# Thermal Processing of Peanut Grains Impairs Their Mimicked Gastrointestinal Digestion While Downstream Defatting Treatments Affect Digestomic Profiles

**DOI:** 10.3390/foods8100463

**Published:** 2019-10-10

**Authors:** Ivana Prodić, Katarina Smiljanić, Ana Simović, Jelena Radosavljević, Tanja Ćirković Veličković

**Affiliations:** 1 University of Belgrade—Faculty of Chemistry, Innovation Center Ltd, 11000 Belgrade, Serbia; iprodic@chem.bg.ac.rs; 2 University of Belgrade—Faculty of Chemistry, Center of Excellence for Molecular Food Sciences & Department of Biochemistry, 11000 Belgrade, Serbia; katarinas@chem.bg.ac.rs (K.S.); radosavljevic@chem.bg.ac.rs (J.R.); 3 Ghent University Global Campus, Incheon 406-840, Korea; 4 Ghent University, Faculty of Bioscience Engineering, 9000 Ghent, Belgium; 5 Serbian Academy of Sciences and Arts, 11000 Belgrade, Serbia

**Keywords:** peanut allergens, thermal processing, food matrix, compliance with the protocol, defatting of peanuts, gastrointestinal digestion

## Abstract

Resistance to digestion by digestive proteases represents a critical property of many food allergens. Recently, a harmonized INFOGEST protocol was proposed for solid food digestion. The protocol proposes digestion conditions suitable for all kinds of solid and liquid foods. However, peanuts, as a lipid-rich food, represent a challenge for downstream analyses of the digestome. This is particularly reflected in the methodological difficulties in analyzing proteins and peptides in the presence of lipids. Therefore, the removal of the lipids seems to be a prerequisite for the downstream analysis of digestomes of lipid-rich foods. Here, we aimed to compare the digestomes of raw and thermally treated (boiled and roasted) peanuts, resulting from the INFOGEST digestion protocol for solid food, upon defatting the digests in two different manners. The most reproducible results of peanut digests were obtained in downstream analyses on TCA/acetone defatting. Unfortunately, defatting, even with an optimized TCA/acetone procedure, leads to the loss of proteins and peptides. The results of our study reveal that different thermal treatments of peanuts affect protein extraction and gastric/gastrointestinal digestion. Roasting of peanuts seems to enhance the extraction of proteins during intestinal digestion to a notable extent. The increased intestinal digestion is a consequence of the delayed extraction of thermally treated peanut proteins, which are poorly soluble in acidic gastric digestion juice but are easily extracted when the pH of the media is raised as in the subsequent intestinal phase of the digestion. Thermal processing of peanuts impaired the gastrointestinal digestion of the peanut proteins, especially in the case of roasted samples.

## 1. Introduction

Peanuts (*Arachis hypogaea* L.) are considered a valuable source of proteins, lipids, dietary fiber, vitamins, minerals, phenolic compounds, and phytosterols. Despite their economic importance as a protein source, the peanut is renowned to frequently provoke allergic reactions in sensitive individuals. Among the sixteen known peanut allergens, Ara h 1 is responsible for 35–95% of peanut-allergic patients, and Ara h 2 is responsible for 95% of allergic reaction in peanut-allergic patients [1], of the class I allergens (complete allergens) that are able to sensitize and elicit severe, generalized symptoms [2].

Schmitt and his colleagues have extensively studied the structural stability and IgE binding of thermally processed peanuts (boiled, fried, and roasted), and found that protein solubility is reduced in all thermal treatments and that this trend increases with time of heat exposure due to protein aggregation [3].

In addition to thermal and non-thermal food processing, which alter protein structure and stability [4], resistance to digestion by digestive proteases represents a critical property of many food allergens. In vitro digestion models simulating the digestion process in the human gastrointestinal system are a useful tool to assess digestion stability, especially if the protocol is applied correctly and transparently. Different digestion protocols have been carried out on purified proteins or defatted aqueous extracts of food proteins, paying no attention to the impact of the food matrix, and differing in the amount of pepsin/pepsin activity units applied per unit mass of the proteins and pH values of the digestion mixtures, as described in the study by Fu and colleagues [5].

In the consensus paper guided by Minekus and colleagues [6], undertaken during the COST INFOGEST action, the general standardized in vitro static digestion method was introduced. This paper suggests the conditions for food digestion which are closest to the physiological conditions found in the gastrointestinal tract. Moreover, the impact of the food matrix in the complete food was taken into account, since this protocol suggests the use of the whole food in the digestion assays.

Combining the effect of the thermal processing of peanuts with stepwise gastrointestinal digestion based on the Minekus’ protocol, Rao and colleagues [7] assessed the effects of the boiling and roasting of whole peanut grains on the peanut protein extraction and binding of IgE from human sera. However, before any functional test, it is of utmost importance to comprehensively characterize the extractome and digestome of the whole food, strictly complying with the INFOGEST protocol. This is the primary step because of the protective effects of the food matrix; allergens from whole raw peanut grains are 500 times more resistant to gastric digestion compared to extracts or purified peanut allergens [8]. The need for strict compliance with the INFOGEST protocol has already been stressed by di Stasio and colleagues in their expression of concerns regarding the relevance of existing digestion results, since protein aggregation and the interaction of allergens with non-protein components, which occurs naturally in the food matrix, affect the bioavailability of allergenic determinants [9].

Therefore, it is not advisable to defat the food samples prior to oral, gastric, and gastrointestinal digestion if the goal is to investigate the digestion susceptibility of the proteins present in the food. However, the downstream processing of the digested food samples, most of the time, requires a defatting step in order to make the proteins and peptides suitable for analysis [8,9]. Even though several studies have performed various post-digestion defatting steps prior to the downstream peanut proteins assessment [7,8,9], the need to compare different delipidation protocol arises, since it is important to be aware of what has been lost from the full digests during defatting.

In this study, we tested the outcomes of different post-digestion defatting procedures of raw, boiled, and roasted peanut grains. In addition, we assessed the outcomes of gastric digestion of both fat- and protein-rich foods, defatted in the optimal manner (spinach, chicken breasts, milk, walnuts, and hazelnuts). We aimed to challenge the methodological difficulties in solid food digestion assays related to the loss of the food matrix and digestome that occur if a lipid-rich food, such as peanuts, is defatted prior to the performed digestion. Finally, we showed how different thermal treatments of peanut grains alter the protein extraction and digestibility of the major peanut allergens by monitoring the stepwise gastrointestinal digestion effects through determination of the protein concentration, gel electrophoreses, and high-resolution mass spectrometry, while strictly complying with the in vitro static INFOGEST protocol, mimicking physiological conditions [6].

## 2. Materials and Methods

### 2.1. Reagents and Materials

α-Amylase from human saliva (A0521-500 UN; Type IX-A, lyophilized powder 1000–3000 U/mg protein), porcine pepsin from gastric mucosa (P6887-1G, lyophilized powder 3200–4500 U/mg protein), pancreatin (P7545, lyophilized powder, 8 X USP), and porcine bile extract (B8631, lyophilized powder) were purchased from Sigma–Aldrich (Saint-Louis, MO, USA). AEBSF- 4-(2-aminoethyl) benzene sulfonyl fluoride hydrochloride was purchased from Fluka, Ref: 76307 (Sigma-Aldrich, Saint-Louis, MO, USA). All other chemicals were of analytical reagent grade and purchased from Sigma–Aldrich (Saint-Louis, MO, USA), unless stated otherwise. In all experiments, ultra-pure deionized water (18 mΩ) was used (Smart2Pure3™ Barnstead aqua purification system (Thermo Fisher Scientific, Waltham, MA, USA)). The enzyme activities were measured according to the assays detailed by Brodkorb and colleagues [10]. A mixture of seven native proteins (14.4–116 kDa), unstained for use as size standards in protein electrophoresis, were purchased from Thermo Fisher Scientific (Waltham, MA, USA).

### 2.2. Thermal Processing

Raw peanuts (*Arachis hypogaea* L.) were purchased from a local grocery and were roasted for 20 min at 170 °C in a pre-warmed home oven. Raw peanut beans (1:10 *w*/*v*) were boiled in water (100 °C) for 20 min [11]. All three peanut preparations were milled using a coffee grinder (800 W, Bosh) three times for 5 min, to obtain a particle size < 1.5 mm. All preparations were kept in 50 mL falcon tubes at 4 °C to avoid extra water evaporation and for use in later experiments. Please see Supporting Materials for the details of the fat- and protein-rich food preparation and processing (spinach, chicken breast, milk, walnut, and hazelnut).

### 2.3. Simulated Oral, Gastric, and Intestinal In Vitro Digestion Conditions

#### 2.3.1. Oral Phase

Ground peanut (0.2 g) was mixed with 100 μL of 2× concentrated simulated salivary fluid (SSF) stock solution. Human salivary α-amylase (20 μL, 1500 U/mL in water) was added to achieve a final concentration of 75 U/mL in the digestion mixture, followed by the addition of CaCl_2_ (20 μL, 15 mM) to obtain the final 0.75 mM concentration. Sixty, fourteen, and ninety μL of water was added to raw, boiled and roasted peanuts, respectively, to compensate for the water loss during the thermal processing and to achieve 200 μL of 1 × SSF concentration. All reagents were pre-warmed at 37 °C for 15 min. Appropriate controls without amylase (only solid peanuts with fluids) were also included. The reaction mixture was incubated for 2 min at 37 °C with agitation, and the gastric phase proceeded after this phase.

#### 2.3.2. Gastric Phase

Completed oral phase material was mixed with 0.4 mL of simulated gastric fluid (SGF) stock solution (1× concentrated SGF) and 20 μL of CaCl_2_ (3 mM in 1× SGF) to achieve a final concentration of 75 µM in the digestion mixture. Porcine pepsin (356 μL; 4500 U/mL in 1× SGF) was added, reaching a final concentration of 2000 U/mL in the digestion mixture. The pH of the mixture was adjusted to 3.0 ± 0.2 with 1 M HCl, and 1 × SGF was added to the final volume of 0.8 mL. The reaction mixture was incubated for 120 min at 37 °C with intense agitation (600 rpm). Control samples were run in the same manner: peanut control (oral bolus without amylase with the addition of 356 μL 1× SGF instead of pepsin solution) at 120 min (Ct 120’), and pepsin control (with 0.2 mL of 1× SSF stock solution and 0.2 g of sand instead of oral bolus at 0 min (pepsin 0’) and 120 min (pepsin 120’)). Digestion was stopped by the addition of 200 μL 2 M NaHCO_3_ to change the pH of the final reaction mixture to 8.0.

#### 2.3.3. Intestinal Phase

The complete gastric chyme of 0.8 mL after 120 min of digestion was mixed with a pancreatic suspension prepared in 400 μL of 1× concentrated simulated intestinal fluid (SIF), with 400 U/mL of trypsin activity in the fluid (100 U/mL in the final digestion mixture). The bile solution (61 μL; 263 mM) and CaCl2 (50 μL; 20 mM) were added to reach a final concentration of 10 mM and 0.3 mM in the digestion mixture, respectively. The necessary volume of 1 M NaOH (100 μL) was added to adjust the pH to 7.0. Finally, 189 μL of deionized water was added to adjust the volume of the mixture so a 1× SIF concentration was achieved. Samples were incubated on a rotating wheel for 2 h at 37 °C. Digestion was stopped by the addition of 50 μL AEBSF (32 mM) to obtain a final concentration of 1 mM in the gastrointestinal mixture.

### 2.4. Post-Gastric or Post-Gastrointestinal Defatting Treatments

After the gastric or gastrointestinal digestion phase, the samples were centrifuged at 10,000 g for 20 min, and the liquid phases were separated. Every sample was divided into three equal aliquots for the assessment of different defatting treatments. One aliquot was kept non-defatted. The second aliquot was defatted by TCA/acetone: 200 μL of liquid phase was mixed with cold 20% TCA/acetone (1:1, *v*/*v*) and left overnight at −20 °C. After removing the supernatant by centrifugation at 13,000 g for 30 min, the pelleted proteins were washed twice with 1 mL of cold acetone, and intensively vortexed and centrifuged at 13,000 g for 15 min. The pellet was dried for 1 h at RT and re-suspended in 200 μL of 1.5× Laemmli sample buffer (reducing and non-reducing). The third aliquot of the liquid phase (400 μL) was defatted with n-hexane: nine volumes of n-hexane were added to the aliquot and mixed for 60 min at RT, and centrifuged for 30 min at 4000 rpm. The upper n-hexane layer was discarded, and the re-extraction of lipids by n-hexane was repeated twice.

### 2.5. Insoluble Peanut Fractions

Pellets (0.2 g) obtained after centrifugation of the samples subjected to gastric digestion were considered insoluble fractions and were extracted with denaturing buffer (8 mL; 7 M urea, 2 M Thiourea, 2% CHAPS, 1% Triton X-100 in 200 mM NaHCO3, pH 8.0). The samples were vortexed (three × 30 s) and incubated in an ultrasound bath for 45 min. The pellet was titrated with 1 M NaHCO3, pH 9.0, to pH 8.5, and left overnight (18 h). In the morning, the pH was confirmed by indicator paper and samples were centrifuged at 13,000 rpm for 20 min. The supernatant was collected and used for SDS-PAGE. To each well, a 10 µL sample was applied for electrophoresis.

### 2.6. SDS-PAGE

SDS-PAGE was carried out on 14% polyacrylamide gel according to the Laemmli protocol [12], and gels were stained with Coomassie Brilliant Blue R-250 (CBB). Digestive enzymes (pepsin and pancreatin) in 0 and 120 min were also run as controls. Particular care was taken to apply the same volume of the samples in the gel.

### 2.7. Determination of Protein Concentration 

#### 2.7.1. Bicinchoninic Acid (BCA) Assay

Protein concentration was determined by BCA assay (Thermo Fisher Scientific Inc., Bremen, Germany) after diluting the liquid phases of non-defatted and hexane-defatted digestion mixtures 20-fold in phosphate-buffered saline (PBS). After TCA/acetone precipitation, the obtained precipitate was dissolved in the same volume of PBS, and upon a 20-fold dilution in PBS, the protein concentration was determined by BCA with standard curves with samples being heated to 60 °C to favor complex formations with peptide bonds [13].

#### 2.7.2. Densitometry with ImageQuant TL Version 8.1

Imaging and analyses of SDS-PAG electrophoretic profiles of thermally treated peanut digests were performed with a laser biomolecular imager (Typhoon FLA 7000 series) and Image 1D Quant TL 8.1 software (GE Healthcare, Howard County, MA, USA). To quantify the intensity of the CBB-stained protein bands, a non-calibrated mode pixel inverter function was chosen with automatic-width peak (band) detection, a peak slope of 75–125, and a noise reduction level of 5. A minimum height of the peak was detected at ≥2% of the highest peak in the sample, and a minimum profile was used for background subtraction. Thermo Scientific™ Pierce™ unstained protein molecular weight markers (8 µL), a mixture of seven native proteins (14.4 kDa to 116 kDa, final concentration 1 mg/mL), were used to normalize protein band volumes in a collective mode (volume = area × pixel intensity, seven marker bands, total volume equal to 8 µg). The cubic spline model of the molecular weight calibration curve was chosen as the best-fitting model. This approach allowed the exclusion of certain bands from the quantification (such as pepsin or pancreatic protein bands). Figure S1 provides a representative example of the entire process of digitalization (pixelization) and volume quantification of the bands in the electrophoretic profiles of the gastric and gastrointestinal digestion of thermally processed peanuts.

### 2.8. Identification of Peanut Proteins by Nano-LC-MS/MS

#### 2.8.1. Band Excision and Preparation for MS

The entire CBB stained gel strips of the controls and gastric digestion of raw peanut liquid phases, defatted with TCA/acetone, were cut into dozens of gel bands. Protein gel bands were washed, reduced with dithiothreitol, and alkylated with iodoacetamide, followed by in gel trypsin-digestion [14] with minor modifications. The amount of trypsin was leveled against a densitometry-measured quantity of proteins in the gel bands so as to keep the same trypsin/sample ratio of 1:30 (*w*/*w*). The working concentration of MS-grade trypsin diluted in 25 mM ammonium bicarbonate buffer was 10 ng/µL. The resulting tryptic, semi tryptic and atryptic peptides were analyzed by high-resolution, nano-liquid chromatography (LC)-tandem mass spectrometry (MS/MS).

#### 2.8.2. Nano-LC-MS/MS

The peptide samples were chromatographically separated on an EASY-nLC II system (Thermo Fisher Scientific Inc., Bremen, Germany) with two columns set up: a trap column C18-A1, 2 cm (SC001, Thermo Fisher Scientific, Waltham, MA, USA) and an analytical column PepMap C18, 15 cm × 75 µm, 3 µm particles, and 100 Å pore size (ES800, Thermo Fisher Scientific, Waltham, MA, USA). Samples (4–8 μL) were analyzed with an LTQ Orbitrap XL hybrid mass spectrometer (Thermo Fisher Scientific Inc., Bremen, Germany). MS data were acquired in the data-dependent MS^2^ mode. Data acquisition was controlled by XCalibur 2.1 software (Thermo Fisher Scientific Inc., Bremen, Germany).

#### 2.8.3. Protein Database and PEAKS X Suite Search Parameters

The identification of peanut proteins was performed by PEAKS Suite X (Bioinformatics Solutions Inc., Canada). Signature MS/MS spectra were searched using the PEAKS post-translational modification (PTM) algorithm against a peanut (*Arachis hypogaea* taxon identifier 3818) database consisting of UniProtKB reviewed and non-reviewed sequences, downloaded on 01/10/2018 from http://www.uniprot.org/, and the Max Quant contaminant database (downloaded on 01/03/2019 from http://www.coxdocs.org/doku.php?id=maxquant:start_downloads.htm).

In the PEAKS DB algorithm, which is essential for a database protein search within the PEAKS X Suite platform, the following modifications were taken into account as variables: oxidation (Met), deamidation (Gln, Asn), and hydroxylation (Pro), while carbamidomethylation (Cys) was set as a fixed modification. Up to two missed trypsin cleavages were allowed per peptide. A non-specific mode of trypsin cleavage was chosen at both ends of the peptides, enabling tryptic, semi-tryptic, and atryptic peptide detection. Mass tolerances were set to ±10 ppm for parent ions and ±0.5 Da for fragment ions. Protein filters were set to protein −10 log P ≥ 20, proteins’ unique peptides ≥ 1, and an AScore for confident PTM identification of at least 20. The peptide filter was set to a false discovery rate for peptide-spectrum matches <0.1%; therefore, the resulting false discovery rate of the peptide sequence was lower than 0.5%, and the de novo alignment local confidence score was ≥80%. In the PEAKS PTM algorithm, an unrestricted, variable PTM search was undertaken using an available list of 313 naturally occurring modifications from Unimod.

### 2.9. Assessment of Pepsin Efficacy

The pepsin efficacy was assessed by densitometry comparison, using Image 1D Quant TL 8.1 software (GE Healthcare, Howard County, MA, USA), of the protein content in the lanes corresponding to the control and the matched digested samples. The efficacy percentage was expressed as: (intensity of the band(s) in the control sample—intensity of the corresponding matched band(s) in the digested sample) / intensity of the band(s) in the control sample × 100.

### 2.10. Statistical Analyses

All analyses, including normality testing, two-way ANOVA with matched samples and the post-tests of multiple comparisons such as Dunnett’s and Sidak’s, were undertaken by GraphPad Prism 7.0 software (GraphPad, San Diego, CA, USA).

## 3. Results and Discussion

In this study, the boiling and roasting of whole peanut grains were combined with gastrointestinal digestion according to the in vitro static protocol mimicking physiological conditions in order to understand the impact of the food matrix and common thermal treatments on the digestion stability of major peanut allergens [6]. Moreover, TCA/acetone and n-hexane defatting approaches were applied to the digested samples, and the effect of the defatting procedure on the recovery of proteins from the peanut extractome (extracted proteins in simulated gastric and intestinal juices without digestive enzymes, i.e. controls) and digestome (extracted and digested proteins in simulated gastric and intestinal juices with digestive enzymes) is discussed. Changes in the solubility of peanut proteins in simulated gastric and intestinal juices due to thermal treatments were assessed by measuring protein contents with a BCA assay and densitometry of the SDS-PAGE profiles. The detailed proteome investigation of the gastric digestion of raw peanuts was studied by high-resolution mass spectrometry and an improved PEAKS X PTM algorithm. Our results showed specific cleavage sites originating from pepsin activity and shed a light on post-translational modifications of the MS-detected peptides present in the raw peanut digestome.

### 3.1. Effects of TCA/Acetone and N-Hexane Defatting on the Protein Concentration of Gastric and Gastrointestinal Digests of Whole Peanuts

**Figure 1:**
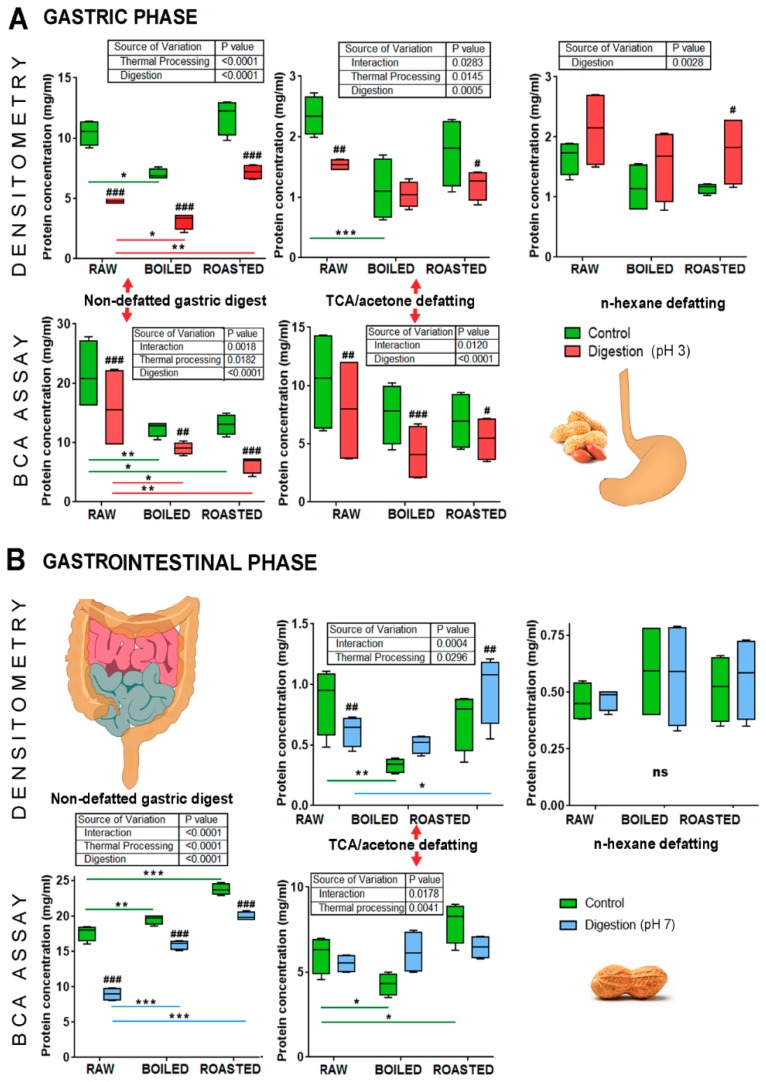
Protein concentrations in the full liquid phases of the control (extraction), gastric and gastrointestinal digested raw, boiled, and defatted peanuts and following n-hexane and TCA/acetone defatting. **A**—Gastric phase—protein content determined by densitometry and BCA assay, before and after defatting with n-hexane and TCA precipitation. **B**—Intestinal phase—protein content determined by densitometry and BCA assay, before and after defatting with n-hexane and TCA precipitation. A two-way ANOVA with matched measurements was employed on at least two independently performed digestions (followed by defatting treatment, if done). Both BCA assay and ImageQuant TL densitometry included technical duplicates. Multiple comparison testing was done following two-way ANOVA with repeated (matched) measures via Sidak’s and Dunnett’s post-tests to test differences in digestion within a single thermal treatment and across thermal treatments, respectively; ns—not significant (e.g., *p* > 0.05): #, ##, and ###—significantly different digested sample from its control at *p* < 0.05, *p* < 0.005, and *p* < 0.0001, respectively. *, **, and ***—significantly different boiled or roasted peanut sample compared to a raw peanut sample at *p* < 0.05, *p* < 0.005, and *p* < 0.0001, respectively.

**Figure 2:**
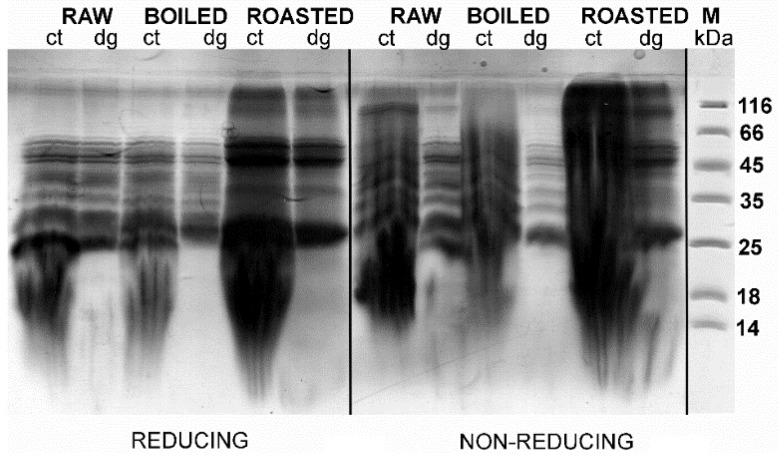
SDS-PAGE protein profiles of non-defatted raw, boiled, and roasted peanut gastric digests and corresponding controls. SDS-PAGE was performed in reducing and non-reducing conditions on 14% gels; ct—control at 120 min; dg—digest at 120 min; M—molecular weight markers.

We compared two defatting treatments, n-hexane and TCA/acetone, with the full, non-defatted liquid gastric and gastrointestinal digestion phases of raw, boiled, and roasted peanuts. The methodological prerequisite for assessing the extraction efficacy and digestibility of peanut proteins in simulated juices is a defatting procedure. According to our results, the choice of defatting procedure alters the results when examining protein concentration, extraction, and gastrointestinal digestion of differently thermally treated peanuts. Protein concentration was assessed by BCA assay and densitometry tests (Figure 1). Both tests have their advantages and disadvantages when examining complex proteomes and digestomes, such as the full food matrix of non-defatted, gastric digestion phase of raw, boiled, and roasted peanuts being electrophoretically resolved and are presented in Figure 2. The BCA test, on the other hand, is suitable for digestomic/peptide concentration measurements, and achieves a better performance compared to other colorimetric tests in resisting the interference to other substances, though it is not devoid of interferences [13], and many times we have found falsely high results with the BCA test in complex protein mixtures rich in polyphenolics and sugars (unpublished data).

In Figure 1A, it can be seen that the protein concentrations as measured by BCA assay in non-defatted control peanut samples are 21.0 mg/mL for raw and 13.0 mg/mL for boiled and roasted, the former being similar to the 16.6 mg/mL of extracted peanut proteins at pH 3.0 [15]. The trend we observed is in agreement with the one described by Schmitt and colleagues in 2010 [3]. Lower protein concentrations in boiled peanut samples can be only partially attributed to the leaching of peanut proteins into the cooking water [11,16,17]. These results were supported by our experiments, in which 5 mg of proteins leached into 50 ml of water during 20 min of boiling, representing 2.6% of the maximal amount of extracted boiled peanut proteins under the conditions set for gastric digestion. Different extraction conditions (neutral pH, different buffers, longer time) and longer boiling time were employed (30 min) in other studies [16,17], resulting in an increased percent of leached proteins of 4% and 5.2% out of total extracted boiled peanut proteins, respectively. A decrease in protein concentration in the case of non-defatted digested roasted peanut is, most probably, the result of diminished protein solubility due to the excessive protein aggregation promoted by the Maillard reaction during roasting [11,18]. The protein content in the differently thermally treated peanut samples subjected to digestion showed a similar trend to the control samples, but with lower values (16.0 mg/mL, 9.0 mg/mL, and 6.0 mg/mL for the raw, boiled, and roasted peanuts, respectively). In addition, higher protein concentrations in the control compared to digested samples were observed in all full liquid phases, regardless of the assessment methodology (Figure 1A,B). We assume that this phenomenon is a consequence of the decreased content of peptide bonds imposed by pepsin action and, hence, a diminished ability to form colored complexes with BCA molecules (e.g., single amino acids and dimers are out of the test sensitivity when 60 °C heating is applied during incubation). On the other hand, small peptides (below four amino acids) easily migrate after the dye front (bromophenol blue line), with a possibility of running out of SDS-PAGE and are thus excluded from densitometry measurements.

In contrast to the case of simulated gastric digestion, the opposite trend was observed across thermally treated peanuts in simulated intestinal digestion; the raw peanut samples exhibited the lowest protein concentrations and the roasted samples the highest (BCA assay, Figure 1A,B). TCA/acetone defatting treatment replicated the trends imposed by digestion and thermal processing seen in non-defatted gastric digestion samples, with a change of trend in the intestinal digestion, whereby digested samples of boiled and roasted peanuts had higher protein concentrations compared to their control counterparts as detected by both techniques (Figure 1A,B). Interestingly, n-hexane defatting of gastric digests diminished the protein concentrations in control samples to levels lower than the concentrations in the digested samples. However, n-hexane treatment did not alter the protein concentration trends when thermal treatments were compared (Figure 1A). In addition, the absolute values of protein concentrations were higher in all TCA/acetone defatted samples compared to the respective counterparts of n-hexane defatted samples (Figure 1A,B). Therefore, even at this stage of the assessment, a preference for the choice of TCA/acetone as the more suitable defatting treatment of peanut digests was noted.

### 3.2. Effects of TCA/Acetone and N-Hexane Defatting on SDS-PAGE Profiles of Gastric and Gastrointestinal Digests of Whole Peanuts

**Figure 3:**
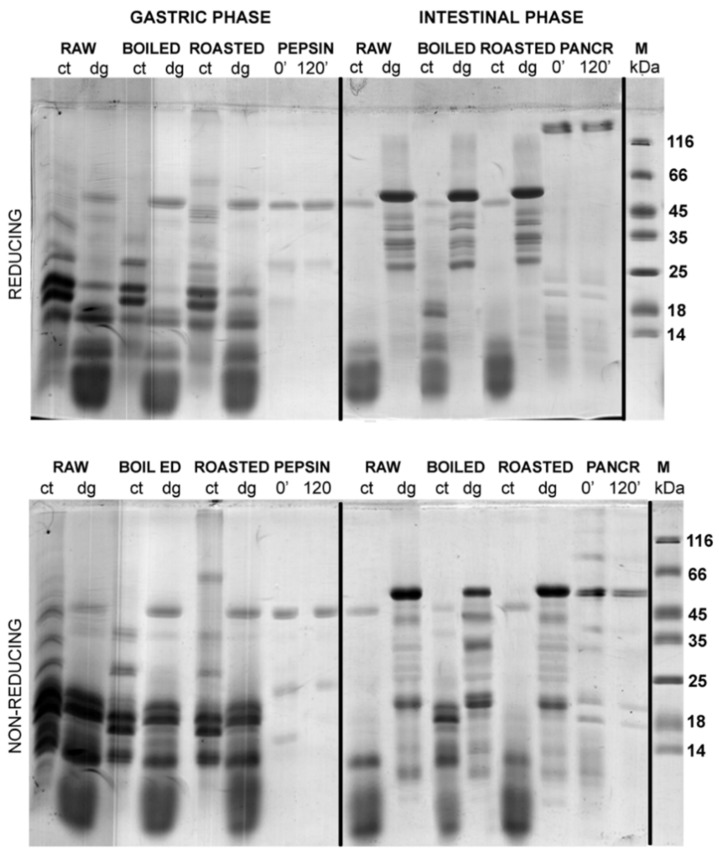
SDS-PAGE protein profiles of n-hexane-defatted raw, boiled, and roasted peanut gastric and intestinal digests and corresponding controls. SDS-PAGE was performed in reducing and non-reducing conditions on 14% gels. ct—control at 120 min; dg—digest at 120 min; PEPSIN—controls containing only pepsin at 0 and 120 min; PANCR—controls containing only pancreatin at 0 and 120 min; M—molecular weight markers.

**Figure 4:**
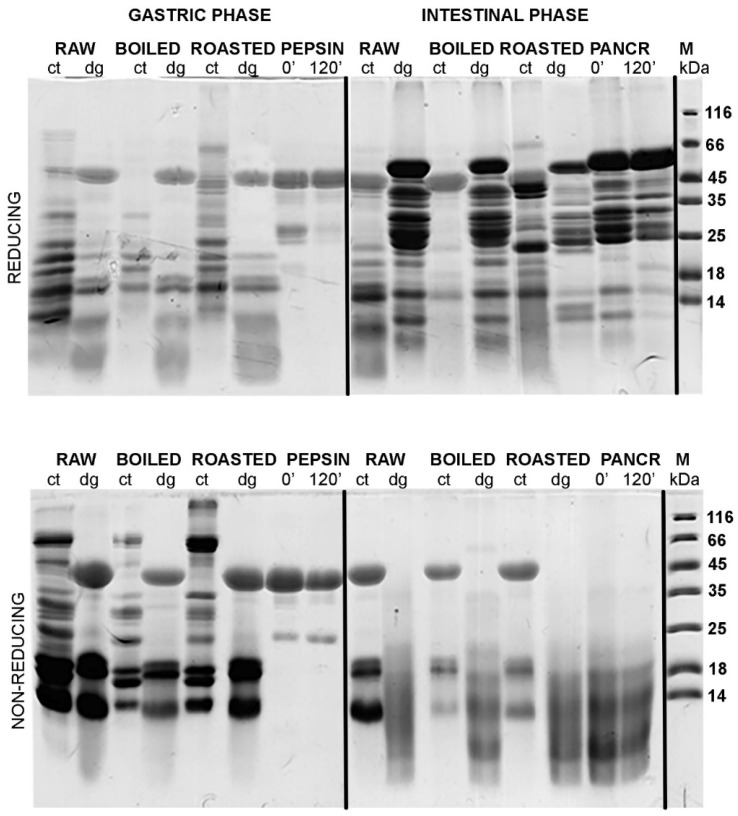
SDS-PAGE protein profiles of TCA/acetone defatting and protein precipitation of raw, boiled and roasted liquid peanut gastric and intestinal digests and their controls. SDS-PAGE was performed in reducing and non-reducing conditions on 14% gels. ct—control at 120 min; dg—digest at 120 min; PEPSIN—controls containing only pepsin at 0 and 120 min; PANCR—controls containing only pancreatin at 0 and 120 min; M—molecular weight markers.

A comparison of the efficacy of defatting procedures in preserving the proteins in defatted gastric digests and corresponding controls was undertaken by densitometry analysis (Figure 1A, Table S1) that included the results following normalizing against non-defatted samples (Figure 2, Figure 3 and Figure 4). In raw, boiled, and roasted peanut gastric digestion controls, protein concentrations were reduced to 76%, 76%, and 85% of their value upon TCA precipitation, respectively. In the case of gastric digests of raw, boiled, and roasted peanuts, protein loss upon TCA/acetone defatting was 68%, 66%, and 81% of the protein concentration without defatting, respectively. The same calculation was applied to the n-hexane treatment and, in this case, the situation was slightly more unfavorable: for the gastric digestion control of raw, boiled, and roasted peanut samples, protein loss was 84%, 83%, and 90%, respectively. The samples of raw, boiled, and roasted peanuts digested in conditions simulating gastric digestion lost 55%, 50%, and 75% of their protein content, respectively, upon n-hexane defatting. However, this was a consequence of the inverse trend between the control and digested samples upon n-hexane defatting, observed in the gastric phase (Figure 1A). This is also an additional argument favoring the TCA/acetone defatting step over n-hexane defatting when this step is mandatory for downstream applications.

Figure 3 and Figure 4 show the protein profiles of the thermally treated control and digested peanut samples in reducing and non-reducing conditions after defatting with n-hexane and TCA/acetone, respectively. Generally, TCA/acetone defatting provided samples with similar SDS-PAGE profiles (Figure 4) to the ones observed in the non-defatted gastric digests in Figure 2. Within all lanes in both Figure 3 and Figure 4, the usual smears of lipids in lower molecular masses are absent when compared to the non-defatted control samples (Figure 2), facilitating observance of lower molecular mass proteins, such as Ara h 2 and Ara h 6, which are present as triplets in the range of 15–18 kDa.

A study conducted by Wacyzk and colleagues [19] on the defatting of raw and thermally processed peanuts followed by protein extraction showed that the defatting procedure affects subsequent extraction of proteins, with defatting with n-hexane providing a higher yield of Ara h 1 and Ara h 2 allergens in the peanut extract. However, in our case, when defatting was undertaken upon extraction, Ara h 1 was more preserved in the sample if TCA/acetone defatting was applied (Figure 3 and Figure 4).

The additional reason we opted for TCA/acetone precipitation of proteins as a favorable defatting procedure for the follow-up of this study was that our main focus was to investigate the digestion effects on larger fragments of molecular masses higher than 10 kDa. We are aware that for the investigation of a whole peanut digestome, it is necessary to identify small digestion-resistant fragments [8]. Since TCA/acetone precipitation is not quantitative and leads to the loss of small fragments, this may mislead us in concluding whether changes come from the difference in digestibility of differently treated peanuts or from washing out small peptides. However, TCA/acetone precipitation was exploited in our study because the amount of recovered larger digestion fragments was higher than upon defatting with n-hexane.

### 3.3. Effects of the Lipid Content in the Food Matrices on Gastric Digestion

In Figure S2, various fresh and processed foods are presented as examples of different food matrices that were digested according to the in vitro static INFOGEST protocol for gastric digestion: fresh spinach, pasteurized milk, raw walnuts, raw hazelnuts, and boiled chicken breasts. Upon digestion, the samples were defatted with TCA/acetone in the same way as the raw and thermally processed peanuts. Even though the majority of the protein content was lost, as in the peanuts (Figure 1, Figure 2, and Figure 4), similar SDS-PAGE profiles were obtained when compared with the corresponding non-defatted samples (data not shown). Figure S2 represents the capabilities of different food matrices to protect proteins present in them from degradation. In our experimental setup, walnut and, to a slightly lesser extent, milk matrices were the most efficient in preserving proteins during gastric digestion, as the largest protein fragments are seen in these digests. This observance is probably due to the higher content of lipids present, especially in walnuts compared to chicken breasts and spinach. The SDS-PAGE profiles of peanuts, walnuts, and hazelnuts (Figure 2, Figure 3 and Figure 4, and Figure S2) indicate that the lipids are an important factor in hampering simulated gastrointestinal digestion. Though the use of gastric lipase is proposed by the novel, updated INFOGEST 2.0 digestion protocol, we did not use it since, at the time of conductance of this research, this protocol had not been published [10]. However, the inclusion of gastric lipase in the simulated gastric digestion is highly recommended, since it will contribute to more realistic digestion conditions, especially in lipid-rich food samples. The hydrolysis of lipids might also indirectly affect pepsin digestion. Moreover, the use of lipase would probably ease the manipulation of digested samples, since the prerequisite defatting steps might be able to be omitted.

When the food samples digested in simulated gastric and gastrointestinal conditions were thoroughly defatted in preparation for SDS-PAGE analysis, the loss of some proportion of the allergenic fragments and intact proteins was hard to avoid. We believe that the different defatting approaches for food samples and food digests are an important cause of the discrepancy observed in peanut allergen digestion resistance reported by different research groups, even when the same INFOGEST protocol is applied for the peanut digestion [7,8,9]. In our view, the most reproducible results were obtained when non-repetitive defatting with TCA/acetone was used on digested samples. In our current work on hazelnuts, we have succeeded in developing a 2D SDS-PAGE protocol with non-defatted samples. We strongly recommend opting for the optimized electrophoretic protocols instead of defatting the samples, whenever possible. In addition, it is possible to run shotgun proteomics and safeguards against excessive loss of peptides analyzing big and small fragments at once, as shown in the study of whole peanut grain digestome by Di Stasio and colleagues [9].

### 3.4. Effect of Thermal Processing on Protein Extraction and Digestion in the Gastric and Gastrointestinal Digestion Conditions

Our data show that the thermal processing of peanuts (i.e., boiling and roasting) decreases the solubility of peanut proteins and hampers their 120 min extraction by simulated gastric fluid (control samples in Figure 1A, Figure 3, and Figure 4). This conclusion is supported by the statistical evaluation of protein concentrations of the gastric digestion control samples, where the protein concentrations of the samples extracted from raw peanuts were significantly higher than the protein concentrations of boiled and roasted peanuts. Though there is no statistically significant difference among the protein concentrations in the samples extracted from the boiled and the roasted peanuts, it is obvious from our data that the roasted samples had a slightly higher concentration of extracted proteins compared to the boiled samples (Figure 1A).

However, this is not the general conclusion regarding the solubility and efficacy of the extraction of the peanut proteins upon thermal treatment. After extraction in simulated intestinal fluid digestion, the opposite—an increasing trend of solubility/extraction of the peanut proteins—was observed, with the proteins from roasted peanuts being the most easily extracted and providing the highest concentrations in the samples (Figure 1B). We assume that the peanut proteins of the thermally treated samples are extracted with a delay in comparison to the raw peanut proteins. Moreover, we suggest that alkaline solutions (mimicking intestinal conditions) facilitate the extraction of the peanut proteins more than the acidic conditions of simulated gastric fluid. This phenomenon might be particularly relevant for understanding the sensitizing potential and the allergenicity of peanuts. Stemming from studies performed on animal models, limited proteolysis of proteins is crucial for allergenicity [20]. Here, we have observed that the thermal treatment of peanuts enhances solubility/extraction of large fragments and digestion-resistant peptides in the intestines, where sensitization occurs through the interaction of large proteinaceous entities with the immune system.

Generally, the protein extraction trend of the digestion control samples across the thermal treatments was replicated in the digested samples (Figure 1). Overall, gastric digestion efficacy, assessed by BCA tests and densitometry (Figure 1A, Table S1), ranges between 30–55% for raw and boiled samples and 40–50% for roasted peanut samples, depending on the applied protein estimation approach. The differences in digestion efficacy of the proteins of raw, boiled, and roasted peanuts is not statistically significant (Figure 1A). However, upon completion of the intestinal phase of digestion, significant changes in the digestion efficacy are observed; the overall efficacy was 50%, 18%, and 16% for the raw, boiled, and roasted peanut samples, respectively (Figure 1B, Table S1). Pepsin possesses superior digestion efficacy for raw peanuts compared to thermally treated peanuts. However, we should not forget that the extraction of proteins from these two preparations was also higher than from raw peanuts. Finally, the effect observed through the difference of gastrointestinal efficacy is significant, because the absolute concentration values representing the digested portions are 8.5 mg/mL, 3.5 mg/mL, and 3.7 mg/mL for the raw, boiled, and roasted samples, respectively, as assessed by densitometry (Figure 1B, Table S1).

In the non-defatted control samples of the gastric digestion of roasted peanuts, protein bands on the molecular weights higher than 100 kDa were observed in the SDS-PAGE profiles (Figure 2); these bands were more prominent in non-reducing conditions than in reducing conditions. The identity of these bands in boiled and roasted peanut preparations has not been confirmed with mass spectrometry; however, these bands could contain oligomers, as reported by Maleki and colleagues upon the roasting of the peanuts, because of the corresponding molecular weight (2000) [18]. The abundance of these protein bands with potential oligomers decreased upon both defatting treatments (Figure 3 and Figure 4). In addition, these high molecular mass bands are susceptible to the action of pepsin, since they disappear after simulated gastric digestion in all defatting preparations and electrophoretic conditions (Figure 2, Figure 3 and Figure 4).

A protein band around 65 kDa, corresponding to the protein Ara h 1, was visible in the control samples of the gastric digestion of raw and roasted peanuts, in both reducing and non-reducing conditions (Figure 4). Like other proteins from the cupins family, Ara h 1 is thermostable and undergoes irreversible denaturation and extensive aggregation after passing through the dominant endothermal transition at temperatures above 80 °C [21]. For our experiments, we used peanuts roasted for 20 min at 170 °C, which represents extensive thermal treatment. SDS-PAGE analysis indicated that Ara h 1 is one of the proteins that were most affected by roasting (Figure 4). We anticipate that the thermal treatment we applied for roasting the peanuts not only favored the aggregation of Ara h 1, but also might enhance the IgE-binding capacity of this protein since the critical temperature for alteration of the IgE-binding properties is reported to be 140 °C [17].

The basic subunit of Ara h 3 (25 kDa) is present in all digestion control samples of raw, boiled, and roasted peanuts (Table S2) [22]. However, it is easily digested by pepsin, since the corresponding band is missing in the digested samples (Figure 4, Table S2). It is easy to identify the acidic subunit of Ara h 3 in the digestion control samples of all three peanut protein preparations since it is represented as a myriad of bands in the 25–45 kDa region (Figure 2, Figure 3 and Figure 4, and Figure 7, and Table S2.) [23]. In the samples obtained upon gastric/gastrointestinal digestion, only one band in that region is distinguished (Figure 2, Figure 3 and Figure 4, and Figure 7). Moreover, it is obvious that the thermal processing dramatically affected the pepsin resistance of the acidic subunit of Ara h 3, since the 35 kDa bands are visible in the digests of the raw peanuts, but not in the digests of boiled peanuts (Figure 4). The roasting of peanuts affected Ara h 3 in a similar manner as Ara h 1; aggregates were formed and the ability to bind the IgE antibodies was increased [24].

Ara h 2 is seen in the SDS-PAGE as a doublet of bands of 17 kDa and 19 kDa [25], and we observed it in all control samples of gastric/gastrointestinal digestions of peanuts, regardless of thermal treatment or electrophoretic (reducing or non-reducing) conditions (Figure 4). Hence, our findings support the previously reported thermostable nature of this protein. However, a lower abundance of Ara h 2 was noticed in the boiled peanut preparations, at least in part due to the leaching of proteins from the peanuts during cooking [16,17], and the effect increased with the duration of boiling [16]. Figure S3 shows the protein profile of peanut proteins that leaked into cooking water (water was ten times concentrated before the SDS-PAGE). Only fragments of 25 kDa and lower are observed (Figure S3), with a prominent Ara h 2 band at 18 kDa, which is in accordance with previous studies [16, 17].

Peanut conglutins (i.e., Ara h 2 and Ara h 6) are pepsin-digestion resistant not only due to their conserved globular structure but also due to the protective features of the lipid-rich peanut matrix. This specificity of the peanut matrix results in the delayed extraction of the proteins and makes conglutins unavailable for digestion by pepsin at the early onset of gastric digestion. Our results are completely in line with widely accepted knowledge on Ara h 6 as an extremely stable and proteolytically-resistant protein since this protein is detected in all control and gastric-digested samples of raw, boiled, and roasted peanuts. In contrast to gastric digestion, intestinal digestion of Ara h 2 and Ara h 6 by pancreatin results in significant degradation of these two proteins.

**Figure 5:**
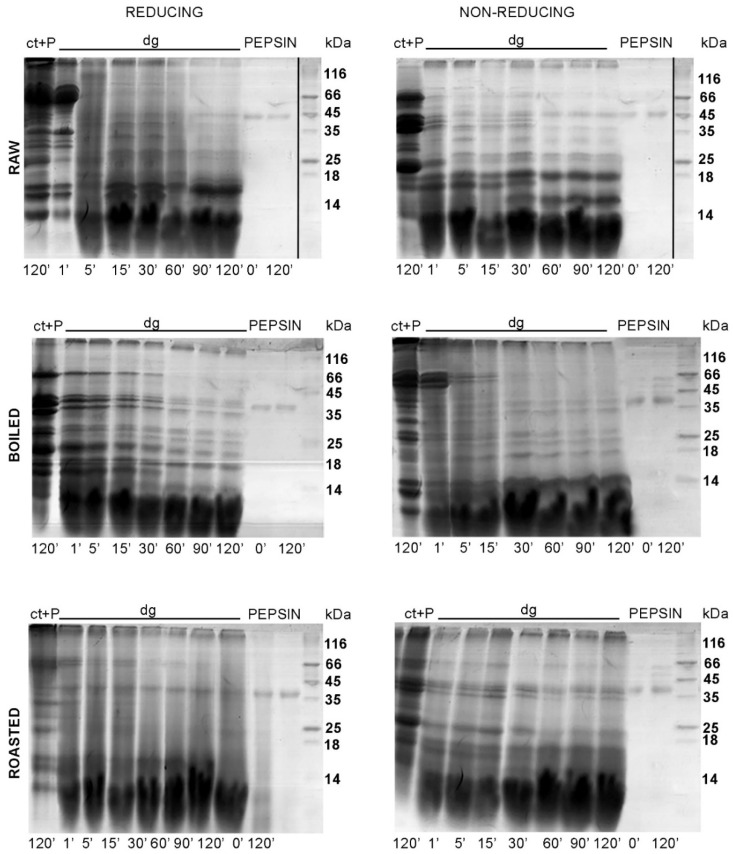
SDS-PAGE protein profiles after the TCA/acetone defatting of raw, boiled, and roasted peanuts digested in conditions mimicking gastric digestion at different time points. SDS-PAGE was performed in reducing and non-reducing conditions on 14% gels. ct + P—control at 120 min containing pepsin; dg—digest at 120 min; PEPSIN—controls containing only pepsin; M—molecular weight markers. The different time points are indicated below the gels.

We also aimed to characterize the gastric digestion of thermally treated peanuts over a 2 h time course. Our focus was on peanut proteins of higher molecular weights, such as Ara h 1 and Ara h 3. In raw peanuts, these two proteins were digested by pepsin in between 60 and 90 min (Figure 5). At those critical time points only Ara h 1 (approximately 65 kDa) was digested in the boiled and roasted peanuts, while the fragments derived from Ara h 3 were still visible in the gastric digests of thermally treated peanuts. Moreover, in all preparations, large protein fragments were visible in the region of 25–45 kDa. Our data suggest that the monitoring of the gastric digestion of peanuts should be extended for a period of 120 min, in contrast to the 10 min gastric digestion in the study by Rao and colleagues [7].

### 3.5. SDS-PAGE Protein Profiles of Insoluble Fractions of Gastric Digests of Thermally Treated Peanut Samples

**Figure 6:**
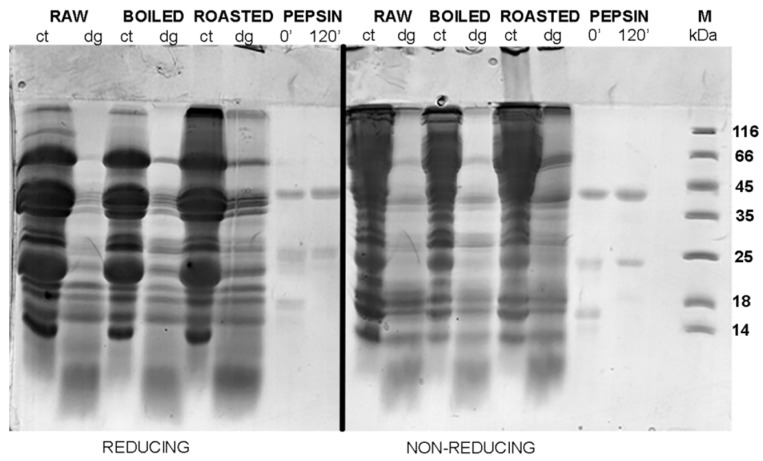
SDS-PAGE protein profiles of insoluble fractions after gastric digestion of raw, boiled, and roasted peanut preparations. SDS-PAGE was performed in reducing and non-reducing conditions, on 14% gels. ct—control at 120 min; dg—digest at 120 min; PEPSIN—controls containing only pepsin at 0 and 120 min; M—molecular weight markers.

The SDS-PAGE profiles of pellets of control and pepsin-digested raw, boiled, and roasted peanuts are shown in Figure 6. A pellet is the insoluble material/precipitate upon centrifugation of obtained digests. The protein profiles of the samples obtained by the digestion of the raw and boiled samples were quite similar. Protein aggregates of very high molecular weights (smear in the concentrating gel) were the most prominent in non-reducing conditions of the roasted peanut digestion control. Proteins Ara h 1 (63 kDa), Ara h 2 (16–18 kDa), Ara h 3 (basic subunit 25 kDa, acidic subunit 25–45 kDa), and Ara h 6 (15 kDa), in all digestion control samples of thermally treated peanuts, were detected at their expected molecular weights, regardless of the electrophoretic (reducing or non-reducing) conditions. We suggest that during intestinal digestion, the proteins from roasted peanuts are more easily extracted than proteins from raw and boiled peanuts. Our findings support the higher sensitization capacity of roasted peanuts when compared with boiled or raw peanuts.

### 3.6. Raw Peanut Gastric Digestion Assessment by the Improved NLC-MS/MS Method and Bioinformatics Approach 

A thorough assessment of the gastrointestinal digestion efficacy solely by the densitometry analysis of SDS-PAGE profiles is prone to erroneous estimations, due to the possible overlapping of the different protein fragments at the same position in the gel. Moreover, this is of great importance when assessing the digestion efficacy of the proteins of lower molecular weights, since the presence of the fragments derived from the proteins of higher molecular weights in the same position in the gel could cause false readings, and higher quantities could be assigned to the lower molecular-weight protein.

**Figure 7:**
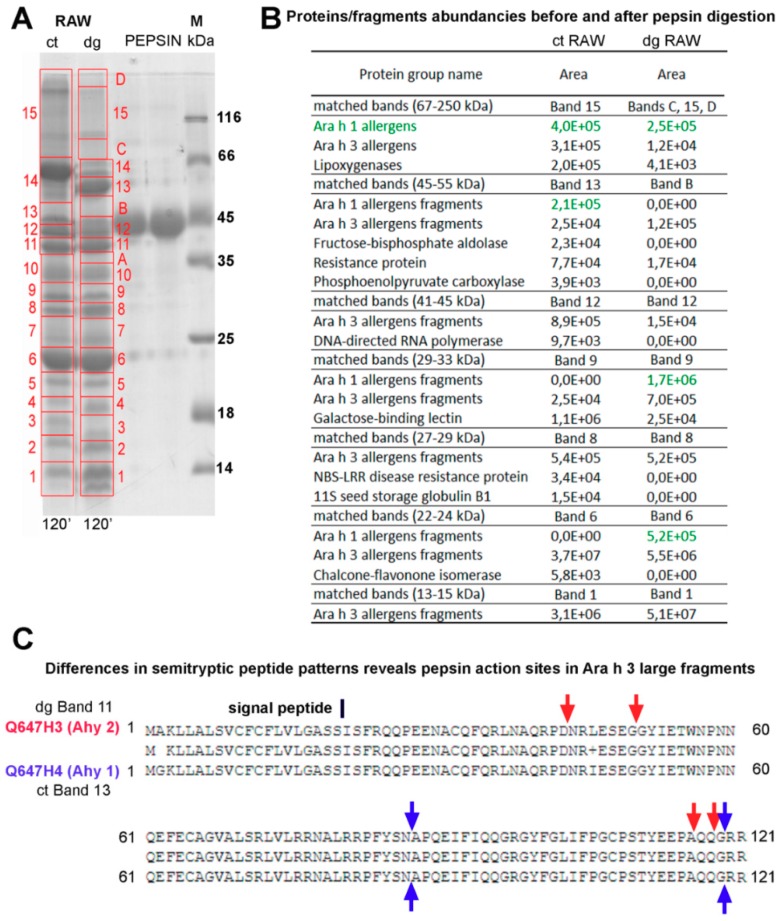
Protein and fragment identification in gastric digestion control and digested samples of raw peanuts by high-resolution tandem mass spectrometry. **A**—Excision map with protein/fragment identifications and their abundancies per excised band. SDS-PAGE was performed in reducing and non-reducing conditions on 14% gels. ct—control at 120 min; dg—digest at 120 min; PEPSIN—controls containing only pepsin at 0 and 120 min; M—molecular weight markers. **B**—Protein/fragment abundancies before and after pepsin digestion. **C**—Sequence alignment of Ara h 3 acidic subunit fragment homologs found at molecular weights of at least 40 kDa, showing nonspecific proteolysis with blue arrows and those unique to the digested sample (a result of the pepsin action) with red arrows.

Therefore, we have applied a pilot assessment using high-resolution tandem mass spectrometry and the PEAKS Suite X software’s post-translational modification search engine (PEAKS PTM) to assess the pepsin digestion efficacy of raw peanuts. This approach is, unlike the label-free in solution approach, only semi-quantitative and these results should be taken with caution. However, we have compared the amount of each protein or allergen to matched gel bands (Figure 7A). Moreover, the comparison of the detected peptide sequence profiles of the same protein and its fragments from the control and digested raw peanuts provided us with information on the specific sites of the pepsin action (Figure 7B).

The intact form of Ara h 1 (65 kDa) was partially digested and its digestion pattern is given in Figure 7A (band 15 and green letters). In contrast to Ara h 1, Ara h 3, which was also detected in this region in the control sample, was almost completely digested. We have obtained, also, that lipoxygenase (band 15), galactose-binding lectin (band 9), and 11S seed storage protein (band 8) were almost completely digested by pepsin (Figure 7A). These findings are in the agreement with the widely accepted consideration of these proteins as being susceptible to pepsin digestion.

Regarding the digestion fate of the major allergen Ara h 1, the accumulation of Ara h 1-derived fragments occurs upon pepsin action. More specifically, Ara h 1-derived fragments were detected in bands nine (approximately 29–33 kDa range) and six (approximately 22–24 kDa range) of the digested sample, while Ara h 1-derived fragments were completely absent in the control within the corresponding regions. In contrast to Ara h 1, which is resistant to pepsin digestion to a decent extent, Ara h 3 was almost completely digested by pepsin, even in the case of band six where it seems to be unaffected by pepsin (Figure 7A). In this band, we detected digestion fragments of the acidic Ara h 3 subunit and accumulation of other Ara h 3-derived fragments. Finally, band one (13–15 kDa range) of the digested sample shows an accumulation of small Ara h 3 peptides to a high extent. A detailed examination of Table S2 provides information on fragments accounting for the accumulation in certain gel bands in the digested sample, and fragments that were originally present in the specific mass range, by matching them to the proteins in the control sample band.

Additionally, we succeeded in identifying the pepsin proteolytic sites by comparing homologs N-terminal fragments of the acidic Ara h 3 subunit found in the 25–45 kDa region, and they included peptide bonds between the following amino acids: aspartic acid and asparagine, glycine and glycine, alanine and glutamine, and glutamine and glycine (Figure 7B). In addition, no difference in the PTM qualitative appearance between the control and digested preparation of raw peanuts was observed (Table S2).

## 4. Conclusions

Resistance to gastric digestion and limited proteolysis in the intestinal tract are characteristics of a typical food allergen. Moreover, the assessment of the digestibility of foods should be tested in the most realistic conditions possible; that is, in the presence of the food matrix, which is a prerequisite to properly judging a protein’s digestion stability. Additionally, analyzing the effects of thermal treatments on the extraction efficacy and digestibility of peanut proteins will provide information on processing conditions that may increase the allergenicity of peanuts. The lack of strict compliance with the in vitro digestion protocols and lack of detailed instructions for post-digestion defatting procedures enables unwanted diversification of data on peanut allergen gastrointestinal digestion stability/resistance. According to our study, a single, non-repetitive TCA/acetone defatting/protein precipitation protocol is proposed as a method of choice over h-hexane defatting. Inclusion of gastric lipase, as proposed already in the novel INFOGEST 2.0 protocol, might help in avoiding the defatting steps, which lead to the loss of the proteins.

The solubility of proteins and extraction efficacy, as observed in digested and control samples, is profoundly altered upon the thermal processing of peanuts. The thermal treatment of peanuts decreased the solubility and extraction efficacy of the peanut proteins in the acidic buffer of the simulated gastric juice.

The extraction of peanut proteins with higher molecular masses, Ara h 1 and Ara h 3 upon the thermal treatment, was impaired in simulated gastric juices, most probably due to aggregation.

The extraction of peanut proteins in intestinal conditions (i.e., controls of gastrointestinal digestion) had the opposite trend observed in the gastric phase; the proteins from raw peanuts were extracted to the lowest extent, while the extraction of proteins from roasted peanuts was favored in these conditions.

Thermal treatments of peanuts impaired the gastrointestinal digestion of the peanut proteins, especially in the case of roasted samples.

## Supplementary Materials

The following are available online at https://www.mdpi.com/2304-8158/8/10/463/s1, Figure S1: Representative example of the entire process of digitalization (pixelization) and volume quantification of the bands in electrophoretic profiles of TCA/acetone defatted gastric and gastrointestinal digests of raw and thermally processed peanuts by ImageQuantTL version 8.1 (GE Healthcare, Howard County, MA, USA), Figure S2: A. 14% SDS-PAGE profiles after TCA/acetone defatting of liquid phase upon gastric digestion of spinach, walnut, milk, and chicken breast preparations in reducing conditions; B. 14% SDS-PAGE profiles of TCA/Acetone defatted; and C. 14% SDS-PAGE profiles of non-defatted liquid phases upon 120’ - gastric digestion of raw hazelnut. Twenty microliter/samples were applied to the gels in both reducing and non/reducing conditions, Figure S3: Peanut proteins profiles leaking in a cooking water (water was 10 times concentrated) on 14 % SDS-PAGE under reducing conditions, stained with CBB R250. Five grams of raw peanut was boiled in 50 mL of water as already described in Materials and Methods (20 min boiling on 100 °C in a closed dish). Water was concentrated to one tenth of volume in two manners, with speed vac and by pelleting proteins with TCA/acetone method (as already described), Table S1: Effect of TCA/acetone and n-hexane defatting procedures on protein concentration in gastric and gastrointestinal digests (and corresponding controls) of whole-grain of thermally processed peanuts. Determined by BCA assay and Image Quant 1D TL 8.1 software, Table S2: Identification of proteins in raw peanut control and gastric digest protein bands by PEAKS X PTM algorithm.
