# Supplementary material for: Thermal Processing of Peanut Grains Impairs Their Mimicked Gastrointestinal Digestion While Downstream Defatting Treatments Affect Digestomic Profiles
Source: Foods. 2019 Oct 10;8(10):463. doi: 10.3390/foods8100463 (PMC6836028; doi:10.3390/foods8100463)
Supplement: Supplementary file 1 [file foods-08-00463-s001.zip › supplementary/supplementary.docx]

## Supplementary Material

Thermal Processing of Peanut Grains Impairs Their Mimicked Gastrointestinal Digestion While Downstream Defatting Treatments Affect Digestomic Profiles

Ivana Prodić^1#^, Katarina Smiljanić^2#^, Ana Simović^2^, Jelena Radosavljević^2^, Tanja Ćirković Veličković^2, 3, 4, 5*^

^1^ University of Belgrade–Faculty of Chemistry, Centre of Excellence for Molecular Food Sciences & Innovation Center Ltd, Belgrade, Serbia

^2^ University of Belgrade–Faculty of Chemistry, Centre of Excellence for Molecular Food Sciences & Department of Biochemistry, Belgrade, Serbia

^3^ Ghent University Global Campus, Incheon, South Korea

^4^ Ghent University, Faculty of Bioscience Engineering, Ghent, Belgium

^5^ Serbian Academy of Sciences and Arts, Belgrade, Serbia

^#^equally contributing

* Correspondence: [tcirkov@chem.bg.ac.rs](mailto:tcirkov@chem.bg.ac.rs); [Tanja.Velickovic@ghent.ac.kr](mailto:Tanja.Velickovic@ghent.ac.kr)

### Abbreviations

AEBSF – 4-(2-aminoethyl) benzenesulfonyl fluoride hydrochloride

1D – one dimensional

CBB – Coomassie Brilliant Blue

DTT- dithiothreitol

GIT – gastrointestinal tract

IAA- iodoacetamide

kDa – kilo Daltons

SDS–PAGE – sodium dodecyl sulphate polyacrylamide gel electrophoresis

SSF – simulated salivary fluid

SGF – simulated gastric fluid

SIF – simulated intestinal fluid

RT – room temperature (between 20°C and 25°C)

TCA – trichloroacetic acid

## Methods

### Materials

α-Amylase from human saliva (A0521-500 UN; Type IX-A, lyophilized powder 1000-3000 U/mg protein), porcine pepsin from gastric mucosa (P6887-1G, lyophilized powder 3200-4500 U/mg protein), pancreatin (P7545, lyophilized powder, 8 X USP) and porcine bile extract (B8631, lyophilized powder) were purchased from Sigma–Aldrich (Saint-Louis, MO, USA). The enzyme activities were measured according to the assays described by Minekus et al. [[1](#_ENREF_1)]. AEBSF- 4-(2-aminoethyl) benzenesulfonyl fluoride hydrochloride was purchased from Fluka, Sigma-Aldrich, Ref: 76307. All other chemicals were of analytical grade and purchased from Sigma-Aldrich (Saint-Louis, MO, USA) unless stated otherwise. In all experiments, ultra-pure deionized water (18 mΩ) was used (Smart2Pure3™ Barnstead aqua puriﬁcation system (Thermo Fisher Scientific, Waltham, MA, USA)).

### Preparation and processing of spinach, chicken breast, walnut, hazelnut and milk before pepsin digestion

Raw spinach (*Spinacia oleracea* L.), raw common walnut (*Juglans regia* L.), hazelnut (*Corylus avellane* L.) and chicken breasts were purchased from a local grocery. Chicken breasts were cooked for 30 minutes at 75 °C [[2](#_ENREF_2)]. All preparations were milled using a coffee grinder (800 W, Bosh), 3 times for 5 minutes to reach particle size of the material < 1.5 mm. Pasteurized milk (3.2% percent of fat) from a local grocery was used (not mechanically pre-treated).

### Simulated oral, gastric and intestinal in vitro digestion conditions

Oral Phase

Raw spinach and boiled chicken breast (0.2 g) were mixed with 100 μL of 2×stock solution of simulated salivary fluid (SSF), separately. Pasteurized milk (0.2 mL) was mixed with 100 μL of 2×stock solution of simulated salivary fluid (SSF). In all four preparations, human salivary α-amylase (20 μL, 1500 U/mL in water) was added to achieve the final concentration of 75 U/mL in the digestion mixture, followed by addition of CaCl_2_ (20 μL, 15 mM) to obtain the final 0.75 mM concentration. Water was added to reach 200 μL of 1× SSF concentration. The reaction mixture was incubated for 2 minutes at 37 °C with agitation. All reagents were pre-warmed at 37 °C for 15 minutes.

Gastric phase

Complete oral phase material was mixed with 0.4 mL of simulated gastric fluid (SGF) stock solution (1× concentrated SGF) and 20 μL of CaCl_2_ (3 mM in 1× SGF) to achieve a final concentration of 75 µM in the digestion mixture. Porcine pepsin (356 μL; 4500 U/mL in 1× SGF) was added, to achieve the final concentration of 2000 U/mL in the digestion mixture. The mixture was adjusted to pH 3.0 ± 0.2 with 1 M HCl, and 1xSGF was added to the volume of 0.8 mL of whole digestion mixture. The reaction mixture was incubated for 120 minutes at 37 °C with intense agitation (600 rpm). Control samples were run in parallel: control for each food (oral bolus without amylase with the addition of 356 μL 1× SGF instead of pepsin solution) at 120` (Ct120), and pepsin control (with 0.2 mL of 1× SSF stock solution and 0.2 g of sand instead of oral bolus at 0 min (Dg-pepsin 0’) and 120 min (Dg-pepsin 120’)). Digestion was stopped by addition of 200 μL 2 M NaHCO_3_ to achieve the final pH of the reaction mixture of 8.0.

Intestinal phase

Complete gastric chyme of 0.8 mL after 120 minutes of digestion was mixed with pancreatic suspension prepared in 400 μL of 1 × concentrated simulated intestinal fluid (SIF), with 400 U/mL of trypsin activity in the fluid (100 U/mL in the final digestion mixture). The bile solution (61 μL; 263 mM) and CaCl_2_ (50 μL, 20mM) were added to reach the final concentration of 10 mM and 0.3 mM in the digestion mixture, respectively. Necessary volume of 1 M NaOH (100 μL) was added to adjust the pH to 7.0. Finally, 189 μL of deionized water was added to adjust the volume of the mixture so the 1× SIF concentration is achieved. Samples were incubated on a rotating wheel for 2 h, at 37 °C. Digestion was stopped by addition of 50 μL AEBSF (32 mM) to obtain the final concentration of 1 mM in the gastrointestinal mixture.

### Post gastric or post gastrointestinal defatting treatment

The samples after the gastric phase digestion were centrifuged at 10,000 g for 20 minutes, and the liquid phase was separated. The liquid phase (200 μL) was mixed with cold 20% trichloroacetic acid (TCA)/acetone (1:1, v/v) and left overnight at -20 °C. After removing the supernatant by centrifugation at 13,000 g for 30 minutes, the pelleted proteins were washed twice with 1 mL of cold acetone, intensively vortexed and centrifuged at 13,000 g for 15 minutes. Pellet was dried for 1 h at RT (room temperature) and resuspended in corresponding volume of 1.5× Laemmli sample buffer. Ten microliters of each sample were applied on 14% SDS PAGE gel (Figure S2).

## Supplementary Figures and Tables


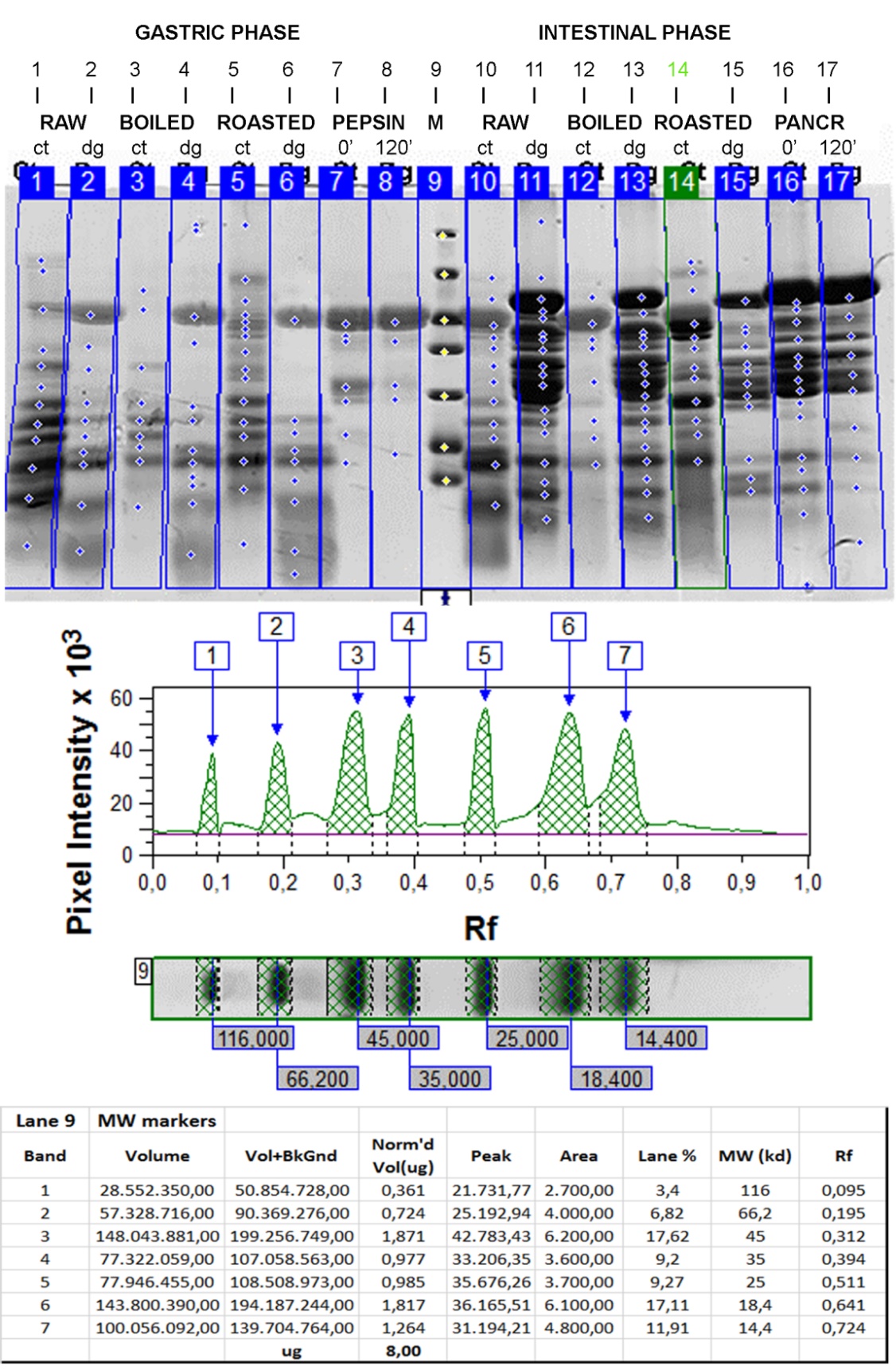


**Figure S1.** Representative example of the entire process of digitalization (pixelization) and volume quantification of the bands in electrophoretic profiles of TCA/acetone defatted gastric and gastrointestinal digests of raw and thermally processed peanuts by ImageQuantTL version 8.1 (GE Healthcare, USA).





**Figure S2.** A. 14% SDS-PAGE profiles after TCA/acetone defatting of liquid phase upon gastric digestion of spinach, walnut, milk, and chicken breast preparations in reducing conditions; B. 14% SDS-PAGE profiles of TCA/Acetone defatted; and C. 14% SDS-PAGE profiles of non-defatted liquid phases upon 120’ - gastric digestion of raw hazelnut. Twenty microliter/samples were applied to the gels in both reducing and non/reducing conditions. Ct - control at 120 minutes; Dg - digest at 120 minutes; Pepsin 0’ and 120’- enzyme controls at 0 and 120 minutes, respectively, M - molecular weight markers.


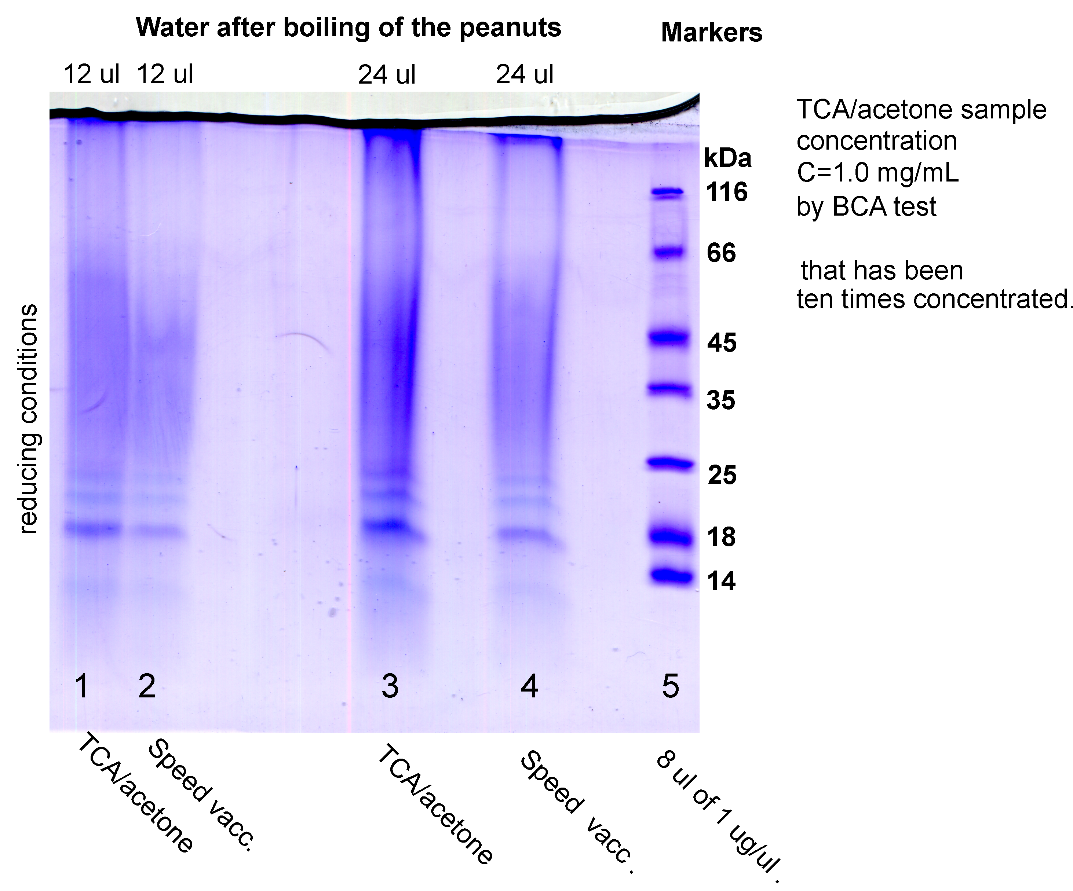


**Figure S3.** Peanut cooking water (10 times concentrated) profiles on 14 % SDS-PAGE under reducing conditions, stained with CBB R250. Five grams of raw peanut was boiled in 50 ml of water as already described in Materials and Methods (20 minutes boiling on 100 °C in a closed dish). Water was concentrated to one tenth of volume in two manners, with speed vac and by pelleting proteins with TCA/acetone method (as already described).

**Table S1.** Effect of TCA/acetone and n-hexane defatting procedures on protein concentration in gastric and gastrointestinal digests (and corresponding controls) of whole-grain of thermally processed peanuts. Determined by BCA assay and Image Quant 1D TL 8.1 software.

## References:

1. Minekus, M.; Alminger, M.; Alvito, P.; Ballance, S.; Bohn, T.; Bourlieu, C.; Carriere, F.; Boutrou, R.; Corredig, M.; Dupont, D., et al. A standardised static in vitro digestion method suitable for food - an international consensus. Food Funct 2014, 5, 1113-1124, doi:10.1039/c3fo60702j.

2. Wen, S.; Zhou, G.; Song, S.; Xu, X.; Voglmeir, J.; Liu, L.; Zhao, F.; Li, M.; Li, L.; Yu, X., et al. Discrimination of in vitro and in vivo digestion products of meat proteins from pork, beef, chicken, and fish. PROTEOMICS 2015, 15, 3688-3698, doi:10.1002/pmic.201500179.

3. Candiano, G.; Bruschi, M.; Musante, L.; Santucci, L.; Ghiggeri, G.M.; Carnemolla, B.; Orecchia, P.; Zardi, L.; Righetti, P.G. Blue silver: A very sensitive colloidal Coomassie G-250 staining for proteome analysis. ELECTROPHORESIS 2004, 25, 1327-1333, doi:10.1002/elps.200305844.
