# Supplementary material for: Thermal Processing of Peanut Grains Impairs Their Mimicked Gastrointestinal Digestion While Downstream Defatting Treatments Affect Digestomic Profiles
Source: Foods. 2019 Oct 10;8(10):463. doi: 10.3390/foods8100463 (PMC6836028; doi:10.3390/foods8100463)
Supplement: Supplementary file 1 [file foods-08-00463-s001.zip › supplementary/Table S2 full.pdf]

Table S2- Identification of proteins in raw peanut control and gastric digest protein bands by PEAKS X PTM algorithm

Band #1 13-15 kDa of SDSPAGE resolved Gastric CONTROL on 120'

Protein Group	Protein ID	Accession	Description	Allergome	Avg, Mass	-10lgP	Coverage (%)	Area	#Peptides	#Unique	#Spec counts
1	20574	A1DZF0	Arachin 6 OS=Arachis hypogaea (	Ara h 3	60375	140,52	8	3,46E+05	6	6	39
1	20576	Q9FZ11	Gly1 OS=Arachis hypogaea OX=3	Ara h 3	60449	140,52	8	3,46E+05	6	6	39
1	20578	B5TYU1	Arachin Arah3 isoform OS=Arach	Ara h 3	60624	140,52	8	3,46E+05	6	6	39
1	20579	Q5I6T2	Arachin Ahy-4 OS=Arachis hypog	Ara h 3	60736	140,52	8	3,46E+05	6	6	39
1	20580	Q647H4	Arachin Ahy-1 OS=Arachis hypog	Ara h 3	61506	140,52	7	3,46E+05	6	6	39
1	20581	Q647H3	Arachin Ahy-2 OS=Arachis hypog	Ara h 3	61532	140,52	7	3,46E+05	6	6	39
1	20582	Q8LKN1	Allergen Arah3/Arah4 OS=Arachi	Ara h 3	61738	140,52	7	3,46E+05	6	6	39
1	20577	Q9SQH7	Glycinin OS=Arachis hypogaea O	Ara h 3	61011	140,52	8	3,46E+05	6	6	39
1	20575	Q8LL03	Trypsin inhibitor (Fragment) OS=.	Ara h 3	25499	140,52	18	3,46E+05	6	6	39
								**3,11E+06**			

Band #1 13-15 kDa of SDSPAGE resolved Gastric DIGESTION on 120' matched to Band *#1 in control*

Protein Group	Protein ID	Accession	Description	Allergome	Avg, Mass	-10lgP	Coverage (%)	Area	#Peptides	#Unique	#Spec
1	20574	A1DZF0	Arachin 6 OS=Arachis hypogaea (	Ara h 3	60375	203,54	18	1,05E+07	13	12	235
1	20578	B5TYU1	Arachin Arah3 isoform OS=Arach	Ara h 3	60624	203,54	18	1,05E+07	13	12	235
1	20577	Q9SQH7	Glycinin OS=Arachis hypogaea O	Ara h 3	61011	203,54	18	1,05E+07	13	12	235
2	20593	Q6IWG5	Glycinin (Fragment) OS=Arachis h	Ara h 3	58061	129,76	19	6,63E+06	10	9	77
2	20595	E5G077	Ara h 3 allergen OS=Arachis hypo	Ara h 3	58305	129,76	19	6,63E+06	10	9	77
2	20594	Q0GM57	Iso-Ara h3 OS=Arachis hypogaea	Ara h 3	58263	129,76	19	6,63E+06	10	9	77
								**5,14E+07**			

Band #2 15-17 kDa of SDSPAGE resolved Gastric CONTROL on 120'

No identification

Band #2 15-17 kDa of SDSPAGE resolved gastric DIGESTION on 120' matched to Band #2 in control

Protein Group	Protein ID	Accession	Description	Allergome	Avg, Mass	-10lgP	Coverage (%)	Area	#Peptides	#Unique	#Spec
1	20574	A1DZF0	Arachin 6 OS=Arachis hypogaea (	Ara h 3	60375	108,77	20	1,03E+05	6	6	17
3	20593	Q6IWG5	Glycinin (Fragment) OS=Arachis h	Ara h 3	58061	33,67	7	0,00E+00	1	1	2
3	20595	E5G077	Ara h 3 allergen OS=Arachis hypo	Ara h 3	58305	33,67	7	0,00E+00	1	1	2
3	20594	Q0GM57	Iso-Ara h3 OS=Arachis hypogaea	Ara h 3	58263	33,67	7	0,00E+00	1	1	2
								**1,03E+05**			

Band #3 17-20 kDa of SDSPAGE resolved Gastric CONTROL on 120'

Protein Group	Protein ID	Accession	Description?	Allergome	Avg, Mass	-10lgP	Coverage (%)	Area	#Peptides	#Unique	#Spec
2	20879	A0A290FZ Y6	Resistance protein (Fragment) OS=Arachis hypogaea	n/a	87223	29,52	1	2,64E+04	1	1	1

Band #3 17-18 kDa of SDSPAGE resolved Gastric DIGESTION on 120' matched to Band #3 in control

Protein Group	Protein ID	Accession	Description?	Allergome	Avg, Mass	-10lgP	Coverage (%)	Area	#Peptides	#Unique	#Spec
3	22696	E9LFE7	7S conarachin (Fragment) OS=Ar	Ara h 1	15873	47,17	9	3,05E+03	1	1	1
3	22695	Q6PSU5	Conarachin (Fragment) OS=Aracl	Ara h 1	33604	47,17	4	3,05E+03	1	1	1
3	21739	Q6PSU6	Conarachin (Fragment) OS=Aracl	Ara h 1	34133	47,17	4	3,05E+03	1	1	1
3	22400	Q6PSU4	Conarachin (Fragment) OS=Aracl	Ara h 1	48095	47,17	3	3,05E+03	1	1	1
3	20849	Q6PSU3	Conarachin (Fragment) OS=Aracl	Ara h 1	66575	47,17	2	3,05E+03	1	1	1
3	20850	P43237	Allergen Ara h 1 clone P17 OS=A	Ara h 1	70283	47,17	2	3,05E+03	1	1	1
3	20851	B3IXL2	Main allergen Ara h1 OS=Arachis	Ara h 1	70283	47,17	2	3,05E+03	1	1	1
3	20852	E5G076	Ara h 1 allergen OS=Arachis hypc	Ara h 1	70788	47,17	2	3,05E+03	1	1	1
3	20853	N1NG13	Seed storage protein Ara h1 OS=	Ara h 1	71345	47,17	2	3,05E+03	1	1	1
3	20854	P43238	Allergen Ara h 1 clone P41B OS=	Ara h 1	71345	47,17	2	3,05E+03	1	1	1
								**3,05E+04**			

Band #4 21-22 kDaof SDSPAGE resolved Gastric CONTROL on 120'

Protein Group	Protein ID	Accession	Description	Allergome	Avg, Mass	-10lgP	Coverage (%)	Area	#Peptides	#Unique	#Spec
7	21031	Q9M5D3	Lipoxygenase OS=Arachis hypoga	n/a	97616	84,68	5	7,73E+03	3	3	3
9	20574	A1DZF0	Arachin 6 OS=Arachis hypogaea	Ara h 3	60375	72,5	2	6,09E+03	1	1	2
9	20576	Q9FZ11	Gly1 OS=Arachis hypogaea OX=3	Ara h 3	60449	72,5	2	6,09E+03	1	1	2
9	20578	B5TYU1	Arachin Arah3 isoform OS=Arach	Ara h 3	60624	72,5	2	6,09E+03	1	1	2
9	20577	Q9SQH7	Glycinin OS=Arachis hypogaea O	Ara h 3	61011	72,5	2	6,09E+03	1	1	2
9	20579	Q5I6T2	Arachin Ahy-4 OS=Arachis hypog	Ara h 3	60736	72,5	2	6,09E+03	1	1	2
9	20580	Q647H4	Arachin Ahy-1 OS=Arachis hypog	Ara h 3	61506	72,5	2	6,09E+03	1	1	2
9	20591	Q6T2T4	Storage protein OS=Arachis hypo	Ara h 3	61499	72,5	2	6,09E+03	1	1	2
9	20581	Q647H3	Arachin Ahy-2 OS=Arachis hypog	Ara h 3	61532	72,5	2	6,09E+03	1	1	2
9	20582	Q8LKN1	Allergen Arah3/Arah4 OS=Arachi	Ara h 3	61738	72,5	2	6,09E+03	1	1	2
4	21037	E9LFE8	11S arachin (Fragment) OS=Arac	n/a	28290	94,35	15	5,44E+03	2	2	4
7	21032	Q4JME6	Lipoxygenase OS=Arachis hypoga	n/a	97476	77,44	4	4,88E+03	2	2	2
7	21033	Q4JME7	Lipoxygenase OS=Arachis hypoga	n/a	97596	77,44	4	4,88E+03	2	2	2
11	21034	Q38711	Galactose-binding lectin (Fragme	n/a	29134	53,52	5	1,59E+03	1	1	1
11	21036	A0A089ZXI	Peanut agglutinin variant OS=Ara	n/a	29407	53,52	5	1,59E+03	1	1	1
11	21035	P02872	Galactose-binding lectin OS=Ara	n/a	29325	53,52	5	1,59E+03	1	1	1
10	20593	Q6IWG5	Glycinin (Fragment) OS=Arachis h	Ara h 3	58061	31,78	5	1,33E+03	1	1	2
10	20595	E5G077	Ara h 3 allergen OS=Arachis hypo	Ara h 3	58305	31,78	5	1,33E+03	1	1	2
10	20594	Q0GM57	Iso-Ara h3 OS=Arachis hypogaea	Ara h 3	58263	31,78	5	1,33E+03	1	1	2
							total	**8,65E+04**			
							Ara h 3	5,88E+04			

Band #4 18-20 kDa of SDSPAGE resolved Gastric DIGESTSION on 120' matched to Band #4 in control

No identification

10	20595	E5G077	Ara h 3 allergen OS=Arachis hypc	Ara h 3	58305	31,78	5	1,33E+03	1	1	2
10	20594	Q0GM57	Iso-Ara h3 OS=Arachis hypogaea	Ara h 3	58263	31,78	5	1,33E+03	1	1	2
							total	**8,65E+04**			
							Ara h 3	5,88E+04			

Band #4 18-20 kDa of SDSPAGE resolved Gastric DIGESTSION on 120' matched to Band #4 in control

No identification

Band #5 20-22 kDa of SDSPAGE resolved Gastric CONTROL on 120'

Protein Group	Protein ID	Accession	Description	Allergome	Avg, Mass	-10lgP	Coverage (%)	Area	#Peptides	#Unique	#Spec
3	21693	A0A290G0J	Resistance protein (Fragment) OS=Arachis hy		57776	22,10	4	3,00E+03	1	1	1
4	21619	A0A290GKJ	Resistance protein (Fragment) OS=Arachis hy		54561	20,35	2	2,50E+03	1	1	1
								**5,50E+03**			

Band #5 20-22 kDa of SDSPAGE resolved Gastric DIGESTSION on 120' matched to band #5 in control

Protein Group	Protein ID	Accession	Description	Allergome	Avg, Mass	-10lgP	Coverage (%)	Area	#Peptides	#Unique	#Spec
6	20593	Q6IWG5	Glycinin (Fragment) OS=Arachis h	Ara h 3	58061	269,16	35	3,67E+07	27	25	176
6	20594	Q0GM57	Iso-Ara h3 OS=Arachis hypogaea	Ara h 3	58263	269,16	35	3,67E+07	27	25	176
8	20582	Q8LKN1	Allergen Arah3/Arah4 OS=Arachi	Ara h 3	61738	282,23	28	7,49E+06	27	2	140
2	20581	Q647H3	Arachin Ahy-2 OS=Arachis hypog	Ara h 3	61532	309,17	34	5,51E+06	29	4	249
1	20574	A1DZF0	Arachin 6 OS=Arachis hypogaea	Ara h 3	60375	305,89	34	3,92E+06	32	2	259
7	20578	B5TYU1	Arachin Arah3 isoform OS=Arach	Ara h 3	60624	300,00	31	2,11E+06	26	2	217
5	20576	Q9FZ11	Gly1 OS=Arachis hypogaea OX=3	Ara h 3	60449	293,74	28	1,91E+05	24	1	216
9	20590	O82580	Glycinin (Fragment) OS=Arachis h	Ara h 3	58350	229,09	24	0,00E+00	14	1	50
								**9,26E+07**			

Band #6 22-24 kDa of SDSPAGE resolved Gastric CONTROL on 120'

Protein Group	Protein ID		Description	Allergome	Avg, Mass	-10lgP	Coverage (%)	Area Control 6	#Peptides	#Unique	#Spec
9	20593	Q6IWG5	Glycinin (Fragment) OS=Arachis h	Ara h 3	58061	258,08	31	1,34E+07	20	15	198
9	20594	Q0GM57	Iso-Ara h3 OS=Arachis hypogaea	Ara h 3	58263	258,08	31	1,34E+07	20	15	198
3	20581	Q647H3	Arachin Ahy-2 OS=Arachis hypog	Ara h 3	61532	301,28	34	7,31E+06	32	4	403
4	20578	B5TYU1	Arachin Arah3 isoform OS=Arach	Ara h 3	60624	293,74	32	1,01E+06	30	3	400
10	20590	O82580	Glycinin (Fragment) OS=Arachis h	Ara h 3	58350	251,87	22	6,69E+05	20	3	159
6	20582	Q8LKN1	Allergen Arah3/Arah4 OS=Arachi	Ara h 3	61738	277,56	31	3,61E+05	30	2	366
7	20576	Q9FZ11	Gly1 OS=Arachis hypogaea OX=3	Ara h 3	60449	286,21	26	2,20E+05	25	2	367
11	20968	HY3_ARAH	Arachin Ahy-3 OS=Arachis hypog	Ara h 3	54569	130,71	7	2,17E+05	4	2	12
19	21915	J9QGM3	Chalcone-flavonone isomerase f	n/a	27626	27,75	11	5,78E+03	1	1	1
							total	**3,66E+07**			
							Ara h 3	3,66E+07			

Band #6 22-24 kDa of SDSPAGE resolved Gastric DIGESTSION on 120' matched to Band #6 in control

Protein Group	Protein ID	Accession	Description	Allergome	Avg, Mass	-10lgP	Coverage (%)	Area	#Peptides	#Unique	#Spec
3	20581	Q647H3	Arachin Ahy-2 OS=Arachis hypog	Ara h 3	61532	235,17	34	1,63E+05	18	3	123
6	20578	B5TYU1	Arachin Arah3 isoform OS=Arach	Ara h 3	60624	231,54	32	1,17E+03	16	1	94
5	20582	Q8LKN1	Allergen Arah3/Arah4 OS=Arachi	Ara h 3	61738	214,35	34	3,57E+05	15	2	93
8	20590	O82580	Glycinin (Fragment) OS=Arachis h	Ara h 3	58350	182,12	23	3,67E+05	13	4	34
7	20593	Q6IWG5	Glycinin (Fragment) OS=Arachis h	Ara h 3	58061	172,18	24	2,29E+06	8	7	83
7	20594	Q0GM57	Iso-Ara h3 OS=Arachis hypogaea	Ara h 3	58263	172,18	23	2,29E+06	8	7	83
9	22400	Q6PSU4	Conarachin (Fragment) OS=Aracl	Ara h 1	48095	96,82	17	1,24E+05	5	5	8
9	20853	N1NG13	Seed storage protein Ara h1 OS=	Ara h 1	71345	96,82	12	1,24E+05	5	5	8
9	20854	P43238	Allergen Ara h 1 clone P41B OS=	Ara h 1	71345	96,82	12	1,24E+05	5	5	8
							total	**5,84E+06**			
							Ara h 1	3,72E+05			
							Ara h 3	5,47E+06			

Band #7 of 24-27 kDa SDSPAGE resolved Gastric CONTROL on 120'

Protein Group	Protein ID	Accession	Description	Allergome	Avg, Mass	-10lgP	Coverage (%)	Area	#Peptides	#Unique	#Spec
2	20576	Q9FZ11	Gly1 OS=Arachis hypogaea OX=3	Ara h 3	60449	8	8	3,77E+03	3	1	9
2	20579	Q5I6T2	Arachin Ahy-4 OS=Arachis hypog	Ara h 3	60736	8	8	3,77E+03	3	1	9
2	20581	Q647H3	Arachin Ahy-2 OS=Arachis hypog	Ara h 3	61532	8	8	3,77E+03	3	1	9
1	20574	A1DZF0	Arachin 6 OS=Arachis hypogaea	Ara h 3	60375	8	8	2,24E+03	3	1	11
1	20578	B5TYU1	Arachin Arah3 isoform OS=Arach	Ara h 3	60624	8	8	2,24E+03	3	1	11
1	20580	Q647H4	Arachin Ahy-1 OS=Arachis hypog	Ara h 3	61506	8	8	2,24E+03	3	1	11
1	20591	Q6T2T4	Storage protein OS=Arachis hypc	Ara h 3	61499	8	8	2,24E+03	3	1	11
								**2,03E+04**			

Band #7 24-27 kDa of SDSPAGE resolved Gastric DIGESTSION on 120' matched to Band #7 in control

Protein Group	Protein ID	Accession	Description	Allergome	Avg, Mass	-10lgP	Coverage (%)	Area	#Peptides	#Unique	#Spec
6	20593	Q6IWG5	Glycinin (Fragment) OS=Arachis h	Ara h 3	58061	95	18	3,06E+05	5	3	15
6	20594	Q0GM57	Iso-Ara h3 OS=Arachis hypogaea	Ara h 3	58263	95	18	3,06E+05	5	3	15
6	20595	E5G077	Ara h 3 allergen OS=Arachis hypc	Ara h 3	58305	95	18	3,06E+05	5	3	15
1	20581	Q647H3	Arachin Ahy-2 OS=Arachis hypog	Ara h 3	61532	285,21	51	2,93E+05	30	5	232
3	20580	Q647H4	Arachin Ahy-1 OS=Arachis hypog	Ara h 3	61506	278,6	42	1,17E+05	28	2	214
12	20968	Q647H2	Arachin Ahy-3 OS=Arachis hypog	3 (alpha ch	54569	23,43	7	2,36E+04	1	1	1
14	25842	0A109QJM	Long chain acyl-CoA synthetase	n/a	74266	27,33	2	6,54E+03	1	1	1
4	20574	A1DZF0	Arachin 6 OS=Arachis hypogaea	Ara h 3	60375	275,05	42	5,84E+03	28	2	206
13	21034	Q38711	Galactose-binding lectin (Fragme	n/a	29134	56,3	5	5,67E+03	1	1	1
13	21036	A0A089ZXL	Peanut agglutinin variant OS=Ara	n/a	29407	56,3	5	5,67E+03	1	1	1
13	21035	P02872	Galactose-binding lectin OS=Ara	n/a	29325	56,3	5	5,67E+03	1	1	1
15	22368	A1E2B0	11S seed storage globulin B1 OS=	n/a	33520	24,77	4	5,41E+03	1	1	1
5	20590	O82580	Glycinin (Fragment) OS=Arachis h	Ara h 3	58350	217,48	25	2,50E+03	16	1	101
							total	**1,39E+06**			
							Ara h 3	1,36E+06			

Band #8 27-29 kDa of SDSPAGE resolved Gastric CONTROL on 120'

Band #8 27-29 kDa of SDSPAGE resolved Gastric CONTROL on 120'

Protein Group	Protein ID	Accession	Description	Allergome	Avg, Mass	-10lgP	Coverage (%)	Area	#Peptides	#Unique	#Spec
1	20574	A1DZF0	Arachin 6 OS=Arachis hypogaea (	Ara h 3	60375	194,95	35	4,03E+05	11	7	67
6	21689	Q2KQ45	Resistance protein PLTR (Fragme	n/a	19632	22,16	7	2,03E+05	1	1	1
2	20581	Q647H3	Arachin Ahy-2 OS=Arachis hypog	Ara h 3	61532	181,57	29	6,87E+04	10	3	32
4	20593	Q6IWG5	Glycinin (Fragment) OS=Arachis h	Ara h 3	58061	43,35	11	3,29E+04	2	2	7
4	20594	Q0GM57	Iso-Ara h3 OS=Arachis hypogaea	Ara h 3	58263	43,35	11	3,29E+04	2	2	7
5	22368	A1E2B0	11S seed storage globulin B1 OS=	n/a	33520	25,85	4	1,52E+04	1	1	1
3	20579	Q5I6T2	Arachin Ahy-4 OS=Arachis hypog	Ara h 3	60736	174,98	29	0,00E+00	9	2	25
							total	**7,56E+05**			
							Ara h 3	5,38E+05			

Band #8 27-29 kDa of SDSPAGE resolved Gastric DIGESTSION on 120' matched to Band #8

Protein Group	Protein ID	Accession	Description	Allergome	Avg, Mass	-10lgP	Coverage (%)	Area	#Peptides	#Unique	#Spec
1	20579	Q5I6T2	Arachin Ahy-4 OS=Arachis hypog	Ara h 3	60736	93,44	5	1,08E+05	1	1	23
1	20576	Q9FZ11	Gly1 OS=Arachis hypogaea OX=3	Ara h 3	60449	93,44	5	1,08E+05	1	1	23
1	20581	Q647H3	Arachin Ahy-2 OS=Arachis hypog	Ara h 3	61532	93,44	4	1,08E+05	1	1	23
5	20834	\0A1B3TNS	NBS-LRR type disease resistance	n/a	108583	21,13	1	3,40E+04	1	1	1
3	20593	Q6IWG5	Glycinin (Fragment) OS=Arachis h	Ara h 3	58061	38,36	8	3,39E+04	2	2	4
3	20595	E5G077	Ara h 3 allergen OS=Arachis hypo	Ara h 3	58305	38,36	8	3,39E+04	2	2	4
3	20594	Q0GM57	Iso-Ara h3 OS=Arachis hypogaea	Ara h 3	58263	38,36	8	3,39E+04	2	2	4
2	20578	B5TYU1	Arachin Arah3 isoform OS=Arach	Ara h 3	60624	80,27	5	2,33E+04	1	1	13
2	20574	A1DZF0	Arachin 6 OS=Arachis hypogaea (	Ara h 3	60375	80,27	5	2,33E+04	1	1	13
2	20580	Q647H4	Arachin Ahy-1 OS=Arachis hypog	Ara h 3	61506	80,27	4	2,33E+04	1	1	13
2	20591	Q6T2T4	Storage protein OS=Arachis hypo	Ara h 3	61499	80,27	4	2,33E+04	1	1	13
							total	**5,53E+05**			
							Ara h 3	5,19E+05			

Band #9 29-33 kDa of SDSPAGE resolved Gastric CONTROL on 120'

Protein Group	Protein ID	Accession	Description	Allergome	Avg, Mass	-10lgP	Coverage (%)	Area	#Peptides	#Unique	#Spec
1	21035	P02872	Galactose-binding lectin OS=Ara	n/a	29325	215,68	70	5,53E+05	19	18	71
1	21034	Q38711	Galactose-binding lectin (Fragme	n/a	29134	215,68	70	5,53E+05	19	18	71
2	21349	Q43373	Galactose-binding lectin OS=Ara	n/a	29566	96,69	14	1,80E+04	3	2	13
10	20593	Q6IWG5	Glycinin (Fragment) OS=Arachis h	Ara h 3	58061	36,74	5	8,49E+03	1	1	2
10	20595	E5G077	Ara h 3 allergen OS=Arachis hypo	Ara h 3	58305	36,74	5	8,49E+03	1	1	2
10	20594	Q0GM57	Iso-Ara h3 OS=Arachis hypogaea	Ara h 3	58263	36,74	5	8,49E+03	1	1	2
7	20591	Q6T2T4	Storage protein OS=Arachis hypo	Ara h 3	61499	87,78	4	0,00E+00	2	1	2
7	20574	A1DZF0	Arachin 6 OS=Arachis hypogaea	Ara h 3	60375	87,78	5	0,00E+00	2	1	2
7	20578	B5TYU1	Arachin Arah3 isoform OS=Arach	Ara h 3	60624	87,78	5	0,00E+00	2	1	2
7	20580	Q647H4	Arachin Ahy-1 OS=Arachis hypog	Ara h 3	61506	87,78	4	0,00E+00	2	1	2
8	20576	Q9FZ11	Gly1 OS=Arachis hypogaea OX=3	Ara h 3	60449	80,32	5	0,00E+00	2	1	2
8	20581	Q647H3	Arachin Ahy-2 OS=Arachis hypog	Ara h 3	61532	80,32	4	0,00E+00	2	1	2
8	20579	Q5I6T2	Arachin Ahy-4 OS=Arachis hypog	Ara h 3	60736	80,32	5	0,00E+00	2	1	2
							total	**1,15E+06**			
							Galactose	**1,12E+06**			
							Ara h 3	2,55E+04			

Band #9 29-33 kDa of SDSPAGE resolved Gastric DIGESTSION on 120' matched with Band #9 in control

Protein Group	Protein ID	Accession	Description	Allergome	Avg, Mass	-10lgP	Coverage (%)	Coverage (%) Digest 9,	Area	#Peptides	#Unique
1	20853	N1NG13	Seed storage protein Ara h1 OS=	Ara h 1	71345	114,12	5	5	2,11E+05	4	4
1	20854	P43238	Allergen Ara h 1 clone P41B OS=	Ara h 1	71345	114,12	5	5	2,11E+05	4	4
1	22400	Q6PSU4	Conarachin (Fragment) OS=Aracl	Ara h 1	48095	114,12	7	7	2,11E+05	4	4
1	20849	Q6PSU3	Conarachin (Fragment) OS=Aracl	Ara h 1	66575	114,12	6	6	2,11E+05	4	4
1	20850	P43237	Allergen Ara h 1 clone P17 OS=A	Ara h 1	70283	114,12	5	5	2,11E+05	4	4
1	20851	B3IXL2	Main allergen Ara h1 OS=Arachis	Ara h 1	70283	114,12	5	5	2,11E+05	4	4
1	21739	Q6PSU6	Conarachin (Fragment) OS=Aracl	Ara h 1	34133	114,12	11	11	2,11E+05	4	4
1	22695	Q6PSU5	Conarachin (Fragment) OS=Aracl	Ara h 1	33604	114,12	11	11	2,11E+05	4	4
3	20593	Q6IWG5	Glycinin (Fragment) OS=Arachis h	Ara h 3	58061	106,08	10	10	1,80E+05	3	3
3	20594	Q0GM57	Iso-Ara h3 OS=Arachis hypogaea	Ara h 3	58263	106,08	10	10	1,80E+05	3	3
3	20595	E5G077	Ara h 3 allergen OS=Arachis hypc	Ara h 3	58305	106,08	10	10	1,80E+05	3	3
2	20591	Q6T2T4	Storage protein OS=Arachis hypc	Ara h 3	61499	107,8	4	4	3,12E+04	2	1
2	20580	Q647H4	Arachin Ahy-1 OS=Arachis hypog	Ara h 3	61506	107,8	4	4	3,12E+04	2	1
2	20574	A1DZF0	Arachin 6 OS=Arachis hypogaea (	Ara h 3	60375	107,8	5	5	3,12E+04	2	1
2	20578	B5TYU1	Arachin Arah3 isoform OS=Arach	Ara h 3	60624	107,8	5	5	3,12E+04	2	1
8	21034	Q38711	Galactose-binding lectin (Fragme	n/a	29134	95,54	9	9	1,42E+04	2	2
8	21035	P02872	Galactose-binding lectin OS=Ara	n/a	29325	95,54	9	9	1,42E+04	2	2
4	20579	Q5I6T2	Arachin Ahy-4 OS=Arachis hypog	Ara h 3	60736	107,12	5	5	1,12E+04	2	1
4	20576	Q9FZ11	Gly1 OS=Arachis hypogaea OX=3	Ara h 3	60449	107,12	5	5	1,12E+04	2	1
4	20581	Q647H3	Arachin Ahy-2 OS=Arachis hypog	Ara h 3	61532	107,12	4	4	1,12E+04	2	1
								total	**2,41E+06**		
								Ara h1	**1,69E+06**		
								Galactose	**2,54E+04**		
								Ara h 3	6,98E+05		

Band #10 33-38 kDa of SDSPAGE resolved Gastric CONTROL on 120'

No detection

Band #10 35-38 kDa of SDSPAGE resolved Gastric DIGESTSION on 120' matched to Band #10 in control

No detection

Band A 33-35 kDa of SDSPAGE resolved Gastric DIGESTION on 120' matched with Band #10 in control

Protein Group	Protein ID	Accession	Description	Allergome	Avg, Mass	-10lgP	Coverage (%)	Area	#Peptides	#Unique	#Spec
1	20574	A1DZF0	Arachin 6 OS=Arachis hypogaea (	Ara h 3	60375	118,86	12	2,14E+04	4	3	9
1	20580	Q647H4	Arachin Ahy-1 OS=Arachis hypog	Ara h 3	61506	118,86	11	2,14E+04	4	3	9
1	20591	Q6T2T4	Storage protein OS=Arachis hypc	Ara h 3	61499	118,86	11	2,14E+04	4	3	9
4	20576	Q9FZ11	Gly1 OS=Arachis hypogaea OX=3	Ara h 3	60449	93,24	5	5,49E+03	2	1	3
4	20581	Q647H3	Arachin Ahy-2 OS=Arachis hypog	Ara h 3	61532	93,24	4	5,49E+03	2	1	3
4	20579	Q5I6T2	Arachin Ahy-4 OS=Arachis hypog	Ara h 3	60736	93,24	5	5,49E+03	2	1	3
3	20593	Q6IWG5	Glycinin (Fragment) OS=Arachis I	Ara h 3	58061	36,32	5	9,07E+04	1	1	5
3	20595	E5G077	Ara h 3 allergen OS=Arachis hypc	Ara h 3	58305	36,32	5	9,07E+04	1	1	5
3	20594	Q0GM57	Iso-Ara h3 OS=Arachis hypogaea	Ara h 3	58263	36,32	5	9,07E+04	1	1	5
								**3,53E+05**			

Band #11 38-41 kDa of SDSPAGE resolved Gastric CONTROL on 120'

No detection

Band #11 38-41 kDa of SDSPAGE resolved Gastric DIGESTSION on 120' matched to Band #11 in control

Protein Group	Protein ID	Accession	Description	Allergome	Avg, Mass	-10lgP	Coverage (%)	Area	#Peptides	#Unique	#Spec
7	20595	E5G077	Ara h 3 allergen OS=Arachis hypc	Ara h 3	58305	93,15	16	8,13E+05	4	3	14
7	20594	Q0GM57	Iso-Ara h3 OS=Arachis hypogaea	Ara h 3	58263	93,15	16	8,13E+05	4	3	14
7	20593	Q6IWG5	Glycinin (Fragment) OS=Arachis I	Ara h 3	58061	93,15	16	8,13E+05	4	3	14
5	22697	T2B9M0	Fructose-bisphosphate aldolase	n/a	38383	134,89	19	4,75E+05	3	3	15
6	20849	Q6PSU3	Conarachin (Fragment) OS=Aracl	Ara h 1	66575	144,98	18	4,03E+05	7	7	13
6	20850	P43237	Allergen Ara h 1 clone P17 OS=A	Ara h 1	70283	144,98	17	4,03E+05	7	7	13
6	20851	B3IXL2	Main allergen Ara h1 OS=Arachis	Ara h 1	70283	144,98	17	4,03E+05	7	7	13
1	20581	Q647H3	Arachin Ahy-2 OS=Arachis hypog	Ara h 3	61532	245,95	45	1,90E+05	19	3	216
2	20576	Q9FZ11	Gly1 OS=Arachis hypogaea OX=3	Ara h 3	60449	249,49	46	6,66E+04	20	3	219
12	21994	\0A0A6ZDT(	Glyceraldehyde-3-phosphate del	n/a	20099	61,73	8	6,80E+03	1	1	1
12	21405	\0A0A6ZDP:	Glyceraldehyde-3-phosphate del	n/a	20101	61,73	8	6,80E+03	1	1	1
							total	**4,4E+06**			
							Ara h 1	1,2E+06			
							Ara h 3	2,7E+06			

Band #12 41-45 kDa of SDSPAGE resolved Gastric CONTROL on 120'

Protein Group	Protein ID	Accession	Description	Allergome	Avg, Mass	-10lgP	Coverage (%)	Area	#Peptides	#Unique	#Spec
1	20593	Q6IWG5	Glycinin (Fragment) OS=Arachis h	Ara h 3	58061	123,43	17	2,72E+05	4	4	33
1	20594	Q0GM57	Iso-Ara h3 OS=Arachis hypogaea	Ara h 3	58263	123,43	17	2,72E+05	4	4	33
1	20595	E5G077	Ara h 3 allergen OS=Arachis hypo	Ara h 3	58305	123,43	17	2,72E+05	4	4	33
2	20590	O82580	Glycinin (Fragment) OS=Arachis h	Ara h 3	58350	168,47	23	7,13E+04	10	1	92
3	20787	A0A191UJ6	DNA-directed RNA polymerase s	n/a	156977	25,61	1	9,71E+03	1	1	1
							total	**9,0E+05**			
							Ara h 3	8,9E+05			

Band #12 41-45 kDa of SDSPAGE resolved Gastric DIGESTION on 120' matched to Band# 12 in control

Protein Group	Protein ID	Accession	Description	Allergome	Avg, Mass	-10lgP	Coverage (%)	Area	#Peptides	#Unique	#Spec
1	20576	Q9FZ11	Gly1 OS=Arachis hypogaea OX=3	Ara h 3	60449	40,98	4	1,65E+03	1	1	1
1	20578	B5TYU1	Arachin Arah3 isoform OS=Arach	Ara h 3	60624	40,98	4	1,65E+03	1	1	1
1	20579	Q5I6T2	Arachin Ahy-4 OS=Arachis hypog	Ara h 3	60736	40,98	4	1,65E+03	1	1	1
1	20581	Q647H3	Arachin Ahy-2 OS=Arachis hypog	Ara h 3	61532	40,98	4	1,65E+03	1	1	1
1	20574	A1DZF0	Arachin 6 OS=Arachis hypogaea	Ara h 3	60375	40,98	4	1,65E+03	1	1	1
1	20577	Q9SQH7	Glycinin OS=Arachis hypogaea O	Ara h 3	61011	40,98	4	1,65E+03	1	1	1
1	20580	Q647H4	Arachin Ahy-1 OS=Arachis hypog	Ara h 3	61506	40,98	4	1,65E+03	1	1	1
1	20582	Q8LKN1	Allergen Arah3/Arah4 OS=Arachi	Ara h 3	61738	40,98	4	1,65E+03	1	1	1
1	20575	Q8LL03	Trypsin inhibitor (Fragment) OS=	Ara h 3	25499	40,98	11	1,65E+03	1	1	1
							Ara h 3	**1,49E+04**			

Band #13 45-55 kDa of SDSPAGE resolved Gastric CONTROL on 120'

Protein Group	Protein ID	Accession	Description	Allergome	Avg, Mass	-10lgP	Coverage (%)	Area	#Peptides	#Unique	#Spec
9	22400	Q6PSU4	Conarachin (Fragment) OS=Aracl	Ara h 1	48095	129,6	18	3,50E+04	7	7	8
9	20853	N1NG13	Seed storage protein Ara h1 OS=	Ara h 1	71345	129,6	12	3,50E+04	7	7	8
9	20854	P43238	Allergen Ara h 1 clone P41B OS=	Ara h 1	71345	129,6	12	3,50E+04	7	7	8
9	20849	Q6PSU3	Conarachin (Fragment) OS=Aracl	Ara h 1	66575	127,53	13	3,50E+04	7	7	8
9	20850	P43237	Allergen Ara h 1 clone P17 OS=A	Ara h 1	70283	127,53	12	3,50E+04	7	7	8
9	20851	B3IXL2	Main allergen Ara h1 OS=Arachis	Ara h 1	70283	127,53	12	3,50E+04	7	7	8
1	20580	Q647H4	Arachin Ahy-1 OS=Arachis hypog	Ara h 3	61506	233,78	54	2,45E+04	23	2	221
14	22697	T2B9M0	Fructose-bisphosphate aldolase	n/a	38383	35,36	6	2,30E+04	1	1	2
85	21693	A0A290G0J[illegible]	Resistance protein (Fragment) O	n/a	57776	27,13	2	7,72E+04	1	1	1
17	22425	C9W981	Phosphoenolpyruvate carboxyla	n/a	116425	22,72	1	3,94E+03	1	1	1
							total	**3,39E+05**			
							Ara h 1	2,10E+05			
							Ara h 3	2,45E+04			

Band B 47-55 kDa of SDSPAGE resolved Gastric DIGESTION on 120' matched with Band #13 in control

Protein Group	Protein ID	Accession	Description	Allergome	Avg, Mass	-10lgP	Coverage (%)	Area	#Peptides	#Unique	#Spec
1	20593	Q6IWG5	Glycinin (Fragment) OS=Arachis h	Ara h 3	58061	43,22	5	9,75E+04	1	1	7
1	20595	E5G077	Ara h 3 allergen OS=Arachis hypc	Ara h 3	58305	43,22	5	9,75E+04	1	1	7
1	20594	Q0GM57	Iso-Ara h3 OS=Arachis hypogaea	Ara h 3	58263	43,22	5	9,75E+04	1	1	7
2	20576	Q9FZ11	Gly1 OS=Arachis hypogaea OX=3	Ara h 3	60449	103,35	7	2,70E+04	2	2	3
85	21693	A0A290G0J[illegible]	Resistance protein (Fragment) O	n/a	57776	27,13	2	1,72E+04	1	1	1
2	20580	Q647H4	Arachin Ahy-1 OS=Arachis hypog	Ara h 3	61506	89,78	2	1,28E+04	1	1	2
2	20591	Q6T2T4	Storage protein OS=Arachis hypc	Ara h 3	61499	89,78	2	1,28E+04	1	1	2
2	20582	Q8LKN1	Allergen Arah3/Arah4 OS=Arachi	Ara h 3	61738	89,78	2	1,28E+04	1	1	2
2	20581	Q647H3	Arachin Ahy-2 OS=Arachis hypog	Ara h 3	61532	89,78	2	1,28E+04	1	1	2
2	20574	A1DZF0	Arachin 6 OS=Arachis hypogaea	Ara h 3	60375	89,78	2	1,28E+04	1	1	2
2	20578	B5TYU1	Arachin Arah3 isoform OS=Arach	Ara h 3	60624	89,78	2	1,28E+04	1	1	2
2	20579	Q5I6T2	Arachin Ahy-4 OS=Arachis hypog	Ara h 3	60736	89,78	2	1,28E+04	1	1	2
2	20577	Q9SQH7	Glycinin OS=Arachis hypogaea O	Ara h 3	61011	89,78	2	1,28E+04	1	1	2
							total	**3,37E+05**			
							Ara h 3	1,25E+05			

Band #14 of SDSPAGE resolved Gastric CONTROL on 120'

No determination

Band #13 55-60 kDa of SDSPAGE resolved Gastric DIGESTION on 120' matched to Band #14 in control

Protein Group	Protein ID	Accession	Description	Allergome	Avg. Mass	-10lgP	Coverage (%)	Area	#Peptides	#Unique	#Spec
1	20853	N1NG13	Seed storage protein Ara h1 OS=	Ara h 1	71345	367,01	54	9,50E+06	56	15	423
1	20854	P43238	Allergen Ara h 1 clone P41B OS=	Ara h 1	71345	367,01	54	9,50E+06	56	15	423
2	20849	Q6PSU3	Conarachin (Fragment) OS=Aracl	Ara h 1	66575	354,63	56	3,88E+06	50	9	379
2	20850	P43237	Allergen Ara h 1 clone P17 OS=A	Ara h 1	70283	354,63	53	3,88E+06	50	9	379
2	20851	B3IXL2	Main allergen Ara h1 OS=Arachis	Ara h 1	70283	354,63	53	3,88E+06	50	9	379
5	20577	Q9SQH7	Glycinin OS=Arachis hypogaea O.	Ara h 3	61011	88,43	2	1,85E+04	1	1	1
5	20581	Q647H3	Arachin Ahy-2 OS=Arachis hypog	Ara h 3	61532	88,43	2	1,85E+04	1	1	1
5	20580	Q647H4	Arachin Ahy-1 OS=Arachis hypog	Ara h 3	61506	88,43	2	1,85E+04	1	1	1
5	20582	Q8LKN1	Allergen Arah3/Arah4 OS=Arachi	Ara h 3	61738	88,43	2	1,85E+04	1	1	1
5	20576	Q9FZ11	Gly1 OS=Arachis hypogaea OX=3	Ara h 3	60449	88,43	2	1,85E+04	1	1	1
5	20579	Q5I6T2	Arachin Ahy-4 OS=Arachis hypog	Ara h 3	60736	88,43	2	1,85E+04	1	1	1
5	20591	Q6T2T4	Storage protein OS=Arachis hypc	Ara h 3	61499	88,43	2	1,85E+04	1	1	1
5	20574	A1DZF0	Arachin 6 OS=Arachis hypogaea (	Ara h 3	60375	88,43	2	1,85E+04	1	1	1
5	20578	B5TYU1	Arachin Arah3 isoform OS=Arach	Ara h 3	60624	88,43	2	1,85E+04	1	1	1
							total	**3,08E+07**			
							Ara h 1	3,06E+07			
							Ara h 3	1,67E+05			

Band #14 60-66 kDa of SDSPAGE resolved Gastric DIGESTION on 120' matched to Band #14 in control

Protein Group	Protein ID	Accession	Description	Allergome	Avg. Mass	-10lgP	Coverage (%)	Area	#Peptides	#Unique	#Spec
1	20853	N1NG13	Seed storage protein Ara h1 OS=	Ara h 1	71345	75,17	14	1,09E+05	4	4	14
1	20854	P43238	Allergen Ara h 1 clone P41B OS=	Ara h 1	71345	75,17	14	1,09E+05	4	4	14
1	22400	Q6PSU4	Conarachin (Fragment) OS=Aracl	Ara h 1	48095	75,17	21	1,09E+05	4	4	14
1	20849	Q6PSU3	Conarachin (Fragment) OS=Aracl	Ara h 1	66575	75,17	15	1,09E+05	4	4	14
1	20850	P43237	Allergen Ara h 1 clone P17 OS=A	Ara h 1	70283	75,17	14	1,09E+05	4	4	14
1	20851	B3IXL2	Main allergen Ara h1 OS=Arachis	Ara h 1	70283	75,17	14	1,09E+05	4	4	14
2	21956	E5FHY1	Late embryogenesis abundant pı	n/a	10140	30,2	14	0,00E+00	1	1	1
							Ara h 1	**6,54E+05**			

Band #15 67-250 kDa of SDSPAGE resolved Gastric CONTROL on 120'

Protein Group	Protein ID	Accession	Description?	Allergome	Avg, Mass	-10lgP	Coverage (%)	Area	#Peptides	#Unique	#Spec
2	20850	P43237	Allergen Ara h 1 clone P17 OS=A	Ara h 1	70283	173,86	17	9,78E+04	9	1	53
2	20851	B3IXL2	Main allergen Ara h1 OS=Arachis	Ara h 1	70283	173,86	17	9,78E+04	9	1	53
2	20849	Q6PSU3	Conarachin (Fragment) OS=Aracl	Ara h 1	66575	173,86	18	9,78E+04	9	1	53
6	20593	Q6IWG5	Glycinin (Fragment) OS=Arachis I	Ara h 3	58061	57,81	5	9,74E+04	1	1	7
6	20595	E5G077	Ara h 3 allergen OS=Arachis hypc	Ara h 3	58305	57,81	5	9,74E+04	1	1	7
6	20594	Q0GM57	Iso-Ara h3 OS=Arachis hypogaea	Ara h 3	58263	57,81	5	9,74E+04	1	1	7
3	21031	Q9M5D3	Lipoxygenase OS=Arachis hypoga	n/a	97616	170,26	8	6,68E+04	5	5	16
3	21032	Q4JME6	Lipoxygenase OS=Arachis hypoga	n/a	97476	170,26	8	6,68E+04	5	5	16
3	21033	Q4JME7	Lipoxygenase OS=Arachis hypoga	n/a	97596	170,26	8	6,68E+04	5	5	16
1	20853	N1NG13	Seed storage protein Ara h1 OS=	Ara h 1	71345	172,12	16	5,11E+04	9	1	53
1	20854	P43238	Allergen Ara h 1 clone P41B OS=	Ara h 1	71345	172,12	16	5,11E+04	9	1	53
5	20574	A1DZF0	Arachin 6 OS=Arachis hypogaea (	Ara h 3	60375	126,79	8	4,28E+03	3	1	8
5	20578	B5TYU1	Arachin Arah3 isoform OS=Arach	Ara h 3	60624	126,79	8	4,28E+03	3	1	8
5	20580	Q647H4	Arachin Ahy-1 OS=Arachis hypog	Ara h 3	61506	126,79	8	4,28E+03	3	1	8
5	20591	Q6T2T4	Storage protein OS=Arachis hypc	Ara h 3	61499	126,79	8	4,28E+03	3	1	8
4	20579	Q5I6T2	Arachin Ahy-4 OS=Arachis hypog	Ara h 3	60736	139,76	8	7,99E+02	3	1	9
4	20576	Q9FZ11	Gly1 OS=Arachis hypogaea OX=3	Ara h 3	60449	139,76	8	7,99E+02	3	1	9
4	20581	Q647H3	Arachin Ahy-2 OS=Arachis hypog	Ara h 3	61532	139,76	8	7,99E+02	3	1	9
							total	**9,08E+05**			
							Ara h 1	3,96E+05			
							Ara h 3	3,12E+05			
							Lipoxygen	2,00E+05			

Band C 67-80 kDa of SDSPAGE resolved Gastric DIGESTION on 120' matched with Band #15 in control

Protein Group	Protein ID	Accession	Description	Allergome	Avg, Mass	-10lgP	Coverage (%)	Area	#Peptides	#Unique	#Spec
9	22753	\0A126DIH(	DnaJ OS=Arachis hypogaea OX=3	n/a	39071	23,66	2	1,33E+04	1	1	1
3	20853	N1NG13	Seed storage protein Ara h1 OS=	Ara h 1	71345	59,25	2	3,97E+03	1	1	3
3	20854	P43238	Allergen Ara h 1 clone P41B OS=	Ara h 1	71345	59,25	2	3,97E+03	1	1	3
3	20850	P43237	Allergen Ara h 1 clone P17 OS=A	Ara h 1	70283	59,25	2	3,97E+03	1	1	3
3	20851	B3IXL2	Main allergen Ara h1 OS=Arachis	Ara h 1	70283	59,25	2	3,97E+03	1	1	3
3	20849	Q6PSU3	Conarachin (Fragment) OS=Aracl	Ara h 1	66575	59,25	2	3,97E+03	1	1	3
3	22400	Q6PSU4	Conarachin (Fragment) OS=Aracl	Ara h 1	48095	59,25	3	3,97E+03	1	1	3
7	20593	Q6IWG5	Glycinin (Fragment) OS=Arachis I	Ara h 3	58061	21,90	5	3,91E+03	1	1	1
7	20595	E5G077	Ara h 3 allergen OS=Arachis hypc	Ara h 3	58305	21,90	5	3,91E+03	1	1	1
7	20594	Q0GM57	Iso-Ara h3 OS=Arachis hypogaea	Ara h 3	58263	21,90	5	3,91E+03	1	1	1

Band C 67-80 kDa of SDSPAGE resolved Gastric DIGESTION on 120' matched with Band #15 in control

Protein Group	Protein ID	Accession	Description	Allergome	Avg, Mass	-10lgP	Coverage (%)	Area	#Peptides	#Unique	#Spec
5	21031	Q9M5D3	Lipoxygenase OS=Arachis hypog	n/a	97616	66,63	2	1,35E+03	1	1	1
5	21032	Q4JME6	Lipoxygenase OS=Arachis hypog	n/a	97476	66,63	2	1,35E+03	1	1	1
5	21033	Q4JME7	Lipoxygenase OS=Arachis hypog	n/a	97596	66,63	2	1,35E+03	1	1	1
							total	**5,29E+04**			
							Ara h 1	2,38E+04			
							Ara h 3	1,17E+04			
							Lipoxygen	4,05E+03			

Band #15 80-150 kDa of SDSPAGE resolved Gastric DIGESTION on 120' matched to Band #15 in control

Protein Group	Protein ID	Accession	Description	Allergome	Avg, Mass	-10lgP	Coverage (%)	Area	#Peptides	#Unique	#Spec
1	20853	N1NG13	Seed storage protein Ara h1 OS=	Ara h 1	71345	51,75	10	3,80E+04	2	2	9
1	20854	P43238	Allergen Ara h 1 clone P41B OS=	Ara h 1	71345	51,75	10	3,80E+04	2	2	9
1	20849	Q6PSU3	Conarachin (Fragment) OS=Aracl	Ara h 1	66575	51,75	11	3,80E+04	2	2	9
1	20850	P43237	Allergen Ara h 1 clone P17 OS=A	Ara h 1	70283	51,75	10	3,80E+04	2	2	9
1	20851	B3IXL2	Main allergen Ara h1 OS=Arachis	Ara h 1	70283	51,75	10	3,80E+04	2	2	9
1	22400	Q6PSU4	Conarachin (Fragment) OS=Aracl	Ara h 1	48095	51,75	14	3,80E+04	2	2	9
							Ara h 1	**2,28E+05**			

Band D 150-250 kDa of SDSPAGE resolved Gastric DIGESTION on 120' matched with Band #15 in control

Protein Group	Protein ID	Accession	Description	-10lgP	Allergome	Avg, Mass	Coverage (%)	Area	#Peptides	#Unique	#Spec
4	24521	\0A191UJC(	NAD(P)H-quinone oxidoreductas	26,72	n/a	145505	3	1,64E+04	1	1	1

Summary

1. Notes Band #1 Control 13-15 kDa

2. Result Statistics

Figure 1. False discovery rate (FDR) curve. X axis is the number of peptide-spectrum matches (PSM) being kept. Y axis is the corresponding FDR.

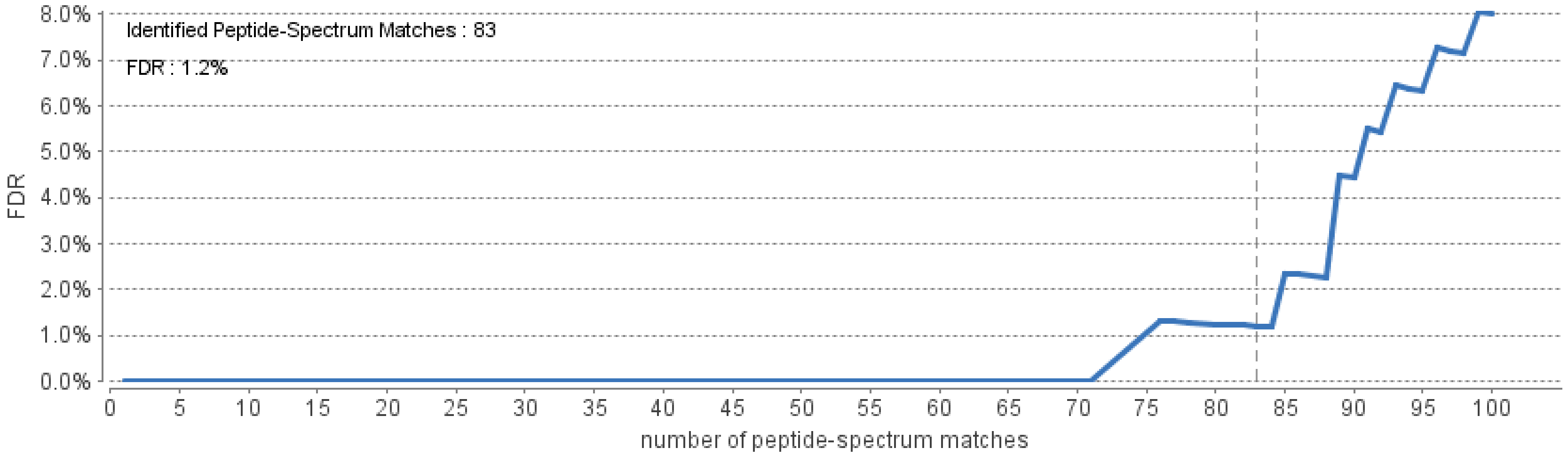

Figure 2. PSM score distribution. **(a)** Distribution of PEAKS peptide score; **(b)** Scatterplot of PEAKS peptide score versus precursor mass error.

(a)

(b)

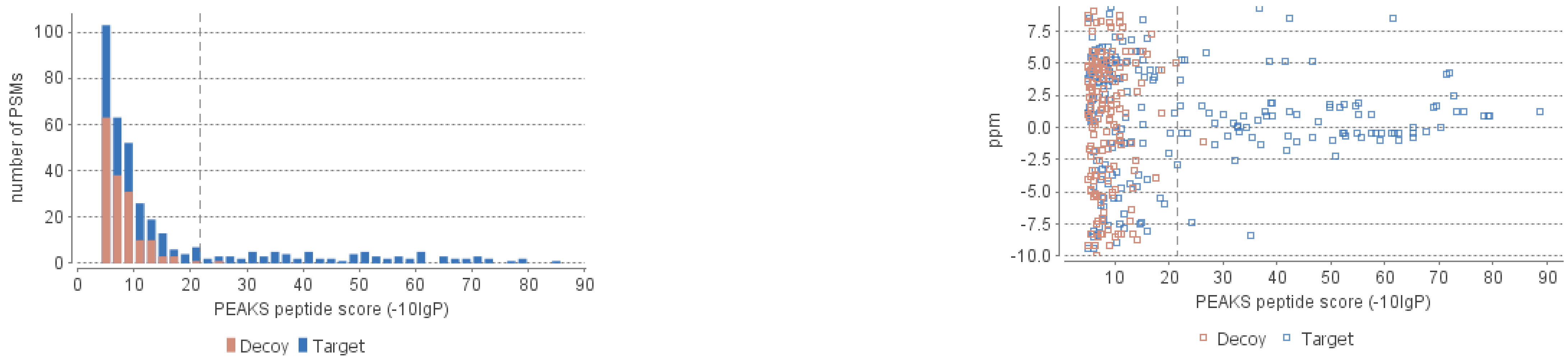

Figure 3. Distribution of peptide feature detection. **(a)** Feature m/z distribution; **(b)** Feature RT distribution

(a)

(b)

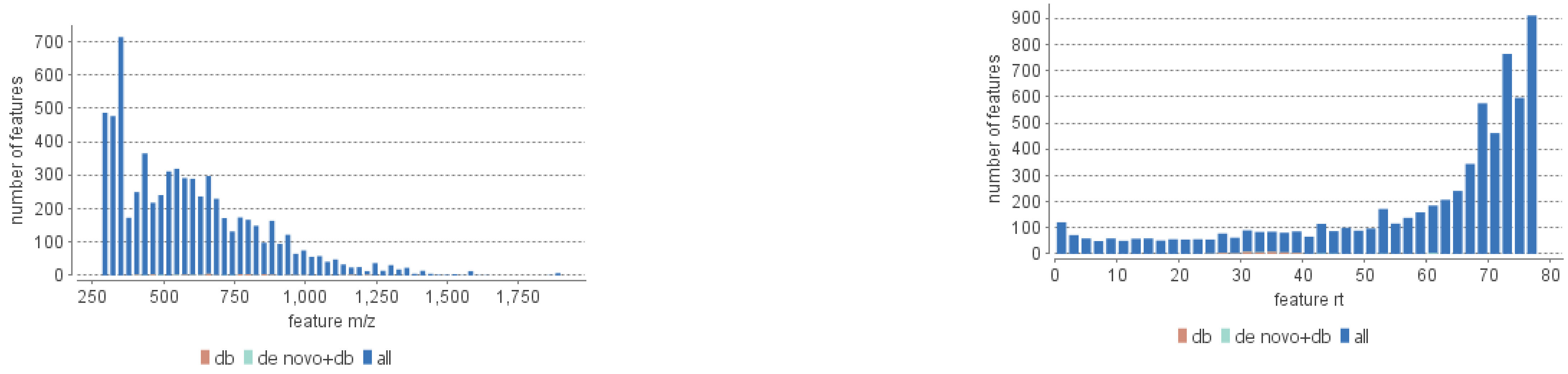

Figure 4. Distribution of identified peptide features. **(a)** Feature abundance distribution **(b)** *De novo* sequencing validation.

(a)

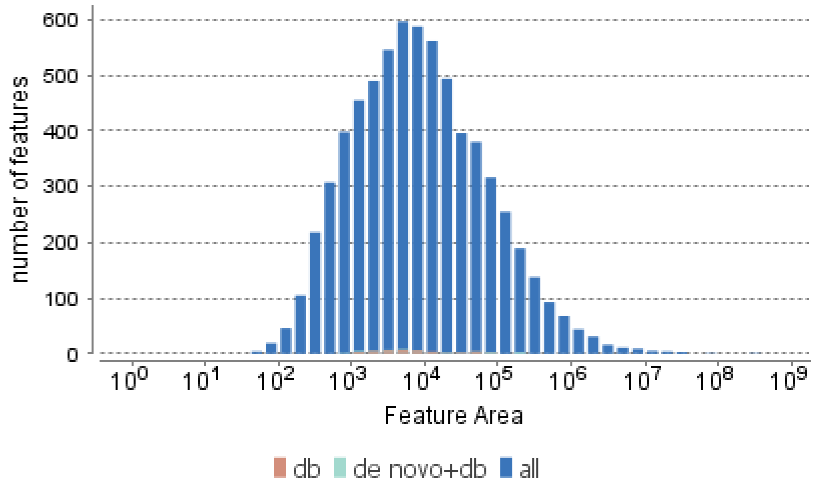

(b)

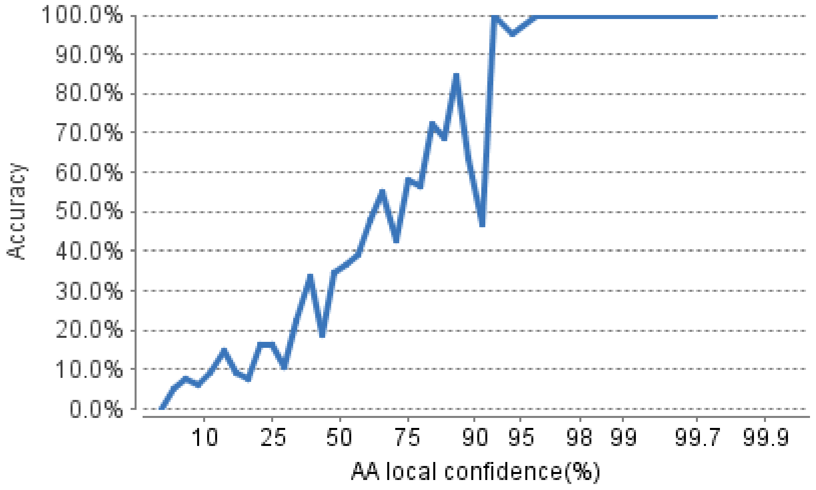

Table 1. Statistics of data.

# of MS scans	2929
# of MS/MS scans	1416
# of Features	6788
# of Chimera scans	87

Table 2. Result filtration parameters.

Peptide -10lgP	≥21.6
PTM Ascore	≥20
Protein -10lgP	≥20
Proteins unique peptides	≥1
De novo ALC Score	≥50%

Table 4. PTM profile.

Name	ΔMass	Position	#PSM	-10lgP	Abundance	AScore
Deamidation	.98	NQ	39	70.15	3.56E3	12.28
Carbamidomethyl	57.02	C	31	70.15	3.56E3	1000.00
HydPro	15.99	P	3	28.39	1.99E3	6.67
Oxidation	15.99	M	3	71.21	4E3	1000.00
Carboxymethyl	58.01	C	2	71.81	1.55E3	1000.00
Carbamylation	43.01	N-term	1	36.32	2.12E3	1000.00
Methylation(KR)	14.02	R	1	36.32	2.12E3	78.93

Amidation	-.98	C-term	1	23.30	2.79E4	1000.00

Table 3. Statistics of filtered result.

Peptide-Spectrum Matches	83
Peptide sequences	45
Protein groups	1
Proteins	9
Proteins (#Unique Peptides)	9 (>2); 0 (=2); 0 (=1);
FDR (Peptide-Spectrum Matches)	1.2%
FDR (Peptide Sequences)	2.2%
FDR (Protein Group)	0.0%
De Novo Only Spectra	12
# of identified Features	49
# of identified MS/MS scans	83

3. Experiment Control

Figure 5. Precursor mass error of peptide-spectrum matches (PSM) in filtered result. **(a)** Distribution of precursor mass error in ppm; **(b)** Scatterplot of precursor m/z versus precursor mass error in ppm.

(a)

(b)

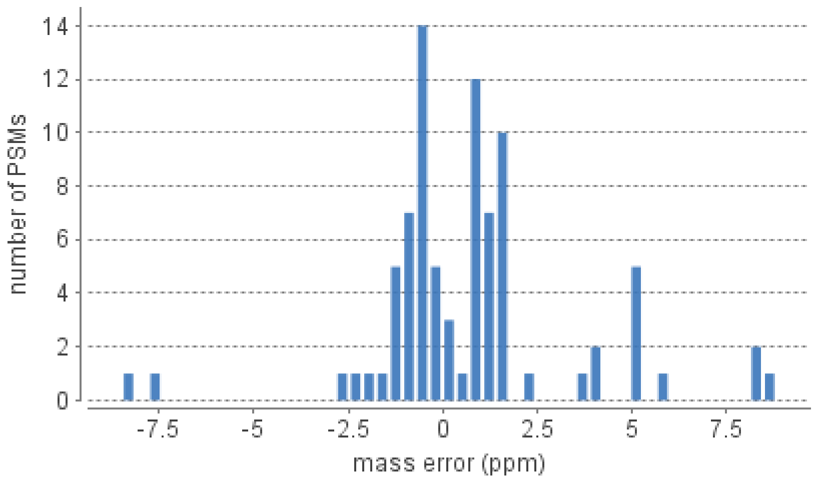

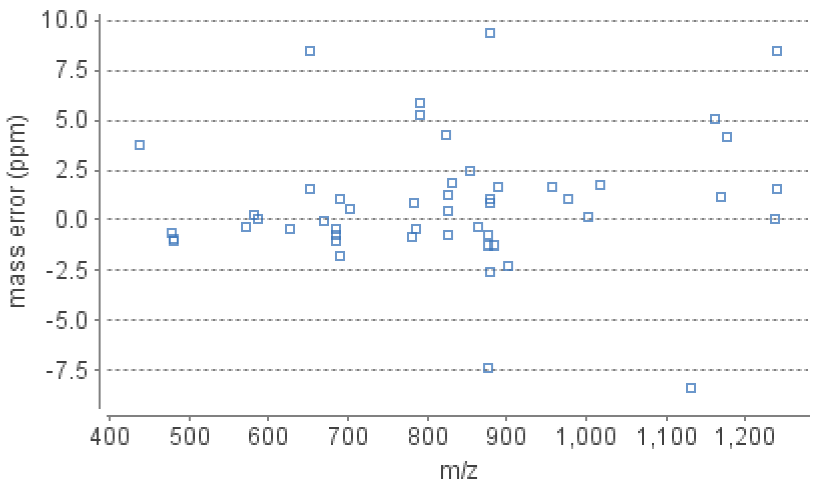

Table 5. Number of identified peptides in each sample by the number of missed cleavages

Missed Cleavages	0	1	2	3	4+
Kontrola 1 traka odozdole najmanja masa	32	12	1	0	0

4. Other Information

Table 6. Search parameters.

Search Engine Name: PEAKS
Parent Mass Error Tolerance: 10.0 ppm
Fragment Mass Error Tolerance: 0.5 Da
Precursor Mass Search Type: monoisotopic
Enzyme: Trypsin
Max Missed Cleavages: 2

Table 7. Instrument parameters.

Fractions: OB4030.raw
Ion Source: ESI(nano-spray)
Fragmentation Mode: CID, CAD(y and b ions)
MS Scan Mode: FT-ICR/Orbitrap
MS/MS Scan Mode: Linear Ion Trap

Digest Mode: Unspecific
Fixed Modifications:
  Carbamidomethylation: 57.02
Variable Modifications:
  Deamidation (NQ): 0.98
  Oxidation (M): 15.99
  Hydroxylation Pro: 15.99
  Acetylation (K): 42.01
  Acetylation (Protein N-term): 42.01
  Acetylation (N-term): 42.01
  Amidation: -0.98
  Beta-methylthiolation: 45.99
  and 305 more...
Max Variable PTM Per Peptide: 5
Database: Uniprot_Peanut-3818_Jul18
Taxon: All
Contaminant Database: contaminantsMQ_mar19
Searched Entry: 1723
FDR Estimation: Enabled
De novo score (ALC%) threshold: 15
Peptide hit threshold (-10logP): 30.0
Peaks run ID: 427
Merge Options: no merge
Precursor Options: corrected
Charge Options: no correction
Filter Charge: 2 - 8
Process: true
Associate chimera: yes

Protein List

Protein Accession Contains:

Protein Description Contains:

Peptide Sample Area >=

Protein Ptm Contains:

Protein Group	Protein ID	Accession	-10lgP	Coverage (%)	Coverage (%) Kontrola 1 traka odozdole najmanja masa	Area Kontrola 1 traka odozdole najmanja masa	#Peptides	#Unique	#Spec Kontrola 1 traka odozdole najmanja masa	PTM	Avg. Mass	Description
**1**	20574	tr|A1DZF0|A1DZF0_ARAHY	140.52	8	8	3.46E5	6	6	39	Y	60375	Arachin 6 OS=Arachis hypogaea OX=3818 PE=2 SV=1
**1**	20576	tr|Q9FZ11|Q9FZ11_ARAHY	140.52	8	8	3.46E5	6	6	39	Y	60449	Gly1 OS=Arachis hypogaea OX=3818 GN=Gly1 PE=2 SV=1
**1**	20578	tr|B5TYU1|B5TYU1_ARAHY	140.52	8	8	3.46E5	6	6	39	Y	60624	Arachin Arah3 isoform OS=Arachis hypogaea OX=3818 PE=1 SV=1
**1**	20579	tr|Q5I6T2|Q5I6T2_ARAHY	140.52	8	8	3.46E5	6	6	39	Y	60736	Arachin Ahy-4 OS=Arachis hypogaea OX=3818 PE=2 SV=1
**1**	20580	tr|Q647H4|Q647H4_ARAHY	140.52	7	7	3.46E5	6	6	39	Y	61506	Arachin Ahy-1 OS=Arachis hypogaea OX=3818 PE=2 SV=1
**1**	20581	tr|Q647H3|Q647H3_ARAHY	140.52	7	7	3.46E5	6	6	39	Y	61532	Arachin Ahy-2 OS=Arachis hypogaea OX=3818 PE=2 SV=1
**1**	20582	tr|Q8LKN1|Q8LKN1_ARAHY	140.52	7	7	3.46E5	6	6	39	Y	61738	Allergen Arah3/Arah4 OS=Arachis hypogaea OX=3818 PE=3 SV=1
**1**	20577	tr|Q9SQH7|Q9SQH7_ARAHY	140.52	8	8	3.46E5	6	6	39	Y	61011	Glycinin OS=Arachis hypogaea OX=3818 GN=Arah4 PE=2 SV=1
**1**	20575	tr|Q8LL03|Q8LL03_ARAHY	140.52	18	18	3.46E5	6	6	39	Y	25499	Trypsin inhibitor (Fragment) OS=Arachis hypogaea OX=3818 PE=2 SV=1
**total 9 proteins**												

tr|A1DZF0|A1DZF0_ARAHY
back to list

| Protein Coverage | Supporting Peptides |
Protein Coverage:

1 MAKLLELSFC FCFLVLGASS ISFRQQPEEN ACQFQRLNAQ RPDNRIESEG GYIETWNPNN QEFECAGVAL SRLVLRRNAL

c Carbamidomethylation (+57.02)
d Deamidation (NQ) (+0.98)
* Carbamylation (+43.01), Methylation(KR) (+14.02)

108

81 R**RPFYSNAPQ EIFIQQGRGY FGLIFPGCPS TYEEPAQQGR R**YQSQRPPRR LQEEDQSQQQ QDSHQKVHRF NEGDLIAVPT

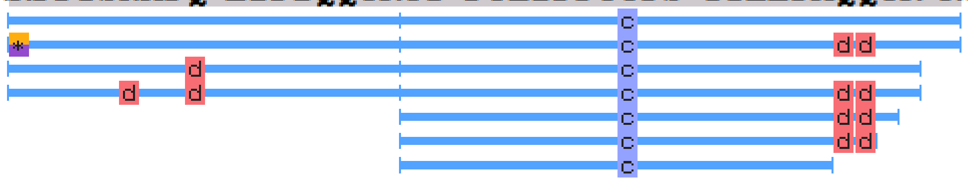

161 GVAFWLYNDH DTDVVAVSLT DTNNNDNQLD QFPRRFNLAG NHEQEFLRYQ QQSRQSRRRS LPLSPYSPQP GQEDREFSPQ

241 GQHGRRERAG QEQENEGGNI FSGFTSEFLA QAFQVDDRQI VQNLRGENES EEQGAIVTVK GGLRILSPDR KSPDEEEEYD

321 EDEYAEEERQ QDRRRGRGSR GSGNGIEETI CTATVKKNIG RNRSPDIYNP QAGSLKTANE LNLLILRWLG LSAEYGNLYR

401 NALFVPHYNT NAHSIIYALR GRAHVQVVDS NGNRVYDEEL QEGHVLVVPQ NFAVAGKSQS ENFEYVAFKT DSRPSIANLA

481 GENSFIDNLP EEVVANSYGL PREQARQLKN NNPFKFFVPP FQQSPRAVA

Supporting Peptides:

Peptide	Uniq	-10lgP	Mass	Length	ppm	m/z	z	RT	Fraction	Scan	Source File	Area Kontrola 1 traka odozdole najmanja masa	#Feature	#Feature Kontrola 1 traka odozdole najmanja masa	Start	End	PTM
R.GYFGLIFPGC(+57.02)PSTYEEPAQQ(+.98)GR.R	Y	70.15	2474.1211	22	0.0	1238.0679	2	34.97	2	1883	OB4030.raw	1.44E4	2	2	99	120	Carbamidomethylation
R.RPFYSNAPQEIFIQQGR.G	Y	65.25	2050.0383	17	-0.4	684.3531	3	30.06	2	1588	OB4030.raw	5.95E4	1	1	82	98	
R.RPFYSNAPQ(+.98)EIFIQQGR.G	Y	61.14	2051.0225	17	-0.4	684.6812	3	30.66	2	1626	OB4030.raw	5.04E4	1	1	82	98	Deamidation (NQ)
R.RPFYSNAPQEIFIQQ(+.98)GR.G	Y	57.62	2051.0225	17	-0.4	684.6812	3	30.66	2	1609	OB4030.raw	5.04E4	1	1	82	98	
R.GYFGLIFPGC(+57.02)PSTYEEPAQ(+.98)Q(+.98)GR.R	Y	52.75	2475.1052	22	-0.7	826.0417	3	35.14	2	1891	OB4030.raw	1.88E4	2	2	99	120	Carbamidomethylation; Deamidation (NQ)
R.RPFYSNAPQEIFIQ(+.98)QGR.G	Y	52.48	2051.0225	17	-0.4	684.6812	3	30.66	2	1606	OB4030.raw	5.04E4	1	1	82	98	
R.RPFYSN(+.98)APQ(+.98)EIFIQQGR.G	Y	50.21	2052.0063	17	-1.1	685.0087	3	31.20	2	1655	OB4030.raw	7.74E3	1	1	82	98	Deamidation (NQ)
R.GYFGLIFPGC(+57.02)PSTYEEPAQQGR.R	Y	47.59	2473.1372	22	0.5	825.3867	3	34.86	2	1877	OB4030.raw	0	0	0	99	120	Carbamidomethylation
R.GYFGLIFPGC(+57.02)PSTYEEPAQ(+.98)Q(+.98)G.R	Y	46.44	2319.0042	21	5.1	1160.5153	2	37.26	2	2021	OB4030.raw	4.5E3	1	1	99	119	Carbamidomethylation; Deamidation (NQ)
R.GYFGLIFPGC(+57.02)PSTYEEPAQ(+.98)Q(+.98)GRR.Y	Y	39.17	2631.2063	23	0.8	878.0768	3	33.51	2	1800	OB4030.raw	5.89E4	2	2	99	121	Carbamidomethylation; Deamidation (NQ)
R.GYFGLIFPGC(+57.02)PSTYEEPAQ(+.98)QGR.R	Y	37.65	2474.1211	22	1.2	825.7153	3	34.97	2	1882	OB4030.raw	1.08E4	1	1	99	120	Carbamidomethylation

Peptide	Uniq	-10lgP	Mass	Length	ppm	m/z	z	RT	Fraction	Scan	Source File	Area Kontrola 1 traka odozdole najmanja masa	#Feature	#Feature Kontrola 1 traka odozdole najmanja masa	Start	End	PTM
R.GYFGLIFPGC(+57.02)PSTYEEPAQQ(+.98)GRR.Y	Y	36.99	2630.2224	23	-1.3	877.7469	3	33.35	2	1785	OB4030.raw	0	0	0	99	121	Carbamidomethylation
R.R(+43.01)(+14.02)PFYSNAPQEIFIQQGR.G	Y	36.32	2107.0598	17	0.5	703.3609	3	30.11	2	1592	OB4030.raw	2.12E3	1	1	82	98	Carbamylation; Methylation(KR)
R.GYFGLIFPGC(+57.02)PSTYEEPAQ(+.98)QGRR.Y	Y	35.45	2630.2224	23	-0.7	877.7474	3	33.38	2	1787	OB4030.raw	0	0	0	99	121	Carbamidomethylation
R.GYFGLIFPGC(+57.02)PSTYEEPAQ(+.98)Q(+.98).G	Y	35.11	2261.9827	20	-8.4	1131.9891	2	37.33	2	2030	OB4030.raw	0	0	0	99	118	Carbamidomethylation; Deamidation (NQ)
R.GYFGLIFPGC(+57.02)PSTYEEPA.Q	Y	32.67	2003.8975	18	0.1	1002.9561	2	37.60	2	2047	OB4030.raw	0	0	0	99	116	Carbamidomethylation
R.GYFGLIFPGC(+57.02)PSTYEEP(+15.99)AQQ(+.98)GRR.Y	Y	28.39	2646.2173	23	-1.3	883.0786	3	34.00	2	1824	OB4030.raw	1.99E3	1	1	99	121	Carbamidomethylation
R.GYFGLIFP(+15.99)GC(+57.02)P(+15.99)STYEEPAQQ(+.98)GRR.Y	Y	26.14	2662.2122	23	1.6	888.4128	3	33.52	2	1793	OB4030.raw	7.79E3	1	1	99	121	Carbamidomethylation
R.GYFGLIFPGC(+57.02)P(+15.99)STYEEP(+15.99)AQQ(+.98)GRR.Y	Y	21.97	2662.2122	23	1.6	888.4128	3	33.52	2	1796	OB4030.raw	7.79E3	1	1	99	121	Carbamidomethylation
**total 19 peptides**																	

tr|Q9FZ11|Q9FZ11_ARAHY
back to list

| Protein Coverage | Supporting Peptides |

Protein Coverage:

■ Carbamidomethylation (+57.02)
■ Deamidation (NQ) (+0.98)
■ Carbamylation (+43.01), Methylation(KR) (+14.02)

```
  1 MIRGRLALSV CFCFLVLGAS SISFRQQPEE NACQFQRLNA QRPDNRLESE GGYIETWNPN NQEFECAGVA LSRLVLRRNA
 81 LRRPFYSNAP QEIFIQQGRG YFGLIFPGCP STYEEPAQQG RRHQSQRAPR RFEGEDQSQQ QQQDSHQKVR RFDEGDLIAV
```

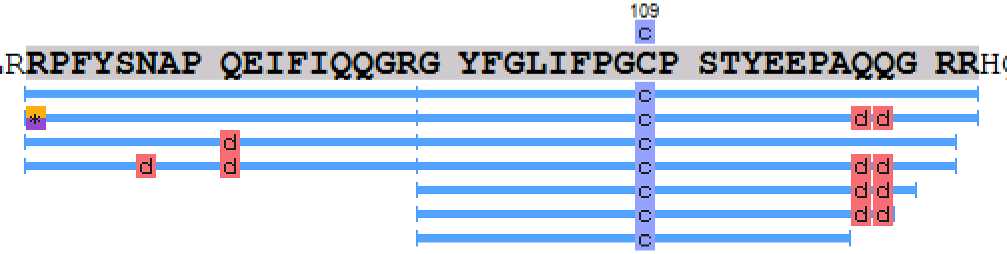

```
161 PTGVALWMFN DHDTDVVAVS LTDTNNNDNQ LDQFPRRFNL AGNHEQEFLR YQQQSRRRSL PYSPYSPQSQ PRQEEREFSP
241 RGQHSRRERA GQEEENEGGN IFSGFTPEFL AQAFQVDDRQ IVQNLRGENE SEEEGAIVTV KGGLRILSPD RKRGADEEEE
321 YDEDEYEYDE EDRRRGRGSR GRGNGIEETI CTASVKKNIG RNRSPDIYNP QAGSLKTAND LNLLILRWLG LSAEYGNLYR
401 NALFVPHYNT NAHSIIYALR GRAHVQVVDS NGNRVYDEEL QEGHVLVVPQ NFAVAGKSQS DNFEYVAFKT DSRPNIANFA
481 GENSIIDNLP EEVVANSYGL PREQARQLKN NNPFKFFVPP SQQSLRAVA
```

Supporting Peptides:

Peptide	Uniq	-10lgP	Mass	Length	ppm	m/z	z	RT	Fraction	Scan	Source File	Area Kontrola 1 traka odozdole najmanja masa	#Feature	#Feature Kontrola 1 traka odozdole najmanja masa	Start	End	PTM
R.GYFGLIFPGC(+57.02)PSTYEEPAQQ(+.98)GR.R	Y	70.15	2474.1211	22	0.0	1238.0679	2	34.97	2	1883	OB4030.raw	1.44E4	2	2	100	121	Carbamidomethylation
R.RPFYSNAPQEIFIQQGR.G	Y	65.25	2050.0383	17	-0.4	684.3531	3	30.06	2	1588	OB4030.raw	5.95E4	1	1	83	99	
R.RPFYSNAPQ(+.98)EIFIQQGR.G	Y	61.14	2051.0225	17	-0.4	684.6812	3	30.66	2	1626	OB4030.raw	5.04E4	1	1	83	99	Deamidation (NQ)
R.RPFYSNAPQEIFIQQ(+.98)GR.G	Y	57.62	2051.0225	17	-0.4	684.6812	3	30.66	2	1609	OB4030.raw	5.04E4	1	1	83	99	
R.GYFGLIFPGC(+57.02)PSTYEEPAQ(+.98)Q(+.98)GR.R	Y	52.75	2475.1052	22	-0.7	826.0417	3	35.14	2	1891	OB4030.raw	1.88E4	2	2	100	121	Carbamidomethylation; Deamidation (NQ)
R.RPFYSNAPQEIFIQ(+.98)QGR.G	Y	52.48	2051.0225	17	-0.4	684.6812	3	30.66	2	1606	OB4030.raw	5.04E4	1	1	83	99	
R.RPFYSN(+.98)APQ(+.98)EIFIQQGR.G	Y	50.21	2052.0063	17	-1.1	685.0087	3	31.20	2	1655	OB4030.raw	7.74E3	1	1	83	99	Deamidation (NQ)
R.GYFGLIFPGC(+57.02)PSTYEEPAQQGR.R	Y	47.59	2473.1372	22	0.5	825.3867	3	34.86	2	1877	OB4030.raw	0	0	0	100	121	Carbamidomethylation
R.GYFGLIFPGC(+57.02)PSTYEEPAQ(+.98)Q(+.98)G.R	Y	46.44	2319.0042	21	5.1	1160.5153	2	37.26	2	2021	OB4030.raw	4.5E3	1	1	100	120	Carbamidomethylation; Deamidation (NQ)
R.GYFGLIFPGC(+57.02)PSTYEEPAQ(+.98)Q(+.98)GRR.H	Y	39.17	2631.2063	23	0.8	878.0768	3	33.51	2	1800	OB4030.raw	5.89E4	2	2	100	122	Carbamidomethylation; Deamidation (NQ)
R.GYFGLIFPGC(+57.02)PSTYEEPAQ(+.98)QGR.R	Y	37.65	2474.1211	22	1.2	825.7153	3	34.97	2	1882	OB4030.raw	1.08E4	1	1	100	121	Carbamidomethylation

Peptide	Uniq	-10lgP	Mass	Length	ppm	m/z	z	RT	Fraction	Scan	Source File	Area Kontrola 1 traka odozdole najmanja masa	#Feature	#Feature Kontrola 1 traka odozdole najmanja masa	Start	End	PTM
R.GYFGLIFPGC(+57.02)PSTYEEPAQQ(+.98)GRR.H	Y	36.99	2630.2224	23	-1.3	877.7469	3	33.35	2	1785	OB4030.raw	0	0	0	100	122	Carbamidomethylation
R.R(+43.01)(+14.02)PFYSNAPQEIFIQQGR.G	Y	36.32	2107.0598	17	0.5	703.3609	3	30.11	2	1592	OB4030.raw	2.12E3	1	1	83	99	Carbamylation; Methylation(KR)
R.GYFGLIFPGC(+57.02)PSTYEEPAQ(+.98)QGRR.H	Y	35.45	2630.2224	23	-0.7	877.7474	3	33.38	2	1787	OB4030.raw	0	0	0	100	122	Carbamidomethylation
R.GYFGLIFPGC(+57.02)PSTYEEPAQ(+.98)Q(+.98).G	Y	35.11	2261.9827	20	-8.4	1131.9891	2	37.33	2	2030	OB4030.raw	0	0	0	100	119	Carbamidomethylation; Deamidation (NQ)
R.GYFGLIFPGC(+57.02)PSTYEEPA.Q	Y	32.67	2003.8975	18	0.1	1002.9561	2	37.60	2	2047	OB4030.raw	0	0	0	100	117	Carbamidomethylation
R.GYFGLIFPGC(+57.02)PSTYEEP(+15.99)AQQ(+.98)GRR.H	Y	28.39	2646.2173	23	-1.3	883.0786	3	34.00	2	1824	OB4030.raw	1.99E3	1	1	100	122	Carbamidomethylation
R.GYFGLIFP(+15.99)GC(+57.02)P(+15.99)STYEEPAQQ(+.98)GRR.H	Y	26.14	2662.2122	23	1.6	888.4128	3	33.52	2	1793	OB4030.raw	7.79E3	1	1	100	122	Carbamidomethylation
R.GYFGLIFPGC(+57.02)P(+15.99)STYEEP(+15.99)AQQ(+.98)GRR.H	Y	21.97	2662.2122	23	1.6	888.4128	3	33.52	2	1796	OB4030.raw	7.79E3	1	1	100	122	Carbamidomethylation
**total 19 peptides**																	

tr|B5TYU1|B5TYU1_ARAHY

back to list

| Protein Coverage | Supporting Peptides |

Protein Coverage:

1 MAKLLELSFC FCFLVLGASS ISFRQQPEEN ACQFQRLNAQ RPDNRIESEG GYIETWNPNN QEFECAGVAL SRLVLRRNAL

81 RRPFYSNAPQ EIFIQQGRGY FGLIFPGCPS TYEEPAQQGR RYQSQRPPRR LQEEDQSQQQ QDSHQKVHRF NEGDLIAVPT

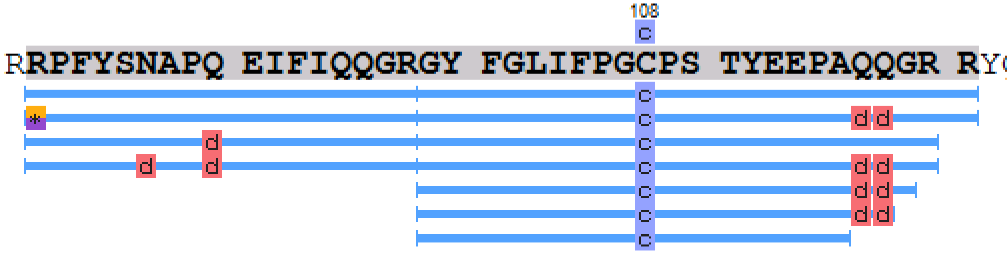

161 GVAFWLYNDH DTDVVAVSLT DTNNNDNQLD QFPRRFNLAG NHEQEFLRYQ QQSRQSRRRS LPYSPYSPQS QPRQEEREFS

241 PRGQHSRRER AGQEEENEGG NIFSGFTPEF LAQAFQVDDR QIVQNLRGEN ESEEQGAIVT VRGGLRILSP DRKRGADEEE

321 EYDEDEYEYD EEDRRRGRGS RGSGNGIEET ICTATVKKNI GRNRSPDIYN PQAGSLKTAN ELNLLILRWL GLSAEYGNLY

401 RNALFVPHYN TNAHSIIYAL RGRAHVQVVD SNGNRVYDEE LQEGHVLVVP QNFAVAGKSQ SDNFEYVAFK TDSRPSIANL

481 AGENSVIDNL PEEVVANSYG LPREQARQLK NNNPFKFFVP PSQQSPRAVA

c Carbamidomethylation (+57.02)
d Deamidation (NQ) (+0.98)
* Carbamylation (+43.01), Methylation(KR) (+14.02)

Supporting Peptides:

Peptide	Uniq	-10lgP	Mass	Length	ppm	m/z	z	RT	Fraction	Scan	Source File	Area Kontrola 1 traka odozdole najmanja masa	#Feature	#Feature Kontrola 1 traka odozdole najmanja masa	Start	End	PTM
R.GYFGLIFPGC(+57.02)PSTYEEPAQQ(+.98)GR.R	Y	70.15	2474.1211	22	0.0	1238.0679	2	34.97	2	1883	OB4030.raw	1.44E4	2	2	99	120	Carbamidomethylation
R.RPFYSNAPQEIFIQQGR.G	Y	65.25	2050.0383	17	-0.4	684.3531	3	30.06	2	1588	OB4030.raw	5.95E4	1	1	82	98	
R.RPFYSNAPQ(+.98)EIFIQQGR.G	Y	61.14	2051.0225	17	-0.4	684.6812	3	30.66	2	1626	OB4030.raw	5.04E4	1	1	82	98	Deamidation (NQ)
R.RPFYSNAPQEIFIQQ(+.98)GR.G	Y	57.62	2051.0225	17	-0.4	684.6812	3	30.66	2	1609	OB4030.raw	5.04E4	1	1	82	98	
R.GYFGLIFPGC(+57.02)PSTYEEPAQ(+.98)Q(+.98)GR.R	Y	52.75	2475.1052	22	-0.7	826.0417	3	35.14	2	1891	OB4030.raw	1.88E4	2	2	99	120	Carbamidomethylation; Deamidation (NQ)
R.RPFYSNAPQEIFIQ(+.98)QGR.G	Y	52.48	2051.0225	17	-0.4	684.6812	3	30.66	2	1606	OB4030.raw	5.04E4	1	1	82	98	
R.RPFYSN(+.98)APQ(+.98)EIFIQQGR.G	Y	50.21	2052.0063	17	-1.1	685.0087	3	31.20	2	1655	OB4030.raw	7.74E3	1	1	82	98	Deamidation (NQ)
R.GYFGLIFPGC(+57.02)PSTYEEPAQQGR.R	Y	47.59	2473.1372	22	0.5	825.3867	3	34.86	2	1877	OB4030.raw	0	0	0	99	120	Carbamidomethylation
R.GYFGLIFPGC(+57.02)PSTYEEPAQ(+.98)Q(+.98)G.R	Y	46.44	2319.0042	21	5.1	1160.5153	2	37.26	2	2021	OB4030.raw	4.5E3	1	1	99	119	Carbamidomethylation; Deamidation (NQ)
R.GYFGLIFPGC(+57.02)PSTYEEPAQ(+.98)Q(+.98)GRR.Y	Y	39.17	2631.2063	23	0.8	878.0768	3	33.51	2	1800	OB4030.raw	5.89E4	2	2	99	121	Carbamidomethylation; Deamidation (NQ)
R.GYFGLIFPGC(+57.02)PSTYEEPAQ(+.98)QGR.R	Y	37.65	2474.1211	22	1.2	825.7153	3	34.97	2	1882	OB4030.raw	1.08E4	1	1	99	120	Carbamidomethylation

Peptide	Uniq	-10lgP	Mass	Length	ppm	m/z	z	RT	Fraction	Scan	Source File	Area Kontrola 1 traka odozdole najmanja masa	#Feature	#Feature Kontrola 1 traka odozdole najmanja masa	Start	End	PTM
R.GYFGLIFPGC(+57.02)PSTYEEPAQQ(+.98)GRR.Y	Y	36.99	2630.2224	23	-1.3	877.7469	3	33.35	2	1785	OB4030.raw	0	0	0	99	121	Carbamidomethylation
R.R(+43.01)(+14.02)PFYSNAPQEIFIQQGR.G	Y	36.32	2107.0598	17	0.5	703.3609	3	30.11	2	1592	OB4030.raw	2.12E3	1	1	82	98	Carbamylation; Methylation(KR)
R.GYFGLIFPGC(+57.02)PSTYEEPAQ(+.98)QGRR.Y	Y	35.45	2630.2224	23	-0.7	877.7474	3	33.38	2	1787	OB4030.raw	0	0	0	99	121	Carbamidomethylation
R.GYFGLIFPGC(+57.02)PSTYEEPAQ(+.98)Q(+.98).G	Y	35.11	2261.9827	20	-8.4	1131.9891	2	37.33	2	2030	OB4030.raw	0	0	0	99	118	Carbamidomethylation; Deamidation (NQ)
R.GYFGLIFPGC(+57.02)PSTYEEPA.Q	Y	32.67	2003.8975	18	0.1	1002.9561	2	37.60	2	2047	OB4030.raw	0	0	0	99	116	Carbamidomethylation
R.GYFGLIFPGC(+57.02)PSTYEEP(+15.99)AQQ(+.98)GRR.Y	Y	28.39	2646.2173	23	-1.3	883.0786	3	34.00	2	1824	OB4030.raw	1.99E3	1	1	99	121	Carbamidomethylation
R.GYFGLIFP(+15.99)GC(+57.02)P(+15.99)STYEEPAQQ(+.98)GRR.Y	Y	26.14	2662.2122	23	1.6	888.4128	3	33.52	2	1793	OB4030.raw	7.79E3	1	1	99	121	Carbamidomethylation
R.GYFGLIFPGC(+57.02)P(+15.99)STYEEP(+15.99)AQQ(+.98)GRR.Y	Y	21.97	2662.2122	23	1.6	888.4128	3	33.52	2	1796	OB4030.raw	7.79E3	1	1	99	121	Carbamidomethylation
**total 19 peptides**																	

tr|Q5I6T2|Q5I6T2_ARAHY

back to list

| Protein Coverage | Supporting Peptides |

Protein Coverage:

1 MAKLLELSFC FCFLVLGASS ISFRQQPEEN ACQFQRLNAQ RPDNRIESEG GYIETWNPNN QEFECAGVAL SRLVLRRNAL

81 RRPFYSNAPQ EIFIQQGRGY FGLIFPGCPS TYEEPAQQGR RSQSQRPPRR LQGEDQSQQQ QDSHQKVHRF DEGDLIAVPT

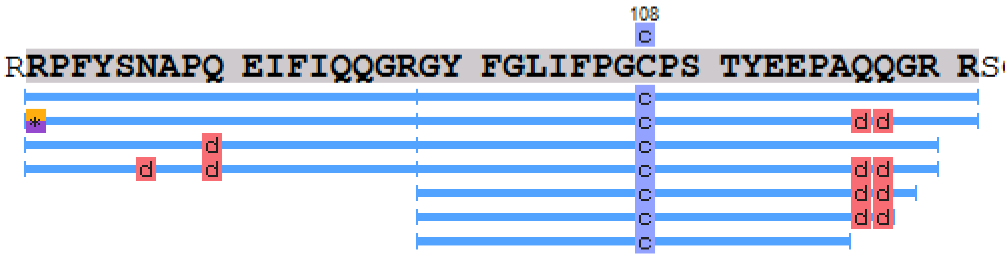

c Carbamidomethylation (+57.02)
d Deamidation (NQ) (+0.98)
* Carbamylation (+43.01), Methylation(KR) (+14.02)

161 GVAFWLYNDH DTDVVAVSLT DTNNNDNQLD QFPRRFNLAG NHEQEFLRYQ QQSRQSRRRS LPYSPYSPQS QPRQEEREFS
241 PRGQHSRRER AGQEEENEGG NIFSGFTPEF LEQAFQVDDR QIVQNLRGEN ESEEEGAIVT VRGGLRILSP DRKRGADEEE
321 EYDEDEYEYD EEDRRRGRGS RGRGNGIEET ICTASVKKNI GRNRSPDIYN PQAGSLKTAN DLNLLILRWL GLSAEYGNLY
401 RNALFVPHYN TNAHSIIYAL RGRAHVQVVD SNGNRVYDEE LQEGHVLVVP QNFAVAGKSQ SDNFEYVAFK TDSRPSIANL
481 AGENSVIDNL PEEVVANSYG LQREQARQQL KNNNPFKFFV PPSQQSPRAV A

Supporting Peptides:

Peptide	Uniq	-10lgP	Mass	Length	ppm	m/z	z	RT	Fraction	Scan	Source File	Area Kontrola 1 traka odozdole najmanja masa	#Feature	#Feature Kontrola 1 traka odozdole najmanja masa	Start	End	PTM
R.GYFGLIFPGC(+57.02)PSTYEEPAQQ(+.98)GR.R	Y	70.15	2474.1211	22	0.0	1238.0679	2	34.97	2	1883	OB4030.raw	1.44E4	2	2	99	120	Carbamidomethylation
R.RPFYSNAPQEIFIQQGR.G	Y	65.25	2050.0383	17	-0.4	684.3531	3	30.06	2	1588	OB4030.raw	5.95E4	1	1	82	98	
R.RPFYSNAPQ(+.98)EIFIQQGR.G	Y	61.14	2051.0225	17	-0.4	684.6812	3	30.66	2	1626	OB4030.raw	5.04E4	1	1	82	98	Deamidation (NQ)
R.RPFYSNAPQEIFIQQ(+.98)GR.G	Y	57.62	2051.0225	17	-0.4	684.6812	3	30.66	2	1609	OB4030.raw	5.04E4	1	1	82	98	
R.GYFGLIFPGC(+57.02)PSTYEEPAQ(+.98)Q(+.98)GR.R	Y	52.75	2475.1052	22	-0.7	826.0417	3	35.14	2	1891	OB4030.raw	1.88E4	2	2	99	120	Carbamidomethylation; Deamidation (NQ)
R.RPFYSNAPQEIFIQ(+.98)QGR.G	Y	52.48	2051.0225	17	-0.4	684.6812	3	30.66	2	1606	OB4030.raw	5.04E4	1	1	82	98	
R.RPFYSN(+.98)APQ(+.98)EIFIQQGR.G	Y	50.21	2052.0063	17	-1.1	685.0087	3	31.20	2	1655	OB4030.raw	7.74E3	1	1	82	98	Deamidation (NQ)
R.GYFGLIFPGC(+57.02)PSTYEEPAQQGR.R	Y	47.59	2473.1372	22	0.5	825.3867	3	34.86	2	1877	OB4030.raw	0	0	0	99	120	Carbamidomethylation
R.GYFGLIFPGC(+57.02)PSTYEEPAQ(+.98)Q(+.98)G.R	Y	46.44	2319.0042	21	5.1	1160.5153	2	37.26	2	2021	OB4030.raw	4.5E3	1	1	99	119	Carbamidomethylation; Deamidation (NQ)
R.GYFGLIFPGC(+57.02)PSTYEEPAQ(+.98)Q(+.98)GRR.S	Y	39.17	2631.2063	23	0.8	878.0768	3	33.51	2	1800	OB4030.raw	5.89E4	2	2	99	121	Carbamidomethylation; Deamidation (NQ)
R.GYFGLIFPGC(+57.02)PSTYEEPAQ(+.98)QGR.R	Y	37.65	2474.1211	22	1.2	825.7153	3	34.97	2	1882	OB4030.raw	1.08E4	1	1	99	120	Carbamidomethylation

Peptide	Uniq	-10lgP	Mass	Length	ppm	m/z	z	RT	Fraction	Scan	Source File	Area Kontrola 1 traka odozdole najmanja masa	#Feature	#Feature Kontrola 1 traka odozdole najmanja masa	Start	End	PTM
R.GYFGLIFPGC(+57.02)PSTYEEPAQQ(+.98)GRR.S	Y	36.99	2630.2224	23	-1.3	877.7469	3	33.35	2	1785	OB4030.raw	0	0	0	99	121	Carbamidomethylation
R.R(+43.01)(+14.02)PFYSNAPQEIFIQQGR.G	Y	36.32	2107.0598	17	0.5	703.3609	3	30.11	2	1592	OB4030.raw	2.12E3	1	1	82	98	Carbamylation; Methylation(KR)
R.GYFGLIFPGC(+57.02)PSTYEEPAQ(+.98)QGRR.S	Y	35.45	2630.2224	23	-0.7	877.7474	3	33.38	2	1787	OB4030.raw	0	0	0	99	121	Carbamidomethylation
R.GYFGLIFPGC(+57.02)PSTYEEPAQ(+.98)Q(+.98).G	Y	35.11	2261.9827	20	-8.4	1131.9891	2	37.33	2	2030	OB4030.raw	0	0	0	99	118	Carbamidomethylation; Deamidation (NQ)
R.GYFGLIFPGC(+57.02)PSTYEEPA.Q	Y	32.67	2003.8975	18	0.1	1002.9561	2	37.60	2	2047	OB4030.raw	0	0	0	99	116	Carbamidomethylation
R.GYFGLIFPGC(+57.02)PSTYEEP(+15.99)AQQ(+.98)GRR.S	Y	28.39	2646.2173	23	-1.3	883.0786	3	34.00	2	1824	OB4030.raw	1.99E3	1	1	99	121	Carbamidomethylation
R.GYFGLIFP(+15.99)GC(+57.02)P(+15.99)STYEEPAQQ(+.98)GRR.S	Y	26.14	2662.2122	23	1.6	888.4128	3	33.52	2	1793	OB4030.raw	7.79E3	1	1	99	121	Carbamidomethylation
R.GYFGLIFPGC(+57.02)P(+15.99)STYEEP(+15.99)AQQ(+.98)GRR.S	Y	21.97	2662.2122	23	1.6	888.4128	3	33.52	2	1796	OB4030.raw	7.79E3	1	1	99	121	Carbamidomethylation
**total 19 peptides**																	

tr|Q647H4|Q647H4_ARAHY

back to list

| Protein Coverage | Supporting Peptides |

Protein Coverage:

1 MGKLLALSVC FCFLVLGASS ISFRQQPEEN ACQFQRLNAQ RPDNRIESEG GYIETWNPNN QEFECAGVAL SRLVLRRNAL

81 RRPFYSNAPQ EIFIQQGRGY FGLIFPGCPS TYEEPAQQGR RHQSQRPPRR FQGQDQSQQQ QDSHQKVHRF DEGDLIAVPT

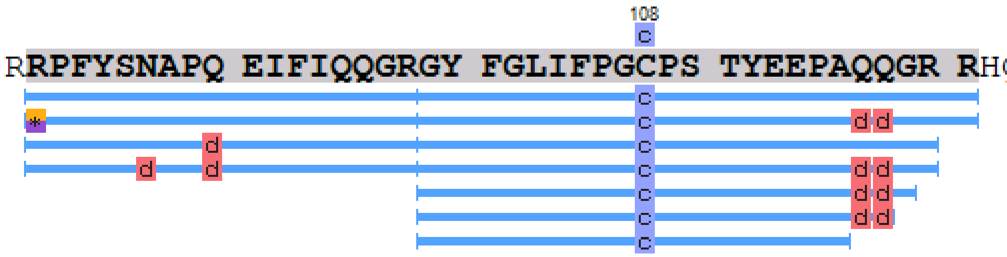

c Carbamidomethylation (+57.02)
d Deamidation (NQ) (+0.98)
* Carbamylation (+43.01), Methylation(KR) (+14.02)

161 GVAFWMYNDH DTDVVAVSLT DTNNNDNQLD QFPRRFNLAG NHEQEFLRYQ QQSRRRSLPY SPYSPQTQPK QEDREFSPRG

241 QHGRRERAGQ EQENEGGNIF SGFTPEFLAQ AFQVDDRQIL QNLRGENESD EQGAIVTVRG GLRILSPDRK RRQQYERPDE

321 EEEYDEDEYE YDEEERQHDR RRGRGSRGSG NGIEETICTA SFKKNIGRNR SPDIYNPQAG SLKTANELNL LILRWLGLSA

401 EYGNLYRNAL FVPHYNTNAH SIIYALRGRA HVQVVDSNGD RVFDEELQEG HVLVVPQNFA VAGKSQSENF EYVAFKTDSR

481 PSIANLAGEN SFIDNLPEEV VANSYGLPRE QARQLKNNNP FKFFVPPSEQ SLRAVA

Supporting Peptides:

Peptide	Uniq	-10lgP	Mass	Length	ppm	m/z	z	RT	Fraction	Scan	Source File	Area Kontrola 1 traka odozdole najmanja masa	#Feature	#Feature Kontrola 1 traka odozdole najmanja masa	Start	End	PTM
R.GYFGLIFPGC(+57.02)PSTYEEPAQQ(+.98)GR.R	Y	70.15	2474.1211	22	0.0	1238.0679	2	34.97	2	1883	OB4030.raw	1.44E4	2	2	99	120	Carbamidomethylation
R.RPFYSNAPQEIFIQQGR.G	Y	65.25	2050.0383	17	-0.4	684.3531	3	30.06	2	1588	OB4030.raw	5.95E4	1	1	82	98	
R.RPFYSNAPQ(+.98)EIFIQQGR.G	Y	61.14	2051.0225	17	-0.4	684.6812	3	30.66	2	1626	OB4030.raw	5.04E4	1	1	82	98	Deamidation (NQ)
R.RPFYSNAPQEIFIQQ(+.98)GR.G	Y	57.62	2051.0225	17	-0.4	684.6812	3	30.66	2	1609	OB4030.raw	5.04E4	1	1	82	98	
R.GYFGLIFPGC(+57.02)PSTYEEPAQ(+.98)Q(+.98)GR.R	Y	52.75	2475.1052	22	-0.7	826.0417	3	35.14	2	1891	OB4030.raw	1.88E4	2	2	99	120	Carbamidomethylation; Deamidation (NQ)
R.RPFYSNAPQEIFIQ(+.98)QGR.G	Y	52.48	2051.0225	17	-0.4	684.6812	3	30.66	2	1606	OB4030.raw	5.04E4	1	1	82	98	
R.RPFYSN(+.98)APQ(+.98)EIFIQQGR.G	Y	50.21	2052.0063	17	-1.1	685.0087	3	31.20	2	1655	OB4030.raw	7.74E3	1	1	82	98	Deamidation (NQ)
R.GYFGLIFPGC(+57.02)PSTYEEPAQQGR.R	Y	47.59	2473.1372	22	0.5	825.3867	3	34.86	2	1877	OB4030.raw	0	0	0	99	120	Carbamidomethylation
R.GYFGLIFPGC(+57.02)PSTYEEPAQ(+.98)Q(+.98)G.R	Y	46.44	2319.0042	21	5.1	1160.5153	2	37.26	2	2021	OB4030.raw	4.5E3	1	1	99	119	Carbamidomethylation; Deamidation (NQ)
R.GYFGLIFPGC(+57.02)PSTYEEPAQ(+.98)Q(+.98)GRR.H	Y	39.17	2631.2063	23	0.8	878.0768	3	33.51	2	1800	OB4030.raw	5.89E4	2	2	99	121	Carbamidomethylation; Deamidation (NQ)
R.GYFGLIFPGC(+57.02)PSTYEEPAQ(+.98)QGR.R	Y	37.65	2474.1211	22	1.2	825.7153	3	34.97	2	1882	OB4030.raw	1.08E4	1	1	99	120	Carbamidomethylation

Peptide	Uniq	-10lgP	Mass	Length	ppm	m/z	z	RT	Fraction	Scan	Source File	Area Kontrola 1 traka odozdole najmanja masa	#Feature	#Feature Kontrola 1 traka odozdole najmanja masa	Start	End	PTM
R.GYFGLIFPGC(+57.02)PSTYEEPAQQ(+.98)GRR.H	Y	36.99	2630.2224	23	-1.3	877.7469	3	33.35	2	1785	OB4030.raw	0	0	0	99	121	Carbamidomethylation
R.R(+43.01)(+14.02)PFYSNAPQEIFIQQGR.G	Y	36.32	2107.0598	17	0.5	703.3609	3	30.11	2	1592	OB4030.raw	2.12E3	1	1	82	98	Carbamylation; Methylation(KR)
R.GYFGLIFPGC(+57.02)PSTYEEPAQ(+.98)QGRR.H	Y	35.45	2630.2224	23	-0.7	877.7474	3	33.38	2	1787	OB4030.raw	0	0	0	99	121	Carbamidomethylation
R.GYFGLIFPGC(+57.02)PSTYEEPAQ(+.98)Q(+.98).G	Y	35.11	2261.9827	20	-8.4	1131.9891	2	37.33	2	2030	OB4030.raw	0	0	0	99	118	Carbamidomethylation; Deamidation (NQ)
R.GYFGLIFPGC(+57.02)PSTYEEPA.Q	Y	32.67	2003.8975	18	0.1	1002.9561	2	37.60	2	2047	OB4030.raw	0	0	0	99	116	Carbamidomethylation
R.GYFGLIFPGC(+57.02)PSTYEEP(+15.99)AQQ(+.98)GRR.H	Y	28.39	2646.2173	23	-1.3	883.0786	3	34.00	2	1824	OB4030.raw	1.99E3	1	1	99	121	Carbamidomethylation
R.GYFGLIFP(+15.99)GC(+57.02)P(+15.99)STYEEPAQQ(+.98)GRR.H	Y	26.14	2662.2122	23	1.6	888.4128	3	33.52	2	1793	OB4030.raw	7.79E3	1	1	99	121	Carbamidomethylation
R.GYFGLIFPGC(+57.02)P(+15.99)STYEEP(+15.99)AQQ(+.98)GRR.H	Y	21.97	2662.2122	23	1.6	888.4128	3	33.52	2	1796	OB4030.raw	7.79E3	1	1	99	121	Carbamidomethylation
**total 19 peptides**																	

tr|Q647H3|Q647H3_ARAHY

back to list

| Protein Coverage | Supporting Peptides |

Protein Coverage:

1 MAKLLALSVC FCFLVLGASS ISFRQQPEEN ACQFQRLNAQ RPDNRLESEG GYIETWNPNN QEFECAGVAL SRLVLRRNAL

c Carbamidomethylation (+57.02)
d Deamidation (NQ) (+0.98)
* Carbamylation (+43.01), Methylation(KR) (+14.02)

81 RRPFYSNAPQ EIFIQQGRGY FGLIFPGCPS TYEEPAQQGR RHQSQRAPRR FEGEDQSQQQ QQDSHQKVRR FDEGDLIAVP

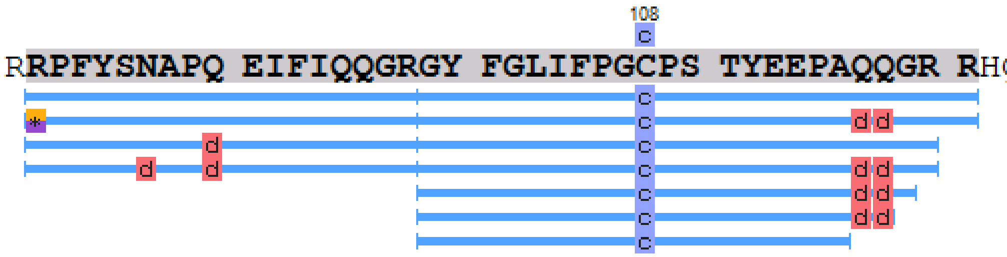

161 TGVALWMYND HDTDVVAVSL TDTNNNDNQL DQFPRRFNLA GNHEQEFLRY QQQSRRRSLP YSPYSPQSQP RQEEREFSPR
241 GQHSRRERAG QEQENEGGNI FSGFTPEFLA QAFQVDDRQI LQNLRGENES DEQGAIVTVR GGLRILSPDR KRRQQYERPD
321 EEEEYDEDEY EYDEEERQQD RRRGRGSRGR GNGIEETICT ASVKKNIGRN RSPDIYNPQA GSLKTANDLN LLILRWLGLS
401 AEYGNLYRNA LFVPHYNTNA HSIIYALRGR AHVQVVDSNG NRVYDEELQE GHVLVVPQNF AVAGKSQSDN FEYVAFKTDS
481 RPSIANLAGE NSIIDNLPEE VVANSYGLPR EQARQLKNNN PFKFFVPPSQ QSLGAVA

Supporting Peptides:

Peptide	Uniq	-10lgP	Mass	Length	ppm	m/z	z	RT	Fraction	Scan	Source File	Area Kontrola 1 traka odozdole najmanja masa	#Feature	#Feature Kontrola 1 traka odozdole najmanja masa	Start	End	PTM
R.GYFGLIFPGC(+57.02)PSTYEEPAQQ(+.98)GR.R	Y	70.15	2474.1211	22	0.0	1238.0679	2	34.97	2	1883	OB4030.raw	1.44E4	2	2	99	120	Carbamidomethylation
R.RPFYSNAPQEIFIQQGR.G	Y	65.25	2050.0383	17	-0.4	684.3531	3	30.06	2	1588	OB4030.raw	5.95E4	1	1	82	98	
R.RPFYSNAPQ(+.98)EIFIQQGR.G	Y	61.14	2051.0225	17	-0.4	684.6812	3	30.66	2	1626	OB4030.raw	5.04E4	1	1	82	98	Deamidation (NQ)
R.RPFYSNAPQEIFIQQ(+.98)GR.G	Y	57.62	2051.0225	17	-0.4	684.6812	3	30.66	2	1609	OB4030.raw	5.04E4	1	1	82	98	
R.GYFGLIFPGC(+57.02)PSTYEEPAQ(+.98)Q(+.98)GR.R	Y	52.75	2475.1052	22	-0.7	826.0417	3	35.14	2	1891	OB4030.raw	1.88E4	2	2	99	120	Carbamidomethylation; Deamidation (NQ)
R.RPFYSNAPQEIFIQ(+.98)QGR.G	Y	52.48	2051.0225	17	-0.4	684.6812	3	30.66	2	1606	OB4030.raw	5.04E4	1	1	82	98	
R.RPFYSN(+.98)APQ(+.98)EIFIQQGR.G	Y	50.21	2052.0063	17	-1.1	685.0087	3	31.20	2	1655	OB4030.raw	7.74E3	1	1	82	98	Deamidation (NQ)
R.GYFGLIFPGC(+57.02)PSTYEEPAQQGR.R	Y	47.59	2473.1372	22	0.5	825.3867	3	34.86	2	1877	OB4030.raw	0	0	0	99	120	Carbamidomethylation
R.GYFGLIFPGC(+57.02)PSTYEEPAQ(+.98)Q(+.98)G.R	Y	46.44	2319.0042	21	5.1	1160.5153	2	37.26	2	2021	OB4030.raw	4.5E3	1	1	99	119	Carbamidomethylation; Deamidation (NQ)
R.GYFGLIFPGC(+57.02)PSTYEEPAQ(+.98)Q(+.98)GRR.H	Y	39.17	2631.2063	23	0.8	878.0768	3	33.51	2	1800	OB4030.raw	5.89E4	2	2	99	121	Carbamidomethylation; Deamidation (NQ)
R.GYFGLIFPGC(+57.02)PSTYEEPAQ(+.98)QGR.R	Y	37.65	2474.1211	22	1.2	825.7153	3	34.97	2	1882	OB4030.raw	1.08E4	1	1	99	120	Carbamidomethylation

Peptide	Uniq	-10lgP	Mass	Length	ppm	m/z	z	RT	Fraction	Scan	Source File	Area Kontrola 1 traka odozdole najmanja masa	#Feature	#Feature Kontrola 1 traka odozdole najmanja masa	Start	End	PTM
R.GYFGLIFPGC(+57.02)PSTYEEPAQQ(+.98)GRR.H	Y	36.99	2630.2224	23	-1.3	877.7469	3	33.35	2	1785	OB4030.raw	0	0	0	99	121	Carbamidomethylation
R.R(+43.01)(+14.02)PFYSNAPQEIFIQQGR.G	Y	36.32	2107.0598	17	0.5	703.3609	3	30.11	2	1592	OB4030.raw	2.12E3	1	1	82	98	Carbamylation; Methylation(KR)
R.GYFGLIFPGC(+57.02)PSTYEEPAQ(+.98)QGRR.H	Y	35.45	2630.2224	23	-0.7	877.7474	3	33.38	2	1787	OB4030.raw	0	0	0	99	121	Carbamidomethylation
R.GYFGLIFPGC(+57.02)PSTYEEPAQ(+.98)Q(+.98).G	Y	35.11	2261.9827	20	-8.4	1131.9891	2	37.33	2	2030	OB4030.raw	0	0	0	99	118	Carbamidomethylation; Deamidation (NQ)
R.GYFGLIFPGC(+57.02)PSTYEEPA.Q	Y	32.67	2003.8975	18	0.1	1002.9561	2	37.60	2	2047	OB4030.raw	0	0	0	99	116	Carbamidomethylation
R.GYFGLIFPGC(+57.02)PSTYEEP(+15.99)AQQ(+.98)GRR.H	Y	28.39	2646.2173	23	-1.3	883.0786	3	34.00	2	1824	OB4030.raw	1.99E3	1	1	99	121	Carbamidomethylation
R.GYFGLIFP(+15.99)GC(+57.02)P(+15.99)STYEEPAQQ(+.98)GRR.H	Y	26.14	2662.2122	23	1.6	888.4128	3	33.52	2	1793	OB4030.raw	7.79E3	1	1	99	121	Carbamidomethylation
R.GYFGLIFPGC(+57.02)P(+15.99)STYEEP(+15.99)AQQ(+.98)GRR.H	Y	21.97	2662.2122	23	1.6	888.4128	3	33.52	2	1796	OB4030.raw	7.79E3	1	1	99	121	Carbamidomethylation
**total 19 peptides**																	

tr|Q8LKN1|Q8LKN1_ARAHY

back to list

| Protein Coverage | Supporting Peptides |

Protein Coverage:

1 MGKLLALSVC FCFLVLGASS ISFRQQPEEN ACQFQRLNAQ RPDNRIESEG GYIETWNPNN QEFECAGVAL SRLVLRRNAL

81 R**RPFYSNAPQ EIFIQQGRGY FGLIFPGCPS TYEEPAQQGR R**HQSQRPPRR FQGQDQSQQQ QDSHQKVHRF DEGDLIAVPT

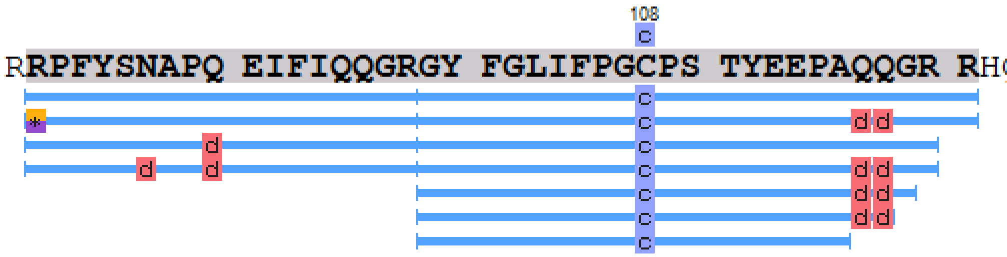

161 GVAFWMYNDH DTDVVAVSLT DTNNNDNQLD QFPRRFNLAG NHEQEFLRYQ QQSRRRSLPY SPYSPQTQPK QEDREFSPRG

241 QHGRRERAGQ EQENEGGNIF SGFTPEFLAQ AFQVDDRQIL QNLRGENESD EQGAIVTVRG GLRILSPDRK RRQQYERPDE

321 EEEYDEDEYE YDEEERQQDR RRGRGSRGSG NGIEETICTA SFKKNIGRNR SPDIYNPQAG SLKTANELQL NLLILRWLGL

401 SAEYGNLYRN ALFVPHYNTN AHSIIYALRG RAHVQVVDSN GDRVFDEELQ EGHVLVVPQN FAVAGKSQSE NFEYVAFKTD

481 SRPSIANLAG ENSFIDNLPE EVVANSYGLP REQARQLKNN NPFKFFVPPS EQSLRAVA

c Carbamidomethylation (+57.02)
d Deamidation (NQ) (+0.98)
* Carbamylation (+43.01), Methylation(KR) (+14.02)

Supporting Peptides:

Peptide	Uniq	-10lgP	Mass	Length	ppm	m/z	z	RT	Fraction	Scan	Source File	Area Kontrola 1 traka odozdole najmanja masa	#Feature	#Feature Kontrola 1 traka odozdole najmanja masa	Start	End	PTM
R.GYFGLIFPGC(+57.02)PSTYEEPAQQ(+.98)GR.R	Y	70.15	2474.1211	22	0.0	1238.0679	2	34.97	2	1883	OB4030.raw	1.44E4	2	2	99	120	Carbamidomethylation
R.RPFYSNAPQEIFIQQGR.G	Y	65.25	2050.0383	17	-0.4	684.3531	3	30.06	2	1588	OB4030.raw	5.95E4	1	1	82	98	
R.RPFYSNAPQ(+.98)EIFIQQGR.G	Y	61.14	2051.0225	17	-0.4	684.6812	3	30.66	2	1626	OB4030.raw	5.04E4	1	1	82	98	Deamidation (NQ)
R.RPFYSNAPQEIFIQQ(+.98)GR.G	Y	57.62	2051.0225	17	-0.4	684.6812	3	30.66	2	1609	OB4030.raw	5.04E4	1	1	82	98	
R.GYFGLIFPGC(+57.02)PSTYEEPAQ(+.98)Q(+.98)GR.R	Y	52.75	2475.1052	22	-0.7	826.0417	3	35.14	2	1891	OB4030.raw	1.88E4	2	2	99	120	Carbamidomethylation; Deamidation (NQ)
R.RPFYSNAPQEIFIQ(+.98)QGR.G	Y	52.48	2051.0225	17	-0.4	684.6812	3	30.66	2	1606	OB4030.raw	5.04E4	1	1	82	98	
R.RPFYSN(+.98)APQ(+.98)EIFIQQGR.G	Y	50.21	2052.0063	17	-1.1	685.0087	3	31.20	2	1655	OB4030.raw	7.74E3	1	1	82	98	Deamidation (NQ)
R.GYFGLIFPGC(+57.02)PSTYEEPAQQGR.R	Y	47.59	2473.1372	22	0.5	825.3867	3	34.86	2	1877	OB4030.raw	0	0	0	99	120	Carbamidomethylation
R.GYFGLIFPGC(+57.02)PSTYEEPAQ(+.98)Q(+.98)G.R	Y	46.44	2319.0042	21	5.1	1160.5153	2	37.26	2	2021	OB4030.raw	4.5E3	1	1	99	119	Carbamidomethylation; Deamidation (NQ)
R.GYFGLIFPGC(+57.02)PSTYEEPAQ(+.98)Q(+.98)GRR.H	Y	39.17	2631.2063	23	0.8	878.0768	3	33.51	2	1800	OB4030.raw	5.89E4	2	2	99	121	Carbamidomethylation; Deamidation (NQ)
R.GYFGLIFPGC(+57.02)PSTYEEPAQ(+.98)QGR.R	Y	37.65	2474.1211	22	1.2	825.7153	3	34.97	2	1882	OB4030.raw	1.08E4	1	1	99	120	Carbamidomethylation

Peptide	Uniq	-10lgP	Mass	Length	ppm	m/z	z	RT	Fraction	Scan	Source File	Area Kontrola 1 traka odozdole najmanja masa	#Feature	#Feature Kontrola 1 traka odozdole najmanja masa	Start	End	PTM
R.GYFGLIFPGC(+57.02)PSTYEEPAQQ(+.98)GRR.H	Y	36.99	2630.2224	23	-1.3	877.7469	3	33.35	2	1785	OB4030.raw	0	0	0	99	121	Carbamidomethylation
R.R(+43.01)(+14.02)PFYSNAPQEIFIQQGR.G	Y	36.32	2107.0598	17	0.5	703.3609	3	30.11	2	1592	OB4030.raw	2.12E3	1	1	82	98	Carbamylation; Methylation(KR)
R.GYFGLIFPGC(+57.02)PSTYEEPAQ(+.98)QGRR.H	Y	35.45	2630.2224	23	-0.7	877.7474	3	33.38	2	1787	OB4030.raw	0	0	0	99	121	Carbamidomethylation
R.GYFGLIFPGC(+57.02)PSTYEEPAQ(+.98)Q(+.98).G	Y	35.11	2261.9827	20	-8.4	1131.9891	2	37.33	2	2030	OB4030.raw	0	0	0	99	118	Carbamidomethylation; Deamidation (NQ)
R.GYFGLIFPGC(+57.02)PSTYEEPA.Q	Y	32.67	2003.8975	18	0.1	1002.9561	2	37.60	2	2047	OB4030.raw	0	0	0	99	116	Carbamidomethylation
R.GYFGLIFPGC(+57.02)PSTYEEP(+15.99)AQQ(+.98)GRR.H	Y	28.39	2646.2173	23	-1.3	883.0786	3	34.00	2	1824	OB4030.raw	1.99E3	1	1	99	121	Carbamidomethylation
R.GYFGLIFP(+15.99)GC(+57.02)P(+15.99)STYEEPAQQ(+.98)GRR.H	Y	26.14	2662.2122	23	1.6	888.4128	3	33.52	2	1793	OB4030.raw	7.79E3	1	1	99	121	Carbamidomethylation
R.GYFGLIFPGC(+57.02)P(+15.99)STYEEP(+15.99)AQQ(+.98)GRR.H	Y	21.97	2662.2122	23	1.6	888.4128	3	33.52	2	1796	OB4030.raw	7.79E3	1	1	99	121	Carbamidomethylation
**total 19 peptides**																	

tr|Q9SQH7|Q9SQH7_ARAHY

back to list

| Protein Coverage | Supporting Peptides |

Protein Coverage:

Carbamidomethylation (+57.02)
Deamidation (NQ) (+0.98)
Carbamylation (+43.01), Methylation(KR) (+14.02)

```
  1  MAKLLELSFC FCFLVLGASS ISFRQQPEEN ACQFQRLNAQ RPDNRIESEG GYIETWNPNN QEFECAGVAL SRLVLRRNAL
 81  RRPFYSNAPQ EIFIQQGRGY FGLIFPGCPS TYEEPAQQGR RYQSQRPPRR LQEEDQSQQQ QDSHQKVHRF NEGDLIAVPT
```

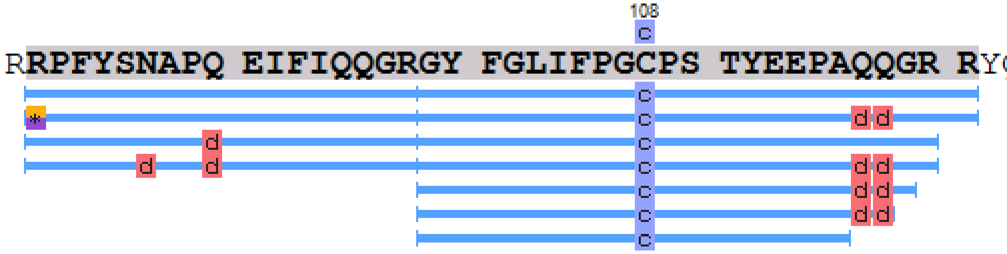


```
161  GVAFWLYNDH DTDVVAVSLT DTNNNDNQLD QFPRRFNLAG NHEQEFLRYQ QQSRQSRRRS LPYSPYSPHS RPRREEREFR
241  PRGQHSRRER AGQEEEDEGG NIFSGFTPEF LEQAFQVDDR QIVQNLWGEN ESEEEGAIVT VRGGLRILSP DGTRGADEEE
321  EYDEDQYEYH EQDGRRGRGS RGGGNGIEET ICTACVKKNI GGNRSPHIYD PQRWFTQNCH DLNLLILRWL GLSAEYGNLY
401  RNALFVPHYN TNAHSIIYAL RGRAHVQVVD SNGNRVYDEE LQEGHVLVVP QNFAVAGKSQ SENFEYVAFK TDSRPSIANF
481  AGENSFIDNL PEEVVANSYG LPREQARQLK NNNPFKFFVP PFQQSPRAVA
```

Supporting Peptides:

Peptide	Uniq	-10lgP	Mass	Length	ppm	m/z	z	RT	Fraction	Scan	Source File	Area Kontrola 1 traka odozdole najmanja masa	#Feature	#Feature Kontrola 1 traka odozdole najmanja masa	Start	End	PTM
R.GYFGLIFPGC(+57.02)PSTYEEPAQQ(+.98)GR.R	Y	70.15	2474.1211	22	0.0	1238.0679	2	34.97	2	1883	OB4030.raw	1.44E4	2	2	99	120	Carbamidomethylation
R.RPFYSNAPQEIFIQQGR.G	Y	65.25	2050.0383	17	-0.4	684.3531	3	30.06	2	1588	OB4030.raw	5.95E4	1	1	82	98	
R.RPFYSNAPQ(+.98)EIFIQQGR.G	Y	61.14	2051.0225	17	-0.4	684.6812	3	30.66	2	1626	OB4030.raw	5.04E4	1	1	82	98	Deamidation (NQ)
R.RPFYSNAPQEIFIQQ(+.98)GR.G	Y	57.62	2051.0225	17	-0.4	684.6812	3	30.66	2	1609	OB4030.raw	5.04E4	1	1	82	98	
R.GYFGLIFPGC(+57.02)PSTYEEPAQ(+.98)Q(+.98)GR.R	Y	52.75	2475.1052	22	-0.7	826.0417	3	35.14	2	1891	OB4030.raw	1.88E4	2	2	99	120	Carbamidomethylation; Deamidation (NQ)
R.RPFYSNAPQEIFIQ(+.98)QGR.G	Y	52.48	2051.0225	17	-0.4	684.6812	3	30.66	2	1606	OB4030.raw	5.04E4	1	1	82	98	
R.RPFYSN(+.98)APQ(+.98)EIFIQQGR.G	Y	50.21	2052.0063	17	-1.1	685.0087	3	31.20	2	1655	OB4030.raw	7.74E3	1	1	82	98	Deamidation (NQ)
R.GYFGLIFPGC(+57.02)PSTYEEPAQQGR.R	Y	47.59	2473.1372	22	0.5	825.3867	3	34.86	2	1877	OB4030.raw	0	0	0	99	120	Carbamidomethylation
R.GYFGLIFPGC(+57.02)PSTYEEPAQ(+.98)Q(+.98)G.R	Y	46.44	2319.0042	21	5.1	1160.5153	2	37.26	2	2021	OB4030.raw	4.5E3	1	1	99	119	Carbamidomethylation; Deamidation (NQ)
R.GYFGLIFPGC(+57.02)PSTYEEPAQ(+.98)Q(+.98)GRR.Y	Y	39.17	2631.2063	23	0.8	878.0768	3	33.51	2	1800	OB4030.raw	5.89E4	2	2	99	121	Carbamidomethylation; Deamidation (NQ)
R.GYFGLIFPGC(+57.02)PSTYEEPAQ(+.98)QGR.R	Y	37.65	2474.1211	22	1.2	825.7153	3	34.97	2	1882	OB4030.raw	1.08E4	1	1	99	120	Carbamidomethylation

Peptide	Uniq	-10lgP	Mass	Length	ppm	m/z	z	RT	Fraction	Scan	Source File	Area Kontrola 1 traka odozdole najmanja masa	#Feature	#Feature Kontrola 1 traka odozdole najmanja masa	Start	End	PTM
R.GYFGLIFPGC(+57.02)PSTYEEPAQQ(+.98)GRR.Y	Y	36.99	2630.2224	23	-1.3	877.7469	3	33.35	2	1785	OB4030.raw	0	0	0	99	121	Carbamidomethylation
R.R(+43.01)(+14.02)PFYSNAPQEIFIQQGR.G	Y	36.32	2107.0598	17	0.5	703.3609	3	30.11	2	1592	OB4030.raw	2.12E3	1	1	82	98	Carbamylation; Methylation(KR)
R.GYFGLIFPGC(+57.02)PSTYEEPAQ(+.98)QGRR.Y	Y	35.45	2630.2224	23	-0.7	877.7474	3	33.38	2	1787	OB4030.raw	0	0	0	99	121	Carbamidomethylation
R.GYFGLIFPGC(+57.02)PSTYEEPAQ(+.98)Q(+.98).G	Y	35.11	2261.9827	20	-8.4	1131.9891	2	37.33	2	2030	OB4030.raw	0	0	0	99	118	Carbamidomethylation; Deamidation (NQ)
R.GYFGLIFPGC(+57.02)PSTYEEPA.Q	Y	32.67	2003.8975	18	0.1	1002.9561	2	37.60	2	2047	OB4030.raw	0	0	0	99	116	Carbamidomethylation
R.GYFGLIFPGC(+57.02)PSTYEEP(+15.99)AQQ(+.98)GRR.Y	Y	28.39	2646.2173	23	-1.3	883.0786	3	34.00	2	1824	OB4030.raw	1.99E3	1	1	99	121	Carbamidomethylation
R.GYFGLIFP(+15.99)GC(+57.02)P(+15.99)STYEEPAQQ(+.98)GRR.Y	Y	26.14	2662.2122	23	1.6	888.4128	3	33.52	2	1793	OB4030.raw	7.79E3	1	1	99	121	Carbamidomethylation
R.GYFGLIFPGC(+57.02)P(+15.99)STYEEP(+15.99)AQQ(+.98)GRR.Y	Y	21.97	2662.2122	23	1.6	888.4128	3	33.52	2	1796	OB4030.raw	7.79E3	1	1	99	121	Carbamidomethylation
**total 19 peptides**																	

tr|Q8LL03|Q8LL03_ARAHY

back to list

| Protein Coverage | Supporting Peptides |

Protein Coverage:

1 NYLHMLLALS VCFCFLVLGA SSISFRQQPE ENACQFQRLN AQRPDNRIES EGGYIETWNP NNQEFECAGV ALSRLVLRRN

81 ALRRPFYSNA PQEIFIQQGR GYFGLIFPGC PSTYEEPAQQ GRRHQSQRPP RRFQGQDQSQ QQDSHQKVH RFDEGDLIAV

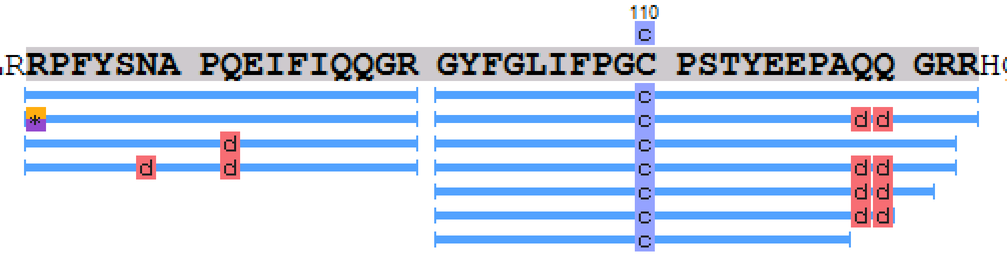

161 PTGVAFWMYN DHDTDVVAVS LTDTNNNDNQ LDQFPRRFNL AGNHEQEFLR YQQQSRRRS

c Carbamidomethylation (+57.02)
d Deamidation (NQ) (+0.98)
* Carbamylation (+43.01), Methylation(KR) (+14.02)

Supporting Peptides:

Peptide	Uniq	-10lgP	Mass	Length	ppm	m/z	z	RT	Fraction	Scan	Source File	Area Kontrola 1 traka odozdole najmanja masa	#Feature	#Feature Kontrola 1 traka odozdole najmanja masa	Start	End	PTM
R.GYFGLIFPGC(+57.02)PSTYEEPAQQ(+.98)GR.R	Y	70.15	2474.1211	22	0.0	1238.0679	2	34.97	2	1883	OB4030.raw	1.44E4	2	2	101	122	Carbamidomethylation
R.RPFYSNAPQEIFIQQGR.G	Y	65.25	2050.0383	17	-0.4	684.3531	3	30.06	2	1588	OB4030.raw	5.95E4	1	1	84	100	
R.RPFYSNAPQ(+.98)EIFIQQGR.G	Y	61.14	2051.0225	17	-0.4	684.6812	3	30.66	2	1626	OB4030.raw	5.04E4	1	1	84	100	Deamidation (NQ)
R.RPFYSNAPQEIFIQQ(+.98)GR.G	Y	57.62	2051.0225	17	-0.4	684.6812	3	30.66	2	1609	OB4030.raw	5.04E4	1	1	84	100	
R.GYFGLIFPGC(+57.02)PSTYEEPAQ(+.98)Q(+.98)GR.R	Y	52.75	2475.1052	22	-0.7	826.0417	3	35.14	2	1891	OB4030.raw	1.88E4	2	2	101	122	Carbamidomethylation; Deamidation (NQ)
R.RPFYSNAPQEIFIQ(+.98)QGR.G	Y	52.48	2051.0225	17	-0.4	684.6812	3	30.66	2	1606	OB4030.raw	5.04E4	1	1	84	100	
R.RPFYSN(+.98)APQ(+.98)EIFIQQGR.G	Y	50.21	2052.0063	17	-1.1	685.0087	3	31.20	2	1655	OB4030.raw	7.74E3	1	1	84	100	Deamidation (NQ)
R.GYFGLIFPGC(+57.02)PSTYEEPAQQGR.R	Y	47.59	2473.1372	22	0.5	825.3867	3	34.86	2	1877	OB4030.raw	0	0	0	101	122	Carbamidomethylation
R.GYFGLIFPGC(+57.02)PSTYEEPAQ(+.98)Q(+.98)G.R	Y	46.44	2319.0042	21	5.1	1160.5153	2	37.26	2	2021	OB4030.raw	4.5E3	1	1	101	121	Carbamidomethylation; Deamidation (NQ)
R.GYFGLIFPGC(+57.02)PSTYEEPAQ(+.98)Q(+.98)GRR.H	Y	39.17	2631.2063	23	0.8	878.0768	3	33.51	2	1800	OB4030.raw	5.89E4	2	2	101	123	Carbamidomethylation; Deamidation (NQ)
R.GYFGLIFPGC(+57.02)PSTYEEPAQ(+.98)QGR.R	Y	37.65	2474.1211	22	1.2	825.7153	3	34.97	2	1882	OB4030.raw	1.08E4	1	1	101	122	Carbamidomethylation
R.GYFGLIFPGC(+57.02)PSTYEEPAQQ(+.98)GRR.H	Y	36.99	2630.2224	23	-1.3	877.7469	3	33.35	2	1785	OB4030.raw	0	0	0	101	123	Carbamidomethylation
R.R(+43.01)(+14.02)PFYSNAPQEIFIQQGR.G	Y	36.32	2107.0598	17	0.5	703.3609	3	30.11	2	1592	OB4030.raw	2.12E3	1	1	84	100	Carbamylation; Methylation(KR)
R.GYFGLIFPGC(+57.02)PSTYEEPAQ(+.98)QGRR.H	Y	35.45	2630.2224	23	-0.7	877.7474	3	33.38	2	1787	OB4030.raw	0	0	0	101	123	Carbamidomethylation
R.GYFGLIFPGC(+57.02)PSTYEEPAQ(+.98)Q(+.98).G	Y	35.11	2261.9827	20	-8.4	1131.9891	2	37.33	2	2030	OB4030.raw	0	0	0	101	120	Carbamidomethylation; Deamidation (NQ)
R.GYFGLIFPGC(+57.02)PSTYEEPA.Q	Y	32.67	2003.8975	18	0.1	1002.9561	2	37.60	2	2047	OB4030.raw	0	0	0	101	118	Carbamidomethylation
R.GYFGLIFPGC(+57.02)PSTYEEP(+15.99)AQQ(+.98)GRR.H	Y	28.39	2646.2173	23	-1.3	883.0786	3	34.00	2	1824	OB4030.raw	1.99E3	1	1	101	123	Carbamidomethylation
R.GYFGLIFP(+15.99)GC(+57.02)P(+15.99)STYEEPAQQ(+.98)GRR.H	Y	26.14	2662.2122	23	1.6	888.4128	3	33.52	2	1793	OB4030.raw	7.79E3	1	1	101	123	Carbamidomethylation
R.GYFGLIFPGC(+57.02)P(+15.99)STYEEP(+15.99)AQQ(+.98)GRR.H	Y	21.97	2662.2122	23	1.6	888.4128	3	33.52	2	1796	OB4030.raw	7.79E3	1	1	101	123	Carbamidomethylation
**total 19 peptides**																	

Peptide List

Summary

1. Notes Gastric Digest Raw peanut Band #1 13-15 kDa

2. Result Statistics

Figure 1. False discovery rate (FDR) curve. X axis is the number of peptide-spectrum matches (PSM) being kept. Y axis is the corresponding FDR.

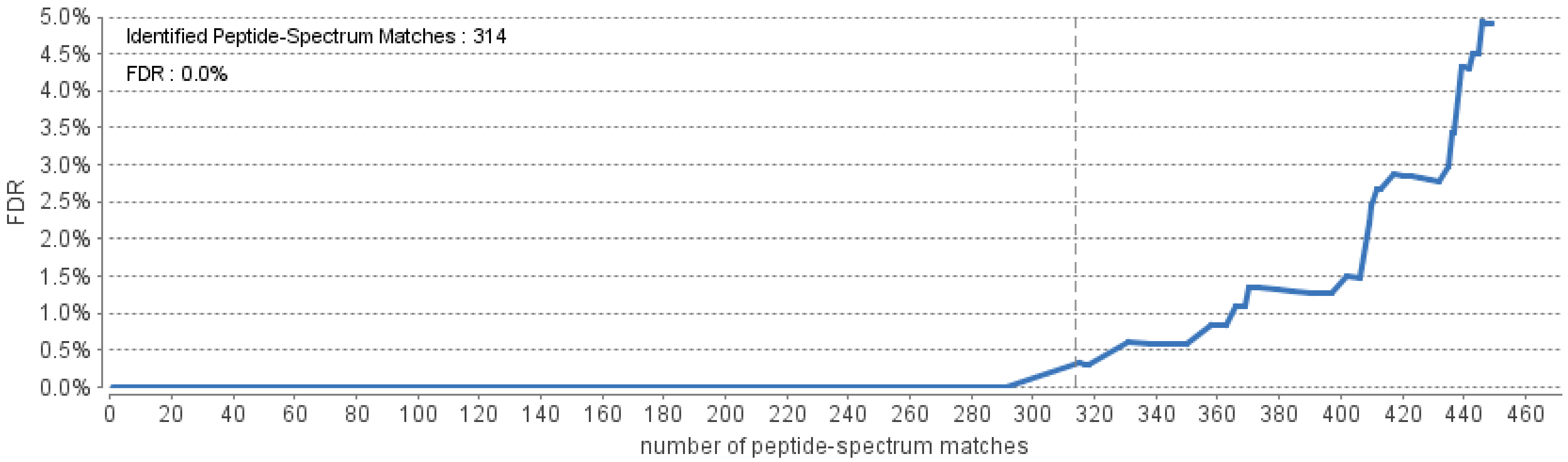

Figure 2. PSM score distribution. **(a)** Distribution of PEAKS peptide score; **(b)** Scatterplot of PEAKS peptide score versus precursor mass error.

(a)

(b)

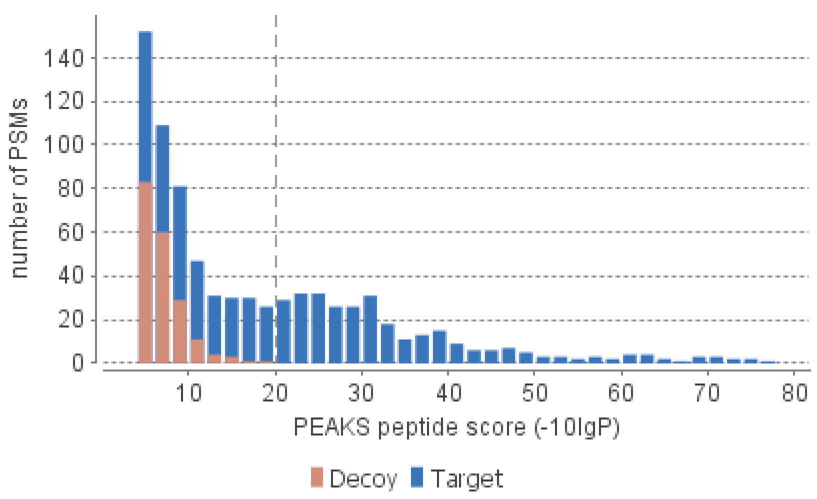

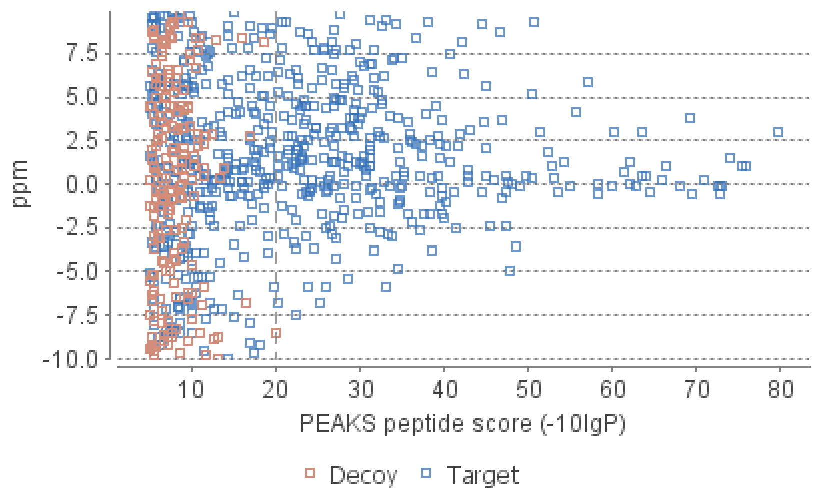

Figure 3. Distribution of peptide feature detection. **(a)** Feature m/z distribution; **(b)** Feature RT distribution

(a)

(b)

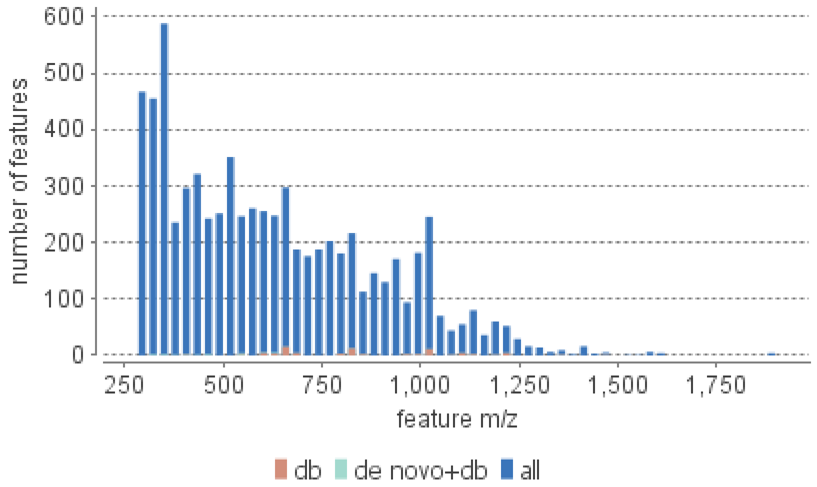

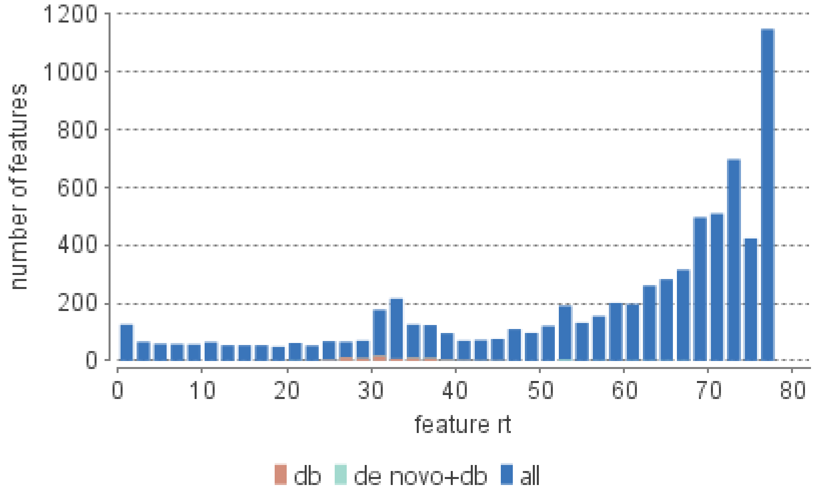

Figure 4. Distribution of identified peptide features. **(a)** Feature abundance distribution **(b)** *De novo* sequencing validation.

(a)

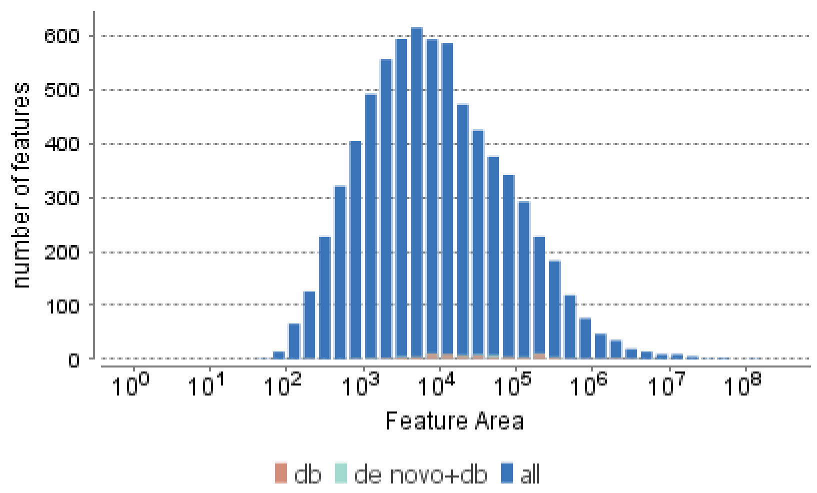

(b)

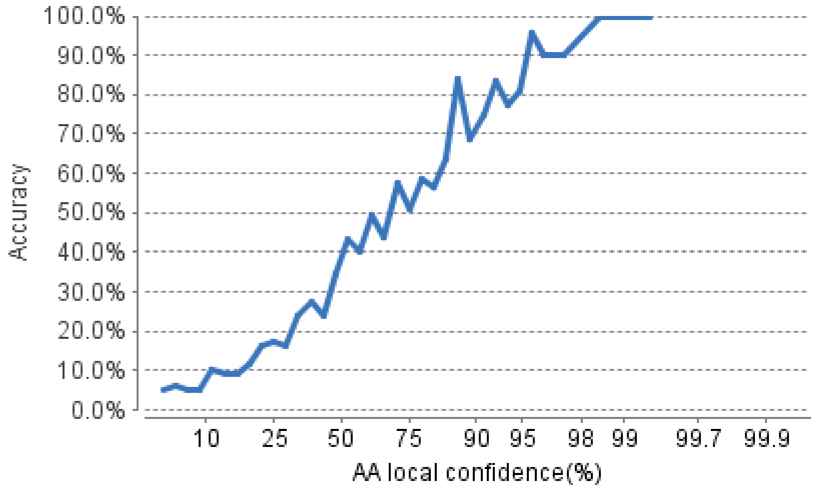

Table 1. Statistics of data.

# of MS scans	2590
# of MS/MS scans	1761
# of Features	7256
# of Chimera scans	269

Table 2. Result filtration parameters.

Peptide -10lgP	≥20.1
PTM Ascore	≥20
Protein -10lgP	≥20
Proteins unique peptides	≥1
De novo ALC Score	≥50%

Table 4. PTM profile.

Name	ΔMass	Position	#PSM	-10lgP	Abundance	AScore
Deamidation	.98	NQ	234	75.95	2.67E5	22.45
Carbamidomethyl	57.02	C	226	79.67	2.94E4	1000.00
HydPro	15.99	P	57	38.58		9.45
Methylation(KR)	14.02	R	11	53.50	5.02E4	122.77
Carbamylation	43.01	N-term	9	53.50	5.02E4	1000.00
Methylation(others)	14.02	E	3	28.03		0.00
Acetylation	42.01	Protein N-term	3	26.78		1000.00

Table 3. Statistics of filtered result.

Peptide-Spectrum Matches	314
Peptide sequences	168
Protein groups	2
Proteins	6
Proteins (#Unique Peptides)	6 (>2); 0 (=2); 0 (=1);
FDR (Peptide-Spectrum Matches)	0.0%
FDR (Peptide Sequences)	0.0%
FDR (Protein Group)	0.0%
De Novo Only Spectra	29
# of identified Features	89
# of identified MS/MS scans	292

Ubiquitin	114.04	C	1	23.25	6.19E3	14.04
Acetylation	42.01	N-term	1	21.91		1000.00
Dimethylation(KR)	28.03	R	1	20.18		12.42
Carbofuran	58.03	S	1	31.50		1000.00
Propionamide	71.04	C	1	22.66	5.12E5	1000.00
Dehydration	-18.01	T	1	25.93	1.43E4	14.84
Deamidation	.98	R	1	21.10	6.36E4	7.21
Sodium	21.98	D	1	25.93	1.43E4	0.00

3. Experiment Control

Figure 5. Precursor mass error of peptide-spectrum matches (PSM) in filtered result. **(a)** Distribution of precursor mass error in ppm; **(b)** Scatterplot of precursor m/z versus precursor mass error in ppm.

(a)

(b)

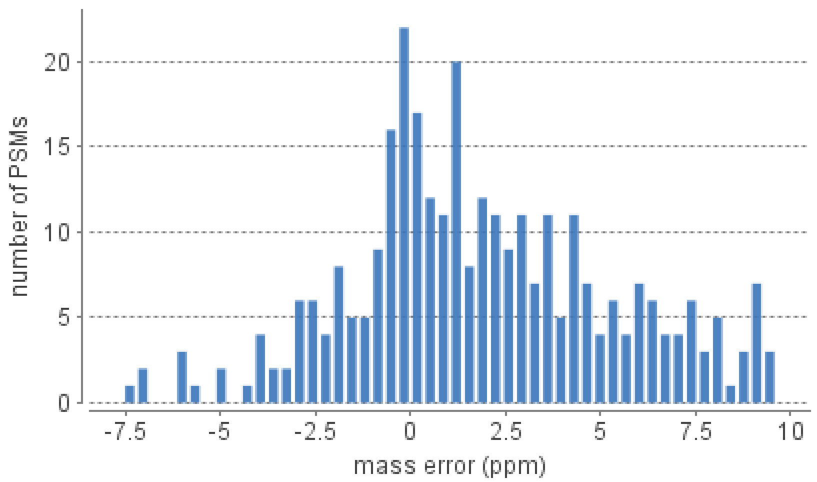

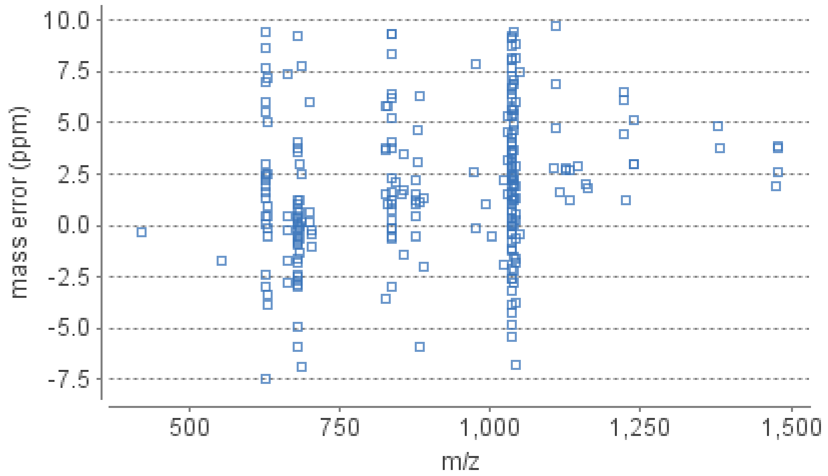

Table 5. Number of identified peptides in each sample by the number of missed cleavages

Missed Cleavages	0	1	2	3	4+
Digest 1. traka 13-15 kDa	35	132	1	0	0

4. Other Information

Table 6. Search parameters.

Search Engine Name: PEAKS
Parent Mass Error Tolerance: 10.0 ppm
Fragment Mass Error Tolerance: 0.5 Da
Precursor Mass Search Type: monoisotopic
Enzyme: Trypsin
Max Missed Cleavages: 2
Digest Mode: Unspecific

Table 7. Instrument parameters.

Fractions: OB4051.raw
Ion Source: ESI(nano-spray)
Fragmentation Mode: CID, CAD(y and b ions)
MS Scan Mode: FT-ICR/Orbitrap
MS/MS Scan Mode: Linear Ion Trap

Fixed Modifications:
  Carbamidomethylation: 57.02
Variable Modifications:
  Deamidation (NQ): 0.98
  Oxidation (M): 15.99
  Hydroxylation Pro: 15.99
  Acetylation (K): 42.01
  Acetylation (Protein N-term): 42.01
  Acetylation (N-term): 42.01
  Amidation: -0.98
  Beta-methylthiolation: 45.99
  and 305 more...
Max Variable PTM Per Peptide: 5
Database: Uniprot_Peanut-3818_Jul18
Taxon: All
Contaminant Database: contaminantsMQ_mar19
Searched Entry: 1723
FDR Estimation: Enabled
De novo score (ALC%) threshold: 15
Peptide hit threshold (-10logP): 30.0
Peaks run ID: 479
Merge Options: no merge
Precursor Options: corrected
Charge Options: no correction
Filter Charge: 2 - 8
Process: true
Associate chimera: yes

Protein List

Protein Accession Contains:

Protein Description Contains:

Peptide Sample Area >=

Protein Ptm Contains:

Protein Group	Protein ID	Accession	-10lgP	Coverage (%)	Coverage (%) Digest 1. traka 13-15 kDa	Area Digest 1. traka 13-15 kDa	#Peptides	#Unique	#Spec Digest 1. traka 13-15 kDa	PTM	Avg. Mass	Description
1	20574	tr|A1DZF0|A1DZF0_ARAHY	203.54	18	18	1.05E7	13	12	235	Y	60375	Arachin 6 OS=Arachis hypogaea OX=3818 PE=2 SV=1
1	20578	tr|B5TYU1|B5TYU1_ARAHY	203.54	18	18	1.05E7	13	12	235	Y	60624	Arachin Arah3 isoform OS=Arachis hypogaea OX=3818 PE=1 SV=1
1	20577	tr|Q9SQH7|Q9SQH7_ARAHY	203.54	18	18	1.05E7	13	12	235	Y	61011	Glycinin OS=Arachis hypogaea OX=3818 GN=Arah4 PE=2 SV=1
2	20593	tr|Q6IWG5|Q6IWG5_ARAHY	129.76	19	19	6.63E6	10	9	77	Y	58061	Glycinin (Fragment) OS=Arachis hypogaea OX=3818 PE=2 SV=1
2	20595	tr|E5G077|E5G077_ARAHY	129.76	19	19	6.63E6	10	9	77	Y	58305	Ara h 3 allergen OS=Arachis hypogaea OX=3818 GN=ara h 3 PE=3 SV=1
2	20594	tr|Q0GM57|Q0GM57_ARAHY	129.76	19	19	6.63E6	10	9	77	Y	58263	Iso-Ara h3 OS=Arachis hypogaea OX=3818 PE=2 SV=1
**total 6 proteins**												

tr|A1DZF0|A1DZF0_ARAHY

back to list

| Protein Coverage | Supporting Peptides |

Protein Coverage:

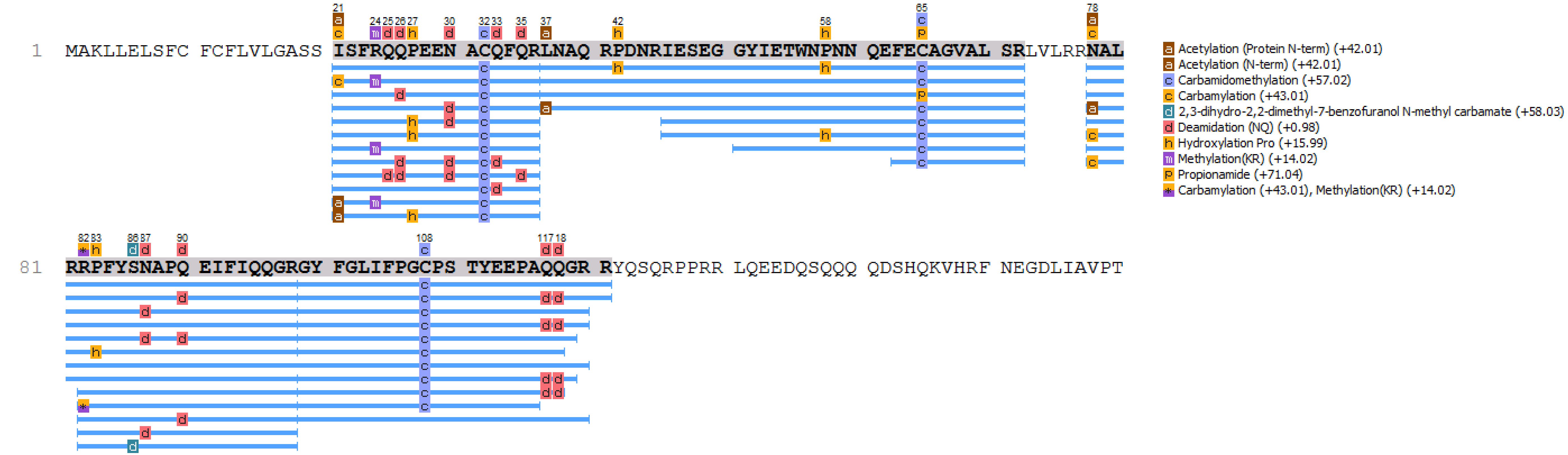

161 GVAFWLYNDH DTDVVAVSLT DTNNNDNQLD QFPRRFNLAG NHEQEFLRYQ QQSRQSRRRS LPLSPYSPQP GQEDREFSPQ

241 GQHGRRERAG QEQENEGGNI FSGFTSEFLA QAFQVDDRQI VQNLRGENES EEQGAIVTVK GGLRILSPDR KSPDEEEEYD

321 EDEYAEEERQ QDRRRGRGSR GSGNGIEETI CTATVKKNIG RNRSPDIYNP QAGSLKTANE LNLLILRWLG LSAEYGNLYR

401 NALFVPHYNT NAHSIIYALR GRAHVQVVDS NGNRVYDEEL QEGHVLVVPQ NFAVAGKSQS ENFEYVAFKT DSRPSIANLA

481 GENSFIDNLP EEVVANSYGL PREQARQLKN NNPFKFFVPP FQQSPRAVA

Supporting Peptides:

Peptide	Uniq	-10lgP	Mass	Length	ppm	m/z	z	RT	Fraction	Scan	Source File	Area Digest 1. traka 13-15 kDa	#Feature	#Feature Digest 1. traka 13-15 kDa	Start	End	PTM
R.GYFGLIFPGC(+57.02)PSTYEEPAQQGR.R	Y	79.67	2473.1372	22	3.0	1237.5796	2	34.90	23	1858	OB4051.raw	2.94E4	1	1	99	120	Carbamidomethylation

Peptide	Uniq	-10lgP	Mass	Length	ppm	m/z	z	RT	Fraction	Scan	Source File	Area Digest 1. traka 13-15 kDa	#Feature	#Feature Digest 1. traka 13-15 kDa	Start	End	PTM
R.NALRRPFYSN(+.98)APQEIFIQQGR.G	Y	75.95	2505.2876	21	1.0	836.1040	3	30.01	23	1564	OB4051.raw	2.67E5	1	1	78	98	Deamidation (NQ)
R.RPFYSNAPQEIFIQQGR.G	Y	73.07	2050.0383	17	-0.1	684.3533	3	30.09	23	1551	OB4051.raw	3.6E5	1	1	82	98	
R.RPFYSNAPQ(+.98)EIFIQQGR.G	Y	72.83	2051.0225	17	-0.6	684.6810	3	30.68	23	1600	OB4051.raw	2.49E5	1	1	82	98	Deamidation (NQ)
R.NALRRPFYSNAPQEIFIQQGR.G	Y	72.48	2504.3037	21	-0.1	835.7751	3	28.99	23	1488	OB4051.raw	2.43E6	4	4	78	98	
S.ISFRQQPEENAC(+57.02)QFQR.L	Y	64.02	2036.9486	16	0.5	679.9905	3	26.48	23	1325	OB4051.raw	9.82E4	2	2	21	36	Carbamidomethylation
S.ISFRQ(+.98)QPEENAC(+57.02)QFQR.L	Y	62.11	2037.9326	16	0.0	680.3182	3	26.91	23	1368	OB4051.raw	2.38E5	2	2	21	36	Carbamidomethylation
S.ISFRQQ(+.98)PEENAC(+57.02)QFQR.L	Y	58.19	2037.9326	16	0.0	680.3182	3	26.91	23	1357	OB4051.raw	2.25E5	1	1	21	36	Deamidation (NQ); Carbamidomethylation
R.GYFGLIFPGC(+57.02)PSTYEEPAQ(+.98)QGR.R	Y	57.05	2474.1211	22	5.8	825.7191	3	35.00	23	1864	OB4051.raw	1.59E5	2	2	99	120	Carbamidomethylation
R.N(+.98)ALRRPFYSNAPQEIFIQQGR.G	Y	55.69	2505.2876	21	4.1	836.1066	3	31.18	23	1625	OB4051.raw	4.28E3	2	2	78	98	
R.R(+43.01)(+14.02)PFYSNAPQEIFIQQGR.G	Y	53.50	2107.0598	17	-0.5	703.3602	3	30.09	23	1554	OB4051.raw	5.02E4	1	1	82	98	Carbamylation; Methylation(KR)
S.ISFRQQ(+.98)PEEN(+.98)AC(+57.02)QFQR.L	Y	53.01	2038.9166	16	0.3	680.6464	3	27.28	23	1386	OB4051.raw	1.86E5	2	2	21	36	Deamidation (NQ); Carbamidomethylation
R.GYFGLIFPGC(+57.02)PSTYEEPAQ(+.98)Q(+.98)G.R	Y	52.28	2319.0042	21	1.8	1160.5115	2	37.27	23	2003	OB4051.raw	2.38E4	1	1	99	119	Carbamidomethylation; Deamidation (NQ)
R.GYFGLIFPGC(+57.02)PSTYEEPAQ(+.98)Q(+.98)GR.R	Y	51.37	2475.1052	22	3.0	1238.5636	2	35.20	23	1876	OB4051.raw	4.43E4	1	1	99	120	Carbamidomethylation; Deamidation (NQ)
R.RPFYSN(+.98)APQ(+.98)EIFIQQGR.G	Y	49.05	2052.0063	17	-0.2	685.0093	3	31.25	23	1630	OB4051.raw	6.55E4	1	1	82	98	Deamidation (NQ)
S.ISFRQQPEEN(+.98)AC(+57.02)QFQR.L	Y	47.83	2037.9326	16	-4.9	680.3148	3	28.27	23	1442	OB4051.raw	1.31E4	1	1	21	36	Deamidation (NQ); Carbamidomethylation
R.LNAQ(+.98)RPDNRIESEGGYIETWN(+.98)PNNQ(+.98)EFEC(+57.02)AGVALSR.L	Y	46.74	4136.8770	36	8.8	1035.2356	4	32.19	23	1690	OB4051.raw	0	0	0	37	72	Carbamidomethylation
R.LN(+.98)AQRPDNRIESEGGYIETWN(+.98)PNNQEFEC(+57.02)AGVALSR.L	Y	45.09	4135.8931	36	5.6	1034.9863	4	32.01	23	1678	OB4051.raw	0	0	0	37	72	Carbamidomethylation
R.GYFGLIFPGC(+57.02)PSTYEEPAQQ(+.98)G.R	Y	44.88	2318.0200	21	2.0	1160.0197	2	37.17	23	1993	OB4051.raw	1.14E4	1	1	99	119	Carbamidomethylation
S.ISFRQ(+.98)QPEEN(+.98)AC(+57.02)QFQR.L	Y	44.79	2038.9166	16	3.5	680.6486	3	29.66	23	1529	OB4051.raw	9.26E2	1	1	21	36	Deamidation (NQ); Carbamidomethylation
R.NALRRPFYSNAPQ(+.98)EIFIQQGR.G	Y	44.58	2505.2876	21	-0.4	836.1028	3	32.63	23	1718	OB4051.raw	0	0	0	78	98	Deamidation (NQ)
S.ISFRQ(+.98)QPEEN(+.98)AC(+57.02)Q(+.98)FQR.L	Y	44.56	2039.9006	16	9.2	680.9804	3	28.92	23	1483	OB4051.raw	0	0	0	21	36	Deamidation (NQ); Carbamidomethylation
R.GYFGLIFPGC(+57.02)PSTYEEPAQ(+.98)Q(+.98)GRR.Y	Y	43.19	2631.2063	23	3.1	878.0787	3	33.53	23	1774	OB4051.raw	0	0	0	99	121	Carbamidomethylation; Deamidation (NQ)

Peptide	Uniq	-10lgP	Mass	Length	ppm	m/z	z	RT	Fraction	Scan	Source File	Area Digest 1. traka 13-15 kDa	#Feature	#Feature Digest 1. traka 13-15 kDa	Start	End	PTM
R.LN(+.98)AQRPDNRIESEGGYIETWNPNNQEFEC(+57.02)AGVALSR.L	Y	41.87	4134.9087	36	2.9	1034.7374	4	31.53	23	1648	OB4051.raw	3.08E4	2	2	37	72	Carbamidomethylation
R.GYFGLIFPGC(+57.02)PSTYEEPAQQ(+.98)GRR.Y	Y	41.54	2630.2224	23	2.2	877.7500	3	34.22	23	1816	OB4051.raw	3.68E5	1	1	99	121	Carbamidomethylation
S.ISFRQQ(+.98)PEENAC(+57.02)Q(+.98)FQR.L	Y	41.38	2038.9166	16	-2.5	680.6445	3	28.10	23	1431	OB4051.raw	0	0	0	21	36	Carbamidomethylation; Deamidation (NQ)
S.ISFRQQ(+.98)PEEN(+.98)AC(+57.02)Q(+.98)FQR.L	Y	41.26	2039.9006	16	-0.5	680.9738	3	27.71	23	1397	OB4051.raw	7.37E4	1	1	21	36	Deamidation (NQ); Carbamidomethylation
R.R(+43.01)(+14.02)PFYSNAPQ(+.98)EIFIQQGR.G	Y	40.22	2108.0439	17	-1.0	703.6879	3	30.76	23	1595	OB4051.raw	3.2E4	1	1	82	98	Carbamylation; Methylation(KR)
R.IESEGGYIETWNPNNQEFEC(+57.02)AGVALSR.L	Y	40.07	3069.3774	27	-1.9	1024.1311	3	33.27	23	1757	OB4051.raw	0	0	0	46	72	Carbamidomethylation
S.ISFRQQPEENAC(+57.02)QFQ(+.98)R.L	Y	39.69	2037.9326	16	-0.2	680.3180	3	28.34	23	1446	OB4051.raw	0	0	0	21	36	Carbamidomethylation
R.LNAQRPDN(+.98)RIESEGGYIETWNPNNQEFEC(+57.02)AGVALSR.L	Y	39.68	4134.9087	36	3.7	1034.7383	4	33.05	23	1744	OB4051.raw	2.12E4	1	1	37	72	Carbamidomethylation
R.LNAQRPDNRIESEGGYIETWN(+.98)PN(+.98)NQEFEC(+57.02)AGVALSR.L	Y	39.41	4135.8931	36	2.3	1034.9829	4	34.12	23	1810	OB4051.raw	0	0	0	37	72	Carbamidomethylation
R.NALRRPFYSNAP(+15.99)QEIFIQQGR.G	Y	38.58	2520.2986	21	-3.8	631.0795	4	29.10	23	1495	OB4051.raw	0	0	0	78	98	
R.LNAQ(+.98)RPDNRIESEGGYIETWNPNNQEFEC(+57.02)AGVALSR.L	Y	38.53	4134.9087	36	0.4	1034.7349	4	36.39	23	1948	OB4051.raw	0	0	0	37	72	Carbamidomethylation
R.N(+.98)ALRRPFYSN(+.98)APQ(+.98)EIFIQQGR.G	Y	38.15	2507.2556	21	9.4	836.7670	3	30.48	23	1602	OB4051.raw	6.36E4	1	1	78	98	Deamidation (NQ)
R.LNAQRPDNRIESEGGYIETWNPNN(+.98)QEFEC(+57.02)AGVALSR.L	Y	37.30	4134.9087	36	2.2	1034.7367	4	36.98	23	1984	OB4051.raw	0	0	0	37	72	Carbamidomethylation
R.NALRRPFYSN(+.98)APQ(+.98)EIFIQQGR.G	Y	36.66	2506.2717	21	8.4	836.4382	3	41.06	23	2232	OB4051.raw	1.61E6	2	2	78	98	Deamidation (NQ)
R.LNAQRPDN(+.98)RIESEGGYIETWNP(+15.99)NNQEFEC(+57.02)AGVALSR.L	Y	36.19	4150.9038	36	-2.2	1038.7310	4	31.09	23	1619	OB4051.raw	0	0	0	37	72	Carbamidomethylation
R.LNAQRPDNRIESEGGYIETWNPN(+.98)NQEFEC(+57.02)AGVALSR.L	Y	35.80	4134.9087	36	3.6	1034.7382	4	37.81	23	2034	OB4051.raw	0	0	0	37	72	Carbamidomethylation
R.NALRRP(+15.99)FYSNAPQEIFIQQGR.G	Y	35.26	2520.2986	21	0.5	631.0822	4	29.17	23	1499	OB4051.raw	0	0	0	78	98	
S.I(+43.01)SFR(+14.02)QQPEENAC(+57.02)QFQR.L	Y	35.15	2093.9700	16	0.7	698.9977	3	26.67	23	1335	OB4051.raw	1.01E4	1	1	21	36	Carbamylation; Methylation(KR); Carbamidomethylation
R.LNAQRPDN(+.98)RIESEGGYIETWNPN(+.98)NQEFEC(+57.02)AGVALSR.L	Y	34.92	4135.8931	36	-0.1	1034.9805	4	40.13	23	2176	OB4051.raw	0	0	0	37	72	Carbamidomethylation
R.GYFGLIFPGC(+57.02)PSTYEEPAQ(+.98)Q(+.98).G	Y	34.77	2261.9827	20	1.2	1132.0000	2	37.47	23	2014	OB4051.raw	8.2E3	1	1	99	118	Carbamidomethylation; Deamidation (NQ)
R.R(+43.01)(+14.02)PFYSNAPQEIFIQ(+.98)QGR.G	Y	34.75	2108.0439	17	-1.0	703.6879	3	30.76	23	1583	OB4051.raw	3.2E4	1	1	82	98	Carbamylation; Methylation(KR)
R.LNAQRPDNRIESEGGYIETWNP(+15.99)N(+.98)NQEFEC(+57.02)AGVALSR.L	Y	34.61	4150.9038	36	2.0	1038.7354	4	30.83	23	1603	OB4051.raw	0	0	0	37	72	Carbamidomethylation
R.LNAQRPDNRIESEGGYIETWNPNNQEFEC(+57.02)AGVALSR.L	Y	34.48	4133.9248	36	-4.8	1034.4835	4	36.92	23	1980	OB4051.raw	1.59E4	1	1	37	72	Carbamidomethylation

Peptide	Uniq	-10lgP	Mass	Length	ppm	m/z	z	RT	Fraction	Scan	Source File	Area Digest 1. traka 13-15 kDa	#Feature	#Feature Digest 1. traka 13-15 kDa	Start	End	PTM
R.LNAQRPDN(+.98)RIESEGGYIETWN(+.98)PNNQEFEC(+57.02)AGVALSR.L	Y	34.30	4135.8931	36	9.3	1034.9901	4	35.72	23	1907	OB4051.raw	0	0	0	37	72	Carbamidomethylation
R.LN(+.98)AQRPDNRIESEGGYIETWNPNNQ(+.98)EFEC(+57.02)AGVALSR.L	Y	34.13	4135.8931	36	7.2	1034.9880	4	36.21	23	1937	OB4051.raw	0	0	0	37	72	Carbamidomethylation
S.ISFRQ(+.98)Q(+.98)PEEN(+.98)AC(+57.02)QFQ(+.98)R.L	Y	33.31	2040.8846	16	-2.9	681.3002	3	27.96	23	1423	OB4051.raw	2.33E4	1	1	21	36	Deamidation (NQ); Carbamidomethylation
S.ISFRQ(+.98)QPEENAC(+57.02)Q(+.98)FQR.L	Y	33.09	2038.9166	16	-5.9	680.6422	3	30.14	23	1560	OB4051.raw	0	0	0	21	36	Carbamidomethylation; Deamidation (NQ)
R.LNAQRPDN(+.98)RIESEGGYIETWN(+.98)P(+15.99)NNQEFEC(+57.02)AGVALSR.L	Y	32.99	4151.8877	36	9.5	1038.9890	4	31.01	23	1614	OB4051.raw	0	0	0	37	72	Carbamidomethylation
R.N(+.98)ALRRPFYSN(+.98)APQEIFIQQGR.G	Y	32.73	2506.2717	21	6.2	836.4363	3	31.45	23	1643	OB4051.raw	0	0	0	78	98	
R.LNAQRP(+15.99)DNRIESEGGYIETWN(+.98)PNNQEFEC(+57.02)AGVALSR.L	Y	32.26	4150.9038	36	-2.8	1038.7303	4	34.46	23	1830	OB4051.raw	0	0	0	37	72	Carbamidomethylation
R.RPFYS(+58.03)NAPQEIFIQQGR.G	Y	31.50	2108.0676	17	-0.2	703.6964	3	31.14	23	1622	OB4051.raw	0	0	0	82	98	2,3-dihydro-2,2-dimethyl-7-benzofuranol N-methyl carbamate
R.GYFGLIFPGC(+57.02)PSTYEEPAQ(+.98)QGRR.Y	Y	31.26	2630.2224	23	1.0	877.7490	3	34.87	23	1855	OB4051.raw	0	0	0	99	121	Carbamidomethylation
R.LNAQRPDNR(+14.02)IESEGGYIETWNP(+15.99)NNQEFEC(+57.02)AGVALSR.L	Y	30.81	4163.9355	36	-1.6	1041.9895	4	33.36	23	1763	OB4051.raw	0	0	0	37	72	Carbamidomethylation
R.GYFGLIFPGC(+57.02)PSTYEEP(+15.99)AQQGRR.Y	Y	30.42	2645.2332	23	1.1	882.7526	3	33.56	23	1776	OB4051.raw	0	0	0	99	121	Carbamidomethylation
S.ISFRQQ(+.98)PEEN(+.98)AC(+57.02)QFQ(+.98)R.L	Y	29.88	2039.9006	16	1.2	680.9750	3	29.35	23	1508	OB4051.raw	3.32E3	1	1	21	36	Carbamidomethylation
R.N(+43.01)ALRRP(+15.99)FYSNAPQEIFIQQGR.G	Y	29.70	2563.3042	21	3.4	855.4449	3	30.68	23	1596	OB4051.raw	1.48E4	1	1	78	98	Carbamylation; Hydroxylation Pro
R.NALRRPFYSNAP(+15.99)Q(+.98)EIFIQQGR.G	Y	29.57	2521.2825	21	-0.1	631.3278	4	30.10	23	1558	OB4051.raw	0	0	0	78	98	
R.LNAQ(+.98)RPDNRIESEGGYIETWNP(+15.99)NNQEFEC(+57.02)AGVALSR.L	Y	29.52	4150.9038	36	4.6	1038.7380	4	31.90	23	1812	OB4051.raw	8.71E5	1	1	37	72	Carbamidomethylation
R.LNAQRPDNRIESEGGYIETWNPNNQ(+.98)EFEC(+57.02)AGVALSR.L	Y	29.43	4134.9087	36	8.1	1034.7428	4	36.60	23	1961	OB4051.raw	0	0	0	37	72	Carbamidomethylation
S.ISFRQ(+.98)Q(+.98)PEENAC(+57.02)QFQR.L	Y	29.41	2038.9166	16	-0.9	680.6456	3	29.88	23	1544	OB4051.raw	0	0	0	21	36	Carbamidomethylation
R.N(+.98)ALRRPFYSNAPQ(+.98)EIFIQQGR.G	Y	28.90	2506.2717	21	7.7	627.5800	4	30.01	23	1573	OB4051.raw	1.61E6	1	1	78	98	
G.GYIETWNPNN(+.98)QEFEC(+57.02)AGVALSR.L	Y	28.67	2555.1387	22	1.5	852.7214	3	33.02	23	1736	OB4051.raw	6.62E3	1	1	51	72	Carbamidomethylation
R.LNAQ(+.98)RPDN(+.98)RIESEGGYIETWNPNN(+.98)QEFEC(+57.02)AGVALSR.L	Y	28.06	4136.8770	36	5.8	828.3875	5	31.70	23	1646	OB4051.raw	3.18E4	1	1	37	72	Carbamidomethylation
R.LNAQRPDN(+.98)RIESEGGYIETWNPNNQEFE(+14.02)C(+57.02)AGVALSR.L	Y	28.03	4148.9243	36	5.6	1038.2441	4	33.09	23	1746	OB4051.raw	0	0	0	37	72	Carbamidomethylation
R.LN(+.98)AQRPDN(+.98)RIESEGGYIETWNPNNQEFEC(+57.02)AGVALSR.L	Y	27.87	4135.8931	36	1.6	1034.9822	4	39.40	23	2131	OB4051.raw	0	0	0	37	72	Carbamidomethylation
R.GYFGLIFP(+15.99)GC(+57.02)P(+15.99)STYEEPAQQ(+.98)GRR.Y	Y	27.78	2662.2122	23	-2.0	888.4095	3	33.73	23	1786	OB4051.raw	0	0	0	99	121	Carbamidomethylation
R.LNAQRPDNRIESEGGYIETWN(+.98)PNNQEFEC(+57.02)AGVALSR.L	Y	27.74	4134.9087	36	4.9	1379.3169	3	31.34	23	1655	OB4051.raw	5.03E4	2	2	37	72	Carbamidomethylation

Peptide	Uniq	-10lgP	Mass	Length	ppm	m/z	z	RT	Fraction	Scan	Source File	Area Digest 1. traka 13-15 kDa	#Feature	#Feature Digest 1. traka 13-15 kDa	Start	End	PTM
R.GYFGLIFPGC(+57.02)P(+15.99)STYEEP(+15.99)AQ(+.98)QGRR.Y	Y	27.21	2662.2122	23	1.3	888.4125	3	33.54	23	1775	OB4051.raw	0	0	0	99	121	Carbamidomethylation
R.LNAQRPDNRIESEGGYIETWN(+.98)PNN(+.98)QEFEC(+57.02)AGVALSR.L	Y	27.03	4135.8931	36	-4.2	1034.9762	4	39.60	23	2143	OB4051.raw	0	0	0	37	72	Carbamidomethylation
R.LNAQ(+.98)RP(+15.99)DNRIESEGGYIETWNP(+15.99)NNQEFEC(+57.02)AGVALSR.L	Y	26.88	4166.8989	36	-1.8	1042.7301	4	32.02	23	1679	OB4051.raw	0	0	0	37	72	Hydroxylation Pro; Carbamidomethylation
R.LNAQRPDNRIESEGGYIETWNP(+15.99)NNQEFEC(+57.02)AGVALSR.L	Y	26.81	4149.9199	36	2.2	1038.4895	4	32.87	23	1733	OB4051.raw	0	0	0	37	72	Carbamidomethylation
R.LNAQRP(+15.99)DNRIESEGGYIETWN(+.98)P(+15.99)NNQ(+.98)EFEC(+57.02)AGVALSR.L	Y	26.81	4167.8828	36	8.8	1042.9872	4	30.91	23	1608	OB4051.raw	0	0	0	37	72	Hydroxylation Pro; Carbamidomethylation
R.L(+42.01)NAQRP(+15.99)DNRIESEGGYIETWNPN(+.98)NQEFEC(+57.02)AGVALSR.L	Y	26.78	4192.9146	36	7.5	1049.2438	4	32.27	23	1696	OB4051.raw	0	0	0	37	72	Acetylation (N-term); Carbamidomethylation
R.LNAQRP(+15.99)DNRIESEGGYIETWNP(+15.99)NNQ(+.98)EFEC(+57.02)AGVALSR.L	Y	26.30	4166.8989	36	0.3	1042.7323	4	30.28	23	1569	OB4051.raw	0	0	0	37	72	Hydroxylation Pro; Carbamidomethylation
R.GYFGLIFP(+15.99)GC(+57.02)PSTYEEPAQQ(+.98)GRR.Y	Y	26.01	2646.2173	23	-5.9	883.0745	3	34.01	23	1805	OB4051.raw	1.9E4	1	1	99	121	Carbamidomethylation
R.LNAQRPD(+21.98)NRIESEGGYIET(-18.01)WNPNNQEFEC(+57.02)AGVALSR.L	Y	25.93	4137.8965	36	4.4	1035.4860	4	37.86	23	2034	OB4051.raw	1.43E4	1	1	37	72	Carbamidomethylation
E.FEC(+57.02)AGVALSR.L	Y	25.38	1108.5334	10	-1.7	555.2731	2	27.11	23	1369	OB4051.raw	1.24E3	1	1	63	72	Carbamidomethylation
S.ISFRQQP(+15.99)EENAC(+57.02)QFQR.L	Y	25.30	2052.9436	16	-6.8	685.3171	3	28.02	23	1432	OB4051.raw	5.73E3	1	1	21	36	Hydroxylation Pro; Carbamidomethylation
R.IESEGGYIETWNP(+15.99)N(+.98)N(+.98)QEFEC(+57.02)AGVALSR.L	Y	24.66	3087.3403	27	3.2	1030.1240	3	34.08	23	1807	OB4051.raw	0	0	0	46	72	Hydroxylation Pro; Carbamidomethylation
R.LNAQRP(+15.99)DNRIESEGGYIETWNP(+15.99)NNQEFEC(+57.02)AGVALSR.L	Y	24.62	4165.9146	36	2.9	1042.4889	4	31.43	23	1642	OB4051.raw	0	0	0	37	72	Hydroxylation Pro; Carbamidomethylation
R.LNAQ(+.98)RP(+15.99)DNRIESEGGYIETWN(+.98)P(+15.99)NNQEFEC(+57.02)AGVALSR.L	Y	24.48	4167.8828	36	-3.7	1042.9741	4	31.99	23	1679	OB4051.raw	1.81E5	1	1	37	72	Hydroxylation Pro; Carbamidomethylation
S.ISFRQQP(+15.99)EEN(+.98)AC(+57.02)QFQR.L	Y	24.24	2053.9275	16	0.2	685.6499	3	28.24	23	1440	OB4051.raw	0	0	0	21	36	Hydroxylation Pro; Deamidation (NQ); Carbamidomethylation
R.LNAQRP(+15.99)DN(+.98)RIESEGGYIETWNP(+15.99)NNQEFEC(+57.02)AGVALSR.L	Y	24.19	4166.8989	36	1.3	1042.7334	4	30.56	23	1587	OB4051.raw	0	0	0	37	72	Hydroxylation Pro; Carbamidomethylation
R.N(+.98)ALRRPFYSNAP(+15.99)QEIFIQQGR.G	Y	24.11	2521.2825	21	-0.5	631.3276	4	30.21	23	1565	OB4051.raw	0	0	0	78	98	
R.GYFGLIFPGC(+57.02)PSTYEEP(+15.99)AQQ(+.98)GRR.Y	Y	23.79	2646.2173	23	-5.9	883.0745	3	34.01	23	1811	OB4051.raw	1.9E4	1	1	99	121	Carbamidomethylation
S.ISFRQQ(+.98)P(+15.99)EENAC(+57.02)QFQR.L	Y	23.77	2053.9275	16	2.5	685.6515	3	27.42	23	1393	OB4051.raw	7.88E3	1	1	21	36	Hydroxylation Pro; Carbamidomethylation
R.LNAQRPDNRIESEGGYIE(+14.02)TWNPNNQ(+.98)EFEC(+57.02)AGVALSR.L	Y	23.52	4148.9243	36	3.7	1038.2422	4	33.94	23	1799	OB4051.raw	0	0	0	37	72	Carbamidomethylation

Peptide	Uniq	-10lgP	Mass	Length	ppm	m/z	z	RT	Fraction	Scan	Source File	Area Digest 1. traka 13-15 kDa	#Feature	#Feature Digest 1. traka 13-15 kDa	Start	End	PTM
S.I(+42.01)SFRQQP(+15.99)EENAC(+57.02)QFQR.L	Y	23.36	2094.9541	16	0.2	699.3254	3	27.03	23	1365	OB4051.raw	3.45E4	1	1	21	36	Acetylation (Protein N-term); Hydroxylation Pro; Carbamidomethylation
R.GYFGLIFPGC(+114.04)PSTYEEPAQQGR.R	Y	23.25	2530.1587	22	2.2	844.3953	3	34.90	23	1861	OB4051.raw	6.19E3	1	1	99	120	
R.GYFGLIFPGC(+57.02)PSTYEEPA.Q	N	23.12	2003.8975	18	-0.6	1002.9554	2	37.57	23	2023	OB4051.raw	8.65E3	1	1	99	116	Carbamidomethylation
R.LN(+.98)AQRPDNRIESEGGYIETWNPNN(+.98)Q(+.98)EFEC(+57.02)AGVALSR.L	Y	22.78	4136.8770	36	0.8	1035.2273	4	37.68	23	2026	OB4051.raw	0	0	0	37	72	Carbamidomethylation
R.LNAQRPDN(+.98)RIESEGGYIETWNPNN(+.98)Q(+.98)EFEC(+57.02)AGVALSR.L	Y	22.70	4136.8770	36	5.8	828.3875	5	31.70	23	1660	OB4051.raw	3.18E4	1	1	37	72	Carbamidomethylation
R.LNAQRPDNRIESEGGYIETWNPNNQEFEC(+71.04)AGVALSR.L	Y	22.66	4147.9404	36	1.3	1037.9938	4	31.61	23	1756	OB4051.raw	5.12E5	1	1	37	72	Propionamide
S.I(+42.01)SFR(+14.02)QQ(+.98)PEENAC(+57.02)QFQR.L	Y	22.62	2093.9587	16	6.0	698.9977	3	26.67	23	1339	OB4051.raw	1.01E4	1	1	21	36	Acetylation (Protein N-term); Methylation(KR); Carbamidomethylation
R.IESEGGYIETWN(+.98)P(+15.99)NNQEFEC(+57.02)AGVALSR.L	Y	22.43	3086.3562	27	4.6	1029.7974	3	33.78	23	1789	OB4051.raw	0	0	0	46	72	Hydroxylation Pro; Carbamidomethylation
R.GYFGLIFPGC(+57.02)PSTYEEPAQQ(+.98).G	Y	21.93	2260.9985	20	2.7	1131.5096	2	37.27	23	2000	OB4051.raw	6.39E3	1	1	99	118	Carbamidomethylation
R.N(+42.01)ALRRP(+15.99)FYSNAPQEIFIQQGR.G	Y	21.91	2562.3091	21	-1.4	855.1091	3	30.71	23	1596	OB4051.raw	0	0	0	78	98	Acetylation (N-term)
S.ISFRQQ(+.98)PEENAC(+57.02)Q(+.98)FQ(+.98)R.L	Y	21.84	2039.9006	16	1.2	680.9750	3	29.35	23	1522	OB4051.raw	3.32E3	1	1	21	36	Carbamidomethylation
R.GYFGLIFPGC(+57.02)P(+15.99)STYEEPAQQGRR.Y	Y	21.73	2645.2332	23	6.3	882.7572	3	34.59	23	1838	OB4051.raw	0	0	0	99	121	Carbamidomethylation
R.N(+.98)ALRRP(+15.99)FYSNAPQEIFIQQGR.G	Y	21.53	2521.2825	21	5.0	631.3311	4	29.41	23	1505	OB4051.raw	7.76E3	1	1	78	98	
R.LNAQRPDNRIESEGGYIETWNPNN(+.98)Q(+.98)EFEC(+57.02)AGVALSR.L	Y	21.45	4135.8931	36	1.1	828.1868	5	31.71	23	1660	OB4051.raw	0	0	0	37	72	Carbamidomethylation
R.N(+43.01)ALR(+14.02)RPFYSNAPQEIFIQQGR.G	Y	21.38	2561.3250	21	1.7	854.7838	3	30.09	23	1556	OB4051.raw	1.19E4	1	1	78	98	Carbamylation
R.N(+.98)ALRR(+.98)PFYSN(+.98)APQEIFIQQGR.G	Y	21.10	2507.2556	21	9.4	836.7670	3	30.48	23	1625	OB4051.raw	6.36E4	1	1	78	98	Deamidation (NQ)
S.ISFR(+14.02)Q(+.98)QPEENAC(+57.02)QFQR.L	Y	20.79	2051.9482	16	-1.3	684.9891	3	27.68	23	1404	OB4051.raw	0	0	0	21	36	Methylation(KR); Carbamidomethylation
R.GYFGLIFP(+15.99)GC(+57.02)P(+15.99)STYEEPAQ(+.98)QGR.R	Y	20.70	2506.1111	22	-0.1	836.3776	3	35.10	23	1874	OB4051.raw	7.14E3	1	1	99	120	Carbamidomethylation
R.NALRRP(+15.99)FYSNAPQ(+.98)EIFIQQGR.G	Y	20.56	2521.2825	21	7.1	631.3324	4	28.64	23	1466	OB4051.raw	0	0	0	78	98	
R.LNAQRPDNRIESEGGYIETWNPN(+.98)N(+.98)QEFEC(+57.02)AGVALSR.L	Y	20.32	4135.8931	36	6.4	1034.9872	4	40.80	23	2218	OB4051.raw	0	0	0	37	72	Carbamidomethylation
R.LNAQRPDNR(+28.03)IESEGGYIETWNPNNQEFEC(+57.02)AGVALSR.L	Y	20.18	4161.9561	36	-6.8	1041.4893	4	34.49	23	1832	OB4051.raw	0	0	0	37	72	Carbamidomethylation
R.NALRRPFYSNAPQEIFIQQ(+.98)GRGYFGLIFPGC(+57.02)PSTYEEPAQQGR.R	Y	20.13	4960.4141	43	1.0	993.0911	5	34.71	23	1822	OB4051.raw	2.3E5	1	1	78	120	Carbamidomethylation
**total 112 peptides**																	

tr|B5TYU1|B5TYU1_ARAHY
back to list

| Protein Coverage | Supporting Peptides |

Protein Coverage:

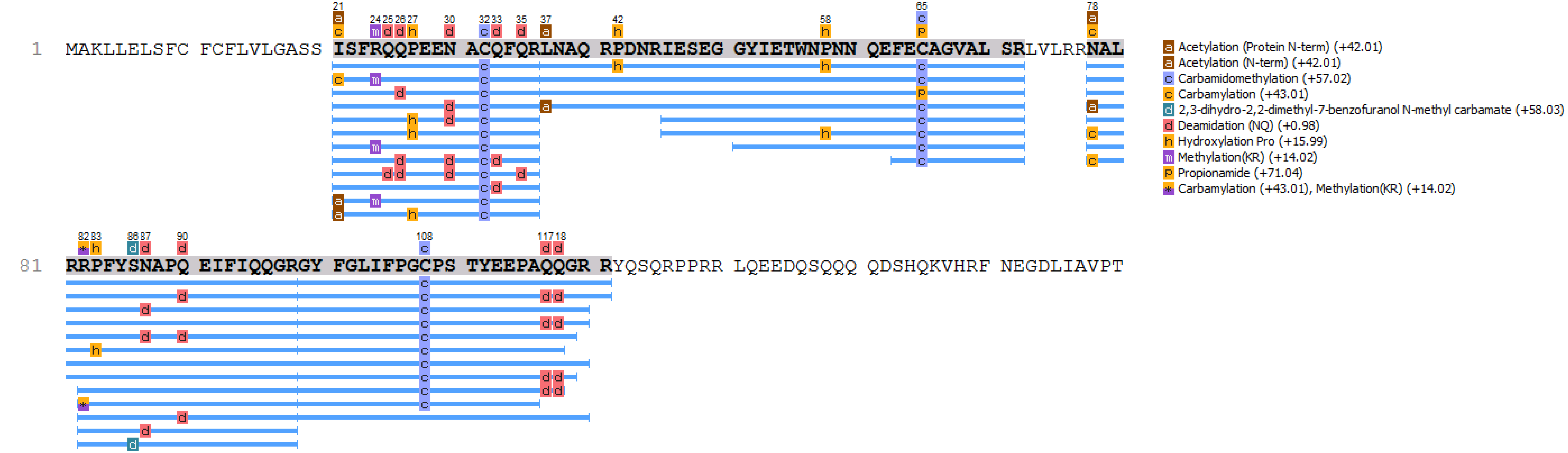

161 GVAFWLYNDH DTDVVAVSLT DTNNNDNQLD QFPRRFNLAG NHEQEFLRYQ QQSRQSRRRS LPYSPYSPQS QPRQEEREFS
241 PRGQHSRRER AGQEEENEGG NIFSGFTPEF LAQAFQVDDR QIVQNLRGEN ESEEQGAIVT VRGGLRILSP DRKRGADEEE
321 EYDEDEYEYD EEDRRRGRGS RGSGNGIEET ICTATVKKNI GRNRSPDIYN PQAGSLKTAN ELNLLILRWL GLSAEYGNLY
401 RNALFVPHYN TNAHSIIYAL RGRAHVQVVD SNGNRVYDEE LQEGHVLVVP QNFAVAGKSQ SDNFEYVAFK TDSRPSIANL
481 AGENSVIDNL PEEVVANSYG LPREQARQLK NNNPFKFFVP PSQQSPRAVA

Supporting Peptides:

Peptide	Uniq	-10lgP	Mass	Length	ppm	m/z	z	RT	Fraction	Scan	Source File	Area Digest 1. traka 13-15 kDa	#Feature	#Feature Digest 1. traka 13-15 kDa	Start	End	PTM
R.GYFGLIFPGC(+57.02)PSTYEEPAQQGR.R	Y	79.67	2473.1372	22	3.0	1237.5796	2	34.90	23	1858	OB4051.raw	2.94E4	1	1	99	120	Carbamidomethylation
R.NALRRPFYSN(+.98)APQEIFIQQGR.G	Y	75.95	2505.2876	21	1.0	836.1040	3	30.01	23	1564	OB4051.raw	2.67E5	1	1	78	98	Deamidation (NQ)
R.RPFYSNAPQEIFIQQGR.G	Y	73.07	2050.0383	17	-0.1	684.3533	3	30.09	23	1551	OB4051.raw	3.6E5	1	1	82	98	
R.RPFYSNAPQ(+.98)EIFIQQGR.G	Y	72.83	2051.0225	17	-0.6	684.6810	3	30.68	23	1600	OB4051.raw	2.49E5	1	1	82	98	Deamidation (NQ)
R.NALRRPFYSNAPQEIFIQQGR.G	Y	72.48	2504.3037	21	-0.1	835.7751	3	28.99	23	1488	OB4051.raw	2.43E6	4	4	78	98	
S.ISFRQQPEENAC(+57.02)QFQR.L	Y	64.02	2036.9486	16	0.5	679.9905	3	26.48	23	1325	OB4051.raw	9.82E4	2	2	21	36	Carbamidomethylation
S.ISFRQ(+.98)QPEENAC(+57.02)QFQR.L	Y	62.11	2037.9326	16	0.0	680.3182	3	26.91	23	1368	OB4051.raw	2.38E5	2	2	21	36	Carbamidomethylation
S.ISFRQQ(+.98)PEENAC(+57.02)QFQR.L	Y	58.19	2037.9326	16	0.0	680.3182	3	26.91	23	1357	OB4051.raw	2.25E5	1	1	21	36	Deamidation (NQ); Carbamidomethylation
R.GYFGLIFPGC(+57.02)PSTYEEPAQ(+.98)QGR.R	Y	57.05	2474.1211	22	5.8	825.7191	3	35.00	23	1864	OB4051.raw	1.59E5	2	2	99	120	Carbamidomethylation
R.N(+.98)ALRRPFYSNAPQEIFIQQGR.G	Y	55.69	2505.2876	21	4.1	836.1066	3	31.18	23	1625	OB4051.raw	4.28E3	2	2	78	98	
R.R(+43.01)(+14.02)PFYSNAPQEIFIQQGR.G	Y	53.50	2107.0598	17	-0.5	703.3602	3	30.09	23	1554	OB4051.raw	5.02E4	1	1	82	98	Carbamylation; Methylation(KR)
S.ISFRQQ(+.98)PEEN(+.98)AC(+57.02)QFQR.L	Y	53.01	2038.9166	16	0.3	680.6464	3	27.28	23	1386	OB4051.raw	1.86E5	2	2	21	36	Deamidation (NQ); Carbamidomethylation
R.GYFGLIFPGC(+57.02)PSTYEEPAQ(+.98)Q(+.98)G.R	Y	52.28	2319.0042	21	1.8	1160.5115	2	37.27	23	2003	OB4051.raw	2.38E4	1	1	99	119	Carbamidomethylation; Deamidation (NQ)
R.GYFGLIFPGC(+57.02)PSTYEEPAQ(+.98)Q(+.98)GR.R	Y	51.37	2475.1052	22	3.0	1238.5636	2	35.20	23	1876	OB4051.raw	4.43E4	1	1	99	120	Carbamidomethylation; Deamidation (NQ)
R.RPFYSN(+.98)APQ(+.98)EIFIQQGR.G	Y	49.05	2052.0063	17	-0.2	685.0093	3	31.25	23	1630	OB4051.raw	6.55E4	1	1	82	98	Deamidation (NQ)
S.ISFRQQPEEN(+.98)AC(+57.02)QFQR.L	Y	47.83	2037.9326	16	-4.9	680.3148	3	28.27	23	1442	OB4051.raw	1.31E4	1	1	21	36	Deamidation (NQ); Carbamidomethylation
R.LNAQ(+.98)RPDNRIESEGGYIETWN(+.98)PNNQ(+.98)EFEC(+57.02)AGVALSR.L	Y	46.74	4136.8770	36	8.8	1035.2356	4	32.19	23	1690	OB4051.raw	0	0	0	37	72	Carbamidomethylation
R.LN(+.98)AQRPDNRIESEGGYIETWN(+.98)PNNQEFEC(+57.02)AGVALSR.L	Y	45.09	4135.8931	36	5.6	1034.9863	4	32.01	23	1678	OB4051.raw	0	0	0	37	72	Carbamidomethylation
R.GYFGLIFPGC(+57.02)PSTYEEPAQQ(+.98)G.R	Y	44.88	2318.0200	21	2.0	1160.0197	2	37.17	23	1993	OB4051.raw	1.14E4	1	1	99	119	Carbamidomethylation
S.ISFRQ(+.98)QPEEN(+.98)AC(+57.02)QFQR.L	Y	44.79	2038.9166	16	3.5	680.6486	3	29.66	23	1529	OB4051.raw	9.26E2	1	1	21	36	Deamidation (NQ); Carbamidomethylation
R.NALRRPFYSNAPQ(+.98)EIFIQQGR.G	Y	44.58	2505.2876	21	-0.4	836.1028	3	32.63	23	1718	OB4051.raw	0	0	0	78	98	Deamidation (NQ)
S.ISFRQ(+.98)QPEEN(+.98)AC(+57.02)Q(+.98)FQR.L	Y	44.56	2039.9006	16	9.2	680.9804	3	28.92	23	1483	OB4051.raw	0	0	0	21	36	Deamidation (NQ); Carbamidomethylation

Peptide	Uniq	-10lgP	Mass	Length	ppm	m/z	z	RT	Fraction	Scan	Source File	Area Digest 1. traka 13-15 kDa	#Feature	#Feature Digest 1. traka 13-15 kDa	Start	End	PTM
R.GYFGLIFPGC(+57.02)PSTYEEPAQ(+.98)Q(+.98)GRR.Y	Y	43.19	2631.2063	23	3.1	878.0787	3	33.53	23	1774	OB4051.raw	0	0	0	99	121	Carbamidomethylation; Deamidation (NQ)
R.LN(+.98)AQRPDNRIESEGGYIETWNPNNQEFEC(+57.02)AGVALSR.L	Y	41.87	4134.9087	36	2.9	1034.7374	4	31.53	23	1648	OB4051.raw	3.08E4	2	2	37	72	Carbamidomethylation
R.GYFGLIFPGC(+57.02)PSTYEEPAQQ(+.98)GRR.Y	Y	41.54	2630.2224	23	2.2	877.7500	3	34.22	23	1816	OB4051.raw	3.68E5	1	1	99	121	Carbamidomethylation
S.ISFRQQ(+.98)PEENAC(+57.02)Q(+.98)FQR.L	Y	41.38	2038.9166	16	-2.5	680.6445	3	28.10	23	1431	OB4051.raw	0	0	0	21	36	Carbamidomethylation; Deamidation (NQ)
S.ISFRQQ(+.98)PEEN(+.98)AC(+57.02)Q(+.98)FQR.L	Y	41.26	2039.9006	16	-0.5	680.9738	3	27.71	23	1397	OB4051.raw	7.37E4	1	1	21	36	Deamidation (NQ); Carbamidomethylation
R.R(+43.01)(+14.02)PFYSNAPQ(+.98)EIFIQQGR.G	Y	40.22	2108.0439	17	-1.0	703.6879	3	30.76	23	1595	OB4051.raw	3.2E4	1	1	82	98	Carbamylation; Methylation(KR)
R.IESEGGYIETWNPNNQEFEC(+57.02)AGVALSR.L	Y	40.07	3069.3774	27	-1.9	1024.1311	3	33.27	23	1757	OB4051.raw	0	0	0	46	72	Carbamidomethylation
S.ISFRQQPEENAC(+57.02)QFQ(+.98)R.L	Y	39.69	2037.9326	16	-0.2	680.3180	3	28.34	23	1446	OB4051.raw	0	0	0	21	36	Carbamidomethylation
R.LNAQRPDN(+.98)RIESEGGYIETWNPNNQEFEC(+57.02)AGVALSR.L	Y	39.68	4134.9087	36	3.7	1034.7383	4	33.05	23	1744	OB4051.raw	2.12E4	1	1	37	72	Carbamidomethylation
R.LNAQRPDNRIESEGGYIETWN(+.98)PN(+.98)NQEFEC(+57.02)AGVALSR.L	Y	39.41	4135.8931	36	2.3	1034.9829	4	34.12	23	1810	OB4051.raw	0	0	0	37	72	Carbamidomethylation
R.NALRRPFYSNAP(+15.99)QEIFIQQGR.G	Y	38.58	2520.2986	21	-3.8	631.0795	4	29.10	23	1495	OB4051.raw	0	0	0	78	98	
R.LNAQ(+.98)RPDNRIESEGGYIETWNPNNQEFEC(+57.02)AGVALSR.L	Y	38.53	4134.9087	36	0.4	1034.7349	4	36.39	23	1948	OB4051.raw	0	0	0	37	72	Carbamidomethylation
R.N(+.98)ALRRPFYSN(+.98)APQ(+.98)EIFIQQGR.G	Y	38.15	2507.2556	21	9.4	836.7670	3	30.48	23	1602	OB4051.raw	6.36E4	1	1	78	98	Deamidation (NQ)
R.LNAQRPDNRIESEGGYIETWNPNN(+.98)QEFEC(+57.02)AGVALSR.L	Y	37.30	4134.9087	36	2.2	1034.7367	4	36.98	23	1984	OB4051.raw	0	0	0	37	72	Carbamidomethylation
R.NALRRPFYSN(+.98)APQ(+.98)EIFIQQGR.G	Y	36.66	2506.2717	21	8.4	836.4382	3	41.06	23	2232	OB4051.raw	1.61E6	2	2	78	98	Deamidation (NQ)
R.LNAQRPDN(+.98)RIESEGGYIETWNP(+15.99)NNQEFEC(+57.02)AGVALSR.L	Y	36.19	4150.9038	36	-2.2	1038.7310	4	31.09	23	1619	OB4051.raw	0	0	0	37	72	Carbamidomethylation
R.LNAQRPDNRIESEGGYIETWNPN(+.98)NQEFEC(+57.02)AGVALSR.L	Y	35.80	4134.9087	36	3.6	1034.7382	4	37.81	23	2034	OB4051.raw	0	0	0	37	72	Carbamidomethylation
R.NALRRP(+15.99)FYSNAPQEIFIQQGR.G	Y	35.26	2520.2986	21	0.5	631.0822	4	29.17	23	1499	OB4051.raw	0	0	0	78	98	
S.I(+43.01)SFR(+14.02)QQPEENAC(+57.02)QFQR.L	Y	35.15	2093.9700	16	0.7	698.9977	3	26.67	23	1335	OB4051.raw	1.01E4	1	1	21	36	Carbamylation; Methylation(KR); Carbamidomethylation
R.LNAQRPDN(+.98)RIESEGGYIETWNPN(+.98)NQEFEC(+57.02)AGVALSR.L	Y	34.92	4135.8931	36	-0.1	1034.9805	4	40.13	23	2176	OB4051.raw	0	0	0	37	72	Carbamidomethylation
R.GYFGLIFPGC(+57.02)PSTYEEPAQ(+.98)Q(+.98).G	Y	34.77	2261.9827	20	1.2	1132.0000	2	37.47	23	2014	OB4051.raw	8.2E3	1	1	99	118	Carbamidomethylation; Deamidation (NQ)
R.R(+43.01)(+14.02)PFYSNAPQEIFIQ(+.98)QGR.G	Y	34.75	2108.0439	17	-1.0	703.6879	3	30.76	23	1583	OB4051.raw	3.2E4	1	1	82	98	Carbamylation; Methylation(KR)
R.LNAQRPDNRIESEGGYIETWNP(+15.99)N(+.98)NQEFEC(+57.02)AGVALSR.L	Y	34.61	4150.9038	36	2.0	1038.7354	4	30.83	23	1603	OB4051.raw	0	0	0	37	72	Carbamidomethylation

Peptide	Uniq	-10lgP	Mass	Length	ppm	m/z	z	RT	Fraction	Scan	Source File	Area Digest 1. traka 13-15 kDa	#Feature	#Feature Digest 1. traka 13-15 kDa	Start	End	PTM
R.LNAQRPDNRIESEGGYIETWNPNNQEFEC(+57.02)AGVALSR.L	Y	34.48	4133.9248	36	-4.8	1034.4835	4	36.92	23	1980	OB4051.raw	1.59E4	1	1	37	72	Carbamidomethylation
R.LNAQRPDN(+.98)RIESEGGYIETWN(+.98)PNNQEFEC(+57.02)AGVALSR.L	Y	34.30	4135.8931	36	9.3	1034.9901	4	35.72	23	1907	OB4051.raw	0	0	0	37	72	Carbamidomethylation
R.LN(+.98)AQRPDNRIESEGGYIETWNPNNQ(+.98)EFEC(+57.02)AGVALSR.L	Y	34.13	4135.8931	36	7.2	1034.9880	4	36.21	23	1937	OB4051.raw	0	0	0	37	72	Carbamidomethylation
S.ISFRQ(+.98)Q(+.98)PEEN(+.98)AC(+57.02)QFQ(+.98)R.L	Y	33.31	2040.8846	16	-2.9	681.3002	3	27.96	23	1423	OB4051.raw	2.33E4	1	1	21	36	Deamidation (NQ); Carbamidomethylation
S.ISFRQ(+.98)QPEENAC(+57.02)Q(+.98)FQR.L	Y	33.09	2038.9166	16	-5.9	680.6422	3	30.14	23	1560	OB4051.raw	0	0	0	21	36	Carbamidomethylation; Deamidation (NQ)
R.LNAQRPDN(+.98)RIESEGGYIETWN(+.98)P(+15.99)NNQEFEC(+57.02)AGVALSR.L	Y	32.99	4151.8877	36	9.5	1038.9890	4	31.01	23	1614	OB4051.raw	0	0	0	37	72	Carbamidomethylation
R.N(+.98)ALRRPFYSN(+.98)APQEIFIQQGR.G	Y	32.73	2506.2717	21	6.2	836.4363	3	31.45	23	1643	OB4051.raw	0	0	0	78	98	
R.LNAQRP(+15.99)DNRIESEGGYIETWN(+.98)PNNQEFEC(+57.02)AGVALSR.L	Y	32.26	4150.9038	36	-2.8	1038.7303	4	34.46	23	1830	OB4051.raw	0	0	0	37	72	Carbamidomethylation
R.RPFYS(+58.03)NAPQEIFIQQGR.G	Y	31.50	2108.0676	17	-0.2	703.6964	3	31.14	23	1622	OB4051.raw	0	0	0	82	98	2,3-dihydro-2,2-dimethyl-7-benzofuranol N-methyl carbamate
R.GYFGLIFPGC(+57.02)PSTYEEPAQ(+.98)QGRR.Y	Y	31.26	2630.2224	23	1.0	877.7490	3	34.87	23	1855	OB4051.raw	0	0	0	99	121	Carbamidomethylation
R.LNAQRPDNR(+14.02)IESEGGYIETWNP(+15.99)NNQEFEC(+57.02)AGVALSR.L	Y	30.81	4163.9355	36	-1.6	1041.9895	4	33.36	23	1763	OB4051.raw	0	0	0	37	72	Carbamidomethylation
R.GYFGLIFPGC(+57.02)PSTYEEP(+15.99)AQQGRR.Y	Y	30.42	2645.2332	23	1.1	882.7526	3	33.56	23	1776	OB4051.raw	0	0	0	99	121	Carbamidomethylation
S.ISFRQQ(+.98)PEEN(+.98)AC(+57.02)QFQ(+.98)R.L	Y	29.88	2039.9006	16	1.2	680.9750	3	29.35	23	1508	OB4051.raw	3.32E3	1	1	21	36	Carbamidomethylation
R.N(+43.01)ALRRP(+15.99)FYSNAPQEIFIQQGR.G	Y	29.70	2563.3042	21	3.4	855.4449	3	30.68	23	1596	OB4051.raw	1.48E4	1	1	78	98	Carbamylation; Hydroxylation Pro
R.NALRRPFYSNAP(+15.99)Q(+.98)EIFIQQGR.G	Y	29.57	2521.2825	21	-0.1	631.3278	4	30.10	23	1558	OB4051.raw	0	0	0	78	98	
R.LNAQ(+.98)RPDNRIESEGGYIETWNP(+15.99)NNQEFEC(+57.02)AGVALSR.L	Y	29.52	4150.9038	36	4.6	1038.7380	4	31.90	23	1812	OB4051.raw	8.71E5	1	1	37	72	Carbamidomethylation
R.LNAQRPDNRIESEGGYIETWNPNNQ(+.98)EFEC(+57.02)AGVALSR.L	Y	29.43	4134.9087	36	8.1	1034.7428	4	36.60	23	1961	OB4051.raw	0	0	0	37	72	Carbamidomethylation
S.ISFRQ(+.98)Q(+.98)PEENAC(+57.02)QFQR.L	Y	29.41	2038.9166	16	-0.9	680.6456	3	29.88	23	1544	OB4051.raw	0	0	0	21	36	Carbamidomethylation
R.N(+.98)ALRRPFYSNAPQ(+.98)EIFIQQGR.G	Y	28.90	2506.2717	21	7.7	627.5800	4	30.01	23	1573	OB4051.raw	1.61E6	1	1	78	98	
G.GYIETWNPNN(+.98)QEFEC(+57.02)AGVALSR.L	Y	28.67	2555.1387	22	1.5	852.7214	3	33.02	23	1736	OB4051.raw	6.62E3	1	1	51	72	Carbamidomethylation
R.LNAQ(+.98)RPDN(+.98)RIESEGGYIETWNPNN(+.98)QEFEC(+57.02)AGVALSR.L	Y	28.06	4136.8770	36	5.8	828.3875	5	31.70	23	1646	OB4051.raw	3.18E4	1	1	37	72	Carbamidomethylation
R.LNAQRPDN(+.98)RIESEGGYIETWNPNNQEFE(+14.02)C(+57.02)AGVALSR.L	Y	28.03	4148.9243	36	5.6	1038.2441	4	33.09	23	1746	OB4051.raw	0	0	0	37	72	Carbamidomethylation
R.LN(+.98)AQRPDN(+.98)RIESEGGYIETWNPNNQEFEC(+57.02)AGVALSR.L	Y	27.87	4135.8931	36	1.6	1034.9822	4	39.40	23	2131	OB4051.raw	0	0	0	37	72	Carbamidomethylation
R.GYFGLIFP(+15.99)GC(+57.02)P(+15.99)STYEEPAQQ(+.98)GRR.Y	Y	27.78	2662.2122	23	-2.0	888.4095	3	33.73	23	1786	OB4051.raw	0	0	0	99	121	Carbamidomethylation

Peptide	Uniq	-10lgP	Mass	Length	ppm	m/z	z	RT	Fraction	Scan	Source File	Area Digest 1. traka 13-15 kDa	#Feature	#Feature Digest 1. traka 13-15 kDa	Start	End	PTM
R.LNAQRPDNRIESEGGYIETWN(+.98)PNNQEFEC(+57.02)AGVALSR.L	Y	27.74	4134.9087	36	4.9	1379.3169	3	31.34	23	1655	OB4051.raw	5.03E4	2	2	37	72	Carbamidomethylation
R.GYFGLIFPGC(+57.02)P(+15.99)STYEEP(+15.99)AQ(+.98)QGRR.Y	Y	27.21	2662.2122	23	1.3	888.4125	3	33.54	23	1775	OB4051.raw	0	0	0	99	121	Carbamidomethylation
R.LNAQRPDNRIESEGGYIETWN(+.98)PNN(+.98)QEFEC(+57.02)AGVALSR.L	Y	27.03	4135.8931	36	-4.2	1034.9762	4	39.60	23	2143	OB4051.raw	0	0	0	37	72	Carbamidomethylation
R.LNAQ(+.98)RP(+15.99)DNRIESEGGYIETWNP(+15.99)NNQEFEC(+57.02)AGVALSR.L	Y	26.88	4166.8989	36	-1.8	1042.7301	4	32.02	23	1679	OB4051.raw	0	0	0	37	72	Hydroxylation Pro; Carbamidomethylation
R.LNAQRPDNRIESEGGYIETWNP(+15.99)NNQEFEC(+57.02)AGVALSR.L	Y	26.81	4149.9199	36	2.2	1038.4895	4	32.87	23	1733	OB4051.raw	0	0	0	37	72	Carbamidomethylation
R.LNAQRP(+15.99)DNRIESEGGYIETWN(+.98)P(+15.99)NNQ(+.98)EFEC(+57.02)AGVALSR.L	Y	26.81	4167.8828	36	8.8	1042.9872	4	30.91	23	1608	OB4051.raw	0	0	0	37	72	Hydroxylation Pro; Carbamidomethylation
R.L(+42.01)NAQRP(+15.99)DNRIESEGGYIETWNPN(+.98)NQEFEC(+57.02)AGVALSR.L	Y	26.78	4192.9146	36	7.5	1049.2438	4	32.27	23	1696	OB4051.raw	0	0	0	37	72	Acetylation (N-term); Carbamidomethylation
R.LNAQRP(+15.99)DNRIESEGGYIETWNP(+15.99)NNQ(+.98)EFEC(+57.02)AGVALSR.L	Y	26.30	4166.8989	36	0.3	1042.7323	4	30.28	23	1569	OB4051.raw	0	0	0	37	72	Hydroxylation Pro; Carbamidomethylation
R.GYFGLIFP(+15.99)GC(+57.02)PSTYEEPAQQ(+.98)GRR.Y	Y	26.01	2646.2173	23	-5.9	883.0745	3	34.01	23	1805	OB4051.raw	1.9E4	1	1	99	121	Carbamidomethylation
R.LNAQRPD(+21.98)NRIESEGGYIET(-18.01)WNPNNQEFEC(+57.02)AGVALSR.L	Y	25.93	4137.8965	36	4.4	1035.4860	4	37.86	23	2034	OB4051.raw	1.43E4	1	1	37	72	Carbamidomethylation
E.FEC(+57.02)AGVALSR.L	Y	25.38	1108.5334	10	-1.7	555.2731	2	27.11	23	1369	OB4051.raw	1.24E3	1	1	63	72	Carbamidomethylation
S.ISFRQQP(+15.99)EENAC(+57.02)QFQR.L	Y	25.30	2052.9436	16	-6.8	685.3171	3	28.02	23	1432	OB4051.raw	5.73E3	1	1	21	36	Hydroxylation Pro; Carbamidomethylation
R.IESEGGYIETWNP(+15.99)N(+.98)N(+.98)QEFEC(+57.02)AGVALSR.L	Y	24.66	3087.3403	27	3.2	1030.1240	3	34.08	23	1807	OB4051.raw	0	0	0	46	72	Hydroxylation Pro; Carbamidomethylation
R.LNAQRP(+15.99)DNRIESEGGYIETWNP(+15.99)NNQEFEC(+57.02)AGVALSR.L	Y	24.62	4165.9146	36	2.9	1042.4889	4	31.43	23	1642	OB4051.raw	0	0	0	37	72	Hydroxylation Pro; Carbamidomethylation
R.LNAQ(+.98)RP(+15.99)DNRIESEGGYIETWN(+.98)P(+15.99)NNQEFEC(+57.02)AGVALSR.L	Y	24.48	4167.8828	36	-3.7	1042.9741	4	31.99	23	1679	OB4051.raw	1.81E5	1	1	37	72	Hydroxylation Pro; Carbamidomethylation
S.ISFRQQP(+15.99)EEN(+.98)AC(+57.02)QFQR.L	Y	24.24	2053.9275	16	0.2	685.6499	3	28.24	23	1440	OB4051.raw	0	0	0	21	36	Hydroxylation Pro; Deamidation (NQ); Carbamidomethylation
R.LNAQRP(+15.99)DN(+.98)RIESEGGYIETWNP(+15.99)NNQEFEC(+57.02)AGVALSR.L	Y	24.19	4166.8989	36	1.3	1042.7334	4	30.56	23	1587	OB4051.raw	0	0	0	37	72	Hydroxylation Pro; Carbamidomethylation
R.N(+.98)ALRRPFYSNAP(+15.99)QEIFIQQGR.G	Y	24.11	2521.2825	21	-0.5	631.3276	4	30.21	23	1565	OB4051.raw	0	0	0	78	98	
R.GYFGLIFPGC(+57.02)PSTYEEP(+15.99)AQQ(+.98)GRR.Y	Y	23.79	2646.2173	23	-5.9	883.0745	3	34.01	23	1811	OB4051.raw	1.9E4	1	1	99	121	Carbamidomethylation
S.ISFRQQ(+.98)P(+15.99)EENAC(+57.02)QFQR.L	Y	23.77	2053.9275	16	2.5	685.6515	3	27.42	23	1393	OB4051.raw	7.88E3	1	1	21	36	Hydroxylation Pro; Carbamidomethylation

Peptide	Uniq	-10lgP	Mass	Length	ppm	m/z	z	RT	Fraction	Scan	Source File	Area Digest 1. traka 13-15 kDa	#Feature	#Feature Digest 1. traka 13-15 kDa	Start	End	PTM
R.LNAQRPDNRIESEGGYIE(+14.02)TWNPNNQ(+.98)EFEC(+57.02)AGVALSR.L	Y	23.52	4148.9243	36	3.7	1038.2422	4	33.94	23	1799	OB4051.raw	0	0	0	37	72	Carbamidomethylation
S.I(+42.01)SFRQQP(+15.99)EENAC(+57.02)QFQR.L	Y	23.36	2094.9541	16	0.2	699.3254	3	27.03	23	1365	OB4051.raw	3.45E4	1	1	21	36	Acetylation (Protein N-term); Hydroxylation Pro; Carbamidomethylation
R.GYFGLIFPGC(+114.04)PSTYEEPAQQGR.R	Y	23.25	2530.1587	22	2.2	844.3953	3	34.90	23	1861	OB4051.raw	6.19E3	1	1	99	120	
R.GYFGLIFPGC(+57.02)PSTYEEPA.Q	N	23.12	2003.8975	18	-0.6	1002.9554	2	37.57	23	2023	OB4051.raw	8.65E3	1	1	99	116	Carbamidomethylation
R.LN(+.98)AQRPDNRIESEGGYIETWNPNN(+.98)Q(+.98)EFEC(+57.02)AGVALSR.L	Y	22.78	4136.8770	36	0.8	1035.2273	4	37.68	23	2026	OB4051.raw	0	0	0	37	72	Carbamidomethylation
R.LNAQRPDN(+.98)RIESEGGYIETWNPNN(+.98)Q(+.98)EFEC(+57.02)AGVALSR.L	Y	22.70	4136.8770	36	5.8	828.3875	5	31.70	23	1660	OB4051.raw	3.18E4	1	1	37	72	Carbamidomethylation
R.LNAQRPDNRIESEGGYIETWNPNNQEFEC(+71.04)AGVALSR.L	Y	22.66	4147.9404	36	1.3	1037.9938	4	31.61	23	1756	OB4051.raw	5.12E5	1	1	37	72	Propionamide
S.I(+42.01)SFR(+14.02)QQ(+.98)PEENAC(+57.02)QFQR.L	Y	22.62	2093.9587	16	6.0	698.9977	3	26.67	23	1339	OB4051.raw	1.01E4	1	1	21	36	Acetylation (Protein N-term); Methylation(KR); Carbamidomethylation
R.IESEGGYIETWN(+.98)P(+15.99)NNQEFEC(+57.02)AGVALSR.L	Y	22.43	3086.3562	27	4.6	1029.7974	3	33.78	23	1789	OB4051.raw	0	0	0	46	72	Hydroxylation Pro; Carbamidomethylation
R.GYFGLIFPGC(+57.02)PSTYEEPAQQ(+.98).G	Y	21.93	2260.9985	20	2.7	1131.5096	2	37.27	23	2000	OB4051.raw	6.39E3	1	1	99	118	Carbamidomethylation
R.N(+42.01)ALRRP(+15.99)FYSNAPQEIFIQQGR.G	Y	21.91	2562.3091	21	-1.4	855.1091	3	30.71	23	1596	OB4051.raw	0	0	0	78	98	Acetylation (N-term)
S.ISFRQQ(+.98)PEENAC(+57.02)Q(+.98)FQ(+.98)R.L	Y	21.84	2039.9006	16	1.2	680.9750	3	29.35	23	1522	OB4051.raw	3.32E3	1	1	21	36	Carbamidomethylation
R.GYFGLIFPGC(+57.02)P(+15.99)STYEEPAQQGRR.Y	Y	21.73	2645.2332	23	6.3	882.7572	3	34.59	23	1838	OB4051.raw	0	0	0	99	121	Carbamidomethylation
R.N(+.98)ALRRP(+15.99)FYSNAPQEIFIQQGR.G	Y	21.53	2521.2825	21	5.0	631.3311	4	29.41	23	1505	OB4051.raw	7.76E3	1	1	78	98	
R.LNAQRPDNRIESEGGYIETWNPNN(+.98)Q(+.98)EFEC(+57.02)AGVALSR.L	Y	21.45	4135.8931	36	1.1	828.1868	5	31.71	23	1660	OB4051.raw	0	0	0	37	72	Carbamidomethylation
R.N(+43.01)ALR(+14.02)RPFYSNAPQEIFIQQGR.G	Y	21.38	2561.3250	21	1.7	854.7838	3	30.09	23	1556	OB4051.raw	1.19E4	1	1	78	98	Carbamylation
R.N(+.98)ALRR(+.98)PFYSN(+.98)APQEIFIQQGR.G	Y	21.10	2507.2556	21	9.4	836.7670	3	30.48	23	1625	OB4051.raw	6.36E4	1	1	78	98	Deamidation (NQ)
S.ISFR(+14.02)Q(+.98)QPEENAC(+57.02)QFQR.L	Y	20.79	2051.9482	16	-1.3	684.9891	3	27.68	23	1404	OB4051.raw	0	0	0	21	36	Methylation(KR); Carbamidomethylation
R.GYFGLIFP(+15.99)GC(+57.02)P(+15.99)STYEEPAQ(+.98)QGR.R	Y	20.70	2506.1111	22	-0.1	836.3776	3	35.10	23	1874	OB4051.raw	7.14E3	1	1	99	120	Carbamidomethylation
R.NALRRP(+15.99)FYSNAPQ(+.98)EIFIQQGR.G	Y	20.56	2521.2825	21	7.1	631.3324	4	28.64	23	1466	OB4051.raw	0	0	0	78	98	
R.LNAQRPDNRIESEGGYIETWNPN(+.98)N(+.98)QEFEC(+57.02)AGVALSR.L	Y	20.32	4135.8931	36	6.4	1034.9872	4	40.80	23	2218	OB4051.raw	0	0	0	37	72	Carbamidomethylation
R.LNAQRPDNR(+28.03)IESEGGYIETWNPNNQEFEC(+57.02)AGVALSR.L	Y	20.18	4161.9561	36	-6.8	1041.4893	4	34.49	23	1832	OB4051.raw	0	0	0	37	72	Carbamidomethylation
R.NALRRPFYSNAPQEIFIQQ(+.98)GRGYFGLIFPGC(+57.02)PSTYEEPAQQGR.R	Y	20.13	4960.4141	43	1.0	993.0911	5	34.71	23	1822	OB4051.raw	2.3E5	1	1	78	120	Carbamidomethylation

Peptide	Uniq	-10lgP	Mass	Length	ppm	m/z	z	RT	Fraction	Scan	Source File	Area Digest 1. traka 13-15 kDa	#Feature	#Feature Digest 1. traka 13-15 kDa	Start	End	PTM
total 112 peptides																	

tr|Q9SQH7|Q9SQH7_ARAHY

back to list

| Protein Coverage | Supporting Peptides |

Protein Coverage:

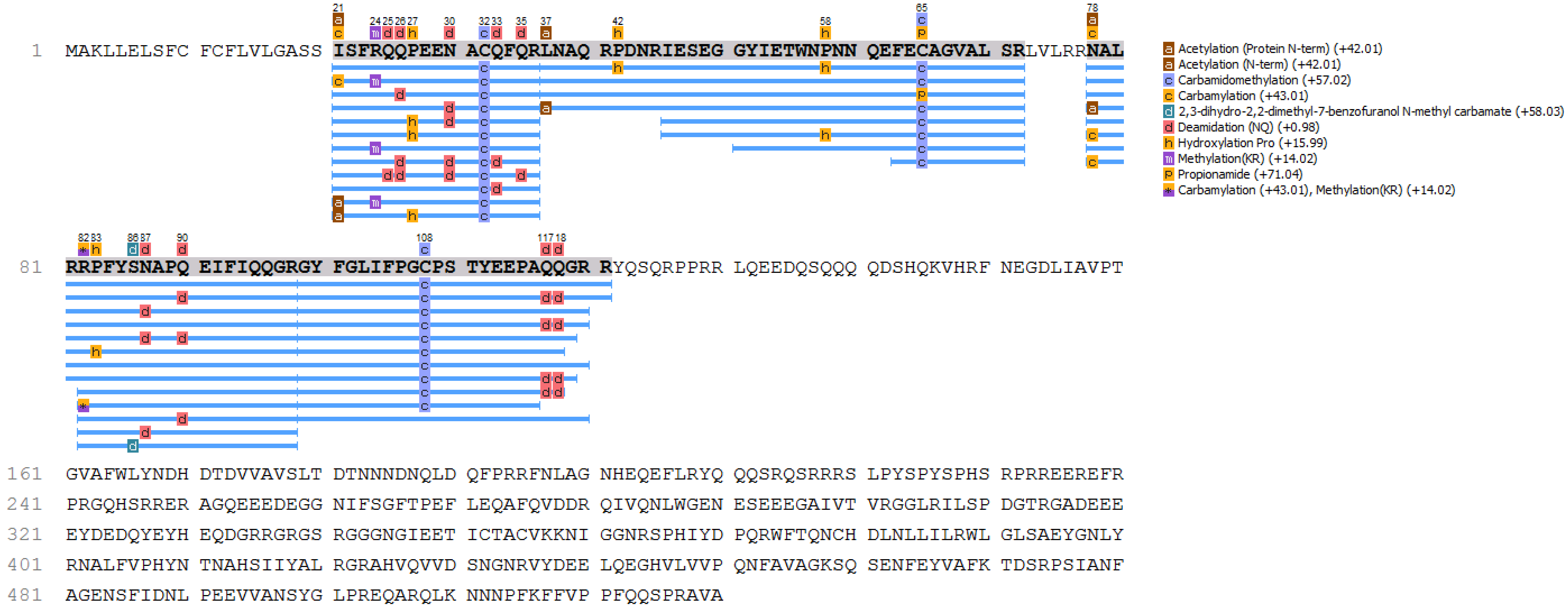

Supporting Peptides:

Peptide	Uniq	-10lgP	Mass	Length	ppm	m/z	z	RT	Fraction	Scan	Source File	Area Digest 1. traka 13-15 kDa	#Feature	#Feature Digest 1. traka 13-15 kDa	Start	End	PTM
R.GYFGLIFPGC(+57.02)PSTYEEPAQQGR.R	Y	79.67	2473.1372	22	3.0	1237.5796	2	34.90	23	1858	OB4051.raw	2.94E4	1	1	99	120	Carbamidomethylation

Peptide	Uniq	-10lgP	Mass	Length	ppm	m/z	z	RT	Fraction	Scan	Source File	Area Digest 1. traka 13-15 kDa	#Feature	#Feature Digest 1. traka 13-15 kDa	Start	End	PTM
R.NALRRPFYSN(+.98)APQEIFIQQGR.G	Y	75.95	2505.2876	21	1.0	836.1040	3	30.01	23	1564	OB4051.raw	2.67E5	1	1	78	98	Deamidation (NQ)
R.RPFYSNAPQEIFIQQGR.G	Y	73.07	2050.0383	17	-0.1	684.3533	3	30.09	23	1551	OB4051.raw	3.6E5	1	1	82	98	
R.RPFYSNAPQ(+.98)EIFIQQGR.G	Y	72.83	2051.0225	17	-0.6	684.6810	3	30.68	23	1600	OB4051.raw	2.49E5	1	1	82	98	Deamidation (NQ)
R.NALRRPFYSNAPQEIFIQQGR.G	Y	72.48	2504.3037	21	-0.1	835.7751	3	28.99	23	1488	OB4051.raw	2.43E6	4	4	78	98	
S.ISFRQQPEENAC(+57.02)QFQR.L	Y	64.02	2036.9486	16	0.5	679.9905	3	26.48	23	1325	OB4051.raw	9.82E4	2	2	21	36	Carbamidomethylation
S.ISFRQ(+.98)QPEENAC(+57.02)QFQR.L	Y	62.11	2037.9326	16	0.0	680.3182	3	26.91	23	1368	OB4051.raw	2.38E5	2	2	21	36	Carbamidomethylation
S.ISFRQQ(+.98)PEENAC(+57.02)QFQR.L	Y	58.19	2037.9326	16	0.0	680.3182	3	26.91	23	1357	OB4051.raw	2.25E5	1	1	21	36	Deamidation (NQ); Carbamidomethylation
R.GYFGLIFPGC(+57.02)PSTYEEPAQ(+.98)QGR.R	Y	57.05	2474.1211	22	5.8	825.7191	3	35.00	23	1864	OB4051.raw	1.59E5	2	2	99	120	Carbamidomethylation
R.N(+.98)ALRRPFYSNAPQEIFIQQGR.G	Y	55.69	2505.2876	21	4.1	836.1066	3	31.18	23	1625	OB4051.raw	4.28E3	2	2	78	98	
R.R(+43.01)(+14.02)PFYSNAPQEIFIQQGR.G	Y	53.50	2107.0598	17	-0.5	703.3602	3	30.09	23	1554	OB4051.raw	5.02E4	1	1	82	98	Carbamylation; Methylation(KR)
S.ISFRQQ(+.98)PEEN(+.98)AC(+57.02)QFQR.L	Y	53.01	2038.9166	16	0.3	680.6464	3	27.28	23	1386	OB4051.raw	1.86E5	2	2	21	36	Deamidation (NQ); Carbamidomethylation
R.GYFGLIFPGC(+57.02)PSTYEEPAQ(+.98)Q(+.98)G.R	Y	52.28	2319.0042	21	1.8	1160.5115	2	37.27	23	2003	OB4051.raw	2.38E4	1	1	99	119	Carbamidomethylation; Deamidation (NQ)
R.GYFGLIFPGC(+57.02)PSTYEEPAQ(+.98)Q(+.98)GR.R	Y	51.37	2475.1052	22	3.0	1238.5636	2	35.20	23	1876	OB4051.raw	4.43E4	1	1	99	120	Carbamidomethylation; Deamidation (NQ)
R.RPFYSN(+.98)APQ(+.98)EIFIQQGR.G	Y	49.05	2052.0063	17	-0.2	685.0093	3	31.25	23	1630	OB4051.raw	6.55E4	1	1	82	98	Deamidation (NQ)
S.ISFRQQPEEN(+.98)AC(+57.02)QFQR.L	Y	47.83	2037.9326	16	-4.9	680.3148	3	28.27	23	1442	OB4051.raw	1.31E4	1	1	21	36	Deamidation (NQ); Carbamidomethylation
R.LNAQ(+.98)RPDNRIESEGGYIETWN(+.98)PNNQ(+.98)EFEC(+57.02)AGVALSR.L	Y	46.74	4136.8770	36	8.8	1035.2356	4	32.19	23	1690	OB4051.raw	0	0	0	37	72	Carbamidomethylation
R.LN(+.98)AQRPDNRIESEGGYIETWN(+.98)PNNQEFEC(+57.02)AGVALSR.L	Y	45.09	4135.8931	36	5.6	1034.9863	4	32.01	23	1678	OB4051.raw	0	0	0	37	72	Carbamidomethylation
R.GYFGLIFPGC(+57.02)PSTYEEPAQQ(+.98)G.R	Y	44.88	2318.0200	21	2.0	1160.0197	2	37.17	23	1993	OB4051.raw	1.14E4	1	1	99	119	Carbamidomethylation
S.ISFRQ(+.98)QPEEN(+.98)AC(+57.02)QFQR.L	Y	44.79	2038.9166	16	3.5	680.6486	3	29.66	23	1529	OB4051.raw	9.26E2	1	1	21	36	Deamidation (NQ); Carbamidomethylation
R.NALRRPFYSNAPQ(+.98)EIFIQQGR.G	Y	44.58	2505.2876	21	-0.4	836.1028	3	32.63	23	1718	OB4051.raw	0	0	0	78	98	Deamidation (NQ)
S.ISFRQ(+.98)QPEEN(+.98)AC(+57.02)Q(+.98)FQR.L	Y	44.56	2039.9006	16	9.2	680.9804	3	28.92	23	1483	OB4051.raw	0	0	0	21	36	Deamidation (NQ); Carbamidomethylation
R.GYFGLIFPGC(+57.02)PSTYEEPAQ(+.98)Q(+.98)GRR.Y	Y	43.19	2631.2063	23	3.1	878.0787	3	33.53	23	1774	OB4051.raw	0	0	0	99	121	Carbamidomethylation; Deamidation (NQ)

Peptide	Uniq	-10lgP	Mass	Length	ppm	m/z	z	RT	Fraction	Scan	Source File	Area Digest 1. traka 13-15 kDa	#Feature	#Feature Digest 1. traka 13-15 kDa	Start	End	PTM
R.LN(+.98)AQRPDNRIESEGGYIETWNPNNQEFEC(+57.02)AGVALSR.L	Y	41.87	4134.9087	36	2.9	1034.7374	4	31.53	23	1648	OB4051.raw	3.08E4	2	2	37	72	Carbamidomethylation
R.GYFGLIFPGC(+57.02)PSTYEEPAQQ(+.98)GRR.Y	Y	41.54	2630.2224	23	2.2	877.7500	3	34.22	23	1816	OB4051.raw	3.68E5	1	1	99	121	Carbamidomethylation
S.ISFRQQ(+.98)PEENAC(+57.02)Q(+.98)FQR.L	Y	41.38	2038.9166	16	-2.5	680.6445	3	28.10	23	1431	OB4051.raw	0	0	0	21	36	Carbamidomethylation; Deamidation (NQ)
S.ISFRQQ(+.98)PEEN(+.98)AC(+57.02)Q(+.98)FQR.L	Y	41.26	2039.9006	16	-0.5	680.9738	3	27.71	23	1397	OB4051.raw	7.37E4	1	1	21	36	Deamidation (NQ); Carbamidomethylation
R.R(+43.01)(+14.02)PFYSNAPQ(+.98)EIFIQQGR.G	Y	40.22	2108.0439	17	-1.0	703.6879	3	30.76	23	1595	OB4051.raw	3.2E4	1	1	82	98	Carbamylation; Methylation(KR)
R.IESEGGYIETWNPNNQEFEC(+57.02)AGVALSR.L	Y	40.07	3069.3774	27	-1.9	1024.1311	3	33.27	23	1757	OB4051.raw	0	0	0	46	72	Carbamidomethylation
S.ISFRQQPEENAC(+57.02)QFQ(+.98)R.L	Y	39.69	2037.9326	16	-0.2	680.3180	3	28.34	23	1446	OB4051.raw	0	0	0	21	36	Carbamidomethylation
R.LNAQRPDN(+.98)RIESEGGYIETWNPNNQEFEC(+57.02)AGVALSR.L	Y	39.68	4134.9087	36	3.7	1034.7383	4	33.05	23	1744	OB4051.raw	2.12E4	1	1	37	72	Carbamidomethylation
R.LNAQRPDNRIESEGGYIETWN(+.98)PN(+.98)NQEFEC(+57.02)AGVALSR.L	Y	39.41	4135.8931	36	2.3	1034.9829	4	34.12	23	1810	OB4051.raw	0	0	0	37	72	Carbamidomethylation
R.NALRRPFYSNAP(+15.99)QEIFIQQGR.G	Y	38.58	2520.2986	21	-3.8	631.0795	4	29.10	23	1495	OB4051.raw	0	0	0	78	98	
R.LNAQ(+.98)RPDNRIESEGGYIETWNPNNQEFEC(+57.02)AGVALSR.L	Y	38.53	4134.9087	36	0.4	1034.7349	4	36.39	23	1948	OB4051.raw	0	0	0	37	72	Carbamidomethylation
R.N(+.98)ALRRPFYSN(+.98)APQ(+.98)EIFIQQGR.G	Y	38.15	2507.2556	21	9.4	836.7670	3	30.48	23	1602	OB4051.raw	6.36E4	1	1	78	98	Deamidation (NQ)
R.LNAQRPDNRIESEGGYIETWNPNN(+.98)QEFEC(+57.02)AGVALSR.L	Y	37.30	4134.9087	36	2.2	1034.7367	4	36.98	23	1984	OB4051.raw	0	0	0	37	72	Carbamidomethylation
R.NALRRPFYSN(+.98)APQ(+.98)EIFIQQGR.G	Y	36.66	2506.2717	21	8.4	836.4382	3	41.06	23	2232	OB4051.raw	1.61E6	2	2	78	98	Deamidation (NQ)
R.LNAQRPDN(+.98)RIESEGGYIETWNP(+15.99)NNQEFEC(+57.02)AGVALSR.L	Y	36.19	4150.9038	36	-2.2	1038.7310	4	31.09	23	1619	OB4051.raw	0	0	0	37	72	Carbamidomethylation
R.LNAQRPDNRIESEGGYIETWNPN(+.98)NQEFEC(+57.02)AGVALSR.L	Y	35.80	4134.9087	36	3.6	1034.7382	4	37.81	23	2034	OB4051.raw	0	0	0	37	72	Carbamidomethylation
R.NALRRP(+15.99)FYSNAPQEIFIQQGR.G	Y	35.26	2520.2986	21	0.5	631.0822	4	29.17	23	1499	OB4051.raw	0	0	0	78	98	
S.I(+43.01)SFR(+14.02)QQPEENAC(+57.02)QFQR.L	Y	35.15	2093.9700	16	0.7	698.9977	3	26.67	23	1335	OB4051.raw	1.01E4	1	1	21	36	Carbamylation; Methylation(KR); Carbamidomethylation
R.LNAQRPDN(+.98)RIESEGGYIETWNPN(+.98)NQEFEC(+57.02)AGVALSR.L	Y	34.92	4135.8931	36	-0.1	1034.9805	4	40.13	23	2176	OB4051.raw	0	0	0	37	72	Carbamidomethylation
R.GYFGLIFPGC(+57.02)PSTYEEPAQ(+.98)Q(+.98).G	Y	34.77	2261.9827	20	1.2	1132.0000	2	37.47	23	2014	OB4051.raw	8.2E3	1	1	99	118	Carbamidomethylation; Deamidation (NQ)
R.R(+43.01)(+14.02)PFYSNAPQEIFIQ(+.98)QGR.G	Y	34.75	2108.0439	17	-1.0	703.6879	3	30.76	23	1583	OB4051.raw	3.2E4	1	1	82	98	Carbamylation; Methylation(KR)
R.LNAQRPDNRIESEGGYIETWNP(+15.99)N(+.98)NQEFEC(+57.02)AGVALSR.L	Y	34.61	4150.9038	36	2.0	1038.7354	4	30.83	23	1603	OB4051.raw	0	0	0	37	72	Carbamidomethylation
R.LNAQRPDNRIESEGGYIETWNPNNQEFEC(+57.02)AGVALSR.L	Y	34.48	4133.9248	36	-4.8	1034.4835	4	36.92	23	1980	OB4051.raw	1.59E4	1	1	37	72	Carbamidomethylation

Peptide	Uniq	-10lgP	Mass	Length	ppm	m/z	z	RT	Fraction	Scan	Source File	Area Digest 1. traka 13-15 kDa	#Feature	#Feature Digest 1. traka 13-15 kDa	Start	End	PTM
R.LNAQRPDN(+.98)RIESEGGYIETWN(+.98)PNNQEFEC(+57.02)AGVALSR.L	Y	34.30	4135.8931	36	9.3	1034.9901	4	35.72	23	1907	OB4051.raw	0	0	0	37	72	Carbamidomethylation
R.LN(+.98)AQRPDNRIESEGGYIETWNPNNQ(+.98)EFEC(+57.02)AGVALSR.L	Y	34.13	4135.8931	36	7.2	1034.9880	4	36.21	23	1937	OB4051.raw	0	0	0	37	72	Carbamidomethylation
S.ISFRQ(+.98)Q(+.98)PEEN(+.98)AC(+57.02)QFQ(+.98)R.L	Y	33.31	2040.8846	16	-2.9	681.3002	3	27.96	23	1423	OB4051.raw	2.33E4	1	1	21	36	Deamidation (NQ); Carbamidomethylation
S.ISFRQ(+.98)QPEENAC(+57.02)Q(+.98)FQR.L	Y	33.09	2038.9166	16	-5.9	680.6422	3	30.14	23	1560	OB4051.raw	0	0	0	21	36	Carbamidomethylation; Deamidation (NQ)
R.LNAQRPDN(+.98)RIESEGGYIETWN(+.98)P(+15.99)NNQEFEC(+57.02)AGVALSR.L	Y	32.99	4151.8877	36	9.5	1038.9890	4	31.01	23	1614	OB4051.raw	0	0	0	37	72	Carbamidomethylation
R.N(+.98)ALRRPFYSN(+.98)APQEIFIQQGR.G	Y	32.73	2506.2717	21	6.2	836.4363	3	31.45	23	1643	OB4051.raw	0	0	0	78	98	
R.LNAQRP(+15.99)DNRIESEGGYIETWN(+.98)PNNQEFEC(+57.02)AGVALSR.L	Y	32.26	4150.9038	36	-2.8	1038.7303	4	34.46	23	1830	OB4051.raw	0	0	0	37	72	Carbamidomethylation
R.RPFYS(+58.03)NAPQEIFIQQGR.G	Y	31.50	2108.0676	17	-0.2	703.6964	3	31.14	23	1622	OB4051.raw	0	0	0	82	98	2,3-dihydro-2,2-dimethyl-7-benzofuranol N-methyl carbamate
R.GYFGLIFPGC(+57.02)PSTYEEPAQ(+.98)QGRR.Y	Y	31.26	2630.2224	23	1.0	877.7490	3	34.87	23	1855	OB4051.raw	0	0	0	99	121	Carbamidomethylation
R.LNAQRPDNR(+14.02)IESEGGYIETWNP(+15.99)NNQEFEC(+57.02)AGVALSR.L	Y	30.81	4163.9355	36	-1.6	1041.9895	4	33.36	23	1763	OB4051.raw	0	0	0	37	72	Carbamidomethylation
R.GYFGLIFPGC(+57.02)PSTYEEP(+15.99)AQQGRR.Y	Y	30.42	2645.2332	23	1.1	882.7526	3	33.56	23	1776	OB4051.raw	0	0	0	99	121	Carbamidomethylation
S.ISFRQQ(+.98)PEEN(+.98)AC(+57.02)QFQ(+.98)R.L	Y	29.88	2039.9006	16	1.2	680.9750	3	29.35	23	1508	OB4051.raw	3.32E3	1	1	21	36	Carbamidomethylation
R.N(+43.01)ALRRP(+15.99)FYSNAPQEIFIQQGR.G	Y	29.70	2563.3042	21	3.4	855.4449	3	30.68	23	1596	OB4051.raw	1.48E4	1	1	78	98	Carbamylation; Hydroxylation Pro
R.NALRRPFYSNAP(+15.99)Q(+.98)EIFIQQGR.G	Y	29.57	2521.2825	21	-0.1	631.3278	4	30.10	23	1558	OB4051.raw	0	0	0	78	98	
R.LNAQ(+.98)RPDNRIESEGGYIETWNP(+15.99)NNQEFEC(+57.02)AGVALSR.L	Y	29.52	4150.9038	36	4.6	1038.7380	4	31.90	23	1812	OB4051.raw	8.71E5	1	1	37	72	Carbamidomethylation
R.LNAQRPDNRIESEGGYIETWNPNNQ(+.98)EFEC(+57.02)AGVALSR.L	Y	29.43	4134.9087	36	8.1	1034.7428	4	36.60	23	1961	OB4051.raw	0	0	0	37	72	Carbamidomethylation
S.ISFRQ(+.98)Q(+.98)PEENAC(+57.02)QFQR.L	Y	29.41	2038.9166	16	-0.9	680.6456	3	29.88	23	1544	OB4051.raw	0	0	0	21	36	Carbamidomethylation
R.N(+.98)ALRRPFYSNAPQ(+.98)EIFIQQGR.G	Y	28.90	2506.2717	21	7.7	627.5800	4	30.01	23	1573	OB4051.raw	1.61E6	1	1	78	98	
G.GYIETWNPNN(+.98)QEFEC(+57.02)AGVALSR.L	Y	28.67	2555.1387	22	1.5	852.7214	3	33.02	23	1736	OB4051.raw	6.62E3	1	1	51	72	Carbamidomethylation
R.LNAQ(+.98)RPDN(+.98)RIESEGGYIETWNPNN(+.98)QEFEC(+57.02)AGVALSR.L	Y	28.06	4136.8770	36	5.8	828.3875	5	31.70	23	1646	OB4051.raw	3.18E4	1	1	37	72	Carbamidomethylation
R.LNAQRPDN(+.98)RIESEGGYIETWNPNNQEFE(+14.02)C(+57.02)AGVALSR.L	Y	28.03	4148.9243	36	5.6	1038.2441	4	33.09	23	1746	OB4051.raw	0	0	0	37	72	Carbamidomethylation
R.LN(+.98)AQRPDN(+.98)RIESEGGYIETWNPNNQEFEC(+57.02)AGVALSR.L	Y	27.87	4135.8931	36	1.6	1034.9822	4	39.40	23	2131	OB4051.raw	0	0	0	37	72	Carbamidomethylation
R.GYFGLIFP(+15.99)GC(+57.02)P(+15.99)STYEEPAQQ(+.98)GRR.Y	Y	27.78	2662.2122	23	-2.0	888.4095	3	33.73	23	1786	OB4051.raw	0	0	0	99	121	Carbamidomethylation
R.LNAQRPDNRIESEGGYIETWN(+.98)PNNQEFEC(+57.02)AGVALSR.L	Y	27.74	4134.9087	36	4.9	1379.3169	3	31.34	23	1655	OB4051.raw	5.03E4	2	2	37	72	Carbamidomethylation

Peptide	Uniq	-10lgP	Mass	Length	ppm	m/z	z	RT	Fraction	Scan	Source File	Area Digest 1. traka 13-15 kDa	#Feature	#Feature Digest 1. traka 13-15 kDa	Start	End	PTM
R.GYFGLIFPGC(+57.02)P(+15.99)STYEEP(+15.99)AQ(+.98)QGRR.Y	Y	27.21	2662.2122	23	1.3	888.4125	3	33.54	23	1775	OB4051.raw	0	0	0	99	121	Carbamidomethylation
R.LNAQRPDNRIESEGGYIETWN(+.98)PNN(+.98)QEFEC(+57.02)AGVALSR.L	Y	27.03	4135.8931	36	-4.2	1034.9762	4	39.60	23	2143	OB4051.raw	0	0	0	37	72	Carbamidomethylation
R.LNAQ(+.98)RP(+15.99)DNRIESEGGYIETWNP(+15.99)NNQEFEC(+57.02)AGVALSR.L	Y	26.88	4166.8989	36	-1.8	1042.7301	4	32.02	23	1679	OB4051.raw	0	0	0	37	72	Hydroxylation Pro; Carbamidomethylation
R.LNAQRPDNRIESEGGYIETWNP(+15.99)NNQEFEC(+57.02)AGVALSR.L	Y	26.81	4149.9199	36	2.2	1038.4895	4	32.87	23	1733	OB4051.raw	0	0	0	37	72	Carbamidomethylation
R.LNAQRP(+15.99)DNRIESEGGYIETWN(+.98)P(+15.99)NNQ(+.98)EFEC(+57.02)AGVALSR.L	Y	26.81	4167.8828	36	8.8	1042.9872	4	30.91	23	1608	OB4051.raw	0	0	0	37	72	Hydroxylation Pro; Carbamidomethylation
R.L(+42.01)NAQRP(+15.99)DNRIESEGGYIETWNPN(+.98)NQEFEC(+57.02)AGVALSR.L	Y	26.78	4192.9146	36	7.5	1049.2438	4	32.27	23	1696	OB4051.raw	0	0	0	37	72	Acetylation (N-term); Carbamidomethylation
R.LNAQRP(+15.99)DNRIESEGGYIETWNP(+15.99)NNQ(+.98)EFEC(+57.02)AGVALSR.L	Y	26.30	4166.8989	36	0.3	1042.7323	4	30.28	23	1569	OB4051.raw	0	0	0	37	72	Hydroxylation Pro; Carbamidomethylation
R.GYFGLIFP(+15.99)GC(+57.02)PSTYEEPAQQ(+.98)GRR.Y	Y	26.01	2646.2173	23	-5.9	883.0745	3	34.01	23	1805	OB4051.raw	1.9E4	1	1	99	121	Carbamidomethylation
R.LNAQRPD(+21.98)NRIESEGGYIET(-18.01)WNPNNQEFEC(+57.02)AGVALSR.L	Y	25.93	4137.8965	36	4.4	1035.4860	4	37.86	23	2034	OB4051.raw	1.43E4	1	1	37	72	Carbamidomethylation
E.FEC(+57.02)AGVALSR.L	Y	25.38	1108.5334	10	-1.7	555.2731	2	27.11	23	1369	OB4051.raw	1.24E3	1	1	63	72	Carbamidomethylation
S.ISFRQQP(+15.99)EENAC(+57.02)QFQR.L	Y	25.30	2052.9436	16	-6.8	685.3171	3	28.02	23	1432	OB4051.raw	5.73E3	1	1	21	36	Hydroxylation Pro; Carbamidomethylation
R.IESEGGYIETWNP(+15.99)N(+.98)N(+.98)QEFEC(+57.02)AGVALSR.L	Y	24.66	3087.3403	27	3.2	1030.1240	3	34.08	23	1807	OB4051.raw	0	0	0	46	72	Hydroxylation Pro; Carbamidomethylation
R.LNAQRP(+15.99)DNRIESEGGYIETWNP(+15.99)NNQEFEC(+57.02)AGVALSR.L	Y	24.62	4165.9146	36	2.9	1042.4889	4	31.43	23	1642	OB4051.raw	0	0	0	37	72	Hydroxylation Pro; Carbamidomethylation
R.LNAQ(+.98)RP(+15.99)DNRIESEGGYIETWN(+.98)P(+15.99)NNQEFEC(+57.02)AGVALSR.L	Y	24.48	4167.8828	36	-3.7	1042.9741	4	31.99	23	1679	OB4051.raw	1.81E5	1	1	37	72	Hydroxylation Pro; Carbamidomethylation
S.ISFRQQP(+15.99)EEN(+.98)AC(+57.02)QFQR.L	Y	24.24	2053.9275	16	0.2	685.6499	3	28.24	23	1440	OB4051.raw	0	0	0	21	36	Hydroxylation Pro; Deamidation (NQ); Carbamidomethylation
R.LNAQRP(+15.99)DN(+.98)RIESEGGYIETWNP(+15.99)NNQEFEC(+57.02)AGVALSR.L	Y	24.19	4166.8989	36	1.3	1042.7334	4	30.56	23	1587	OB4051.raw	0	0	0	37	72	Hydroxylation Pro; Carbamidomethylation
R.N(+.98)ALRRPFYSNAP(+15.99)QEIFIQQGR.G	Y	24.11	2521.2825	21	-0.5	631.3276	4	30.21	23	1565	OB4051.raw	0	0	0	78	98	
R.GYFGLIFPGC(+57.02)PSTYEEP(+15.99)AQQ(+.98)GRR.Y	Y	23.79	2646.2173	23	-5.9	883.0745	3	34.01	23	1811	OB4051.raw	1.9E4	1	1	99	121	Carbamidomethylation
S.ISFRQQ(+.98)P(+15.99)EENAC(+57.02)QFQR.L	Y	23.77	2053.9275	16	2.5	685.6515	3	27.42	23	1393	OB4051.raw	7.88E3	1	1	21	36	Hydroxylation Pro; Carbamidomethylation
R.LNAQRPDNRIESEGGYIE(+14.02)TWNPNNQ(+.98)EFEC(+57.02)AGVALSR.L	Y	23.52	4148.9243	36	3.7	1038.2422	4	33.94	23	1799	OB4051.raw	0	0	0	37	72	Carbamidomethylation

Peptide	Uniq	-10lgP	Mass	Length	ppm	m/z	z	RT	Fraction	Scan	Source File	Area Digest 1. traka 13-15 kDa	#Feature	#Feature Digest 1. traka 13-15 kDa	Start	End	PTM
S.I(+42.01)SFRQQP(+15.99)EENAC(+57.02)QFQR.L	Y	23.36	2094.9541	16	0.2	699.3254	3	27.03	23	1365	OB4051.raw	3.45E4	1	1	21	36	Acetylation (Protein N-term); Hydroxylation Pro; Carbamidomethylation
R.GYFGLIFPGC(+114.04)PSTYEEPAQQGR.R	Y	23.25	2530.1587	22	2.2	844.3953	3	34.90	23	1861	OB4051.raw	6.19E3	1	1	99	120	
R.GYFGLIFPGC(+57.02)PSTYEEPA.Q	N	23.12	2003.8975	18	-0.6	1002.9554	2	37.57	23	2023	OB4051.raw	8.65E3	1	1	99	116	Carbamidomethylation
R.LN(+.98)AQRPDNRIESEGGYIETWNPNN(+.98)Q(+.98)EFEC(+57.02)AGVALSR.L	Y	22.78	4136.8770	36	0.8	1035.2273	4	37.68	23	2026	OB4051.raw	0	0	0	37	72	Carbamidomethylation
R.LNAQRPDN(+.98)RIESEGGYIETWNPNN(+.98)Q(+.98)EFEC(+57.02)AGVALSR.L	Y	22.70	4136.8770	36	5.8	828.3875	5	31.70	23	1660	OB4051.raw	3.18E4	1	1	37	72	Carbamidomethylation
R.LNAQRPDNRIESEGGYIETWNPNNQEFEC(+71.04)AGVALSR.L	Y	22.66	4147.9404	36	1.3	1037.9938	4	31.61	23	1756	OB4051.raw	5.12E5	1	1	37	72	Propionamide
S.I(+42.01)SFR(+14.02)QQ(+.98)PEENAC(+57.02)QFQR.L	Y	22.62	2093.9587	16	6.0	698.9977	3	26.67	23	1339	OB4051.raw	1.01E4	1	1	21	36	Acetylation (Protein N-term); Methylation(KR); Carbamidomethylation
R.IESEGGYIETWN(+.98)P(+15.99)NNQEFEC(+57.02)AGVALSR.L	Y	22.43	3086.3562	27	4.6	1029.7974	3	33.78	23	1789	OB4051.raw	0	0	0	46	72	Hydroxylation Pro; Carbamidomethylation
R.GYFGLIFPGC(+57.02)PSTYEEPAQQ(+.98).G	Y	21.93	2260.9985	20	2.7	1131.5096	2	37.27	23	2000	OB4051.raw	6.39E3	1	1	99	118	Carbamidomethylation
R.N(+42.01)ALRRP(+15.99)FYSNAPQEIFIQQGR.G	Y	21.91	2562.3091	21	-1.4	855.1091	3	30.71	23	1596	OB4051.raw	0	0	0	78	98	Acetylation (N-term)
S.ISFRQQ(+.98)PEENAC(+57.02)Q(+.98)FQ(+.98)R.L	Y	21.84	2039.9006	16	1.2	680.9750	3	29.35	23	1522	OB4051.raw	3.32E3	1	1	21	36	Carbamidomethylation
R.GYFGLIFPGC(+57.02)P(+15.99)STYEEPAQQGRR.Y	Y	21.73	2645.2332	23	6.3	882.7572	3	34.59	23	1838	OB4051.raw	0	0	0	99	121	Carbamidomethylation
R.N(+.98)ALRRP(+15.99)FYSNAPQEIFIQQGR.G	Y	21.53	2521.2825	21	5.0	631.3311	4	29.41	23	1505	OB4051.raw	7.76E3	1	1	78	98	
R.LNAQRPDNRIESEGGYIETWNPNN(+.98)Q(+.98)EFEC(+57.02)AGVALSR.L	Y	21.45	4135.8931	36	1.1	828.1868	5	31.71	23	1660	OB4051.raw	0	0	0	37	72	Carbamidomethylation
R.N(+43.01)ALR(+14.02)RPFYSNAPQEIFIQQGR.G	Y	21.38	2561.3250	21	1.7	854.7838	3	30.09	23	1556	OB4051.raw	1.19E4	1	1	78	98	Carbamylation
R.N(+.98)ALRR(+.98)PFYSN(+.98)APQEIFIQQGR.G	Y	21.10	2507.2556	21	9.4	836.7670	3	30.48	23	1625	OB4051.raw	6.36E4	1	1	78	98	Deamidation (NQ)
S.ISFR(+14.02)Q(+.98)QPEENAC(+57.02)QFQR.L	Y	20.79	2051.9482	16	-1.3	684.9891	3	27.68	23	1404	OB4051.raw	0	0	0	21	36	Methylation(KR); Carbamidomethylation
R.GYFGLIFP(+15.99)GC(+57.02)P(+15.99)STYEEPAQ(+.98)QGR.R	Y	20.70	2506.1111	22	-0.1	836.3776	3	35.10	23	1874	OB4051.raw	7.14E3	1	1	99	120	Carbamidomethylation
R.NALRRP(+15.99)FYSNAPQ(+.98)EIFIQQGR.G	Y	20.56	2521.2825	21	7.1	631.3324	4	28.64	23	1466	OB4051.raw	0	0	0	78	98	
R.LNAQRPDNRIESEGGYIETWNPN(+.98)N(+.98)QEFEC(+57.02)AGVALSR.L	Y	20.32	4135.8931	36	6.4	1034.9872	4	40.80	23	2218	OB4051.raw	0	0	0	37	72	Carbamidomethylation
R.LNAQRPDNR(+28.03)IESEGGYIETWNPNNQEFEC(+57.02)AGVALSR.L	Y	20.18	4161.9561	36	-6.8	1041.4893	4	34.49	23	1832	OB4051.raw	0	0	0	37	72	Carbamidomethylation
R.NALRRPFYSNAPQEIFIQQ(+.98)GRGYFGLIFPGC(+57.02)PSTYEEPAQQGR.R	Y	20.13	4960.4141	43	1.0	993.0911	5	34.71	23	1822	OB4051.raw	2.3E5	1	1	78	120	Carbamidomethylation
**total 112 peptides**																	

tr|Q6IWG5|Q6IWG5_ARAHY
back to list

| Protein Coverage | Supporting Peptides |

Protein Coverage:

1 KLLALSLCFC VLVLGASS**VT FRQGGEENEC QFQRLNAQRP DNRIESEGGY IETWNPNNQE FQCAGVALSR** TVLRR**NALRR**

81 **PFYSNAPLEI YVQQGSGYFG LIFPGCPSTY EEPAQEGRR**Y QSQKPSRRFQ VGQDDPSQQQ QDSHQKVHRF DEGDLIAVPT

161 GVAFWMYNDE DTDVVTVTLS DTSSIHNQLD QFPRRFYLAG NQEQEFLRYQ QQQGSRPHYR QISPRVRGDE QENEGSNIFS

241 GFAQEFLQHA FQVDRQTVEN LRGENEREEQ GAIVTVKGGL RILSPDEEDE SSRSPPSRRE EFDEDRSRPQ QRGKYDENRR

321 GYKNGIEETI CSASVKKNLG RSSNPDIYNP QAGSLRSVNE LDLPILGWLG LSAQHGTIYR NAMFVPHYTL NAHTIVVALN

401 GRAHVQVVDS NGNRVYDEEL QEGHVLVVPQ NFAVAAKAQS ENYEYLAFKT DSRPSIANLA GENSIIDNLP EEVVANSYRL

481 PREQARQLKN NNPFKFFVPP FDHQSMREVA

c Carbamidomethylation (+57.02)
c Carbamylation (+43.01)
d Deamidation (NQ) (+0.98)
h Hydroxylation Pro (+15.99)

Supporting Peptides:

Peptide	Uniq	-10lgP	Mass	Length	ppm	m/z	z	RT	Fraction	Scan	Source File	Area Digest 1. traka 13-15 kDa	#Feature	#Feature Digest 1. traka 13-15 kDa	Start	End	PTM
R.LNAQRPDNRIESEGGYIETWNPNNQEFQC(+57.02)AGVALSR.T	Y	52.73	4132.9409	36	1.0	1034.2435	4	31.17	23	1624	OB4051.raw	0	0	0	35	70	Carbamidomethylat
S.VTFRQGGEENEC(+57.02)QFQR.L	Y	47.41	1983.8857	16	0.4	662.3028	3	24.90	23	1231	OB4051.raw	3.28E4	1	1	19	34	Carbamidomethylat
R.LNAQRPDNRIESEGGYIETWNPN(+.98)NQ(+.98)EFQC(+57.02)AGVALSR.T	Y	42.21	4134.9087	36	8.2	1034.7429	4	33.83	23	1792	OB4051.raw	0	0	0	35	70	Carbamidomethylat
S.VTFRQGGEEN(+.98)EC(+57.02)QFQR.L	Y	40.64	1984.8698	16	7.4	662.6354	3	24.81	23	1229	OB4051.raw	2.27E4	1	1	19	34	Deamidation (NQ); Carbamidomethylat

Peptide	Uniq	-10lgP	Mass	Length	ppm	m/z	z	RT	Fraction	Scan	Source File	Area Digest 1. traka 13-15 kDa	#Feature	#Feature Digest 1. traka 13-15 kDa	Start	End	PTM
R.IESEGGYIETWNPNN(+.98)QEFQC(+57.02)AGVALSR.T	Y	40.14	3069.3774	27	2.2	1024.1354	3	33.04	23	1751	OB4051.raw	1.47E4	1	1	44	70	Carbamidomethylat
S.VTFRQGGEENEC(+57.02)Q(+.98)FQR.L	Y	39.75	1984.8698	16	-1.7	662.6294	3	25.37	23	1256	OB4051.raw	2.27E4	1	1	19	34	Carbamidomethylat<br>Deamidation (NQ)
R.LNAQRPDNRIESEGGYIETWNPNNQ(+.98)EFQC(+57.02)AGVALSR.T	Y	39.59	4133.9248	36	1.5	1034.4900	4	31.52	23	1636	OB4051.raw	2.82E6	1	1	35	70	Carbamidomethylat
R.LNAQRPDN(+.98)RIESEGGYIETWNPNNQ(+.98)EFQC(+57.02)AGVALSR.T	Y	39.44	4134.9087	36	-1.3	1034.7332	4	35.06	23	1867	OB4051.raw	0	0	0	35	70	Carbamidomethylat
R.RPFYSNAPLEIYVQQGSGYFGLIFPGC(+57.02)PSTYEEPAQEGR.R	Y	39.29	4424.0845	39	2.8	1107.0315	4	36.52	23	1956	OB4051.raw	0	0	0	80	118	Carbamidomethylat
R.N(+.98)ALRRPFYSNAPLEIYVQQGSGYFGLIFPGC(+57.02)PSTYEEPAQEGR.R	Y	39.03	4879.3340	43	4.4	1220.8462	4	35.98	23	1924	OB4051.raw	1.1E6	1	1	76	118	Carbamidomethylat
R.LNAQRPDNRIESEGGYIETWNPNN(+.98)Q(+.98)EFQC(+57.02)AGVALSR.T	Y	37.75	4134.9087	36	4.1	1034.7386	4	35.22	23	1877	OB4051.raw	0	0	0	35	70	Carbamidomethylat
D.NRIESEGGYIETWNPN(+.98)NQEFQC(+57.02)AGVALSR.T	Y	37.33	3339.5215	29	1.6	1114.1829	3	32.00	23	1682	OB4051.raw	4.41E4	1	1	42	70	Carbamidomethylat
R.RPFYSNAPLEIYVQQGSGYFGLIFPGC(+57.02)PSTYEEPAQEGRR.Y	Y	36.00	4580.1860	40	2.9	1146.0571	4	35.70	23	1906	OB4051.raw	0	0	0	80	119	Carbamidomethylat
S.VTFRQ(+.98)GGEENEC(+57.02)QFQR.L	Y	35.73	1984.8698	16	-1.7	662.6294	3	25.37	23	1270	OB4051.raw	2.27E4	1	1	19	34	Carbamidomethylat
R.LNAQRPDNRIESEGGYIETWN(+.98)PN(+.98)NQ(+.98)EFQC(+57.02)AGVALSR.T	Y	35.10	4135.8931	36	7.2	1034.9880	4	39.81	23	2156	OB4051.raw	0	0	0	35	70	Carbamidomethylat
S.VTFRQ(+.98)GGEENEC(+57.02)Q(+.98)FQR.L	Y	34.67	1985.8538	16	-0.2	662.9584	3	25.77	23	1287	OB4051.raw	1.03E4	1	1	19	34	Deamidation (NQ);<br>Carbamidomethylat
R.LNAQ(+.98)RPDNRIESEGGYIETWNPNNQ(+.98)EFQC(+57.02)AGVALSR.T	Y	34.61	4134.9087	36	-1.1	1034.7333	4	37.22	23	1998	OB4051.raw	0	0	0	35	70	Carbamidomethylat
R.LNAQRPDN(+.98)RIESEGGYIETWNP(+15.99)NNQ(+.98)EFQC(+57.02)AGVALSR.T	Y	33.90	4150.9038	36	7.1	1038.7406	4	30.51	23	1584	OB4051.raw	0	0	0	35	70	Carbamidomethylat
R.LNAQRPDNRIESEGGYIETWN(+.98)PN(+.98)N(+.98)Q(+.98)EFQC(+57.02)AGVALSR.T	Y	33.50	4136.8770	36	-0.1	1035.2264	4	42.89	23	2341	OB4051.raw	0	0	0	35	70	Carbamidomethylat
R.LNAQRPDNRIESEGGYIETWNPN(+.98)N(+.98)Q(+.98)EFQC(+57.02)AGVALSR.T	Y	32.85	4135.8931	36	0.2	1034.9807	4	34.95	23	1860	OB4051.raw	0	0	0	35	70	Carbamidomethylat
R.RPFYSN(+.98)APLEIYVQQ(+.98)GSGYFGLIFPGC(+57.02)PSTYEEPAQEGR.R	Y	32.75	4426.0527	39	2.6	1476.3621	3	37.32	23	2004	OB4051.raw	0	0	0	80	118	Carbamidomethylat
R.RPFYSN(+.98)APLEIYVQQGSGYFGLIFPGC(+57.02)PSTYEEPAQEGR.R	Y	32.70	4425.0688	39	4.8	1107.2798	4	36.67	23	1992	OB4051.raw	2.72E5	2	2	80	118	Carbamidomethylat
R.LNAQ(+.98)RPDN(+.98)RIESEGGYIETWNPNNQEFQC(+57.02)AGVALSR.T	Y	31.63	4134.9087	36	5.4	1034.7400	4	35.50	23	1894	OB4051.raw	0	0	0	35	70	Carbamidomethylat
R.N(+.98)ALRRPFYSNAPLEIYVQQ(+.98)GSGYFGLIFPGC(+57.02)PSTYEEPAQEGR.R	Y	31.61	4880.3179	43	6.1	1221.0942	4	36.89	23	1978	OB4051.raw	0	0	0	76	118	Carbamidomethylat
R.LNAQRPDN(+.98)RIESEGGYIETWN(+.98)P(+15.99)NNQEFQC(+57.02)AGVALSR.T	Y	31.44	4150.9038	36	6.9	1038.7404	4	31.21	23	1627	OB4051.raw	0	0	0	35	70	Carbamidomethylat
R.RPFYSNAPLEIYVQQGSGYFGLIFPG.C	Y	31.28	2919.4595	26	2.6	974.1630	3	38.46	23	2074	OB4051.raw	3.72E4	1	1	80	105	
R.RPFYSNAPLEIYVQ(+.98)QGSGYFGLIFPGC(+57.02)PSTYEEPAQ(+.98)EGR.R	Y	31.19	4426.0527	39	6.9	1107.5281	4	37.20	23	1997	OB4051.raw	0	0	0	80	118	Carbamidomethylat<br>Deamidation (NQ)
R.LNAQRPDN(+.98)RIESEGGYIETWN(+.98)PNNQ(+.98)EFQC(+57.02)AGVALSR.T	Y	30.54	4135.8931	36	6.8	1034.9875	4	36.78	23	1972	OB4051.raw	0	0	0	35	70	Carbamidomethylat
R.RPFYSNAPLEIYVQQ(+.98)GSGYFGLIFPGC(+57.02)PSTYEEPAQ(+.98)EGR.R	Y	30.00	4426.0527	39	3.8	1476.3638	3	37.10	23	1991	OB4051.raw	0	0	0	80	118	Carbamidomethylat<br>Deamidation (NQ)

Peptide	Uniq	-10lgP	Mass	Length	ppm	m/z	z	RT	Fraction	Scan	Source File	Area Digest 1. traka 13-15 kDa	#Feature	#Feature Digest 1. traka 13-15 kDa	Start	End	PTM
R.LNAQRPDNRIESEGGYIETWN(+.98)P(+15.99)NN(+.98)Q(+.98)EFQC(+57.02)AGVALSR.T	Y	29.72	4151.8877	36	5.5	1038.9849	4	30.63	23	1591	OB4051.raw	0	0	0	35	70	Carbamidomethylat
R.LNAQRPDN(+.98)RIESEGGYIETWNPNN(+.98)QEFQC(+57.02)AGVALSR.T	Y	29.37	4134.9087	36	4.3	1034.7389	4	42.10	23	2306	OB4051.raw	9.6E3	1	1	35	70	Carbamidomethylat
R.RPFYSNAPLEIYVQQGSGYFGLIFPGC(+57.02)PSTYEEPAQ(+.98)EGR.R	Y	29.16	4425.0688	39	3.8	1476.0359	3	36.58	23	1958	OB4051.raw	1.03E5	1	1	80	118	Carbamidomethylat
R.LNAQRPDN(+.98)RIESEGGYIETWNPN(+.98)NQEFQC(+57.02)AGVALSR.T	Y	28.73	4134.9087	36	3.2	1034.7378	4	39.28	23	2081	OB4051.raw	2.12E4	1	1	35	70	Carbamidomethylat
R.LNAQRPDN(+.98)RIESEGGYIETWNP(+15.99)NNQEFQC(+57.02)AGVALSR.T	Y	28.13	4149.9199	36	0.0	1038.4873	4	34.77	23	1849	OB4051.raw	0	0	0	35	70	Carbamidomethylat
R.LN(+.98)AQRPDN(+.98)RIESEGGYIETWNP(+15.99)NNQEFQC(+57.02)AGVALSR.T	Y	28.01	4150.9038	36	-1.5	1038.7317	4	30.73	23	1597	OB4051.raw	0	0	0	35	70	Carbamidomethylat
R.LN(+.98)AQRPDN(+.98)RIESEGGYIETWNPNNQEFQC(+57.02)AGVALSR.T	Y	27.78	4134.9087	36	3.2	1034.7378	4	39.28	23	2087	OB4051.raw	2.12E4	1	1	35	70	Carbamidomethylat
R.RPFYSN(+.98)APLEIYVQ(+.98)QGSGYFGLIFPGC(+57.02)PSTYEEPAQEGR.R	Y	27.51	4426.0527	39	9.8	1107.5312	4	37.50	23	2015	OB4051.raw	0	0	0	80	118	Carbamidomethylat
R.NALRRPFYSN(+.98)APLEIYVQQGSGYFGLIFPG.C	Y	27.25	3374.7087	30	2.8	1125.9133	3	37.37	23	2002	OB4051.raw	1.79E5	1	1	76	105	
R.RPFYSNAPLEIYVQQ(+.98)GSGYFGLIFPGC(+57.02)PSTYEEPAQEGR.R	Y	27.08	4425.0688	39	4.8	1107.2798	4	36.67	23	1966	OB4051.raw	1.7E5	1	1	80	118	Carbamidomethylat
R.LN(+.98)AQRPDNRIESEGGYIETWNP(+15.99)NNQEFQC(+57.02)AGVALSR.T	Y	26.84	4149.9199	36	5.0	1038.4924	4	33.40	23	1765	OB4051.raw	0	0	0	35	70	Carbamidomethylat
R.NALRRPFYSNAPLEIYVQ(+.98)QGSGYFGLIFPGC(+57.02)PSTYEEPAQEGR.R	Y	26.81	4879.3340	43	4.4	1220.8462	4	35.98	23	2033	OB4051.raw	1.1E6	1	1	76	118	Carbamidomethylat
R.LNAQRPDNRIESEGGYIETWNP(+15.99)NNQEFQC(+57.02)AGVALSR.T	Y	26.66	4148.9355	36	1.2	1038.2424	4	31.54	23	1649	OB4051.raw	0	0	0	35	70	Carbamidomethylat
R.NALRRPFYSNAPLEIYVQ(+.98)Q(+.98)GSGYFGLIFPGC(+57.02)PSTYEEPAQEGR.R	Y	26.58	4880.3179	43	6.5	1221.0947	4	37.08	23	1990	OB4051.raw	0	0	0	76	118	Carbamidomethylat
R.L(+43.01)NAQR(+14.02)PDNRIESEGGYIETWN(+.98)PNNQEFQC(+57.02)AGVALSR.T	Y	26.02	4190.9463	36	-0.4	1048.7434	4	31.43	23	1637	OB4051.raw	2.42E5	1	1	35	70	Carbamylation; Carbamidomethylat
R.RPFYSNAPLEIYVQQ(+.98)GSGYFGLIFPG.C	Y	25.69	2920.4434	26	7.8	974.4960	3	38.40	23	2070	OB4051.raw	0	0	0	80	105	
R.IESEGGYIETWNP(+15.99)NNQ(+.98)EFQC(+57.02)AGVALSR.T	Y	25.67	3085.3723	27	1.6	1029.4663	3	33.90	23	1796	OB4051.raw	1.33E4	1	1	44	70	Hydroxylation Pro; Carbamidomethylat
R.LNAQRPDNRIESEGGYIETWN(+.98)PN(+.98)N(+.98)QEFQC(+57.02)AGVALSR.T	Y	25.58	4135.8931	36	6.7	1034.9874	4	38.31	23	2064	OB4051.raw	0	0	0	35	70	Carbamidomethylat
R.LNAQRP(+15.99)DNRIESEGGYIETWNP(+15.99)NN(+.98)Q(+.98)EFQC(+57.02)AGVALSR.T	Y	24.34	4166.8989	36	8.1	1042.7405	4	30.23	23	1566	OB4051.raw	0	0	0	35	70	Hydroxylation Pro; Carbamidomethylat
S.GYFGLIFPGC(+57.02)PSTYEEPA.Q	N	23.12	2003.8975	18	-0.6	1002.9554	2	37.57	23	2023	OB4051.raw	8.65E3	1	1	97	114	Carbamidomethylat
R.LNAQRPDN(+.98)RIESEGGYIETWNPNNQEFQC(+57.02)AGVALSR.T	Y	23.05	4133.9248	36	1.5	1034.4900	4	40.45	23	2197	OB4051.raw	0	0	0	35	70	Carbamidomethylat
R.LNAQ(+.98)RPDN(+.98)RIESEGGYIETWN(+.98)PN(+.98)N(+.98)QEFQC(+57.02)AGVALSR.T	Y	22.92	4137.8608	36	8.8	1035.4816	4	38.36	23	2064	OB4051.raw	7.24E3	1	1	35	70	Carbamidomethylat
R.LNAQRPDNRIESEGGYIETWN(+.98)PN(+.98)NQEFQC(+57.02)AGVALSR.T	Y	22.51	4134.9087	36	1.9	1034.7365	4	43.14	23	2356	OB4051.raw	0	0	0	35	70	Carbamidomethylat
R.NALRRPFYSNAPLEIYVQQGSGYFGLIFP(+15.99)GC(+57.02)PSTYEEPAQEGR.R	Y	22.42	4894.3447	43	1.2	1224.5950	4	35.88	23	1962	OB4051.raw	1.86E5	1	1	76	118	Carbamidomethylat
R.LN(+.98)AQ(+.98)RP(+15.99)DN(+.98)RIESEGGYIETWNP(+15.99)NNQEFQC(+57.02)AGVALSR.T	Y	22.35	4167.8828	36	-3.7	1042.9741	4	31.99	23	1694	OB4051.raw	1.81E5	1	1	35	70	Hydroxylation Pro; Carbamidomethylat

Peptide	Uniq	-10lgP	Mass	Length	ppm	m/z	z	RT	Fraction	Scan	Source File	Area Digest 1. traka 13-15 kDa	#Feature	#Feature Digest 1. traka 13-15 kDa	Start	End	PTM
R.NALRRPFYSNAPLEIYVQQGSGYFGLIFPG.C	Y	21.43	3373.7246	30	2.7	1125.5852	3	36.90	23	1979	OB4051.raw	0	0	0	76	105	
**total 55 peptides**																	

tr|E5G077|E5G077_ARAHY

back to list

| Protein Coverage | Supporting Peptides |

Protein Coverage:

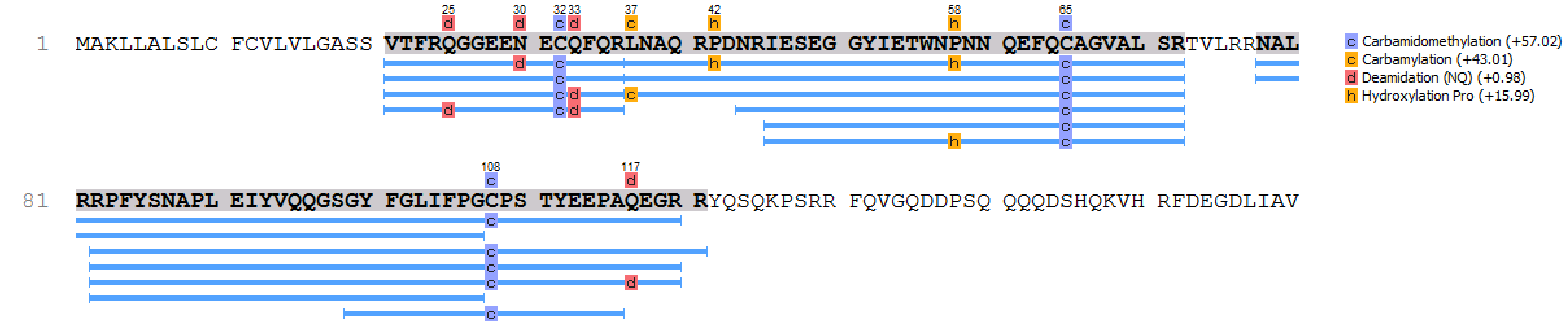

161 PTGVAFWMYN DEDTDVVTVT LSDTSSIHNQ LDQFPRRFYL AGNQEQEFLR YQQQQGSRPH YRQISPRVRG DEQENEGSNI
241 FSGFAQEFLQ HAFQVDRQTV ENLRGENERE EQGAIVTVKG GLRILSPDEE DESSRSPPNR REEFDEDRSR PQQRGKYDEN
321 RRGYKNGIEE TICSASVKKN LGRSSNPDIY NPQAGSLRSV NELDLPILGW LGLSAQHGTI YRNAMFVPHY TLNAHTIVVA
401 LNGRAHVQVV DSNGNRVYDE ELQEGHVLVV PQNFAVAAKA QSENYEYLAF KTDSRPSIAN QAGENSIIDN LPEEVVANSY
481 RLPREQARQL KNNNPFKFFV PPFDHQSMRE VA

Supporting Peptides:

Peptide	Uniq	-10lgP	Mass	Length	ppm	m/z	z	RT	Fraction	Scan	Source File	Area Digest 1. traka 13-15 kDa	#Feature	#Feature Digest 1. traka 13-15 kDa	Start	End	PTM
R.LNAQRPDNRIESEGGYIETWNPNNQEFQC(+57.02)AGVALSR.T	Y	52.73	4132.9409	36	1.0	1034.2435	4	31.17	23	1624	OB4051.raw	0	0	0	37	72	Carbamidomethylat
S.VTFRQGGEENEC(+57.02)QFQR.L	Y	47.41	1983.8857	16	0.4	662.3028	3	24.90	23	1231	OB4051.raw	3.28E4	1	1	21	36	Carbamidomethylat
R.LNAQRPDNRIESEGGYIETWNPN(+.98)NQ(+.98)EFQC(+57.02)AGVALSR.T	Y	42.21	4134.9087	36	8.2	1034.7429	4	33.83	23	1792	OB4051.raw	0	0	0	37	72	Carbamidomethylat
S.VTFRQGGEEN(+.98)EC(+57.02)QFQR.L	Y	40.64	1984.8698	16	7.4	662.6354	3	24.81	23	1229	OB4051.raw	2.27E4	1	1	21	36	Deamidation (NQ); Carbamidomethylat
R.IESEGGYIETWNPNN(+.98)QEFQC(+57.02)AGVALSR.T	Y	40.14	3069.3774	27	2.2	1024.1354	3	33.04	23	1751	OB4051.raw	1.47E4	1	1	46	72	Carbamidomethylat
S.VTFRQGGEENEC(+57.02)Q(+.98)FQR.L	Y	39.75	1984.8698	16	-1.7	662.6294	3	25.37	23	1256	OB4051.raw	2.27E4	1	1	21	36	Carbamidomethylat Deamidation (NQ)
R.LNAQRPDNRIESEGGYIETWNPNNQ(+.98)EFQC(+57.02)AGVALSR.T	Y	39.59	4133.9248	36	1.5	1034.4900	4	31.52	23	1636	OB4051.raw	2.82E6	1	1	37	72	Carbamidomethylat
R.LNAQRPDN(+.98)RIESEGGYIETWNPNNQ(+.98)EFQC(+57.02)AGVALSR.T	Y	39.44	4134.9087	36	-1.3	1034.7332	4	35.06	23	1867	OB4051.raw	0	0	0	37	72	Carbamidomethylat
R.RPFYSNAPLEIYVQQGSGYFGLIFPGC(+57.02)PSTYEEPAQEGR.R	Y	39.29	4424.0845	39	2.8	1107.0315	4	36.52	23	1956	OB4051.raw	0	0	0	82	120	Carbamidomethylat
R.N(+.98)ALRRPFYSNAPLEIYVQQGSGYFGLIFPGC(+57.02)PSTYEEPAQEGR.R	Y	39.03	4879.3340	43	4.4	1220.8462	4	35.98	23	1924	OB4051.raw	1.1E6	1	1	78	120	Carbamidomethylat
R.LNAQRPDNRIESEGGYIETWNPNN(+.98)Q(+.98)EFQC(+57.02)AGVALSR.T	Y	37.75	4134.9087	36	4.1	1034.7386	4	35.22	23	1877	OB4051.raw	0	0	0	37	72	Carbamidomethylat
D.NRIESEGGYIETWNPN(+.98)NQEFQC(+57.02)AGVALSR.T	Y	37.33	3339.5215	29	1.6	1114.1829	3	32.00	23	1682	OB4051.raw	4.41E4	1	1	44	72	Carbamidomethylat
R.RPFYSNAPLEIYVQQGSGYFGLIFPGC(+57.02)PSTYEEPAQEGRR.Y	Y	36.00	4580.1860	40	2.9	1146.0571	4	35.70	23	1906	OB4051.raw	0	0	0	82	121	Carbamidomethylat
S.VTFRQ(+.98)GGEENEC(+57.02)QFQR.L	Y	35.73	1984.8698	16	-1.7	662.6294	3	25.37	23	1270	OB4051.raw	2.27E4	1	1	21	36	Carbamidomethylat
R.LNAQRPDNRIESEGGYIETWN(+.98)PN(+.98)NQ(+.98)EFQC(+57.02)AGVALSR.T	Y	35.10	4135.8931	36	7.2	1034.9880	4	39.81	23	2156	OB4051.raw	0	0	0	37	72	Carbamidomethylat
S.VTFRQ(+.98)GGEENEC(+57.02)Q(+.98)FQR.L	Y	34.67	1985.8538	16	-0.2	662.9584	3	25.77	23	1287	OB4051.raw	1.03E4	1	1	21	36	Deamidation (NQ); Carbamidomethylat
R.LNAQ(+.98)RPDNRIESEGGYIETWNPNNQ(+.98)EFQC(+57.02)AGVALSR.T	Y	34.61	4134.9087	36	-1.1	1034.7333	4	37.22	23	1998	OB4051.raw	0	0	0	37	72	Carbamidomethylat
R.LNAQRPDN(+.98)RIESEGGYIETWNP(+15.99)NNQ(+.98)EFQC(+57.02)AGVALSR.T	Y	33.90	4150.9038	36	7.1	1038.7406	4	30.51	23	1584	OB4051.raw	0	0	0	37	72	Carbamidomethylat
R.LNAQRPDNRIESEGGYIETWN(+.98)PN(+.98)N(+.98)Q(+.98)EFQC(+57.02)AGVALSR.T	Y	33.50	4136.8770	36	-0.1	1035.2264	4	42.89	23	2341	OB4051.raw	0	0	0	37	72	Carbamidomethylat
R.LNAQRPDNRIESEGGYIETWNPN(+.98)N(+.98)Q(+.98)EFQC(+57.02)AGVALSR.T	Y	32.85	4135.8931	36	0.2	1034.9807	4	34.95	23	1860	OB4051.raw	0	0	0	37	72	Carbamidomethylat
R.RPFYSN(+.98)APLEIYVQQ(+.98)GSGYFGLIFPGC(+57.02)PSTYEEPAQEGR.R	Y	32.75	4426.0527	39	2.6	1476.3621	3	37.32	23	2004	OB4051.raw	0	0	0	82	120	Carbamidomethylat
R.RPFYSN(+.98)APLEIYVQQGSGYFGLIFPGC(+57.02)PSTYEEPAQEGR.R	Y	32.70	4425.0688	39	4.8	1107.2798	4	36.67	23	1992	OB4051.raw	2.72E5	2	2	82	120	Carbamidomethylat
R.LNAQ(+.98)RPDN(+.98)RIESEGGYIETWNPNNQEFQC(+57.02)AGVALSR.T	Y	31.63	4134.9087	36	5.4	1034.7400	4	35.50	23	1894	OB4051.raw	0	0	0	37	72	Carbamidomethylat
R.N(+.98)ALRRPFYSNAPLEIYVQQ(+.98)GSGYFGLIFPGC(+57.02)PSTYEEPAQEGR.R	Y	31.61	4880.3179	43	6.1	1221.0942	4	36.89	23	1978	OB4051.raw	0	0	0	78	120	Carbamidomethylat
R.LNAQRPDN(+.98)RIESEGGYIETWN(+.98)P(+15.99)NNQEFQC(+57.02)AGVALSR.T	Y	31.44	4150.9038	36	6.9	1038.7404	4	31.21	23	1627	OB4051.raw	0	0	0	37	72	Carbamidomethylat
R.RPFYSNAPLEIYVQQGSGYFGLIFPG.C	Y	31.28	2919.4595	26	2.6	974.1630	3	38.46	23	2074	OB4051.raw	3.72E4	1	1	82	107	

Peptide	Uniq	-10lgP	Mass	Length	ppm	m/z	z	RT	Fraction	Scan	Source File	Area Digest 1. traka 13-15 kDa	#Feature	#Feature Digest 1. traka 13-15 kDa	Start	End	PTM
R.RPFYSNAPLEIYVQ(+.98)QGSGYFGLIFPGC(+57.02)PSTYEEPAQ(+.98)EGR.R	Y	31.19	4426.0527	39	6.9	1107.5281	4	37.20	23	1997	OB4051.raw	0	0	0	82	120	Carbamidomethylat Deamidation (NQ)
R.LNAQRPDN(+.98)RIESEGGYIETWN(+.98)PNNQ(+.98)EFQC(+57.02)AGVALSR.T	Y	30.54	4135.8931	36	6.8	1034.9875	4	36.78	23	1972	OB4051.raw	0	0	0	37	72	Carbamidomethylat
R.RPFYSNAPLEIYVQQ(+.98)GSGYFGLIFPGC(+57.02)PSTYEEPAQ(+.98)EGR.R	Y	30.00	4426.0527	39	3.8	1476.3638	3	37.10	23	1991	OB4051.raw	0	0	0	82	120	Carbamidomethylat Deamidation (NQ)
R.LNAQRPDNRIESEGGYIETWN(+.98)P(+15.99)NN(+.98)Q(+.98)EFQC(+57.02)AGVALSR.T	Y	29.72	4151.8877	36	5.5	1038.9849	4	30.63	23	1591	OB4051.raw	0	0	0	37	72	Carbamidomethylat
R.LNAQRPDN(+.98)RIESEGGYIETWNPNN(+.98)QEFQC(+57.02)AGVALSR.T	Y	29.37	4134.9087	36	4.3	1034.7389	4	42.10	23	2306	OB4051.raw	9.6E3	1	1	37	72	Carbamidomethylat
R.RPFYSNAPLEIYVQQGSGYFGLIFPGC(+57.02)PSTYEEPAQ(+.98)EGR.R	Y	29.16	4425.0688	39	3.8	1476.0359	3	36.58	23	1958	OB4051.raw	1.03E5	1	1	82	120	Carbamidomethylat
R.LNAQRPDN(+.98)RIESEGGYIETWNPN(+.98)NQEFQC(+57.02)AGVALSR.T	Y	28.73	4134.9087	36	3.2	1034.7378	4	39.28	23	2081	OB4051.raw	2.12E4	1	1	37	72	Carbamidomethylat
R.LNAQRPDN(+.98)RIESEGGYIETWNP(+15.99)NNQEFQC(+57.02)AGVALSR.T	Y	28.13	4149.9199	36	0.0	1038.4873	4	34.77	23	1849	OB4051.raw	0	0	0	37	72	Carbamidomethylat
R.LN(+.98)AQRPDN(+.98)RIESEGGYIETWNP(+15.99)NNQEFQC(+57.02)AGVALSR.T	Y	28.01	4150.9038	36	-1.5	1038.7317	4	30.73	23	1597	OB4051.raw	0	0	0	37	72	Carbamidomethylat
R.LN(+.98)AQRPDN(+.98)RIESEGGYIETWNPNNQEFQC(+57.02)AGVALSR.T	Y	27.78	4134.9087	36	3.2	1034.7378	4	39.28	23	2087	OB4051.raw	2.12E4	1	1	37	72	Carbamidomethylat
R.RPFYSN(+.98)APLEIYVQ(+.98)QGSGYFGLIFPGC(+57.02)PSTYEEPAQEGR.R	Y	27.51	4426.0527	39	9.8	1107.5312	4	37.50	23	2015	OB4051.raw	0	0	0	82	120	Carbamidomethylat
R.NALRRPFYSN(+.98)APLEIYVQQGSGYFGLIFPG.C	Y	27.25	3374.7087	30	2.8	1125.9133	3	37.37	23	2002	OB4051.raw	1.79E5	1	1	78	107	
R.RPFYSNAPLEIYVQQ(+.98)GSGYFGLIFPGC(+57.02)PSTYEEPAQEGR.R	Y	27.08	4425.0688	39	4.8	1107.2798	4	36.67	23	1966	OB4051.raw	1.7E5	1	1	82	120	Carbamidomethylat
R.LN(+.98)AQRPDNRIESEGGYIETWNP(+15.99)NNQEFQC(+57.02)AGVALSR.T	Y	26.84	4149.9199	36	5.0	1038.4924	4	33.40	23	1765	OB4051.raw	0	0	0	37	72	Carbamidomethylat
R.NALRRPFYSNAPLEIYVQ(+.98)QGSGYFGLIFPGC(+57.02)PSTYEEPAQEGR.R	Y	26.81	4879.3340	43	4.4	1220.8462	4	35.98	23	2033	OB4051.raw	1.1E6	1	1	78	120	Carbamidomethylat
R.LNAQRPDNRIESEGGYIETWNP(+15.99)NNQEFQC(+57.02)AGVALSR.T	Y	26.66	4148.9355	36	1.2	1038.2424	4	31.54	23	1649	OB4051.raw	0	0	0	37	72	Carbamidomethylat
R.NALRRPFYSNAPLEIYVQ(+.98)Q(+.98)GSGYFGLIFPGC(+57.02)PSTYEEPAQEGR.R	Y	26.58	4880.3179	43	6.5	1221.0947	4	37.08	23	1990	OB4051.raw	0	0	0	78	120	Carbamidomethylat
R.L(+43.01)NAQR(+14.02)PDNRIESEGGYIETWN(+.98)PNNQEFQC(+57.02)AGVALSR.T	Y	26.02	4190.9463	36	-0.4	1048.7434	4	31.43	23	1637	OB4051.raw	2.42E5	1	1	37	72	Carbamylation; Carbamidomethylat
R.RPFYSNAPLEIYVQQ(+.98)GSGYFGLIFPG.C	Y	25.69	2920.4434	26	7.8	974.4960	3	38.40	23	2070	OB4051.raw	0	0	0	82	107	
R.IESEGGYIETWNP(+15.99)NNQ(+.98)EFQC(+57.02)AGVALSR.T	Y	25.67	3085.3723	27	1.6	1029.4663	3	33.90	23	1796	OB4051.raw	1.33E4	1	1	46	72	Hydroxylation Pro; Carbamidomethylat
R.LNAQRPDNRIESEGGYIETWN(+.98)PN(+.98)N(+.98)QEFQC(+57.02)AGVALSR.T	Y	25.58	4135.8931	36	6.7	1034.9874	4	38.31	23	2064	OB4051.raw	0	0	0	37	72	Carbamidomethylat
R.LNAQRP(+15.99)DNRIESEGGYIETWNP(+15.99)NN(+.98)Q(+.98)EFQC(+57.02)AGVALSR.T	Y	24.34	4166.8989	36	8.1	1042.7405	4	30.23	23	1566	OB4051.raw	0	0	0	37	72	Hydroxylation Pro; Carbamidomethylat
S.GYFGLIFPGC(+57.02)PSTYEEPA.Q	N	23.12	2003.8975	18	-0.6	1002.9554	2	37.57	23	2023	OB4051.raw	8.65E3	1	1	99	116	Carbamidomethylat
R.LNAQRPDN(+.98)RIESEGGYIETWNPNNQEFQC(+57.02)AGVALSR.T	Y	23.05	4133.9248	36	1.5	1034.4900	4	40.45	23	2197	OB4051.raw	0	0	0	37	72	Carbamidomethylat
R.LNAQ(+.98)RPDN(+.98)RIESEGGYIETWN(+.98)PN(+.98)N(+.98)QEFQC(+57.02)AGVALSR.T	Y	22.92	4137.8608	36	8.8	1035.4816	4	38.36	23	2064	OB4051.raw	7.24E3	1	1	37	72	Carbamidomethylat

Peptide	Uniq	-10lgP	Mass	Length	ppm	m/z	z	RT	Fraction	Scan	Source File	Area Digest 1. traka 13-15 kDa	#Feature	#Feature Digest 1. traka 13-15 kDa	Start	End	PTM
R.LNAQRPDNRIESEGGYIETWN(+.98)PN(+.98)NQEFQC(+57.02)AGVALSR.T	Y	22.51	4134.9087	36	1.9	1034.7365	4	43.14	23	2356	OB4051.raw	0	0	0	37	72	Carbamidomethylat
R.NALRRPFYSNAPLEIYVQQGSGYFGLIFP(+15.99)GC(+57.02)PSTYEEPAQEGR.R	Y	22.42	4894.3447	43	1.2	1224.5950	4	35.88	23	1962	OB4051.raw	1.86E5	1	1	78	120	Carbamidomethylat
R.LN(+.98)AQ(+.98)RP(+15.99)DN(+.98)RIESEGGYIETWNP(+15.99)NNQEFQC(+57.02)AGVALSR.T	Y	22.35	4167.8828	36	-3.7	1042.9741	4	31.99	23	1694	OB4051.raw	1.81E5	1	1	37	72	Hydroxylation Pro; Carbamidomethylat
R.NALRRPFYSNAPLEIYVQQGSGYFGLIFPG.C	Y	21.43	3373.7246	30	2.7	1125.5852	3	36.90	23	1979	OB4051.raw	0	0	0	78	107	
**total 55 peptides**																	

tr|Q0GM57|Q0GM57_ARAHY

back to list

| Protein Coverage | Supporting Peptides |

Protein Coverage:

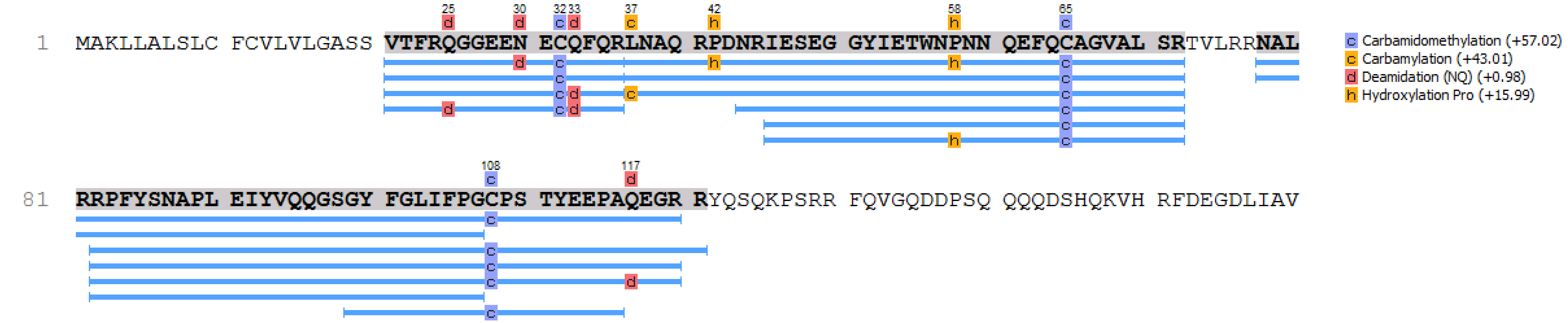

161 PTGVAFWMYN DEDTDVVTVT LSDTSSIHNQ LDQFPRRFYL AGNQEQEFLR YQQQQGSRPH YRQISPRVRG DEQENEGSNI
241 FSGFAQEFLQ HAFQVDRQTV ENLRGENERE EQGAIVTVKG GLRILSPDEE DESSRSPPSR REEFDEDRSR PQQRGKYDEN
321 RRGYKNGIEE TICSASVKKN LGRSSNPDIY NPQAGSLRSV NELDLPILGW LGLSAQHGTI YRNAMFVPHY TLNAHTIVVA
401 LNGRAHVQVV DSNGNRVYDE ELQEGHVLVV PQNFAVAAKA QSENYEYLAF KTDSRPSIAN LAGENSIIDN LPEEVVANSY
481 RLPREQARQL KNNNPFKFFV PPFDHQSMRE VA

Supporting Peptides:

Peptide	Uniq	-10lgP	Mass	Length	ppm	m/z	z	RT	Fraction	Scan	Source File	Area Digest 1. traka 13-15 kDa	#Feature	#Feature Digest 1. traka 13-15 kDa	Start	End	PTM
R.LNAQRPDNRIESEGGYIETWNPNNQEFQC(+57.02)AGVALSR.T	Y	52.73	4132.9409	36	1.0	1034.2435	4	31.17	23	1624	OB4051.raw	0	0	0	37	72	Carbamidomethylat
S.VTFRQGGEENEC(+57.02)QFQR.L	Y	47.41	1983.8857	16	0.4	662.3028	3	24.90	23	1231	OB4051.raw	3.28E4	1	1	21	36	Carbamidomethylat
R.LNAQRPDNRIESEGGYIETWNPN(+.98)NQ(+.98)EFQC(+57.02)AGVALSR.T	Y	42.21	4134.9087	36	8.2	1034.7429	4	33.83	23	1792	OB4051.raw	0	0	0	37	72	Carbamidomethylat
S.VTFRQGGEEN(+.98)EC(+57.02)QFQR.L	Y	40.64	1984.8698	16	7.4	662.6354	3	24.81	23	1229	OB4051.raw	2.27E4	1	1	21	36	Deamidation (NQ); Carbamidomethylat
R.IESEGGYIETWNPNN(+.98)QEFQC(+57.02)AGVALSR.T	Y	40.14	3069.3774	27	2.2	1024.1354	3	33.04	23	1751	OB4051.raw	1.47E4	1	1	46	72	Carbamidomethylat
S.VTFRQGGEENEC(+57.02)Q(+.98)FQR.L	Y	39.75	1984.8698	16	-1.7	662.6294	3	25.37	23	1256	OB4051.raw	2.27E4	1	1	21	36	Carbamidomethylat Deamidation (NQ)
R.LNAQRPDNRIESEGGYIETWNPNNQ(+.98)EFQC(+57.02)AGVALSR.T	Y	39.59	4133.9248	36	1.5	1034.4900	4	31.52	23	1636	OB4051.raw	2.82E6	1	1	37	72	Carbamidomethylat

Peptide	Uniq	-10lgP	Mass	Length	ppm	m/z	z	RT	Fraction	Scan	Source File	Area Digest 1. traka 13-15 kDa	#Feature	#Feature Digest 1. traka 13-15 kDa	Start	End	PTM
R.LNAQRPDN(+.98)RIESEGGYIETWNPNNQ(+.98)EFQC(+57.02)AGVALSR.T	Y	39.44	4134.9087	36	-1.3	1034.7332	4	35.06	23	1867	OB4051.raw	0	0	0	37	72	Carbamidomethylat
R.RPFYSNAPLEIYVQQGSGYFGLIFPGC(+57.02)PSTYEEPAQEGR.R	Y	39.29	4424.0845	39	2.8	1107.0315	4	36.52	23	1956	OB4051.raw	0	0	0	82	120	Carbamidomethylat
R.N(+.98)ALRRPFYSNAPLEIYVQQGSGYFGLIFPGC(+57.02)PSTYEEPAQEGR.R	Y	39.03	4879.3340	43	4.4	1220.8462	4	35.98	23	1924	OB4051.raw	1.1E6	1	1	78	120	Carbamidomethylat
R.LNAQRPDNRIESEGGYIETWNPNN(+.98)Q(+.98)EFQC(+57.02)AGVALSR.T	Y	37.75	4134.9087	36	4.1	1034.7386	4	35.22	23	1877	OB4051.raw	0	0	0	37	72	Carbamidomethylat
D.NRIESEGGYIETWNPN(+.98)NQEFQC(+57.02)AGVALSR.T	Y	37.33	3339.5215	29	1.6	1114.1829	3	32.00	23	1682	OB4051.raw	4.41E4	1	1	44	72	Carbamidomethylat
R.RPFYSNAPLEIYVQQGSGYFGLIFPGC(+57.02)PSTYEEPAQEGRR.Y	Y	36.00	4580.1860	40	2.9	1146.0571	4	35.70	23	1906	OB4051.raw	0	0	0	82	121	Carbamidomethylat
S.VTFRQ(+.98)GGEENEC(+57.02)QFQR.L	Y	35.73	1984.8698	16	-1.7	662.6294	3	25.37	23	1270	OB4051.raw	2.27E4	1	1	21	36	Carbamidomethylat
R.LNAQRPDNRIESEGGYIETWN(+.98)PN(+.98)NQ(+.98)EFQC(+57.02)AGVALSR.T	Y	35.10	4135.8931	36	7.2	1034.9880	4	39.81	23	2156	OB4051.raw	0	0	0	37	72	Carbamidomethylat
S.VTFRQ(+.98)GGEENEC(+57.02)Q(+.98)FQR.L	Y	34.67	1985.8538	16	-0.2	662.9584	3	25.77	23	1287	OB4051.raw	1.03E4	1	1	21	36	Deamidation (NQ); Carbamidomethylat
R.LNAQ(+.98)RPDNRIESEGGYIETWNPNNQ(+.98)EFQC(+57.02)AGVALSR.T	Y	34.61	4134.9087	36	-1.1	1034.7333	4	37.22	23	1998	OB4051.raw	0	0	0	37	72	Carbamidomethylat
R.LNAQRPDN(+.98)RIESEGGYIETWNP(+15.99)NNQ(+.98)EFQC(+57.02)AGVALSR.T	Y	33.90	4150.9038	36	7.1	1038.7406	4	30.51	23	1584	OB4051.raw	0	0	0	37	72	Carbamidomethylat
R.LNAQRPDNRIESEGGYIETWN(+.98)PN(+.98)N(+.98)Q(+.98)EFQC(+57.02)AGVALSR.T	Y	33.50	4136.8770	36	-0.1	1035.2264	4	42.89	23	2341	OB4051.raw	0	0	0	37	72	Carbamidomethylat
R.LNAQRPDNRIESEGGYIETWNPN(+.98)N(+.98)Q(+.98)EFQC(+57.02)AGVALSR.T	Y	32.85	4135.8931	36	0.2	1034.9807	4	34.95	23	1860	OB4051.raw	0	0	0	37	72	Carbamidomethylat
R.RPFYSN(+.98)APLEIYVQQ(+.98)GSGYFGLIFPGC(+57.02)PSTYEEPAQEGR.R	Y	32.75	4426.0527	39	2.6	1476.3621	3	37.32	23	2004	OB4051.raw	0	0	0	82	120	Carbamidomethylat
R.RPFYSN(+.98)APLEIYVQQGSGYFGLIFPGC(+57.02)PSTYEEPAQEGR.R	Y	32.70	4425.0688	39	4.8	1107.2798	4	36.67	23	1992	OB4051.raw	2.72E5	2	2	82	120	Carbamidomethylat
R.LNAQ(+.98)RPDN(+.98)RIESEGGYIETWNPNNQEFQC(+57.02)AGVALSR.T	Y	31.63	4134.9087	36	5.4	1034.7400	4	35.50	23	1894	OB4051.raw	0	0	0	37	72	Carbamidomethylat
R.N(+.98)ALRRPFYSNAPLEIYVQQ(+.98)GSGYFGLIFPGC(+57.02)PSTYEEPAQEGR.R	Y	31.61	4880.3179	43	6.1	1221.0942	4	36.89	23	1978	OB4051.raw	0	0	0	78	120	Carbamidomethylat
R.LNAQRPDN(+.98)RIESEGGYIETWN(+.98)P(+15.99)NNQEFQC(+57.02)AGVALSR.T	Y	31.44	4150.9038	36	6.9	1038.7404	4	31.21	23	1627	OB4051.raw	0	0	0	37	72	Carbamidomethylat
R.RPFYSNAPLEIYVQQGSGYFGLIFPG.C	Y	31.28	2919.4595	26	2.6	974.1630	3	38.46	23	2074	OB4051.raw	3.72E4	1	1	82	107	
R.RPFYSNAPLEIYVQ(+.98)QGSGYFGLIFPGC(+57.02)PSTYEEPAQ(+.98)EGR.R	Y	31.19	4426.0527	39	6.9	1107.5281	4	37.20	23	1997	OB4051.raw	0	0	0	82	120	Carbamidomethylat Deamidation (NQ)
R.LNAQRPDN(+.98)RIESEGGYIETWN(+.98)PNNQ(+.98)EFQC(+57.02)AGVALSR.T	Y	30.54	4135.8931	36	6.8	1034.9875	4	36.78	23	1972	OB4051.raw	0	0	0	37	72	Carbamidomethylat
R.RPFYSNAPLEIYVQQ(+.98)GSGYFGLIFPGC(+57.02)PSTYEEPAQ(+.98)EGR.R	Y	30.00	4426.0527	39	3.8	1476.3638	3	37.10	23	1991	OB4051.raw	0	0	0	82	120	Carbamidomethylat Deamidation (NQ)
R.LNAQRPDNRIESEGGYIETWN(+.98)P(+15.99)NN(+.98)Q(+.98)EFQC(+57.02)AGVALSR.T	Y	29.72	4151.8877	36	5.5	1038.9849	4	30.63	23	1591	OB4051.raw	0	0	0	37	72	Carbamidomethylat
R.LNAQRPDN(+.98)RIESEGGYIETWNPNN(+.98)QEFQC(+57.02)AGVALSR.T	Y	29.37	4134.9087	36	4.3	1034.7389	4	42.10	23	2306	OB4051.raw	9.6E3	1	1	37	72	Carbamidomethylat
R.RPFYSNAPLEIYVQQGSGYFGLIFPGC(+57.02)PSTYEEPAQ(+.98)EGR.R	Y	29.16	4425.0688	39	3.8	1476.0359	3	36.58	23	1958	OB4051.raw	1.03E5	1	1	82	120	Carbamidomethylat
R.LNAQRPDN(+.98)RIESEGGYIETWNPN(+.98)NQEFQC(+57.02)AGVALSR.T	Y	28.73	4134.9087	36	3.2	1034.7378	4	39.28	23	2081	OB4051.raw	2.12E4	1	1	37	72	Carbamidomethylat

Peptide	Uniq	-10lgP	Mass	Length	ppm	m/z	z	RT	Fraction	Scan	Source File	Area Digest 1. traka 13-15 kDa	#Feature	#Feature Digest 1. traka 13-15 kDa	Start	End	PTM
R.LNAQRPDN(+.98)RIESEGGYIETWNP(+15.99)NNQEFQC(+57.02)AGVALSR.T	Y	28.13	4149.9199	36	0.0	1038.4873	4	34.77	23	1849	OB4051.raw	0	0	0	37	72	Carbamidomethylat
R.LN(+.98)AQRPDN(+.98)RIESEGGYIETWNP(+15.99)NNQEFQC(+57.02)AGVALSR.T	Y	28.01	4150.9038	36	-1.5	1038.7317	4	30.73	23	1597	OB4051.raw	0	0	0	37	72	Carbamidomethylat
R.LN(+.98)AQRPDN(+.98)RIESEGGYIETWNPNNQEFQC(+57.02)AGVALSR.T	Y	27.78	4134.9087	36	3.2	1034.7378	4	39.28	23	2087	OB4051.raw	2.12E4	1	1	37	72	Carbamidomethylat
R.RPFYSN(+.98)APLEIYVQ(+.98)QGSGYFGLIFPGC(+57.02)PSTYEEPAQEGR.R	Y	27.51	4426.0527	39	9.8	1107.5312	4	37.50	23	2015	OB4051.raw	0	0	0	82	120	Carbamidomethylat
R.NALRRPFYSN(+.98)APLEIYVQQGSGYFGLIFPG.C	Y	27.25	3374.7087	30	2.8	1125.9133	3	37.37	23	2002	OB4051.raw	1.79E5	1	1	78	107	
R.RPFYSNAPLEIYVQQ(+.98)GSGYFGLIFPGC(+57.02)PSTYEEPAQEGR.R	Y	27.08	4425.0688	39	4.8	1107.2798	4	36.67	23	1966	OB4051.raw	1.7E5	1	1	82	120	Carbamidomethylat
R.LN(+.98)AQRPDNRIESEGGYIETWNP(+15.99)NNQEFQC(+57.02)AGVALSR.T	Y	26.84	4149.9199	36	5.0	1038.4924	4	33.40	23	1765	OB4051.raw	0	0	0	37	72	Carbamidomethylat
R.NALRRPFYSNAPLEIYVQ(+.98)QGSGYFGLIFPGC(+57.02)PSTYEEPAQEGR.R	Y	26.81	4879.3340	43	4.4	1220.8462	4	35.98	23	2033	OB4051.raw	1.1E6	1	1	78	120	Carbamidomethylat
R.LNAQRPDNRIESEGGYIETWNP(+15.99)NNQEFQC(+57.02)AGVALSR.T	Y	26.66	4148.9355	36	1.2	1038.2424	4	31.54	23	1649	OB4051.raw	0	0	0	37	72	Carbamidomethylat
R.NALRRPFYSNAPLEIYVQ(+.98)Q(+.98)GSGYFGLIFPGC(+57.02)PSTYEEPAQEGR.R	Y	26.58	4880.3179	43	6.5	1221.0947	4	37.08	23	1990	OB4051.raw	0	0	0	78	120	Carbamidomethylat
R.L(+43.01)NAQR(+14.02)PDNRIESEGGYIETWN(+.98)PNNQEFQC(+57.02)AGVALSR.T	Y	26.02	4190.9463	36	-0.4	1048.7434	4	31.43	23	1637	OB4051.raw	2.42E5	1	1	37	72	Carbamylation; Carbamidomethylat
R.RPFYSNAPLEIYVQQ(+.98)GSGYFGLIFPG.C	Y	25.69	2920.4434	26	7.8	974.4960	3	38.40	23	2070	OB4051.raw	0	0	0	82	107	
R.IESEGGYIETWNP(+15.99)NNQ(+.98)EFQC(+57.02)AGVALSR.T	Y	25.67	3085.3723	27	1.6	1029.4663	3	33.90	23	1796	OB4051.raw	1.33E4	1	1	46	72	Hydroxylation Pro; Carbamidomethylat
R.LNAQRPDNRIESEGGYIETWN(+.98)PN(+.98)N(+.98)QEFQC(+57.02)AGVALSR.T	Y	25.58	4135.8931	36	6.7	1034.9874	4	38.31	23	2064	OB4051.raw	0	0	0	37	72	Carbamidomethylat
R.LNAQRP(+15.99)DNRIESEGGYIETWNP(+15.99)NN(+.98)Q(+.98)EFQC(+57.02)AGVALSR.T	Y	24.34	4166.8989	36	8.1	1042.7405	4	30.23	23	1566	OB4051.raw	0	0	0	37	72	Hydroxylation Pro; Carbamidomethylat
S.GYFGLIFPGC(+57.02)PSTYEEPA.Q	N	23.12	2003.8975	18	-0.6	1002.9554	2	37.57	23	2023	OB4051.raw	8.65E3	1	1	99	116	Carbamidomethylat
R.LNAQRPDN(+.98)RIESEGGYIETWNPNNQEFQC(+57.02)AGVALSR.T	Y	23.05	4133.9248	36	1.5	1034.4900	4	40.45	23	2197	OB4051.raw	0	0	0	37	72	Carbamidomethylat
R.LNAQ(+.98)RPDN(+.98)RIESEGGYIETWN(+.98)PN(+.98)N(+.98)QEFQC(+57.02)AGVALSR.T	Y	22.92	4137.8608	36	8.8	1035.4816	4	38.36	23	2064	OB4051.raw	7.24E3	1	1	37	72	Carbamidomethylat
R.LNAQRPDNRIESEGGYIETWN(+.98)PN(+.98)NQEFQC(+57.02)AGVALSR.T	Y	22.51	4134.9087	36	1.9	1034.7365	4	43.14	23	2356	OB4051.raw	0	0	0	37	72	Carbamidomethylat
R.NALRRPFYSNAPLEIYVQQGSGYFGLIFP(+15.99)GC(+57.02)PSTYEEPAQEGR.R	Y	22.42	4894.3447	43	1.2	1224.5950	4	35.88	23	1962	OB4051.raw	1.86E5	1	1	78	120	Carbamidomethylat
R.LN(+.98)AQ(+.98)RP(+15.99)DN(+.98)RIESEGGYIETWNP(+15.99)NNQEFQC(+57.02)AGVALSR.T	Y	22.35	4167.8828	36	-3.7	1042.9741	4	31.99	23	1694	OB4051.raw	1.81E5	1	1	37	72	Hydroxylation Pro; Carbamidomethylat
R.NALRRPFYSNAPLEIYVQQGSGYFGLIFPG.C	Y	21.43	3373.7246	30	2.7	1125.5852	3	36.90	23	1979	OB4051.raw	0	0	0	78	107	
**total 55 peptides**																	

Peptide List

Prepared with PEAKS ™ (bioinfor.com)

Summary

1. Notes Gastric Digest Raw peanut Band #2 15-17 kDa

2. Result Statistics

Figure 1. False discovery rate (FDR) curve. X axis is the number of peptide-spectrum matches (PSM) being kept. Y axis is the corresponding FDR.

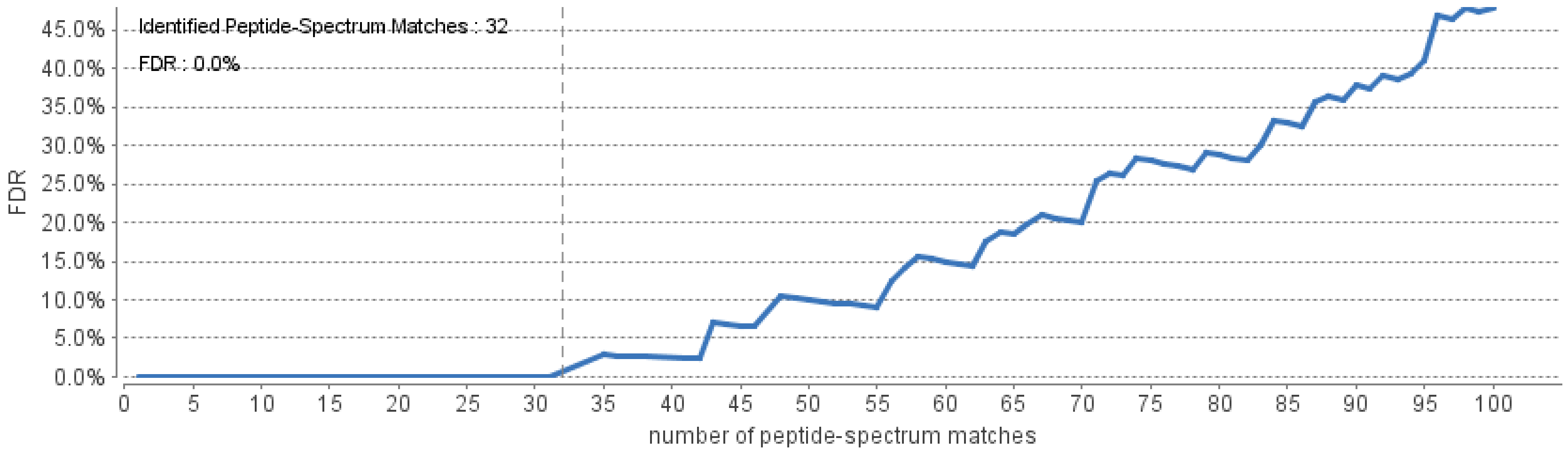

Figure 2. PSM score distribution. **(a)** Distribution of PEAKS peptide score; **(b)** Scatterplot of PEAKS peptide score versus precursor mass error.

(a)

(b)

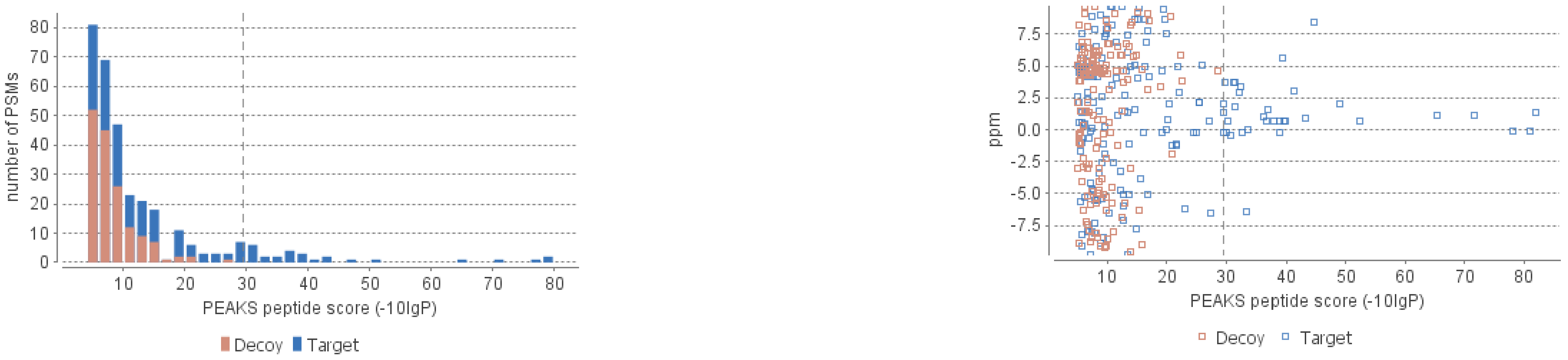

Figure 3. Distribution of peptide feature detection. **(a)** Feature m/z distribution; **(b)** Feature RT distribution

(a) **(b)**

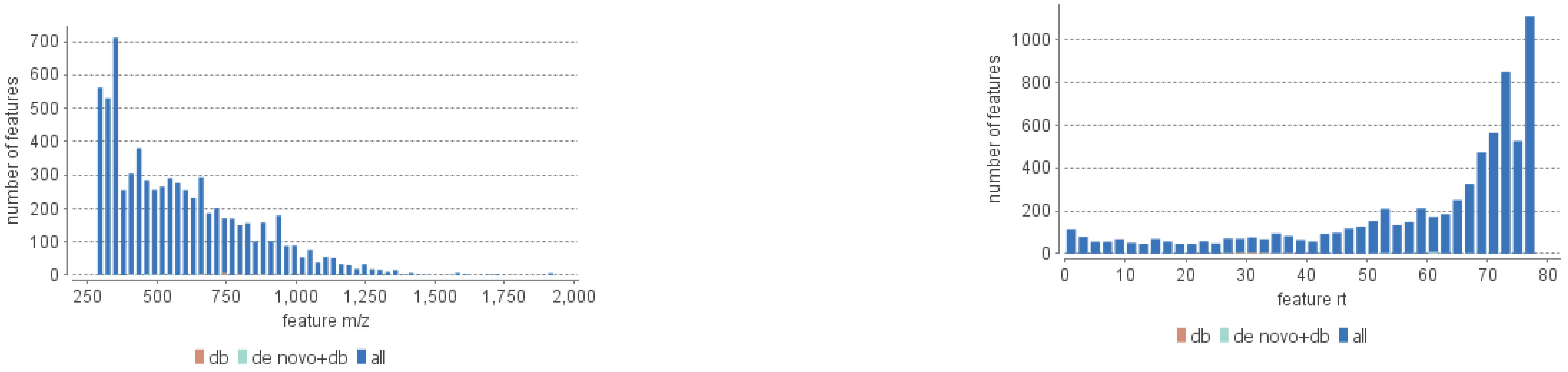

Figure 4. Distribution of identified peptide features. **(a)** Feature abundance distribution **(b)** *De novo* sequencing validation.

(a)

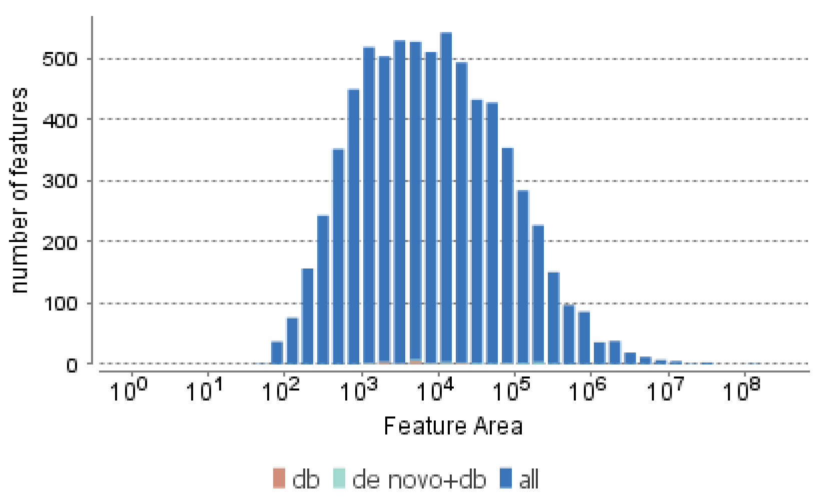

(b)

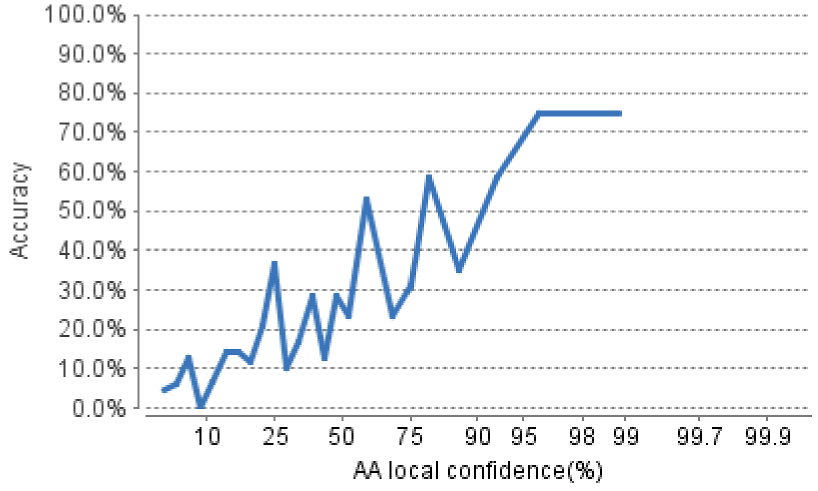

Table 1. Statistics of data.

# of MS scans	3089
# of MS/MS scans	1041
# of Features	7127
# of Chimera scans	77

Table 2. Result filtration parameters.

Peptide -10lgP	≥29.5
PTM Ascore	≥20
Protein -10lgP	≥20
Proteins unique peptides	≥1
De novo ALC Score	≥50%

Table 4. PTM profile.

Name	ΔMass	Position	#PSM	-10lgP	Abundance	AScore
Deamidation	.98	NQ	19	52.37	6.35E3	1000.00
Carbamidomethyl	57.02	C	12	71.58	6.12E3	1000.00
HydPro	15.99	P	8	52.37	6.35E3	57.45
Dimethylation(KR)	28.03	K	3	80.90	4.12E3	1000.00
EtOH	44.03	K	1	30.01	1.18E4	55.39

Table 3. Statistics of filtered result.

Peptide-Spectrum Matches	32
Peptide sequences	26
Protein groups	2
Proteins	4
Proteins (#Unique Peptides)	1 (>2); 0 (=2); 3 (=1);
FDR (Peptide-Spectrum Matches)	0.0%
FDR (Peptide Sequences)	0.0%
FDR (Protein Group)	0.0%
De Novo Only Spectra	32
# of identified Features	36
# of identified MS/MS scans	32

3. Experiment Control

Figure 5. Precursor mass error of peptide-spectrum matches (PSM) in filtered result. **(a)** Distribution of precursor mass error in ppm; **(b)** Scatterplot of precursor m/z versus precursor mass error in ppm.

(a)

(b)

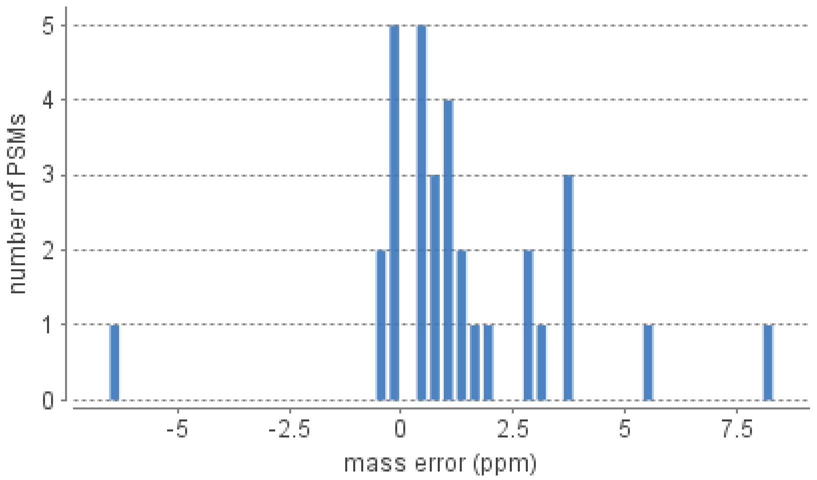

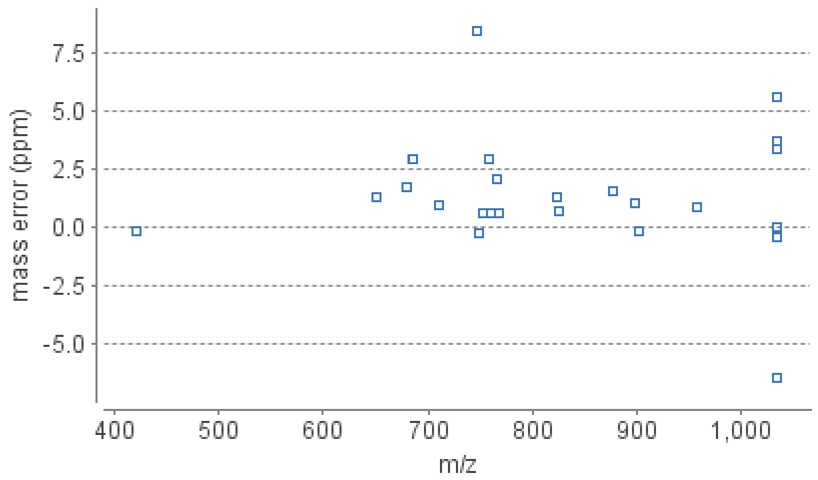

Table 5. Number of identified peptides in each sample by the number of missed cleavages

Missed Cleavages	0	1	2	3	4+
Digest 2. traka 16 kDa	15	11	0	0	0

4. Other Information

Table 6. Search parameters.

Search Engine Name: PEAKS
Parent Mass Error Tolerance: 10.0 ppm
Fragment Mass Error Tolerance: 0.5 Da
Precursor Mass Search Type: monoisotopic
Enzyme: Trypsin
Max Missed Cleavages: 2
Digest Mode: Unspecific
Fixed Modifications:

Table 7. Instrument parameters.

Fractions: OB4052.raw
Ion Source: ESI(nano-spray)
Fragmentation Mode: CID, CAD(y and b ions)
MS Scan Mode: FT-ICR/Orbitrap
MS/MS Scan Mode: Linear Ion Trap

Carbamidomethylation: 57.02
Variable Modifications:
Deamidation (NQ): 0.98
Oxidation (M): 15.99
Hydroxylation Pro: 15.99
Acetylation (K): 42.01
Acetylation (Protein N-term): 42.01
Acetylation (N-term): 42.01
Amidation: -0.98
Beta-methylthiolation: 45.99
and 305 more...
Max Variable PTM Per Peptide: 5
Database: Uniprot_Peanut-3818_Jul18
Taxon: All
Contaminant Database: contaminantsMQ_mar19
Searched Entry: 1723
FDR Estimation: Enabled
De novo score (ALC%) threshold: 15
Peptide hit threshold (-10logP): 30.0
Peaks run ID: 466
Merge Options: no merge
Precursor Options: corrected
Charge Options: no correction
Filter Charge: 2 - 8
Process: true
Associate chimera: yes

Protein List

Protein Accession Contains:
Protein Description Contains:
Peptide Sample Area >=
Protein Ptm Contains:

Protein Group	Protein ID	Accession	-10lgP	Coverage (%)	Coverage (%) Digest 2. traka 16 kDa	Area Digest 2. traka 16 kDa	#Peptides	#Unique	#Spec Digest 2. traka 16 kDa	PTM	Avg. Mass	Description
**1**	20574	tr|A1DZF0|A1DZF0_ARAHY	108.77	20	20	1.03E5	6	6	17	Y	60375	Arachin 6 OS=Arachis hypogaea OX=3818 PE=2 SV=1
**3**	20593	tr|Q6IWG5|Q6IWG5_ARAHY	33.67	7	7	0E0	1	1	2	Y	58061	Glycinin (Fragment) OS=Arachis hypogaea OX=3818 PE=2 SV=1
**3**	20595	tr|E5G077|E5G077_ARAHY	33.67	7	7	0E0	1	1	2	Y	58305	Ara h 3 allergen OS=Arachis hypogaea OX=3818 GN=ara h 3 PE=3 SV=1
**3**	20594	tr|Q0GM57|Q0GM57_ARAHY	33.67	7	7	0E0	1	1	2	Y	58263	Iso-Ara h3 OS=Arachis hypogaea OX=3818 PE=2 SV=1
**total 4 proteins**												

tr|A1DZF0|A1DZF0_ARAHY

back to list

| **Protein Coverage** | **Supporting Peptides** |

Protein Coverage:

```
  1 MAKLLELSFC FCFLVLGASS ISFRQQPEEN ACQFQRLNAQ RPDNRIESEG GYIETWNPNN QEFECAGVAL SRLVLRRNAL
 81 RRPFYSNAPQ EIFIQQGRGY FGLIFPGCPS TYEEPAQQGR RYQSQRPPRR LQEEDQSQQQ QDSHQKVHRF NEGDLIAVPT
161 GVAFWLYNDH DTDVVAVSLT DTNNNDNQLD QFPRRFNLAG NHEQEFLRYQ QQSRQSRRRS LPLSPYSPQP GQEDREFSPQ
241 GQHGRRERAG QEQENEGGNI FSGFTSEFLA QAFQVDDRQI VQNLRGENES EEQGAIVTVK GGLRILSPDR KSPDEEEEYD
321 EDEYAEEERQ QDRRRGRGSR GSGNGIEETI CTATVKKNIG RNRSPDIYNP QAGSLKTANE LNLLILRWLG LSAEYGNLYR
401 NALFVPHYNT NAHSIIYALR GRAHVQVVDS NGNRVYDEEL QEGHVLVVPQ NFAVAGKSQS ENFEYVAFKT DSRPSIANLA
481 GENSFIDNLP EEVVANSYGL PREQARQLKN NNPFKFFVPP FQQSPRAVA
```

c Carbamidomethylation (+57.02)
d Deamidation (NQ) (+0.98)
h Hydroxylation Pro (+15.99)

Supporting Peptides:

Peptide	Uniq	-10lgP	Mass	Length	ppm	m/z	z	RT	Fraction	Scan	Source File	Area Digest 2. traka 16 kDa	#Feature	#Feature Digest 2. traka 16 kDa	Start	End	PTM
R.SLPLSP(+15.99)YSPQ(+.98)PGQ(+.98)E.D	Y	52.37	1516.6932	14	0.6	759.3544	2	30.74	24	1548	OB4052.raw	6.35E3	1	1	220	233	Hydroxylation Pro; Deamidation (NQ)
R.SLPLSP(+15.99)YSP(+15.99)QPGQ(+.98)E.D	Y	48.93	1531.7041	14	2.1	766.8609	2	29.52	24	1474	OB4052.raw	5.77E3	1	1	220	233	Hydroxylation Pro
R.RPFYSNAPQ(+.98)EIFIQQGR.G	Y	41.34	2051.0225	17	3.0	684.6835	3	30.71	24	1538	OB4052.raw	1.96E3	1	1	82	98	
R.SLPLSP(+15.99)YSP(+15.99)Q(+.98)PGQ(+.98)E.D	Y	39.89	1532.6881	14	0.6	767.3518	2	29.82	24	1502	OB4052.raw	1.87E4	1	1	220	233	Hydroxylation Pro; Deamidation (NQ)
R.SLPLSP(+15.99)YSP(+15.99)Q(+.98)PGQ(+.98)ED.R	Y	39.68	1647.7151	15	0.7	824.8654	2	29.89	24	1497	OB4052.raw	5.66E3	1	1	220	234	Hydroxylation Pro; Deamidation (NQ)
R.LNAQ(+.98)RPDNRIESEGGYIETWN(+.98)PNN(+.98)QEFEC(+57.02)AGVALSR.L	Y	39.39	4136.8770	36	5.6	1035.2323	4	31.77	24	1609	OB4052.raw	0	0	0	37	72	Carbamidomethylation
R.GYFGLIFPGC(+57.02)PSTYEEPAQ(+.98)Q(+.98)GRR.Y	Y	37.00	2631.2063	23	1.5	878.0774	3	33.53	24	1707	OB4052.raw	5.44E3	1	1	99	121	Carbamidomethylation; Deamidation (NQ)
R.LNAQRPDNRIESEGGYIETWN(+.98)PNNQEFEC(+57.02)AGVALSR.L	Y	32.38	4134.9087	36	3.4	1034.7379	4	31.42	24	1588	OB4052.raw	0	0	0	37	72	Carbamidomethylation
R.SLPLSP(+15.99)YSPQ(+.98)PGQE.D	Y	32.20	1515.7092	14	3.0	758.8641	2	30.43	24	1531	OB4052.raw	1.63E3	1	1	220	233	Hydroxylation Pro
R.LN(+.98)AQRPDNRIESEGGYIETWNPNNQ(+.98)EFEC(+57.02)AGVALSR.L	Y	31.50	4135.8931	36	3.7	1034.9844	4	31.68	24	1631	OB4052.raw	1.74E4	1	1	37	72	Carbamidomethylation
S.ISFRQ(+.98)QPEENAC(+57.02)Q(+.98)FQR.L	Y	31.48	2038.9166	16	1.7	680.6473	3	27.27	24	1357	OB4052.raw	3.93E3	1	1	21	36	Carbamidomethylation; Deamidation (NQ)
R.LN(+.98)AQRPDNRIESEGGYIETWNPNN(+.98)QEFEC(+57.02)AGVALSR.L	Y	31.28	4135.8931	36	3.7	1034.9844	4	31.68	24	1615	OB4052.raw	1.74E4	1	1	37	72	Carbamidomethylation
R.LNAQRPDNRIESEGGYIETWNPNNQ(+.98)EFEC(+57.02)AGVALSR.L	Y	30.64	4134.9087	36	-0.4	1034.7340	4	31.55	24	1596	OB4052.raw	0	0	0	37	72	Carbamidomethylation
R.LNAQRPDN(+.98)RIESEGGYIETWN(+.98)PNNQEFEC(+57.02)AGVALSR.L	Y	29.79	4135.8931	36	3.7	1034.9844	4	31.68	24	1612	OB4052.raw	1.74E4	1	1	37	72	Carbamidomethylation
R.SLPLSP(+15.99)YSP(+15.99)Q(+.98)PGQED.R	Y	29.58	1646.7311	15	1.3	824.3739	2	29.57	24	1479	OB4052.raw	1.63E3	1	1	220	234	Hydroxylation Pro
**total 15 peptides**																	

tr|Q6IWG5|Q6IWG5_ARAHY
back to list

| Protein Coverage | Supporting Peptides |

Protein Coverage:

63
C

1 KLLALSLCFC VLVLGASSVT FRQGGEENEC QFQR**LNAQRP DNRIESEGGY IETWNPNNQE FQCAGVALSR** TVLRRNALRR

C Carbamidomethylation (+57.02)

81 PFYSNAPLEI YVQQGSGYFG LIFPGCPSTY EEPAQEGRRY QSQKPSRRFQ VGQDDPSQQQ QDSHQKVHRF DEGDLIAVPT
161 GVAFWMYNDE DTDVVTVTLS DTSSIHNQLD QFPRRFYLAG NQEQEFLRYQ QQQGSRPHYR QISPRVRGDE QENEGSNIFS
241 GFAQEFLQHA FQVDRQTVEN LRGENEREEQ GAIVTVKGGL RILSPDEEDE SSRSPPSRRE EFDEDRSRPQ QRGKYDENRR
321 GYKNGIEETI CSASVKKNLG RSSNPDIYNP QAGSLRSVNE LDLPILGWLG LSAQHGTIYR NAMFVPHYTL NAHTIVVALN
401 GRAHVQVVDS NGNRVYDEEL QEGHVLVVPQ NFAVAAKAQS ENYEYLAFKT DSRPSIANLA GENSIIDNLP EEVVANSYRL
481 PREQARQLKN NNPFKFFVPP FDHQSMREVA

Supporting Peptides:

Peptide	Uniq	-10lgP	Mass	Length	ppm	m/z	z	RT	Fraction	Scan	Source File	Area Digest 2. traka 16 kDa	#Feature	#Feature Digest 2. traka 16 kDa	Start	End	PTM
R.LNAQRPDNRIESEGGYIETWNPN(+.98)N(+.98)QEFQC(+57.02)AGVALSR.T	Y	33.67	4134.9087	36	0.0	1034.7345	4	31.69	24	1604	OB4052.raw	0	0	0	35	70	Carbamidomethylation
R.LNAQRPDNRIESEGGYIETWNPNNQEFQC(+57.02)AGVALSR.T	Y	33.40	4132.9409	36	-6.5	1034.2358	4	31.45	24	1590	OB4052.raw	0	0	0	35	70	Carbamidomethylation
**total 2 peptides**																	

tr|E5G077|E5G077_ARAHY
back to list

| Protein Coverage | Supporting Peptides |

Protein Coverage:

65
C

1 MAKLLALSLC FCVLVLGASS VTFRQGGEEN ECQFQR**LNAQ RPDNRIESEG GYIETWNPNN QEFQCAGVAL SR**TVLRRNAL

C Carbamidomethylation (+57.02)

81 RRPFYSNAPL EIYVQQGSGY FGLIFPGCPS TYEEPAQEGR RYQSQKPSRR FQVGQDDPSQ QQQDSHQKVH RFDEGDLIAV
161 PTGVAFWMYN DEDTDVVTVT LSDTSSIHNQ LDQFPRRFYL AGNQEQEFLR YQQQQGSRPH YRQISPRVRG DEQENEGSNI
241 FSGFAQEFLQ HAFQVDRQTV ENLRGENERE EQGAIVTVKG GLRILSPDEE DESSRSPPNR REEFDEDRSR PQQRGKYDEN
321 RRGYKNGIEE TICSASVKKN LGRSSNPDIY NPQAGSLRSV NELDLPILGW LGLSAQHGTI YRNAMFVPHY TLNAHTIVVA
401 LNGRAHVQVV DSNGNRVYDE ELQEGHVLVV PQNFAVAAKA QSENYEYLAF KTDSRPSIAN QAGENSIIDN LPEEVVANSY
481 RLPREQARQL KNNNPFKFFV PPFDHQSMRE VA

Supporting Peptides:

Peptide	Uniq	-10lgP	Mass	Length	ppm	m/z	z	RT	Fraction	Scan	Source File	Area Digest 2. traka 16 kDa	#Feature	#Feature Digest 2. traka 16 kDa	Start	End	PTM
R.LNAQRPDNRIESEGGYIETWNPN(+.98)N(+.98)QEFQC(+57.02)AGVALSR.T	Y	33.67	4134.9087	36	0.0	1034.7345	4	31.69	24	1604	OB4052.raw	0	0	0	37	72	Carbamidomethylation
R.LNAQRPDNRIESEGGYIETWNPNNQEFQC(+57.02)AGVALSR.T	Y	33.40	4132.9409	36	-6.5	1034.2358	4	31.45	24	1590	OB4052.raw	0	0	0	37	72	Carbamidomethylation
**total 2 peptides**																	

tr|Q0GM57|Q0GM57_ARAHY

back to list

| Protein Coverage | Supporting Peptides |

Protein Coverage:

65 C

```
  1 MAKLLALSLC FCVLVLGASS VTFRQGGEEN ECQFQRLNAQ RPDNRIESEG GYIETWNPNN QEFQCAGVAL SRTVLRRNAL
 81 RRPFYSNAPL EIYVQQGSGY FGLIFPGCPS TYEEPAQEGR RYQSQKPSRR FQVGQDDPSQ QQQDSHQKVH RFDEGDLIAV
161 PTGVAFWMYN DEDTDVVTVT LSDTSSIHNQ LDQFPRRFYL AGNQEQEFLR YQQQQGSRPH YRQISPRVRG DEQENEGSNI
241 FSGFAQEFLQ HAFQVDRQTV ENLRGENERE EQGAIVTVKG GLRILSPDEE DESSRSPPSR REEFDEDRSR PQQRGKYDEN
321 RRGYKNGIEE TICSASVKKN LGRSSNPDIY NPQAGSLRSV NELDLPILGW LGLSAQHGTI YRNAMFVPHY TLNAHTIVVA
401 LNGRAHVQVV DSNGNRVYDE ELQEGHVLVV PQNFAVAAKA QSENYEYLAF KTDSRPSIAN LAGENSIIDN LPEEVVANSY
481 RLPREQARQL KNNNPFKFFV PPFDHQSMRE VA
```

C Carbamidomethylation (+57.02)

Supporting Peptides:

Peptide	Uniq	-10lgP	Mass	Length	ppm	m/z	z	RT	Fraction	Scan	Source File	Area Digest 2. traka 16 kDa	#Feature	#Feature Digest 2. traka 16 kDa	Start	End	PTM
R.LNAQRPDNRIESEGGYIETWNPN(+.98)N(+.98)QEFQC(+57.02)AGVALSR.T	Y	33.67	4134.9087	36	0.0	1034.7345	4	31.69	24	1604	OB4052.raw	0	0	0	37	72	Carbamidomethylation
R.LNAQRPDNRIESEGGYIETWNPNNQEFQC(+57.02)AGVALSR.T	Y	33.40	4132.9409	36	-6.5	1034.2358	4	31.45	24	1590	OB4052.raw	0	0	0	37	72	Carbamidomethylation
**total 2 peptides**																	

Peptide List

Prepared with PEAKS ™ (bioinfor.com)

1. Notes Gastric Digest Raw peanut Band #3 17-18 kDa

2. Result Statistics

Figure 1. False discovery rate (FDR) curve. X axis is the number of peptide-spectrum matches (PSM) being kept. Y axis is the corresponding FDR.

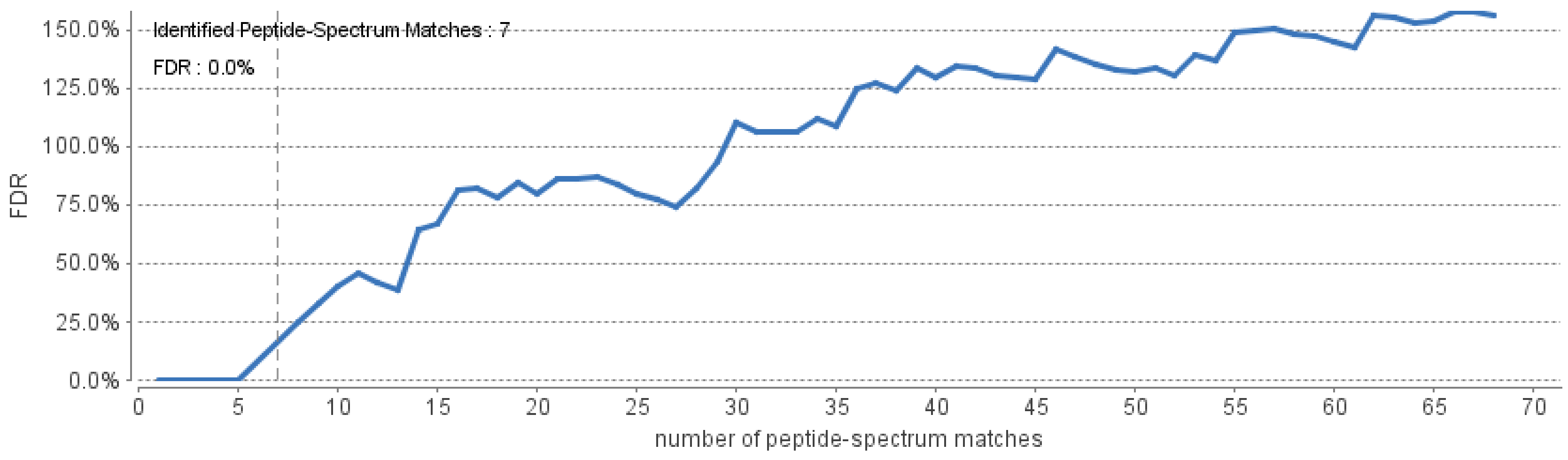

Figure 2. PSM score distribution. **(a)** Distribution of PEAKS peptide score; **(b)** Scatterplot of PEAKS peptide score versus precursor mass error.

(a)

(b)

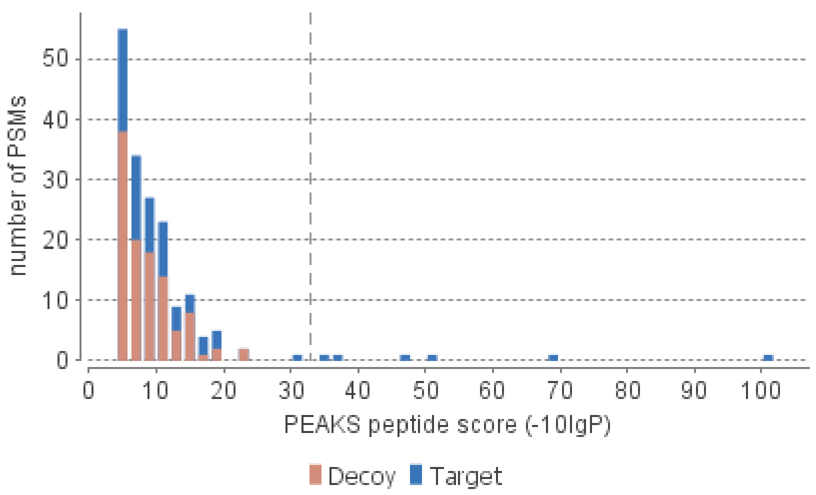

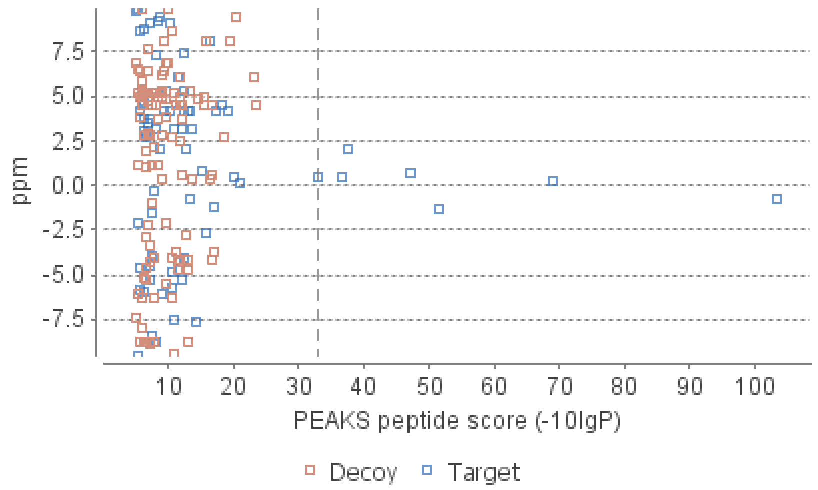

Figure 3. Distribution of peptide feature detection. **(a)** Feature m/z distribution; **(b)** Feature RT distribution

(a)

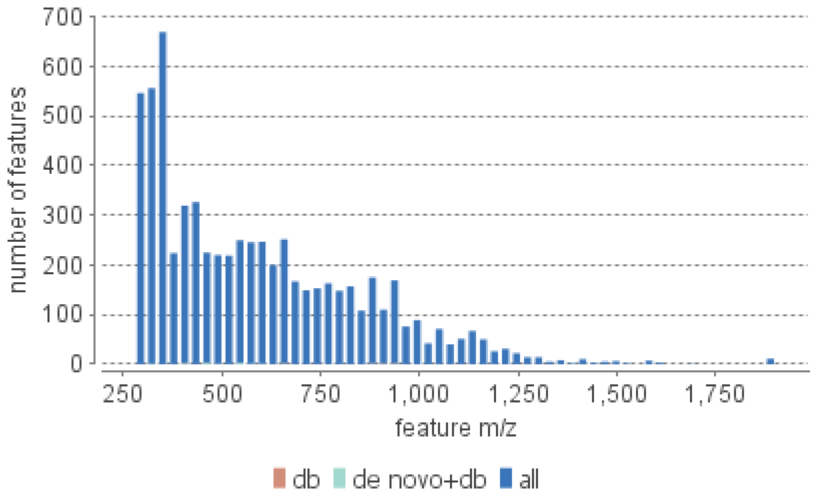

(b)

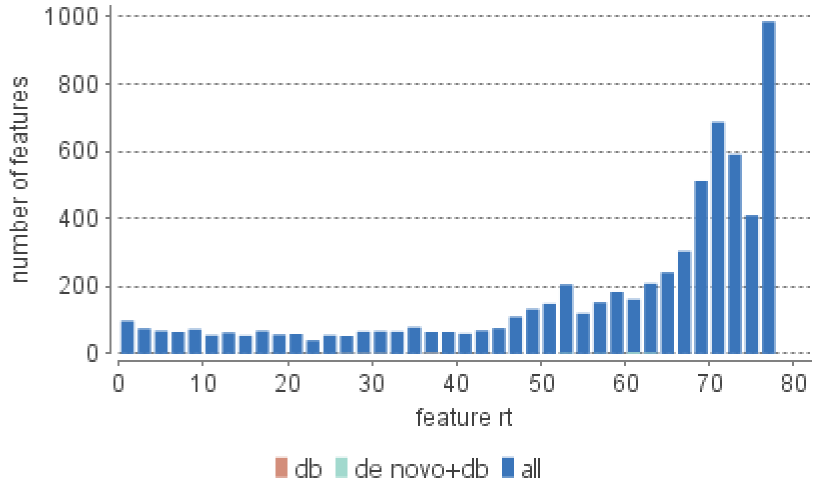

Figure 4. Distribution of identified peptide features. **(a)** Feature abundance distribution **(b)** *De novo* sequencing validation.

(a)

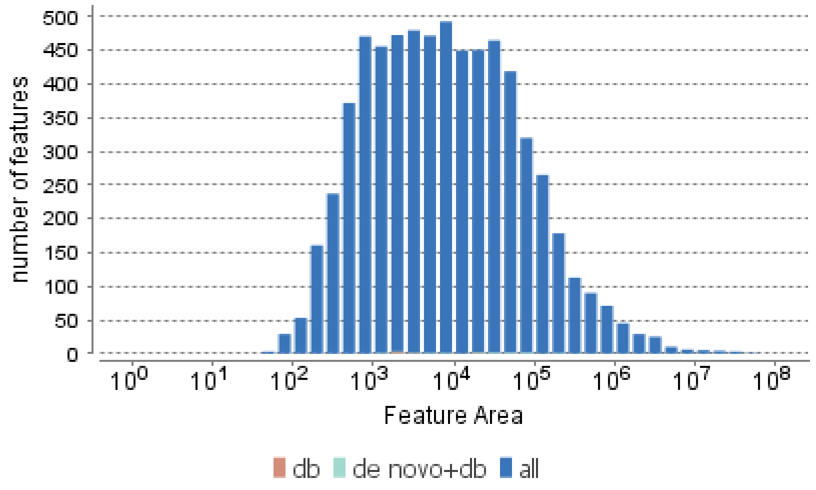

(b)

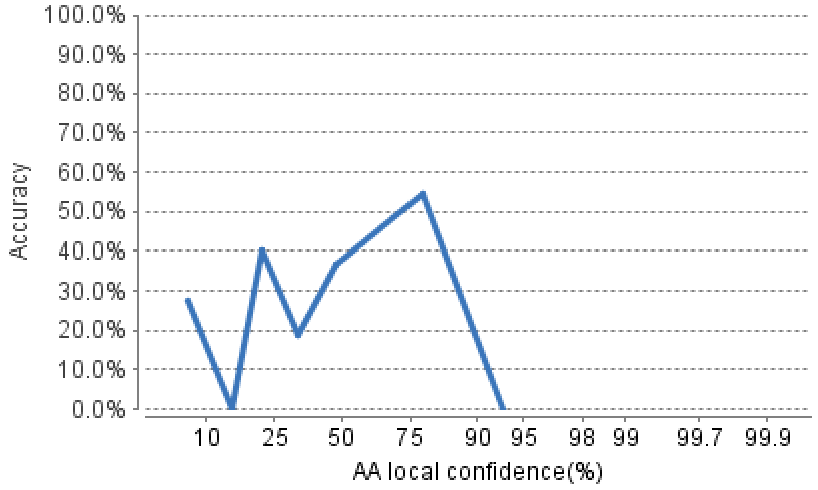

Table 1. Statistics of data.

# of MS scans	3210
# of MS/MS scans	780
# of Features	6650
# of Chimera scans	63

Table 2. Result filtration parameters.

Peptide -10lgP	≥32.9
PTM Ascore	≥20
Protein -10lgP	≥20
Proteins unique peptides	≥1
De novo ALC Score	≥50%

Table 4. PTM profile.

Name	ΔMass	Position	#PSM	-10lgP	Abundance	AScore
Deamidation	.98	Q	1	47.17	3.05E3	1000.00
Carbamidomethyl	57.02	C	1	69.09	2.44E3	1000.00

Table 3. Statistics of filtered result.

Peptide-Spectrum Matches	7
Peptide sequences	6
Protein groups	1
Proteins	10
Proteins (#Unique Peptides)	0 (>2); 0 (=2); 10 (=1);
FDR (Peptide-Spectrum Matches)	0.0%
FDR (Peptide Sequences)	0.0%
FDR (Protein Group)	0.0%
De Novo Only Spectra	15
# of identified Features	16
# of identified MS/MS scans	7

3. Experiment Control

Figure 5. Precursor mass error of peptide-spectrum matches (PSM) in filtered result. **(a)** Distribution of precursor mass error in ppm; **(b)** Scatterplot of precursor m/z versus precursor mass error in ppm.

(a)

(b)

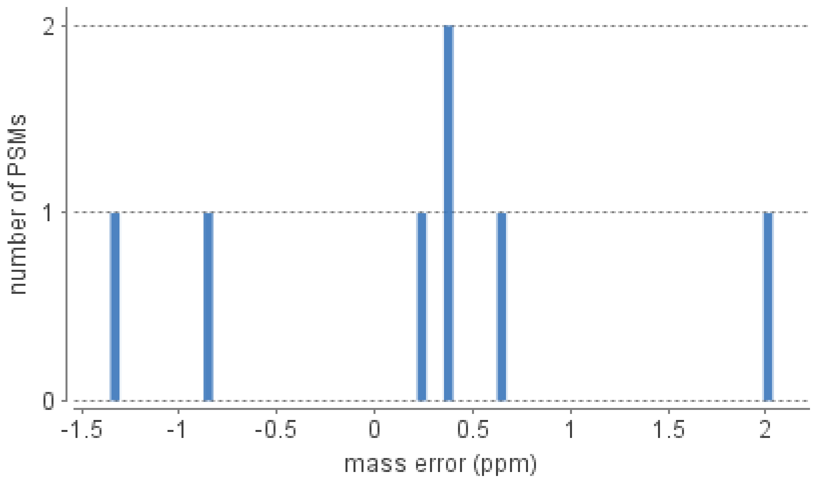

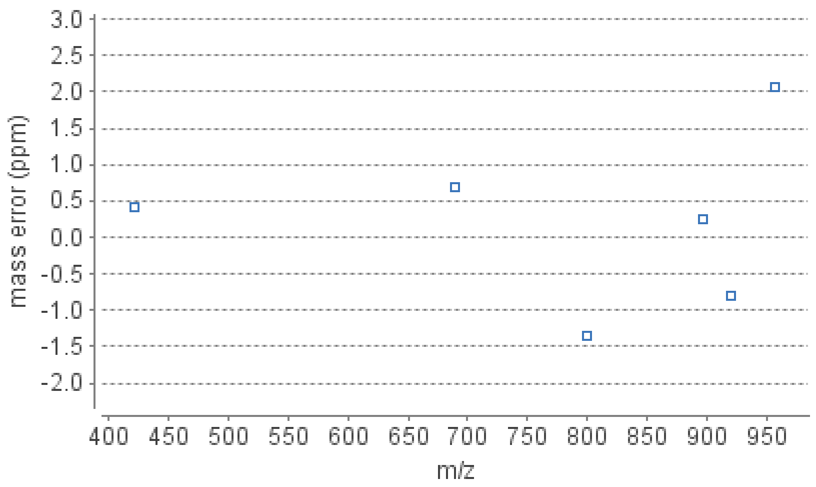

Table 5. Number of identified peptides in each sample by the number of missed cleavages

Missed Cleavages	0	1	2	3	4+
Digest 3. traka 18 kDa	6	0	0	0	0

4. Other Information

Table 6. Search parameters.

Search Engine Name: PEAKS
Parent Mass Error Tolerance: 10.0 ppm
Fragment Mass Error Tolerance: 0.5 Da
Precursor Mass Search Type: monoisotopic
Enzyme: Trypsin
Max Missed Cleavages: 2
Digest Mode: Unspecific
Fixed Modifications:

Table 7. Instrument parameters.

Fractions: OB4053.raw
Ion Source: ESI(nano-spray)
Fragmentation Mode: CID, CAD(y and b ions)
MS Scan Mode: FT-ICR/Orbitrap
MS/MS Scan Mode: Linear Ion Trap

Carbamidomethylation: 57.02
Variable Modifications:
Deamidation (NQ): 0.98
Oxidation (M): 15.99
Hydroxylation Pro: 15.99
Acetylation (K): 42.01
Acetylation (Protein N-term): 42.01
Acetylation (N-term): 42.01
Amidation: -0.98
Beta-methylthiolation: 45.99
and 305 more...
Max Variable PTM Per Peptide: 5
Database: Uniprot_Peanut-3818_Jul18
Taxon: All
Contaminant Database: contaminantsMQ_mar19
Searched Entry: 1723
FDR Estimation: Enabled
De novo score (ALC%) threshold: 15
Peptide hit threshold (-10logP): 30.0
Peaks run ID: 467
Merge Options: no merge
Precursor Options: corrected
Charge Options: no correction
Filter Charge: 2 - 8
Process: true
Associate chimera: yes

Protein List

Protein Accession Contains:
Protein Description Contains:
Peptide Sample Area >=
Protein Ptm Contains:

Protein Group	Protein ID	Accession	-10lgP	Coverage (%)	Coverage (%) Digest 3. traka 18 kDa	Area Digest 3. traka 18 kDa	#Peptides	#Unique	#Spec Digest 3. traka 18 kDa	PTM	Avg. Mass	Description
**3**	22696	tr|E9LFE7|E9LFE7_ARAHY	47.17	9	9	3.05E3	1	1	1	Y	15873	7S conarachin (Fragment) OS=Arachis hypogaea OX=3818 PE=2 SV=1
**3**	22695	tr|Q6PSU5|Q6PSU5_ARAHY	47.17	4	4	3.05E3	1	1	1	Y	33604	Conarachin (Fragment) OS=Arachis hypogaea OX=3818 PE=2 SV=1
**3**	21739	tr|Q6PSU6|Q6PSU6_ARAHY	47.17	4	4	3.05E3	1	1	1	Y	34133	Conarachin (Fragment) OS=Arachis hypogaea OX=3818 PE=2 SV=1
**3**	22400	tr|Q6PSU4|Q6PSU4_ARAHY	47.17	3	3	3.05E3	1	1	1	Y	48095	Conarachin (Fragment) OS=Arachis hypogaea OX=3818 PE=2 SV=1
**3**	20849	tr|Q6PSU3|Q6PSU3_ARAHY	47.17	2	2	3.05E3	1	1	1	Y	66575	Conarachin (Fragment) OS=Arachis hypogaea OX=3818 PE=4 SV=1
**3**	20850	P43237|ALL11_ARAHY	47.17	2	2	3.05E3	1	1	1	Y	70283	Allergen Ara h 1, clone P17 OS=Arachis hypogaea OX=3818 PE=1 SV=1
**3**	20851	tr|B3IXL2|B3IXL2_ARAHY	47.17	2	2	3.05E3	1	1	1	Y	70283	Main allergen Ara h1 OS=Arachis hypogaea OX=3818 PE=2 SV=1
**3**	20852	tr|E5G076|E5G076_ARAHY	47.17	2	2	3.05E3	1	1	1	Y	70788	Ara h 1 allergen OS=Arachis hypogaea OX=3818 GN=ara h 1 PE=4 SV=1
**3**	20853	tr|N1NG13|N1NG13_ARAHY	47.17	2	2	3.05E3	1	1	1	Y	71345	Seed storage protein Ara h1 OS=Arachis hypogaea OX=3818 GN=ARAX_AHF417E07-017 PE=4 SV=1
**3**	20854	P43238|ALL12_ARAHY	47.17	2	2	3.05E3	1	1	1	Y	71345	Allergen Ara h 1, clone P41B OS=Arachis hypogaea OX=3818 PE=1 SV=1
**total 10 proteins**												

tr|E9LFE7|E9LFE7_ARAHY

back to list

| **Protein Coverage** | **Supporting Peptides** |

Protein Coverage:

```
 1  WEEEEEDEEE EGSNREVRRY TARLKEGDVF IMPAAHPVAI NASSELHLLG FGINAENNHR IFLAGDKDNV IDQIEKQAKD
                                89
                                 d
81  LAFPGSGEQV EKLIKNQRES HFVSARPQSQ SQFPSSPEKE DQEEENQGGK GPLLSILKAF N
                                 d
```

d Deamidation (NQ) (+0.98)

Supporting Peptides:

Peptide	Uniq	-10lgP	Mass	Length	ppm	m/z	z	RT	Fraction	Scan	Source File	Area Digest 3. traka 18 kDa	#Feature	#Feature Digest 3. traka 18 kDa	Start	End	PTM
K.DLAFPGSGEQ(+.98)VEK.L	Y	47.17	1376.6459	13	0.7	689.3307	2	29.47	25	1456	OB4053.raw	3.05E3	1	1	80	92	Deamidation (NQ)
**total 1 peptides**																	

tr|Q6PSU5|Q6PSU5_ARAHY

back to list

| Protein Coverage | Supporting Peptides |

Protein Coverage:

d Deamidation (NQ) (+0.98)

```
  1  LEAAFNAEFN EIRRVLLEEN AGGEQEERGQ RRWSTRSSEN NEGVIVKVSK EHVEELTKHA KSVSKKGSEE EGDITNPINL
 81  REGEPDLSNN FGKLFEVKPD KKNPQLQDLD MMLTCVEIKE GALMLPHFNS KAMVIVVVNK GTGNLELVAV RKEQQQRGRR
161  EEEEDEDQEE EGSNREVRRY TARLKEGDVF IMPAAHPVAI NASSELHLLG FGINAENNHR IFLAGDKDNV IDQIEKQAKD
                 249
                  d
241  LAFPGSGEQV EKLIKNQRES HFVSARPQSQ SPSSPEKEDQ EEENQGGKGP LLSILKAFN
                  d
```

Supporting Peptides:

Peptide	Uniq	-10lgP	Mass	Length	ppm	m/z	z	RT	Fraction	Scan	Source File	Area Digest 3. traka 18 kDa	#Feature	#Feature Digest 3. traka 18 kDa	Start	End	PTM
K.DLAFPGSGEQ(+.98)VEK.L	Y	47.17	1376.6459	13	0.7	689.3307	2	29.47	25	1456	OB4053.raw	3.05E3	1	1	240	252	Deamidation (NQ)
**total 1 peptides**																	

tr|Q6PSU6|Q6PSU6_ARAHY

back to list

| Protein Coverage | Supporting Peptides |

Protein Coverage:

d Deamidation (NQ) (+0.98)

```
  1  GNTLEAAFNA EFNEIRRVLL EENAGGEQEE RGQRRRSTRS SDNEGVIVKV SKEHVQELTK HAKSVSKKGS EEEDITNPIN
 81  LRDGEPDLSN NFGRLFEVKP DKKNPQLQDL DMMLTCVEIK EGALMLPHFN SKAMVIVVVN KGTGNLELVA VRKEQQQRGR
161  REQEWEEEEE DEEEEGSNRE VRRYTARLKE GDVFIMPAAH PVAINASSEL HLLGFGINAE NNHRIFLAGD KDNVIDQIEK
                        253
                         d
241  QAKDLAFPGS GEQVEKLIKN QRESHFVSAR PQSQSPSSPE KEDQEEENQG GKGPLLSILK AFN
                         d
```

Supporting Peptides:

Peptide	Uniq	-10lgP	Mass	Length	ppm	m/z	z	RT	Fraction	Scan	Source File	Area Digest 3. traka 18 kDa	#Feature	#Feature Digest 3. traka 18 kDa	Start	End	PTM
K.DLAFPGSGEQ(+.98)VEK.L	Y	47.17	1376.6459	13	0.7	689.3307	2	29.47	25	1456	OB4053.raw	3.05E3	1	1	244	256	Deamidation (NQ)

Peptide	Uniq	-10lgP	Mass	Length	ppm	m/z	z	RT	Fraction	Scan	Source File	Area Digest 3. traka 18 kDa	#Feature	#Feature Digest 3. traka 18 kDa	Start	End	PTM
**total 1 peptides**																	

tr|Q6PSU4|Q6PSU4_ARAHY
back to list

| Protein Coverage | Supporting Peptides |

Protein Coverage:

```
  1  GFDQRSRQFQ NLQNHRIVQI EAKPNTLVLP KHADADNILV IQQGQATVTV ANGNNRKSFN LDEGHALRIP SGFISYILNR
 81  HDNQNLRVAK ISMPVNTPGQ FEDFFPASSR DQSSYLQGFS RNTLEAAFNA EFNEIRRVLL EENAGGEQEE RGQRRWSTRS
161  SENNEGVIVK VSKEHVEELT KHAKSVSKKG SEEGDITNPI NLREGEPDLS NNFGKLFEVK PDKKNPQLQD LDMMLTCVEI
241  KEGALMLPHF NSKAMVIVVV NKGTGNLELV AVRKEQQQRG RREEEEDEDE EEEGSNREVR RYTARLKEGD VFIMPAAHPV
321  AINASSELHL LGFGINAENN HRIFLAGDKD NVIDQIEKQA KDLAFPGSGE QVEKLIKNQK ESHFVSARPQ SQSQSPSSPE
401  KESPEKEDQE EENQGGKGPL LSILKAFN
```

371 d

d Deamidation (NQ) (+0.98)

Supporting Peptides:

Peptide	Uniq	-10lgP	Mass	Length	ppm	m/z	z	RT	Fraction	Scan	Source File	Area Digest 3. traka 18 kDa	#Feature	#Feature Digest 3. traka 18 kDa	Start	End	PTM
K.DLAFPGSGEQ(+.98)VEK.L	Y	47.17	1376.6459	13	0.7	689.3307	2	29.47	25	1456	OB4053.raw	3.05E3	1	1	362	374	Deamidation (NQ)
**total 1 peptides**																	

tr|Q6PSU3|Q6PSU3_ARAHY
back to list

| Protein Coverage | Supporting Peptides |

Protein Coverage:

1 MRGRVSPLML LLGILVLASV SATQAKSPYR KTENPCAQRC LQSCQQEPDD LKQKACESRC TKLEYDPRCV YDTGATNQRH

d Deamidation (NQ) (+0.98)

81 PPGERTRGRQ PGDYDDDRRQ PRREEGGRWG PAEPRERERE EDWRQPREDW RRPSHQQPRK IRPEGREGEQ EWGTPGSEVR

161 EETSRNNPFY FPSRRFSTRY GNQNGRIRVL QRFDQRSKQF QNLQNHRIVQ IEARPNTLVL PKHADADNIL VIQQGQATVT

241 VANGNNRKSF NLDEGHALRI PSGFISYILN RHDNQNLRVA KISMPVNTPG QFEDFFPASS RDQSSYLQGF SRNTLEAAFN

321 AEFNEIRRVL LEENAGGEQE ERGQRRRSTR SSDNEGVIVK VSKEHVQELT KHAKSVSKKG SEEEDITNPI NLRDGEPDLS

401 NNFGRLFEVK PDKKNPQLQD LDMMLTCVEI KEGALMLPHF NSKAMVIVVV NKGTGNLELV AVRKEQQQRG RREQEWEEEE

481 EDEEEEGSNR EVRRYTARLK EGDVFIMPAA HPVAINASSE LHLLGFGINA ENNHRIFLAG DKDNVIDQIE KQAK**DLAFPG**

564
d

561 **SGEQVEK**LIK NQRESHFVSA

d

Supporting Peptides:

Peptide	Uniq	-10lgP	Mass	Length	ppm	m/z	z	RT	Fraction	Scan	Source File	Area Digest 3. traka 18 kDa	#Feature	#Feature Digest 3. traka 18 kDa	Start	End	PTM
K.DLAFPGSGEQ(+.98)VEK.L	Y	47.17	1376.6459	13	0.7	689.3307	2	29.47	25	1456	OB4053.raw	3.05E3	1	1	555	567	Deamidation (NQ)
**total 1 peptides**																	

P43237|ALL11_ARAHY

back to list

| **Protein Coverage** | **Supporting Peptides** |

Protein Coverage:

1	MRGRVSPLML	LLGILVLASV	SATQAKSPYR	KTENPCAQRC	LQSCQQEPDD	LKQKACESRC	TKLEYDPRCV	YDTGATNQRH	d Deamidation (NQ) (+0.98)
81	PPGERTRGRQ	PGDYDDDRRQ	PRREEGGRWG	PAEPRERERE	EDWRQPREDW	RRPSHQQPRK	IRPEGREGEQ	EWGTPGSEVR	
161	EETSRNNPFY	FPSRRFSTRY	GNQNGRIRVL	QRFDQRSKQF	QNLQNHRIVQ	IEARPNTLVL	PKHADADNIL	VIQQGQATVT	
241	VANGNNRKSF	NLDEGHALRI	PSGFISYILN	RHDNQNLRVA	KISMPVNTPG	QFEDFFPASS	RDQSSYLQGF	SRNTLEAAFN	
321	AEFNEIRRVL	LEENAGGEQE	ERGQRRRSTR	SSDNEGVIVK	VSKEHVQELT	KHAKSVSKKG	SEEEDITNPI	NLRDGEPDLS	
401	NNFGRLFEVK	PDKKNPQLQD	LDMMLTCVEI	KEGALMLPHF	NSKAMVIVVV	NKGTGNLELV	AVRKEQQQRG	RREQEWEEEE	
481	EDEEEEGSNR	EVRRYTARLK	EGDVFIMPAA	HPVAINASSE	LHLLGFGINA	ENNHRIFLAG	DKDNVIDQIE	KQAK**DLAFPG**	
561	**SGEQVEK**LIK	NQRESHFVSA	RPQSQPSSSP	EKEDQEEENQ	GGKGPLLSIL	KAFN			

564 d (SGEQVEK)

Supporting Peptides:

Peptide	Uniq	-10lgP	Mass	Length	ppm	m/z	z	RT	Fraction	Scan	Source File	Area Digest 3. traka 18 kDa	#Feature	#Feature Digest 3. traka 18 kDa	Start	End	PTM
K.DLAFPGSGEQ(+.98)VEK.L	Y	47.17	1376.6459	13	0.7	689.3307	2	29.47	25	1456	OB4053.raw	3.05E3	1	1	555	567	Deamidation (NQ)
**total 1 peptides**																	

tr|B3IXL2|B3IXL2_ARAHY

[back to list]

| [Protein Coverage] | [Supporting Peptides] |

Protein Coverage:

d Deamidation (NQ) (+0.98)

1 MRGRVSPLML LLGILVLASV SATQAKSPYR KTENPCAQRC LQSCQQEPDD LKQKACESRC TKLEYDPRCV YDTGATNQRH
81 PPGERTRGRQ PGDYDDDRRQ PRREEGGRWG PAEPRERERE EDWRQPREDW RRPSHQQPRK IRPEGREGEQ EWGTPGSEVR
161 EETSRNNPFY FPSRRFSTRY GNQNGRIRVL QRFDQRSKQF QNLQNHRIVQ IEARPNTLVL PKHADADNIL VIQQGQATVT
241 VANGNNRKSF NLDEGHALRI PSGFISYILN RHDNQNLRVA KISMPVNTPG QFEDFFPASS RDQSSYLQGF SRNTLEAAFN
321 AEFNEIRRVL LEENAGGEQE ERGQRRRSTR SSDNEGVIVK VSKEHVQELT KHAKSVSKKG SEEEDITNPI NLRDGEPDLS
401 NNFGRLFEVK PDKKNPQLQD LDMMLTCVEI KEGALMLPHF NSKAMVIVVV NKGTGNLELV AVRKEQQQRG RREQEWEEEE
481 EDEEEEGSNR EVRRYTARLK EGDVFIMPAA HPVAINASSE LHLLGFGINA ENNHRIFLAG DKDNVIDQIE KQAK**DLAFPG**

564
d

561 **SGEQVEK**LIK NQRESHFVSA RPQSQSPSSP EKEDQEEENQ GGKGPLLSIL KAFN

d

Supporting Peptides:

Peptide	Uniq	-10lgP	Mass	Length	ppm	m/z	z	RT	Fraction	Scan	Source File	Area Digest 3. traka 18 kDa	#Feature	#Feature Digest 3. traka 18 kDa	Start	End	PTM
K.DLAFPGSGEQ(+.98)VEK.L	Y	47.17	1376.6459	13	0.7	689.3307	2	29.47	25	1456	OB4053.raw	3.05E3	1	1	555	567	Deamidation (NQ)
**total 1 peptides**																	

tr|E5G076|E5G076_ARAHY

back to list

| Protein Coverage | Supporting Peptides |

Protein Coverage:

										d Deamidation (NQ) (+0.98)
1	MRGRVSPLML	LLGILVLASV	SATHAKSSPY	QKKTENPCAQ	RCLQSCQQEP	DDLKQKACES	RCTKLEYDPR	CVYDPRGHTG		
81	TTNQRSPPGE	RTRGRQPGDY	DDDRRQPRRE	EGGRWGPAGP	REREREEDWR	QPREDWRRPS	HQQPRKIRPE	GREGEQEWGT		
161	PGSHVREETS	RNNPFYFPSR	RFSTRYGNQN	GRIRVLQRFD	QRSRQFQNLQ	NHRIVQIEAK	PNTLVLPKHA	DADNILVIQQ		
241	GQATVTVANG	NNRRALILTR	AMHSESHPFH	FLHLDDMTPE	LRVAKSHAVN	TPGQFEDFFP	ASSRDQSSYL	QGFSRNTLEA		
321	AFNAEFNEIR	RVLLEENAGG	EQEERGQRRW	STRSSENNEG	VIVEVSKEHV	EELTKHAKSV	SKKGSEEEGD	ITNPINLREG		
401	EPDLSDNFGR	LFEVKPDKKN	PQLQDLDMML	TCVEIKEGAL	MLPHFNSKAM	VIVVINKGTG	NLELVAVRKE	QQQRGRREQE		
481	WEEEEEDEEE	EGSNREVRRY	TARLKEGDVF	IMPAAHPVAI	NASSELHLLG	FGINAENNHR	IFLAGDKDNV	VDQIEKQAKD		
561	LAFPGSGEQV	EKLIKNQRES	HFVSARPQSQ	SPSSPEKEDQ	EEENQGGKGP	LLSILKAFN				

569 d

Supporting Peptides:

Peptide	Uniq	-10lgP	Mass	Length	ppm	m/z	z	RT	Fraction	Scan	Source File	Area Digest 3. traka 18 kDa	#Feature	#Feature Digest 3. traka 18 kDa	Start	End	PTM
K.DLAFPGSGEQ(+.98)VEK.L	Y	47.17	1376.6459	13	0.7	689.3307	2	29.47	25	1456	OB4053.raw	3.05E3	1	1	560	572	Deamidation (NQ)
total 1 peptides																	

tr|N1NG13|N1NG13_ARAHY
back to list

| Protein Coverage | Supporting Peptides |

Protein Coverage:

d Deamidation (NQ) (+0.98)

```
  1 MRGRVSPLML LLGILVLASV SATHAKSSPY QKKTENPCAQ RCLQSCQQEP DDLKQKACES RCTKLEYDPR CVYDPRGHTG
 81 TTNQRSPPGE RTRGRQPGDY DDDRRQPRRE EGGRWGPAGP REREREEDWR QPREDWRRPS HQQPRKIRPE GREGEQEWGT
161 PGSHVREETS RNNPFYFPSR RFSTRYGNQN GRIRVLQRFD QRSRQFQNLQ NHRIVQIEAK PNTLVLPKHA DADNILVIQQ
241 GQATVTVANG NNRKSFNLDE GHALRIPSGF ISYILNRHDN QNLRVAKISM PVNTPGQFED FFPASSRDQS SYLQGFSRNT
321 LEAAFNAEFN EIRRVLLEEN AGGEQEERGQ RRWSTRSSEN NEGVIVKVSK EHVEELTKHA KSVSKKGSEE EGDITNPINL
401 REGEPDLSNN FGKLFEVKPD KKNPQLQDLD MMLTCVEIKE GALMLPHFNS KAMVIVVVNK GTGNLELVAV RKEQQQRGRR
481 EEEEDEDEEE EGSNREVRRY TARLKEGDVF IMPAAHPVAI NASSELHLLG FGINAENNHR IFLAGDKDNV IDQIEKQAKD

              569
               d
561 LAFPGSGEQV EKLIKNQKES HFVSARPQSQ SQSPSSPEKE SPEKEDQEEE NQGGKGPLLS ILKAFN
               d
```

Supporting Peptides:

Peptide	Uniq	-10lgP	Mass	Length	ppm	m/z	z	RT	Fraction	Scan	Source File	Area Digest 3. traka 18 kDa	#Feature	#Feature Digest 3. traka 18 kDa	Start	End	PTM
K.DLAFPGSGEQ(+.98)VEK.L	Y	47.17	1376.6459	13	0.7	689.3307	2	29.47	25	1456	OB4053.raw	3.05E3	1	1	560	572	Deamidation (NQ)
**total 1 peptides**																	

P43238|ALL12_ARAHY

back to list

| **Protein Coverage** | **Supporting Peptides** |

Protein Coverage:

d Deamidation (NQ) (+0.98)

1 MRGRVSPLML LLGILVLASV SATHAKSSPY QKKTENPCAQ RCLQSCQQEP DDLKQKACES RCTKLEYDPR CVYDPRGHTG
81 TTNQRSPPGE RTRGRQPGDY DDDRRQPRRE EGGRWGPAGP REREREEDWR QPREDWRRPS HQQPRKIRPE GREGEQEWGT
161 PGSHVREETS RNNPFYFPSR RFSTRYGNQN GRIRVLQRFD QRSRQFQNLQ NHRIVQIEAK PNTLVLPKHA DADNILVIQQ
241 GQATVTVANG NNRKSFNLDE GHALRIPSGF ISYILNRHDN QNLRVAKISM PVNTPGQFED FFPASSRDQS SYLQGFSRNT
321 LEAAFNAEFN EIRRVLLEEN AGGEQEERGQ RRWSTRSSEN NEGVIVKVSK EHVEELTKHA KSVSKKGSEE EGDITNPINL
401 REGEPDLSNN FGKLFEVKPD KKNPQLQDLD MMLTCVEIKE GALMLPHFNS KAMVIVVVNK GTGNLELVAV RKEQQQRGRR
481 EEEEDEDEEE EGSNREVRRY TARLKEGDVF IMPAAHPVAI NASSELHLLG FGINAENNHR IFLAGDKDNV IDQIEKQAK**D**

569
d
561 **LAFPGSGEQV EK**LIKNQKES HFVSARPQSQ SQSPSSPEKE SPEKEDQEEE NQGGKGPLLS ILKAFN
d

Supporting Peptides:

Peptide	Uniq	-10lgP	Mass	Length	ppm	m/z	z	RT	Fraction	Scan	Source File	Area Digest 3. traka 18 kDa	#Feature	#Feature Digest 3. traka 18 kDa	Start	End	PTM
K.DLAFPGSGEQ(+.98)VEK.L	Y	47.17	1376.6459	13	0.7	689.3307	2	29.47	25	1456	OB4053.raw	3.05E3	1	1	560	572	Deamidation (NQ)
**total 1 peptides**																	

Peptide List

1. Notes Gastric Control Raw peanut Band #4 21-22 kDa

2. Result Statistics

Figure 1. False discovery rate (FDR) curve. X axis is the number of peptide-spectrum matches (PSM) being kept. Y axis is the corresponding FDR.

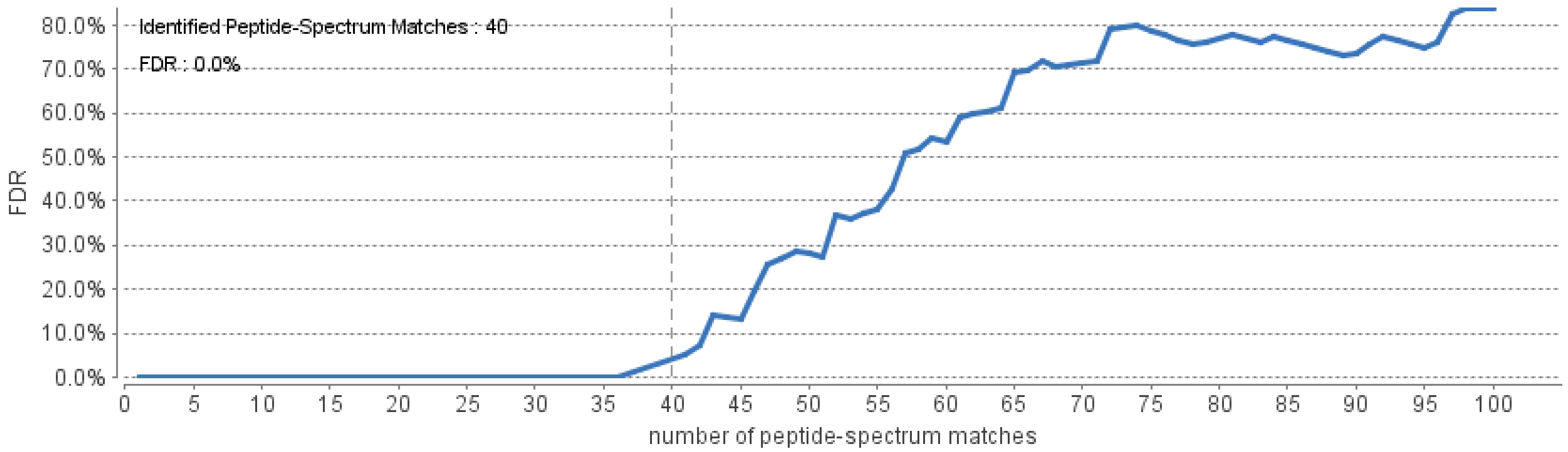

Figure 2. PSM score distribution. **(a)** Distribution of PEAKS peptide score; **(b)** Scatterplot of PEAKS peptide score versus precursor mass error.

(a)

(b)

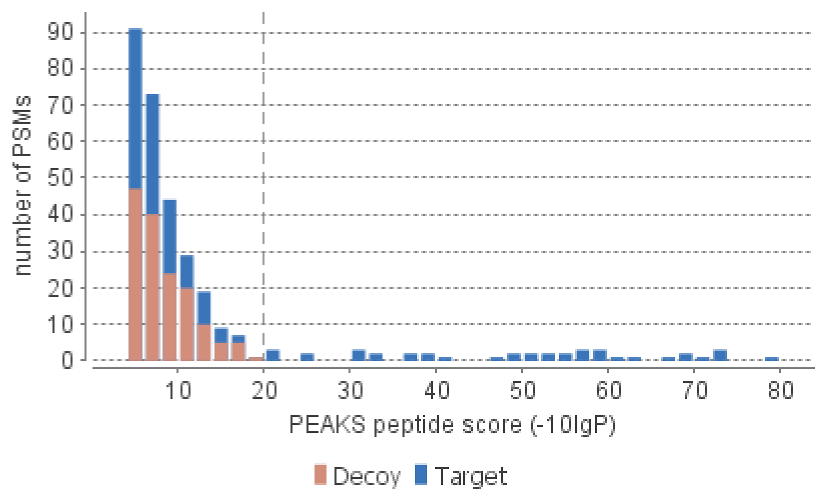

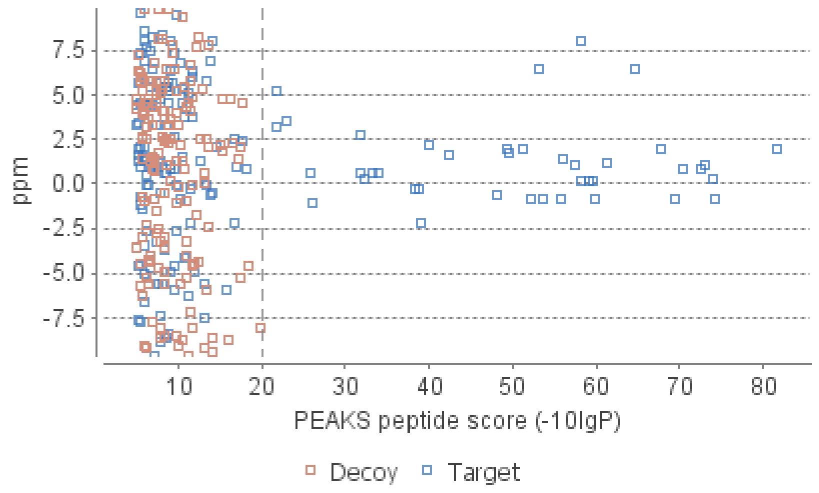

Figure 3. Distribution of peptide feature detection. **(a)** Feature m/z distribution; **(b)** Feature RT distribution

(a)

(b)

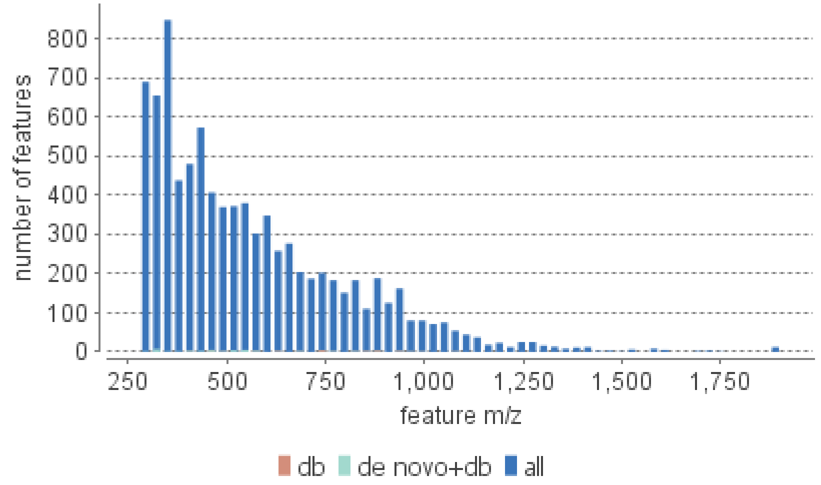

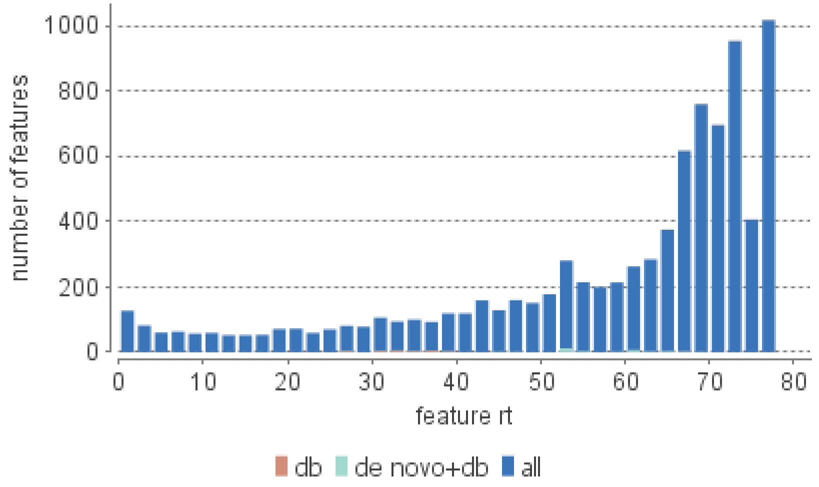

Figure 4. Distribution of identified peptide features. **(a)** Feature abundance distribution **(b)** *De novo* sequencing validation.

(a)

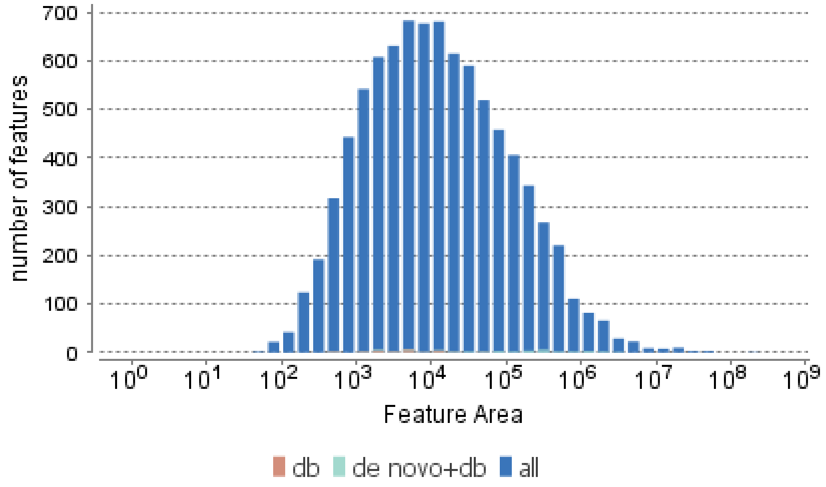

(b)

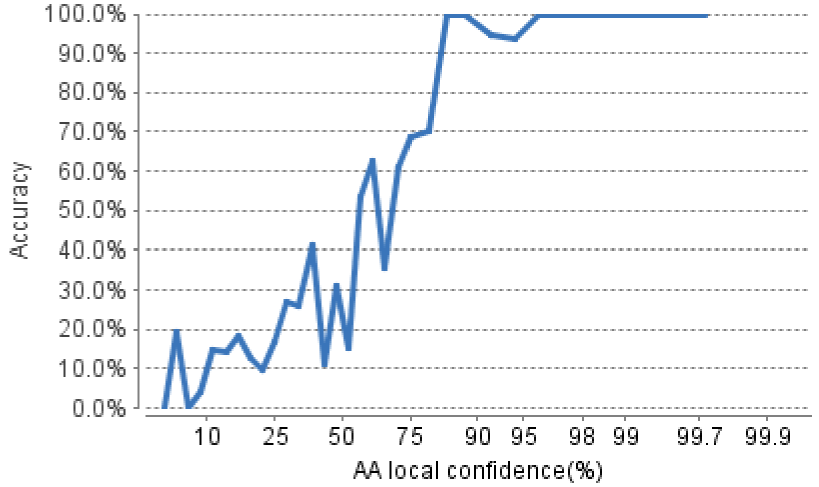

Table 1. Statistics of data.

# of MS scans	3177
# of MS/MS scans	1237
# of Features	8728
# of Chimera scans	87

Table 2. Result filtration parameters.

Peptide -10lgP	≥20
PTM Ascore	≥20
Protein -10lgP	≥20
Proteins unique peptides	≥1
De novo ALC Score	≥50%

Table 4. PTM profile.

Name	ΔMass	Position	#PSM	-10lgP	Abundance	AScore
Deamidation	.98	NQ	5	58.14		0.00
Dimethylation(KR)	28.03	KR	3	55.99	2.25E3	1000.00
Carbamidomethyl	57.02	C	1	49.50	1.5E3	1000.00
Amidation	-.98	C-term	1	21.71	2.85E3	1000.00

Table 3. Statistics of filtered result.

Peptide-Spectrum Matches	40
Peptide sequences	27
Protein groups	5
Proteins	19
Proteins (#Unique Peptides)	1 (>2); 3 (=2); 15 (=1);
FDR (Peptide-Spectrum Matches)	0.0%
FDR (Peptide Sequences)	0.0%
FDR (Protein Group)	0.0%
De Novo Only Spectra	65
# of identified Features	56
# of identified MS/MS scans	40

3. Experiment Control

Figure 5. Precursor mass error of peptide-spectrum matches (PSM) in filtered result. **(a)** Distribution of precursor mass error in ppm; **(b)** Scatterplot of precursor m/z versus precursor mass error in ppm.

(a)

(b)

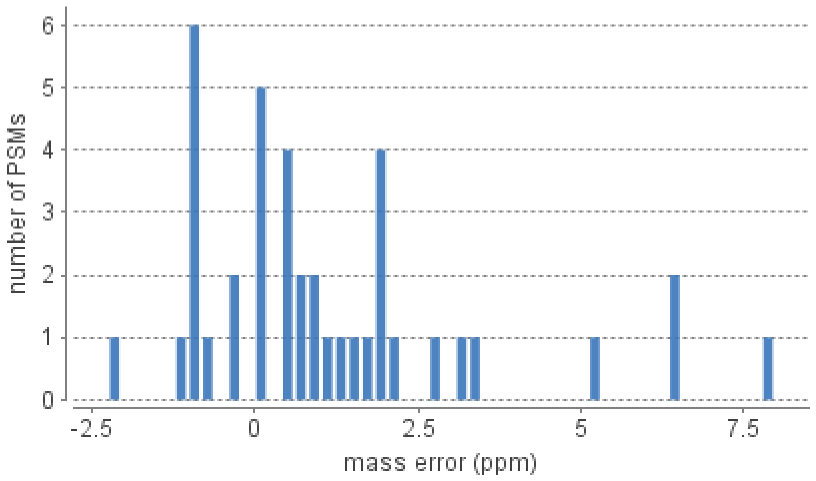

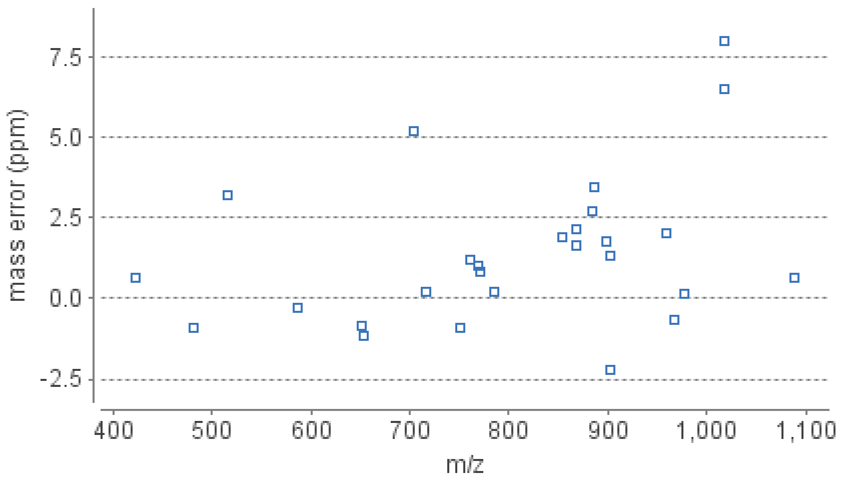

Table 5. Number of identified peptides in each sample by the number of missed cleavages

Missed Cleavages	0	1	2	3	4+
Kontrola 4. traka 20 kDa	20	7	0	0	0

4. Other Information

Table 6. Search parameters.

Search Engine Name: PEAKS
Parent Mass Error Tolerance: 10.0 ppm
Fragment Mass Error Tolerance: 0.5 Da
Precursor Mass Search Type: monoisotopic
Enzyme: Trypsin
Max Missed Cleavages: 2
Digest Mode: Unspecific

Table 7. Instrument parameters.

Fractions: OB4033.raw
Ion Source: ESI(nano-spray)
Fragmentation Mode: CID, CAD(y and b ions)
MS Scan Mode: FT-ICR/Orbitrap
MS/MS Scan Mode: Linear Ion Trap

Fixed Modifications:
  Carbamidomethylation: 57.02
Variable Modifications:
  Deamidation (NQ): 0.98
  Oxidation (M): 15.99
  Hydroxylation Pro: 15.99
  Acetylation (K): 42.01
  Acetylation (Protein N-term): 42.01
  Acetylation (N-term): 42.01
  Amidation: -0.98
  Beta-methylthiolation: 45.99
  and 305 more...
Max Variable PTM Per Peptide: 5
Database: Uniprot_Peanut-3818_Jul18
Taxon: All
Contaminant Database: contaminantsMQ_mar19
Searched Entry: 1723
FDR Estimation: Enabled
De novo score (ALC%) threshold: 15
Peptide hit threshold (-10logP): 30.0
Peaks run ID: 431
Merge Options: no merge
Precursor Options: corrected
Charge Options: no correction
Filter Charge: 2 - 8
Process: true
Associate chimera: yes

Protein List

Protein Accession Contains:
Protein Description Contains:
Peptide Sample Area >=
Protein Ptm Contains:

Protein Group	Protein ID	Accession	-10lgP	Coverage (%)	Coverage (%) Kontrola 4. traka 20 kDa	Area Kontrola 4. traka 20 kDa	#Peptides	#Unique	#Spec Kontrola 4. traka 20 kDa	PTM	Avg. Mass	Description
**4**	21037	tr|E9LFE8|E9LFE8_ARAHY	94.35	15	15	5.44E3	2	2	4	Y	28290	11S arachin (Fragment) OS=Arachis hypogaea OX=3818 PE=2 SV=1
**7**	21031	tr|Q9M5D3|Q9M5D3_ARAHY	84.68	5	5	7.73E3	3	3	3	Y	97616	Lipoxygenase OS=Arachis hypogaea OX=3818 PE=2 SV=1
**7**	21032	tr|Q4JME6|Q4JME6_ARAHY	77.44	4	4	4.88E3	2	2	2	N	97476	Lipoxygenase OS=Arachis hypogaea OX=3818 GN=Pnlox3 PE=2 SV=1
**7**	21033	tr|Q4JME7|Q4JME7_ARAHY	77.44	4	4	4.88E3	2	2	2	N	97596	Lipoxygenase OS=Arachis hypogaea OX=3818 GN=Pnlox2 PE=2 SV=1
**9**	20574	tr|A1DZF0|A1DZF0_ARAHY	72.50	2	2	6.09E3	1	1	2	N	60375	Arachin 6 OS=Arachis hypogaea OX=3818 PE =2 SV=1
**9**	20576	tr|Q9FZ11|Q9FZ11_ARAHY	72.50	2	2	6.09E3	1	1	2	N	60449	Gly1 OS=Arachis hypogaea OX=3818 GN=Gly 1 PE=2 SV=1
**9**	20578	tr|B5TYU1|B5TYU1_ARAHY	72.50	2	2	6.09E3	1	1	2	N	60624	Arachin Arah3 isoform OS=Arachis hypogaea OX=3818 PE=1 SV=1
**9**	20577	tr|Q9SQH7|Q9SQH7_ARAHY	72.50	2	2	6.09E3	1	1	2	N	61011	Glycinin OS=Arachis hypogaea OX=3818 GN= Arah4 PE=2 SV=1
**9**	20579	tr|Q5I6T2|Q5I6T2_ARAHY	72.50	2	2	6.09E3	1	1	2	N	60736	Arachin Ahy-4 OS=Arachis hypogaea OX=381 8 PE=2 SV=1
**9**	20580	tr|Q647H4|Q647H4_ARAHY	72.50	2	2	6.09E3	1	1	2	N	61506	Arachin Ahy-1 OS=Arachis hypogaea OX=381 8 PE=2 SV=1
**9**	20591	tr|Q6T2T4|Q6T2T4_ARAHY	72.50	2	2	6.09E3	1	1	2	N	61499	Storage protein OS=Arachis hypogaea OX=38 18 PE=2 SV=1
**9**	20581	tr|Q647H3|Q647H3_ARAHY	72.50	2	2	6.09E3	1	1	2	N	61532	Arachin Ahy-2 OS=Arachis hypogaea OX=381 8 PE=2 SV=1
**9**	20582	tr|Q8LKN1|Q8LKN1_ARAHY	72.50	2	2	6.09E3	1	1	2	N	61738	Allergen Arah3/Arah4 OS=Arachis hypogaea O X=3818 PE=3 SV=1
**11**	21034	tr|Q38711|Q38711_ARAHY	53.52	5	5	1.59E3	1	1	1	N	29134	Galactose-binding lectin (Fragment) OS=Arac his hypogaea OX=3818 GN=lec PE=2 SV=1
**11**	21036	tr|A0A089ZXL7|A0A089ZXL7_ARAHY	53.52	5	5	1.59E3	1	1	1	N	29407	Peanut agglutinin variant OS=Arachis hypogae a OX=3818 PE=2 SV=1
**11**	21035	P02872|LECG_ARAHY	53.52	5	5	1.59E3	1	1	1	N	29325	Galactose-binding lectin OS=Arachis hypogaea OX=3818 PE=1 SV=3
**10**	20593	tr|Q6IWG5|Q6IWG5_ARAHY	31.78	5	5	1.33E3	1	1	2	Y	58061	Glycinin (Fragment) OS=Arachis hypogaea OX =3818 PE=2 SV=1
**10**	20595	tr|E5G077|E5G077_ARAHY	31.78	5	5	1.33E3	1	1	2	Y	58305	Ara h 3 allergen OS=Arachis hypogaea OX=38 18 GN=ara h 3 PE=3 SV=1

Protein Group	Protein ID	Accession	-10lgP	Coverage (%)	Coverage (%) Kontrola 4. traka 20 kDa	Area Kontrola 4. traka 20 kDa	#Peptides	#Unique	#Spec Kontrola 4. traka 20 kDa	PTM	Avg. Mass	Description
**10**	20594	tr|Q0GM57|Q0GM57_ARAHY	31.78	5	5	1.33E3	1	1	2	Y	58263	Iso-Ara h3 OS=Arachis hypogaea OX=3818 PE=2 SV=1
**total 19 proteins**												

tr|E9LFE8|E9LFE8_ARAHY
back to list

| Protein Coverage | Supporting Peptides |

Protein Coverage:

```
  1  KSRKFFLGGK PQEEKGEEGN MFSGLELKTV AESLGIDMGI AGKVQGVDDP RGSIIIVEDE LETLSPAVEE SGNGNGLDET
 81  LCTLRLVHQL AESTDADKYN PRAGFLTALN TPNLPVLQYV QLGADRGVFY KNAVMAPHYN LNCHAVIYGT EGRGWIEVVG
161  ENGRKVYEGE VREGQILIVP QQFMVAKKAA EGSDEGFGWI AVKTSDNPMI SPLAGKLSLI RAMPLPVLMN SFRLTAEEAI
241  NLKKRGELTF FSPDPAHTQI
```

d Dimethylation(KR) (+28.03)

Supporting Peptides:

Peptide	Uniq	-10lgP	Mass	Length	ppm	m/z	z	RT	Fraction	Scan	Source File	Area Kontrola 4. traka 20 kDa	#Feature	#Feature Kontrola 4. traka 20 kDa	Start	End	PTM
K.AAEGSDEGFGWIAVK.T	Y	73.13	1535.7256	15	1.0	768.8708	2	32.35	5	1666	OB4033.raw	5E3	1	1	189	203	
R.AGFLTALN(+.98)TPNLPVLQYVQLGADR(+28.03).G	Y	42.45	2599.4009	24	1.6	867.4756	3	38.88	5	2038	OB4033.raw	0	0	0	103	126	Dimethylation(KR)
R.AGFLTALNTPN(+.98)LPVLQYVQLGADR(+28.03).G	Y	40.02	2599.4009	24	2.1	867.4761	3	38.63	5	2023	OB4033.raw	4.42E2	1	1	103	126	Dimethylation(KR)
**total 3 peptides**																	

tr|Q9M5D3|Q9M5D3_ARAHY
back to list

| Protein Coverage | Supporting Peptides |

Protein Coverage:

1 MVSVRRSAGI RGRAGILNRG HKIK**WTVVLM RK**NVLDVDTF TDVAATANIG GLIGTGIN**VI GSTVDALTAF LGR**SVSLQLI

Amidation (-0.98)

81 SSTQSDVNGN GKVVKDTFLE GIIASLPTLE LENLAFSIHF EWDDSMGIPG AFYIKNYMQV EFFLKTLTLE DVPNQGTIHF

161 VCNSWVYNSK LYKSPR**IFFS NKPYLPSETP APLVK**YREED LKILRGDGKG ERQEHERIYD YDVYNDLGNP DRNENHARPI

241 LGGSTTFLTS QGRTGRYPAR NDPNSEKPGD VYVPRDENFG HLKSSDFLAN SIKFLTRYVL PAFESVFDLN LTPNEFDSFQ

321 DVRDLYEGGI RLPTEVISTK RRLLVIKELF RTDGEQVLKF PPPRFLQVNE ENSAWMTDEE FVIINELLVS HPCMDPTIYG

401 DQNSKIPAEV LDLEGCSLEE AINGRRLFIL DYHDVFMPYV RRINETHAKA YATRTILFLK EDGTLNPVAI ELSLPHPDGD

481 KSGAISDVIL PAKGRCRKHN LATSQSLCHI NDSCYHHLMS HWLNTHAVIE PFVIATNRQL SVIHPIYKLL SPHYRDTMNI

561 NALARQNLIN SDGIIERTFL PSKFSVEMSS AVYKNWVFTD QALPADLIKR GMAVEDSSSP YGIRLVIEDY PYAVDGLEIW

641 FAIKEWVQDY VSLYYPTDND LKKDPELQNW WKEAVEVGHG DLKDKPWWPK MQTVEELVES CTTIIWTASA LHAAVNFGQY

721 PYGGLILNRP TLSRRLLPEQ GTAEYEEMVK SHQKAYLRTI TPKLETLIDL TTIEILSKHA SDEVYLGERD NPHWTFDSRA

801 LEAFQRFGNK LSEIEEKLTE KNKDGRLSNR IGPVELPYTL LHPTSNEGLT FRGVPNSISI

Supporting Peptides:

Peptide	Uniq	-10lgP	Mass	Length	ppm	m/z	z	RT	Fraction	Scan	Source File	Area Kontrola 4. traka 20 kDa	#Feature	#Feature Kontrola 4. traka 20 kDa	Start	End	PTM
N.VIGSTVDALTAFLGR.S	Y	61.33	1518.8406	15	1.2	760.4285	2	35.89	5	1866	OB4033.raw	4.42E3	1	1	59	73	
R.IFFSNKPYLPSETPAPLVK.Y	Y	32.22	2147.1665	19	0.2	716.7296	3	31.56	5	1622	OB4033.raw	4.54E2	1	1	177	195	
K.WTVVLMRK(-.98).N	Y	21.71	1030.6110	8	3.2	516.3144	2	36.30	5	1889	OB4033.raw	2.85E3	1	1	25	32	Amidation
**total 3 peptides**																	

tr|Q4JME6|Q4JME6_ARAHY

back to list

| Protein Coverage | Supporting Peptides |

Protein Coverage:

1 MFSGVAGILN RGHKIKGTVV LMRKNVLDVD TFTDVAATAN IGGLIGTGIN **VIGSTVDALT AFLGR**SVSLQ LISSTQSDEN

81 GNGKVVKDTF LEGIIASLPT LGAGESAFSI HFEWDDSMGI PGAFYIKNYM QVEFFLKTLT LEDVPNQGTI HFVCNSWVYN

161 SKLYKSPR**IF FSNKPYLPSE TPAPLVK**YRE EDLKILRGDG KGERQEHERI YDYDVYNDLG NPDRNENHAR PILGGSTTFP

241 YPRRGRTGRY PARNDPNSEK PGDVYVPRDE NFGHLKSSDF LANSIKFLTR YVLPAFESVF DLNLTPNEFD SFQDVRDLYE

321 GGIRLPTEVI STISPLPVIK ELFRTDGEQV LKFPPPHIIQ VNKSAWMTDE EFAREMIAGV NPCMIRSLQE FPPKSNLDPT

401 IYGDQNSKIP AEVLDLEGCS LEEAINGRRL FILDYHDVFM PYVRRINETH AKAYATRTIL FLKEDGTLKP VAIELSLPHP

481 DGDKSGAISE VILPAKEGVE STIWLLAKAY VIVNDSCYHQ LMSHWLNTHA VIEPFVIATN RQLSVIHPIY KLLSPHYLDT

561 MNINALARQN LINSDGIIER TFLPSKFSVE MSSAVYKNWV FTDQALPADL IKRGMAVEDS SSPYGIRLVI EDYPYAVDGL

641 EIWFAIKEWV QDYVSLYYPT DNDLKKDPEL QNWWKEAVEV GHGDLKDKPW WPKMQTVEEL VESCTTIIWT ASALHAAVNF

721 GQYPYGGLIL NRPTLSRRLL PEQGTAEYEE MVKSHQKAYL RTITPKLETL IDLTTIEILS KHASDEVYLG ERDNPHWTFD

801 SRALEAFQRF GNKLSEIEEK LTEKNKDGRL SNRIGPVELP YTLLHPTSNE GLTFRGVPNS ISI

Supporting Peptides:

Peptide	Uniq	-10lgP	Mass	Length	ppm	m/z	z	RT	Fraction	Scan	Source File	Area Kontrola 4. traka 20 kDa	#Feature	#Feature Kontrola 4. traka 20 kDa	Start	End	PTM
N.VIGSTVDALTAFLGR.S	Y	61.33	1518.8406	15	1.2	760.4285	2	35.89	5	1866	OB4033.raw	4.42E3	1	1	51	65	
R.IFFSNKPYLPSETPAPLVK.Y	Y	32.22	2147.1665	19	0.2	716.7296	3	31.56	5	1622	OB4033.raw	4.54E2	1	1	169	187	
**total 2 peptides**																	

tr|Q4JME7|Q4JME7_ARAHY

back to list

| Protein Coverage | Supporting Peptides |

Protein Coverage:

1 MFSGVTGMLN RGHKIKGTVV LMRKNVLDVD TFTDVVATAN IGGLIGTGIN **VIGSTVDALT AFLGR**SVSLQ LISSTQSDEN

81 GNGKVVKDTF LEGIIASLPT LGAGESAFSI HFEWDDSMGI PGAFYIKNYM QVEFFLKTLT LEDVPNQGTI HFVCNSWVYN

161 SKLYKSPR**IF FSNKPYLPSE TPAPLVK**YRE EDLKNLRGDG KGERQEHERI YDYDVYNDLG NPDRNENHAR PILGGSTTFP

241 YPRRGRTGRY PARNDPNSEK PGDVYVPRDE NFGHLKSSDF LANSIKFLTR YVLPAFESVF DLNLTPNEFD SFQDVRDLYE

321 GGIRLPTEVI STISPLPVIK ELFRTDGEQV LKFPPPHIIQ VNKSAWMTDE EFAREMIAGV NPCMIRSLQE FPPKSNLDPT

401 IYGDQNSKIP AEVLDLEGCS LEEAINGRRL FILDYHDVFM PYVRRINETH AKAYATRTIL FLKEDGTLKP VAIELSLPHP

481 DGDKSGAISE VILPAKEGVE STIWLLAKAY VIVNDSCYHQ LMSHWLNTHA VIEPFVIATN RQLSVIHPIY KLLSPHYRDT

561 MNINALARQN LINSDGIIER TFLPSKFSVE MSSAVYKNWV FTDQALPADL IKRGMAVEDS SSPYGIRLVI EDYPYAVDGL

641 EIWFAIKEWV QDYVSLYYPT DNDLKKDPEL QNWWKEAVEV GHGDLKDKPW WPKMQTVEEL VESCTTIIWT ASALHAAVNF

721 GQYPYGGLIL NRPTLSRRLL PEQGTAEYEE MVKSHQKAYL RTITPKLETL IDLTTIEILS KHASDEVYLG ERDNPHWTFD

801 SRALEAFQRF GNKLSEIEEK LTEKNKDGRL SNRIGPVELP YTLLHPTSNE GLTFRGVPNS ISI

Supporting Peptides:

Peptide	Uniq	-10lgP	Mass	Length	ppm	m/z	z	RT	Fraction	Scan	Source File	Area Kontrola 4. traka 20 kDa	#Feature	#Feature Kontrola 4. traka 20 kDa	Start	End	PTM
N.VIGSTVDALTAFLGR.S	Y	61.33	1518.8406	15	1.2	760.4285	2	35.89	5	1866	OB4033.raw	4.42E3	1	1	51	65	
R.IFFSNKPYLPSETPAPLVK.Y	Y	32.22	2147.1665	19	0.2	716.7296	3	31.56	5	1622	OB4033.raw	4.54E2	1	1	169	187	
**total 2 peptides**																	

tr|A1DZF0|A1DZF0_ARAHY

back to list

| Protein Coverage | Supporting Peptides |

Protein Coverage:

1 MAKLLELSFC FCFLVLGASS ISFRQQPEEN ACQFQRLNAQ RPDNRIESEG GYIETWNPNN QEFECAGVAL SRLVLRRNAL

81 RRPFYSNAPQ EIFIQQGRGY FGLIFPGCPS TYEEPAQQGR RYQSQRPPRR LQEEDQSQQQ QDSHQKVHRF NEGDLIAVPT

161 GVAFWLYNDH DTDVVAVSLT DTNNNDNQLD QFPRRFNLAG NHEQEFLRYQ QQSRQSRRRS LPLSPYSPQP GQEDREFSPQ

241 GQHGRRERAG QEQENEGGNI FSGFTSEFLA QAFQVDDRQI VQNLRGENES EEQGAIVTVK GGLRILSPDR KSPDEEEEYD

321 EDEYAEEERQ QDRRRGRGSR GSGNGIEETI CTATVKKNIG RNRSPDIYNP QAGSLKTANE LNLLILR**WLG LSAEYGNLYR**

401 NALFVPHYNT NAHSIIYALR GRAHVQVVDS NGNRVYDEEL QEGHVLVVPQ NFAVAGKSQS ENFEYVAFKT DSRPSIANLA

481 GENSFIDNLP EEVVANSYGL PREQARQLKN NNPFKFFVPP FQQSPRAVA

Supporting Peptides:

Peptide	Uniq	-10lgP	Mass	Length	ppm	m/z	z	RT	Fraction	Scan	Source File	Area Kontrola 4. traka 20 kDa	#Feature	#Feature Kontrola 4. traka 20 kDa	Start	End	PTM
R.WLGLSAEYGNLYR.N	Y	72.50	1540.7673	13	0.9	771.3916	2	33.32	5	1721	OB4033.raw	6.09E3	1	1	388	400	
**total 1 peptides**																	

tr|Q9FZ11|Q9FZ11_ARAHY

back to list

| Protein Coverage | Supporting Peptides |

Protein Coverage:

1 MIRGRLALSV CFCFLVLGAS SISFRQQPEE NACQFQRLNA QRPDNRLESE GGYIETWNPN NQEFECAGVA LSRLVLRRNA

81 LRRPFYSNAP QEIFIQQGRG YFGLIFPGCP STYEEPAQQG RRHQSQRAPR RFEGEDQSQQ QQQDSHQKVR RFDEGDLIAV

161 PTGVALWMFN DHDTDVVAVS LTDTNNNDNQ LDQFPRRFNL AGNHEQEFLR YQQQSRRRSL PYSPYSPQSQ PRQEEREFSP

241 RGQHSRRERA GQEEENEGGN IFSGFTPEFL AQAFQVDDRQ IVQNLRGENE SEEEGAIVTV KGGLRILSPD RKRGADEEEE

321 YDEDEYEYDE EDRRRGRGSR GRGNGIEETI CTASVKKNIG RNRSPDIYNP QAGSLKTAND LNLLILR**WLG LSAEYGNLYR**

401 NALFVPHYNT NAHSIIYALR GRAHVQVVDS NGNRVYDEEL QEGHVLVVPQ NFAVAGKSQS DNFEYVAFKT DSRPNIANFA

481 GENSIIDNLP EEVVANSYGL PREQARQLKN NNPFKFFVPP SQQSLRAVA

Supporting Peptides:

Peptide	Uniq	-10lgP	Mass	Length	ppm	m/z	z	RT	Fraction	Scan	Source File	Area Kontrola 4. traka 20 kDa	#Feature	#Feature Kontrola 4. traka 20 kDa	Start	End	PTM
R.WLGLSAEYGNLYR.N	Y	72.50	1540.7673	13	0.9	771.3916	2	33.32	5	1721	OB4033.raw	6.09E3	1	1	388	400	
**total 1 peptides**																	

tr|B5TYU1|B5TYU1_ARAHY

[back to list]

| Protein Coverage | Supporting Peptides |

Protein Coverage:

```
  1 MAKLLELSFC FCFLVLGASS ISFRQQPEEN ACQFQRLNAQ RPDNRIESEG GYIETWNPNN QEFECAGVAL SRLVLRRNAL
 81 RRPFYSNAPQ EIFIQQGRGY FGLIFPGCPS TYEEPAQQGR RYQSQRPPRR LQEEDQSQQQ QDSHQKVHRF NEGDLIAVPT
161 GVAFWLYNDH DTDVVAVSLT DTNNNDNQLD QFPRRFNLAG NHEQEFLRYQ QQSRQSRRRS LPYSPYSPQS QPRQEEREFS
241 PRGQHSRRER AGQEEENEGG NIFSGFTPEF LAQAFQVDDR QIVQNLRGEN ESEEQGAIVT VRGGLRILSP DRKRGADEEE
321 EYDEDEYEYD EEDRRRGRGS RGSGNGIEET ICTATVKKNI GRNRSPDIYN PQAGSLKTAN ELNLLILRWL GLSAEYGNLY
401 RNALFVPHYN TNAHSIIYAL RGRAHVQVVD SNGNRVYDEE LQEGHVLVVP QNFAVAGKSQ SDNFEYVAFK TDSRPSIANL
481 AGENSVIDNL PEEVVANSYG LPREQARQLK NNNPFKFFVP PSQQSPRAVA
```

Supporting Peptides:

Peptide	Uniq	-10lgP	Mass	Length	ppm	m/z	z	RT	Fraction	Scan	Source File	Area Kontrola 4. traka 20 kDa	#Feature	#Feature Kontrola 4. traka 20 kDa	Start	End	PTM
R.WLGLSAEYGNLYR.N	Y	72.50	1540.7673	13	0.9	771.3916	2	33.32	5	1721	OB4033.raw	6.09E3	1	1	389	401	
**total 1 peptides**																	

tr|Q9SQH7|Q9SQH7_ARAHY

[back to list]

| Protein Coverage | Supporting Peptides |

Protein Coverage:

```
  1 MAKLLELSFC FCFLVLGASS ISFRQQPEEN ACQFQRLNAQ RPDNRIESEG GYIETWNPNN QEFECAGVAL SRLVLRRNAL
 81 RRPFYSNAPQ EIFIQQGRGY FGLIFPGCPS TYEEPAQQGR RYQSQRPPRR LQEEDQSQQQ QDSHQKVHRF NEGDLIAVPT
161 GVAFWLYNDH DTDVVAVSLT DTNNNDNQLD QFPRRFNLAG NHEQEFLRYQ QQSRQSRRRS LPYSPYSPHS RPRREEREFR
241 PRGQHSRRER AGQEEEDEGG NIFSGFTPEF LEQAFQVDDR QIVQNLWGEN ESEEEGAIVT VRGGLRILSP DGTRGADEEE
321 EYDEDQYEYH EQDGRRGRGS RGGGNGIEET ICTACVKKNI GGNRSPHIYD PQRWFTQNCH DLNLLILRWL GLSAEYGNLY
401 RNALFVPHYN TNAHSIIYAL RGRAHVQVVD SNGNRVYDEE LQEGHVLVVP QNFAVAGKSQ SENFEYVAFK TDSRPSIANF
481 AGENSFIDNL PEEVVANSYG LPREQARQLK NNNPFKFFVP PFQQSPRAVA
```

Supporting Peptides:

Peptide	Uniq	-10lgP	Mass	Length	ppm	m/z	z	RT	Fraction	Scan	Source File	Area Kontrola 4. traka 20 kDa	#Feature	#Feature Kontrola 4. traka 20 kDa	Start	End	PTM
R.WLGLSAEYGNLYR.N	Y	72.50	1540.7673	13	0.9	771.3916	2	33.32	5	1721	OB4033.raw	6.09E3	1	1	389	401	
**total 1 peptides**																	

tr|Q5I6T2|Q5I6T2_ARAHY

back to list

| **Protein Coverage** | **Supporting Peptides** |

Protein Coverage:

```
  1 MAKLLELSFC FCFLVLGASS ISFRQQPEEN ACQFQRLNAQ RPDNRIESEG GYIETWNPNN QEFECAGVAL SRLVLRRNAL
 81 RRPFYSNAPQ EIFIQQGRGY FGLIFPGCPS TYEEPAQQGR RSQSQRPPRR LQGEDQSQQQ QDSHQKVHRF DEGDLIAVPT
161 GVAFWLYNDH DTDVVAVSLT DTNNNDNQLD QFPRRFNLAG NHEQEFLRYQ QQSRQSRRRS LPYSPYSPQS QPRQEEREFS
241 PRGQHSRRER AGQEEENEGG NIFSGFTPEF LEQAFQVDDR QIVQNLRGEN ESEEEGAIVT VRGGLRILSP DRKRGADEEE
321 EYDEDEYEYD EEDRRRGRGS RGRGNGIEET ICTASVKKNI GRNRSPDIYN PQAGSLKTAN DLNLLILRWL GLSAEYGNLY
401 RNALFVPHYN TNAHSIIYAL RGRAHVQVVD SNGNRVYDEE LQEGHVLVVP QNFAVAGKSQ SDNFEYVAFK TDSRPSIANL
481 AGENSVIDNL PEEVVANSYG LQREQARQQL KNNNPFKFFV PPSQQSPRAV A
```

Supporting Peptides:

Peptide	Uniq	-10lgP	Mass	Length	ppm	m/z	z	RT	Fraction	Scan	Source File	Area Kontrola 4. traka 20 kDa	#Feature	#Feature Kontrola 4. traka 20 kDa	Start	End	PTM
R.WLGLSAEYGNLYR.N	Y	72.50	1540.7673	13	0.9	771.3916	2	33.32	5	1721	OB4033.raw	6.09E3	1	1	389	401	
**total 1 peptides**																	

tr|Q647H4|Q647H4_ARAHY

back to list

| **Protein Coverage** | **Supporting Peptides** |

Protein Coverage:

```
  1 MGKLLALSVC FCFLVLGASS ISFRQQPEEN ACQFQRLNAQ RPDNRIESEG GYIETWNPNN QEFECAGVAL SRLVLRRNAL
 81 RRPFYSNAPQ EIFIQQGRGY FGLIFPGCPS TYEEPAQQGR RHQSQRPPRR FQGQDQSQQQ QDSHQKVHRF DEGDLIAVPT
161 GVAFWMYNDH DTDVVAVSLT DTNNNDNQLD QFPRRFNLAG NHEQEFLRYQ QQSRRRSLPY SPYSPQTQPK QEDREFSPRG
241 QHGRRERAGQ EQENEGGNIF SGFTPEFLAQ AFQVDDRQIL QNLRGENESD EQGAIVTVRG GLRILSPDRK RRQQYERPDE
321 EEEYDEDEYE YDEEERQHDR RRGRGSRGSG NGIEETICTA SFKKNIGRNR SPDIYNPQAG SLKTANELNL LILRWLGLSA
401 EYGNLYRNAL FVPHYNTNAH SIIYALRGRA HVQVVDSNGD RVFDEELQEG HVLVVPQNFA VAGKSQSENF EYVAFKTDSR
481 PSIANLAGEN SFIDNLPEEV VANSYGLPRE QARQLKNNNP FKFFVPPSEQ SLRAVA
```

Supporting Peptides:

Peptide	Uniq	-10lgP	Mass	Length	ppm	m/z	z	RT	Fraction	Scan	Source File	Area Kontrola 4. traka 20 kDa	#Feature	#Feature Kontrola 4. traka 20 kDa	Start	End	PTM
R.WLGLSAEYGNLYR.N	Y	72.50	1540.7673	13	0.9	771.3916	2	33.32	5	1721	OB4033.raw	6.09E3	1	1	395	407	
**total 1 peptides**																	

tr|Q6T2T4|Q6T2T4_ARAHY

back to list

| Protein Coverage | Supporting Peptides |

Protein Coverage:

```
  1 MGKLLALSVC FCFLVLGASS ISFRQQPEEN ACQFQRLNAQ RPDNRIESEG GYIETWNPNN QEFECAGVAL SRLVLRRNAL
 81 RRPFYSNAPQ EIFIQQGRAY FGLIFLGCPS TYEEPAQQGR RHQSQRPPRR FQGQDQSQQQ QDSHQKVHRF DEGDLIAVPT
161 GVAFWMYNDH DTDVVAVSLT DTNNNDNQLD QFPRRFNLAG NHEQEFLRYQ QQSRRRSLPY SPYSPQTQPK QEDREFSPRG
241 QHGRRERAGQ EQENEGGNIF SGFTPEFLAQ AFQVDDRQIL QNLRGENESD EQGAIVTVRG GLRILSPDRK KRQQYERPDE
321 EEEYDEDEYE YDEEERQQDR RRGRGSRGSG NGIEETICTA SFKKNIGRNR SPDIYNPQAG SLKTANELNL LILRWLGLSA
401 EYGNLYRNAL FVPHYNTNAH SIIYALRGRA HVQVVDSNGD RVFDEELQEG HVLVVPQNFA VAGKSQSENF EYVAFKTDSR
481 PSIANLAGEN SFIDNLPEEV VANSYGLPRE QARQLKNNNP FKFFVPPSEQ SLRAVA
```

Supporting Peptides:

Peptide	Uniq	-10lgP	Mass	Length	ppm	m/z	z	RT	Fraction	Scan	Source File	Area Kontrola 4. traka 20 kDa	#Feature	#Feature Kontrola 4. traka 20 kDa	Start	End	PTM
R.WLGLSAEYGNLYR.N	Y	72.50	1540.7673	13	0.9	771.3916	2	33.32	5	1721	OB4033.raw	6.09E3	1	1	395	407	
**total 1 peptides**																	

tr|Q647H3|Q647H3_ARAHY
back to list

| Protein Coverage | Supporting Peptides |

Protein Coverage:

```
  1 MAKLLALSVC FCFLVLGASS ISFRQQPEEN ACQFQRLNAQ RPDNRLESEG GYIETWNPNN QEFECAGVAL SRLVLRRNAL
 81 RRPFYSNAPQ EIFIQQGRGY FGLIFPGCPS TYEEPAQQGR RHQSQRAPRR FEGEDQSQQQ QQDSHQKVRR FDEGDLIAVP
161 TGVALWMYND HDTDVVAVSL TDTNNNDNQL DQFPRRFNLA GNHEQEFLRY QQQSRRRSLP YSPYSPQSQP RQEEREFSPR
241 GQHSRRERAG QEQENEGGNI FSGFTPEFLA QAFQVDDRQI LQNLRGENES DEQGAIVTVR GGLRILSPDR KRRQQYERPD
321 EEEEYDEDEY EYDEEERQQD RRRGRGSRGR GNGIEETICT ASVKKNIGRN RSPDIYNPQA GSLKTANDLN LLILRWLGLS
401 AEYGNLYRNA LFVPHYNTNA HSIIYALRGR AHVQVVDSNG NRVYDEELQE GHVLVVPQNF AVAGKSQSDN FEYVAFKTDS
481 RPSIANLAGE NSIIDNLPEE VVANSYGLPR EQARQLKNNN PFKFFVPPSQ QSLGAVA
```

Supporting Peptides:

Peptide	Uniq	-10lgP	Mass	Length	ppm	m/z	z	RT	Fraction	Scan	Source File	Area Kontrola 4. traka 20 kDa	#Feature	#Feature Kontrola 4. traka 20 kDa	Start	End	PTM
R.WLGLSAEYGNLYR.N	Y	72.50	1540.7673	13	0.9	771.3916	2	33.32	5	1721	OB4033.raw	6.09E3	1	1	396	408	
**total 1 peptides**																	

tr|Q8LKN1|Q8LKN1_ARAHY
back to list

| Protein Coverage | Supporting Peptides |

Protein Coverage:

```
  1 MGKLLALSVC FCFLVLGASS ISFRQQPEEN ACQFQRLNAQ RPDNRIESEG GYIETWNPNN QEFECAGVAL SRLVLRRNAL
 81 RRPFYSNAPQ EIFIQQGRGY FGLIFPGCPS TYEEPAQQGR RHQSQRPPRR FQGQDQSQQQ QDSHQKVHRF DEGDLIAVPT
161 GVAFWMYNDH DTDVVAVSLT DTNNNDNQLD QFPRRFNLAG NHEQEFLRYQ QQSRRRSLPY SPYSPQTQPK QEDREFSPRG
241 QHGRRERAGQ EQENEGGNIF SGFTPEFLAQ AFQVDDRQIL QNLRGENESD EQGAIVTVRG GLRILSPDRK RRQQYERPDE
321 EEEYDEDEYE YDEEERQQDR RRGRGSRGSG NGIEETICTA SFKKNIGRNR SPDIYNPQAG SLKTANELQL NLLILRWLGL
401 SAEYGNLYRN ALFVPHYNTN AHSIIYALRG RAHVQVVDSN GDRVFDEELQ EGHVLVVPQN FAVAGKSQSE NFEYVAFKTD
481 SRPSIANLAG ENSFIDNLPE EVVANSYGLP REQARQLKNN NPFKFFVPPS EQSLRAVA
```

Supporting Peptides:

Peptide	Uniq	-10lgP	Mass	Length	ppm	m/z	z	RT	Fraction	Scan	Source File	Area Kontrola 4. traka 20 kDa	#Feature	#Feature Kontrola 4. traka 20 kDa	Start	End	PTM
R.WLGLSAEYGNLYR.N	Y	72.50	1540.7673	13	0.9	771.3916	2	33.32	5	1721	OB4033.raw	6.09E3	1	1	397	409	
**total 1 peptides**																	

tr|Q38711|Q38711_ARAHY

back to list

| Protein Coverage | Supporting Peptides |

Protein Coverage:

```
  1 KPFCVFLTFF LLLAASKKVN SAETVSFNFN SFSEGNPAIN FQGDVTVLSN GNIQLTNLNK VNSVGRVLYA MPVRIWSSAT
 81 GNVASFLTSF SFEMKDIKDY DPADGIIFFI APEDTQIPAG SIGGGTLGVS DTKGAGHFVG VEFDTYSNSE YNDPPTDHVG
161 IDVNSVDSVK TVPWNSVSGA VVQVTVIYDS STKTLSVAVT NENGDITTIA QVVDLKAKLP ERVKFGFSAS GSLGGRQIHL
241 IRSWSFTSTL ITTTRRSIDN NEKKIMNMAT A
```

Supporting Peptides:

Peptide	Uniq	-10lgP	Mass	Length	ppm	m/z	z	RT	Fraction	Scan	Source File	Area Kontrola 4. traka 20 kDa	#Feature	#Feature Kontrola 4. traka 20 kDa	Start	End	PTM
R.SWSFTSTLITTTR.R	Y	53.52	1499.7620	13	-0.9	750.8876	2	33.15	5	1713	OB4033.raw	1.59E3	1	1	243	255	
**total 1 peptides**																	

tr|A0A089ZXL7|A0A089ZXL7_ARAHY

back to list

| Protein Coverage | Supporting Peptides |

Protein Coverage:

```
  1 MKPFCVFLTF FLLLAASSKK VNSAETVSFN FNSFSEGNPA INFQGDVTVL SNGNIQLTNL NKVNSVGRVL YAMPVRIWSS
 81 ATGNVASFLT SFSFEMKDIK DYDPADGIIF FIAPEDTQIP AGSIGGGTLG VSDTKGAGYF VGVEFDTYSN SEYNDPPTDH
161 VGIDVNSVDS VKTVPWNSVS GAVVKVTVIY DSSTKTLSVA VTNDNGDITT IAQVVDLKAK LPERVKFGFS ASGSRAGRQI
241 HLIRSWSFTS TLITTTRRSI DNNEKKIMNM ASA
```

Supporting Peptides:

Peptide	Uniq	-10lgP	Mass	Length	ppm	m/z	z	RT	Fraction	Scan	Source File	Area Kontrola 4. traka 20 kDa	#Feature	#Feature Kontrola 4. traka 20 kDa	Start	End	PTM
R.SWSFTSTLITTTR.R	Y	53.52	1499.7620	13	-0.9	750.8876	2	33.15	5	1713	OB4033.raw	1.59E3	1	1	245	257	
**total 1 peptides**																	

P02872|LECG_ARAHY

back to list

| Protein Coverage | Supporting Peptides |

Protein Coverage:

```
  1 MKPFCVFLTF FLLLAASSKK VDSAETVSFN FNSFSEGNPA INFQGDVTVL SNGNIQLTNL NKVNSVGRVL YAMPVRIWSS
 81 ATGNVASFLT SFSFEMKDIK DYDPADGIIF FIAPEDTQIP AGSIGGGTLG VSDTKGAGHF VGVEFDTYSN SEYNDPPTDH
161 VGIDVNSVDS VKTVPWNSVS GAVVKVTVIY DSSTKTLSVA VTNDNGDITT IAQVVDLKAK LPERVKFGFS ASGSLGGRQI
241 HLIRSWSFTS TLITTTRRSI DNNEKKIMNM ASA
```

Supporting Peptides:

Peptide	Uniq	-10lgP	Mass	Length	ppm	m/z	z	RT	Fraction	Scan	Source File	Area Kontrola 4. traka 20 kDa	#Feature	#Feature Kontrola 4. traka 20 kDa	Start	End	PTM
R.SWSFTSTLITTTR.R	Y	53.52	1499.7620	13	-0.9	750.8876	2	33.15	5	1713	OB4033.raw	1.59E3	1	1	245	257	
**total 1 peptides**																	

tr|Q6IWG5|Q6IWG5_ARAHY

back to list

| Protein Coverage | Supporting Peptides |

Protein Coverage:

```
  1 KLLALSLCFC VLVLGASSVT FRQGGEENEC QFQRLNAQRP DNRIESEGGY IETWNPNNQE FQCAGVALSR TVLRRNALRR
 81 PFYSNAPLEI YVQQGSGYFG LIFPGCPSTY EEPAQEGRRY QSQKPSRRFQ VGQDDPSQQQ QDSHQKVHRF DEGDLIAVPT
161 GVAFWMYNDE DTDVVTVTLS DTSSIHNQLD QFPRRFYLAG NQEQEFLRYQ QQQGSRPHYR QISPRVRGDE QENEGSNIFS
241 GFAQEFLQHA FQVDRQTVEN LRGENEREEQ GAIVTVKGGL RILSPDEEDE SSRSPPSRRE EFDEDRSRPQ QRGKYDENRR
321 GYKNGIEETI CSASVKKNLG RSSNPDIYNP QAGSLRSVNE LDLPILGWLG LSAQHGTIYR NAMFVPHYTL NAHTIVVALN
401 GRAHVQVVDS NGNRVYDEEL QEGHVLVVPQ NFAVAAKAQS ENYEYLAFKT DSRPSIANLA GENSIIDNLP EEVVANSYRL
481 PREQARQLKN NNPFKFFVPP FDHQSMREVA
```

d Deamidation (NQ) (+0.98)

Supporting Peptides:

Peptide	Uniq	-10lgP	Mass	Length	ppm	m/z	z	RT	Fraction	Scan	Source File	Area Kontrola 4. traka 20 kDa	#Feature	#Feature Kontrola 4. traka 20 kDa	Start	End	PTM
R.SVNELDLPILGWLGLSAQHGTIYR.N	Y	31.78	2651.4070	24	2.7	884.8120	3	38.46	5	2013	OB4033.raw	1.33E3	1	1	357	380	
R.SVN(+.98)ELDLPILGWLGLSAQ(+.98)HGTIYR.N	Y	22.89	2653.3750	24	3.5	885.4687	3	38.91	5	2040	OB4033.raw	0	0	0	357	380	Deamidation (NQ)
**total 2 peptides**																	

tr|E5G077|E5G077_ARAHY

back to list

| Protein Coverage | Supporting Peptides |

Protein Coverage:

```
  1 MAKLLALSLC FCVLVLGASS VTFRQGGEEN ECQFQRLNAQ RPDNRIESEG GYIETWNPNN QEFQCAGVAL SRTVLRRNAL
 81 RRPFYSNAPL EIYVQQGSGY FGLIFPGCPS TYEEPAQEGR RYQSQKPSRR FQVGQDDPSQ QQQDSHQKVH RFDEGDLIAV
161 PTGVAFWMYN DEDTDVVTVT LSDTSSIHNQ LDQFPRRFYL AGNQEQEFLR YQQQQGSRPH YRQISPRVRG DEQENEGSNI
241 FSGFAQEFLQ HAFQVDRQTV ENLRGENERE EQGAIVTVKG GLRILSPDEE DESSRSPPNR REEFDEDRSR PQQRGKYDEN
321 RRGYKNGIEE TICSASVKKN LGRSSNPDIY NPQAGSLRSV NELDLPILGW LGLSAQHGTI YRNAMFVPHY TLNAHTIVVA
401 LNGRAHVQVV DSNGNRVYDE ELQEGHVLVV PQNFAVAAKA QSENYEYLAF KTDSRPSIAN QAGENSIIDN LPEEVVANSY
481 RLPREQARQL KNNNPFKFFV PPFDHQSMRE VA
```

d Deamidation (NQ) (+0.98)

Supporting Peptides:

Peptide	Uniq	-10lgP	Mass	Length	ppm	m/z	z	RT	Fraction	Scan	Source File	Area Kontrola 4. traka 20 kDa	#Feature	#Feature Kontrola 4. traka 20 kDa	Start	End	PTM
R.SVNELDLPILGWLGLSAQHGTIYR.N	Y	31.78	2651.4070	24	2.7	884.8120	3	38.46	5	2013	OB4033.raw	1.33E3	1	1	359	382	
R.SVN(+.98)ELDLPILGWLGLSAQ(+.98)HGTIYR.N	Y	22.89	2653.3750	24	3.5	885.4687	3	38.91	5	2040	OB4033.raw	0	0	0	359	382	Deamidation (NQ)
**total 2 peptides**																	

tr|Q0GM57|Q0GM57_ARAHY
back to list

| **Protein Coverage** | **Supporting Peptides** |

Protein Coverage:

```
  1   MAKLLALSLC FCVLVLGASS VTFRQGGEEN ECQFQRLNAQ RPDNRIESEG GYIETWNPNN QEFQCAGVAL SRTVLRRNAL
 81   RRPFYSNAPL EIYVQQGSGY FGLIFPGCPS TYEEPAQEGR RYQSQKPSRR FQVGQDDPSQ QQQDSHQKVH RFDEGDLIAV
161   PTGVAFWMYN DEDTDVVTVT LSDTSSIHNQ LDQFPRRFYL AGNQEQEFLR YQQQQGSRPH YRQISPRVRG DEQENEGSNI
241   FSGFAQEFLQ HAFQVDRQTV ENLRGENERE EQGAIVTVKG GLRILSPDEE DESSRSPPSR REEFDEDRSR PQQRGKYDEN
321   RRGYKNGIEE TICSASVKKN LGRSSNPDIY NPQAGSLRSV NELDLPILGW LGLSAQHGTI YRNAMFVPHY TLNAHTIVVA
                                                  d                 d
401   LNGRAHVQVV DSNGNRVYDE ELQEGHVLVV PQNFAVAAKA QSENYEYLAF KTDSRPSIAN LAGENSIIDN LPEEVVANSY
481   RLPREQARQL KNNNPFKFFV PPFDHQSMRE VA
```

d Deamidation (NQ) (+0.98)

Supporting Peptides:

Peptide	Uniq	-10lgP	Mass	Length	ppm	m/z	z	RT	Fraction	Scan	Source File	Area Kontrola 4. traka 20 kDa	#Feature	#Feature Kontrola 4. traka 20 kDa	Start	End	PTM
R.SVNELDLPILGWLGLSAQHGTIYR.N	Y	31.78	2651.4070	24	2.7	884.8120	3	38.46	5	2013	OB4033.raw	1.33E3	1	1	359	382	
R.SVN(+.98)ELDLPILGWLGLSAQ(+.98)HGTIYR.N	Y	22.89	2653.3750	24	3.5	885.4687	3	38.91	5	2040	OB4033.raw	0	0	0	359	382	Deamidation (NQ)
**total 2 peptides**																	

Peptide List

1. Notes Gastric Digest Raw peanut Band #5 20-22 kDa

2. Result Statistics

Figure 1. False discovery rate (FDR) curve. X axis is the number of peptide-spectrum matches (PSM) being kept. Y axis is the corresponding FDR.

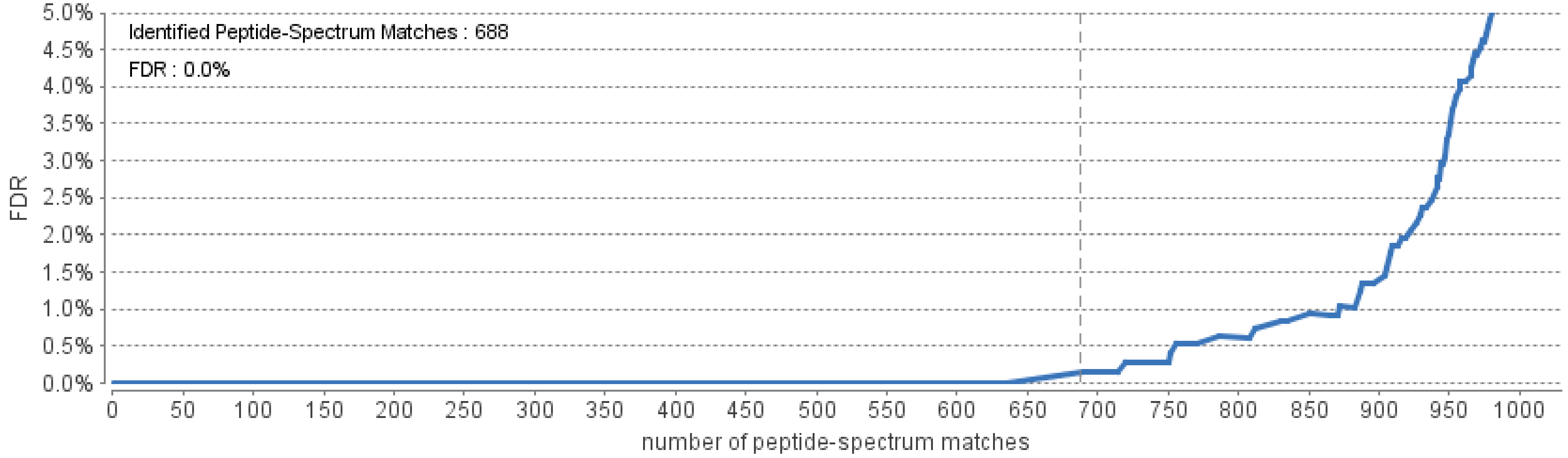

Figure 2. PSM score distribution. **(a)** Distribution of PEAKS peptide score; **(b)** Scatterplot of PEAKS peptide score versus precursor mass error.

(a)

(b)

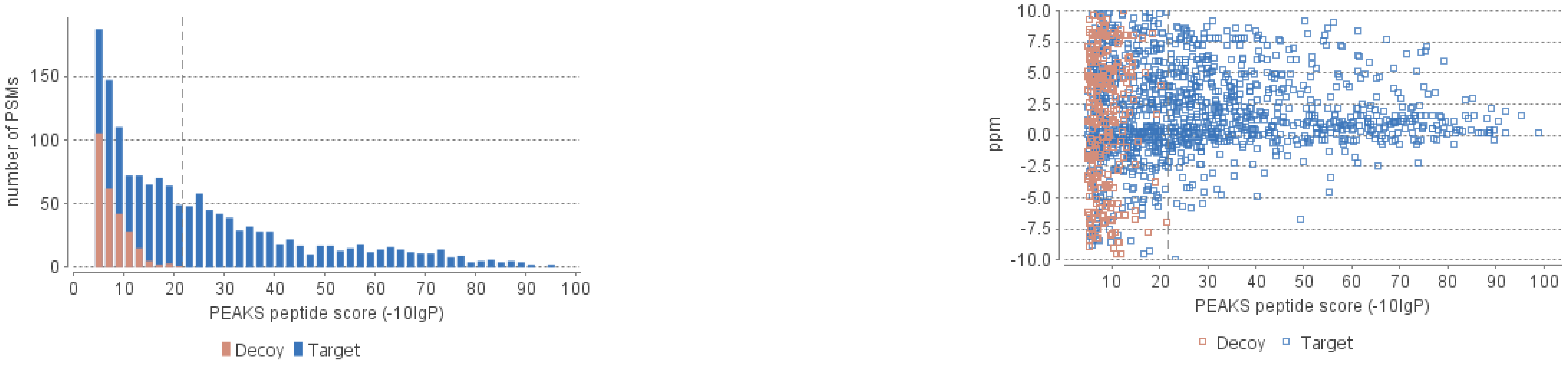

Figure 3. Distribution of peptide feature detection. **(a)** Feature m/z distribution; **(b)** Feature RT distribution

(a) **(b)**

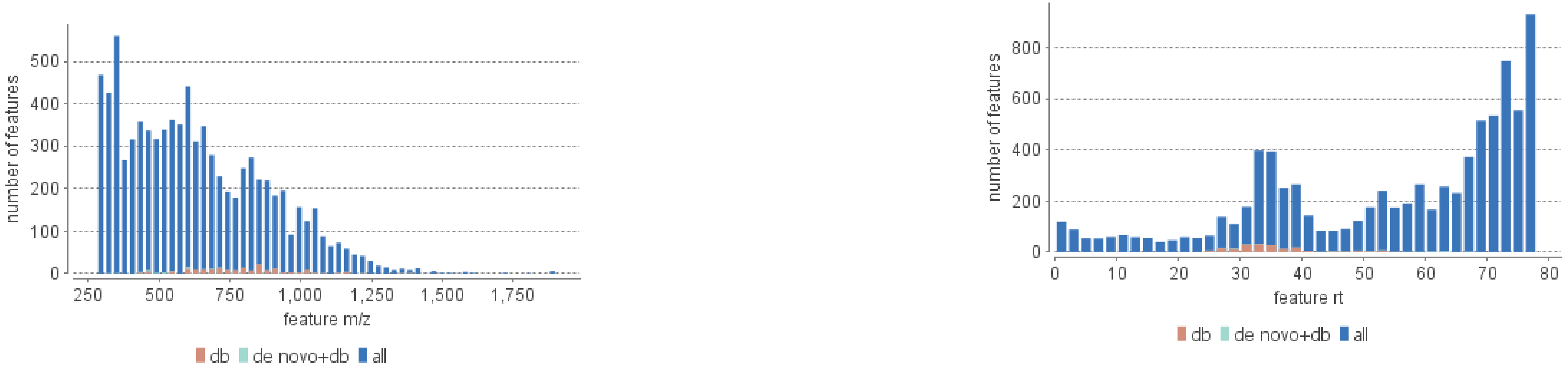

Figure 4. Distribution of identified peptide features. **(a)** Feature abundance distribution **(b)** *De novo* sequencing validation.

(a)

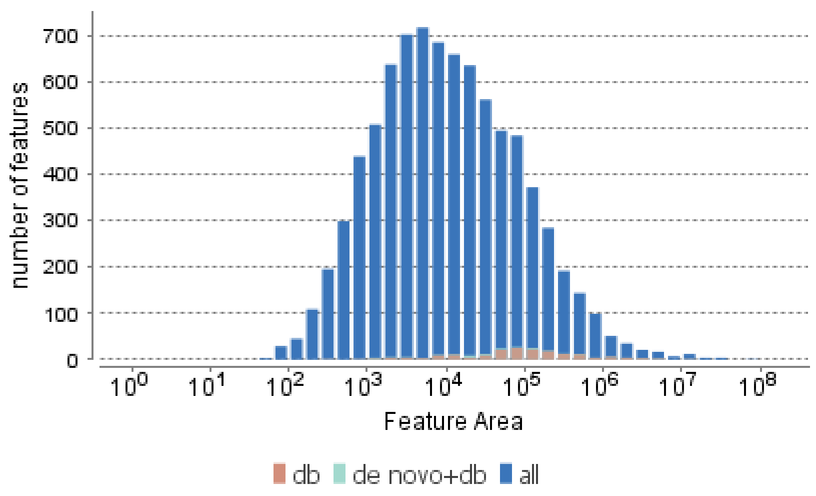

(b)

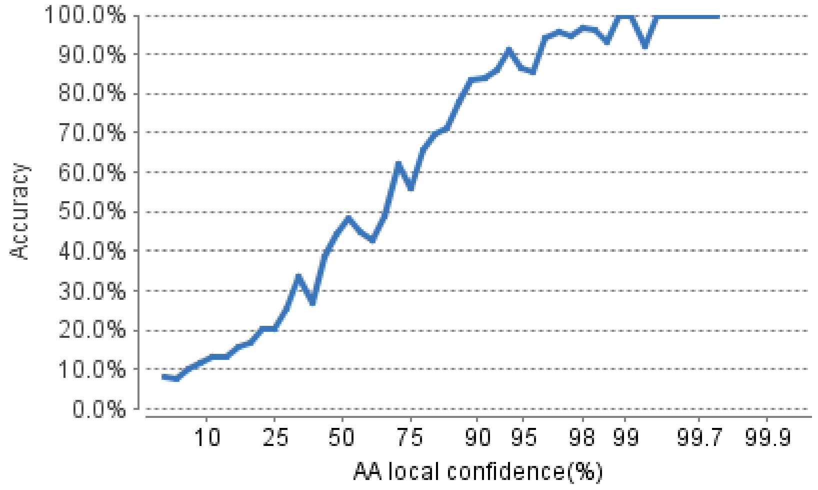

Table 1. Statistics of data.

# of MS scans	2342
# of MS/MS scans	2105
# of Features	8453
# of Chimera scans	429

Table 2. Result filtration parameters.

Peptide -10lgP	≥21.6
PTM Ascore	≥20
Protein -10lgP	≥20
Proteins unique peptides	≥1
De novo ALC Score	≥50%

Table 4. PTM profile.

Name	ΔMass	Position	#PSM	-10lgP	Abundance	AScore
Deamidation	.98	NQ	407	89.03	6.12E4	83.60
HydPro	15.99	P	45	57.00		0.00
Carbamidomethyl	57.02	C	33	77.66	1.3E5	1000.00
Oxidation	15.99	HW	7	35.89	8.59E3	1000.00
Methylation(KR)	14.02	KR	5	37.66	1.57E4	61.26
Pyro-glu from Q	-17.03	N-term	4	32.75	4.47E4	1000.00
Dihydroxy	31.99	PRW	3	33.32	9.71E4	0.00

Table 3. Statistics of filtered result.

Peptide-Spectrum Matches	688
Peptide sequences	289
Protein groups	7
Proteins	8
Proteins (#Unique Peptides)	3 (>2); 3 (=2); 2 (=1);
FDR (Peptide-Spectrum Matches)	0.0%
FDR (Peptide Sequences)	0.0%
FDR (Protein Group)	0.0%
De Novo Only Spectra	42
# of identified Features	201
# of identified MS/MS scans	621

Oxidation	15.99	M	2	69.67	8.15E4	1000.00
Propionamide	71.04	C	2	46.25	3.51E4	1000.00
Propionamide	71.04	N-term	2	42.77	3.7E4	1000.00
Ethyl	28.03	E	2	37.16	1.79E5	0.00
Ubiquitin	114.04	T	1	27.03	2.47E4	69.29
Carbamylation	43.01	N-term	1	53.67	7.92E4	1000.00
Carboxymethyl	58.01	C	1	37.75	1.22E4	1000.00
ser_thr_DAET	87.05	S	1	25.32	1.83E5	7.25
Cation:Fe[II]	53.92	E	1	27.19	6.3E4	0.00
Methylation(others)	14.02	T	1	26.25		0.00
ISD_z+2_ion	-15.01	N-term	1	24.82		1000.00
Carboxylation	43.99	W	1	25.59		15.76
B-methylthiol	45.99	N	1	30.26		83.43

3. Experiment Control

Figure 5. Precursor mass error of peptide-spectrum matches (PSM) in filtered result. **(a)** Distribution of precursor mass error in ppm; **(b)** Scatterplot of precursor m/z versus precursor mass error in ppm.

(a)

(b)

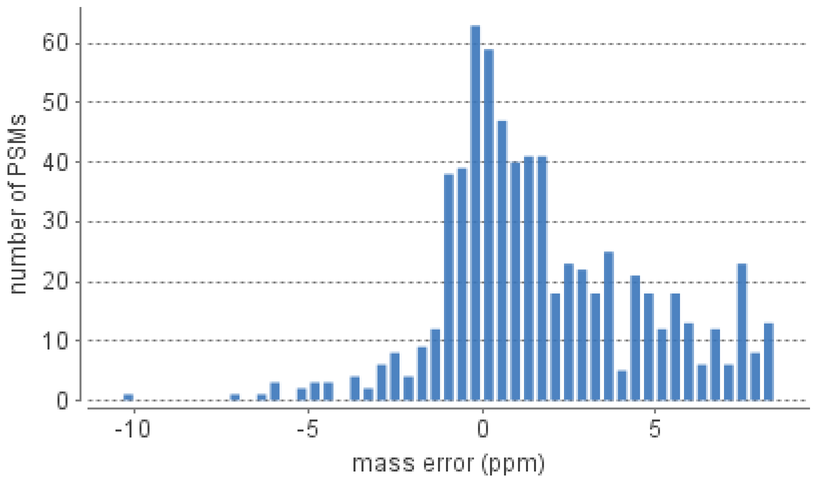

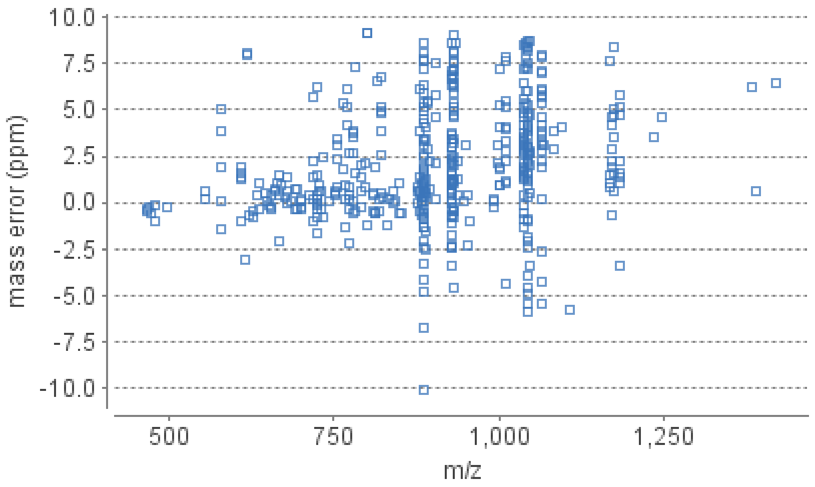

Table 5. Number of identified peptides in each sample by the number of missed cleavages

Missed Cleavages	0	1	2	3	4+
Digest 5 traka 22 kDa	90	98	101	0	0

4. Other Information

Table 6. Search parameters.

Search Engine Name: PEAKS
Parent Mass Error Tolerance: 10.0 ppm
Fragment Mass Error Tolerance: 0.5 Da
Precursor Mass Search Type: monoisotopic
Enzyme: Trypsin
Max Missed Cleavages: 2
Digest Mode: Unspecific
Fixed Modifications:

Table 7. Instrument parameters.

Fractions: OB4055.raw
Ion Source: ESI(nano-spray)
Fragmentation Mode: CID, CAD(y and b ions)
MS Scan Mode: FT-ICR/Orbitrap
MS/MS Scan Mode: Linear Ion Trap

Carbamidomethylation: 57.02
Variable Modifications:
Deamidation (NQ): 0.98
Oxidation (M): 15.99
Hydroxylation Pro: 15.99
Acetylation (K): 42.01
Acetylation (Protein N-term): 42.01
Acetylation (N-term): 42.01
Amidation: -0.98
Beta-methylthiolation: 45.99
and 305 more...
Max Variable PTM Per Peptide: 5
Database: Uniprot_Peanut-3818_Jul18
Taxon: All
Contaminant Database: contaminantsMQ_mar19
Searched Entry: 1723
FDR Estimation: Enabled
De novo score (ALC%) threshold: 15
Peptide hit threshold (-10logP): 30.0
Peaks run ID: 476
Merge Options: no merge
Precursor Options: corrected
Charge Options: no correction
Filter Charge: 2 - 8
Process: true
Associate chimera: yes

Protein List

Protein Accession Contains:
Protein Description Contains:
Peptide Sample Area >=
Protein Ptm Contains:

Protein Group	Protein ID	Accession	-10lgP	Coverage (%)	Coverage (%) Digest 5 traka 22 kDa	Area Digest 5 traka 22 kDa	#Peptides	#Unique	#Spec Digest 5 traka 22 kDa	PTM	Avg. Mass	Description
**2**	20581	tr|Q647H3|Q647H3_ARAHY	309.17	34	34	5.51E6	29	4	249	Y	61532	Arachin Ahy-2 OS=Arachis hypogaea OX=3818 PE=2 SV=1
**1**	20574	tr|A1DZF0|A1DZF0_ARAHY	305.89	34	34	3.92E6	32	2	259	Y	60375	Arachin 6 OS=Arachis hypogaea OX=3818 PE=2 SV=1
**7**	20578	tr|B5TYU1|B5TYU1_ARAHY	300.00	31	31	2.11E6	26	2	217	Y	60624	Arachin Arah3 isoform OS=Arachis hypogaea OX=3818 PE=1 SV=1
**5**	20576	tr|Q9FZ11|Q9FZ11_ARAHY	293.74	28	28	1.91E5	24	1	216	Y	60449	Gly1 OS=Arachis hypogaea OX=3818 GN=Gly1 PE=2 SV=1
**8**	20582	tr|Q8LKN1|Q8LKN1_ARAHY	282.23	28	28	7.49E6	27	2	140	Y	61738	Allergen Arah3/Arah4 OS=Arachis hypogaea OX=3818 PE=3 SV=1
**6**	20593	tr|Q6IWG5|Q6IWG5_ARAHY	269.16	35	35	3.67E7	27	25	176	Y	58061	Glycinin (Fragment) OS=Arachis hypogaea OX=3818 PE=2 SV=1
**6**	20594	tr|Q0GM57|Q0GM57_ARAHY	269.16	35	35	3.67E7	27	25	176	Y	58263	Iso-Ara h3 OS=Arachis hypogaea OX=3818 PE=2 SV=1
**9**	20590	tr|O82580|O82580_ARAHY	229.09	24	24	0E0	14	1	50	Y	58350	Glycinin (Fragment) OS=Arachis hypogaea OX=3818 GN=Arah3 PE=2 SV=1
**total 8 proteins**												

tr|Q647H3|Q647H3_ARAHY
back to list

| **Protein Coverage** | **Supporting Peptides** |

Protein Coverage:

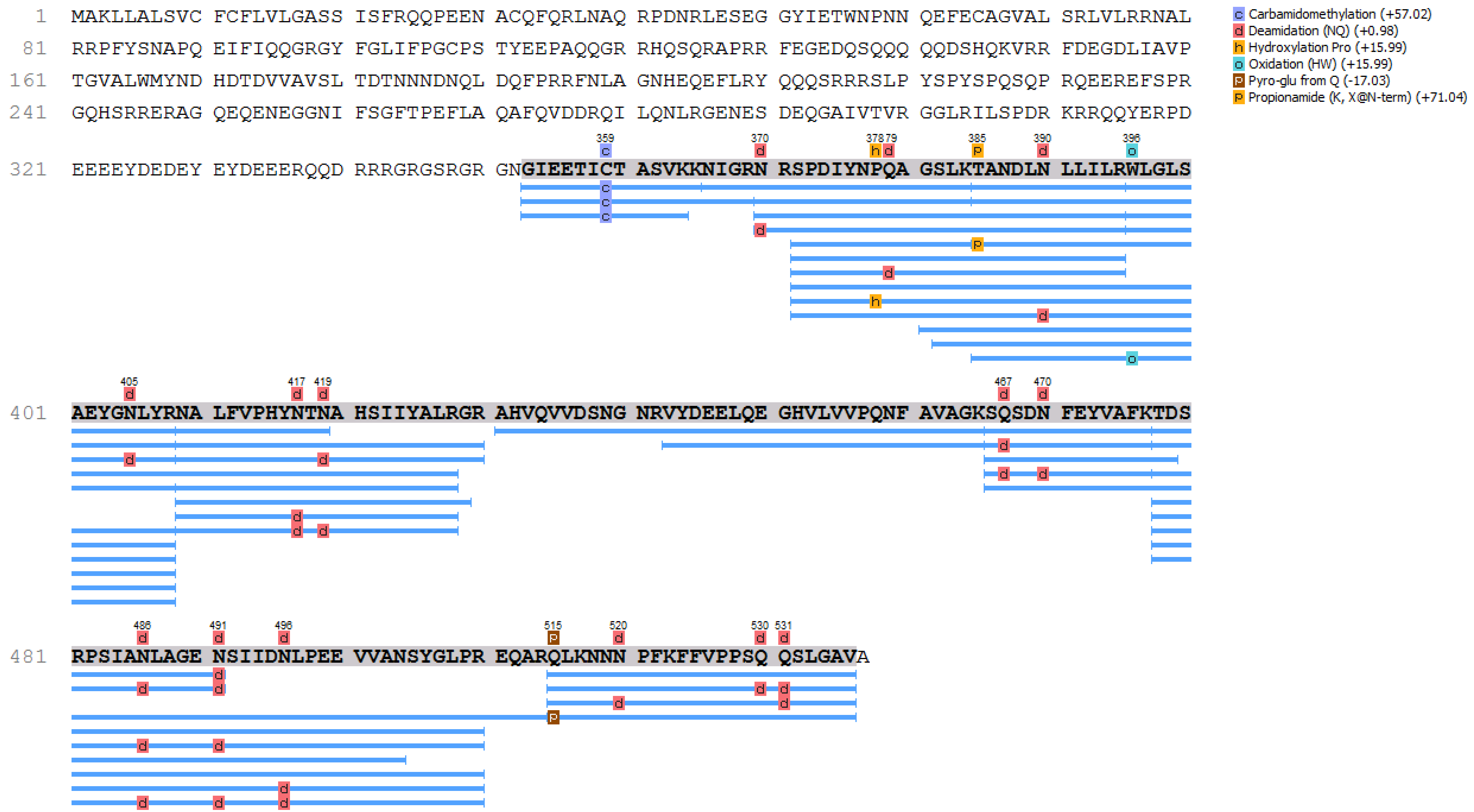
1 MAKLLALSVC FCFLVLGASS ISFRQQPEEN ACQFQRLNAQ RPDNRLESEG GYIETWNPNN QEFECAGVAL SRLVLRRNAL
81 RRPFYSNAPQ EIFIQQGRGY FGLIFPGCPS TYEEPAQQGR RHQSQRAPRR FEGEDQSQQQ QQDSHQKVRR FDEGDLIAVP
161 TGVALWMYND HDTDVVAVSL TDTNNNDNQL DQFPRRFNLA GNHEQEFLRY QQQSRRRSLP YSPYSPQSQP RQEEREFSPR
241 GQHSRRERAG QEQENEGGNI FSGFTPEFLA QAFQVDDRQI LQNLRGENES DEQGAIVTVR GGLRILSPDR KRRQQYERPD
321 EEEEYDEDEY EYDEEERQQD RRRGRGSRGR GNGIEETICT ASVKKNIGRN RSPDIYNPQA GSLKTANDLN LLILRWLGLS
401 AEYGNLYRNA LFVPHYNTNA HSIIYALRGR AHVQVVDSNG NRVYDEELQE GHVLVVPQNF AVAGKSQSDN FEYVAFKTDS
481 RPSIANLAGE NSIIDNLPEE VVANSYGLPR EQARQLKNNN PFKFFVPPSQ QSLGAVA
c Carbamidomethylation (+57.02)
d Deamidation (NQ) (+0.98)
h Hydroxylation Pro (+15.99)
o Oxidation (HW) (+15.99)
p Pyro-glu from Q (-17.03)
p Propionamide (K, X@N-term) (+71.04)

Supporting Peptides:

Peptide	Uniq	-10lgP	Mass	Length	ppm	m/z	z	RT	Fraction	Scan	Source File	Area Digest 5 traka 22 kDa	#Feature	#Feature Digest 5 traka 22 kDa	Start	End	PTM
R.WLGLSAEYGNLYR.N	N	98.82	1540.7673	13	0.1	771.3911	2	33.33	27	1749	OB4055.raw	1.54E6	1	1	396	408	
K.TANDLNLLILRWLGLSAEYGNLYR.N	N	95.42	2777.4863	24	1.6	926.8375	3	39.71	27	2197	OB4055.raw	4.05E6	4	4	385	408	
K.TDSRPSIANLAGENSIIDNLPEEVVANSYGLPR.E	Y	91.97	3510.7590	33	2.0	1171.2626	3	35.65	27	1921	OB4055.raw	0	0	0	478	510	
K.SQ(+.98)SDNFEYVAFK.T	N	89.03	1434.6302	12	2.2	718.3240	2	30.79	27	1580	OB4055.raw	6.12E4	1	1	466	477	Deamidation (NQ)
K.SQSDNFEYVAFK.T	N	88.10	1433.6462	12	1.3	717.8314	2	30.42	27	1552	OB4055.raw	1.4E5	1	1	466	477	
K.TANDLN(+.98)LLILRWLGLSAEYGNLYR.N	N	85.28	2778.4705	24	3.0	927.1669	3	40.36	27	2227	OB4055.raw	6.5E3	3	3	385	408	
R.SPDIYNPQ(+.98)AGSLKTANDLNLLILR.W	N	79.73	2626.3965	24	0.0	876.4728	3	33.97	27	1802	OB4055.raw	2.51E5	1	1	372	395	Deamidation (NQ)
R.NALFVPHYNTNAHSIIYALR.G	N	77.97	2313.2019	20	0.4	772.0749	3	31.16	27	1603	OB4055.raw	2.7E6	2	2	409	428	
R.SPDIYNPQ(+.98)AGSLK.T	N	77.51	1389.6776	13	-0.4	695.8458	2	27.96	27	1407	OB4055.raw	1.22E5	1	1	372	384	
N.GIEETIC(+57.02)TASVK.K	N	77.10	1306.6438	12	-0.3	654.3290	2	27.67	27	1393	OB4055.raw	1.37E5	1	1	353	364	Carbamidomethylation
R.SPDIYNPQAGSLKTANDLNLLILR.W	N	75.09	2625.4126	24	0.8	876.1455	3	33.57	27	1780	OB4055.raw	9.38E4	1	1	372	395	
R.WLGLSAEYGN(+.98)LYR.N	N	73.75	1541.7513	13	-2.2	771.8812	2	33.65	27	1783	OB4055.raw	0	0	0	396	408	Deamidation (NQ)
R.SPDIYNPQAGSLK.T	N	72.90	1388.6936	13	-0.4	695.3538	2	27.38	27	1370	OB4055.raw	9.44E4	1	1	372	384	
K.TDSRPSIANLAGENSIIDN(+.98)LPEEVVANSYGLPR.E	Y	70.20	3511.7429	33	2.9	1171.5917	3	36.02	27	1933	OB4055.raw	7.82E5	2	2	478	510	Deamidation (NQ)
R.VYDEELQEGHVLVVPQN(+.98)FAVAGK.S	N	68.51	2541.2751	23	-0.6	848.0985	3	31.98	27	1660	OB4055.raw	6.03E4	1	1	443	465	
R.NALFVPHYN(+.98)TNAHSIIYALR.G	N	68.06	2314.1858	20	0.9	772.4032	3	31.89	27	1665	OB4055.raw	1.45E6	2	2	409	428	Deamidation (NQ)
R.SPDIYNPQ(+.98)AGSLKTAN(+.98)DLNLLILR.W	N	66.93	2627.3806	24	0.7	876.8014	3	34.40	27	1838	OB4055.raw	1.05E5	1	1	372	395	
R.NRSPDIYNPQAGSLKTANDLNLLILR.W	N	66.35	2895.5566	26	-0.1	724.8964	4	32.20	27	1678	OB4055.raw	0	0	0	370	395	
K.SQSDNFEYVAFKTD.S	N	63.84	1649.7209	14	0.3	825.8680	2	30.79	27	1587	OB4055.raw	3.23E4	1	1	466	479	
K.TDSRPSIAN(+.98)LAGEN(+.98)SIIDN(+.98)LPEEVVANSYGLPR.E	Y	61.16	3513.7109	33	8.3	1172.2540	3	36.60	27	1981	OB4055.raw	0	0	0	478	510	Deamidation (NQ)
K.TDSRPSIANLAGEN(+.98)SIIDN(+.98)LPEEVVANSYGLPR.E	Y	58.74	3512.7271	33	4.1	1171.9211	3	36.31	27	1963	OB4055.raw	0	0	0	478	510	Deamidation (NQ)
R.NRSPDIYNPQAGSLK.T	N	58.59	1658.8376	15	-1.3	830.4250	2	25.53	27	1270	OB4055.raw	2.35E5	2	2	370	384	
R.NALFVPHYN(+.98)TN(+.98)AHSIIYALR.G	N	58.26	2315.1699	20	-0.3	772.7303	3	32.31	27	1687	OB4055.raw	1.33E5	1	1	409	428	Deamidation (NQ)
R.SPDIYN(+.98)PQAGSLK.T	N	53.73	1389.6776	13	-0.4	695.8458	2	27.96	27	1456	OB4055.raw	1.22E5	1	1	372	384	
N.GIEETIC(+57.02)TASVKK.N	N	52.94	1434.7388	13	0.2	718.3768	2	25.91	27	1293	OB4055.raw	3.94E5	3	3	353	365	Carbamidomethylation
R.SPDIYNPQAGSLKTANDLNLLILRWLGLSAEYGNLYR.N	N	52.19	4148.1694	37	1.3	1038.0510	4	39.63	27	2149	OB4055.raw	4.13E6	1	1	372	408	
K.SQ(+.98)SDN(+.98)FEYVAFK.T	N	51.74	1435.6143	12	5.7	718.8185	2	31.21	27	1611	OB4055.raw	2.16E3	1	1	466	477	Deamidation (NQ)
K.TDSRPSIANLAGEN(+.98).S	N	50.16	1444.6793	14	0.0	723.3469	2	28.45	27	1439	OB4055.raw	3.06E4	1	1	478	491	Deamidation (NQ)
R.NRSPDIYNPQ(+.98)AGSLKTANDLNLLILR.W	N	47.56	2896.5405	26	-0.1	725.1423	4	32.56	27	1700	OB4055.raw	3.94E5	1	1	370	395	

Peptide	Uniq	-10lgP	Mass	Length	ppm	m/z	z	RT	Fraction	Scan	Source File	Area Digest 5 traka 22 kDa	#Feature	#Feature Digest 5 traka 22 kDa	Start	End	PTM
R.SPDIYNPQAGSLKTANDLN(+.98)LLILRWLGLSAEYGNLYR.N	N	46.71	4149.1533	37	4.2	1038.2999	4	44.01	27	2449	OB4055.raw	0	0	0	372	408	Deamidation (NQ)
K.TDSRPSIAN(+.98)LAGEN(+.98).S	N	46.58	1445.6633	14	-1.6	723.8378	2	29.14	27	1479	OB4055.raw	6.66E3	1	1	478	491	Deamidation (NQ)
R.NRSPDIYN(+.98)PQAGSLKTAN(+.98)DLNLLILR.W	N	45.54	2897.5247	26	1.5	725.3895	4	33.43	27	1754	OB4055.raw	9.81E4	1	1	370	395	
R.QLKNN(+.98)NPFKFFVPPSQQ(+.98)SLGAV.A	Y	45.40	2461.2642	22	4.8	821.4326	3	31.96	27	1653	OB4055.raw	9.9E4	1	1	515	536	
G.SLKTANDLNLLILRWLGLSAEYGNLYR.N	N	45.10	3105.6975	27	0.1	777.4317	4	38.47	27	2116	OB4055.raw	4.25E5	1	1	382	408	
R.SPDIYNPQ(+.98)AGSLKTANDLNLLILRWLGLSAEYGNLYR.N	N	44.86	4149.1533	37	2.6	1038.2983	4	39.73	27	2186	OB4055.raw	2.09E3	1	1	372	408	
R.VYDEELQEGHVLVVPQNFAVAGK.S	N	43.94	2540.2910	23	1.1	847.7719	3	31.62	27	1640	OB4055.raw	4.8E4	1	1	443	465	
G.SLKTANDLN(+.98)LLILRWLGLSAEYGNLYR.N	N	43.59	3106.6814	27	3.8	777.6805	4	39.50	27	2170	OB4055.raw	0	0	0	382	408	
K.T(+71.04)ANDLNLLILRWLGLSAEYGNLYR.N	N	42.77	2848.5234	24	0.4	950.5155	3	40.15	27	2219	OB4055.raw	3.7E4	1	1	385	408	Propionamide (K, X@N-term)
R.SPDIYNPQAGSLKTAN(+.98)DLNLLILRWLGLSAEYGNLYR.N	N	42.71	4149.1533	37	3.7	1038.2994	4	39.48	27	2168	OB4055.raw	0	0	0	372	408	
R.SPDIYNP(+15.99)QAGSLKTANDLNLLILRWLGLSAEYGNLYR.N	N	42.27	4164.1641	37	3.0	1042.0514	4	40.64	27	2245	OB4055.raw	0	0	0	372	408	Hydroxylation Pro
K.TDSRPSIANLAGENSIIDNLPEEVVANSYGLPREQ(+.98)AR.Q	Y	42.01	3995.9824	37	1.8	1000.0046	4	34.49	27	1843	OB4055.raw	0	0	0	478	514	
R.SPDIYN(+.98)PQAGSLKTANDLNLLILRWLGLSAEYGNLYR.N	N	41.28	4149.1533	37	1.8	1038.2975	4	43.13	27	2396	OB4055.raw	0	0	0	372	408	
R.NRSPDIYN(+.98)PQ(+.98)AGSLKTAN(+.98)DLNLLILR.W	N	41.16	2898.5085	26	6.2	725.6389	4	33.08	27	1754	OB4055.raw	1.98E5	1	1	370	395	
K.TDSRPSIANLAGEN(+.98)SIIDNLPEEVVANSYGLPREQ(+.98)AR.Q	Y	40.84	3996.9663	37	1.9	1000.2508	4	34.85	27	1867	OB4055.raw	0	0	0	478	514	
R.Q(+.98)LKN(+.98)NNPFKFFVPPSQ(+.98)Q(+.98)SLGAV.A	Y	40.57	2463.2322	22	6.7	822.0902	3	32.66	27	1713	OB4055.raw	4.12E5	1	1	515	536	Deamidation (NQ)
R.SPDIYNPQ(+.98)AGSLKTANDLN(+.98)LLILRWLGLSAEYGNLYR.N	N	40.27	4150.1372	37	7.8	1038.5497	4	44.34	27	2469	OB4055.raw	7.17E3	1	1	372	408	
R.NALFVPHYNTN(+.98).A	N	39.43	1289.6040	11	0.5	645.8096	2	29.63	27	1510	OB4055.raw	8.74E3	1	1	409	419	
R.QLKN(+.98)N(+.98)N(+.98)PFKFFVPPSQ(+.98)Q(+.98)SLGAV.A	Y	39.06	2464.2161	22	5.2	822.4169	3	33.25	27	1753	OB4055.raw	0	0	0	515	536	Deamidation (NQ)
R.NRSPDIYN(+.98)PQ(+.98)AGSLKTANDLNLLILR.W	N	38.72	2897.5247	26	1.5	725.3895	4	33.43	27	1773	OB4055.raw	9.81E4	1	1	370	395	
R.QLKNN(+.98)NPFKFFVPPSQ(+.98)Q(+.98)SLGAV.A	Y	37.57	2462.2480	22	4.9	821.7607	3	32.66	27	1676	OB4055.raw	2.29E6	2	2	515	536	Deamidation (NQ)
R.QLKN(+.98)NN(+.98)PFKFFVPPSQ(+.98)QSLGAV.A	Y	36.92	2462.2480	22	4.9	821.7607	3	32.66	27	1695	OB4055.raw	3.18E5	1	1	515	536	
R.SPDIYNPQAGSLKTAN(+.98)DLN(+.98)LLILRWLGLSAEYGNLYR.N	N	36.34	4150.1372	37	7.8	1038.5497	4	43.61	27	2425	OB4055.raw	7.17E3	1	1	372	408	
R.NRSPDIYNPQAGSLKTAN(+.98)DLN(+.98)LLILR.W	N	35.98	2897.5247	26	1.5	725.3895	4	33.43	27	1763	OB4055.raw	9.81E4	1	1	370	395	
K.TANDLNLLILRW(+15.99)LGLSAEYGNLYR.N	N	35.89	2793.4812	24	8.6	932.1757	3	44.65	27	2481	OB4055.raw	8.59E3	1	1	385	408	Oxidation (HW)
K.TDSRPSIANLAGENSIIDN(+.98)LPEEVVANSYGLPREQ(+.98)AR.Q	Y	35.63	3996.9663	37	1.9	1000.2508	4	34.86	27	1868	OB4055.raw	0	0	0	478	514	
K.TDSRPSIANLAGEN(+.98)SIIDNLPEEVVAN(+.98)SYGLPREQAR.Q	Y	35.27	3996.9663	37	0.9	1000.2498	4	35.90	27	1937	OB4055.raw	0	0	0	478	514	
R.QLKNNNPFKFFVPPSQQ(+.98)SLGAV.A	Y	35.02	2460.2800	22	3.9	821.1038	3	31.42	27	1625	OB4055.raw	0	0	0	515	536	
K.TDSRPSIANLAGENSIIDNLPEEVVANSYGLPREQAR.Q	Y	33.30	3994.9983	37	1.0	999.7578	4	34.48	27	1831	OB4055.raw	8.29E5	1	1	478	514	

Peptide	Uniq	-10lgP	Mass	Length	ppm	m/z	z	RT	Fraction	Scan	Source File	Area Digest 5 traka 22 kDa	#Feature	#Feature Digest 5 traka 22 kDa	Start	End	PTM
N.GIEETIC(+57.02)TASVKKNIGR.N	N	31.74	1874.9884	17	-0.2	469.7543	4	26.50	27	1347	OB4055.raw	5.22E5	2	2	353	369	Carbamidomethylation
K.SQSDNFEYVAFKTDSRPSIANLAGENSIIDN(+.98)LPEEVVANSYGLPR.E	Y	31.65	4927.3789	45	3.5	1232.8563	4	35.92	27	1924	OB4055.raw	3.3E5	1	1	466	510	
R.N(+.98)RSPDIYN(+.98)PQAGSLKTANDLN(+.98)LLILR.W	N	31.43	2898.5085	26	6.2	725.6389	4	33.08	27	1763	OB4055.raw	1.98E5	1	1	370	395	Deamidation (NQ)
R.WLGLSAEYGNLYRNALFVPHYN(+.98)TN(+.98)AHSIIYALR.G	N	30.70	3837.9268	33	4.2	768.5958	5	35.09	27	1913	OB4055.raw	1.63E5	1	1	396	428	
R.SPDIYNPQ(+.98)AGSLKTAN(+.98)DLNLLILRWLGLSAEYGNLYR.N	N	30.15	4150.1372	37	5.3	1038.5471	4	42.52	27	2360	OB4055.raw	7.17E3	1	1	372	408	
R.NALFVPHYNTN(+.98)AHSIIYALRGR.A	N	29.99	2527.3083	22	0.0	843.4434	3	31.16	27	1609	OB4055.raw	4.64E4	1	1	409	430	Deamidation (NQ)
K.TANDLNLLILRWLGLSAEYGN(+.98)LYR.N	N	29.79	2778.4705	24	6.3	927.1699	3	50.07	27	2826	OB4055.raw	4.35E3	1	1	385	408	
K.TDSRPSIAN(+.98)LAGEN(+.98)SIIDNLPEEVVANSYGLPR.E	Y	29.71	3512.7271	33	4.6	1171.9216	3	35.00	27	1863	OB4055.raw	1.01E4	1	1	478	510	Deamidation (NQ)
R.WLGLSAEYGN(+.98)LYRNALFVPHYN(+.98)TN(+.98)AHSIIYALR.G	N	29.39	3838.9106	33	6.1	768.7941	5	35.52	27	1913	OB4055.raw	0	0	0	396	428	
R.SPDIYN(+.98)PQAGSLKTAN(+.98)DLNLLILRWLGLSAEYGNLYR.N	N	28.95	4150.1372	37	6.3	1384.3950	3	39.63	27	2213	OB4055.raw	1.07E5	1	1	372	408	
R.WLGLSAEYGNLYRN(+.98)ALFVPHYNTN(+.98)AHSIIYALR.G	N	28.44	3837.9268	33	4.2	768.5958	5	35.09	27	1907	OB4055.raw	1.63E5	1	1	396	428	
G.SLKTAN(+.98)DLNLLILRWLGLSAEYGNLYR.N	N	28.33	3106.6814	27	3.8	777.6806	4	41.37	27	2290	OB4055.raw	0	0	0	382	408	
K.TDSRPSIANLAGENSIIDN(+.98)LPEEVVAN(+.98).S	N	28.08	2839.3723	27	3.1	947.4677	3	35.64	27	1904	OB4055.raw	3.6E4	1	1	478	504	
R.SPDIYN(+.98)PQ(+.98)AGSLKTANDLNLLILRWLGLSAEYGNLYR.N	N	27.98	4150.1372	37	7.3	1038.5492	4	45.23	27	2522	OB4055.raw	0	0	0	372	408	
R.NALFVPHYNTNAHSIIYALRG.R	N	27.77	2370.2231	21	1.4	791.0828	3	31.62	27	1624	OB4055.raw	8.89E4	1	1	409	429	
K.TDSRPSIAN(+.98)LAGENSIIDN(+.98)LPEEVVANSYGLPR.E	Y	27.62	3512.7271	33	6.1	879.1944	4	36.08	27	1948	OB4055.raw	0	0	0	478	510	
K.TDSRPSIAN(+.98)LAGENSIIDNLPEEVVANSYGLPR.E	Y	27.58	3511.7429	33	3.9	878.9464	4	35.83	27	1948	OB4055.raw	1.63E5	1	1	478	510	
R.WLGLSAEYGNLYRN(+.98)ALFVPHYN(+.98)TN(+.98)AHSIIYALR.G	N	27.29	3838.9106	33	5.2	768.7934	5	35.43	27	1907	OB4055.raw	0	0	0	396	428	
K.NIGRNRSPDIYN(+.98)PQAGSLK.T	N	25.83	2100.0710	19	0.2	701.0311	3	25.59	27	1264	OB4055.raw	0	0	0	366	384	
K.TANDLNLLILR.W	N	25.78	1254.7296	11	-0.8	628.3716	2	33.40	27	1789	OB4055.raw	2.37E5	1	1	385	395	
R.SPDIYN(+.98)PQAGSLKTANDLN(+.98)LLILRWLGLSAEYGNLYR.N	N	25.56	4150.1372	37	8.4	1038.5503	4	48.41	27	2712	OB4055.raw	7.17E3	1	1	372	408	
K.TDSRPSIANLAGEN(+.98)SIIDNLPEEVVAN(+.98)SYGLPR.E	Y	25.50	3512.7271	33	0.6	879.1896	4	36.17	27	1954	OB4055.raw	0	0	0	478	510	
A.GSLKTAN(+.98)DLNLLILRWLGLSAEYGNLYR.N	N	25.38	3163.7029	28	2.0	791.9346	4	39.16	27	2147	OB4055.raw	0	0	0	381	408	
K.N(+.98)IGRNRSPDIYNPQAGSLK.T	N	24.58	2100.0710	19	0.1	701.0310	3	25.67	27	1269	OB4055.raw	0	0	0	366	384	
R.QLKNN(+.98)N(+.98)PFKFFVPPSQ(+.98)Q(+.98)SLGAV.A	Y	24.46	2463.2322	22	7.9	616.8202	4	32.67	27	1711	OB4055.raw	0	0	0	515	536	Deamidation (NQ)
R.NALFVPHYN(+.98)TNAHSIIYALRGR.A	N	24.45	2527.3083	22	0.4	632.8346	4	31.16	27	1597	OB4055.raw	1.55E6	1	1	409	430	
R.Q(-17.03)LKNN(+.98)NPFKFFVPPSQQ(+.98)SLGAV.A	Y	22.49	2444.2375	22	6.6	815.7585	3	33.48	27	1791	OB4055.raw	1.15E5	1	1	515	536	Pyro-glu from Q
R.AHVQVVDSN(+.98)GNRVYDEELQEGHVLVVPQNFAVAGK.S	N	22.21	3817.9023	35	-1.0	955.4819	4	31.24	27	1613	OB4055.raw	0	0	0	431	465	
R.AHVQVVDSN(+.98)GNRVYDEELQ(+.98)EGHVLVVPQ(+.98)NFAVAGK.S	N	22.19	3819.8704	35	5.4	764.9855	5	31.35	27	1643	OB4055.raw	1.55E5	1	1	431	465	

Peptide	Uniq	-10lgP	Mass	Length	ppm	m/z	z	RT	Fraction	Scan	Source File	Area Digest 5 traka 22 kDa	#Feature	#Feature Digest 5 traka 22 kDa	Start	End	PTM
K.TDSRPSIANLAGEN(+.98)SIIDNLPEEVVANSYGLPR.E	Y	22.08	3511.7429	33	3.9	878.9464	4	35.83	27	1954	OB4055.raw	1.63E5	1	1	478	510	
R.AHVQVVDSNGN(+.98)RVYDEELQ(+.98)EGHVLVVPQNFAVAGK.S	N	21.90	3818.8862	35	1.8	764.7859	5	31.36	27	1622	OB4055.raw	0	0	0	431	465	
**total 89 peptides**																	

tr|A1DZF0|A1DZF0_ARAHY

back to list

| Protein Coverage | Supporting Peptides |

Protein Coverage:

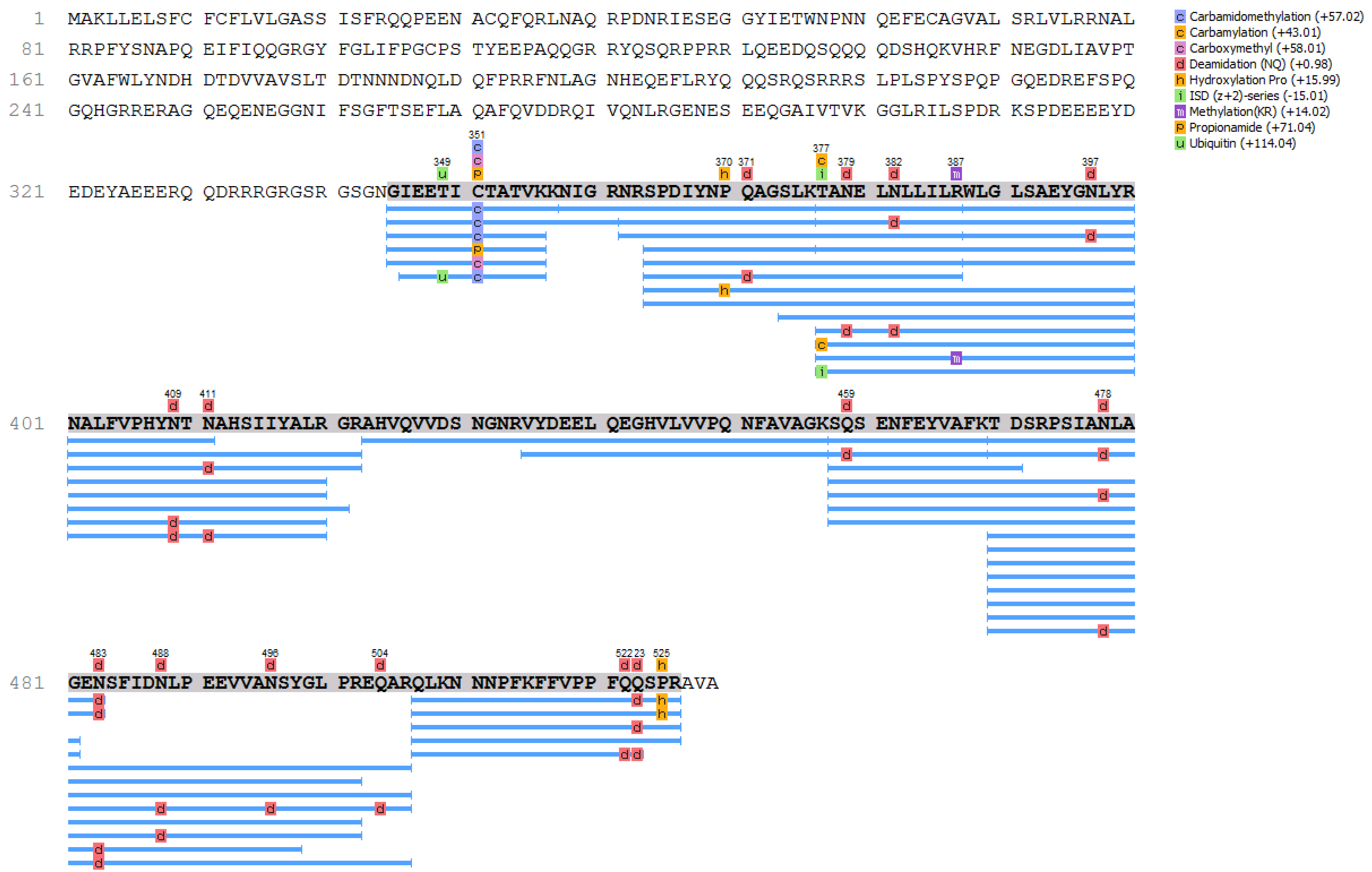
1 MAKLLELSFC FCFLVLGASS ISFRQQPEEN ACQFQRLNAQ RPDNRIESEG GYIETWNPNN QEFECAGVAL SRLVLRRNAL
81 RRPFYSNAPQ EIFIQQGRGY FGLIFPGCPS TYEEPAQQGR RYQSQRPPRR LQEEDQSQQQ QDSHQKVHRF NEGDLIAVPT
161 GVAFWLYNDH DTDVVAVSLT DTNNNDNQLD QFPRRFNLAG NHEQEFLRYQ QQSRQSRRRS LPLSPYSPQP GQEDREFSPQ
241 GQHGRRERAG QEQENEGGNI FSGFTSEFLA QAFQVDDRQI VQNLRGENES EEQGAIVTVK GGLRILSPDR KSPDEEEEYD
321 EDEYAEEERQ QDRRRGRGSR GSGNGIEETI CTATVKKNIG RNRSPDIYNP QAGSLKTANE LNLLILRWLG LSAEYGNLYR
401 NALFVPHYNT NAHSIIYALR GRAHVQVVDS NGNRVYDEEL QEGHVLVVPQ NFAVAGKSQS ENFEYVAFKT DSRPSIANLA
481 GENSFIDNLP EEVVANSYGL PREQARQLKN NNPFKFFVPP FQQSPRAVA
c Carbamidomethylation (+57.02)
c Carbamylation (+43.01)
c Carboxymethyl (+58.01)
d Deamidation (NQ) (+0.98)
h Hydroxylation Pro (+15.99)
i ISD (z+2)-series (-15.01)
m Methylation(KR) (+14.02)
P Propionamide (+71.04)
u Ubiquitin (+114.04)

Supporting Peptides:

Peptide	Uniq	-10lgP	Mass	Length	ppm	m/z	z	RT	Fraction	Scan	Source File	Area Digest 5 traka 22 kDa	#Feature	#Feature Digest 5 traka 22 kDa	Start	End	PTM
R.WLGLSAEYGNLYR.N	N	98.82	1540.7673	13	0.1	771.3911	2	33.33	27	1749	OB4055.raw	1.54E6	1	1	388	400	
K.TANELNLLILRWLGLSAEYGNLYR.N	N	90.49	2791.5020	24	1.1	931.5090	3	39.71	27	2150	OB4055.raw	2.13E6	1	1	377	400	
K.SQSENFEYVAFK.T	N	89.61	1447.6619	12	0.4	724.8385	2	30.22	27	1543	OB4055.raw	2.25E5	1	1	458	469	
R.SPDIYNPQ(+.98)AGSLKTANELNLLILR.W	N	83.05	2640.4121	24	0.3	881.1449	3	34.30	27	1825	OB4055.raw	2.21E5	1	1	364	387	
K.TAN(+.98)ELNLLILRWLGLSAEYGNLYR.N	N	79.11	2792.4861	24	5.9	931.8415	3	40.73	27	2251	OB4055.raw	1.4E4	1	1	377	400	
K.TDSRPSIANLAGENSFIDNLPEEVVANSYGLPR.E	N	78.92	3544.7434	33	1.0	1182.5896	3	36.12	27	1936	OB4055.raw	5.39E5	1	1	470	502	
R.NALFVPHYNTNAHSIIYALR.G	N	77.97	2313.2019	20	0.4	772.0749	3	31.16	27	1603	OB4055.raw	2.7E6	2	2	401	420	
N.GIEETIC(+57.02)TATVK.K	N	77.66	1320.6595	12	0.7	661.3375	2	28.16	27	1418	OB4055.raw	1.3E5	1	1	345	356	Carbamidomethylation
R.SPDIYNPQ(+.98)AGSLK.T	N	77.51	1389.6776	13	-0.4	695.8458	2	27.96	27	1407	OB4055.raw	1.22E5	1	1	364	376	
K.TANELN(+.98)LLILRWLGLSAEYGNLYR.N	N	77.43	2792.4861	24	3.4	931.8391	3	40.54	27	2239	OB4055.raw	1.4E4	1	1	377	400	Deamidation (NQ)
R.WLGLSAEYGN(+.98)LYR.N	N	73.75	1541.7513	13	-2.2	771.8812	2	33.65	27	1783	OB4055.raw	0	0	0	388	400	Deamidation (NQ)
K.SQ(+.98)SENFEYVAFK.T	N	73.36	1448.6459	12	-0.8	725.3297	2	30.52	27	1573	OB4055.raw	1.12E5	1	1	458	469	Deamidation (NQ)
R.SPDIYNPQAGSLK.T	N	72.90	1388.6936	13	-0.4	695.3538	2	27.38	27	1370	OB4055.raw	9.44E4	1	1	364	376	
K.TDSRPSIANLAGENSFIDN(+.98)LPEEVVANSYGLPR.E	N	70.54	3545.7273	33	2.3	1182.9191	3	36.12	27	1951	OB4055.raw	0	0	0	470	502	Deamidation (NQ)
R.SPDIYN(+.98)PQ(+.98)AGSLKTANELNLLILR.W	N	68.90	2641.3962	24	1.0	881.4736	3	34.66	27	1851	OB4055.raw	8.13E4	1	1	364	387	Deamidation (NQ)
R.VYDEELQEGHVLVVPQN(+.98)FAVAGK.S	N	68.51	2541.2751	23	-0.6	848.0985	3	31.98	27	1660	OB4055.raw	6.03E4	1	1	435	457	
R.NALFVPHYN(+.98)TNAHSIIYALR.G	N	68.06	2314.1858	20	0.9	772.4032	3	31.89	27	1665	OB4055.raw	1.45E6	2	2	401	420	Deamidation (NQ)
K.TDSRPSIANLAGEN(+.98)SFIDN(+.98)LPEEVVANSYGLPR.E	N	65.76	3546.7114	33	4.7	1183.2500	3	36.50	27	1975	OB4055.raw	0	0	0	470	502	
R.SPDIYN(+.98)PQAGSLKTANELNLLILRWLGLSAEYGNLYR.N	N	65.10	4163.1689	37	3.1	1041.8027	4	39.91	27	2198	OB4055.raw	4.68E4	1	1	364	400	
R.SPDIYNPQAGSLKTANELNLLILR.W	N	63.60	2639.4282	24	-0.1	880.8166	3	33.89	27	1804	OB4055.raw	9.02E4	1	1	364	387	
K.TDSRPSIANLAGEN(+.98)SFIDNLPEEVVANSYGLPR.E	N	63.54	3545.7273	33	5.8	1182.9232	3	37.11	27	2013	OB4055.raw	0	0	0	470	502	
R.SPDIYNPQAGSLKTANELN(+.98)LLILRWLGLSAEYGNLYR.N	N	61.94	4163.1689	37	4.6	1041.8043	4	39.65	27	2180	OB4055.raw	4.68E4	1	1	364	400	
R.SPDIYNPQAGSLKTANELNLLILRWLGLSAEYGNLYR.N	N	61.02	4162.1851	37	1.6	1041.5552	4	39.79	27	2169	OB4055.raw	3.44E6	1	1	364	400	
R.NRSPDIYNPQAGSLK.T	N	58.59	1658.8376	15	-1.3	830.4250	2	25.53	27	1270	OB4055.raw	2.35E5	2	2	362	376	
R.NALFVPHYN(+.98)TN(+.98)AHSIIYALR.G	N	58.26	2315.1699	20	-0.3	772.7303	3	32.31	27	1687	OB4055.raw	1.33E5	1	1	401	420	Deamidation (NQ)
R.NRSPDIYNPQ(+.98)AGSLKTANELNLLILR.W	N	58.23	2910.5562	26	0.9	728.6470	4	32.90	27	1723	OB4055.raw	3.88E5	1	1	362	387	
K.TAN(+.98)ELN(+.98)LLILRWLGLSAEYGNLYR.N	N	57.65	2793.4700	24	7.2	932.1707	3	40.44	27	2228	OB4055.raw	1.72E5	1	1	377	400	Deamidation (NQ)

Peptide	Uniq	-10lgP	Mass	Length	ppm	m/z	z	RT	Fraction	Scan	Source File	Area Digest 5 traka 22 kDa	#Feature	#Feature Digest 5 traka 22 kDa	Start	End	PTM
R.NRSPDIYN(+.98)PQ(+.98)AGSLKTANELNLLILR.W	N	56.64	2911.5403	26	0.7	728.8928	4	33.24	27	1779	OB4055.raw	2.18E5	1	1	362	387	
K.SQSENFEYVAFKTD.S	N	55.46	1663.7366	14	0.2	832.8757	2	30.70	27	1581	OB4055.raw	4.71E4	1	1	458	471	
K.TDSRPSIAN(+.98)LAGEN(+.98)SFIDN(+.98)LPEEVVANSYGLPR.E	N	55.41	3547.6953	33	-3.4	1183.5684	3	36.99	27	2005	OB4055.raw	0	0	0	470	502	Deamidation (NQ)
R.NRSPDIYNPQAGSLKTAN(+.98)ELNLLILR.W	N	55.23	2910.5562	26	0.9	728.6470	4	32.90	27	1742	OB4055.raw	3.88E5	1	1	362	387	
R.SPDIYN(+.98)PQAGSLK.T	N	53.73	1389.6776	13	-0.4	695.8458	2	27.96	27	1456	OB4055.raw	1.22E5	1	1	364	376	
K.T(+43.01)ANELNLLILRWLGLSAEYGNLYR.N	N	53.67	2834.5078	24	0.1	945.8433	3	39.96	27	2203	OB4055.raw	7.92E4	1	1	377	400	Carbamylation
R.NRSPDIYNPQAGSLKTANELNLLILR.W	N	52.49	2909.5723	26	0.7	728.4008	4	32.54	27	1703	OB4055.raw	0	0	0	362	387	
K.TDSRPSIANLAGEN(+.98).S	N	50.16	1444.6793	14	0.0	723.3469	2	28.45	27	1439	OB4055.raw	3.06E4	1	1	470	483	Deamidation (NQ)
R.SPDIYNPQ(+.98)AGSLKTANELNLLILRWLGLSAEYGNLYR.N	N	49.91	4163.1689	37	4.0	1041.8037	4	42.23	27	2342	OB4055.raw	4.68E4	1	1	364	400	
N.GIEETIC(+57.02)TATVKK.N	N	48.27	1448.7545	13	-0.4	725.3843	2	26.38	27	1317	OB4055.raw	5.13E4	1	1	345	357	Carbamidomethylation
R.SPDIYNPQAGSLKTAN(+.98)ELNLLILRWLGLSAEYGNLYR.N	N	48.23	4163.1689	37	6.0	1041.8058	4	47.15	27	2617	OB4055.raw	4.68E4	1	1	364	400	
K.TDSRPSIAN(+.98)LAGEN(+.98).S	N	46.58	1445.6633	14	-1.6	723.8378	2	29.14	27	1479	OB4055.raw	6.66E3	1	1	470	483	Deamidation (NQ)
N.GIEETIC(+71.04)TATVK.K	N	46.25	1334.6752	12	1.0	668.3455	2	28.36	27	1436	OB4055.raw	3.51E4	1	1	345	356	Propionamide
G.SLKTANELN(+.98)LLILRWLGLSAEYGNLYR.N	N	44.57	3120.6970	27	1.6	781.1828	4	39.10	27	2143	OB4055.raw	0	0	0	374	400	
R.VYDEELQEGHVLVVPQNFAVAGK.S	N	43.94	2540.2910	23	1.1	847.7719	3	31.62	27	1640	OB4055.raw	4.8E4	1	1	435	457	
R.QLKNN(+.98)N(+.98)PFKFFVPPFQ(+.98)Q(+.98)SP(+15.99)R.A	Y	41.91	2452.2063	20	-3.0	614.0570	4	33.80	27	1781	OB4055.raw	1.39E5	1	1	507	526	Deamidation (NQ); Hydroxylation Pro
R.Q(+.98)LKNN(+.98)NPFKFFVPPFQ(+.98)Q(+.98)SPR.A	Y	41.72	2436.2114	20	1.6	610.0611	4	33.89	27	1807	OB4055.raw	5.2E5	1	1	507	526	Deamidation (NQ)
K.TDSRPSIANLAGEN(+.98)SFIDNLPEEVVANSYGLPREQAR.Q	N	40.05	4029.9668	37	4.1	1008.5031	4	36.96	27	2003	OB4055.raw	0	0	0	470	506	Deamidation (NQ)
K.SQSENFEYVAFKTDSRPSIANLAG.E	N	39.51	2630.2612	24	0.5	877.7615	3	31.62	27	1639	OB4055.raw	6.96E4	1	1	458	481	
R.NALFVPHYNTN(+.98).A	N	39.43	1289.6040	11	0.5	645.8096	2	29.63	27	1510	OB4055.raw	8.74E3	1	1	401	411	
K.TDSRPSIANLAGENSFIDNLPEEVVANSYGLPREQAR.Q	N	39.23	4028.9827	37	1.2	1008.2542	4	34.76	27	1849	OB4055.raw	1.21E6	1	1	470	506	
K.TANELNLLILR.W	N	38.99	1268.7452	11	-0.3	635.3797	2	33.64	27	1785	OB4055.raw	2.08E5	1	1	377	387	
G.SLKTANELNLLILRWLGLSAEYGNLYR.N	N	38.88	3119.7131	27	-0.5	780.9352	4	38.55	27	2104	OB4055.raw	1.85E5	1	1	374	400	
R.Q(+.98)LKN(+.98)NNPFKFFVPPFQ(+.98)Q(+.98)SPR.A	Y	38.08	2436.2114	20	1.6	610.0611	4	33.89	27	1892	OB4055.raw	5.2E5	1	1	507	526	
R.Q(+.98)LKN(+.98)NN(+.98)PFKFFVPPFQ(+.98)Q(+.98)SPR.A	Y	37.77	2437.1953	20	1.4	610.3069	4	34.48	27	1855	OB4055.raw	5.17E4	1	1	507	526	Deamidation (NQ)
N.GIEETIC(+58.01)TATVK.K	N	37.75	1321.6436	12	0.6	661.8295	2	28.75	27	1459	OB4055.raw	1.22E4	1	1	345	356	Carboxymethyl
K.TANELN(+.98)LLILR(+14.02)WLGLSAEYGNLYR.N	N	37.66	2806.5017	24	0.4	936.5082	3	40.72	27	2260	OB4055.raw	1.57E4	1	1	377	400	Methylation(KR)

Peptide	Uniq	-10lgP	Mass	Length	ppm	m/z	z	RT	Fraction	Scan	Source File	Area Digest 5 traka 22 kDa	#Feature	#Feature Digest 5 traka 22 kDa	Start	End	PTM
K.TDSRPSIANLAGEN(+.98)SFIDNLPEEVVAN(+.98)SYGLPREQ(+.98)AR.Q	N	37.47	4031.9348	37	7.8	1008.9988	4	34.84	27	2003	OB4055.raw	2.53E6	1	1	470	506	
R.QLKNNNPFKFFVPPFQ(+.98)Q(+.98)SPR.A	Y	37.32	2434.2432	20	-1.0	609.5674	4	33.24	27	1807	OB4055.raw	3.8E5	1	1	507	526	
R.SPDIYN(+.98)PQAGSLKTAN(+.98)ELNLLILRWLGLSAEYGNLYR.N	N	36.79	4164.1528	37	7.2	1042.0530	4	44.53	27	2480	OB4055.raw	0	0	0	364	400	
K.TDSRPSIANLAGEN(+.98)SFIDNLPEEVVANSYGLPREQ(+.98)AR.Q	N	36.60	4030.9507	37	3.3	1008.7483	4	36.71	27	1988	OB4055.raw	0	0	0	470	506	
K.TDSRPSIANLAGEN(+.98)SFIDN(+.98)LPEEVVANSYGLPREQ(+.98)AR.Q	N	36.33	4031.9348	37	7.8	1008.9988	4	34.84	27	1885	OB4055.raw	2.53E6	1	1	470	506	
K.TDSRPSIANLAGENSFIDNLPEEVVANSYGLPREQ(+.98)AR.Q	N	35.87	4029.9668	37	2.2	1008.5012	4	34.77	27	1861	OB4055.raw	0	0	0	470	506	
K.TDSRPSIANLAGENSFIDN(+.98)LPEEVVANSYGLPREQ(+.98)AR.Q	N	35.49	4030.9507	37	2.8	1008.7477	4	35.10	27	1885	OB4055.raw	0	0	0	470	506	
R.SPDIYN(+.98)PQ(+.98)AGSLKTANELNLLILRWLGLSAEYGNLYR.N	N	33.97	4164.1528	37	8.4	1042.0542	4	45.83	27	2558	OB4055.raw	0	0	0	364	400	
K.TANELNLLILR(+14.02)WLGLSAEYGNLYR.N	N	33.37	2805.5176	24	2.2	936.1819	3	40.25	27	2216	OB4055.raw	6.97E4	1	1	377	400	Methylation(KR)
R.QLKNN(+.98)N(+.98)PFKFFVPPFQQ(+.98)SPR.A	Y	33.21	2435.2273	20	1.3	609.8149	4	33.34	27	1760	OB4055.raw	0	0	0	507	526	
K.TDSRPSIAN(+.98)LAGENSFIDN(+.98)LPEEVVANSYGLPREQAR.Q	N	33.01	4030.9507	37	1.0	1008.7460	4	37.29	27	2024	OB4055.raw	0	0	0	470	506	
R.SPDIYNP(+15.99)Q(+.98)AGSLKTANELNLLILRWLGLSAEYGNLYR.N	N	32.18	4179.1641	37	0.5	1045.7988	4	41.39	27	2291	OB4055.raw	0	0	0	364	400	Hydroxylation Pro
K.TDSRPSIANLAGEN(+.98)SFIDNLPEEVVANSY.G	N	32.14	3122.4680	29	1.3	1041.8313	3	36.49	27	1985	OB4055.raw	8.84E3	1	1	470	498	Deamidation (NQ)
R.Q(+.98)LKN(+.98)NN(+.98)PFKFFVPPFQQ(+.98)SPR.A	Y	31.82	2436.2114	20	1.6	610.0611	4	33.89	27	1795	OB4055.raw	5.2E5	1	1	507	526	
K.TDSRPSIAN(+.98)LAGEN(+.98)SFIDN(+.98)LPEEVVANSYGLPREQAR.Q	N	31.53	4031.9348	37	7.8	1008.9988	4	34.84	27	2024	OB4055.raw	2.53E6	1	1	470	506	
G.SLKTAN(+.98)ELN(+.98)LLILRWLGLSAEYGNLYR.N	N	31.03	3121.6812	27	3.8	1041.5717	3	39.09	27	2146	OB4055.raw	1.61E4	1	1	374	400	
R.QLKN(+.98)N(+.98)NPFKFFVPPFQ(+.98)Q(+.98)SP(+15.99)R.A	Y	31.02	2452.2063	20	-3.0	614.0570	4	33.80	27	1797	OB4055.raw	1.39E5	1	1	507	526	Hydroxylation Pro
R.WLGLSAEYGNLYRNALFVPHYN(+.98)TN(+.98)AHSIIYALR.G	N	30.70	3837.9268	33	4.2	768.5958	5	35.09	27	1913	OB4055.raw	1.63E5	1	1	388	420	
K.TDSRPSIAN(+.98)LAGENSFIDNLPEEVVAN(+.98)SYGLPR.E	N	30.44	3546.7114	33	1.3	887.6863	4	36.40	27	1979	OB4055.raw	7.87E4	1	1	470	502	
R.NALFVPHYNTN(+.98)AHSIIYALRGR.A	N	29.99	2527.3083	22	0.0	843.4434	3	31.16	27	1609	OB4055.raw	4.64E4	1	1	401	422	Deamidation (NQ)
K.TANELN(+.98)LLILR.W	N	29.92	1269.7292	11	1.0	635.8726	2	34.22	27	1822	OB4055.raw	6.28E4	1	1	377	387	
R.SPDIYNP(+15.99)QAGSLKTANELNLLILRWLGLSAEYGNLYR.N	N	29.68	4178.1797	37	2.6	1045.5549	4	40.15	27	2159	OB4055.raw	1.36E5	1	1	364	400	Hydroxylation Pro
R.Q(+.98)LKNNN(+.98)PFKFFVPPFQ(+.98)Q(+.98)SPR.A	Y	29.55	2436.2114	20	1.6	610.0611	4	33.89	27	1918	OB4055.raw	5.2E5	1	1	507	526	
R.WLGLSAEYGN(+.98)LYRNALFVPHYN(+.98)TN(+.98)AHSIIYALR.G	N	29.39	3838.9106	33	6.1	768.7941	5	35.52	27	1913	OB4055.raw	0	0	0	388	420	
R.WLGLSAEYGNLYRN(+.98)ALFVPHYNTN(+.98)AHSIIYALR.G	N	28.44	3837.9268	33	4.2	768.5958	5	35.09	27	1907	OB4055.raw	1.63E5	1	1	388	420	
R.QLKNN(+.98)NPFKFFVPPFQQ(+.98)SPR.A	Y	28.41	2434.2432	20	-1.0	609.5674	4	33.24	27	1760	OB4055.raw	3.8E5	1	1	507	526	
R.NALFVPHYNTNAHSIIYALRG.R	N	27.77	2370.2231	21	1.4	791.0828	3	31.62	27	1624	OB4055.raw	8.89E4	1	1	401	421	
R.QLKNNN(+.98)PFKFFVPPFQQ(+.98)SPR.A	Y	27.58	2434.2432	20	-1.0	609.5674	4	33.24	27	1777	OB4055.raw	3.8E5	1	1	507	526	

Peptide	Uniq	-10lgP	Mass	Length	ppm	m/z	z	RT	Fraction	Scan	Source File	Area Digest 5 traka 22 kDa	#Feature	#Feature Digest 5 traka 22 kDa	Start	End	PTM
R.WLGLSAEYGNLYRN(+.98)ALFVPHYN(+.98)TN(+.98)AHSIIYALR.G	N	27.29	3838.9106	33	5.2	768.7934	5	35.43	27	1907	OB4055.raw	0	0	0	388	420	
G.IEET(+114.04)IC(+57.02)TATVK.K	N	27.03	1377.6810	11	0.7	689.8483	2	28.26	27	1432	OB4055.raw	2.47E4	1	1	346	356	Ubiquitin; Carbamidomethylation
K.SQSENFEYVAFKTDSRPSIANLAGEN(+.98)SFIDNLPEEVVANSYGLPR.E	N	26.57	4975.3789	45	4.6	1244.8577	4	35.96	27	1941	OB4055.raw	0	0	0	458	502	
R.SPDIYNPQ(+.98)AGSLKTANELN(+.98)LLILRWLGLSAEYGNLYR.N	N	26.55	4164.1528	37	2.5	1042.0481	4	47.88	27	2681	OB4055.raw	0	0	0	364	400	
K.SQSENFEYVAFKTDSRPSIAN(+.98)LAG.E	N	26.38	2631.2451	24	1.4	878.0902	3	32.06	27	1672	OB4055.raw	4.56E4	1	1	458	481	Deamidation (NQ)
K.T(+14.02)ANELNLLILRWLGLSAEYGNLYR.N	N	26.25	2805.5176	24	1.2	936.1810	3	40.59	27	2242	OB4055.raw	0	0	0	377	400	
N.GIEETIC(+57.02)TATVKKNIGR.N	N	26.11	1889.0040	17	-0.6	473.2580	4	26.89	27	1346	OB4055.raw	2.81E5	1	1	345	361	Carbamidomethylation
K.TDSRPSIANLAGENSFIDN(+.98)LPEEVVAN(+.98)SYGLPREQ(+.98)AR.Q	N	26.09	4031.9348	37	7.8	1008.9988	4	34.84	27	1849	OB4055.raw	2.53E6	1	1	470	506	Deamidation (NQ)
K.NIGRNRSPDIYN(+.98)PQAGSLK.T	N	25.83	2100.0710	19	0.2	701.0311	3	25.59	27	1264	OB4055.raw	0	0	0	358	376	
R.Q(+.98)LKNNNPFKFFVPPFQ(+.98)Q(+.98).S	Y	25.30	2095.0415	17	-0.3	699.3542	3	35.17	27	1898	OB4055.raw	1.51E5	1	1	507	523	Deamidation (NQ)
R.SPDIYNP(+15.99)Q(+.98)AGSLKTAN(+.98)ELNLLILRWLGLSAEYGNLYR.N	N	25.16	4180.1479	37	4.7	1046.0492	4	42.38	27	2351	OB4055.raw	0	0	0	364	400	Hydroxylation Pro
K.TDSRPSIANLAGEN(+.98)SFIDN(+.98)LPEEVVANSY.G	N	24.92	3123.4519	29	5.2	1042.1633	3	36.99	27	1985	OB4055.raw	2.77E4	1	1	470	498	
R.Q(+.98)LKNN(+.98)N(+.98)PFKFFVPPFQ(+.98)Q(+.98)SPR.A	Y	24.89	2437.1953	20	1.4	610.3069	4	34.48	27	1844	OB4055.raw	5.17E4	1	1	507	526	
K.T(-15.01)ANELNLLILRWLGLSAEYGNLYR.N	N	24.82	2776.4912	24	-3.4	926.5012	3	46.65	27	2607	OB4055.raw	0	0	0	377	400	ISD (z+2)-series
K.N(+.98)IGRNRSPDIYNPQAGSLK.T	N	24.58	2100.0710	19	0.1	701.0310	3	25.67	27	1269	OB4055.raw	0	0	0	358	376	
R.NALFVPHYN(+.98)TNAHSIIYALRGR.A	N	24.45	2527.3083	22	0.4	632.8346	4	31.16	27	1597	OB4055.raw	1.55E6	1	1	401	422	
R.SPDIYNPQ(+.98)AGSLKTAN(+.98)ELN(+.98)LLILRWLGLSAEYGNLYR.N	N	23.22	4165.1372	37	3.0	1042.2947	4	49.37	27	2769	OB4055.raw	0	0	0	364	400	
G.SLKTAN(+.98)ELNLLILRWLGLSAEYGNLYR.N	N	23.19	3120.6970	27	7.3	781.1873	4	41.07	27	2272	OB4055.raw	0	0	0	374	400	
K.TDSRPSIAN(+.98)LAGEN(+.98)SFIDNLPEEVVANSYGLPR.E	N	22.42	3546.7114	33	1.3	887.6863	4	36.40	27	1971	OB4055.raw	7.87E4	1	1	470	502	
R.Q(+.98)LKN(+.98)NNPFKFFVPPFQ(+.98)Q(+.98).S	Y	22.27	2096.0254	17	-0.4	699.6821	3	35.74	27	1939	OB4055.raw	1.71E5	1	1	507	523	Deamidation (NQ)
R.AHVQVVDSN(+.98)GNRVYDEELQEGHVLVVPQNFAVAGK.S	N	22.21	3817.9023	35	-1.0	955.4819	4	31.24	27	1613	OB4055.raw	0	0	0	423	457	
R.AHVQVVDSN(+.98)GNRVYDEELQ(+.98)EGHVLVVPQ(+.98)NFAVAGK.S	N	22.19	3819.8704	35	5.4	764.9855	5	31.35	27	1643	OB4055.raw	1.55E5	1	1	423	457	
K.SQSENFEYVAFKTDSRPSIAN(+.98)LAGENSFIDNLPEEVVANSYGLPREQ(+.98)AR.Q	N	22.13	5460.6021	49	4.0	1093.1321	5	35.14	27	1888	OB4055.raw	0	0	0	458	506	
R.AHVQVVDSNGN(+.98)RVYDEELQ(+.98)EGHVLVVPQNFAVAGK.S	N	21.90	3818.8862	35	1.8	764.7859	5	31.36	27	1622	OB4055.raw	0	0	0	423	457	
**total 106 peptides**																	

tr|B5TYU1|B5TYU1_ARAHY

back to list

Protein Coverage:

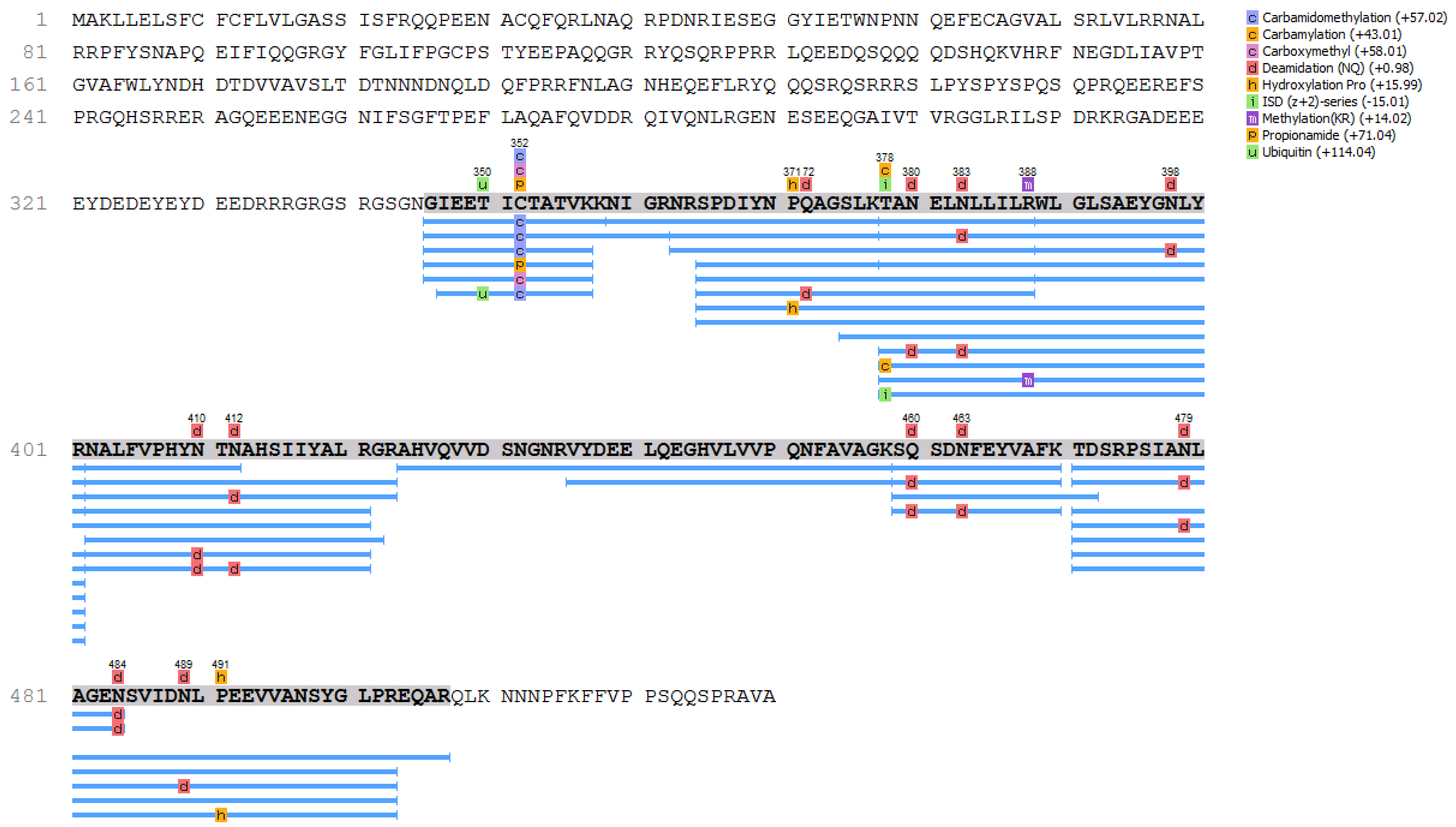
1 MAKLLELSFC FCFLVLGASS ISFRQQPEEN ACQFQRLNAQ RPDNRIESEG GYIETWNPNN QEFECAGVAL SRLVLRRNAL
81 RRPFYSNAPQ EIFIQQGRGY FGLIFPGCPS TYEEPAQQGR RYQSQRPPRR LQEEDQSQQQ QDSHQKVHRF NEGDLIAVPT
161 GVAFWLYNDH DTDVVAVSLT DTNNNDNQLD QFPRRFNLAG NHEQEFLRYQ QQSRQSRRRS LPYSPYSPQS QPRQEEREFS
241 PRGQHSRRER AGQEEENEGG NIFSGFTPEF LAQAFQVDDR QIVQNLRGEN ESEEQGAIVT VRGGLRILSP DRKRGADEEE
321 EYDEDEYEYD EEDRRRGRGS RGSGNGIEET ICTATVKKNI GRNRSPDIYN PQAGSLKTAN ELNLLILRWL GLSAEYGNLY
401 RNALFVPHYN TNAHSIIYAL RGRAHVQVVD SNGNRVYDEE LQEGHVLVVP QNFAVAGKSQ SDNFEYVAFK TDSRPSIANL
481 AGENSVIDNL PEEVVANSYG LPREQARQLK NNNPFKFFVP PSQQSPRAVA
350 352 371 372 378 380 383 388 398
410 412 460 463 479
484 489 491
Carbamidomethylation (+57.02)
Carbamylation (+43.01)
Carboxymethyl (+58.01)
Deamidation (NQ) (+0.98)
Hydroxylation Pro (+15.99)
ISD (z+2)-series (-15.01)
Methylation(KR) (+14.02)
Propionamide (+71.04)
Ubiquitin (+114.04)

Supporting Peptides:

Peptide	Uniq	-10lgP	Mass	Length	ppm	m/z	z	RT	Fraction	Scan	Source File	Area Digest 5 traka 22 kDa	#Feature	#Feature Digest 5 traka 22 kDa	Start	End	PTM
R.WLGLSAEYGNLYR.N	N	98.82	1540.7673	13	0.1	771.3911	2	33.33	27	1749	OB4055.raw	1.54E6	1	1	389	401	
K.TANELNLLILRWLGLSAEYGNLYR.N	N	90.49	2791.5020	24	1.1	931.5090	3	39.71	27	2150	OB4055.raw	2.13E6	1	1	378	401	
K.SQ(+.98)SDNFEYVAFK.T	N	89.03	1434.6302	12	2.2	718.3240	2	30.79	27	1580	OB4055.raw	6.12E4	1	1	459	470	Deamidation (NQ)
K.SQSDNFEYVAFK.T	N	88.10	1433.6462	12	1.3	717.8314	2	30.42	27	1552	OB4055.raw	1.4E5	1	1	459	470	
R.SPDIYNPQ(+.98)AGSLKTANELNLLILR.W	N	83.05	2640.4121	24	0.3	881.1449	3	34.30	27	1825	OB4055.raw	2.21E5	1	1	365	388	
K.TAN(+.98)ELNLLILRWLGLSAEYGNLYR.N	N	79.11	2792.4861	24	5.9	931.8415	3	40.73	27	2251	OB4055.raw	1.4E4	1	1	378	401	
K.TDSRPSIANLAGEN(+.98)SVIDN(+.98)LPEEVVANSYGLPR.E	Y	78.57	3498.7114	33	1.4	1167.2461	3	35.76	27	1928	OB4055.raw	0	0	0	471	503	
R.NALFVPHYNTNAHSIIYALR.G	N	77.97	2313.2019	20	0.4	772.0749	3	31.16	27	1603	OB4055.raw	2.7E6	2	2	402	421	
N.GIEETIC(+57.02)TATVK.K	N	77.66	1320.6595	12	0.7	661.3375	2	28.16	27	1418	OB4055.raw	1.3E5	1	1	346	357	Carbamidomethylation
R.SPDIYNPQ(+.98)AGSLK.T	N	77.51	1389.6776	13	-0.4	695.8458	2	27.96	27	1407	OB4055.raw	1.22E5	1	1	365	377	
K.TANELN(+.98)LLILRWLGLSAEYGNLYR.N	N	77.43	2792.4861	24	3.4	931.8391	3	40.54	27	2239	OB4055.raw	1.4E4	1	1	378	401	Deamidation (NQ)
R.WLGLSAEYGN(+.98)LYR.N	N	73.75	1541.7513	13	-2.2	771.8812	2	33.65	27	1783	OB4055.raw	0	0	0	389	401	Deamidation (NQ)
R.SPDIYNPQAGSLK.T	N	72.90	1388.6936	13	-0.4	695.3538	2	27.38	27	1370	OB4055.raw	9.44E4	1	1	365	377	
R.SPDIYN(+.98)PQ(+.98)AGSLKTANELNLLILR.W	N	68.90	2641.3962	24	1.0	881.4736	3	34.66	27	1851	OB4055.raw	8.13E4	1	1	365	388	Deamidation (NQ)
R.VYDEELQEGHVLVVPQN(+.98)FAVAGK.S	N	68.51	2541.2751	23	-0.6	848.0985	3	31.98	27	1660	OB4055.raw	6.03E4	1	1	436	458	
R.NALFVPHYN(+.98)TNAHSIIYALR.G	N	68.06	2314.1858	20	0.9	772.4032	3	31.89	27	1665	OB4055.raw	1.45E6	2	2	402	421	Deamidation (NQ)
R.SPDIYN(+.98)PQAGSLKTANELNLLILRWLGLSAEYGNLYR.N	N	65.10	4163.1689	37	3.1	1041.8027	4	39.91	27	2198	OB4055.raw	4.68E4	1	1	365	401	
K.SQSDNFEYVAFKTD.S	N	63.84	1649.7209	14	0.3	825.8680	2	30.79	27	1587	OB4055.raw	3.23E4	1	1	459	472	
R.SPDIYNPQAGSLKTANELNLLILR.W	N	63.60	2639.4282	24	-0.1	880.8166	3	33.89	27	1804	OB4055.raw	9.02E4	1	1	365	388	
R.SPDIYNPQAGSLKTANELN(+.98)LLILRWLGLSAEYGNLYR.N	N	61.94	4163.1689	37	4.6	1041.8043	4	39.65	27	2180	OB4055.raw	4.68E4	1	1	365	401	
R.SPDIYNPQAGSLKTANELNLLILRWLGLSAEYGNLYR.N	N	61.02	4162.1851	37	1.6	1041.5552	4	39.79	27	2169	OB4055.raw	3.44E6	1	1	365	401	
R.NRSPDIYNPQAGSLK.T	N	58.59	1658.8376	15	-1.3	830.4250	2	25.53	27	1270	OB4055.raw	2.35E5	2	2	363	377	
R.NALFVPHYN(+.98)TN(+.98)AHSIIYALR.G	N	58.26	2315.1699	20	-0.3	772.7303	3	32.31	27	1687	OB4055.raw	1.33E5	1	1	402	421	Deamidation (NQ)
R.NRSPDIYNPQ(+.98)AGSLKTANELNLLILR.W	N	58.23	2910.5562	26	0.9	728.6470	4	32.90	27	1723	OB4055.raw	3.88E5	1	1	363	388	
K.TAN(+.98)ELN(+.98)LLILRWLGLSAEYGNLYR.N	N	57.65	2793.4700	24	7.2	932.1707	3	40.44	27	2228	OB4055.raw	1.72E5	1	1	378	401	Deamidation (NQ)
K.TDSRP(+15.99)SIANLAGENSVIDNLPEEVVANSYGLPR.E	Y	57.00	3512.7383	33	2.2	1171.9226	3	36.79	27	1993	OB4055.raw	0	0	0	471	503	
R.NRSPDIYN(+.98)PQ(+.98)AGSLKTANELNLLILR.W	N	56.64	2911.5403	26	0.7	728.8928	4	33.24	27	1779	OB4055.raw	2.18E5	1	1	363	388	
R.NRSPDIYNPQAGSLKTAN(+.98)ELNLLILR.W	N	55.23	2910.5562	26	0.9	728.6470	4	32.90	27	1742	OB4055.raw	3.88E5	1	1	363	388	
R.SPDIYN(+.98)PQAGSLK.T	N	53.73	1389.6776	13	-0.4	695.8458	2	27.96	27	1456	OB4055.raw	1.22E5	1	1	365	377	

Peptide	Uniq	-10lgP	Mass	Length	ppm	m/z	z	RT	Fraction	Scan	Source File	Area Digest 5 traka 22 kDa	#Feature	#Feature Digest 5 traka 22 kDa	Start	End	PTM
K.T(+43.01)ANELNLLILRWLGLSAEYGNLYR.N	N	53.67	2834.5078	24	0.1	945.8433	3	39.96	27	2203	OB4055.raw	7.92E4	1	1	378	401	Carbamylation
K.TDSRPSIANLAGENSVIDN(+.98)LPEEVVANSYGLPR.E	Y	53.42	3497.7273	33	0.8	1166.9174	3	35.51	27	1912	OB4055.raw	0	0	0	471	503	
R.NRSPDIYNPQAGSLKTANELNLLILR.W	N	52.49	2909.5723	26	0.7	728.4008	4	32.54	27	1703	OB4055.raw	0	0	0	363	388	
K.SQ(+.98)SDN(+.98)FEYVAFK.T	N	51.74	1435.6143	12	5.7	718.8185	2	31.21	27	1611	OB4055.raw	2.16E3	1	1	459	470	Deamidation (NQ)
K.TDSRPSIANLAGENSVIDNLPEEVVANSYGLPR.E	Y	50.49	3496.7434	33	1.0	1166.5896	3	35.17	27	1912	OB4055.raw	7.71E4	1	1	471	503	
K.TDSRPSIANLAGEN(+.98).S	N	50.16	1444.6793	14	0.0	723.3469	2	28.45	27	1439	OB4055.raw	3.06E4	1	1	471	484	Deamidation (NQ)
R.SPDIYNPQ(+.98)AGSLKTANELNLLILRWLGLSAEYGNLYR.N	N	49.91	4163.1689	37	4.0	1041.8037	4	42.23	27	2342	OB4055.raw	4.68E4	1	1	365	401	
N.GIEETIC(+57.02)TATVKK.N	N	48.27	1448.7545	13	-0.4	725.3843	2	26.38	27	1317	OB4055.raw	5.13E4	1	1	346	358	Carbamidomethylation
R.SPDIYNPQAGSLKTAN(+.98)ELNLLILRWLGLSAEYGNLYR.N	N	48.23	4163.1689	37	6.0	1041.8058	4	47.15	27	2617	OB4055.raw	4.68E4	1	1	365	401	
K.TDSRPSIAN(+.98)LAGEN(+.98).S	N	46.58	1445.6633	14	-1.6	723.8378	2	29.14	27	1479	OB4055.raw	6.66E3	1	1	471	484	Deamidation (NQ)
N.GIEETIC(+71.04)TATVK.K	N	46.25	1334.6752	12	1.0	668.3455	2	28.36	27	1436	OB4055.raw	3.51E4	1	1	346	357	Propionamide
K.TDSRPSIANLAGEN(+.98)SVIDN(+.98)LPEEVVAN(+.98)SYGLPR.E	Y	44.66	3499.6953	33	7.6	1167.5813	3	35.64	27	1928	OB4055.raw	1.08E5	1	1	471	503	Deamidation (NQ)
G.SLKTANELN(+.98)LLILRWLGLSAEYGNLYR.N	N	44.57	3120.6970	27	1.6	781.1828	4	39.10	27	2143	OB4055.raw	0	0	0	375	401	
R.VYDEELQEGHVLVVPQNFAVAGK.S	N	43.94	2540.2910	23	1.1	847.7719	3	31.62	27	1640	OB4055.raw	4.8E4	1	1	436	458	
K.TDSRPSIAN(+.98)LAGEN(+.98)SVIDN(+.98)LPEEVVANSYGLPR.E	Y	41.58	3499.6953	33	7.6	1167.5813	3	35.64	27	1912	OB4055.raw	1.08E5	1	1	471	503	Deamidation (NQ)
R.NALFVPHYNTN(+.98).A	N	39.43	1289.6040	11	0.5	645.8096	2	29.63	27	1510	OB4055.raw	8.74E3	1	1	402	412	
K.TANELNLLILR.W	N	38.99	1268.7452	11	-0.3	635.3797	2	33.64	27	1785	OB4055.raw	2.08E5	1	1	378	388	
G.SLKTANELNLLILRWLGLSAEYGNLYR.N	N	38.88	3119.7131	27	-0.5	780.9352	4	38.55	27	2104	OB4055.raw	1.85E5	1	1	375	401	
N.GIEETIC(+58.01)TATVK.K	N	37.75	1321.6436	12	0.6	661.8295	2	28.75	27	1459	OB4055.raw	1.22E4	1	1	346	357	Carboxymethyl
K.TANELN(+.98)LLILR(+14.02)WLGLSAEYGNLYR.N	N	37.66	2806.5017	24	0.4	936.5082	3	40.72	27	2260	OB4055.raw	1.57E4	1	1	378	401	Methylation(KR)
R.SPDIYN(+.98)PQAGSLKTAN(+.98)ELNLLILRWLGLSAEYGNLYR.N	N	36.79	4164.1528	37	7.2	1042.0530	4	44.53	27	2480	OB4055.raw	0	0	0	365	401	
K.TDSRPSIANLAGENSVIDNLP(+15.99)EEVVANSYGLPR.E	Y	34.63	3512.7383	33	-0.4	879.1915	4	36.29	27	1961	OB4055.raw	0	0	0	471	503	Hydroxylation Pro
R.SPDIYN(+.98)PQ(+.98)AGSLKTANELNLLILRWLGLSAEYGNLYR.N	N	33.97	4164.1528	37	8.4	1042.0542	4	45.83	27	2558	OB4055.raw	0	0	0	365	401	
K.TANELNLLILR(+14.02)WLGLSAEYGNLYR.N	N	33.37	2805.5176	24	2.2	936.1819	3	40.25	27	2216	OB4055.raw	6.97E4	1	1	378	401	Methylation(KR)
R.SPDIYNP(+15.99)Q(+.98)AGSLKTANELNLLILRWLGLSAEYGNLYR.N	N	32.18	4179.1641	37	0.5	1045.7988	4	41.39	27	2291	OB4055.raw	0	0	0	365	401	Hydroxylation Pro
K.TDSRPSIANLAGEN(+.98)SVIDNLPEEVVANSYGLPREQ(+.98)AR.Q	Y	31.25	3982.9507	37	2.1	996.7471	4	34.52	27	1845	OB4055.raw	0	0	0	471	507	
G.SLKTAN(+.98)ELN(+.98)LLILRWLGLSAEYGNLYR.N	N	31.03	3121.6812	27	3.8	1041.5717	3	39.09	27	2146	OB4055.raw	1.61E4	1	1	375	401	
R.WLGLSAEYGNLYRNALFVPHYN(+.98)TN(+.98)AHSIIYALR.G	N	30.70	3837.9268	33	4.2	768.5958	5	35.09	27	1913	OB4055.raw	1.63E5	1	1	389	421	
R.NALFVPHYNTN(+.98)AHSIIYALRGR.A	N	29.99	2527.3083	22	0.0	843.4434	3	31.16	27	1609	OB4055.raw	4.64E4	1	1	402	423	Deamidation (NQ)

Peptide	Uniq	-10lgP	Mass	Length	ppm	m/z	z	RT	Fraction	Scan	Source File	Area Digest 5 traka 22 kDa	#Feature	#Feature Digest 5 traka 22 kDa	Start	End	PTM
K.TANELN(+.98)LLILR.W	N	29.92	1269.7292	11	1.0	635.8726	2	34.22	27	1822	OB4055.raw	6.28E4	1	1	378	388	
R.SPDIYNP(+15.99)QAGSLKTANELNLLILRWLGLSAEYGNLYR.N	N	29.68	4178.1797	37	2.6	1045.5549	4	40.15	27	2159	OB4055.raw	1.36E5	1	1	365	401	Hydroxylation Pro
R.WLGLSAEYGN(+.98)LYRNALFVPHYN(+.98)TN(+.98)AHSIIYALR.G	N	29.39	3838.9106	33	6.1	768.7941	5	35.52	27	1913	OB4055.raw	0	0	0	389	421	
R.WLGLSAEYGNLYRN(+.98)ALFVPHYNTN(+.98)AHSIIYALR.G	N	28.44	3837.9268	33	4.2	768.5958	5	35.09	27	1907	OB4055.raw	1.63E5	1	1	389	421	
R.NALFVPHYNTNAHSIIYALRG.R	N	27.77	2370.2231	21	1.4	791.0828	3	31.62	27	1624	OB4055.raw	8.89E4	1	1	402	422	
K.TDSRPSIANLAGENSVIDNLP(+15.99)EEVVANSYGLPREQ(+.98)AR.Q	Y	27.62	3997.9617	37	7.2	1000.5049	4	34.84	27	1994	OB4055.raw	1.46E6	1	1	471	507	
K.TDSRPSIANLAGEN(+.98)SVIDNLPEEVVANSYGLPREQAR.Q	Y	27.56	3981.9668	37	3.1	996.5021	4	34.04	27	1845	OB4055.raw	3.61E5	1	1	471	507	
R.WLGLSAEYGNLYRN(+.98)ALFVPHYN(+.98)TN(+.98)AHSIIYALR.G	N	27.29	3838.9106	33	5.2	768.7934	5	35.43	27	1907	OB4055.raw	0	0	0	389	421	
G.IEET(+114.04)IC(+57.02)TATVK.K	N	27.03	1377.6810	11	0.7	689.8483	2	28.26	27	1432	OB4055.raw	2.47E4	1	1	347	357	Ubiquitin; Carbamidomethylation
R.SPDIYNPQ(+.98)AGSLKTANELN(+.98)LLILRWLGLSAEYGNLYR.N	N	26.55	4164.1528	37	2.5	1042.0481	4	47.88	27	2681	OB4055.raw	0	0	0	365	401	
K.T(+14.02)ANELNLLILRWLGLSAEYGNLYR.N	N	26.25	2805.5176	24	1.2	936.1810	3	40.59	27	2242	OB4055.raw	0	0	0	378	401	
N.GIEETIC(+57.02)TATVKKNIGR.N	N	26.11	1889.0040	17	-0.6	473.2580	4	26.89	27	1346	OB4055.raw	2.81E5	1	1	346	362	Carbamidomethylation
K.NIGRNRSPDIYN(+.98)PQAGSLK.T	N	25.83	2100.0710	19	0.2	701.0311	3	25.59	27	1264	OB4055.raw	0	0	0	359	377	
R.SPDIYNP(+15.99)Q(+.98)AGSLKTAN(+.98)ELNLLILRWLGLSAEYGNLYR.N	N	25.16	4180.1479	37	4.7	1046.0492	4	42.38	27	2351	OB4055.raw	0	0	0	365	401	Hydroxylation Pro
K.T(-15.01)ANELNLLILRWLGLSAEYGNLYR.N	N	24.82	2776.4912	24	-3.4	926.5012	3	46.65	27	2607	OB4055.raw	0	0	0	378	401	ISD (z+2)-series
K.N(+.98)IGRNRSPDIYNPQAGSLK.T	N	24.58	2100.0710	19	0.1	701.0310	3	25.67	27	1269	OB4055.raw	0	0	0	359	377	
R.NALFVPHYN(+.98)TNAHSIIYALRGR.A	N	24.45	2527.3083	22	0.4	632.8346	4	31.16	27	1597	OB4055.raw	1.55E6	1	1	402	423	
R.SPDIYNPQ(+.98)AGSLKTAN(+.98)ELN(+.98)LLILRWLGLSAEYGNLYR.N	N	23.22	4165.1372	37	3.0	1042.2947	4	49.37	27	2769	OB4055.raw	0	0	0	365	401	
G.SLKTAN(+.98)ELNLLILRWLGLSAEYGNLYR.N	N	23.19	3120.6970	27	7.3	781.1873	4	41.07	27	2272	OB4055.raw	0	0	0	375	401	
R.AHVQVVDSN(+.98)GNRVYDEELQEGHVLVVPQNFAVAGK.S	N	22.21	3817.9023	35	-1.0	955.4819	4	31.24	27	1613	OB4055.raw	0	0	0	424	458	
R.AHVQVVDSN(+.98)GNRVYDEELQ(+.98)EGHVLVVPQ(+.98)NFAVAGK.S	N	22.19	3819.8704	35	5.4	764.9855	5	31.35	27	1643	OB4055.raw	1.55E5	1	1	424	458	
R.AHVQVVDSNGN(+.98)RVYDEELQ(+.98)EGHVLVVPQNFAVAGK.S	N	21.90	3818.8862	35	1.8	764.7859	5	31.36	27	1622	OB4055.raw	0	0	0	424	458	
**total 80 peptides**																	

tr|Q9FZ11|Q9FZ11_ARAHY

back to list

| Protein Coverage | Supporting Peptides |

Protein Coverage:

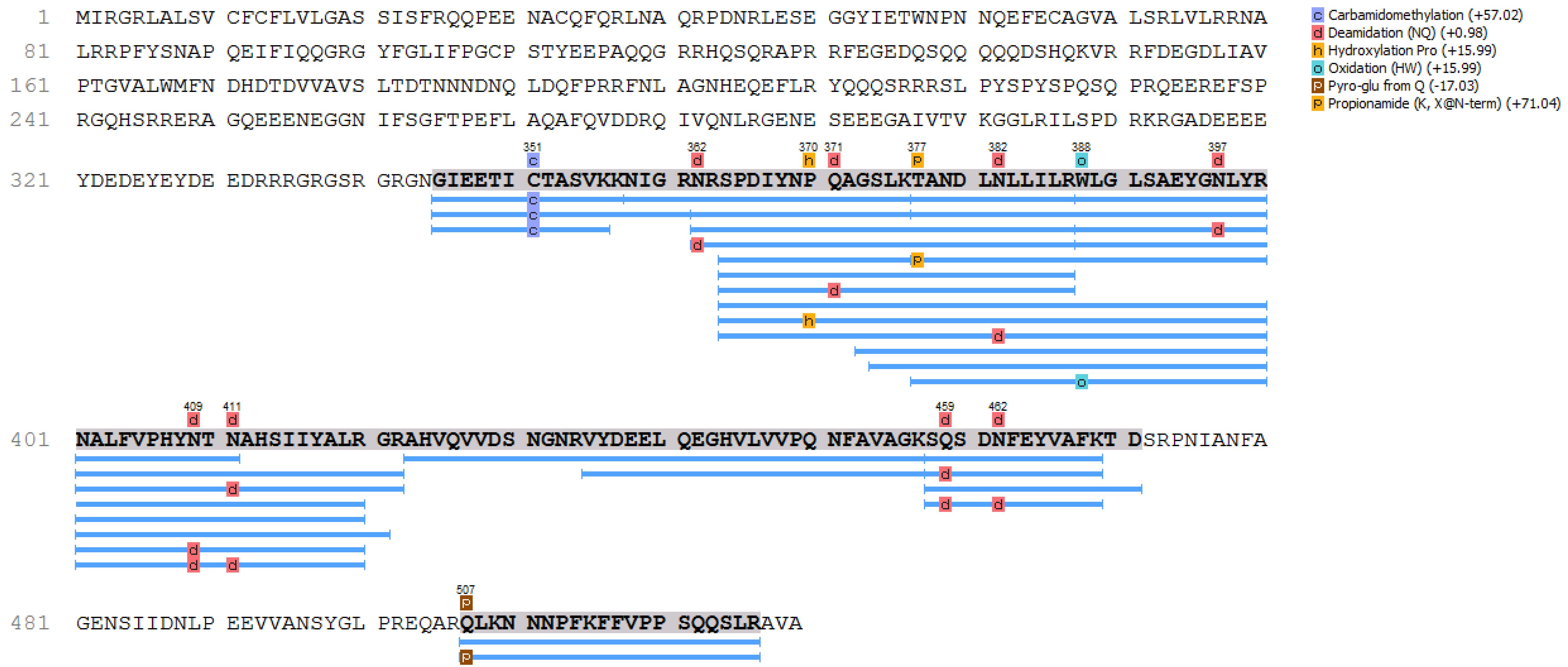

Supporting Peptides:

Peptide	Uniq	-10lgP	Mass	Length	ppm	m/z	z	RT	Fraction	Scan	Source File	Area Digest 5 traka 22 kDa	#Feature	#Feature Digest 5 traka 22 kDa	Start	End	PTM
R.WLGLSAEYGNLYR.N	N	98.82	1540.7673	13	0.1	771.3911	2	33.33	27	1749	OB4055.raw	1.54E6	1	1	388	400	
K.TANDLNLLILRWLGLSAEYGNLYR.N	N	95.42	2777.4863	24	1.6	926.8375	3	39.71	27	2197	OB4055.raw	4.05E6	4	4	377	400	
K.SQ(+.98)SDNFEYVAFK.T	N	89.03	1434.6302	12	2.2	718.3240	2	30.79	27	1580	OB4055.raw	6.12E4	1	1	458	469	Deamidation (NQ)

Peptide	Uniq	-10lgP	Mass	Length	ppm	m/z	z	RT	Fraction	Scan	Source File	Area Digest 5 traka 22 kDa	#Feature	#Feature Digest 5 traka 22 kDa	Start	End	PTM
K.SQSDNFEYVAFK.T	N	88.10	1433.6462	12	1.3	717.8314	2	30.42	27	1552	OB4055.raw	1.4E5	1	1	458	469	
K.TANDLN(+.98)LLILRWLGLSAEYGNLYR.N	N	85.28	2778.4705	24	3.0	927.1669	3	40.36	27	2227	OB4055.raw	6.5E3	3	3	377	400	
R.SPDIYNPQ(+.98)AGSLKTANDLNLLILR.W	N	79.73	2626.3965	24	0.0	876.4728	3	33.97	27	1802	OB4055.raw	2.51E5	1	1	364	387	Deamidation (NQ)
R.NALFVPHYNTNAHSIIYALR.G	N	77.97	2313.2019	20	0.4	772.0749	3	31.16	27	1603	OB4055.raw	2.7E6	2	2	401	420	
R.SPDIYNPQ(+.98)AGSLK.T	N	77.51	1389.6776	13	-0.4	695.8458	2	27.96	27	1407	OB4055.raw	1.22E5	1	1	364	376	
N.GIEETIC(+57.02)TASVK.K	N	77.10	1306.6438	12	-0.3	654.3290	2	27.67	27	1393	OB4055.raw	1.37E5	1	1	345	356	Carbamidomethylation
R.SPDIYNPQAGSLKTANDLNLLILR.W	N	75.09	2625.4126	24	0.8	876.1455	3	33.57	27	1780	OB4055.raw	9.38E4	1	1	364	387	
R.WLGLSAEYGN(+.98)LYR.N	N	73.75	1541.7513	13	-2.2	771.8812	2	33.65	27	1783	OB4055.raw	0	0	0	388	400	Deamidation (NQ)
R.SPDIYNPQAGSLK.T	N	72.90	1388.6936	13	-0.4	695.3538	2	27.38	27	1370	OB4055.raw	9.44E4	1	1	364	376	
R.VYDEELQEGHVLVVPQN(+.98)FAVAGK.S	N	68.51	2541.2751	23	-0.6	848.0985	3	31.98	27	1660	OB4055.raw	6.03E4	1	1	435	457	
R.NALFVPHYN(+.98)TNAHSIIYALR.G	N	68.06	2314.1858	20	0.9	772.4032	3	31.89	27	1665	OB4055.raw	1.45E6	2	2	401	420	Deamidation (NQ)
R.SPDIYNPQ(+.98)AGSLKTAN(+.98)DLNLLILR.W	N	66.93	2627.3806	24	0.7	876.8014	3	34.40	27	1838	OB4055.raw	1.05E5	1	1	364	387	
R.NRSPDIYNPQAGSLKTANDLNLLILR.W	N	66.35	2895.5566	26	-0.1	724.8964	4	32.20	27	1678	OB4055.raw	0	0	0	362	387	
K.SQSDNFEYVAFKTD.S	N	63.84	1649.7209	14	0.3	825.8680	2	30.79	27	1587	OB4055.raw	3.23E4	1	1	458	471	
R.NRSPDIYNPQAGSLK.T	N	58.59	1658.8376	15	-1.3	830.4250	2	25.53	27	1270	OB4055.raw	2.35E5	2	2	362	376	
R.NALFVPHYN(+.98)TN(+.98)AHSIIYALR.G	N	58.26	2315.1699	20	-0.3	772.7303	3	32.31	27	1687	OB4055.raw	1.33E5	1	1	401	420	Deamidation (NQ)
R.SPDIYN(+.98)PQAGSLK.T	N	53.73	1389.6776	13	-0.4	695.8458	2	27.96	27	1456	OB4055.raw	1.22E5	1	1	364	376	
N.GIEETIC(+57.02)TASVKK.N	N	52.94	1434.7388	13	0.2	718.3768	2	25.91	27	1293	OB4055.raw	3.94E5	3	3	345	357	Carbamidomethylation
R.SPDIYNPQAGSLKTANDLNLLILRWLGLSAEYGNLYR.N	N	52.19	4148.1694	37	1.3	1038.0510	4	39.63	27	2149	OB4055.raw	4.13E6	1	1	364	400	
K.SQ(+.98)SDN(+.98)FEYVAFK.T	N	51.74	1435.6143	12	5.7	718.8185	2	31.21	27	1611	OB4055.raw	2.16E3	1	1	458	469	Deamidation (NQ)
R.NRSPDIYNPQ(+.98)AGSLKTANDLNLLILR.W	N	47.56	2896.5405	26	-0.1	725.1423	4	32.56	27	1700	OB4055.raw	3.94E5	1	1	362	387	
R.Q(+.98)LKNNN(+.98)PFKFFVPPSQQ(+.98)SLR.A	Y	46.87	2391.2222	20	2.1	798.0830	3	32.22	27	1688	OB4055.raw	1.46E5	1	1	507	526	
R.SPDIYNPQAGSLKTANDLN(+.98)LLILRWLGLSAEYGNLYR.N	N	46.71	4149.1533	37	4.2	1038.2999	4	44.01	27	2449	OB4055.raw	0	0	0	364	400	Deamidation (NQ)
R.NRSPDIYN(+.98)PQAGSLKTAN(+.98)DLNLLILR.W	N	45.54	2897.5247	26	1.5	725.3895	4	33.43	27	1754	OB4055.raw	9.81E4	1	1	362	387	
G.SLKTANDLNLLILRWLGLSAEYGNLYR.N	N	45.10	3105.6975	27	0.1	777.4317	4	38.47	27	2116	OB4055.raw	4.25E5	1	1	374	400	
R.SPDIYNPQ(+.98)AGSLKTANDLNLLILRWLGLSAEYGNLYR.N	N	44.86	4149.1533	37	2.6	1038.2983	4	39.73	27	2186	OB4055.raw	2.09E3	1	1	364	400	
R.VYDEELQEGHVLVVPQNFAVAGK.S	N	43.94	2540.2910	23	1.1	847.7719	3	31.62	27	1640	OB4055.raw	4.8E4	1	1	435	457	
G.SLKTANDLN(+.98)LLILRWLGLSAEYGNLYR.N	N	43.59	3106.6814	27	3.8	777.6805	4	39.50	27	2170	OB4055.raw	0	0	0	374	400	
K.T(+71.04)ANDLNLLILRWLGLSAEYGNLYR.N	N	42.77	2848.5234	24	0.4	950.5155	3	40.15	27	2219	OB4055.raw	3.7E4	1	1	377	400	Propionamide (K, X@N-term)

Peptide	Uniq	-10lgP	Mass	Length	ppm	m/z	z	RT	Fraction	Scan	Source File	Area Digest 5 traka 22 kDa	#Feature	#Feature Digest 5 traka 22 kDa	Start	End	PTM
R.SPDIYNPQAGSLKTAN(+.98)DLNLLILRWLGLSAEYGNLYR.N	N	42.71	4149.1533	37	3.7	1038.2994	4	39.48	27	2168	OB4055.raw	0	0	0	364	400	
R.SPDIYNP(+15.99)QAGSLKTANDLNLLILRWLGLSAEYGNLYR.N	N	42.27	4164.1641	37	3.0	1042.0514	4	40.64	27	2245	OB4055.raw	0	0	0	364	400	Hydroxylation Pro
R.SPDIYN(+.98)PQAGSLKTANDLNLLILRWLGLSAEYGNLYR.N	N	41.28	4149.1533	37	1.8	1038.2975	4	43.13	27	2396	OB4055.raw	0	0	0	364	400	
R.NRSPDIYN(+.98)PQ(+.98)AGSLKTAN(+.98)DLNLLILR.W	N	41.16	2898.5085	26	6.2	725.6389	4	33.08	27	1754	OB4055.raw	1.98E5	1	1	362	387	
R.SPDIYNPQ(+.98)AGSLKTANDLN(+.98)LLILRWLGLSAEYGNLYR.N	N	40.27	4150.1372	37	7.8	1038.5497	4	44.34	27	2469	OB4055.raw	7.17E3	1	1	364	400	
R.NALFVPHYNTN(+.98).A	N	39.43	1289.6040	11	0.5	645.8096	2	29.63	27	1510	OB4055.raw	8.74E3	1	1	401	411	
R.NRSPDIYN(+.98)PQ(+.98)AGSLKTANDLNLLILR.W	N	38.72	2897.5247	26	1.5	725.3895	4	33.43	27	1773	OB4055.raw	9.81E4	1	1	362	387	
R.SPDIYNPQAGSLKTAN(+.98)DLN(+.98)LLILRWLGLSAEYGNLYR.N	N	36.34	4150.1372	37	7.8	1038.5497	4	43.61	27	2425	OB4055.raw	7.17E3	1	1	364	400	
R.NRSPDIYNPQAGSLKTAN(+.98)DLN(+.98)LLILR.W	N	35.98	2897.5247	26	1.5	725.3895	4	33.43	27	1763	OB4055.raw	9.81E4	1	1	362	387	
K.TANDLNLLILRW(+15.99)LGLSAEYGNLYR.N	N	35.89	2793.4812	24	8.6	932.1757	3	44.65	27	2481	OB4055.raw	8.59E3	1	1	377	400	Oxidation (HW)
N.GIEETIC(+57.02)TASVKKNIGR.N	N	31.74	1874.9884	17	-0.2	469.7543	4	26.50	27	1347	OB4055.raw	5.22E5	2	2	345	361	Carbamidomethylation
R.N(+.98)RSPDIYN(+.98)PQAGSLKTANDLN(+.98)LLILR.W	N	31.43	2898.5085	26	6.2	725.6389	4	33.08	27	1763	OB4055.raw	1.98E5	1	1	362	387	Deamidation (NQ)
R.WLGLSAEYGNLYRNALFVPHYN(+.98)TN(+.98)AHSIIYALR.G	N	30.70	3837.9268	33	4.2	768.5958	5	35.09	27	1913	OB4055.raw	1.63E5	1	1	388	420	
R.SPDIYNPQ(+.98)AGSLKTAN(+.98)DLNLLILRWLGLSAEYGNLYR.N	N	30.15	4150.1372	37	5.3	1038.5471	4	42.52	27	2360	OB4055.raw	7.17E3	1	1	364	400	
R.NALFVPHYNTN(+.98)AHSIIYALRGR.A	N	29.99	2527.3083	22	0.0	843.4434	3	31.16	27	1609	OB4055.raw	4.64E4	1	1	401	422	Deamidation (NQ)
K.TANDLNLLILRWLGLSAEYGN(+.98)LYR.N	N	29.79	2778.4705	24	6.3	927.1699	3	50.07	27	2826	OB4055.raw	4.35E3	1	1	377	400	
R.WLGLSAEYGN(+.98)LYRNALFVPHYN(+.98)TN(+.98)AHSIIYALR.G	N	29.39	3838.9106	33	6.1	768.7941	5	35.52	27	1913	OB4055.raw	0	0	0	388	420	
R.SPDIYN(+.98)PQAGSLKTAN(+.98)DLNLLILRWLGLSAEYGNLYR.N	N	28.95	4150.1372	37	6.3	1384.3950	3	39.63	27	2213	OB4055.raw	1.07E5	1	1	364	400	
R.WLGLSAEYGNLYRN(+.98)ALFVPHYNTN(+.98)AHSIIYALR.G	N	28.44	3837.9268	33	4.2	768.5958	5	35.09	27	1907	OB4055.raw	1.63E5	1	1	388	420	
G.SLKTAN(+.98)DLNLLILRWLGLSAEYGNLYR.N	N	28.33	3106.6814	27	3.8	777.6806	4	41.37	27	2290	OB4055.raw	0	0	0	374	400	
R.SPDIYN(+.98)PQ(+.98)AGSLKTANDLNLLILRWLGLSAEYGNLYR.N	N	27.98	4150.1372	37	7.3	1038.5492	4	45.23	27	2522	OB4055.raw	0	0	0	364	400	
R.NALFVPHYNTNAHSIIYALRG.R	N	27.77	2370.2231	21	1.4	791.0828	3	31.62	27	1624	OB4055.raw	8.89E4	1	1	401	421	
R.WLGLSAEYGNLYRN(+.98)ALFVPHYN(+.98)TN(+.98)AHSIIYALR.G	N	27.29	3838.9106	33	5.2	768.7934	5	35.43	27	1907	OB4055.raw	0	0	0	388	420	
K.NIGRNRSPDIYN(+.98)PQAGSLK.T	N	25.83	2100.0710	19	0.2	701.0311	3	25.59	27	1264	OB4055.raw	0	0	0	358	376	
K.TANDLNLLILR.W	N	25.78	1254.7296	11	-0.8	628.3716	2	33.40	27	1789	OB4055.raw	2.37E5	1	1	377	387	
R.SPDIYN(+.98)PQAGSLKTANDLN(+.98)LLILRWLGLSAEYGNLYR.N	N	25.56	4150.1372	37	8.4	1038.5503	4	48.41	27	2712	OB4055.raw	7.17E3	1	1	364	400	
A.GSLKTAN(+.98)DLNLLILRWLGLSAEYGNLYR.N	N	25.38	3163.7029	28	2.0	791.9346	4	39.16	27	2147	OB4055.raw	0	0	0	373	400	
K.N(+.98)IGRNRSPDIYNPQAGSLK.T	N	24.58	2100.0710	19	0.1	701.0310	3	25.67	27	1269	OB4055.raw	0	0	0	358	376	
R.NALFVPHYN(+.98)TNAHSIIYALRGR.A	N	24.45	2527.3083	22	0.4	632.8346	4	31.16	27	1597	OB4055.raw	1.55E6	1	1	401	422	

Peptide	Uniq	-10lgP	Mass	Length	ppm	m/z	z	RT	Fraction	Scan	Source File	Area Digest 5 traka 22 kDa	#Feature	#Feature Digest 5 traka 22 kDa	Start	End	PTM
R.Q(-17.03)LKN(+.98)NNPFKFFVPPSQQSLR.A	Y	23.14	2372.2275	20	0.7	791.7503	3	33.16	27	1774	OB4055.raw	4.47E4	1	1	507	526	Pyro-glu from Q
R.AHVQVVDSN(+.98)GNRVYDEELQEGHVLVVPQNFAVAGK.S	N	22.21	3817.9023	35	-1.0	955.4819	4	31.24	27	1613	OB4055.raw	0	0	0	423	457	
R.AHVQVVDSN(+.98)GNRVYDEELQ(+.98)EGHVLVVPQ(+.98)NFAVAGK.S	N	22.19	3819.8704	35	5.4	764.9855	5	31.35	27	1643	OB4055.raw	1.55E5	1	1	423	457	
R.AHVQVVDSNGN(+.98)RVYDEELQ(+.98)EGHVLVVPQNFAVAGK.S	N	21.90	3818.8862	35	1.8	764.7859	5	31.36	27	1622	OB4055.raw	0	0	0	423	457	
**total 65 peptides**																	

tr|Q8LKN1|Q8LKN1_ARAHY
back to list

| Protein Coverage | Supporting Peptides |

Protein Coverage:

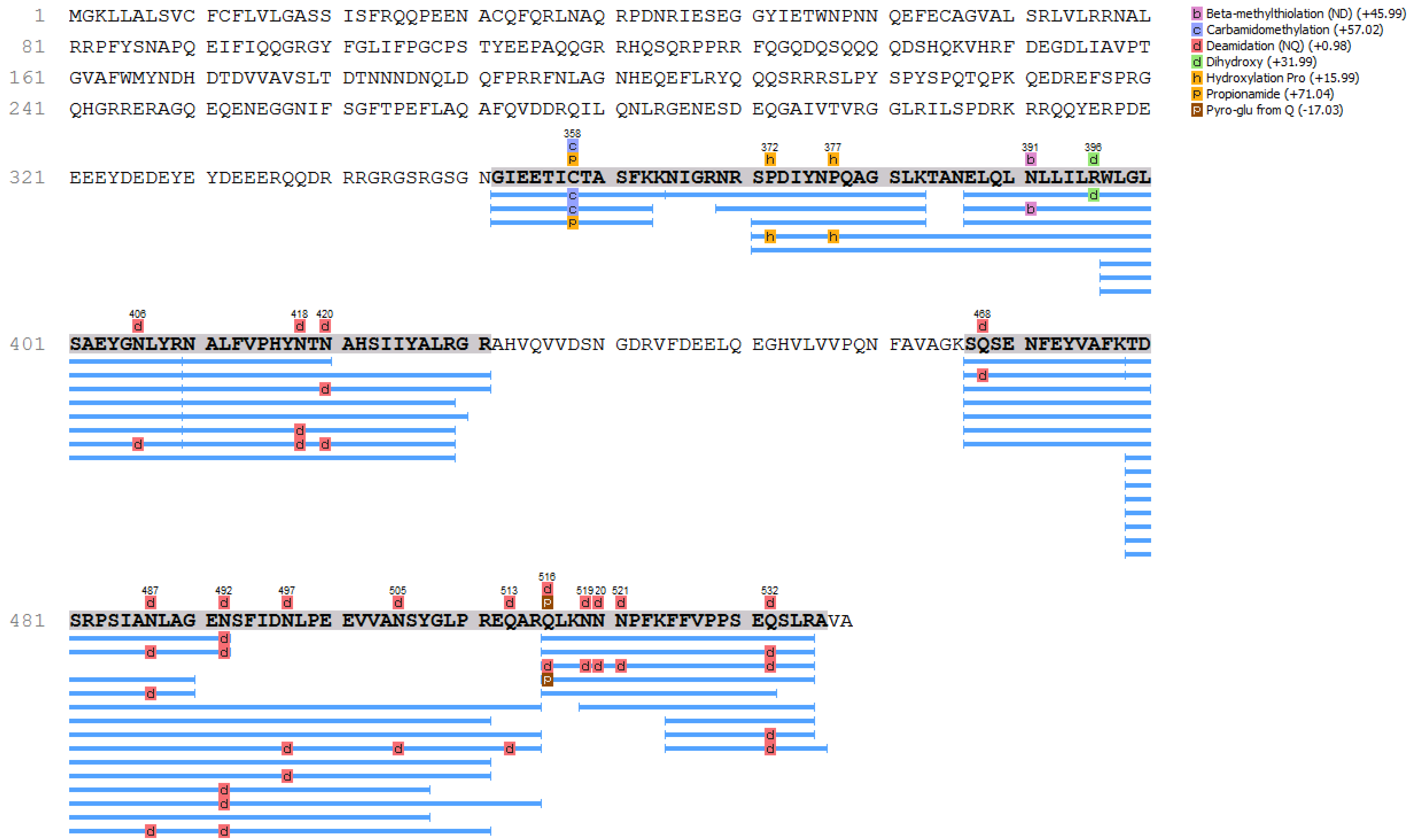
1 MGKLLALSVC FCFLVLGASS ISFRQQPEEN ACQFQRLNAQ RPDNRIESEG GYIETWNPNN QEFECAGVAL SRLVLRRNAL
81 RRPFYSNAPQ EIFIQQGRGY FGLIFPGCPS TYEEPAQQGR RHQSQRPPRR FQGQDQSQQQ QDSHQKVHRF DEGDLIAVPT
161 GVAFWMYNDH DTDVVAVSLT DTNNNDNQLD QFPRRFNLAG NHEQEFLRYQ QQSRRRSLPY SPYSPQTQPK QEDREFSPRG
241 QHGRRERAGQ EQENEGGNIF SGFTPEFLAQ AFQVDDRQIL QNLRGENESD EQGAIVTVRG GLRILSPDRK RRQQYERPDE
358 c p
372 h
377 h
391 b
396 d
321 EEEYDEDEYE YDEEERQQDR RRGRGSRGSG NGIEETICTA SFKKNIGRNR SPDIYNPQAG SLKTANELQL NLLILRWLGL
406 d
418 d
420 d
468 d
401 SAEYGNLYRN ALFVPHYNTN AHSIIYALRG RAHVQVVDSN GDRVFDEELQ EGHVLVVPQN FAVAGKSQSE NFEYVAFKTD
487 d
492 d
497 d
505 d
513 d
516 d p
519 d
20 d
521 d
532 d
481 SRPSIANLAG ENSFIDNLPE EVVANSYGLP REQARQLKNN NPFKFFVPPS EQSLRAVA
b Beta-methylthiolation (ND) (+45.99)
c Carbamidomethylation (+57.02)
d Deamidation (NQ) (+0.98)
d Dihydroxy (+31.99)
h Hydroxylation Pro (+15.99)
p Propionamide (+71.04)
p Pyro-glu from Q (-17.03)

Supporting Peptides:

Peptide	Uniq	-10lgP	Mass	Length	ppm	m/z	z	RT	Fraction	Scan	Source File	Area Digest 5 traka 22 kDa	#Feature	#Feature Digest 5 traka 22 kDa	Start	End	PTM
R.WLGLSAEYGNLYR.N	N	98.82	1540.7673	13	0.1	771.3911	2	33.33	27	1749	OB4055.raw	1.54E6	1	1	397	409	
K.SQSENFEYVAFK.T	N	89.61	1447.6619	12	0.4	724.8385	2	30.22	27	1543	OB4055.raw	2.25E5	1	1	467	478	
K.TDSRPSIANLAGENSFIDNLPEEVVANSYGLPR.E	N	78.92	3544.7434	33	1.0	1182.5896	3	36.12	27	1936	OB4055.raw	5.39E5	1	1	479	511	
R.NALFVPHYNTNAHSIIYALR.G	N	77.97	2313.2019	20	0.4	772.0749	3	31.16	27	1603	OB4055.raw	2.7E6	2	2	410	429	
R.SPDIYNPQ(+.98)AGSLK.T	N	77.51	1389.6776	13	-0.4	695.8458	2	27.96	27	1407	OB4055.raw	1.22E5	1	1	371	383	
R.WLGLSAEYGN(+.98)LYR.N	N	73.75	1541.7513	13	-2.2	771.8812	2	33.65	27	1783	OB4055.raw	0	0	0	397	409	Deamidation (NQ)
K.SQ(+.98)SENFEYVAFK.T	N	73.36	1448.6459	12	-0.8	725.3297	2	30.52	27	1573	OB4055.raw	1.12E5	1	1	467	478	Deamidation (NQ)
R.SPDIYNPQAGSLK.T	N	72.90	1388.6936	13	-0.4	695.3538	2	27.38	27	1370	OB4055.raw	9.44E4	1	1	371	383	
N.GIEETIC(+57.02)TASFK.K	N	72.28	1354.6438	12	1.3	678.3301	2	29.63	27	1513	OB4055.raw	1.87E4	1	1	352	363	Carbamidomethyla
K.TDSRPSIANLAGENSFIDN(+.98)LPEEVVANSYGLPR.E	N	70.54	3545.7273	33	2.3	1182.9191	3	36.12	27	1951	OB4055.raw	0	0	0	479	511	Deamidation (NQ)
R.NALFVPHYN(+.98)TNAHSIIYALR.G	N	68.06	2314.1858	20	0.9	772.4032	3	31.89	27	1665	OB4055.raw	1.45E6	2	2	410	429	Deamidation (NQ)
K.TDSRPSIANLAGEN(+.98)SFIDN(+.98)LPEEVVANSYGLPR.E	N	65.76	3546.7114	33	4.7	1183.2500	3	36.50	27	1975	OB4055.raw	0	0	0	479	511	
K.TDSRPSIANLAGEN(+.98)SFIDNLPEEVVANSYGLPR.E	N	63.54	3545.7273	33	5.8	1182.9232	3	37.11	27	2013	OB4055.raw	0	0	0	479	511	
R.NRSPDIYNPQAGSLK.T	N	58.59	1658.8376	15	-1.3	830.4250	2	25.53	27	1270	OB4055.raw	2.35E5	2	2	369	383	
R.NALFVPHYN(+.98)TN(+.98)AHSIIYALR.G	N	58.26	2315.1699	20	-0.3	772.7303	3	32.31	27	1687	OB4055.raw	1.33E5	1	1	410	429	Deamidation (NQ)
N.GIEETIC(+57.02)TASFKK.N	N	56.80	1482.7388	13	0.1	742.3767	2	27.67	27	1396	OB4055.raw	2.93E5	2	2	352	364	Carbamidomethyla
K.SQSENFEYVAFKTD.S	N	55.46	1663.7366	14	0.2	832.8757	2	30.70	27	1581	OB4055.raw	4.71E4	1	1	467	480	
K.TDSRPSIAN(+.98)LAGEN(+.98)SFIDN(+.98)LPEEVVANSYGLPR.E	N	55.41	3547.6953	33	-3.4	1183.5684	3	36.99	27	2005	OB4055.raw	0	0	0	479	511	Deamidation (NQ)
R.SPDIYN(+.98)PQAGSLK.T	N	53.73	1389.6776	13	-0.4	695.8458	2	27.96	27	1456	OB4055.raw	1.22E5	1	1	371	383	
K.TDSRPSIANLAGEN(+.98).S	N	50.16	1444.6793	14	0.0	723.3469	2	28.45	27	1439	OB4055.raw	3.06E4	1	1	479	492	Deamidation (NQ)
R.Q(+.98)LKN(+.98)N(+.98)NPFKFFVPPSEQ(+.98)SLR.A	N	50.10	2393.1902	20	9.1	798.7446	3	33.01	27	1737	OB4055.raw	0	0	0	516	535	Deamidation (NQ)
R.Q(+.98)LKN(+.98)NNPFKFFVPPSEQ(+.98)SLR.A	N	49.87	2392.2063	20	0.8	798.4100	3	32.48	27	1719	OB4055.raw	2.19E5	1	1	516	535	Deamidation (NQ)
K.TDSRPSIAN(+.98)LAGEN(+.98).S	N	46.58	1445.6633	14	-1.6	723.8378	2	29.14	27	1479	OB4055.raw	6.66E3	1	1	479	492	Deamidation (NQ)
K.FFVPPSEQ(+.98)SLRA.V	N	44.42	1377.6927	12	-0.4	689.8534	2	30.52	27	1563	OB4055.raw	9.82E4	1	1	525	536	Deamidation (NQ)
R.Q(+.98)LKN(+.98)NN(+.98)PFKFFVPPSEQ(+.98)SLR.A	N	44.04	2393.1902	20	-1.3	798.7363	3	33.29	27	1756	OB4055.raw	0	0	0	516	535	Deamidation (NQ)
K.TDSRPSIANLAGEN(+.98)SFIDNLPEEVVANSYGLPREQAR.Q	N	40.05	4029.9668	37	4.1	1008.5031	4	36.96	27	2003	OB4055.raw	0	0	0	479	515	Deamidation (NQ)
K.SQSENFEYVAFKTDSRPSIANLAG.E	N	39.51	2630.2612	24	0.5	877.7615	3	31.62	27	1639	OB4055.raw	6.96E4	1	1	467	490	

Peptide	Uniq	-10lgP	Mass	Length	ppm	m/z	z	RT	Fraction	Scan	Source File	Area Digest 5 traka 22 kDa	#Feature	#Feature Digest 5 traka 22 kDa	Start	End	PTM
R.NALFVPHYNTN(+.98).A	N	39.43	1289.6040	11	0.5	645.8096	2	29.63	27	1510	OB4055.raw	8.74E3	1	1	410	420	
K.TDSRPSIANLAGENSFIDNLPEEVVANSYGLPREQAR.Q	N	39.23	4028.9827	37	1.2	1008.2542	4	34.76	27	1849	OB4055.raw	1.21E6	1	1	479	515	
N.GIEETIC(+71.04)TASFK.K	N	39.07	1368.6595	12	0.1	685.3371	2	29.88	27	1527	OB4055.raw	0	0	0	352	363	Propionamide
R.Q(+.98)LKN(+.98)NNPFKFFVPPSEQSLR.A	N	38.96	2391.2222	20	2.1	798.0830	3	32.22	27	1684	OB4055.raw	1.46E5	1	1	516	535	
K.TDSRPSIANLAGEN(+.98)SFIDNLPEEVVAN(+.98)SYGLPREQ(+.98)AR.Q	N	37.47	4031.9348	37	7.8	1008.9988	4	34.84	27	2003	OB4055.raw	2.53E6	1	1	479	515	
K.TDSRPSIANLAGEN(+.98)SFIDNLPEEVVANSYGLPREQ(+.98)AR.Q	N	36.60	4030.9507	37	3.3	1008.7483	4	36.71	27	1988	OB4055.raw	0	0	0	479	515	
K.TDSRPSIANLAGEN(+.98)SFIDN(+.98)LPEEVVANSYGLPREQ(+.98)AR.Q	N	36.33	4031.9348	37	7.8	1008.9988	4	34.84	27	1885	OB4055.raw	2.53E6	1	1	479	515	
K.TDSRPSIANLAGENSFIDNLPEEVVANSYGLPREQ(+.98)AR.Q	N	35.87	4029.9668	37	2.2	1008.5012	4	34.77	27	1861	OB4055.raw	0	0	0	479	515	
K.TDSRPSIANLAGENSFIDN(+.98)LPEEVVANSYGLPREQ(+.98)AR.Q	N	35.49	4030.9507	37	2.8	1008.7477	4	35.10	27	1885	OB4055.raw	0	0	0	479	515	
K.FFVPPSEQ(+.98)SLR.A	N	34.94	1306.6556	11	0.4	654.3353	2	30.03	27	1531	OB4055.raw	2.36E4	1	1	525	535	Deamidation (NQ)
R.SP(+15.99)DIYNP(+15.99)QAGSLKTANELQLNLLILRWLGLSAEYGNLYR.N	Y	33.96	4435.3174	39	0.3	888.0710	5	38.73	27	2336	OB4055.raw	1.92E6	2	2	371	409	Hydroxylation Pro
K.TDSRPSIAN(+.98)LAGENSFIDN(+.98)LPEEVVANSYGLPREQAR.Q	N	33.01	4030.9507	37	1.0	1008.7460	4	37.29	27	2024	OB4055.raw	0	0	0	479	515	
R.Q(-17.03)LKNNNPFKFFVPPSEQSLR.A	N	32.75	2372.2275	20	0.7	791.7503	3	33.16	27	1745	OB4055.raw	4.47E4	1	1	516	535	Pyro-glu from Q
R.Q(-17.03)LKNN(+.98)NPFKFFVPPSEQSLR.A	N	32.63	2373.2117	20	-0.2	792.0776	3	33.73	27	1792	OB4055.raw	5.33E4	1	1	516	535	Pyro-glu from Q
R.SPDIYNP(+15.99)QAGSLK(+14.02)TANELQLNLLILRWLGLSAEYGNLYR.N	Y	32.44	4433.3384	39	-1.5	887.6736	5	42.13	27	2336	OB4055.raw	0	0	0	371	409	
R.QLKNN(+.98)NPFKFFVPPSEQ(+.98)SLR.A	N	32.36	2391.2222	20	2.1	798.0830	3	32.22	27	1666	OB4055.raw	1.46E5	1	1	516	535	Deamidation (NQ)
K.TDSRPSIANLAGEN(+.98)SFIDNLPEEVVANSY.G	N	32.14	3122.4680	29	1.3	1041.8313	3	36.49	27	1985	OB4055.raw	8.84E3	1	1	479	507	Deamidation (NQ)
K.NNNPFKFFVPPSEQSLR.A	N	31.82	2020.0166	17	0.3	674.3463	3	32.31	27	1691	OB4055.raw	1.36E4	1	1	519	535	
K.TDSRPSIAN(+.98)LAGEN(+.98)SFIDN(+.98)LPEEVVANSYGLPREQAR.Q	N	31.53	4031.9348	37	7.8	1008.9988	4	34.84	27	2024	OB4055.raw	2.53E6	1	1	479	515	
R.QLKN(+.98)NN(+.98)PFKFFVPPSEQ(+.98)SLR.A	N	31.46	2392.2063	20	0.8	798.4100	3	32.48	27	1696	OB4055.raw	2.19E5	1	1	516	535	
R.Q(+.98)LKN(+.98)N(+.98)N(+.98)PFKFFVPPSEQ(+.98)SLR.A	N	30.92	2394.1743	20	9.2	799.0727	3	33.15	27	1737	OB4055.raw	6.06E4	1	1	516	535	Deamidation (NQ)
R.WLGLSAEYGNLYRNALFVPHYN(+.98)TN(+.98)AHSIIYALR.G	N	30.70	3837.9268	33	4.2	768.5958	5	35.09	27	1913	OB4055.raw	1.63E5	1	1	397	429	
K.TDSRPSIAN(+.98)LAGENSFIDNLPEEVVAN(+.98)SYGLPR.E	N	30.44	3546.7114	33	1.3	887.6863	4	36.40	27	1979	OB4055.raw	7.87E4	1	1	479	511	
N.ELQLN(+45.99)LLILRWLGLSAEYGNLYR.N	Y	30.26	2792.5046	23	5.0	931.8468	3	44.66	27	2488	OB4055.raw	0	0	0	387	409	Beta-methylthiolat (ND)
R.SPDIYNP(+15.99)QAGSLK(+14.02)TANELQ(+.98)LNLLILRWLGLSAEYGNLYR.N	Y	30.03	4434.3223	39	0.7	887.8724	5	39.55	27	2173	OB4055.raw	0	0	0	371	409	
R.NALFVPHYNTN(+.98)AHSIIYALRGR.A	N	29.99	2527.3083	22	0.0	843.4434	3	31.16	27	1609	OB4055.raw	4.64E4	1	1	410	431	Deamidation (NQ)
R.WLGLSAEYGN(+.98)LYRNALFVPHYN(+.98)TN(+.98)AHSIIYALR.G	N	29.39	3838.9106	33	6.1	768.7941	5	35.52	27	1913	OB4055.raw	0	0	0	397	429	

Peptide	Uniq	-10lgP	Mass	Length	ppm	m/z	z	RT	Fraction	Scan	Source File	Area Digest 5 traka 22 kDa	#Feature	#Feature Digest 5 traka 22 kDa	Start	End	PTM
R.WLGLSAEYGNLYRN(+.98)ALFVPHYNTN(+.98)AHSIIYALR.G	N	28.44	3837.9268	33	4.2	768.5958	5	35.09	27	1907	OB4055.raw	1.63E5	1	1	397	429	
R.NALFVPHYNTNAHSIIYALRG.R	N	27.77	2370.2231	21	1.4	791.0828	3	31.62	27	1624	OB4055.raw	8.89E4	1	1	410	430	
R.WLGLSAEYGNLYRN(+.98)ALFVPHYN(+.98)TN(+.98)AHSIIYALR.G	N	27.29	3838.9106	33	5.2	768.7934	5	35.43	27	1907	OB4055.raw	0	0	0	397	429	
K.SQSENFEYVAFKTDSRPSIANLAGEN(+.98)SFIDNLPEEVVANSYGLPR.E	N	26.57	4975.3789	45	4.6	1244.8577	4	35.96	27	1941	OB4055.raw	0	0	0	467	511	
N.ELQ(+.98)LNLLILR(+31.99)WLGLSAEYGNLYR.N	Y	26.47	2779.4907	23	3.5	927.5074	3	46.55	27	2601	OB4055.raw	0	0	0	387	409	Dihydroxy
K.SQSENFEYVAFKTDSRPSIAN(+.98)LAG.E	N	26.38	2631.2451	24	1.4	878.0902	3	32.06	27	1672	OB4055.raw	4.56E4	1	1	467	490	Deamidation (NQ)
K.TDSRPSIANLAGENSFIDN(+.98)LPEEVVAN(+.98)SYGLPREQ(+.98)AR.Q	N	26.09	4031.9348	37	7.8	1008.9988	4	34.84	27	1849	OB4055.raw	2.53E6	1	1	479	515	Deamidation (NQ)
K.NIGRNRSPDIYN(+.98)PQAGSLK.T	N	25.83	2100.0710	19	0.2	701.0311	3	25.59	27	1264	OB4055.raw	0	0	0	365	383	
R.SP(+15.99)DIYN(+.98)P(+15.99)Q(+.98)AGSLKTAN(+.98)ELQLNLLILRWLGLSAEYGNLYR.N	Y	25.49	4438.2695	39	3.4	888.6642	5	44.11	27	2457	OB4055.raw	7.01E3	1	1	371	409	Hydroxylation Pro
R.SPDIYNP(+15.99)Q(+.98)AGSLKTAN(+.98)ELQ(+.98)LN(+.98)LLILRWLGLSAEYGNLYR.N	Y	25.48	4423.2588	39	8.7	885.6667	5	38.82	27	2125	OB4055.raw	1.1E6	1	1	371	409	
K.TDSRPSIANLAGEN(+.98)SFIDN(+.98)LPEEVVANSY.G	N	24.92	3123.4519	29	5.2	1042.1633	3	36.99	27	1985	OB4055.raw	2.77E4	1	1	479	507	
R.Q(+.98)LKNN(+.98)NPFKFFVPPSEQ(+.98).S	N	24.89	2035.9890	17	0.0	679.6703	3	33.40	27	1761	OB4055.raw	1.85E5	1	1	516	532	
R.SP(+15.99)DIYNPQAGSLKTANELQLNLLILRWLGLSAEYGNLYR.N	Y	24.77	4419.3228	39	-5.7	1105.8317	4	38.47	27	2249	OB4055.raw	7.68E5	1	1	371	409	
K.N(+.98)IGRNRSPDIYNPQAGSLK.T	N	24.58	2100.0710	19	0.1	701.0310	3	25.67	27	1269	OB4055.raw	0	0	0	365	383	
R.NALFVPHYN(+.98)TNAHSIIYALRGR.A	N	24.45	2527.3083	22	0.4	632.8346	4	31.16	27	1597	OB4055.raw	1.55E6	1	1	410	431	
R.SPDIYNP(+15.99)QAGSLKTANELQLNLLILRWLGLSAEYGNLYR.N	Y	24.45	4419.3228	39	-2.1	884.8700	5	38.37	27	2125	OB4055.raw	1.49E6	1	1	371	409	
K.FFVPPSEQSLR.A	N	24.33	1305.6716	11	-0.4	653.8428	2	29.53	27	1508	OB4055.raw	8.55E3	1	1	525	535	
R.SP(+15.99)DIYN(+.98)P(+15.99)QAGSLKTANELQLNLLILRWLGLSAEYGNLYR.N	Y	24.10	4436.3013	39	-2.6	888.2653	5	48.73	27	2731	OB4055.raw	0	0	0	371	409	Hydroxylation Pro
R.SPDIYN(+.98)P(+15.99)Q(+.98)AGSLKTAN(+.98)ELQ(+.98)LNLLILRWLGLSAEYGNLYR.N	Y	23.95	4423.2588	39	8.7	885.6667	5	38.82	27	2167	OB4055.raw	1.1E6	1	1	371	409	
R.SPDIYN(+.98)P(+15.99)QAGSLKTAN(+.98)ELQ(+.98)LN(+.98)LLILRWLGLSAEYGNLYR.N	Y	23.79	4423.2588	39	8.7	885.6667	5	38.82	27	2137	OB4055.raw	1.1E6	1	1	371	409	
K.TDSRPSIAN(+.98)LAGEN(+.98)SFIDNLPEEVVANSYGLPR.E	N	22.42	3546.7114	33	1.3	887.6863	4	36.40	27	1971	OB4055.raw	7.87E4	1	1	479	511	
N.ELQLNLLILRW(+31.99)LGLSAEYGNLYR.N	Y	22.29	2778.5068	23	-0.8	927.1755	3	49.41	27	2772	OB4055.raw	0	0	0	387	409	
K.SQSENFEYVAFKTDSRPSIAN(+.98)LAGENSFIDNLPEEVVANSYGLPREQ(+.98)AR.Q	N	22.13	5460.6021	49	4.0	1093.1321	5	35.14	27	1888	OB4055.raw	0	0	0	467	515	
**total 77 peptides**																	

tr|Q6IWG5|Q6IWG5_ARAHY

back to list

| Protein Coverage | Supporting Peptides |

Protein Coverage:

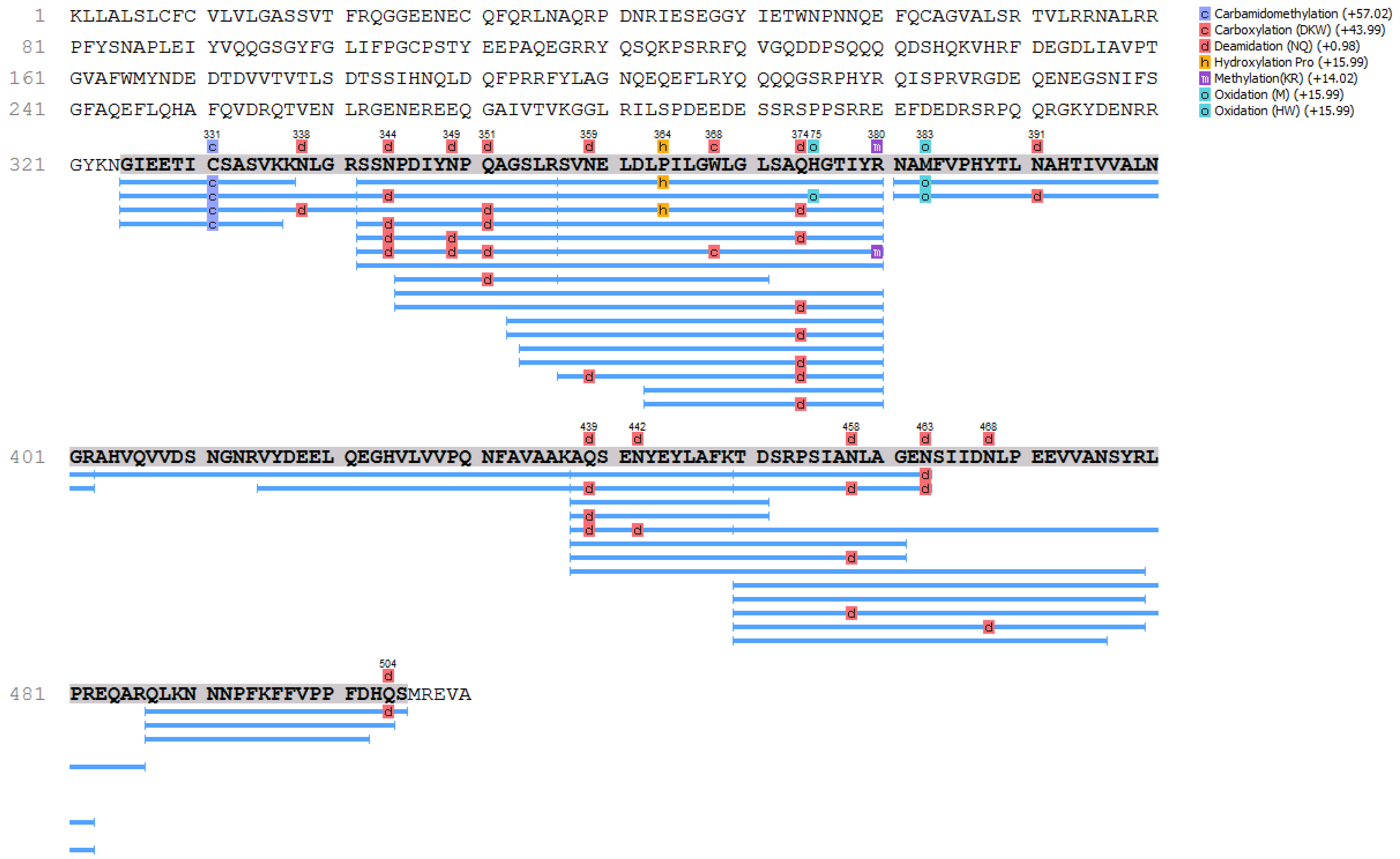
1 KLLALSLCFC VLVLGASSVT FRQGGEENEC QFQRLNAQRP DNRIESEGGY IETWNPNNQE FQCAGVALSR TVLRRNALRR
81 PFYSNAPLEI YVQQGSGYFG LIFPGCPSTY EEPAQEGRRY QSQKPSRRFQ VGQDDPSQQQ QDSHQKVHRF DEGDLIAVPT
161 GVAFWMYNDE DTDVVTVTLS DTSSIHNQLD QFPRRFYLAG NQEQEFLRYQ QQQGSRPHYR QISPRVRGDE QENEGSNIFS
241 GFAQEFLQHA FQVDRQTVEN LRGENEREEQ GAIVTVKGGL RILSPDEEDE SSRSPPSRRE EFDEDRSRPQ QRGKYDENRR
321 GYKNGIEETI CSASVKKNLG RSSNPDIYNP QAGSLRSVNE LDLPILGWLG LSAQHGTIYR NAMFVPHYTL NAHTIVVALN
331 338 344 349 351 359 364 368 374 75 380 383 391
401 GRAHVQVVDS NGNRVYDEEL QEGHVLVVPQ NFAVAAKAQS ENYEYLAFKT DSRPSIANLA GENSIIDNLP EEVVANSYRL
439 442 458 463 468
481 PREQARQLKN NNPFKFFVPP FDHQSMREVA
504
Carbamidomethylation (+57.02)
Carboxylation (DKW) (+43.99)
Deamidation (NQ) (+0.98)
Hydroxylation Pro (+15.99)
Methylation(KR) (+14.02)
Oxidation (M) (+15.99)
Oxidation (HW) (+15.99)

Supporting Peptides:

Peptide	Uniq	-10lgP	Mass	Length	ppm	m/z	z	RT	Fraction	Scan	Source File	Area Digest 5 traka 22 kDa	#Feature	#Feature Digest 5 traka 22 kDa	Start	End	PTM
K.AQSENYEYLAFK.T	Y	89.64	1461.6776	12	-0.4	731.8458	2	30.22	27	1544	OB4055.raw	1.31E5	1	1	438	449	
R.SSNPDIYNPQAGSLR.S	Y	84.93	1617.7747	15	0.4	809.8949	2	27.38	27	1382	OB4055.raw	2.93E5	1	1	342	356	
K.TDSRPSIAN(+.98)LAGENSIIDN(+.98)LPEEVVANSYR.L	Y	83.57	3245.5686	30	2.9	1082.8666	3	35.49	27	1911	OB4055.raw	0	0	0	450	479	Deamidation (NQ)
K.AQ(+.98)SENYEYLAFK.T	Y	81.11	1462.6616	12	-0.8	732.3375	2	30.52	27	1574	OB4055.raw	6.97E4	1	1	438	449	Deamidation (NQ)
R.SSNPDIYNPQ(+.98)AGSLR.S	Y	80.35	1618.7587	15	0.5	810.3870	2	27.96	27	1405	OB4055.raw	4.37E5	1	1	342	356	Deamidation (NQ)
R.SSN(+.98)PDIYNPQAGSLR.S	Y	76.62	1618.7587	15	0.5	810.3870	2	27.96	27	1395	OB4055.raw	4.37E5	1	1	342	356	Deamidation (NQ)
K.TDSRPSIAN(+.98)LAGENSIIDNLPEEVVANSYR.L	Y	74.94	3244.5847	30	3.8	1082.5397	3	34.91	27	1911	OB4055.raw	1.41E5	1	1	450	479	
K.AQSENYEYLAFKTD.S	Y	74.54	1677.7522	14	0.6	839.8839	2	30.60	27	1569	OB4055.raw	3.02E4	1	1	438	451	
R.SSN(+.98)PDIYNPQ(+.98)AGSLR.S	Y	73.68	1619.7427	15	-0.5	810.8782	2	28.36	27	1435	OB4055.raw	1.31E5	1	1	342	356	Deamidation (NQ)
R.NAM(+15.99)FVPHYTLNAHTIVVALNGR.A	Y	69.67	2453.2637	22	0.2	818.7620	3	31.62	27	1635	OB4055.raw	8.15E4	1	1	381	402	Oxidation (M)
R.SSN(+.98)PDIYN(+.98)PQAGSLR.S	Y	66.47	1619.7427	15	1.9	810.8802	2	28.77	27	1460	OB4055.raw	0	0	0	342	356	Deamidation (NQ)
R.NAM(+15.99)FVPHYTLN(+.98)AHTIVVALNGR.A	Y	66.07	2454.2478	22	-0.5	819.0895	3	32.31	27	1685	OB4055.raw	8.68E4	1	1	381	402	Oxidation (M); Deamidation (NQ)
N.GIEETIC(+57.02)SASVK.K	Y	64.68	1292.6282	12	0.1	647.3214	2	27.77	27	1401	OB4055.raw	3.01E4	1	1	325	336	Carbamidomethylation
N.GIEETIC(+57.02)SASVKK.N	Y	64.34	1420.7231	13	0.4	711.3691	2	26.01	27	1292	OB4055.raw	1.49E4	1	1	325	337	Carbamidomethylation
K.AQ(+.98)SEN(+.98)YEYLAFK.T	Y	64.02	1463.6456	12	2.4	732.8318	2	30.94	27	1593	OB4055.raw	0	0	0	438	449	Deamidation (NQ)
R.SSNPDIYN(+.98)PQAGSLR.S	Y	60.32	1618.7587	15	0.5	810.3870	2	27.96	27	1514	OB4055.raw	4.37E5	1	1	342	356	
R.SVNELDLPILGWLGLSAQHGTIYR.N	Y	60.10	2651.4070	24	0.9	884.8104	3	38.37	27	2083	OB4055.raw	1.29E7	6	6	357	380	
N.PDIYNPQ(+.98)AGSLR.S	Y	59.64	1330.6517	12	1.4	666.3341	2	28.16	27	1423	OB4055.raw	1.58E4	1	1	345	356	Deamidation (NQ)
D.LPILGWLGLSAQHGTIYR.N	Y	58.06	1994.1101	18	0.4	665.7109	3	35.26	27	1891	OB4055.raw	8.47E4	1	1	363	380	
K.TDSRPSIANLAGEN(+.98)SIIDNLPEEVVANSYR.L	Y	56.84	3244.5847	30	3.8	1082.5397	3	34.91	27	1856	OB4055.raw	1.41E5	1	1	450	479	
R.SVNELDLPILGWLGLSAQ(+.98)HGTIYR.N	Y	52.54	2652.3911	24	5.4	885.1424	3	48.44	27	2714	OB4055.raw	1.46E3	1	1	357	380	Deamidation (NQ)
K.TDSRPSIANLAGEN(+.98).S	N	50.16	1444.6793	14	0.0	723.3469	2	28.45	27	1439	OB4055.raw	3.06E4	1	1	450	463	Deamidation (NQ)
D.LPILGWLGLSAQ(+.98)HGTIYR.N	Y	49.72	1995.0941	18	-2.1	666.0372	3	35.54	27	1917	OB4055.raw	3.5E4	1	1	363	380	Deamidation (NQ)
K.TDSRPSIAN(+.98)LAGEN(+.98).S	N	46.58	1445.6633	14	-1.6	723.8378	2	29.14	27	1479	OB4055.raw	6.66E3	1	1	450	463	Deamidation (NQ)
R.SSNPDIYN(+.98)PQAGSLRSVN(+.98)ELDLPILGWLGLSAQHGTIYR.N	Y	44.17	4253.1392	39	6.5	1418.7295	3	37.55	27	2053	OB4055.raw	6.57E4	1	1	342	380	
K.TDSRPSIANLAGENSIIDNLPEEVVANSYRLPR.E	Y	43.94	3609.8386	33	0.2	903.4671	4	34.78	27	1862	OB4055.raw	0	0	0	450	482	
R.SSN(+.98)PDIYN(+.98)PQ(+.98)AGSLR.S	Y	43.68	1620.7267	15	-0.6	811.3701	2	28.84	27	1465	OB4055.raw	7.02E3	1	1	342	356	Deamidation (NQ)

Peptide	Uniq	-10lgP	Mass	Length	ppm	m/z	z	RT	Fraction	Scan	Source File	Area Digest 5 traka 22 kDa	#Feature	#Feature Digest 5 traka 22 kDa	Start	End	PTM
G.SLRSVNELDLPILGWLGLSAQ(+.98)HGTIYR.N	Y	43.61	3008.6084	27	0.8	753.1600	4	37.37	27	2029	OB4055.raw	0	0	0	354	380	Deamidation (NQ)
K.TDSRPSIANLAGEN(+.98)SIIDNLPEEVVANSYRLPR.E	Y	43.38	3610.8225	33	4.7	903.7171	4	35.09	27	1897	OB4055.raw	9.37E5	1	1	450	482	
G.SLRSVNELDLPILGWLGLSAQHGTIYR.N	Y	43.13	3007.6243	27	0.6	752.9138	4	37.16	27	2029	OB4055.raw	3.93E5	1	1	354	380	
R.SVNELDLP(+15.99)ILGWLGLSAQHGTIYR.N	Y	40.14	2667.4021	24	2.9	890.1439	3	36.12	27	2042	OB4055.raw	2.1E5	1	1	357	380	Hydroxylation Pro
R.SVNELDLPILGWLGL.S	Y	39.12	1637.9028	15	0.5	819.9591	2	44.80	27	2501	OB4055.raw	6.92E3	1	1	357	371	
K.AQ(+.98)SENYEYLAFKTD.S	Y	38.11	1678.7362	14	0.0	840.3754	2	30.88	27	1582	OB4055.raw	1.11E4	1	1	438	451	Deamidation (NQ)
K.TDSRPSIANLAGENSIIDNLPEEVVAN(+.98)SYRLPR.E	Y	37.80	3610.8225	33	4.7	903.7171	4	35.09	27	1879	OB4055.raw	9.37E5	1	1	450	482	
R.SSNPDIYNPQAGSLRSVN(+.98)ELDLPILGWLGLSAQHGTIYR.N	Y	37.68	4252.1553	39	2.2	1064.0485	4	37.74	27	2035	OB4055.raw	5.25E6	1	1	342	380	
N.GIEETIC(+57.02)SASVKKNLGR.S	Y	37.38	1860.9727	17	-0.7	621.3311	3	26.79	27	1334	OB4055.raw	6.05E5	2	2	325	341	Carbamidomethylation
R.SSNPDIYN(+.98)PQ(+.98)AGSLRSVNELDLPILGWLGLSAQ(+.98)HGTIYR.N	Y	37.37	4254.1230	39	6.1	1064.5446	4	38.23	27	2084	OB4055.raw	0	0	0	342	380	
R.SVNE(+28.03)LDLPILGWLGLSAQHGTIYR.N	Y	37.16	2679.4385	24	0.7	894.1541	3	39.18	27	2145	OB4055.raw	1.79E5	1	1	357	380	
N.PDIYNPQ(+.98)AGSLRSVNELDLPILGWLGLSAQ(+.98)HGTIYR.N	Y	36.48	3965.0322	36	0.0	992.2654	4	38.18	27	2080	OB4055.raw	0	0	0	345	380	Deamidation (NQ)
K.AQSEN(+.98)YEYLAFKTDSRPSIANLAGEN(+.98)SIIDNLPEEVVANSYR.L	Y	35.66	4689.2358	42	1.6	1173.3181	4	35.05	27	1882	OB4055.raw	0	0	0	438	479	
R.SSNPDIYNPQAGSLRSVN(+.98)ELDLPILGWLGLSAQ(+.98)HGTIYR.N	Y	35.41	4253.1392	39	3.6	1064.2959	4	37.85	27	2059	OB4055.raw	0	0	0	342	380	
K.AQSEN(+.98)YEYLAFKTDSRPSIANLAGENSIIDNLPEEVVANSYR.L	Y	35.01	4688.2515	42	3.5	1173.0742	4	34.84	27	1882	OB4055.raw	1.39E5	1	1	438	479	
R.SVNELDLPILGWLGLSAQH(+15.99)GTIYR.N	Y	34.68	2667.4021	24	2.9	890.1439	3	36.12	27	2020	OB4055.raw	2.1E5	1	1	357	380	Oxidation (HW)
R.SVN(+.98)ELDLPILGWLGLSAQ(+.98)HGTIYR.N	Y	34.39	2653.3750	24	4.4	885.4695	3	48.58	27	2714	OB4055.raw	1.23E4	1	1	357	380	Deamidation (NQ)
R.Q(+.98)LKNNNPFKFFVPPFD.H	Y	34.30	1951.9832	16	-0.2	651.6682	3	35.36	27	1919	OB4055.raw	4.32E4	1	1	487	502	
R.SSNPDIYNPQ(+.98)AGSLRSVN(+.98)ELDLPILGWLGLSAQHGTIYR.N	Y	33.43	4253.1392	39	4.2	1064.2965	4	40.47	27	2234	OB4055.raw	0	0	0	342	380	
R.SVNELDLP(+31.99)ILGWLGLSAQHGTIYR.N	Y	33.32	2683.3970	24	2.2	895.4749	3	36.49	27	1977	OB4055.raw	9.71E4	1	1	357	380	
R.VYDEELQEGHVLVVPQN(+.98)FAVAAK.A	Y	32.32	2555.2908	23	-0.6	852.7704	3	32.15	27	1679	OB4055.raw	6.74E4	1	1	415	437	
R.SVNELDLPILGW(+15.99)LGLSAQHGTIYR.N	Y	31.61	2667.4021	24	2.9	890.1439	3	36.12	27	1984	OB4055.raw	2.1E5	1	1	357	380	
R.SSNPDIYNPQAGSLRSVNELDLPILGWLGLSAQHGTIYR.N	Y	31.56	4251.1714	39	0.2	1063.8003	4	37.09	27	2011	OB4055.raw	0	0	0	342	380	
K.AQSENYEYLAFKTDSRPSIANLAGENSIIDNLPEEVVANSYR.L	Y	30.66	4687.2676	42	0.6	1172.8248	4	34.79	27	1863	OB4055.raw	0	0	0	438	479	
N.PDIYNPQ(+.98)AGSLRSVN(+.98)ELDLPILGWLGLSAQHGTIYR.N	Y	29.70	3965.0322	36	0.2	992.2655	4	37.97	27	2067	OB4055.raw	0	0	0	345	380	
K.TDSRPSIAN(+.98)LAGEN(+.98)SIIDNLPEEVVANSYRLPR.E	Y	29.51	3611.8066	33	2.1	903.9608	4	35.46	27	1909	OB4055.raw	0	0	0	450	482	Deamidation (NQ)
R.SVNELDLP(+15.99)ILGWLGLSAQ(+.98)HGTIYR.N	Y	28.70	2668.3860	24	5.4	890.4741	3	36.95	27	2002	OB4055.raw	0	0	0	357	380	Hydroxylation Pro; Deamidation (NQ)

Peptide	Uniq	-10lgP	Mass	Length	ppm	m/z	z	RT	Fraction	Scan	Source File	Area Digest 5 traka 22 kDa	#Feature	#Feature Digest 5 traka 22 kDa	Start	End	PTM
A.GSLRSVNELDLPILGWLGLSAQ(+.98)HGTIYR.N	Y	28.67	3065.6296	28	-1.4	767.4136	4	37.53	27	2039	OB4055.raw	0	0	0	353	380	Deamidation (NQ)
A.GSLRSVNELDLPILGWLGLSAQHGTIYR.N	Y	28.27	3064.6458	28	0.2	767.1689	4	36.98	27	2039	OB4055.raw	8.69E4	1	1	353	380	
K.TDSRPSIANLAGENSIIDN(+.98)LPEEVVAN(+.98).S	N	28.08	2839.3723	27	3.1	947.4677	3	35.64	27	1904	OB4055.raw	3.6E4	1	1	450	476	
K.AQSENYEYLAFKTDSRPSIANLAG.E	Y	27.92	2644.2769	24	0.1	882.4330	3	31.53	27	1634	OB4055.raw	5.91E4	1	1	438	461	
R.SVNE(+53.92)LDLPILGWLGLSAQHGTIYR.N	Y	27.19	2705.3264	24	0.6	677.3393	4	38.11	27	2079	OB4055.raw	6.3E4	1	1	357	380	
R.SSNPDIYN(+.98)PQ(+.98)AGSLRSVNELDLPILGWLGLSAQHGTIYR.N	Y	26.92	4253.1392	39	6.9	1064.2994	4	44.74	27	2493	OB4055.raw	0	0	0	342	380	
R.SSNPDIYNPQ(+.98)AGSLRSVNELDLPILGWLGLSAQHGTIYR.N	Y	26.18	4252.1553	39	2.2	1064.0485	4	45.81	27	2557	OB4055.raw	5.25E6	1	1	342	380	
R.SSNPDIYNPQ(+.98)AGSLRSVNELDLPILGWLGLSAQ(+.98)HGTIYR.N	Y	26.15	4253.1392	39	7.9	1064.3004	4	48.94	27	2743	OB4055.raw	0	0	0	342	380	
R.SSNPDIYNPQAGSLRSVNELDLPILGWLGLSAQ(+.98)HGTIYR.N	Y	26.13	4252.1553	39	-4.2	1064.0416	4	44.43	27	2474	OB4055.raw	5.25E6	1	1	342	380	
R.SVNELDLPILGW(+43.99)LGLSAQHGTIYR(+14.02).N	Y	25.59	2709.4126	24	7.5	904.1516	3	39.99	27	2204	OB4055.raw	0	0	0	357	380	Carboxylation (DKW); Methylation(KR)
N.PDIYNPQAGSLRSVN(+.98)ELDLPILGWLGLSAQHGTIYR.N	Y	25.40	3964.0481	36	-0.3	992.0190	4	37.64	27	2056	OB4055.raw	1.51E5	1	1	345	380	
K.AQSENYEYLAFKTDSRPSIAN(+.98)LAG.E	Y	25.12	2645.2607	24	-0.7	882.7603	3	31.98	27	1661	OB4055.raw	8.26E4	1	1	438	461	Deamidation (NQ)
R.QLKN(+.98)N(+.98)NPFKFFVPPFDHQ(+.98)S.M	Y	24.58	2306.1008	19	-1.4	577.5317	4	33.89	27	1811	OB4055.raw	1.19E5	1	1	487	505	Deamidation (NQ)
R.Q(+.98)LKN(+.98)NNPFKFFVPPFD.H	Y	24.10	1952.9672	16	-0.2	651.9962	3	36.02	27	1945	OB4055.raw	6.23E4	1	1	487	502	
K.TDSRPSIANLAGENSIIDNLPEEVVANSYRLPREQ(+.98)AR.Q	Y	24.08	4095.0620	37	2.6	820.0218	5	33.97	27	1787	OB4055.raw	5.2E5	1	1	450	486	
R.SSNPDIYN(+.98)PQAGSLRSVNELDLPILGWLGLSAQHGTIYR.N	Y	23.67	4252.1553	39	-5.5	1064.0403	4	47.28	27	2645	OB4055.raw	0	0	0	342	380	
K.AQSENYEYLAFKTDSRPSIAN(+.98)LAGEN(+.98)SIIDNLPEEVVANSYR.L	Y	23.41	4689.2358	42	5.0	1173.3221	4	35.34	27	1901	OB4055.raw	0	0	0	438	479	
N.GIEETIC(+57.02)SASVKKN(+.98)LGR.S	Y	23.36	1861.9567	17	-0.5	466.4962	4	27.48	27	1381	OB4055.raw	6.34E4	1	1	325	341	Carbamidomethylation; Deamidation (NQ)
R.AHVQVVDSN(+.98)GN(+.98)RVYDEELQEGHVLVVPQNFAVAAK.A	Y	22.90	3832.9019	35	1.0	767.5884	5	30.99	27	1600	OB4055.raw	4.64E4	1	1	403	437	
R.Q(+.98)LKNNN(+.98)PFKFFVPPFDHQ(+.98).S	Y	22.42	2219.0686	18	0.6	555.7748	4	34.12	27	1819	OB4055.raw	9.1E4	1	1	487	504	
R.SSNPDIYN(+.98)PQ(+.98)AGSLRSVN(+.98)ELDLPILGWLGLSAQHGTIYR.N	Y	22.10	4254.1230	39	8.0	1064.5465	4	47.58	27	2663	OB4055.raw	0	0	0	342	380	
R.SVN(+.98)ELDLPILGWLGLSAQHGTIYR.N	Y	22.06	2652.3911	24	7.6	885.1443	3	55.52	27	3147	OB4055.raw	6.57E2	1	1	357	380	
R.SSNPDIYNP(+15.99)Q(+.98)AGSLRSVN(+.98)ELDLPILGWLGLSAQHGTIYR.N	Y	21.89	4269.1343	39	3.1	1068.2942	4	37.49	27	2037	OB4055.raw	0	0	0	342	380	
R.SSNPDIYNPQ(+.98)AGSLRSVN(+.98)ELDLPILGWLGLSAQ(+.98)HGTIYR.N	Y	21.73	4254.1230	39	3.1	1064.5414	4	46.13	27	2576	OB4055.raw	0	0	0	342	380	
**total 78 peptides**																	

tr|Q0GM57|Q0GM57_ARAHY
back to list

| **Protein Coverage** | **Supporting Peptides** |

Protein Coverage:

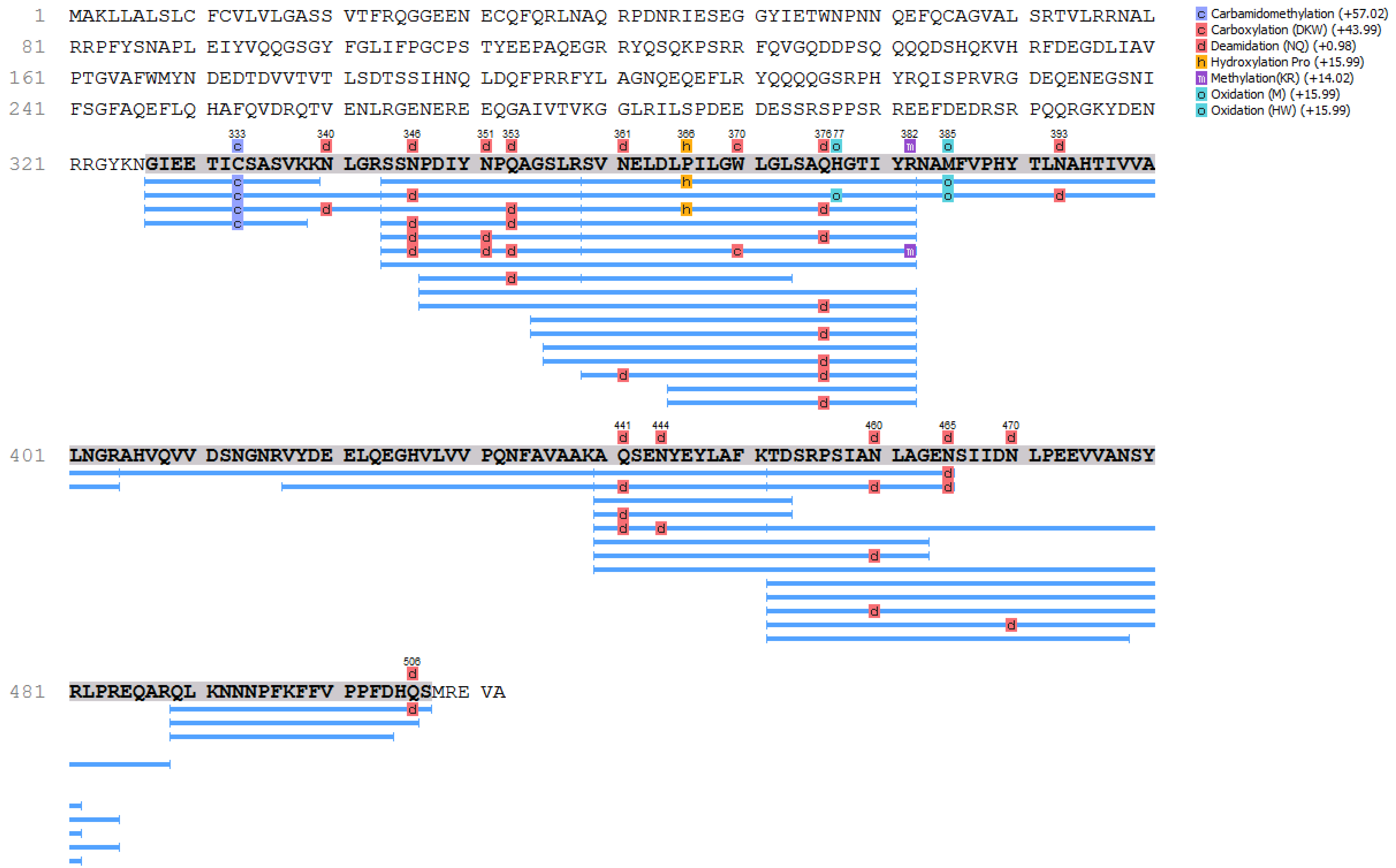
1 MAKLLALSLC FCVLVLGASS VTFRQGGEEN ECQFQRLNAQ RPDNRIESEG GYIETWNPNN QEFQCAGVAL SRTVLRRNAL
81 RRPFYSNAPL EIYVQQGSGY FGLIFPGCPS TYEEPAQEGR RYQSQKPSRR FQVGQDDPSQ QQQDSHQKVH RFDEGDLIAV
161 PTGVAFWMYN DEDTDVVTVT LSDTSSIHNQ LDQFPRRFYL AGNQEQEFLR YQQQQGSRPH YRQISPRVRG DEQENEGSNI
241 FSGFAQEFLQ HAFQVDRQTV ENLRGENERE EQGAIVTVKG GLRILSPDEE DESSRSPPSR REEFDEDRSR PQQRGKYDEN
321 RRGYKNGIEE TICSASVKKN LGRSSNPDIY NPQAGSLRSV NELDLPILGW LGLSAQHGTI YRNAMFVPHY TLNAHTIVVA
401 LNGRAHVQVV DSNGNRVYDE ELQEGHVLVV PQNFAVAAKA QSENYEYLAF KTDSRPSIAN LAGENSIIDN LPEEVVANSY
481 RLPREQARQL KNNNPFKFFV PPFDHQSMRE VA
c Carbamidomethylation (+57.02)
c Carboxylation (DKW) (+43.99)
d Deamidation (NQ) (+0.98)
h Hydroxylation Pro (+15.99)
m Methylation(KR) (+14.02)
o Oxidation (M) (+15.99)
o Oxidation (HW) (+15.99)

Supporting Peptides:

Peptide	Uniq	-10lgP	Mass	Length	ppm	m/z	z	RT	Fraction	Scan	Source File	Area Digest 5 traka 22 kDa	#Feature	#Feature Digest 5 traka 22 kDa	Start	End	PTM
K.AQSENYEYLAFK.T	Y	89.64	1461.6776	12	-0.4	731.8458	2	30.22	27	1544	OB4055.raw	1.31E5	1	1	440	451	
R.SSNPDIYNPQAGSLR.S	Y	84.93	1617.7747	15	0.4	809.8949	2	27.38	27	1382	OB4055.raw	2.93E5	1	1	344	358	
K.TDSRPSIAN(+.98)LAGENSIIDN(+.98)LPEEVVANSYR.L	Y	83.57	3245.5686	30	2.9	1082.8666	3	35.49	27	1911	OB4055.raw	0	0	0	452	481	Deamidation (NQ)
K.AQ(+.98)SENYEYLAFK.T	Y	81.11	1462.6616	12	-0.8	732.3375	2	30.52	27	1574	OB4055.raw	6.97E4	1	1	440	451	Deamidation (NQ)
R.SSNPDIYNPQ(+.98)AGSLR.S	Y	80.35	1618.7587	15	0.5	810.3870	2	27.96	27	1405	OB4055.raw	4.37E5	1	1	344	358	Deamidation (NQ)
R.SSN(+.98)PDIYNPQAGSLR.S	Y	76.62	1618.7587	15	0.5	810.3870	2	27.96	27	1395	OB4055.raw	4.37E5	1	1	344	358	Deamidation (NQ)
K.TDSRPSIAN(+.98)LAGENSIIDNLPEEVVANSYR.L	Y	74.94	3244.5847	30	3.8	1082.5397	3	34.91	27	1911	OB4055.raw	1.41E5	1	1	452	481	
K.AQSENYEYLAFKTD.S	Y	74.54	1677.7522	14	0.6	839.8839	2	30.60	27	1569	OB4055.raw	3.02E4	1	1	440	453	
R.SSN(+.98)PDIYNPQ(+.98)AGSLR.S	Y	73.68	1619.7427	15	-0.5	810.8782	2	28.36	27	1435	OB4055.raw	1.31E5	1	1	344	358	Deamidation (NQ)
R.NAM(+15.99)FVPHYTLNAHTIVVALNGR.A	Y	69.67	2453.2637	22	0.2	818.7620	3	31.62	27	1635	OB4055.raw	8.15E4	1	1	383	404	Oxidation (M)
R.SSN(+.98)PDIYN(+.98)PQAGSLR.S	Y	66.47	1619.7427	15	1.9	810.8802	2	28.77	27	1460	OB4055.raw	0	0	0	344	358	Deamidation (NQ)
R.NAM(+15.99)FVPHYTLN(+.98)AHTIVVALNGR.A	Y	66.07	2454.2478	22	-0.5	819.0895	3	32.31	27	1685	OB4055.raw	8.68E4	1	1	383	404	Oxidation (M); Deamidation (NQ)
N.GIEETIC(+57.02)SASVK.K	Y	64.68	1292.6282	12	0.1	647.3214	2	27.77	27	1401	OB4055.raw	3.01E4	1	1	327	338	Carbamidomethylation
N.GIEETIC(+57.02)SASVKK.N	Y	64.34	1420.7231	13	0.4	711.3691	2	26.01	27	1292	OB4055.raw	1.49E4	1	1	327	339	Carbamidomethylation
K.AQ(+.98)SEN(+.98)YEYLAFK.T	Y	64.02	1463.6456	12	2.4	732.8318	2	30.94	27	1593	OB4055.raw	0	0	0	440	451	Deamidation (NQ)
R.SSNPDIYN(+.98)PQAGSLR.S	Y	60.32	1618.7587	15	0.5	810.3870	2	27.96	27	1514	OB4055.raw	4.37E5	1	1	344	358	
R.SVNELDLPILGWLGLSAQHGTIYR.N	Y	60.10	2651.4070	24	0.9	884.8104	3	38.37	27	2083	OB4055.raw	1.29E7	6	6	359	382	
N.PDIYNPQ(+.98)AGSLR.S	Y	59.64	1330.6517	12	1.4	666.3341	2	28.16	27	1423	OB4055.raw	1.58E4	1	1	347	358	Deamidation (NQ)
D.LPILGWLGLSAQHGTIYR.N	Y	58.06	1994.1101	18	0.4	665.7109	3	35.26	27	1891	OB4055.raw	8.47E4	1	1	365	382	
K.TDSRPSIANLAGEN(+.98)SIIDNLPEEVVANSYR.L	Y	56.84	3244.5847	30	3.8	1082.5397	3	34.91	27	1856	OB4055.raw	1.41E5	1	1	452	481	
R.SVNELDLPILGWLGLSAQ(+.98)HGTIYR.N	Y	52.54	2652.3911	24	5.4	885.1424	3	48.44	27	2714	OB4055.raw	1.46E3	1	1	359	382	Deamidation (NQ)
K.TDSRPSIANLAGEN(+.98).S	N	50.16	1444.6793	14	0.0	723.3469	2	28.45	27	1439	OB4055.raw	3.06E4	1	1	452	465	Deamidation (NQ)
D.LPILGWLGLSAQ(+.98)HGTIYR.N	Y	49.72	1995.0941	18	-2.1	666.0372	3	35.54	27	1917	OB4055.raw	3.5E4	1	1	365	382	Deamidation (NQ)
K.TDSRPSIAN(+.98)LAGEN(+.98).S	N	46.58	1445.6633	14	-1.6	723.8378	2	29.14	27	1479	OB4055.raw	6.66E3	1	1	452	465	Deamidation (NQ)
R.SSNPDIYN(+.98)PQAGSLRSVN(+.98)ELDLPILGWLGLSAQHGTIYR.N	Y	44.17	4253.1392	39	6.5	1418.7295	3	37.55	27	2053	OB4055.raw	6.57E4	1	1	344	382	
K.TDSRPSIANLAGENSIIDNLPEEVVANSYRLPR.E	Y	43.94	3609.8386	33	0.2	903.4671	4	34.78	27	1862	OB4055.raw	0	0	0	452	484	
R.SSN(+.98)PDIYN(+.98)PQ(+.98)AGSLR.S	Y	43.68	1620.7267	15	-0.6	811.3701	2	28.84	27	1465	OB4055.raw	7.02E3	1	1	344	358	Deamidation (NQ)

Peptide	Uniq	-10lgP	Mass	Length	ppm	m/z	z	RT	Fraction	Scan	Source File	Area Digest 5 traka 22 kDa	#Feature	#Feature Digest 5 traka 22 kDa	Start	End	PTM
G.SLRSVNELDLPILGWLGLSAQ(+.98)HGTIYR.N	Y	43.61	3008.6084	27	0.8	753.1600	4	37.37	27	2029	OB4055.raw	0	0	0	356	382	Deamidation (NQ)
K.TDSRPSIANLAGEN(+.98)SIIDNLPEEVVANSYRLPR.E	Y	43.38	3610.8225	33	4.7	903.7171	4	35.09	27	1897	OB4055.raw	9.37E5	1	1	452	484	
G.SLRSVNELDLPILGWLGLSAQHGTIYR.N	Y	43.13	3007.6243	27	0.6	752.9138	4	37.16	27	2029	OB4055.raw	3.93E5	1	1	356	382	
R.SVNELDLP(+15.99)ILGWLGLSAQHGTIYR.N	Y	40.14	2667.4021	24	2.9	890.1439	3	36.12	27	2042	OB4055.raw	2.1E5	1	1	359	382	Hydroxylation Pro
R.SVNELDLPILGWLGL.S	Y	39.12	1637.9028	15	0.5	819.9591	2	44.80	27	2501	OB4055.raw	6.92E3	1	1	359	373	
K.AQ(+.98)SENYEYLAFKTD.S	Y	38.11	1678.7362	14	0.0	840.3754	2	30.88	27	1582	OB4055.raw	1.11E4	1	1	440	453	Deamidation (NQ)
K.TDSRPSIANLAGENSIIDNLPEEVVAN(+.98)SYRLPR.E	Y	37.80	3610.8225	33	4.7	903.7171	4	35.09	27	1879	OB4055.raw	9.37E5	1	1	452	484	
R.SSNPDIYNPQAGSLRSVN(+.98)ELDLPILGWLGLSAQHGTIYR.N	Y	37.68	4252.1553	39	2.2	1064.0485	4	37.74	27	2035	OB4055.raw	5.25E6	1	1	344	382	
N.GIEETIC(+57.02)SASVKKNLGR.S	Y	37.38	1860.9727	17	-0.7	621.3311	3	26.79	27	1334	OB4055.raw	6.05E5	2	2	327	343	Carbamidomethylation
R.SSNPDIYN(+.98)PQ(+.98)AGSLRSVNELDLPILGWLGLSAQ(+.98)HGTIYR.N	Y	37.37	4254.1230	39	6.1	1064.5446	4	38.23	27	2084	OB4055.raw	0	0	0	344	382	
R.SVNE(+28.03)LDLPILGWLGLSAQHGTIYR.N	Y	37.16	2679.4385	24	0.7	894.1541	3	39.18	27	2145	OB4055.raw	1.79E5	1	1	359	382	
N.PDIYNPQ(+.98)AGSLRSVNELDLPILGWLGLSAQ(+.98)HGTIYR.N	Y	36.48	3965.0322	36	0.0	992.2654	4	38.18	27	2080	OB4055.raw	0	0	0	347	382	Deamidation (NQ)
K.AQSEN(+.98)YEYLAFKTDSRPSIANLAGEN(+.98)SIIDNLPEEVVANSYR.L	Y	35.66	4689.2358	42	1.6	1173.3181	4	35.05	27	1882	OB4055.raw	0	0	0	440	481	
R.SSNPDIYNPQAGSLRSVN(+.98)ELDLPILGWLGLSAQ(+.98)HGTIYR.N	Y	35.41	4253.1392	39	3.6	1064.2959	4	37.85	27	2059	OB4055.raw	0	0	0	344	382	
K.AQSEN(+.98)YEYLAFKTDSRPSIANLAGENSIIDNLPEEVVANSYR.L	Y	35.01	4688.2515	42	3.5	1173.0742	4	34.84	27	1882	OB4055.raw	1.39E5	1	1	440	481	
R.SVNELDLPILGWLGLSAQH(+15.99)GTIYR.N	Y	34.68	2667.4021	24	2.9	890.1439	3	36.12	27	2020	OB4055.raw	2.1E5	1	1	359	382	Oxidation (HW)
R.SVN(+.98)ELDLPILGWLGLSAQ(+.98)HGTIYR.N	Y	34.39	2653.3750	24	4.4	885.4695	3	48.58	27	2714	OB4055.raw	1.23E4	1	1	359	382	Deamidation (NQ)
R.Q(+.98)LKNNNPFKFFVPPFD.H	Y	34.30	1951.9832	16	-0.2	651.6682	3	35.36	27	1919	OB4055.raw	4.32E4	1	1	489	504	
R.SSNPDIYNPQ(+.98)AGSLRSVN(+.98)ELDLPILGWLGLSAQHGTIYR.N	Y	33.43	4253.1392	39	4.2	1064.2965	4	40.47	27	2234	OB4055.raw	0	0	0	344	382	
R.SVNELDLP(+31.99)ILGWLGLSAQHGTIYR.N	Y	33.32	2683.3970	24	2.2	895.4749	3	36.49	27	1977	OB4055.raw	9.71E4	1	1	359	382	
R.VYDEELQEGHVLVVPQN(+.98)FAVAAK.A	Y	32.32	2555.2908	23	-0.6	852.7704	3	32.15	27	1679	OB4055.raw	6.74E4	1	1	417	439	
R.SVNELDLPILGW(+15.99)LGLSAQHGTIYR.N	Y	31.61	2667.4021	24	2.9	890.1439	3	36.12	27	1984	OB4055.raw	2.1E5	1	1	359	382	
R.SSNPDIYNPQAGSLRSVNELDLPILGWLGLSAQHGTIYR.N	Y	31.56	4251.1714	39	0.2	1063.8003	4	37.09	27	2011	OB4055.raw	0	0	0	344	382	
K.AQSENYEYLAFKTDSRPSIANLAGENSIIDNLPEEVVANSYR.L	Y	30.66	4687.2676	42	0.6	1172.8248	4	34.79	27	1863	OB4055.raw	0	0	0	440	481	
N.PDIYNPQ(+.98)AGSLRSVN(+.98)ELDLPILGWLGLSAQHGTIYR.N	Y	29.70	3965.0322	36	0.2	992.2655	4	37.97	27	2067	OB4055.raw	0	0	0	347	382	
K.TDSRPSIAN(+.98)LAGEN(+.98)SIIDNLPEEVVANSYRLPR.E	Y	29.51	3611.8066	33	2.1	903.9608	4	35.46	27	1909	OB4055.raw	0	0	0	452	484	Deamidation (NQ)
R.SVNELDLP(+15.99)ILGWLGLSAQ(+.98)HGTIYR.N	Y	28.70	2668.3860	24	5.4	890.4741	3	36.95	27	2002	OB4055.raw	0	0	0	359	382	Hydroxylation Pro; Deamidation (NQ)

Peptide	Uniq	-10lgP	Mass	Length	ppm	m/z	z	RT	Fraction	Scan	Source File	Area Digest 5 traka 22 kDa	#Feature	#Feature Digest 5 traka 22 kDa	Start	End	PTM
A.GSLRSVNELDLPILGWLGLSAQ(+.98)HGTIYR.N	Y	28.67	3065.6296	28	-1.4	767.4136	4	37.53	27	2039	OB4055.raw	0	0	0	355	382	Deamidation (NQ)
A.GSLRSVNELDLPILGWLGLSAQHGTIYR.N	Y	28.27	3064.6458	28	0.2	767.1689	4	36.98	27	2039	OB4055.raw	8.69E4	1	1	355	382	
K.TDSRPSIANLAGENSIIDN(+.98)LPEEVVAN(+.98).S	N	28.08	2839.3723	27	3.1	947.4677	3	35.64	27	1904	OB4055.raw	3.6E4	1	1	452	478	
K.AQSENYEYLAFKTDSRPSIANLAG.E	Y	27.92	2644.2769	24	0.1	882.4330	3	31.53	27	1634	OB4055.raw	5.91E4	1	1	440	463	
R.SVNE(+53.92)LDLPILGWLGLSAQHGTIYR.N	Y	27.19	2705.3264	24	0.6	677.3393	4	38.11	27	2079	OB4055.raw	6.3E4	1	1	359	382	
R.SSNPDIYN(+.98)PQ(+.98)AGSLRSVNELDLPILGWLGLSAQHGTIYR.N	Y	26.92	4253.1392	39	6.9	1064.2994	4	44.74	27	2493	OB4055.raw	0	0	0	344	382	
R.SSNPDIYNPQ(+.98)AGSLRSVNELDLPILGWLGLSAQHGTIYR.N	Y	26.18	4252.1553	39	2.2	1064.0485	4	45.81	27	2557	OB4055.raw	5.25E6	1	1	344	382	
R.SSNPDIYNPQ(+.98)AGSLRSVNELDLPILGWLGLSAQ(+.98)HGTIYR.N	Y	26.15	4253.1392	39	7.9	1064.3004	4	48.94	27	2743	OB4055.raw	0	0	0	344	382	
R.SSNPDIYNPQAGSLRSVNELDLPILGWLGLSAQ(+.98)HGTIYR.N	Y	26.13	4252.1553	39	-4.2	1064.0416	4	44.43	27	2474	OB4055.raw	5.25E6	1	1	344	382	
R.SVNELDLPILGW(+43.99)LGLSAQHGTIYR(+14.02).N	Y	25.59	2709.4126	24	7.5	904.1516	3	39.99	27	2204	OB4055.raw	0	0	0	359	382	Carboxylation (DKW); Methylation(KR)
N.PDIYNPQAGSLRSVN(+.98)ELDLPILGWLGLSAQHGTIYR.N	Y	25.40	3964.0481	36	-0.3	992.0190	4	37.64	27	2056	OB4055.raw	1.51E5	1	1	347	382	
K.AQSENYEYLAFKTDSRPSIAN(+.98)LAG.E	Y	25.12	2645.2607	24	-0.7	882.7603	3	31.98	27	1661	OB4055.raw	8.26E4	1	1	440	463	Deamidation (NQ)
R.QLKN(+.98)N(+.98)NPFKFFVPPFDHQ(+.98)S.M	Y	24.58	2306.1008	19	-1.4	577.5317	4	33.89	27	1811	OB4055.raw	1.19E5	1	1	489	507	Deamidation (NQ)
R.Q(+.98)LKN(+.98)NNPFKFFVPPFD.H	Y	24.10	1952.9672	16	-0.2	651.9962	3	36.02	27	1945	OB4055.raw	6.23E4	1	1	489	504	
K.TDSRPSIANLAGENSIIDNLPEEVVANSYRLPREQ(+.98)AR.Q	Y	24.08	4095.0620	37	2.6	820.0218	5	33.97	27	1787	OB4055.raw	5.2E5	1	1	452	488	
R.SSNPDIYN(+.98)PQAGSLRSVNELDLPILGWLGLSAQHGTIYR.N	Y	23.67	4252.1553	39	-5.5	1064.0403	4	47.28	27	2645	OB4055.raw	0	0	0	344	382	
K.AQSENYEYLAFKTDSRPSIAN(+.98)LAGEN(+.98)SIIDNLPEEVVANSYR.L	Y	23.41	4689.2358	42	5.0	1173.3221	4	35.34	27	1901	OB4055.raw	0	0	0	440	481	
N.GIEETIC(+57.02)SASVKKN(+.98)LGR.S	Y	23.36	1861.9567	17	-0.5	466.4962	4	27.48	27	1381	OB4055.raw	6.34E4	1	1	327	343	Carbamidomethylation; Deamidation (NQ)
R.AHVQVVDSN(+.98)GN(+.98)RVYDEELQEGHVLVVPQNFAVAAK.A	Y	22.90	3832.9019	35	1.0	767.5884	5	30.99	27	1600	OB4055.raw	4.64E4	1	1	405	439	
R.Q(+.98)LKNNN(+.98)PFKFFVPPFDHQ(+.98).S	Y	22.42	2219.0686	18	0.6	555.7748	4	34.12	27	1819	OB4055.raw	9.1E4	1	1	489	506	
R.SSNPDIYN(+.98)PQ(+.98)AGSLRSVN(+.98)ELDLPILGWLGLSAQHGTIYR.N	Y	22.10	4254.1230	39	8.0	1064.5465	4	47.58	27	2663	OB4055.raw	0	0	0	344	382	
R.SVN(+.98)ELDLPILGWLGLSAQHGTIYR.N	Y	22.06	2652.3911	24	7.6	885.1443	3	55.52	27	3147	OB4055.raw	6.57E2	1	1	359	382	
R.SSNPDIYNP(+15.99)Q(+.98)AGSLRSVN(+.98)ELDLPILGWLGLSAQHGTIYR.N	Y	21.89	4269.1343	39	3.1	1068.2942	4	37.49	27	2037	OB4055.raw	0	0	0	344	382	
R.SSNPDIYNPQ(+.98)AGSLRSVN(+.98)ELDLPILGWLGLSAQ(+.98)HGTIYR.N	Y	21.73	4254.1230	39	3.1	1064.5414	4	46.13	27	2576	OB4055.raw	0	0	0	344	382	
**total 78 peptides**																	

tr|O82580|O82580_ARAHY

back to list

| **Protein Coverage** | **Supporting Peptides** |

Protein Coverage:

d Deamidation (NQ) (+0.98)

```
  1   RQQPEENACQ FQRLNAQRPD NRIESEGGYI ETWNPNNQEF ECAGVALSRL VLRRNALRRP FYSNAPQEIF IQQGRGYFGL
 81   IFPGCPRHYE EPHTQGRRSQ SQRPPRRLQG EDQSQQQRDS HQKVHRFDEG DLIAVPTGVA FWLYNDHDTD VVAVSLTDTN
161   NNDNQLDQFP RRFNLAGNTE QEFLRYQQQS RQSRRRSLPY SPYSPQSQPR QEEREFSPRG QHSRRERAGQ EEENEGGNIF
241   SGFTPEFLEQ AFQVDDRQIV QNLRGETESE EEGAIVTVRG GLRILSPDRK RRADEEEEYD EDEYEYDEED RRRGRGSRGR
                           340      349
321   GNGIEETICT ASAKKNIGRN RSPDIYNPQA GSLKTANDLN LLILRWLGPS AEYGNLYRNA LFVAHYNTNA HSIIYRLRGR
                                  437                                      456       461
401   AHVQVVDSNG NRVYDEELQE GHVLVVPQNF AVAGKSQSEN FEYVAFKTDS RPSIANLAGE NSVIDNLPEE VVANSYGLQR
481   EQARQLKNNN PFKFFVPPSQ QSPRAVA
```

Supporting Peptides:

Peptide	Uniq	-10lgP	Mass	Length	ppm	m/z	z	RT	Fraction	Scan	Source File	Area Digest 5 traka 22 kDa	#Feature	#Feature Digest 5 traka 22 kDa	Start	End	PTM
K.SQSENFEYVAFK.T	N	89.61	1447.6619	12	0.4	724.8385	2	30.22	27	1543	OB4055.raw	2.25E5	1	1	436	447	
R.SPDIYNPQ(+.98)AGSLKTANDLNLLILR.W	N	79.73	2626.3965	24	0.0	876.4728	3	33.97	27	1802	OB4055.raw	2.51E5	1	1	342	365	Deamidation (NQ)
R.SPDIYNPQ(+.98)AGSLK.T	N	77.51	1389.6776	13	-0.4	695.8458	2	27.96	27	1407	OB4055.raw	1.22E5	1	1	342	354	
R.SPDIYNPQAGSLKTANDLNLLILR.W	N	75.09	2625.4126	24	0.8	876.1455	3	33.57	27	1780	OB4055.raw	9.38E4	1	1	342	365	
K.SQ(+.98)SENFEYVAFK.T	N	73.36	1448.6459	12	-0.8	725.3297	2	30.52	27	1573	OB4055.raw	1.12E5	1	1	436	447	Deamidation (NQ)
R.SPDIYNPQAGSLK.T	N	72.90	1388.6936	13	-0.4	695.3538	2	27.38	27	1370	OB4055.raw	9.44E4	1	1	342	354	

Peptide	Uniq	-10lgP	Mass	Length	ppm	m/z	z	RT	Fraction	Scan	Source File	Area Digest 5 traka 22 kDa	#Feature	#Feature Digest 5 traka 22 kDa	Start	End	PTM
R.VYDEELQEGHVLVVPQN(+.98)FAVAGK.S	N	68.51	2541.2751	23	-0.6	848.0985	3	31.98	27	1660	OB4055.raw	6.03E4	1	1	413	435	
R.SPDIYNPQ(+.98)AGSLKTAN(+.98)DLNLLILR.W	N	66.93	2627.3806	24	0.7	876.8014	3	34.40	27	1838	OB4055.raw	1.05E5	1	1	342	365	
R.NRSPDIYNPQAGSLKTANDLNLLILR.W	N	66.35	2895.5566	26	-0.1	724.8964	4	32.20	27	1678	OB4055.raw	0	0	0	340	365	
R.NRSPDIYNPQAGSLK.T	N	58.59	1658.8376	15	-1.3	830.4250	2	25.53	27	1270	OB4055.raw	2.35E5	2	2	340	354	
K.SQSENFEYVAFKTD.S	N	55.46	1663.7366	14	0.2	832.8757	2	30.70	27	1581	OB4055.raw	4.71E4	1	1	436	449	
R.SPDIYN(+.98)PQAGSLK.T	N	53.73	1389.6776	13	-0.4	695.8458	2	27.96	27	1456	OB4055.raw	1.22E5	1	1	342	354	
K.TDSRPSIANLAGEN(+.98).S	N	50.16	1444.6793	14	0.0	723.3469	2	28.45	27	1439	OB4055.raw	3.06E4	1	1	448	461	Deamidation (NQ)
R.NRSPDIYNPQ(+.98)AGSLKTANDLNLLILR.W	N	47.56	2896.5405	26	-0.1	725.1423	4	32.56	27	1700	OB4055.raw	3.94E5	1	1	340	365	
K.TDSRPSIAN(+.98)LAGEN(+.98).S	N	46.58	1445.6633	14	-1.6	723.8378	2	29.14	27	1479	OB4055.raw	6.66E3	1	1	448	461	Deamidation (NQ)
R.NRSPDIYN(+.98)PQAGSLKTAN(+.98)DLNLLILR.W	N	45.54	2897.5247	26	1.5	725.3895	4	33.43	27	1754	OB4055.raw	9.81E4	1	1	340	365	
R.VYDEELQEGHVLVVPQNFAVAGK.S	N	43.94	2540.2910	23	1.1	847.7719	3	31.62	27	1640	OB4055.raw	4.8E4	1	1	413	435	
R.NRSPDIYN(+.98)PQ(+.98)AGSLKTAN(+.98)DLNLLILR.W	N	41.16	2898.5085	26	6.2	725.6389	4	33.08	27	1754	OB4055.raw	1.98E5	1	1	340	365	
K.SQSENFEYVAFKTDSRPSIANLAG.E	N	39.51	2630.2612	24	0.5	877.7615	3	31.62	27	1639	OB4055.raw	6.96E4	1	1	436	459	
R.NRSPDIYN(+.98)PQ(+.98)AGSLKTANDLNLLILR.W	N	38.72	2897.5247	26	1.5	725.3895	4	33.43	27	1773	OB4055.raw	9.81E4	1	1	340	365	
R.SPDIYNPQAGSLKTANDLN(+.98)LLILRWLGP(+15.99)SAEYGNLYR.N	Y	36.87	4149.1172	37	5.6	1038.2924	4	42.81	27	2377	OB4055.raw	0	0	0	342	378	
R.NRSPDIYNPQAGSLKTAN(+.98)DLN(+.98)LLILR.W	N	35.98	2897.5247	26	1.5	725.3895	4	33.43	27	1763	OB4055.raw	9.81E4	1	1	340	365	
R.SPDIYNPQAGSLKTANDLNLLILRWLGP(+15.99)SAEYGNLYR.N	Y	31.68	4148.1328	37	1.8	1038.0424	4	44.93	27	2504	OB4055.raw	0	0	0	342	378	
R.N(+.98)RSPDIYN(+.98)PQAGSLKTANDLN(+.98)LLILR.W	N	31.43	2898.5085	26	6.2	725.6389	4	33.08	27	1763	OB4055.raw	1.98E5	1	1	340	365	Deamidation (NQ)
K.SQSENFEYVAFKTDSRPSIAN(+.98)LAG.E	N	26.38	2631.2451	24	1.4	878.0902	3	32.06	27	1672	OB4055.raw	4.56E4	1	1	436	459	Deamidation (NQ)
K.NIGRNRSPDIYN(+.98)PQAGSLK.T	N	25.83	2100.0710	19	0.2	701.0311	3	25.59	27	1264	OB4055.raw	0	0	0	336	354	
K.TANDLNLLILR.W	N	25.78	1254.7296	11	-0.8	628.3716	2	33.40	27	1789	OB4055.raw	2.37E5	1	1	355	365	
K.TDSRPSIANLAGENSVIDNLPEEVVANS(+87.05)YGLQR.E	N	25.32	3614.7998	33	5.8	904.7125	4	35.17	27	1897	OB4055.raw	1.83E5	1	1	448	480	
K.N(+.98)IGRNRSPDIYNPQAGSLK.T	N	24.58	2100.0710	19	0.1	701.0310	3	25.67	27	1269	OB4055.raw	0	0	0	336	354	
R.AHVQVVDSN(+.98)GNRVYDEELQEGHVLVVPQNFAVAGK.S	N	22.21	3817.9023	35	-1.0	955.4819	4	31.24	27	1613	OB4055.raw	0	0	0	401	435	
R.AHVQVVDSN(+.98)GNRVYDEELQ(+.98)EGHVLVVPQ(+.98)NFAVAGK.S	N	22.19	3819.8704	35	5.4	764.9855	5	31.35	27	1643	OB4055.raw	1.55E5	1	1	401	435	
R.AHVQVVDSNGN(+.98)RVYDEELQ(+.98)EGHVLVVPQNFAVAGK.S	N	21.90	3818.8862	35	1.8	764.7859	5	31.36	27	1622	OB4055.raw	0	0	0	401	435	
**total 32 peptides**																	

Peptide List

Prepared with PEAKS ™ (bioinfor.com)

1. Notes Gastric Control Raw peanut Band #6 22-24 kDa (PTM456)

2. Result Statistics

Figure 1. False discovery rate (FDR) curve. X axis is the number of peptide-spectrum matches (PSM) being kept. Y axis is the corresponding FDR.

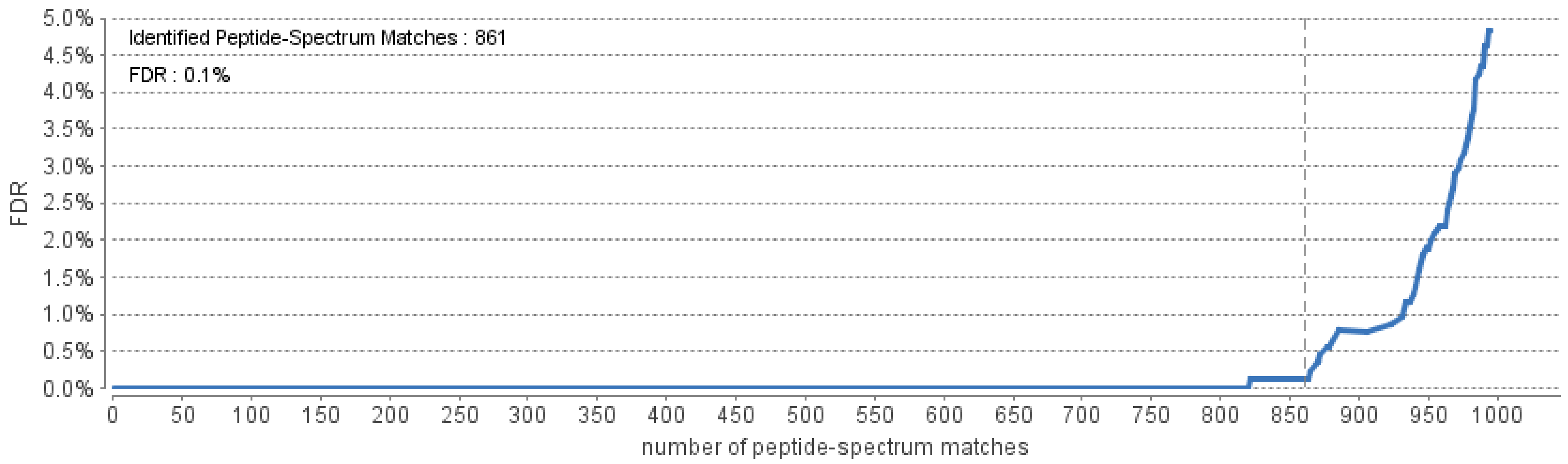

Figure 2. PSM score distribution. **(a)** Distribution of PEAKS peptide score; **(b)** Scatterplot of PEAKS peptide score versus precursor mass error.

(a)

(b)

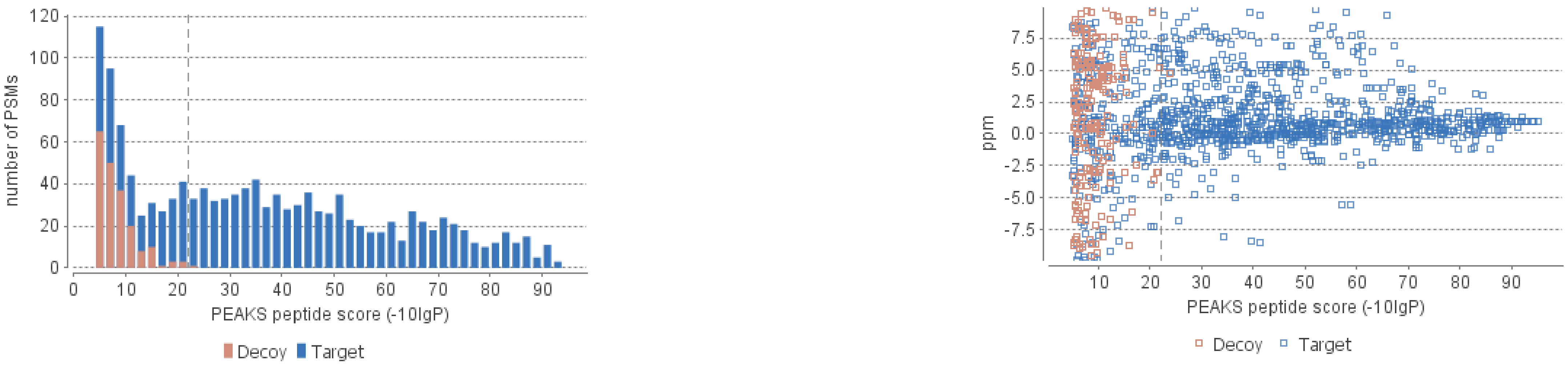

Figure 3. Distribution of peptide feature detection. **(a)** Feature m/z distribution; **(b)** Feature RT distribution

(a)

(b)

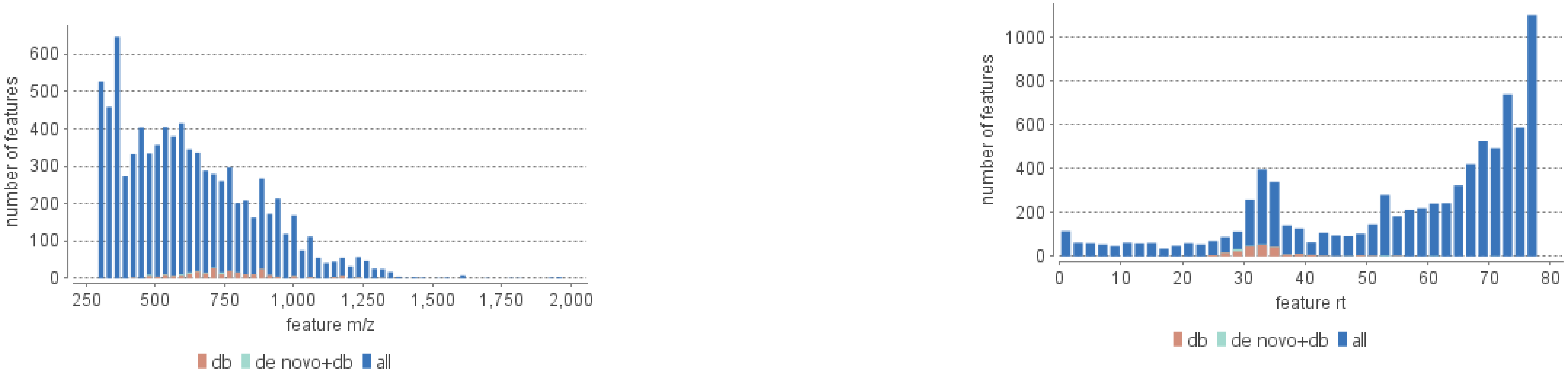

Figure 4. Distribution of identified peptide features. **(a)** Feature abundance distribution **(b)** *De novo* sequencing validation.

(a)

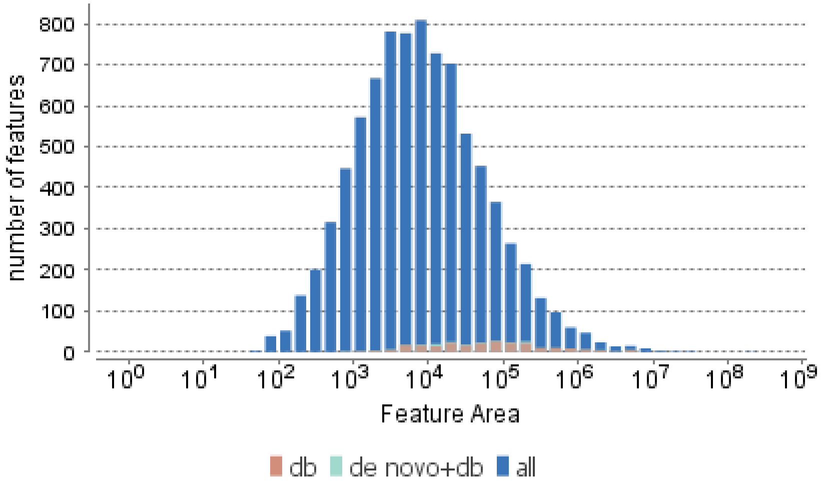

(b)

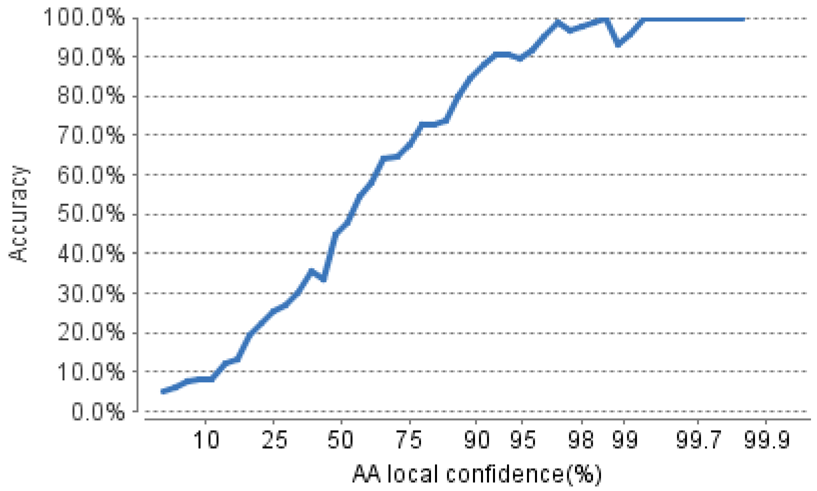

Table 1. Statistics of data.

# of MS scans	2333
# of MS/MS scans	2174
# of Features	8470
# of Chimera scans	256

Table 2. Result filtration parameters.

Peptide -10lgP	≥22
PTM Ascore	≥20
Protein -10lgP	≥20
Proteins unique peptides	≥1
De novo ALC Score	≥50%

Table 4. PTM profile.

Name	ΔMass	Position	#PSM	-10lgP	Abundance	AScore
Deamidation	.98	NQ	378	85.76	3.56E5	57.45
HydPro	15.99	P	28	61.14		1000.00
Carbamidomethyl	57.02	C	22	72.36	6.45E4	1000.00
Oxidation	15.99	M	16	78.91	2.22E5	1000.00
Methylation(others)	14.02	EST	14	64.20		60.92
Acetylation	42.01	N-term	12	48.25	1.13E5	1000.00
Cation:Fe[II]	53.92	DE	9	56.14	5.46E5	111.20

Table 3. Statistics of filtered result.

Peptide-Spectrum Matches	861
Peptide sequences	307
Protein groups	8
Proteins	9
Proteins (#Unique Peptides)	5 (>2); 3 (=2); 1 (=1);
FDR (Peptide-Spectrum Matches)	0.1%
FDR (Peptide Sequences)	0.3%
FDR (Protein Group)	0.0%
De Novo Only Spectra	45
# of identified Features	259
# of identified MS/MS scans	785

Sodium	21.98	DE	9	51.31	7.34E3	28.64
Dehydration	-18.01	STY	8	41.07	3.31E4	26.36
Ethyl	28.03	DE	8	74.46	1.15E5	94.27
Carbamylation	43.01	N-term	7	49.43	1.74E5	1000.00
Methylation(KR)	14.02	R	5	46.67	2.22E4	82.05
Oxidation	15.99	HW	5	42.47	2.2E4	15.08
Propionamide	71.04	C	4	58.59	1.63E4	1000.00
Methyl+Deamidated	15.00	Q	3	44.11	4.55E4	57.45
Carboxymethyl	58.01	C	2	35.77	9.22E3	1000.00
Kynurenin	3.99	W	2	29.24	9.89E4	1000.00
Acetyl	42.01	HY	2	36.41	9.72E4	14.02
Dihydroxy	31.99	W	2	37.09	1.18E4	34.30
Delta:H(5)C(2)	29.04	P	2	36.60	2.63E5	1000.00
Ubiquitin	114.04	T	1	26.88	9.08E3	44.84
Carbofuran	58.03	S	1	25.07	4.21E3	1000.00
Benzoyl	104.03	N-term	1	28.35	7.62E3	1000.00
Aminotyrosine	15.01	Y	1	39.61	1.91E4	63.97

Propionamide	71.04	N-term	1	39.86	4.25E3	1000.00
Amidation	-.98	C-term	1	23.42		1000.00
LG-pyrrole	316.20	N-term	1	27.27		1000.00
Ethanolamine	43.04	E	1	22.60	9.33E4	151.71
Deamidation	.98	R	1	24.62		1000.00
ISD_z+2_ion	-15.01	N-term	1	23.61		1000.00
Acetylation	42.01	K	1	36.23	4.79E4	1000.00
Amidine	41.03	N-term	1	42.04	5.56E3	77.78
MethylamineST	13.03	S	1	39.18	2.63E5	1000.00
Carboxymethyl	58.01	N-term	1	34.07	1.9E5	1000.00
Argglutamicsealde	-43.05	R	1	22.54	4.89E4	26.75

3. Experiment Control

Figure 5. Precursor mass error of peptide-spectrum matches (PSM) in filtered result. **(a)** Distribution of precursor mass error in ppm; **(b)** Scatterplot of precursor m/z versus precursor mass error in ppm.

(a)

(b)

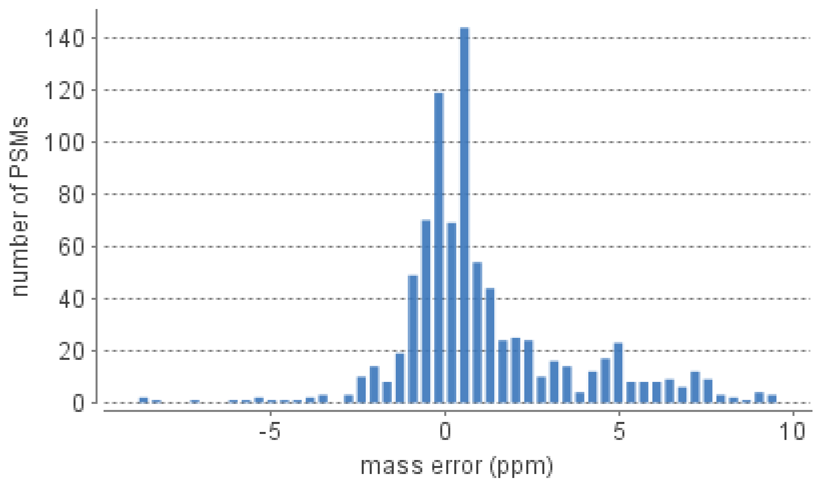

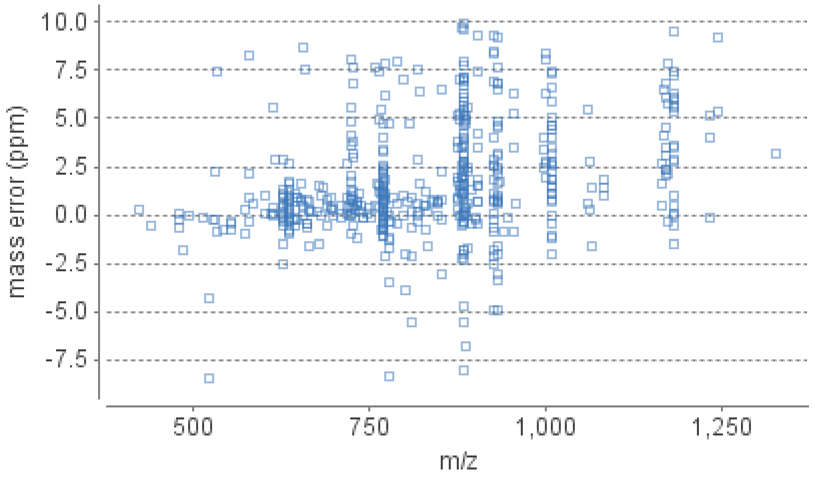

Table 5. Number of identified peptides in each sample by the number of missed cleavages

Missed Cleavages	0	1	2	3	4+
KOntrola 6. traka	164	116	27	0	0

4. Other Information

Table 6. Search parameters.

Search Engine Name: PEAKS
Parent Mass Error Tolerance: 10.0 ppm
Fragment Mass Error Tolerance: 0.5 Da
Precursor Mass Search Type: monoisotopic
Enzyme: Trypsin
Max Missed Cleavages: 2
Digest Mode: Unspecific
Fixed Modifications:

Table 7. Instrument parameters.

Fractions: OB4035.raw
Ion Source: ESI(nano-spray)
Fragmentation Mode: CID, CAD(y and b ions)
MS Scan Mode: FT-ICR/Orbitrap
MS/MS Scan Mode: Linear Ion Trap

Carbamidomethylation: 57.02
Variable Modifications:
Deamidation (NQ): 0.98
Oxidation (M): 15.99
Hydroxylation Pro: 15.99
Acetylation (K): 42.01
Acetylation (Protein N-term): 42.01
Acetylation (N-term): 42.01
Amidation: -0.98
Beta-methylthiolation: 45.99
and 305 more...
Max Variable PTM Per Peptide: 5
Database: Uniprot_Peanut-3818_Jul18
Taxon: All
Contaminant Database: contaminantsMQ_mar19
Searched Entry: 1723
FDR Estimation: Enabled
De novo score (ALC%) threshold: 15
Peptide hit threshold (-10logP): 30.0
Peaks run ID: 438
Merge Options: no merge
Precursor Options: corrected
Charge Options: no correction
Filter Charge: 2 - 8
Process: true
Associate chimera: yes

Protein List

Protein Accession Contains:
Protein Description Contains:
Peptide Sample Area >=
Protein Ptm Contains:

Protein Group	Protein ID	Accession	-10lgP	Coverage (%)	Coverage (%) KOntrola 6. traka	Area KOntrola 6. traka	#Peptides	#Unique	#Spec KOntrola 6. traka	PTM	Avg. Mass	Description
**3**	20581	tr|Q647H3|Q647H3_ARAHY	301.28	34	34	7.31E6	32	4	403	Y	61532	Arachin Ahy-2 OS=Arachis hypogaea OX=3818 PE=2 SV=1
**4**	20578	tr|B5TYU1|B5TYU1_ARAHY	293.74	32	32	1.01E6	30	3	400	Y	60624	Arachin Arah3 isoform OS=Arachis hypogaea OX=3818 PE=1 SV=1
**7**	20576	tr|Q9FZ11|Q9FZ11_ARAHY	286.21	26	26	2.2E5	25	2	367	Y	60449	Gly1 OS=Arachis hypogaea OX=3818 GN=Gly1 PE=2 SV=1
**6**	20582	tr|Q8LKN1|Q8LKN1_ARAHY	277.56	31	31	3.61E5	30	2	366	Y	61738	Allergen Arah3/Arah4 OS=Arachis hypogaea OX=3818 PE=3 SV=1
**9**	20593	tr|Q6IWG5|Q6IWG5_ARAHY	258.08	31	31	1.34E7	20	15	198	Y	58061	Glycinin (Fragment) OS=Arachis hypogaea OX=3818 PE=2 SV=1
**9**	20594	tr|Q0GM57|Q0GM57_ARAHY	258.08	31	31	1.34E7	20	15	198	Y	58263	Iso-Ara h3 OS=Arachis hypogaea OX=3818 PE=2 SV=1
**10**	20590	tr|O82580|O82580_ARAHY	251.87	22	22	6.69E5	20	3	159	Y	58350	Glycinin (Fragment) OS=Arachis hypogaea OX=3818 GN=Arah3 PE=2 SV=1
**11**	20968	Q647H2|AHY3_ARAHY	130.71	7	7	2.17E5	4	2	12	Y	54569	Arachin Ahy-3 OS=Arachis hypogaea OX=3818 PE=1 SV=1
**19**	21915	tr|J9QGM3|J9QGM3_ARAHY	27.75	11	11	5.78E3	1	1	1	Y	27626	Chalcone-flavonone isomerase family protein OS=Arachis hypogaea OX=3818 GN=CHI-I PE=2 SV=1
**total 9 proteins**												

tr|Q647H3|Q647H3_ARAHY
back to list

| Protein Coverage | Supporting Peptides |
Protein Coverage:

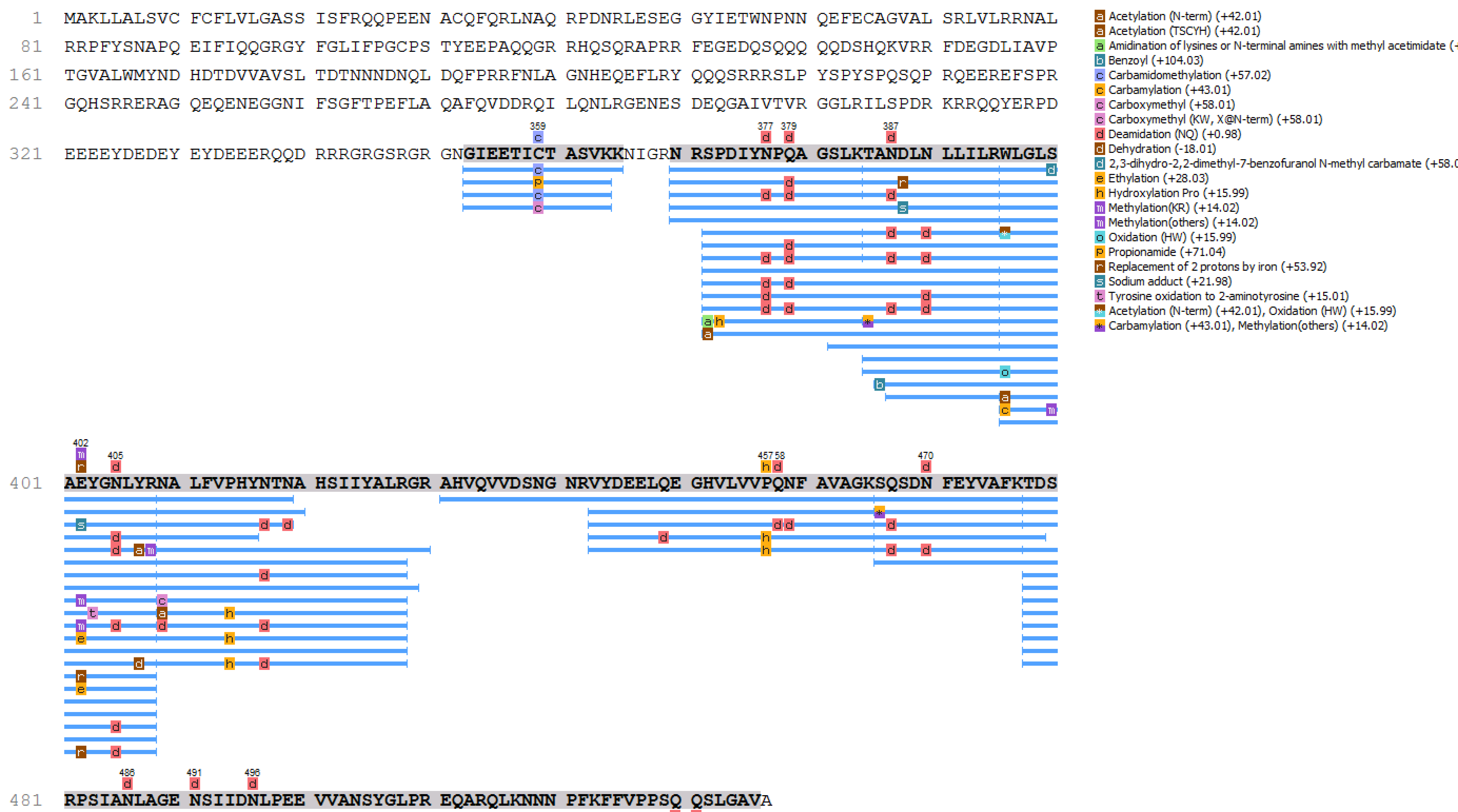

1 MAKLLALSVC FCFLVLGASS ISFRQQPEEN ACQFQRLNAQ RPDNRLESEG GYIETWNPNN QEFECAGVAL SRLVLRRNAL
81 RRPFYSNAPQ EIFIQQGRGY FGLIFPGCPS TYEEPAQQGR RHQSQRAPRR FEGEDQSQQQ QQDSHQKVRR FDEGDLIAVP
161 TGVALWMYND HDTDVVAVSL TDTNNNDNQL DQFPRRFNLA GNHEQEFLRY QQQSRRRSLP YSPYSPQSQP RQEEREFSPR
241 GQHSRRERAG QEQENEGGNI FSGFTPEFLA QAFQVDDRQI LQNLRGENES DEQGAIVTVR GGLRILSPDR KRRQQYERPD
359 377 379 387
321 EEEEYDEDEY EYDEEERQQD RRRGRGSRGR GNGIEETICT ASVKKNIGRN RSPDIYNPQA GSLKTANDLN LLILRWLGLS
402 405 457 58 470
401 AEYGNLYRNA LFVPHYNTNA HSIIYALRGR AHVQVVDSNG NRVYDEELQE GHVLVVPQNF AVAGKSQSDN FEYVAFKTDS
486 491 496
481 RPSIANLAGE NSIIDNLPEE VVANSYGLPR EQARQLKNNN PFKFFVPPSQ QSLGAVA
Acetylation (N-term) (+42.01)
Acetylation (TSCYH) (+42.01)
Amidination of lysines or N-terminal amines with methyl acetimidate (
Benzoyl (+104.03)
Carbamidomethylation (+57.02)
Carbamylation (+43.01)
Carboxymethyl (+58.01)
Carboxymethyl (KW, X@N-term) (+58.01)
Deamidation (NQ) (+0.98)
Dehydration (-18.01)
2,3-dihydro-2,2-dimethyl-7-benzofuranol N-methyl carbamate (+58.0
Ethylation (+28.03)
Hydroxylation Pro (+15.99)
Methylation(KR) (+14.02)
Methylation(others) (+14.02)
Oxidation (HW) (+15.99)
Propionamide (+71.04)
Replacement of 2 protons by iron (+53.92)
Sodium adduct (+21.98)
Tyrosine oxidation to 2-aminotyrosine (+15.01)
Acetylation (N-term) (+42.01), Oxidation (HW) (+15.99)
Carbamylation (+43.01), Methylation(others) (+14.02)

Supporting Peptides:

Peptide	Uniq	-10lgP	Mass	Length	ppm	m/z	z	RT	Fraction	Scan	Source File	Area KOntrola 6. traka	#Feature	#Feature KOntrola 6. traka	Start	End	PTM
R.WLGLSAEYGNLYR.N	N	95.34	1540.7673	13	1.0	771.3917	2	33.34	7	1963	OB4035.raw	1.45E7	5	5	396	408	
K.SQSDNFEYVAFK.T	N	86.92	1433.6462	12	0.9	717.8311	2	30.43	7	1599	OB4035.raw	8.2E5	1	1	466	477	
R.SPDIYNPQAGSLKTANDLNLLILR.W	N	86.57	2625.4126	24	0.8	876.1455	3	33.65	7	1826	OB4035.raw	2.37E5	1	1	372	395	
K.TDSRPSIANLAGENSIIDN(+.98)LPEEVVANSYGLPR.E	Y	84.47	3511.7429	33	3.0	1171.5918	3	35.98	7	1998	OB4035.raw	0	0	0	478	510	Deamidation (NQ)
K.TANDLNLLILRWLGLSAEYGNLYR.N	N	84.26	2777.4863	24	0.8	926.8368	3	39.78	7	2235	OB4035.raw	2.55E5	2	2	385	408	
R.SPDIYNPQ(+.98)AGSLKTANDLNLLILR.W	N	83.88	2626.3965	24	0.8	876.4735	3	34.04	7	1854	OB4035.raw	1.03E6	1	1	372	395	
R.N(+.98)ALFVPHYN(+.98)TNAHSIIYALR.G	N	83.79	2315.1699	20	1.1	772.7314	3	32.66	7	1752	OB4035.raw	2.44E5	1	1	409	428	Deamidation (NQ)
K.SQ(+.98)SDNFEYVAFK.T	N	79.67	1434.6302	12	0.3	718.3226	2	30.79	7	1627	OB4035.raw	3.08E5	1	1	466	477	Deamidation (NQ)
K.TANDLN(+.98)LLILRWLGLSAEYGNLYR.N	N	74.56	2778.4705	24	4.0	927.1678	3	40.91	7	2304	OB4035.raw	0	0	0	385	408	
R.WLGLSAE(+28.03)YGNLYR.N	N	74.46	1568.7987	13	0.9	785.4073	2	34.89	7	1929	OB4035.raw	1.15E5	1	1	396	408	Ethylation
R.NALFVPHYN(+.98)TNAHSIIYALR.G	N	74.22	2314.1858	20	1.8	772.4039	3	31.94	7	1699	OB4035.raw	9.78E6	4	4	409	428	Deamidation (NQ)
K.TDSRPSIANLAGENSIIDNLPEEVVANSYGLPR.E	Y	74.00	3510.7590	33	2.4	1171.2631	3	35.69	7	1974	OB4035.raw	4.34E5	1	1	478	510	
R.SPDIYNPQAGSLK.T	N	72.75	1388.6936	13	0.2	695.3542	2	27.39	7	1421	OB4035.raw	1.04E5	1	1	372	384	
R.NALFVPHYNTNAHSIIYALR.G	N	72.27	2313.2019	20	0.8	772.0752	3	31.23	7	1675	OB4035.raw	5.74E6	2	2	409	428	
R.SPDIYNPQ(+.98)AGSLK.T	N	71.57	1389.6776	13	0.9	695.8467	2	28.07	7	1461	OB4035.raw	1.89E5	1	1	372	384	Deamidation (NQ)
N.GIEETIC(+57.02)TASVK.K	N	71.53	1306.6438	12	0.7	654.3297	2	27.80	7	1440	OB4035.raw	5.54E4	1	1	353	364	Carbamidomethylation
R.SPDIYN(+.98)PQAGSLKTANDLN(+.98)LLILR.W	N	70.53	2627.3806	24	5.1	876.8053	3	34.72	7	1912	OB4035.raw	0	0	0	372	395	Deamidation (NQ)
R.SPDIYN(+.98)PQ(+.98)AGSLKTANDLNLLILR.W	N	68.73	2627.3806	24	1.7	876.8023	3	34.36	7	1884	OB4035.raw	0	0	0	372	395	Deamidation (NQ)
R.VYDEELQEGHVLVVPQN(+.98)FAVAGK.S	N	68.59	2541.2751	23	0.7	848.0996	3	32.01	7	1729	OB4035.raw	2.05E5	1	1	443	465	
K.TAN(+.98)DLNLLILRWLGLSAEYGNLYR.N	N	67.21	2778.4705	24	7.0	927.1705	3	41.57	7	2344	OB4035.raw	0	0	0	385	408	
R.SPDIYN(+.98)PQAGSLK.T	N	66.40	1389.6776	13	0.9	695.8467	2	28.07	7	1450	OB4035.raw	1.89E5	1	1	372	384	
K.SQSDNFEYVAFKTD.S	N	65.50	1649.7209	14	0.4	825.8681	2	30.88	7	1634	OB4035.raw	6.42E4	1	1	466	479	
R.WLGLSAE(+14.02)YGNLYR.N	N	64.20	1554.7831	13	-0.4	778.3985	2	34.13	7	1867	OB4035.raw	0	0	0	396	408	Methylation(others)
R.SPDIYN(+.98)PQ(+.98)AGSLK.T	N	63.44	1390.6616	13	0.4	696.3384	2	28.62	7	1501	OB4035.raw	3.67E4	1	1	372	384	Deamidation (NQ)
R.SPDIYN(+.98)PQAGSLKTANDLNLLILR.W	N	62.73	2626.3965	24	0.3	876.4730	3	35.57	7	1972	OB4035.raw	0	0	0	372	395	
K.TDSRPSIANLAGEN(+.98)SIIDN(+.98)LPEEVVANSYGLPR.E	Y	61.05	3512.7271	33	6.9	1171.9243	3	35.97	7	2022	OB4035.raw	5.02E5	1	1	478	510	Deamidation (NQ)
K.TDSRPSIAN(+.98)LAGENSIIDN(+.98)LPEEVVANSYGLPR.E	Y	56.28	3512.7271	33	4.6	1171.9216	3	36.74	7	2046	OB4035.raw	0	0	0	478	510	Deamidation (NQ)
R.WLGLSAE(+53.92)YGNLYR.N	N	56.14	1594.6866	13	-0.3	532.5693	3	33.34	7	1819	OB4035.raw	5.46E5	1	1	396	408	Replacement of 2 protons by iron
R.NRSPDIYNPQ(+.98)AGSLKTAN(+.98)DLNLLILR.W	N	56.12	2897.5247	26	4.9	725.3920	4	34.35	7	1904	OB4035.raw	3.34E4	1	1	370	395	

Peptide	Uniq	-10lgP	Mass	Length	ppm	m/z	z	RT	Fraction	Scan	Source File	Area KOntrola 6. traka	#Feature	#Feature KOntrola 6. traka	Start	End	PTM
R.NRSPDIYNPQAGSLK.T	N	54.77	1658.8376	15	0.5	830.4265	2	25.62	7	1308	OB4035.raw	9.56E4	3	3	370	384	
K.TAN(+.98)DLN(+.98)LLILRWLGLSAEYGNLYR.N	N	54.63	2779.4543	24	2.9	927.4948	3	41.27	7	2326	OB4035.raw	0	0	0	385	408	Deamidation (NQ)
N.GIEETIC(+57.02)TASVKK.N	N	53.72	1434.7388	13	0.1	718.3767	2	25.95	7	1325	OB4035.raw	7.84E4	2	2	353	365	Carbamidomethylation
R.NRSPDIYN(+.98)PQAGSLKTAN(+.98)DLNLLILR.W	N	53.50	2897.5247	26	4.1	725.3914	4	33.94	7	1852	OB4035.raw	0	0	0	370	395	
R.NRSPDIYNPQ(+.98)AGSLKTANDLNLLILR.W	N	52.65	2896.5405	26	0.8	725.1430	4	32.66	7	1765	OB4035.raw	8.81E5	1	1	370	395	
R.SPDIYN(+.98)PQ(+.98)AGSLKTAN(+.98)DLN(+.98)LLILR.W	N	52.36	2629.3486	24	7.1	877.4630	3	34.83	7	1912	OB4035.raw	1.17E5	1	1	372	395	Deamidation (NQ)
R.VYDEELQEGHVLVVPQNFAVAGK.S	N	51.87	2540.2910	23	0.6	847.7714	3	31.67	7	1692	OB4035.raw	1.53E5	1	1	443	465	
R.NRSPDIYNPQ(+.98)AGSLK.T	N	51.37	1659.8217	15	-0.8	830.9175	2	26.31	7	1347	OB4035.raw	1.5E5	2	2	370	384	Deamidation (NQ)
K.TANDLNLLILR.W	N	50.78	1254.7296	11	0.0	628.3721	2	33.43	7	1928	OB4035.raw	5.74E6	1	1	385	395	
R.NRSPDIYN(+.98)PQ(+.98)AGSLKTAN(+.98)DLNLLILR.W	N	50.70	2898.5085	26	1.8	725.6357	4	33.73	7	1852	OB4035.raw	8.77E4	1	1	370	395	
K.TDSRPSIANLAGEN(+.98).S	N	49.56	1444.6793	14	0.0	723.3469	2	28.43	7	1486	OB4035.raw	2.32E4	1	1	478	491	Deamidation (NQ)
R.NALFVP(+15.99)HYN(+.98)TNAHSIIYALR.G	N	49.25	2330.1807	20	0.0	777.7341	3	33.43	7	1816	OB4035.raw	1.08E5	1	1	409	428	Hydroxylation Pro; Deamidation (NQ)
K.T(+43.01)(+14.02)ANDLNLLILR.W	N	48.90	1311.7510	11	0.4	656.8831	2	33.65	7	1825	OB4035.raw	1.5E5	1	1	385	395	Carbamylation; Methylation(others)
K.S(+43.01)(+14.02)QSDNFEYVAFK.T	N	46.98	1490.6677	12	0.1	746.3412	2	30.52	7	1618	OB4035.raw	3.2E4	1	1	466	477	Carbamylation; Methylation(others)
K.TDSRPSIAN(+.98)LAGEN(+.98).S	N	46.28	1445.6633	14	0.1	723.8390	2	29.02	7	1514	OB4035.raw	2.64E4	1	1	478	491	Deamidation (NQ)
K.SQ(+.98)SDN(+.98)FEYVAFK.T	N	46.28	1435.6143	12	2.7	718.8163	2	31.28	7	1664	OB4035.raw	7.74E3	1	1	466	477	Deamidation (NQ)
R.NRSPDIYNPQAGSLKTAN(+.98)DLNLLILR.W	N	46.23	2896.5405	26	0.8	725.1430	4	32.66	7	1753	OB4035.raw	8.81E5	1	1	370	395	
R.WLGLSAE(+14.02)YGN(+.98)LYR.N	N	45.38	1555.7671	13	-1.2	778.8899	2	34.58	7	1900	OB4035.raw	1.91E4	1	1	396	408	Methylation(others); Deamidation (NQ)
R.NALFVP(+15.99)HYNTNAHSIIYALR.G	N	44.60	2329.1968	20	0.1	777.4063	3	32.92	7	1784	OB4035.raw	1.3E5	2	2	409	428	Hydroxylation Pro
R.NRSPDIYNPQAGSLKTANDLNLLILR.W	N	43.70	2895.5566	26	0.7	724.8970	4	32.30	7	1734	OB4035.raw	0	0	0	370	395	
K.TAN(+.98)DLNLLILR.W	N	43.37	1255.7136	11	1.3	628.8649	2	34.04	7	1848	OB4035.raw	1.97E6	1	1	385	395	Deamidation (NQ)
A.NDLNLLILR.W	N	43.35	1082.6448	9	-0.8	542.3292	2	33.34	7	1810	OB4035.raw	2.03E4	1	1	387	395	
K.TAND(+28.03)LNLLILR.W	N	43.32	1282.7609	11	-0.2	642.3876	2	34.83	7	1924	OB4035.raw	9.42E4	1	1	385	395	
R.AHVQVVDSN(+.98)GN(+.98)RVYDEELQ(+.98)EGHVLVVPQNFAVAGK.S	N	43.28	3819.8704	35	5.2	955.9799	4	31.40	7	1672	OB4035.raw	0	0	0	431	465	
R.VYDEELQEGHVLVVPQ(+.98)N(+.98)FAVAGK.S	N	42.89	2542.2590	23	-0.1	848.4269	3	32.47	7	1755	OB4035.raw	1.18E5	1	1	443	465	Deamidation (NQ)
R.NRSPDIYN(+.98)PQAGSLK.T	N	42.75	1659.8217	15	-0.4	554.2809	3	25.82	7	1316	OB4035.raw	1.71E5	2	2	370	384	
R.NRSPDIYNP(+15.99)QAGSLKTANDLNLLILR.W	N	42.57	2911.5515	26	3.0	728.8973	4	34.73	7	1936	OB4035.raw	7.19E4	1	1	370	395	

Peptide	Uniq	-10lgP	Mass	Length	ppm	m/z	z	RT	Fraction	Scan	Source File	Area KOntrola 6. traka	#Feature	#Feature KOntrola 6. traka	Start	End	PTM
R.S(+41.03)P(+15.99)DIYNPQAGSLK.T	N	42.04	1445.7150	13	0.0	723.8647	2	27.55	7	1427	OB4035.raw	5.56E3	1	1	372	384	Amidination of lysines or N-terminal amines with methyl acetimidate; Hydroxylation Pro
K.TAN(+.98)DLNLLILRW(+15.99)LGLSAEYGNLYR.N	N	41.41	2794.4653	24	9.2	932.5043	3	41.41	7	2334	OB4035.raw	0	0	0	385	408	Oxidation (HW)
R.WLGLSAE(+21.98)YGNLYR.N	N	41.38	1562.7494	13	-8.5	521.9193	3	33.34	7	1807	OB4035.raw	1.16E5	2	2	396	408	Sodium adduct
R.NALFVPHYNTN(+.98).A	N	41.37	1289.6040	11	0.3	645.8094	2	29.66	7	1549	OB4035.raw	3.19E4	1	1	409	419	
K.TDSRPSIAN(+.98)LAGENSIIDNLPEEVVAN(+.98)SYGLPR.E	Y	41.33	3512.7271	33	2.1	1171.9187	3	35.07	7	1933	OB4035.raw	2.15E4	1	1	478	510	
R.WLGLSAEYGNLY(-18.01)R.N	N	41.07	1522.7568	13	1.7	762.3870	2	39.61	7	2227	OB4035.raw	3.31E4	1	1	396	408	Dehydration
K.TANDLNLLILRWLGLSAE(+28.03)YGNLYR.N	N	40.66	2805.5176	24	1.7	936.1814	3	40.41	7	2270	OB4035.raw	2.22E4	1	1	385	408	Ethylation
R.AHVQVVDSN(+.98)GNRVYDEELQ(+.98)EGHVLVVPQNFAVAGK.S	N	40.63	3818.8862	35	3.5	955.7322	4	31.54	7	1682	OB4035.raw	0	0	0	431	465	
R.NRSPDIYNP(+15.99)QAGSLKTAN(+.98)DLNLLILR.W	N	40.29	2912.5354	26	3.8	729.1439	4	33.34	7	1836	OB4035.raw	6.68E5	1	1	370	395	
R.WLGLSAEY(+15.01)GNLYR.N	N	39.61	1555.7783	13	-8.4	778.8899	2	34.58	7	1894	OB4035.raw	1.91E4	1	1	396	408	Tyrosine oxidation to 2-aminotyrosine
R.NRSPDIYNP(+15.99)QAGSLKTANDLN(+.98)LLILR.W	N	39.51	2912.5354	26	3.8	729.1439	4	33.34	7	1818	OB4035.raw	6.68E5	1	1	370	395	
R.N(+.98)ALFVPHYNTNAHSIIYALR.G	N	39.24	2314.1858	20	2.1	579.5549	4	31.94	7	1776	OB4035.raw	4.84E6	1	1	409	428	
K.TAN(+.98)DLN(+.98)LLILR.W	N	39.22	1256.6976	11	2.9	629.3579	2	34.54	7	1897	OB4035.raw	3.72E4	1	1	385	395	Deamidation (NQ)
K.TDSRPSIANLAGENSIIDNLPEEVVANSYGLPREQ(+.98)AR.Q	Y	39.00	3995.9824	37	2.7	1000.0056	4	34.53	7	1898	OB4035.raw	0	0	0	478	514	
R.S(+42.01)P(+15.99)DIYNPQAGSLKTANDLNLLILR.W	N	38.78	2683.4180	24	-0.5	895.4795	3	34.11	7	1864	OB4035.raw	9.81E4	1	1	372	395	Acetylation (N-term)
R.NRSPDIYNPQAGSLKTAN(+.98)DLN(+.98)LLILR.W	N	38.68	2897.5247	26	1.0	725.3892	4	33.45	7	1814	OB4035.raw	0	0	0	370	395	
K.TDSRPSIANLAGENSIIDN(+.98)LPEEVVANSYGLPREQ(+.98)AR.Q	Y	37.55	3996.9663	37	1.9	1000.2507	4	34.83	7	1921	OB4035.raw	1.96E6	1	1	478	514	
K.TAND(+53.92)LNLLILR.W	N	37.22	1308.6488	11	0.0	655.3317	2	33.43	7	1821	OB4035.raw	5.21E4	1	1	385	395	Replacement of 2 protons by iron
K.TDSRPSIAN(+.98)LAGEN.S	N	36.99	1444.6793	14	0.0	723.3469	2	28.43	7	1499	OB4035.raw	2.32E4	1	1	478	491	Deamidation (NQ)
K.TDSRPSIAN(+.98)LAGENSIIDNLPEEVVANSYGLPR.E	Y	36.57	3511.7429	33	5.3	878.9476	4	35.88	7	2001	OB4035.raw	1.34E5	1	1	478	510	
R.AHVQVVDSN(+.98)GNRVYDEELQEGHVLVVPQNFAVAGK.S	N	36.49	3817.9023	35	-0.8	955.4821	4	31.34	7	1648	OB4035.raw	2.77E5	1	1	431	465	
R.NALFVP(+15.99)H(+42.01)YNTNAHSIIYALR.G	N	36.41	2371.2073	20	8.0	791.4160	3	31.23	7	1662	OB4035.raw	9.72E4	1	1	409	428	Hydroxylation Pro
R.AHVQVVDSNGN(+.98)RVYDEELQEGHVLVVPQNFAVAGK.S	N	36.28	3817.9023	35	-0.8	955.4821	4	31.34	7	1663	OB4035.raw	2.77E5	1	1	431	465	
K.SQSDNFEYVAFKTDSRPSIANLAGENSIIDNLPEEVVANSYGLPR.E	Y	35.90	4926.3945	45	-0.2	1232.6057	4	35.65	7	1977	OB4035.raw	0	0	0	466	510	
R.WLGLSAEYGN(+.98)LY(+42.01)R(+14.02).N	N	35.60	1597.7776	13	7.0	799.9017	2	33.73	7	1791	OB4035.raw	6.17E5	1	1	396	408	Deamidation (NQ); Acetylation (TSCYH); Methylation(KR)

Peptide	Uniq	-10lgP	Mass	Length	ppm	m/z	z	RT	Fraction	Scan	Source File	Area KOntrola 6. traka	#Feature	#Feature KOntrola 6. traka	Start	End	PTM
K.TDSRPSIANLAGE.N	N	35.36	1329.6525	13	0.7	665.8340	2	28.33	7	1479	OB4035.raw	1.91E4	1	1	478	490	
G.SLKTANDLN(+.98)LLILR.W	N	35.26	1583.9246	14	-0.3	528.9820	3	32.66	7	1759	OB4035.raw	5.11E4	1	1	382	395	
K.TDSRPSIANLAGEN(+.98)SIIDNLPEEVVANSYGLPREQ(+.98)AR.Q	Y	35.21	3996.9663	37	1.9	1000.2507	4	34.83	7	2032	OB4035.raw	1.96E6	1	1	478	514	
R.WLGLSAEYGNLYRN(+.98)ALFVPHYN(+.98)TN(+.98)AHSIIYALR.G	N	35.11	3838.9106	33	1.1	768.7903	5	35.86	7	1990	OB4035.raw	0	0	0	396	428	
R.NALFVPHYN(+.98)TN(+.98).A	N	34.82	1290.5880	11	-0.2	646.3011	2	29.97	7	1580	OB4035.raw	5.15E3	1	1	409	419	Deamidation (NQ)
R.W(+43.01)LGLS(+14.02)AEYGNLYR.N	N	34.45	1597.7888	13	0.0	799.9017	2	33.73	7	1849	OB4035.raw	6.17E5	1	1	396	408	Carbamylation; Methylation(others)
K.TDSRPSIAN(+.98)LAG.E	N	34.14	1201.5939	12	1.0	601.8048	2	28.72	7	1485	OB4035.raw	4.76E4	1	1	478	489	Deamidation (NQ)
R.N(+58.01)ALFVPHYNTNAHSIIYALR.G	N	34.07	2371.2073	20	-0.5	791.4093	3	32.37	7	1735	OB4035.raw	1.9E5	1	1	409	428	Carboxymethyl (KW, X@N-term)
R.WLGLSAEYGN(+.98)LYR.N	N	34.02	1541.7513	13	6.2	771.8878	2	33.66	7	1830	OB4035.raw	0	0	0	396	408	Deamidation (NQ)
K.TDSRPSIANLAGEN(+.98)SIIDNLPEEVVAN(+.98)SYGLPREQ(+.98)AR.Q	Y	32.63	3997.9504	37	2.7	1000.4976	4	35.26	7	1951	OB4035.raw	0	0	0	478	514	
R.VYDEELQ(+.98)EGHVLVVP(+15.99)QNFAVAGK.S	N	32.20	2557.2700	23	-3.0	853.4280	3	32.95	7	1778	OB4035.raw	0	0	0	443	465	Deamidation (NQ); Hydroxylation Pro
K.SQSDNFEYVAFKTDSRPSIANLAGENSIIDN(+.98)LPEEVVANSYGLPR.E	Y	32.12	4927.3789	45	5.2	1232.8584	4	35.88	7	1977	OB4035.raw	2.51E5	1	1	466	510	
R.WLGLSAE(+53.92)YGN(+.98)LYR.N	N	32.10	1595.6707	13	2.2	532.8987	3	33.75	7	1837	OB4035.raw	0	0	0	396	408	Replacement of 2 protons by iron; Deamidation (NQ)
R.NALFVPHYN(+.98)TNA.H	N	32.09	1360.6411	12	1.0	681.3285	2	29.71	7	1559	OB4035.raw	5.2E3	1	1	409	420	
R.N(+42.01)ALFVP(+15.99)HYNTNAHSIIYALR.G	N	31.91	2371.2073	20	-0.5	791.4093	3	32.37	7	1748	OB4035.raw	1.9E5	1	1	409	428	Acetylation (N-term); Hydroxylation Pro
R.SPDIYN(+.98)PQ(+.98)AGSLKTAN(+.98)DLNLLILR.W	N	31.82	2628.3645	24	7.6	658.1034	4	34.43	7	1880	OB4035.raw	1.37E4	1	1	372	395	
K.TDSRPSIANLA.G	N	31.18	1143.5884	11	0.3	572.8016	2	27.88	7	1465	OB4035.raw	1.36E4	1	1	478	488	
R.AHVQVVDSNGNRVYDEELQ(+.98)EGHVLVVPQNFAVAGK.S	N	31.15	3817.9023	35	1.2	764.5887	5	31.42	7	1628	OB4035.raw	1.05E6	1	1	431	465	
K.TDSRPSIANLAGEN(+.98)SIIDN(+.98)LPEEVVANSYGLPREQ(+.98)AR.Q	Y	30.82	3997.9504	37	8.0	1000.5029	4	35.93	7	1995	OB4035.raw	0	0	0	478	514	
R.AHVQVVDSNGNRVYDEELQEGHVLVVPQNFAVAGK.S	N	30.77	3816.9182	35	0.4	764.3912	5	30.69	7	1624	OB4035.raw	0	0	0	431	465	
R.VYDEELQ(+.98)EGHVLVVP(+15.99)QN(+.98)FAVAGK.S	N	30.74	2558.2539	23	2.3	853.7605	3	32.92	7	1778	OB4035.raw	7.47E3	1	1	443	465	Deamidation (NQ); Hydroxylation Pro
R.SPD(+21.98)IYNPQAGS(-18.01)LKTANDLNLLILR.W	N	30.41	2629.3840	24	1.5	877.4700	3	34.43	7	1884	OB4035.raw	1.89E5	1	1	372	395	
K.TANDLN(+.98)LLILRW(+15.99)LGLSAEYGNLYR.N	N	30.14	2794.4653	24	4.6	932.5000	3	42.12	7	2378	OB4035.raw	0	0	0	385	408	Oxidation (HW)
R.NRSPDIYN(+.98)P(+15.99)Q(+.98)AGSLKTANDLNLLILR.W	N	29.40	2913.5195	26	-0.5	729.3868	4	34.64	7	1905	OB4035.raw	0	0	0	370	395	
K.TDSRPSIANLAGEN(+.98)SIIDN(+.98)LPEEVVAN.S	N	29.30	2839.3723	27	-0.2	947.4645	3	35.50	7	1957	OB4035.raw	3.01E4	1	1	478	504	

Peptide	Uniq	-10lgP	Mass	Length	ppm	m/z	z	RT	Fraction	Scan	Source File	Area KOntrola 6. traka	#Feature	#Feature KOntrola 6. traka	Start	End	PTM
R.NALFVPHYNTNAHSIIYALRG.R	N	29.09	2370.2231	21	0.6	791.0821	3	31.77	7	1662	OB4035.raw	1.7E5	1	1	409	429	
R.AHVQVVDSNGNRVYDEELQ(+.98)EGHVLVVPQ(+.98)NFAVAGK.S	N	28.70	3818.8862	35	4.9	764.7883	5	31.34	7	1668	OB4035.raw	0	0	0	431	465	
T.A(+104.03)NDLNLLILRWLGLSAEYGNLYR.N	N	28.35	2780.4648	23	2.6	927.8313	3	40.90	7	2321	OB4035.raw	7.62E3	1	1	386	408	Benzoyl
K.TDSRPSIANLAGENSIIDNLPEEVVAN(+.98)SYGLPREQ(+.98)AR.Q	Y	28.17	3996.9663	37	1.9	1000.2507	4	34.83	7	2056	OB4035.raw	1.96E6	1	1	478	514	
R.NALFVPHY.N	N	27.94	959.4865	8	-0.7	480.7502	2	30.43	7	1610	OB4035.raw	2.39E4	1	1	409	416	
R.WLGLSAEYGNLYRN(+.98)ALFVPHYN(+.98)TNAHSIIYALR.G	N	27.92	3837.9268	33	-0.8	768.5920	5	35.14	7	1960	OB4035.raw	2.05E5	1	1	396	428	
R.W(+42.01)LGLSAE(+14.02)YGN(+.98)LYR.N	N	27.76	1597.7776	13	7.4	533.6038	3	33.73	7	1837	OB4035.raw	7.42E3	1	1	396	408	Acetylation (N-term); Deamidation (NQ)
R.NALFVPHYNTNA.H	N	27.57	1359.6571	12	0.0	680.8358	2	29.29	7	1532	OB4035.raw	7.88E3	1	1	409	420	
K.TDSRPSIAN(+.98)LA.G	N	27.12	1144.5724	11	-1.0	573.2929	2	28.82	7	1508	OB4035.raw	3.26E4	1	1	478	488	Deamidation (NQ)
R.NRSPDIYNPQAGSLKT(-18.01)AND(+21.98)LNLLILR.W	N	26.92	2899.5281	26	2.0	725.8907	4	33.01	7	1797	OB4035.raw	4.04E5	2	2	370	395	Sodium adduct
R.NALFVPHYNTNAHSIIYALRGR.A	N	26.87	2526.3245	22	0.6	632.5887	4	30.52	7	1611	OB4035.raw	5.28E4	1	1	409	430	
R.AHVQVVDSN(+.98)GN(+.98)RVYDEELQEGHVLVVPQNFAVAGK.S	N	26.20	3818.8862	35	6.3	955.7348	4	30.87	7	1636	OB4035.raw	0	0	0	431	465	
R.WLGLSAEYGN(+.98)LYRNALFVPHYN(+.98)TN(+.98)AHSIIYALR.G	N	25.93	3838.9106	33	5.5	768.7936	5	35.67	7	1978	OB4035.raw	0	0	0	396	428	
R.VYDEELQEGHVLVVP(+15.99)QNFAVAGK.S	N	25.47	2556.2859	23	6.6	853.1082	3	33.01	7	1778	OB4035.raw	7.76E3	1	1	443	465	Hydroxylation Pro
R.WLGLS(+58.03)AEYGNLYR.N	N	25.07	1598.7966	13	-3.9	800.4025	2	33.19	7	1791	OB4035.raw	4.21E3	1	1	396	408	2,3-dihydro-2,2-dimethyl-7-benzofuranol N-methyl carbamate
R.QLKN(+.98)N(+.98)NPFKFFVPPSQ(+.98)Q(+.98)SLGAV.A	Y	24.54	2463.2322	22	6.4	822.0899	3	32.75	7	1766	OB4035.raw	8E4	1	1	515	536	Deamidation (NQ)
R.WLGLS(-18.01)AEYGNLYR.N	N	24.29	1522.7568	13	1.7	762.3870	2	39.61	7	2224	OB4035.raw	3.31E4	1	1	396	408	
R.NRSPDIYN(+.98)PQ(+.98)AGSLK.T	N	23.21	1660.8057	15	-0.7	554.6088	3	27.13	7	1410	OB4035.raw	1.72E4	1	1	370	384	Deamidation (NQ)
K.TDSRPSIANLAG.E	N	23.17	1200.6099	12	0.1	601.3123	2	27.97	7	1447	OB4035.raw	2.3E4	1	1	478	489	
N.GIEETIC(+71.04)TASVK.K	N	22.64	1320.6595	12	-0.7	661.3366	2	27.68	7	1436	OB4035.raw	0	0	0	353	364	Propionamide
R.W(+42.01)(+15.99)LGLSAEYGNLYR.N	N	22.56	1598.7728	13	-2.0	800.3921	2	34.11	7	1862	OB4035.raw	4.12E4	1	1	396	408	Acetylation (N-term); Oxidation (HW)
N.GIEETIC(+58.01)TASVK.K	N	22.53	1307.6278	12	1.7	654.8223	2	28.82	7	1505	OB4035.raw	5.31E3	1	1	353	364	Carboxymethyl
R.AHVQ(+.98)VVDSNGNRVYDEELQEGHVLVVPQNFAVAGK.S	N	22.40	3817.9023	35	1.2	764.5887	5	31.42	7	1642	OB4035.raw	1.05E6	1	1	431	465	
R.SPDIYNPQAGSLKT(-18.01)AND(+21.98)LNLLILR.W	N	22.35	2629.3840	24	1.5	877.4700	3	34.43	7	1912	OB4035.raw	1.89E5	1	1	372	395	
R.AHVQVVDSNGNRVYDEELQEGHVLVVPQ(+.98)N(+.98)FAVAGK.S	N	22.34	3818.8862	35	1.5	764.7856	5	31.51	7	1680	OB4035.raw	0	0	0	431	465	
R.N(+.98)RSPDIYNPQAGSLK.T	N	22.33	1659.8217	15	-0.5	554.2809	3	26.23	7	1389	OB4035.raw	1.47E5	1	1	370	384	
R.AHVQ(+.98)VVDSNGN(+.98)RVYDEELQEGHVLVVPQN(+.98)FAVAGK.S	N	22.20	3819.8704	35	7.5	764.9871	5	32.04	7	1717	OB4035.raw	0	0	0	431	465	

Peptide	Uniq	-10lgP	Mass	Length	ppm	m/z	z	RT	Fraction	Scan	Source File	Area KOntrola 6. traka	#Feature	#Feature KOntrola 6. traka	Start	End	PTM
total 133 peptides																	

tr|B5TYU1|B5TYU1_ARAHY
back to list

| Protein Coverage | Supporting Peptides |

Protein Coverage:

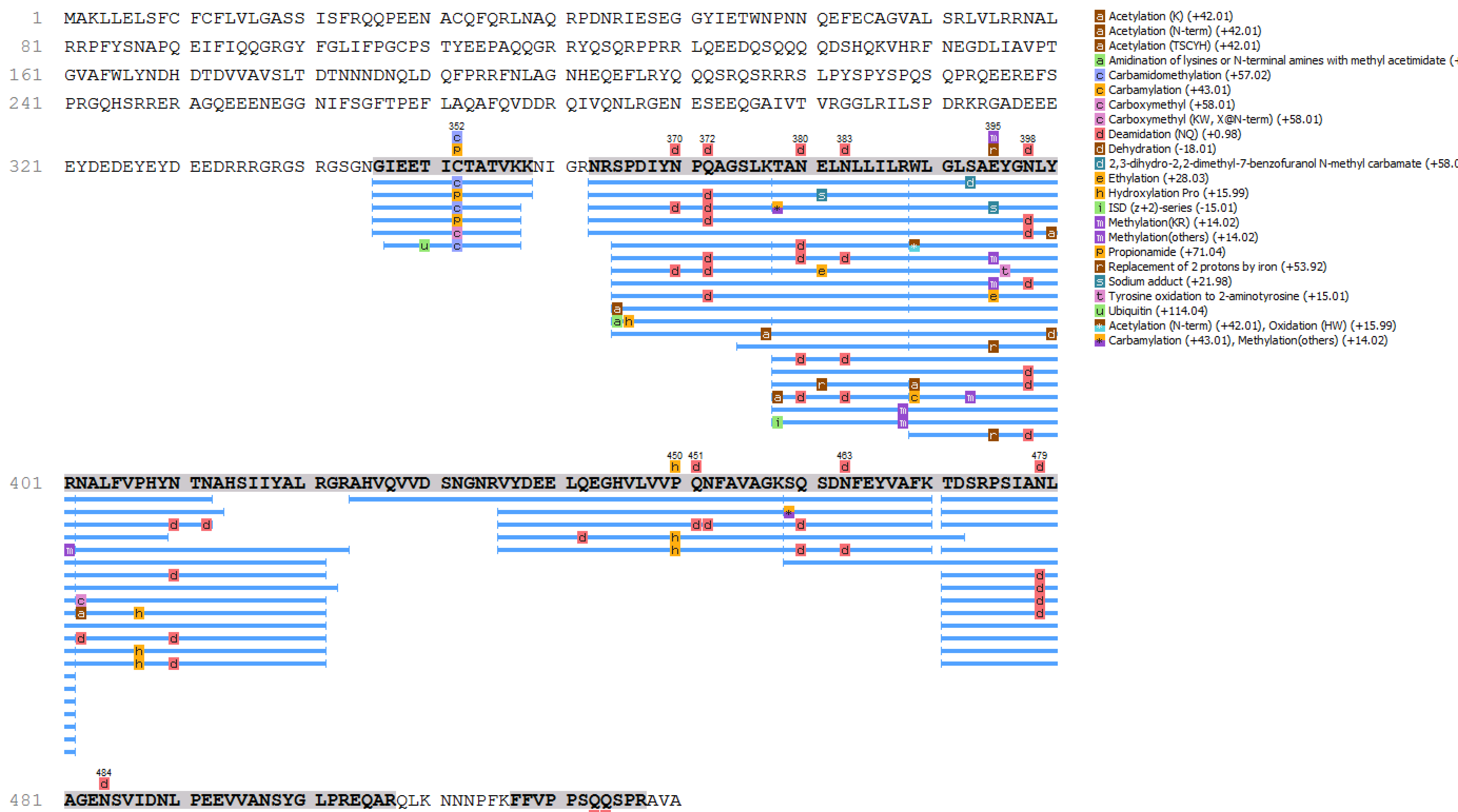
1 MAKLLELSFC FCFLVLGASS ISFRQQPEEN ACQFQRLNAQ RPDNRIESEG GYIETWNPNN QEFECAGVAL SRLVLRRNAL
81 RRPFYSNAPQ EIFIQQGRGY FGLIFPGCPS TYEEPAQQGR RYQSQRPPRR LQEEDQSQQQ QDSHQKVHRF NEGDLIAVPT
161 GVAFWLYNDH DTDVVAVSLT DTNNNDNQLD QFPRRFNLAG NHEQEFLRYQ QQSRQSRRRS LPYSPYSPQS QPRQEEREFS
241 PRGQHSRRER AGQEEENEGG NIFSGFTPEF LAQAFQVDDR QIVQNLRGEN ESEEQGAIVT VRGGLRILSP DRKRGADEEE
321 EYDEDEYEYD EEDRRRGRGS RGSGNGIEET ICTATVKKNI GRNRSPDIYN PQAGSLKTAN ELNLLILRWL GLSAEYGNLY
352
370
372
380
383
395
398
401 RNALFVPHYN TNAHSIIYAL RGRAHVQVVD SNGNRVYDEE LQEGHVLVVP QNFAVAGKSQ SDNFEYVAFK TDSRPSIANL
450
451
463
479
481 AGENSVIDNL PEEVVANSYG LPREQARQLK NNNPFKFFVP PSQQSPRAVA
484
a Acetylation (K) (+42.01)
a Acetylation (N-term) (+42.01)
a Acetylation (TSCYH) (+42.01)
a Amidination of lysines or N-terminal amines with methyl acetimidate (
c Carbamidomethylation (+57.02)
c Carbamylation (+43.01)
c Carboxymethyl (+58.01)
c Carboxymethyl (KW, X@N-term) (+58.01)
d Deamidation (NQ) (+0.98)
d Dehydration (-18.01)
d 2,3-dihydro-2,2-dimethyl-7-benzofuranol N-methyl carbamate (+58.0
e Ethylation (+28.03)
h Hydroxylation Pro (+15.99)
i ISD (z+2)-series (-15.01)
m Methylation(KR) (+14.02)
m Methylation(others) (+14.02)
p Propionamide (+71.04)
r Replacement of 2 protons by iron (+53.92)
s Sodium adduct (+21.98)
t Tyrosine oxidation to 2-aminotyrosine (+15.01)
u Ubiquitin (+114.04)
Acetylation (N-term) (+42.01), Oxidation (HW) (+15.99)
Carbamylation (+43.01), Methylation(others) (+14.02)

Supporting Peptides:

Peptide	Uniq	-10lgP	Mass	Length	ppm	m/z	z	RT	Fraction	Scan	Source File	Area KOntrola 6. traka	#Feature	#Feature KOntrola 6. traka	Start	End	PTM
R.WLGLSAEYGNLYR.N	N	95.34	1540.7673	13	1.0	771.3917	2	33.34	7	1963	OB4035.raw	1.45E7	5	5	389	401	
K.SQSDNFEYVAFK.T	N	86.92	1433.6462	12	0.9	717.8311	2	30.43	7	1599	OB4035.raw	8.2E5	1	1	459	470	
R.N(+.98)ALFVPHYN(+.98)TNAHSIIYALR.G	N	83.79	2315.1699	20	1.1	772.7314	3	32.66	7	1752	OB4035.raw	2.44E5	1	1	402	421	Deamidation (NQ)
R.SPDIYNPQ(+.98)AGSLKTANELNLLILR.W	N	82.41	2640.4121	24	1.1	881.1456	3	34.26	7	1873	OB4035.raw	1.23E6	1	1	365	388	Deamidation (NQ)
R.SPDIYNPQAGSLKTANELNLLILR.W	N	81.51	2639.4282	24	0.0	880.8167	3	33.88	7	1845	OB4035.raw	3.87E5	1	1	365	388	
K.TDSRPSIANLAGEN(+.98)SVIDNLPEEVVANSYGLPR.E	Y	80.09	3497.7273	33	1.6	1166.9182	3	35.54	7	1970	OB4035.raw	6.3E4	1	1	471	503	Deamidation (NQ)
K.SQ(+.98)SDNFEYVAFK.T	N	79.67	1434.6302	12	0.3	718.3226	2	30.79	7	1627	OB4035.raw	3.08E5	1	1	459	470	Deamidation (NQ)
K.TDSRPSIANLAGENSVIDNLPEEVVANSYGLPR.E	Y	75.23	3496.7434	33	0.5	1166.5890	3	35.14	7	1944	OB4035.raw	1.21E5	1	1	471	503	
K.TDSRPSIANLAGENSVIDN(+.98)LPEEVVANSYGLPR.E	Y	74.64	3497.7273	33	2.6	1166.9194	3	35.34	7	1956	OB4035.raw	0	0	0	471	503	
R.WLGLSAE(+28.03)YGNLYR.N	N	74.46	1568.7987	13	0.9	785.4073	2	34.89	7	1929	OB4035.raw	1.15E5	1	1	389	401	Ethylation
R.NALFVPHYN(+.98)TNAHSIIYALR.G	N	74.22	2314.1858	20	1.8	772.4039	3	31.94	7	1699	OB4035.raw	9.78E6	4	4	402	421	Deamidation (NQ)
K.TANELNLLILRWLGLSAEYGNLYR.N	N	74.10	2791.5020	24	0.9	931.5087	3	41.01	7	2310	OB4035.raw	2.03E5	2	2	378	401	
R.SPDIYNPQAGSLK.T	N	72.75	1388.6936	13	0.2	695.3542	2	27.39	7	1421	OB4035.raw	1.04E5	1	1	365	377	
N.GIEETIC(+57.02)TATVK.K	N	72.36	1320.6595	12	0.8	661.3376	2	28.07	7	1473	OB4035.raw	6.45E4	1	1	346	357	Carbamidomethylation
R.NALFVPHYNTNAHSIIYALR.G	N	72.27	2313.2019	20	0.8	772.0752	3	31.23	7	1675	OB4035.raw	5.74E6	2	2	402	421	
R.SPDIYN(+.98)PQAGSLKTANELNLLILR.W	N	72.25	2640.4121	24	1.9	881.1463	3	35.63	7	1976	OB4035.raw	0	0	0	365	388	
R.SPDIYNPQ(+.98)AGSLK.T	N	71.57	1389.6776	13	0.9	695.8467	2	28.07	7	1461	OB4035.raw	1.89E5	1	1	365	377	Deamidation (NQ)
R.SPDIYN(+.98)PQAGSLKTAN(+.98)ELNLLILR.W	N	68.83	2641.3962	24	1.5	881.4740	3	34.68	7	1908	OB4035.raw	0	0	0	365	388	
R.VYDEELQEGHVLVVPQN(+.98)FAVAGK.S	N	68.59	2541.2751	23	0.7	848.0996	3	32.01	7	1729	OB4035.raw	2.05E5	1	1	436	458	
R.SPDIYN(+.98)PQAGSLK.T	N	66.40	1389.6776	13	0.9	695.8467	2	28.07	7	1450	OB4035.raw	1.89E5	1	1	365	377	
K.SQSDNFEYVAFKTD.S	N	65.50	1649.7209	14	0.4	825.8681	2	30.88	7	1634	OB4035.raw	6.42E4	1	1	459	472	
K.TANELN(+.98)LLILRWLGLSAEYGNLYR.N	N	65.11	2792.4861	24	0.8	931.8367	3	40.81	7	2298	OB4035.raw	5.19E3	1	1	378	401	
R.WLGLSAE(+14.02)YGNLYR.N	N	64.20	1554.7831	13	-0.4	778.3985	2	34.13	7	1867	OB4035.raw	0	0	0	389	401	Methylation(others)
R.SPDIYN(+.98)PQ(+.98)AGSLK.T	N	63.44	1390.6616	13	0.4	696.3384	2	28.62	7	1501	OB4035.raw	3.67E4	1	1	365	377	Deamidation (NQ)
K.TDSRPSIANLAGEN(+.98)SVIDN(+.98)LPEEVVANSYGLPR.E	Y	63.16	3498.7114	33	6.5	1167.2520	3	35.78	7	1986	OB4035.raw	1.66E5	1	1	471	503	
R.NRSPDIYN(+.98)PQAGSLKTANELNLLILR.W	N	60.37	2910.5562	26	0.7	728.6469	4	32.92	7	1796	OB4035.raw	8.77E5	1	1	363	388	
K.TAN(+.98)ELNLLILRWLGLSAEYGNLYR.N	N	60.11	2792.4861	24	6.8	931.8423	3	43.14	7	2443	OB4035.raw	4.31E3	1	1	378	401	
R.NRSPDIYNPQ(+.98)AGSLKTANELNLLILR.W	N	60.10	2910.5562	26	0.7	728.6469	4	32.92	7	1782	OB4035.raw	8.77E5	1	1	363	388	Deamidation (NQ)
K.TANELNLLILR.W	N	59.29	1268.7452	11	-0.1	635.3798	2	33.73	7	1910	OB4035.raw	4.83E6	2	2	378	388	

Peptide	Uniq	-10lgP	Mass	Length	ppm	m/z	z	RT	Fraction	Scan	Source File	Area KOntrola 6. traka	#Feature	#Feature KOntrola 6. traka	Start	End	PTM
N.GIEETIC(+57.02)TATVKK.N	N	57.16	1448.7545	13	0.2	725.3847	2	26.53	7	1361	OB4035.raw	7.77E3	1	1	346	358	Carbamidomethylation
R.WLGLSAE(+53.92)YGNLYR.N	N	56.14	1594.6866	13	-0.3	532.5693	3	33.34	7	1819	OB4035.raw	5.46E5	1	1	389	401	Replacement of 2 protons by iron
R.SPDIYN(+.98)PQ(+.98)AGSLKTANELNLLILR.W	N	55.71	2641.3962	24	2.5	881.4749	3	38.06	7	2127	OB4035.raw	0	0	0	365	388	Deamidation (NQ)
R.NRSPDIYNPQAGSLK.T	N	54.77	1658.8376	15	0.5	830.4265	2	25.62	7	1308	OB4035.raw	9.56E4	3	3	363	377	
R.SPDIYN(+.98)PQ(+.98)AGSLKTAN(+.98)ELNLLILR.W	N	52.00	2642.3801	24	9.7	881.8092	3	38.10	7	2127	OB4035.raw	7.46E3	1	1	365	388	
R.VYDEELQEGHVLVVPQNFAVAGK.S	N	51.87	2540.2910	23	0.6	847.7714	3	31.67	7	1692	OB4035.raw	1.53E5	1	1	436	458	
R.NRSPDIYNPQ(+.98)AGSLK.T	N	51.37	1659.8217	15	-0.8	830.9175	2	26.31	7	1347	OB4035.raw	1.5E5	2	2	363	377	Deamidation (NQ)
K.TANE(+28.03)LNLLILR.W	N	51.01	1296.7765	11	0.9	649.3961	2	35.69	7	1983	OB4035.raw	3.28E4	1	1	378	388	Ethylation
N.GIEETIC(+71.04)TATVK.K	N	50.49	1334.6752	12	0.2	668.3450	2	28.43	7	1483	OB4035.raw	3.08E4	1	1	346	357	Propionamide
K.TAN(+.98)ELNLLILR.W	N	50.21	1269.7292	11	0.7	635.8723	2	34.35	7	1872	OB4035.raw	1.24E6	1	1	378	388	Deamidation (NQ)
K.TANELNLLILRWLGLSAEYGN(+.98)LYR.N	N	49.96	2792.4861	24	4.9	931.8405	3	42.51	7	2406	OB4035.raw	5.19E3	1	1	378	401	Deamidation (NQ)
R.NRSPDIYNPQ(+.98)AGSLKTAN(+.98)ELNLLILR.W	N	49.63	2911.5403	26	1.2	728.8932	4	33.51	7	1818	OB4035.raw	7.19E4	1	1	363	388	
K.TDSRPSIANLAGEN(+.98).S	N	49.56	1444.6793	14	0.0	723.3469	2	28.43	7	1486	OB4035.raw	2.32E4	1	1	471	484	Deamidation (NQ)
K.T(+43.01)(+14.02)ANELNLLILR.W	N	49.43	1325.7667	11	0.2	663.8907	2	33.96	7	1855	OB4035.raw	1.74E5	1	1	378	388	Carbamylation; Methylation(others)
R.NALFVP(+15.99)HYN(+.98)TNAHSIIYALR.G	N	49.25	2330.1807	20	0.0	777.7341	3	33.43	7	1816	OB4035.raw	1.08E5	1	1	402	421	Hydroxylation Pro; Deamidation (NQ)
R.NRSPDIYNPQAGSLKTAN(+.98)ELNLLILR.W	N	49.05	2910.5562	26	0.7	728.6469	4	32.92	7	1770	OB4035.raw	8.77E5	1	1	363	388	
K.TANELN(+.98)LLILR.W	N	48.70	1269.7292	11	0.7	635.8723	2	34.35	7	1890	OB4035.raw	1.24E6	1	1	378	388	
R.S(+42.01)P(+15.99)DIYNPQAGSLKTANELNLLILR.W	N	48.25	2697.4336	24	-0.3	900.1516	3	34.35	7	1882	OB4035.raw	1.13E5	1	1	365	388	Acetylation (N-term)
K.TAN(+.98)ELN(+.98)LLILRWLGLSAEYGNLYR.N	N	47.96	2793.4700	24	4.5	932.1682	3	42.32	7	2391	OB4035.raw	0	0	0	378	401	Deamidation (NQ)
R.NRSPDIYNPQAGSLKTANELNLLILR.W	N	47.32	2909.5723	26	1.2	728.4012	4	32.69	7	1760	OB4035.raw	0	0	0	363	388	
K.TANE(+28.03)LNLLILRWLGLSAEYGNLYR.N	N	47.29	2819.5334	24	-0.9	940.8510	3	40.80	7	2296	OB4035.raw	6.75E3	1	1	378	401	
K.S(+43.01)(+14.02)QSDNFEYVAFK.T	N	46.98	1490.6677	12	0.1	746.3412	2	30.52	7	1618	OB4035.raw	3.2E4	1	1	459	470	Carbamylation; Methylation(others)
K.TANELNLLILR(+14.02)WLGLSAEYGNLYR.N	N	46.67	2805.5176	24	1.7	936.1814	3	40.41	7	2265	OB4035.raw	2.22E4	1	1	378	401	Methylation(KR)
K.TDSRPSIAN(+.98)LAGEN(+.98).S	N	46.28	1445.6633	14	0.1	723.8390	2	29.02	7	1514	OB4035.raw	2.64E4	1	1	471	484	Deamidation (NQ)
K.SQ(+.98)SDN(+.98)FEYVAFK.T	N	46.28	1435.6143	12	2.7	718.8163	2	31.28	7	1664	OB4035.raw	7.74E3	1	1	459	470	Deamidation (NQ)
R.NRSPDIYN(+.98)PQ(+.98)AGSLKTANELNLLILR.W	N	45.68	2911.5403	26	0.5	728.8927	4	33.74	7	1836	OB4035.raw	0	0	0	363	388	
R.WLGLSAE(+14.02)YGN(+.98)LYR.N	N	45.38	1555.7671	13	-1.2	778.8899	2	34.58	7	1900	OB4035.raw	1.91E4	1	1	389	401	Methylation(others); Deamidation (NQ)

Peptide	Uniq	-10lgP	Mass	Length	ppm	m/z	z	RT	Fraction	Scan	Source File	Area KOntrola 6. traka	#Feature	#Feature KOntrola 6. traka	Start	End	PTM
K.TAN(+.98)ELN(+.98)LLILR.W	N	45.08	1270.7133	11	2.7	636.3656	2	34.89	7	1922	OB4035.raw	9.56E4	1	1	378	388	Deamidation (NQ)
R.NALFVP(+15.99)HYNTNAHSIIYALR.G	N	44.60	2329.1968	20	0.1	777.4063	3	32.92	7	1784	OB4035.raw	1.3E5	2	2	402	421	Hydroxylation Pro
R.AHVQVVDSN(+.98)GN(+.98)RVYDEELQ(+.98)EGHVLVVPQNFAVAGK.S	N	43.28	3819.8704	35	5.2	955.9799	4	31.40	7	1672	OB4035.raw	0	0	0	424	458	
R.VYDEELQEGHVLVVPQ(+.98)N(+.98)FAVAGK.S	N	42.89	2542.2590	23	-0.1	848.4269	3	32.47	7	1755	OB4035.raw	1.18E5	1	1	436	458	Deamidation (NQ)
R.NRSPDIYN(+.98)PQAGSLK.T	N	42.75	1659.8217	15	-0.4	554.2809	3	25.82	7	1316	OB4035.raw	1.71E5	2	2	363	377	
R.S(+41.03)P(+15.99)DIYNPQAGSLK.T	N	42.04	1445.7150	13	0.0	723.8647	2	27.55	7	1427	OB4035.raw	5.56E3	1	1	365	377	Amidination of lysines or N-terminal amines with methyl acetimidate; Hydroxylation Pro
R.WLGLSAE(+21.98)YGNLYR.N	N	41.38	1562.7494	13	-8.5	521.9193	3	33.34	7	1807	OB4035.raw	1.16E5	2	2	389	401	Sodium adduct
R.NALFVPHYNTN(+.98).A	N	41.37	1289.6040	11	0.3	645.8094	2	29.66	7	1549	OB4035.raw	3.19E4	1	1	402	412	
R.WLGLSAEYGNLY(-18.01)R.N	N	41.07	1522.7568	13	1.7	762.3870	2	39.61	7	2227	OB4035.raw	3.31E4	1	1	389	401	Dehydration
R.AHVQVVDSN(+.98)GNRVYDEELQ(+.98)EGHVLVVPQNFAVAGK.S	N	40.63	3818.8862	35	3.5	955.7322	4	31.54	7	1682	OB4035.raw	0	0	0	424	458	
R.NRSPDIYN(+.98)PQ(+.98)AGSLKTAN(+.98)ELNLLILR.W	N	39.62	2912.5242	26	7.7	729.1439	4	33.34	7	1796	OB4035.raw	6.68E5	1	1	363	388	
R.WLGLSAEY(+15.01)GNLYR.N	N	39.61	1555.7783	13	-8.4	778.8899	2	34.58	7	1894	OB4035.raw	1.91E4	1	1	389	401	Tyrosine oxidation to 2-aminotyrosine
K.TDSRPSIANLAGEN(+.98)SVIDNLPEEVVAN(+.98)SYGLPREQ(+.98)AR.Q	Y	39.59	3983.9348	37	2.4	996.9934	4	34.74	7	1914	OB4035.raw	0	0	0	471	507	Deamidation (NQ)
R.N(+.98)ALFVPHYNTNAHSIIYALR.G	N	39.24	2314.1858	20	2.1	579.5549	4	31.94	7	1776	OB4035.raw	4.84E6	1	1	402	421	
K.TDSRPSIANLAGENSVIDNLPEEVVAN(+.98)SYGLPREQ(+.98)AR.Q	Y	37.03	3982.9507	37	3.4	996.7484	4	34.35	7	1892	OB4035.raw	5.31E5	1	1	471	507	
K.TDSRPSIAN(+.98)LAGEN.S	N	36.99	1444.6793	14	0.0	723.3469	2	28.43	7	1499	OB4035.raw	2.32E4	1	1	471	484	Deamidation (NQ)
K.TANE(+53.92)LNLLILR.W	N	36.59	1322.6646	11	-0.5	662.3392	2	33.73	7	1839	OB4035.raw	9.25E4	2	2	378	388	Replacement of 2 protons by iron
R.AHVQVVDSN(+.98)GNRVYDEELQEGHVLVVPQNFAVAGK.S	N	36.49	3817.9023	35	-0.8	955.4821	4	31.34	7	1648	OB4035.raw	2.77E5	1	1	424	458	
R.NALFVP(+15.99)H(+42.01)YNTNAHSIIYALR.G	N	36.41	2371.2073	20	8.0	791.4160	3	31.23	7	1662	OB4035.raw	9.72E4	1	1	402	421	Hydroxylation Pro
R.AHVQVVDSNGN(+.98)RVYDEELQEGHVLVVPQNFAVAGK.S	N	36.28	3817.9023	35	-0.8	955.4821	4	31.34	7	1663	OB4035.raw	2.77E5	1	1	424	458	
R.SPDIYNP(+15.99)Q(+.98)AGSLK(+42.01)TANELNLLILR.W	N	36.23	2698.4177	24	-0.2	900.4797	3	34.83	7	1918	OB4035.raw	4.79E4	1	1	365	388	Acetylation (K)
R.N(+.98)RSPDIYNPQ(+.98)AGSLKTAN(+.98)ELNLLILR.W	N	36.03	2912.5242	26	1.2	729.1392	4	34.04	7	1876	OB4035.raw	1.27E5	1	1	363	388	
N.GIEETIC(+58.01)TATVK.K	N	35.77	1321.6436	12	-0.5	661.8287	2	28.83	7	1507	OB4035.raw	9.22E3	1	1	346	357	Carboxymethyl
R.WLGLSAEYGN(+.98)LY(+42.01)R(+14.02).N	N	35.60	1597.7776	13	7.0	799.9017	2	33.73	7	1791	OB4035.raw	6.17E5	1	1	389	401	Deamidation (NQ); Acetylation (TSCYH); Methylation(KR)
K.TDSRPSIANLAGE.N	N	35.36	1329.6525	13	0.7	665.8340	2	28.33	7	1479	OB4035.raw	1.91E4	1	1	471	483	

Peptide	Uniq	-10lgP	Mass	Length	ppm	m/z	z	RT	Fraction	Scan	Source File	Area KOntrola 6. traka	#Feature	#Feature KOntrola 6. traka	Start	End	PTM
R.WLGLSAEYGNLYRN(+.98)ALFVPHYN(+.98)TN(+.98)AHSIIYALR.G	N	35.11	3838.9106	33	1.1	768.7903	5	35.86	7	1990	OB4035.raw	0	0	0	389	421	
R.NALFVPHYN(+.98)TN(+.98).A	N	34.82	1290.5880	11	-0.2	646.3011	2	29.97	7	1580	OB4035.raw	5.15E3	1	1	402	412	Deamidation (NQ)
R.W(+43.01)LGLS(+14.02)AEYGNLYR.N	N	34.45	1597.7888	13	0.0	799.9017	2	33.73	7	1849	OB4035.raw	6.17E5	1	1	389	401	Carbamylation; Methylation(others)
K.TDSRPSIAN(+.98)LAG.E	N	34.14	1201.5939	12	1.0	601.8048	2	28.72	7	1485	OB4035.raw	4.76E4	1	1	471	482	Deamidation (NQ)
R.N(+58.01)ALFVPHYNTNAHSIIYALR.G	N	34.07	2371.2073	20	-0.5	791.4093	3	32.37	7	1735	OB4035.raw	1.9E5	1	1	402	421	Carboxymethyl (KW, X@N-term)
R.WLGLSAEYGN(+.98)LYR.N	N	34.02	1541.7513	13	6.2	771.8878	2	33.66	7	1830	OB4035.raw	0	0	0	389	401	Deamidation (NQ)
K.TDSRPSIAN(+.98)LAGENSVIDNLPEEVVANSYGLPR.E	Y	33.08	3497.7273	33	3.8	875.4424	4	35.42	7	1972	OB4035.raw	6.3E4	1	1	471	503	
R.VYDEELQ(+.98)EGHVLVVP(+15.99)QNFAVAGK.S	N	32.20	2557.2700	23	-3.0	853.4280	3	32.95	7	1778	OB4035.raw	0	0	0	436	458	Deamidation (NQ); Hydroxylation Pro
R.WLGLSAE(+53.92)YGN(+.98)LYR.N	N	32.10	1595.6707	13	2.2	532.8987	3	33.75	7	1837	OB4035.raw	0	0	0	389	401	Replacement of 2 protons by iron; Deamidation (NQ)
R.NALFVPHYN(+.98)TNA.H	N	32.09	1360.6411	12	1.0	681.3285	2	29.71	7	1559	OB4035.raw	5.2E3	1	1	402	413	
R.N(+42.01)ALFVP(+15.99)HYNTNAHSIIYALR.G	N	31.91	2371.2073	20	-0.5	791.4093	3	32.37	7	1748	OB4035.raw	1.9E5	1	1	402	421	Acetylation (N-term); Hydroxylation Pro
K.TDSRPSIANLA.G	N	31.18	1143.5884	11	0.3	572.8016	2	27.88	7	1465	OB4035.raw	1.36E4	1	1	471	481	
R.AHVQVVDSNGNRVYDEELQ(+.98)EGHVLVVPQNFAVAGK.S	N	31.15	3817.9023	35	1.2	764.5887	5	31.42	7	1628	OB4035.raw	1.05E6	1	1	424	458	
K.TDSRPSIANLAGENSVIDNLPEEVVAN(+.98)SYGLPR.E	Y	30.96	3497.7273	33	3.8	875.4424	4	35.42	7	1959	OB4035.raw	6.3E4	1	1	471	503	
R.AHVQVVDSNGNRVYDEELQEGHVLVVPQNFAVAGK.S	N	30.77	3816.9182	35	0.4	764.3912	5	30.69	7	1624	OB4035.raw	0	0	0	424	458	
R.VYDEELQ(+.98)EGHVLVVP(+15.99)QN(+.98)FAVAGK.S	N	30.74	2558.2539	23	2.3	853.7605	3	32.92	7	1778	OB4035.raw	7.47E3	1	1	436	458	Deamidation (NQ); Hydroxylation Pro
R.NALFVPHYNTNAHSIIYALRG.R	N	29.09	2370.2231	21	0.6	791.0821	3	31.77	7	1662	OB4035.raw	1.7E5	1	1	402	422	
R.AHVQVVDSNGNRVYDEELQ(+.98)EGHVLVVPQ(+.98)NFAVAGK.S	N	28.70	3818.8862	35	4.9	764.7883	5	31.34	7	1668	OB4035.raw	0	0	0	424	458	
N.GIEETIC(+71.04)TATVKK.N	N	28.06	1462.7701	13	0.5	732.3927	2	26.79	7	1382	OB4035.raw	3.19E3	1	1	346	358	Propionamide
R.NALFVPHY.N	N	27.94	959.4865	8	-0.7	480.7502	2	30.43	7	1610	OB4035.raw	2.39E4	1	1	402	409	
R.WLGLSAEYGNLYRN(+.98)ALFVPHYN(+.98)TNAHSIIYALR.G	N	27.92	3837.9268	33	-0.8	768.5920	5	35.14	7	1960	OB4035.raw	2.05E5	1	1	389	421	
R.W(+42.01)LGLSAE(+14.02)YGN(+.98)LYR.N	N	27.76	1597.7776	13	7.4	533.6038	3	33.73	7	1837	OB4035.raw	7.42E3	1	1	389	401	Acetylation (N-term); Deamidation (NQ)
R.NALFVPHYNTNA.H	N	27.57	1359.6571	12	0.0	680.8358	2	29.29	7	1532	OB4035.raw	7.88E3	1	1	402	413	
K.TDSRPSIAN(+.98)LA.G	N	27.12	1144.5724	11	-1.0	573.2929	2	28.82	7	1508	OB4035.raw	3.26E4	1	1	471	481	Deamidation (NQ)

Peptide	Uniq	-10lgP	Mass	Length	ppm	m/z	z	RT	Fraction	Scan	Source File	Area KOntrola 6. traka	#Feature	#Feature KOntrola 6. traka	Start	End	PTM
G.IEET(+114.04)IC(+57.02)TATVK.K	N	26.88	1377.6810	11	-0.5	689.8474	2	28.17	7	1481	OB4035.raw	9.08E3	1	1	347	357	Ubiquitin; Carbamidomethylation
R.NALFVPHYNTNAHSIIYALRGR.A	N	26.87	2526.3245	22	0.6	632.5887	4	30.52	7	1611	OB4035.raw	5.28E4	1	1	402	423	
R.AHVQVVDSN(+.98)GN(+.98)RVYDEELQEGHVLVVPQNFAVAGK.S	N	26.20	3818.8862	35	6.3	955.7348	4	30.87	7	1636	OB4035.raw	0	0	0	424	458	
R.WLGLSAEYGN(+.98)LYRNALFVPHYN(+.98)TN(+.98)AHSIIYALR.G	N	25.93	3838.9106	33	5.5	768.7936	5	35.67	7	1978	OB4035.raw	0	0	0	389	421	
G.SLKTANELN(+.98)LLILR.W	N	25.84	1597.9403	14	-0.9	533.6536	3	32.92	7	1777	OB4035.raw	4.54E4	1	1	375	388	
R.VYDEELQEGHVLVVP(+15.99)QNFAVAGK.S	N	25.47	2556.2859	23	6.6	853.1082	3	33.01	7	1778	OB4035.raw	7.76E3	1	1	436	458	Hydroxylation Pro
K.T(+42.01)AN(+.98)ELN(+.98)LLILR.W	N	25.44	1312.7238	11	8.7	657.3749	2	34.04	7	1880	OB4035.raw	4.57E4	1	1	378	388	Acetylation (N-term); Deamidation (NQ)
K.FFVPPSQ(+.98)Q(+.98)SPR.A	N	25.37	1290.6244	11	-0.6	646.3191	2	28.17	7	1475	OB4035.raw	4.4E3	1	1	517	527	Deamidation (NQ)
R.WLGLS(+58.03)AEYGNLYR.N	N	25.07	1598.7966	13	-3.9	800.4025	2	33.19	7	1791	OB4035.raw	4.21E3	1	1	389	401	2,3-dihydro-2,2-dimethyl-7-benzofuranol N-methyl carbamate
K.SQSDNFEYVAFKTDSRP(+15.99)SIANLAGENSVIDNLPEEVVANSYGLPR.E	Y	24.56	4928.3740	45	4.0	1233.1057	4	36.24	7	2014	OB4035.raw	0	0	0	459	503	
R.WLGLS(-18.01)AEYGNLYR.N	N	24.29	1522.7568	13	1.7	762.3870	2	39.61	7	2224	OB4035.raw	3.31E4	1	1	389	401	
K.T(-15.01)ANELNLLILR(+14.02)WLGLSAEYGNLYR.N	N	23.61	2790.5068	24	-4.9	931.1716	3	42.54	7	2404	OB4035.raw	0	0	0	378	401	ISD (z+2)-series; Methylation(KR)
K.TANE(+21.98)LNLLILR.W	N	23.43	1290.7272	11	-0.2	646.3707	2	33.73	7	1840	OB4035.raw	1.63E4	1	1	378	388	Sodium adduct
R.NRSPDIYN(+.98)PQ(+.98)AGSLK.T	N	23.21	1660.8057	15	-0.7	554.6088	3	27.13	7	1410	OB4035.raw	1.72E4	1	1	363	377	Deamidation (NQ)
K.TDSRPSIANLAG.E	N	23.17	1200.6099	12	0.1	601.3123	2	27.97	7	1447	OB4035.raw	2.3E4	1	1	471	482	
R.W(+42.01)(+15.99)LGLSAEYGNLYR.N	N	22.56	1598.7728	13	-2.0	800.3921	2	34.11	7	1862	OB4035.raw	4.12E4	1	1	389	401	Acetylation (N-term); Oxidation (HW)
R.AHVQ(+.98)VVDSNGNRVYDEELQEGHVLVVPQNFAVAGK.S	N	22.40	3817.9023	35	1.2	764.5887	5	31.42	7	1642	OB4035.raw	1.05E6	1	1	424	458	
R.AHVQVVDSNGNRVYDEELQEGHVLVVPQ(+.98)N(+.98)FAVAGK.S	N	22.34	3818.8862	35	1.5	764.7856	5	31.51	7	1680	OB4035.raw	0	0	0	424	458	
R.N(+.98)RSPDIYNPQAGSLK.T	N	22.33	1659.8217	15	-0.5	554.2809	3	26.23	7	1389	OB4035.raw	1.47E5	1	1	363	377	
R.AHVQ(+.98)VVDSNGN(+.98)RVYDEELQEGHVLVVPQN(+.98)FAVAGK.S	N	22.20	3819.8704	35	7.5	764.9871	5	32.04	7	1717	OB4035.raw	0	0	0	424	458	
**total 125 peptides**																	

tr|Q9FZ11|Q9FZ11_ARAHY
back to list

| Protein Coverage | Supporting Peptides |

Protein Coverage:

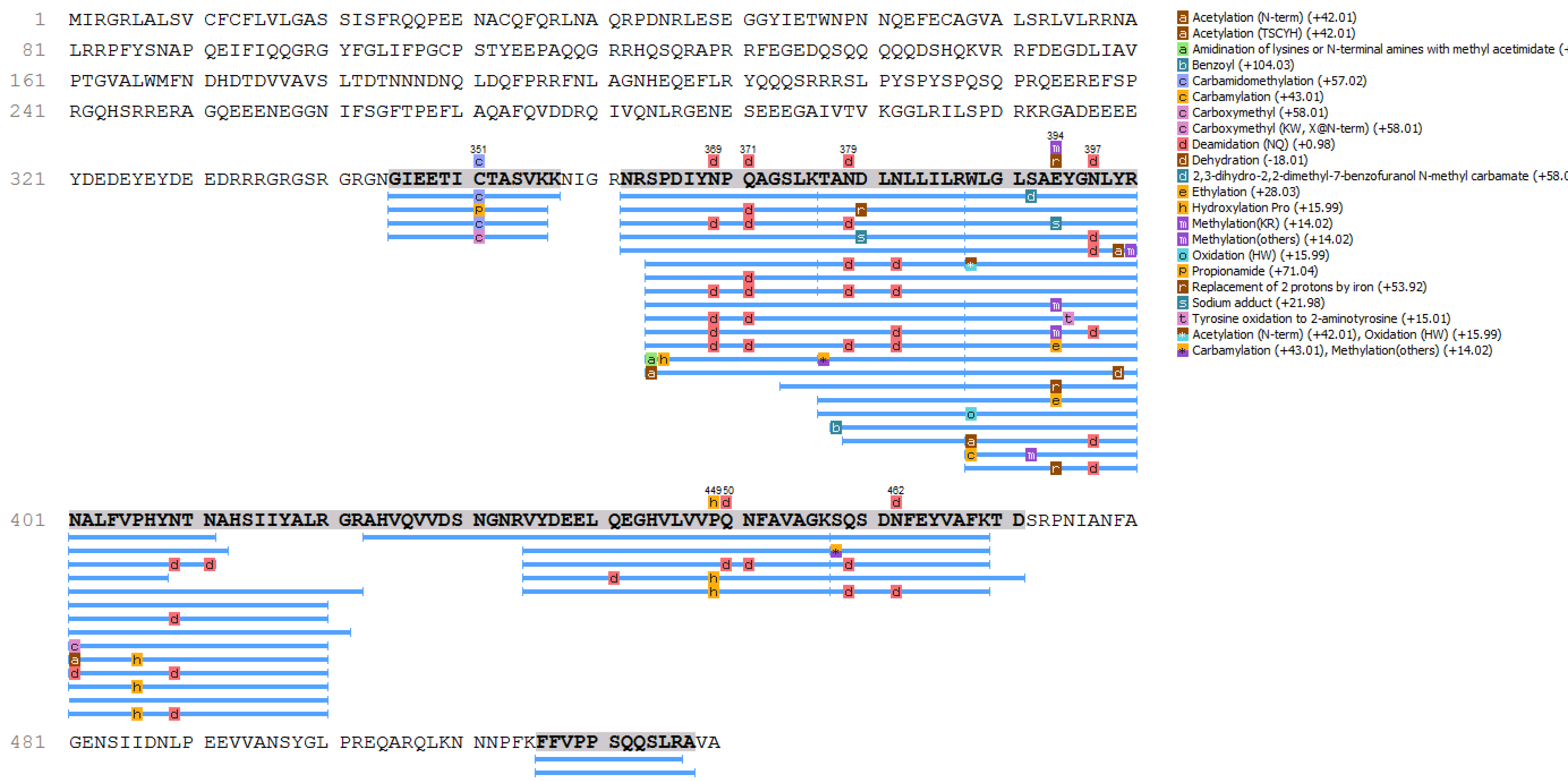
1 MIRGRLALSV CFCFLVLGAS SISFRQQPEE NACQFQRLNA QRPDNRLESE GGYIETWNPN NQEFECAGVA LSRLVLRRNA
81 LRRPFYSNAP QEIFIQQGRG YFGLIFPGCP STYEEPAQQG RRHQSQRAPR RFEGEDQSQQ QQQDSHQKVR RFDEGDLIAV
161 PTGVALWMFN DHDTDVVAVS LTDTNNNDNQ LDQFPRRFNL AGNHEQEFLR YQQQSRRRSL PYSPYSPQSQ PRQEEREFSP
241 RGQHSRRERA GQEEENEGGN IFSGFTPEFL AQAFQVDDRQ IVQNLRGENE SEEEGAIVTV KGGLRILSPD RKRGADEEEE
351 369 371 379 394 397
321 YDEDEYEYDE EDRRRGRGSR GRGNGIEETI CTASVKKNIG RNRSPDIYNP QAGSLKTAND LNLLILRWLG LSAEYGNLYR
449 450 462
401 NALFVPHYNT NAHSIIYALR GRAHVQVVDS NGNRVYDEEL QEGHVLVVPQ NFAVAGKSQS DNFEYVAFKT DSRPNIANFA
481 GENSIIDNLP EEVVANSYGL PREQARQLKN NNPFKFFVPP SQQSLRAVA
a Acetylation (N-term) (+42.01)
a Acetylation (TSCYH) (+42.01)
a Amidination of lysines or N-terminal amines with methyl acetimidate (
b Benzoyl (+104.03)
c Carbamidomethylation (+57.02)
c Carbamylation (+43.01)
c Carboxymethyl (+58.01)
c Carboxymethyl (KW, X@N-term) (+58.01)
d Deamidation (NQ) (+0.98)
d Dehydration (-18.01)
d 2,3-dihydro-2,2-dimethyl-7-benzofuranol N-methyl carbamate (+58.
e Ethylation (+28.03)
h Hydroxylation Pro (+15.99)
m Methylation(KR) (+14.02)
m Methylation(others) (+14.02)
o Oxidation (HW) (+15.99)
p Propionamide (+71.04)
r Replacement of 2 protons by iron (+53.92)
s Sodium adduct (+21.98)
t Tyrosine oxidation to 2-aminotyrosine (+15.01)
* Acetylation (N-term) (+42.01), Oxidation (HW) (+15.99)
* Carbamylation (+43.01), Methylation(others) (+14.02)

Supporting Peptides:

Peptide	Uniq	-10lgP	Mass	Length	ppm	m/z	z	RT	Fraction	Scan	Source File	Area KOntrola 6. traka	#Feature	#Feature KOntrola 6. traka	Start	End	PTM
R.WLGLSAEYGNLYR.N	N	95.34	1540.7673	13	1.0	771.3917	2	33.34	7	1963	OB4035.raw	1.45E7	5	5	388	400	
K.SQSDNFEYVAFK.T	N	86.92	1433.6462	12	0.9	717.8311	2	30.43	7	1599	OB4035.raw	8.2E5	1	1	458	469	
R.SPDIYNPQAGSLKTANDLNLLILR.W	N	86.57	2625.4126	24	0.8	876.1455	3	33.65	7	1826	OB4035.raw	2.37E5	1	1	364	387	
K.TANDLNLLILRWLGLSAEYGNLYR.N	N	84.26	2777.4863	24	0.8	926.8368	3	39.78	7	2235	OB4035.raw	2.55E5	2	2	377	400	
R.SPDIYNPQ(+.98)AGSLKTANDLNLLILR.W	N	83.88	2626.3965	24	0.8	876.4735	3	34.04	7	1854	OB4035.raw	1.03E6	1	1	364	387	
R.N(+.98)ALFVPHYN(+.98)TNAHSIIYALR.G	N	83.79	2315.1699	20	1.1	772.7314	3	32.66	7	1752	OB4035.raw	2.44E5	1	1	401	420	Deamidation (NQ)
K.SQ(+.98)SDNFEYVAFK.T	N	79.67	1434.6302	12	0.3	718.3226	2	30.79	7	1627	OB4035.raw	3.08E5	1	1	458	469	Deamidation (NQ)
K.TANDLN(+.98)LLILRWLGLSAEYGNLYR.N	N	74.56	2778.4705	24	4.0	927.1678	3	40.91	7	2304	OB4035.raw	0	0	0	377	400	
R.WLGLSAE(+28.03)YGNLYR.N	N	74.46	1568.7987	13	0.9	785.4073	2	34.89	7	1929	OB4035.raw	1.15E5	1	1	388	400	Ethylation
R.NALFVPHYN(+.98)TNAHSIIYALR.G	N	74.22	2314.1858	20	1.8	772.4039	3	31.94	7	1699	OB4035.raw	9.78E6	4	4	401	420	Deamidation (NQ)
R.SPDIYNPQAGSLK.T	N	72.75	1388.6936	13	0.2	695.3542	2	27.39	7	1421	OB4035.raw	1.04E5	1	1	364	376	
R.NALFVPHYNTNAHSIIYALR.G	N	72.27	2313.2019	20	0.8	772.0752	3	31.23	7	1675	OB4035.raw	5.74E6	2	2	401	420	
R.SPDIYNPQ(+.98)AGSLK.T	N	71.57	1389.6776	13	0.9	695.8467	2	28.07	7	1461	OB4035.raw	1.89E5	1	1	364	376	Deamidation (NQ)
N.GIEETIC(+57.02)TASVK.K	N	71.53	1306.6438	12	0.7	654.3297	2	27.80	7	1440	OB4035.raw	5.54E4	1	1	345	356	Carbamidomethylation
R.SPDIYN(+.98)PQAGSLKTANDLN(+.98)LLILR.W	N	70.53	2627.3806	24	5.1	876.8053	3	34.72	7	1912	OB4035.raw	0	0	0	364	387	Deamidation (NQ)
R.SPDIYN(+.98)PQ(+.98)AGSLKTANDLNLLILR.W	N	68.73	2627.3806	24	1.7	876.8023	3	34.36	7	1884	OB4035.raw	0	0	0	364	387	Deamidation (NQ)
R.VYDEELQEGHVLVVPQN(+.98)FAVAGK.S	N	68.59	2541.2751	23	0.7	848.0996	3	32.01	7	1729	OB4035.raw	2.05E5	1	1	435	457	
K.TAN(+.98)DLNLLILRWLGLSAEYGNLYR.N	N	67.21	2778.4705	24	7.0	927.1705	3	41.57	7	2344	OB4035.raw	0	0	0	377	400	
R.SPDIYN(+.98)PQAGSLK.T	N	66.40	1389.6776	13	0.9	695.8467	2	28.07	7	1450	OB4035.raw	1.89E5	1	1	364	376	
K.SQSDNFEYVAFKTD.S	N	65.50	1649.7209	14	0.4	825.8681	2	30.88	7	1634	OB4035.raw	6.42E4	1	1	458	471	
R.WLGLSAE(+14.02)YGNLYR.N	N	64.20	1554.7831	13	-0.4	778.3985	2	34.13	7	1867	OB4035.raw	0	0	0	388	400	Methylation(others)
R.SPDIYN(+.98)PQ(+.98)AGSLK.T	N	63.44	1390.6616	13	0.4	696.3384	2	28.62	7	1501	OB4035.raw	3.67E4	1	1	364	376	Deamidation (NQ)
R.SPDIYN(+.98)PQAGSLKTANDLNLLILR.W	N	62.73	2626.3965	24	0.3	876.4730	3	35.57	7	1972	OB4035.raw	0	0	0	364	387	
R.WLGLSAE(+53.92)YGNLYR.N	N	56.14	1594.6866	13	-0.3	532.5693	3	33.34	7	1819	OB4035.raw	5.46E5	1	1	388	400	Replacement of 2 protons by iron
R.NRSPDIYNPQ(+.98)AGSLKTAN(+.98)DLNLLILR.W	N	56.12	2897.5247	26	4.9	725.3920	4	34.35	7	1904	OB4035.raw	3.34E4	1	1	362	387	
R.NRSPDIYNPQAGSLK.T	N	54.77	1658.8376	15	0.5	830.4265	2	25.62	7	1308	OB4035.raw	9.56E4	3	3	362	376	
K.TAN(+.98)DLN(+.98)LLILRWLGLSAEYGNLYR.N	N	54.63	2779.4543	24	2.9	927.4948	3	41.27	7	2326	OB4035.raw	0	0	0	377	400	Deamidation (NQ)
N.GIEETIC(+57.02)TASVKK.N	N	53.72	1434.7388	13	0.1	718.3767	2	25.95	7	1325	OB4035.raw	7.84E4	2	2	345	357	Carbamidomethylation
R.NRSPDIYN(+.98)PQAGSLKTAN(+.98)DLNLLILR.W	N	53.50	2897.5247	26	4.1	725.3914	4	33.94	7	1852	OB4035.raw	0	0	0	362	387	

Peptide	Uniq	-10lgP	Mass	Length	ppm	m/z	z	RT	Fraction	Scan	Source File	Area KOntrola 6. traka	#Feature	#Feature KOntrola 6. traka	Start	End	PTM
R.NRSPDIYNPQ(+.98)AGSLKTANDLNLLILR.W	N	52.65	2896.5405	26	0.8	725.1430	4	32.66	7	1765	OB4035.raw	8.81E5	1	1	362	387	
R.SPDIYN(+.98)PQ(+.98)AGSLKTAN(+.98)DLN(+.98)LLILR.W	N	52.36	2629.3486	24	7.1	877.4630	3	34.83	7	1912	OB4035.raw	1.17E5	1	1	364	387	Deamidation (NQ)
R.VYDEELQEGHVLVVPQNFAVAGK.S	N	51.87	2540.2910	23	0.6	847.7714	3	31.67	7	1692	OB4035.raw	1.53E5	1	1	435	457	
R.NRSPDIYNPQ(+.98)AGSLK.T	N	51.37	1659.8217	15	-0.8	830.9175	2	26.31	7	1347	OB4035.raw	1.5E5	2	2	362	376	Deamidation (NQ)
K.TANDLNLLILR.W	N	50.78	1254.7296	11	0.0	628.3721	2	33.43	7	1928	OB4035.raw	5.74E6	1	1	377	387	
R.NRSPDIYN(+.98)PQ(+.98)AGSLKTAN(+.98)DLNLLILR.W	N	50.70	2898.5085	26	1.8	725.6357	4	33.73	7	1852	OB4035.raw	8.77E4	1	1	362	387	
R.NALFVP(+15.99)HYN(+.98)TNAHSIIYALR.G	N	49.25	2330.1807	20	0.0	777.7341	3	33.43	7	1816	OB4035.raw	1.08E5	1	1	401	420	Hydroxylation Pro; Deamidation (NQ)
K.T(+43.01)(+14.02)ANDLNLLILR.W	N	48.90	1311.7510	11	0.4	656.8831	2	33.65	7	1825	OB4035.raw	1.5E5	1	1	377	387	Carbamylation; Methylation(others)
K.S(+43.01)(+14.02)QSDNFEYVAFK.T	N	46.98	1490.6677	12	0.1	746.3412	2	30.52	7	1618	OB4035.raw	3.2E4	1	1	458	469	Carbamylation; Methylation(others)
K.SQ(+.98)SDN(+.98)FEYVAFK.T	N	46.28	1435.6143	12	2.7	718.8163	2	31.28	7	1664	OB4035.raw	7.74E3	1	1	458	469	Deamidation (NQ)
R.NRSPDIYNPQAGSLKTAN(+.98)DLNLLILR.W	N	46.23	2896.5405	26	0.8	725.1430	4	32.66	7	1753	OB4035.raw	8.81E5	1	1	362	387	
R.WLGLSAE(+14.02)YGN(+.98)LYR.N	N	45.38	1555.7671	13	-1.2	778.8899	2	34.58	7	1900	OB4035.raw	1.91E4	1	1	388	400	Methylation(others); Deamidation (NQ)
R.NALFVP(+15.99)HYNTNAHSIIYALR.G	N	44.60	2329.1968	20	0.1	777.4063	3	32.92	7	1784	OB4035.raw	1.3E5	2	2	401	420	Hydroxylation Pro
R.NRSPDIYNPQAGSLKTANDLNLLILR.W	N	43.70	2895.5566	26	0.7	724.8970	4	32.30	7	1734	OB4035.raw	0	0	0	362	387	
K.TAN(+.98)DLNLLILR.W	N	43.37	1255.7136	11	1.3	628.8649	2	34.04	7	1848	OB4035.raw	1.97E6	1	1	377	387	Deamidation (NQ)
A.NDLNLLILR.W	N	43.35	1082.6448	9	-0.8	542.3292	2	33.34	7	1810	OB4035.raw	2.03E4	1	1	379	387	
K.TAND(+28.03)LNLLILR.W	N	43.32	1282.7609	11	-0.2	642.3876	2	34.83	7	1924	OB4035.raw	9.42E4	1	1	377	387	
R.AHVQVVDSN(+.98)GN(+.98)RVYDEELQ(+.98)EGHVLVVPQNFAVAGK.S	N	43.28	3819.8704	35	5.2	955.9799	4	31.40	7	1672	OB4035.raw	0	0	0	423	457	
R.VYDEELQEGHVLVVPQ(+.98)N(+.98)FAVAGK.S	N	42.89	2542.2590	23	-0.1	848.4269	3	32.47	7	1755	OB4035.raw	1.18E5	1	1	435	457	Deamidation (NQ)
R.NRSPDIYN(+.98)PQAGSLK.T	N	42.75	1659.8217	15	-0.4	554.2809	3	25.82	7	1316	OB4035.raw	1.71E5	2	2	362	376	
R.NRSPDIYNP(+15.99)QAGSLKTANDLNLLILR.W	N	42.57	2911.5515	26	3.0	728.8973	4	34.73	7	1936	OB4035.raw	7.19E4	1	1	362	387	
R.S(+41.03)P(+15.99)DIYNPQAGSLK.T	N	42.04	1445.7150	13	0.0	723.8647	2	27.55	7	1427	OB4035.raw	5.56E3	1	1	364	376	Amidination of lysines or N-terminal amines with methyl acetimidate; Hydroxylation Pro
K.TAN(+.98)DLNLLILRW(+15.99)LGLSAEYGNLYR.N	N	41.41	2794.4653	24	9.2	932.5043	3	41.41	7	2334	OB4035.raw	0	0	0	377	400	Oxidation (HW)
R.WLGLSAE(+21.98)YGNLYR.N	N	41.38	1562.7494	13	-8.5	521.9193	3	33.34	7	1807	OB4035.raw	1.16E5	2	2	388	400	Sodium adduct
R.NALFVPHYNTN(+.98).A	N	41.37	1289.6040	11	0.3	645.8094	2	29.66	7	1549	OB4035.raw	3.19E4	1	1	401	411	

Peptide	Uniq	-10lgP	Mass	Length	ppm	m/z	z	RT	Fraction	Scan	Source File	Area KOntrola 6. traka	#Feature	#Feature KOntrola 6. traka	Start	End	PTM
R.WLGLSAEYGNLY(-18.01)R.N	N	41.07	1522.7568	13	1.7	762.3870	2	39.61	7	2227	OB4035.raw	3.31E4	1	1	388	400	Dehydration
K.TANDLNLLILRWLGLSAE(+28.03)YGNLYR.N	N	40.66	2805.5176	24	1.7	936.1814	3	40.41	7	2270	OB4035.raw	2.22E4	1	1	377	400	Ethylation
R.AHVQVVDSN(+.98)GNRVYDEELQ(+.98)EGHVLVVPQNFAVAGK.S	N	40.63	3818.8862	35	3.5	955.7322	4	31.54	7	1682	OB4035.raw	0	0	0	423	457	
R.NRSPDIYNP(+15.99)QAGSLKTAN(+.98)DLNLLILR.W	N	40.29	2912.5354	26	3.8	729.1439	4	33.34	7	1836	OB4035.raw	6.68E5	1	1	362	387	
R.WLGLSAEY(+15.01)GNLYR.N	N	39.61	1555.7783	13	-8.4	778.8899	2	34.58	7	1894	OB4035.raw	1.91E4	1	1	388	400	Tyrosine oxidation to 2-aminotyrosine
R.NRSPDIYNP(+15.99)QAGSLKTANDLN(+.98)LLILR.W	N	39.51	2912.5354	26	3.8	729.1439	4	33.34	7	1818	OB4035.raw	6.68E5	1	1	362	387	
R.N(+.98)ALFVPHYNTNAHSIIYALR.G	N	39.24	2314.1858	20	2.1	579.5549	4	31.94	7	1776	OB4035.raw	4.84E6	1	1	401	420	
K.TAN(+.98)DLN(+.98)LLILR.W	N	39.22	1256.6976	11	2.9	629.3579	2	34.54	7	1897	OB4035.raw	3.72E4	1	1	377	387	Deamidation (NQ)
R.S(+42.01)P(+15.99)DIYNPQAGSLKTANDLNLLILR.W	N	38.78	2683.4180	24	-0.5	895.4795	3	34.11	7	1864	OB4035.raw	9.81E4	1	1	364	387	Acetylation (N-term)
R.NRSPDIYNPQAGSLKTAN(+.98)DLN(+.98)LLILR.W	N	38.68	2897.5247	26	1.0	725.3892	4	33.45	7	1814	OB4035.raw	0	0	0	362	387	
K.TAND(+53.92)LNLLILR.W	N	37.22	1308.6488	11	0.0	655.3317	2	33.43	7	1821	OB4035.raw	5.21E4	1	1	377	387	Replacement of 2 protons by iron
R.AHVQVVDSN(+.98)GNRVYDEELQEGHVLVVPQNFAVAGK.S	N	36.49	3817.9023	35	-0.8	955.4821	4	31.34	7	1648	OB4035.raw	2.77E5	1	1	423	457	
R.NALFVP(+15.99)H(+42.01)YNTNAHSIIYALR.G	N	36.41	2371.2073	20	8.0	791.4160	3	31.23	7	1662	OB4035.raw	9.72E4	1	1	401	420	Hydroxylation Pro
R.AHVQVVDSNGN(+.98)RVYDEELQEGHVLVVPQNFAVAGK.S	N	36.28	3817.9023	35	-0.8	955.4821	4	31.34	7	1663	OB4035.raw	2.77E5	1	1	423	457	
R.WLGLSAEYGN(+.98)LY(+42.01)R(+14.02).N	N	35.60	1597.7776	13	7.0	799.9017	2	33.73	7	1791	OB4035.raw	6.17E5	1	1	388	400	Deamidation (NQ); Acetylation (TSCYH); Methylation(KR)
G.SLKTANDLN(+.98)LLILR.W	N	35.26	1583.9246	14	-0.3	528.9820	3	32.66	7	1759	OB4035.raw	5.11E4	1	1	374	387	
R.WLGLSAEYGNLYRN(+.98)ALFVPHYN(+.98)TN(+.98)AHSIIYALR.G	N	35.11	3838.9106	33	1.1	768.7903	5	35.86	7	1990	OB4035.raw	0	0	0	388	420	
K.FFVPPSQQ(+.98)SLR.A	Y	34.93	1305.6716	11	0.8	653.8436	2	29.65	7	1537	OB4035.raw	7.54E4	1	1	516	526	
R.NALFVPHYN(+.98)TN(+.98).A	N	34.82	1290.5880	11	-0.2	646.3011	2	29.97	7	1580	OB4035.raw	5.15E3	1	1	401	411	Deamidation (NQ)
R.W(+43.01)LGLS(+14.02)AEYGNLYR.N	N	34.45	1597.7888	13	0.0	799.9017	2	33.73	7	1849	OB4035.raw	6.17E5	1	1	388	400	Carbamylation; Methylation(others)
R.N(+58.01)ALFVPHYNTNAHSIIYALR.G	N	34.07	2371.2073	20	-0.5	791.4093	3	32.37	7	1735	OB4035.raw	1.9E5	1	1	401	420	Carboxymethyl (KW, X@N-term)
R.WLGLSAEYGN(+.98)LYR.N	N	34.02	1541.7513	13	6.2	771.8878	2	33.66	7	1830	OB4035.raw	0	0	0	388	400	Deamidation (NQ)
R.VYDEELQ(+.98)EGHVLVVP(+15.99)QNFAVAGK.S	N	32.20	2557.2700	23	-3.0	853.4280	3	32.95	7	1778	OB4035.raw	0	0	0	435	457	Deamidation (NQ); Hydroxylation Pro
R.WLGLSAE(+53.92)YGN(+.98)LYR.N	N	32.10	1595.6707	13	2.2	532.8987	3	33.75	7	1837	OB4035.raw	0	0	0	388	400	Replacement of 2 protons by iron; Deamidation (NQ)

Peptide	Uniq	-10lgP	Mass	Length	ppm	m/z	z	RT	Fraction	Scan	Source File	Area KOntrola 6. traka	#Feature	#Feature KOntrola 6. traka	Start	End	PTM
R.NALFVPHYN(+.98)TNA.H	N	32.09	1360.6411	12	1.0	681.3285	2	29.71	7	1559	OB4035.raw	5.2E3	1	1	401	412	
R.N(+42.01)ALFVP(+15.99)HYNTNAHSIIYALR.G	N	31.91	2371.2073	20	-0.5	791.4093	3	32.37	7	1748	OB4035.raw	1.9E5	1	1	401	420	Acetylation (N-term); Hydroxylation Pro
R.SPDIYN(+.98)PQ(+.98)AGSLKTAN(+.98)DLNLLILR.W	N	31.82	2628.3645	24	7.6	658.1034	4	34.43	7	1880	OB4035.raw	1.37E4	1	1	364	387	
R.AHVQVVDSNGNRVYDEELQ(+.98)EGHVLVVPQNFAVAGK.S	N	31.15	3817.9023	35	1.2	764.5887	5	31.42	7	1628	OB4035.raw	1.05E6	1	1	423	457	
R.AHVQVVDSNGNRVYDEELQEGHVLVVPQNFAVAGK.S	N	30.77	3816.9182	35	0.4	764.3912	5	30.69	7	1624	OB4035.raw	0	0	0	423	457	
R.VYDEELQ(+.98)EGHVLVVP(+15.99)QN(+.98)FAVAGK.S	N	30.74	2558.2539	23	2.3	853.7605	3	32.92	7	1778	OB4035.raw	7.47E3	1	1	435	457	Deamidation (NQ); Hydroxylation Pro
R.SPD(+21.98)IYNPQAGS(-18.01)LKTANDLNLLILR.W	N	30.41	2629.3840	24	1.5	877.4700	3	34.43	7	1884	OB4035.raw	1.89E5	1	1	364	387	
K.TANDLN(+.98)LLILRW(+15.99)LGLSAEYGNLYR.N	N	30.14	2794.4653	24	4.6	932.5000	3	42.12	7	2378	OB4035.raw	0	0	0	377	400	Oxidation (HW)
R.NRSPDIYN(+.98)P(+15.99)Q(+.98)AGSLKTANDLNLLILR.W	N	29.40	2913.5195	26	-0.5	729.3868	4	34.64	7	1905	OB4035.raw	0	0	0	362	387	
R.NALFVPHYNTNAHSIIYALRG.R	N	29.09	2370.2231	21	0.6	791.0821	3	31.77	7	1662	OB4035.raw	1.7E5	1	1	401	421	
R.AHVQVVDSNGNRVYDEELQ(+.98)EGHVLVVPQ(+.98)NFAVAGK.S	N	28.70	3818.8862	35	4.9	764.7883	5	31.34	7	1668	OB4035.raw	0	0	0	423	457	
T.A(+104.03)NDLNLLILRWLGLSAEYGNLYR.N	N	28.35	2780.4648	23	2.6	927.8313	3	40.90	7	2321	OB4035.raw	7.62E3	1	1	378	400	Benzoyl
R.NALFVPHY.N	N	27.94	959.4865	8	-0.7	480.7502	2	30.43	7	1610	OB4035.raw	2.39E4	1	1	401	408	
R.WLGLSAEYGNLYRN(+.98)ALFVPHYN(+.98)TNAHSIIYALR.G	N	27.92	3837.9268	33	-0.8	768.5920	5	35.14	7	1960	OB4035.raw	2.05E5	1	1	388	420	
R.W(+42.01)LGLSAE(+14.02)YGN(+.98)LYR.N	N	27.76	1597.7776	13	7.4	533.6038	3	33.73	7	1837	OB4035.raw	7.42E3	1	1	388	400	Acetylation (N-term); Deamidation (NQ)
R.NALFVPHYNTNA.H	N	27.57	1359.6571	12	0.0	680.8358	2	29.29	7	1532	OB4035.raw	7.88E3	1	1	401	412	
R.NRSPDIYNPQAGSLKT(-18.01)AND(+21.98)LNLLILR.W	N	26.92	2899.5281	26	2.0	725.8907	4	33.01	7	1797	OB4035.raw	4.04E5	2	2	362	387	Sodium adduct
R.NALFVPHYNTNAHSIIYALRGR.A	N	26.87	2526.3245	22	0.6	632.5887	4	30.52	7	1611	OB4035.raw	5.28E4	1	1	401	422	
K.FFVPPSQQ(+.98)SLRA.V	Y	26.50	1376.7087	12	0.8	689.3622	2	30.07	7	1573	OB4035.raw	1.45E5	1	1	516	527	
R.AHVQVVDSN(+.98)GN(+.98)RVYDEELQEGHVLVVPQNFAVAGK.S	N	26.20	3818.8862	35	6.3	955.7348	4	30.87	7	1636	OB4035.raw	0	0	0	423	457	
R.WLGLSAEYGN(+.98)LYRNALFVPHYN(+.98)TN(+.98)AHSIIYALR.G	N	25.93	3838.9106	33	5.5	768.7936	5	35.67	7	1978	OB4035.raw	0	0	0	388	420	
R.VYDEELQEGHVLVVP(+15.99)QNFAVAGK.S	N	25.47	2556.2859	23	6.6	853.1082	3	33.01	7	1778	OB4035.raw	7.76E3	1	1	435	457	Hydroxylation Pro
R.WLGLS(+58.03)AEYGNLYR.N	N	25.07	1598.7966	13	-3.9	800.4025	2	33.19	7	1791	OB4035.raw	4.21E3	1	1	388	400	2,3-dihydro-2,2-dimethyl-7-benzofuranol N-methyl carbamate
R.WLGLS(-18.01)AEYGNLYR.N	N	24.29	1522.7568	13	1.7	762.3870	2	39.61	7	2224	OB4035.raw	3.31E4	1	1	388	400	
R.NRSPDIYN(+.98)PQ(+.98)AGSLK.T	N	23.21	1660.8057	15	-0.7	554.6088	3	27.13	7	1410	OB4035.raw	1.72E4	1	1	362	376	Deamidation (NQ)
N.GIEETIC(+71.04)TASVK.K	N	22.64	1320.6595	12	-0.7	661.3366	2	27.68	7	1436	OB4035.raw	0	0	0	345	356	Propionamide

Peptide	Uniq	-10lgP	Mass	Length	ppm	m/z	z	RT	Fraction	Scan	Source File	Area KOntrola 6. traka	#Feature	#Feature KOntrola 6. traka	Start	End	PTM
R.W(+42.01)(+15.99)LGLSAEYGNLYR.N	N	22.56	1598.7728	13	-2.0	800.3921	2	34.11	7	1862	OB4035.raw	4.12E4	1	1	388	400	Acetylation (N-term); Oxidation (HW)
N.GIEETIC(+58.01)TASVK.K	N	22.53	1307.6278	12	1.7	654.8223	2	28.82	7	1505	OB4035.raw	5.31E3	1	1	345	356	Carboxymethyl
R.AHVQ(+.98)VVDSNGNRVYDEELQEGHVLVVPQNFAVAGK.S	N	22.40	3817.9023	35	1.2	764.5887	5	31.42	7	1642	OB4035.raw	1.05E6	1	1	423	457	
R.SPDIYNPQAGSLKT(-18.01)AND(+21.98)LNLLILR.W	N	22.35	2629.3840	24	1.5	877.4700	3	34.43	7	1912	OB4035.raw	1.89E5	1	1	364	387	
R.AHVQVVDSNGNRVYDEELQEGHVLVVPQ(+.98)N(+.98)FAVAGK.S	N	22.34	3818.8862	35	1.5	764.7856	5	31.51	7	1680	OB4035.raw	0	0	0	423	457	
R.N(+.98)RSPDIYNPQAGSLK.T	N	22.33	1659.8217	15	-0.5	554.2809	3	26.23	7	1389	OB4035.raw	1.47E5	1	1	362	376	
R.AHVQ(+.98)VVDSNGN(+.98)RVYDEELQEGHVLVVPQN(+.98)FAVAGK.S	N	22.20	3819.8704	35	7.5	764.9871	5	32.04	7	1717	OB4035.raw	0	0	0	423	457	
**total 111 peptides**																	

tr|Q8LKN1|Q8LKN1_ARAHY
back to list

| Protein Coverage | Supporting Peptides |

Protein Coverage:

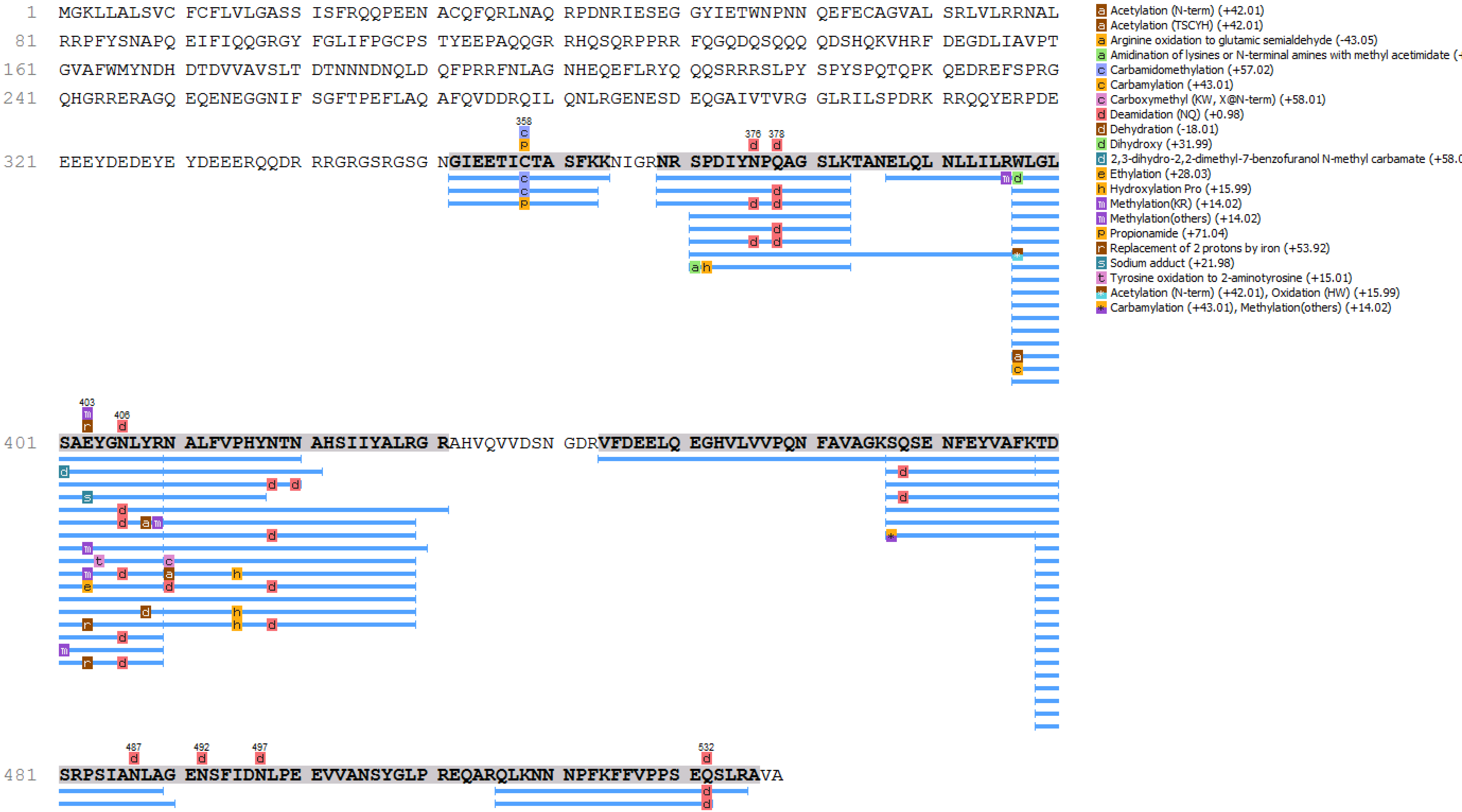
1 MGKLLALSVC FCFLVLGASS ISFRQQPEEN ACQFQRLNAQ RPDNRIESEG GYIETWNPNN QEFECAGVAL SRLVLRRNAL
81 RRPFYSNAPQ EIFIQQGRGY FGLIFPGCPS TYEEPAQQGR RHQSQRPPRR FQGQDQSQQQ QDSHQKVHRF DEGDLIAVPT
161 GVAFWMYNDH DTDVVAVSLT DTNNNDNQLD QFPRRFNLAG NHEQEFLRYQ QQSRRRSLPY SPYSPQTQPK QEDREFSPRG
241 QHGRRERAGQ EQENEGGNIF SGFTPEFLAQ AFQVDDRQIL QNLRGENESD EQGAIVTVRG GLRILSPDRK RRQQYERPDE
358
376 378
321 EEEYDEDEYE YDEEERQQDR RRGRGSRGSG NGIEETICTA SFKKNIGRNR SPDIYNPQAG SLKTANELQL NLLILRWLGL
403 406
401 SAEYGNLYRN ALFVPHYNTN AHSIIYALRG RAHVQVVDSN GDRVFDEELQ EGHVLVVPQN FAVAGKSQSE NFEYVAFKTD
487 492 497 532
481 SRPSIANLAG ENSFIDNLPE EVVANSYGLP REQARQLKNN NPFKFFVPPS EQSLRAVA
Acetylation (N-term) (+42.01)
Acetylation (TSCYH) (+42.01)
Arginine oxidation to glutamic semialdehyde (-43.05)
Amidination of lysines or N-terminal amines with methyl acetimidate (
Carbamidomethylation (+57.02)
Carbamylation (+43.01)
Carboxymethyl (KW, X@N-term) (+58.01)
Deamidation (NQ) (+0.98)
Dehydration (-18.01)
Dihydroxy (+31.99)
2,3-dihydro-2,2-dimethyl-7-benzofuranol N-methyl carbamate (+58.
Ethylation (+28.03)
Hydroxylation Pro (+15.99)
Methylation(KR) (+14.02)
Methylation(others) (+14.02)
Propionamide (+71.04)
Replacement of 2 protons by iron (+53.92)
Sodium adduct (+21.98)
Tyrosine oxidation to 2-aminotyrosine (+15.01)
Acetylation (N-term) (+42.01), Oxidation (HW) (+15.99)
Carbamylation (+43.01), Methylation(others) (+14.02)

Supporting Peptides:

Peptide	Uniq	-10lgP	Mass	Length	ppm	m/z	z	RT	Fraction	Scan	Source File	Area KOntrola 6. traka	#Feature	#Feature KOntrola 6. traka	Start	End	PTM
R.WLGLSAEYGNLYR.N	N	95.34	1540.7673	13	1.0	771.3917	2	33.34	7	1963	OB4035.raw	1.45E7	5	5	397	409	
K.SQSENFEYVAFK.T	N	92.19	1447.6619	12	1.3	724.8392	2	30.26	7	1587	OB4035.raw	1.74E6	1	1	467	478	
K.SQ(+.98)SENFEYVAFK.T	N	84.36	1448.6459	12	0.1	725.3303	2	30.61	7	1614	OB4035.raw	4.98E5	2	2	467	478	Deamidation (N
R.N(+.98)ALFVPHYN(+.98)TNAHSIIYALR.G	N	83.79	2315.1699	20	1.1	772.7314	3	32.66	7	1752	OB4035.raw	2.44E5	1	1	410	429	Deamidation (N
R.WLGLSAE(+28.03)YGNLYR.N	N	74.46	1568.7987	13	0.9	785.4073	2	34.89	7	1929	OB4035.raw	1.15E5	1	1	397	409	Ethylation
R.NALFVPHYN(+.98)TNAHSIIYALR.G	N	74.22	2314.1858	20	1.8	772.4039	3	31.94	7	1699	OB4035.raw	9.78E6	4	4	410	429	Deamidation (N
R.SPDIYNPQAGSLK.T	N	72.75	1388.6936	13	0.2	695.3542	2	27.39	7	1421	OB4035.raw	1.04E5	1	1	371	383	
K.TDSRPSIANLAGENSFIDNLPEEVVANSYGLPR.E	N	72.28	3544.7434	33	1.0	1182.5896	3	35.89	7	1992	OB4035.raw	3.21E5	1	1	479	511	
R.NALFVPHYNTNAHSIIYALR.G	N	72.27	2313.2019	20	0.8	772.0752	3	31.23	7	1675	OB4035.raw	5.74E6	2	2	410	429	
R.SPDIYNPQ(+.98)AGSLK.T	N	71.57	1389.6776	13	0.9	695.8467	2	28.07	7	1461	OB4035.raw	1.89E5	1	1	371	383	Deamidation (N
K.TDSRPSIANLAGENSFIDN(+.98)LPEEVVANSYGLPR.E	N	70.66	3545.7273	33	3.6	1182.9207	3	36.16	7	2010	OB4035.raw	8.95E5	1	1	479	511	Deamidation (N
K.TDSRPSIANLAGEN(+.98)SFIDN(+.98)LPEEVVANSYGLPR.E	N	68.32	3546.7114	33	5.5	1183.2510	3	38.04	7	2126	OB4035.raw	0	0	0	479	511	Deamidation (N
N.GIEETIC(+57.02)TASFK.K	N	67.74	1354.6438	12	1.5	678.3302	2	29.65	7	1562	OB4035.raw	4.41E4	1	1	352	363	Carbamidometh
R.SPDIYN(+.98)PQAGSLK.T	N	66.40	1389.6776	13	0.9	695.8467	2	28.07	7	1450	OB4035.raw	1.89E5	1	1	371	383	
K.SQSENFEYVAFKTD.S	N	66.17	1663.7366	14	0.2	832.8757	2	30.71	7	1635	OB4035.raw	1.38E5	1	1	467	480	
R.WLGLSAE(+14.02)YGNLYR.N	N	64.20	1554.7831	13	-0.4	778.3985	2	34.13	7	1867	OB4035.raw	0	0	0	397	409	Methylation(oth
K.TDSRPSIANLAGEN(+.98)SFIDNLPEEVVANSYGLPR.E	N	63.77	3545.7273	33	3.6	1182.9207	3	36.16	7	2114	OB4035.raw	8.95E5	1	1	479	511	
R.SPDIYN(+.98)PQ(+.98)AGSLK.T	N	63.44	1390.6616	13	0.4	696.3384	2	28.62	7	1501	OB4035.raw	3.67E4	1	1	371	383	Deamidation (N
N.GIEETIC(+71.04)TASFK.K	N	58.59	1368.6595	12	1.4	685.3380	2	29.81	7	1566	OB4035.raw	1.63E4	1	1	352	363	Propionamide
R.WLGLSAE(+53.92)YGNLYR.N	N	56.14	1594.6866	13	-0.3	532.5693	3	33.34	7	1819	OB4035.raw	5.46E5	1	1	397	409	Replacement of protons by iron
R.NRSPDIYNPQAGSLK.T	N	54.77	1658.8376	15	0.5	830.4265	2	25.62	7	1308	OB4035.raw	9.56E4	3	3	369	383	
K.TDSRPSIAN(+.98)LAGEN(+.98)SFIDN(+.98)LPEEVVANSYGLPR.E	N	54.30	3547.6953	33	5.4	1183.5787	3	36.54	7	2077	OB4035.raw	2.93E5	2	2	479	511	Deamidation (N
R.NRSPDIYNPQ(+.98)AGSLK.T	N	51.37	1659.8217	15	-0.8	830.9175	2	26.31	7	1347	OB4035.raw	1.5E5	2	2	369	383	Deamidation (N
K.TDSRPSIAN(+.98)LAGEN(+.98)SFIDNLPEEVVANSYGLPR.E	N	51.29	3546.7114	33	1.0	1183.2456	3	38.34	7	2145	OB4035.raw	0	0	0	479	511	Deamidation (N
K.SQSE(+28.03)NFEYVAFK.T	N	51.18	1475.6932	12	0.5	738.8542	2	32.29	7	1738	OB4035.raw	7.72E4	1	1	467	478	
K.TDSRPSIANLAGEN(+.98).S	N	49.56	1444.6793	14	0.0	723.3469	2	28.43	7	1486	OB4035.raw	2.32E4	1	1	479	492	Deamidation (N
K.FFVPPSEQ(+.98)SLR.A	N	49.38	1306.6556	11	0.7	654.3356	2	30.07	7	1584	OB4035.raw	1.99E5	1	1	525	535	Deamidation (N
R.NALFVP(+15.99)HYN(+.98)TNAHSIIYALR.G	N	49.25	2330.1807	20	0.0	777.7341	3	33.43	7	1816	OB4035.raw	1.08E5	1	1	410	429	Hydroxylation P Deamidation (N

Peptide	Uniq	-10lgP	Mass	Length	ppm	m/z	z	RT	Fraction	Scan	Source File	Area KOntrola 6. traka	#Feature	#Feature KOntrola 6. traka	Start	End	PTM
N.GIEETIC(+57.02)TASFKK.N	N	48.17	1482.7388	13	-0.2	742.3765	2	27.79	7	1441	OB4035.raw	1.6E4	1	1	352	364	Carbamidometh
K.TDSRPSIANLAGEN(+.98)SFIDN(+.98)LPEEVVAN(+.98)SYGLPR.E	N	46.57	3547.6953	33	5.4	1183.5787	3	36.54	7	2126	OB4035.raw	2.79E5	1	1	479	511	Deamidation (N
K.TDSRPSIANLAGEN(+.98)SFIDN(+.98)LPEEVVANSYGLPREQAR.Q	N	46.44	4030.9507	37	2.8	1008.7477	4	36.18	7	2011	OB4035.raw	0	0	0	479	515	
K.TDSRPSIAN(+.98)LAGEN(+.98).S	N	46.28	1445.6633	14	0.1	723.8390	2	29.02	7	1514	OB4035.raw	2.64E4	1	1	479	492	Deamidation (N
R.WLGLSAE(+14.02)YGN(+.98)LYR.N	N	45.38	1555.7671	13	-1.2	778.8899	2	34.58	7	1900	OB4035.raw	1.91E4	1	1	397	409	Methylation(oth<br>Deamidation (N
K.TDSRPSIANLAGENSFIDNLPEEVVANSYGLPREQ(+.98)AR.Q	N	45.37	4029.9668	37	1.1	1008.5001	4	34.97	7	1920	OB4035.raw	2.35E6	2	2	479	515	
K.SQ(+.98)SENFEYVAFKTD.S	N	45.20	1664.7206	14	-1.6	833.3662	2	31.06	7	1647	OB4035.raw	2.27E4	1	1	467	480	Deamidation (N
R.NALFVP(+15.99)HYNTNAHSIIYALR.G	N	44.60	2329.1968	20	0.1	777.4063	3	32.92	7	1784	OB4035.raw	1.3E5	2	2	410	429	Hydroxylation F
K.S(+43.01)(+14.02)QSENFEYVAFK.T	N	44.52	1504.6833	12	-0.1	753.3489	2	30.43	7	1603	OB4035.raw	7.06E4	1	1	467	478	Carbamylation;<br>Methylation(oth
K.TDSRPSIANLAGEN(+.98)SFIDNLPEEVVAN(+.98)SYGLPR.E	N	44.36	3546.7114	33	5.1	887.6897	4	36.99	7	2061	OB4035.raw	0	0	0	479	511	
K.TDSRPSIANLAGEN(+.98)SFIDN(+.98)LPEEVVANSYGLPREQ(+.98)AR.Q	N	44.03	4031.9348	37	2.7	1008.9937	4	35.43	7	1962	OB4035.raw	0	0	0	479	515	Deamidation (N
K.SQSENFE(+53.92)YVAFK.T	N	43.57	1501.5812	12	0.4	751.7982	2	30.26	7	1592	OB4035.raw	2.51E4	1	1	467	478	
R.NRSPDIYN(+.98)PQAGSLK.T	N	42.75	1659.8217	15	-0.4	554.2809	3	25.82	7	1316	OB4035.raw	1.71E5	2	2	369	383	
R.S(+41.03)P(+15.99)DIYNPQAGSLK.T	N	42.04	1445.7150	13	0.0	723.8647	2	27.55	7	1427	OB4035.raw	5.56E3	1	1	371	383	Amidination of<br>or N-terminal a<br>with methyl<br>acetimidate;<br>Hydroxylation F
R.WLGLSAE(+21.98)YGNLYR.N	N	41.38	1562.7494	13	-8.5	521.9193	3	33.34	7	1807	OB4035.raw	1.16E5	2	2	397	409	Sodium adduct
R.NALFVPHYNTN(+.98).A	N	41.37	1289.6040	11	0.3	645.8094	2	29.66	7	1549	OB4035.raw	3.19E4	1	1	410	420	
R.WLGLSAEYGNLY(-18.01)R.N	N	41.07	1522.7568	13	1.7	762.3870	2	39.61	7	2227	OB4035.raw	3.31E4	1	1	397	409	Dehydration
K.TDSRPSIANLAGENSFIDN(+.98)LPEEVVANSYGLPREQ(+.98)AR.Q	N	39.96	4030.9507	37	3.4	1008.7484	4	36.37	7	2023	OB4035.raw	0	0	0	479	515	
R.WLGLSAEY(+15.01)GNLYR.N	N	39.61	1555.7783	13	-8.4	778.8899	2	34.58	7	1894	OB4035.raw	1.91E4	1	1	397	409	Tyrosine oxidat<br>2-aminotyrosin
R.N(+.98)ALFVPHYNTNAHSIIYALR.G	N	39.24	2314.1858	20	2.1	579.5549	4	31.94	7	1776	OB4035.raw	4.84E6	1	1	410	429	
K.TDSRPSIANLAGEN(+.98)SFIDNLPEEVVAN(+.98)SYGLPREQ(+.98)AR.Q	N	38.91	4031.9348	37	3.1	1008.9941	4	34.19	7	1869	OB4035.raw	1.07E5	1	1	479	515	
K.FFVPPSEQ(+.98)SLRA.V	N	38.81	1377.6927	12	0.4	689.8539	2	30.52	7	1666	OB4035.raw	4.37E5	1	1	525	536	Deamidation (N
K.SQSENFEYVAFKTDSRPSIANLAG.E	N	37.87	2630.2612	24	0.0	877.7610	3	31.67	7	1694	OB4035.raw	4.54E4	1	1	467	490	
K.FFVPPSEQSLR.A	N	37.18	1305.6716	11	0.8	653.8436	2	29.65	7	1572	OB4035.raw	7.54E4	1	1	525	535	
K.TDSRPSIAN(+.98)LAGEN.S	N	36.99	1444.6793	14	0.0	723.3469	2	28.43	7	1499	OB4035.raw	2.32E4	1	1	479	492	Deamidation (N

Peptide	Uniq	-10lgP	Mass	Length	ppm	m/z	z	RT	Fraction	Scan	Source File	Area KOntrola 6. traka	#Feature	#Feature KOntrola 6. traka	Start	End	PTM
K.TDSRPSIANLAGENSFIDNLPEEVVANSYGLPREQAR.Q	N	36.48	4028.9827	37	0.8	1008.2538	4	34.60	7	1903	OB4035.raw	0	0	0	479	515	
R.NALFVP(+15.99)H(+42.01)YNTNAHSIIYALR.G	N	36.41	2371.2073	20	8.0	791.4160	3	31.23	7	1662	OB4035.raw	9.72E4	1	1	410	429	Hydroxylation P
K.TDSRPSIANLAGENSFIDNLPEEVVAN(+.98)SYGLPREQ(+.98)AR.Q	N	36.12	4030.9507	37	-0.5	1008.7444	4	37.54	7	2095	OB4035.raw	0	0	0	479	515	
K.SQSE(+21.98)NFEYVAFK.T	N	35.67	1469.6438	12	1.1	735.8300	2	30.26	7	1606	OB4035.raw	1.19E4	1	1	467	478	
R.WLGLSAEYGN(+.98)LY(+42.01)R(+14.02).N	N	35.60	1597.7776	13	7.0	799.9017	2	33.73	7	1791	OB4035.raw	6.17E5	1	1	397	409	Deamidation (N<br>Acetylation (TS<br>Methylation(KR
K.FFVPPSEQSLRA.V	N	35.57	1376.7087	12	0.8	689.3622	2	30.07	7	1600	OB4035.raw	1.45E5	1	1	525	536	
K.TDSRPSIANLAGEN(+.98)SFIDNLPEEVVANSYGLPREQ(+.98)AR.Q	N	35.47	4030.9507	37	2.5	1008.7475	4	35.07	7	1938	OB4035.raw	0	0	0	479	515	
K.TDSRPSIANLAGE.N	N	35.36	1329.6525	13	0.7	665.8340	2	28.33	7	1479	OB4035.raw	1.91E4	1	1	479	491	
R.WLGLSAEYGNLYRN(+.98)ALFVPHYN(+.98)TN(+.98)AHSIIYALR.G	N	35.11	3838.9106	33	1.1	768.7903	5	35.86	7	1990	OB4035.raw	0	0	0	397	429	
R.NALFVPHYN(+.98)TN(+.98).A	N	34.82	1290.5880	11	-0.2	646.3011	2	29.97	7	1580	OB4035.raw	5.15E3	1	1	410	420	Deamidation (N
R.W(+43.01)LGLS(+14.02)AEYGNLYR.N	N	34.45	1597.7888	13	0.0	799.9017	2	33.73	7	1849	OB4035.raw	6.17E5	1	1	397	409	Carbamylation;<br>Methylation(oth
K.TDSRPSIAN(+.98)LAG.E	N	34.14	1201.5939	12	1.0	601.8048	2	28.72	7	1485	OB4035.raw	4.76E4	1	1	479	490	Deamidation (N
R.N(+58.01)ALFVPHYNTNAHSIIYALR.G	N	34.07	2371.2073	20	-0.5	791.4093	3	32.37	7	1735	OB4035.raw	1.9E5	1	1	410	429	Carboxymethyl<br>X@N-term)
R.WLGLSAEYGN(+.98)LYR.N	N	34.02	1541.7513	13	6.2	771.8878	2	33.66	7	1830	OB4035.raw	0	0	0	397	409	Deamidation (N
R.Q(+.98)LKN(+.98)N(+.98)NPFKFFVPPSEQ(+.98)SLR.A	N	32.77	2393.1902	20	1.0	798.7381	3	33.09	7	1791	OB4035.raw	1.02E4	1	1	516	535	Deamidation (N
K.SQSEN(+.98)FEYVAFKTDSRPSIANLAGENSFIDNLPEEVVANSYGLPR.E	N	32.54	4975.3789	45	5.3	1244.8586	4	35.88	7	2005	OB4035.raw	2.16E5	1	1	467	511	
R.VFDEELQEGHVLVVPQNFAVAGK.S	N	32.49	2524.2961	23	0.8	842.4400	3	32.48	7	1750	OB4035.raw	2.13E4	1	1	444	466	
R.WLGLSAE(+53.92)YGN(+.98)LYR.N	N	32.10	1595.6707	13	2.2	532.8987	3	33.75	7	1837	OB4035.raw	0	0	0	397	409	Replacement of<br>protons by iron<br>Deamidation (N
R.NALFVPHYN(+.98)TNA.H	N	32.09	1360.6411	12	1.0	681.3285	2	29.71	7	1559	OB4035.raw	5.2E3	1	1	410	421	
R.N(+42.01)ALFVP(+15.99)HYNTNAHSIIYALR.G	N	31.91	2371.2073	20	-0.5	791.4093	3	32.37	7	1748	OB4035.raw	1.9E5	1	1	410	429	Acetylation (N-<br>Hydroxylation P
K.TDSRPSIANLA.G	N	31.18	1143.5884	11	0.3	572.8016	2	27.88	7	1465	OB4035.raw	1.36E4	1	1	479	489	
R.NALFVPHYNTNAHSIIYALRG.R	N	29.09	2370.2231	21	0.6	791.0821	3	31.77	7	1662	OB4035.raw	1.7E5	1	1	410	430	
K.TDSRPSIAN(+.98)LAGENSFIDN(+.98)LPEEVVANSYG.L	N	28.77	3180.4734	30	5.5	1061.1709	3	36.81	7	2050	OB4035.raw	0	0	0	479	508	
K.TDSRPSIANLAGEN(+.98)SFIDNLPEEVVANSYG.L	N	28.60	3179.4895	30	0.6	1060.8378	3	36.54	7	2050	OB4035.raw	1.11E4	1	1	479	508	
R.NALFVPHY.N	N	27.94	959.4865	8	-0.7	480.7502	2	30.43	7	1610	OB4035.raw	2.39E4	1	1	410	417	

Peptide	Uniq	-10lgP	Mass	Length	ppm	m/z	z	RT	Fraction	Scan	Source File	Area KOntrola 6. traka	#Feature	#Feature KOntrola 6. traka	Start	End	PTM
R.WLGLSAEYGNLYRN(+.98)ALFVPHYN(+.98)TNAHSIIYALR.G	N	27.92	3837.9268	33	-0.8	768.5920	5	35.14	7	1960	OB4035.raw	2.05E5	1	1	397	429	
R.W(+42.01)LGLSAE(+14.02)YGN(+.98)LYR.N	N	27.76	1597.7776	13	7.4	533.6038	3	33.73	7	1837	OB4035.raw	7.42E3	1	1	397	409	Acetylation (N-<br>Deamidation (N
R.NALFVPHYNTNA.H	N	27.57	1359.6571	12	0.0	680.8358	2	29.29	7	1532	OB4035.raw	7.88E3	1	1	410	421	
K.TDSRPSIAN(+.98)LA.G	N	27.12	1144.5724	11	-1.0	573.2929	2	28.82	7	1508	OB4035.raw	3.26E4	1	1	479	489	Deamidation (N
K.TDSRPSIANLAGENSFIDNLPEEVVAN(+.98)SYGLPR.E	N	26.98	3545.7273	33	0.4	887.4395	4	37.76	7	2109	OB4035.raw	0	0	0	479	511	
K.SQSEN(+.98)FEYVAFKTDSRPSIAN(+.98)LAGEN(+.98)SFIDN(+.98)LPEEVVANSYGLPR.E	N	26.96	4978.3306	45	9.2	1245.6013	4	36.39	7	2024	OB4035.raw	0	0	0	467	511	
R.NALFVPHYNTNAHSIIYALRGR.A	N	26.87	2526.3245	22	0.6	632.5887	4	30.52	7	1611	OB4035.raw	5.28E4	1	1	410	431	
K.TDSRPSIANLAGEN(+.98)SFIDN(+.98)LPEEVVAN(+.98)SYGLPREQAR.Q	N	26.02	4031.9348	37	7.4	1008.9984	4	38.73	7	2170	OB4035.raw	0	0	0	479	515	
R.WLGLSAEYGN(+.98)LYRNALFVPHYN(+.98)TN(+.98)AHSIIYALR.G	N	25.93	3838.9106	33	5.5	768.7936	5	35.67	7	1978	OB4035.raw	0	0	0	397	429	
K.TDSRPSIANLAGENSFIDN(+.98)LPEEVVAN(+.98)SYGLPREQ(+.98)AR.Q	N	25.85	4031.9348	37	1.5	1008.9925	4	38.90	7	2181	OB4035.raw	0	0	0	479	515	
N.ELQLNLLILR(+14.02)W(+31.99)LGLSAEYGNLYR.N	Y	25.47	2792.5225	23	-3.0	931.8453	3	42.19	7	2383	OB4035.raw	0	0	0	387	409	Methylation(KR<br>Dihydroxy
R.WLGLS(+58.03)AEYGNLYR.N	N	25.07	1598.7966	13	-3.9	800.4025	2	33.19	7	1791	OB4035.raw	4.21E3	1	1	397	409	2,3-dihydro-2,2<br>dimethyl-7-<br>benzofuranol N<br>methyl carbam
R.WLGLS(-18.01)AEYGNLYR.N	N	24.29	1522.7568	13	1.7	762.3870	2	39.61	7	2224	OB4035.raw	3.31E4	1	1	397	409	
R.NRSPDIYN(+.98)PQ(+.98)AGSLK.T	N	23.21	1660.8057	15	-0.7	554.6088	3	27.13	7	1410	OB4035.raw	1.72E4	1	1	369	383	Deamidation (N
K.TDSRPSIANLAG.E	N	23.17	1200.6099	12	0.1	601.3123	2	27.97	7	1447	OB4035.raw	2.3E4	1	1	479	490	
K.TDSRPSIAN(+.98)LAGEN(+.98)SFIDN(+.98)LPEEVVAN(+.98)SYGLPREQ(+.98)AR.Q	N	23.14	4033.9028	37	6.6	1009.4897	4	35.54	7	1962	OB4035.raw	9.79E5	1	1	479	515	Deamidation (N
R.SP(+15.99)DIYN(+.98)PQAGSLKTAN(+.98)ELQ(+.98)LNLLILR.W	Y	23.04	2899.5178	26	5.5	725.8907	4	33.01	7	1852	OB4035.raw	3.61E5	1	1	371	396	
K.SQ(+.98)SENFEYVAFKTDSRPSIANLAGENSFIDNLPEEVVANSYGLPR.E	N	22.63	4975.3789	45	5.3	1244.8586	4	35.88	7	1987	OB4035.raw	2.16E5	1	1	467	511	
R.QLKN(+.98)N(+.98)NPFKFFVPPSEQ(+.98).S	N	22.61	2035.9890	17	-1.5	679.6693	3	33.43	7	1822	OB4035.raw	5.05E4	1	1	516	532	Deamidation (N
R.W(+42.01)(+15.99)LGLSAEYGNLYR.N	N	22.56	1598.7728	13	-2.0	800.3921	2	34.11	7	1862	OB4035.raw	4.12E4	1	1	397	409	Acetylation (N-<br>Oxidation (HW)
K.TDSR(-43.05)PSIANLAGENSFIDNLPEEVVANSYGLPR.E	N	22.54	3501.6899	33	4.1	1168.2421	3	36.08	7	1986	OB4035.raw	4.89E4	1	1	479	511	Arginine oxidat<br>glutamic<br>semialdehyde
R.N(+.98)RSPDIYNPQAGSLK.T	N	22.33	1659.8217	15	-0.5	554.2809	3	26.23	7	1389	OB4035.raw	1.47E5	1	1	369	383	
**total 100 peptides**																	

tr|Q6IWG5|Q6IWG5_ARAHY

back to list

Protein Coverage:

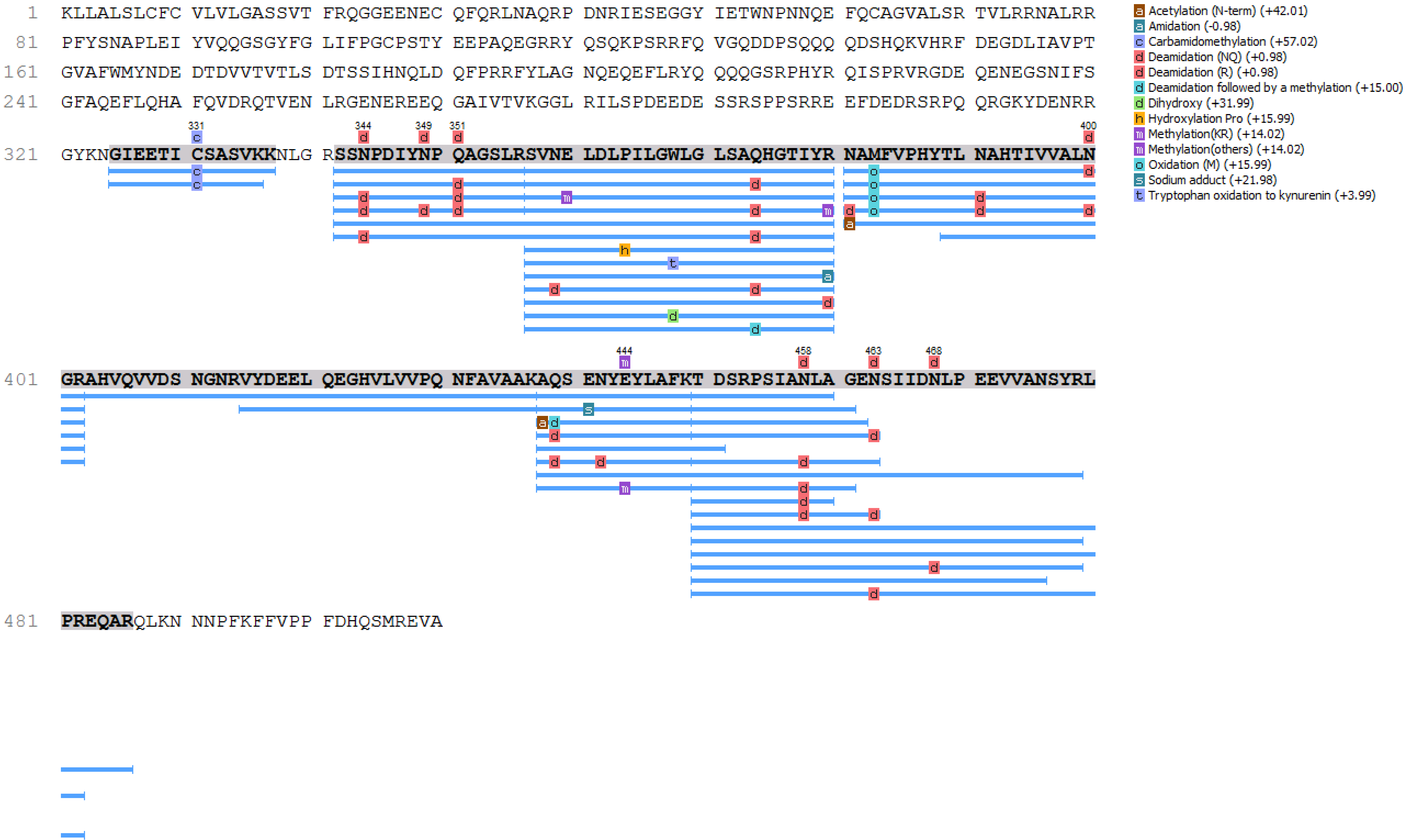
1 KLLALSLCFC VLVLGASSVT FRQGGEENEC QFQRLNAQRP DNRIESEGGY IETWNPNNQE FQCAGVALSR TVLRRNALRR
81 PFYSNAPLEI YVQQGSGYFG LIFPGCPSTY EEPAQEGRRY QSQKPSRRFQ VGQDDPSQQQ QDSHQKVHRF DEGDLIAVPT
161 GVAFWMYNDE DTDVVTVTLS DTSSIHNQLD QFPRRFYLAG NQEQEFLRYQ QQQGSRPHYR QISPRVRGDE QENEGSNIFS
241 GFAQEFLQHA FQVDRQTVEN LRGENEREEQ GAIVTVKGGL RILSPDEEDE SSRSPPSRRE EFDEDRSRPQ QRGKYDENRR
331 344 349 351 400
321 GYKNGIEETI CSASVKKNLG RSSNPDIYNP QAGSLRSVNE LDLPILGWLG LSAQHGTIYR NAMFVPHYTL NAHTIVVALN
444 458 463 468
401 GRAHVQVVDS NGNRVYDEEL QEGHVLVVPQ NFAVAAKAQS ENYEYLAFKT DSRPSIANLA GENSIIDNLP EEVVANSYRL
481 PREQARQLKN NNPFKFFVPP FDHQSMREVA
a Acetylation (N-term) (+42.01)
a Amidation (-0.98)
c Carbamidomethylation (+57.02)
d Deamidation (NQ) (+0.98)
d Deamidation (R) (+0.98)
d Deamidation followed by a methylation (+15.00)
d Dihydroxy (+31.99)
h Hydroxylation Pro (+15.99)
m Methylation(KR) (+14.02)
m Methylation(others) (+14.02)
o Oxidation (M) (+15.99)
s Sodium adduct (+21.98)
t Tryptophan oxidation to kynurenin (+3.99)

Supporting Peptides:

Peptide	Uniq	-10lgP	Mass	Length	ppm	m/z	z	RT	Fraction	Scan	Source File	Area KOntrola 6. traka	#Feature	#Feature KOntrola 6. traka	Start	End	PTM
K.AQSENYEYLAFK.T	Y	87.80	1461.6776	12	0.3	731.8463	2	30.26	7	1597	OB4035.raw	8.74E5	1	1	438	449	
K.TDSRPSIANLAGENSIIDNLPEEVVANSYR.L	Y	87.43	3243.6006	30	1.4	1082.2090	3	34.89	7	1916	OB4035.raw	3.79E5	1	1	450	479	
K.AQ(+.98)SENYEYLAFK.T	Y	85.76	1462.6616	12	-1.2	732.3372	2	30.52	7	1608	OB4035.raw	4.29E5	2	2	438	449	Deamidation (NQ)
K.TDSRPSIANLAGEN(+.98)SIIDNLPEEVVANSYR.L	Y	83.56	3244.5847	30	1.8	1082.5375	3	34.98	7	1932	OB4035.raw	0	0	0	450	479	
R.SVNELDLPILGWLGLSAQHGTIYR.N	Y	83.04	2651.4070	24	3.2	1326.7150	2	38.27	7	2146	OB4035.raw	6.12E6	9	9	357	380	
R.NAM(+15.99)FVPHYTLN(+.98)AHTIVVALNGR.A	Y	78.91	2454.2478	22	0.2	819.0900	3	32.29	7	1732	OB4035.raw	7.2E5	2	2	381	402	Oxidation (M); Deamidation (NQ)
R.SSNPDIYNPQAGSLR.S	Y	78.72	1617.7747	15	0.2	809.8948	2	27.39	7	1414	OB4035.raw	9.3E4	1	1	342	356	
R.SSNPDIYNPQ(+.98)AGSLR.S	Y	76.31	1618.7587	15	0.9	810.3873	2	27.97	7	1462	OB4035.raw	2.04E5	1	1	342	356	Deamidation (NQ)
K.AQSENYEYLAFKTD.S	Y	72.09	1677.7522	14	0.6	839.8839	2	30.61	7	1617	OB4035.raw	8.79E4	1	1	438	451	
R.NAM(+15.99)FVPHYTLNAHTIVVALNGR.A	Y	70.98	2453.2637	22	0.5	818.7623	3	31.67	7	1689	OB4035.raw	7.58E5	2	2	381	402	Oxidation (M)
R.NAM(+15.99)FVPHYTLNAHTIVVALN(+.98)GR.A	Y	70.79	2454.2478	22	2.9	819.0922	3	32.06	7	1718	OB4035.raw	6.42E5	3	3	381	402	Oxidation (M); Deamidation (NQ)
K.TDSRPSIAN(+.98)LAGENSIIDN(+.98)LPEEVVANSYR.L	Y	69.17	3245.5686	30	0.9	1082.8645	3	35.47	7	1965	OB4035.raw	0	0	0	450	479	Deamidation (NQ)
R.SSN(+.98)PDIYNPQ(+.98)AGSLR.S	Y	68.10	1619.7427	15	-0.7	810.8781	2	28.44	7	1477	OB4035.raw	7.94E4	1	1	342	356	Deamidation (NQ)
K.AQ(+.98)SEN(+.98)YEYLAFK.T	Y	65.17	1463.6456	12	0.7	732.8306	2	30.92	7	1640	OB4035.raw	2.91E4	1	1	438	449	Deamidation (NQ)
K.AQSENYE(+14.02)YLAFK.T	Y	62.97	1475.6932	12	0.1	738.8540	2	31.23	7	1665	OB4035.raw	6.54E4	1	1	438	449	Methylation(others)
N.GIEETIC(+57.02)SASVK.K	Y	62.95	1292.6282	12	0.9	647.3220	2	27.82	7	1445	OB4035.raw	0	0	0	325	336	Carbamidomethylatio
R.SVNELDLPILGWLGLSAQ(+.98)HGTIYR.N	Y	57.06	2652.3911	24	2.5	885.1399	3	38.57	7	2160	OB4035.raw	2.86E4	4	4	357	380	Deamidation (NQ)
R.SSNPDIYN(+.98)PQ(+.98)AGSLR.S	Y	53.78	1619.7427	15	-0.7	810.8781	2	28.44	7	1489	OB4035.raw	7.94E4	1	1	342	356	Deamidation (NQ)
R.SSNPDIYN(+.98)PQAGSLR.S	Y	51.72	1618.7587	15	0.9	810.3873	2	27.97	7	1570	OB4035.raw	2.04E5	1	1	342	356	
R.SSN(+.98)PDIYN(+.98)PQ(+.98)AGSLR.S	Y	51.49	1620.7267	15	-2.1	811.3689	2	28.82	7	1510	OB4035.raw	1.04E4	1	1	342	356	Deamidation (NQ)
K.AQSE(+21.98)NYEYLAFK.T	Y	51.31	1483.6595	12	-0.1	742.8370	2	30.26	7	1594	OB4035.raw	7.34E3	1	1	438	449	Sodium adduct
K.TDSRPSIANLAGEN(+.98).S	N	49.56	1444.6793	14	0.0	723.3469	2	28.43	7	1486	OB4035.raw	2.32E4	1	1	450	463	Deamidation (NQ)
R.VYDEELQEGHVLVVPQNFAVAAK.A	Y	49.06	2554.3066	23	0.8	852.4435	3	31.85	7	1704	OB4035.raw	1.12E5	1	1	415	437	
R.N(+.98)AM(+15.99)FVPHYTLN(+.98)AHTIVVALN(+.98)GR.A	Y	48.26	2456.2158	22	5.5	615.0646	4	33.01	7	1809	OB4035.raw	5.82E4	1	1	381	402	Deamidation (NQ); Oxidation (M)
R.AHVQVVDSNGN(+.98)RVYDEELQ(+.98)EGHVLVVPQNFAVAAK.A	Y	47.58	3832.9019	35	0.6	959.2333	4	31.16	7	1653	OB4035.raw	2.01E5	1	1	403	437	
R.VYDEELQEGHVLVVPQ(+.98)NFAVAAK.A	Y	46.99	2555.2908	23	-0.3	852.7706	3	32.20	7	1723	OB4035.raw	1.11E5	1	1	415	437	
K.TDSRPSIAN(+.98)LAGEN(+.98).S	N	46.28	1445.6633	14	0.1	723.8390	2	29.02	7	1514	OB4035.raw	2.64E4	1	1	450	463	Deamidation (NQ)

Peptide	Uniq	-10lgP	Mass	Length	ppm	m/z	z	RT	Fraction	Scan	Source File	Area KOntrola 6. traka	#Feature	#Feature KOntrola 6. traka	Start	End	PTM
N.GIEETIC(+57.02)SASVKK.N	Y	45.60	1420.7231	13	-0.1	711.3688	2	26.10	7	1337	OB4035.raw	6.78E3	1	1	325	337	Carbamidomethylation
K.A(+42.01)Q(+15.00)SENYEYLAFK.T	Y	44.11	1518.6877	12	7.6	760.3569	2	30.26	7	1591	OB4035.raw	4.55E4	1	1	438	449	Acetylation (N-term); Deamidation followed by a methylation
R.SVNELDLPILGWLGLSAQH(+15.99)GTIYR.N	Y	42.47	2667.4021	24	2.3	890.1433	3	39.33	7	2251	OB4035.raw	2.2E4	1	1	357	380	
K.TDSRPSIANLAGEN(+.98)SIIDNLPEEVVANSYRLPR.E	Y	38.66	3610.8225	33	1.5	903.7142	4	36.15	7	2008	OB4035.raw	0	0	0	450	482	
K.AQ(+.98)SENYEYLAFKTDSRPSIAN(+.98)LAGEN(+.98)SIIDNLPEEVVANSYR.L	Y	37.97	4690.2197	42	7.8	1173.5714	4	35.37	7	1958	OB4035.raw	0	0	0	438	479	
K.AQSENYEYLAFKTDSRPSIANLAGEN(+.98)SIIDNLPEEVVANSYR.L	Y	37.88	4688.2515	42	2.1	1173.0726	4	34.99	7	1933	OB4035.raw	0	0	0	438	479	
K.TDSRPSIANLAGEN(+.98)SIIDN(+.98)LPEEVVANSYRLPR.E	Y	37.32	3611.8066	33	4.2	903.9628	4	35.51	7	1968	OB4035.raw	0	0	0	450	482	Deamidation (NQ)
K.TDSRPSIANLAGENSIIDN(+.98)LPEEVVANSYRLPR.E	Y	37.13	3610.8225	33	0.7	903.7136	4	36.41	7	2025	OB4035.raw	0	0	0	450	482	
R.SVNELDLPILGW(+31.99)LGLSAQHGTIYR.N	Y	37.09	2683.3970	24	1.3	895.4741	3	36.44	7	2037	OB4035.raw	1.18E4	1	1	357	380	Dihydroxy
K.TDSRPSIAN(+.98)LAGEN.S	N	36.99	1444.6793	14	0.0	723.3469	2	28.43	7	1499	OB4035.raw	2.32E4	1	1	450	463	Deamidation (NQ)
K.TDSRPSIANLAGE.N	N	35.36	1329.6525	13	0.7	665.8340	2	28.33	7	1479	OB4035.raw	1.91E4	1	1	450	462	
K.AQ(+.98)SENYEYLAFKTDSRPSIAN(+.98)LAGENSIIDNLPEEVVANSYR.L	Y	34.85	4689.2358	42	5.8	1173.3230	4	35.31	7	1954	OB4035.raw	0	0	0	438	479	
R.SVNE(+14.02)LDLPILGWLGLSAQHGTIYR.N	Y	34.54	2665.4229	24	-1.7	889.4800	3	38.70	7	2174	OB4035.raw	3.13E4	1	1	357	380	Methylation(others)
K.AQSENYEYLAFKTDSRPSIANLAGENSIIDNLPEEVVANSYR.L	Y	34.45	4687.2676	42	-0.4	1172.8237	4	34.83	7	1958	OB4035.raw	2.96E5	1	1	438	479	
K.TDSRPSIAN(+.98)LAG.E	N	34.14	1201.5939	12	1.0	601.8048	2	28.72	7	1485	OB4035.raw	4.76E4	1	1	450	461	Deamidation (NQ)
K.TDSRPSIANLAGENSIIDNLPEEVVANSYRLPR.E	Y	32.84	3609.8386	33	-0.4	903.4666	4	34.97	7	1923	OB4035.raw	7.53E5	1	1	450	482	
R.SVNELDLP(+15.99)ILGWLGLSAQHGTIYR.N	Y	31.79	2667.4021	24	2.5	890.1435	3	40.21	7	2269	OB4035.raw	3.7E4	2	2	357	380	Hydroxylation Pro
K.TDSRPSIANLA.G	N	31.18	1143.5884	11	0.3	572.8016	2	27.88	7	1465	OB4035.raw	1.36E4	1	1	450	460	
K.AQSE(+53.92)NYEYLAFK.T	Y	30.07	1515.5968	12	0.5	758.8060	2	30.27	7	1588	OB4035.raw	1.29E4	1	1	438	449	
K.TDSRPSIANLAGENSIIDNLPEEVVANSYRLPREQ(+.98)AR.Q	Y	29.63	4095.0620	37	0.0	820.0197	5	34.04	7	1868	OB4035.raw	4.09E5	1	1	450	486	
R.SVN(+.98)ELDLPILGWLGLSAQ(+.98)HGTIYR.N	Y	29.39	2653.3750	24	2.4	885.4677	3	51.28	7	2957	OB4035.raw	0	0	0	357	380	Deamidation (NQ)
K.TDSRPSIANLAGEN(+.98)SIIDN(+.98)LPEEVVAN.S	N	29.30	2839.3723	27	-0.2	947.4645	3	35.50	7	1957	OB4035.raw	3.01E4	1	1	450	476	
K.TDSRPSIAN(+.98)LAGENSIIDN(+.98)LPEEVVANSYRLPR.E	Y	29.28	3611.8066	33	1.5	903.9603	4	37.10	7	2068	OB4035.raw	0	0	0	450	482	
R.SVNELDLPILGW(+3.99)LGLSAQHGTIYR.N	Y	29.24	2655.4021	24	-0.1	886.1412	3	39.71	7	2262	OB4035.raw	2.52E5	2	2	357	380	Tryptophan oxidation to kynurenin
R.SSNPDIYNPQAGSLRSVN(+.98)ELDLPILGWLGLSAQ(+.98)HGTIYR.N	Y	29.20	4253.1392	39	2.8	1064.2950	4	37.70	7	2103	OB4035.raw	1.66E5	1	1	342	380	
K.TDSRPSIANLAGENSIIDN(+.98)LPEEVVANSYRLPREQ(+.98)AR.Q	Y	28.74	4096.0459	37	1.6	820.2178	5	34.14	7	1868	OB4035.raw	0	0	0	450	486	
R.SVNELDLPILGWLGLSAQ(+15.00)HGTIYR.N	Y	28.73	2666.4067	24	0.5	889.8100	3	40.33	7	2269	OB4035.raw	0	0	0	357	380	Deamidation followed by a methylation

Peptide	Uniq	-10lgP	Mass	Length	ppm	m/z	z	RT	Fraction	Scan	Source File	Area KOntrola 6. traka	#Feature	#Feature KOntrola 6. traka	Start	End	PTM
Y.TLNAHTIVVALNGR.A	Y	27.51	1477.8365	14	0.0	493.6194	3	29.97	7	1582	OB4035.raw	3.15E3	1	1	389	402	
R.SSN(+.98)PDIYN(+.98)PQ(+.98)AGSLRSVNELDLPILGWLGLSAQHGTIYR.N	Y	27.17	4254.1230	39	0.3	1064.5383	4	38.12	7	2131	OB4035.raw	0	0	0	342	380	
K.TDSRPSIAN(+.98)LA.G	N	27.12	1144.5724	11	-1.0	573.2929	2	28.82	7	1508	OB4035.raw	3.26E4	1	1	450	460	Deamidation (NQ)
R.SVNELDLPILGWLGLSAQ(+.98)HGTIYR(+14.02).N	Y	25.55	2666.4067	24	5.6	889.8145	3	38.98	7	2186	OB4035.raw	0	0	0	357	380	Deamidation (NQ); Methylation(KR)
K.TDSRPSIANLAGEN(+.98)SIIDNLPEEVVAN(+.98)SYRLPR.E	Y	24.86	3611.8066	33	7.5	903.9657	4	37.91	7	2118	OB4035.raw	0	0	0	450	482	
R.SVNELDLPILGWLGLSAQHGTIYR(+.98).N	Y	24.62	2652.3911	24	2.6	885.1400	3	46.87	7	2681	OB4035.raw	0	0	0	357	380	Deamidation (R)
R.SVNELDLPILGWLGLSAQ(+.98)HGTIYR(-.98).N	Y	23.42	2651.4070	24	0.2	884.8098	3	45.16	7	2571	OB4035.raw	0	0	0	357	380	Amidation
K.TDSRPSIANLAG.E	N	23.17	1200.6099	12	0.1	601.3123	2	27.97	7	1447	OB4035.raw	2.3E4	1	1	450	461	
R.SSN(+.98)PDIYN(+.98)PQ(+.98)AGSLRSVNELDLPILGWLGLSAQ(+.98)HGTIYR.N	Y	22.87	4255.1074	39	-1.6	1064.7825	4	38.49	7	2155	OB4035.raw	0	0	0	342	380	Deamidation (NQ)
K.TDSRPSIANLAGEN(+.98)SIIDN(+.98)LPEEVVAN(+.98)SYRLPR.E	Y	22.74	3612.7908	33	9.3	904.2134	4	37.40	7	2086	OB4035.raw	0	0	0	450	482	
R.N(+.98)AM(+15.99)FVPHYTLNAHTIVVALN(+.98)GR.A	Y	22.58	2455.2317	22	7.5	819.4240	3	33.34	7	1850	OB4035.raw	1.75E4	1	1	381	402	Oxidation (M); Deamidation (NQ)
R.N(+42.01)AMFVPHYTLNAHT(-18.01)IVVALN(+.98)GR.A	Y	22.58	2462.2529	22	2.9	616.5723	4	32.43	7	1742	OB4035.raw	0	0	0	381	402	Acetylation (N-term)
R.SSN(+.98)PDIYN(+.98)PQ(+.98)AGSLRSVN(+.98)ELDLPILGWLGLSAQHGTIYR.N	Y	22.03	4255.1074	39	1.4	1064.7856	4	38.28	7	2142	OB4035.raw	0	0	0	342	380	
**total 67 peptides**																	

tr|Q0GM57|Q0GM57_ARAHY
back to list

| Protein Coverage | Supporting Peptides |

Protein Coverage:

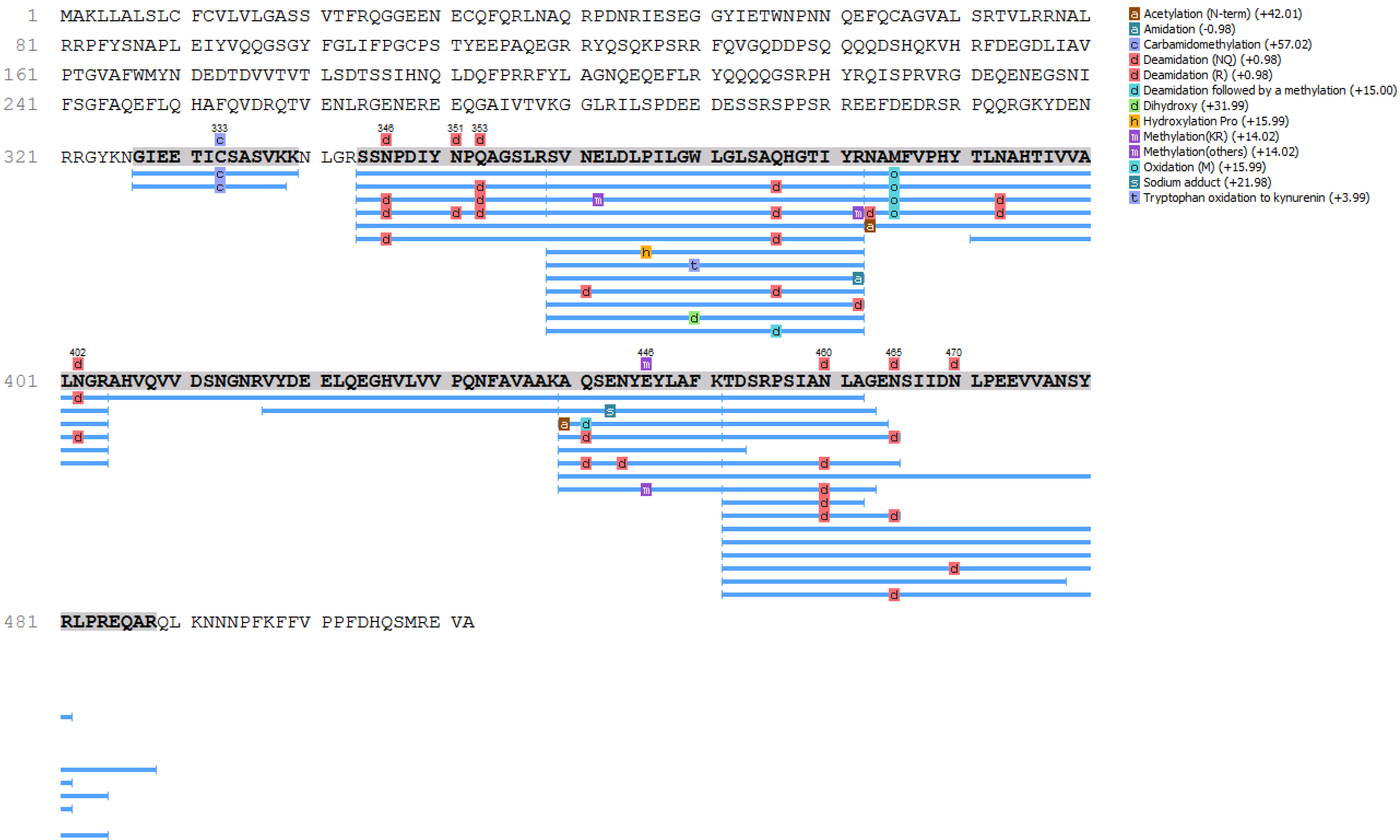
1 MAKLLALSLC FCVLVLGASS VTFRQGGEEN ECQFQRLNAQ RPDNRIESEG GYIETWNPNN QEFQCAGVAL SRTVLRRNAL
81 RRPFYSNAPL EIYVQQGSGY FGLIFPGCPS TYEEPAQEGR RYQSQKPSRR FQVGQDDPSQ QQQDSHQKVH RFDEGDLIAV
161 PTGVAFWMYN DEDTDVVTVT LSDTSSIHNQ LDQFPRRFYL AGNQEQEFLR YQQQQGSRPH YRQISPRVRG DEQENEGSNI
241 FSGFAQEFLQ HAFQVDRQTV ENLRGENERE EQGAIVTVKG GLRILSPDEE DESSRSPPSR REEFDEDRSR PQQRGKYDEN
321 RRGYKNGIEE TICSASVKKN LGRSSNPDIY NPQAGSLRSV NELDLPILGW LGLSAQHGTI YRNAMFVPHY TLNAHTIVVA
401 LNGRAHVQVV DSNGNRVYDE ELQEGHVLVV PQNFAVAAKA QSENYEYLAF KTDSRPSIAN LAGENSIIDN LPEEVVANSY
481 RLPREQARQL KNNNPFKFFV PPFDHQSMRE VA
333
346
351
353
402
446
460
465
470
Acetylation (N-term) (+42.01)
Amidation (-0.98)
Carbamidomethylation (+57.02)
Deamidation (NQ) (+0.98)
Deamidation (R) (+0.98)
Deamidation followed by a methylation (+15.00)
Dihydroxy (+31.99)
Hydroxylation Pro (+15.99)
Methylation(KR) (+14.02)
Methylation(others) (+14.02)
Oxidation (M) (+15.99)
Sodium adduct (+21.98)
Tryptophan oxidation to kynurenin (+3.99)

Supporting Peptides:

Peptide	Uniq	-10lgP	Mass	Length	ppm	m/z	z	RT	Fraction	Scan	Source File	Area KOntrola 6. traka	#Feature	#Feature KOntrola 6. traka	Start	End	PTM
K.AQSENYEYLAFK.T	Y	87.80	1461.6776	12	0.3	731.8463	2	30.26	7	1597	OB4035.raw	8.74E5	1	1	440	451	
K.TDSRPSIANLAGENSIIDNLPEEVVANSYR.L	Y	87.43	3243.6006	30	1.4	1082.2090	3	34.89	7	1916	OB4035.raw	3.79E5	1	1	452	481	
K.AQ(+.98)SENYEYLAFK.T	Y	85.76	1462.6616	12	-1.2	732.3372	2	30.52	7	1608	OB4035.raw	4.29E5	2	2	440	451	Deamidation (NQ)
K.TDSRPSIANLAGEN(+.98)SIIDNLPEEVVANSYR.L	Y	83.56	3244.5847	30	1.8	1082.5375	3	34.98	7	1932	OB4035.raw	0	0	0	452	481	
R.SVNELDLPILGWLGLSAQHGTIYR.N	Y	83.04	2651.4070	24	3.2	1326.7150	2	38.27	7	2146	OB4035.raw	6.12E6	9	9	359	382	
R.NAM(+15.99)FVPHYTLN(+.98)AHTIVVALNGR.A	Y	78.91	2454.2478	22	0.2	819.0900	3	32.29	7	1732	OB4035.raw	7.2E5	2	2	383	404	Oxidation (M); Deamidation (NQ)
R.SSNPDIYNPQAGSLR.S	Y	78.72	1617.7747	15	0.2	809.8948	2	27.39	7	1414	OB4035.raw	9.3E4	1	1	344	358	
R.SSNPDIYNPQ(+.98)AGSLR.S	Y	76.31	1618.7587	15	0.9	810.3873	2	27.97	7	1462	OB4035.raw	2.04E5	1	1	344	358	Deamidation (NQ)
K.AQSENYEYLAFKTD.S	Y	72.09	1677.7522	14	0.6	839.8839	2	30.61	7	1617	OB4035.raw	8.79E4	1	1	440	453	
R.NAM(+15.99)FVPHYTLNAHTIVVALNGR.A	Y	70.98	2453.2637	22	0.5	818.7623	3	31.67	7	1689	OB4035.raw	7.58E5	2	2	383	404	Oxidation (M)
R.NAM(+15.99)FVPHYTLNAHTIVVALN(+.98)GR.A	Y	70.79	2454.2478	22	2.9	819.0922	3	32.06	7	1718	OB4035.raw	6.42E5	3	3	383	404	Oxidation (M); Deamidation (NQ)
K.TDSRPSIAN(+.98)LAGENSIIDN(+.98)LPEEVVANSYR.L	Y	69.17	3245.5686	30	0.9	1082.8645	3	35.47	7	1965	OB4035.raw	0	0	0	452	481	Deamidation (NQ)
R.SSN(+.98)PDIYNPQ(+.98)AGSLR.S	Y	68.10	1619.7427	15	-0.7	810.8781	2	28.44	7	1477	OB4035.raw	7.94E4	1	1	344	358	Deamidation (NQ)
K.AQ(+.98)SEN(+.98)YEYLAFK.T	Y	65.17	1463.6456	12	0.7	732.8306	2	30.92	7	1640	OB4035.raw	2.91E4	1	1	440	451	Deamidation (NQ)
K.AQSENYE(+14.02)YLAFK.T	Y	62.97	1475.6932	12	0.1	738.8540	2	31.23	7	1665	OB4035.raw	6.54E4	1	1	440	451	Methylation(others)
N.GIEETIC(+57.02)SASVK.K	Y	62.95	1292.6282	12	0.9	647.3220	2	27.82	7	1445	OB4035.raw	0	0	0	327	338	Carbamidomethylatior
R.SVNELDLPILGWLGLSAQ(+.98)HGTIYR.N	Y	57.06	2652.3911	24	2.5	885.1399	3	38.57	7	2160	OB4035.raw	2.86E4	4	4	359	382	Deamidation (NQ)
R.SSNPDIYN(+.98)PQ(+.98)AGSLR.S	Y	53.78	1619.7427	15	-0.7	810.8781	2	28.44	7	1489	OB4035.raw	7.94E4	1	1	344	358	Deamidation (NQ)
R.SSNPDIYN(+.98)PQAGSLR.S	Y	51.72	1618.7587	15	0.9	810.3873	2	27.97	7	1570	OB4035.raw	2.04E5	1	1	344	358	
R.SSN(+.98)PDIYN(+.98)PQ(+.98)AGSLR.S	Y	51.49	1620.7267	15	-2.1	811.3689	2	28.82	7	1510	OB4035.raw	1.04E4	1	1	344	358	Deamidation (NQ)
K.AQSE(+21.98)NYEYLAFK.T	Y	51.31	1483.6595	12	-0.1	742.8370	2	30.26	7	1594	OB4035.raw	7.34E3	1	1	440	451	Sodium adduct
K.TDSRPSIANLAGEN(+.98).S	N	49.56	1444.6793	14	0.0	723.3469	2	28.43	7	1486	OB4035.raw	2.32E4	1	1	452	465	Deamidation (NQ)
R.VYDEELQEGHVLVVPQNFAVAAK.A	Y	49.06	2554.3066	23	0.8	852.4435	3	31.85	7	1704	OB4035.raw	1.12E5	1	1	417	439	
R.N(+.98)AM(+15.99)FVPHYTLN(+.98)AHTIVVALN(+.98)GR.A	Y	48.26	2456.2158	22	5.5	615.0646	4	33.01	7	1809	OB4035.raw	5.82E4	1	1	383	404	Deamidation (NQ); Oxidation (M)
R.AHVQVVDSNGN(+.98)RVYDEELQ(+.98)EGHVLVVPQNFAVAAK.A	Y	47.58	3832.9019	35	0.6	959.2333	4	31.16	7	1653	OB4035.raw	2.01E5	1	1	405	439	
R.VYDEELQEGHVLVVPQ(+.98)NFAVAAK.A	Y	46.99	2555.2908	23	-0.3	852.7706	3	32.20	7	1723	OB4035.raw	1.11E5	1	1	417	439	
K.TDSRPSIAN(+.98)LAGEN(+.98).S	N	46.28	1445.6633	14	0.1	723.8390	2	29.02	7	1514	OB4035.raw	2.64E4	1	1	452	465	Deamidation (NQ)

Peptide	Uniq	-10lgP	Mass	Length	ppm	m/z	z	RT	Fraction	Scan	Source File	Area KOntrola 6. traka	#Feature	#Feature KOntrola 6. traka	Start	End	PTM
N.GIEETIC(+57.02)SASVKK.N	Y	45.60	1420.7231	13	-0.1	711.3688	2	26.10	7	1337	OB4035.raw	6.78E3	1	1	327	339	Carbamidomethylation
K.A(+42.01)Q(+15.00)SENYEYLAFK.T	Y	44.11	1518.6877	12	7.6	760.3569	2	30.26	7	1591	OB4035.raw	4.55E4	1	1	440	451	Acetylation (N-term); Deamidation followed by a methylation
R.SVNELDLPILGWLGLSAQH(+15.99)GTIYR.N	Y	42.47	2667.4021	24	2.3	890.1433	3	39.33	7	2251	OB4035.raw	2.2E4	1	1	359	382	
K.TDSRPSIANLAGEN(+.98)SIIDNLPEEVVANSYRLPR.E	Y	38.66	3610.8225	33	1.5	903.7142	4	36.15	7	2008	OB4035.raw	0	0	0	452	484	
K.AQ(+.98)SENYEYLAFKTDSRPSIAN(+.98)LAGEN(+.98)SIIDNLPEEVVANSYR.L	Y	37.97	4690.2197	42	7.8	1173.5714	4	35.37	7	1958	OB4035.raw	0	0	0	440	481	
K.AQSENYEYLAFKTDSRPSIANLAGEN(+.98)SIIDNLPEEVVANSYR.L	Y	37.88	4688.2515	42	2.1	1173.0726	4	34.99	7	1933	OB4035.raw	0	0	0	440	481	
K.TDSRPSIANLAGEN(+.98)SIIDN(+.98)LPEEVVANSYRLPR.E	Y	37.32	3611.8066	33	4.2	903.9628	4	35.51	7	1968	OB4035.raw	0	0	0	452	484	Deamidation (NQ)
K.TDSRPSIANLAGENSIIDN(+.98)LPEEVVANSYRLPR.E	Y	37.13	3610.8225	33	0.7	903.7136	4	36.41	7	2025	OB4035.raw	0	0	0	452	484	
R.SVNELDLPILGW(+31.99)LGLSAQHGTIYR.N	Y	37.09	2683.3970	24	1.3	895.4741	3	36.44	7	2037	OB4035.raw	1.18E4	1	1	359	382	Dihydroxy
K.TDSRPSIAN(+.98)LAGEN.S	N	36.99	1444.6793	14	0.0	723.3469	2	28.43	7	1499	OB4035.raw	2.32E4	1	1	452	465	Deamidation (NQ)
K.TDSRPSIANLAGE.N	N	35.36	1329.6525	13	0.7	665.8340	2	28.33	7	1479	OB4035.raw	1.91E4	1	1	452	464	
K.AQ(+.98)SENYEYLAFKTDSRPSIAN(+.98)LAGENSIIDNLPEEVVANSYR.L	Y	34.85	4689.2358	42	5.8	1173.3230	4	35.31	7	1954	OB4035.raw	0	0	0	440	481	
R.SVNE(+14.02)LDLPILGWLGLSAQHGTIYR.N	Y	34.54	2665.4229	24	-1.7	889.4800	3	38.70	7	2174	OB4035.raw	3.13E4	1	1	359	382	Methylation(others)
K.AQSENYEYLAFKTDSRPSIANLAGENSIIDNLPEEVVANSYR.L	Y	34.45	4687.2676	42	-0.4	1172.8237	4	34.83	7	1958	OB4035.raw	2.96E5	1	1	440	481	
K.TDSRPSIAN(+.98)LAG.E	N	34.14	1201.5939	12	1.0	601.8048	2	28.72	7	1485	OB4035.raw	4.76E4	1	1	452	463	Deamidation (NQ)
K.TDSRPSIANLAGENSIIDNLPEEVVANSYRLPR.E	Y	32.84	3609.8386	33	-0.4	903.4666	4	34.97	7	1923	OB4035.raw	7.53E5	1	1	452	484	
R.SVNELDLP(+15.99)ILGWLGLSAQHGTIYR.N	Y	31.79	2667.4021	24	2.5	890.1435	3	40.21	7	2269	OB4035.raw	3.7E4	2	2	359	382	Hydroxylation Pro
K.TDSRPSIANLA.G	N	31.18	1143.5884	11	0.3	572.8016	2	27.88	7	1465	OB4035.raw	1.36E4	1	1	452	462	
K.AQSE(+53.92)NYEYLAFK.T	Y	30.07	1515.5968	12	0.5	758.8060	2	30.27	7	1588	OB4035.raw	1.29E4	1	1	440	451	
K.TDSRPSIANLAGENSIIDNLPEEVVANSYRLPREQ(+.98)AR.Q	Y	29.63	4095.0620	37	0.0	820.0197	5	34.04	7	1868	OB4035.raw	4.09E5	1	1	452	488	
R.SVN(+.98)ELDLPILGWLGLSAQ(+.98)HGTIYR.N	Y	29.39	2653.3750	24	2.4	885.4677	3	51.28	7	2957	OB4035.raw	0	0	0	359	382	Deamidation (NQ)
K.TDSRPSIANLAGEN(+.98)SIIDN(+.98)LPEEVVAN.S	N	29.30	2839.3723	27	-0.2	947.4645	3	35.50	7	1957	OB4035.raw	3.01E4	1	1	452	478	
K.TDSRPSIAN(+.98)LAGENSIIDN(+.98)LPEEVVANSYRLPR.E	Y	29.28	3611.8066	33	1.5	903.9603	4	37.10	7	2068	OB4035.raw	0	0	0	452	484	
R.SVNELDLPILGW(+3.99)LGLSAQHGTIYR.N	Y	29.24	2655.4021	24	-0.1	886.1412	3	39.71	7	2262	OB4035.raw	2.52E5	2	2	359	382	Tryptophan oxidation to kynurenin
R.SSNPDIYNPQAGSLRSVN(+.98)ELDLPILGWLGLSAQ(+.98)HGTIYR.N	Y	29.20	4253.1392	39	2.8	1064.2950	4	37.70	7	2103	OB4035.raw	1.66E5	1	1	344	382	
K.TDSRPSIANLAGENSIIDN(+.98)LPEEVVANSYRLPREQ(+.98)AR.Q	Y	28.74	4096.0459	37	1.6	820.2178	5	34.14	7	1868	OB4035.raw	0	0	0	452	488	
R.SVNELDLPILGWLGLSAQ(+15.00)HGTIYR.N	Y	28.73	2666.4067	24	0.5	889.8100	3	40.33	7	2269	OB4035.raw	0	0	0	359	382	Deamidation followed by a methylation

Peptide	Uniq	-10lgP	Mass	Length	ppm	m/z	z	RT	Fraction	Scan	Source File	Area KOntrola 6. traka	#Feature	#Feature KOntrola 6. traka	Start	End	PTM
Y.TLNAHTIVVALNGR.A	Y	27.51	1477.8365	14	0.0	493.6194	3	29.97	7	1582	OB4035.raw	3.15E3	1	1	391	404	
R.SSN(+.98)PDIYN(+.98)PQ(+.98)AGSLRSVNELDLPILGWLGLSAQHGTIYR.N	Y	27.17	4254.1230	39	0.3	1064.5383	4	38.12	7	2131	OB4035.raw	0	0	0	344	382	
K.TDSRPSIAN(+.98)LA.G	N	27.12	1144.5724	11	-1.0	573.2929	2	28.82	7	1508	OB4035.raw	3.26E4	1	1	452	462	Deamidation (NQ)
R.SVNELDLPILGWLGLSAQ(+.98)HGTIYR(+14.02).N	Y	25.55	2666.4067	24	5.6	889.8145	3	38.98	7	2186	OB4035.raw	0	0	0	359	382	Deamidation (NQ); Methylation(KR)
K.TDSRPSIANLAGEN(+.98)SIIDNLPEEVVAN(+.98)SYRLPR.E	Y	24.86	3611.8066	33	7.5	903.9657	4	37.91	7	2118	OB4035.raw	0	0	0	452	484	
R.SVNELDLPILGWLGLSAQHGTIYR(+.98).N	Y	24.62	2652.3911	24	2.6	885.1400	3	46.87	7	2681	OB4035.raw	0	0	0	359	382	Deamidation (R)
R.SVNELDLPILGWLGLSAQ(+.98)HGTIYR(-.98).N	Y	23.42	2651.4070	24	0.2	884.8098	3	45.16	7	2571	OB4035.raw	0	0	0	359	382	Amidation
K.TDSRPSIANLAG.E	N	23.17	1200.6099	12	0.1	601.3123	2	27.97	7	1447	OB4035.raw	2.3E4	1	1	452	463	
R.SSN(+.98)PDIYN(+.98)PQ(+.98)AGSLRSVNELDLPILGWLGLSAQ(+.98)HGTIYR.N	Y	22.87	4255.1074	39	-1.6	1064.7825	4	38.49	7	2155	OB4035.raw	0	0	0	344	382	Deamidation (NQ)
K.TDSRPSIANLAGEN(+.98)SIIDN(+.98)LPEEVVAN(+.98)SYRLPR.E	Y	22.74	3612.7908	33	9.3	904.2134	4	37.40	7	2086	OB4035.raw	0	0	0	452	484	
R.N(+.98)AM(+15.99)FVPHYTLNAHTIVVALN(+.98)GR.A	Y	22.58	2455.2317	22	7.5	819.4240	3	33.34	7	1850	OB4035.raw	1.75E4	1	1	383	404	Oxidation (M); Deamidation (NQ)
R.N(+42.01)AMFVPHYTLNAHT(-18.01)IVVALN(+.98)GR.A	Y	22.58	2462.2529	22	2.9	616.5723	4	32.43	7	1742	OB4035.raw	0	0	0	383	404	Acetylation (N-term)
R.SSN(+.98)PDIYN(+.98)PQ(+.98)AGSLRSVN(+.98)ELDLPILGWLGLSAQHGTIYR.N	Y	22.03	4255.1074	39	1.4	1064.7856	4	38.28	7	2142	OB4035.raw	0	0	0	344	382	
**total 67 peptides**																	

tr|O82580|O82580_ARAHY

back to list

| Protein Coverage | Supporting Peptides |

Protein Coverage:

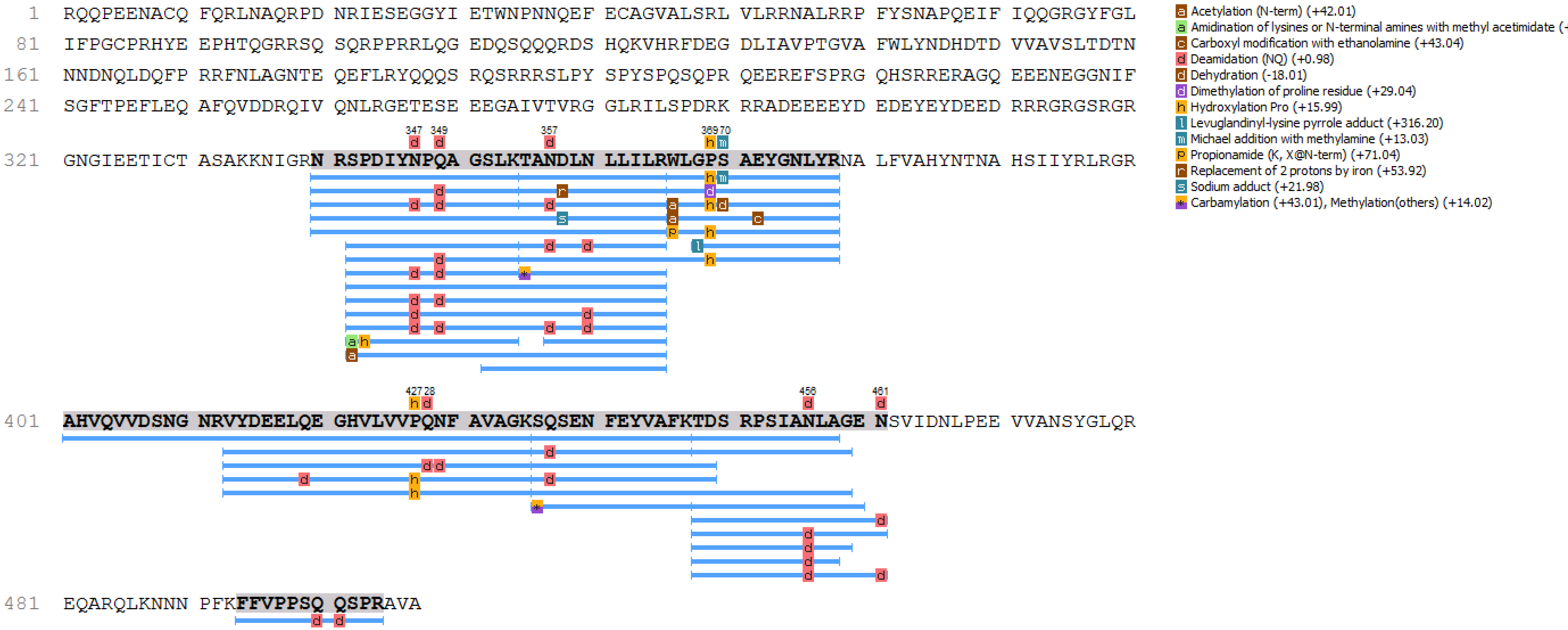

Supporting Peptides:

Peptide	Uniq	-10lgP	Mass	Length	ppm	m/z	z	RT	Fraction	Scan	Source File	Area KOntrola 6. traka	#Feature	#Feature KOntrola 6. traka	Start	End	PTM
K.SQSENFEYVAFK.T	N	92.19	1447.6619	12	1.3	724.8392	2	30.26	7	1587	OB4035.raw	1.74E6	1	1	436	447	
R.SPDIYNPQAGSLKTANDLNLLILR.W	N	86.57	2625.4126	24	0.8	876.1455	3	33.65	7	1826	OB4035.raw	2.37E5	1	1	342	365	

Peptide	Uniq	-10lgP	Mass	Length	ppm	m/z	z	RT	Fraction	Scan	Source File	Area KOntrola 6. traka	#Feature	#Feature KOntrola 6. traka	Start	End	PTM
K.SQ(+.98)SENFEYVAFK.T	N	84.36	1448.6459	12	0.1	725.3303	2	30.61	7	1614	OB4035.raw	4.98E5	2	2	436	447	Deamidation (NQ)
R.SPDIYNPQ(+.98)AGSLKTANDLNLLILR.W	N	83.88	2626.3965	24	0.8	876.4735	3	34.04	7	1854	OB4035.raw	1.03E6	1	1	342	365	
R.SPDIYNPQAGSLK.T	N	72.75	1388.6936	13	0.2	695.3542	2	27.39	7	1421	OB4035.raw	1.04E5	1	1	342	354	
R.SPDIYNPQ(+.98)AGSLK.T	N	71.57	1389.6776	13	0.9	695.8467	2	28.07	7	1461	OB4035.raw	1.89E5	1	1	342	354	Deamidation (NQ)
R.SPDIYN(+.98)PQAGSLKTANDLN(+.98)LLILR.W	N	70.53	2627.3806	24	5.1	876.8053	3	34.72	7	1912	OB4035.raw	0	0	0	342	365	Deamidation (NQ)
R.SPDIYN(+.98)PQ(+.98)AGSLKTANDLNLLILR.W	N	68.73	2627.3806	24	1.7	876.8023	3	34.36	7	1884	OB4035.raw	0	0	0	342	365	Deamidation (NQ)
R.VYDEELQEGHVLVVPQN(+.98)FAVAGK.S	N	68.59	2541.2751	23	0.7	848.0996	3	32.01	7	1729	OB4035.raw	2.05E5	1	1	413	435	
R.SPDIYN(+.98)PQAGSLK.T	N	66.40	1389.6776	13	0.9	695.8467	2	28.07	7	1450	OB4035.raw	1.89E5	1	1	342	354	
K.SQSENFEYVAFKTD.S	N	66.17	1663.7366	14	0.2	832.8757	2	30.71	7	1635	OB4035.raw	1.38E5	1	1	436	449	
R.SPDIYN(+.98)PQ(+.98)AGSLK.T	N	63.44	1390.6616	13	0.4	696.3384	2	28.62	7	1501	OB4035.raw	3.67E4	1	1	342	354	Deamidation (NQ)
R.SPDIYN(+.98)PQAGSLKTANDLNLLILR.W	N	62.73	2626.3965	24	0.3	876.4730	3	35.57	7	1972	OB4035.raw	0	0	0	342	365	
K.TANDLNLLILRWLGP(+15.99)SAEYGNLYR.N	Y	61.14	2777.4500	24	2.6	926.8263	3	42.24	7	2386	OB4035.raw	0	0	0	355	378	Hydroxylation Pro
R.NRSPDIYNPQ(+.98)AGSLKTAN(+.98)DLNLLILR.W	N	56.12	2897.5247	26	4.9	725.3920	4	34.35	7	1904	OB4035.raw	3.34E4	1	1	340	365	
R.NRSPDIYNPQAGSLK.T	N	54.77	1658.8376	15	0.5	830.4265	2	25.62	7	1308	OB4035.raw	9.56E4	3	3	340	354	
R.NRSPDIYN(+.98)PQAGSLKTAN(+.98)DLNLLILR.W	N	53.50	2897.5247	26	4.1	725.3914	4	33.94	7	1852	OB4035.raw	0	0	0	340	365	
R.NRSPDIYNPQ(+.98)AGSLKTANDLNLLILR.W	N	52.65	2896.5405	26	0.8	725.1430	4	32.66	7	1765	OB4035.raw	8.81E5	1	1	340	365	
R.SPDIYN(+.98)PQ(+.98)AGSLKTAN(+.98)DLN(+.98)LLILR.W	N	52.36	2629.3486	24	7.1	877.4630	3	34.83	7	1912	OB4035.raw	1.17E5	1	1	342	365	Deamidation (NQ)
R.VYDEELQEGHVLVVPQNFAVAGK.S	N	51.87	2540.2910	23	0.6	847.7714	3	31.67	7	1692	OB4035.raw	1.53E5	1	1	413	435	
R.NRSPDIYNPQ(+.98)AGSLK.T	N	51.37	1659.8217	15	-0.8	830.9175	2	26.31	7	1347	OB4035.raw	1.5E5	2	2	340	354	Deamidation (NQ)
K.SQSE(+28.03)NFEYVAFK.T	N	51.18	1475.6932	12	0.5	738.8542	2	32.29	7	1738	OB4035.raw	7.72E4	1	1	436	447	
K.TANDLNLLILR.W	N	50.78	1254.7296	11	0.0	628.3721	2	33.43	7	1928	OB4035.raw	5.74E6	1	1	355	365	
R.NRSPDIYN(+.98)PQ(+.98)AGSLKTAN(+.98)DLNLLILR.W	N	50.70	2898.5085	26	1.8	725.6357	4	33.73	7	1852	OB4035.raw	8.77E4	1	1	340	365	
K.TDSRPSIANLAGEN(+.98).S	N	49.56	1444.6793	14	0.0	723.3469	2	28.43	7	1486	OB4035.raw	2.32E4	1	1	448	461	Deamidation (NQ)
K.T(+43.01)(+14.02)ANDLNLLILR.W	N	48.90	1311.7510	11	0.4	656.8831	2	33.65	7	1825	OB4035.raw	1.5E5	1	1	355	365	Carbamylation; Methylation(others)
K.TDSRPSIAN(+.98)LAGEN(+.98).S	N	46.28	1445.6633	14	0.1	723.8390	2	29.02	7	1514	OB4035.raw	2.64E4	1	1	448	461	Deamidation (NQ)
R.NRSPDIYNPQAGSLKTAN(+.98)DLNLLILR.W	N	46.23	2896.5405	26	0.8	725.1430	4	32.66	7	1753	OB4035.raw	8.81E5	1	1	340	365	
K.SQ(+.98)SENFEYVAFKTD.S	N	45.20	1664.7206	14	-1.6	833.3662	2	31.06	7	1647	OB4035.raw	2.27E4	1	1	436	449	Deamidation (NQ)
K.S(+43.01)(+14.02)QSENFEYVAFK.T	N	44.52	1504.6833	12	-0.1	753.3489	2	30.43	7	1603	OB4035.raw	7.06E4	1	1	436	447	Carbamylation; Methylation(others)
R.NRSPDIYNPQAGSLKTANDLNLLILR.W	N	43.70	2895.5566	26	0.7	724.8970	4	32.30	7	1734	OB4035.raw	0	0	0	340	365	

Peptide	Uniq	-10lgP	Mass	Length	ppm	m/z	z	RT	Fraction	Scan	Source File	Area KOntrola 6. traka	#Feature	#Feature KOntrola 6. traka	Start	End	PTM
K.SQSENFE(+53.92)YVAFK.T	N	43.57	1501.5812	12	0.4	751.7982	2	30.26	7	1592	OB4035.raw	2.51E4	1	1	436	447	
K.TAN(+.98)DLNLLILR.W	N	43.37	1255.7136	11	1.3	628.8649	2	34.04	7	1848	OB4035.raw	1.97E6	1	1	355	365	Deamidation (NQ)
A.NDLNLLILR.W	N	43.35	1082.6448	9	-0.8	542.3292	2	33.34	7	1810	OB4035.raw	2.03E4	1	1	357	365	
K.TAND(+28.03)LNLLILR.W	N	43.32	1282.7609	11	-0.2	642.3876	2	34.83	7	1924	OB4035.raw	9.42E4	1	1	355	365	
R.AHVQVVDSN(+.98)GN(+.98)RVYDEELQ(+.98)EGHVLVVPQNFAVAGK.S	N	43.28	3819.8704	35	5.2	955.9799	4	31.40	7	1672	OB4035.raw	0	0	0	401	435	
R.VYDEELQEGHVLVVPQ(+.98)N(+.98)FAVAGK.S	N	42.89	2542.2590	23	-0.1	848.4269	3	32.47	7	1755	OB4035.raw	1.18E5	1	1	413	435	Deamidation (NQ)
R.NRSPDIYN(+.98)PQAGSLK.T	N	42.75	1659.8217	15	-0.4	554.2809	3	25.82	7	1316	OB4035.raw	1.71E5	2	2	340	354	
R.NRSPDIYNP(+15.99)QAGSLKTANDLNLLILR.W	N	42.57	2911.5515	26	3.0	728.8973	4	34.73	7	1936	OB4035.raw	7.19E4	1	1	340	365	
R.S(+41.03)P(+15.99)DIYNPQAGSLK.T	N	42.04	1445.7150	13	0.0	723.8647	2	27.55	7	1427	OB4035.raw	5.56E3	1	1	342	354	Amidination of lysines or N-terminal amines with methyl acetimidate; Hydroxylation Pro
R.AHVQVVDSN(+.98)GNRVYDEELQ(+.98)EGHVLVVPQNFAVAGK.S	N	40.63	3818.8862	35	3.5	955.7322	4	31.54	7	1682	OB4035.raw	0	0	0	401	435	
R.NRSPDIYNP(+15.99)QAGSLKTAN(+.98)DLNLLILR.W	N	40.29	2912.5354	26	3.8	729.1439	4	33.34	7	1836	OB4035.raw	6.68E5	1	1	340	365	
R.W(+71.04)LGP(+15.99)SAEYGNLYR.N	Y	39.86	1611.7681	13	4.8	806.8951	2	37.80	7	2113	OB4035.raw	4.25E3	1	1	366	378	Propionamide (K, X@N-term); Hydroxylation Pro
R.NRSPDIYNP(+15.99)QAGSLKTANDLN(+.98)LLILR.W	N	39.51	2912.5354	26	3.8	729.1439	4	33.34	7	1818	OB4035.raw	6.68E5	1	1	340	365	
K.TAN(+.98)DLN(+.98)LLILR.W	N	39.22	1256.6976	11	2.9	629.3579	2	34.54	7	1897	OB4035.raw	3.72E4	1	1	355	365	Deamidation (NQ)
R.WLGP(+15.99)S(+13.03)AEYGNLYR.N	Y	39.18	1553.7626	13	4.7	777.8922	2	34.19	7	1867	OB4035.raw	2.63E5	1	1	366	378	Hydroxylation Pro; Michael addition with methylamine
R.S(+42.01)P(+15.99)DIYNPQAGSLKTANDLNLLILR.W	N	38.78	2683.4180	24	-0.5	895.4795	3	34.11	7	1864	OB4035.raw	9.81E4	1	1	342	365	Acetylation (N-term)
R.NRSPDIYNPQAGSLKTAN(+.98)DLN(+.98)LLILR.W	N	38.68	2897.5247	26	1.0	725.3892	4	33.45	7	1814	OB4035.raw	0	0	0	340	365	
K.SQSENFEYVAFKTDSRPSIANLAG.E	N	37.87	2630.2612	24	0.0	877.7610	3	31.67	7	1694	OB4035.raw	4.54E4	1	1	436	459	
K.TAND(+53.92)LNLLILR.W	N	37.22	1308.6488	11	0.0	655.3317	2	33.43	7	1821	OB4035.raw	5.21E4	1	1	355	365	Replacement of 2 protons by iron
K.TDSRPSIAN(+.98)LAGEN.S	N	36.99	1444.6793	14	0.0	723.3469	2	28.43	7	1499	OB4035.raw	2.32E4	1	1	448	461	Deamidation (NQ)
R.WLGP(+29.04)SAEYGNLYR.N	Y	36.60	1553.7753	13	-3.5	777.8922	2	34.19	7	1900	OB4035.raw	2.63E5	1	1	366	378	Dimethylation of proline residue
R.AHVQVVDSN(+.98)GNRVYDEELQEGHVLVVPQNFAVAGK.S	N	36.49	3817.9023	35	-0.8	955.4821	4	31.34	7	1648	OB4035.raw	2.77E5	1	1	401	435	
R.AHVQVVDSNGN(+.98)RVYDEELQEGHVLVVPQNFAVAGK.S	N	36.28	3817.9023	35	-0.8	955.4821	4	31.34	7	1663	OB4035.raw	2.77E5	1	1	401	435	

Peptide	Uniq	-10lgP	Mass	Length	ppm	m/z	z	RT	Fraction	Scan	Source File	Area KOntrola 6. traka	#Feature	#Feature KOntrola 6. traka	Start	End	PTM
K.SQSE(+21.98)NFEYVAFK.T	N	35.67	1469.6438	12	1.1	735.8300	2	30.26	7	1606	OB4035.raw	1.19E4	1	1	436	447	
K.TDSRPSIANLAGE.N	N	35.36	1329.6525	13	0.7	665.8340	2	28.33	7	1479	OB4035.raw	1.91E4	1	1	448	460	
G.SLKTANDLN(+.98)LLILR.W	N	35.26	1583.9246	14	-0.3	528.9820	3	32.66	7	1759	OB4035.raw	5.11E4	1	1	352	365	
K.TDSRPSIAN(+.98)LAG.E	N	34.14	1201.5939	12	1.0	601.8048	2	28.72	7	1485	OB4035.raw	4.76E4	1	1	448	459	Deamidation (NQ)
R.VYDEELQ(+.98)EGHVLVVP(+15.99)QNFAVAGK.S	N	32.20	2557.2700	23	-3.0	853.4280	3	32.95	7	1778	OB4035.raw	0	0	0	413	435	Deamidation (NQ); Hydroxylation Pro
R.SPDIYN(+.98)PQ(+.98)AGSLKTAN(+.98)DLNLLILR.W	N	31.82	2628.3645	24	7.6	658.1034	4	34.43	7	1880	OB4035.raw	1.37E4	1	1	342	365	
R.W(+42.01)LGP(+15.99)S(-18.01)AEYGNLYR.N	Y	31.58	1564.7310	13	-4.3	522.5820	3	33.34	7	1807	OB4035.raw	4.58E4	1	1	366	378	Acetylation (N-term); Hydroxylation Pro; Dehydration
K.TDSRPSIANLA.G	N	31.18	1143.5884	11	0.3	572.8016	2	27.88	7	1465	OB4035.raw	1.36E4	1	1	448	458	
R.AHVQVVDSNGNRVYDEELQ(+.98)EGHVLVVPQNFAVAGK.S	N	31.15	3817.9023	35	1.2	764.5887	5	31.42	7	1628	OB4035.raw	1.05E6	1	1	401	435	
R.AHVQVVDSNGNRVYDEELQEGHVLVVPQNFAVAGK.S	N	30.77	3816.9182	35	0.4	764.3912	5	30.69	7	1624	OB4035.raw	0	0	0	401	435	
R.VYDEELQ(+.98)EGHVLVVP(+15.99)QN(+.98)FAVAGK.S	N	30.74	2558.2539	23	2.3	853.7605	3	32.92	7	1778	OB4035.raw	7.47E3	1	1	413	435	Deamidation (NQ); Hydroxylation Pro
R.SPD(+21.98)IYNPQAGS(-18.01)LKTANDLNLLILR.W	N	30.41	2629.3840	24	1.5	877.4700	3	34.43	7	1884	OB4035.raw	1.89E5	1	1	342	365	
R.NRSPDIYN(+.98)P(+15.99)Q(+.98)AGSLKTANDLNLLILR.W	N	29.40	2913.5195	26	-0.5	729.3868	4	34.64	7	1905	OB4035.raw	0	0	0	340	365	
R.AHVQVVDSNGNRVYDEELQ(+.98)EGHVLVVPQ(+.98)NFAVAGK.S	N	28.70	3818.8862	35	4.9	764.7883	5	31.34	7	1668	OB4035.raw	0	0	0	401	435	
L.G(+316.20)PSAEYGNLYR.N	Y	27.27	1541.7766	11	-2.2	771.8939	2	49.80	7	2866	OB4035.raw	0	0	0	368	378	Levuglandinyl-lysine pyrrole adduct
K.TDSRPSIAN(+.98)LA.G	N	27.12	1144.5724	11	-1.0	573.2929	2	28.82	7	1508	OB4035.raw	3.26E4	1	1	448	458	Deamidation (NQ)
R.NRSPDIYNPQAGSLKT(-18.01)AND(+21.98)LNLLILR.W	N	26.92	2899.5281	26	2.0	725.8907	4	33.01	7	1797	OB4035.raw	4.04E5	2	2	340	365	Sodium adduct
R.AHVQVVDSN(+.98)GN(+.98)RVYDEELQEGHVLVVPQNFAVAGK.S	N	26.20	3818.8862	35	6.3	955.7348	4	30.87	7	1636	OB4035.raw	0	0	0	401	435	
R.VYDEELQEGHVLVVP(+15.99)QNFAVAGK.S	N	25.47	2556.2859	23	6.6	853.1082	3	33.01	7	1778	OB4035.raw	7.76E3	1	1	413	435	Hydroxylation Pro
K.FFVPPSQ(+.98)Q(+.98)SPR.A	N	25.37	1290.6244	11	-0.6	646.3191	2	28.17	7	1475	OB4035.raw	4.4E3	1	1	494	504	Deamidation (NQ)
R.NRSPDIYN(+.98)PQ(+.98)AGSLK.T	N	23.21	1660.8057	15	-0.7	554.6088	3	27.13	7	1410	OB4035.raw	1.72E4	1	1	340	354	Deamidation (NQ)
K.TDSRPSIANLAG.E	N	23.17	1200.6099	12	0.1	601.3123	2	27.97	7	1447	OB4035.raw	2.3E4	1	1	448	459	
R.W(+42.01)LGPSAE(+43.04)YGNLYR.N	Y	22.60	1609.7888	13	0.7	805.9022	2	37.42	7	2088	OB4035.raw	9.33E4	1	1	366	378	Acetylation (N-term); Carboxyl modification with ethanolamine
R.AHVQ(+.98)VVDSNGNRVYDEELQEGHVLVVPQNFAVAGK.S	N	22.40	3817.9023	35	1.2	764.5887	5	31.42	7	1642	OB4035.raw	1.05E6	1	1	401	435	
R.SPDIYNPQAGSLKT(-18.01)AND(+21.98)LNLLILR.W	N	22.35	2629.3840	24	1.5	877.4700	3	34.43	7	1912	OB4035.raw	1.89E5	1	1	342	365	

Peptide	Uniq	-10lgP	Mass	Length	ppm	m/z	z	RT	Fraction	Scan	Source File	Area KOntrola 6. traka	#Feature	#Feature KOntrola 6. traka	Start	End	PTM
R.AHVQVVDSNGNRVYDEELQEGHVLVVPQ(+.98)N(+.98)FAVAGK.S	N	22.34	3818.8862	35	1.5	764.7856	5	31.51	7	1680	OB4035.raw	0	0	0	401	435	
R.N(+.98)RSPDIYNPQAGSLK.T	N	22.33	1659.8217	15	-0.5	554.2809	3	26.23	7	1389	OB4035.raw	1.47E5	1	1	340	354	
R.AHVQ(+.98)VVDSNGN(+.98)RVYDEELQEGHVLVVPQN(+.98)FAVAGK.S	N	22.20	3819.8704	35	7.5	764.9871	5	32.04	7	1717	OB4035.raw	0	0	0	401	435	
**total 82 peptides**																	

Q647H2|AHY3_ARAHY

back to list

| Protein Coverage | Supporting Peptides |

Protein Coverage:

```
  1 MAKLLALSVC FCFLVLGASS VTFRQQGEEN ECQFQRLNAQ RPDNCIESEG GYIETWNPNN QEFQCAGVAL SRFVLRRNAL
 81 RRPFYSNAPQ EIFIYQGSGY FGLIFPGCPG TFEEPIQGSE QFQRPSRHFQ GQDQSQRPLD THQKVHGFRE GDLIAVPHGV
161 AFWIYNDQDT DVVAISVLHT NSLHNQLDQF PRRFNLAGKQ EQEFLRYQQR SGRQSPKGEE QEQEQENEGG NVFSGFSTEF
241 LSHGFQVNED IVRNLRGENE REEQGAIVTV KGGLSILVPP EWRQSYQQPG RGDKDFNNGI EETICTATVK MNIGKSTSAD
321 IYNPQAGSVR TVNELDLPIL NRLGLSAEYG SIHRDAMFVP HYNMNANSMI YALHGGAHVQ VVDCNGNRVF DEELQEGQSL
401 VVPQNFAVAA KSQSEHFLYV AFKTNSRASI SNLAGKNSYM WNLPEDVVAN SYGLQYEQAR QLKNNNPFTF LVPPQDSQMI
481 RTVA
```

c Carbamidomethylation (+57.02)
c Carboxymethyl (+58.01)
d Deamidation (NQ) (+0.98)
p Propionamide (+71.04)
u Ubiquitin (+114.04)

Supporting Peptides:

Peptide	Uniq	-10lgP	Mass	Length	ppm	m/z	z	RT	Fraction	Scan	Source File	Area KOntrola 6. traka	#Feature	#Feature KOntrola 6. traka	Start	End	PTM
R.TVN(+.98)ELDLPILNR.L	Y	76.49	1396.7561	12	0.2	699.3855	2	33.62	7	1827	OB4035.raw	3.66E4	1	1	331	342	Deamidation (NQ)
N.GIEETIC(+57.02)TATVK.M	N	72.36	1320.6595	12	0.8	661.3376	2	28.07	7	1473	OB4035.raw	6.45E4	1	1	299	310	Carbamidomethylation
R.TVNELDLPILNR.L	Y	70.12	1395.7721	12	0.0	698.8933	2	33.20	7	1788	OB4035.raw	1.58E5	1	1	331	342	

Peptide	Uniq	-10lgP	Mass	Length	ppm	m/z	z	RT	Fraction	Scan	Source File	Area KOntrola 6. traka	#Feature	#Feature KOntrola 6. traka	Start	End	PTM
N.GIEETIC(+71.04)TATVK.M	N	50.49	1334.6752	12	0.2	668.3450	2	28.43	7	1483	OB4035.raw	3.08E4	1	1	299	310	Propionamide
N.GIEETIC(+58.01)TATVK.M	N	35.77	1321.6436	12	-0.5	661.8287	2	28.83	7	1507	OB4035.raw	9.22E3	1	1	299	310	Carboxymethyl
K.SQSEHFLYVAFK.T	Y	33.96	1454.7194	12	-1.8	485.9129	3	30.34	7	1605	OB4035.raw	2.27E4	1	1	412	423	
G.IEET(+114.04)IC(+57.02)TATVK.M	N	26.88	1377.6810	11	-0.5	689.8474	2	28.17	7	1481	OB4035.raw	9.08E3	1	1	300	310	Ubiquitin; Carbamidomethylation
**total 7 peptides**																	

tr|J9QGM3|J9QGM3_ARAHY
back to list

| Protein Coverage | Supporting Peptides |

Protein Coverage:

```
  1 MVLPSSLSAV QVDNVTFPAT AKPPGSDKTF FLGGAGVRGL QIDDKFVKFT AIAVYLQDNA VPSLAVKWNG KTPSELTESV
 81 DFFRDIVTGP FEKFMQVTMI LPLTGQQYSQ KVSENCVAIW KHLGIYTDQE ANAIDKFLSV FKDQNFPPDS SILFTLLPNG
161 SLVIGFSKDG SIPEAGTAVI ENKLLSEAVL ESMIGKHGVS PAAKQSLATR LYELFKQSGH DDTADKKLHD FDTDNGLHHT
241 KSGNSVAEDA AEKLS
```

d Deamidation (NQ) (+0.98)

Supporting Peptides:

Peptide	Uniq	-10lgP	Mass	Length	ppm	m/z	z	RT	Fraction	Scan	Source File	Area KOntrola 6. traka	#Feature	#Feature KOntrola 6. traka	Start	End	PTM
K.LLSEAVLESMIGKHGVSPAAKQ(+.98)SLATR.L	Y	27.75	2793.5059	27	3.2	932.1788	3	41.79	7	2359	OB4035.raw	5.78E3	1	1	184	210	Deamidation (NQ)
**total 1 peptides**																	

Peptide List

Prepared with PEAKS ™ (bioinfor.com)

Summary

1. Notes Gastric Digest Raw peanut Band #6 22-24 kDa (PTM493)

2. Result Statistics

Figure 1. False discovery rate (FDR) curve. X axis is the number of peptide-spectrum matches (PSM) being kept. Y axis is the corresponding FDR.

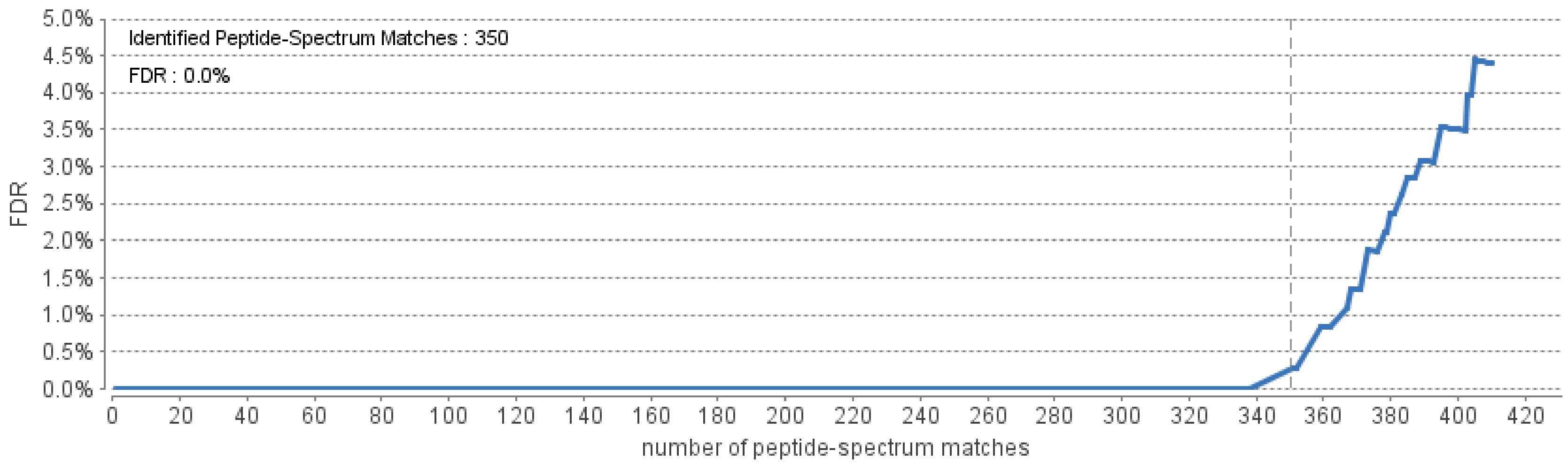

Figure 2. PSM score distribution. **(a)** Distribution of PEAKS peptide score; **(b)** Scatterplot of PEAKS peptide score versus precursor mass error.

(a) **(b)**

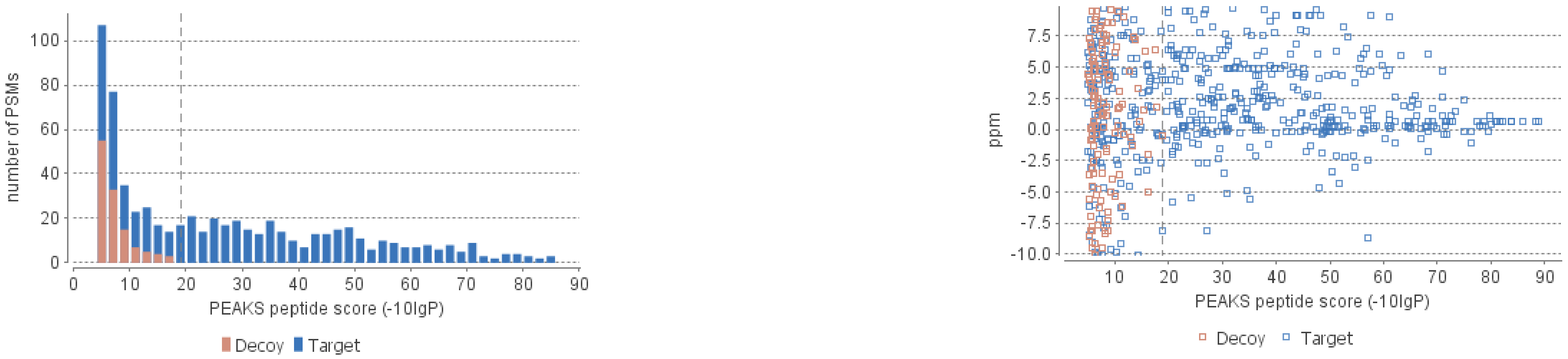

Figure 3. Distribution of peptide feature detection. **(a)** Feature m/z distribution; **(b)** Feature RT distribution

(a)

(b)

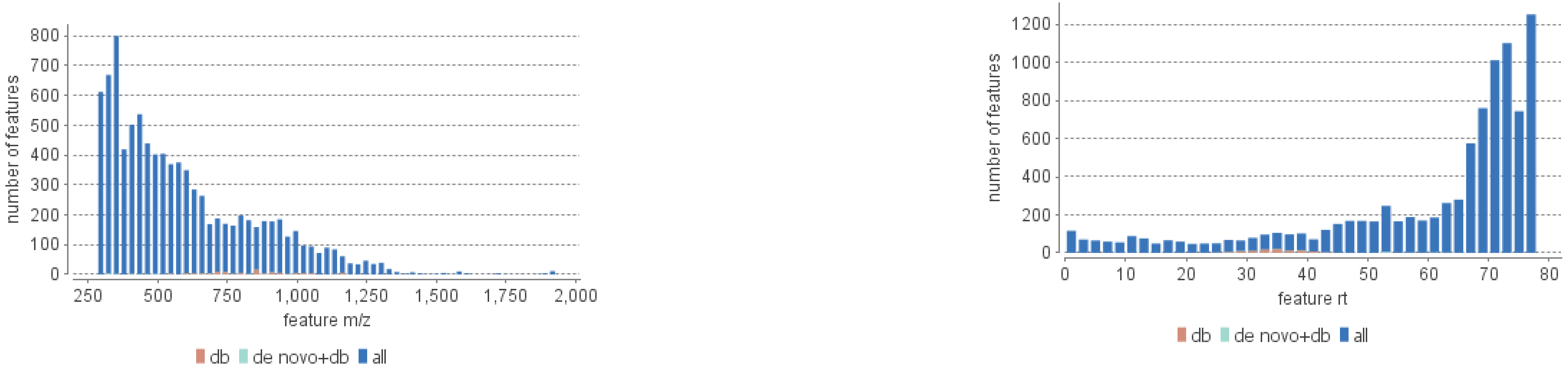

Figure 4. Distribution of identified peptide features. **(a)** Feature abundance distribution **(b)** *De novo* sequencing validation.

(a)

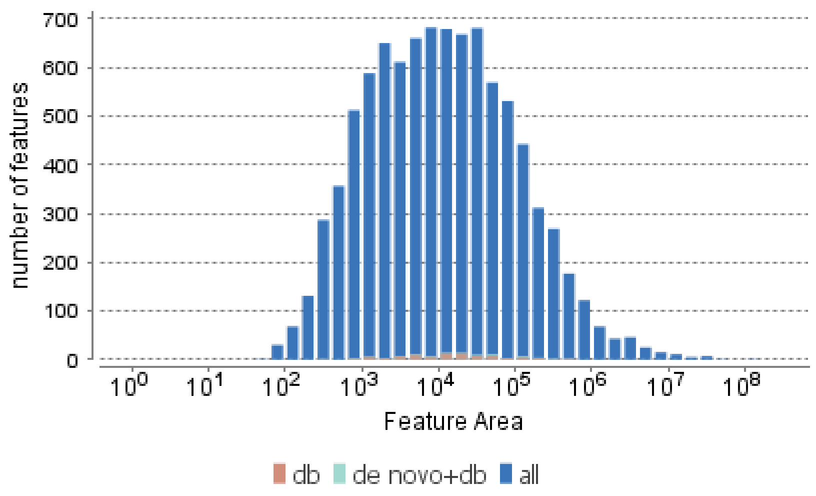

(b)

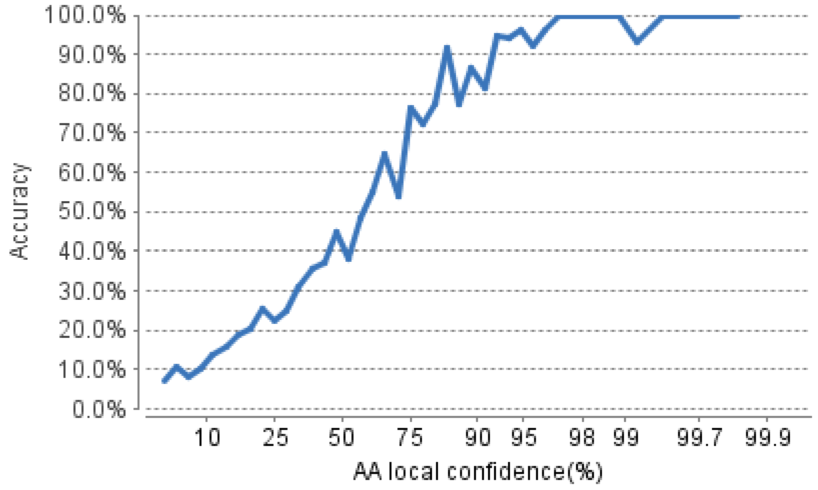

Table 1. Statistics of data.

# of MS scans	2956
# of MS/MS scans	1344
# of Features	9245
# of Chimera scans	168

Table 2. Result filtration parameters.

Peptide -10lgP	≥19
PTM Ascore	≥20
Protein -10lgP	≥20
Proteins unique peptides	≥1
De novo ALC Score	≥50%

Table 4. PTM profile.

Name	ΔMass	Position	#PSM	-10lgP	Abundance	AScore
Deamidation	.98	NQ	227	76.21	2.81E4	1000.00
HydPro	15.99	P	19	37.91		15.48
Carbamidomethyl	57.02	C	12	60.31	1.41E3	1000.00
Ammonium	17.03	E	7	30.75	5.01E4	13.13
Dihydroxy	31.99	RW	6	35.25		7.21
Oxidation	15.99	M	5	36.99	2.6E3	1000.00
LG-pyrrole	316.20	N-term	2	27.23	5.01E4	1000.00

Table 3. Statistics of filtered result.

Peptide-Spectrum Matches	350
Peptide sequences	182
Protein groups	6
Proteins	9
Proteins (#Unique Peptides)	7 (>2); 1 (=2); 1 (=1);
FDR (Peptide-Spectrum Matches)	0.0%
FDR (Peptide Sequences)	0.0%
FDR (Protein Group)	0.0%
De Novo Only Spectra	28
# of identified Features	105
# of identified MS/MS scans	300

Methylation(KR)	14.02	KR	2	30.40	1.14E5	18.31
Amidation	-.98	C-term	1	30.84		1000.00

3. Experiment Control

Figure 5. Precursor mass error of peptide-spectrum matches (PSM) in filtered result. **(a)** Distribution of precursor mass error in ppm; **(b)** Scatterplot of precursor m/z versus precursor mass error in ppm.

(a)

(b)

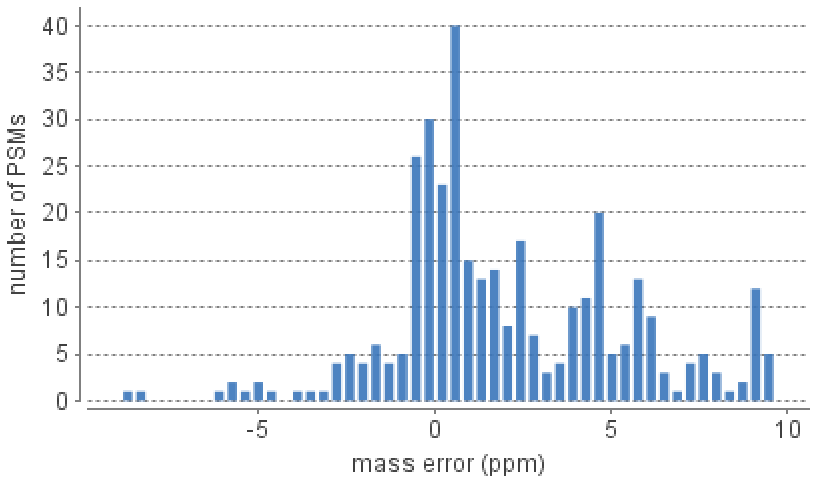

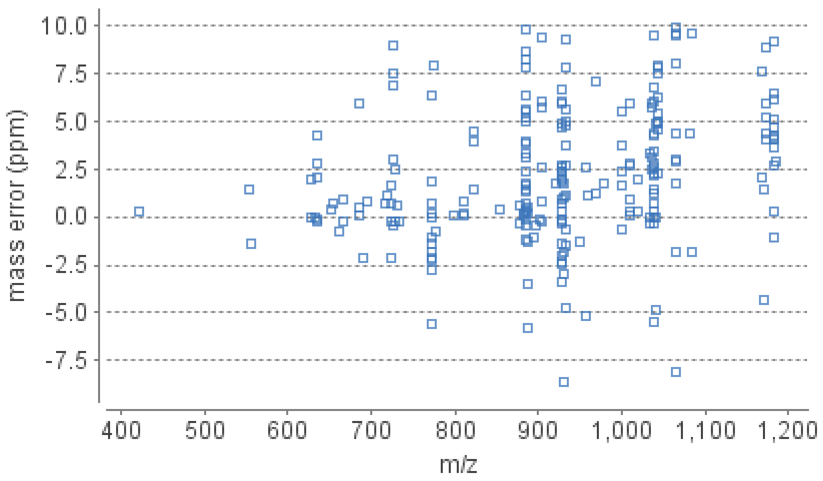

Table 5. Number of identified peptides in each sample by the number of missed cleavages

Missed Cleavages	0	1	2	3	4+
Digest 6. traka 23-24 kDa	73	63	46	0	0

4. Other Information

Table 6. Search parameters.

Search Engine Name: PEAKS
Parent Mass Error Tolerance: 10.0 ppm
Fragment Mass Error Tolerance: 0.5 Da
Precursor Mass Search Type: monoisotopic
Enzyme: Trypsin
Max Missed Cleavages: 2
Digest Mode: Unspecific

Table 7. Instrument parameters.

Fractions: OB4056.raw
Ion Source: ESI(nano-spray)
Fragmentation Mode: CID, CAD(y and b ions)
MS Scan Mode: FT-ICR/Orbitrap
MS/MS Scan Mode: Linear Ion Trap

Fixed Modifications:
  Carbamidomethylation: 57.02
Variable Modifications:
  Deamidation (NQ): 0.98
  Oxidation (M): 15.99
  Hydroxylation Pro: 15.99
  Acetylation (K): 42.01
  Acetylation (Protein N-term): 42.01
  Acetylation (N-term): 42.01
  Amidation: -0.98
  Beta-methylthiolation: 45.99
  and 305 more...
Max Variable PTM Per Peptide: 5
Database: Uniprot_Peanut-3818_Jul18
Taxon: All
Contaminant Database: contaminantsMQ_mar19
Searched Entry: 1723
FDR Estimation: Enabled
De novo score (ALC%) threshold: 15
Peptide hit threshold (-10logP): 30.0
Peaks run ID: 481
Merge Options: no merge
Precursor Options: corrected
Charge Options: no correction
Filter Charge: 2 - 8
Process: true
Associate chimera: yes

Protein List

Protein Accession Contains:

Protein Description Contains:

Peptide Sample Area >=

Protein Ptm Contains:

Protein Group	Protein ID	Accession	-10lgP	Coverage (%)	Coverage (%) Digest 6. traka 23-24 kDa	Area Digest 6. traka 23-24 kDa	#Peptides	#Unique	#Spec Digest 6. traka 23-24 kDa	PTM	Avg. Mass	Description
**3**	20581	tr|Q647H3|Q647H3_ARAHY	235.17	34	34	1.63E5	18	3	123	Y	61532	Arachin Ahy-2 OS=Arachis hypogaea OX=3818 PE=2 SV=1
**6**	20578	tr|B5TYU1|B5TYU1_ARAHY	231.54	32	32	1.17E3	16	1	94	Y	60624	Arachin Arah3 isoform OS=Arachis hypogaea OX=3818 PE=1 SV=1
**5**	20582	tr|Q8LKN1|Q8LKN1_ARAHY	214.35	34	34	3.57E5	15	2	93	Y	61738	Allergen Arah3/Arah4 OS=Arachis hypogaea OX=3818 PE=3 SV=1
**8**	20590	tr|O82580|O82580_ARAHY	182.12	23	23	3.67E5	13	4	34	Y	58350	Glycinin (Fragment) OS=Arachis hypogaea OX=3818 GN=Arah3 PE=2 SV=1
**7**	20593	tr|Q6IWG5|Q6IWG5_ARAHY	172.18	24	24	2.29E6	8	7	83	Y	58061	Glycinin (Fragment) OS=Arachis hypogaea OX=3818 PE=2 SV=1
**7**	20594	tr|Q0GM57|Q0GM57_ARAHY	172.18	23	23	2.29E6	8	7	83	Y	58263	Iso-Ara h3 OS=Arachis hypogaea OX=3818 PE=2 SV=1
**9**	22400	tr|Q6PSU4|Q6PSU4_ARAHY	96.82	17	17	1.24E5	5	5	8	Y	48095	Conarachin (Fragment) OS=Arachis hypogaea OX=3818 PE=2 SV=1
**9**	20853	tr|N1NG13|N1NG13_ARAHY	96.82	12	12	1.24E5	5	5	8	Y	71345	Seed storage protein Ara h1 OS=Arachis hypogaea OX=3818 GN=ARAX_AHF417E07-017 PE=4 SV=1
**9**	20854	P43238|ALL12_ARAHY	96.82	12	12	1.24E5	5	5	8	Y	71345	Allergen Ara h 1, clone P41B OS=Arachis hypogaea OX=3818 PE=1 SV=1
**total 9 proteins**												

tr|Q647H3|Q647H3_ARAHY
back to list

| Protein Coverage | Supporting Peptides |

Protein Coverage:

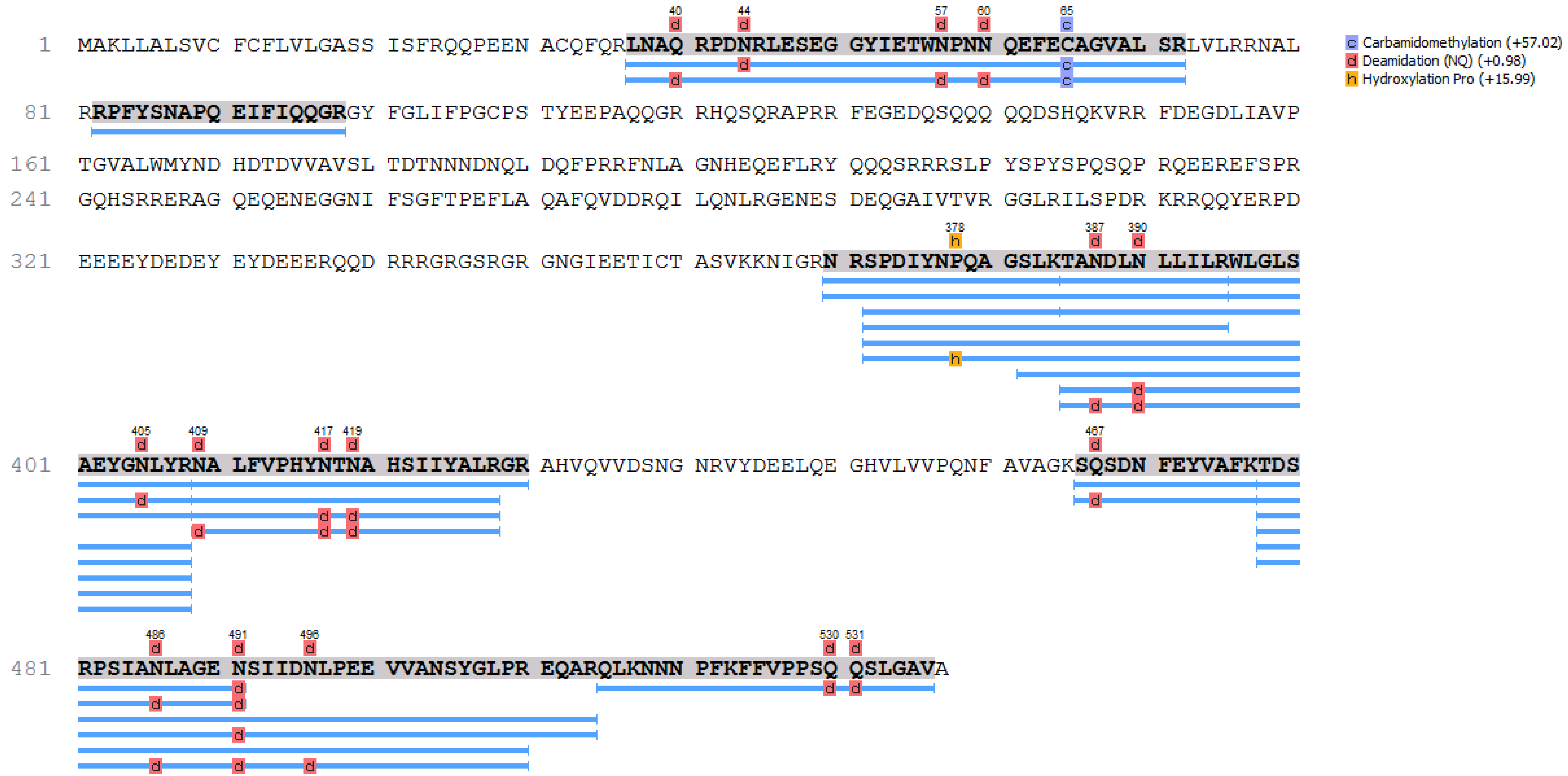

Supporting Peptides:

Peptide	Uniq	-10lgP	Mass	Length	ppm	m/z	z	RT	Fraction	Scan	Source File	Area Digest 6. traka 23-24 kDa	#Feature	#Feature Digest 6. traka 23-24 kDa	Start	End	PTM
R.WLGLSAEYGNLYR.N	N	88.77	1540.7673	13	0.7	771.3915	2	33.17	28	1701	OB4056.raw	5E5	1	1	396	408	
K.TANDLNLLILRWLGLSAEYGNLYR.N	N	78.42	2777.4863	24	0.3	926.8363	3	39.72	28	2101	OB4056.raw	2.35E5	2	2	385	408	
R.WLGLSAEYGN(+.98)LYR.N	N	76.21	1541.7513	13	-1.1	771.8821	2	33.52	28	1720	OB4056.raw	2.81E4	1	1	396	408	Deamidation (NQ)
K.TANDLN(+.98)LLILRWLGLSAEYGNLYR.N	N	74.92	2778.4705	24	2.3	927.1663	3	40.03	28	2125	OB4056.raw	6.94E2	1	1	385	408	Deamidation (NQ)
K.TDSRPSIANLAGEN(+.98)SIIDNLPEEVVANSYGLPR.E	Y	73.37	3511.7429	33	1.5	1171.5900	3	35.69	28	1854	OB4056.raw	1.34E4	1	1	478	510	
K.SQSDNFEYVAFK.T	N	72.81	1433.6462	12	0.7	717.8309	2	30.26	28	1521	OB4056.raw	6.85E3	1	1	466	477	
K.TAN(+.98)DLNLLILRWLGLSAEYGNLYR.N	N	66.39	2778.4705	24	2.1	927.1660	3	41.49	28	2216	OB4056.raw	6.94E2	1	1	385	408	
R.SPDIYNPQ(+.98)AGSLK.T	N	61.16	1389.6776	13	0.8	695.8466	2	27.83	28	1387	OB4056.raw	5.83E3	1	1	372	384	
K.SQ(+.98)SDNFEYVAFK.T	N	61.13	1434.6302	12	1.2	718.3232	2	30.66	28	1546	OB4056.raw	3.03E3	1	1	466	477	Deamidation (NQ)
K.TDSRPSIANLAGEN(+.98)SIIDN(+.98)LPEEVVANSYGLPR.E	Y	59.80	3512.7271	33	5.2	1171.9224	3	36.17	28	1898	OB4056.raw	2.47E4	1	1	478	510	
R.NALFVPHYNTNAHSIIYALR.G	N	56.65	2313.2019	20	0.0	772.0746	3	31.12	28	1571	OB4056.raw	2.81E4	1	1	409	428	
R.RPFYSNAPQ(+.98)EIFIQQGR.G	N	56.36	2051.0225	17	0.0	684.6815	3	30.59	28	1544	OB4056.raw	0	0	0	82	98	
R.SPDIYNPQ(+.98)AGSLKTAN(+.98)DLNLLILR.W	N	55.70	2627.3806	24	-0.3	876.8005	3	34.26	28	1763	OB4056.raw	1.44E4	1	1	372	395	
R.NRSPDIYNPQ(+.98)AGSLKTANDLNLLILR.W	N	53.30	2896.5405	26	7.5	725.1478	4	32.15	28	1639	OB4056.raw	0	0	0	370	395	
R.NALFVPHYN(+.98)TN(+.98)AHSIIYALR.G	N	52.81	2315.1699	20	-2.2	772.7289	3	32.47	28	1657	OB4056.raw	4.59E3	2	2	409	428	Deamidation (NQ)
K.TDSRPSIANLAGENSIIDN(+.98)LPEEVVANSYGLPR.E	Y	51.62	3511.7429	33	-4.3	1171.5833	3	35.90	28	1866	OB4056.raw	0	0	0	478	510	
R.NALFVPHYN(+.98)TNAHSIIYALR.G	N	51.39	2314.1858	20	0.1	772.4026	3	32.45	28	1657	OB4056.raw	2.88E4	1	1	409	428	
R.SPDIYNPQ(+.98)AGSLKTANDLNLLILR.W	N	49.51	2626.3965	24	0.6	876.4733	3	33.87	28	1745	OB4056.raw	1.12E4	1	1	372	395	
K.TDSRPSIAN(+.98)LAGEN(+.98)SIIDN(+.98)LPEEVVANSYGLPR.E	Y	49.46	3513.7109	33	4.4	1172.2494	3	36.30	28	1891	OB4056.raw	0	0	0	478	510	Deamidation (NQ)
K.TANDLNLLILR.W	N	49.10	1254.7296	11	0.0	628.3721	2	33.27	28	1702	OB4056.raw	7.58E4	1	1	385	395	
K.TAN(+.98)DLN(+.98)LLILRWLGLSAEYGNLYR.N	N	48.65	2779.4543	24	-0.4	927.4917	3	40.83	28	2175	OB4056.raw	0	0	0	385	408	Deamidation (NQ)
R.NALFVPHYNTN(+.98)AHSIIYALR.G	N	46.36	2314.1858	20	-1.4	772.4015	3	32.32	28	1649	OB4056.raw	0	0	0	409	428	
R.N(+.98)ALFVPHYN(+.98)TN(+.98)AHSIIYALR.G	N	44.58	2316.1538	20	8.0	773.0647	3	32.32	28	1649	OB4056.raw	1.28E3	1	1	409	428	Deamidation (NQ)
R.NRSPDIYN(+.98)PQAGSLKTAN(+.98)DLNLLILR.W	N	43.64	2897.5247	26	6.9	725.3934	4	32.47	28	1669	OB4056.raw	1.04E4	1	1	370	395	
R.RPFYSNAPQEIFIQQGR.G	N	42.32	2050.0383	17	0.5	684.3538	3	29.96	28	1513	OB4056.raw	6.68E3	1	1	82	98	
R.SPDIYN(+.98)PQAGSLKTANDLNLLILRWLGLSAEYGNLYR.N	N	41.26	4149.1533	37	0.8	1038.2964	4	39.72	28	2095	OB4056.raw	2.67E5	1	1	372	408	
R.SPDIYN(+.98)PQ(+.98)AGSLKTANDLNLLILRWLGLSAEYGNLYR.N	N	40.71	4150.1372	37	2.2	1038.5438	4	39.75	28	2107	OB4056.raw	0	0	0	372	408	
R.SPDIYNPQAGSLKTANDLNLLILRWLGLSAEYGNLYR.N	N	39.13	4148.1694	37	4.4	1038.0542	4	39.33	28	2081	OB4056.raw	0	0	0	372	408	

Peptide	Uniq	-10lgP	Mass	Length	ppm	m/z	z	RT	Fraction	Scan	Source File	Area Digest 6. traka 23-24 kDa	#Feature	#Feature Digest 6. traka 23-24 kDa	Start	End	PTM
R.SPDIYNP(+15.99)Q(+.98)AGSLKTANDLNLLILRWLGLSAEYGNLYR.N	N	37.91	4165.1484	37	6.3	1042.3009	4	40.96	28	2183	OB4056.raw	0	0	0	372	408	
R.SPDIYNPQAGSLKTAN(+.98)DLN(+.98)LLILRWLGLSAEYGNLYR.N	N	37.76	4150.1372	37	3.5	1038.5452	4	40.38	28	2147	OB4056.raw	0	0	0	372	408	
R.SPDIYN(+.98)PQ(+.98)AGSLKTAN(+.98)DLNLLILRWLGLSAEYGNLYR.N	N	36.89	4151.1216	37	4.3	1038.7921	4	40.51	28	2155	OB4056.raw	0	0	0	372	408	
R.SPDIYN(+.98)P(+15.99)Q(+.98)AGSLKTANDLNLLILRWLGLSAEYGNLYR.N	N	36.71	4166.1323	37	4.9	1042.5454	4	40.27	28	2141	OB4056.raw	5.68E4	1	1	372	408	Hydroxylation Pro
K.TDSRPSIANLAGEN(+.98).S	N	36.29	1444.6793	14	-2.1	723.3454	2	28.25	28	1412	OB4056.raw	3.88E3	1	1	478	491	Deamidation (NQ)
R.SPDIYN(+.98)PQAGSLKTAN(+.98)DLNLLILRWLGLSAEYGNLYR.N	N	35.78	4150.1372	37	2.3	1038.5439	4	40.33	28	2144	OB4056.raw	0	0	0	372	408	
R.SPDIYNPQAGSLKTAN(+.98)DLNLLILRWLGLSAEYGNLYR.N	N	35.54	4149.1533	37	0.8	1038.2964	4	39.72	28	2172	OB4056.raw	2.67E5	1	1	372	408	
R.SPDIYNPQAGSLKTANDLN(+.98)LLILRWLGLSAEYGNLYR.N	N	35.34	4149.1533	37	0.8	1038.2964	4	39.72	28	2119	OB4056.raw	2.67E5	1	1	372	408	
R.SPDIYNP(+15.99)Q(+.98)AGSLKTAN(+.98)DLNLLILRWLGLSAEYGNLYR.N	N	34.90	4166.1323	37	4.9	1042.5454	4	40.27	28	2159	OB4056.raw	5.68E4	1	1	372	408	Hydroxylation Pro
K.TDSRPSIANLAGENSIIDNLPEEVVANSYGLPREQ(+.98)AR.Q	Y	34.60	3995.9824	37	1.6	1000.0045	4	34.46	28	1771	OB4056.raw	9.47E4	1	1	478	514	
R.SPDIYNPQ(+.98)AGSLKTANDLN(+.98)LLILRWLGLSAEYGNLYR.N	N	32.30	4150.1372	37	1.5	1038.5431	4	41.44	28	2213	OB4056.raw	0	0	0	372	408	
R.SPDIYNP(+15.99)Q(+.98)AGSLKTANDLN(+.98)LLILRWLGLSAEYGNLYR.N	N	32.14	4166.1323	37	4.9	1042.5454	4	40.27	28	2143	OB4056.raw	5.68E4	1	1	372	408	Hydroxylation Pro
R.SPDIYNPQ(+.98)AGSLKTAN(+.98)DLNLLILRWLGLSAEYGNLYR.N	N	31.28	4150.1372	37	6.8	1038.5486	4	41.52	28	2218	OB4056.raw	0	0	0	372	408	
R.LNAQ(+.98)RPDNRLESEGGYIETWN(+.98)PNN(+.98)QEFEC(+57.02)AGVALSR.L	N	30.16	4136.8770	36	5.9	1035.2327	4	32.03	28	1632	OB4056.raw	0	0	0	37	72	Deamidation (NQ); Carbamidomethylation
K.TDSRPSIAN(+.98)LAGEN(+.98).S	N	28.45	1445.6633	14	0.7	723.8395	2	28.96	28	1450	OB4056.raw	6.9E2	1	1	478	491	Deamidation (NQ)
K.TDSRPSIANLAGEN(+.98)SIIDNLPEEVVAN(+.98)SYGLPREQ(+.98)AR.Q	Y	28.06	3997.9504	37	2.4	1000.4973	4	34.90	28	1805	OB4056.raw	0	0	0	478	514	Deamidation (NQ)
R.QLKN(+.98)N(+.98)NPFKFFVPPSQ(+.98)Q(+.98)SLGAV.A	Y	27.40	2463.2322	22	4.5	822.0884	3	32.56	28	1663	OB4056.raw	7.4E3	1	1	515	536	Deamidation (NQ)
R.LNAQRPDN(+.98)RLESEGGYIETWNPNNQEFEC(+57.02)AGVALSR.L	N	26.80	4134.9087	36	-0.3	1034.7341	4	31.42	28	1595	OB4056.raw	0	0	0	37	72	Deamidation (NQ); Carbamidomethylation
R.SPDIYNPQ(+.98)AGSLKTANDLNLLILRWLGLSAEYGNLYR.N	N	26.23	4149.1533	37	1.2	1038.2969	4	42.12	28	2255	OB4056.raw	0	0	0	372	408	
R.QLKNN(+.98)NPFKFFVPPSQ(+.98)Q(+.98)SLGAV.A	Y	26.18	2462.2480	22	4.0	821.7599	3	32.17	28	1674	OB4056.raw	1.16E4	1	1	515	536	Deamidation (NQ)
K.TDSRPSIANLAGEN(+.98)SIIDN(+.98)LPEEVVANSYGLPREQ(+.98)AR.Q	Y	26.08	3997.9504	37	3.7	1000.4986	4	34.77	28	1797	OB4056.raw	0	0	0	478	514	
K.TDSRPSIAN(+.98)LAGEN(+.98)SIIDN(+.98)LPEEVVANSYGLPREQ(+.98)AR.Q	Y	25.94	3998.9343	37	5.5	1000.7463	4	35.10	28	1817	OB4056.raw	0	0	0	478	514	
R.N(+.98)RSPDIYNPQ(+.98)AGSLKTANDLNLLILR.W	N	25.28	2897.5247	26	-0.4	725.3881	4	32.93	28	1686	OB4056.raw	0	0	0	370	395	
R.QLKNN(+.98)N(+.98)PFKFFVPPSQ(+.98)Q(+.98)SLGAV.A	Y	24.62	2463.2322	22	1.5	822.0859	3	32.74	28	1674	OB4056.raw	0	0	0	515	536	Deamidation (NQ)
K.TDSRPSIANLAGENSIIDNLPEEVVANSYGLPREQAR.Q	Y	21.65	3994.9983	37	-0.6	999.7562	4	34.24	28	1765	OB4056.raw	0	0	0	478	514	
R.NALFVPHYNTNAHSIIYALRGR.A	N	21.07	2526.3245	22	0.0	632.5884	4	29.97	28	1505	OB4056.raw	0	0	0	409	430	
K.TAN(+.98)DLNLLILR.W	N	20.82	1255.7136	11	1.9	628.8653	2	33.87	28	1744	OB4056.raw	3.85E4	1	1	385	395	

Peptide	Uniq	-10lgP	Mass	Length	ppm	m/z	z	RT	Fraction	Scan	Source File	Area Digest 6. traka 23-24 kDa	#Feature	#Feature Digest 6. traka 23-24 kDa	Start	End	PTM
R.NRSPDIYNPQAGSLK.T	N	19.79	1658.8376	15	1.5	553.9540	3	25.42	28	1243	OB4056.raw	1.39E4	1	1	370	384	
G.SLKTANDLNLLILRWLGLSAEYGNLYR.N	N	19.77	3105.6975	27	-0.8	777.4310	4	38.33	28	2028	OB4056.raw	4.91E3	1	1	382	408	
R.QLKNNN(+.98)PFKFFVPPSQ(+.98)Q(+.98)SLGAV.A	Y	19.55	2462.2480	22	4.0	821.7599	3	32.17	28	1667	OB4056.raw	1.16E4	1	1	515	536	Deamidation (NQ)
**total 58 peptides**																	

tr|B5TYU1|B5TYU1_ARAHY
back to list

| Protein Coverage | Supporting Peptides |

Protein Coverage:

c Carbamidomethylation (+57.02)
d Deamidation (NQ) (+0.98)

1 MAKLLELSFC FCFLVLGASS ISFRQQPEEN ACQFQR**LNAQ RPDNRIESEG GYIETWNPNN QEFECAGVAL SR**LVLRRNAL

81 R**RPFYSNAPQ EIFIQQGR**GY FGLIFPGCPS TYEEPAQQGR RYQSQRPPRR LQEEDQSQQQ QDSHQKVHRF NEGDLIAVPT

161 GVAFWLYNDH DTDVVAVSLT DTNNNDNQLD QFPRRFNLAG NHEQEFLRYQ QQSRQSRRRS LPYSPYSPQS QPRQEEREFS

241 PRGQHSRRER AGQEEENEGG NIFSGFTPEF LAQAFQVDDR QIVQNLRGEN ESEEQGAIVT VRGGLRILSP DRKRGADEEE

321 EYDEDEYEYD EEDRRRGRGS RGSGN**GIEET ICTATVK**KNI GR**NRSPDIYN PQAGSLKTAN ELNLLILRWL GLSAEYGNLY**

401 **RNALFVPHYN TNAHSIIYAL RGR**AHVQVVD SNGNRVYDEE LQEGHVLVVP QNFAVAGK**SQ SDNFEYVAFK TDSRPSIANL**

481 **AGENSVIDNL PEEVVANSYG LPR**EQARQLK NNNPFKFFVP PSQQSPRAVA

Supporting Peptides:

Peptide	Uniq	-10lgP	Mass	Length	ppm	m/z	z	RT	Fraction	Scan	Source File	Area Digest 6. traka 23-24 kDa	#Feature	#Feature Digest 6. traka 23-24 kDa	Start	End	PTM
R.WLGLSAEYGNLYR.N	N	88.77	1540.7673	13	0.7	771.3915	2	33.17	28	1701	OB4056.raw	5E5	1	1	389	401	
K.TANELNLLILRWLGLSAEYGNLYR.N	N	79.71	2791.5020	24	1.1	931.5089	3	39.72	28	2096	OB4056.raw	1.32E5	2	2	378	401	
R.WLGLSAEYGN(+.98)LYR.N	N	76.21	1541.7513	13	-1.1	771.8821	2	33.52	28	1720	OB4056.raw	2.81E4	1	1	389	401	Deamidation (NQ)
K.SQSDNFEYVAFK.T	N	72.81	1433.6462	12	0.7	717.8309	2	30.26	28	1521	OB4056.raw	6.85E3	1	1	459	470	
K.TANELN(+.98)LLILRWLGLSAEYGNLYR.N	N	67.00	2792.4861	24	-0.7	931.8353	3	40.62	28	2162	OB4056.raw	0	0	0	378	401	
R.SPDIYN(+.98)PQAGSLKTAN(+.98)ELNLLILR.W	N	64.96	2641.3962	24	0.1	881.4728	3	34.56	28	1787	OB4056.raw	1.75E4	1	1	365	388	
R.SPDIYN(+.98)PQAGSLKTANELNLLILR.W	N	64.51	2640.4121	24	0.1	881.1448	3	34.16	28	1757	OB4056.raw	2.89E4	1	1	365	388	
R.SPDIYNPQ(+.98)AGSLK.T	N	61.16	1389.6776	13	0.8	695.8466	2	27.83	28	1387	OB4056.raw	5.83E3	1	1	365	377	
K.SQ(+.98)SDNFEYVAFK.T	N	61.13	1434.6302	12	1.2	718.3232	2	30.66	28	1546	OB4056.raw	3.03E3	1	1	459	470	Deamidation (NQ)
N.GIEETIC(+57.02)TATVK.K	N	60.31	1320.6595	12	-0.8	661.3365	2	28.06	28	1396	OB4056.raw	1.41E3	1	1	346	357	Carbamidomethylation
R.NALFVPHYNTNAHSIIYALR.G	N	56.65	2313.2019	20	0.0	772.0746	3	31.12	28	1571	OB4056.raw	2.81E4	1	1	402	421	
R.RPFYSNAPQ(+.98)EIFIQQGR.G	N	56.36	2051.0225	17	0.0	684.6815	3	30.59	28	1544	OB4056.raw	0	0	0	82	98	
K.TAN(+.98)ELNLLILRWLGLSAEYGNLYR.N	N	54.66	2792.4861	24	3.7	931.8394	3	41.35	28	2207	OB4056.raw	0	0	0	378	401	
R.NALFVPHYN(+.98)TN(+.98)AHSIIYALR.G	N	52.81	2315.1699	20	-2.2	772.7289	3	32.47	28	1657	OB4056.raw	4.59E3	2	2	402	421	Deamidation (NQ)
K.TANELNLLILR.W	N	52.38	1268.7452	11	-0.2	635.3798	2	33.57	28	1747	OB4056.raw	1.21E5	1	1	378	388	
R.NALFVPHYN(+.98)TNAHSIIYALR.G	N	51.39	2314.1858	20	0.1	772.4026	3	32.45	28	1657	OB4056.raw	2.88E4	1	1	402	421	
R.NRSPDIYNPQ(+.98)AGSLKTANELNLLILR.W	N	48.74	2910.5562	26	-0.3	728.6461	4	32.87	28	1675	OB4056.raw	1.71E4	1	1	363	388	
R.NALFVPHYNTN(+.98)AHSIIYALR.G	N	46.36	2314.1858	20	-1.4	772.4015	3	32.32	28	1649	OB4056.raw	0	0	0	402	421	
K.TAN(+.98)ELN(+.98)LLILRWLGLSAEYGNLYR.N	N	45.50	2793.4700	24	7.9	932.1713	3	40.35	28	2156	OB4056.raw	3.33E4	1	1	378	401	Deamidation (NQ)
R.SPDIYNPQAGSLKTAN(+.98)ELNLLILRWLGLSAEYGNLYR.N	N	45.06	4163.1689	37	4.9	1041.8046	4	39.79	28	2159	OB4056.raw	1.74E5	1	1	365	401	
R.N(+.98)ALFVPHYN(+.98)TN(+.98)AHSIIYALR.G	N	44.58	2316.1538	20	8.0	773.0647	3	32.32	28	1649	OB4056.raw	1.28E3	1	1	402	421	Deamidation (NQ)
R.SPDIYNPQ(+.98)AGSLKTANELN(+.98)LLILRWLGLSAEYGNLYR.N	N	44.03	4164.1528	37	4.6	1042.0503	4	40.31	28	2143	OB4056.raw	0	0	0	365	401	
R.RPFYSNAPQEIFIQQGR.G	N	42.32	2050.0383	17	0.5	684.3538	3	29.96	28	1513	OB4056.raw	6.68E3	1	1	82	98	
R.SPDIYNPQ(+.98)AGSLKTANELNLLILRWLGLSAEYGNLYR.N	N	40.91	4163.1689	37	4.9	1041.8046	4	39.79	28	2072	OB4056.raw	1.74E5	1	1	365	401	
R.SPDIYNPQAGSLKTAN(+.98)ELN(+.98)LLILRWLGLSAEYGNLYR.N	N	39.84	4164.1528	37	7.8	1042.0536	4	40.28	28	2141	OB4056.raw	0	0	0	365	401	
R.SPDIYNPQAGSLKTANELN(+.98)LLILRWLGLSAEYGNLYR.N	N	39.62	4163.1689	37	4.9	1041.8046	4	39.79	28	2173	OB4056.raw	1.74E5	1	1	365	401	
K.TAN(+.98)ELNLLILR.W	N	39.07	1269.7292	11	2.8	635.8737	2	34.42	28	1776	OB4056.raw	4.16E4	1	1	378	388	Deamidation (NQ)
R.SPDIYN(+.98)PQAGSLKTANELNLLILRWLGLSAEYGNLYR.N	N	37.84	4163.1689	37	4.9	1041.8046	4	39.79	28	2161	OB4056.raw	1.74E5	1	1	365	401	

Peptide	Uniq	-10lgP	Mass	Length	ppm	m/z	z	RT	Fraction	Scan	Source File	Area Digest 6. traka 23-24 kDa	#Feature	#Feature Digest 6. traka 23-24 kDa	Start	End	PTM
R.SPDIYN(+.98)PQ(+.98)AGSLKTAN(+.98)ELN(+.98)LLILRWLGLSAEYGNLYR.N	N	37.57	4166.1211	37	7.5	1042.5454	4	40.27	28	2129	OB4056.raw	5.68E4	1	1	365	401	Deamidation (NQ)
R.NRSPDIYNPQ(+.98)AGSLKTAN(+.98)ELNLLILR.W	N	36.76	2911.5403	26	2.5	728.8942	4	33.23	28	1704	OB4056.raw	0	0	0	363	388	
R.SPDIYNPQAGSLKTANELNLLILRWLGLSAEYGNLYR.N	N	36.36	4162.1851	37	-0.1	1041.5535	4	39.31	28	2080	OB4056.raw	0	0	0	365	401	
K.TDSRPSIANLAGEN(+.98).S	N	36.29	1444.6793	14	-2.1	723.3454	2	28.25	28	1412	OB4056.raw	3.88E3	1	1	471	484	Deamidation (NQ)
K.TANELN(+.98)LLILR.W	N	36.25	1269.7292	11	4.3	635.8746	2	34.64	28	1789	OB4056.raw	4.16E4	1	1	378	388	
R.SPDIYNPQ(+.98)AGSLKTAN(+.98)ELNLLILRWLGLSAEYGNLYR.N	N	32.54	4164.1528	37	5.0	1042.0507	4	41.15	28	2195	OB4056.raw	0	0	0	365	401	
K.TDSRPSIAN(+.98)LAGEN(+.98)SVIDNLPEEVVAN(+.98)SYGLPR.E	Y	30.71	3499.6953	33	7.6	1167.5813	3	35.70	28	1857	OB4056.raw	1.17E3	1	1	471	503	Deamidation (NQ)
R.LNAQ(+.98)RPDNRIESEGGYIETWN(+.98)PNN(+.98)QEFEC(+57.02)AGVALSR.L	N	30.16	4136.8770	36	5.9	1035.2327	4	32.03	28	1632	OB4056.raw	0	0	0	37	72	Carbamidomethylation
K.TDSRPSIAN(+.98)LAGEN(+.98).S	N	28.45	1445.6633	14	0.7	723.8395	2	28.96	28	1450	OB4056.raw	6.9E2	1	1	471	484	Deamidation (NQ)
R.LNAQRPDN(+.98)RIESEGGYIETWNPNNQEFEC(+57.02)AGVALSR.L	N	26.80	4134.9087	36	-0.3	1034.7341	4	31.42	28	1595	OB4056.raw	0	0	0	37	72	Carbamidomethylation
K.TAN(+.98)ELN(+.98)LLILRWLGLSAEYGN(+.98)LYR.N	N	26.68	2794.4541	24	9.3	932.5006	3	40.82	28	2180	OB4056.raw	1.68E3	1	1	378	401	Deamidation (NQ)
K.TDSRPSIAN(+.98)LAGEN(+.98)SVIDNLPEEVVANSYGLPR.E	Y	21.65	3498.7114	33	2.1	1167.2468	3	35.75	28	1857	OB4056.raw	0	0	0	471	503	Deamidation (NQ)
R.NALFVPHYNTNAHSIIYALRGR.A	N	21.07	2526.3245	22	0.0	632.5884	4	29.97	28	1505	OB4056.raw	0	0	0	402	423	
R.NRSPDIYNPQAGSLK.T	N	19.79	1658.8376	15	1.5	553.9540	3	25.42	28	1243	OB4056.raw	1.39E4	1	1	363	377	
**total 42 peptides**																	

tr|Q8LKN1|Q8LKN1_ARAHY
back to list

| Protein Coverage | Supporting Peptides |

Protein Coverage:

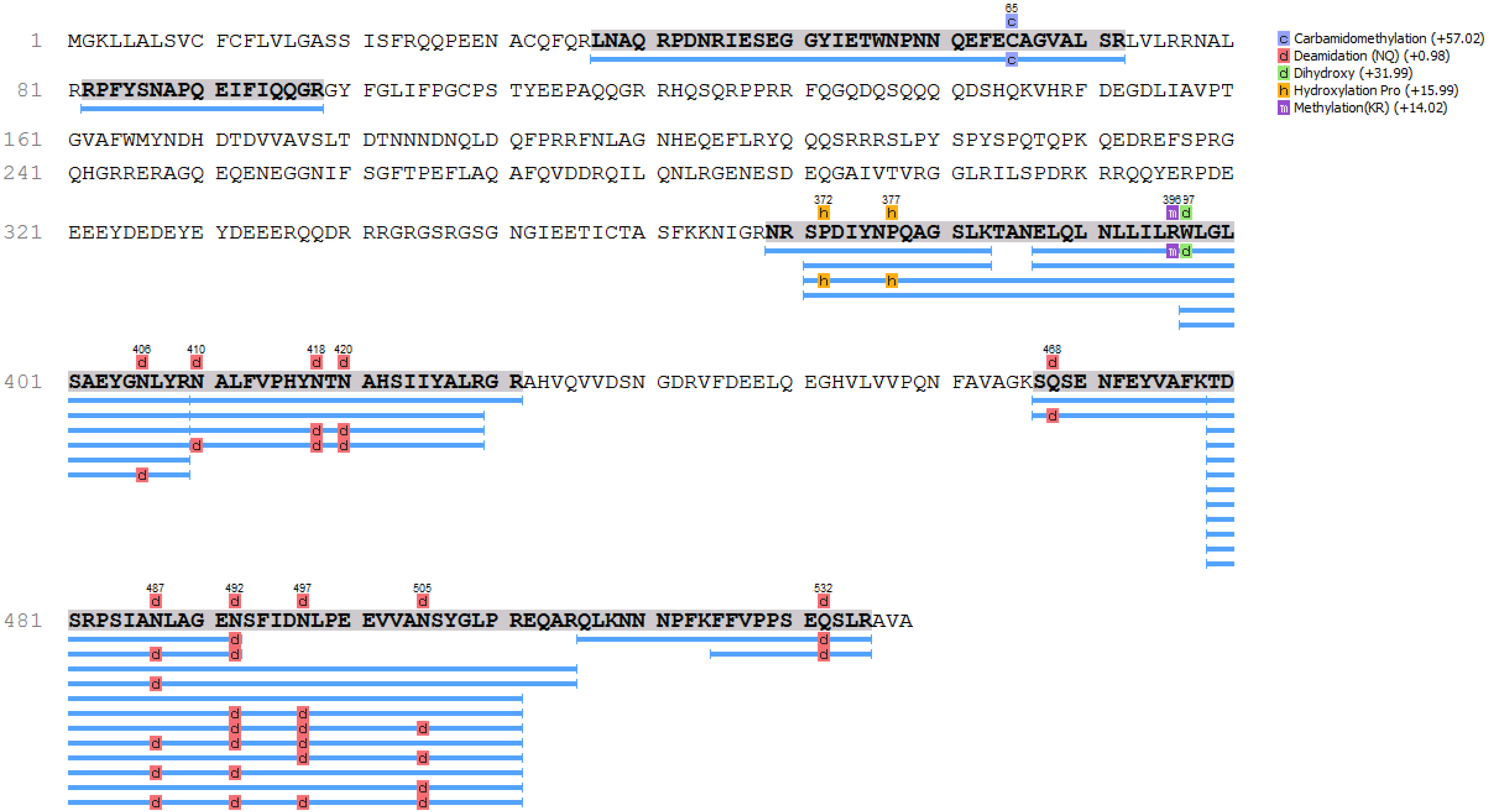

Supporting Peptides:

Peptide	Uniq	-10lgP	Mass	Length	ppm	m/z	z	RT	Fraction	Scan	Source File	Area Digest 6. traka 23-24 kDa	#Feature	#Feature Digest 6. traka 23-24 kDa	Start	End	PTM
R.WLGLSAEYGNLYR.N	N	88.77	1540.7673	13	0.7	771.3915	2	33.17	28	1701	OB4056.raw	5E5	1	1	397	409	
K.SQSENFEYVAFK.T	N	79.46	1447.6619	12	-0.2	724.8381	2	30.14	28	1512	OB4056.raw	2.33E4	2	2	467	478	
R.WLGLSAEYGN(+.98)LYR.N	N	76.21	1541.7513	13	-1.1	771.8821	2	33.52	28	1720	OB4056.raw	2.81E4	1	1	397	409	Deamidation (NQ)
K.SQ(+.98)SENFEYVAFK.T	N	71.75	1448.6459	12	-0.4	725.3299	2	30.40	28	1531	OB4056.raw	7.21E3	1	1	467	478	Deamidation (NQ)
K.TDSRPSIANLAGEN(+.98)SFIDNLPEEVVANSYGLPR.E	N	68.35	3545.7273	33	6.1	1182.9236	3	35.95	28	1865	OB4056.raw	1.52E4	1	1	479	511	
K.TDSRPSIANLAGEN(+.98)SFIDN(+.98)LPEEVVANSYGLPR.E	N	62.12	3546.7114	33	-1.1	1183.2432	3	36.13	28	1881	OB4056.raw	0	0	0	479	511	Deamidation (NQ)
R.SPDIYNPQ(+.98)AGSLK.T	N	61.16	1389.6776	13	0.8	695.8466	2	27.83	28	1387	OB4056.raw	5.83E3	1	1	371	383	
K.TDSRPSIANLAGENSFIDNLPEEVVANSYGLPR.E	N	60.87	3544.7434	33	0.3	1182.5887	3	35.78	28	1859	OB4056.raw	0	0	0	479	511	
K.TDSRPSIANLAGENSFIDN(+.98)LPEEVVANSYGLPR.E	N	59.15	3545.7273	33	6.1	1182.9236	3	35.95	28	1871	OB4056.raw	1.52E4	1	1	479	511	
K.TDSRPSIAN(+.98)LAGEN(+.98)SFIDNLPEEVVAN(+.98)SYGLPR.E	N	57.60	3547.6953	33	4.7	1183.5779	3	36.71	28	1917	OB4056.raw	0	0	0	479	511	Deamidation (NQ)
K.TDSRPSIAN(+.98)LAGEN(+.98)SFIDN(+.98)LPEEVVANSYGLPR.E	N	57.58	3547.6953	33	9.2	1183.5833	3	36.20	28	1889	OB4056.raw	5.06E4	1	1	479	511	Deamidation (NQ)
R.NALFVPHYNTNAHSIIYALR.G	N	56.65	2313.2019	20	0.0	772.0746	3	31.12	28	1571	OB4056.raw	2.81E4	1	1	410	429	
R.RPFYSNAPQ(+.98)EIFIQQGR.G	N	56.36	2051.0225	17	0.0	684.6815	3	30.59	28	1544	OB4056.raw	0	0	0	82	98	
R.NALFVPHYN(+.98)TN(+.98)AHSIIYALR.G	N	52.81	2315.1699	20	-2.2	772.7289	3	32.47	28	1657	OB4056.raw	4.59E3	2	2	410	429	Deamidation (NQ)
K.TDSRPSIAN(+.98)LAGEN(+.98)SFIDNLPEEVVANSYGLPR.E	N	52.29	3546.7114	33	4.3	1183.2495	3	36.40	28	1897	OB4056.raw	0	0	0	479	511	Deamidation (NQ)
R.NALFVPHYN(+.98)TNAHSIIYALR.G	N	51.39	2314.1858	20	0.1	772.4026	3	32.45	28	1657	OB4056.raw	2.88E4	1	1	410	429	
K.TDSRPSIANLAGEN(+.98)SFIDN(+.98)LPEEVVAN(+.98)SYGLPR.E	N	48.30	3547.6953	33	9.2	1183.5833	3	36.20	28	1881	OB4056.raw	5.06E4	1	1	479	511	Deamidation (NQ)
K.TDSRPSIANLAGEN(+.98)SFIDNLPEEVVAN(+.98)SYGLPR.E	N	47.94	3546.7114	33	4.1	1183.2493	3	36.55	28	1907	OB4056.raw	0	0	0	479	511	
R.NALFVPHYNTN(+.98)AHSIIYALR.G	N	46.36	2314.1858	20	-1.4	772.4015	3	32.32	28	1649	OB4056.raw	0	0	0	410	429	
K.TDSRPSIAN(+.98)LAGEN(+.98)SFIDN(+.98)LPEEVVAN(+.98)SYGLPR.E	N	45.98	3548.6794	33	2.9	1183.9038	3	36.86	28	1927	OB4056.raw	0	0	0	479	511	Deamidation (NQ)
K.TDSRPSIAN(+.98)LAGENSFIDN(+.98)LPEEVVANSYGLPR.E	N	45.07	3546.7114	33	4.2	1183.2494	3	36.58	28	1909	OB4056.raw	0	0	0	479	511	
R.N(+.98)ALFVPHYN(+.98)TN(+.98)AHSIIYALR.G	N	44.58	2316.1538	20	8.0	773.0647	3	32.32	28	1649	OB4056.raw	1.28E3	1	1	410	429	Deamidation (NQ)
K.TDSRPSIAN(+.98)LAGENSFIDN(+.98)LPEEVVAN(+.98)SYGLPR.E	N	44.11	3547.6953	33	9.2	1183.5833	3	36.20	28	1909	OB4056.raw	5.06E4	1	1	479	511	Deamidation (NQ)
R.RPFYSNAPQEIFIQQGR.G	N	42.32	2050.0383	17	0.5	684.3538	3	29.96	28	1513	OB4056.raw	6.68E3	1	1	82	98	
K.TDSRPSIANLAGEN(+.98)SFIDNLPEEVVANSYGLPREQAR.Q	N	36.41	4029.9668	37	6.0	1008.5050	4	34.54	28	1783	OB4056.raw	0	0	0	479	515	
K.TDSRPSIANLAGEN(+.98).S	N	36.29	1444.6793	14	-2.1	723.3454	2	28.25	28	1412	OB4056.raw	3.88E3	1	1	479	492	Deamidation (NQ)
N.ELQLNLLILR(+31.99)WLGLSAEYGNLYR.N	Y	35.25	2778.5068	23	-1.4	927.1749	3	41.62	28	2224	OB4056.raw	0	0	0	387	409	
K.TDSRPSIANLAGENSFIDN(+.98)LPEEVVANSYGLPREQAR.Q	N	34.64	4029.9668	37	0.9	1008.4999	4	34.76	28	1791	OB4056.raw	1.25E5	1	1	479	515	

Peptide	Uniq	-10lgP	Mass	Length	ppm	m/z	z	RT	Fraction	Scan	Source File	Area Digest 6. traka 23-24 kDa	#Feature	#Feature Digest 6. traka 23-24 kDa	Start	End	PTM
K.TDSRPSIANLAGEN(+.98)SFIDN(+.98)LPEEVVANSYGLPREQ(+.98)AR.Q	N	32.40	4031.9348	37	2.8	1008.9938	4	35.24	28	1826	OB4056.raw	0	0	0	479	515	
R.QLKN(+.98)N(+.98)NPFKFFVPPSEQ(+.98)SLR.A	N	31.06	2392.2063	20	0.1	798.4094	3	32.37	28	1651	OB4056.raw	2.45E3	1	1	516	535	Deamidation (NQ)
R.SPDIYNP(+15.99)QAGSLK(+14.02)TANELQLNLLILRWLGLSAEYGNLYR.N	Y	30.40	4433.3384	39	-3.5	887.6719	5	38.69	28	2055	OB4056.raw	1.14E5	1	1	371	409	
R.LNAQ(+.98)RPDNRIESEGGYIETWN(+.98)PNN(+.98)QEFEC(+57.02)AGVALSR.L	N	30.16	4136.8770	36	5.9	1035.2327	4	32.03	28	1632	OB4056.raw	0	0	0	37	72	Carbamidomethylation
N.ELQLNLLILR(+14.02)W(+31.99)LGLSAEYGNLYR.N	Y	29.56	2792.5225	23	-1.5	931.8467	3	41.02	28	2187	OB4056.raw	0	0	0	387	409	Methylation(KR); Dihydroxy
K.TDSRPSIAN(+.98)LAGEN(+.98)SFIDNLPEEVVANSYGLPREQ(+.98)AR.Q	N	29.44	4031.9348	37	2.7	1008.9937	4	35.40	28	1836	OB4056.raw	0	0	0	479	515	Deamidation (NQ)
N.ELQLN(+.98)LLILRW(+31.99)LGLSAEYGNLYR.N	Y	29.38	2779.4907	23	-2.3	927.5020	3	42.30	28	2266	OB4056.raw	0	0	0	387	409	
K.TDSRPSIAN(+.98)LAGEN(+.98).S	N	28.45	1445.6633	14	0.7	723.8395	2	28.96	28	1450	OB4056.raw	6.9E2	1	1	479	492	Deamidation (NQ)
K.FFVPPSEQ(+.98)SLR.A	N	28.34	1306.6556	11	0.7	654.3356	2	29.84	28	1497	OB4056.raw	1.45E3	1	1	525	535	Deamidation (NQ)
R.SPDIYNP(+15.99)Q(+.98)AGSLKTAN(+.98)ELQLNLLILRWLGLSAEYGNLYR.N	Y	28.19	4421.2905	39	-0.4	885.2650	5	38.76	28	2038	OB4056.raw	9.53E4	1	1	371	409	
R.LNAQRPDN(+.98)RIESEGGYIETWNPNNQEFEC(+57.02)AGVALSR.L	N	26.80	4134.9087	36	-0.3	1034.7341	4	31.42	28	1595	OB4056.raw	0	0	0	37	72	Carbamidomethylation
R.SP(+15.99)DIYNP(+15.99)QAGSLKTANELQLNLLILRWLGLSAEYGNLYR.N	Y	25.35	4435.3174	39	0.5	888.0712	5	39.67	28	2102	OB4056.raw	0	0	0	371	409	Hydroxylation Pro
K.TDSRPSIANLAGENSFIDN(+.98)LPEEVVAN(+.98)SYGLPREQ(+.98)AR.Q	N	24.24	4031.9348	37	0.3	1008.9913	4	35.64	28	1850	OB4056.raw	0	0	0	479	515	
N.ELQ(+.98)LNLLILR(+31.99)WLGLSAEYGNLYR.N	Y	23.03	2779.4907	23	4.9	927.5087	3	41.87	28	2239	OB4056.raw	0	0	0	387	409	
R.SPDIYNP(+15.99)Q(+.98)AGSLKTANELQLNLLILRWLGLSAEYGNLYR.N	Y	22.86	4420.3066	39	0.3	885.0689	5	39.79	28	2098	OB4056.raw	8.8E3	1	1	371	409	
R.SP(+15.99)DIYNPQAGSLKTANELQLN(+.98)LLILRWLGLSAEYGNLYR.N	Y	22.74	4420.3066	39	0.3	885.0689	5	39.79	28	2089	OB4056.raw	8.8E3	1	1	371	409	
K.TDSRPSIANLAGENSFIDNLPEEVVANSYGLPREQ(+.98)AR.Q	N	22.55	4029.9668	37	0.1	1008.4991	4	35.82	28	1861	OB4056.raw	0	0	0	479	515	
R.SPDIYN(+.98)P(+15.99)QAGSLKTANELQLNLLILRWLGLSAEYGNLYR.N	Y	22.52	4420.3066	39	0.3	885.0689	5	39.79	28	2114	OB4056.raw	8.8E3	1	1	371	409	
R.SPDIYNP(+15.99)QAGSLKTANELQ(+.98)LN(+.98)LLILRWLGLSAEYGNLYR.N	Y	21.61	4421.2905	39	-0.4	885.2650	5	38.76	28	2020	OB4056.raw	9.53E4	1	1	371	409	
R.SP(+15.99)DIYNPQ(+.98)AGSLKTANELQLN(+.98)LLILRWLGLSAEYGNLYR.N	Y	21.53	4421.2905	39	5.7	885.2704	5	38.18	28	1989	OB4056.raw	2.1E4	1	1	371	409	
R.NALFVPHYNTNAHSIIYALRGR.A	N	21.07	2526.3245	22	0.0	632.5884	4	29.97	28	1505	OB4056.raw	0	0	0	410	431	
R.NRSPDIYNPQAGSLK.T	N	19.79	1658.8376	15	1.5	553.9540	3	25.42	28	1243	OB4056.raw	1.39E4	1	1	369	383	
R.SP(+15.99)DIYN(+.98)PQAGSLKTANELQLNLLILRWLGLSAEYGNLYR.N	Y	19.00	4420.3066	39	-0.4	885.0682	5	37.68	28	1976	OB4056.raw	4.95E3	1	1	371	409	
**total 51 peptides**																	

tr|O82580|O82580_ARAHY

back to list

| Protein Coverage | Supporting Peptides |

Protein Coverage:

```
  1 RQQPEENACQ FQRLNAQRPD NRIESEGGYI ETWNPNNQEF ECAGVALSRL VLRRNALRRP FYSNAPQEIF IQQGRGYFGL
 81 IFPGCPRHYE EPHTQGRRSQ SQRPPRRLQG EDQSQQQRDS HQKVHRFDEG DLIAVPTGVA FWLYNDHDTD VVAVSLTDTN
161 NNDNQLDQFP RRFNLAGNTE QEFLRYQQQS RQSRRRSLPY SPYSPQSQPR QEEREFSPRG QHSRRERAGQ EEENEGGNIF
241 SGFTPEFLEQ AFQVDDRQIV QNLRGETESE EEGAIVTVRG GLRILSPDRK RRADEEEEYD EDEYEYDEED RRRGRGSRGR
321 GNGIEETICT ASAKKNIGRN RSPDIYNPQA GSLKTANDLN LLILRWLGPS AEYGNLYRNA LFVAHYNTNA HSIIYRLRGR
401 AHVQVVDSNG NRVYDEELQE GHVLVVPQNF AVAGKSQSEN FEYVAFKTDS RPSIANLAGE NSVIDNLPEE VVANSYGLQR
481 EQARQLKNNN PFKFFVPPSQ QSPRAVA
```

Modification sites: 42 c; 368 l; 372 r; 437 d; 456 d; 461 d

c Carbamidomethylation (+57.02)
d Deamidation (NQ) (+0.98)
l Levuglandinyl-lysine pyrrole adduct (+316.20)
r Replacement of proton with ammonium ion (+17.03)

Supporting Peptides:

Peptide	Uniq	-10lgP	Mass	Length	ppm	m/z	z	RT	Fraction	Scan	Source File	Area Digest 6. traka 23-24 kDa	#Feature	#Feature Digest 6. traka 23-24 kDa	Start	End	PTM
K.SQSENFEYVAFK.T	N	79.46	1447.6619	12	-0.2	724.8381	2	30.14	28	1512	OB4056.raw	2.33E4	2	2	436	447	
K.SQ(+.98)SENFEYVAFK.T	N	71.75	1448.6459	12	-0.4	725.3299	2	30.40	28	1531	OB4056.raw	7.21E3	1	1	436	447	Deamidation (NQ)
R.SPDIYNPQ(+.98)AGSLK.T	N	61.16	1389.6776	13	0.8	695.8466	2	27.83	28	1387	OB4056.raw	5.83E3	1	1	342	354	
R.RPFYSNAPQ(+.98)EIFIQQGR.G	N	56.36	2051.0225	17	0.0	684.6815	3	30.59	28	1544	OB4056.raw	0	0	0	59	75	
R.SPDIYNPQ(+.98)AGSLKTAN(+.98)DLNLLILR.W	N	55.70	2627.3806	24	-0.3	876.8005	3	34.26	28	1763	OB4056.raw	1.44E4	1	1	342	365	
R.NRSPDIYNPQ(+.98)AGSLKTANDLNLLILR.W	N	53.30	2896.5405	26	7.5	725.1478	4	32.15	28	1639	OB4056.raw	0	0	0	340	365	
R.SPDIYNPQ(+.98)AGSLKTANDLNLLILR.W	N	49.51	2626.3965	24	0.6	876.4733	3	33.87	28	1745	OB4056.raw	1.12E4	1	1	342	365	
K.TANDLNLLILR.W	N	49.10	1254.7296	11	0.0	628.3721	2	33.27	28	1702	OB4056.raw	7.58E4	1	1	355	365	
R.NRSPDIYN(+.98)PQAGSLKTAN(+.98)DLNLLILR.W	N	43.64	2897.5247	26	6.9	725.3934	4	32.47	28	1669	OB4056.raw	1.04E4	1	1	340	365	
R.RPFYSNAPQEIFIQQGR.G	N	42.32	2050.0383	17	0.5	684.3538	3	29.96	28	1513	OB4056.raw	6.68E3	1	1	59	75	

Peptide	Uniq	-10lgP	Mass	Length	ppm	m/z	z	RT	Fraction	Scan	Source File	Area Digest 6. traka 23-24 kDa	#Feature	#Feature Digest 6. traka 23-24 kDa	Start	End	PTM
K.TDSRPSIANLAGEN(+.98).S	N	36.29	1444.6793	14	-2.1	723.3454	2	28.25	28	1412	OB4056.raw	3.88E3	1	1	448	461	Deamidation (NQ)
R.WLGPSAE(+17.03)YGNLYR.N	Y	30.75	1541.7626	13	6.4	771.8935	2	33.87	28	1844	OB4056.raw	5.01E4	1	1	366	378	Replacement of proton with ammonium ion
R.LNAQ(+.98)RPDNRIESEGGYIETWN(+.98)PNN(+.98)QEFEC(+57.02)AGVALSR.L	N	30.16	4136.8770	36	5.9	1035.2327	4	32.03	28	1632	OB4056.raw	0	0	0	14	49	Carbamidomethylation
K.TDSRPSIAN(+.98)LAGEN(+.98).S	N	28.45	1445.6633	14	0.7	723.8395	2	28.96	28	1450	OB4056.raw	6.9E2	1	1	448	461	Deamidation (NQ)
L.G(+316.20)PSAEYGNLYR.N	Y	27.23	1541.7766	11	-2.7	771.8935	2	33.87	28	1743	OB4056.raw	5.01E4	1	1	368	378	Levuglandinyl-lysine pyrrole adduct
R.LNAQRPDN(+.98)RIESEGGYIETWNPNNQEFEC(+57.02)AGVALSR.L	N	26.80	4134.9087	36	-0.3	1034.7341	4	31.42	28	1595	OB4056.raw	0	0	0	14	49	Carbamidomethylation
R.N(+.98)RSPDIYNPQ(+.98)AGSLKTANDLNLLILR.W	N	25.28	2897.5247	26	-0.4	725.3881	4	32.93	28	1686	OB4056.raw	0	0	0	340	365	
R.SPDIYN(+.98)P(+15.99)QAGSLKTANDLNLLILRWLGPSAEYGNLYR.N	Y	23.98	4149.1172	37	9.5	1038.2964	4	39.72	28	2181	OB4056.raw	2.67E5	1	1	342	378	
K.TAN(+.98)DLNLLILR.W	N	20.82	1255.7136	11	1.9	628.8653	2	33.87	28	1744	OB4056.raw	3.85E4	1	1	355	365	
K.TAN(+.98)DLNLLILRWLGPSAE(+17.03)YGNLYR.N	Y	20.42	2779.4656	24	6.7	927.5020	3	42.54	28	2282	OB4056.raw	0	0	0	355	378	
R.NRSPDIYNPQAGSLK.T	N	19.79	1658.8376	15	1.5	553.9540	3	25.42	28	1243	OB4056.raw	1.39E4	1	1	340	354	
**total 21 peptides**																	

tr|Q6IWG5|Q6IWG5_ARAHY

back to list

| Protein Coverage | Supporting Peptides |

Protein Coverage:

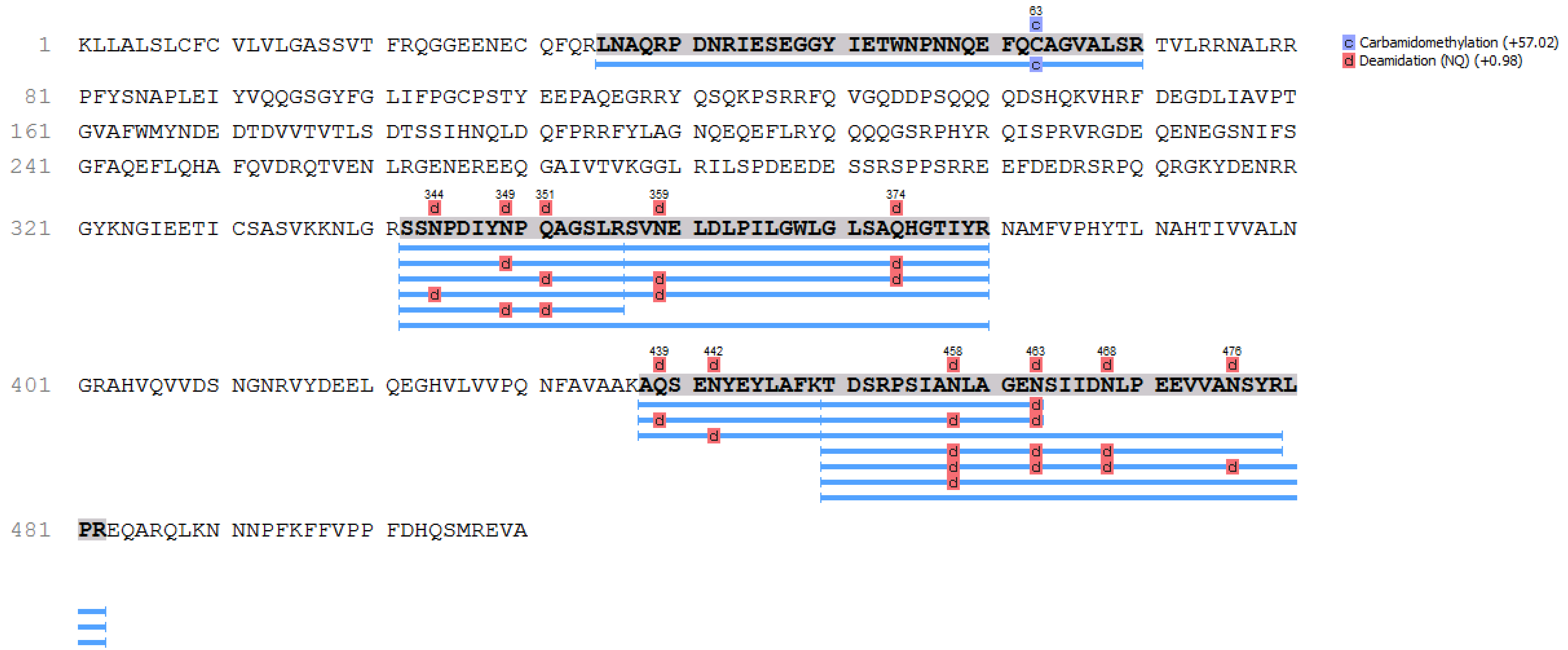

Supporting Peptides:

Peptide	Uniq	-10lgP	Mass	Length	ppm	m/z	z	RT	Fraction	Scan	Source File	Area Digest 6. traka 23-24 kDa	#Feature	#Feature Digest 6. traka 23-24 kDa	Start	End	PTM
K.AQSENYEYLAFK.T	Y	77.36	1461.6776	12	0.7	731.8466	2	30.07	28	1517	OB4056.raw	1.34E4	1	1	438	449	
K.AQ(+.98)SENYEYLAFK.T	Y	74.85	1462.6616	12	-0.2	732.3380	2	30.32	28	1529	OB4056.raw	1.48E4	1	1	438	449	Deamidation (NQ)
R.SSNPDIYNPQAGSLR.S	Y	69.84	1617.7747	15	0.1	809.8947	2	27.20	28	1355	OB4056.raw	1.07E4	1	1	342	356	

Peptide	Uniq	-10lgP	Mass	Length	ppm	m/z	z	RT	Fraction	Scan	Source File	Area Digest 6. traka 23-24 kDa	#Feature	#Feature Digest 6. traka 23-24 kDa	Start	End	PTM
R.SSNPDIYNPQ(+.98)AGSLR.S	Y	68.02	1618.7587	15	0.2	810.3868	2	27.81	28	1386	OB4056.raw	2.32E4	1	1	342	356	Deamidation (NQ)
K.TDSRPSIANLAGEN(+.98)SIIDNLPEEVVANSYR.L	Y	56.35	3244.5847	30	4.4	1082.5403	3	34.85	28	1785	OB4056.raw	2.19E4	1	1	450	479	
R.SVNELDLPILGWLGLSAQHGTIYR.N	Y	56.19	2651.4070	24	1.6	884.8110	3	38.09	28	2005	OB4056.raw	1.62E5	4	4	357	380	
R.SVN(+.98)ELDLPILGWLGLSAQ(+.98)HGTIYR.N	Y	52.54	2653.3750	24	8.2	885.4729	3	38.33	28	2038	OB4056.raw	1.8E5	1	1	357	380	Deamidation (NQ)
R.SVNELDLPILGWLGLSAQ(+.98)HGTIYR.N	Y	51.29	2652.3911	24	6.3	885.1432	3	42.33	28	2268	OB4056.raw	3E4	2	2	357	380	Deamidation (NQ)
R.SSN(+.98)PDIYNPQ(+.98)AGSLR.S	Y	50.25	1619.7427	15	0.8	810.8793	2	28.28	28	1406	OB4056.raw	1.08E4	1	1	342	356	Deamidation (NQ)
K.AQSEN(+.98)YEYLAFK.T	Y	49.79	1462.6616	12	-0.2	732.3380	2	30.32	28	1547	OB4056.raw	1.48E4	1	1	438	449	Deamidation (NQ)
R.SSNPDIYN(+.98)PQ(+.98)AGSLR.S	Y	48.92	1619.7427	15	0.8	810.8793	2	28.28	28	1420	OB4056.raw	1.08E4	1	1	342	356	Deamidation (NQ)
K.TDSRPSIAN(+.98)LAGEN(+.98)SIIDN(+.98)LPEEVVANSYR.L	Y	47.44	3246.5527	30	9.6	1083.2019	3	35.05	28	1823	OB4056.raw	1.64E4	1	1	450	479	Deamidation (NQ)
R.SSN(+.98)PDIYNPQAGSLR.S	Y	47.04	1618.7587	15	0.2	810.3868	2	27.81	28	1376	OB4056.raw	2.32E4	1	1	342	356	Deamidation (NQ)
K.TDSRPSIAN(+.98)LAGENSIIDNLPEEVVANSYR.L	Y	45.31	3244.5847	30	4.4	1082.5403	3	34.85	28	1811	OB4056.raw	2.19E4	1	1	450	479	
R.SVN(+.98)ELDLPILGWLGLSAQHGTIYR.N	Y	43.16	2652.3911	24	3.4	885.1406	3	38.64	28	2038	OB4056.raw	0	0	0	357	380	Deamidation (NQ)
R.SSNPDIYNPQAGSLRSVN(+.98)ELDLPILGWLGLSAQHGTIYR.N	Y	38.06	4252.1553	39	1.8	1064.0480	4	37.54	28	1956	OB4056.raw	4.94E5	1	1	342	380	
R.LN(+.98)AQRPDNRIESEGGYIETWNPN(+.98)NQ(+.98)EFQC(+57.02)AGVALSR.T	Y	37.64	4135.8931	36	2.5	1034.9832	4	31.47	28	1606	OB4056.raw	4.95E4	1	1	35	70	Carbamidomethylation
R.LN(+.98)AQRPDNRIESEGGYIETWNPN(+.98)NQEFQC(+57.02)AGVALSR.T	Y	37.22	4134.9087	36	-0.1	1034.7344	4	31.60	28	1606	OB4056.raw	0	0	0	35	70	Carbamidomethylation
R.LN(+.98)AQ(+.98)RPDNRIESEGGYIETWN(+.98)PNN(+.98)QEFQC(+57.02)AGVALSR.T	Y	36.48	4136.8770	36	3.1	1035.2297	4	31.92	28	1625	OB4056.raw	0	0	0	35	70	Carbamidomethylation
K.TDSRPSIANLAGEN(+.98).S	N	36.29	1444.6793	14	-2.1	723.3454	2	28.25	28	1412	OB4056.raw	3.88E3	1	1	450	463	Deamidation (NQ)
R.SSNPDIYN(+.98)PQAGSLR.S	Y	36.17	1618.7587	15	0.2	810.3868	2	27.81	28	1379	OB4056.raw	2.32E4	1	1	342	356	Deamidation (NQ)
K.TDSRPSIAN(+.98)LAGEN(+.98)SIIDNLPEEVVANSYRLPR.E	Y	34.83	3611.8066	33	0.8	903.9597	4	35.42	28	1837	OB4056.raw	0	0	0	450	482	
R.LNAQRPDNRIESEGGYIETWN(+.98)PN(+.98)NQ(+.98)EFQC(+57.02)AGVALSR.T	Y	33.19	4135.8931	36	2.5	1034.9832	4	31.47	28	1580	OB4056.raw	4.95E4	1	1	35	70	Carbamidomethylation
R.SSNPDIYNPQ(+.98)AGSLRSVN(+.98)ELDLPILGWLGLSAQHGTIYR.N	Y	32.57	4253.1392	39	2.9	1064.2952	4	37.62	28	1975	OB4056.raw	0	0	0	342	380	
R.SSNPDIYNPQAGSLRSVNELDLPILGWLGLSAQ(+.98)HGTIYR.N	Y	32.01	4252.1553	39	1.8	1064.0480	4	37.54	28	2026	OB4056.raw	4.94E5	1	1	342	380	
R.LNAQRPDNRIESEGGYIETWNPNNQ(+.98)EFQC(+57.02)AGVALSR.T	Y	29.53	4133.9248	36	3.4	1034.4919	4	31.17	28	1580	OB4056.raw	0	0	0	35	70	Carbamidomethylation
R.LN(+.98)AQRPDNRIESEGGYIETWNPN(+.98)N(+.98)Q(+.98)EFQC(+57.02)AGVALSR.T	Y	28.76	4136.8770	36	5.7	1035.2324	4	31.73	28	1614	OB4056.raw	0	0	0	35	70	Carbamidomethylation
K.TDSRPSIAN(+.98)LAGEN(+.98).S	N	28.45	1445.6633	14	0.7	723.8395	2	28.96	28	1450	OB4056.raw	6.9E2	1	1	450	463	Deamidation (NQ)
R.LN(+.98)AQRPDN(+.98)RIESEGGYIETWNPN(+.98)NQEFQC(+57.02)AGVALSR.T	Y	27.55	4135.8931	36	2.5	1034.9832	4	31.47	28	1595	OB4056.raw	4.95E4	1	1	35	70	Carbamidomethylation
K.TDSRPSIANLAGENSIIDN(+.98)LPEEVVANSYRLPR.E	Y	27.33	3610.8225	33	5.7	903.7181	4	34.76	28	1803	OB4056.raw	4.73E4	1	1	450	482	
R.SSNPDIYNPQ(+.98)AGSLRSVN(+.98)ELDLPILGWLGLSAQ(+.98)HGTIYR.N	Y	26.98	4254.1230	39	9.6	1064.5482	4	38.79	28	2048	OB4056.raw	0	0	0	342	380	

Peptide	Uniq	-10lgP	Mass	Length	ppm	m/z	z	RT	Fraction	Scan	Source File	Area Digest 6. traka 23-24 kDa	#Feature	#Feature Digest 6. traka 23-24 kDa	Start	End	PTM
K.TDSRPSIAN(+.98)LAGEN(+.98)SIIDN(+.98)LPEEVVANSYRLPR.E	Y	26.57	3612.7908	33	-0.2	904.2048	4	35.50	28	1842	OB4056.raw	0	0	0	450	482	Deamidation (NQ)
K.TDSRPSIANLAGEN(+.98)SIIDN(+.98)LPEEVVANSYRLPR.E	Y	25.95	3611.8066	33	2.6	903.9613	4	35.06	28	1815	OB4056.raw	0	0	0	450	482	
R.SVNELDLPILGW(+31.99)LGLSAQHGTIYR.N	Y	24.60	2683.3970	24	-1.1	895.4720	3	37.23	28	1950	OB4056.raw	0	0	0	357	380	
R.SSNPDIYNPQ(+.98)AGSLRSVNELDLPILGWLGLSAQHGTIYR.N	Y	21.86	4252.1553	39	1.8	1064.0480	4	37.54	28	2086	OB4056.raw	4.94E5	1	1	342	380	
R.SSNPDIYNPQ(+.98)AGSLRSVNELDLPILGWLGLSAQ(+.98)HGTIYR.N	Y	21.34	4253.1392	39	4.4	1064.2968	4	38.05	28	2002	OB4056.raw	0	0	0	342	380	
R.SSNPDIYNPQAGSLRSVNELDLPILGWLGLSAQHGTIYR.N	Y	20.83	4251.1714	39	-1.8	1063.7982	4	37.03	28	1938	OB4056.raw	0	0	0	342	380	
R.SSNPDIYN(+.98)PQ(+.98)AGSLRSVN(+.98)ELDLPILGWLGLSAQHGTIYR.N	Y	20.51	4254.1230	39	8.1	1064.5466	4	38.24	28	2014	OB4056.raw	0	0	0	342	380	
R.SSNPDIYN(+.98)PQAGSLRSVN(+.98)ELDLPILGWLGLSAQ(+.98)HGTIYR.N	Y	20.01	4254.1230	39	9.9	1064.5486	4	40.54	28	2149	OB4056.raw	1.03E4	1	1	342	380	
K.TDSRPSIAN(+.98)LAGEN(+.98)SIIDN(+.98)LPEEVVAN(+.98)SYRLPR.E	Y	19.98	3613.7747	33	9.4	904.4595	4	35.24	28	1828	OB4056.raw	2.37E4	1	1	450	482	Deamidation (NQ)
R.SSNPDIYN(+.98)PQ(+.98)AGSLRSVNELDLPILGWLGLSAQHGTIYR.N	Y	19.88	4253.1392	39	3.0	1064.2953	4	38.67	28	2040	OB4056.raw	0	0	0	342	380	
**total 41 peptides**																	

tr|Q0GM57|Q0GM57_ARAHY

back to list

| Protein Coverage | Supporting Peptides |

Protein Coverage:

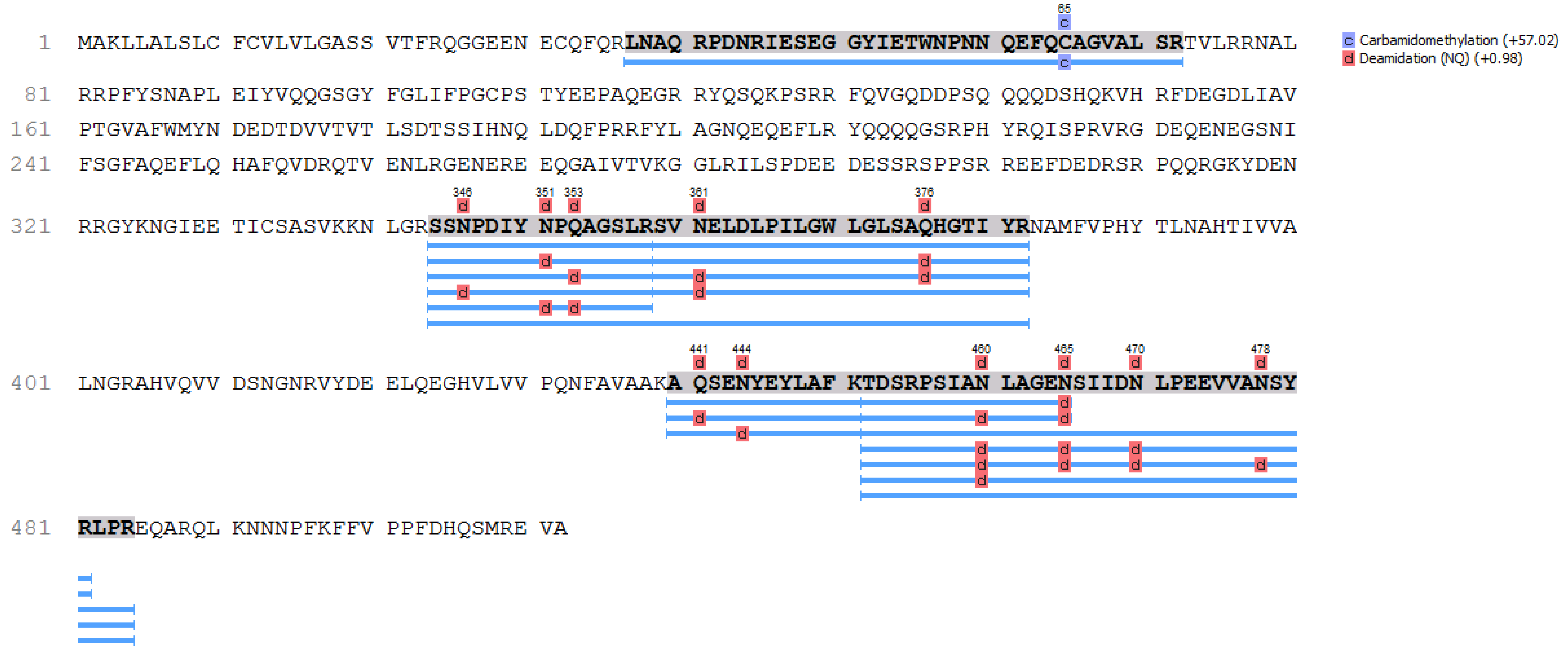

Supporting Peptides:

Peptide	Uniq	-10lgP	Mass	Length	ppm	m/z	z	RT	Fraction	Scan	Source File	Area Digest 6. traka 23-24 kDa	#Feature	#Feature Digest 6. traka 23-24 kDa	Start	End	PTM
K.AQSENYEYLAFK.T	Y	77.36	1461.6776	12	0.7	731.8466	2	30.07	28	1517	OB4056.raw	1.34E4	1	1	440	451	
K.AQ(+.98)SENYEYLAFK.T	Y	74.85	1462.6616	12	-0.2	732.3380	2	30.32	28	1529	OB4056.raw	1.48E4	1	1	440	451	Deamidation (NQ)
R.SSNPDIYNPQAGSLR.S	Y	69.84	1617.7747	15	0.1	809.8947	2	27.20	28	1355	OB4056.raw	1.07E4	1	1	344	358	

Peptide	Uniq	-10lgP	Mass	Length	ppm	m/z	z	RT	Fraction	Scan	Source File	Area Digest 6. traka 23-24 kDa	#Feature	#Feature Digest 6. traka 23-24 kDa	Start	End	PTM
R.SSNPDIYNPQ(+.98)AGSLR.S	Y	68.02	1618.7587	15	0.2	810.3868	2	27.81	28	1386	OB4056.raw	2.32E4	1	1	344	358	Deamidation (NQ)
K.TDSRPSIANLAGEN(+.98)SIIDNLPEEVVANSYR.L	Y	56.35	3244.5847	30	4.4	1082.5403	3	34.85	28	1785	OB4056.raw	2.19E4	1	1	452	481	
R.SVNELDLPILGWLGLSAQHGTIYR.N	Y	56.19	2651.4070	24	1.6	884.8110	3	38.09	28	2005	OB4056.raw	1.62E5	4	4	359	382	
R.SVN(+.98)ELDLPILGWLGLSAQ(+.98)HGTIYR.N	Y	52.54	2653.3750	24	8.2	885.4729	3	38.33	28	2038	OB4056.raw	1.8E5	1	1	359	382	Deamidation (NQ)
R.SVNELDLPILGWLGLSAQ(+.98)HGTIYR.N	Y	51.29	2652.3911	24	6.3	885.1432	3	42.33	28	2268	OB4056.raw	3E4	2	2	359	382	Deamidation (NQ)
R.SSN(+.98)PDIYNPQ(+.98)AGSLR.S	Y	50.25	1619.7427	15	0.8	810.8793	2	28.28	28	1406	OB4056.raw	1.08E4	1	1	344	358	Deamidation (NQ)
K.AQSEN(+.98)YEYLAFK.T	Y	49.79	1462.6616	12	-0.2	732.3380	2	30.32	28	1547	OB4056.raw	1.48E4	1	1	440	451	Deamidation (NQ)
R.SSNPDIYN(+.98)PQ(+.98)AGSLR.S	Y	48.92	1619.7427	15	0.8	810.8793	2	28.28	28	1420	OB4056.raw	1.08E4	1	1	344	358	Deamidation (NQ)
K.TDSRPSIAN(+.98)LAGEN(+.98)SIIDN(+.98)LPEEVVANSYR.L	Y	47.44	3246.5527	30	9.6	1083.2019	3	35.05	28	1823	OB4056.raw	1.64E4	1	1	452	481	Deamidation (NQ)
R.SSN(+.98)PDIYNPQAGSLR.S	Y	47.04	1618.7587	15	0.2	810.3868	2	27.81	28	1376	OB4056.raw	2.32E4	1	1	344	358	Deamidation (NQ)
K.TDSRPSIAN(+.98)LAGENSIIDNLPEEVVANSYR.L	Y	45.31	3244.5847	30	4.4	1082.5403	3	34.85	28	1811	OB4056.raw	2.19E4	1	1	452	481	
R.SVN(+.98)ELDLPILGWLGLSAQHGTIYR.N	Y	43.16	2652.3911	24	3.4	885.1406	3	38.64	28	2038	OB4056.raw	0	0	0	359	382	Deamidation (NQ)
R.SSNPDIYNPQAGSLRSVN(+.98)ELDLPILGWLGLSAQHGTIYR.N	Y	38.06	4252.1553	39	1.8	1064.0480	4	37.54	28	1956	OB4056.raw	4.94E5	1	1	344	382	
R.LN(+.98)AQRPDNRIESEGGYIETWNPN(+.98)NQ(+.98)EFQC(+57.02)AGVALSR.T	Y	37.64	4135.8931	36	2.5	1034.9832	4	31.47	28	1606	OB4056.raw	4.95E4	1	1	37	72	Carbamidomethylation
R.LN(+.98)AQRPDNRIESEGGYIETWNPN(+.98)NQEFQC(+57.02)AGVALSR.T	Y	37.22	4134.9087	36	-0.1	1034.7344	4	31.60	28	1606	OB4056.raw	0	0	0	37	72	Carbamidomethylation
R.LN(+.98)AQ(+.98)RPDNRIESEGGYIETWN(+.98)PNN(+.98)QEFQC(+57.02)AGVALSR.T	Y	36.48	4136.8770	36	3.1	1035.2297	4	31.92	28	1625	OB4056.raw	0	0	0	37	72	Carbamidomethylation
K.TDSRPSIANLAGEN(+.98).S	N	36.29	1444.6793	14	-2.1	723.3454	2	28.25	28	1412	OB4056.raw	3.88E3	1	1	452	465	Deamidation (NQ)
R.SSNPDIYN(+.98)PQAGSLR.S	Y	36.17	1618.7587	15	0.2	810.3868	2	27.81	28	1379	OB4056.raw	2.32E4	1	1	344	358	Deamidation (NQ)
K.TDSRPSIAN(+.98)LAGEN(+.98)SIIDNLPEEVVANSYRLPR.E	Y	34.83	3611.8066	33	0.8	903.9597	4	35.42	28	1837	OB4056.raw	0	0	0	452	484	
R.LNAQRPDNRIESEGGYIETWN(+.98)PN(+.98)NQ(+.98)EFQC(+57.02)AGVALSR.T	Y	33.19	4135.8931	36	2.5	1034.9832	4	31.47	28	1580	OB4056.raw	4.95E4	1	1	37	72	Carbamidomethylation
R.SSNPDIYNPQ(+.98)AGSLRSVN(+.98)ELDLPILGWLGLSAQHGTIYR.N	Y	32.57	4253.1392	39	2.9	1064.2952	4	37.62	28	1975	OB4056.raw	0	0	0	344	382	
R.SSNPDIYNPQAGSLRSVNELDLPILGWLGLSAQ(+.98)HGTIYR.N	Y	32.01	4252.1553	39	1.8	1064.0480	4	37.54	28	2026	OB4056.raw	4.94E5	1	1	344	382	
R.LNAQRPDNRIESEGGYIETWNPNNQ(+.98)EFQC(+57.02)AGVALSR.T	Y	29.53	4133.9248	36	3.4	1034.4919	4	31.17	28	1580	OB4056.raw	0	0	0	37	72	Carbamidomethylation
R.LN(+.98)AQRPDNRIESEGGYIETWNPN(+.98)N(+.98)Q(+.98)EFQC(+57.02)AGVALSR.T	Y	28.76	4136.8770	36	5.7	1035.2324	4	31.73	28	1614	OB4056.raw	0	0	0	37	72	Carbamidomethylation
K.TDSRPSIAN(+.98)LAGEN(+.98).S	N	28.45	1445.6633	14	0.7	723.8395	2	28.96	28	1450	OB4056.raw	6.9E2	1	1	452	465	Deamidation (NQ)
R.LN(+.98)AQRPDN(+.98)RIESEGGYIETWNPN(+.98)NQEFQC(+57.02)AGVALSR.T	Y	27.55	4135.8931	36	2.5	1034.9832	4	31.47	28	1595	OB4056.raw	4.95E4	1	1	37	72	Carbamidomethylation
K.TDSRPSIANLAGENSIIDN(+.98)LPEEVVANSYRLPR.E	Y	27.33	3610.8225	33	5.7	903.7181	4	34.76	28	1803	OB4056.raw	4.73E4	1	1	452	484	
R.SSNPDIYNPQ(+.98)AGSLRSVN(+.98)ELDLPILGWLGLSAQ(+.98)HGTIYR.N	Y	26.98	4254.1230	39	9.6	1064.5482	4	38.79	28	2048	OB4056.raw	0	0	0	344	382	

Peptide	Uniq	-10lgP	Mass	Length	ppm	m/z	z	RT	Fraction	Scan	Source File	Area Digest 6. traka 23-24 kDa	#Feature	#Feature Digest 6. traka 23-24 kDa	Start	End	PTM
K.TDSRPSIAN(+.98)LAGEN(+.98)SIIDN(+.98)LPEEVVANSYRLPR.E	Y	26.57	3612.7908	33	-0.2	904.2048	4	35.50	28	1842	OB4056.raw	0	0	0	452	484	Deamidation (NQ)
K.TDSRPSIANLAGEN(+.98)SIIDN(+.98)LPEEVVANSYRLPR.E	Y	25.95	3611.8066	33	2.6	903.9613	4	35.06	28	1815	OB4056.raw	0	0	0	452	484	
R.SVNELDLPILGW(+31.99)LGLSAQHGTIYR.N	Y	24.60	2683.3970	24	-1.1	895.4720	3	37.23	28	1950	OB4056.raw	0	0	0	359	382	
R.SSNPDIYNPQ(+.98)AGSLRSVNELDLPILGWLGLSAQHGTIYR.N	Y	21.86	4252.1553	39	1.8	1064.0480	4	37.54	28	2086	OB4056.raw	4.94E5	1	1	344	382	
R.SSNPDIYNPQ(+.98)AGSLRSVNELDLPILGWLGLSAQ(+.98)HGTIYR.N	Y	21.34	4253.1392	39	4.4	1064.2968	4	38.05	28	2002	OB4056.raw	0	0	0	344	382	
R.SSNPDIYNPQAGSLRSVNELDLPILGWLGLSAQHGTIYR.N	Y	20.83	4251.1714	39	-1.8	1063.7982	4	37.03	28	1938	OB4056.raw	0	0	0	344	382	
R.SSNPDIYN(+.98)PQ(+.98)AGSLRSVN(+.98)ELDLPILGWLGLSAQHGTIYR.N	Y	20.51	4254.1230	39	8.1	1064.5466	4	38.24	28	2014	OB4056.raw	0	0	0	344	382	
R.SSNPDIYN(+.98)PQAGSLRSVN(+.98)ELDLPILGWLGLSAQ(+.98)HGTIYR.N	Y	20.01	4254.1230	39	9.9	1064.5486	4	40.54	28	2149	OB4056.raw	1.03E4	1	1	344	382	
K.TDSRPSIAN(+.98)LAGEN(+.98)SIIDN(+.98)LPEEVVAN(+.98)SYRLPR.E	Y	19.98	3613.7747	33	9.4	904.4595	4	35.24	28	1828	OB4056.raw	2.37E4	1	1	452	484	Deamidation (NQ)
R.SSNPDIYN(+.98)PQ(+.98)AGSLRSVNELDLPILGWLGLSAQHGTIYR.N	Y	19.88	4253.1392	39	3.0	1064.2953	4	38.67	28	2040	OB4056.raw	0	0	0	344	382	
**total 41 peptides**																	

tr|Q6PSU4|Q6PSU4_ARAHY

back to list

| Protein Coverage | Supporting Peptides |

Protein Coverage:

```
  1 GFDQRSRQFQ NLQNHRIVQI EAKPNTLVLP KHADADNILV IQQGQATVTV ANGNNRKSFN LDEGHALRIP SGFISYILNR
 81 HDNQNLRVAK ISMPVNTPGQ FEDFFPASSR DQSSYLQGFS RNTLEAAFNA EFNEIRRVLL EENAGGEQEE RGQRRWSTRS
161 SENNEGVIVK VSKEHVEELT KHAKSVSKKG SEEGDITNPI NLREGEPDLS NNFGKLFEVK PDKKNPQLQD LDMMLTCVEI
241 KEGALMLPHF NSKAMVIVVV NKGTGNLELV AVRKEQQQRG RREEEDEDE EEEGSNREVR RYTARLKEGD VFIMPAAHPV
321 AINASSELHL LGFGINAENN HRIFLAGDKD NVIDQIEKQA KDLAFPGSGE QVEKLIKNQK ESHFVSARPQ SQSQSPSSPE
401 KESPEKEDQE EENQGGKGPL LSILKAFN
```

Modification sites marked: 19 d, 25 d; 93 o, 96 d, 100 d, 112 d, 117 d

d Deamidation (NQ) (+0.98)
o Oxidation (M) (+15.99)

Supporting Peptides:

Peptide	Uniq	-10lgP	Mass	Length	ppm	m/z	z	RT	Fraction	Scan	Source File	Area Digest 6. traka 23-24 kDa	#Feature	#Feature Digest 6. traka 23-24 kDa	Start	End	PTM
R.IPSGFISYILNR.H	Y	54.33	1378.7609	12	-2.1	690.3862	2	35.23	28	1830	OB4056.raw	5.9E3	1	1	69	80	
R.NTLEAAFNAEFN(+.98)EIRR.V	Y	38.53	1894.9172	16	-1.2	948.4647	2	33.37	28	1716	OB4056.raw	3.4E3	1	1	122	137	
K.ISM(+15.99)PVNTPGQ(+.98)FEDFFPA.S	Y	36.99	1912.8552	17	2.6	957.4374	2	36.07	28	1878	OB4056.raw	2.6E3	1	1	91	107	Oxidation (M); Deamidation (NQ)
K.ISM(+15.99)PVNTPGQ(+.98)FEDFFPASSRDQ(+.98)SSYLQ(+.98)GFSR.N	Y	26.07	3513.5669	31	6.0	1172.2032	3	34.06	28	1758	OB4056.raw	4.91E4	1	1	91	121	Oxidation (M)
K.ISM(+15.99)PVN(+.98)TPGQ(+.98)FEDFFPASSRDQ(+.98)SSYLQ(+.98)GFSR.N	Y	25.69	3514.5510	31	4.1	1172.5291	3	34.50	28	1781	OB4056.raw	0	0	0	91	121	Oxidation (M); Deamidation (NQ)
K.ISM(+15.99)PVN(+.98)TPGQ(+.98)FEDFFPASSRDQSSYLQ(+.98)GFSR.N	Y	23.89	3513.5669	31	6.0	1172.2032	3	34.06	28	1774	OB4056.raw	4.91E4	1	1	91	121	Oxidation (M)
R.IVQ(+.98)IEAKPN(+.98)TLVLPK.H	Y	21.84	1663.9760	15	-1.4	555.6652	3	29.67	28	1486	OB4056.raw	1.4E4	1	1	17	31	Deamidation (NQ)
**total 7 peptides**																	

tr|N1NG13|N1NG13_ARAHY
back to list

| Protein Coverage | Supporting Peptides |

Protein Coverage:

d Deamidation (NQ) (+0.98)
o Oxidation (M) (+15.99)

```
1   MRGRVSPLML LLGILVLASV SATHAKSSPY QKKTENPCAQ RCLQSCQQEP DDLKQKACES RCTKLEYDPR CVYDPRGHTG
81  TTNQRSPPGE RTRGRQPGDY DDDRRQPRRE EGGRWGPAGP REREREEDWR QPREDWRRPS HQQPRKIRPE GREGEQEWGT
161 PGSHVREETS RNNPFYFPSR RFSTRYGNQN GRIRVLQRFD QRSRQFQNLQ NHRIVQIEAK PNTLVLPKHA DADNILVIQQ
241 GQATVTVANG NNRKSFNLDE GHALRIPSGF ISYILNRHDN QNLRVAKISM PVNTPGQFED FFPASSRDQS SYLQGFSRNT
321 LEAAFNAEFN EIRRVLLEEN AGGEQEERGQ RRWSTRSSEN NEGVIVKVSK EHVEELTKHA KSVSKKGSEE EGDITNPINL
401 REGEPDLSNN FGKLFEVKPD KKNPQLQDLD MMLTCVEIKE GALMLPHFNS KAMVIVVVNK GTGNLELVAV RKEQQQRGRR
481 EEEEDEDEEE EGSNREVRRY TARLKEGDVF IMPAAHPVAI NASSELHLLG FGINAENNHR IFLAGDKDNV IDQIEKQAKD
561 LAFPGSGEQV EKLIKNQKES HFVSARPQSQ SQSPSSPEKE SPEKEDQEEE NQGGKGPLLS ILKAFN
```

Supporting Peptides:

Peptide	Uniq	-10lgP	Mass	Length	ppm	m/z	z	RT	Fraction	Scan	Source File	Area Digest 6. traka 23-24 kDa	#Feature	#Feature Digest 6. traka 23-24 kDa	Start	End	PTM
R.IPSGFISYILNR.H	Y	54.33	1378.7609	12	-2.1	690.3862	2	35.23	28	1830	OB4056.raw	5.9E3	1	1	266	277	
R.NTLEAAFNAEFN(+.98)EIRR.V	Y	38.53	1894.9172	16	-1.2	948.4647	2	33.37	28	1716	OB4056.raw	3.4E3	1	1	319	334	
K.ISM(+15.99)PVNTPGQ(+.98)FEDFFPA.S	Y	36.99	1912.8552	17	2.6	957.4374	2	36.07	28	1878	OB4056.raw	2.6E3	1	1	288	304	Oxidation (M); Deamidation (NQ)
K.ISM(+15.99)PVNTPGQ(+.98)FEDFFPASSRDQ(+.98)SSYLQ(+.98)GFSR.N	Y	26.07	3513.5669	31	6.0	1172.2032	3	34.06	28	1758	OB4056.raw	4.91E4	1	1	288	318	Oxidation (M)
K.ISM(+15.99)PVN(+.98)TPGQ(+.98)FEDFFPASSRDQ(+.98)SSYLQ(+.98)GFSR.N	Y	25.69	3514.5510	31	4.1	1172.5291	3	34.50	28	1781	OB4056.raw	0	0	0	288	318	Oxidation (M); Deamidation (NQ)
K.ISM(+15.99)PVN(+.98)TPGQ(+.98)FEDFFPASSRDQSSYLQ(+.98)GFSR.N	Y	23.89	3513.5669	31	6.0	1172.2032	3	34.06	28	1774	OB4056.raw	4.91E4	1	1	288	318	Oxidation (M)
R.IVQ(+.98)IEAKPN(+.98)TLVLPK.H	Y	21.84	1663.9760	15	-1.4	555.6652	3	29.67	28	1486	OB4056.raw	1.4E4	1	1	214	228	Deamidation (NQ)
**total 7 peptides**																	

P43238|ALL12_ARAHY

back to list

| **Protein Coverage** | **Supporting Peptides** |

Protein Coverage:

d Deamidation (NQ) (+0.98)
o Oxidation (M) (+15.99)

```
  1  MRGRVSPLML LLGILVLASV SATHAKSSPY QKKTENPCAQ RCLQSCQQEP DDLKQKACES RCTKLEYDPR CVYDPRGHTG
 81  TTNQRSPPGE RTRGRQPGDY DDDRRQPRRE EGGRWGPAGP REREREEDWR QPREDWRRPS HQQPRKIRPE GREGEQEWGT
161  PGSHVREETS RNNPFYFPSR RFSTRYGNQN GRIRVLQRFD QRSRQFQNLQ NHRIVQIEAK PNTLVLPKHA DADNILVIQQ
241  GQATVTVANG NNRKSFNLDE GHALRIPSGF ISYILNRHDN QNLRVAKISM PVNTPGQFED FFPASSRDQS SYLQGFSRNT
321  LEAAFNAEFN EIRRVLLEEN AGGEQEERGQ RRWSTRSSEN NEGVIVKVSK EHVEELTKHA KSVSKKGSEE EGDITNPINL
401  REGEPDLSNN FGKLFEVKPD KKNPQLQDLD MMLTCVEIKE GALMLPHFNS KAMVIVVVNK GTGNLELVAV RKEQQQRGRR
481  EEEEDEDEEE EGSNREVRRY TARLKEGDVF IMPAAHPVAI NASSELHLLG FGINAENNHR IFLAGDKDNV IDQIEKQAKD
561  LAFPGSGEQV EKLIKNQKES HFVSARPQSQ SQSPSSPEKE SPEKEDQEEE NQGGKGPLLS ILKAFN
```

Supporting Peptides:

Peptide	Uniq	-10lgP	Mass	Length	ppm	m/z	z	RT	Fraction	Scan	Source File	Area Digest 6. traka 23-24 kDa	#Feature	#Feature Digest 6. traka 23-24 kDa	Start	End	PTM
R.IPSGFISYILNR.H	Y	54.33	1378.7609	12	-2.1	690.3862	2	35.23	28	1830	OB4056.raw	5.9E3	1	1	266	277	
R.NTLEAAFNAEFN(+.98)EIRR.V	Y	38.53	1894.9172	16	-1.2	948.4647	2	33.37	28	1716	OB4056.raw	3.4E3	1	1	319	334	
K.ISM(+15.99)PVNTPGQ(+.98)FEDFFPA.S	Y	36.99	1912.8552	17	2.6	957.4374	2	36.07	28	1878	OB4056.raw	2.6E3	1	1	288	304	Oxidation (M); Deamidation (NQ)
K.ISM(+15.99)PVNTPGQ(+.98)FEDFFPASSRDQ(+.98)SSYLQ(+.98)GFSR.N	Y	26.07	3513.5669	31	6.0	1172.2032	3	34.06	28	1758	OB4056.raw	4.91E4	1	1	288	318	Oxidation (M)
K.ISM(+15.99)PVN(+.98)TPGQ(+.98)FEDFFPASSRDQ(+.98)SSYLQ(+.98)GFSR.N	Y	25.69	3514.5510	31	4.1	1172.5291	3	34.50	28	1781	OB4056.raw	0	0	0	288	318	Oxidation (M); Deamidation (NQ)
K.ISM(+15.99)PVN(+.98)TPGQ(+.98)FEDFFPASSRDQSSYLQ(+.98)GFSR.N	Y	23.89	3513.5669	31	6.0	1172.2032	3	34.06	28	1774	OB4056.raw	4.91E4	1	1	288	318	Oxidation (M)

Peptide	Uniq	-10lgP	Mass	Length	ppm	m/z	z	RT	Fraction	Scan	Source File	Area Digest 6. traka 23-24 kDa	#Feature	#Feature Digest 6. traka 23-24 kDa	Start	End	PTM
R.IVQ(+.98)IEAKPN(+.98)TLVLPK.H	Y	21.84	1663.9760	15	-1.4	555.6652	3	29.67	28	1486	OB4056.raw	1.4E4	1	1	214	228	Deamidation (NQ)
**total 7 peptides**																	

Peptide List

Prepared with PEAKS ™ (bioinfor.com)

1. Notes Gastric Control Raw peanut Band #7 24-27 kDa (PTM446)

2. Result Statistics

Figure 1. False discovery rate (FDR) curve. X axis is the number of peptide-spectrum matches (PSM) being kept. Y axis is the corresponding FDR.

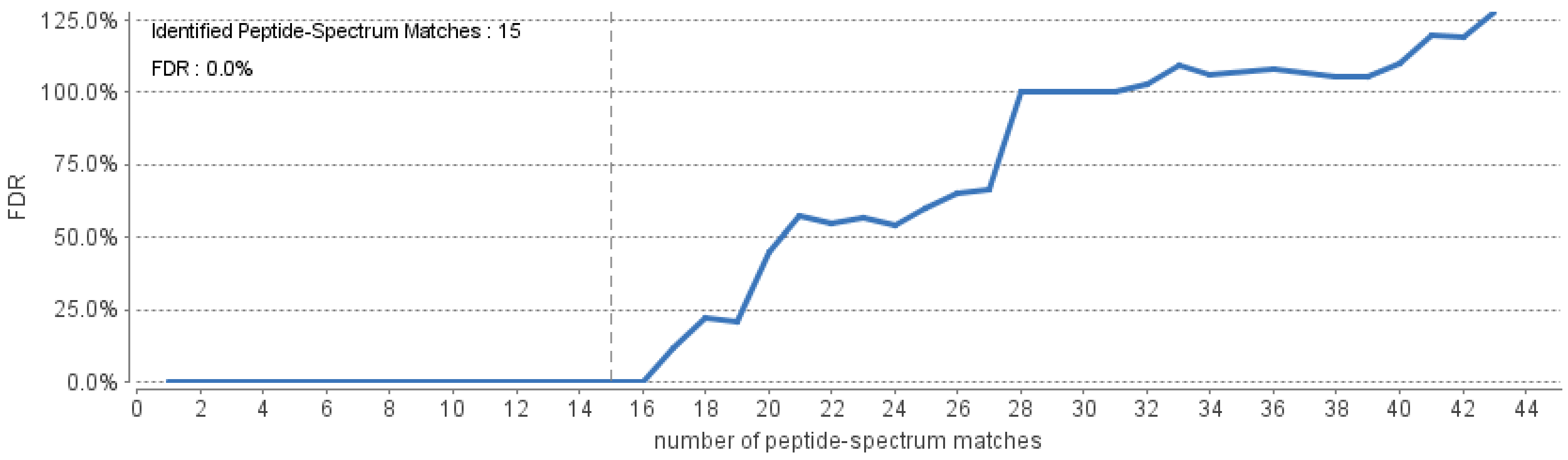

Figure 2. PSM score distribution. **(a)** Distribution of PEAKS peptide score; **(b)** Scatterplot of PEAKS peptide score versus precursor mass error.

(a)

(b)

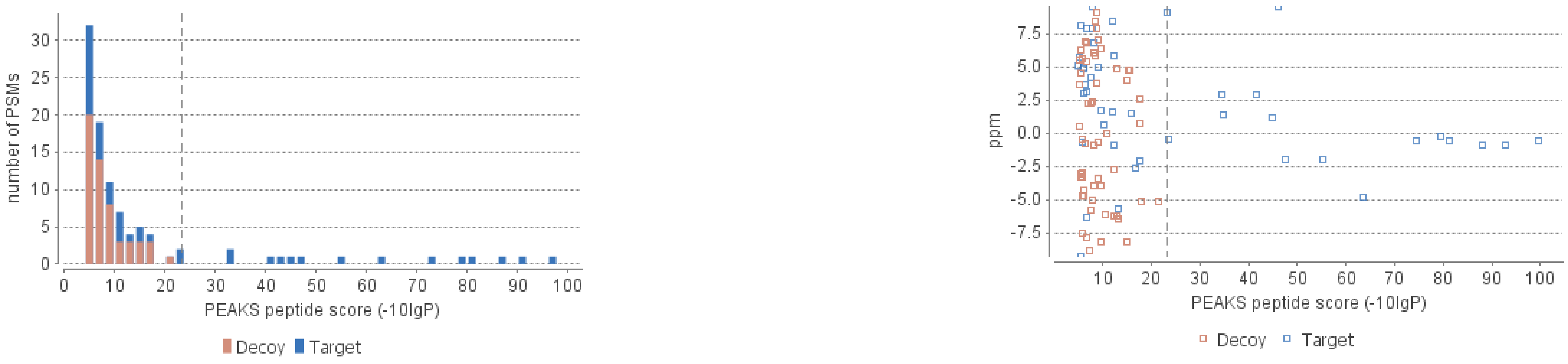

Figure 3. Distribution of peptide feature detection. **(a)** Feature m/z distribution; **(b)** Feature RT distribution

(a) **(b)**

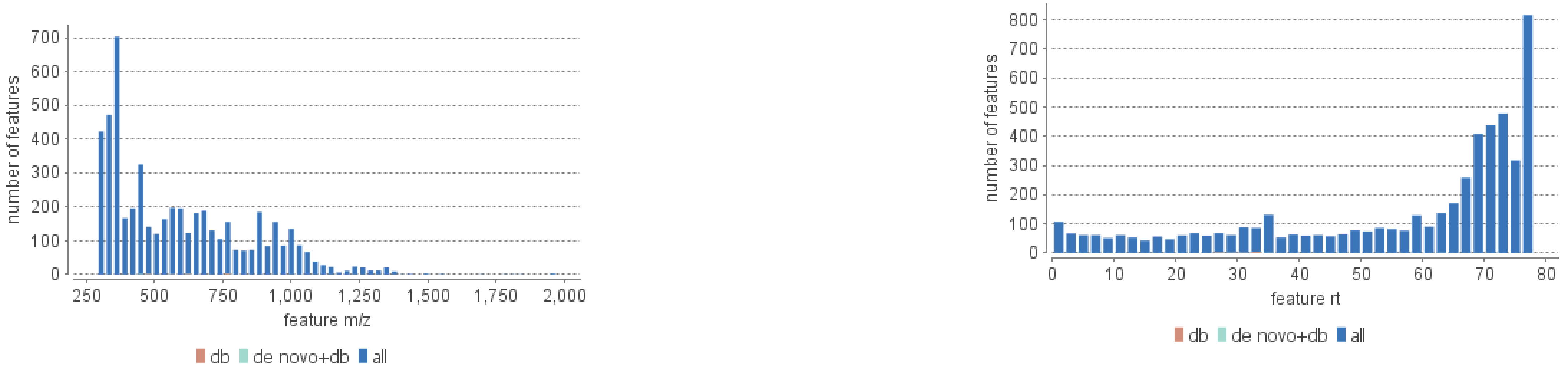

Figure 4. Distribution of identified peptide features. **(a)** Feature abundance distribution **(b)** *De novo* sequencing validation.

(a)

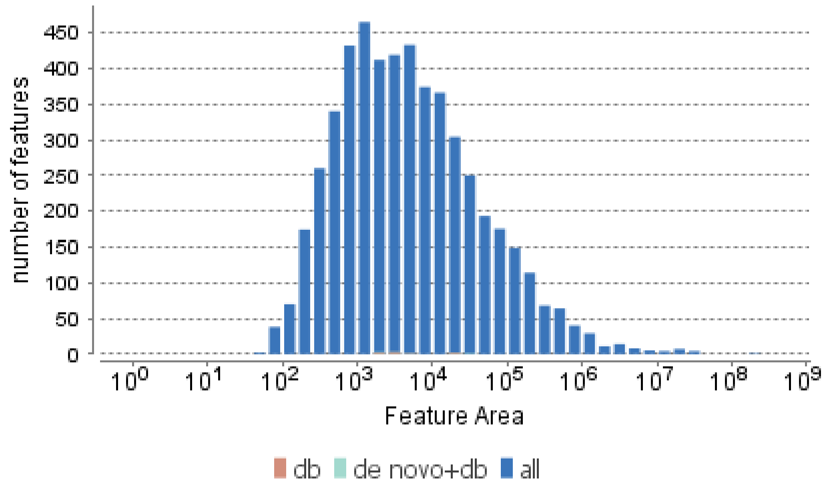

(b)

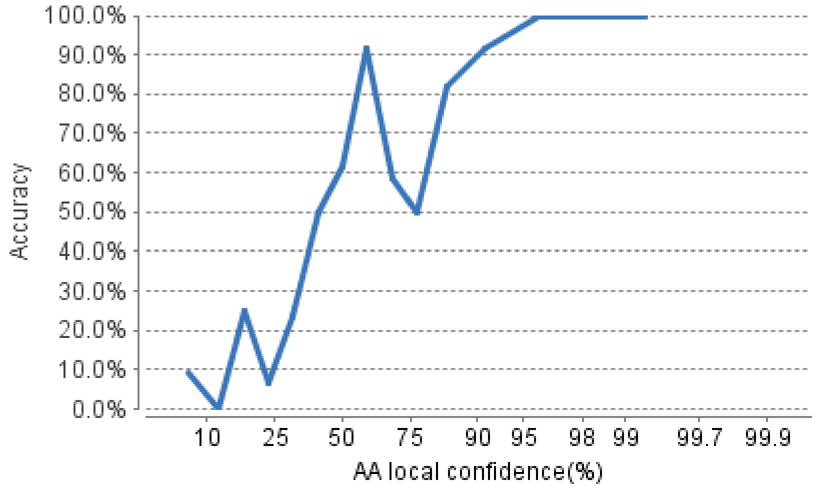

Table 1. Statistics of data.

# of MS scans	3286
# of MS/MS scans	679
# of Features	5225
# of Chimera scans	23

Table 2. Result filtration parameters.

Peptide -10lgP	≥23.4
PTM Ascore	≥20
Protein -10lgP	≥20
Proteins unique peptides	≥1
De novo ALC Score	≥50%

Table 4. PTM profile.

Name	ΔMass	Position	#PSM	-10lgP	Abundance	AScore
Deamidation	.98	N	1	63.52		1000.00

Table 3. Statistics of filtered result.

Peptide-Spectrum Matches	15
Peptide sequences	8
Protein groups	2
Proteins	7
Proteins (#Unique Peptides)	0 (>2); 0 (=2); 7 (=1);
FDR (Peptide-Spectrum Matches)	0.0%
FDR (Peptide Sequences)	0.0%
FDR (Protein Group)	0.0%
De Novo Only Spectra	2
# of identified Features	10
# of identified MS/MS scans	15

3. Experiment Control

Figure 5. Precursor mass error of peptide-spectrum matches (PSM) in filtered result. **(a)** Distribution of precursor mass error in ppm; **(b)** Scatterplot of precursor m/z versus precursor mass error in ppm.

(a)

(b)

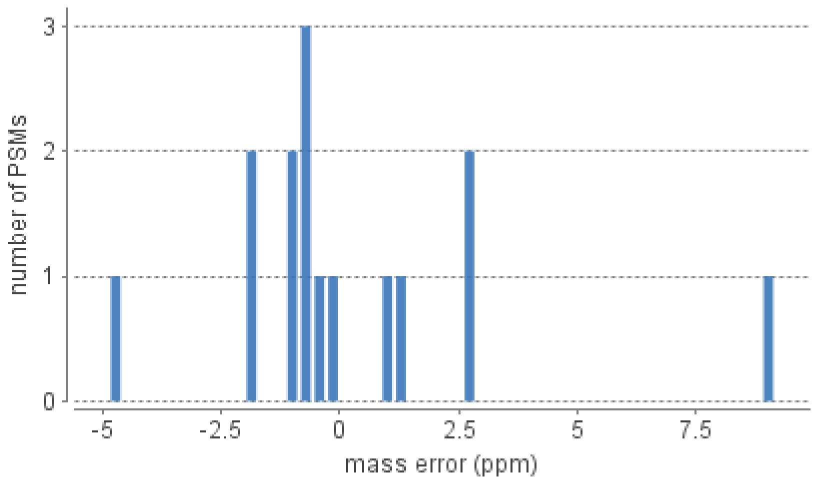

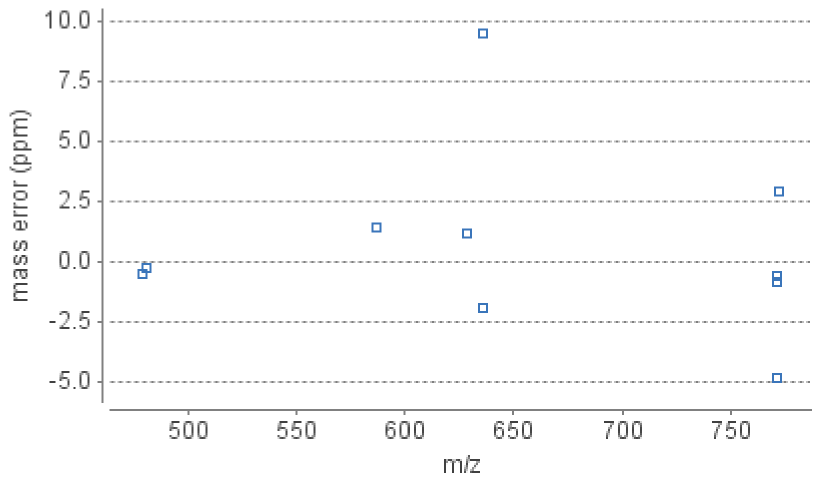

Table 5. Number of identified peptides in each sample by the number of missed cleavages

Missed Cleavages	0	1	2	3	4+
KOntrola 7. traka 25 kDa	6	1	1	0	0

4. Other Information

Table 6. Search parameters.

Search Engine Name: PEAKS
Parent Mass Error Tolerance: 10.0 ppm
Fragment Mass Error Tolerance: 0.5 Da
Precursor Mass Search Type: monoisotopic
Enzyme: Trypsin
Max Missed Cleavages: 2
Digest Mode: Unspecific

Table 7. Instrument parameters.
Fractions: OB4036.raw
Ion Source: ESI(nano-spray)
Fragmentation Mode: CID, CAD(y and b ions)
MS Scan Mode: FT-ICR/Orbitrap
MS/MS Scan Mode: Linear Ion Trap

Fixed Modifications:
  Carbamidomethylation: 57.02
Variable Modifications:
  Deamidation (NQ): 0.98
  Oxidation (M): 15.99
  Hydroxylation Pro: 15.99
  Acetylation (K): 42.01
  Acetylation (Protein N-term): 42.01
  Acetylation (N-term): 42.01
  Amidation: -0.98
  Beta-methylthiolation: 45.99
  and 305 more...
Max Variable PTM Per Peptide: 5
Database: Uniprot_Peanut-3818_Jul18
Taxon: All
Contaminant Database: contaminantsMQ_mar19
Searched Entry: 1723
FDR Estimation: Enabled
De novo score (ALC%) threshold: 15
Peptide hit threshold (-10logP): 30.0
Peaks run ID: 432
Merge Options: no merge
Precursor Options: corrected
Charge Options: no correction
Filter Charge: 2 - 8
Process: true
Associate chimera: yes

Protein List

Protein Accession Contains:
Protein Description Contains:
Peptide Sample Area >=
Protein Ptm Contains:

Protein Group	Protein ID	Accession	-10lgP	Coverage (%)	Coverage (%) KOntrola 7. traka 25 kDa	Area KOntrola 7. traka 25 kDa	#Peptides	#Unique	#Spec KOntrola 7. traka 25 kDa	PTM	Avg. Mass	Description
**1**	20574	tr|A1DZF0|A1DZF0_ARAHY	141.15	8	8	2.24E3	3	1	11	Y	60375	Arachin 6 OS=Arachis hypogaea OX=3818 PE=2 SV=1
**1**	20578	tr|B5TYU1|B5TYU1_ARAHY	141.15	8	8	2.24E3	3	1	11	Y	60624	Arachin Arah3 isoform OS=Arachis hypogaea OX=3818 PE=1 SV=1
**1**	20580	tr|Q647H4|Q647H4_ARAHY	141.15	8	8	2.24E3	3	1	11	Y	61506	Arachin Ahy-1 OS=Arachis hypogaea OX=3818 PE=2 SV=1
**1**	20591	tr|Q6T2T4|Q6T2T4_ARAHY	141.15	8	8	2.24E3	3	1	11	Y	61499	Storage protein OS=Arachis hypogaea OX=3818 PE=2 SV=1
**2**	20576	tr|Q9FZ11|Q9FZ11_ARAHY	136.05	8	8	3.77E3	3	1	9	Y	60449	Gly1 OS=Arachis hypogaea OX=3818 GN=Gly1 PE=2 SV=1
**2**	20579	tr|Q5I6T2|Q5I6T2_ARAHY	136.05	8	8	3.77E3	3	1	9	Y	60736	Arachin Ahy-4 OS=Arachis hypogaea OX=3818 PE=2 SV=1
**2**	20581	tr|Q647H3|Q647H3_ARAHY	136.05	8	8	3.77E3	3	1	9	Y	61532	Arachin Ahy-2 OS=Arachis hypogaea OX=3818 PE=2 SV=1
**total 7 proteins**												

tr|A1DZF0|A1DZF0_ARAHY

back to list

| Protein Coverage | Supporting Peptides |

Protein Coverage:

```
  1  MAKLLELSFC FCFLVLGASS ISFRQQPEEN ACQFQRLNAQ RPDNRIESEG GYIETWNPNN QEFECAGVAL SRLVLRRNAL
 81  RRPFYSNAPQ EIFIQQGRGY FGLIFPGCPS TYEEPAQQGR RYQSQRPPRR LQEEDQSQQQ QDSHQKVHRF NEGDLIAVPT
161  GVAFWLYNDH DTDVVAVSLT DTNNNDNQLD QFPRRFNLAG NHEQEFLRYQ QQSRQSRRRS LPLSPYSPQP GQEDREFSPQ
241  GQHGRRERAG QEQENEGGNI FSGFTSEFLA QAFQVDDRQI VQNLRGENES EEQGAIVTVK GGLRILSPDR KSPDEEEEYD
321  EDEYAEEERQ QDRRRGRGSR GSGNGIEETI CTATVKKNIG RNRSPDIYNP QAGSLKTANE LNLLILRWLG LSAEYGNLYR
401  NALFVPHYNT NAHSIIYALR GRAHVQVVDS NGNRVYDEEL QEGHVLVVPQ NFAVAGKSQS ENFEYVAFKT DSRPSIANLA
481  GENSFIDNLP EEVVANSYGL PREQARQLKN NNPFKFFVPP FQQSPRAVA
```

d Deamidation (NQ) (+0.98)

Supporting Peptides:

Peptide	Uniq	-10lgP	Mass	Length	ppm	m/z	z	RT	Fraction	Scan	Source File	Area KOntrola 7. traka 25 kDa	#Feature	#Feature KOntrola 7. traka 25 kDa	Start	End	PTM
R.WLGLSAEYGNLYR.N	N	99.73	1540.7673	13	-0.6	771.3905	2	33.43	8	1688	OB4036.raw	2.2E4	2	2	388	400	
R.WLGLSAEYGN(+.98)LYR.N	N	63.52	1541.7513	13	-4.8	771.8792	2	33.78	8	1711	OB4036.raw	0	0	0	388	400	Deamidation (NQ)
K.TANELNLLILR.W	Y	55.14	1268.7452	11	-1.9	635.3787	2	33.77	8	1709	OB4036.raw	2.24E3	1	1	377	387	
R.NALFVPHYNTNAHSIIYALR.G	N	41.55	2313.2019	20	2.9	772.0768	3	30.98	8	1554	OB4036.raw	2.97E3	1	1	401	420	
**total 4 peptides**																	

tr|B5TYU1|B5TYU1_ARAHY
back to list

| Protein Coverage | Supporting Peptides |

Protein Coverage:

```
  1  MAKLLELSFC FCFLVLGASS ISFRQQPEEN ACQFQRLNAQ RPDNRIESEG GYIETWNPNN QEFECAGVAL SRLVLRRNAL
 81  RRPFYSNAPQ EIFIQQGRGY FGLIFPGCPS TYEEPAQQGR RYQSQRPPRR LQEEDQSQQQ QDSHQKVHRF NEGDLIAVPT
161  GVAFWLYNDH DTDVVAVSLT DTNNNDNQLD QFPRRFNLAG NHEQEFLRYQ QQSRQSRRRS LPYSPYSPQS QPRQEEREFS
241  PRGQHSRRER AGQEEENEGG NIFSGFTPEF LAQAFQVDDR QIVQNLRGEN ESEEQGAIVT VRGGLRILSP DRKRGADEEE
321  EYDEDEYEYD EEDRRRGRGS RGSGNGIEET ICTATVKKNI GRNRSPDIYN PQAGSLKTAN ELNLLILRWL GLSAEYGNLY
401  RNALFVPHYN TNAHSIIYAL RGRAHVQVVD SNGNRVYDEE LQEGHVLVVP QNFAVAGKSQ SDNFEYVAFK TDSRPSIANL
481  AGENSVIDNL PEEVVANSYG LPREQARQLK NNNPFKFFVP PSQQSPRAVA
```

d Deamidation (NQ) (+0.98)

Supporting Peptides:

Peptide	Uniq	-10lgP	Mass	Length	ppm	m/z	z	RT	Fraction	Scan	Source File	Area KOntrola 7. traka 25 kDa	#Feature	#Feature KOntrola 7. traka 25 kDa	Start	End	PTM
R.WLGLSAEYGNLYR.N	N	99.73	1540.7673	13	-0.6	771.3905	2	33.43	8	1688	OB4036.raw	2.2E4	2	2	389	401	
R.WLGLSAEYGN(+.98)LYR.N	N	63.52	1541.7513	13	-4.8	771.8792	2	33.78	8	1711	OB4036.raw	0	0	0	389	401	Deamidation (NQ)
K.TANELNLLILR.W	Y	55.14	1268.7452	11	-1.9	635.3787	2	33.77	8	1709	OB4036.raw	2.24E3	1	1	378	388	
R.NALFVPHYNTNAHSIIYALR.G	N	41.55	2313.2019	20	2.9	772.0768	3	30.98	8	1554	OB4036.raw	2.97E3	1	1	402	421	
**total 4 peptides**																	

tr|Q647H4|Q647H4_ARAHY
back to list

| Protein Coverage | Supporting Peptides |

Protein Coverage:

```
  1 MGKLLALSVC FCFLVLGASS ISFRQQPEEN ACQFQRLNAQ RPDNRIESEG GYIETWNPNN QEFECAGVAL SRLVLRRNAL
 81 RRPFYSNAPQ EIFIQQGRGY FGLIFPGCPS TYEEPAQQGR RHQSQRPPRR FQGQDQSQQQ QDSHQKVHRF DEGDLIAVPT
161 GVAFWMYNDH DTDVVAVSLT DTNNNDNQLD QFPRRFNLAG NHEQEFLRYQ QQSRRRSLPY SPYSPQTQPK QEDREFSPRG
241 QHGRRERAGQ EQENEGGNIF SGFTPEFLAQ AFQVDDRQIL QNLRGENESD EQGAIVTVRG GLRILSPDRK RRQQYERPDE
321 EEEYDEDEYE YDEEERQHDR RRGRGSRGSG NGIEETICTA SFKKNIGRNR SPDIYNPQAG SLKTANELNL LILRWLGLSA
401 EYGNLYRNAL FVPHYNTNAH SIIYALRGRA HVQVVDSNGD RVFDEELQEG HVLVVPQNFA VAGKSQSENF EYVAFKTDSR
481 PSIANLAGEN SFIDNLPEEV VANSYGLPRE QARQLKNNNP FKFFVPPSEQ SLRAVA
```

d Deamidation (NQ) (+0.98)

Supporting Peptides:

Peptide	Uniq	-10lgP	Mass	Length	ppm	m/z	z	RT	Fraction	Scan	Source File	Area KOntrola 7. traka 25 kDa	#Feature	#Feature KOntrola 7. traka 25 kDa	Start	End	PTM
R.WLGLSAEYGNLYR.N	N	99.73	1540.7673	13	-0.6	771.3905	2	33.43	8	1688	OB4036.raw	2.2E4	2	2	395	407	
R.WLGLSAEYGN(+.98)LYR.N	N	63.52	1541.7513	13	-4.8	771.8792	2	33.78	8	1711	OB4036.raw	0	0	0	395	407	Deamidation (NQ)
K.TANELNLLILR.W	Y	55.14	1268.7452	11	-1.9	635.3787	2	33.77	8	1709	OB4036.raw	2.24E3	1	1	384	394	
R.NALFVPHYNTNAHSIIYALR.G	N	41.55	2313.2019	20	2.9	772.0768	3	30.98	8	1554	OB4036.raw	2.97E3	1	1	408	427	
**total 4 peptides**																	

tr|Q6T2T4|Q6T2T4_ARAHY

back to list

| Protein Coverage | Supporting Peptides |

Protein Coverage:

1 MGKLLALSVC FCFLVLGASS ISFRQQPEEN ACQFQRLNAQ RPDNRIESEG GYIETWNPNN QEFECAGVAL SRLVLRRNAL

81 RRPFYSNAPQ EIFIQQGRAY FGLIFLGCPS TYEEPAQQGR RHQSQRPPRR FQGQDQSQQQ QDSHQKVHRF DEGDLIAVPT

161 GVAFWMYNDH DTDVVAVSLT DTNNNDNQLD QFPRRFNLAG NHEQEFLRYQ QQSRRRSLPY SPYSPQTQPK QEDREFSPRG

241 QHGRRERAGQ EQENEGGNIF SGFTPEFLAQ AFQVDDRQIL QNLRGENESD EQGAIVTVRG GLRILSPDRK KRQQYERPDE

321 EEEYDEDEYE YDEEERQQDR RRGRGSRGSG NGIEETICTA SFKKNIGRNR SPDIYNPQAG SLK**TANELNL LILRWLGLSA**

401 **EYGNLYRNAL FVPHYNTNAH SIIYALR**GRA HVQVVDSNGD RVFDEELQEG HVLVVPQNFA VAGKSQSENF EYVAFKTDSR

d

481 PSIANLAGEN SFIDNLPEEV VANSYGLPRE QARQLKNNNP FKFFVPPSEQ SLRAVA

d Deamidation (NQ) (+0.98)

Supporting Peptides:

Peptide	Uniq	-10lgP	Mass	Length	ppm	m/z	z	RT	Fraction	Scan	Source File	Area KOntrola 7. traka 25 kDa	#Feature	#Feature KOntrola 7. traka 25 kDa	Start	End	PTM
R.WLGLSAEYGNLYR.N	N	99.73	1540.7673	13	-0.6	771.3905	2	33.43	8	1688	OB4036.raw	2.2E4	2	2	395	407	
R.WLGLSAEYGN(+.98)LYR.N	N	63.52	1541.7513	13	-4.8	771.8792	2	33.78	8	1711	OB4036.raw	0	0	0	395	407	Deamidation (NQ)
K.TANELNLLILR.W	Y	55.14	1268.7452	11	-1.9	635.3787	2	33.77	8	1709	OB4036.raw	2.24E3	1	1	384	394	
R.NALFVPHYNTNAHSIIYALR.G	N	41.55	2313.2019	20	2.9	772.0768	3	30.98	8	1554	OB4036.raw	2.97E3	1	1	408	427	
**total 4 peptides**																	

tr|Q9FZ11|Q9FZ11_ARAHY

back to list

| Protein Coverage | Supporting Peptides |

Protein Coverage:

1 MIRGRLALSV CFCFLVLGAS SISFRQQPEE NACQFQRLNA QRPDNRLESE GGYIETWNPN NQEFECAGVA LSRLVLRRNA

d Deamidation (NQ) (+0.98)

81 LRRPFYSNAP QEIFIQQGRG YFGLIFPGCP STYEEPAQQG RRHQSQRAPR RFEGEDQSQQ QQQDSHQKVR RFDEGDLIAV

161 PTGVALWMFN DHDTDVVAVS LTDTNNNDNQ LDQFPRRFNL AGNHEQEFLR YQQQSRRRSL PYSPYSPQSQ PRQEEREFSP

241 RGQHSRRERA GQEEENEGGN IFSGFTPEFL AQAFQVDDRQ IVQNLRGENE SEEEGAIVTV KGGLRILSPD RKRGADEEEE

321 YDEDEYEYDE EDRRRGRGSR GRGNGIEETI CTASVKKNIG RNRSPDIYNP QAGSLK**TAND LNLLILRWLG LSAEYGNLYR**

d

401 **NALFVPHYNT NAHSIIYALR** GRAHVQVVDS NGNRVYDEEL QEGHVLVVPQ NFAVAGKSQS DNFEYVAFKT DSRPNIANFA

481 GENSIIDNLP EEVVANSYGL PREQARQLKN NNPFKFFVPP SQQSLRAVA

Supporting Peptides:

Peptide	Uniq	-10lgP	Mass	Length	ppm	m/z	z	RT	Fraction	Scan	Source File	Area KOntrola 7. traka 25 kDa	#Feature	#Feature KOntrola 7. traka 25 kDa	Start	End	PTM
R.WLGLSAEYGNLYR.N	N	99.73	1540.7673	13	-0.6	771.3905	2	33.43	8	1688	OB4036.raw	2.2E4	2	2	388	400	
R.WLGLSAEYGN(+.98)LYR.N	N	63.52	1541.7513	13	-4.8	771.8792	2	33.78	8	1711	OB4036.raw	0	0	0	388	400	Deamidation (NQ)
K.TANDLNLLILR.W	Y	44.92	1254.7296	11	1.1	628.3728	2	33.51	8	1694	OB4036.raw	3.77E3	1	1	377	387	
R.NALFVPHYNTNAHSIIYALR.G	N	41.55	2313.2019	20	2.9	772.0768	3	30.98	8	1554	OB4036.raw	2.97E3	1	1	401	420	
**total 4 peptides**																	

tr|Q5I6T2|Q5I6T2_ARAHY

back to list

| Protein Coverage | Supporting Peptides |

Protein Coverage:

1 MAKLLELSFC FCFLVLGASS ISFRQQPEEN ACQFQRLNAQ RPDNRIESEG GYIETWNPNN QEFECAGVAL SRLVLRRNAL

d Deamidation (NQ) (+0.98)

81 RRPFYSNAPQ EIFIQQGRGY FGLIFPGCPS TYEEPAQQGR RSQSQRPPRR LQGEDQSQQQ QDSHQKVHRF DEGDLIAVPT

161 GVAFWLYNDH DTDVVAVSLT DTNNNDNQLD QFPRRFNLAG NHEQEFLRYQ QQSRQSRRRS LPYSPYSPQS QPRQEEREFS

241 PRGQHSRRER AGQEEENEGG NIFSGFTPEF LEQAFQVDDR QIVQNLRGEN ESEEEGAIVT VRGGLRILSP DRKRGADEEE

321 EYDEDEYEYD EEDRRRGRGS RGRGNGIEET ICTASVKKNI GRNRSPDIYN PQAGSLK**TAN DLNLLILRWL GLSAEYGNLY**

d

401 **RNALFVPHYN TNAHSIIYAL R**GRAHVQVVD SNGNRVYDEE LQEGHVLVVP QNFAVAGKSQ SDNFEYVAFK TDSRPSIANL

481 AGENSVIDNL PEEVVANSYG LQREQARQQL KNNNPFKFFV PPSQQSPRAV A

Supporting Peptides:

Peptide	Uniq	-10lgP	Mass	Length	ppm	m/z	z	RT	Fraction	Scan	Source File	Area KOntrola 7. traka 25 kDa	#Feature	#Feature KOntrola 7. traka 25 kDa	Start	End	PTM
R.WLGLSAEYGNLYR.N	N	99.73	1540.7673	13	-0.6	771.3905	2	33.43	8	1688	OB4036.raw	2.2E4	2	2	389	401	
R.WLGLSAEYGN(+.98)LYR.N	N	63.52	1541.7513	13	-4.8	771.8792	2	33.78	8	1711	OB4036.raw	0	0	0	389	401	Deamidation (NQ)
K.TANDLNLLILR.W	Y	44.92	1254.7296	11	1.1	628.3728	2	33.51	8	1694	OB4036.raw	3.77E3	1	1	378	388	
R.NALFVPHYNTNAHSIIYALR.G	N	41.55	2313.2019	20	2.9	772.0768	3	30.98	8	1554	OB4036.raw	2.97E3	1	1	402	421	
**total 4 peptides**																	

tr|Q647H3|Q647H3_ARAHY

back to list

| Protein Coverage | Supporting Peptides |

Protein Coverage:

d Deamidation (NQ) (+0.98)

```
  1 MAKLLALSVC FCFLVLGASS ISFRQQPEEN ACQFQRLNAQ RPDNRLESEG GYIETWNPNN QEFECAGVAL SRLVLRRNAL
 81 RRPFYSNAPQ EIFIQQGRGY FGLIFPGCPS TYEEPAQQGR RHQSQRAPRR FEGEDQSQQQ QQDSHQKVRR FDEGDLIAVP
161 TGVALWMYND HDTDVVAVSL TDTNNNDNQL DQFPRRFNLA GNHEQEFLRY QQQSRRRSLP YSPYSPQSQP RQEEREFSPR
241 GQHSRRERAG QEQENEGGNI FSGFTPEFLA QAFQVDDRQI LQNLRGENES DEQGAIVTVR GGLRILSPDR KRRQQYERPD
321 EEEEYDEDEY EYDEEERQQD RRRGRGSRGR GNGIEETICT ASVKKNIGRN RSPDIYNPQA GSLKTANDLN LLILRWLGLS
401 AEYGNLYRNA LFVPHYNTNA HSIIYALRGR AHVQVVDSNG NRVYDEELQE GHVLVVPQNF AVAGKSQSDN FEYVAFKTDS
481 RPSIANLAGE NSIIDNLPEE VVANSYGLPR EQARQLKNNN PFKFFVPPSQ QSLGAVA
```

Supporting Peptides:

Peptide	Uniq	-10lgP	Mass	Length	ppm	m/z	z	RT	Fraction	Scan	Source File	Area KOntrola 7. traka 25 kDa	#Feature	#Feature KOntrola 7. traka 25 kDa	Start	End	PTM
R.WLGLSAEYGNLYR.N	N	99.73	1540.7673	13	-0.6	771.3905	2	33.43	8	1688	OB4036.raw	2.2E4	2	2	396	408	
R.WLGLSAEYGN(+.98)LYR.N	N	63.52	1541.7513	13	-4.8	771.8792	2	33.78	8	1711	OB4036.raw	0	0	0	396	408	Deamidation (NQ)
K.TANDLNLLILR.W	Y	44.92	1254.7296	11	1.1	628.3728	2	33.51	8	1694	OB4036.raw	3.77E3	1	1	385	395	
R.NALFVPHYNTNAHSIIYALR.G	N	41.55	2313.2019	20	2.9	772.0768	3	30.98	8	1554	OB4036.raw	2.97E3	1	1	409	428	
**total 4 peptides**																	

Peptide List

Summary

1. Notes Gastric Digest Raw peanut Band #7 24-27 kDa (PTM492)

2. Result Statistics

Figure 1. False discovery rate (FDR) curve. X axis is the number of peptide-spectrum matches (PSM) being kept. Y axis is the corresponding FDR.

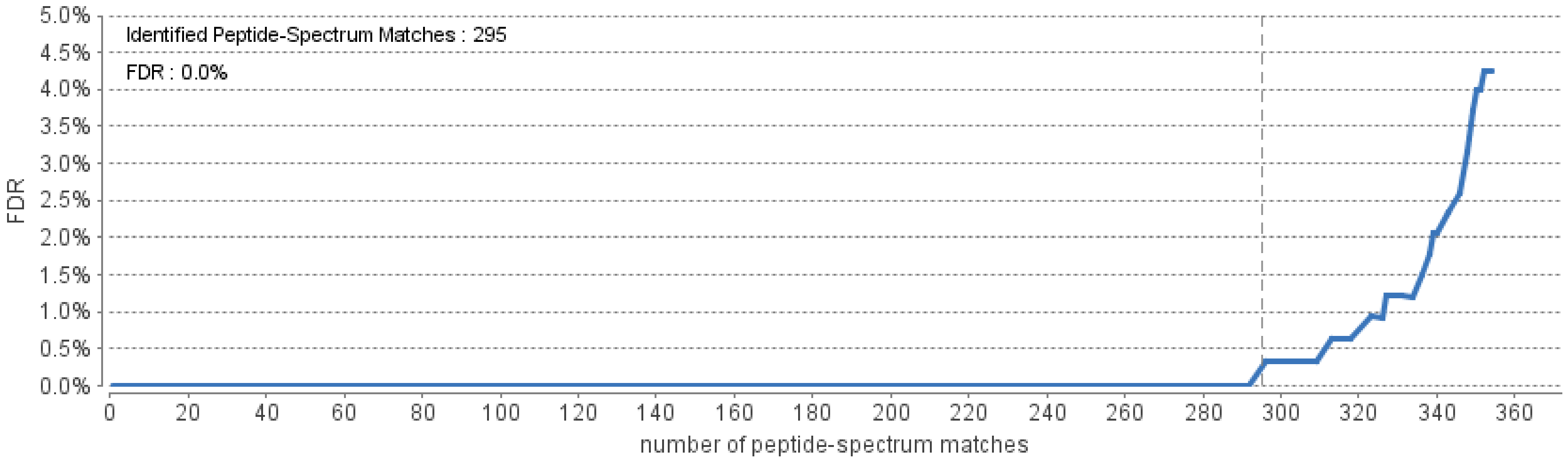

Figure 2. PSM score distribution. **(a)** Distribution of PEAKS peptide score; **(b)** Scatterplot of PEAKS peptide score versus precursor mass error.

(a)

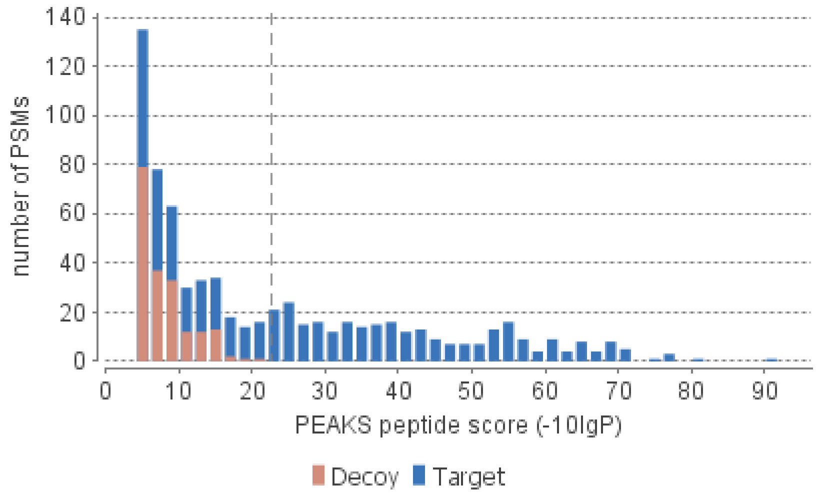

(b)

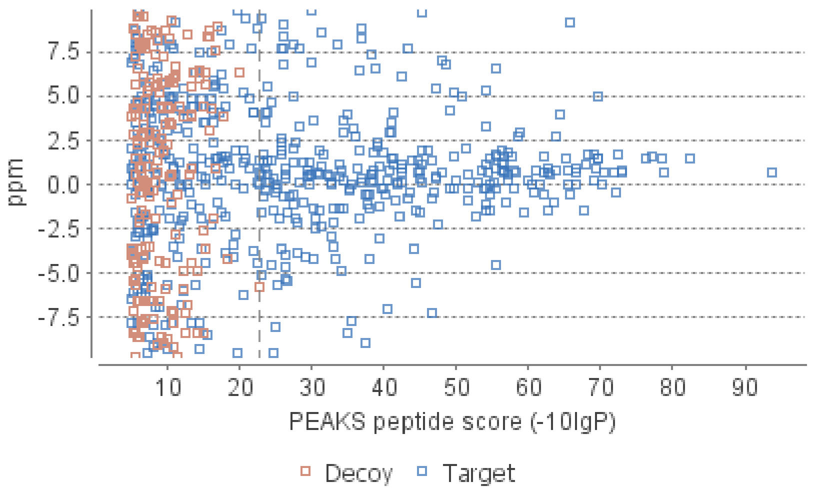

Figure 3. Distribution of peptide feature detection. **(a)** Feature m/z distribution; **(b)** Feature RT distribution

(a) (b)

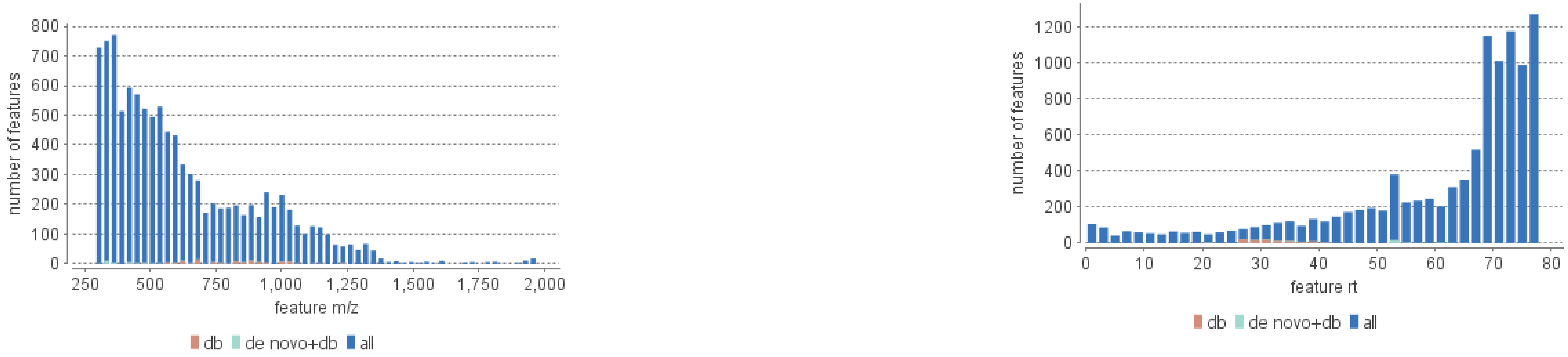

Figure 4. Distribution of identified peptide features. **(a)** Feature abundance distribution **(b)** *De novo* sequencing validation.

(a) (b)

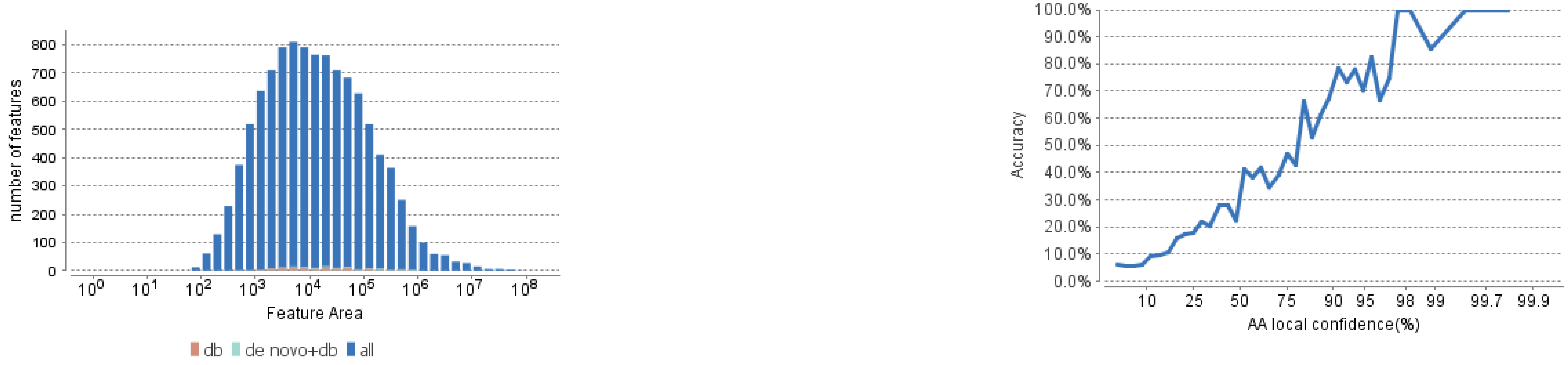

Table 1. Statistics of data.

# of MS scans	2904
# of MS/MS scans	1532
# of Features	10623
# of Chimera scans	205

Table 2. Result filtration parameters.

Peptide -10lgP	≥22.7

Table 4. PTM profile.

Name	ΔMass	Position	#PSM	-10lgP	Abundance	AScore
Deamidation	.98	NQ	222	72.96	1.32E5	16.65
Carbamidomethyl	57.02	C	106	77.03		1000.00
HydPro	15.99	P	49	67.87	5.43E4	69.71
Methylation(KR)	14.02	R	4	35.31		16.78

PTM Ascore	≥20
Protein -10lgP	≥20
Proteins unique peptides	≥1
De novo ALC Score	≥50%

Table 3. Statistics of filtered result.

Peptide-Spectrum Matches	295
Peptide sequences	187
Protein groups	9
Proteins	13
Proteins (#Unique Peptides)	4 (>2); 2 (=2); 7 (=1);
FDR (Peptide-Spectrum Matches)	0.0%
FDR (Peptide Sequences)	0.0%
FDR (Protein Group)	0.0%
De Novo Only Spectra	66
# of identified Features	138
# of identified MS/MS scans	278

Oxidation	15.99	M	3	25.96	7.52E3	1000.00
Carbamylation	43.01	N-term	2	33.80	2.68E3	1000.00
Acetylation	42.01	N-term	2	26.80	3.53E3	1000.00
Pyro-glu from Q	-17.03	N-term	2	25.82		1000.00
Dimethylation(KR)	28.03	K	1	69.27	5.4E3	1000.00
EtOH	44.03	K	1	28.65	1.06E4	85.84
Dehydration	-18.01	Y	1	23.18		3.64
Dihydroxy	31.99	R	1	25.71		0.00
Sodium	21.98	D	1	23.18		0.00

3. Experiment Control

Figure 5. Precursor mass error of peptide-spectrum matches (PSM) in filtered result. **(a)** Distribution of precursor mass error in ppm; **(b)** Scatterplot of precursor m/z versus precursor mass error in ppm.

(a)

(b)

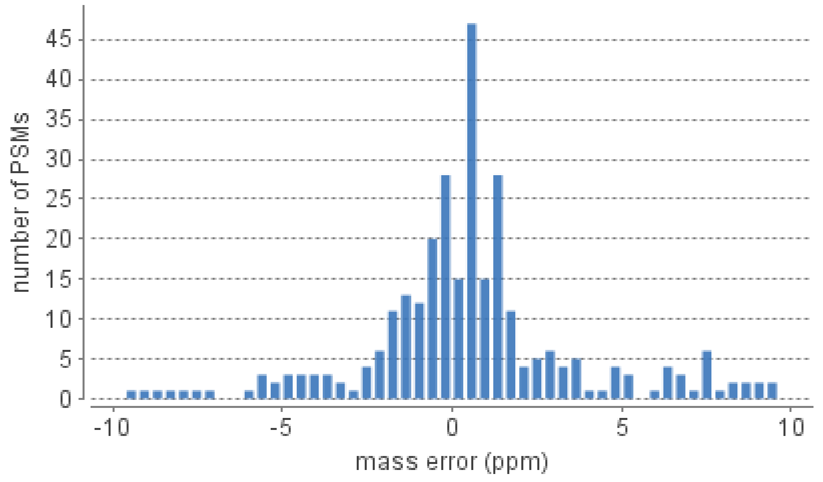

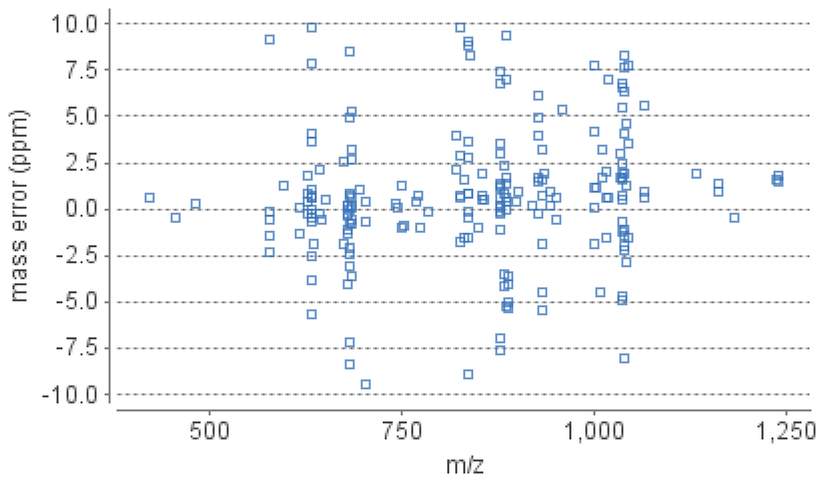

Table 5. Number of identified peptides in each sample by the number of missed cleavages

Missed Cleavages	0	1	2	3	4+
Digest 7. traka 25-27 kDa	56	104	27	0	0

4. Other Information

Table 6. Search parameters.

Search Engine Name: PEAKS
Parent Mass Error Tolerance: 10.0 ppm
Fragment Mass Error Tolerance: 0.5 Da
Precursor Mass Search Type: monoisotopic
Enzyme: Trypsin
Max Missed Cleavages: 2
Digest Mode: Unspecific
Fixed Modifications:
  Carbamidomethylation: 57.02
Variable Modifications:
  Deamidation (NQ): 0.98
  Oxidation (M): 15.99
  Hydroxylation Pro: 15.99
  Acetylation (K): 42.01
  Acetylation (Protein N-term): 42.01
  Acetylation (N-term): 42.01
  Amidation: -0.98
  Beta-methylthiolation: 45.99
  and 305 more...
Max Variable PTM Per Peptide: 5

Table 7. Instrument parameters.

Fractions: OB4057.raw
Ion Source: ESI(nano-spray)
Fragmentation Mode: CID, CAD(y and b ions)
MS Scan Mode: FT-ICR/Orbitrap
MS/MS Scan Mode: Linear Ion Trap

Database: Uniprot_Peanut-3818_Jul18
Taxon: All
Contaminant Database: contaminantsMQ_mar19
Searched Entry: 1723
FDR Estimation: Enabled
De novo score (ALC%) threshold: 15
Peptide hit threshold (-10logP): 30.0
Peaks run ID: 478
Merge Options: no merge
Precursor Options: corrected
Charge Options: no correction
Filter Charge: 2 - 8
Process: true
Associate chimera: yes

Protein List

Protein Accession Contains:
Protein Description Contains:
Peptide Sample Area >=
Protein Ptm Contains:

Protein Group	Protein ID	Accession	-10lgP	Coverage (%)	Coverage (%) Digest 7. traka 25-27 kDa	Area Digest 7. traka 25-27 kDa	#Peptides	#Unique	#Spec Digest 7. traka 25-27 kDa	PTM	Avg. Mass	Description
**1**	20581	tr|Q647H3|Q647H3_ARAHY	285.21	51	51	2.93E5	30	5	232	Y	61532	Arachin Ahy-2 OS=Arachis hypogaea OX=3818 PE=2 SV=1
**3**	20580	tr|Q647H4|Q647H4_ARAHY	278.60	42	42	1.17E5	28	2	214	Y	61506	Arachin Ahy-1 OS=Arachis hypogaea OX=3818 PE=2 SV=1
**4**	20574	tr|A1DZF0|A1DZF0_ARAHY	275.05	42	42	5.84E3	28	2	206	Y	60375	Arachin 6 OS=Arachis hypogaea OX=3818 PE=2 SV=1
**5**	20590	tr|O82580|O82580_ARAHY	217.48	25	25	0E0	16	1	101	Y	58350	Glycinin (Fragment) OS=Arachis hypogaea OX=3818 GN=Arah3 PE=2 SV=1
**6**	20593	tr|Q6IWG5|Q6IWG5_ARAHY	95.00	18	18	3.06E5	5	3	15	Y	58061	Glycinin (Fragment) OS=Arachis hypogaea OX=3818 PE=2 SV=1
**6**	20594	tr|Q0GM57|Q0GM57_ARAHY	95.00	18	18	3.06E5	5	3	15	Y	58263	Iso-Ara h3 OS=Arachis hypogaea OX=3818 PE=2 SV=1
**6**	20595	tr|E5G077|E5G077_ARAHY	95.00	18	18	3.06E5	5	3	15	Y	58305	Ara h 3 allergen OS=Arachis hypogaea OX=3818 GN=ara h 3 PE=3 SV=1
**13**	21034	tr|Q38711|Q38711_ARAHY	56.30	5	5	5.67E3	1	1	1	N	29134	Galactose-binding lectin (Fragment) OS=Arachis hypogaea OX=3818 GN=lec PE=2 SV=1
**13**	21036	tr|A0A089ZXL7|A0A089ZXL7_ARAHY	56.30	5	5	5.67E3	1	1	1	N	29407	Peanut agglutinin variant OS=Arachis hypogaea OX=3818 PE=2 SV=1
**13**	21035	P02872|LECG_ARAHY	56.30	5	5	5.67E3	1	1	1	N	29325	Galactose-binding lectin OS=Arachis hypogaea OX=3818 PE=1 SV=3
**14**	25842	tr|A0A109QJM5|A0A109QJM5_ARAHY	27.33	2	2	6.54E3	1	1	1	Y	74266	Long chain acyl-CoA synthetase 4 OS=Arachis hypogaea OX=3818 GN=LACS4 PE=2 SV=1
**15**	22368	tr|A1E2B0|A1E2B0_ARAHY	24.77	4	4	5.41E3	1	1	1	N	33520	11S seed storage globulin B1 OS=Arachis hypogaea OX=3818 PE=2 SV=1
**12**	20968	Q647H2|AHY3_ARAHY	23.43	7	7	2.36E4	1	1	1	Y	54569	Arachin Ahy-3 OS=Arachis hypogaea OX=3818 PE=1 SV=1

Protein Group	Protein ID	Accession	-10lgP	Coverage (%)	Coverage (%) Digest 7. traka 25-27 kDa	Area Digest 7. traka 25-27 kDa	#Peptides	#Unique	#Spec Digest 7. traka 25-27 kDa	PTM	Avg. Mass	Description
total 13 proteins												

tr|Q647H3|Q647H3_ARAHY

back to list

| Protein Coverage | Supporting Peptides |

Protein Coverage:

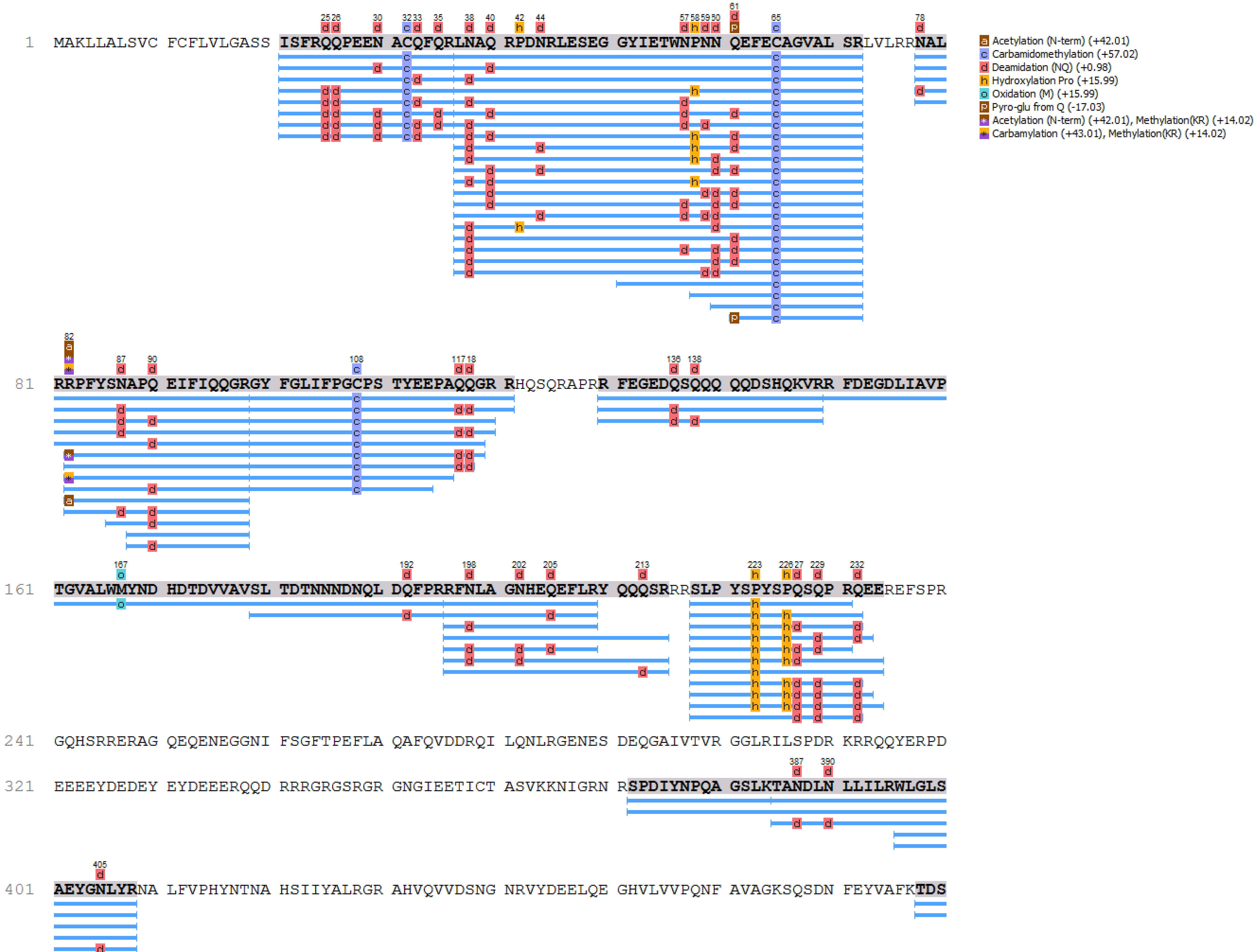
1 MAKLLALSVC FCFLVLGASS ISFRQQPEEN ACQFQRLNAQ RPDNRLESEG GYIETWNPNN QEFECAGVAL SRLVLRRNAL
81 RRPFYSNAPQ EIFIQQGRGY FGLIFPGCPS TYEEPAQQGR RHQSQRAPRR FEGEDQSQQQ QQDSHQKVRR FDEGDLIAVP
161 TGVALWMYND HDTDVVAVSL TDTNNNDNQL DQFPRRFNLA GNHEQEFLRY QQQSRRRSLP YSPYSPQSQP RQEEREFSPR
241 GQHSRRERAG QEQENEGGNI FSGFTPEFLA QAFQVDDRQI LQNLRGENES DEQGAIVTVR GGLRILSPDR KRRQQYERPD
321 EEEEYDEDEY EYDEEERQQD RRRGRGSRGR GNGIEETICT ASVKKNIGRN RSPDIYNPQA GSLKTANDLN LLILRWLGLS
401 AEYGNLYRNA LFVPHYNTNA HSIIYALRGR AHVQVVDSNG NRVYDEELQE GHVLVVPQNF AVAGKSQSDN FEYVAFKTDS
a Acetylation (N-term) (+42.01)
c Carbamidomethylation (+57.02)
d Deamidation (NQ) (+0.98)
h Hydroxylation Pro (+15.99)
o Oxidation (M) (+15.99)
p Pyro-glu from Q (-17.03)
* Acetylation (N-term) (+42.01), Methylation(KR) (+14.02)
* Carbamylation (+43.01), Methylation(KR) (+14.02)

Supporting Peptides:

Peptide	Uniq	-10lgP	Mass	Length	ppm	m/z	z	RT	Fraction	Scan	Source File	Area Digest 7. traka 25-27 kDa	#Feature	#Feature Digest 7. traka 25-27 kDa	Start	End	PTM
R.WLGLSAEYGNLYR.N	N	93.88	1540.7673	13	0.7	771.3915	2	33.33	29	1738	OB4057.raw	2.5E4	1	1	396	408	
K.TANDLNLLILRWLGLSAEYGNLYR.N	Y	82.38	2777.4863	24	1.5	926.8374	3	39.78	29	2153	OB4057.raw	5.17E4	1	1	385	408	
R.GYFGLIFPGC(+57.02)PSTYEEPAQQGR.R	N	77.03	2473.1372	22	1.6	1237.5779	2	34.90	29	1839	OB4057.raw	0	0	0	99	120	Carbamidomethylation
R.NALRRPFYSN(+.98)APQEIFIQQGR.G	N	72.96	2505.2876	21	0.8	836.1038	3	30.04	29	1537	OB4057.raw	1.34E5	2	2	78	98	Deamidation (NQ)
R.RPFYSNAPQEIFIQQGR.G	N	72.96	2050.0383	17	0.6	684.3538	3	30.09	29	1546	OB4057.raw	1.76E5	1	1	82	98	
K.TAN(+.98)DLNLLILRWLGLSAEYGNLYR.N	Y	72.33	2778.4705	24	1.7	927.1657	3	40.44	29	2204	OB4057.raw	1.91E4	2	2	385	408	
R.NALRRPFYSNAPQEIFIQQGR.G	N	71.98	2504.3037	21	-0.5	835.7748	3	29.54	29	1503	OB4057.raw	5.44E5	2	2	78	98	
R.RPFYSNAPQ(+.98)EIFIQQGR.G	N	70.26	2051.0225	17	0.0	684.6815	3	30.74	29	1580	OB4057.raw	2.52E5	1	1	82	98	Deamidation (NQ)
N.NQ(+.98)EFEC(+57.02)AGVALSR.L	N	69.12	1480.6616	13	0.2	741.3383	2	28.82	29	1460	OB4057.raw	5.61E3	1	1	60	72	Carbamidomethylation
K.TANDLN(+.98)LLILRWLGLSAEYGNLYR.N	Y	68.94	2778.4705	24	1.7	927.1657	3	40.44	29	2185	OB4057.raw	1.91E4	2	2	385	408	
R.RFNLAGNHEQ(+.98)EFLR.Y	N	67.53	1730.8488	14	-1.5	577.9560	3	28.20	29	1404	OB4057.raw	4.97E4	1	1	196	209	Deamidation (NQ)
R.RFNLAGN(+.98)HEQ(+.98)EFLR.Y	N	65.72	1731.8329	14	9.2	578.2902	3	28.98	29	1473	OB4057.raw	0	0	0	196	209	Deamidation (NQ)
R.RPFYSN(+.98)APQ(+.98)EIFIQQGR.G	N	65.72	2052.0063	17	-0.6	685.0090	3	31.27	29	1614	OB4057.raw	1.11E5	2	2	82	98	Deamidation (NQ)
K.TAN(+.98)DLN(+.98)LLILRWLGLSAEYGNLYR.N	Y	64.43	2779.4543	24	4.0	927.4957	3	40.41	29	2193	OB4057.raw	0	0	0	385	408	Deamidation (NQ)
N.PN(+.98)NQ(+.98)EFEC(+57.02)AGVALSR.L	N	63.50	1692.7413	15	-1.0	847.3771	2	29.54	29	1509	OB4057.raw	2.96E3	1	1	58	72	Carbamidomethylation
R.SLPYSP(+15.99)YSP(+15.99)Q(+.98)SQ(+.98)PRQ(+.98).E	N	62.95	1768.7791	15	0.6	885.3973	2	26.79	29	1334	OB4057.raw	6.62E4	1	1	218	232	Hydroxylation Pro; Deamidation (NQ)
R.RFN(+.98)LAGNHEQEFLR.Y	N	62.67	1730.8488	14	-1.5	577.9560	3	28.20	29	1449	OB4057.raw	4.97E4	1	1	196	209	Deamidation (NQ)
R.RFNLAGNHEQEFLR.Y	N	62.20	1729.8647	14	-0.6	577.6285	3	27.45	29	1373	OB4057.raw	2.42E4	1	1	196	209	
R.SLPYSP(+15.99)YSP(+15.99)Q(+.98)SQ(+.98)PRQ(+.98)E.E	N	62.00	1897.8217	16	0.6	949.9187	2	26.89	29	1338	OB4057.raw	2.05E4	1	1	218	233	Hydroxylation Pro; Deamidation (NQ)
R.RPFYSNAPQEIFIQQ(+.98)GR.G	N	61.45	2051.0225	17	0.0	684.6815	3	30.74	29	1558	OB4057.raw	2.52E5	1	1	82	98	
R.NALRRPFYSN(+.98)APQ(+.98)EIFIQQGR.G	N	61.01	2506.2717	21	0.8	836.4319	3	30.86	29	1581	OB4057.raw	1.12E5	1	1	78	98	Deamidation (NQ)
R.N(+.98)ALRRPFYSNAPQ(+.98)EIFIQQGR.G	N	59.37	2506.2717	21	-1.6	836.4299	3	31.54	29	1635	OB4057.raw	6.41E3	1	1	78	98	Deamidation (NQ)
R.GYFGLIFPGC(+57.02)PSTYEEPAQ(+.98)Q(+.98)GR.R	N	58.83	2475.1052	22	2.9	826.0447	3	35.24	29	1860	OB4057.raw	5.56E4	1	1	99	120	Carbamidomethylation; Deamidation (NQ)
R.GYFGLIFPGC(+57.02)PSTYEEPAQ(+.98)QGR.R	N	58.17	2474.1211	22	0.7	825.7148	3	35.01	29	1841	OB4057.raw	5.09E4	2	2	99	120	Carbamidomethylation
N.APQEIFIQQGR.G	N	57.98	1285.6779	11	-0.2	643.8461	2	28.00	29	1415	OB4057.raw	2.55E3	1	1	88	98	

Peptide	Uniq	-10lgP	Mass	Length	ppm	m/z	z	RT	Fraction	Scan	Source File	Area Digest 7. traka 25-27 kDa	#Feature	#Feature Digest 7. traka 25-27 kDa	Start	End	PTM
R.WLGLSAEYGN(+.98)LYR.N	N	56.70	1541.7513	13	-1.0	771.8821	2	33.70	29	1766	OB4057.raw	0	0	0	396	408	Deamidation (NQ)
R.N(+.98)ALRRPFYSN(+.98)APQEIFIQQGR.G	N	56.60	2506.2717	21	0.8	836.4319	3	30.86	29	1598	OB4057.raw	1.19E5	2	2	78	98	Deamidation (NQ)
R.SLPYSP(+15.99)YSP(+15.99)Q(+.98)SQPRQ(+.98).E	N	56.49	1767.7950	15	1.6	884.9062	2	26.44	29	1317	OB4057.raw	1.61E4	1	1	218	232	Hydroxylation Pro; Deamidation (NQ)
R.SLPYSP(+15.99)YSP(+15.99)Q(+.98)SQ(+.98)PRQ(+.98)EE.R	N	56.48	2026.8643	17	0.7	1014.4401	2	26.99	29	1346	OB4057.raw	2.68E4	1	1	218	234	Hydroxylation Pro; Deamidation (NQ)
R.NALRRPFYSN(+.98)APQEIFIQQ(+.98)GR.G	N	56.36	2506.2717	21	0.8	836.4319	3	30.86	29	1575	OB4057.raw	1.12E5	1	1	78	98	
R.SLPYSP(+15.99)YSP(+15.99)QSQ(+.98)PRQ(+.98).E	N	55.47	1767.7950	15	1.6	884.9062	2	26.44	29	1315	OB4057.raw	1.61E4	1	1	218	232	Hydroxylation Pro
S.ISFRQ(+.98)QPEENAC(+57.02)QFQR.L	N	55.40	2037.9326	16	-0.2	680.3180	3	26.89	29	1336	OB4057.raw	3.68E4	1	1	21	36	Carbamidomethylation
R.LNAQ(+.98)RPDNRLESEGGYIETWN(+.98)PNNQ(+.98)EFEC(+57.02)AGVALSR.L	N	55.37	4136.8770	36	6.5	1035.2333	4	31.90	29	1667	OB4057.raw	3.94E5	1	1	37	72	Deamidation (NQ); Carbamidomethylation
R.RFNLAGN(+.98)HEQEFLR.Y	N	54.72	1730.8488	14	-1.5	577.9560	3	28.20	29	1420	OB4057.raw	4.97E4	1	1	196	209	
R.RPFYSN(+.98)APQEIFIQQGR.G	N	54.60	2051.0225	17	0.0	684.6815	3	30.74	29	1688	OB4057.raw	2.52E5	1	1	82	98	
R.RPFYSNAPQ(+.98)EIFIQ(+.98)QGR.G	N	54.53	2052.0063	17	-0.6	685.0090	3	31.27	29	1601	OB4057.raw	1.08E5	1	1	82	98	
N.APQ(+.98)EIFIQQGR.G	N	53.97	1286.6619	11	-0.2	644.3381	2	28.64	29	1452	OB4057.raw	6.63E3	1	1	88	98	Deamidation (NQ)
S.ISFRQQPEENAC(+57.02)QFQR.L	N	53.95	2036.9486	16	-1.1	679.9894	3	26.44	29	1318	OB4057.raw	5.84E3	1	1	21	36	Carbamidomethylation
S.ISFRQQ(+.98)PEENAC(+57.02)QFQR.L	N	53.83	2037.9326	16	-0.2	680.3180	3	26.89	29	1344	OB4057.raw	3.68E4	1	1	21	36	Carbamidomethylation
S.ISFRQQ(+.98)PEENAC(+57.02)Q(+.98)FQR.L	N	52.87	2038.9166	16	0.1	680.6462	3	27.25	29	1372	OB4057.raw	1.04E5	1	1	21	36	Carbamidomethylation; Deamidation (NQ)
S.ISFRQ(+.98)QPEEN(+.98)AC(+57.02)QFQR.L	N	52.86	2038.9166	16	0.1	680.6462	3	27.25	29	1361	OB4057.raw	1.04E5	1	1	21	36	Deamidation (NQ); Carbamidomethylation
R.RPFYSN(+.98)APQ(+.98)EIFIQ(+.98)QGR.G	N	51.51	2052.9905	17	-0.9	685.3369	3	31.47	29	1634	OB4057.raw	1.41E4	1	1	82	98	Deamidation (NQ)
R.RFN(+.98)LAGNHEQ(+.98)EFLR.Y	N	49.63	1731.8329	14	-0.2	578.2848	3	28.72	29	1459	OB4057.raw	1.88E4	1	1	196	209	Deamidation (NQ)
R.LN(+.98)AQRPDNRLESEGGYIETWN(+.98)PNNQEFEC(+57.02)AGVALSR.L	N	49.62	4135.8931	36	1.6	1034.9822	4	31.82	29	1652	OB4057.raw	0	0	0	37	72	Deamidation (NQ); Carbamidomethylation
V.SLTDTNN(+.98)N(+.98)DN(+.98)QLDQ(+.98)FPR.R	N	49.16	1994.8341	17	4.1	998.4285	2	30.46	29	1566	OB4057.raw	0	0	0	179	195	Deamidation (NQ)
R.LNAQ(+.98)RPDNRLESEGGYIETWNPN(+.98)N(+.98)Q(+.98)EFEC(+57.02)AGVALSR.L	N	48.61	4137.8608	36	6.8	1035.4795	4	32.47	29	1691	OB4057.raw	0	0	0	37	72	Deamidation (NQ); Carbamidomethylation

Peptide	Uniq	-10lgP	Mass	Length	ppm	m/z	z	RT	Fraction	Scan	Source File	Area Digest 7. traka 25-27 kDa	#Feature	#Feature Digest 7. traka 25-27 kDa	Start	End	PTM
R.RFN(+.98)LAGN(+.98)HEQ(+.98)EFLR.Y	N	47.43	1732.8169	14	-2.3	578.6116	3	29.08	29	1481	OB4057.raw	4.14E3	1	1	196	209	Deamidation (NQ)
R.LNAQ(+.98)RPDN(+.98)RLESEGGYIETWNPNN(+.98)Q(+.98)EFEC(+57.02)AGVALSR.L	N	47.19	4137.8608	36	5.5	1035.4781	4	32.37	29	1685	OB4057.raw	0	0	0	37	72	Deamidation (NQ); Carbamidomethylation
S.ISFRQ(+.98)Q(+.98)PEENAC(+57.02)Q(+.98)FQR.L	N	46.46	2039.9006	16	-0.8	680.9736	3	27.61	29	1392	OB4057.raw	1.5E5	1	1	21	36	Deamidation (NQ); Carbamidomethylation
S.ISFRQ(+.98)QPEEN(+.98)AC(+57.02)Q(+.98)FQR.L	N	46.46	2039.9006	16	-7.2	680.9692	3	28.84	29	1464	OB4057.raw	0	0	0	21	36	Carbamidomethylation; Deamidation (NQ)
R.GYFGLIFPGC(+57.02)PSTYEEPAQ(+.98)Q(+.98)G.R	N	46.43	2319.0042	21	1.4	1160.5110	2	37.30	29	1999	OB4057.raw	1.95E4	1	1	99	119	Carbamidomethylation; Deamidation (NQ)
G.GYIETWNPN(+.98)NQEFEC(+57.02)AGVALSR.L	N	46.05	2555.1387	22	1.9	852.7218	3	33.01	29	1724	OB4057.raw	0	0	0	51	72	Carbamidomethylation
R.GYFGLIFPGC(+57.02)PSTYEEPAQ(+.98)Q(+.98).G	N	45.23	2261.9827	20	1.9	1132.0007	2	37.44	29	2005	OB4057.raw	2.43E3	1	1	99	118	Carbamidomethylation; Deamidation (NQ)
R.GYFGLIFPGC(+57.02)PSTYEEPA.Q	N	45.16	2003.8975	18	1.1	1002.9572	2	37.61	29	2011	OB4057.raw	6.78E3	1	1	99	116	Carbamidomethylation
R.GYFGLIFPGC(+57.02)PSTYEEPAQ(+.98)QGRR.H	N	45.10	2630.2224	23	0.2	877.7482	3	33.53	29	1771	OB4057.raw	2.51E5	2	2	99	121	Carbamidomethylation
R.SLPYSP(+15.99)YSP(+15.99)Q(+.98)SQPRQ(+.98)EE.R	N	44.77	2025.8802	17	-1.6	1013.9458	2	26.69	29	1329	OB4057.raw	8.53E3	1	1	218	234	Hydroxylation Pro; Deamidation (NQ)
S.ISFRQ(+.98)Q(+.98)PEEN(+.98)AC(+57.02)QFQ(+.98)R.L	N	44.56	2040.8846	16	0.7	681.3026	3	28.00	29	1419	OB4057.raw	6.08E4	2	2	21	36	Deamidation (NQ); Carbamidomethylation
R.LN(+.98)AQRPDN(+.98)RLESEGGYIETWNP(+15.99)NNQ(+.98)EFEC(+57.02)AGVALSR.L	N	43.32	4152.8721	36	7.7	1039.2333	4	31.96	29	1673	OB4057.raw	3.37E4	1	1	37	72	Deamidation (NQ); Hydroxylation Pro; Carbamidomethylation
S.ISFRQQ(+.98)PEEN(+.98)AC(+57.02)QFQ(+.98)R.L	N	43.09	2039.9006	16	-0.8	680.9736	3	27.61	29	1428	OB4057.raw	1.5E5	1	1	21	36	Deamidation (NQ); Carbamidomethylation
R.GYFGLIFPGC(+57.02)PSTYEEPAQ(+.98)QG.R	N	42.45	2318.0200	21	0.9	1160.0183	2	37.15	29	1981	OB4057.raw	0	0	0	99	119	Carbamidomethylation
R.GYFGLIFPGC(+57.02)PSTYEEPAQ(+.98)Q(+.98)GRR.H	N	42.43	2631.2063	23	1.2	878.0771	3	33.54	29	1756	OB4057.raw	0	0	0	99	121	Carbamidomethylation; Deamidation (NQ)

Peptide	Uniq	-10lgP	Mass	Length	ppm	m/z	z	RT	Fraction	Scan	Source File	Area Digest 7. traka 25-27 kDa	#Feature	#Feature Digest 7. traka 25-27 kDa	Start	End	PTM
Y.SNAPQ(+.98)EIFIQQGR.G	N	42.42	1487.7368	13	0.1	744.8757	2	29.44	29	1506	OB4057.raw	2.04E3	1	1	86	98	Deamidation (NQ)
R.GYFGLIFPGC(+57.02)PSTYEEPAQQ(+.98)GRR.H	N	42.26	2630.2224	23	0.7	877.7487	3	33.34	29	1744	OB4057.raw	2.48E5	1	1	99	121	Carbamidomethylation
S.ISFRQ(+.98)Q(+.98)PEENAC(+57.02)QFQR.L	N	41.78	2038.9166	16	0.1	680.6462	3	27.25	29	1432	OB4057.raw	1.04E5	1	1	21	36	Deamidation (NQ); Carbamidomethylation
R.RFN(+.98)LAGN(+.98)HEQ(+.98)EFLRYQQ(+.98)Q(+.98)SR.R	N	41.31	2525.1570	20	4.0	632.2991	4	29.73	29	1519	OB4057.raw	0	0	0	196	215	Deamidation (NQ)
R.GYFGLIFP(+15.99)GC(+57.02)PSTYEEPAQQGR.R	N	41.18	2489.1321	22	1.5	830.7192	3	35.31	29	1863	OB4057.raw	3.8E3	1	1	99	120	Carbamidomethylation
R.GYFGLIFPGC(+57.02)P(+15.99)STYEEPAQQGR.R	N	40.91	2489.1321	22	1.5	830.7192	3	35.31	29	1878	OB4057.raw	3.8E3	1	1	99	120	Carbamidomethylation
R.LN(+.98)AQRPDNRLESEGGYIETWNPNNQEFEC(+57.02)AGVALSR.L	N	40.89	4134.9087	36	3.0	1034.7375	4	31.51	29	1640	OB4057.raw	1.17E5	1	1	37	72	Deamidation (NQ); Carbamidomethylation
S.ISFRQ(+.98)Q(+.98)PEENAC(+57.02)QFQ(+.98)R.L	N	40.78	2039.9006	16	0.8	680.9747	3	28.66	29	1453	OB4057.raw	0	0	0	21	36	Deamidation (NQ); Carbamidomethylation
R.LNAQ(+.98)RPDNRLESEGGYIETWNPNNQEFEC(+57.02)AGVALSR.L	N	40.63	4134.9087	36	3.0	1034.7375	4	31.51	29	1628	OB4057.raw	1.17E5	1	1	37	72	Deamidation (NQ); Carbamidomethylation
R.GYFGLIFPGC(+57.02)PSTYEE.P	N	40.42	1835.8076	16	0.2	918.9113	2	37.63	29	2015	OB4057.raw	1.67E3	1	1	99	114	Carbamidomethylation
R.GYFGLIFPGC(+57.02)PSTYEEPAQQGRR.H	N	40.40	2629.2383	23	-7.0	877.4139	3	33.26	29	1739	OB4057.raw	0	0	0	99	121	Carbamidomethylation
N.APQ(+.98)EIFIQ(+.98)QGR.G	N	40.15	1287.6459	11	-0.6	644.8298	2	29.21	29	1486	OB4057.raw	1.52E3	1	1	88	98	Deamidation (NQ)
S.ISFRQ(+.98)Q(+.98)PEEN(+.98)AC(+57.02)Q(+.98)FQ(+.98)R.L	N	39.36	2041.8687	16	-3.1	681.6281	3	28.45	29	1440	OB4057.raw	3.98E3	1	1	21	36	Deamidation (NQ); Carbamidomethylation
R.SPDIYNPQ(+.98)AGSLK.T	N	39.27	1389.6776	13	1.0	695.8468	2	28.06	29	1416	OB4057.raw	0	0	0	372	384	
R.LNAQRPDN(+.98)RLESEGGYIETWN(+.98)PN(+.98)N(+.98)QEFEC(+57.02)AGVALSR.L	N	39.23	4137.8608	36	-1.3	1035.4712	4	32.66	29	1703	OB4057.raw	0	0	0	37	72	Deamidation (NQ); Carbamidomethylation
R.LN(+.98)AQRPDNRLESEGGYIETWNPNN(+.98)Q(+.98)EFEC(+57.02)AGVALSR.L	N	39.15	4136.8770	36	2.4	1035.2290	4	33.65	29	1763	OB4057.raw	0	0	0	37	72	Deamidation (NQ); Carbamidomethylation
R.SLPYSP(+15.99)YSP(+15.99)Q(+.98)SQ(+.98)PR.Q	N	39.01	1639.7365	14	2.1	820.8773	2	26.59	29	1330	OB4057.raw	5.55E3	1	1	218	231	Hydroxylation Pro; Deamidation (NQ)
R.LN(+.98)AQRPDNRLESEGGYIETWN(+.98)PN(+.98)NQEFEC(+57.02)AGVALSR.L	N	38.91	4136.8770	36	6.5	1035.2333	4	31.90	29	1708	OB4057.raw	3.94E5	1	1	37	72	Deamidation (NQ);

Peptide	Uniq	-10lgP	Mass	Length	ppm	m/z	z	RT	Fraction	Scan	Source File	Area Digest 7. traka 25-27 kDa	#Feature	#Feature Digest 7. traka 25-27 kDa	Start	End	PTM
																	Carbamidomethylation
R.LN(+.98)AQRPDNRLESEGGYIETWNPN(+.98)N(+.98)QEFEC(+57.02)AGVALSR.L	N	38.53	4136.8770	36	0.8	1035.2273	4	34.69	29	1826	OB4057.raw	0	0	0	37	72	Deamidation (NQ); Carbamidomethylation
R.GYFGLIFPGC(+57.02)PSTYEEP(+15.99)AQQGRR.H	N	38.08	2645.2332	23	-4.2	882.7480	3	33.96	29	1777	OB4057.raw	4.66E4	1	1	99	121	Carbamidomethylation
R.RFNLAGN(+.98)HEQEFLRYQ(+.98)Q(+.98)QSR.R	N	37.97	2523.1890	20	-0.5	631.8042	4	28.35	29	1434	OB4057.raw	0	0	0	196	215	
R.RFEGEDQ(+.98)SQ(+.98)Q(+.98)Q(+.98)QQDSHQ(+.98)KVR.R	Y	37.91	2462.0581	20	-1.3	616.5210	4	21.45	29	1056	OB4057.raw	1.97E4	1	1	130	149	Deamidation (NQ)
R.NALRRPFYSNAPQ(+.98)EIFIQQGR.G	N	37.39	2505.2876	21	-8.9	836.0957	3	33.41	29	1748	OB4057.raw	8.09E5	1	1	78	98	
R.SLPYSP(+15.99)YSP(+15.99)QSQ(+.98)PRQ(+.98)E.E	N	37.26	1896.8376	16	-0.6	949.4255	2	26.49	29	1322	OB4057.raw	5.37E3	1	1	218	233	Hydroxylation Pro; Deamidation (NQ)
R.SPDIYN(+.98)PQ(+.98)AGSLKTANDLNLLILRWLGLSAEYGNLYR.N	Y	36.78	4150.1372	37	8.3	1038.5502	4	39.90	29	2160	OB4057.raw	0	0	0	372	408	
R.LN(+.98)AQRPDNRLESEGGYIETWNP(+15.99)NN(+.98)QEFEC(+57.02)AGVALSR.L	N	36.67	4151.8877	36	6.4	1038.9858	4	32.27	29	1679	OB4057.raw	0	0	0	37	72	Deamidation (NQ); Hydroxylation Pro; Carbamidomethylation
R.LNAQ(+.98)RPDNRLESEGGYIETWN(+.98)PNN(+.98)Q(+.98)EFEC(+57.02)AGVALSR.L	N	36.45	4137.8608	36	1.9	1035.4745	4	32.59	29	1698	OB4057.raw	0	0	0	37	72	Deamidation (NQ); Carbamidomethylation
R.RFN(+.98)LAGNHEQ(+.98)EFLRYQ(+.98)Q(+.98)QSR.R	N	35.14	2524.1731	20	-0.3	632.0504	4	29.48	29	1504	OB4057.raw	0	0	0	196	215	
R.SLPYSP(+15.99)YSP(+15.99)Q(+.98)SQPR.Q	N	34.93	1638.7524	14	3.9	820.3867	2	26.35	29	1310	OB4057.raw	0	0	0	218	231	Hydroxylation Pro
R.RFEGEDQ(+.98)SQQQ(+.98)Q(+.98)Q(+.98)DSHQ(+.98)KVR.R	Y	34.31	2462.0581	20	-1.3	616.5210	4	21.45	29	1034	OB4057.raw	1.97E4	1	1	130	149	
R.GYFGLIFPGC(+57.02)P(+15.99)STYEEPAQQ(+.98)GRR.H	N	34.17	2646.2173	23	0.8	883.0804	3	34.27	29	1801	OB4057.raw	0	0	0	99	121	Carbamidomethylation
R.RFEGEDQ(+.98)SQ(+.98)Q(+.98)QQ(+.98)QDSHQ(+.98)KVR.R	Y	33.84	2462.0581	20	-1.3	616.5210	4	21.45	29	1052	OB4057.raw	1.97E4	1	1	130	149	Deamidation (NQ)
R.R(+43.01)(+14.02)PFYSNAPQEIFIQQGR.G	N	33.80	2107.0598	17	0.4	703.3608	3	30.09	29	1541	OB4057.raw	2.68E3	1	1	82	98	Carbamylation; Methylation(KR)
R.RFEGEDQ(+.98)SQQ(+.98)Q(+.98)Q(+.98)QDSHQ(+.98)KVR.R	Y	33.69	2462.0581	20	-1.3	616.5210	4	21.45	29	1046	OB4057.raw	1.97E4	1	1	130	149	
R.RFN(+.98)LAGNHEQ(+.98)EFLRYQ(+.98)Q(+.98)Q(+.98)SR.R	N	33.42	2525.1570	20	7.9	632.3015	4	29.03	29	1480	OB4057.raw	2.4E4	1	1	196	215	
R.GYFGLIFPGC(+57.02)P(+15.99)STYEEPAQQGRR.H	N	33.29	2645.2332	23	-4.2	882.7480	3	33.96	29	1772	OB4057.raw	4.66E4	1	1	99	121	Carbamidomethylation
R.RFEGEDQ(+.98)SQQ(+.98)Q(+.98)Q(+.98)Q(+.98)DSHQKVR.R	Y	33.06	2462.0581	20	-1.3	616.5210	4	21.45	29	1039	OB4057.raw	1.97E4	1	1	130	149	
R.SPDIYNPQAGSLKTANDLNLLILRWLGLSAEYGNLYR.N	Y	32.83	4148.1694	37	-2.2	1038.0474	4	39.68	29	2146	OB4057.raw	0	0	0	372	408	
S.ISFRQ(+.98)Q(+.98)PEEN(+.98)AC(+57.02)Q(+.98)FQR.L	N	32.58	2040.8846	16	0.0	681.3021	3	28.64	29	1453	OB4057.raw	2.82E3	1	1	21	36	Deamidation (NQ); Carbamidomethylation
R.RFNLAGN(+.98)HEQ(+.98)EFLRYQ(+.98)QQSR.R	N	31.37	2523.1890	20	1.0	631.8052	4	28.55	29	1446	OB4057.raw	0	0	0	196	215	
S.ISFRQ(+.98)Q(+.98)PEEN(+.98)AC(+57.02)QFQR.L	N	31.15	2039.9006	16	-2.4	680.9725	3	29.92	29	1531	OB4057.raw	0	0	0	21	36	Carbamidomethylation

Peptide	Uniq	-10lgP	Mass	Length	ppm	m/z	z	RT	Fraction	Scan	Source File	Area Digest 7. traka 25-27 kDa	#Feature	#Feature Digest 7. traka 25-27 kDa	Start	End	PTM
R.LNAQRPDNRLESEGGYIETWNP(+15.99)NNQEFEC(+57.02)AGVALSR.L	N	31.12	4149.9199	36	1.9	1038.4893	4	31.82	29	1673	OB4057.raw	6.01E4	1	1	37	72	Hydroxylation Pro; Carbamidomethylation
R.GYFGLIFP(+15.99)GC(+57.02)PSTYEEPAQQ(+.98)GR.R	N	30.90	2490.1160	22	-1.5	831.0447	3	35.67	29	1886	OB4057.raw	0	0	0	99	120	Carbamidomethylation
R.RFNLAGN(+.98)HEQ(+.98)EFLRYQ(+.98)Q(+.98)QSR.R	N	30.35	2524.1731	20	-2.5	632.0490	4	28.87	29	1466	OB4057.raw	0	0	0	196	215	
R.RFN(+.98)LAGN(+.98)HEQEFLRYQ(+.98)QQ(+.98)SR.R	N	30.27	2524.1731	20	3.7	632.0529	4	29.09	29	1480	OB4057.raw	0	0	0	196	215	Deamidation (NQ)
S.ISFRQQ(+.98)PEEN(+.98)AC(+57.02)QFQR.L	N	30.06	2038.9166	16	-1.3	680.6453	3	29.22	29	1488	OB4057.raw	0	0	0	21	36	Carbamidomethylation
R.RFNLAGNHEQ(+.98)EFLRYQ(+.98)Q(+.98)QSR.R	N	29.87	2523.1890	20	9.8	631.8107	4	28.40	29	1437	OB4057.raw	0	0	0	196	215	
R.SLPYSPYSP(+15.99)Q(+.98)SQ(+.98)PRQ(+.98).E	N	29.62	1752.7842	15	0.1	877.3994	2	27.92	29	1406	OB4057.raw	1.81E4	1	1	218	232	Deamidation (NQ)
R.RFNLAGN(+.98)HEQEFLRYQQQ(+.98)SR.R	N	29.43	2522.2051	20	0.8	631.5590	4	28.08	29	1417	OB4057.raw	0	0	0	196	215	
R.GYFGLIFPGC(+57.02)P(+15.99)STYEEP(+15.99)AQQGRR.H	N	29.12	2661.2280	23	-4.0	888.0797	3	33.53	29	1750	OB4057.raw	2.5E4	1	1	99	121	Carbamidomethylation
R.RFEGEDQ(+.98)SQQ(+.98)Q(+.98)QQ(+.98)DSHQKVR.R	Y	28.33	2461.0742	20	0.1	616.2759	4	21.03	29	1030	OB4057.raw	6.04E3	1	1	130	149	
R.LN(+.98)AQ(+.98)RPDNRLESEGGYIETWNP(+15.99)NNQ(+.98)EFEC(+57.02)AGVALSR.L	N	28.15	4152.8721	36	7.7	1039.2333	4	31.96	29	1668	OB4057.raw	3.37E4	1	1	37	72	Deamidation (NQ); Hydroxylation Pro; Carbamidomethylation
R.RFN(+.98)LAGNHEQ(+.98)EFLRYQQ(+.98)Q(+.98)SR.R	N	28.03	2524.1731	20	-3.8	632.0482	4	29.55	29	1508	OB4057.raw	0	0	0	196	215	
R.GYFGLIFPGC(+57.02)PSTYEEP(+15.99)AQ(+.98)QGRR.H	N	27.80	2646.2173	23	2.4	883.0818	3	34.48	29	1814	OB4057.raw	0	0	0	99	121	Carbamidomethylation
K.TDSRPSIAN(+.98)LAGENSIIDNLPEEVVANSYGLPREQ(+.98)AR.Q	Y	27.72	3996.9663	37	-1.9	1000.2469	4	34.65	29	1824	OB4057.raw	0	0	0	478	514	Deamidation (NQ)
R.LN(+.98)AQ(+.98)RPDNRLESEGGYIETWNP(+15.99)NNQEFEC(+57.02)AGVALSR.L	N	27.49	4151.8877	36	-1.1	1038.9780	4	32.39	29	1686	OB4057.raw	0	0	0	37	72	Deamidation (NQ); Hydroxylation Pro; Carbamidomethylation
R.RFNLAGN(+.98)HEQ(+.98)EFLRYQ(+.98)Q(+.98)Q(+.98)SR.R	N	27.28	2525.1570	20	7.9	632.3015	4	29.03	29	1466	OB4057.raw	2.4E4	1	1	196	215	
R.R(+42.01)P(+15.99)FYSNAPQEIFIQQGR.G	N	26.80	2108.0439	17	-0.7	703.6881	3	30.76	29	1577	OB4057.raw	3.53E3	1	1	82	98	Acetylation (N-term)
R.SLPYSP(+15.99)YSP(+15.99)Q(+.98)SQ(+.98)PRQEE.R	N	26.49	2025.8802	17	-1.6	1013.9458	2	26.69	29	1341	OB4057.raw	8.53E3	1	1	218	234	Hydroxylation Pro
R.GYFGLIFPGC(+57.02)P(+15.99)STYEEP(+15.99)AQQ(+.98)GRR.H	N	26.49	2662.2122	23	-5.4	888.4066	3	33.81	29	1773	OB4057.raw	0	0	0	99	121	Carbamidomethylation
R.LN(+.98)AQRPDNRLESEGGYIETWNPNNQ(+.98)EFEC(+57.02)AGVALSR.L	N	26.34	4135.8931	36	-4.7	1034.9757	4	33.17	29	1734	OB4057.raw	0	0	0	37	72	Deamidation (NQ); Carbamidomethylation
R.LN(+.98)AQRPDNRLESEGGYIETWN(+.98)PNN(+.98)Q(+.98)EFEC(+57.02)AGVALSR.L	N	26.06	4137.8608	36	-0.7	1035.4718	4	33.49	29	1753	OB4057.raw	0	0	0	37	72	Deamidation (NQ); Carbamidomethylation

Peptide	Uniq	-10lgP	Mass	Length	ppm	m/z	z	RT	Fraction	Scan	Source File	Area Digest 7. traka 25-27 kDa	#Feature	#Feature Digest 7. traka 25-27 kDa	Start	End	PTM
R.N(+.98)ALRRPFYSN(+.98)APQEIFIQ(+.98)Q(+.98)GR.G	N	25.99	2508.2397	21	8.4	837.0942	3	31.36	29	1621	OB4057.raw	1.89E4	1	1	78	98	
R.RFDEGDLIAVPTGVALWM(+15.99)YNDHDTDVVAVSLTDTN(+.98)N(+.98)NDN(+.98)Q(+.98)LDQFPR.R	Y	25.96	5210.3574	46	7.7	1043.0868	5	35.88	29	1902	OB4057.raw	7.52E3	1	1	150	195	Oxidation (M)
R.NALRRPFYSN(+.98)APQ(+.98)EIFIQQ(+.98)GR.G	N	25.95	2507.2556	21	9.1	836.7667	3	32.01	29	1663	OB4057.raw	0	0	0	78	98	
N.Q(-17.03)EFEC(+57.02)AGVALSR.L	N	25.82	1348.6082	12	2.6	675.3131	2	31.68	29	1643	OB4057.raw	1.38E3	1	1	61	72	Pyro-glu from Q; Carbamidomethylation
K.TDSRPSIAN(+.98)LAGEN(+.98)SIIDNLPEEVVANSYGLPREQ(+.98)AR.Q	Y	25.78	3997.9504	37	7.7	1000.5026	4	34.76	29	1824	OB4057.raw	1.75E4	1	1	478	514	
R.GYFGLIFPGC(+57.02)PSTYEEPAQQGR(+31.99)R.H	N	25.71	2661.2280	23	-3.6	888.0801	3	33.19	29	1735	OB4057.raw	0	0	0	99	121	Carbamidomethylation
R.LNAQRPDNRLESEGGYIETWNPNNQEFEC(+57.02)AGVALSR.L	N	25.66	4133.9248	36	1.7	1034.4902	4	31.34	29	1622	OB4057.raw	0	0	0	37	72	Carbamidomethylation
R.RFN(+.98)LAGN(+.98)HEQEFLRYQ(+.98)Q(+.98)Q(+.98)SR.R	N	25.15	2525.1570	20	-5.6	632.2930	4	29.68	29	1516	OB4057.raw	2.4E3	1	1	196	215	
R.LN(+.98)AQRP(+15.99)DNRLESEGGYIETWNPNN(+.98)QEFEC(+57.02)AGVALSR.L	N	25.01	4151.8877	36	-8.1	1038.9708	4	32.88	29	1716	OB4057.raw	0	0	0	37	72	Deamidation (NQ); Hydroxylation Pro; Carbamidomethylation
R.R(+42.01)(+14.02)PFYSNAPQEIFIQQGR.G	N	24.55	2106.0647	17	-9.4	703.0222	3	30.07	29	1541	OB4057.raw	0	0	0	82	98	Acetylation (N-term); Methylation(KR)
R.SPDIYNPQAGSLKTANDLN(+.98)LLILRWLGLSAEYGNLYR.N	Y	23.90	4149.1533	37	4.0	1038.2998	4	39.72	29	2167	OB4057.raw	3.67E4	1	1	372	408	
R.SPDIYN(+.98)PQAGSLKTANDLNLLILRWLGLSAEYGNLYR.N	Y	23.52	4149.1533	37	4.0	1038.2998	4	39.72	29	2144	OB4057.raw	3.67E4	1	1	372	408	
R.RFNLAGNHEQ(+.98)EFLRYQQ(+.98)QSR.R	N	23.33	2522.2051	20	-0.7	631.5581	4	28.18	29	1423	OB4057.raw	0	0	0	196	215	
R.LNAQRPD(+21.98)NRLESEGGY(-18.01)IETWNPNNQEFEC(+57.02)AGVALSR.L	N	23.18	4137.8965	36	1.7	1035.4832	4	33.31	29	1742	OB4057.raw	0	0	0	37	72	Carbamidomethylation
R.GYFGLIFPGC(+57.02)P(+15.99)STYEEP(+15.99)AQ(+.98)Q(+.98)GRR.H	N	23.01	2663.1960	23	-5.1	888.7348	3	34.20	29	1797	OB4057.raw	0	0	0	99	121	Carbamidomethylation; Deamidation (NQ)
R.RFDEGDLIAVPTGVALWM(+15.99)YNDHDTDVVAVSLTDTNN(+.98)NDN(+.98)Q(+.98)LDQFPR.R	Y	22.83	5209.3735	46	-1.5	1042.8804	5	35.93	29	1902	OB4057.raw	0	0	0	150	195	Oxidation (M)
G.GYIETWN(+.98)PNN(+.98)QEFEC(+57.02)AGVALSR.L	N	22.78	2556.1226	22	0.7	853.0487	3	33.23	29	1746	OB4057.raw	8.99E3	1	1	51	72	Carbamidomethylation
**total 140 peptides**																	

tr|Q647H4|Q647H4_ARAHY
back to list

| Protein Coverage | Supporting Peptides |

Protein Coverage:

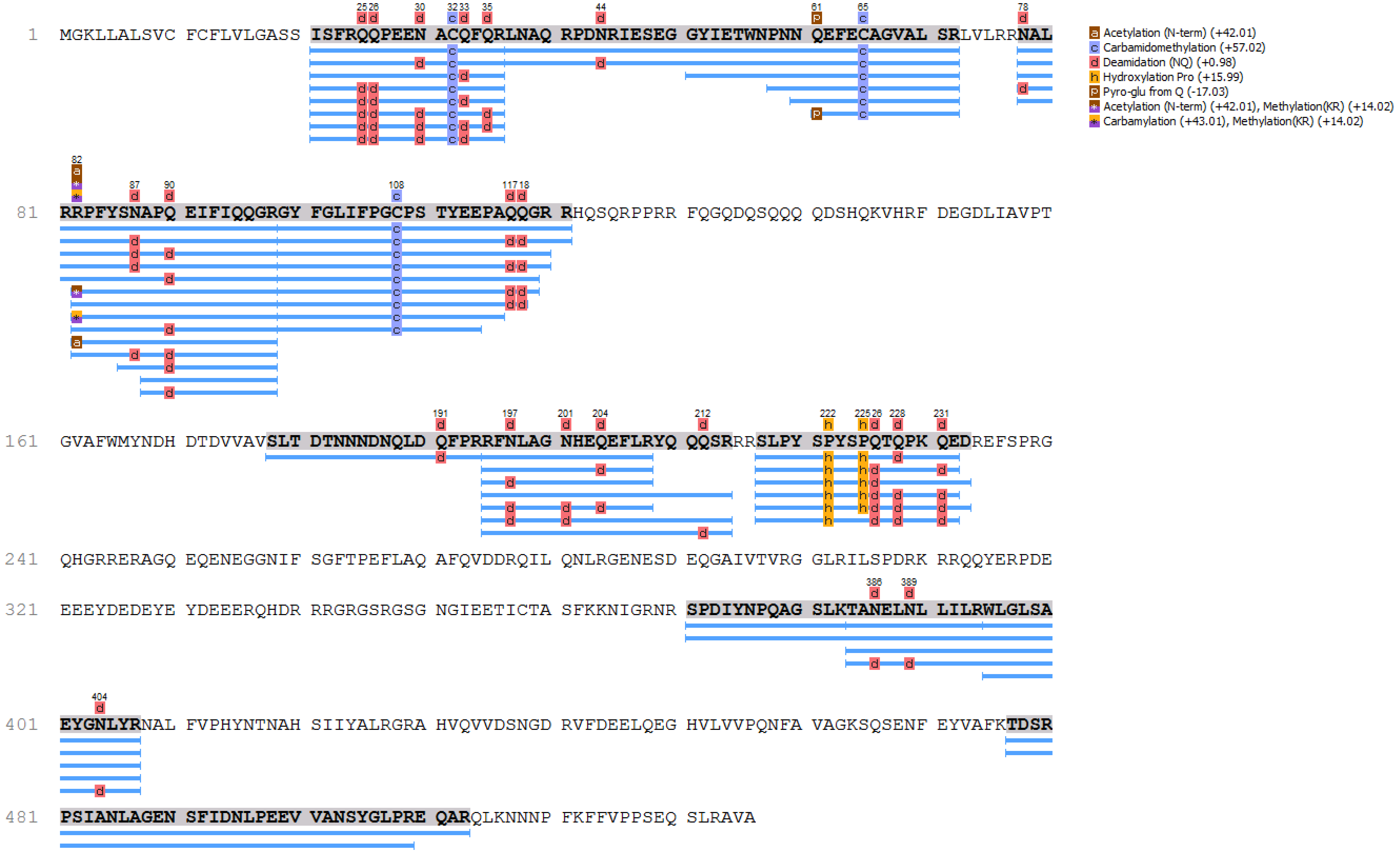

Supporting Peptides:

Peptide	Uniq	-10lgP	Mass	Length	ppm	m/z	z	RT	Fraction	Scan	Source File	Area Digest 7. traka 25-27 kDa	#Feature	#Feature Digest 7. traka 25-27 kDa	Start	End	PTM
R.WLGLSAEYGNLYR.N	N	93.88	1540.7673	13	0.7	771.3915	2	33.33	29	1738	OB4057.raw	2.5E4	1	1	395	407	
R.GYFGLIFPGC(+57.02)PSTYEEPAQQGR.R	N	77.03	2473.1372	22	1.6	1237.5779	2	34.90	29	1839	OB4057.raw	0	0	0	99	120	Carbamidomethylation
R.NALRRPFYSN(+.98)APQEIFIQQGR.G	N	72.96	2505.2876	21	0.8	836.1038	3	30.04	29	1537	OB4057.raw	1.34E5	2	2	78	98	Deamidation (NQ)
R.RPFYSNAPQEIFIQQGR.G	N	72.96	2050.0383	17	0.6	684.3538	3	30.09	29	1546	OB4057.raw	1.76E5	1	1	82	98	
R.NALRRPFYSNAPQEIFIQQGR.G	N	71.98	2504.3037	21	-0.5	835.7748	3	29.54	29	1503	OB4057.raw	5.44E5	2	2	78	98	
K.TANELNLLILRWLGLSAEYGNLYR.N	N	70.48	2791.5020	24	0.7	931.5086	3	39.84	29	2175	OB4057.raw	2.08E4	1	1	384	407	
R.RPFYSNAPQ(+.98)EIFIQQGR.G	N	70.26	2051.0225	17	0.0	684.6815	3	30.74	29	1580	OB4057.raw	2.52E5	1	1	82	98	Deamidation (NQ)
N.NQ(+.98)EFEC(+57.02)AGVALSR.L	N	69.12	1480.6616	13	0.2	741.3383	2	28.82	29	1460	OB4057.raw	5.61E3	1	1	60	72	Carbamidomethylation
R.SLPYSP(+15.99)YSP(+15.99)Q(+.98)TQ(+.98)PKQ(+.98)ED.R	Y	67.87	1998.8582	17	1.1	1000.4375	2	26.99	29	1348	OB4057.raw	5.43E4	1	1	217	233	Hydroxylation Pro; Deamidation (NQ)
R.RFNLAGNHEQ(+.98)EFLR.Y	N	67.53	1730.8488	14	-1.5	577.9560	3	28.20	29	1404	OB4057.raw	4.97E4	1	1	195	208	Deamidation (NQ)
R.SLPYSP(+15.99)YSP(+15.99)Q(+.98)TQ(+.98)PKQ(+.98)E.D	Y	66.43	1883.8312	16	1.0	942.9238	2	26.89	29	1340	OB4057.raw	3.79E4	1	1	217	232	Hydroxylation Pro; Deamidation (NQ)
R.RFNLAGN(+.98)HEQ(+.98)EFLR.Y	N	65.72	1731.8329	14	9.2	578.2902	3	28.98	29	1473	OB4057.raw	0	0	0	195	208	Deamidation (NQ)
R.RPFYSN(+.98)APQ(+.98)EIFIQQGR.G	N	65.72	2052.0063	17	-0.6	685.0090	3	31.27	29	1614	OB4057.raw	1.11E5	2	2	82	98	Deamidation (NQ)
N.PN(+.98)NQ(+.98)EFEC(+57.02)AGVALSR.L	N	63.50	1692.7413	15	-1.0	847.3771	2	29.54	29	1509	OB4057.raw	2.96E3	1	1	58	72	Carbamidomethylation
R.RFN(+.98)LAGNHEQEFLR.Y	N	62.67	1730.8488	14	-1.5	577.9560	3	28.20	29	1449	OB4057.raw	4.97E4	1	1	195	208	Deamidation (NQ)
R.RFNLAGNHEQEFLR.Y	N	62.20	1729.8647	14	-0.6	577.6285	3	27.45	29	1373	OB4057.raw	2.42E4	1	1	195	208	
K.TAN(+.98)ELNLLILRWLGLSAEYGNLYR.N	N	62.19	2792.4861	24	1.5	931.8374	3	40.46	29	2201	OB4057.raw	4.9E3	1	1	384	407	
R.RPFYSNAPQEIFIQQ(+.98)GR.G	N	61.45	2051.0225	17	0.0	684.6815	3	30.74	29	1558	OB4057.raw	2.52E5	1	1	82	98	
R.NALRRPFYSN(+.98)APQ(+.98)EIFIQQGR.G	N	61.01	2506.2717	21	0.8	836.4319	3	30.86	29	1581	OB4057.raw	1.12E5	1	1	78	98	Deamidation (NQ)
R.SLPYSP(+15.99)YSP(+15.99)Q(+.98)TQPKQ(+.98)E.D	Y	59.96	1882.8472	16	0.2	942.4310	2	26.59	29	1328	OB4057.raw	7.29E3	1	1	217	232	Hydroxylation Pro; Deamidation (NQ)
R.N(+.98)ALRRPFYSNAPQ(+.98)EIFIQQGR.G	N	59.37	2506.2717	21	-1.6	836.4299	3	31.54	29	1635	OB4057.raw	6.41E3	1	1	78	98	Deamidation (NQ)
R.GYFGLIFPGC(+57.02)PSTYEEPAQ(+.98)Q(+.98)GR.R	N	58.83	2475.1052	22	2.9	826.0447	3	35.24	29	1860	OB4057.raw	5.56E4	1	1	99	120	Carbamidomethylation; Deamidation (NQ)
R.GYFGLIFPGC(+57.02)PSTYEEPAQ(+.98)QGR.R	N	58.17	2474.1211	22	0.7	825.7148	3	35.01	29	1841	OB4057.raw	5.09E4	2	2	99	120	Carbamidomethylation
N.APQEIFIQQGR.G	N	57.98	1285.6779	11	-0.2	643.8461	2	28.00	29	1415	OB4057.raw	2.55E3	1	1	88	98	
K.TANELN(+.98)LLILRWLGLSAEYGNLYR.N	N	57.78	2792.4861	24	1.5	931.8374	3	40.46	29	2195	OB4057.raw	4.9E3	1	1	384	407	
R.WLGLSAEYGN(+.98)LYR.N	N	56.70	1541.7513	13	-1.0	771.8821	2	33.70	29	1766	OB4057.raw	0	0	0	395	407	Deamidation (NQ)
R.N(+.98)ALRRPFYSN(+.98)APQEIFIQQGR.G	N	56.60	2506.2717	21	0.8	836.4319	3	30.86	29	1598	OB4057.raw	1.19E5	2	2	78	98	Deamidation (NQ)
R.NALRRPFYSN(+.98)APQEIFIQQ(+.98)GR.G	N	56.36	2506.2717	21	0.8	836.4319	3	30.86	29	1575	OB4057.raw	1.12E5	1	1	78	98	
R.SLPYSP(+15.99)YSPQ(+.98)TQ(+.98)PKQ(+.98)E.D	Y	56.18	1867.8363	16	1.9	934.9272	2	27.82	29	1401	OB4057.raw	0	0	0	217	232	Hydroxylation Pro; Deamidation (NQ)
S.ISFRQ(+.98)QPEENAC(+57.02)QFQR.L	N	55.40	2037.9326	16	-0.2	680.3180	3	26.89	29	1336	OB4057.raw	3.68E4	1	1	21	36	Carbamidomethylation
R.LNAQ(+.98)RPDNRIESEGGYIETWN(+.98)PNNQ(+.98)EFEC(+57.02)AGVALSR.L	N	55.37	4136.8770	36	6.5	1035.2333	4	31.90	29	1667	OB4057.raw	3.94E5	1	1	37	72	Carbamidomethylation
R.SLPYSP(+15.99)YSP(+15.99)Q(+.98)TQPKQ(+.98)ED.R	Y	55.11	1997.8741	17	0.1	999.9445	2	26.69	29	1335	OB4057.raw	1.02E4	1	1	217	233	Hydroxylation Pro; Deamidation (NQ)
R.RFNLAGN(+.98)HEQEFLR.Y	N	54.72	1730.8488	14	-1.5	577.9560	3	28.20	29	1420	OB4057.raw	4.97E4	1	1	195	208	
R.RPFYSN(+.98)APQEIFIQQGR.G	N	54.60	2051.0225	17	0.0	684.6815	3	30.74	29	1688	OB4057.raw	2.52E5	1	1	82	98	
R.RPFYSNAPQ(+.98)EIFIQ(+.98)QGR.G	N	54.53	2052.0063	17	-0.6	685.0090	3	31.27	29	1601	OB4057.raw	1.08E5	1	1	82	98	
N.APQ(+.98)EIFIQQGR.G	N	53.97	1286.6619	11	-0.2	644.3381	2	28.64	29	1452	OB4057.raw	6.63E3	1	1	88	98	Deamidation (NQ)

Peptide	Uniq	-10lgP	Mass	Length	ppm	m/z	z	RT	Fraction	Scan	Source File	Area Digest 7. traka 25-27 kDa	#Feature	#Feature Digest 7. traka 25-27 kDa	Start	End	PTM
S.ISFRQQPEENAC(+57.02)QFQR.L	N	53.95	2036.9486	16	-1.1	679.9894	3	26.44	29	1318	OB4057.raw	5.84E3	1	1	21	36	Carbamidomethylation
S.ISFRQQ(+.98)PEENAC(+57.02)QFQR.L	N	53.83	2037.9326	16	-0.2	680.3180	3	26.89	29	1344	OB4057.raw	3.68E4	1	1	21	36	Carbamidomethylation
S.ISFRQQ(+.98)PEENAC(+57.02)Q(+.98)FQR.L	N	52.87	2038.9166	16	0.1	680.6462	3	27.25	29	1372	OB4057.raw	1.04E5	1	1	21	36	Carbamidomethylation; Deamidation (NQ)
S.ISFRQ(+.98)QPEEN(+.98)AC(+57.02)QFQR.L	N	52.86	2038.9166	16	0.1	680.6462	3	27.25	29	1361	OB4057.raw	1.04E5	1	1	21	36	Deamidation (NQ); Carbamidomethylation
R.RPFYSN(+.98)APQ(+.98)EIFIQ(+.98)QGR.G	N	51.51	2052.9905	17	-0.9	685.3369	3	31.47	29	1634	OB4057.raw	1.41E4	1	1	82	98	Deamidation (NQ)
R.SLPYSP(+15.99)YSP(+15.99)QTQ(+.98)PKQ(+.98)E.D	Y	49.80	1882.8472	16	0.2	942.4310	2	26.59	29	1323	OB4057.raw	7.29E3	1	1	217	232	Hydroxylation Pro; Deamidation (NQ)
R.RFN(+.98)LAGNHEQ(+.98)EFLR.Y	N	49.63	1731.8329	14	-0.2	578.2848	3	28.72	29	1459	OB4057.raw	1.88E4	1	1	195	208	Deamidation (NQ)
R.LN(+.98)AQRPDNRIESEGGYIETWN(+.98)PNNQEFEC(+57.02)AGVALSR.L	N	49.62	4135.8931	36	1.6	1034.9822	4	31.82	29	1652	OB4057.raw	0	0	0	37	72	Carbamidomethylation
V.SLTDTNN(+.98)N(+.98)DN(+.98)QLDQ(+.98)FPR.R	N	49.16	1994.8341	17	4.1	998.4285	2	30.46	29	1566	OB4057.raw	0	0	0	178	194	Deamidation (NQ)
R.LNAQ(+.98)RPDNRIESEGGYIETWNPN(+.98)N(+.98)Q(+.98)EFEC(+57.02)AGVALSR.L	N	48.61	4137.8608	36	6.8	1035.4795	4	32.47	29	1691	OB4057.raw	0	0	0	37	72	Carbamidomethylation
R.RFN(+.98)LAGN(+.98)HEQ(+.98)EFLR.Y	N	47.43	1732.8169	14	-2.3	578.6116	3	29.08	29	1481	OB4057.raw	4.14E3	1	1	195	208	Deamidation (NQ)
R.LNAQ(+.98)RPDN(+.98)RIESEGGYIETWNPNN(+.98)Q(+.98)EFEC(+57.02)AGVALSR.L	N	47.19	4137.8608	36	5.5	1035.4781	4	32.37	29	1685	OB4057.raw	0	0	0	37	72	Carbamidomethylation
S.ISFRQ(+.98)Q(+.98)PEENAC(+57.02)Q(+.98)FQR.L	N	46.46	2039.9006	16	-0.8	680.9736	3	27.61	29	1392	OB4057.raw	1.5E5	1	1	21	36	Deamidation (NQ); Carbamidomethylation
S.ISFRQ(+.98)QPEEN(+.98)AC(+57.02)Q(+.98)FQR.L	N	46.46	2039.9006	16	-7.2	680.9692	3	28.84	29	1464	OB4057.raw	0	0	0	21	36	Carbamidomethylation; Deamidation (NQ)
R.GYFGLIFPGC(+57.02)PSTYEEPAQ(+.98)Q(+.98)G.R	N	46.43	2319.0042	21	1.4	1160.5110	2	37.30	29	1999	OB4057.raw	1.95E4	1	1	99	119	Carbamidomethylation; Deamidation (NQ)
G.GYIETWNPN(+.98)NQEFEC(+57.02)AGVALSR.L	N	46.05	2555.1387	22	1.9	852.7218	3	33.01	29	1724	OB4057.raw	0	0	0	51	72	Carbamidomethylation
R.GYFGLIFPGC(+57.02)PSTYEEPAQ(+.98)Q(+.98).G	N	45.23	2261.9827	20	1.9	1132.0007	2	37.44	29	2005	OB4057.raw	2.43E3	1	1	99	118	Carbamidomethylation; Deamidation (NQ)
R.GYFGLIFPGC(+57.02)PSTYEEPA.Q	N	45.16	2003.8975	18	1.1	1002.9572	2	37.61	29	2011	OB4057.raw	6.78E3	1	1	99	116	Carbamidomethylation
R.GYFGLIFPGC(+57.02)PSTYEEPAQ(+.98)QGRR.H	N	45.10	2630.2224	23	0.2	877.7482	3	33.53	29	1771	OB4057.raw	2.51E5	2	2	99	121	Carbamidomethylation
S.ISFRQ(+.98)Q(+.98)PEEN(+.98)AC(+57.02)QFQ(+.98)R.L	N	44.56	2040.8846	16	0.7	681.3026	3	28.00	29	1419	OB4057.raw	6.08E4	2	2	21	36	Deamidation (NQ); Carbamidomethylation
R.LN(+.98)AQRPDN(+.98)RIESEGGYIETWNP(+15.99)NNQ(+.98)EFEC(+57.02)AGVALSR.L	N	43.32	4152.8721	36	7.7	1039.2333	4	31.96	29	1673	OB4057.raw	3.37E4	1	1	37	72	Deamidation (NQ); Carbamidomethylation
S.ISFRQQ(+.98)PEEN(+.98)AC(+57.02)QFQ(+.98)R.L	N	43.09	2039.9006	16	-0.8	680.9736	3	27.61	29	1428	OB4057.raw	1.5E5	1	1	21	36	Deamidation (NQ); Carbamidomethylation
R.GYFGLIFPGC(+57.02)PSTYEEPAQ(+.98)QG.R	N	42.45	2318.0200	21	0.9	1160.0183	2	37.15	29	1981	OB4057.raw	0	0	0	99	119	Carbamidomethylation
R.GYFGLIFPGC(+57.02)PSTYEEPAQ(+.98)Q(+.98)GRR.H	N	42.43	2631.2063	23	1.2	878.0771	3	33.54	29	1756	OB4057.raw	0	0	0	99	121	Carbamidomethylation; Deamidation (NQ)
Y.SNAPQ(+.98)EIFIQQGR.G	N	42.42	1487.7368	13	0.1	744.8757	2	29.44	29	1506	OB4057.raw	2.04E3	1	1	86	98	Deamidation (NQ)
R.GYFGLIFPGC(+57.02)PSTYEEPAQQ(+.98)GRR.H	N	42.26	2630.2224	23	0.7	877.7487	3	33.34	29	1744	OB4057.raw	2.48E5	1	1	99	121	Carbamidomethylation
K.TANELNLLILR.W	N	42.07	1268.7452	11	-1.8	635.3787	2	33.72	29	1764	OB4057.raw	4.73E3	1	1	384	394	
S.ISFRQ(+.98)Q(+.98)PEENAC(+57.02)QFQR.L	N	41.78	2038.9166	16	0.1	680.6462	3	27.25	29	1432	OB4057.raw	1.04E5	1	1	21	36	Deamidation (NQ); Carbamidomethylation
R.RFN(+.98)LAGN(+.98)HEQ(+.98)EFLRYQQ(+.98)Q(+.98)SR.R	N	41.31	2525.1570	20	4.0	632.2991	4	29.73	29	1519	OB4057.raw	0	0	0	195	214	Deamidation (NQ)
R.GYFGLIFP(+15.99)GC(+57.02)PSTYEEPAQQGR.R	N	41.18	2489.1321	22	1.5	830.7192	3	35.31	29	1863	OB4057.raw	3.8E3	1	1	99	120	Carbamidomethylation
R.GYFGLIFPGC(+57.02)P(+15.99)STYEEPAQQGR.R	N	40.91	2489.1321	22	1.5	830.7192	3	35.31	29	1878	OB4057.raw	3.8E3	1	1	99	120	Carbamidomethylation
R.LN(+.98)AQRPDNRIESEGGYIETWNPNNQEFEC(+57.02)AGVALSR.L	N	40.89	4134.9087	36	3.0	1034.7375	4	31.51	29	1640	OB4057.raw	1.17E5	1	1	37	72	Carbamidomethylation

Peptide	Uniq	-10lgP	Mass	Length	ppm	m/z	z	RT	Fraction	Scan	Source File	Area Digest 7. traka 25-27 kDa	#Feature	#Feature Digest 7. traka 25-27 kDa	Start	End	PTM
S.ISFRQ(+.98)Q(+.98)PEENAC(+57.02)QFQ(+.98)R.L	N	40.78	2039.9006	16	0.8	680.9747	3	28.66	29	1453	OB4057.raw	0	0	0	21	36	Deamidation (NQ); Carbamidomethylation
R.LNAQ(+.98)RPDNRIESEGGYIETWNPNNQEFEC(+57.02)AGVALSR.L	N	40.63	4134.9087	36	3.0	1034.7375	4	31.51	29	1628	OB4057.raw	1.17E5	1	1	37	72	Carbamidomethylation
R.GYFGLIFPGC(+57.02)PSTYEE.P	N	40.42	1835.8076	16	0.2	918.9113	2	37.63	29	2015	OB4057.raw	1.67E3	1	1	99	114	Carbamidomethylation
R.GYFGLIFPGC(+57.02)PSTYEEPAQQGRR.H	N	40.40	2629.2383	23	-7.0	877.4139	3	33.26	29	1739	OB4057.raw	0	0	0	99	121	Carbamidomethylation
N.APQ(+.98)EIFIQ(+.98)QGR.G	N	40.15	1287.6459	11	-0.6	644.8298	2	29.21	29	1486	OB4057.raw	1.52E3	1	1	88	98	Deamidation (NQ)
S.ISFRQ(+.98)Q(+.98)PEEN(+.98)AC(+57.02)Q(+.98)FQ(+.98)R.L	N	39.36	2041.8687	16	-3.1	681.6281	3	28.45	29	1440	OB4057.raw	3.98E3	1	1	21	36	Deamidation (NQ); Carbamidomethylation
R.SPDIYNPQ(+.98)AGSLK.T	N	39.27	1389.6776	13	1.0	695.8468	2	28.06	29	1416	OB4057.raw	0	0	0	371	383	
R.LNAQRPDN(+.98)RIESEGGYIETWN(+.98)PN(+.98)N(+.98)QEFEC(+57.02)AGVALSR.L	N	39.23	4137.8608	36	-1.3	1035.4712	4	32.66	29	1703	OB4057.raw	0	0	0	37	72	Carbamidomethylation
R.LN(+.98)AQRPDNRIESEGGYIETWNPNN(+.98)Q(+.98)EFEC(+57.02)AGVALSR.L	N	39.15	4136.8770	36	2.4	1035.2290	4	33.65	29	1763	OB4057.raw	0	0	0	37	72	Carbamidomethylation
R.LN(+.98)AQRPDNRIESEGGYIETWN(+.98)PN(+.98)NQEFEC(+57.02)AGVALSR.L	N	38.91	4136.8770	36	6.5	1035.2333	4	31.90	29	1708	OB4057.raw	3.94E5	1	1	37	72	Carbamidomethylation
R.LN(+.98)AQRPDNRIESEGGYIETWNPN(+.98)N(+.98)QEFEC(+57.02)AGVALSR.L	N	38.53	4136.8770	36	0.8	1035.2273	4	34.69	29	1826	OB4057.raw	0	0	0	37	72	Carbamidomethylation
R.GYFGLIFPGC(+57.02)PSTYEEP(+15.99)AQQGRR.H	N	38.08	2645.2332	23	-4.2	882.7480	3	33.96	29	1777	OB4057.raw	4.66E4	1	1	99	121	Carbamidomethylation
R.RFNLAGN(+.98)HEQEFLRYQ(+.98)Q(+.98)QSR.R	N	37.97	2523.1890	20	-0.5	631.8042	4	28.35	29	1434	OB4057.raw	0	0	0	195	214	
K.TDSRPSIANLAGENSFIDN(+.98)LPEEVVANSYGLPR.E	N	37.62	3545.7273	33	-0.5	1182.9158	3	36.13	29	1914	OB4057.raw	0	0	0	477	509	
R.NALRRPFYSNAPQ(+.98)EIFIQQGR.G	N	37.39	2505.2876	21	-8.9	836.0957	3	33.41	29	1748	OB4057.raw	8.09E5	1	1	78	98	
R.LN(+.98)AQRPDNRIESEGGYIETWNP(+15.99)NN(+.98)QEFEC(+57.02)AGVALSR.L	N	36.67	4151.8877	36	6.4	1038.9858	4	32.27	29	1679	OB4057.raw	0	0	0	37	72	Carbamidomethylation
K.TAN(+.98)ELN(+.98)LLILRWLGLSAEYGNLYR.N	N	36.48	2793.4700	24	-1.9	932.1622	3	40.89	29	2223	OB4057.raw	0	0	0	384	407	Deamidation (NQ)
R.LNAQ(+.98)RPDNRIESEGGYIETWN(+.98)PNN(+.98)Q(+.98)EFEC(+57.02)AGVALSR.L	N	36.45	4137.8608	36	1.9	1035.4745	4	32.59	29	1698	OB4057.raw	0	0	0	37	72	Carbamidomethylation
R.RFN(+.98)LAGNHEQ(+.98)EFLRYQ(+.98)Q(+.98)QSR.R	N	35.14	2524.1731	20	-0.3	632.0504	4	29.48	29	1504	OB4057.raw	0	0	0	195	214	
R.GYFGLIFPGC(+57.02)P(+15.99)STYEEPAQQ(+.98)GRR.H	N	34.17	2646.2173	23	0.8	883.0804	3	34.27	29	1801	OB4057.raw	0	0	0	99	121	Carbamidomethylation
R.R(+43.01)(+14.02)PFYSNAPQEIFIQQGR.G	N	33.80	2107.0598	17	0.4	703.3608	3	30.09	29	1541	OB4057.raw	2.68E3	1	1	82	98	Carbamylation; Methylation(KR)
R.RFN(+.98)LAGNHEQ(+.98)EFLRYQ(+.98)Q(+.98)Q(+.98)SR.R	N	33.42	2525.1570	20	7.9	632.3015	4	29.03	29	1480	OB4057.raw	2.4E4	1	1	195	214	
R.GYFGLIFPGC(+57.02)P(+15.99)STYEEPAQQGRR.H	N	33.29	2645.2332	23	-4.2	882.7480	3	33.96	29	1772	OB4057.raw	4.66E4	1	1	99	121	Carbamidomethylation
R.SPDIYNPQAGSLKTANELNLLILRWLGLSAEYGNLYR.N	N	32.64	4162.1851	37	-2.9	1041.5505	4	39.87	29	2158	OB4057.raw	0	0	0	371	407	
S.ISFRQ(+.98)Q(+.98)PEEN(+.98)AC(+57.02)Q(+.98)FQR.L	N	32.58	2040.8846	16	0.0	681.3021	3	28.64	29	1453	OB4057.raw	2.82E3	1	1	21	36	Deamidation (NQ); Carbamidomethylation
K.TDSRPSIANLAGEN(+.98)SFIDN(+.98)LPEEVVANSYGLPREQ(+.98)AR.Q	N	31.61	4031.9348	37	1.7	1008.9927	4	35.16	29	1855	OB4057.raw	0	0	0	477	513	
R.RFNLAGN(+.98)HEQ(+.98)EFLRYQ(+.98)QQSR.R	N	31.37	2523.1890	20	1.0	631.8052	4	28.55	29	1446	OB4057.raw	0	0	0	195	214	
S.ISFRQ(+.98)Q(+.98)PEEN(+.98)AC(+57.02)QFQR.L	N	31.15	2039.9006	16	-2.4	680.9725	3	29.92	29	1531	OB4057.raw	0	0	0	21	36	Carbamidomethylation
R.LNAQRPDNRIESEGGYIETWNP(+15.99)NNQEFEC(+57.02)AGVALSR.L	N	31.12	4149.9199	36	1.9	1038.4893	4	31.82	29	1673	OB4057.raw	6.01E4	1	1	37	72	Carbamidomethylation
R.GYFGLIFP(+15.99)GC(+57.02)PSTYEEPAQQ(+.98)GR.R	N	30.90	2490.1160	22	-1.5	831.0447	3	35.67	29	1886	OB4057.raw	0	0	0	99	120	Carbamidomethylation
R.RFNLAGN(+.98)HEQ(+.98)EFLRYQ(+.98)Q(+.98)QSR.R	N	30.35	2524.1731	20	-2.5	632.0490	4	28.87	29	1466	OB4057.raw	0	0	0	195	214	
R.RFN(+.98)LAGN(+.98)HEQEFLRYQ(+.98)QQ(+.98)SR.R	N	30.27	2524.1731	20	3.7	632.0529	4	29.09	29	1480	OB4057.raw	0	0	0	195	214	Deamidation (NQ)
S.ISFRQQ(+.98)PEEN(+.98)AC(+57.02)QFQR.L	N	30.06	2038.9166	16	-1.3	680.6453	3	29.22	29	1488	OB4057.raw	0	0	0	21	36	Carbamidomethylation
R.RFNLAGNHEQ(+.98)EFLRYQ(+.98)Q(+.98)QSR.R	N	29.87	2523.1890	20	9.8	631.8107	4	28.40	29	1437	OB4057.raw	0	0	0	195	214	
R.SPDIYN(+.98)PQAGSLKTANELNLLILRWLGLSAEYGNLYR.N	N	29.56	4163.1689	37	1.2	1041.8008	4	39.78	29	2164	OB4057.raw	2.62E4	1	1	371	407	
R.RFNLAGN(+.98)HEQEFLRYQQQ(+.98)SR.R	N	29.43	2522.2051	20	0.8	631.5590	4	28.08	29	1417	OB4057.raw	0	0	0	195	214	
R.GYFGLIFPGC(+57.02)P(+15.99)STYEEP(+15.99)AQQGRR.H	N	29.12	2661.2280	23	-4.0	888.0797	3	33.53	29	1750	OB4057.raw	2.5E4	1	1	99	121	Carbamidomethylation

Peptide	Uniq	-10lgP	Mass	Length	ppm	m/z	z	RT	Fraction	Scan	Source File	Area Digest 7. traka 25-27 kDa	#Feature	#Feature Digest 7. traka 25-27 kDa	Start	End	PTM
R.SPDIYNPQAGSLKTAN(+.98)ELNLLILRWLGLSAEYGNLYR.N	N	28.83	4163.1689	37	1.2	1041.8008	4	39.78	29	2178	OB4057.raw	2.62E4	1	1	371	407	
K.TDSRPSIAN(+.98)LAGENSFIDN(+.98)LPEEVVANSYGLPREQAR.Q	N	28.27	4030.9507	37	3.3	1008.7482	4	34.85	29	1869	OB4057.raw	3.58E4	1	1	477	513	
R.LN(+.98)AQ(+.98)RPDNRIESEGGYIETWNP(+15.99)NNQ(+.98)EFEC(+57.02)AGVALSR.L	N	28.15	4152.8721	36	7.7	1039.2333	4	31.96	29	1668	OB4057.raw	3.37E4	1	1	37	72	Carbamidomethylation
R.RFN(+.98)LAGNHEQ(+.98)EFLRYQQ(+.98)Q(+.98)SR.R	N	28.03	2524.1731	20	-3.8	632.0482	4	29.55	29	1508	OB4057.raw	0	0	0	195	214	
R.GYFGLIFPGC(+57.02)PSTYEEP(+15.99)AQ(+.98)QGRR.H	N	27.80	2646.2173	23	2.4	883.0818	3	34.48	29	1814	OB4057.raw	0	0	0	99	121	Carbamidomethylation
R.LN(+.98)AQ(+.98)RPDNRIESEGGYIETWNP(+15.99)NNQEFEC(+57.02)AGVALSR.L	N	27.49	4151.8877	36	-1.1	1038.9780	4	32.39	29	1686	OB4057.raw	0	0	0	37	72	Carbamidomethylation
R.RFNLAGN(+.98)HEQ(+.98)EFLRYQ(+.98)Q(+.98)Q(+.98)SR.R	N	27.28	2525.1570	20	7.9	632.3015	4	29.03	29	1466	OB4057.raw	2.4E4	1	1	195	214	
R.R(+42.01)P(+15.99)FYSNAPQEIFIQQGR.G	N	26.80	2108.0439	17	-0.7	703.6881	3	30.76	29	1577	OB4057.raw	3.53E3	1	1	82	98	Acetylation (N-term)
R.GYFGLIFPGC(+57.02)P(+15.99)STYEEP(+15.99)AQQ(+.98)GRR.H	N	26.49	2662.2122	23	-5.4	888.4066	3	33.81	29	1773	OB4057.raw	0	0	0	99	121	Carbamidomethylation
R.LN(+.98)AQRPDNRIESEGGYIETWNPNNQ(+.98)EFEC(+57.02)AGVALSR.L	N	26.34	4135.8931	36	-4.7	1034.9757	4	33.17	29	1734	OB4057.raw	0	0	0	37	72	Carbamidomethylation
R.LN(+.98)AQRPDNRIESEGGYIETWN(+.98)PNN(+.98)Q(+.98)EFEC(+57.02)AGVALSR.L	N	26.06	4137.8608	36	-0.7	1035.4718	4	33.49	29	1753	OB4057.raw	0	0	0	37	72	Carbamidomethylation
R.N(+.98)ALRRPFYSN(+.98)APQEIFIQ(+.98)Q(+.98)GR.G	N	25.99	2508.2397	21	8.4	837.0942	3	31.36	29	1621	OB4057.raw	1.89E4	1	1	78	98	
R.NALRRPFYSN(+.98)APQ(+.98)EIFIQQ(+.98)GR.G	N	25.95	2507.2556	21	9.1	836.7667	3	32.01	29	1663	OB4057.raw	0	0	0	78	98	
N.Q(-17.03)EFEC(+57.02)AGVALSR.L	N	25.82	1348.6082	12	2.6	675.3131	2	31.68	29	1643	OB4057.raw	1.38E3	1	1	61	72	Pyro-glu from Q; Carbamidomethylation
R.GYFGLIFPGC(+57.02)PSTYEEPAQQGR(+31.99)R.H	N	25.71	2661.2280	23	-3.6	888.0801	3	33.19	29	1735	OB4057.raw	0	0	0	99	121	Carbamidomethylation
R.LNAQRPDNRIESEGGYIETWNPNNQEFEC(+57.02)AGVALSR.L	N	25.66	4133.9248	36	1.7	1034.4902	4	31.34	29	1622	OB4057.raw	0	0	0	37	72	Carbamidomethylation
R.RFN(+.98)LAGN(+.98)HEQEFLRYQ(+.98)Q(+.98)Q(+.98)SR.R	N	25.15	2525.1570	20	-5.6	632.2930	4	29.68	29	1516	OB4057.raw	2.4E3	1	1	195	214	
R.LN(+.98)AQRP(+15.99)DNRIESEGGYIETWNPNN(+.98)QEFEC(+57.02)AGVALSR.L	N	25.01	4151.8877	36	-8.1	1038.9708	4	32.88	29	1716	OB4057.raw	0	0	0	37	72	Carbamidomethylation
R.R(+42.01)(+14.02)PFYSNAPQEIFIQQGR.G	N	24.55	2106.0647	17	-9.4	703.0222	3	30.07	29	1541	OB4057.raw	0	0	0	82	98	Acetylation (N-term); Methylation(KR)
R.SPDIYNPQ(+.98)AGSLKTANELN(+.98)LLILRWLGLSAEYGNLYR.N	N	24.48	4164.1528	37	4.6	1042.0503	4	40.02	29	2168	OB4057.raw	0	0	0	371	407	
K.TDSRPSIANLAGENSFIDN(+.98)LPEEVVANSYGLPREQAR.Q	N	24.32	4029.9668	37	-4.5	1008.4944	4	35.02	29	1847	OB4057.raw	0	0	0	477	513	
R.LNAQRPD(+21.98)NRIESEGGY(-18.01)IETWNPNNQEFEC(+57.02)AGVALSR.L	N	23.34	4137.8965	36	1.7	1035.4832	4	33.31	29	1742	OB4057.raw	0	0	0	37	72	Carbamidomethylation
R.RFNLAGNHEQ(+.98)EFLRYQQ(+.98)QSR.R	N	23.33	2522.2051	20	-0.7	631.5581	4	28.18	29	1423	OB4057.raw	0	0	0	195	214	
R.GYFGLIFPGC(+57.02)P(+15.99)STYEEP(+15.99)AQ(+.98)Q(+.98)GRR.H	N	23.01	2663.1960	23	-5.1	888.7348	3	34.20	29	1797	OB4057.raw	0	0	0	99	121	Carbamidomethylation; Deamidation (NQ)
G.GYIETWN(+.98)PNN(+.98)QEFEC(+57.02)AGVALSR.L	N	22.78	2556.1226	22	0.7	853.0487	3	33.23	29	1746	OB4057.raw	8.99E3	1	1	51	72	Carbamidomethylation
**total 130 peptides**																	

tr|A1DZF0|A1DZF0_ARAHY

back to list

| Protein Coverage | Supporting Peptides |

Protein Coverage:

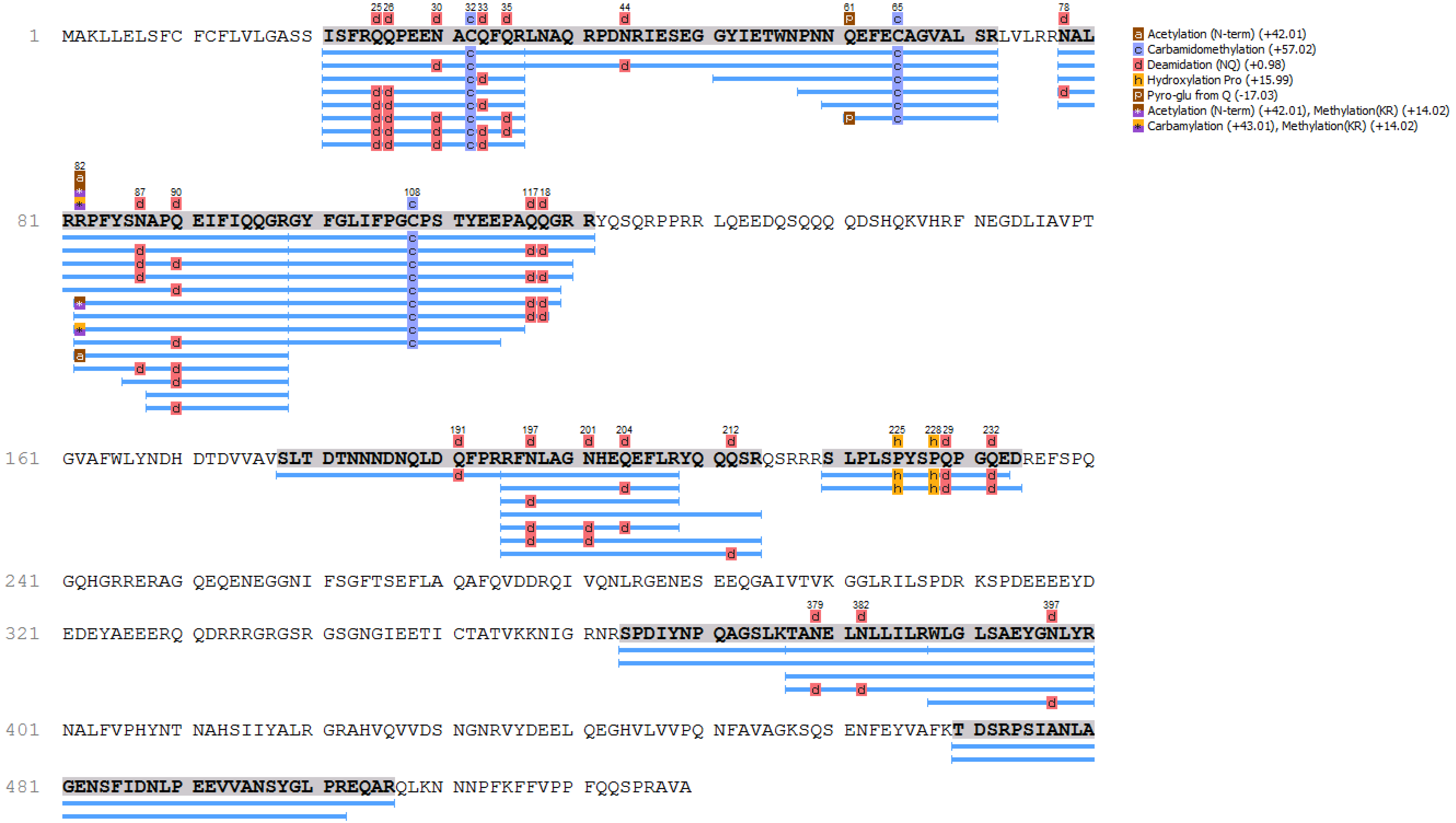

Supporting Peptides:

Peptide	Uniq	-10lgP	Mass	Length	ppm	m/z	z	RT	Fraction	Scan	Source File	Area Digest 7. traka 25-27 kDa	#Feature	#Feature Digest 7. traka 25-27 kDa	Start	End	PTM
R.WLGLSAEYGNLYR.N	N	93.88	1540.7673	13	0.7	771.3915	2	33.33	29	1738	OB4057.raw	2.5E4	1	1	388	400	
R.GYFGLIFPGC(+57.02)PSTYEEPAQQGR.R	N	77.03	2473.1372	22	1.6	1237.5779	2	34.90	29	1839	OB4057.raw	0	0	0	99	120	Carbamidomethylation

Peptide	Uniq	-10lgP	Mass	Length	ppm	m/z	z	RT	Fraction	Scan	Source File	Area Digest 7. traka 25-27 kDa	#Feature	#Feature Digest 7. traka 25-27 kDa	Start	End	PTM
R.NALRRPFYSN(+.98)APQEIFIQQGR.G	N	72.96	2505.2876	21	0.8	836.1038	3	30.04	29	1537	OB4057.raw	1.34E5	2	2	78	98	Deamidation (NQ)
R.RPFYSNAPQEIFIQQGR.G	N	72.96	2050.0383	17	0.6	684.3538	3	30.09	29	1546	OB4057.raw	1.76E5	1	1	82	98	
R.NALRRPFYSNAPQEIFIQQGR.G	N	71.98	2504.3037	21	-0.5	835.7748	3	29.54	29	1503	OB4057.raw	5.44E5	2	2	78	98	
K.TANELNLLILRWLGLSAEYGNLYR.N	N	70.48	2791.5020	24	0.7	931.5086	3	39.84	29	2175	OB4057.raw	2.08E4	1	1	377	400	
R.RPFYSNAPQ(+.98)EIFIQQGR.G	N	70.26	2051.0225	17	0.0	684.6815	3	30.74	29	1580	OB4057.raw	2.52E5	1	1	82	98	Deamidation (NQ)
N.NQ(+.98)EFEC(+57.02)AGVALSR.L	N	69.12	1480.6616	13	0.2	741.3383	2	28.82	29	1460	OB4057.raw	5.61E3	1	1	60	72	Carbamidomethylation
R.RFNLAGNHEQ(+.98)EFLR.Y	N	67.53	1730.8488	14	-1.5	577.9560	3	28.20	29	1404	OB4057.raw	4.97E4	1	1	195	208	Deamidation (NQ)
R.RFNLAGN(+.98)HEQ(+.98)EFLR.Y	N	65.72	1731.8329	14	9.2	578.2902	3	28.98	29	1473	OB4057.raw	0	0	0	195	208	Deamidation (NQ)
R.RPFYSN(+.98)APQ(+.98)EIFIQQGR.G	N	65.72	2052.0063	17	-0.6	685.0090	3	31.27	29	1614	OB4057.raw	1.11E5	2	2	82	98	Deamidation (NQ)
N.PN(+.98)NQ(+.98)EFEC(+57.02)AGVALSR.L	N	63.50	1692.7413	15	-1.0	847.3771	2	29.54	29	1509	OB4057.raw	2.96E3	1	1	58	72	Carbamidomethylation
R.RFN(+.98)LAGNHEQEFLR.Y	N	62.67	1730.8488	14	-1.5	577.9560	3	28.20	29	1449	OB4057.raw	4.97E4	1	1	195	208	Deamidation (NQ)
R.RFNLAGNHEQEFLR.Y	N	62.20	1729.8647	14	-0.6	577.6285	3	27.45	29	1373	OB4057.raw	2.42E4	1	1	195	208	
K.TAN(+.98)ELNLLILRWLGLSAEYGNLYR.N	N	62.19	2792.4861	24	1.5	931.8374	3	40.46	29	2201	OB4057.raw	4.9E3	1	1	377	400	
R.RPFYSNAPQEIFIQQ(+.98)GR.G	N	61.45	2051.0225	17	0.0	684.6815	3	30.74	29	1558	OB4057.raw	2.52E5	1	1	82	98	
R.NALRRPFYSN(+.98)APQ(+.98)EIFIQQGR.G	N	61.01	2506.2717	21	0.8	836.4319	3	30.86	29	1581	OB4057.raw	1.12E5	1	1	78	98	Deamidation (NQ)
R.N(+.98)ALRRPFYSNAPQ(+.98)EIFIQQGR.G	N	59.37	2506.2717	21	-1.6	836.4299	3	31.54	29	1635	OB4057.raw	6.41E3	1	1	78	98	Deamidation (NQ)
R.GYFGLIFPGC(+57.02)PSTYEEPAQ(+.98)Q(+.98)GR.R	N	58.83	2475.1052	22	2.9	826.0447	3	35.24	29	1860	OB4057.raw	5.56E4	1	1	99	120	Carbamidomethylation; Deamidation (NQ)
R.GYFGLIFPGC(+57.02)PSTYEEPAQ(+.98)QGR.R	N	58.17	2474.1211	22	0.7	825.7148	3	35.01	29	1841	OB4057.raw	5.09E4	2	2	99	120	Carbamidomethylation
N.APQEIFIQQGR.G	N	57.98	1285.6779	11	-0.2	643.8461	2	28.00	29	1415	OB4057.raw	2.55E3	1	1	88	98	
K.TANELN(+.98)LLILRWLGLSAEYGNLYR.N	N	57.78	2792.4861	24	1.5	931.8374	3	40.46	29	2195	OB4057.raw	4.9E3	1	1	377	400	
R.WLGLSAEYGN(+.98)LYR.N	N	56.70	1541.7513	13	-1.0	771.8821	2	33.70	29	1766	OB4057.raw	0	0	0	388	400	Deamidation (NQ)
R.N(+.98)ALRRPFYSN(+.98)APQEIFIQQGR.G	N	56.60	2506.2717	21	0.8	836.4319	3	30.86	29	1598	OB4057.raw	1.19E5	2	2	78	98	Deamidation (NQ)
R.NALRRPFYSN(+.98)APQEIFIQQ(+.98)GR.G	N	56.36	2506.2717	21	0.8	836.4319	3	30.86	29	1575	OB4057.raw	1.12E5	1	1	78	98	
S.ISFRQ(+.98)QPEENAC(+57.02)QFQR.L	N	55.40	2037.9326	16	-0.2	680.3180	3	26.89	29	1336	OB4057.raw	3.68E4	1	1	21	36	Carbamidomethylation
R.LNAQ(+.98)RPDNRIESEGGYIETWN(+.98)PNNQ(+.98)EFEC(+57.02)AGVALSR.L	N	55.37	4136.8770	36	6.5	1035.2333	4	31.90	29	1667	OB4057.raw	3.94E5	1	1	37	72	Carbamidomethylation
R.RFNLAGN(+.98)HEQEFLR.Y	N	54.72	1730.8488	14	-1.5	577.9560	3	28.20	29	1420	OB4057.raw	4.97E4	1	1	195	208	
R.RPFYSN(+.98)APQEIFIQQGR.G	N	54.60	2051.0225	17	0.0	684.6815	3	30.74	29	1688	OB4057.raw	2.52E5	1	1	82	98	
R.RPFYSNAPQ(+.98)EIFIQ(+.98)QGR.G	N	54.53	2052.0063	17	-0.6	685.0090	3	31.27	29	1601	OB4057.raw	1.08E5	1	1	82	98	
N.APQ(+.98)EIFIQQGR.G	N	53.97	1286.6619	11	-0.2	644.3381	2	28.64	29	1452	OB4057.raw	6.63E3	1	1	88	98	Deamidation (NQ)
S.ISFRQQPEENAC(+57.02)QFQR.L	N	53.95	2036.9486	16	-1.1	679.9894	3	26.44	29	1318	OB4057.raw	5.84E3	1	1	21	36	Carbamidomethylation
S.ISFRQQ(+.98)PEENAC(+57.02)QFQR.L	N	53.83	2037.9326	16	-0.2	680.3180	3	26.89	29	1344	OB4057.raw	3.68E4	1	1	21	36	Carbamidomethylation
S.ISFRQQ(+.98)PEENAC(+57.02)Q(+.98)FQR.L	N	52.87	2038.9166	16	0.1	680.6462	3	27.25	29	1372	OB4057.raw	1.04E5	1	1	21	36	Carbamidomethylation; Deamidation (NQ)
S.ISFRQ(+.98)QPEEN(+.98)AC(+57.02)QFQR.L	N	52.86	2038.9166	16	0.1	680.6462	3	27.25	29	1361	OB4057.raw	1.04E5	1	1	21	36	Deamidation (NQ); Carbamidomethylation
R.SLPLSP(+15.99)YSP(+15.99)Q(+.98)PGQ(+.98)ED.R	Y	52.54	1647.7151	15	-1.8	824.8633	2	29.90	29	1530	OB4057.raw	0	0	0	220	234	Hydroxylation Pro; Deamidation (NQ)
R.RPFYSN(+.98)APQ(+.98)EIFIQ(+.98)QGR.G	N	51.51	2052.9905	17	-0.9	685.3369	3	31.47	29	1634	OB4057.raw	1.41E4	1	1	82	98	Deamidation (NQ)
R.RFN(+.98)LAGNHEQ(+.98)EFLR.Y	N	49.63	1731.8329	14	-0.2	578.2848	3	28.72	29	1459	OB4057.raw	1.88E4	1	1	195	208	Deamidation (NQ)
R.LN(+.98)AQRPDNRIESEGGYIETWN(+.98)PNNQEFEC(+57.02)AGVALSR.L	N	49.62	4135.8931	36	1.6	1034.9822	4	31.82	29	1652	OB4057.raw	0	0	0	37	72	Carbamidomethylation

Peptide	Uniq	-10lgP	Mass	Length	ppm	m/z	z	RT	Fraction	Scan	Source File	Area Digest 7. traka 25-27 kDa	#Feature	#Feature Digest 7. traka 25-27 kDa	Start	End	PTM
V.SLTDTNN(+.98)N(+.98)DN(+.98)QLDQ(+.98)FPR.R	N	49.16	1994.8341	17	4.1	998.4285	2	30.46	29	1566	OB4057.raw	0	0	0	178	194	Deamidation (NQ)
R.LNAQ(+.98)RPDNRIESEGGYIETWNPN(+.98)N(+.98)Q(+.98)EFEC(+57.02)AGVALSR.L	N	48.61	4137.8608	36	6.8	1035.4795	4	32.47	29	1691	OB4057.raw	0	0	0	37	72	Carbamidomethylation
R.RFN(+.98)LAGN(+.98)HEQ(+.98)EFLR.Y	N	47.43	1732.8169	14	-2.3	578.6116	3	29.08	29	1481	OB4057.raw	4.14E3	1	1	195	208	Deamidation (NQ)
R.LNAQ(+.98)RPDN(+.98)RIESEGGYIETWNPNN(+.98)Q(+.98)EFEC(+57.02)AGVALSR.L	N	47.19	4137.8608	36	5.5	1035.4781	4	32.37	29	1685	OB4057.raw	0	0	0	37	72	Carbamidomethylation
S.ISFRQ(+.98)Q(+.98)PEENAC(+57.02)Q(+.98)FQR.L	N	46.46	2039.9006	16	-0.8	680.9736	3	27.61	29	1392	OB4057.raw	1.5E5	1	1	21	36	Deamidation (NQ); Carbamidomethylation
S.ISFRQ(+.98)QPEEN(+.98)AC(+57.02)Q(+.98)FQR.L	N	46.46	2039.9006	16	-7.2	680.9692	3	28.84	29	1464	OB4057.raw	0	0	0	21	36	Carbamidomethylation; Deamidation (NQ)
R.GYFGLIFPGC(+57.02)PSTYEEPAQ(+.98)Q(+.98)G.R	N	46.43	2319.0042	21	1.4	1160.5110	2	37.30	29	1999	OB4057.raw	1.95E4	1	1	99	119	Carbamidomethylation; Deamidation (NQ)
G.GYIETWNPN(+.98)NQEFEC(+57.02)AGVALSR.L	N	46.05	2555.1387	22	1.9	852.7218	3	33.01	29	1724	OB4057.raw	0	0	0	51	72	Carbamidomethylation
R.GYFGLIFPGC(+57.02)PSTYEEPAQ(+.98)Q(+.98).G	N	45.23	2261.9827	20	1.9	1132.0007	2	37.44	29	2005	OB4057.raw	2.43E3	1	1	99	118	Carbamidomethylation; Deamidation (NQ)
R.GYFGLIFPGC(+57.02)PSTYEEPA.Q	N	45.16	2003.8975	18	1.1	1002.9572	2	37.61	29	2011	OB4057.raw	6.78E3	1	1	99	116	Carbamidomethylation
R.GYFGLIFPGC(+57.02)PSTYEEPAQ(+.98)QGRR.Y	N	45.10	2630.2224	23	0.2	877.7482	3	33.53	29	1771	OB4057.raw	2.51E5	2	2	99	121	Carbamidomethylation
R.SLPLSP(+15.99)YSP(+15.99)Q(+.98)PGQ(+.98)E.D	Y	44.87	1532.6881	14	0.4	767.3516	2	29.81	29	1522	OB4057.raw	5.84E3	1	1	220	233	Hydroxylation Pro; Deamidation (NQ)
S.ISFRQ(+.98)Q(+.98)PEEN(+.98)AC(+57.02)QFQ(+.98)R.L	N	44.56	2040.8846	16	0.7	681.3026	3	28.00	29	1419	OB4057.raw	6.08E4	2	2	21	36	Deamidation (NQ); Carbamidomethylation
R.LN(+.98)AQRPDN(+.98)RIESEGGYIETWNP(+15.99)NNQ(+.98)EFEC(+57.02)AGVALSR.L	N	43.32	4152.8721	36	7.7	1039.2333	4	31.96	29	1673	OB4057.raw	3.37E4	1	1	37	72	Deamidation (NQ); Carbamidomethylation
S.ISFRQQ(+.98)PEEN(+.98)AC(+57.02)QFQ(+.98)R.L	N	43.09	2039.9006	16	-0.8	680.9736	3	27.61	29	1428	OB4057.raw	1.5E5	1	1	21	36	Deamidation (NQ); Carbamidomethylation
R.GYFGLIFPGC(+57.02)PSTYEEPAQ(+.98)QG.R	N	42.45	2318.0200	21	0.9	1160.0183	2	37.15	29	1981	OB4057.raw	0	0	0	99	119	Carbamidomethylation
R.GYFGLIFPGC(+57.02)PSTYEEPAQ(+.98)Q(+.98)GRR.Y	N	42.43	2631.2063	23	1.2	878.0771	3	33.54	29	1756	OB4057.raw	0	0	0	99	121	Carbamidomethylation; Deamidation (NQ)
Y.SNAPQ(+.98)EIFIQQGR.G	N	42.42	1487.7368	13	0.1	744.8757	2	29.44	29	1506	OB4057.raw	2.04E3	1	1	86	98	Deamidation (NQ)
R.GYFGLIFPGC(+57.02)PSTYEEPAQQ(+.98)GRR.Y	N	42.26	2630.2224	23	0.7	877.7487	3	33.34	29	1744	OB4057.raw	2.48E5	1	1	99	121	Carbamidomethylation
K.TANELNLLILR.W	N	42.07	1268.7452	11	-1.8	635.3787	2	33.72	29	1764	OB4057.raw	4.73E3	1	1	377	387	
S.ISFRQ(+.98)Q(+.98)PEENAC(+57.02)QFQR.L	N	41.78	2038.9166	16	0.1	680.6462	3	27.25	29	1432	OB4057.raw	1.04E5	1	1	21	36	Deamidation (NQ); Carbamidomethylation
R.RFN(+.98)LAGN(+.98)HEQ(+.98)EFLRYQQ(+.98)Q(+.98)SR.Q	N	41.31	2525.1570	20	4.0	632.2991	4	29.73	29	1519	OB4057.raw	0	0	0	195	214	Deamidation (NQ)
R.GYFGLIFP(+15.99)GC(+57.02)PSTYEEPAQQGR.R	N	41.18	2489.1321	22	1.5	830.7192	3	35.31	29	1863	OB4057.raw	3.8E3	1	1	99	120	Carbamidomethylation
R.GYFGLIFPGC(+57.02)P(+15.99)STYEEPAQQGR.R	N	40.91	2489.1321	22	1.5	830.7192	3	35.31	29	1878	OB4057.raw	3.8E3	1	1	99	120	Carbamidomethylation
R.LN(+.98)AQRPDNRIESEGGYIETWNPNNQEFEC(+57.02)AGVALSR.L	N	40.89	4134.9087	36	3.0	1034.7375	4	31.51	29	1640	OB4057.raw	1.17E5	1	1	37	72	Carbamidomethylation
S.ISFRQ(+.98)Q(+.98)PEENAC(+57.02)QFQ(+.98)R.L	N	40.78	2039.9006	16	0.8	680.9747	3	28.66	29	1453	OB4057.raw	0	0	0	21	36	Deamidation (NQ); Carbamidomethylation
R.LNAQ(+.98)RPDNRIESEGGYIETWNPNNQEFEC(+57.02)AGVALSR.L	N	40.63	4134.9087	36	3.0	1034.7375	4	31.51	29	1628	OB4057.raw	1.17E5	1	1	37	72	Carbamidomethylation
R.GYFGLIFPGC(+57.02)PSTYEE.P	N	40.42	1835.8076	16	0.2	918.9113	2	37.63	29	2015	OB4057.raw	1.67E3	1	1	99	114	Carbamidomethylation
R.GYFGLIFPGC(+57.02)PSTYEEPAQQGRR.Y	N	40.40	2629.2383	23	-7.0	877.4139	3	33.26	29	1739	OB4057.raw	0	0	0	99	121	Carbamidomethylation
N.APQ(+.98)EIFIQ(+.98)QGR.G	N	40.15	1287.6459	11	-0.6	644.8298	2	29.21	29	1486	OB4057.raw	1.52E3	1	1	88	98	Deamidation (NQ)
S.ISFRQ(+.98)Q(+.98)PEEN(+.98)AC(+57.02)Q(+.98)FQ(+.98)R.L	N	39.36	2041.8687	16	-3.1	681.6281	3	28.45	29	1440	OB4057.raw	3.98E3	1	1	21	36	Deamidation (NQ); Carbamidomethylation
R.SPDIYNPQ(+.98)AGSLK.T	N	39.27	1389.6776	13	1.0	695.8468	2	28.06	29	1416	OB4057.raw	0	0	0	364	376	

Peptide	Uniq	-10lgP	Mass	Length	ppm	m/z	z	RT	Fraction	Scan	Source File	Area Digest 7. traka 25-27 kDa	#Feature	#Feature Digest 7. traka 25-27 kDa	Start	End	PTM
R.LNAQRPDN(+.98)RIESEGGYIETWN(+.98)PN(+.98)N(+.98)QEFEC(+57.02)AGVALSR.L	N	39.23	4137.8608	36	-1.3	1035.4712	4	32.66	29	1703	OB4057.raw	0	0	0	37	72	Carbamidomethylation
R.LN(+.98)AQRPDNRIESEGGYIETWNPNN(+.98)Q(+.98)EFEC(+57.02)AGVALSR.L	N	39.15	4136.8770	36	2.4	1035.2290	4	33.65	29	1763	OB4057.raw	0	0	0	37	72	Carbamidomethylation
R.LN(+.98)AQRPDNRIESEGGYIETWN(+.98)PN(+.98)NQEFEC(+57.02)AGVALSR.L	N	38.91	4136.8770	36	6.5	1035.2333	4	31.90	29	1708	OB4057.raw	3.94E5	1	1	37	72	Carbamidomethylation
R.LN(+.98)AQRPDNRIESEGGYIETWNPN(+.98)N(+.98)QEFEC(+57.02)AGVALSR.L	N	38.53	4136.8770	36	0.8	1035.2273	4	34.69	29	1826	OB4057.raw	0	0	0	37	72	Carbamidomethylation
R.GYFGLIFPGC(+57.02)PSTYEEP(+15.99)AQQGRR.Y	N	38.08	2645.2332	23	-4.2	882.7480	3	33.96	29	1777	OB4057.raw	4.66E4	1	1	99	121	Carbamidomethylation
R.RFNLAGN(+.98)HEQEFLRYQ(+.98)Q(+.98)QSR.Q	N	37.97	2523.1890	20	-0.5	631.8042	4	28.35	29	1434	OB4057.raw	0	0	0	195	214	
K.TDSRPSIANLAGENSFIDN(+.98)LPEEVVANSYGLPR.E	N	37.62	3545.7273	33	-0.5	1182.9158	3	36.13	29	1914	OB4057.raw	0	0	0	470	502	
R.NALRRPFYSNAPQ(+.98)EIFIQQGR.G	N	37.39	2505.2876	21	-8.9	836.0957	3	33.41	29	1748	OB4057.raw	8.09E5	1	1	78	98	
R.LN(+.98)AQRPDNRIESEGGYIETWNP(+15.99)NN(+.98)QEFEC(+57.02)AGVALSR.L	N	36.67	4151.8877	36	6.4	1038.9858	4	32.27	29	1679	OB4057.raw	0	0	0	37	72	Carbamidomethylation
K.TAN(+.98)ELN(+.98)LLILRWLGLSAEYGNLYR.N	N	36.48	2793.4700	24	-1.9	932.1622	3	40.89	29	2223	OB4057.raw	0	0	0	377	400	Deamidation (NQ)
R.LNAQ(+.98)RPDNRIESEGGYIETWN(+.98)PNN(+.98)Q(+.98)EFEC(+57.02)AGVALSR.L	N	36.45	4137.8608	36	1.9	1035.4745	4	32.59	29	1698	OB4057.raw	0	0	0	37	72	Carbamidomethylation
R.RFN(+.98)LAGNHEQ(+.98)EFLRYQ(+.98)Q(+.98)QSR.Q	N	35.14	2524.1731	20	-0.3	632.0504	4	29.48	29	1504	OB4057.raw	0	0	0	195	214	
R.GYFGLIFPGC(+57.02)P(+15.99)STYEEPAQQ(+.98)GRR.Y	N	34.17	2646.2173	23	0.8	883.0804	3	34.27	29	1801	OB4057.raw	0	0	0	99	121	Carbamidomethylation
R.R(+43.01)(+14.02)PFYSNAPQEIFIQQGR.G	N	33.80	2107.0598	17	0.4	703.3608	3	30.09	29	1541	OB4057.raw	2.68E3	1	1	82	98	Carbamylation; Methylation(KR)
R.RFN(+.98)LAGNHEQ(+.98)EFLRYQ(+.98)Q(+.98)Q(+.98)SR.Q	N	33.42	2525.1570	20	7.9	632.3015	4	29.03	29	1480	OB4057.raw	2.4E4	1	1	195	214	
R.GYFGLIFPGC(+57.02)P(+15.99)STYEEPAQQGRR.Y	N	33.29	2645.2332	23	-4.2	882.7480	3	33.96	29	1772	OB4057.raw	4.66E4	1	1	99	121	Carbamidomethylation
R.SPDIYNPQAGSLKTANELNLLILRWLGLSAEYGNLYR.N	N	32.64	4162.1851	37	-2.9	1041.5505	4	39.87	29	2158	OB4057.raw	0	0	0	364	400	
S.ISFRQ(+.98)Q(+.98)PEEN(+.98)AC(+57.02)Q(+.98)FQR.L	N	32.58	2040.8846	16	0.0	681.3021	3	28.64	29	1453	OB4057.raw	2.82E3	1	1	21	36	Deamidation (NQ); Carbamidomethylation
K.TDSRPSIANLAGEN(+.98)SFIDN(+.98)LPEEVVANSYGLPREQ(+.98)AR.Q	N	31.61	4031.9348	37	1.7	1008.9927	4	35.16	29	1855	OB4057.raw	0	0	0	470	506	
R.RFNLAGN(+.98)HEQ(+.98)EFLRYQ(+.98)QQSR.Q	N	31.37	2523.1890	20	1.0	631.8052	4	28.55	29	1446	OB4057.raw	0	0	0	195	214	
S.ISFRQ(+.98)Q(+.98)PEEN(+.98)AC(+57.02)QFQR.L	N	31.15	2039.9006	16	-2.4	680.9725	3	29.92	29	1531	OB4057.raw	0	0	0	21	36	Carbamidomethylation
R.LNAQRPDNRIESEGGYIETWNP(+15.99)NNQEFEC(+57.02)AGVALSR.L	N	31.12	4149.9199	36	1.9	1038.4893	4	31.82	29	1673	OB4057.raw	6.01E4	1	1	37	72	Carbamidomethylation
R.GYFGLIFP(+15.99)GC(+57.02)PSTYEEPAQQ(+.98)GR.R	N	30.90	2490.1160	22	-1.5	831.0447	3	35.67	29	1886	OB4057.raw	0	0	0	99	120	Carbamidomethylation
R.RFNLAGN(+.98)HEQ(+.98)EFLRYQ(+.98)Q(+.98)QSR.Q	N	30.35	2524.1731	20	-2.5	632.0490	4	28.87	29	1466	OB4057.raw	0	0	0	195	214	
R.RFN(+.98)LAGN(+.98)HEQEFLRYQ(+.98)QQ(+.98)SR.Q	N	30.27	2524.1731	20	3.7	632.0529	4	29.09	29	1480	OB4057.raw	0	0	0	195	214	Deamidation (NQ)
S.ISFRQQ(+.98)PEEN(+.98)AC(+57.02)QFQR.L	N	30.06	2038.9166	16	-1.3	680.6453	3	29.22	29	1488	OB4057.raw	0	0	0	21	36	Carbamidomethylation
R.RFNLAGNHEQ(+.98)EFLRYQ(+.98)Q(+.98)QSR.Q	N	29.87	2523.1890	20	9.8	631.8107	4	28.40	29	1437	OB4057.raw	0	0	0	195	214	
R.SPDIYN(+.98)PQAGSLKTANELNLLILRWLGLSAEYGNLYR.N	N	29.56	4163.1689	37	1.2	1041.8008	4	39.78	29	2164	OB4057.raw	2.62E4	1	1	364	400	
R.RFNLAGN(+.98)HEQEFLRYQQQ(+.98)SR.Q	N	29.43	2522.2051	20	0.8	631.5590	4	28.08	29	1417	OB4057.raw	0	0	0	195	214	
R.GYFGLIFPGC(+57.02)P(+15.99)STYEEP(+15.99)AQQGRR.Y	N	29.12	2661.2280	23	-4.0	888.0797	3	33.53	29	1750	OB4057.raw	2.5E4	1	1	99	121	Carbamidomethylation
R.SPDIYNPQAGSLKTAN(+.98)ELNLLILRWLGLSAEYGNLYR.N	N	28.83	4163.1689	37	1.2	1041.8008	4	39.78	29	2178	OB4057.raw	2.62E4	1	1	364	400	
K.TDSRPSIAN(+.98)LAGENSFIDN(+.98)LPEEVVANSYGLPREQAR.Q	N	28.27	4030.9507	37	3.3	1008.7482	4	34.85	29	1869	OB4057.raw	3.58E4	1	1	470	506	
R.LN(+.98)AQ(+.98)RPDNRIESEGGYIETWNP(+15.99)NNQ(+.98)EFEC(+57.02)AGVALSR.L	N	28.15	4152.8721	36	7.7	1039.2333	4	31.96	29	1668	OB4057.raw	3.37E4	1	1	37	72	Carbamidomethylation
R.RFN(+.98)LAGNHEQ(+.98)EFLRYQQ(+.98)Q(+.98)SR.Q	N	28.03	2524.1731	20	-3.8	632.0482	4	29.55	29	1508	OB4057.raw	0	0	0	195	214	
R.GYFGLIFPGC(+57.02)PSTYEEP(+15.99)AQ(+.98)QGRR.Y	N	27.80	2646.2173	23	2.4	883.0818	3	34.48	29	1814	OB4057.raw	0	0	0	99	121	Carbamidomethylation
R.LN(+.98)AQ(+.98)RPDNRIESEGGYIETWNP(+15.99)NNQEFEC(+57.02)AGVALSR.L	N	27.49	4151.8877	36	-1.1	1038.9780	4	32.39	29	1686	OB4057.raw	0	0	0	37	72	Carbamidomethylation
R.RFNLAGN(+.98)HEQ(+.98)EFLRYQ(+.98)Q(+.98)Q(+.98)SR.Q	N	27.28	2525.1570	20	7.9	632.3015	4	29.03	29	1466	OB4057.raw	2.4E4	1	1	195	214	
R.R(+42.01)P(+15.99)FYSNAPQEIFIQQGR.G	N	26.80	2108.0439	17	-0.7	703.6881	3	30.76	29	1577	OB4057.raw	3.53E3	1	1	82	98	Acetylation (N-term)

Peptide	Uniq	-10lgP	Mass	Length	ppm	m/z	z	RT	Fraction	Scan	Source File	Area Digest 7. traka 25-27 kDa	#Feature	#Feature Digest 7. traka 25-27 kDa	Start	End	PTM
R.GYFGLIFPGC(+57.02)P(+15.99)STYEEP(+15.99)AQQ(+.98)GRR.Y	N	26.49	2662.2122	23	-5.4	888.4066	3	33.81	29	1773	OB4057.raw	0	0	0	99	121	Carbamidomethylation
R.LN(+.98)AQRPDNRIESEGGYIETWNPNNQ(+.98)EFEC(+57.02)AGVALSR.L	N	26.34	4135.8931	36	-4.7	1034.9757	4	33.17	29	1734	OB4057.raw	0	0	0	37	72	Carbamidomethylation
R.LN(+.98)AQRPDNRIESEGGYIETWN(+.98)PNN(+.98)Q(+.98)EFEC(+57.02)AGVALSR.L	N	26.06	4137.8608	36	-0.7	1035.4718	4	33.49	29	1753	OB4057.raw	0	0	0	37	72	Carbamidomethylation
R.N(+.98)ALRRPFYSN(+.98)APQEIFIQ(+.98)Q(+.98)GR.G	N	25.99	2508.2397	21	8.4	837.0942	3	31.36	29	1621	OB4057.raw	1.89E4	1	1	78	98	
R.NALRRPFYSN(+.98)APQ(+.98)EIFIQQ(+.98)GR.G	N	25.95	2507.2556	21	9.1	836.7667	3	32.01	29	1663	OB4057.raw	0	0	0	78	98	
N.Q(-17.03)EFEC(+57.02)AGVALSR.L	N	25.82	1348.6082	12	2.6	675.3131	2	31.68	29	1643	OB4057.raw	1.38E3	1	1	61	72	Pyro-glu from Q; Carbamidomethylation
R.GYFGLIFPGC(+57.02)PSTYEEPAQQGR(+31.99)R.Y	N	25.71	2661.2280	23	-3.6	888.0801	3	33.19	29	1735	OB4057.raw	0	0	0	99	121	Carbamidomethylation
R.LNAQRPDNRIESEGGYIETWNPNNQEFEC(+57.02)AGVALSR.L	N	25.66	4133.9248	36	1.7	1034.4902	4	31.34	29	1622	OB4057.raw	0	0	0	37	72	Carbamidomethylation
R.RFN(+.98)LAGN(+.98)HEQEFLRYQ(+.98)Q(+.98)Q(+.98)SR.Q	N	25.15	2525.1570	20	-5.6	632.2930	4	29.68	29	1516	OB4057.raw	2.4E3	1	1	195	214	
R.LN(+.98)AQRP(+15.99)DNRIESEGGYIETWNPNN(+.98)QEFEC(+57.02)AGVALSR.L	N	25.01	4151.8877	36	-8.1	1038.9708	4	32.88	29	1716	OB4057.raw	0	0	0	37	72	Carbamidomethylation
R.R(+42.01)(+14.02)PFYSNAPQEIFIQQGR.G	N	24.55	2106.0647	17	-9.4	703.0222	3	30.07	29	1541	OB4057.raw	0	0	0	82	98	Acetylation (N-term); Methylation(KR)
R.SPDIYNPQ(+.98)AGSLKTANELN(+.98)LLILRWLGLSAEYGNLYR.N	N	24.48	4164.1528	37	4.6	1042.0503	4	40.02	29	2168	OB4057.raw	0	0	0	364	400	
K.TDSRPSIANLAGENSFIDN(+.98)LPEEVVANSYGLPREQAR.Q	N	24.32	4029.9668	37	-4.5	1008.4944	4	35.02	29	1847	OB4057.raw	0	0	0	470	506	
R.LNAQRPD(+21.98)NRIESEGGY(-18.01)IETWNPNNQEFEC(+57.02)AGVALSR.L	N	23.34	4137.8965	36	1.7	1035.4832	4	33.31	29	1742	OB4057.raw	0	0	0	37	72	Carbamidomethylation
R.RFNLAGNHEQ(+.98)EFLRYQQ(+.98)QSR.Q	N	23.33	2522.2051	20	-0.7	631.5581	4	28.18	29	1423	OB4057.raw	0	0	0	195	214	
R.GYFGLIFPGC(+57.02)P(+15.99)STYEEP(+15.99)AQ(+.98)Q(+.98)GRR.Y	N	23.01	2663.1960	23	-5.1	888.7348	3	34.20	29	1797	OB4057.raw	0	0	0	99	121	Carbamidomethylation; Deamidation (NQ)
G.GYIETWN(+.98)PNN(+.98)QEFEC(+57.02)AGVALSR.L	N	22.78	2556.1226	22	0.7	853.0487	3	33.23	29	1746	OB4057.raw	8.99E3	1	1	51	72	Carbamidomethylation
**total 126 peptides**																	

tr|O82580|O82580_ARAHY
back to list

| Protein Coverage | Supporting Peptides |

Protein Coverage:

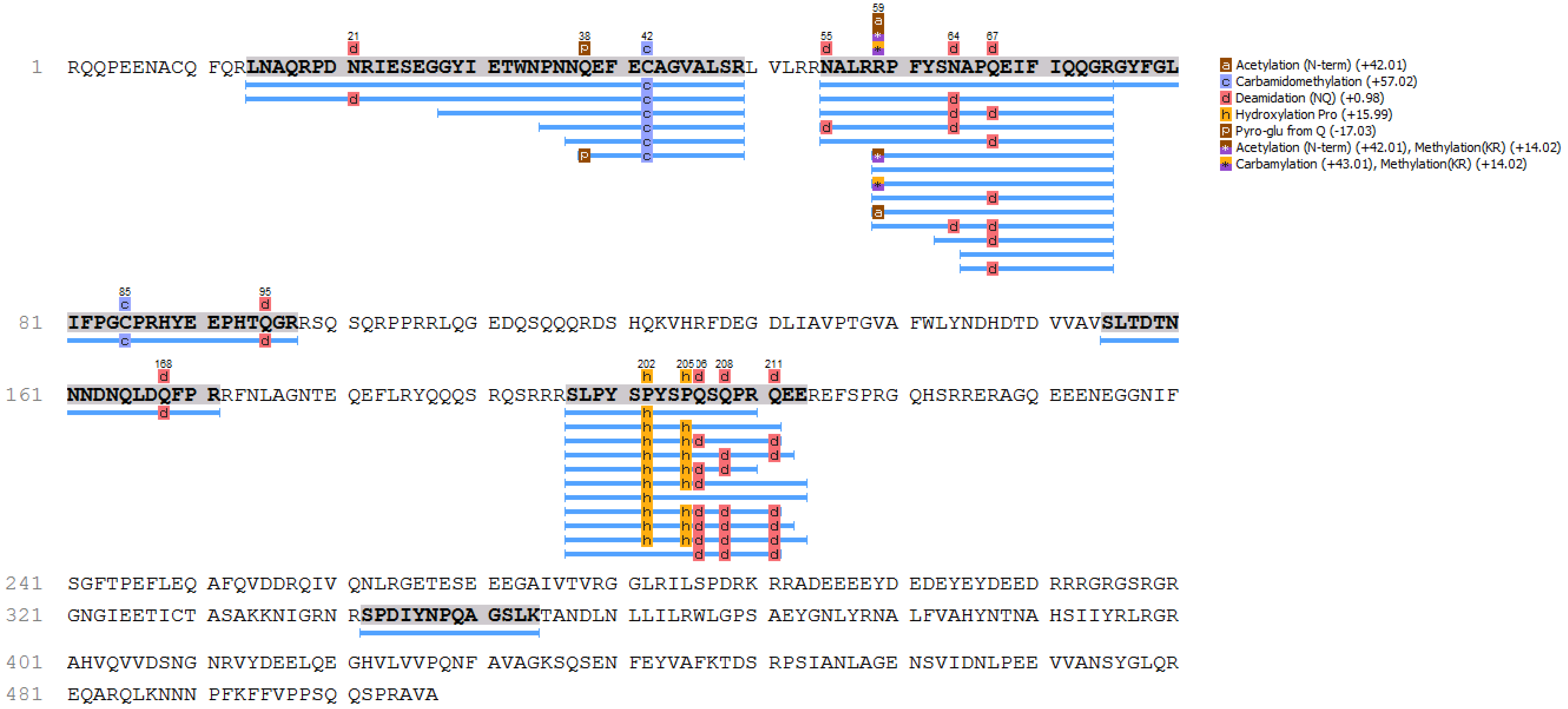

Supporting Peptides:

Peptide	Uniq	-10lgP	Mass	Length	ppm	m/z	z	RT	Fraction	Scan	Source File	Area Digest 7. traka 25-27 kDa	#Feature	#Feature Digest 7. traka 25-27 kDa	Start	End	PTM
R.NALRRPFYSN(+.98)APQEIFIQQGR.G	N	72.96	2505.2876	21	0.8	836.1038	3	30.04	29	1537	OB4057.raw	1.34E5	2	2	55	75	Deamidation (NQ)
R.RPFYSNAPQEIFIQQGR.G	N	72.96	2050.0383	17	0.6	684.3538	3	30.09	29	1546	OB4057.raw	1.76E5	1	1	59	75	
R.NALRRPFYSNAPQEIFIQQGR.G	N	71.98	2504.3037	21	-0.5	835.7748	3	29.54	29	1503	OB4057.raw	5.44E5	2	2	55	75	
R.RPFYSNAPQ(+.98)EIFIQQGR.G	N	70.26	2051.0225	17	0.0	684.6815	3	30.74	29	1580	OB4057.raw	2.52E5	1	1	59	75	Deamidation (NQ)
N.NQ(+.98)EFEC(+57.02)AGVALSR.L	N	69.12	1480.6616	13	0.2	741.3383	2	28.82	29	1460	OB4057.raw	5.61E3	1	1	37	49	Carbamidomethylation
R.RPFYSN(+.98)APQ(+.98)EIFIQQGR.G	N	65.72	2052.0063	17	-0.6	685.0090	3	31.27	29	1614	OB4057.raw	1.11E5	2	2	59	75	Deamidation (NQ)
N.PN(+.98)NQ(+.98)EFEC(+57.02)AGVALSR.L	N	63.50	1692.7413	15	-1.0	847.3771	2	29.54	29	1509	OB4057.raw	2.96E3	1	1	35	49	Carbamidomethylation
R.SLPYSP(+15.99)YSP(+15.99)Q(+.98)SQ(+.98)PRQ(+.98).E	N	62.95	1768.7791	15	0.6	885.3973	2	26.79	29	1334	OB4057.raw	6.62E4	1	1	197	211	Hydroxylation Pro; Deamidation (NQ)

Peptide	Uniq	-10lgP	Mass	Length	ppm	m/z	z	RT	Fraction	Scan	Source File	Area Digest 7. traka 25-27 kDa	#Feature	#Feature Digest 7. traka 25-27 kDa	Start	End	PTM
R.SLPYSP(+15.99)YSP(+15.99)Q(+.98)SQ(+.98)PRQ(+.98)E.E	N	62.00	1897.8217	16	0.6	949.9187	2	26.89	29	1338	OB4057.raw	2.05E4	1	1	197	212	Hydroxylation Pro; Deamidation (NQ)
R.RPFYSNAPQEIFIQQ(+.98)GR.G	N	61.45	2051.0225	17	0.0	684.6815	3	30.74	29	1558	OB4057.raw	2.52E5	1	1	59	75	
R.NALRRPFYSN(+.98)APQ(+.98)EIFIQQGR.G	N	61.01	2506.2717	21	0.8	836.4319	3	30.86	29	1581	OB4057.raw	1.12E5	1	1	55	75	Deamidation (NQ)
R.N(+.98)ALRRPFYSNAPQ(+.98)EIFIQQGR.G	N	59.37	2506.2717	21	-1.6	836.4299	3	31.54	29	1635	OB4057.raw	6.41E3	1	1	55	75	Deamidation (NQ)
N.APQEIFIQQGR.G	N	57.98	1285.6779	11	-0.2	643.8461	2	28.00	29	1415	OB4057.raw	2.55E3	1	1	65	75	
R.N(+.98)ALRRPFYSN(+.98)APQEIFIQQGR.G	N	56.60	2506.2717	21	0.8	836.4319	3	30.86	29	1598	OB4057.raw	1.19E5	2	2	55	75	Deamidation (NQ)
R.SLPYSP(+15.99)YSP(+15.99)Q(+.98)SQPRQ(+.98).E	N	56.49	1767.7950	15	1.6	884.9062	2	26.44	29	1317	OB4057.raw	1.61E4	1	1	197	211	Hydroxylation Pro; Deamidation (NQ)
R.SLPYSP(+15.99)YSP(+15.99)Q(+.98)SQ(+.98)PRQ(+.98)EE.R	N	56.48	2026.8643	17	0.7	1014.4401	2	26.99	29	1346	OB4057.raw	2.68E4	1	1	197	213	Hydroxylation Pro; Deamidation (NQ)
R.NALRRPFYSN(+.98)APQEIFIQQ(+.98)GR.G	N	56.36	2506.2717	21	0.8	836.4319	3	30.86	29	1575	OB4057.raw	1.12E5	1	1	55	75	
R.SLPYSP(+15.99)YSP(+15.99)QSQ(+.98)PRQ(+.98).E	N	55.47	1767.7950	15	1.6	884.9062	2	26.44	29	1315	OB4057.raw	1.61E4	1	1	197	211	Hydroxylation Pro
R.LNAQ(+.98)RPDNRIESEGGYIETWN(+.98)PNNQ(+.98)EFEC(+57.02)AGVALSR.L	N	55.37	4136.8770	36	6.5	1035.2333	4	31.90	29	1667	OB4057.raw	3.94E5	1	1	14	49	Carbamidomethylation
R.RPFYSN(+.98)APQEIFIQQGR.G	N	54.60	2051.0225	17	0.0	684.6815	3	30.74	29	1688	OB4057.raw	2.52E5	1	1	59	75	
R.RPFYSNAPQ(+.98)EIFIQ(+.98)QGR.G	N	54.53	2052.0063	17	-0.6	685.0090	3	31.27	29	1601	OB4057.raw	1.08E5	1	1	59	75	
N.APQ(+.98)EIFIQQGR.G	N	53.97	1286.6619	11	-0.2	644.3381	2	28.64	29	1452	OB4057.raw	6.63E3	1	1	65	75	Deamidation (NQ)
R.RPFYSN(+.98)APQ(+.98)EIFIQ(+.98)QGR.G	N	51.51	2052.9905	17	-0.9	685.3369	3	31.47	29	1634	OB4057.raw	1.41E4	1	1	59	75	Deamidation (NQ)
R.LN(+.98)AQRPDNRIESEGGYIETWN(+.98)PNNQEFEC(+57.02)AGVALSR.L	N	49.62	4135.8931	36	1.6	1034.9822	4	31.82	29	1652	OB4057.raw	0	0	0	14	49	Carbamidomethylation
V.SLTDTNN(+.98)N(+.98)DN(+.98)QLDQ(+.98)FPR.R	N	49.16	1994.8341	17	4.1	998.4285	2	30.46	29	1566	OB4057.raw	0	0	0	155	171	Deamidation (NQ)
R.LNAQ(+.98)RPDNRIESEGGYIETWNPN(+.98)N(+.98)Q(+.98)EFEC(+57.02)AGVALSR.L	N	48.61	4137.8608	36	6.8	1035.4795	4	32.47	29	1691	OB4057.raw	0	0	0	14	49	Carbamidomethylation
R.LNAQ(+.98)RPDN(+.98)RIESEGGYIETWNPNN(+.98)Q(+.98)EFEC(+57.02)AGVALSR.L	N	47.19	4137.8608	36	5.5	1035.4781	4	32.37	29	1685	OB4057.raw	0	0	0	14	49	Carbamidomethylation
G.GYIETWNPN(+.98)NQEFEC(+57.02)AGVALSR.L	N	46.05	2555.1387	22	1.9	852.7218	3	33.01	29	1724	OB4057.raw	0	0	0	28	49	Carbamidomethylation
R.SLPYSP(+15.99)YSP(+15.99)Q(+.98)SQPRQ(+.98)EE.R	N	44.77	2025.8802	17	-1.6	1013.9458	2	26.69	29	1329	OB4057.raw	8.53E3	1	1	197	213	Hydroxylation Pro; Deamidation (NQ)
R.LN(+.98)AQRPDN(+.98)RIESEGGYIETWNP(+15.99)NNQ(+.98)EFEC(+57.02)AGVALSR.L	N	43.32	4152.8721	36	7.7	1039.2333	4	31.96	29	1673	OB4057.raw	3.37E4	1	1	14	49	Deamidation (NQ); Carbamidomethylation
Y.SNAPQ(+.98)EIFIQQGR.G	N	42.42	1487.7368	13	0.1	744.8757	2	29.44	29	1506	OB4057.raw	2.04E3	1	1	63	75	Deamidation (NQ)
R.LN(+.98)AQRPDNRIESEGGYIETWNPNNQEFEC(+57.02)AGVALSR.L	N	40.89	4134.9087	36	3.0	1034.7375	4	31.51	29	1640	OB4057.raw	1.17E5	1	1	14	49	Carbamidomethylation
R.LNAQ(+.98)RPDNRIESEGGYIETWNPNNQEFEC(+57.02)AGVALSR.L	N	40.63	4134.9087	36	3.0	1034.7375	4	31.51	29	1628	OB4057.raw	1.17E5	1	1	14	49	Carbamidomethylation
N.APQ(+.98)EIFIQ(+.98)QGR.G	N	40.15	1287.6459	11	-0.6	644.8298	2	29.21	29	1486	OB4057.raw	1.52E3	1	1	65	75	Deamidation (NQ)
R.SPDIYNPQ(+.98)AGSLK.T	N	39.27	1389.6776	13	1.0	695.8468	2	28.06	29	1416	OB4057.raw	0	0	0	342	354	
R.LNAQRPDN(+.98)RIESEGGYIETWN(+.98)PN(+.98)N(+.98)QEFEC(+57.02)AGVALSR.L	N	39.23	4137.8608	36	-1.3	1035.4712	4	32.66	29	1703	OB4057.raw	0	0	0	14	49	Carbamidomethylation
R.LN(+.98)AQRPDNRIESEGGYIETWNPNN(+.98)Q(+.98)EFEC(+57.02)AGVALSR.L	N	39.15	4136.8770	36	2.4	1035.2290	4	33.65	29	1763	OB4057.raw	0	0	0	14	49	Carbamidomethylation
R.SLPYSP(+15.99)YSP(+15.99)Q(+.98)SQ(+.98)PR.Q	N	39.01	1639.7365	14	2.1	820.8773	2	26.59	29	1330	OB4057.raw	5.55E3	1	1	197	210	Hydroxylation Pro; Deamidation (NQ)
R.LN(+.98)AQRPDNRIESEGGYIETWN(+.98)PN(+.98)NQEFEC(+57.02)AGVALSR.L	N	38.91	4136.8770	36	6.5	1035.2333	4	31.90	29	1708	OB4057.raw	3.94E5	1	1	14	49	Carbamidomethylation
R.LN(+.98)AQRPDNRIESEGGYIETWNPN(+.98)N(+.98)QEFEC(+57.02)AGVALSR.L	N	38.53	4136.8770	36	0.8	1035.2273	4	34.69	29	1826	OB4057.raw	0	0	0	14	49	Carbamidomethylation
R.NALRRPFYSNAPQ(+.98)EIFIQQGR.G	N	37.39	2505.2876	21	-8.9	836.0957	3	33.41	29	1748	OB4057.raw	8.09E5	1	1	55	75	
R.SLPYSP(+15.99)YSP(+15.99)QSQ(+.98)PRQ(+.98)E.E	N	37.26	1896.8376	16	-0.6	949.4255	2	26.49	29	1322	OB4057.raw	5.37E3	1	1	197	212	Hydroxylation Pro; Deamidation (NQ)
R.LN(+.98)AQRPDNRIESEGGYIETWNP(+15.99)NN(+.98)QEFEC(+57.02)AGVALSR.L	N	36.67	4151.8877	36	6.4	1038.9858	4	32.27	29	1679	OB4057.raw	0	0	0	14	49	Carbamidomethylation

Peptide	Uniq	-10lgP	Mass	Length	ppm	m/z	z	RT	Fraction	Scan	Source File	Area Digest 7. traka 25-27 kDa	#Feature	#Feature Digest 7. traka 25-27 kDa	Start	End	PTM
R.LNAQ(+.98)RPDNRIESEGGYIETWN(+.98)PNN(+.98)Q(+.98)EFEC(+57.02)AGVALSR.L	N	36.45	4137.8608	36	1.9	1035.4745	4	32.59	29	1698	OB4057.raw	0	0	0	14	49	Carbamidomethylation
R.GYFGLIFPGC(+57.02)PRHYEEPHTQ(+.98)GR(+14.02).R	Y	35.31	2632.2280	22	-7.6	878.4099	3	33.06	29	1727	OB4057.raw	0	0	0	76	97	Carbamidomethylation; Deamidation (NQ)
R.SLPYSP(+15.99)YSP(+15.99)Q(+.98)SQPR.Q	N	34.93	1638.7524	14	3.9	820.3867	2	26.35	29	1310	OB4057.raw	0	0	0	197	210	Hydroxylation Pro
R.R(+43.01)(+14.02)PFYSNAPQEIFIQQGR.G	N	33.80	2107.0598	17	0.4	703.3608	3	30.09	29	1541	OB4057.raw	2.68E3	1	1	59	75	Carbamylation; Methylation(KR)
R.LNAQRPDNRIESEGGYIETWNP(+15.99)NNQEFEC(+57.02)AGVALSR.L	N	31.12	4149.9199	36	1.9	1038.4893	4	31.82	29	1673	OB4057.raw	6.01E4	1	1	14	49	Carbamidomethylation
R.SLPYSPYSP(+15.99)Q(+.98)SQ(+.98)PRQ(+.98).E	N	29.62	1752.7842	15	0.1	877.3994	2	27.92	29	1406	OB4057.raw	1.81E4	1	1	197	211	Deamidation (NQ)
R.LN(+.98)AQ(+.98)RPDNRIESEGGYIETWNP(+15.99)NNQ(+.98)EFEC(+57.02)AGVALSR.L	N	28.15	4152.8721	36	7.7	1039.2333	4	31.96	29	1668	OB4057.raw	3.37E4	1	1	14	49	Carbamidomethylation
R.LN(+.98)AQ(+.98)RPDNRIESEGGYIETWNP(+15.99)NNQEFEC(+57.02)AGVALSR.L	N	27.49	4151.8877	36	-1.1	1038.9780	4	32.39	29	1686	OB4057.raw	0	0	0	14	49	Carbamidomethylation
R.R(+42.01)P(+15.99)FYSNAPQEIFIQQGR.G	N	26.80	2108.0439	17	-0.7	703.6881	3	30.76	29	1577	OB4057.raw	3.53E3	1	1	59	75	Acetylation (N-term)
R.SLPYSP(+15.99)YSP(+15.99)Q(+.98)SQ(+.98)PRQEE.R	N	26.49	2025.8802	17	-1.6	1013.9458	2	26.69	29	1341	OB4057.raw	8.53E3	1	1	197	213	Hydroxylation Pro
R.LN(+.98)AQRPDNRIESEGGYIETWNPNNQ(+.98)EFEC(+57.02)AGVALSR.L	N	26.34	4135.8931	36	-4.7	1034.9757	4	33.17	29	1734	OB4057.raw	0	0	0	14	49	Carbamidomethylation
R.LN(+.98)AQRPDNRIESEGGYIETWN(+.98)PNN(+.98)Q(+.98)EFEC(+57.02)AGVALSR.L	N	26.06	4137.8608	36	-0.7	1035.4718	4	33.49	29	1753	OB4057.raw	0	0	0	14	49	Carbamidomethylation
R.N(+.98)ALRRPFYSN(+.98)APQEIFIQ(+.98)Q(+.98)GR.G	N	25.99	2508.2397	21	8.4	837.0942	3	31.36	29	1621	OB4057.raw	1.89E4	1	1	55	75	
R.NALRRPFYSN(+.98)APQ(+.98)EIFIQQ(+.98)GR.G	N	25.95	2507.2556	21	9.1	836.7667	3	32.01	29	1663	OB4057.raw	0	0	0	55	75	
N.Q(-17.03)EFEC(+57.02)AGVALSR.L	N	25.82	1348.6082	12	2.6	675.3131	2	31.68	29	1643	OB4057.raw	1.38E3	1	1	38	49	Pyro-glu from Q; Carbamidomethylation
R.LNAQRPDNRIESEGGYIETWNPNNQEFEC(+57.02)AGVALSR.L	N	25.66	4133.9248	36	1.7	1034.4902	4	31.34	29	1622	OB4057.raw	0	0	0	14	49	Carbamidomethylation
R.LN(+.98)AQRP(+15.99)DNRIESEGGYIETWNPNN(+.98)QEFEC(+57.02)AGVALSR.L	N	25.01	4151.8877	36	-8.1	1038.9708	4	32.88	29	1716	OB4057.raw	0	0	0	14	49	Carbamidomethylation
R.R(+42.01)(+14.02)PFYSNAPQEIFIQQGR.G	N	24.55	2106.0647	17	-9.4	703.0222	3	30.07	29	1541	OB4057.raw	0	0	0	59	75	Acetylation (N-term); Methylation(KR)
R.LNAQRPD(+21.98)NRIESEGGY(-18.01)IETWNPNNQEFEC(+57.02)AGVALSR.L	N	23.34	4137.8965	36	1.7	1035.4832	4	33.31	29	1742	OB4057.raw	0	0	0	14	49	Carbamidomethylation
G.GYIETWN(+.98)PNN(+.98)QEFEC(+57.02)AGVALSR.L	N	22.78	2556.1226	22	0.7	853.0487	3	33.23	29	1746	OB4057.raw	8.99E3	1	1	28	49	Carbamidomethylation
**total 63 peptides**																	

tr|Q6IWG5|Q6IWG5_ARAHY

back to list

| Protein Coverage | Supporting Peptides |

Protein Coverage:

1 KLLALSLCFC VLVLGASSVT FRQGGEENEC QFQR**LNAQRP DNRIESEGGY IETWNPNNQE FQCAGVALSR** TVLRRNALRR

81 PFYSNAPLEI YVQQGS**GYFG LIFPGCPSTY EEPA**QEGRRY QSQKPSRRFQ VGQDDPSQQQ QDSHQKVHRF DEGDLIAVPT

161 GVAFWMYNDE DTDVVTVTLS DTSSIHNQLD QFPRRFYLAG NQEQEFLRYQ QQQGSRPHYR QISPRVRGDE QENEGSNIFS

241 GFAQEFLQHA FQVDRQTVEN LRGENEREEQ GAIVTVKGGL RILSPDEEDE SSRSPPSRRE EFDEDRSRPQ QRGKYDENRR

321 GYKNGIEETI CSASVKKNLG R**SSNPDIYNP QAGSLRSVNE LDLPILGWLG LSAQHGTIYR** NAMFVPHYTL NAHTIVVALN

401 GRAHVQVVDS NGNRVYDEEL QEGHVLVVPQ NFAVAAKAQS ENYEYLAFKT DSRPSIANLA GENSIIDNLP EEVVANSYRL

481 PREQARQLKN NNPFKFFVPP FDHQSMREVA

Modification sites: 63 c, 106 c, 359 d, 374 d

c Carbamidomethylation (+57.02)
d Deamidation (NQ) (+0.98)

Supporting Peptides:

Peptide	Uniq	-10lgP	Mass	Length	ppm	m/z	z	RT	Fraction	Scan	Source File	Area Digest 7. traka 25-27 kDa	#Feature	#Feature Digest 7. traka 25-27 kDa	Start	End	PTM
R.SVNELDLPILGWLGLSAQ(+.98)HGTIYR.N	Y	47.19	2652.3911	24	0.4	885.1380	3	38.48	29	2067	OB4057.raw	7.57E4	1	1	357	380	Deamidation (NQ)
S.GYFGLIFPGC(+57.02)PSTYEEPA.Q	N	45.16	2003.8975	18	1.1	1002.9572	2	37.61	29	2011	OB4057.raw	6.78E3	1	1	97	114	Carbamidomethylation
R.SVNELDLPILGWLGLSAQHGTIYR.N	Y	41.94	2651.4070	24	0.0	884.8096	3	38.25	29	2047	OB4057.raw	4.26E4	2	2	357	380	
S.GYFGLIFPGC(+57.02)PSTYEE.P	N	40.42	1835.8076	16	0.2	918.9113	2	37.63	29	2015	OB4057.raw	1.67E3	1	1	97	112	Carbamidomethylation
R.SVN(+.98)ELDLPILGWLGLSAQHGTIYR.N	Y	36.81	2652.3911	24	0.4	885.1380	3	38.48	29	2079	OB4057.raw	7.67E4	2	2	357	380	Deamidation (NQ)
R.SSNPDIYN(+.98)PQAGSLRSVN(+.98)ELDLPILGWLGLSAQHGTIYR.N	Y	26.42	4253.1392	39	1.0	1064.2931	4	37.75	29	2022	OB4057.raw	9.71E4	1	1	342	380	
R.LN(+.98)AQRPDNRIESEGGYIETWN(+.98)PNNQ(+.98)EFQC(+57.02)AGVALSR.T	Y	25.73	4135.8931	36	2.4	1034.9830	4	32.98	29	1722	OB4057.raw	0	0	0	35	70	Carbamidomethylation
R.SVN(+.98)ELDLPILGWLGLSAQ(+.98)HGTIYR.N	Y	24.29	2653.3750	24	1.4	885.4669	3	38.86	29	2099	OB4057.raw	1.39E4	1	1	357	380	Deamidation (NQ)
R.SSNPDIYN(+.98)PQ(+.98)AGSLRSVNELDLPILGWLGLSAQ(+.98)HGTIYR.N	Y	23.71	4254.1230	39	5.6	1064.5439	4	38.06	29	2042	OB4057.raw	0	0	0	342	380	
R.SSNPDIYNPQAGSLRSVN(+.98)ELDLPILGWLGLSAQHGTIYR.N	Y	22.88	4252.1553	39	0.6	1064.0468	4	37.58	29	2010	OB4057.raw	0	0	0	342	380	
**total 10 peptides**																	

tr|Q0GM57|Q0GM57_ARAHY

back to list

| Protein Coverage | Supporting Peptides |

Protein Coverage:

```
                                                                       65
                                                                       c
  1 MAKLLALSLC FCVLVLGASS VTFRQGGEEN ECQFQRLNAQ RPDNRIESEG GYIETWNPNN QEFQCAGVAL SRTVLRRNAL

                          108
                          c
 81 RRPFYSNAPL EIYVQQGSGY FGLIFPGCPS TYEEPAQEGR RYQSQKPSRR FQVGQDDPSQ QQQDSHQKVH RFDEGDLIAV

161 PTGVAFWMYN DEDTDVVTVT LSDTSSIHNQ LDQFPRRFYL AGNQEQEFLR YQQQQGSRPH YRQISPRVRG DEQENEGSNI
241 FSGFAQEFLQ HAFQVDRQTV ENLRGENERE EQGAIVTVKG GLRILSPDEE DESSRSPPSR REEFDEDRSR PQQRGKYDEN
                                               361            376
                                               d              d
321 RRGYKNGIEE TICSASVKKN LGRSSNPDIY NPQAGSLRSV NELDLPILGW LGLSAQHGTI YRNAMFVPHY TLNAHTIVVA

401 LNGRAHVQVV DSNGNRVYDE ELQEGHVLVV PQNFAVAAKA QSENYEYLAF KTDSRPSIAN LAGENSIIDN LPEEVVANSY
481 RLPREQARQL KNNNPFKFFV PPFDHQSMRE VA
```

c Carbamidomethylation (+57.02)
d Deamidation (NQ) (+0.98)

Supporting Peptides:

Peptide	Uniq	-10lgP	Mass	Length	ppm	m/z	z	RT	Fraction	Scan	Source File	Area Digest 7. traka 25-27 kDa	#Feature	#Feature Digest 7. traka 25-27 kDa	Start	End	PTM
R.SVNELDLPILGWLGLSAQ(+.98)HGTIYR.N	Y	47.19	2652.3911	24	0.4	885.1380	3	38.48	29	2067	OB4057.raw	7.57E4	1	1	359	382	Deamidation (NQ)
S.GYFGLIFPGC(+57.02)PSTYEEPA.Q	N	45.16	2003.8975	18	1.1	1002.9572	2	37.61	29	2011	OB4057.raw	6.78E3	1	1	99	116	Carbamidomethylation
R.SVNELDLPILGWLGLSAQHGTIYR.N	Y	41.94	2651.4070	24	0.0	884.8096	3	38.25	29	2047	OB4057.raw	4.26E4	2	2	359	382	
S.GYFGLIFPGC(+57.02)PSTYEE.P	N	40.42	1835.8076	16	0.2	918.9113	2	37.63	29	2015	OB4057.raw	1.67E3	1	1	99	114	Carbamidomethylation
R.SVN(+.98)ELDLPILGWLGLSAQHGTIYR.N	Y	36.81	2652.3911	24	0.4	885.1380	3	38.48	29	2079	OB4057.raw	7.67E4	2	2	359	382	Deamidation (NQ)
R.SSNPDIYN(+.98)PQAGSLRSVN(+.98)ELDLPILGWLGLSAQHGTIYR.N	Y	26.42	4253.1392	39	1.0	1064.2931	4	37.75	29	2022	OB4057.raw	9.71E4	1	1	344	382	
R.LN(+.98)AQRPDNRIESEGGYIETWN(+.98)PNNQ(+.98)EFQC(+57.02)AGVALSR.T	Y	25.73	4135.8931	36	2.4	1034.9830	4	32.98	29	1722	OB4057.raw	0	0	0	37	72	Carbamidomethylation
R.SVN(+.98)ELDLPILGWLGLSAQ(+.98)HGTIYR.N	Y	24.29	2653.3750	24	1.4	885.4669	3	38.86	29	2099	OB4057.raw	1.39E4	1	1	359	382	Deamidation (NQ)
R.SSNPDIYN(+.98)PQ(+.98)AGSLRSVNELDLPILGWLGLSAQ(+.98)HGTIYR.N	Y	23.71	4254.1230	39	5.6	1064.5439	4	38.06	29	2042	OB4057.raw	0	0	0	344	382	
R.SSNPDIYNPQAGSLRSVN(+.98)ELDLPILGWLGLSAQHGTIYR.N	Y	22.88	4252.1553	39	0.6	1064.0468	4	37.58	29	2010	OB4057.raw	0	0	0	344	382	
**total 10 peptides**																	

tr|E5G077|E5G077_ARAHY

back to list

| Protein Coverage | Supporting Peptides |

Protein Coverage:

65 c

1 MAKLLALSLC FCVLVLGASS VTFRQGGEEN ECQFQR**LNAQ RPDNRIESEG GYIETWNPNN QEFQCAGVAL SR**TVLRRNAL

c Carbamidomethylation (+57.02)
d Deamidation (NQ) (+0.98)

108 c

81 RRPFYSNAPL EIYVQQGS**GY FGLIFPGCPS TYEEPA**QEGR RYQSQKPSRR FQVGQDDPSQ QQQDSHQKVH RFDEGDLIAV

161 PTGVAFWMYN DEDTDVVTVT LSDTSSIHNQ LDQFPRRFYL AGNQEQEFLR YQQQQGSRPH YRQISPRVRG DEQENEGSNI

241 FSGFAQEFLQ HAFQVDRQTV ENLRGENERE EQGAIVTVKG GLRILSPDEE DESSRSPPNR REEFDEDRSR PQQRGKYDEN

361 d 376 d

321 RRGYKNGIEE TICSASVKKN LGR**SSNPDIY NPQAGSLRSV NELDLPILGW LGLSAQHGTI YR**NAMFVPHY TLNAHTIVVA

401 LNGRAHVQVV DSNGNRVYDE ELQEGHVLVV PQNFAVAAKA QSENYEYLAF KTDSRPSIAN QAGENSIIDN LPEEVVANSY

481 RLPREQARQL KNNNPFKFFV PPFDHQSMRE VA

Supporting Peptides:

Peptide	Uniq	-10lgP	Mass	Length	ppm	m/z	z	RT	Fraction	Scan	Source File	Area Digest 7. traka 25-27 kDa	#Feature	#Feature Digest 7. traka 25-27 kDa	Start	End	PTM
R.SVNELDLPILGWLGLSAQ(+.98)HGTIYR.N	Y	47.19	2652.3911	24	0.4	885.1380	3	38.48	29	2067	OB4057.raw	7.57E4	1	1	359	382	Deamidation (NQ)
S.GYFGLIFPGC(+57.02)PSTYEEPA.Q	N	45.16	2003.8975	18	1.1	1002.9572	2	37.61	29	2011	OB4057.raw	6.78E3	1	1	99	116	Carbamidomethylation
R.SVNELDLPILGWLGLSAQHGTIYR.N	Y	41.94	2651.4070	24	0.0	884.8096	3	38.25	29	2047	OB4057.raw	4.26E4	2	2	359	382	
S.GYFGLIFPGC(+57.02)PSTYEE.P	N	40.42	1835.8076	16	0.2	918.9113	2	37.63	29	2015	OB4057.raw	1.67E3	1	1	99	114	Carbamidomethylation
R.SVN(+.98)ELDLPILGWLGLSAQHGTIYR.N	Y	36.81	2652.3911	24	0.4	885.1380	3	38.48	29	2079	OB4057.raw	7.67E4	2	2	359	382	Deamidation (NQ)
R.SSNPDIYN(+.98)PQAGSLRSVN(+.98)ELDLPILGWLGLSAQHGTIYR.N	Y	26.42	4253.1392	39	1.0	1064.2931	4	37.75	29	2022	OB4057.raw	9.71E4	1	1	344	382	
R.LN(+.98)AQRPDNRIESEGGYIETWN(+.98)PNNQ(+.98)EFQC(+57.02)AGVALSR.T	Y	25.73	4135.8931	36	2.4	1034.9830	4	32.98	29	1722	OB4057.raw	0	0	0	37	72	Carbamidomethylation
R.SVN(+.98)ELDLPILGWLGLSAQ(+.98)HGTIYR.N	Y	24.29	2653.3750	24	1.4	885.4669	3	38.86	29	2099	OB4057.raw	1.39E4	1	1	359	382	Deamidation (NQ)
R.SSNPDIYN(+.98)PQ(+.98)AGSLRSVNELDLPILGWLGLSAQ(+.98)HGTIYR.N	Y	23.71	4254.1230	39	5.6	1064.5439	4	38.06	29	2042	OB4057.raw	0	0	0	344	382	
R.SSNPDIYNPQAGSLRSVN(+.98)ELDLPILGWLGLSAQHGTIYR.N	Y	22.88	4252.1553	39	0.6	1064.0468	4	37.58	29	2010	OB4057.raw	0	0	0	344	382	
**total 10 peptides**																	

tr|Q38711|Q38711_ARAHY

back to list

| Protein Coverage | Supporting Peptides |

Protein Coverage:

1 KPFCVFLTFF LLLAASKKVN SAETVSFNFN SFSEGNPAIN FQGDVTVLSN GNIQLTNLNK VNSVGRVLYA MPVRIWSSAT

81 GNVASFLTSF SFEMKDIKDY DPADGIIFFI APEDTQIPAG SIGGGTLGVS DTKGAGHFVG VEFDTYSNSE YNDPPTDHVG

161 IDVNSVDSVK TVPWNSVSGA VVQVTVIYDS STKTLSVAVT NENGDITTIA QVVDLKAKLP ERVKFGFSAS GSLGGRQIHL

241 IR**SWSFTSTL ITTTR**RSIDN NEKKIMNMAT A

Supporting Peptides:

Peptide	Uniq	-10lgP	Mass	Length	ppm	m/z	z	RT	Fraction	Scan	Source File	Area Digest 7. traka 25-27 kDa	#Feature	#Feature Digest 7. traka 25-27 kDa	Start	End	PTM
R.SWSFTSTLITTTR.R	Y	56.30	1499.7620	13	1.2	750.8892	2	33.13	29	1736	OB4057.raw	5.67E3	1	1	243	255	

Peptide	Uniq	-10lgP	Mass	Length	ppm	m/z	z	RT	Fraction	Scan	Source File	Area Digest 7. traka 25-27 kDa	#Feature	#Feature Digest 7. traka 25-27 kDa	Start	End	PTM
**total 1 peptides**																	

tr|A0A089ZXL7|A0A089ZXL7_ARAHY

back to list

| Protein Coverage | Supporting Peptides |

Protein Coverage:

```
  1 MKPFCVFLTF FLLLAASSKK VNSAETVSFN FNSFSEGNPA INFQGDVTVL SNGNIQLTNL NKVNSVGRVL YAMPVRIWSS
 81 ATGNVASFLT SFSFEMKDIK DYDPADGIIF FIAPEDTQIP AGSIGGGTLG VSDTKGAGYF VGVEFDTYSN SEYNDPPTDH
161 VGIDVNSVDS VKTVPWNSVS GAVVKVTVIY DSSTKTLSVA VTNDNGDITT IAQVVDLKAK LPERVKFGFS ASGSRAGRQI
241 HLIRSWSFTS TLITTTRRSI DNNEKKIMNM ASA
```

Supporting Peptides:

Peptide	Uniq	-10lgP	Mass	Length	ppm	m/z	z	RT	Fraction	Scan	Source File	Area Digest 7. traka 25-27 kDa	#Feature	#Feature Digest 7. traka 25-27 kDa	Start	End	PTM
R.SWSFTSTLITTTR.R	Y	56.30	1499.7620	13	1.2	750.8892	2	33.13	29	1736	OB4057.raw	5.67E3	1	1	245	257	
**total 1 peptides**																	

P02872|LECG_ARAHY

back to list

| Protein Coverage | Supporting Peptides |

Protein Coverage:

```
  1 MKPFCVFLTF FLLLAASSKK VDSAETVSFN FNSFSEGNPA INFQGDVTVL SNGNIQLTNL NKVNSVGRVL YAMPVRIWSS
 81 ATGNVASFLT SFSFEMKDIK DYDPADGIIF FIAPEDTQIP AGSIGGGTLG VSDTKGAGHF VGVEFDTYSN SEYNDPPTDH
161 VGIDVNSVDS VKTVPWNSVS GAVVKVTVIY DSSTKTLSVA VTNDNGDITT IAQVVDLKAK LPERVKFGFS ASGSLGGRQI
241 HLIRSWSFTS TLITTTRRSI DNNEKKIMNM ASA
```

Supporting Peptides:

Peptide	Uniq	-10lgP	Mass	Length	ppm	m/z	z	RT	Fraction	Scan	Source File	Area Digest 7. traka 25-27 kDa	#Feature	#Feature Digest 7. traka 25-27 kDa	Start	End	PTM
R.SWSFTSTLITTTR.R	Y	56.30	1499.7620	13	1.2	750.8892	2	33.13	29	1736	OB4057.raw	5.67E3	1	1	245	257	
**total 1 peptides**																	

tr|A0A109QJM5|A0A109QJM5_ARAHY

back to list

| Protein Coverage | Supporting Peptides |

Protein Coverage:

1 MAQKRFIVEV EKAKEAEGER PSRGPVYRSL FAKDGFPPPV PGLDNCWDIF RTSVQKYPKN PMLGHREIVD GKPGKYKWKT

h Hydroxylation Pro (+15.99)

81 YEEVYDLVIK IGNSLRSCGY GEGVKCGIYG ANCAEWIISM EAINAHGLYC APLYDTLGAG AVEFIICHAE VSVSFVEEKK

161 IPELLKTFPN TTKYLKTLVS FGKVTPEQKQ EVEKFGLAIY SWDEFSQVGQ NQSFDLPVKK KSDICTIMYT SGTTGDPKGV

241 LISNESILTL LAGVKRLLES VKEELNDKDV YLSYLPLAHI FDRVIEELFI WHGASIGFWR GDVKLLIEDI GELKPTIFCA

369 h

321 VPRVLDRVYT GLTQKVSAGG FLKQALFNFA YSYKLNNMKK GR**KHEEASPL LDK**IVFDKVK QGLGGHVRLI LSGAAPLSPH

h

401 VESYLKVVTC AHVIQGYGLT ETCAGTFVSL PHEMDMVGTV GPPVPNVDVC LESVPDMGYD ALGSTPRGEI CVRGNTLFSG

481 YYKRDDLTNE VLTDGWFHTG DVGEWQPDGS MKIIDRKKNI FKLSQGEYVA VENLESVYRQ LSCIDSIWVY GNSFESFLVA

561 VANPSKPVLE RWAEENGIKI DFDSLCKDSR AKSYILGELT RIAKENKLKG YEFIKDVHLD PVPFDMERDL ITPTYKMKRP

641 QLLKYYKNVI DDMYKSGGKR SA

Supporting Peptides:

Peptide	Uniq	-10lgP	Mass	Length	ppm	m/z	z	RT	Fraction	Scan	Source File	Area Digest 7. traka 25-27 kDa	#Feature	#Feature Digest 7. traka 25-27 kDa	Start	End	PTM
R.KHEEASP(+15.99)LLDK.I	Y	27.33	1281.6564	11	2.1	641.8368	2	30.55	29	1563	OB4057.raw	6.54E3	1	1	363	373	Hydroxylation Pro
**total 1 peptides**																	

tr|A1E2B0|A1E2B0_ARAHY

back to list

| Protein Coverage | Supporting Peptides |

Protein Coverage:

1 MANSLRLAFL VLFQSSLALS LAHRYPTKCS FDKLVALEPT KRVESEGGFT EYWDSKNDQF QCVGVSALRY SIKPK**GLLLP**

81 **HYINAPR**LQY VLQGTGILET VVPGCPETFR EQTRHGDQHQ KIHATREGDV IVVPTGSAQW IYNSGETDMV IFSVIDSANE

161 DNQLDLKVRK FFLGGKPQEE KGEEGNMFSG LELKTVAESL DIDMGIAGKV QGVDDPRGSM IIVEDELETL SPAVEESGNG

241 NGLIETVCTL RLVHQLAEST DADKYNPRAG FLTALTPLTF PSFNMSNLAL IDVFSTECCD GATLH

Supporting Peptides:

Peptide	Uniq	-10lgP	Mass	Length	ppm	m/z	z	RT	Fraction	Scan	Source File	Area Digest 7. traka 25-27 kDa	#Feature	#Feature Digest 7. traka 25-27 kDa	Start	End	PTM
K.GLLLPHYINAPR.L	Y	24.77	1362.7771	12	-0.5	455.2661	3	29.81	29	1523	OB4057.raw	5.41E3	1	1	76	87	
**total 1 peptides**																	

Q647H2|AHY3_ARAHY

back to list

| Protein Coverage | Supporting Peptides |

Protein Coverage:

1	MAKLLALSVC	FCFLVLGASS	VTFRQQGEEN	ECQFQR**LNAQ**	**RPDNCIESEG**	**GYIETWNPNN**	**QEFQCAGVAL**	**SR**FVLRRNAL				

Carbamidomethylation (+57.02)
Hydroxylation Pro (+15.99)

81 RRPFYSNAPQ EIFIYQGSGY FGLIFPGCPG TFEEPIQGSE QFQRPSRHFQ GQDQSQRPLD THQKVHGFRE GDLIAVPHGV
161 AFWIYNDQDT DVVAISVLHT NSLHNQLDQF PRRFNLAGKQ EQEFLRYQQR SGRQSPKGEE QEQEQENEGG NVFSGFSTEF
241 LSHGFQVNED IVRNLRGENE REEQGAIVTV KGGLSILVPP EWRQSYQQPG RGDKDFNNGI EETICTATVK MNIGKSTSAD
321 IYNPQAGSVR TVNELDLPIL NRLGLSAEYG SIHRDAMFVP HYNMNANSMI YALHGGAHVQ VVDCNGNRVF DEELQEGQSL
401 VVPQNFAVAA KSQSEHFLYV AFKTNSRASI SNLAGKNSYM WNLPEDVVAN SYGLQYEQAR QLKNNNPFTF LVPPQDSQMI
481 RTVA

Supporting Peptides:

Peptide	Uniq	-10lgP	Mass	Length	ppm	m/z	z	RT	Fraction	Scan	Source File	Area Digest 7. traka 25-27 kDa	#Feature	#Feature Digest 7. traka 25-27 kDa	Start	End	PTM
R.LNAQRP(+15.99)DNC(+57.02)IESEGGYIETWNP(+15.99)NN(+.98)QEFQC(+57.02)AGVALSR.F	Y	23.43	4169.8442	36	3.6	1043.4720	4	32.06	29	1650	OB4057.raw	2.36E4	1	1	37	72	Hydroxylation Pro; Carbamidomethylation
**total 1 peptides**																	

Peptide List

Prepared with PEAKS ™ (bioinfor.com)

1. Notes Gastric Control Raw peanut Band #8 27-29 kDa (PTM457)

2. Result Statistics

Figure 1. False discovery rate (FDR) curve. X axis is the number of peptide-spectrum matches (PSM) being kept. Y axis is the corresponding FDR.

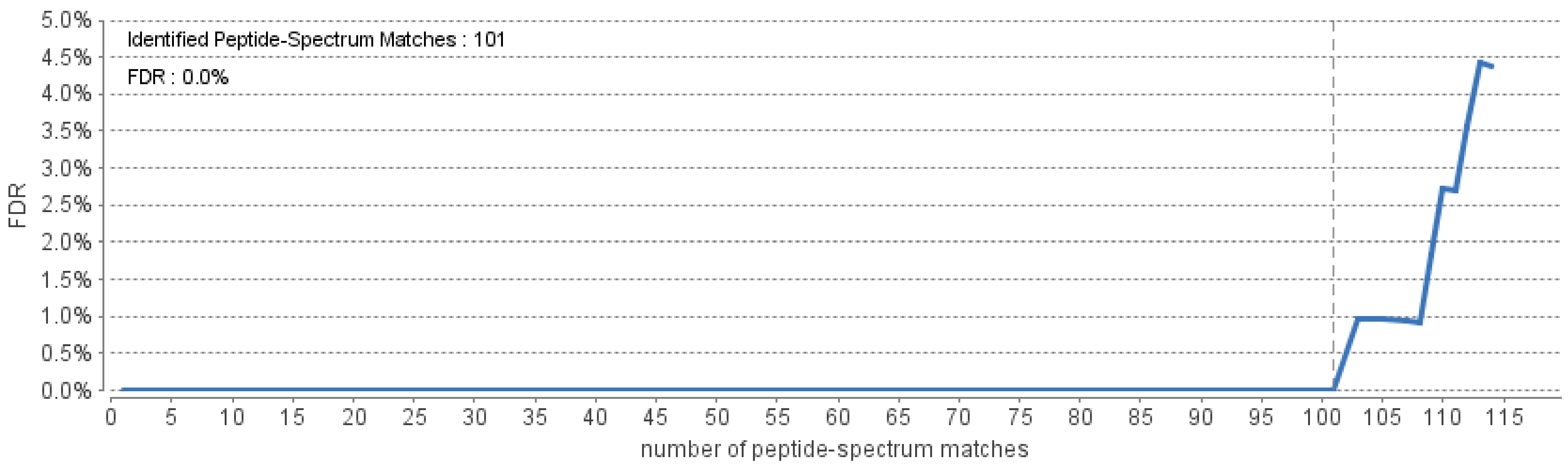

Figure 2. PSM score distribution. **(a)** Distribution of PEAKS peptide score; **(b)** Scatterplot of PEAKS peptide score versus precursor mass error.

(a)

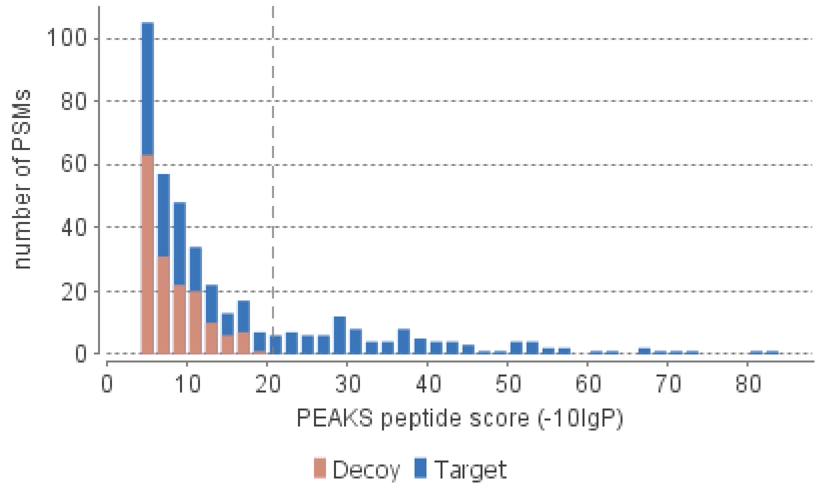

(b)

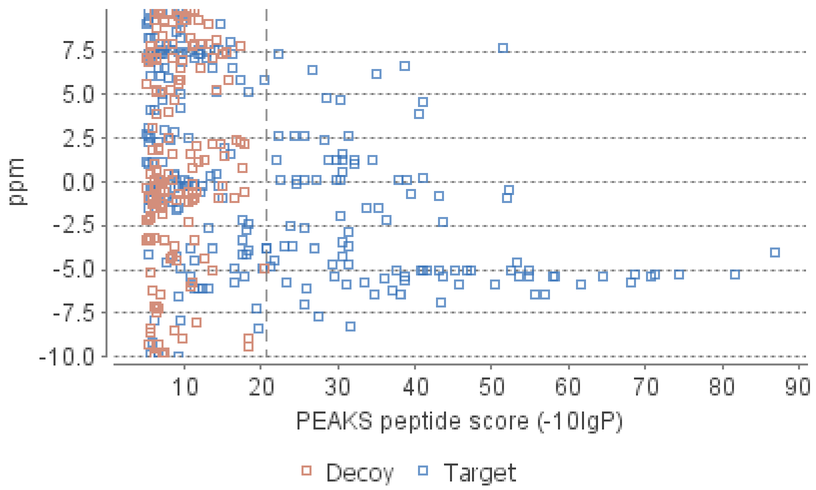

Figure 3. Distribution of peptide feature detection. **(a)** Feature m/z distribution; **(b)** Feature RT distribution

(a)

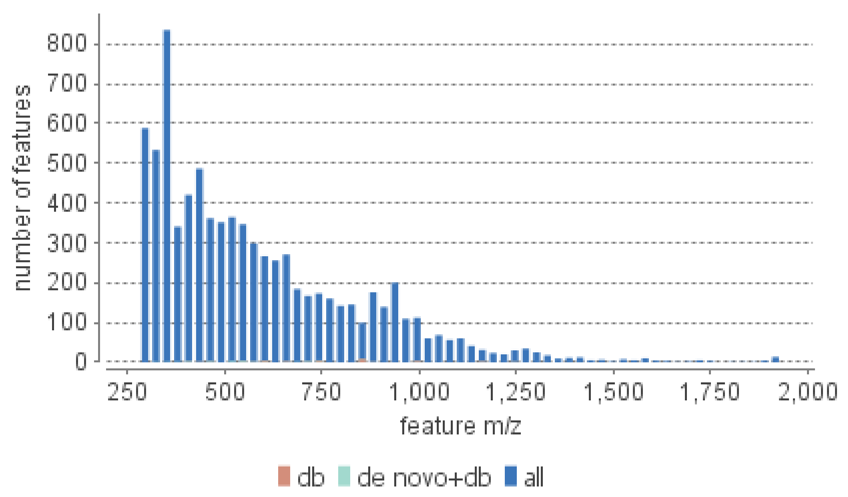

(b)

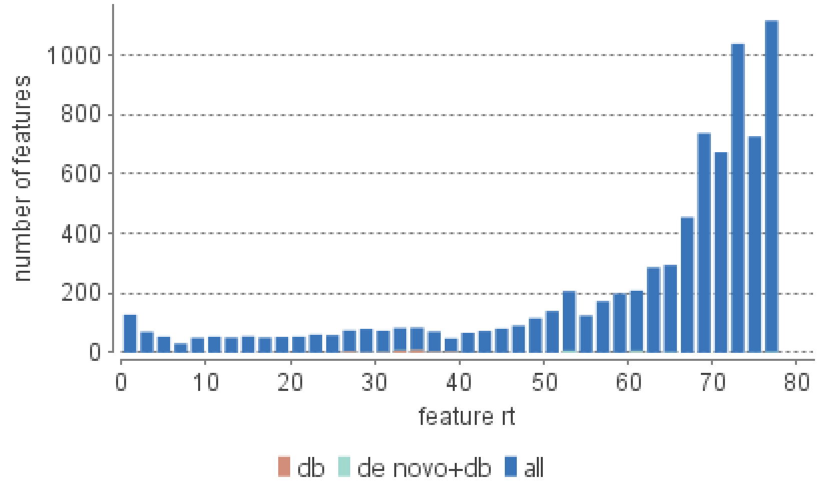

Figure 4. Distribution of identified peptide features. **(a)** Feature abundance distribution **(b)** *De novo* sequencing validation.

(a)

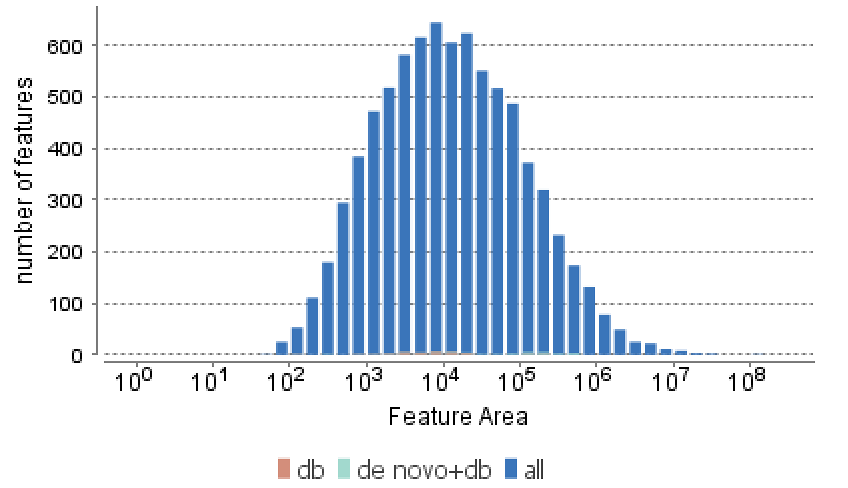

(b)

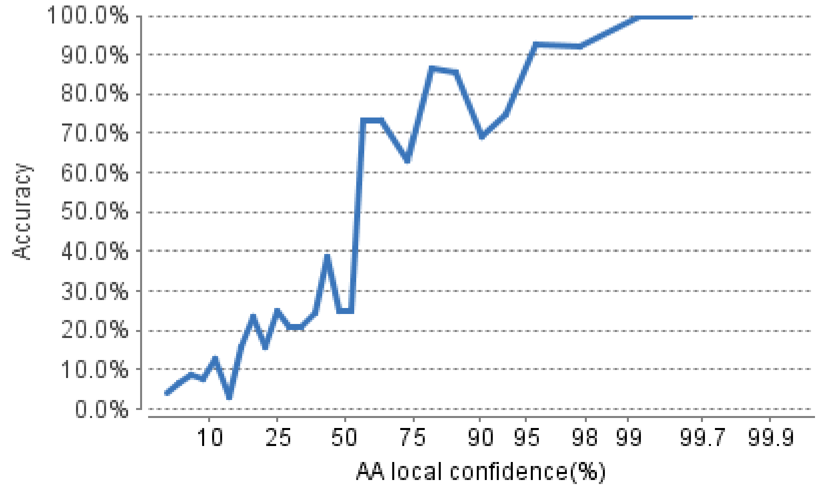

Table 1. Statistics of data.

# of MS scans	2891
# of MS/MS scans	1396
# of Features	8093
# of Chimera scans	110

Table 2. Result filtration parameters.

Peptide -10lgP	≥20.6

Table 4. PTM profile.

Name	ΔMass	Position	#PSM	-10lgP	Abundance	AScore
Deamidation	.98	NQ	76	70.72	1.19E4	6.59
HydPro	15.99	P	3	38.75	2.11E4	30.07

PTM Ascore	≥20
Protein -10lgP	≥20
Proteins unique peptides	≥1
De novo ALC Score	≥50%

Table 3. Statistics of filtered result.

Peptide-Spectrum Matches	101
Peptide sequences	80
Protein groups	6
Proteins	7
Proteins (#Unique Peptides)	2 (>2); 3 (=2); 2 (=1);
FDR (Peptide-Spectrum Matches)	0.0%
FDR (Peptide Sequences)	0.0%
FDR (Protein Group)	0.0%
De Novo Only Spectra	32
# of identified Features	50
# of identified MS/MS scans	97

3. Experiment Control

Figure 5. Precursor mass error of peptide-spectrum matches (PSM) in filtered result. **(a)** Distribution of precursor mass error in ppm; **(b)** Scatterplot of precursor m/z versus precursor mass error in ppm.

(a)

(b)

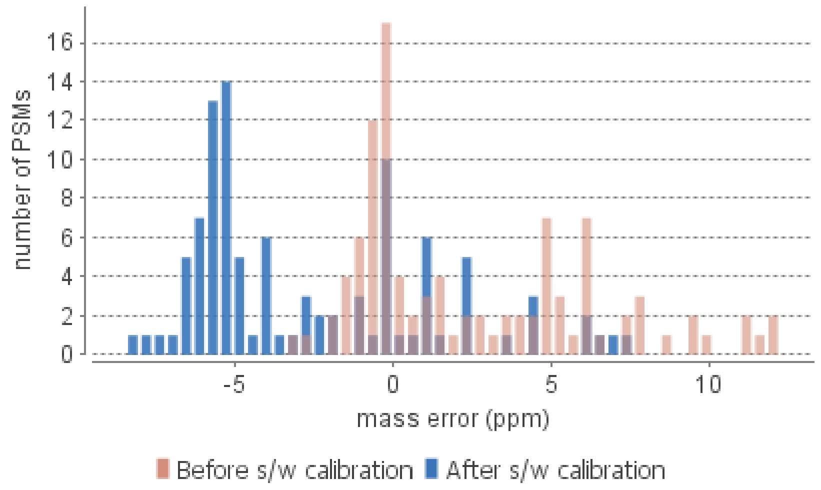

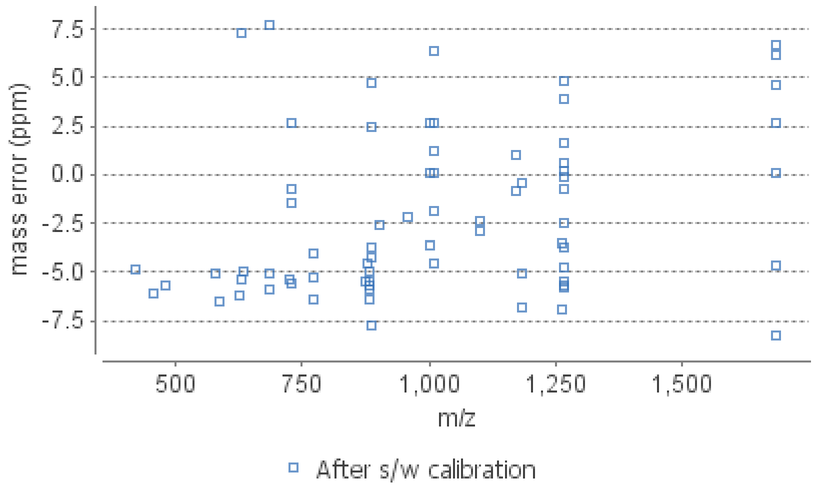

Table 5. Number of identified peptides in each sample by the number of missed cleavages

Missed Cleavages	0	1	2	3	4+
Kontrola 8. traka	44	30	6	0	0

4. Other Information

Table 6. Search parameters.

Search Engine Name: PEAKS
Parent Mass Error Tolerance: 10.0 ppm
Fragment Mass Error Tolerance: 0.5 Da
Precursor Mass Search Type: monoisotopic
Enzyme: Trypsin
Max Missed Cleavages: 2
Digest Mode: Unspecific
Fixed Modifications:
  Carbamidomethylation: 57.02
Variable Modifications:
  Deamidation (NQ): 0.98
  Oxidation (M): 15.99
  Hydroxylation Pro: 15.99
  Acetylation (K): 42.01
  Acetylation (Protein N-term): 42.01
  Acetylation (N-term): 42.01
  Amidation: -0.98
  Beta-methylthiolation: 45.99
  and 305 more...
Max Variable PTM Per Peptide: 5
Database: Uniprot_Peanut-3818_Jul18

Table 7. Instrument parameters.

Fractions: OB4037.raw
Ion Source: ESI(nano-spray)
Fragmentation Mode: CID, CAD(y and b ions)
MS Scan Mode: FT-ICR/Orbitrap
MS/MS Scan Mode: Linear Ion Trap

Taxon: All
Contaminant Database: contaminantsMQ_mar19
Searched Entry: 1723
FDR Estimation: Enabled
De novo score (ALC%) threshold: 15
Peptide hit threshold (-10logP): 30.0
Peaks run ID: 441
Merge Options: no merge
Precursor Options: corrected
Charge Options: no correction
Filter Charge: 2 - 8
Process: true
Associate chimera: yes

Protein List

Protein Accession Contains:
Protein Description Contains:
Peptide Sample Area >=
Protein Ptm Contains:

Protein Group	Protein ID	Accession	-10lgP	Coverage (%)	Coverage (%) Kontrola 8. traka	Area Kontrola 8. traka	#Peptides	#Unique	#Spec Kontrola 8. traka	PTM	Avg. Mass	Description
**1**	20574	tr|A1DZF0|A1DZF0_ARAHY	194.95	35	35	4.03E5	11	7	67	Y	60375	Arachin 6 OS=Arachis hypogaea OX=3818 PE=2 SV=1
**2**	20581	tr|Q647H3|Q647H3_ARAHY	181.57	29	29	6.87E4	10	3	32	Y	61532	Arachin Ahy-2 OS=Arachis hypogaea OX=3818 PE=2 SV=1
**3**	20579	tr|Q5I6T2|Q5I6T2_ARAHY	174.98	29	29	0E0	9	2	25	Y	60736	Arachin Ahy-4 OS=Arachis hypogaea OX=3818 PE=2 SV=1
**4**	20593	tr|Q6IWG5|Q6IWG5_ARAHY	43.35	11	11	3.29E4	2	2	7	Y	58061	Glycinin (Fragment) OS=Arachis hypogaea OX=3818 PE=2 SV=1
**4**	20594	tr|Q0GM57|Q0GM57_ARAHY	43.35	11	11	3.29E4	2	2	7	Y	58263	Iso-Ara h3 OS=Arachis hypogaea OX=3818 PE=2 SV=1
**5**	22368	tr|A1E2B0|A1E2B0_ARAHY	25.85	4	4	1.52E4	1	1	1	N	33520	11S seed storage globulin B1 OS=Arachis hypogaea OX=3818 PE=2 SV=1
**6**	21689	tr|Q2KQ45|Q2KQ45_ARAHY	22.16	7	7	2.03E5	1	1	1	N	19632	Resistance protein PLTR (Fragment) OS=Arachis hypogaea OX=3818 PE=4 SV=1
**total 7 proteins**												

tr|A1DZF0|A1DZF0_ARAHY
back to list

| Protein Coverage | Supporting Peptides |
Protein Coverage:

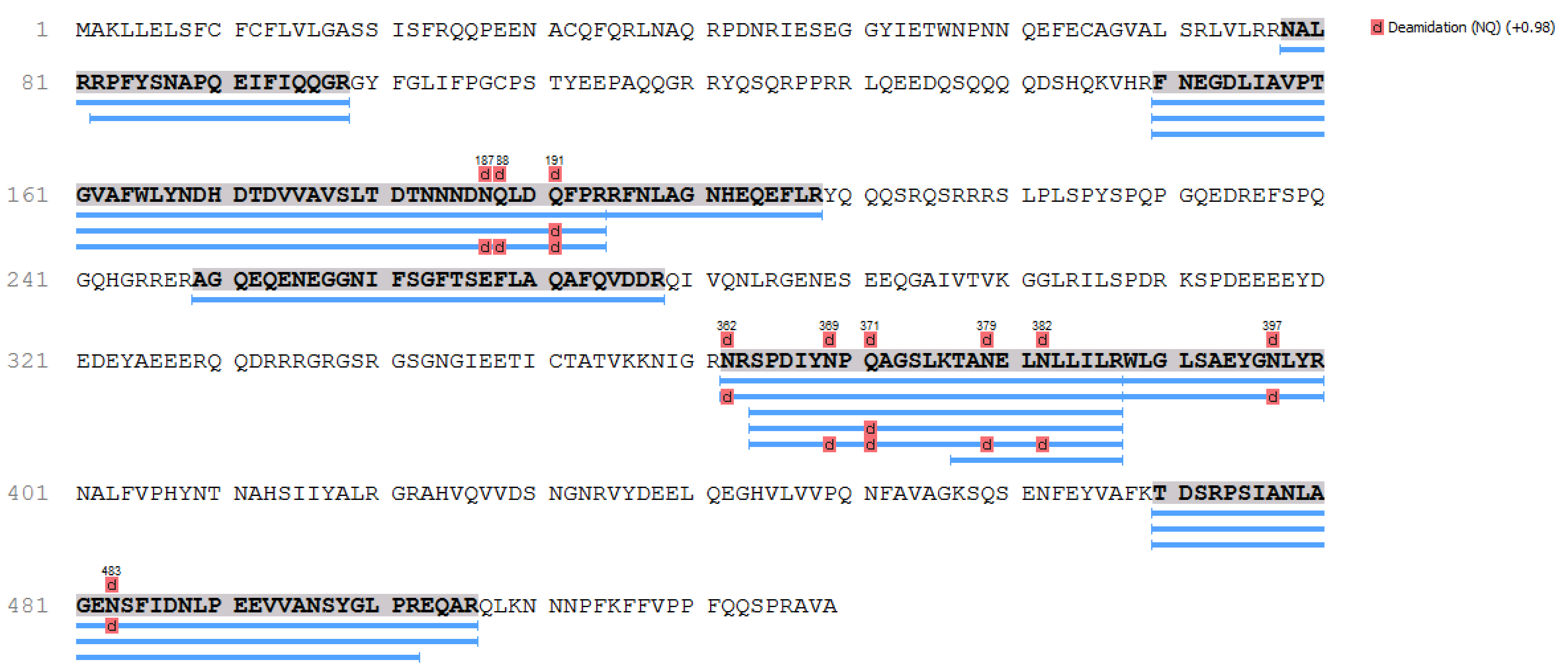

Supporting Peptides:

Peptide	Uniq	-10lgP	Mass	Length	ppm	m/z	z	RT	Fraction	Scan	Source File	Area Kontrola 8. traka	#Feature	#Feature Kontrola 8. traka	Start	End	PTM
R.WLGLSAEYGNLYR.N	N	86.85	1540.7673	13	-4.0	771.3918	2	32.98	9	1648	OB4037.raw	2.84E4	2	2	388	400	
R.SPDIYNPQ(+.98)AGSLKTANELNLLILR.W	Y	70.72	2640.4121	24	-5.4	881.1444	3	34.38	9	1728	OB4037.raw	1.19E4	1	1	364	387	Deamidation (NQ)
R.SPDIYN(+.98)PQAGSLKTANELNLLILR.W	Y	64.60	2640.4121	24	-5.4	881.1444	3	34.38	9	1726	OB4037.raw	1.19E4	1	1	364	387	
R.RPFYSNAPQEIFIQQGR.G	N	61.68	2050.0383	17	-5.9	684.3529	3	30.20	9	1495	OB4037.raw	8.31E3	1	1	82	98	
R.SPDIYN(+.98)PQAGSLKTANELN(+.98)LLILR.W	Y	56.95	2641.3962	24	-6.5	881.4716	3	34.81	9	1755	OB4037.raw	2.79E3	1	1	364	387	
R.WLGLSAEYGN(+.98)LYR.N	N	55.62	1541.7513	13	-6.5	771.8820	2	33.78	9	1693	OB4037.raw	1.46E3	1	1	388	400	Deamidation (NQ)
R.RPFYSNAPQ(+.98)EIFIQQGR.G	N	54.88	2051.0225	17	-5.0	684.6815	3	30.81	9	1534	OB4037.raw	9.16E3	1	1	82	98	
R.RPFYSN(+.98)APQEIFIQQGR.G	N	52.47	2051.0225	17	-5.0	684.6815	3	30.81	9	1530	OB4037.raw	9.16E3	1	1	82	98	
K.TDSRPSIANLAGENSFIDNLPEEVVANSYGLPR.E	Y	52.33	3544.7434	33	-0.4	1182.5940	3	36.01	9	1830	OB4037.raw	0	0	0	470	502	
R.RPFYSN(+.98)APQEIFIQQ(+.98)GR.G	N	51.57	2052.0063	17	7.7	685.0182	3	31.08	9	1544	OB4037.raw	0	0	0	82	98	
R.SPDIYNPQAGSLKTANELNLLILR.W	Y	50.51	2639.4282	24	-5.9	880.8160	3	33.96	9	1706	OB4037.raw	1.78E3	1	1	364	387	
K.TDSRPSIANLAGENSFIDN(+.98)LPEEVVANSYGLPR.E	Y	47.16	3545.7273	33	-5.1	1182.9165	3	36.21	9	1849	OB4037.raw	1.01E4	1	1	470	502	
K.TANELNLLILR.W	Y	45.29	1268.7452	11	-5.0	635.3800	2	33.77	9	1695	OB4037.raw	2.84E3	1	1	377	387	
R.AGQ(+.98)EQEN(+.98)EGGNIFSGFTSEFLAQ(+.98)AFQVDDR.Q	Y	43.59	3293.4272	30	-2.3	1098.8195	3	39.38	9	2029	OB4037.raw	0	0	0	249	278	
R.RFNLAGNHEQEFLR.Y	N	43.03	1729.8647	14	-5.1	577.6289	3	27.56	9	1359	OB4037.raw	4.66E3	1	1	195	208	

Peptide	Uniq	-10lgP	Mass	Length	ppm	m/z	z	RT	Fraction	Scan	Source File	Area Kontrola 8. traka	#Feature	#Feature Kontrola 8. traka	Start	End	PTM
R.FNEGDLIAVPTGVAFWLYNDHDTDVVAVSLTDTN(+.98)N(+.98)NDN(+.98)Q(+.98)LDQ(+.98)FPR.R	Y	42.99	5054.2896	45	-0.8	1264.5852	4	39.20	9	2018	OB4037.raw	0	0	0	150	194	Deamidation (NQ)
R.SPDIYNPQAGSLKTAN(+.98)ELN(+.98)LLILR.W	Y	41.53	2641.3962	24	-5.0	881.4728	3	34.98	9	1766	OB4037.raw	0	0	0	364	387	
R.FNEGDLIAVPTGVAFWLYNDHDTDVVAVSLTDTNN(+.98)NDN(+.98)QLDQFPR.R	Y	40.94	5051.3374	45	0.2	1263.8484	4	38.25	9	1958	OB4037.raw	0	0	0	150	194	
K.TDSRPSIAN(+.98)LAGENSFIDNLPEEVVANSYGLPR.E	Y	40.87	3545.7273	33	-5.1	1182.9165	3	36.21	9	1846	OB4037.raw	1.01E4	1	1	470	502	
R.FNEGDLIAVPTGVAFWLYN(+.98)DHDTDVVAVSLTDTN(+.98)N(+.98)NDNQLDQFPR.R	Y	40.41	5052.3213	45	3.9	1264.0990	4	38.35	9	1965	OB4037.raw	0	0	0	150	194	
R.NRSPDIYN(+.98)PQ(+.98)AGSLKTANELNLLILR.W	Y	39.50	2911.5403	26	-0.7	728.8956	4	33.35	9	1668	OB4037.raw	0	0	0	362	387	
R.FNEGDLIAVPTGVAFWLYNDHDTDVVAVSLTDTN(+.98)N(+.98)N(+.98)DNQLDQFPR.R	Y	39.02	5052.3213	45	0.1	1685.1233	3	38.54	9	1991	OB4037.raw	2.11E4	1	1	150	194	
R.N(+.98)RSPDIYNPQAGSLKTANELN(+.98)LLILR.W	Y	38.78	2911.5403	26	-5.6	728.8920	4	33.38	9	1670	OB4037.raw	0	0	0	362	387	Deamidation (NQ)
R.FNEGDLIAVPTGVAFWLYNDHDTDVVAVSLTDTN(+.98)NNDN(+.98)QLDQ(+.98)FPR.R	Y	37.97	5052.3213	45	0.1	1685.1233	3	38.54	9	1995	OB4037.raw	2.11E4	1	1	150	194	
R.NALRRPFYSNAPQEIFIQQGR.G	N	37.18	2504.3037	21	-6.2	627.0826	4	29.61	9	1463	OB4037.raw	0	0	0	78	98	
R.NRSPDIYN(+.98)PQAGSLKTANELNLLILR.W	Y	35.32	2910.5562	26	-1.5	728.6490	4	33.35	9	1668	OB4037.raw	1.53E2	1	1	362	387	
R.SPDIYN(+.98)PQAGSLKTAN(+.98)ELNLLILR.W	Y	34.86	2641.3962	24	-6.5	881.4716	3	34.81	9	1757	OB4037.raw	2.79E3	1	1	364	387	
K.TDSRPSIANLAGENSFIDN(+.98)LPEEVVAN(+.98)SYGLPREQAR.Q	Y	34.54	4030.9507	37	1.2	1008.7514	4	35.30	9	1788	OB4037.raw	1.85E4	1	1	470	506	
R.N(+.98)RSPDIYNPQAGSLKTANELNLLILR.W	Y	33.66	2910.5562	26	-1.5	728.6490	4	33.35	9	1670	OB4037.raw	1.53E2	1	1	362	387	Deamidation (NQ)
R.SPDIYN(+.98)PQ(+.98)AGSLKTAN(+.98)ELN(+.98)LLILR.W	Y	33.56	2643.3643	24	-5.7	882.1282	3	35.28	9	1786	OB4037.raw	0	0	0	364	387	Deamidation (NQ)
K.TDSRPSIANLAGENSFIDNLPEEVVAN(+.98)SYGLPREQ(+.98)AR.Q	Y	32.12	4030.9507	37	1.2	1008.7514	4	35.30	9	1775	OB4037.raw	1.85E4	1	1	470	506	
R.FNEGDLIAVPTGVAFWLYNDHDTDVVAVSLTDTNNNDN(+.98)QLDQ(+.98)FPR.R	Y	31.76	5051.3374	45	-8.2	1684.7812	3	38.68	9	1986	OB4037.raw	0	0	0	150	194	Deamidation (NQ)
R.AGQEQEN(+.98)EGGNIFSGFTSEFLAQAFQVDDR.Q	Y	31.30	3291.4592	30	-2.9	1098.1628	3	38.78	9	1992	OB4037.raw	0	0	0	249	278	
R.FNEGDLIAVPTGVAFWLYNDHDTDVVAVSLTDTN(+.98)NN(+.98)DNQLDQFPR.R	Y	31.24	5051.3374	45	-3.7	1263.8435	4	38.56	9	1982	OB4037.raw	1.07E5	1	1	150	194	
R.FNEGDLIAVPTGVAFWLYN(+.98)DHDTDVVAVSLTDTNNNDN(+.98)QLDQ(+.98)FPR.R	Y	31.09	5052.3213	45	-5.8	1264.0868	4	38.93	9	2002	OB4037.raw	0	0	0	150	194	Deamidation (NQ)
R.FNEGDLIAVPTGVAFWLYN(+.98)DHDTDVVAVSLTDTNNNDNQLDQFPR.R	Y	30.66	5050.3530	45	-3.5	1263.5977	4	38.18	9	1954	OB4037.raw	0	0	0	150	194	
R.FNEGDLIAVPTGVAFWLYN(+.98)DHDTDVVAVSLTDTN(+.98)NNDN(+.98)Q(+.98)LDQFPR.R	Y	30.57	5053.3052	45	0.6	1264.3408	4	39.32	9	2025	OB4037.raw	0	0	0	150	194	
R.FNEGDLIAVPTGVAFWLYN(+.98)DHDTDVVAVSLTDTN(+.98)N(+.98)NDN(+.98)QLDQ(+.98)FPR.R	Y	30.56	5054.2896	45	1.6	1264.5883	4	39.37	9	2028	OB4037.raw	0	0	0	150	194	
K.TDSRPSIANLAGENSFIDN(+.98)LPEEVVANSYGLPREQ(+.98)AR.Q	Y	30.51	4030.9507	37	1.2	1008.7514	4	35.30	9	1770	OB4037.raw	1.85E4	1	1	470	506	
K.TDSRPSIANLAGENSFIDNLPEEVVANSYGLPREQ(+.98)AR.Q	Y	30.38	4029.9668	37	-1.9	1008.5023	4	35.09	9	1773	OB4037.raw	0	0	0	470	506	
R.FNEGDLIAVPTGVAFWLYNDHDTDVVAVSLTDTN(+.98)N(+.98)N(+.98)DN(+.98)Q(+.98)LDQFPR.R	Y	29.38	5054.2896	45	-4.7	1264.5802	4	39.23	9	2020	OB4037.raw	0	0	0	150	194	
K.TDSRPSIANLAGEN(+.98)SFIDNLPEEVVANSYGLPREQ(+.98)AR.Q	Y	28.84	4030.9507	37	1.2	1008.7514	4	35.30	9	1791	OB4037.raw	1.85E4	1	1	470	506	
K.TDSRPSIAN(+.98)LAGENSFIDN(+.98)LPEEVVANSYGLPREQ(+.98)AR.Q	Y	26.68	4031.9348	37	6.4	1009.0026	4	35.73	9	1814	OB4037.raw	0	0	0	470	506	
R.FNEGDLIAVPTGVAFWLYNDHDTDVVAVSLTDTN(+.98)N(+.98)N(+.98)DNQ(+.98)LDQ(+.98)FPR.R	Y	25.66	5054.2896	45	2.7	1685.7837	3	38.44	9	1971	OB4037.raw	0	0	0	150	194	
R.FNEGDLIAVPTGVAFWLYNDHDTDVVAVSLTDTNN(+.98)NDNQLDQFPR.R	Y	25.51	5050.3530	45	-7.0	1263.5933	4	38.38	9	1967	OB4037.raw	0	0	0	150	194	
R.FNEGDLIAVPTGVAFWLYN(+.98)DHDTDVVAVSLTDTN(+.98)N(+.98)NDN(+.98)Q(+.98)LDQFPR.R	Y	24.50	5054.2896	45	-0.1	1264.5861	4	39.08	9	2011	OB4037.raw	0	0	0	150	194	
R.FNEGDLIAVPTGVAFWLYN(+.98)DHDTDVVAVSLTDTNNNDNQ(+.98)LDQFPR.R	Y	23.74	5051.3374	45	-2.5	1263.8450	4	39.62	9	2042	OB4037.raw	0	0	0	150	194	
R.FNEGDLIAVPTGVAFWLYNDHDTDVVAVSLTDTNNN(+.98)DN(+.98)QLDQ(+.98)FPR.R	Y	23.41	5052.3213	45	-5.7	1264.0869	4	39.05	9	2009	OB4037.raw	0	0	0	150	194	
R.FNEGDLIAVPTGVAFWLYNDHDTDVVAVSLTDTNNNDN(+.98)Q(+.98)LDQFPR.R	Y	23.04	5051.3374	45	-3.7	1263.8435	4	38.56	9	1998	OB4037.raw	1.07E5	1	1	150	194	
R.NRSPDIYNPQAGSLKTAN(+.98)ELN(+.98)LLILR.W	Y	22.16	2911.5403	26	2.6	728.8980	4	33.43	9	1673	OB4037.raw	0	0	0	362	387	
K.TDSRPSIANLAGEN(+.98)SFIDN(+.98)LPEEVVANSYGLPREQAR.Q	Y	21.97	4030.9507	37	1.2	1008.7514	4	35.30	9	1793	OB4037.raw	1.85E4	1	1	470	506	
K.TDSRPSIANLAGEN(+.98)SFIDN(+.98)LPEEVVAN(+.98)SYGLPREQ(+.98)AR.Q	Y	21.65	4032.9187	37	-4.5	1009.2376	4	35.18	9	1779	OB4037.raw	0	0	0	470	506	Deamidation (NQ)

Peptide	Uniq	-10lgP	Mass	Length	ppm	m/z	z	RT	Fraction	Scan	Source File	Area Kontrola 8. traka	#Feature	#Feature Kontrola 8. traka	Start	End	PTM
total 52 peptides																	

tr|Q647H3|Q647H3_ARAHY

back to list

| Protein Coverage | Supporting Peptides |

Protein Coverage:

```
  1 MAKLLALSVC FCFLVLGASS ISFRQQPEEN ACQFQRLNAQ RPDNRLESEG GYIETWNPNN QEFECAGVAL SRLVLRRNAL
 81 RRPFYSNAPQ EIFIQQGRGY FGLIFPGCPS TYEEPAQQGR RHQSQRAPRR FEGEDQSQQQ QQDSHQKVRR FDEGDLIAVP
161 TGVALWMYND HDTDVVAVSL TDTNNNDNQL DQFPRRFNLA GNHEQEFLRY QQQSRRRSLP YSPYSPQSQP RQEEREFSPR
241 GQHSRRERAG QEQENEGGNI FSGFTPEFLA QAFQVDDRQI LQNLRGENES DEQGAIVTVR GGLRILSPDR KRRQQYERPD
321 EEEEYDEDEY EYDEEERQQD RRRGRGSRGR GNGIEETICT ASVKKNIGRN RSPDIYNPQA GSLKTANDLN LLILRWLGLS
401 AEYGNLYRNA LFVPHYNTNA HSIIYALRGR AHVQVVDSNG NRVYDEELQE GHVLVVPQNF AVAGKSQSDN FEYVAFKTDS
481 RPSIANLAGE NSIIDNLPEE VVANSYGLPR EQARQLKNNN PFKFFVPPSQ QSLGAVA
```

Modification sites: 160 h; 377 d, 379 d; 405 d; 486 d, 491 d, 496 d

d Deamidation (NQ) (+0.98)
h Hydroxylation Pro (+15.99)

Supporting Peptides:

Peptide	Uniq	-10lgP	Mass	Length	ppm	m/z	z	RT	Fraction	Scan	Source File	Area Kontrola 8. traka	#Feature	#Feature Kontrola 8. traka	Start	End	PTM
R.WLGLSAEYGNLYR.N	N	86.85	1540.7673	13	-4.0	771.3918	2	32.98	9	1648	OB4037.raw	2.84E4	2	2	396	408	
R.RPFYSNAPQEIFIQQGR.G	N	61.68	2050.0383	17	-5.9	684.3529	3	30.20	9	1495	OB4037.raw	8.31E3	1	1	82	98	
R.SPDIYNPQ(+.98)AGSLKTANDLNLLILR.W	N	58.07	2626.3965	24	-5.4	876.4725	3	34.10	9	1716	OB4037.raw	5.65E3	1	1	372	395	
R.WLGLSAEYGN(+.98)LYR.N	N	55.62	1541.7513	13	-6.5	771.8820	2	33.78	9	1693	OB4037.raw	1.46E3	1	1	396	408	Deamidation (NQ)
R.RPFYSNAPQ(+.98)EIFIQQGR.G	N	54.88	2051.0225	17	-5.0	684.6815	3	30.81	9	1534	OB4037.raw	9.16E3	1	1	82	98	
R.SPDIYN(+.98)PQ(+.98)AGSLKTANDLNLLILR.W	N	53.18	2627.3806	24	-4.6	876.8013	3	34.45	9	1735	OB4037.raw	2.75E3	1	1	372	395	Deamidation (NQ)
R.RPFYSN(+.98)APQEIFIQQGR.G	N	52.47	2051.0225	17	-5.0	684.6815	3	30.81	9	1530	OB4037.raw	9.16E3	1	1	82	98	
K.TDSRPSIANLAGENSIIDN(+.98)LPEEVVANSYGLPR.E	Y	51.84	3511.7429	33	-0.9	1171.5933	3	35.68	9	1816	OB4037.raw	8.32E2	1	1	478	510	
R.RPFYSN(+.98)APQEIFIQQ(+.98)GR.G	N	51.57	2052.0063	17	7.7	685.0182	3	31.08	9	1544	OB4037.raw	0	0	0	82	98	

Peptide	Uniq	-10lgP	Mass	Length	ppm	m/z	z	RT	Fraction	Scan	Source File	Area Kontrola 8. traka	#Feature	#Feature Kontrola 8. traka	Start	End	PTM
R.RFNLAGNHEQEFLR.Y	N	43.03	1729.8647	14	-5.1	577.6289	3	27.56	9	1359	OB4037.raw	4.66E3	1	1	196	209	
R.FDEGDLIAVP(+15.99)TGVALWMYNDHDTDVVAVSLTDTNNN(+.98)DNQLDQ(+.98)FPR.R	Y	38.75	5052.2881	45	6.7	1685.1233	3	38.54	9	1983	OB4037.raw	2.11E4	1	1	151	195	Hydroxylation Pro
K.TANDLNLLILR.W	N	38.66	1254.7296	11	-5.4	628.3719	2	33.48	9	1675	OB4037.raw	3.81E3	1	1	385	395	
R.NALRRPFYSNAPQEIFIQQGR.G	N	37.18	2504.3037	21	-6.2	627.0826	4	29.61	9	1463	OB4037.raw	0	0	0	78	98	
K.TDSRPSIAN(+.98)LAGEN(+.98)SIIDN(+.98)LPEEVVANSYGLPR.E	Y	32.04	3513.7109	33	1.0	1172.2515	3	36.33	9	1847	OB4037.raw	0	0	0	478	510	Deamidation (NQ)
K.TDSRPSIANLAGEN(+.98)SIIDNLPEEVVAN(+.98)SYGLPREQ(+.98)AR.Q	Y	30.39	3997.9504	37	0.1	1000.5001	4	34.81	9	1744	OB4037.raw	8.2E3	1	1	478	514	
K.TDSRPSIAN(+.98)LAGENSIIDNLPEEVVAN(+.98)SYGLPREQ(+.98)AR.Q	Y	29.78	3997.9504	37	0.1	1000.5001	4	34.81	9	1764	OB4037.raw	8.2E3	1	1	478	514	
R.NRSPDIYN(+.98)PQ(+.98)AGSLKTANDLNLLILR.W	N	29.45	2897.5247	26	-5.4	725.3883	4	33.12	9	1656	OB4037.raw	0	0	0	370	395	
R.FDEGDLIAVP(+15.99)TGVALWMYNDHDTDVVAVSLTDTNN(+.98)NDN(+.98)Q(+.98)LDQFPR.R	Y	28.57	5053.2725	45	4.8	1264.3380	4	39.17	9	2016	OB4037.raw	0	0	0	151	195	
K.TDSRPSIANLAGEN(+.98)SIIDN(+.98)LPEEVVANSYGLPREQ(+.98)AR.Q	Y	27.11	3997.9504	37	0.1	1000.5001	4	34.81	9	1746	OB4037.raw	8.2E3	1	1	478	514	
K.TDSRPSIANLAGENSIIDN(+.98)LPEEVVAN(+.98)SYGLPREQ(+.98)AR.Q	Y	24.50	3997.9504	37	0.1	1000.5001	4	34.81	9	1752	OB4037.raw	8.2E3	1	1	478	514	Deamidation (NQ)
K.TDSRPSIAN(+.98)LAGEN(+.98)SIIDNLPEEVVAN(+.98)SYGLPREQ(+.98)AR.Q	Y	24.33	3998.9343	37	2.6	1000.7487	4	35.06	9	1764	OB4037.raw	5.77E3	1	1	478	514	
K.TDSRPSIANLAGEN(+.98)SIIDNLPEEVVANSYGLPREQ(+.98)AR.Q	Y	23.98	3996.9663	37	-3.7	1000.2504	4	34.94	9	1764	OB4037.raw	0	0	0	478	514	
K.TDSRPSIAN(+.98)LAGENSIIDN(+.98)LPEEVVANSYGLPREQ(+.98)AR.Q	Y	22.44	3997.9504	37	0.1	1000.5001	4	34.81	9	1741	OB4037.raw	8.2E3	1	1	478	514	
**total 23 peptides**																	

tr|Q5I6T2|Q5I6T2_ARAHY
back to list

| **Protein Coverage** | **Supporting Peptides** |

Protein Coverage:

d Deamidation (NQ) (+0.98)

```
  1 MAKLLELSFC FCFLVLGASS ISFRQQPEEN ACQFQRLNAQ RPDNRIESEG GYIETWNPNN QEFECAGVAL SRLVLRRNAL
 81 RRPFYSNAPQ EIFIQQGRGY FGLIFPGCPS TYEEPAQQGR RSQSQRPPRR LQGEDQSQQQ QDSHQKVHRF DEGDLIAVPT
161 GVAFWLYNDH DTDVVAVSLT DTNNNDNQLD QFPRRFNLAG NHEQEFLRYQ QQSRQSRRRS LPYSPYSPQS QPRQEEREFS
241 PRGQHSRRER AGQEEENEGG NIFSGFTPEF LEQAFQVDDR QIVQNLRGEN ESEEEGAIVT VRGGLRILSP DRKRGADEEE
                                                                   370 372              398
                                                                    d   d                d
321 EYDEDEYEYD EEDRRRGRGS RGRGNGIEET ICTASVKKNI GRNRSPDIYN PQAGSLKTAN DLNLLILRWL GLSAEYGNLY
                                                                                         d
                                                                    d   d
401 RNALFVPHYN TNAHSIIYAL RGRAHVQVVD SNGNRVYDEE LQEGHVLVVP QNFAVAGKSQ SDNFEYVAFK TDSRPSIANL
481 AGENSVIDNL PEEVVANSYG LQREQARQQL KNNNPFKFFV PPSQQSPRAV A
```

Supporting Peptides:

Peptide	Uniq	-10lgP	Mass	Length	ppm	m/z	z	RT	Fraction	Scan	Source File	Area Kontrola 8. traka	#Feature	#Feature Kontrola 8. traka	Start	End	PTM
R.WLGLSAEYGNLYR.N	N	86.85	1540.7673	13	-4.0	771.3918	2	32.98	9	1648	OB4037.raw	2.84E4	2	2	389	401	
R.RPFYSNAPQEIFIQQGR.G	N	61.68	2050.0383	17	-5.9	684.3529	3	30.20	9	1495	OB4037.raw	8.31E3	1	1	82	98	
R.SPDIYNPQ(+.98)AGSLKTANDLNLLILR.W	N	58.07	2626.3965	24	-5.4	876.4725	3	34.10	9	1716	OB4037.raw	5.65E3	1	1	365	388	
R.WLGLSAEYGN(+.98)LYR.N	N	55.62	1541.7513	13	-6.5	771.8820	2	33.78	9	1693	OB4037.raw	1.46E3	1	1	389	401	Deamidation (NQ)
R.RPFYSNAPQ(+.98)EIFIQQGR.G	N	54.88	2051.0225	17	-5.0	684.6815	3	30.81	9	1534	OB4037.raw	9.16E3	1	1	82	98	
R.SPDIYN(+.98)PQ(+.98)AGSLKTANDLNLLILR.W	N	53.18	2627.3806	24	-4.6	876.8013	3	34.45	9	1735	OB4037.raw	2.75E3	1	1	365	388	Deamidation (NQ)
R.RPFYSN(+.98)APQEIFIQQGR.G	N	52.47	2051.0225	17	-5.0	684.6815	3	30.81	9	1530	OB4037.raw	9.16E3	1	1	82	98	
R.RPFYSN(+.98)APQEIFIQQ(+.98)GR.G	N	51.57	2052.0063	17	7.7	685.0182	3	31.08	9	1544	OB4037.raw	0	0	0	82	98	
R.RFNLAGNHEQEFLR.Y	N	43.03	1729.8647	14	-5.1	577.6289	3	27.56	9	1359	OB4037.raw	4.66E3	1	1	195	208	
R.FDEGDLIAVPTGVAFWLYNDHDTDVVAVSLTDTNN(+.98)N(+.98)DN(+.98)QLDQ(+.98)FPR.R	Y	41.07	5054.2891	45	4.6	1685.7867	3	39.10	9	2012	OB4037.raw	0	0	0	150	194	
K.TANDLNLLILR.W	N	38.66	1254.7296	11	-5.4	628.3719	2	33.48	9	1675	OB4037.raw	3.81E3	1	1	378	388	
R.NALRRPFYSNAPQEIFIQQGR.G	N	37.18	2504.3037	21	-6.2	627.0826	4	29.61	9	1463	OB4037.raw	0	0	0	78	98	
R.FDEGDLIAVPTGVAFWLYN(+.98)DHDTDVVAVSLTDTN(+.98)N(+.98)NDN(+.98)Q(+.98)LDQFPR.R	Y	35.08	5055.2734	45	6.2	1686.1176	3	38.89	9	1999	OB4037.raw	0	0	0	150	194	
R.FDEGDLIAVPTGVAFWLYNDHDTDVVAVSLTDTNNNDNQLDQ(+.98)FPR.R	Y	31.50	5051.3374	45	-4.7	1684.7872	3	38.49	9	1974	OB4037.raw	0	0	0	150	194	
K.TDSRPSIANLAGENSVIDN(+.98)LP(+15.99)EEVVAN(+.98)SYGLQ(+.98)REQAR.Q	Y	31.41	4030.9355	37	2.7	1008.7491	4	34.84	9	1758	OB4037.raw	0	0	0	471	507	
R.NRSPDIYN(+.98)PQ(+.98)AGSLKTANDLNLLILR.W	N	29.45	2897.5247	26	-5.4	725.3883	4	33.12	9	1656	OB4037.raw	0	0	0	363	388	
**total 16 peptides**																	

tr|Q6IWG5|Q6IWG5_ARAHY

back to list

| Protein Coverage | Supporting Peptides |

Protein Coverage:

```
  1 KLLALSLCFC VLVLGASSVT FRQGGEENEC QFQRLNAQRP DNRIESEGGY IETWNPNNQE FQCAGVALSR TVLRRNALRR
 81 PFYSNAPLEI YVQQGSGYFG LIFPGCPSTY EEPAQEGRRY QSQKPSRRFQ VGQDDPSQQQ QDSHQKVHRF DEGDLIAVPT
161 GVAFWMYNDE DTDVVTVTLS DTSSIHNQLD QFPRRFYLAG NQEQEFLRYQ QQQGSRPHYR QISPRVRGDE QENEGSNIFS
241 GFAQEFLQHA FQVDRQTVEN LRGENEREEQ GAIVTVKGGL RILSPDEEDE SSRSPPSRRE EFDEDRSRPQ QRGKYDENRR
                                                            374
                                                            d
321 GYKNGIEETI CSASVKKNLG RSSNPDIYNP QAGSLRSVNE LDLPILGWLG LSAQHGTIYR NAMFVPHYTL NAHTIVVALN
                                                            d
401 GRAHVQVVDS NGNRVYDEEL QEGHVLVVPQ NFAVAAKAQS ENYEYLAFKT DSRPSIANLA GENSIIDNLP EEVVANSYRL
481 PREQARQLKN NNPFKFFVPP FDHQSMREVA
```

d Deamidation (NQ) (+0.98)

Supporting Peptides:

Peptide	Uniq	-10lgP	Mass	Length	ppm	m/z	z	RT	Fraction	Scan	Source File	Area Kontrola 8. traka	#Feature	#Feature Kontrola 8. traka	Start	End	PTM
R.SVNELDLPILGWLGLSAQ(+.98)HGTIYR.N	Y	30.52	2652.3911	24	-4.3	885.1384	3	37.90	9	1938	OB4037.raw	1.21E4	2	2	357	380	Deamidation (NQ)
R.SVNELDLPILGWLGLSAQHGTIYR.N	Y	27.05	2651.4070	24	-3.8	884.8109	3	38.34	9	1976	OB4037.raw	1.03E4	1	1	357	380	
K.TDSRPSIANLAGENSIIDNLPEEVVANSYRLPR.E	Y	25.64	3609.8386	33	-2.6	903.4692	4	34.93	9	1772	OB4037.raw	1.05E4	1	1	450	482	
**total 3 peptides**																	

tr|Q0GM57|Q0GM57_ARAHY

back to list

| Protein Coverage | Supporting Peptides |

Protein Coverage:

d Deamidation (NQ) (+0.98)

1 MAKLLALSLC FCVLVLGASS VTFRQGGEEN ECQFQRLNAQ RPDNRIESEG GYIETWNPNN QEFQCAGVAL SRTVLRRNAL
81 RRPFYSNAPL EIYVQQGSGY FGLIFPGCPS TYEEPAQEGR RYQSQKPSRR FQVGQDDPSQ QQQDSHQKVH RFDEGDLIAV
161 PTGVAFWMYN DEDTDVVTVT LSDTSSIHNQ LDQFPRRFYL AGNQEQEFLR YQQQQGSRPH YRQISPRVRG DEQENEGSNI
241 FSGFAQEFLQ HAFQVDRQTV ENLRGENERE EQGAIVTVKG GLRILSPDEE DESSRSPPSR REEFDEDRSR PQQRGKYDEN
376 d
321 RRGYKNGIEE TICSASVKKN LGRSSNPDIY NPQAGSLR**SV NELDLPILGW LGLSAQHGTI YR**NAMFVPHY TLNAHTIVVA
d
401 LNGRAHVQVV DSNGNRVYDE ELQEGHVLVV PQNFAVAAKA QSENYEYLAF K**TDSRPSIAN LAGENSIIDN LPEEVVANSY**
481 **RLPR**EQARQL KNNNPFKFFV PPFDHQSMRE VA

Supporting Peptides:

Peptide	Uniq	-10lgP	Mass	Length	ppm	m/z	z	RT	Fraction	Scan	Source File	Area Kontrola 8. traka	#Feature	#Feature Kontrola 8. traka	Start	End	PTM
R.SVNELDLPILGWLGLSAQ(+.98)HGTIYR.N	Y	30.52	2652.3911	24	-4.3	885.1384	3	37.90	9	1938	OB4037.raw	1.21E4	2	2	359	382	Deamidation (NQ)
R.SVNELDLPILGWLGLSAQHGTIYR.N	Y	27.05	2651.4070	24	-3.8	884.8109	3	38.34	9	1976	OB4037.raw	1.03E4	1	1	359	382	
K.TDSRPSIANLAGENSIIDNLPEEVVANSYRLPR.E	Y	25.64	3609.8386	33	-2.6	903.4692	4	34.93	9	1772	OB4037.raw	1.05E4	1	1	452	484	
**total 3 peptides**																	

tr|A1E2B0|A1E2B0_ARAHY

back to list

| Protein Coverage | Supporting Peptides |

Protein Coverage:

1 MANSLRLAFL VLFQSSLALS LAHRYPTKCS FDKLVALEPT KRVESEGGFT EYWDSKNDQF QCVGVSALRY SIKPK**GLLLP**
81 **HYINAPR**LQY VLQGTGILET VVPGCPETFR EQTRHGDQHQ KIHATREGDV IVVPTGSAQW IYNSGETDMV IFSVIDSANE
161 DNQLDLKVRK FFLGGKPQEE KGEEGNMFSG LELKTVAESL DIDMGIAGKV QGVDDPRGSM IIVEDELETL SPAVEESGNG
241 NGLIETVCTL RLVHQLAEST DADKYNPRAG FLTALTPLTF PSFNMSNLAL IDVFSTECCD GATLH

Supporting Peptides:

Peptide	Uniq	-10lgP	Mass	Length	ppm	m/z	z	RT	Fraction	Scan	Source File	Area Kontrola 8. traka	#Feature	#Feature Kontrola 8. traka	Start	End	PTM
K.GLLLPHYINAPR.L	Y	25.85	1362.7771	12	-6.1	455.2659	3	30.02	9	1487	OB4037.raw	1.52E4	1	1	76	87	
**total 1 peptides**																	

tr|Q2KQ45|Q2KQ45_ARAHY

back to list

| Protein Coverage | Supporting Peptides |

Protein Coverage:

1 GMGGVGKTTI ARAVFETVRS RFEVTCFLAD VREQCEKKDI VRSQKQLLDQ VNINSNAVHN KYDGRTIIQN SLRLKKVLLV
81 LDDVNHEKLL ENLAGEQDWF GPGSRIIITT RDVEVLKEQA VLETYMVEGL VESEAFNLFC LKAFKQAAEP TEGFLDLSEE
161 VVK**HSGGLPL ALKVLG**

Supporting Peptides:

Peptide	Uniq	-10lgP	Mass	Length	ppm	m/z	z	RT	Fraction	Scan	Source File	Area Kontrola 8. traka	#Feature	#Feature Kontrola 8. traka	Start	End	PTM
K.HSGGLPLALKVLG	Y	22.16	1260.7554	13	7.3	631.3928	2	60.55	9	3132	OB4037.raw	2.03E5	1	1	164	176	
**total 1 peptides**																	

Peptide List

Prepared with PEAKS ™ (bioinfor.com)

Summary

1. Notes Gastric Digest Raw peanut Band #8 27-29 kDa (PTM483)

2. Result Statistics

Figure 1. False discovery rate (FDR) curve. X axis is the number of peptide-spectrum matches (PSM) being kept. Y axis is the corresponding FDR.

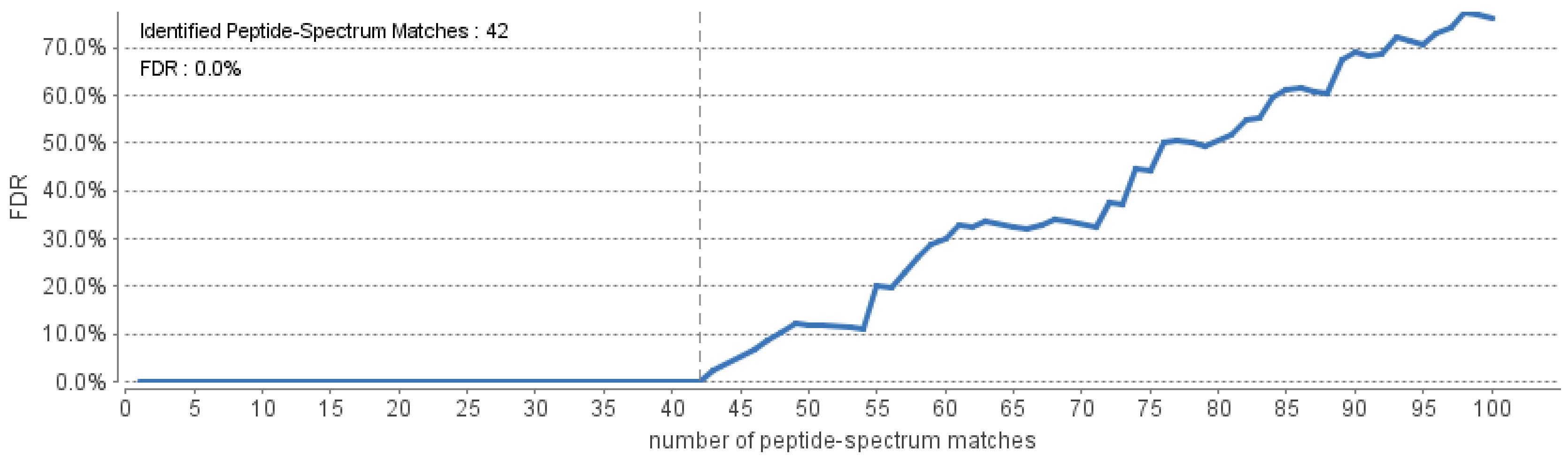

Figure 2. PSM score distribution. **(a)** Distribution of PEAKS peptide score; **(b)** Scatterplot of PEAKS peptide score versus precursor mass error.

(a)

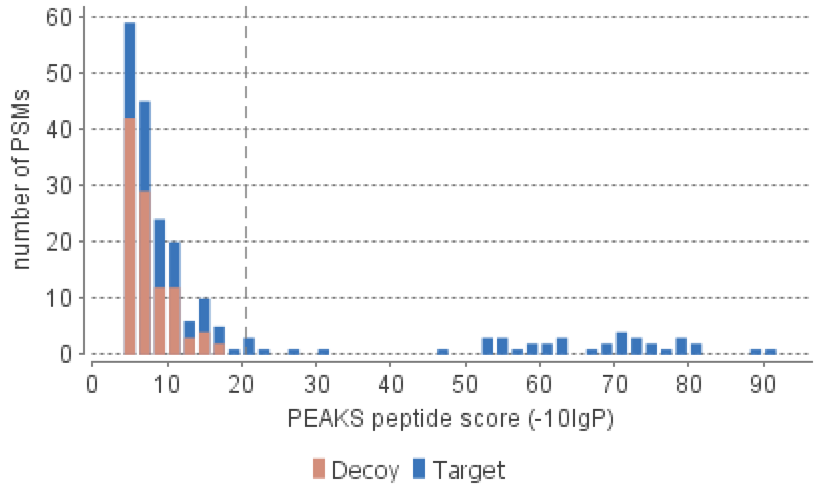

(b)

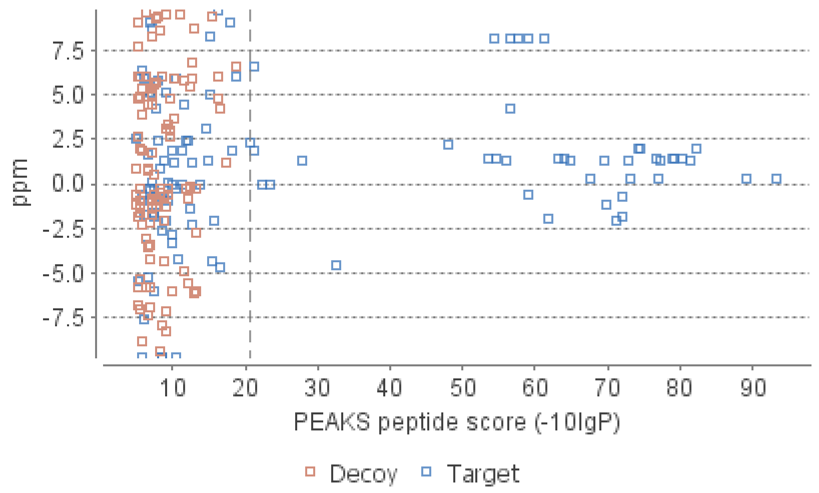

Figure 3. Distribution of peptide feature detection. **(a)** Feature m/z distribution; **(b)** Feature RT distribution

(a)

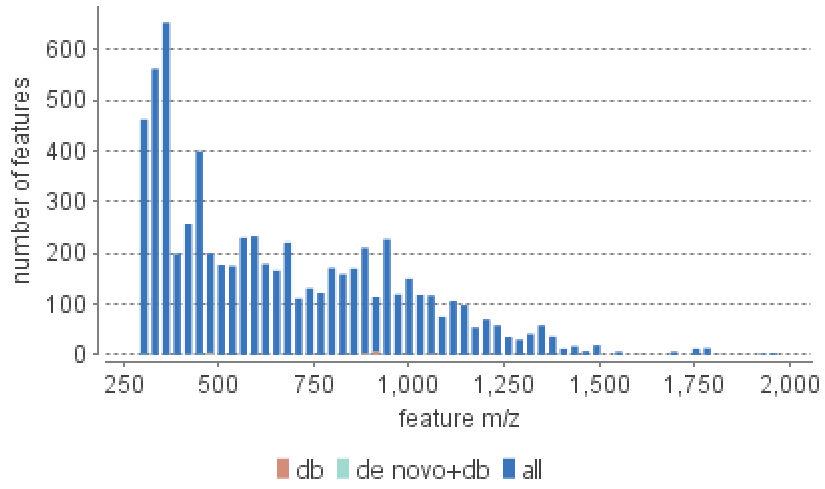

(b)

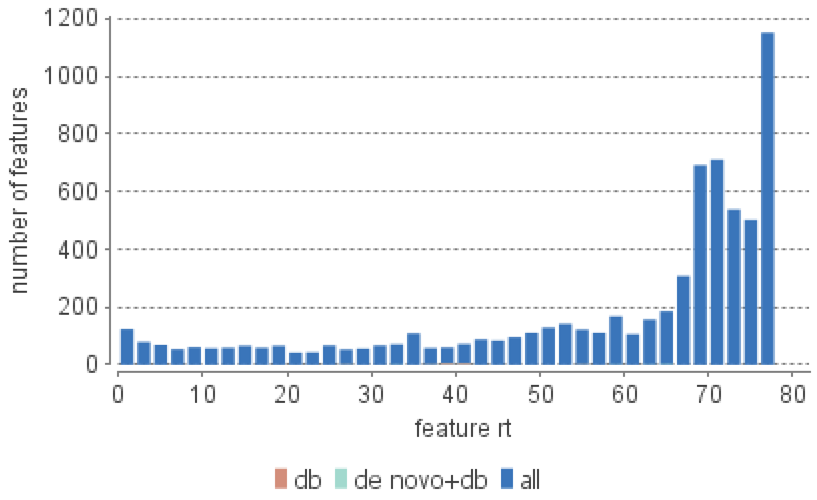

Figure 4. Distribution of identified peptide features. **(a)** Feature abundance distribution **(b)** *De novo* sequencing validation.

(a)

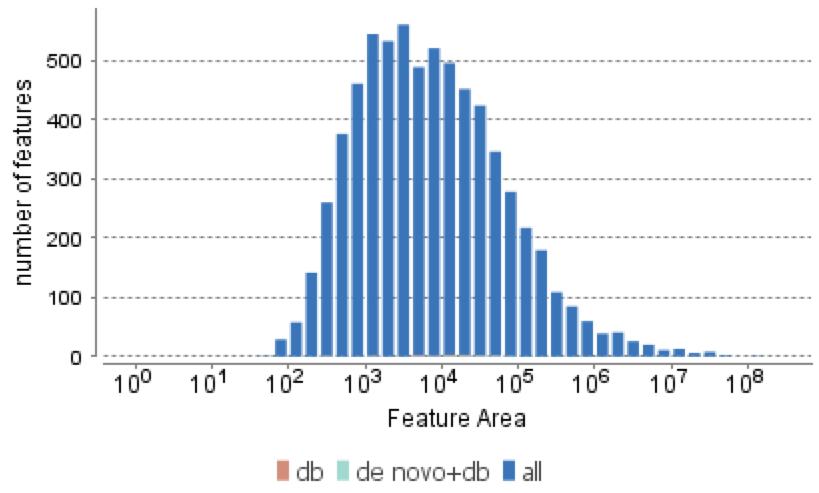

(b)

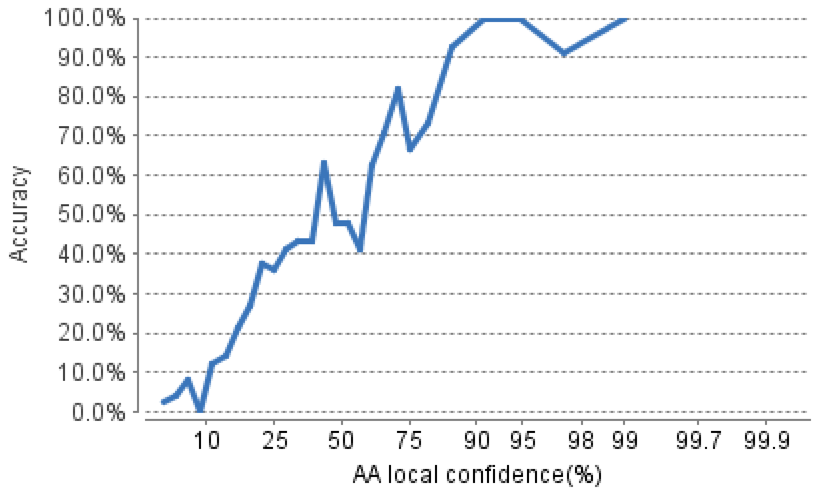

Table 1. Statistics of data.

# of MS scans	3208
# of MS/MS scans	939
# of Features	6790
# of Chimera scans	141

Table 2. Result filtration parameters.

Peptide -10lgP	≥20.7

Table 4. PTM profile.

Name	ΔMass	Position	#PSM	-10lgP	Abundance	AScore
Deamidation	.98	NQ	23	82.27		0.00
ISD_z+2_ion	-15.01	N-term	1	20.71		1000.00
Oxidation	15.99	W	1	32.55		1000.00

PTM Ascore	≥20
Protein -10lgP	≥20
Proteins unique peptides	≥1
De novo ALC Score	≥50%

Table 3. Statistics of filtered result.

Peptide-Spectrum Matches	42
Peptide sequences	16
Protein groups	4
Proteins	11
Proteins (#Unique Peptides)	0 (>2); 3 (=2); 8 (=1);
FDR (Peptide-Spectrum Matches)	0.0%
FDR (Peptide Sequences)	0.0%
FDR (Protein Group)	0.0%
De Novo Only Spectra	16
# of identified Features	16
# of identified MS/MS scans	36

3. Experiment Control

Figure 5. Precursor mass error of peptide-spectrum matches (PSM) in filtered result. **(a)** Distribution of precursor mass error in ppm; **(b)** Scatterplot of precursor m/z versus precursor mass error in ppm.

(a)

(b)

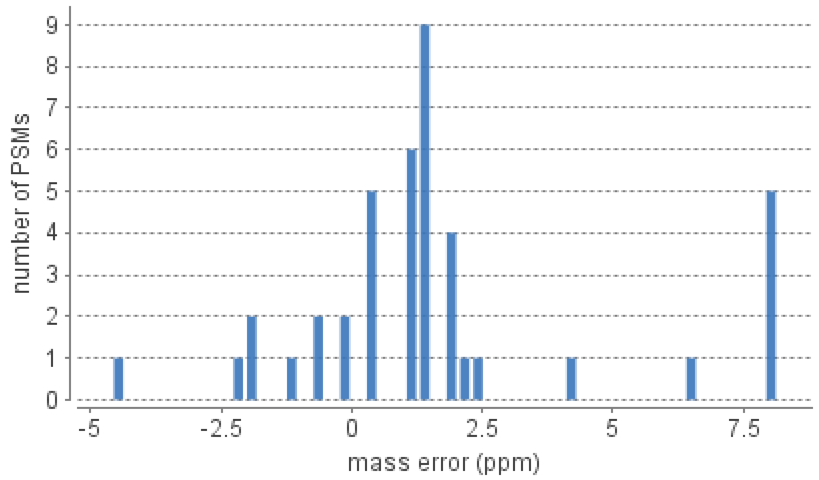

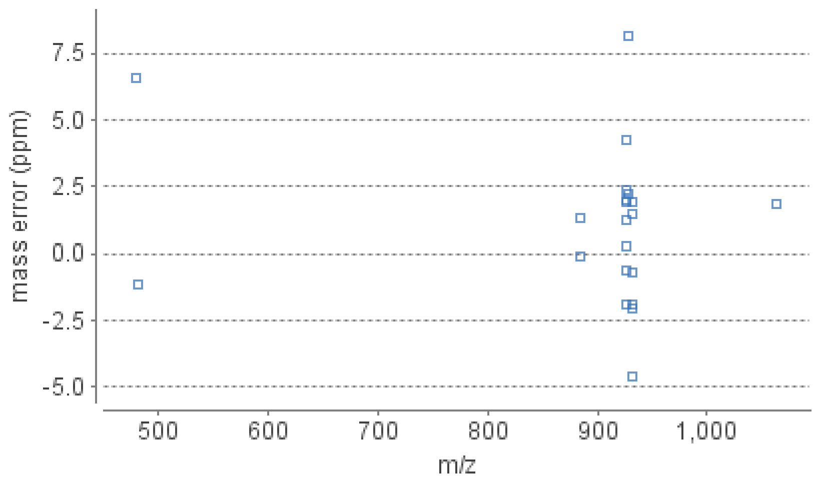

Table 5. Number of identified peptides in each sample by the number of missed cleavages

Missed Cleavages	0	1	2	3	4+
Digest 8. traka	4	12	0	0	0

4. Other Information

Table 6. Search parameters.

Search Engine Name: PEAKS
Parent Mass Error Tolerance: 10.0 ppm
Fragment Mass Error Tolerance: 0.5 Da
Precursor Mass Search Type: monoisotopic
Enzyme: Trypsin
Max Missed Cleavages: 2
Digest Mode: Unspecific
Fixed Modifications:
  Carbamidomethylation: 57.02
Variable Modifications:
  Deamidation (NQ): 0.98
  Oxidation (M): 15.99
  Hydroxylation Pro: 15.99
  Acetylation (K): 42.01
  Acetylation (Protein N-term): 42.01
  Acetylation (N-term): 42.01
  Amidation: -0.98
  Beta-methylthiolation: 45.99
  and 305 more...
Max Variable PTM Per Peptide: 5
Database: Uniprot_Peanut-3818_Jul18

Table 7. Instrument parameters.

Fractions: OB4058.raw
Ion Source: ESI(nano-spray)
Fragmentation Mode: CID, CAD(y and b ions)
MS Scan Mode: FT-ICR/Orbitrap
MS/MS Scan Mode: Linear Ion Trap

Taxon: All
Contaminant Database: contaminantsMQ_mar19
Searched Entry: 1723
FDR Estimation: Enabled
De novo score (ALC%) threshold: 15
Peptide hit threshold (-10logP): 30.0
Peaks run ID: 474
Merge Options: no merge
Precursor Options: corrected
Charge Options: no correction
Filter Charge: 2 - 8
Process: true
Associate chimera: yes

Protein List

Protein Accession Contains:
Protein Description Contains:
Peptide Sample Area >=
Protein Ptm Contains:

Protein Group	Protein ID	Accession	-10lgP	Coverage (%)	Coverage (%) Digest 8. traka	Area Digest 8. traka	#Peptides	#Unique	#Spec Digest 8. traka	PTM	Avg. Mass	Description
**1**	20579	tr|Q5I6T2|Q5I6T2_ARAHY	93.44	5	5	1.08E5	1	1	23	Y	60736	Arachin Ahy-4 OS=Arachis hypogaea OX=3818 PE=2 SV=1
**1**	20576	tr|Q9FZ11|Q9FZ11_ARAHY	93.44	5	5	1.08E5	1	1	23	Y	60449	Gly1 OS=Arachis hypogaea OX=3818 GN=Gly1 PE=2 SV=1
**1**	20581	tr|Q647H3|Q647H3_ARAHY	93.44	4	4	1.08E5	1	1	23	Y	61532	Arachin Ahy-2 OS=Arachis hypogaea OX=3818 PE=2 SV=1
**2**	20578	tr|B5TYU1|B5TYU1_ARAHY	80.27	5	5	2.33E4	1	1	13	Y	60624	Arachin Arah3 isoform OS=Arachis hypogaea OX=3818 PE =1 SV=1
**2**	20574	tr|A1DZF0|A1DZF0_ARAHY	80.27	5	5	2.33E4	1	1	13	Y	60375	Arachin 6 OS=Arachis hypogaea OX=3818 PE=2 SV=1
**2**	20580	tr|Q647H4|Q647H4_ARAHY	80.27	4	4	2.33E4	1	1	13	Y	61506	Arachin Ahy-1 OS=Arachis hypogaea OX=3818 PE=2 SV=1
**2**	20591	tr|Q6T2T4|Q6T2T4_ARAHY	80.27	4	4	2.33E4	1	1	13	Y	61499	Storage protein OS=Arachis hypogaea OX=3818 PE=2 SV= 1
**3**	20593	tr|Q6IWG5|Q6IWG5_ARAHY	38.36	8	8	3.39E4	2	2	4	N	58061	Glycinin (Fragment) OS=Arachis hypogaea OX=3818 PE=2 SV=1
**3**	20595	tr|E5G077|E5G077_ARAHY	38.36	8	8	3.39E4	2	2	4	N	58305	Ara h 3 allergen OS=Arachis hypogaea OX=3818 GN=ara h 3 PE=3 SV=1
**3**	20594	tr|Q0GM57|Q0GM57_ARAHY	38.36	8	8	3.39E4	2	2	4	N	58263	Iso-Ara h3 OS=Arachis hypogaea OX=3818 PE=2 SV=1
**5**	20834	tr|A0A1B3TNS1|A0A1B3TNS1_ARAHY	21.13	1	1	3.4E4	1	1	1	Y	108583	NBS-LRR type disease resistance protein OS=Arachis hypog aea OX=3818 PE=3 SV=1
**total 11 proteins**												

tr|Q5I6T2|Q5I6T2_ARAHY
back to list

| Protein Coverage | Supporting Peptides |
Protein Coverage:

1 MAKLLELSFC FCFLVLGASS ISFRQQPEEN ACQFQRLNAQ RPDNRIESEG GYIETWNPNN QEFECAGVAL SRLVLRRNAL
81 RRPFYSNAPQ EIFIQQGRGY FGLIFPGCPS TYEEPAQQGR RSQSQRPPRR LQGEDQSQQQ QDSHQKVHRF DEGDLIAVPT
161 GVAFWLYNDH DTDVVAVSLT DTNNNDNQLD QFPRRFNLAG NHEQEFLRYQ QQSRQSRRRS LPYSPYSPQS QPRQEEREFS
241 PRGQHSRRER AGQEEENEGG NIFSGFTPEF LEQAFQVDDR QIVQNLRGEN ESEEEGAIVT VRGGLRILSP DRKRGADEEE
321 EYDEDEYEYD EEDRRRGRGS RGRGNGIEET ICTASVKKNI GRNRSPDIYN PQAGSLK**TAN DLNLLILRWL GLSAEYGNLY**
401 **R**NALFVPHYN TNAHSIIYAL RGRAHVQVVD SNGNRVYDEE LQEGHVLVVP QNFAVAGKSQ SDNFEYVAFK TDSRPSIANL
481 AGENSVIDNL PEEVVANSYG LQREQARQQL KNNNPFKFFV PPSQQSPRAV A

(380 d, 383 d, 389 o)

d Deamidation (NQ) (+0.98)
o Oxidation (HW) (+15.99)

Supporting Peptides:

Peptide	Uniq	-10lgP	Mass	Length	ppm	m/z	z	RT	Fraction	Scan	Source File	Area Digest 8. traka	#Feature	#Feature Digest 8. traka	Start	End	PTM
K.TANDLNLLILRWLGLSAEYGNLYR.N	Y	93.44	2777.4863	24	0.3	926.8363	3	39.73	30	2065	OB4058.raw	5.02E4	2	2	378	401	
K.TANDLN(+.98)LLILRWLGLSAEYGNLYR.N	Y	82.27	2778.4705	24	2.0	927.1660	3	40.06	30	2081	OB4058.raw	1.46E4	1	1	378	401	Deamidation (NQ)
K.TAN(+.98)DLNLLILRWLGLSAEYGNLYR.N	Y	69.48	2778.4705	24	1.3	927.1653	3	40.42	30	2093	OB4058.raw	1.46E4	1	1	378	401	
K.TAN(+.98)DLN(+.98)LLILRWLGLSAEYGNLYR.N	Y	61.32	2779.4543	24	8.2	927.4996	3	40.14	30	2093	OB4058.raw	1.5E4	2	2	378	401	Deamidation (NQ)
K.TANDLN(+.98)LLILRWLGLSAEYGN(+.98)LYR.N	Y	56.64	2779.4543	24	8.2	927.4996	3	40.14	30	2081	OB4058.raw	1.37E4	1	1	378	401	
K.TANDLN(+.98)LLILRW(+15.99)LGLSAEYGNLYR.N	Y	32.55	2794.4653	24	-4.6	932.4915	3	40.65	30	2116	OB4058.raw	0	0	0	378	401	Oxidation (HW)
**total 6 peptides**																	

tr|Q9FZ11|Q9FZ11_ARAHY

back to list

| Protein Coverage | Supporting Peptides |

Protein Coverage:

1 MIRGRLALSV CFCFLVLGAS SISFRQQPEE NACQFQRLNA QRPDNRLESE GGYIETWNPN NQEFECAGVA LSRLVLRRNA
81 LRRPFYSNAP QEIFIQQGRG YFGLIFPGCP STYEEPAQQG RRHQSQRAPR RFEGEDQSQQ QQQDSHQKVR RFDEGDLIAV
161 PTGVALWMFN DHDTDVVAVS LTDTNNNDNQ LDQFPRRFNL AGNHEQEFLR YQQQSRRRSL PYSPYSPQSQ PRQEEREFSP
241 RGQHSRRERA GQEEENEGGN IFSGFTPEFL AQAFQVDDRQ IVQNLRGENE SEEEGAIVTV KGGLRILSPD RKRGADEEEE
321 YDEDEYEYDE EDRRRGRGSR GRGNGIEETI CTASVKKNIG RNRSPDIYNP QAGSLK**TAND LNLLILRWLG LSAEYGNLYR**
401 NALFVPHYNT NAHSIIYALR GRAHVQVVDS NGNRVYDEEL QEGHVLVVPQ NFAVAGKSQS DNFEYVAFKT DSRPNIANFA
481 GENSIIDNLP EEVVANSYGL PREQARQLKN NNPFKFFVPP SQQSLRAVA

(379 d, 382 d, 388 o)

d Deamidation (NQ) (+0.98)
o Oxidation (HW) (+15.99)

Supporting Peptides:

Peptide	Uniq	-10lgP	Mass	Length	ppm	m/z	z	RT	Fraction	Scan	Source File	Area Digest 8. traka	#Feature	#Feature Digest 8. traka	Start	End	PTM
K.TANDLNLLILRWLGLSAEYGNLYR.N	Y	93.44	2777.4863	24	0.3	926.8363	3	39.73	30	2065	OB4058.raw	5.02E4	2	2	377	400	
K.TANDLN(+.98)LLILRWLGLSAEYGNLYR.N	Y	82.27	2778.4705	24	2.0	927.1660	3	40.06	30	2081	OB4058.raw	1.46E4	1	1	377	400	Deamidation (NQ)

Peptide	Uniq	-10lgP	Mass	Length	ppm	m/z	z	RT	Fraction	Scan	Source File	Area Digest 8. traka	#Feature	#Feature Digest 8. traka	Start	End	PTM
K.TAN(+.98)DLNLLILRWLGLSAEYGNLYR.N	Y	69.48	2778.4705	24	1.3	927.1653	3	40.42	30	2093	OB4058.raw	1.46E4	1	1	377	400	
K.TAN(+.98)DLN(+.98)LLILRWLGLSAEYGNLYR.N	Y	61.32	2779.4543	24	8.2	927.4996	3	40.14	30	2093	OB4058.raw	1.5E4	2	2	377	400	Deamidation (NQ)
K.TANDLN(+.98)LLILRWLGLSAEYGN(+.98)LYR.N	Y	56.64	2779.4543	24	8.2	927.4996	3	40.14	30	2081	OB4058.raw	1.37E4	1	1	377	400	
K.TANDLN(+.98)LLILRW(+15.99)LGLSAEYGNLYR.N	Y	32.55	2794.4653	24	-4.6	932.4915	3	40.65	30	2116	OB4058.raw	0	0	0	377	400	Oxidation (HW)
**total 6 peptides**																	

tr|Q647H3|Q647H3_ARAHY

back to list

| Protein Coverage | Supporting Peptides |

Protein Coverage:

Deamidation (NQ) (+0.98)
Oxidation (HW) (+15.99)

```
  1  MAKLLALSVC FCFLVLGASS ISFRQQPEEN ACQFQRLNAQ RPDNRLESEG GYIETWNPNN QEFECAGVAL SRLVLRRNAL
 81  RRPFYSNAPQ EIFIQQGRGY FGLIFPGCPS TYEEPAQQGR RHQSQRAPRR FEGEDQSQQQ QQDSHQKVRR FDEGDLIAVP
161  TGVALWMYND HDTDVVAVSL TDTNNNDNQL DQFPRRFNLA GNHEQEFLRY QQQSRRRSLP YSPYSPQSQP RQEEREFSPR
241  GQHSRRERAG QEQENEGGNI FSGFTPEFLA QAFQVDDRQI LQNLRGENES DEQGAIVTVR GGLRILSPDR KRRQQYERPD
                                                                             387 390    396
321  EEEEYDEDEY EYDEEERQQD RRRGRGSRGR GNGIEETICT ASVKKNIGRN RSPDIYNPQA GSLKTANDLN LLILRWLGLS
401  AEYGNLYRNA LFVPHYNTNA HSIIYALRGR AHVQVVDSNG NRVYDEELQE GHVLVVPQNF AVAGKSQSDN FEYVAFKTDS
481  RPSIANLAGE NSIIDNLPEE VVANSYGLPR EQARQLKNNN PFKFFVPPSQ QSLGAVA
```

Supporting Peptides:

Peptide	Uniq	-10lgP	Mass	Length	ppm	m/z	z	RT	Fraction	Scan	Source File	Area Digest 8. traka	#Feature	#Feature Digest 8. traka	Start	End	PTM
K.TANDLNLLILRWLGLSAEYGNLYR.N	Y	93.44	2777.4863	24	0.3	926.8363	3	39.73	30	2065	OB4058.raw	5.02E4	2	2	385	408	
K.TANDLN(+.98)LLILRWLGLSAEYGNLYR.N	Y	82.27	2778.4705	24	2.0	927.1660	3	40.06	30	2081	OB4058.raw	1.46E4	1	1	385	408	Deamidation (NQ)
K.TAN(+.98)DLNLLILRWLGLSAEYGNLYR.N	Y	69.48	2778.4705	24	1.3	927.1653	3	40.42	30	2093	OB4058.raw	1.46E4	1	1	385	408	
K.TAN(+.98)DLN(+.98)LLILRWLGLSAEYGNLYR.N	Y	61.32	2779.4543	24	8.2	927.4996	3	40.14	30	2093	OB4058.raw	1.5E4	2	2	385	408	Deamidation (NQ)
K.TANDLN(+.98)LLILRWLGLSAEYGN(+.98)LYR.N	Y	56.64	2779.4543	24	8.2	927.4996	3	40.14	30	2081	OB4058.raw	1.37E4	1	1	385	408	
K.TANDLN(+.98)LLILRW(+15.99)LGLSAEYGNLYR.N	Y	32.55	2794.4653	24	-4.6	932.4915	3	40.65	30	2116	OB4058.raw	0	0	0	385	408	Oxidation (HW)
**total 6 peptides**																	

tr|B5TYU1|B5TYU1_ARAHY

back to list

| Protein Coverage | Supporting Peptides |

Protein Coverage:

1 MAKLLELSFC FCFLVLGASS ISFRQQPEEN ACQFQRLNAQ RPDNRIESEG GYIETWNPNN QEFECAGVAL SRLVLRRNAL i ISD (z+2)-series (-15.01)

81 RRPFYSNAPQ EIFIQQGRGY FGLIFPGCPS TYEEPAQQGR RYQSQRPPRR LQEEDQSQQQ QDSHQKVHRF NEGDLIAVPT

161 GVAFWLYNDH DTDVVAVSLT DTNNNDNQLD QFPRRFNLAG NHEQEFLRYQ QQSRQSRRRS LPYSPYSPQS QPRQEEREFS

241 PRGQHSRRER AGQEEENEGG NIFSGFTPEF LAQAFQVDDR QIVQNLRGEN ESEEQGAIVT VRGGLRILSP DRKRGADEEE

378

321 EYDEDEYEYD EEDRRRGRGS RGSGNGIEET ICTATVKKNI GRNRSPDIYN PQAGSLK**TAN ELNLLILRWL GLSAEYGNLY**

401 **R**NALFVPHYN TNAHSIIYAL RGRAHVQVVD SNGNRVYDEE LQEGHVLVVP QNFAVAGKSQ SDNFEYVAFK TDSRPSIANL

481 AGENSVIDNL PEEVVANSYG LPREQARQLK NNNPFKFFVP PSQQSPRAVA

Supporting Peptides:

Peptide	Uniq	-10lgP	Mass	Length	ppm	m/z	z	RT	Fraction	Scan	Source File	Area Digest 8. traka	#Feature	#Feature Digest 8. traka	Start	End	PTM
K.TANELNLLILRWLGLSAEYGNLYR.N	Y	80.27	2791.5020	24	1.5	931.5093	3	39.76	30	2057	OB4058.raw	2.33E4	1	1	378	401	
K.TAN(+.98)ELNLLILRWLGLSAEYGNLYR.N	Y	74.16	2792.4861	24	1.9	931.8378	3	40.03	30	2079	OB4058.raw	0	0	0	378	401	
K.TANELN(+.98)LLILRWLGLSAEYGNLYR.N	Y	72.04	2792.4861	24	-0.7	931.8353	3	40.32	30	2097	OB4058.raw	0	0	0	378	401	
K.T(-15.01)ANELNLLILRWLGLSAEYGNLYR.N	Y	20.71	2776.4912	24	2.4	926.5065	3	40.72	30	2120	OB4058.raw	0	0	0	378	401	ISD (z+2)-series
**total 4 peptides**																	

tr|A1DZF0|A1DZF0_ARAHY
back to list

| Protein Coverage | Supporting Peptides |

Protein Coverage:

1 MAKLLELSFC FCFLVLGASS ISFRQQPEEN ACQFQRLNAQ RPDNRIESEG GYIETWNPNN QEFECAGVAL SRLVLRRNAL i ISD (z+2)-series (-15.01)

81 RRPFYSNAPQ EIFIQQGRGY FGLIFPGCPS TYEEPAQQGR RYQSQRPPRR LQEEDQSQQQ QDSHQKVHRF NEGDLIAVPT

161 GVAFWLYNDH DTDVVAVSLT DTNNNDNQLD QFPRRFNLAG NHEQEFLRYQ QQSRQSRRRS LPLSPYSPQP GQEDREFSPQ

241 GQHGRRERAG QEQENEGGNI FSGFTSEFLA QAFQVDDRQI VQNLRGENES EEQGAIVTVK GGLRILSPDR KSPDEEEEYD

377

321 EDEYAEEERQ QDRRRGRGSR GSGNGIEETI CTATVKKNIG RNRSPDIYNP QAGSLK**TANE LNLLILRWLG LSAEYGNLYR**

401 NALFVPHYNT NAHSIIYALR GRAHVQVVDS NGNRVYDEEL QEGHVLVVPQ NFAVAGKSQS ENFEYVAFKT DSRPSIANLA

481 GENSFIDNLP EEVVANSYGL PREQARQLKN NNPFKFFVPP FQQSPRAVA

Supporting Peptides:

Peptide	Uniq	-10lgP	Mass	Length	ppm	m/z	z	RT	Fraction	Scan	Source File	Area Digest 8. traka	#Feature	#Feature Digest 8. traka	Start	End	PTM
K.TANELNLLILRWLGLSAEYGNLYR.N	Y	80.27	2791.5020	24	1.5	931.5093	3	39.76	30	2057	OB4058.raw	2.33E4	1	1	377	400	
K.TAN(+.98)ELNLLILRWLGLSAEYGNLYR.N	Y	74.16	2792.4861	24	1.9	931.8378	3	40.03	30	2079	OB4058.raw	0	0	0	377	400	
K.TANELN(+.98)LLILRWLGLSAEYGNLYR.N	Y	72.04	2792.4861	24	-0.7	931.8353	3	40.32	30	2097	OB4058.raw	0	0	0	377	400	
K.T(-15.01)ANELNLLILRWLGLSAEYGNLYR.N	Y	20.71	2776.4912	24	2.4	926.5065	3	40.72	30	2120	OB4058.raw	0	0	0	377	400	ISD (z+2)-series
**total 4 peptides**																	

tr|Q647H4|Q647H4_ARAHY
back to list

| Protein Coverage | Supporting Peptides |

Protein Coverage:

```
  1 MGKLLALSVC FCFLVLGASS ISFRQQPEEN ACQFQRLNAQ RPDNRIESEG GYIETWNPNN QEFECAGVAL SRLVLRRNAL
 81 RRPFYSNAPQ EIFIQQGRGY FGLIFPGCPS TYEEPAQQGR RHQSQRPPRR FQGQDQSQQQ QDSHQKVHRF DEGDLIAVPT
161 GVAFWMYNDH DTDVVAVSLT DTNNNDNQLD QFPRRFNLAG NHEQEFLRYQ QQSRRRSLPY SPYSPQTQPK QEDREFSPRG
241 QHGRRERAGQ EQENEGGNIF SGFTPEFLAQ AFQVDDRQIL QNLRGENESD EQGAIVTVRG GLRILSPDRK RRQQYERPDE
321 EEEYDEDEYE YDEEERQHDR RRGRGSRGSG NGIEETICTA SFKKNIGRNR SPDIYNPQAG SLKTANELNL LILRWLGLSA
401 EYGNLYRNAL FVPHYNTNAH SIIYALRGRA HVQVVDSNGD RVFDEELQEG HVLVVPQNFA VAGKSQSENF EYVAFKTDSR
481 PSIANLAGEN SFIDNLPEEV VANSYGLPRE QARQLKNNNP FKFFVPPSEQ SLRAVA
```

i ISD (z+2)-series (-15.01)

384 i

Supporting Peptides:

Peptide	Uniq	-10lgP	Mass	Length	ppm	m/z	z	RT	Fraction	Scan	Source File	Area Digest 8. traka	#Feature	#Feature Digest 8. traka	Start	End	PTM
K.TANELNLLILRWLGLSAEYGNLYR.N	Y	80.27	2791.5020	24	1.5	931.5093	3	39.76	30	2057	OB4058.raw	2.33E4	1	1	384	407	
K.TAN(+.98)ELNLLILRWLGLSAEYGNLYR.N	Y	74.16	2792.4861	24	1.9	931.8378	3	40.03	30	2079	OB4058.raw	0	0	0	384	407	
K.TANELN(+.98)LLILRWLGLSAEYGNLYR.N	Y	72.04	2792.4861	24	-0.7	931.8353	3	40.32	30	2097	OB4058.raw	0	0	0	384	407	
K.T(-15.01)ANELNLLILRWLGLSAEYGNLYR.N	Y	20.71	2776.4912	24	2.4	926.5065	3	40.72	30	2120	OB4058.raw	0	0	0	384	407	ISD (z+2)-series
**total 4 peptides**																	

tr|Q6T2T4|Q6T2T4_ARAHY

back to list

| Protein Coverage | Supporting Peptides |

Protein Coverage:

```
  1 MGKLLALSVC FCFLVLGASS ISFRQQPEEN ACQFQRLNAQ RPDNRIESEG GYIETWNPNN QEFECAGVAL SRLVLRRNAL
 81 RRPFYSNAPQ EIFIQQGRAY FGLIFLGCPS TYEEPAQQGR RHQSQRPPRR FQGQDQSQQQ QDSHQKVHRF DEGDLIAVPT
161 GVAFWMYNDH DTDVVAVSLT DTNNNDNQLD QFPRRFNLAG NHEQEFLRYQ QQSRRRSLPY SPYSPQTQPK QEDREFSPRG
241 QHGRRERAGQ EQENEGGNIF SGFTPEFLAQ AFQVDDRQIL QNLRGENESD EQGAIVTVRG GLRILSPDRK KRQQYERPDE
321 EEEYDEDEYE YDEEERQQDR RRGRGSRGSG NGIEETICTA SFKKNIGRNR SPDIYNPQAG SLKTANELNL LILRWLGLSA
401 EYGNLYRNAL FVPHYNTNAH SIIYALRGRA HVQVVDSNGD RVFDEELQEG HVLVVPQNFA VAGKSQSENF EYVAFKTDSR
481 PSIANLAGEN SFIDNLPEEV VANSYGLPRE QARQLKNNNP FKFFVPPSEQ SLRAVA
```

i ISD (z+2)-series (-15.01)

384 i

Supporting Peptides:

Peptide	Uniq	-10lgP	Mass	Length	ppm	m/z	z	RT	Fraction	Scan	Source File	Area Digest 8. traka	#Feature	#Feature Digest 8. traka	Start	End	PTM
K.TANELNLLILRWLGLSAEYGNLYR.N	Y	80.27	2791.5020	24	1.5	931.5093	3	39.76	30	2057	OB4058.raw	2.33E4	1	1	384	407	
K.TAN(+.98)ELNLLILRWLGLSAEYGNLYR.N	Y	74.16	2792.4861	24	1.9	931.8378	3	40.03	30	2079	OB4058.raw	0	0	0	384	407	
K.TANELN(+.98)LLILRWLGLSAEYGNLYR.N	Y	72.04	2792.4861	24	-0.7	931.8353	3	40.32	30	2097	OB4058.raw	0	0	0	384	407	
K.T(-15.01)ANELNLLILRWLGLSAEYGNLYR.N	Y	20.71	2776.4912	24	2.4	926.5065	3	40.72	30	2120	OB4058.raw	0	0	0	384	407	ISD (z+2)-series

Peptide	Uniq	-10lgP	Mass	Length	ppm	m/z	z	RT	Fraction	Scan	Source File	Area Digest 8. traka	#Feature	#Feature Digest 8. traka	Start	End	PTM
total 4 peptides																	

tr|Q6IWG5|Q6IWG5_ARAHY
back to list

| Protein Coverage | Supporting Peptides |

Protein Coverage:

```
  1  KLLALSLCFC VLVLGASSVT FRQGGEENEC QFQRLNAQRP DNRIESEGGY IETWNPNNQE FQCAGVALSR TVLRRNALRR
 81  PFYSNAPLEI YVQQGSGYFG LIFPGCPSTY EEPAQEGRRY QSQKPSRRFQ VGQDDPSQQQ QDSHQKVHRF DEGDLIAVPT
161  GVAFWMYNDE DTDVVTVTLS DTSSIHNQLD QFPRRFYLAG NQEQEFLRYQ QQQGSRPHYR QISPRVRGDE QENEGSNIFS
241  GFAQEFLQHA FQVDRQTVEN LRGENEREEQ GAIVTVKGGL RILSPDEEDE SSRSPPSRRE EFDEDRSRPQ QRGKYDENRR
321  GYKNGIEETI CSASVKKNLG RSSNPDIYNP QAGSLRSVNE LDLPILGWLG LSAQHGTIYR NAMFVPHYTL NAHTIVVALN
401  GRAHVQVVDS NGNRVYDEEL QEGHVLVVPQ NFAVAAKAQS ENYEYLAFKT DSRPSIANLA GENSIIDNLP EEVVANSYRL
481  PREQARQLKN NNPFKFFVPP FDHQSMREVA
```

Supporting Peptides:

Peptide	Uniq	-10lgP	Mass	Length	ppm	m/z	z	RT	Fraction	Scan	Source File	Area Digest 8. traka	#Feature	#Feature Digest 8. traka	Start	End	PTM
R.SVNELDLPILGWLGLSAQHGTIYR.N	Y	27.74	2651.4070	24	1.3	884.8108	3	38.18	30	1981	OB4058.raw	4.18E3	1	1	357	380	
R.SVN(+.98)ELDLPILGWLGLSAQHGTIYR.N	Y	23.47	2652.3911	24	-0.1	885.1376	3	38.40	30	1996	OB4058.raw	6.94E3	1	1	357	380	
R.SVNELDLPILGWLGLSAQ(+.98)HGTIYR.N	Y	22.33	2652.3911	24	-0.1	885.1376	3	38.40	30	1988	OB4058.raw	6.94E3	1	1	357	380	
R.SSNPDIYNPQ(+.98)AGSLRSVNELDLPILGWLGLSAQHGTIYR.N	Y	21.23	4252.1553	39	1.9	1064.0481	4	37.35	30	1928	OB4058.raw	1.59E4	1	1	342	380	
total 4 peptides																	

tr|E5G077|E5G077_ARAHY
back to list

| Protein Coverage | Supporting Peptides |

Protein Coverage:

```
  1  MAKLLALSLC FCVLVLGASS VTFRQGGEEN ECQFQRLNAQ RPDNRIESEG GYIETWNPNN QEFQCAGVAL SRTVLRRNAL
 81  RRPFYSNAPL EIYVQQGSGY FGLIFPGCPS TYEEPAQEGR RYQSQKPSRR FQVGQDDPSQ QQQDSHQKVH RFDEGDLIAV
161  PTGVAFWMYN DEDTDVVTVT LSDTSSIHNQ LDQFPRRFYL AGNQEQEFLR YQQQQGSRPH YRQISPRVRG DEQENEGSNI
241  FSGFAQEFLQ HAFQVDRQTV ENLRGENERE EQGAIVTVKG GLRILSPDEE DESSRSPPNR REEFDEDRSR PQQRGKYDEN
321  RRGYKNGIEE TICSASVKKN LGRSSNPDIY NPQAGSLRSV NELDLPILGW LGLSAQHGTI YRNAMFVPHY TLNAHTIVVA
401  LNGRAHVQVV DSNGNRVYDE ELQEGHVLVV PQNFAVAAKA QSENYEYLAF KTDSRPSIAN QAGENSIIDN LPEEVVANSY
481  RLPREQARQL KNNNPFKFFV PPFDHQSMRE VA
```

Supporting Peptides:

Peptide	Uniq	-10lgP	Mass	Length	ppm	m/z	z	RT	Fraction	Scan	Source File	Area Digest 8. traka	#Feature	#Feature Digest 8. traka	Start	End	PTM
R.SVNELDLPILGWLGLSAQHGTIYR.N	Y	27.74	2651.4070	24	1.3	884.8108	3	38.18	30	1981	OB4058.raw	4.18E3	1	1	359	382	
R.SVN(+.98)ELDLPILGWLGLSAQHGTIYR.N	Y	23.47	2652.3911	24	-0.1	885.1376	3	38.40	30	1996	OB4058.raw	6.94E3	1	1	359	382	
R.SVNELDLPILGWLGLSAQ(+.98)HGTIYR.N	Y	22.33	2652.3911	24	-0.1	885.1376	3	38.40	30	1988	OB4058.raw	6.94E3	1	1	359	382	
R.SSNPDIYNPQ(+.98)AGSLRSVNELDLPILGWLGLSAQHGTIYR.N	Y	21.23	4252.1553	39	1.9	1064.0481	4	37.35	30	1928	OB4058.raw	1.59E4	1	1	344	382	

Peptide	Uniq	-10lgP	Mass	Length	ppm	m/z	z	RT	Fraction	Scan	Source File	Area Digest 8. traka	#Feature	#Feature Digest 8. traka	Start	End	PTM
**total 4 peptides**																	

tr|Q0GM57|Q0GM57_ARAHY
back to list

| Protein Coverage | Supporting Peptides |

Protein Coverage:

```
  1 MAKLLALSLC FCVLVLGASS VTFRQGGEEN ECQFQRLNAQ RPDNRIESEG GYIETWNPNN QEFQCAGVAL SRTVLRRNAL
 81 RRPFYSNAPL EIYVQQGSGY FGLIFPGCPS TYEEPAQEGR RYQSQKPSRR FQVGQDDPSQ QQQDSHQKVH RFDEGDLIAV
161 PTGVAFWMYN DEDTDVVTVT LSDTSSIHNQ LDQFPRRFYL AGNQEQEFLR YQQQQGSRPH YRQISPRVRG DEQENEGSNI
241 FSGFAQEFLQ HAFQVDRQTV ENLRGENERE EQGAIVTVKG GLRILSPDEE DESSRSPPSR REEFDEDRSR PQQRGKYDEN
321 RRGYKNGIEE TICSASVKKN LGRSSNPDIY NPQAGSLRSV NELDLPILGW LGLSAQHGTI YRNAMFVPHY TLNAHTIVVA
401 LNGRAHVQVV DSNGNRVYDE ELQEGHVLVV PQNFAVAAKA QSENYEYLAF KTDSRPSIAN LAGENSIIDN LPEEVVANSY
481 RLPREQARQL KNNNPFKFFV PPFDHQSMRE VA
```

Supporting Peptides:

Peptide	Uniq	-10lgP	Mass	Length	ppm	m/z	z	RT	Fraction	Scan	Source File	Area Digest 8. traka	#Feature	#Feature Digest 8. traka	Start	End	PTM
R.SVNELDLPILGWLGLSAQHGTIYR.N	Y	27.74	2651.4070	24	1.3	884.8108	3	38.18	30	1981	OB4058.raw	4.18E3	1	1	359	382	
R.SVN(+.98)ELDLPILGWLGLSAQHGTIYR.N	Y	23.47	2652.3911	24	-0.1	885.1376	3	38.40	30	1996	OB4058.raw	6.94E3	1	1	359	382	
R.SVNELDLPILGWLGLSAQ(+.98)HGTIYR.N	Y	22.33	2652.3911	24	-0.1	885.1376	3	38.40	30	1988	OB4058.raw	6.94E3	1	1	359	382	
R.SSNPDIYNPQ(+.98)AGSLRSVNELDLPILGWLGLSAQHGTIYR.N	Y	21.23	4252.1553	39	1.9	1064.0481	4	37.35	30	1928	OB4058.raw	1.59E4	1	1	344	382	
**total 4 peptides**																	

tr|A0A1B3TNS1|A0A1B3TNS1_ARAHY
back to list

| Protein Coverage | Supporting Peptides |

Protein Coverage:

1 MAESPVSFLL DKLTTLLQEE VNLQRGVSDD IRHIKGELER HKAILRVADA LEDKDHELQE WIKRVREIAY EMEDAIDEFN

d Deamidation (NQ) (+0.98)

81 VRLVDQHMHG SNSSLTHKIV FTWKTLKARR QIGSHIQEIK SRLDVISMER PSMYGIGSRS SQRLSSRLDS QGDALLLEEA

161 DLVGIDTPKK QLSDLLFKDE PNRDVIAIYG MGGLGKTTLA KQVYDDPKVK KRFRIHAWVI VSQSFKLEEL LRDLVQQLYN

252 d

241 VIGKP**APEAV GQMR**SDKLKE VIKNLLQRSR YLIVLDDVWH VNVWDSVKYA LPNNSRGSRV MLTTRKRNVA MSSCAEFGKV

d

321 YNLEFLSEHE AWSLFCRKTF QGNSCPSHLE QVCWNILKLC GGLPLAIVAI SGASATRDKT NIEEWQMVCR SFGAEMEGND

401 KLEDMKKVLS LSFNELPYYL KSCLLYLSIF PEFHAIEHMR LIRLWIAEGF VVGEDGKTLE EVADSYLKEL LNRSLLQVVQ

481 KTSDGRMKTC RMHDLIREIV TLKSKNQNFA TIAKEPDITW PDKVRRLSVI NTMNNIQQNK TFQLRSLLMF ALSDANHDFS

561 LHAVCSTGYR LLRVLDLQDS PLQVFPAQVV NLYLLKFLSL KNTKVKSIPA SIKKLQHLET LDLKHSNVTE LPVEIVELQR

641 LRHLLVYRYE IESYAYFHSK YGFKVSAPIG KMQSLQKLCF IEVDQGSKAL MIELGKLTQL RRLGIRKMRR EDGAALCFSI

721 EKMINLRSLS ITAINEDEII DIHCISKPPQ YLRQLYLSGR LEKFPQWIQS LKNLAKVHLK WSRLKEDPLV YLQDLPNLRH

801 LEFLQVYVGD KLHFRADKFQ NLKVLGLDEI DGLKSMIMEE GAMPGLKKLI IQRCGALTQV PLGIEHLSKL KSIEFFDMPE

881 ELISALRPNG GKDNWRVQHV PVVYSTYWRD GGWDVYSLDT FGERETDSSA VMRSLELPTL WKV

Supporting Peptides:

Peptide	Uniq	-10lgP	Mass	Length	ppm	m/z	z	RT	Fraction	Scan	Source File	Area Digest 8. traka	#Feature	#Feature Digest 8. traka	Start	End	PTM
P.APEAVGQ(+.98)MR.S	Y	21.13	958.4542	9	6.6	480.2375	2	54.62	30	2918	OB4058.raw	3.4E4	1	1	246	254	Deamidation (NQ)
**total 1 peptides**																	

Peptide List

Prepared with PEAKS ™ (bioinfor.com)

1. Notes Gastric Control Raw peanut Band #9 29-33 kDa (PTM449)

2. Result Statistics

Figure 1. False discovery rate (FDR) curve. X axis is the number of peptide-spectrum matches (PSM) being kept. Y axis is the corresponding FDR.

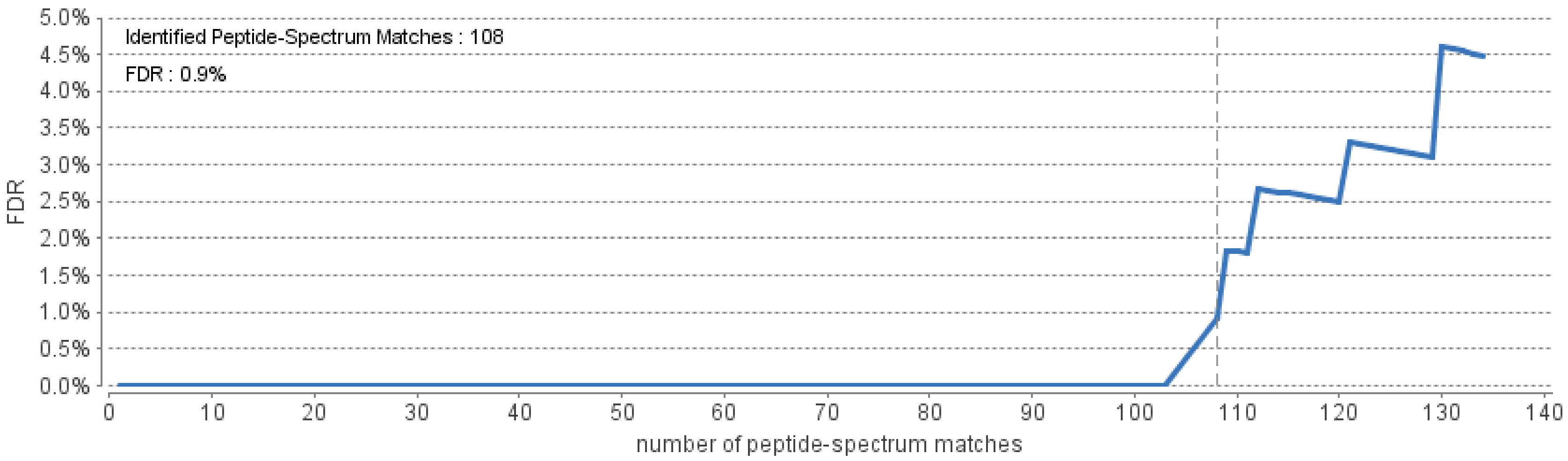

Figure 2. PSM score distribution. **(a)** Distribution of PEAKS peptide score; **(b)** Scatterplot of PEAKS peptide score versus precursor mass error.

(a)

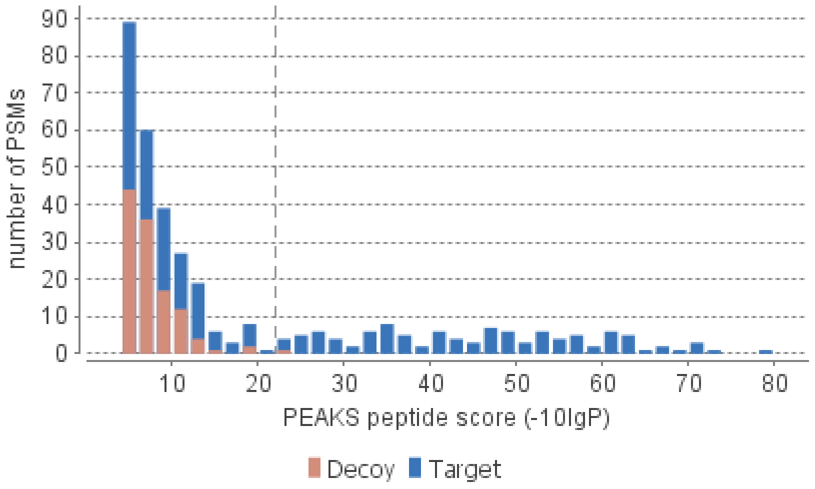

(b)

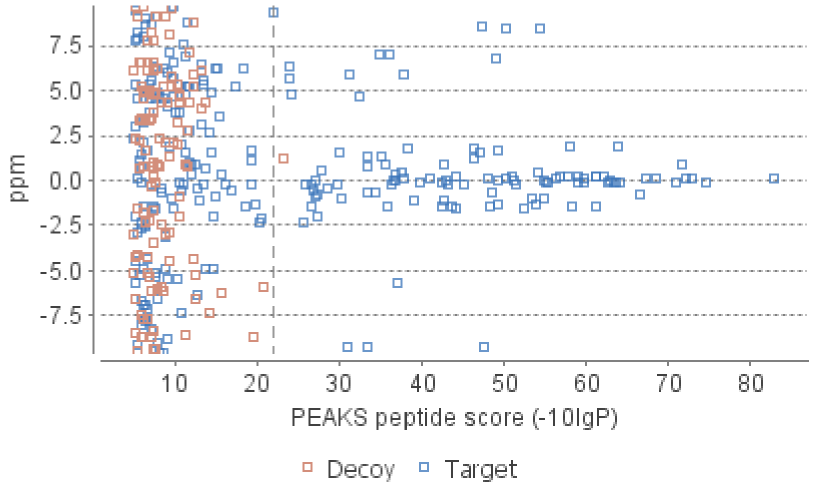

Figure 3. Distribution of peptide feature detection. **(a)** Feature m/z distribution; **(b)** Feature RT distribution

(a)

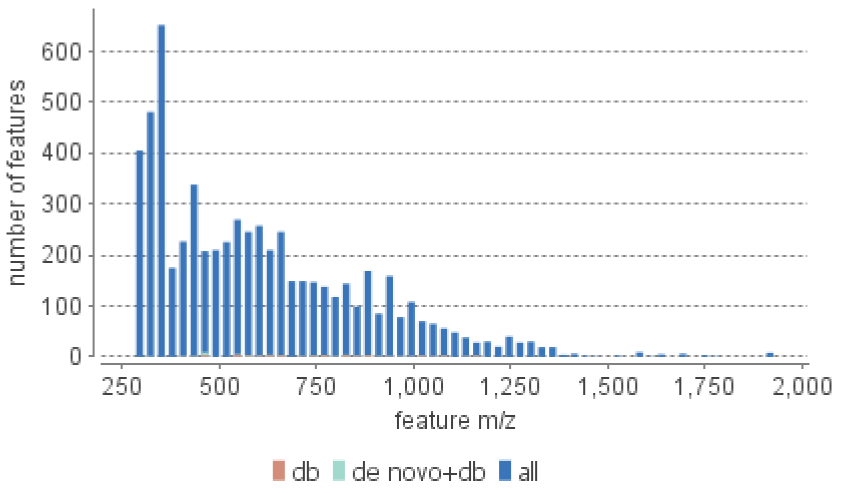

(b)

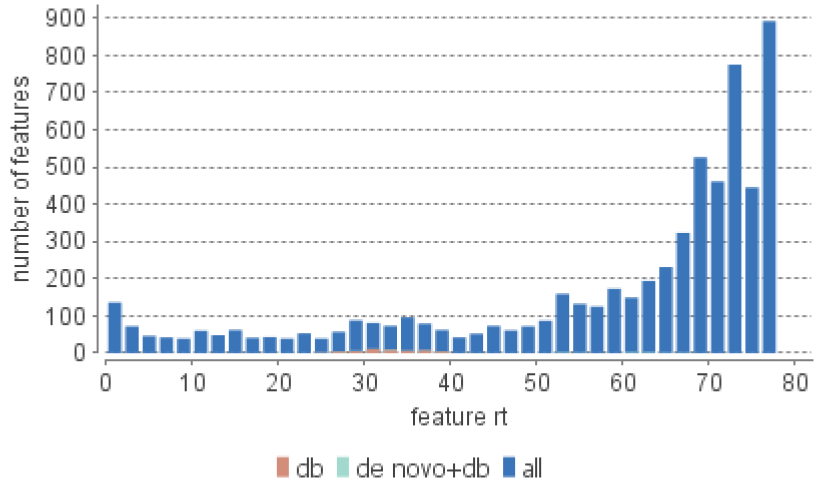

Figure 4. Distribution of identified peptide features. **(a)** Feature abundance distribution **(b)** *De novo* sequencing validation.

(a)

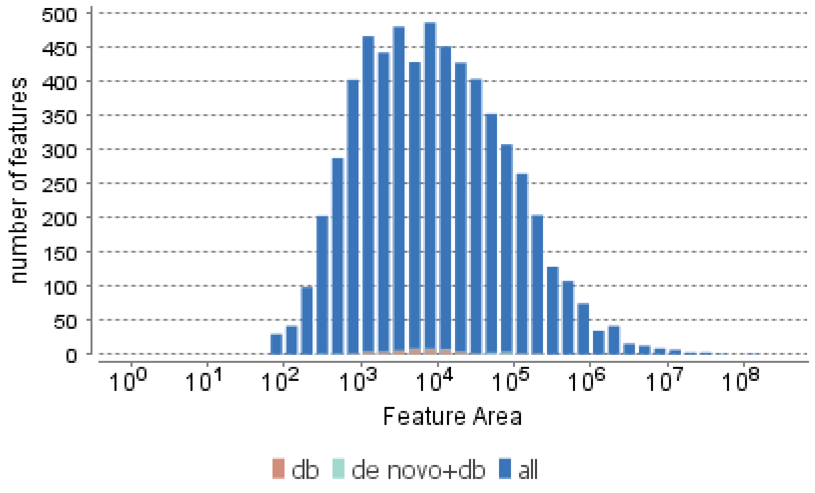

(b)

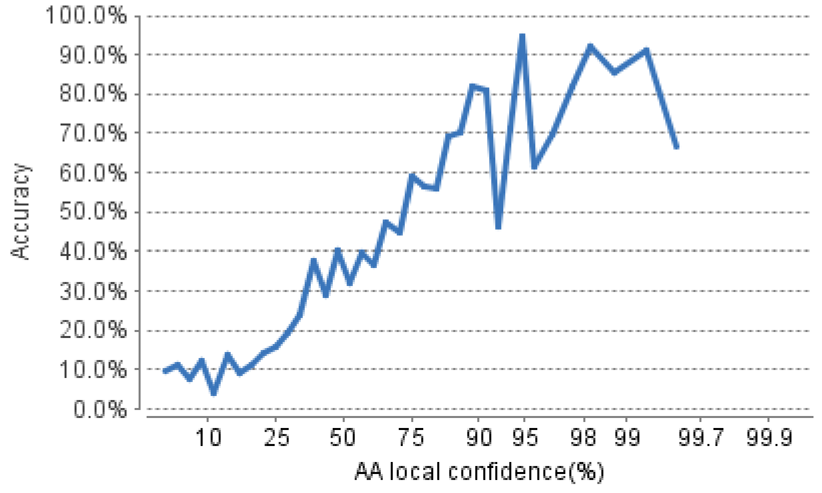

Table 1. Statistics of data.

# of MS scans	2869
# of MS/MS scans	1423
# of Features	6228
# of Chimera scans	101

Table 2. Result filtration parameters.

Peptide -10lgP	≥22

Table 4. PTM profile.

Name	ΔMass	Position	#PSM	-10lgP	Abundance	AScore
Deamidation	.98	NQ	21	62.14	1.91E4	44.59
Oxidation	15.99	M	10	71.54	4.65E3	1000.00
Carbamylation	43.01	N-term	4	47.61	6.37E3	205.29
Carboxylation	43.99	D	3	47.61	6.37E3	73.54

PTM Ascore	≥20
Protein -10lgP	≥20
Proteins unique peptides	≥1
De novo ALC Score	≥50%

Table 3. Statistics of filtered result.

Peptide-Spectrum Matches	108
Peptide sequences	55
Protein groups	5
Proteins	13
Proteins (#Unique Peptides)	2 (>2); 1 (=2); 10 (=1);
FDR (Peptide-Spectrum Matches)	0.9%
FDR (Peptide Sequences)	1.8%
FDR (Protein Group)	0.0%
De Novo Only Spectra	13
# of identified Features	57
# of identified MS/MS scans	102

HydPro	15.99	P	2	24.10		1000.00
Formylation	27.99	N-term	2	48.47	1.79E3	119.03
Acetylation	42.01	N-term	1	25.52	1.36E4	1000.00
Im_biotin	225.09	N-term	1	28.59		78.36
Oxidation	15.99	H	1	25.52	1.36E4	1000.00

3. Experiment Control

Figure 5. Precursor mass error of peptide-spectrum matches (PSM) in filtered result. **(a)** Distribution of precursor mass error in ppm; **(b)** Scatterplot of precursor m/z versus precursor mass error in ppm.

(a)

(b)

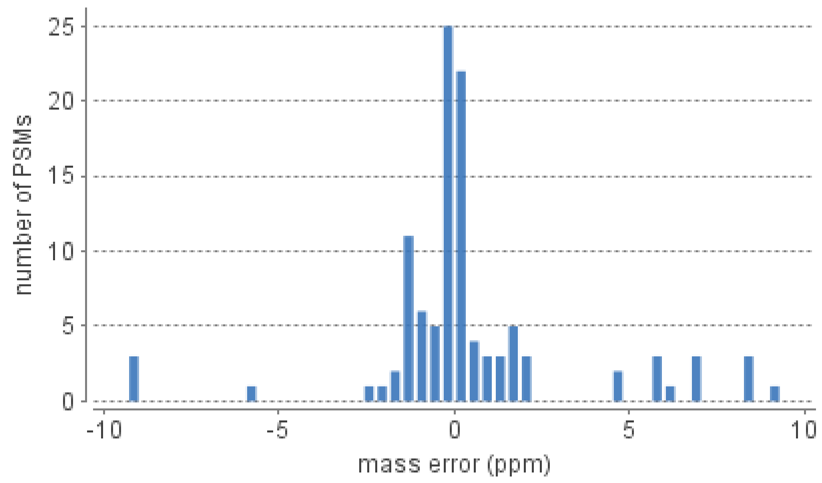

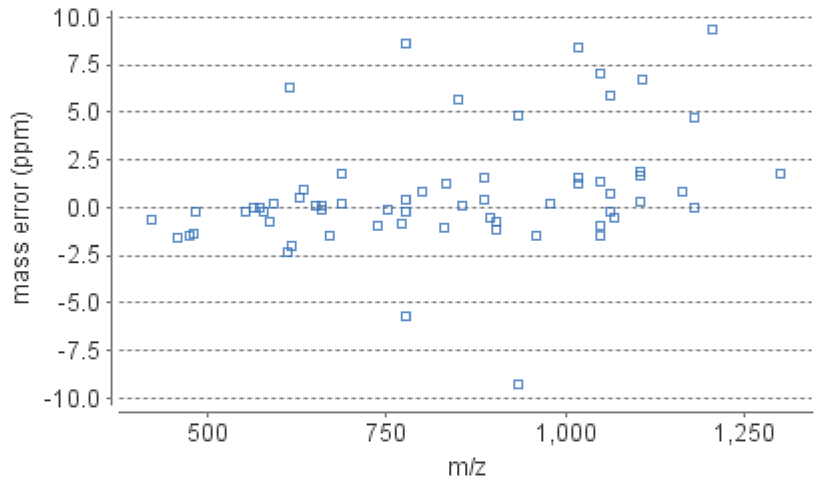

Table 5. Number of identified peptides in each sample by the number of missed cleavages

Missed Cleavages	0	1	2	3	4+
KOntrola 9. traka	35	19	1	0	0

4. Other Information

Table 6. Search parameters.

Search Engine Name: PEAKS
Parent Mass Error Tolerance: 10.0 ppm
Fragment Mass Error Tolerance: 0.5 Da
Precursor Mass Search Type: monoisotopic
Enzyme: Trypsin
Max Missed Cleavages: 2
Digest Mode: Unspecific
Fixed Modifications:
  Carbamidomethylation: 57.02
Variable Modifications:
  Deamidation (NQ): 0.98
  Oxidation (M): 15.99
  Hydroxylation Pro: 15.99
  Acetylation (K): 42.01
  Acetylation (Protein N-term): 42.01
  Acetylation (N-term): 42.01
  Amidation: -0.98
  Beta-methylthiolation: 45.99
  and 305 more...
Max Variable PTM Per Peptide: 5
Database: Uniprot_Peanut-3818_Jul18

Table 7. Instrument parameters.

Fractions: OB4038.raw
Ion Source: ESI(nano-spray)
Fragmentation Mode: CID, CAD(y and b ions)
MS Scan Mode: FT-ICR/Orbitrap
MS/MS Scan Mode: Linear Ion Trap

Taxon: All
Contaminant Database: contaminantsMQ_mar19
Searched Entry: 1723
FDR Estimation: Enabled
De novo score (ALC%) threshold: 15
Peptide hit threshold (-10logP): 30.0
Peaks run ID: 433
Merge Options: no merge
Precursor Options: corrected
Charge Options: no correction
Filter Charge: 2 - 8
Process: true
Associate chimera: yes

Protein List

Protein Accession Contains:
Protein Description Contains:
Peptide Sample Area >=
Protein Ptm Contains:

Protein Group	Protein ID	Accession	-10lgP	Coverage (%)	Coverage (%) KOntrola 9. traka	Area KOntrola 9. traka	#Peptides	#Unique	#Spec KOntrola 9. traka	PTM	Avg. Mass	Description
**1**	21035	P02872|LECG_ARAHY	215.68	70	70	5.53E5	19	18	71	Y	29325	Galactose-binding lectin OS=Arachis hypogaea OX=3818 PE=1 SV=3
**1**	21034	tr|Q38711|Q38711_ARAHY	215.68	70	70	5.53E5	19	18	71	Y	29134	Galactose-binding lectin (Fragment) OS=Arachis hypogaea OX=3818 GN=lec PE=2 SV=1
**2**	21349	tr|Q43373|Q43373_ARAHY	96.69	14	14	1.8E4	3	2	13	Y	29566	Galactose-binding lectin OS=Arachis hypogaea OX=3818 GN=lec PE=2 SV=1
**7**	20591	tr|Q6T2T4|Q6T2T4_ARAHY	87.78	4	4	0E0	2	1	2	N	61499	Storage protein OS=Arachis hypogaea OX=3818 PE=2 SV=1
**7**	20574	tr|A1DZF0|A1DZF0_ARAHY	87.78	5	5	0E0	2	1	2	N	60375	Arachin 6 OS=Arachis hypogaea OX=3818 PE=2 SV=1
**7**	20578	tr|B5TYU1|B5TYU1_ARAHY	87.78	5	5	0E0	2	1	2	N	60624	Arachin Arah3 isoform OS=Arachis hypogaea OX=3818 PE=1 SV=1
**7**	20580	tr|Q647H4|Q647H4_ARAHY	87.78	4	4	0E0	2	1	2	N	61506	Arachin Ahy-1 OS=Arachis hypogaea OX=3818 PE=2 SV=1
**8**	20576	tr|Q9FZ11|Q9FZ11_ARAHY	80.32	5	5	0E0	2	1	2	N	60449	Gly1 OS=Arachis hypogaea OX=3818 GN=Gly1 PE=2 SV=1
**8**	20581	tr|Q647H3|Q647H3_ARAHY	80.32	4	4	0E0	2	1	2	N	61532	Arachin Ahy-2 OS=Arachis hypogaea OX=3818 PE=2 SV=1
**8**	20579	tr|Q5I6T2|Q5I6T2_ARAHY	80.32	5	5	0E0	2	1	2	N	60736	Arachin Ahy-4 OS=Arachis hypogaea OX=3818 PE=2 SV=1
**10**	20593	tr|Q6IWG5|Q6IWG5_ARAHY	36.74	5	5	8.49E3	1	1	2	N	58061	Glycinin (Fragment) OS=Arachis hypogaea OX=3818 PE=2 SV=1
**10**	20595	tr|E5G077|E5G077_ARAHY	36.74	5	5	8.49E3	1	1	2	N	58305	Ara h 3 allergen OS=Arachis hypogaea OX=3818 GN=ara h 3 PE=3 SV=1
**10**	20594	tr|Q0GM57|Q0GM57_ARAHY	36.74	5	5	8.49E3	1	1	2	N	58263	Iso-Ara h3 OS=Arachis hypogaea OX=3818 PE=2 SV=1
**total 13 proteins**												

P02872|LECG_ARAHY
back to list

| Protein Coverage | Supporting Peptides |

Protein Coverage:

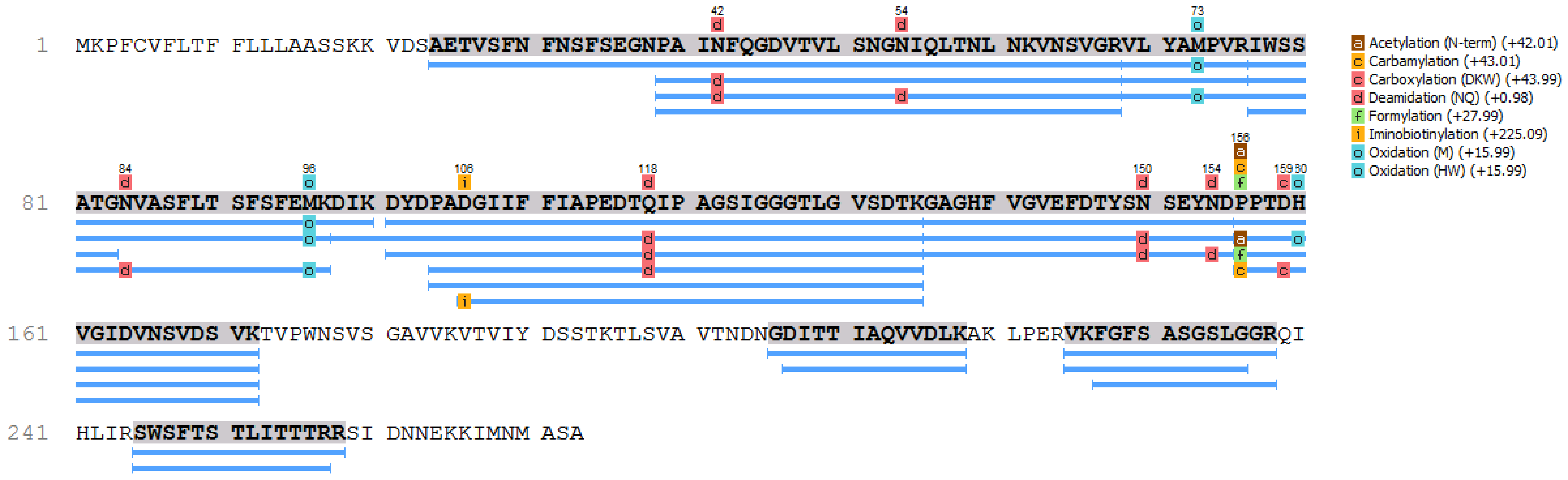

Supporting Peptides:

Peptide	Uniq	-10lgP	Mass	Length	ppm	m/z	z	RT	Fraction	Scan	Source File	Area KOntrola 9. traka	#Feature	#Feature KOntrola 9. traka	Start	End	PTM
R.SWSFTSTLITTTR.R	Y	74.60	1499.7620	13	-0.1	750.8882	2	33.33	10	1792	OB4038.raw	9.23E4	1	1	245	257	
R.IWSSATGNVASFLTSFSFEM(+15.99)K.D	Y	71.54	2325.0986	21	0.9	1163.5576	2	37.29	10	2024	OB4038.raw	1.2E4	2	2	77	97	Oxidation (M)
K.GAGHFVGVEFDTYSNSEYND.P	N	63.79	2206.9080	20	1.9	1104.4634	2	31.40	10	1672	OB4038.raw	1.95E4	2	2	136	155	
K.FGFSASGSLGGR.Q	Y	62.77	1141.5515	12	0.0	571.7830	2	29.46	10	1563	OB4038.raw	3.53E4	1	1	227	238	
K.GAGHFVGVEFDTYSN(+.98)SEYND.P	N	62.14	2207.8918	20	0.3	1104.9535	2	31.74	10	1695	OB4038.raw	1.91E4	1	1	136	155	Deamidation (NQ)
D.PPTDHVGIDVNSVDSVK.T	Y	61.14	1777.8846	17	0.2	593.6356	3	27.79	10	1455	OB4038.raw	1.74E5	1	1	156	172	
G.DITTIAQVVDLK.A	Y	59.77	1314.7395	12	-0.1	658.3770	2	34.02	10	1831	OB4038.raw	2.33E4	1	1	207	218	
D.PADGIIFFIAPEDTQ(+.98)IPAGSIGGGTLGVSDTK.G	Y	54.71	3144.5867	32	-1.0	1049.2018	3	37.06	10	2007	OB4038.raw	9.41E3	2	2	104	135	Deamidation (NQ)
R.VKFGFSASGSLGGR.Q	Y	52.29	1368.7150	14	-1.6	457.2449	3	28.45	10	1494	OB4038.raw	7.07E3	1	1	225	238	
R.IWSSATGN(+.98)VASFLTSFSFEM(+15.99)K.D	Y	51.35	2326.0828	21	-0.2	776.3680	3	37.87	10	2058	OB4038.raw	9.8E2	1	1	77	97	Deamidation (NQ); Oxidation (M)
N.GDITTIAQVVDLK.A	Y	50.11	1371.7609	13	0.2	686.8878	2	33.13	10	1784	OB4038.raw	4.96E3	2	2	206	218	
K.GAGHFVGVEFDTYSN(+.98)SEYN(+.98)D.P	N	48.99	2208.8760	20	6.8	1105.4528	2	31.98	10	1710	OB4038.raw	0	0	0	136	155	Deamidation (NQ)
D.P(+27.99)PTDHVGIDVNSVDSVK.T	Y	48.47	1805.8795	17	-0.7	903.9464	2	30.08	10	1595	OB4038.raw	1.79E3	1	1	156	172	Formylation
R.VLYAMPVR.I	Y	48.33	947.5262	8	-1.4	474.7697	2	28.89	10	1518	OB4038.raw	4.21E3	1	1	69	76	

Peptide	Uniq	-10lgP	Mass	Length	ppm	m/z	z	RT	Fraction	Scan	Source File	Area KOntrola 9. traka	#Feature	#Feature KOntrola 9. traka	Start	End	PTM
D.P(+43.01)PTD(+43.99)HVGIDVNSVDSVK.T	Y	47.61	1864.8802	17	-9.3	933.4387	2	30.81	10	1635	OB4038.raw	6.37E3	1	1	156	172	Carbamylation; Carboxylation (DKW)
K.DIKDYDPADGIIFFIAPEDTQ(+.98)IPAGSIGGGTLGVSDTK.G	Y	46.28	3893.9097	38	1.8	1298.9795	3	37.74	10	2050	OB4038.raw	0	0	0	98	135	Deamidation (NQ)
R.VLYAM(+15.99)PVR.I	Y	45.20	963.5212	8	-0.2	482.7678	2	25.68	10	1351	OB4038.raw	1.05E4	1	1	69	76	Oxidation (M)
K.DYDPADGIIFFIAPEDTQ(+.98)IPAGSIGGGTLGVSDTK.G	Y	42.82	3537.7039	35	0.0	1180.2419	3	38.96	10	2122	OB4038.raw	3.39E3	1	1	101	135	Deamidation (NQ)
N.PAIN(+.98)FQ(+.98)GDVTVLSNGN(+.98)IQLTNLN(+.98)KVNSVGR.V	Y	37.70	3185.6204	30	5.9	1062.8870	3	34.24	10	1847	OB4038.raw	2.9E3	1	1	39	68	Deamidation (NQ)
N.PAINFQ(+.98)GDVTVLSN(+.98)GNIQLTNLN(+.98)KVNSVGR.V	Y	36.41	3184.6365	30	-0.3	1062.5525	3	34.25	10	1850	OB4038.raw	0	0	0	39	68	
D.PADGIIFFIAPEDTQIPAGSIGGGTLGVSDTK.G	Y	35.84	3143.6025	32	-1.4	1048.8733	3	36.89	10	1998	OB4038.raw	0	0	0	104	135	
R.VLYAM(+15.99)PVRIWSSATG.N	Y	35.19	1665.8549	15	1.3	833.9358	2	32.08	10	1717	OB4038.raw	2.76E3	1	1	69	83	Oxidation (M)
N.PAIN(+.98)FQ(+.98)GDVTVLSNGNIQLTNLN(+.98)KVNSVGR.V	Y	33.34	3184.6365	30	0.8	1062.5536	3	34.21	10	1847	OB4038.raw	0	0	0	39	68	Deamidation (NQ)
K.DYDPADGIIFFIAPEDTQIPAGSIGGGTLGVSDTK.G	Y	32.39	3536.7197	35	4.7	1179.9194	3	38.69	10	2111	OB4038.raw	1.36E3	1	1	101	135	
N.PAINFQ(+.98)GDVTVLSN(+.98)GNIQ(+.98)LTNLN(+.98)KVNSVGR.V	Y	31.14	3185.6204	30	5.9	1062.8870	3	34.24	10	1850	OB4038.raw	2.9E3	1	1	39	68	
R.SWSFTSTLITTTRR.S	Y	30.27	1655.8632	14	-1.0	828.9380	2	31.63	10	1685	OB4038.raw	1.35E5	2	2	245	258	
R.VKFGFSASGSLG.G	Y	29.70	1155.5924	12	-0.2	578.8033	2	30.52	10	1621	OB4038.raw	6.59E3	1	1	225	236	
A.D(+225.09)GIIFFIAPEDTQIPAGSIGGGTLGVSDTK.G	Y	28.59	3200.6062	30	-0.5	1067.8755	3	37.21	10	2018	OB4038.raw	0	0	0	106	135	Iminobiotinylation
R.IWSSATGNVASFLTSFSFEM(+15.99)KDIK.D	Y	26.79	2681.3047	24	-0.5	894.7750	3	35.93	10	1944	OB4038.raw	2.34E3	1	1	77	100	Oxidation (M)
D.P(+42.01)PTDH(+15.99)VGIDVNSVDSVK.T	Y	25.52	1835.8901	17	-2.3	612.9692	3	28.10	10	1474	OB4038.raw	1.36E4	1	1	156	172	Acetylation (N-term); Oxidation (HW)
D.P(+43.01)(+15.99)PTDHVGIDVNSVDSVK.T	Y	24.03	1836.8854	17	6.3	613.3063	3	29.00	10	1527	OB4038.raw	0	0	0	156	172	
S.AETVSFNFNSFSEGN(+.98)PAIN(+.98)FQ(+.98)GDVTVLSN(+.98)GNIQLTNLN(+.98)KVNSVGR.V	Y	22.01	4817.2944	45	9.4	1205.3422	4	36.77	10	1991	OB4038.raw	0	0	0	24	68	
**total 32 peptides**																	

tr|Q38711|Q38711_ARAHY

back to list

| Protein Coverage | Supporting Peptides |

Protein Coverage:

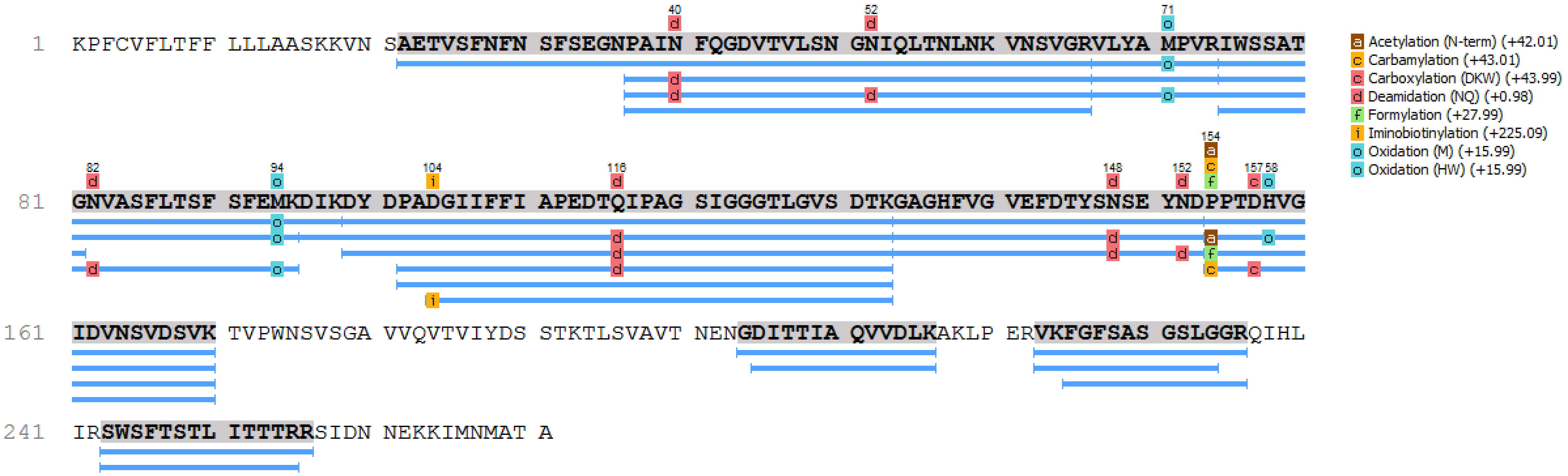

Supporting Peptides:

Peptide	Uniq	-10lgP	Mass	Length	ppm	m/z	z	RT	Fraction	Scan	Source File	Area KOntrola 9. traka	#Feature	#Feature KOntrola 9. traka	Start	End	PTM
R.SWSFTSTLITTTR.R	Y	74.60	1499.7620	13	-0.1	750.8882	2	33.33	10	1792	OB4038.raw	9.23E4	1	1	243	255	
R.IWSSATGNVASFLTSFSFEM(+15.99)K.D	Y	71.54	2325.0986	21	0.9	1163.5576	2	37.29	10	2024	OB4038.raw	1.2E4	2	2	75	95	Oxidation (M)
K.GAGHFVGVEFDTYSNSEYND.P	N	63.79	2206.9080	20	1.9	1104.4634	2	31.40	10	1672	OB4038.raw	1.95E4	2	2	134	153	
K.FGFSASGSLGGR.Q	Y	62.77	1141.5515	12	0.0	571.7830	2	29.46	10	1563	OB4038.raw	3.53E4	1	1	225	236	
K.GAGHFVGVEFDTYSN(+.98)SEYND.P	N	62.14	2207.8918	20	0.3	1104.9535	2	31.74	10	1695	OB4038.raw	1.91E4	1	1	134	153	Deamidation (NQ)
D.PPTDHVGIDVNSVDSVK.T	Y	61.14	1777.8846	17	0.2	593.6356	3	27.79	10	1455	OB4038.raw	1.74E5	1	1	154	170	
G.DITTIAQVVDLK.A	Y	59.77	1314.7395	12	-0.1	658.3770	2	34.02	10	1831	OB4038.raw	2.33E4	1	1	205	216	
D.PADGIIFFIAPEDTQ(+.98)IPAGSIGGGTLGVSDTK.G	Y	54.71	3144.5867	32	-1.0	1049.2018	3	37.06	10	2007	OB4038.raw	9.41E3	2	2	102	133	Deamidation (NQ)
R.VKFGFSASGSLGGR.Q	Y	52.29	1368.7150	14	-1.6	457.2449	3	28.45	10	1494	OB4038.raw	7.07E3	1	1	223	236	
R.IWSSATGN(+.98)VASFLTSFSFEM(+15.99)K.D	Y	51.35	2326.0828	21	-0.2	776.3680	3	37.87	10	2058	OB4038.raw	9.8E2	1	1	75	95	Deamidation (NQ); Oxidation (M)
N.GDITTIAQVVDLK.A	Y	50.11	1371.7609	13	0.2	686.8878	2	33.13	10	1784	OB4038.raw	4.96E3	2	2	204	216	
K.GAGHFVGVEFDTYSN(+.98)SEYN(+.98)D.P	N	48.99	2208.8760	20	6.8	1105.4528	2	31.98	10	1710	OB4038.raw	0	0	0	134	153	Deamidation (NQ)
D.P(+27.99)PTDHVGIDVNSVDSVK.T	Y	48.47	1805.8795	17	-0.7	903.9464	2	30.08	10	1595	OB4038.raw	1.79E3	1	1	154	170	Formylation
R.VLYAMPVR.I	Y	48.33	947.5262	8	-1.4	474.7697	2	28.89	10	1518	OB4038.raw	4.21E3	1	1	67	74	
D.P(+43.01)PTD(+43.99)HVGIDVNSVDSVK.T	Y	47.61	1864.8802	17	-9.3	933.4387	2	30.81	10	1635	OB4038.raw	6.37E3	1	1	154	170	Carbamylation; Carboxylation (DKW)
K.DIKDYDPADGIIFFIAPEDTQ(+.98)IPAGSIGGGTLGVSDTK.G	Y	46.28	3893.9097	38	1.8	1298.9795	3	37.74	10	2050	OB4038.raw	0	0	0	96	133	Deamidation (NQ)
R.VLYAM(+15.99)PVR.I	Y	45.20	963.5212	8	-0.2	482.7678	2	25.68	10	1351	OB4038.raw	1.05E4	1	1	67	74	Oxidation (M)

Peptide	Uniq	-10lgP	Mass	Length	ppm	m/z	z	RT	Fraction	Scan	Source File	Area KOntrola 9. traka	#Feature	#Feature KOntrola 9. traka	Start	End	PTM
K.DYDPADGIIFFIAPEDTQ(+.98)IPAGSIGGGTLGVSDTK.G	Y	42.82	3537.7039	35	0.0	1180.2419	3	38.96	10	2122	OB4038.raw	3.39E3	1	1	99	133	Deamidation (NQ)
N.PAIN(+.98)FQ(+.98)GDVTVLSNGN(+.98)IQLTNLN(+.98)KVNSVGR.V	Y	37.70	3185.6204	30	5.9	1062.8870	3	34.24	10	1847	OB4038.raw	2.9E3	1	1	37	66	Deamidation (NQ)
N.PAINFQ(+.98)GDVTVLSN(+.98)GNIQLTNLN(+.98)KVNSVGR.V	Y	36.41	3184.6365	30	-0.3	1062.5525	3	34.25	10	1850	OB4038.raw	0	0	0	37	66	
D.PADGIIFFIAPEDTQIPAGSIGGGTLGVSDTK.G	Y	35.84	3143.6025	32	-1.4	1048.8733	3	36.89	10	1998	OB4038.raw	0	0	0	102	133	
R.VLYAM(+15.99)PVRIWSSATG.N	Y	35.19	1665.8549	15	1.3	833.9358	2	32.08	10	1717	OB4038.raw	2.76E3	1	1	67	81	Oxidation (M)
N.PAIN(+.98)FQ(+.98)GDVTVLSNGNIQLTNLN(+.98)KVNSVGR.V	Y	33.34	3184.6365	30	0.8	1062.5536	3	34.21	10	1847	OB4038.raw	0	0	0	37	66	Deamidation (NQ)
K.DYDPADGIIFFIAPEDTQIPAGSIGGGTLGVSDTK.G	Y	32.39	3536.7197	35	4.7	1179.9194	3	38.69	10	2111	OB4038.raw	1.36E3	1	1	99	133	
N.PAINFQ(+.98)GDVTVLSN(+.98)GNIQ(+.98)LTNLN(+.98)KVNSVGR.V	Y	31.14	3185.6204	30	5.9	1062.8870	3	34.24	10	1850	OB4038.raw	2.9E3	1	1	37	66	
R.SWSFTSTLITTTRR.S	Y	30.27	1655.8632	14	-1.0	828.9380	2	31.63	10	1685	OB4038.raw	1.35E5	2	2	243	256	
R.VKFGFSASGSLG.G	Y	29.70	1155.5924	12	-0.2	578.8033	2	30.52	10	1621	OB4038.raw	6.59E3	1	1	223	234	
A.D(+225.09)GIIFFIAPEDTQIPAGSIGGGTLGVSDTK.G	Y	28.59	3200.6062	30	-0.5	1067.8755	3	37.21	10	2018	OB4038.raw	0	0	0	104	133	Iminobiotinylation
R.IWSSATGNVASFLTSFSFEM(+15.99)KDIK.D	Y	26.79	2681.3047	24	-0.5	894.7750	3	35.93	10	1944	OB4038.raw	2.34E3	1	1	75	98	Oxidation (M)
D.P(+42.01)PTDH(+15.99)VGIDVNSVDSVK.T	Y	25.52	1835.8901	17	-2.3	612.9692	3	28.10	10	1474	OB4038.raw	1.36E4	1	1	154	170	Acetylation (N-term); Oxidation (HW)
D.P(+43.01)(+15.99)PTDHVGIDVNSVDSVK.T	Y	24.03	1836.8854	17	6.3	613.3063	3	29.00	10	1527	OB4038.raw	0	0	0	154	170	
S.AETVSFNFNSFSEGN(+.98)PAIN(+.98)FQ(+.98)GDVTVLSN(+.98)GNIQLTNLN(+.98)KVNSVGR.V	Y	22.01	4817.2944	45	9.4	1205.3422	4	36.77	10	1991	OB4038.raw	0	0	0	22	66	
**total 32 peptides**																	

tr|Q43373|Q43373_ARAHY

back to list

| Protein Coverage | Supporting Peptides |

Protein Coverage:

d Deamidation (NQ) (+0.98)

1 MKPFCVLLTF FLLLAASSKK VNSAKTETVS FNYNSFNQGN PAISFQGDVT VLSDGNLLLT NLNKSNSVGR VLYATPVRIW

152 d 156 d

81 SSATGNVASF VTSFSFEMKD YNDYDPADGI IFFISPEDTQ IPAGSIGGGT LGVSDTK**GAG HFVGVEFDTY SNSEYND**PPT

d

d d

161 HHVGIDVNSV KSLKTVPWNS VSGALVKVTV IYDSSSKTLS VAVTNENG**DI TTIAEVVDLK AKLPER**VKFG FSASGSAGGR

241 QIHLIRSWSF TSTLITTTTT TSINNNEKKI MNILTA

Supporting Peptides:

Peptide	Uniq	-10lgP	Mass	Length	ppm	m/z	z	RT	Fraction	Scan	Source File	Area KOntrola 9. traka	#Feature	#Feature KOntrola 9. traka	Start	End	PTM
K.GAGHFVGVEFDTYSNSEYND.P	N	63.79	2206.9080	20	1.9	1104.4634	2	31.40	10	1672	OB4038.raw	1.95E4	2	2	138	157	
K.GAGHFVGVEFDTYSN(+.98)SEYND.P	N	62.14	2207.8918	20	0.3	1104.9535	2	31.74	10	1695	OB4038.raw	1.91E4	1	1	138	157	Deamidation (NQ)
K.GAGHFVGVEFDTYSN(+.98)SEYN(+.98)D.P	N	48.99	2208.8760	20	6.8	1105.4528	2	31.98	10	1710	OB4038.raw	0	0	0	138	157	Deamidation (NQ)
G.DITTIAEVVDLK.A	Y	48.03	1315.7235	12	0.1	658.8691	2	34.55	10	1863	OB4038.raw	1.24E4	1	1	209	220	
G.DITTIAEVVDLKAKLPER.V	Y	26.64	2010.1360	18	-1.4	671.0516	3	32.77	10	1757	OB4038.raw	5.56E3	1	1	209	226	
**total 5 peptides**																	

tr|Q6T2T4|Q6T2T4_ARAHY

back to list

| Protein Coverage | Supporting Peptides |

Protein Coverage:

1 MGKLLALSVC FCFLVLGASS ISFRQQPEEN ACQFQRLNAQ RPDNRIESEG GYIETWNPNN QEFECAGVAL SRLVLRRNAL

81 RRPFYSNAPQ EIFIQQGRAY FGLIFLGCPS TYEEPAQQGR RHQSQRPPRR FQGQDQSQQQ QDSHQKVHRF DEGDLIAVPT

161 GVAFWMYNDH DTDVVAVSLT DTNNNDNQLD QFPRRFNLAG NHEQEFLRYQ QQSRRRSLPY SPYSPQTQPK QEDREFSPRG

241 QHGRRERAGQ EQENEGGNIF SGFTPEFLAQ AFQVDDRQIL QNLRGENESD EQGAIVTVRG GLRILSPDRK KRQQYERPDE

321 EEEYDEDEYE YDEEERQQDR RRGRGSRGSG NGIEETICTA SFKKNIGRNR SPDIYNPQAG SLK**TANELNL LILRWLGLSA**

401 **EYGNLYR**NAL FVPHYNTNAH SIIYALRGRA HVQVVDSNGD RVFDEELQEG HVLVVPQNFA VAGKSQSENF EYVAFKTDSR

481 PSIANLAGEN SFIDNLPEEV VANSYGLPRE QARQLKNNNP FKFFVPPSEQ SLRAVA

Supporting Peptides:

Peptide	Uniq	-10lgP	Mass	Length	ppm	m/z	z	RT	Fraction	Scan	Source File	Area KOntrola 9. traka	#Feature	#Feature KOntrola 9. traka	Start	End	PTM
R.WLGLSAEYGNLYR.N	N	66.47	1540.7673	13	-0.8	771.3903	2	33.52	10	1804	OB4038.raw	1.21E4	1	1	395	407	
K.TANELNLLILR.W	Y	42.62	1268.7452	11	0.9	635.3805	2	33.93	10	1829	OB4038.raw	0	0	0	384	394	
**total 2 peptides**																	

tr|A1DZF0|A1DZF0_ARAHY

back to list

| Protein Coverage | Supporting Peptides |

Protein Coverage:

1 MAKLLELSFC FCFLVLGASS ISFRQQPEEN ACQFQRLNAQ RPDNRIESEG GYIETWNPNN QEFECAGVAL SRLVLRRNAL

81 RRPFYSNAPQ EIFIQQGRGY FGLIFPGCPS TYEEPAQQGR RYQSQRPPRR LQEEDQSQQQ QDSHQKVHRF NEGDLIAVPT

161 GVAFWLYNDH DTDVVAVSLT DTNNNDNQLD QFPRRFNLAG NHEQEFLRYQ QQSRQSRRRS LPLSPYSPQP GQEDREFSPQ

241 GQHGRRERAG QEQENEGGNI FSGFTSEFLA QAFQVDDRQI VQNLRGENES EEQGAIVTVK GGLRILSPDR KSPDEEEEYD

321 EDEYAEEERQ QDRRRGRGSR GSGNGIEETI CTATVKKNIG RNRSPDIYNP QAGSLK**TANE LNLLILRWLG LSAEYGNLYR**

401 NALFVPHYNT NAHSIIYALR GRAHVQVVDS NGNRVYDEEL QEGHVLVVPQ NFAVAGKSQS ENFEYVAFKT DSRPSIANLA

481 GENSFIDNLP EEVVANSYGL PREQARQLKN NNPFKFFVPP FQQSPRAVA

Supporting Peptides:

Peptide	Uniq	-10lgP	Mass	Length	ppm	m/z	z	RT	Fraction	Scan	Source File	Area KOntrola 9. traka	#Feature	#Feature KOntrola 9. traka	Start	End	PTM
R.WLGLSAEYGNLYR.N	N	66.47	1540.7673	13	-0.8	771.3903	2	33.52	10	1804	OB4038.raw	1.21E4	1	1	388	400	
K.TANELNLLILR.W	Y	42.62	1268.7452	11	0.9	635.3805	2	33.93	10	1829	OB4038.raw	0	0	0	377	387	
**total 2 peptides**																	

tr|B5TYU1|B5TYU1_ARAHY

back to list

| Protein Coverage | Supporting Peptides |

Protein Coverage:

1 MAKLLELSFC FCFLVLGASS ISFRQQPEEN ACQFQRLNAQ RPDNRIESEG GYIETWNPNN QEFECAGVAL SRLVLRRNAL
81 RRPFYSNAPQ EIFIQQGRGY FGLIFPGCPS TYEEPAQQGR RYQSQRPPRR LQEEDQSQQQ QDSHQKVHRF NEGDLIAVPT
161 GVAFWLYNDH DTDVVAVSLT DTNNNDNQLD QFPRRFNLAG NHEQEFLRYQ QQSRQSRRRS LPYSPYSPQS QPRQEEREFS
241 PRGQHSRRER AGQEEENEGG NIFSGFTPEF LAQAFQVDDR QIVQNLRGEN ESEEQGAIVT VRGGLRILSP DRKRGADEEE
321 EYDEDEYEYD EEDRRRGRGS RGSGNGIEET ICTATVKKNI GRNRSPDIYN PQAGSLK**TAN ELNLLILRWL GLSAEYGNLY**
401 **R**NALFVPHYN TNAHSIIYAL RGRAHVQVVD SNGNRVYDEE LQEGHVLVVP QNFAVAGKSQ SDNFEYVAFK TDSRPSIANL
481 AGENSVIDNL PEEVVANSYG LPREQARQLK NNNPFKFFVP PSQQSPRAVA

Supporting Peptides:

Peptide	Uniq	-10lgP	Mass	Length	ppm	m/z	z	RT	Fraction	Scan	Source File	Area KOntrola 9. traka	#Feature	#Feature KOntrola 9. traka	Start	End	PTM
R.WLGLSAEYGNLYR.N	N	66.47	1540.7673	13	-0.8	771.3903	2	33.52	10	1804	OB4038.raw	1.21E4	1	1	389	401	
K.TANELNLLILR.W	Y	42.62	1268.7452	11	0.9	635.3805	2	33.93	10	1829	OB4038.raw	0	0	0	378	388	
**total 2 peptides**																	

tr|Q647H4|Q647H4_ARAHY

back to list

| Protein Coverage | Supporting Peptides |

Protein Coverage:

1 MGKLLALSVC FCFLVLGASS ISFRQQPEEN ACQFQRLNAQ RPDNRIESEG GYIETWNPNN QEFECAGVAL SRLVLRRNAL
81 RRPFYSNAPQ EIFIQQGRGY FGLIFPGCPS TYEEPAQQGR RHQSQRPPRR FQGQDQSQQQ QDSHQKVHRF DEGDLIAVPT
161 GVAFWMYNDH DTDVVAVSLT DTNNNDNQLD QFPRRFNLAG NHEQEFLRYQ QQSRRRSLPY SPYSPQTQPK QEDREFSPRG
241 QHGRRERAGQ EQENEGGNIF SGFTPEFLAQ AFQVDDRQIL QNLRGENESD EQGAIVTVRG GLRILSPDRK RRQQYERPDE
321 EEEYDEDEYE YDEEERQHDR RRGRGSRGSG NGIEETICTA SFKKNIGRNR SPDIYNPQAG SLK**TANELNL LILRWLGLSA**
401 **EYGNLYR**NAL FVPHYNTNAH SIIYALRGRA HVQVVDSNGD RVFDEELQEG HVLVVPQNFA VAGKSQSENF EYVAFKTDSR
481 PSIANLAGEN SFIDNLPEEV VANSYGLPRE QARQLKNNNP FKFFVPPSEQ SLRAVA

Supporting Peptides:

Peptide	Uniq	-10lgP	Mass	Length	ppm	m/z	z	RT	Fraction	Scan	Source File	Area KOntrola 9. traka	#Feature	#Feature KOntrola 9. traka	Start	End	PTM
R.WLGLSAEYGNLYR.N	N	66.47	1540.7673	13	-0.8	771.3903	2	33.52	10	1804	OB4038.raw	1.21E4	1	1	395	407	
K.TANELNLLILR.W	Y	42.62	1268.7452	11	0.9	635.3805	2	33.93	10	1829	OB4038.raw	0	0	0	384	394	
**total 2 peptides**																	

tr|Q9FZ11|Q9FZ11_ARAHY

back to list

| Protein Coverage | Supporting Peptides |

Protein Coverage:

1 MIRGRLALSV CFCFLVLGAS SISFRQQPEE NACQFQRLNA QRPDNRLESE GGYIETWNPN NQEFECAGVA LSRLVLRRNA
81 LRRPFYSNAP QEIFIQQGRG YFGLIFPGCP STYEEPAQQG RRHQSQRAPR RFEGEDQSQQ QQQDSHQKVR RFDEGDLIAV
161 PTGVALWMFN DHDTDVVAVS LTDTNNNDNQ LDQFPRRFNL AGNHEQEFLR YQQQSRRRSL PYSPYSPQSQ PRQEEREFSP
241 RGQHSRRERA GQEEENEGGN IFSGFTPEFL AQAFQVDDRQ IVQNLRGENE SEEEGAIVTV KGGLRILSPD RKRGADEEEE
321 YDEDEYEYDE EDRRRGRGSR GRGNGIEETI CTASVKKNIG RNRSPDIYNP QAGSLK**TAND LNLLILRWLG LSAEYGNLYR**
401 NALFVPHYNT NAHSIIYALR GRAHVQVVDS NGNRVYDEEL QEGHVLVVPQ NFAVAGKSQS DNFEYVAFKT DSRPNIANFA
481 GENSIIDNLP EEVVANSYGL PREQARQLKN NNPFKFFVPP SQQSLRAVA

Supporting Peptides:

Peptide	Uniq	-10lgP	Mass	Length	ppm	m/z	z	RT	Fraction	Scan	Source File	Area KOntrola 9. traka	#Feature	#Feature KOntrola 9. traka	Start	End	PTM
R.WLGLSAEYGNLYR.N	N	66.47	1540.7673	13	-0.8	771.3903	2	33.52	10	1804	OB4038.raw	1.21E4	1	1	388	400	
K.TANDLNLLILR.W	Y	27.70	1254.7296	11	0.6	628.3724	2	33.63	10	1811	OB4038.raw	0	0	0	377	387	
**total 2 peptides**																	

tr|Q647H3|Q647H3_ARAHY

back to list

| Protein Coverage | Supporting Peptides |

Protein Coverage:

1 MAKLLALSVC FCFLVLGASS ISFRQQPEEN ACQFQRLNAQ RPDNRLESEG GYIETWNPNN QEFECAGVAL SRLVLRRNAL
81 RRPFYSNAPQ EIFIQQGRGY FGLIFPGCPS TYEEPAQQGR RHQSQRAPRR FEGEDQSQQQ QQDSHQKVRR FDEGDLIAVP
161 TGVALWMYND HDTDVVAVSL TDTNNNDNQL DQFPRRFNLA GNHEQEFLRY QQQSRRRSLP YSPYSPQSQP RQEEREFSPR
241 GQHSRRERAG QEQENEGGNI FSGFTPEFLA QAFQVDDRQI LQNLRGENES DEQGAIVTVR GGLRILSPDR KRRQQYERPD
321 EEEEYDEDEY EYDEEERQQD RRRGRGSRGR GNGIEETICT ASVKKNIGRN RSPDIYNPQA GSLK**TANDLN LLILRWLGLS**
401 **AEYGNLYR**NA LFVPHYNTNA HSIIYALRGR AHVQVVDSNG NRVYDEELQE GHVLVVPQNF AVAGKSQSDN FEYVAFKTDS
481 RPSIANLAGE NSIIDNLPEE VVANSYGLPR EQARQLKNNN PFKFFVPPSQ QSLGAVA

Supporting Peptides:

Peptide	Uniq	-10lgP	Mass	Length	ppm	m/z	z	RT	Fraction	Scan	Source File	Area KOntrola 9. traka	#Feature	#Feature KOntrola 9. traka	Start	End	PTM
R.WLGLSAEYGNLYR.N	N	66.47	1540.7673	13	-0.8	771.3903	2	33.52	10	1804	OB4038.raw	1.21E4	1	1	396	408	
K.TANDLNLLILR.W	Y	27.70	1254.7296	11	0.6	628.3724	2	33.63	10	1811	OB4038.raw	0	0	0	385	395	
**total 2 peptides**																	

tr|Q5I6T2|Q5I6T2_ARAHY

back to list

| Protein Coverage | Supporting Peptides |

Protein Coverage:

1 MAKLLELSFC FCFLVLGASS ISFRQQPEEN ACQFQRLNAQ RPDNRIESEG GYIETWNPNN QEFECAGVAL SRLVLRRNAL

81 RRPFYSNAPQ EIFIQQGRGY FGLIFPGCPS TYEEPAQQGR RSQSQRPPRR LQGEDQSQQQ QDSHQKVHRF DEGDLIAVPT

161 GVAFWLYNDH DTDVVAVSLT DTNNNDNQLD QFPRRFNLAG NHEQEFLRYQ QQSRQSRRRS LPYSPYSPQS QPRQEEREFS

241 PRGQHSRRER AGQEEENEGG NIFSGFTPEF LEQAFQVDDR QIVQNLRGEN ESEEEGAIVT VRGGLRILSP DRKRGADEEE

321 EYDEDEYEYD EEDRRRGRGS RGRGNGIEET ICTASVKKNI GRNRSPDIYN PQAGSLK**TAN DLNLLILRWL GLSAEYGNLY**

401 **R**NALFVPHYN TNAHSIIYAL RGRAHVQVVD SNGNRVYDEE LQEGHVLVVP QNFAVAGKSQ SDNFEYVAFK TDSRPSIANL

481 AGENSVIDNL PEEVVANSYG LQREQARQQL KNNNPFKFFV PPSQQSPRAV A

Supporting Peptides:

Peptide	Uniq	-10lgP	Mass	Length	ppm	m/z	z	RT	Fraction	Scan	Source File	Area KOntrola 9. traka	#Feature	#Feature KOntrola 9. traka	Start	End	PTM
R.WLGLSAEYGNLYR.N	N	66.47	1540.7673	13	-0.8	771.3903	2	33.52	10	1804	OB4038.raw	1.21E4	1	1	389	401	
K.TANDLNLLILR.W	Y	27.70	1254.7296	11	0.6	628.3724	2	33.63	10	1811	OB4038.raw	0	0	0	378	388	
**total 2 peptides**																	

tr|Q6IWG5|Q6IWG5_ARAHY

back to list

| Protein Coverage | Supporting Peptides |

Protein Coverage:

1 KLLALSLCFC VLVLGASSVT FRQGGEENEC QFQRLNAQRP DNRIESEGGY IETWNPNNQE FQCAGVALSR TVLRRNALRR

81 PFYSNAPLEI YVQQGSGYFG LIFPGCPSTY EEPAQEGRRY QSQKPSRRFQ VGQDDPSQQQ QDSHQKVHRF DEGDLIAVPT

161 GVAFWMYNDE DTDVVTVTLS DTSSIHNQLD QFPRRFYLAG NQEQEFLRYQ QQQGSRPHYR QISPRVRGDE QENEGSNIFS

241 GFAQEFLQHA FQVDRQTVEN LRGENEREEQ GAIVTVKGGL RILSPDEEDE SSRSPPSRRE EFDEDRSRPQ QRGKYDENRR

321 GYKNGIEETI CSASVKKNLG RSSNPDIYNP QAGSLR**SVNE LDLPILGWLG LSAQHGTIYR** NAMFVPHYTL NAHTIVVALN

401 GRAHVQVVDS NGNRVYDEEL QEGHVLVVPQ NFAVAAKAQS ENYEYLAFKT DSRPSIANLA GENSIIDNLP EEVVANSYRL

481 PREQARQLKN NNPFKFFVPP FDHQSMREVA

Supporting Peptides:

Peptide	Uniq	-10lgP	Mass	Length	ppm	m/z	z	RT	Fraction	Scan	Source File	Area KOntrola 9. traka	#Feature	#Feature KOntrola 9. traka	Start	End	PTM
R.SVNELDLPILGWLGLSAQHGTIYR.N	Y	36.74	2651.4070	24	0.4	884.8099	3	38.40	10	2088	OB4038.raw	4.22E3	1	1	357	380	
R.SVNELDLPILGWLGLSAQ(+.98)HGTIYR.N	Y	30.01	2652.3911	24	1.6	885.1390	3	38.70	10	2102	OB4038.raw	4.27E3	1	1	357	380	
**total 2 peptides**																	

tr|E5G077|E5G077_ARAHY

back to list

| Protein Coverage | Supporting Peptides |

Protein Coverage:

1 MAKLLALSLC FCVLVLGASS VTFRQGGEEN ECQFQRLNAQ RPDNRIESEG GYIETWNPNN QEFQCAGVAL SRTVLRRNAL

81 RRPFYSNAPL EIYVQQGSGY FGLIFPGCPS TYEEPAQEGR RYQSQKPSRR FQVGQDDPSQ QQQDSHQKVH RFDEGDLIAV

161 PTGVAFWMYN DEDTDVVTVT LSDTSSIHNQ LDQFPRRFYL AGNQEQEFLR YQQQQGSRPH YRQISPRVRG DEQENEGSNI

241 FSGFAQEFLQ HAFQVDRQTV ENLRGENERE EQGAIVTVKG GLRILSPDEE DESSRSPPNR REEFDEDRSR PQQRGKYDEN

321 RRGYKNGIEE TICSASVKKN LGRSSNPDIY NPQAGSLR**SV NELDLPILGW LGLSAQHGTI YR**NAMFVPHY TLNAHTIVVA

401 LNGRAHVQVV DSNGNRVYDE ELQEGHVLVV PQNFAVAAKA QSENYEYLAF KTDSRPSIAN QAGENSIIDN LPEEVVANSY

481 RLPREQARQL KNNNPFKFFV PPFDHQSMRE VA

Supporting Peptides:

Peptide	Uniq	-10lgP	Mass	Length	ppm	m/z	z	RT	Fraction	Scan	Source File	Area KOntrola 9. traka	#Feature	#Feature KOntrola 9. traka	Start	End	PTM
R.SVNELDLPILGWLGLSAQHGTIYR.N	Y	36.74	2651.4070	24	0.4	884.8099	3	38.40	10	2088	OB4038.raw	4.22E3	1	1	359	382	
R.SVNELDLPILGWLGLSAQ(+.98)HGTIYR.N	Y	30.01	2652.3911	24	1.6	885.1390	3	38.70	10	2102	OB4038.raw	4.27E3	1	1	359	382	
**total 2 peptides**																	

tr|Q0GM57|Q0GM57_ARAHY

back to list

| Protein Coverage | Supporting Peptides |

Protein Coverage:

1 MAKLLALSLC FCVLVLGASS VTFRQGGEEN ECQFQRLNAQ RPDNRIESEG GYIETWNPNN QEFQCAGVAL SRTVLRRNAL

81 RRPFYSNAPL EIYVQQGSGY FGLIFPGCPS TYEEPAQEGR RYQSQKPSRR FQVGQDDPSQ QQQDSHQKVH RFDEGDLIAV

161 PTGVAFWMYN DEDTDVVTVT LSDTSSIHNQ LDQFPRRFYL AGNQEQEFLR YQQQQGSRPH YRQISPRVRG DEQENEGSNI

241 FSGFAQEFLQ HAFQVDRQTV ENLRGENERE EQGAIVTVKG GLRILSPDEE DESSRSPPSR REEFDEDRSR PQQRGKYDEN

321 RRGYKNGIEE TICSASVKKN LGRSSNPDIY NPQAGSLR**SV NELDLPILGW LGLSAQHGTI YR**NAMFVPHY TLNAHTIVVA

401 LNGRAHVQVV DSNGNRVYDE ELQEGHVLVV PQNFAVAAKA QSENYEYLAF KTDSRPSIAN LAGENSIIDN LPEEVVANSY

481 RLPREQARQL KNNNPFKFFV PPFDHQSMRE VA

Supporting Peptides:

Peptide	Uniq	-10lgP	Mass	Length	ppm	m/z	z	RT	Fraction	Scan	Source File	Area KOntrola 9. traka	#Feature	#Feature KOntrola 9. traka	Start	End	PTM
R.SVNELDLPILGWLGLSAQHGTIYR.N	Y	36.74	2651.4070	24	0.4	884.8099	3	38.40	10	2088	OB4038.raw	4.22E3	1	1	359	382	
R.SVNELDLPILGWLGLSAQ(+.98)HGTIYR.N	Y	30.01	2652.3911	24	1.6	885.1390	3	38.70	10	2102	OB4038.raw	4.27E3	1	1	359	382	
**total 2 peptides**																	

Peptide List

Prepared with PEAKS ™ (bioinfor.com)

Summary

1. Notes Gastric Digest Raw peanut Band #9 29-33 kDa (PTM501)

2. Result Statistics

Figure 1. False discovery rate (FDR) curve. X axis is the number of peptide-spectrum matches (PSM) being kept. Y axis is the corresponding FDR.

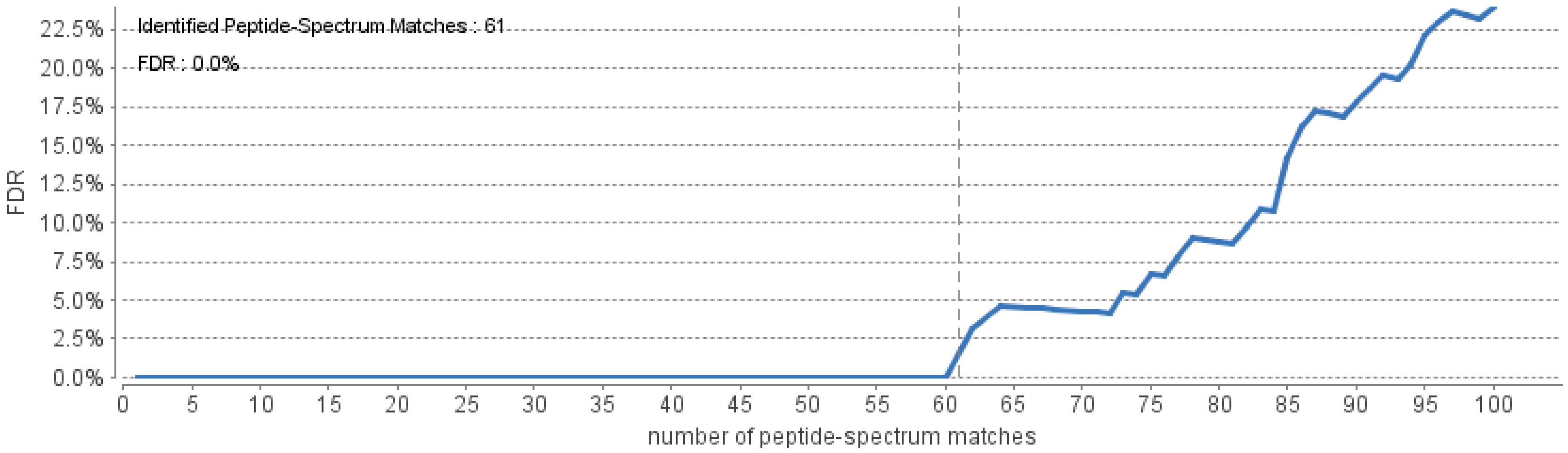

Figure 2. PSM score distribution. **(a)** Distribution of PEAKS peptide score; **(b)** Scatterplot of PEAKS peptide score versus precursor mass error.

(a)

(b)

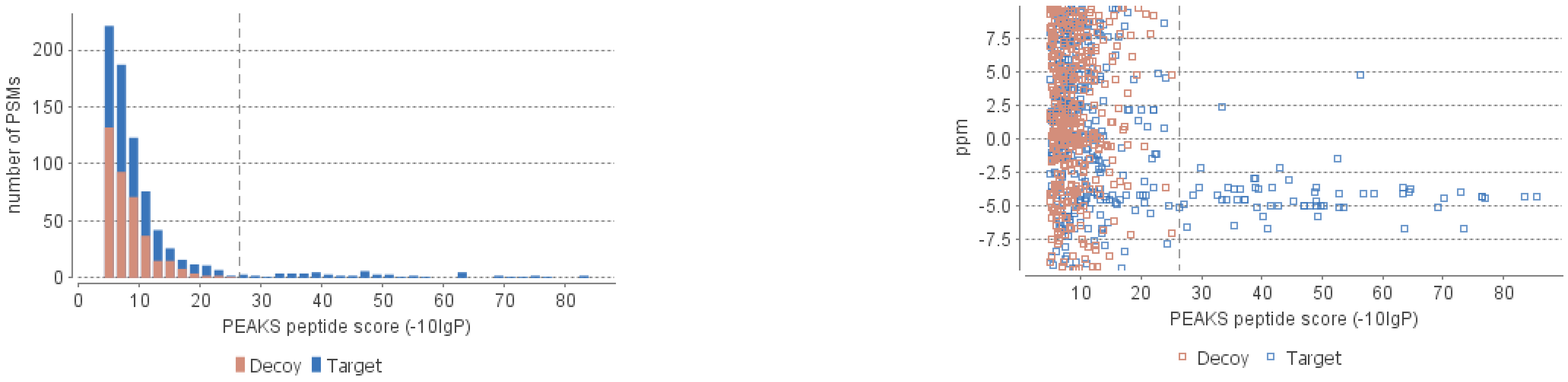

Figure 3. Distribution of peptide feature detection. **(a)** Feature m/z distribution; **(b)** Feature RT distribution

(a)

(b)

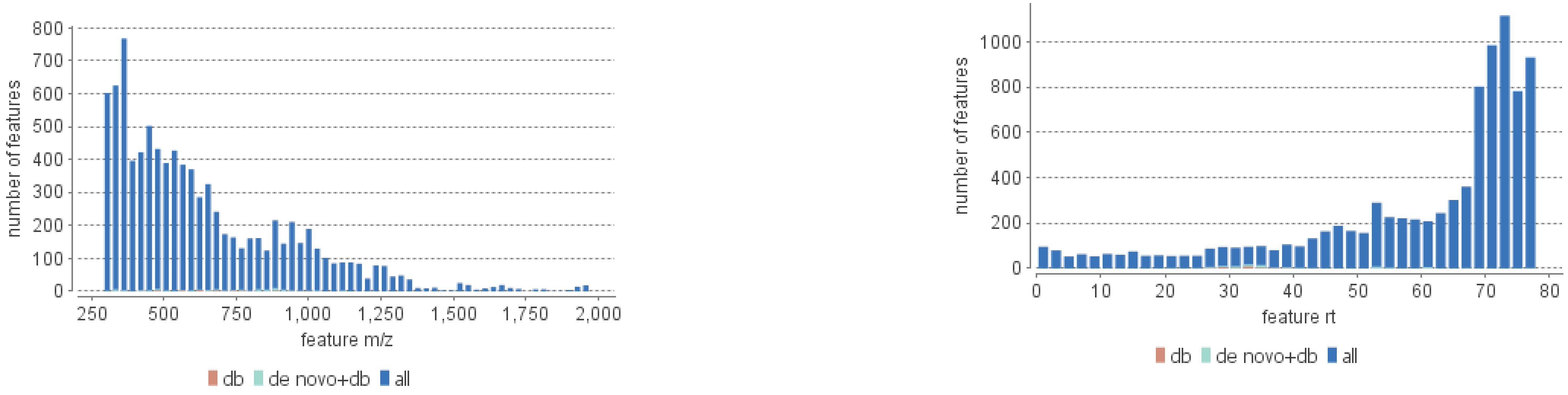

Figure 4. Distribution of identified peptide features. **(a)** Feature abundance distribution **(b)** *De novo* sequencing validation.

(a)

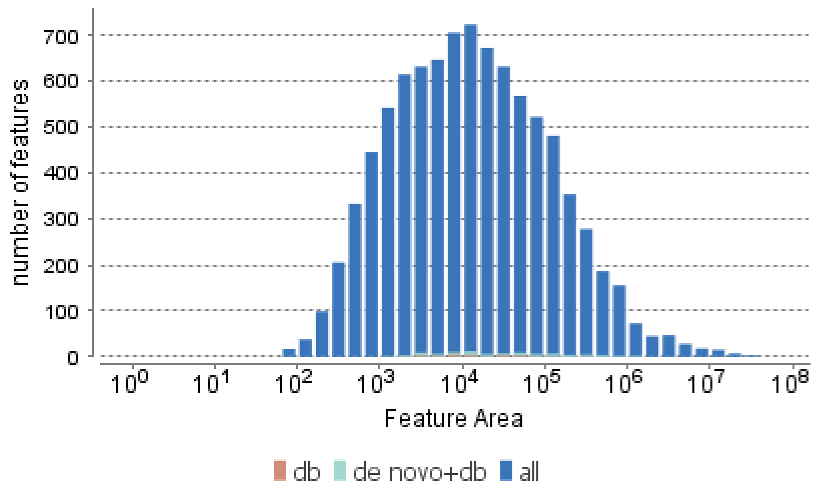

(b)

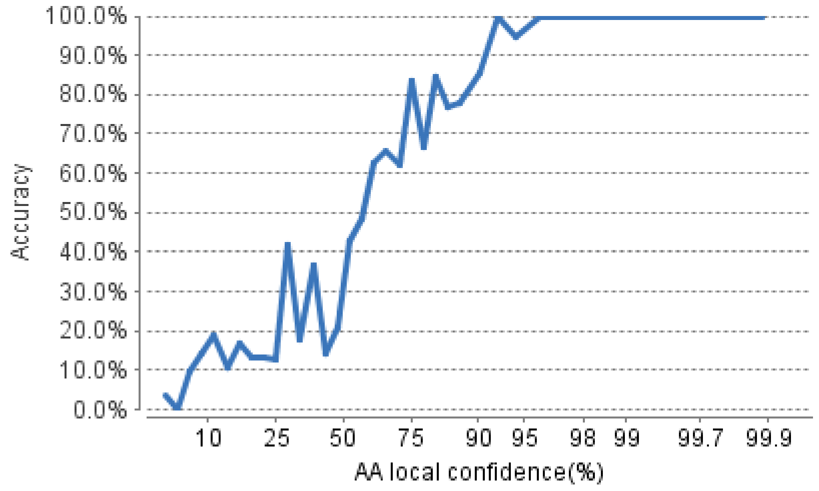

Table 1. Statistics of data.

# of MS scans	2594
# of MS/MS scans	1846
# of Features	9088
# of Chimera scans	187

Table 2. Result filtration parameters.

Peptide -10lgP	≥26.4
PTM Ascore	≥20
Protein -10lgP	≥20
Proteins unique peptides	≥1
De novo ALC Score	≥50%

Table 4. PTM profile.

Name	ΔMass	Position	#PSM	-10lgP	Abundance	AScore
Oxidation	15.99	M	20	69.14	5.02E4	1000.00
Deamidation	.98	NQ	14	73.48	7.08E3	1000.00
Carbamidomethyl	57.02	C	2	36.28	6.16E3	1000.00

Table 3. Statistics of filtered result.

Peptide-Spectrum Matches	61
Peptide sequences	33
Protein groups	6
Proteins	21
Proteins (#Unique Peptides)	12 (>2); 2 (=2); 7 (=1);
FDR (Peptide-Spectrum Matches)	0.0%
FDR (Peptide Sequences)	0.0%
FDR (Protein Group)	0.0%
De Novo Only Spectra	163
# of identified Features	93
# of identified MS/MS scans	61

3. Experiment Control

Figure 5. Precursor mass error of peptide-spectrum matches (PSM) in filtered result. **(a)** Distribution of precursor mass error in ppm; **(b)** Scatterplot of precursor m/z versus precursor mass error in ppm.

(a)

(b)

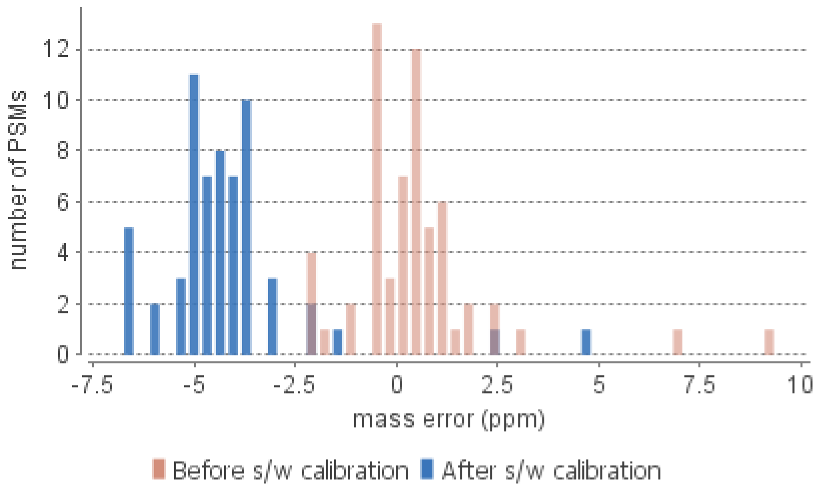

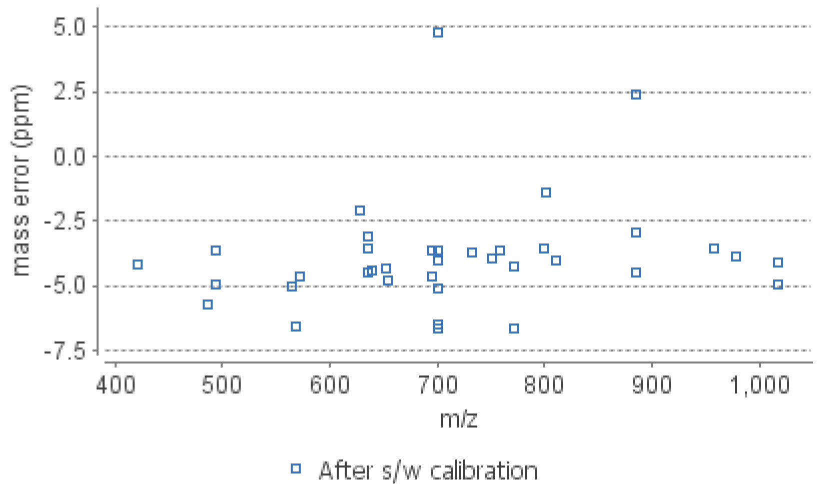

Table 5. Number of identified peptides in each sample by the number of missed cleavages

Missed Cleavages	0	1	2	3	4+
Digest 9. traka	22	11	0	0	0

4. Other Information

Table 6. Search parameters.

Search Engine Name: PEAKS
Parent Mass Error Tolerance: 10.0 ppm
Fragment Mass Error Tolerance: 0.5 Da
Precursor Mass Search Type: monoisotopic
Enzyme: Trypsin
Max Missed Cleavages: 2
Digest Mode: Unspecific
Fixed Modifications:

Table 7. Instrument parameters.

Fractions: OB4059.raw
Ion Source: ESI(nano-spray)
Fragmentation Mode: CID, CAD(y and b ions)
MS Scan Mode: FT-ICR/Orbitrap
MS/MS Scan Mode: Linear Ion Trap

Carbamidomethylation: 57.02
Variable Modifications:
Deamidation (NQ): 0.98
Oxidation (M): 15.99
Hydroxylation Pro: 15.99
Acetylation (K): 42.01
Acetylation (Protein N-term): 42.01
Acetylation (N-term): 42.01
Amidation: -0.98
Beta-methylthiolation: 45.99
and 305 more...
Max Variable PTM Per Peptide: 5
Database: Uniprot_Peanut-3818_Jul18
Taxon: All
Contaminant Database: contaminantsMQ_mar19
Searched Entry: 1723
FDR Estimation: Enabled
De novo score (ALC%) threshold: 15
Peptide hit threshold (-10logP): 30.0
Peaks run ID: 484
Merge Options: no merge
Precursor Options: corrected
Charge Options: no correction
Filter Charge: 2 - 8
Process: true
Associate chimera: yes

Protein List

Protein Accession Contains:
Protein Description Contains:
Peptide Sample Area >=
Protein Ptm Contains:

Protein Group	Protein ID	Accession	-10lgP	Coverage (%)	Coverage (%) Digest 9. traka	Area Digest 9. traka	#Peptides	#Unique	#Spec Digest 9. traka	PTM	Avg. Mass	Description
**1**	20853	tr|N1NG13|N1NG13_ARAHY	114.12	5	5	2.11E5	4	4	22	Y	71345	Seed storage protein Ara h1 OS=Arachis hypogaea OX=3818 GN=ARAX_AHF417E07-017 PE=4 SV=1
**1**	20854	P43238|ALL12_ARAHY	114.12	5	5	2.11E5	4	4	22	Y	71345	Allergen Ara h 1, clone P41B OS=Arachis hypogaea OX=3818 PE=1 SV=1
**1**	22400	tr|Q6PSU4|Q6PSU4_ARAHY	114.12	7	7	2.11E5	4	4	22	Y	48095	Conarachin (Fragment) OS=Arachis hypogaea OX=3818 PE=2 SV=1
**1**	20849	tr|Q6PSU3|Q6PSU3_ARAHY	114.12	6	6	2.11E5	4	4	22	Y	66575	Conarachin (Fragment) OS=Arachis hypogaea OX=3818 PE=4 SV=1
**1**	20850	P43237|ALL11_ARAHY	114.12	5	5	2.11E5	4	4	22	Y	70283	Allergen Ara h 1, clone P17 OS=Arachis hypogaea OX=3818 PE=1 SV=1
**1**	20851	tr|B3IXL2|B3IXL2_ARAHY	114.12	5	5	2.11E5	4	4	22	Y	70283	Main allergen Ara h1 OS=Arachis hypogaea OX=3818 PE=2 SV=1
**1**	21739	tr|Q6PSU6|Q6PSU6_ARAHY	114.12	11	11	2.11E5	4	4	22	Y	34133	Conarachin (Fragment) OS=Arachis hypogaea OX=3818 PE=2 SV=1
**1**	22695	tr|Q6PSU5|Q6PSU5_ARAHY	114.12	11	11	2.11E5	4	4	22	Y	33604	Conarachin (Fragment) OS=Arachis hypogaea OX=3818 PE=2 SV=1
**2**	20591	tr|Q6T2T4|Q6T2T4_ARAHY	107.80	4	4	3.12E4	2	1	10	Y	61499	Storage protein OS=Arachis hypogaea OX=3818 PE=2 SV=1
**2**	20580	tr|Q647H4|Q647H4_ARAHY	107.80	4	4	3.12E4	2	1	10	Y	61506	Arachin Ahy-1 OS=Arachis hypogaea OX=3818 PE=2 SV=1
**2**	20574	tr|A1DZF0|A1DZF0_ARAHY	107.80	5	5	3.12E4	2	1	10	Y	60375	Arachin 6 OS=Arachis hypogaea OX=3818 PE=2 SV=1
**2**	20578	tr|B5TYU1|B5TYU1_ARAHY	107.80	5	5	3.12E4	2	1	10	Y	60624	Arachin Arah3 isoform OS=Arachis hypogaea OX=3818 PE=1 SV=1
**4**	20579	tr|Q5I6T2|Q5I6T2_ARAHY	107.12	5	5	1.12E4	2	1	8	Y	60736	Arachin Ahy-4 OS=Arachis hypogaea OX=3818 PE=2 SV=1
**4**	20576	tr|Q9FZ11|Q9FZ11_ARAHY	107.12	5	5	1.12E4	2	1	8	Y	60449	Gly1 OS=Arachis hypogaea OX=3818 GN=Gly1 PE=2 SV=1
**4**	20581	tr|Q647H3|Q647H3_ARAHY	107.12	4	4	1.12E4	2	1	8	Y	61532	Arachin Ahy-2 OS=Arachis hypogaea OX=3818 PE=2 SV=1
**3**	20593	tr|Q6IWG5|Q6IWG5_ARAHY	106.08	10	10	1.8E5	3	3	8	Y	58061	Glycinin (Fragment) OS=Arachis hypogaea OX=3818 PE=2 SV=1
**3**	20594	tr|Q0GM57|Q0GM57_ARAHY	106.08	10	10	1.8E5	3	3	8	Y	58263	Iso-Ara h3 OS=Arachis hypogaea OX=3818 PE=2 SV=1

Protein Group	Protein ID	Accession	-10lgP	Coverage (%)	Coverage (%) Digest 9. traka	Area Digest 9. traka	#Peptides	#Unique	#Spec Digest 9. traka	PTM	Avg. Mass	Description
**3**	20595	tr|E5G077|E5G077_ARAHY	106.08	10	10	1.8E5	3	3	8	Y	58305	Ara h 3 allergen OS=Arachis hypogaea OX=3818 GN=ara h 3 PE=3 SV=1
**8**	21034	tr|Q38711|Q38711_ARAHY	95.54	9	9	1.42E4	2	2	2	N	29134	Galactose-binding lectin (Fragment) OS=Arachis hypogaea OX=3818 GN=lec PE=2 SV=1
**8**	21035	P02872|LECG_ARAHY	95.54	9	9	1.42E4	2	2	2	N	29325	Galactose-binding lectin OS=Arachis hypogaea OX=3818 PE=1 SV=3
**5**	30641	tr|B4UWA1|B4UWA1_ARAHY	88.96	24	24	2.45E4	4	4	7	Y	22284	Lactoylglutathione lyase (Fragment) OS=Arachis hypogaea OX=3818 PE=2 SV=1
**total 21 proteins**												

tr|N1NG13|N1NG13_ARAHY
back to list

| Protein Coverage | Supporting Peptides |

Protein Coverage:

d Deamidation (NQ) (+0.98)
o Oxidation (M) (+15.99)

1 MRGRVSPLML LLGILVLASV SATHAKSSPY QKKTENPCAQ RCLQSCQQEP DDLKQKACES RCTKLEYDPR CVYDPRGHTG

81 TTNQRSPPGE RTRGRQPGDY DDDRRQPRRE EGGRWGPAGP REREREEDWR QPREDWRRPS HQQPRKIRPE GREGEQEWGT

161 PGSHVREETS RNNPFYFPSR RFSTRYGNQN GRIRVLQRFD QRSRQFQNLQ NHRIVQIEAK PNTLVLPKHA DADNILVIQQ

241 GQATVTVANG NNRKSFNLDE GHALRIPSGF ISYILNRHDN QNLRVAKISM PVNTPGQFED FFPASSRDQS SYLQGFSRNT

321 LEAAFNAEFN EIRRVLLEEN AGGEQEERGQ RRWSTRSSEN NEGVIVKVSK EHVEELTKHA KSVSKKGSEE EGDITNPINL

(453 o, 459 d, 464 d)

401 REGEPDLSNN FGKLFEVKPD KKNPQLQDLD MMLTCVEIKE GALMLPHFNS K**AMVIVVVNK GTGNLELVAV R**KEQQQRGRR

(512 o)

481 EEEEDEDEEE EGSNREVRRY TAR**LKEGDVF IMPAA**HPVAI NASSELHLLG FGINAENNHR IFLAGDKDNV IDQIEKQAKD

561 LAFPGSGEQV EKLIKNQKES HFVSARPQSQ SQSPSSPEKE SPEKEDQEEE NQGGKGPLLS ILKAFN

Supporting Peptides:

Peptide	Uniq	-10lgP	Mass	Length	ppm	m/z	z	RT	Fraction	Scan	Source File	Area Digest 9. traka	#Feature	#Feature Digest 9. traka	Start	End	PTM
K.AM(+15.99)VIVVVNKGTGNLELVAVR.K	Y	69.14	2097.1980	20	-5.1	700.0730	3	31.73	31	1627	OB4059.raw	5.02E4	1	1	452	471	Oxidation (M)
K.AM(+15.99)VIVVVNKGTGN(+.98)LELVAVR.K	Y	64.46	2098.1819	20	-4.0	700.4017	3	32.17	31	1659	OB4059.raw	3.83E4	1	1	452	471	Oxidation (M)
K.AM(+15.99)VIVVVN(+.98)KGTGN(+.98)LELVAVR.K	Y	56.21	2099.1660	20	4.8	700.7359	3	32.50	31	1678	OB4059.raw	0	0	0	452	471	Oxidation (M); Deamidation (NQ)
K.AM(+15.99)VIVVVNK.G	Y	50.27	987.5787	9	-5.0	494.7965	2	26.07	31	1293	OB4059.raw	5.67E4	1	1	452	460	Oxidation (M)
K.AMVIVVVNK.G	Y	49.24	971.5837	9	-5.7	486.7986	2	28.57	31	1430	OB4059.raw	7.07E3	1	1	452	460	
K.AMVIVVVNKGTGNLELVAVR.K	Y	48.98	2081.2031	20	-4.6	694.7417	3	33.03	31	1713	OB4059.raw	7.38E3	1	1	452	471	
K.GTGNLELVAVR.K	Y	39.14	1127.6299	11	-5.0	564.8220	2	29.06	31	1470	OB4059.raw	5.16E4	1	1	461	471	
R.LKEGDVFIM(+15.99)PAA.H	Y	27.17	1305.6638	12	-4.8	653.8391	2	29.79	31	1511	OB4059.raw	0	0	0	504	515	Oxidation (M)
**total 8 peptides**																	

P43238|ALL12_ARAHY
back to list

| Protein Coverage | Supporting Peptides |

Protein Coverage:

```
  1 MRGRVSPLML LLGILVLASV SATHAKSSPY QKKTENPCAQ RCLQSCQQEP DDLKQKACES RCTKLEYDPR CVYDPRGHTG
 81 TTNQRSPPGE RTRGRQPGDY DDDRRQPRRE EGGRWGPAGP REREREEDWR QPREDWRRPS HQQPRKIRPE GREGEQEWGT
161 PGSHVREETS RNNPFYFPSR RFSTRYGNQN GRIRVLQRFD QRSRQFQNLQ NHRIVQIEAK PNTLVLPKHA DADNILVIQQ
241 GQATVTVANG NNRKSFNLDE GHALRIPSGF ISYILNRHDN QNLRVAKISM PVNTPGQFED FFPASSRDQS SYLQGFSRNT
321 LEAAFNAEFN EIRRVLLEEN AGGEQEERGQ RRWSTRSSEN NEGVIVKVSK EHVEELTKHA KSVSKKGSEE EGDITNPINL
401 REGEPDLSNN FGKLFEVKPD KKNPQLQDLD MMLTCVEIKE GALMLPHFNS KAMVIVVVNK GTGNLELVAV RKEQQQRGRR
481 EEEEDEDEEE EGSNREVRRY TARLKEGDVF IMPAAHPVAI NASSELHLLG FGINAENNHR IFLAGDKDNV IDQIEKQAKD
561 LAFPGSGEQV EKLIKNQKES HFVSARPQSQ SQSPSSPEKE SPEKEDQEEE NQGGKGPLLS ILKAFN
```

Modification sites: 453 o, 459 d, 464 d, 512 o

d Deamidation (NQ) (+0.98)
o Oxidation (M) (+15.99)

Supporting Peptides:

Peptide	Uniq	-10lgP	Mass	Length	ppm	m/z	z	RT	Fraction	Scan	Source File	Area Digest 9. traka	#Feature	#Feature Digest 9. traka	Start	End	PTM
K.AM(+15.99)VIVVVNKGTGNLELVAVR.K	Y	69.14	2097.1980	20	-5.1	700.0730	3	31.73	31	1627	OB4059.raw	5.02E4	1	1	452	471	Oxidation (M)
K.AM(+15.99)VIVVVNKGTGN(+.98)LELVAVR.K	Y	64.46	2098.1819	20	-4.0	700.4017	3	32.17	31	1659	OB4059.raw	3.83E4	1	1	452	471	Oxidation (M)
K.AM(+15.99)VIVVVN(+.98)KGTGN(+.98)LELVAVR.K	Y	56.21	2099.1660	20	4.8	700.7359	3	32.50	31	1678	OB4059.raw	0	0	0	452	471	Oxidation (M); Deamidation (NQ)
K.AM(+15.99)VIVVVNK.G	Y	50.27	987.5787	9	-5.0	494.7965	2	26.07	31	1293	OB4059.raw	5.67E4	1	1	452	460	Oxidation (M)
K.AMVIVVVNK.G	Y	49.24	971.5837	9	-5.7	486.7986	2	28.57	31	1430	OB4059.raw	7.07E3	1	1	452	460	
K.AMVIVVVNKGTGNLELVAVR.K	Y	48.98	2081.2031	20	-4.6	694.7417	3	33.03	31	1713	OB4059.raw	7.38E3	1	1	452	471	
K.GTGNLELVAVR.K	Y	39.14	1127.6299	11	-5.0	564.8220	2	29.06	31	1470	OB4059.raw	5.16E4	1	1	461	471	
R.LKEGDVFIM(+15.99)PAA.H	Y	27.17	1305.6638	12	-4.8	653.8391	2	29.79	31	1511	OB4059.raw	0	0	0	504	515	Oxidation (M)
**total 8 peptides**																	

tr|Q6PSU4|Q6PSU4_ARAHY

back to list

| Protein Coverage | Supporting Peptides |

Protein Coverage:

```
  1  GFDQRSRQFQ NLQNHRIVQI EAKPNTLVLP KHADADNILV IQQGQATVTV ANGNNRKSFN LDEGHALRIP SGFISYILNR
 81  HDNQNLRVAK ISMPVNTPGQ FEDFFPASSR DQSSYLQGFS RNTLEAAFNA EFNEIRRVLL EENAGGEQEE RGQRRWSTRS
161  SENNEGVIVK VSKEHVEELT KHAKSVSKKG SEEGDITNPI NLREGEPDLS NNFGKLFEVK PDKKNPQLQD LDMMLTCVEI
241  KEGALMLPHF NSKAMVIVVV NKGTGNLELV AVRKEQQQRG RREEEEDEDE EEEGSNREVR RYTARLKEGD VFIMPAAHPV
321  AINASSELHL LGFGINAENN HRIFLAGDKD NVIDQIEKQA KDLAFPGSGE QVEKLIKNQK ESHFVSARPQ SQSQSPSSPE
401  KESPEKEDQE EENQGGKGPL LSILKAFN
```

Modification sites: 255 (o), 261 (d), 266 (d), 314 (o)

d Deamidation (NQ) (+0.98)
o Oxidation (M) (+15.99)

Supporting Peptides:

Peptide	Uniq	-10lgP	Mass	Length	ppm	m/z	z	RT	Fraction	Scan	Source File	Area Digest 9. traka	#Feature	#Feature Digest 9. traka	Start	End	PTM
K.AM(+15.99)VIVVVNKGTGNLELVAVR.K	Y	69.14	2097.1980	20	-5.1	700.0730	3	31.73	31	1627	OB4059.raw	5.02E4	1	1	254	273	Oxidation (M)
K.AM(+15.99)VIVVVNKGTGN(+.98)LELVAVR.K	Y	64.46	2098.1819	20	-4.0	700.4017	3	32.17	31	1659	OB4059.raw	3.83E4	1	1	254	273	Oxidation (M)
K.AM(+15.99)VIVVVN(+.98)KGTGN(+.98)LELVAVR.K	Y	56.21	2099.1660	20	4.8	700.7359	3	32.50	31	1678	OB4059.raw	0	0	0	254	273	Oxidation (M); Deamidation (NQ)
K.AM(+15.99)VIVVVNK.G	Y	50.27	987.5787	9	-5.0	494.7965	2	26.07	31	1293	OB4059.raw	5.67E4	1	1	254	262	Oxidation (M)
K.AMVIVVVNK.G	Y	49.24	971.5837	9	-5.7	486.7986	2	28.57	31	1430	OB4059.raw	7.07E3	1	1	254	262	
K.AMVIVVVNKGTGNLELVAVR.K	Y	48.98	2081.2031	20	-4.6	694.7417	3	33.03	31	1713	OB4059.raw	7.38E3	1	1	254	273	
K.GTGNLELVAVR.K	Y	39.14	1127.6299	11	-5.0	564.8220	2	29.06	31	1470	OB4059.raw	5.16E4	1	1	263	273	
R.LKEGDVFIM(+15.99)PAA.H	Y	27.17	1305.6638	12	-4.8	653.8391	2	29.79	31	1511	OB4059.raw	0	0	0	306	317	Oxidation (M)
**total 8 peptides**																	

tr|Q6PSU3|Q6PSU3_ARAHY

back to list

| Protein Coverage | Supporting Peptides |

Protein Coverage:

d Deamidation (NQ) (+0.98)
o Oxidation (M) (+15.99)

```
  1 MRGRVSPLML LLGILVLASV SATQAKSPYR KTENPCAQRC LQSCQQEPDD LKQKACESRC TKLEYDPRCV YDTGATNQRH
 81 PPGERTRGRQ PGDYDDDRRQ PRREEGGRWG PAEPRERERE EDWRQPREDW RRPSHQQPRK IRPEGREGEQ EWGTPGSEVR
161 EETSRNNPFY FPSRRFSTRY GNQNGRIRVL QRFDQRSKQF QNLQNHRIVQ IEARPNTLVL PKHADADNIL VIQQGQATVT
241 VANGNNRKSF NLDEGHALRI PSGFISYILN RHDNQNLRVA KISMPVNTPG QFEDFFPASS RDQSSYLQGF SRNTLEAAFN
321 AEFNEIRRVL LEENAGGEQE ERGQRRRSTR SSDNEGVIVK VSKEHVQELT KHAKSVSKKG SEEEDITNPI NLRDGEPDLS
401 NNFGRLFEVK PDKKNPQLQD LDMMLTCVEI KEGALMLPHF NSKAMVIVVV NKGTGNLELV AVRKEQQQRG RREQEWEEEE
481 EDEEEEGSNR EVRRYTARLK EGDVFIMPAA HPVAINASSE LHLLGFGINA ENNHRIFLAG DKDNVIDQIE KQAKDLAFPG
561 SGEQVEKLIK NQRESHFVSA
```

Supporting Peptides:

Peptide	Uniq	-10lgP	Mass	Length	ppm	m/z	z	RT	Fraction	Scan	Source File	Area Digest 9. traka	#Feature	#Feature Digest 9. traka	Start	End	PTM
K.AM(+15.99)VIVVVNKGTGNLELVAVR.K	Y	69.14	2097.1980	20	-5.1	700.0730	3	31.73	31	1627	OB4059.raw	5.02E4	1	1	444	463	Oxidation (M)
K.AM(+15.99)VIVVVNKGTGN(+.98)LELVAVR.K	Y	64.46	2098.1819	20	-4.0	700.4017	3	32.17	31	1659	OB4059.raw	3.83E4	1	1	444	463	Oxidation (M)
K.AM(+15.99)VIVVVN(+.98)KGTGN(+.98)LELVAVR.K	Y	56.21	2099.1660	20	4.8	700.7359	3	32.50	31	1678	OB4059.raw	0	0	0	444	463	Oxidation (M); Deamidation (NQ)
K.AM(+15.99)VIVVVNK.G	Y	50.27	987.5787	9	-5.0	494.7965	2	26.07	31	1293	OB4059.raw	5.67E4	1	1	444	452	Oxidation (M)
K.AMVIVVVNK.G	Y	49.24	971.5837	9	-5.7	486.7986	2	28.57	31	1430	OB4059.raw	7.07E3	1	1	444	452	
K.AMVIVVVNKGTGNLELVAVR.K	Y	48.98	2081.2031	20	-4.6	694.7417	3	33.03	31	1713	OB4059.raw	7.38E3	1	1	444	463	
K.GTGNLELVAVR.K	Y	39.14	1127.6299	11	-5.0	564.8220	2	29.06	31	1470	OB4059.raw	5.16E4	1	1	453	463	
R.LKEGDVFIM(+15.99)PAA.H	Y	27.17	1305.6638	12	-4.8	653.8391	2	29.79	31	1511	OB4059.raw	0	0	0	499	510	Oxidation (M)
**total 8 peptides**																	

P43237|ALL11_ARAHY

back to list

| Protein Coverage | Supporting Peptides |

Protein Coverage:

d Deamidation (NQ) (+0.98)
o Oxidation (M) (+15.99)

1 MRGRVSPLML LLGILVLASV SATQAKSPYR KTENPCAQRC LQSCQQEPDD LKQKACESRC TKLEYDPRCV YDTGATNQRH
81 PPGERTRGRQ PGDYDDDRRQ PRREEGGRWG PAEPRERERE EDWRQPREDW RRPSHQQPRK IRPEGREGEQ EWGTPGSEVR
161 EETSRNNPFY FPSRRFSTRY GNQNGRIRVL QRFDQRSKQF QNLQNHRIVQ IEARPNTLVL PKHADADNIL VIQQGQATVT
241 VANGNNRKSF NLDEGHALRI PSGFISYILN RHDNQNLRVA KISMPVNTPG QFEDFFPASS RDQSSYLQGF SRNTLEAAFN
321 AEFNEIRRVL LEENAGGEQE ERGQRRRSTR SSDNEGVIVK VSKEHVQELT KHAKSVSKKG SEEEDITNPI NLRDGEPDLS
401 NNFGRLFEVK PDKKNPQLQD LDMMLTCVEI KEGALMLPHF NSK**AMVIVVV NKGTGNLELV AVR**KEQQQRG RREQEWEEEE
481 EDEEEEGSNR EVRRYTAR**LK EGDVFIMPAA** HPVAINASSE LHLLGFGINA ENNHRIFLAG DKDNVIDQIE KQAKDLAFPG
561 SGEQVEKLIK NQRESHFVSA RPQSQSPSSP EKEDQEEENQ GGKGPLLSIL KAFN

Supporting Peptides:

Peptide	Uniq	-10lgP	Mass	Length	ppm	m/z	z	RT	Fraction	Scan	Source File	Area Digest 9. traka	#Feature	#Feature Digest 9. traka	Start	End	PTM
K.AM(+15.99)VIVVVNKGTGNLELVAVR.K	Y	69.14	2097.1980	20	-5.1	700.0730	3	31.73	31	1627	OB4059.raw	5.02E4	1	1	444	463	Oxidation (M)
K.AM(+15.99)VIVVVNKGTGN(+.98)LELVAVR.K	Y	64.46	2098.1819	20	-4.0	700.4017	3	32.17	31	1659	OB4059.raw	3.83E4	1	1	444	463	Oxidation (M)
K.AM(+15.99)VIVVVN(+.98)KGTGN(+.98)LELVAVR.K	Y	56.21	2099.1660	20	4.8	700.7359	3	32.50	31	1678	OB4059.raw	0	0	0	444	463	Oxidation (M); Deamidation (NQ)
K.AM(+15.99)VIVVVNK.G	Y	50.27	987.5787	9	-5.0	494.7965	2	26.07	31	1293	OB4059.raw	5.67E4	1	1	444	452	Oxidation (M)
K.AMVIVVVNK.G	Y	49.24	971.5837	9	-5.7	486.7986	2	28.57	31	1430	OB4059.raw	7.07E3	1	1	444	452	
K.AMVIVVVNKGTGNLELVAVR.K	Y	48.98	2081.2031	20	-4.6	694.7417	3	33.03	31	1713	OB4059.raw	7.38E3	1	1	444	463	
K.GTGNLELVAVR.K	Y	39.14	1127.6299	11	-5.0	564.8220	2	29.06	31	1470	OB4059.raw	5.16E4	1	1	453	463	
R.LKEGDVFIM(+15.99)PAA.H	Y	27.17	1305.6638	12	-4.8	653.8391	2	29.79	31	1511	OB4059.raw	0	0	0	499	510	Oxidation (M)
**total 8 peptides**																	

tr|B3IXL2|B3IXL2_ARAHY

back to list

| Protein Coverage | Supporting Peptides |

Protein Coverage:

d Deamidation (NQ) (+0.98)
o Oxidation (M) (+15.99)

```
  1 MRGRVSPLML LLGILVLASV SATQAKSPYR KTENPCAQRC LQSCQQEPDD LKQKACESRC TKLEYDPRCV YDTGATNQRH
 81 PPGERTRGRQ PGDYDDDRRQ PRREEGGRWG PAEPRERERE EDWRQPREDW RRPSHQQPRK IRPEGREGEQ EWGTPGSEVR
161 EETSRNNPFY FPSRRFSTRY GNQNGRIRVL QRFDQRSKQF QNLQNHRIVQ IEARPNTLVL PKHADADNIL VIQQGQATVT
241 VANGNNRKSF NLDEGHALRI PSGFISYILN RHDNQNLRVA KISMPVNTPG QFEDFFPASS RDQSSYLQGF SRNTLEAAFN
321 AEFNEIRRVL LEENAGGEQE ERGQRRRSTR SSDNEGVIVK VSKEHVQELT KHAKSVSKKG SEEEDITNPI NLRDGEPDLS
                                               445       451  456
401 NNFGRLFEVK PDKKNPQLQD LDMMLTCVEI KEGALMLPHF NSKAMVIVVV NKGTGNLELV AVRKEQQQRG RREQEWEEEE
                             507
481 EDEEEEGSNR EVRRYTARLK EGDVFIMPAA HPVAINASSE LHLLGFGINA ENNHRIFLAG DKDNVIDQIE KQAKDLAFPG
561 SGEQVEKLIK NQRESHFVSA RPQSQPSSSP EKEDQEEENQ GGKGPLLSIL KAFN
```

Supporting Peptides:

Peptide	Uniq	-10lgP	Mass	Length	ppm	m/z	z	RT	Fraction	Scan	Source File	Area Digest 9. traka	#Feature	#Feature Digest 9. traka	Start	End	PTM
K.AM(+15.99)VIVVVNKGTGNLELVAVR.K	Y	69.14	2097.1980	20	-5.1	700.0730	3	31.73	31	1627	OB4059.raw	5.02E4	1	1	444	463	Oxidation (M)
K.AM(+15.99)VIVVVNKGTGN(+.98)LELVAVR.K	Y	64.46	2098.1819	20	-4.0	700.4017	3	32.17	31	1659	OB4059.raw	3.83E4	1	1	444	463	Oxidation (M)
K.AM(+15.99)VIVVVN(+.98)KGTGN(+.98)LELVAVR.K	Y	56.21	2099.1660	20	4.8	700.7359	3	32.50	31	1678	OB4059.raw	0	0	0	444	463	Oxidation (M); Deamidation (NQ)
K.AM(+15.99)VIVVVNK.G	Y	50.27	987.5787	9	-5.0	494.7965	2	26.07	31	1293	OB4059.raw	5.67E4	1	1	444	452	Oxidation (M)
K.AMVIVVVNK.G	Y	49.24	971.5837	9	-5.7	486.7986	2	28.57	31	1430	OB4059.raw	7.07E3	1	1	444	452	
K.AMVIVVVNKGTGNLELVAVR.K	Y	48.98	2081.2031	20	-4.6	694.7417	3	33.03	31	1713	OB4059.raw	7.38E3	1	1	444	463	
K.GTGNLELVAVR.K	Y	39.14	1127.6299	11	-5.0	564.8220	2	29.06	31	1470	OB4059.raw	5.16E4	1	1	453	463	
R.LKEGDVFIM(+15.99)PAA.H	Y	27.17	1305.6638	12	-4.8	653.8391	2	29.79	31	1511	OB4059.raw	0	0	0	499	510	Oxidation (M)
**total 8 peptides**																	

tr|Q6PSU6|Q6PSU6_ARAHY

back to list

| [Protein Coverage](#) | [Supporting Peptides](#) |

Protein Coverage:

1 GNTLEAAFNA EFNEIRRVLL EENAGGEQEE RGQRRRSTRS SDNEGVIVKV SKEHVQELTK HAKSVSKKGS EEEDITNPIN

81 LRDGEPDLSN NFGRLFEVKP DKKNPQLQDL DMMLTCVEIK EGALMLPHFN SK**AMVIVVVN KGTGNLELVA VR**KEQQQRGR

161 REQEWEEEEE DEEEEGSNRE VRRYTAR**LKE GDVFIMPAA**H PVAINASSEL HLLGFGINAE NNHRIFLAGD KDNVIDQIEK

241 QAKDLAFPGS GEQVEKLIKN QRESHFVSAR PQSQSPSSPE KEDQEEENQG GKGPLLSILK AFN

Supporting Peptides:

Peptide	Uniq	-10lgP	Mass	Length	ppm	m/z	z	RT	Fraction	Scan	Source File	Area Digest 9. traka	#Feature	#Feature Digest 9. traka	Start	End	PTM
K.AM(+15.99)VIVVVNKGTGNLELVAVR.K	Y	69.14	2097.1980	20	-5.1	700.0730	3	31.73	31	1627	OB4059.raw	5.02E4	1	1	133	152	Oxidation (M)
K.AM(+15.99)VIVVVNKGTGN(+.98)LELVAVR.K	Y	64.46	2098.1819	20	-4.0	700.4017	3	32.17	31	1659	OB4059.raw	3.83E4	1	1	133	152	Oxidation (M)
K.AM(+15.99)VIVVVN(+.98)KGTGN(+.98)LELVAVR.K	Y	56.21	2099.1660	20	4.8	700.7359	3	32.50	31	1678	OB4059.raw	0	0	0	133	152	Oxidation (M); Deamidation (NQ)
K.AM(+15.99)VIVVVNK.G	Y	50.27	987.5787	9	-5.0	494.7965	2	26.07	31	1293	OB4059.raw	5.67E4	1	1	133	141	Oxidation (M)
K.AMVIVVVNK.G	Y	49.24	971.5837	9	-5.7	486.7986	2	28.57	31	1430	OB4059.raw	7.07E3	1	1	133	141	
K.AMVIVVVNKGTGNLELVAVR.K	Y	48.98	2081.2031	20	-4.6	694.7417	3	33.03	31	1713	OB4059.raw	7.38E3	1	1	133	152	
K.GTGNLELVAVR.K	Y	39.14	1127.6299	11	-5.0	564.8220	2	29.06	31	1470	OB4059.raw	5.16E4	1	1	142	152	
R.LKEGDVFIM(+15.99)PAA.H	Y	27.17	1305.6638	12	-4.8	653.8391	2	29.79	31	1511	OB4059.raw	0	0	0	188	199	Oxidation (M)
**total 8 peptides**																	

tr|Q6PSU5|Q6PSU5_ARAHY

[back to list](#)

| [Protein Coverage](#) | [Supporting Peptides](#) |

Protein Coverage:

1 LEAAFNAEFN EIRRVLLEEN AGGEQEERGQ RRWSTRSSEN NEGVIVKVSK EHVEELTKHA KSVSKKGSEE EGDITNPINL

81 REGEPDLSNN FGKLFEVKPD KKNPQLQDLD MMLTCVEIKE GALMLPHFNS K**AMVIVVVNK GTGNLELVAV R**KEQQQRGRR

161 EEEEDEDQEE EGSNREVRRY TAR**LKEGDVF IMPAA**HPVAI NASSELHLLG FGINAENNHR IFLAGDKDNV IDQIEKQAKD

241 LAFPGSGEQV EKLIKNQRES HFVSARPQSQ SPSSPEKEDQ EEENQGGKGP LLSILKAFN

d Deamidation (NQ) (+0.98)
o Oxidation (M) (+15.99)

Supporting Peptides:

Peptide	Uniq	-10lgP	Mass	Length	ppm	m/z	z	RT	Fraction	Scan	Source File	Area Digest 9. traka	#Feature	#Feature Digest 9. traka	Start	End	PTM
K.AM(+15.99)VIVVVNKGTGNLELVAVR.K	Y	69.14	2097.1980	20	-5.1	700.0730	3	31.73	31	1627	OB4059.raw	5.02E4	1	1	132	151	Oxidation (M)
K.AM(+15.99)VIVVVNKGTGN(+.98)LELVAVR.K	Y	64.46	2098.1819	20	-4.0	700.4017	3	32.17	31	1659	OB4059.raw	3.83E4	1	1	132	151	Oxidation (M)
K.AM(+15.99)VIVVVN(+.98)KGTGN(+.98)LELVAVR.K	Y	56.21	2099.1660	20	4.8	700.7359	3	32.50	31	1678	OB4059.raw	0	0	0	132	151	Oxidation (M); Deamidation (NQ)
K.AM(+15.99)VIVVVNK.G	Y	50.27	987.5787	9	-5.0	494.7965	2	26.07	31	1293	OB4059.raw	5.67E4	1	1	132	140	Oxidation (M)
K.AMVIVVVNK.G	Y	49.24	971.5837	9	-5.7	486.7986	2	28.57	31	1430	OB4059.raw	7.07E3	1	1	132	140	
K.AMVIVVVNKGTGNLELVAVR.K	Y	48.98	2081.2031	20	-4.6	694.7417	3	33.03	31	1713	OB4059.raw	7.38E3	1	1	132	151	
K.GTGNLELVAVR.K	Y	39.14	1127.6299	11	-5.0	564.8220	2	29.06	31	1470	OB4059.raw	5.16E4	1	1	141	151	
R.LKEGDVFIM(+15.99)PAA.H	Y	27.17	1305.6638	12	-4.8	653.8391	2	29.79	31	1511	OB4059.raw	0	0	0	184	195	Oxidation (M)
**total 8 peptides**																	

tr|Q6T2T4|Q6T2T4_ARAHY

back to list

| Protein Coverage | Supporting Peptides |

Protein Coverage:

d Deamidation (NQ) (+0.98)

1 MGKLLALSVC FCFLVLGASS ISFRQQPEEN ACQFQRLNAQ RPDNRIESEG GYIETWNPNN QEFECAGVAL SRLVLRRNAL
81 RRPFYSNAPQ EIFIQQGRAY FGLIFLGCPS TYEEPAQQGR RHQSQRPPRR FQGQDQSQQQ QDSHQKVHRF DEGDLIAVPT
161 GVAFWMYNDH DTDVVAVSLT DTNNNDNQLD QFPRRFNLAG NHEQEFLRYQ QQSRRRSLPY SPYSPQTQPK QEDREFSPRG
241 QHGRRERAGQ EQENEGGNIF SGFTPEFLAQ AFQVDDRQIL QNLRGENESD EQGAIVTVRG GLRILSPDRK KRQQYERPDE

386 d

321 EEEYDEDEYE YDEEERQQDR RRGRGSRGSG NGIEETICTA SFKKNIGRNR SPDIYNPQAG SLK**TANELNL LILRWLGLSA**

d

404 d

401 **EYGNLYR**NAL FVPHYNTNAH SIIYALRGRA HVQVVDSNGD RVFDEELQEG HVLVVPQNFA VAGKSQSENF EYVAFKTDSR

d

481 PSIANLAGEN SFIDNLPEEV VANSYGLPRE QARQLKNNNP FKFFVPPSEQ SLRAVA

Supporting Peptides:

Peptide	Uniq	-10lgP	Mass	Length	ppm	m/z	z	RT	Fraction	Scan	Source File	Area Digest 9. traka	#Feature	#Feature Digest 9. traka	Start	End	PTM
R.WLGLSAEYGNLYR.N	N	85.61	1540.7673	13	-4.2	771.3913	2	33.40	31	1730	OB4059.raw	1.41E5	1	1	395	407	
R.WLGLSAEYGN(+.98)LYR.N	N	73.48	1541.7513	13	-6.7	771.8814	2	33.83	31	1759	OB4059.raw	7.08E3	1	1	395	407	Deamidation (NQ)
K.TANELNLLILR.W	Y	44.38	1268.7452	11	-3.1	635.3809	2	34.02	31	1772	OB4059.raw	1.16E4	1	1	384	394	
K.TAN(+.98)ELNLLILR.W	Y	37.10	1269.7292	11	-4.5	635.8720	2	34.37	31	1792	OB4059.raw	9.79E3	1	1	384	394	Deamidation (NQ)
K.TANELN(+.98)LLILR.W	Y	35.88	1269.7292	11	-4.5	635.8720	2	34.37	31	1802	OB4059.raw	9.79E3	1	1	384	394	
**total 5 peptides**																	

tr|Q647H4|Q647H4_ARAHY

back to list

| Protein Coverage | Supporting Peptides |

Protein Coverage:

1 MGKLLALSVC FCFLVLGASS ISFRQQPEEN ACQFQRLNAQ RPDNRIESEG GYIETWNPNN QEFECAGVAL SRLVLRRNAL

81 RRPFYSNAPQ EIFIQQGRGY FGLIFPGCPS TYEEPAQQGR RHQSQRPPRR FQGQDQSQQQ QDSHQKVHRF DEGDLIAVPT

161 GVAFWMYNDH DTDVVAVSLT DTNNNDNQLD QFPRRFNLAG NHEQEFLRYQ QQSRRRSLPY SPYSPQTQPK QEDREFSPRG

241 QHGRRERAGQ EQENEGGNIF SGFTPEFLAQ AFQVDDRQIL QNLRGENESD EQGAIVTVRG GLRILSPDRK RRQQYERPDE

321 EEEYDEDEYE YDEEERQHDR RRGRGSRGSG NGIEETICTA SFKKNIGRNR SPDIYNPQAG SLK**TANELNL LILRWLGLSA**

401 **EYGNLYR**NAL FVPHYNTNAH SIIYALRGRA HVQVVDSNGD RVFDEELQEG HVLVVPQNFA VAGKSQSENF EYVAFKTDSR

481 PSIANLAGEN SFIDNLPEEV VANSYGLPRE QARQLKNNNP FKFFVPPSEQ SLRAVA

d Deamidation (NQ) (+0.98)

386 d

404 d

Supporting Peptides:

Peptide	Uniq	-10lgP	Mass	Length	ppm	m/z	z	RT	Fraction	Scan	Source File	Area Digest 9. traka	#Feature	#Feature Digest 9. traka	Start	End	PTM
R.WLGLSAEYGNLYR.N	N	85.61	1540.7673	13	-4.2	771.3913	2	33.40	31	1730	OB4059.raw	1.41E5	1	1	395	407	
R.WLGLSAEYGN(+.98)LYR.N	N	73.48	1541.7513	13	-6.7	771.8814	2	33.83	31	1759	OB4059.raw	7.08E3	1	1	395	407	Deamidation (NQ)
K.TANELNLLILR.W	Y	44.38	1268.7452	11	-3.1	635.3809	2	34.02	31	1772	OB4059.raw	1.16E4	1	1	384	394	
K.TAN(+.98)ELNLLILR.W	Y	37.10	1269.7292	11	-4.5	635.8720	2	34.37	31	1792	OB4059.raw	9.79E3	1	1	384	394	Deamidation (NQ)
K.TANELN(+.98)LLILR.W	Y	35.88	1269.7292	11	-4.5	635.8720	2	34.37	31	1802	OB4059.raw	9.79E3	1	1	384	394	
**total 5 peptides**																	

tr|A1DZF0|A1DZF0_ARAHY

back to list

| Protein Coverage | Supporting Peptides |

Protein Coverage:

1 MAKLLELSFC FCFLVLGASS ISFRQQPEEN ACQFQRLNAQ RPDNRIESEG GYIETWNPNN QEFECAGVAL SRLVLRRNAL

81 RRPFYSNAPQ EIFIQQGRGY FGLIFPGCPS TYEEPAQQGR RYQSQRPPRR LQEEDQSQQQ QDSHQKVHRF NEGDLIAVPT

161 GVAFWLYNDH DTDVVAVSLT DTNNNDNQLD QFPRRFNLAG NHEQEFLRYQ QQSRQSRRRS LPLSPYSPQP GQEDREFSPQ

241 GQHGRRERAG QEQENEGGNI FSGFTSEFLA QAFQVDDRQI VQNLRGENES EEQGAIVTVK GGLRILSPDR KSPDEEEEYD

379 d 397 d

321 EDEYAEEERQ QDRRRGRGSR GSGNGIEETI CTATVKKNIG RNRSPDIYNP QAGSLK**TANE LNLLILRWLG LSAEYGNLYR**

d d

401 NALFVPHYNT NAHSIIYALR GRAHVQVVDS NGNRVYDEEL QEGHVLVVPQ NFAVAGKSQS ENFEYVAFKT DSRPSIANLA

481 GENSFIDNLP EEVVANSYGL PREQARQLKN NNPFKFFVPP FQQSPRAVA

d Deamidation (NQ) (+0.98)

Supporting Peptides:

Peptide	Uniq	-10lgP	Mass	Length	ppm	m/z	z	RT	Fraction	Scan	Source File	Area Digest 9. traka	#Feature	#Feature Digest 9. traka	Start	End	PTM
R.WLGLSAEYGNLYR.N	N	85.61	1540.7673	13	-4.2	771.3913	2	33.40	31	1730	OB4059.raw	1.41E5	1	1	388	400	
R.WLGLSAEYGN(+.98)LYR.N	N	73.48	1541.7513	13	-6.7	771.8814	2	33.83	31	1759	OB4059.raw	7.08E3	1	1	388	400	Deamidation (NQ)
K.TANELNLLILR.W	Y	44.38	1268.7452	11	-3.1	635.3809	2	34.02	31	1772	OB4059.raw	1.16E4	1	1	377	387	
K.TAN(+.98)ELNLLILR.W	Y	37.10	1269.7292	11	-4.5	635.8720	2	34.37	31	1792	OB4059.raw	9.79E3	1	1	377	387	Deamidation (NQ)
K.TANELN(+.98)LLILR.W	Y	35.88	1269.7292	11	-4.5	635.8720	2	34.37	31	1802	OB4059.raw	9.79E3	1	1	377	387	
**total 5 peptides**																	

tr|B5TYU1|B5TYU1_ARAHY

back to list

| **Protein Coverage** | **Supporting Peptides** |

Protein Coverage:

1 MAKLLELSFC FCFLVLGASS ISFRQQPEEN ACQFQRLNAQ RPDNRIESEG GYIETWNPNN QEFECAGVAL SRLVLRRNAL

81 RRPFYSNAPQ EIFIQQGRGY FGLIFPGCPS TYEEPAQQGR RYQSQRPPRR LQEEDQSQQQ QDSHQKVHRF NEGDLIAVPT

161 GVAFWLYNDH DTDVVAVSLT DTNNNDNQLD QFPRRFNLAG NHEQEFLRYQ QQSRQSRRRS LPYSPYSPQS QPRQEEREFS

241 PRGQHSRRER AGQEEENEGG NIFSGFTPEF LAQAFQVDDR QIVQNLRGEN ESEEQGAIVT VRGGLRILSP DRKRGADEEE

321 EYDEDEYEYD EEDRRRGRGS RGSGNGIEET ICTATVKKNI GRNRSPDIYN PQAGSLK**TAN ELNLLILRWL GLSAEYGNLY**

401 **R**NALFVPHYN TNAHSIIYAL RGRAHVQVVD SNGNRVYDEE LQEGHVLVVP QNFAVAGKSQ SDNFEYVAFK TDSRPSIANL

481 AGENSVIDNL PEEVVANSYG LPREQARQLK NNNPFKFFVP PSQQSPRAVA

d Deamidation (NQ) (+0.98)

Supporting Peptides:

Peptide	Uniq	-10lgP	Mass	Length	ppm	m/z	z	RT	Fraction	Scan	Source File	Area Digest 9. traka	#Feature	#Feature Digest 9. traka	Start	End	PTM
R.WLGLSAEYGNLYR.N	N	85.61	1540.7673	13	-4.2	771.3913	2	33.40	31	1730	OB4059.raw	1.41E5	1	1	389	401	
R.WLGLSAEYGN(+.98)LYR.N	N	73.48	1541.7513	13	-6.7	771.8814	2	33.83	31	1759	OB4059.raw	7.08E3	1	1	389	401	Deamidation (NQ)
K.TANELNLLILR.W	Y	44.38	1268.7452	11	-3.1	635.3809	2	34.02	31	1772	OB4059.raw	1.16E4	1	1	378	388	
K.TAN(+.98)ELNLLILR.W	Y	37.10	1269.7292	11	-4.5	635.8720	2	34.37	31	1792	OB4059.raw	9.79E3	1	1	378	388	Deamidation (NQ)
K.TANELN(+.98)LLILR.W	Y	35.88	1269.7292	11	-4.5	635.8720	2	34.37	31	1802	OB4059.raw	9.79E3	1	1	378	388	
**total 5 peptides**																	

tr|Q5I6T2|Q5I6T2_ARAHY

back to list

| Protein Coverage | Supporting Peptides |

Protein Coverage:

d Deamidation (NQ) (+0.98)

```
  1 MAKLLELSFC FCFLVLGASS ISFRQQPEEN ACQFQRLNAQ RPDNRIESEG GYIETWNPNN QEFECAGVAL SRLVLRRNAL
 81 RRPFYSNAPQ EIFIQQGRGY FGLIFPGCPS TYEEPAQQGR RSQSQRPPRR LQGEDQSQQQ QDSHQKVHRF DEGDLIAVPT
161 GVAFWLYNDH DTDVVAVSLT DTNNNDNQLD QFPRRFNLAG NHEQEFLRYQ QQSRQSRRRS LPYSPYSPQS QPRQEEREFS
241 PRGQHSRRER AGQEEENEGG NIFSGFTPEF LEQAFQVDDR QIVQNLRGEN ESEEEGAIVT VRGGLRILSP DRKRGADEEE
321 EYDEDEYEYD EEDRRRGRGS RGRGNGIEET ICTASVKKNI GRNRSPDIYN PQAGSLKTAN DLNLLILRWL GLSAEYGNLY
401 RNALFVPHYN TNAHSIIYAL RGRAHVQVVD SNGNRVYDEE LQEGHVLVVP QNFAVAGKSQ SDNFEYVAFK TDSRPSIANL
481 AGENSVIDNL PEEVVANSYG LQREQARQQL KNNNPFKFFV PPSQQSPRAV A
```

380 d, 398 d

Supporting Peptides:

Peptide	Uniq	-10lgP	Mass	Length	ppm	m/z	z	RT	Fraction	Scan	Source File	Area Digest 9. traka	#Feature	#Feature Digest 9. traka	Start	End	PTM
R.WLGLSAEYGNLYR.N	N	85.61	1540.7673	13	-4.2	771.3913	2	33.40	31	1730	OB4059.raw	1.41E5	1	1	389	401	
R.WLGLSAEYGN(+.98)LYR.N	N	73.48	1541.7513	13	-6.7	771.8814	2	33.83	31	1759	OB4059.raw	7.08E3	1	1	389	401	Deamidation (NQ)
K.TAN(+.98)DLNLLILR.W	Y	43.03	1255.7136	11	-2.1	628.8657	2	34.08	31	1766	OB4059.raw	1.12E4	1	1	378	388	Deamidation (NQ)
**total 3 peptides**																	

tr|Q9FZ11|Q9FZ11_ARAHY

back to list

| Protein Coverage | Supporting Peptides |

Protein Coverage:

d Deamidation (NQ) (+0.98)

1 MIRGRLALSV CFCFLVLGAS SISFRQQPEE NACQFQRLNA QRPDNRLESE GGYIETWNPN NQEFECAGVA LSRLVLRRNA
81 LRRPFYSNAP QEIFIQQGRG YFGLIFPGCP STYEEPAQQG RRHQSQRAPR RFEGEDQSQQ QQQDSHQKVR RFDEGDLIAV
161 PTGVALWMFN DHDTDVVAVS LTDTNNNDNQ LDQFPRRFNL AGNHEQEFLR YQQQSRRRSL PYSPYSPQSQ PRQEEREFSP
241 RGQHSRRERA GQEEENEGGN IFSGFTPEFL AQAFQVDDRQ IVQNLRGENE SEEEGAIVTV KGGLRILSPD RKRGADEEEE
321 YDEDEYEYDE EDRRRGRGSR GRGNGIEETI CTASVKKNIG RNRSPDIYNP QAGSLK**TAND LNLLILRWLG LSAEYGNLYR**
401 NALFVPHYNT NAHSIIYALR GRAHVQVVDS NGNRVYDEEL QEGHVLVVPQ NFAVAGKSQS DNFEYVAFKT DSRPNIANFA
481 GENSIIDNLP EEVVANSYGL PREQARQLKN NNPFKFFVPP SQQSLRAVA

Supporting Peptides:

Peptide	Uniq	-10lgP	Mass	Length	ppm	m/z	z	RT	Fraction	Scan	Source File	Area Digest 9. traka	#Feature	#Feature Digest 9. traka	Start	End	PTM
R.WLGLSAEYGNLYR.N	N	85.61	1540.7673	13	-4.2	771.3913	2	33.40	31	1730	OB4059.raw	1.41E5	1	1	388	400	
R.WLGLSAEYGN(+.98)LYR.N	N	73.48	1541.7513	13	-6.7	771.8814	2	33.83	31	1759	OB4059.raw	7.08E3	1	1	388	400	Deamidation (NQ)
K.TAN(+.98)DLNLLILR.W	Y	43.03	1255.7136	11	-2.1	628.8657	2	34.08	31	1766	OB4059.raw	1.12E4	1	1	377	387	Deamidation (NQ)
**total 3 peptides**																	

tr|Q647H3|Q647H3_ARAHY

back to list

| Protein Coverage | Supporting Peptides |

Protein Coverage:

1 MAKLLALSVC FCFLVLGASS ISFRQQPEEN ACQFQRLNAQ RPDNRLESEG GYIETWNPNN QEFECAGVAL SRLVLRRNAL

81 RRPFYSNAPQ EIFIQQGRGY FGLIFPGCPS TYEEPAQQGR RHQSQRAPRR FEGEDQSQQQ QQDSHQKVRR FDEGDLIAVP

161 TGVALWMYND HDTDVVAVSL TDTNNNDNQL DQFPRRFNLA GNHEQEFLRY QQQSRRRSLP YSPYSPQSQP RQEEREFSPR

241 GQHSRRERAG QEQENEGGNI FSGFTPEFLA QAFQVDDRQI LQNLRGENES DEQGAIVTVR GGLRILSPDR KRRQQYERPD

321 EEEEYDEDEY EYDEEERQQD RRRGRGSRGR GNGIEETICT ASVKKNIGRN RSPDIYNPQA GSLK**TANDLN LLILRWLGLS**

401 **AEYGNLYR**NA LFVPHYNTNA HSIIYALRGR AHVQVVDSNG NRVYDEELQE GHVLVVPQNF AVAGKSQSDN FEYVAFKTDS

481 RPSIANLAGE NSIIDNLPEE VVANSYGLPR EQARQLKNNN PFKFFVPPSQ QSLGAVA

d Deamidation (NQ) (+0.98)

Supporting Peptides:

Peptide	Uniq	-10lgP	Mass	Length	ppm	m/z	z	RT	Fraction	Scan	Source File	Area Digest 9. traka	#Feature	#Feature Digest 9. traka	Start	End	PTM
R.WLGLSAEYGNLYR.N	N	85.61	1540.7673	13	-4.2	771.3913	2	33.40	31	1730	OB4059.raw	1.41E5	1	1	396	408	
R.WLGLSAEYGN(+.98)LYR.N	N	73.48	1541.7513	13	-6.7	771.8814	2	33.83	31	1759	OB4059.raw	7.08E3	1	1	396	408	Deamidation (NQ)
K.TAN(+.98)DLNLLILR.W	Y	43.03	1255.7136	11	-2.1	628.8657	2	34.08	31	1766	OB4059.raw	1.12E4	1	1	385	395	Deamidation (NQ)
**total 3 peptides**																	

tr|Q6IWG5|Q6IWG5_ARAHY

back to list

| Protein Coverage | Supporting Peptides |

Protein Coverage:

1 KLLALSLCFC VLVLGASSVT FRQGGEENEC QFQRLNAQRP DNRIESEGGY IETWNPNNQE FQCAGVALSR TVLRRNALRR

81 PFYSNAPLEI YVQQGSGYFG LIFPGCPSTY EEPAQEGRRY QSQKPSRRFQ VGQDDPSQQQ QDSHQKVHRF DEGDLIAVPT

161 GVAFWMYNDE DTDVVTVTLS DTSSIHNQLD QFPRRFYLAG NQEQEFLRYQ QQQGSRPHYR QISPRVRGDE QENEGSNIFS

241 GFAQEFLQHA FQVDRQTVEN LRGENEREEQ GAIVTVKGGL RILSPDEEDE SSRSPPSRRE EFDEDRSRPQ QRGKYDENRR

374 d

321 GYKNGIEETI CSASVKKNLG R**SSNPDIYNP QAGSLRSVNE LDLPILGWLG LSAQHGTIYR** NAMFVPHYTL NAHTIVVALN

d

401 GRAHVQVVDS NGNRVYDEEL QEGHVLVVPQ NFAVAAK**AQS ENYEYLAFK**T DSRPSIANLA GENSIIDNLP EEVVANSYRL

481 PREQARQLKN NNPFKFFVPP FDHQSMREVA

d Deamidation (NQ) (+0.98)

Supporting Peptides:

Peptide	Uniq	-10lgP	Mass	Length	ppm	m/z	z	RT	Fraction	Scan	Source File	Area Digest 9. traka	#Feature	#Feature Digest 9. traka	Start	End	PTM
K.AQSENYEYLAFK.T	Y	64.69	1461.6776	12	-3.7	731.8468	2	30.30	31	1547	OB4059.raw	2.43E3	1	1	438	449	
R.SSNPDIYNPQAGSLR.S	Y	56.79	1617.7747	15	-4.0	809.8951	2	27.47	31	1372	OB4059.raw	0	0	0	342	356	
R.SVNELDLPILGWLGLSAQHGTIYR.N	Y	38.97	2651.4070	24	-2.9	884.8112	3	38.34	31	2039	OB4059.raw	2.98E4	1	1	357	380	
R.SVN(+.98)ELDLPILGWLGLSAQHGTIYR.N	Y	37.03	2652.3911	24	-4.5	885.1378	3	38.64	31	2070	OB4059.raw	7.39E4	1	1	357	380	
R.SVNELDLPILGWLGLSAQ(+.98)HGTIYR.N	Y	34.08	2652.3911	24	-4.5	885.1378	3	38.64	31	2055	OB4059.raw	7.39E4	1	1	357	380	Deamidation (NQ)
**total 5 peptides**																	

tr|Q0GM57|Q0GM57_ARAHY

back to list

| Protein Coverage | Supporting Peptides |

Protein Coverage:

d Deamidation (NQ) (+0.98)

```
  1 MAKLLALSLC FCVLVLGASS VTFRQGGEEN ECQFQRLNAQ RPDNRIESEG GYIETWNPNN QEFQCAGVAL SRTVLRRNAL
 81 RRPFYSNAPL EIYVQQGSGY FGLIFPGCPS TYEEPAQEGR RYQSQKPSRR FQVGQDDPSQ QQQDSHQKVH RFDEGDLIAV
161 PTGVAFWMYN DEDTDVVTVT LSDTSSIHNQ LDQFPRRFYL AGNQEQEFLR YQQQQGSRPH YRQISPRVRG DEQENEGSNI
241 FSGFAQEFLQ HAFQVDRQTV ENLRGENERE EQGAIVTVKG GLRILSPDEE DESSRSPPSR REEFDEDRSR PQQRGKYDEN
                                                                    376
                                                                    d
321 RRGYKNGIEE TICSASVKKN LGRSSNPDIY NPQAGSLRSV NELDLPILGW LGLSAQHGTI YRNAMFVPHY TLNAHTIVVA
                                                                    d
401 LNGRAHVQVV DSNGNRVYDE ELQEGHVLVV PQNFAVAAKA QSENYEYLAF KTDSRPSIAN LAGENSIIDN LPEEVVANSY
481 RLPREQARQL KNNNPFKFFV PPFDHQSMRE VA
```

Supporting Peptides:

Peptide	Uniq	-10lgP	Mass	Length	ppm	m/z	z	RT	Fraction	Scan	Source File	Area Digest 9. traka	#Feature	#Feature Digest 9. traka	Start	End	PTM
K.AQSENYEYLAFK.T	Y	64.69	1461.6776	12	-3.7	731.8468	2	30.30	31	1547	OB4059.raw	2.43E3	1	1	440	451	
R.SSNPDIYNPQAGSLR.S	Y	56.79	1617.7747	15	-4.0	809.8951	2	27.47	31	1372	OB4059.raw	0	0	0	344	358	
R.SVNELDLPILGWLGLSAQHGTIYR.N	Y	38.97	2651.4070	24	-2.9	884.8112	3	38.34	31	2039	OB4059.raw	2.98E4	1	1	359	382	
R.SVN(+.98)ELDLPILGWLGLSAQHGTIYR.N	Y	37.03	2652.3911	24	-4.5	885.1378	3	38.64	31	2070	OB4059.raw	7.39E4	1	1	359	382	
R.SVNELDLPILGWLGLSAQ(+.98)HGTIYR.N	Y	34.08	2652.3911	24	-4.5	885.1378	3	38.64	31	2055	OB4059.raw	7.39E4	1	1	359	382	Deamidation (NQ)
**total 5 peptides**																	

tr|E5G077|E5G077_ARAHY

back to list

| Protein Coverage | Supporting Peptides |

Protein Coverage:

1 MAKLLALSLC FCVLVLGASS VTFRQGGEEN ECQFQRLNAQ RPDNRIESEG GYIETWNPNN QEFQCAGVAL SRTVLRRNAL

d Deamidation (NQ) (+0.98)

81 RRPFYSNAPL EIYVQQGSGY FGLIFPGCPS TYEEPAQEGR RYQSQKPSRR FQVGQDDPSQ QQQDSHQKVH RFDEGDLIAV

161 PTGVAFWMYN DEDTDVVTVT LSDTSSIHNQ LDQFPRRFYL AGNQEQEFLR YQQQQGSRPH YRQISPRVRG DEQENEGSNI

241 FSGFAQEFLQ HAFQVDRQTV ENLRGENERE EQGAIVTVKG GLRILSPDEE DESSRSPPNR REEFDEDRSR PQQRGKYDEN

376 d

321 RRGYKNGIEE TICSASVKKN LGR**SSNPDIY NPQAGSLRSV NELDLPILGW LGLSAQHGTI YR**NAMFVPHY TLNAHTIVVA

d

401 LNGRAHVQVV DSNGNRVYDE ELQEGHVLVV PQNFAVAAK**A QSENYEYLAF K**TDSRPSIAN QAGENSIIDN LPEEVVANSY

481 RLPREQARQL KNNNPFKFFV PPFDHQSMRE VA

Supporting Peptides:

Peptide	Uniq	-10lgP	Mass	Length	ppm	m/z	z	RT	Fraction	Scan	Source File	Area Digest 9. traka	#Feature	#Feature Digest 9. traka	Start	End	PTM
K.AQSENYEYLAFK.T	Y	64.69	1461.6776	12	-3.7	731.8468	2	30.30	31	1547	OB4059.raw	2.43E3	1	1	440	451	
R.SSNPDIYNPQAGSLR.S	Y	56.79	1617.7747	15	-4.0	809.8951	2	27.47	31	1372	OB4059.raw	0	0	0	344	358	
R.SVNELDLPILGWLGLSAQHGTIYR.N	Y	38.97	2651.4070	24	-2.9	884.8112	3	38.34	31	2039	OB4059.raw	2.98E4	1	1	359	382	
R.SVN(+.98)ELDLPILGWLGLSAQHGTIYR.N	Y	37.03	2652.3911	24	-4.5	885.1378	3	38.64	31	2070	OB4059.raw	7.39E4	1	1	359	382	
R.SVNELDLPILGWLGLSAQ(+.98)HGTIYR.N	Y	34.08	2652.3911	24	-4.5	885.1378	3	38.64	31	2055	OB4059.raw	7.39E4	1	1	359	382	Deamidation (NQ)
**total 5 peptides**																	

tr|Q38711|Q38711_ARAHY

back to list

| Protein Coverage | Supporting Peptides |

Protein Coverage:

1 KPFCVFLTFF LLLAASKKVN SAETVSFNFN SFSEGNPAIN FQGDVTVLSN GNIQLTNLNK VNSVGRVLYA MPVRIWSSAT

81 GNVASFLTSF SFEMKDIKDY DPADGIIFFI APEDTQIPAG SIGGGTLGVS DTKGAGHFVG VEFDTYSNSE YNDPPTDHVG

161 IDVNSVDSVK TVPWNSVSGA VVQVTVIYDS STKTLSVAVT NENGDITTIA QVVDLKAKLP ERVK**FGFSAS GSLGGR**QIHL

241 IR**SWSFTSTL ITTTR**RSIDN NEKKIMNMAT A

Supporting Peptides:

Peptide	Uniq	-10lgP	Mass	Length	ppm	m/z	z	RT	Fraction	Scan	Source File	Area Digest 9. traka	#Feature	#Feature Digest 9. traka	Start	End	PTM
R.SWSFTSTLITTTR.R	Y	72.93	1499.7620	13	-4.0	750.8888	2	33.21	31	1724	OB4059.raw	9.52E3	1	1	243	255	
K.FGFSASGSLGGR.Q	Y	45.21	1141.5515	12	-4.6	571.7831	2	29.28	31	1480	OB4059.raw	4.73E3	1	1	225	236	
**total 2 peptides**																	

P02872|LECG_ARAHY

back to list

| Protein Coverage | Supporting Peptides |

Protein Coverage:

1 MKPFCVFLTF FLLLAASSKK VDSAETVSFN FNSFSEGNPA INFQGDVTVL SNGNIQLTNL NKVNSVGRVL YAMPVRIWSS

81 ATGNVASFLT SFSFEMKDIK DYDPADGIIF FIAPEDTQIP AGSIGGGTLG VSDTKGAGHF VGVEFDTYSN SEYNDPPTDH

161 VGIDVNSVDS VKTVPWNSVS GAVVKVTVIY DSSTKTLSVA VTNDNGDITT IAQVVDLKAK LPERVK**FGFS ASGSLGGR**QI

241 HLIR**SWSFTS TLITTTR**RSI DNNEKKIMNM ASA

Supporting Peptides:

Peptide	Uniq	-10lgP	Mass	Length	ppm	m/z	z	RT	Fraction	Scan	Source File	Area Digest 9. traka	#Feature	#Feature Digest 9. traka	Start	End	PTM
R.SWSFTSTLITTTR.R	Y	72.93	1499.7620	13	-4.0	750.8888	2	33.21	31	1724	OB4059.raw	9.52E3	1	1	245	257	
K.FGFSASGSLGGR.Q	Y	45.21	1141.5515	12	-4.6	571.7831	2	29.28	31	1480	OB4059.raw	4.73E3	1	1	227	238	
**total 2 peptides**																	

tr|B4UWA1|B4UWA1_ARAHY

back to list

| Protein Coverage | Supporting Peptides |

Protein Coverage:

45 o

1 MAETAQPNAE LLEWPKKDKR RFLHAVYRVG DLDR**TIKFYT EAFGMR**LLRK RDVPEEKYAN AFLGFGPEHS NFVVELTYNY

144 d 153 c 156 o

81 GVTSYDIGTG FGHFAIATPD VYKLVEDIRA KGGNVTREPG PVK**GGSSVIA FVKDPDGYTF ELIQRASTPE PLCQVMLR**VG

161 DLERSIKFYE KALGMKVVKK VDRPEYKYTL LCLDMP

c Carbamidomethylation (+57.02)
d Deamidation (NQ) (+0.98)
o Oxidation (M) (+15.99)

Supporting Peptides:

Peptide	Uniq	-10lgP	Mass	Length	ppm	m/z	z	RT	Fraction	Scan	Source File	Area Digest 9. traka	#Feature	#Feature Digest 9. traka	Start	End	PTM
K.GGSSVIAFVKDPDGYTFELIQ(+.98)R.A	Y	52.51	2399.2009	22	-1.4	800.7435	3	34.11	31	1777	OB4059.raw	0	0	0	124	145	Deamidation (NQ)
K.GGSSVIAFVKDPDGYTFELIQR.A	Y	48.94	2398.2168	22	-3.6	800.4138	3	33.88	31	1767	OB4059.raw	1.06E4	1	1	124	145	
R.ASTPEPLC(+57.02)QVM(+15.99)LR.V	Y	36.28	1516.7378	13	-3.7	759.3770	2	29.28	31	1482	OB4059.raw	6.16E3	1	1	146	158	Carbamidomethylation; Oxidation (M)
R.TIKFYTEAFGM(+15.99)R.L	Y	34.27	1478.7228	12	-3.6	493.9154	3	29.67	31	1501	OB4059.raw	3.94E3	1	1	35	46	Oxidation (M)
K.FYTEAFGM(+15.99)R.L	Y	27.53	1136.4961	9	-6.5	569.2543	2	28.50	31	1431	OB4059.raw	3.76E3	1	1	38	46	Oxidation (M)
**total 5 peptides**																	

Peptide List

Prepared with PEAKS ™ (bioinfor.com)

Summary

1. Notes Gastric Digest Raw peanut Band #A 33-35 kDa (PTM508)

2. Result Statistics

Figure 1. False discovery rate (FDR) curve. X axis is the number of peptide-spectrum matches (PSM) being kept. Y axis is the corresponding FDR.

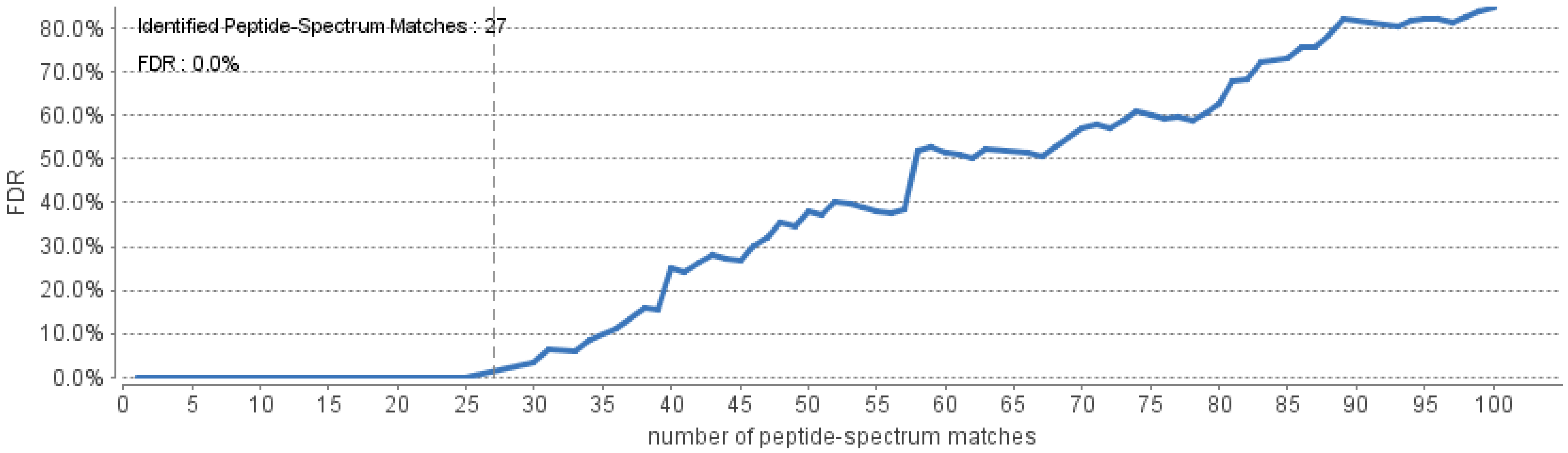

Figure 2. PSM score distribution. **(a)** Distribution of PEAKS peptide score; **(b)** Scatterplot of PEAKS peptide score versus precursor mass error.

(a)

(b)

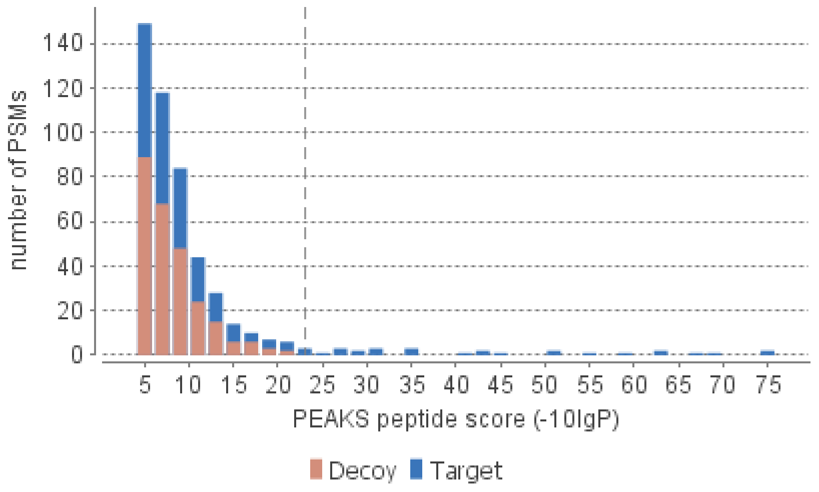

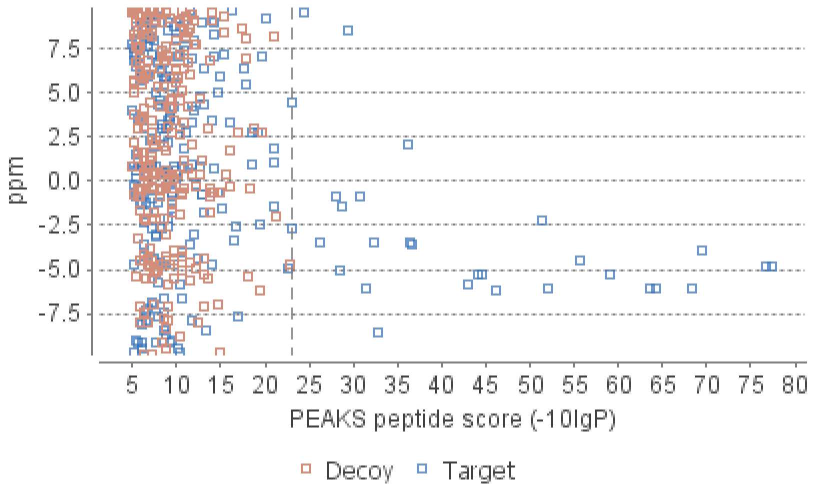

Figure 3. Distribution of peptide feature detection. **(a)** Feature m/z distribution; **(b)** Feature RT distribution

(a)

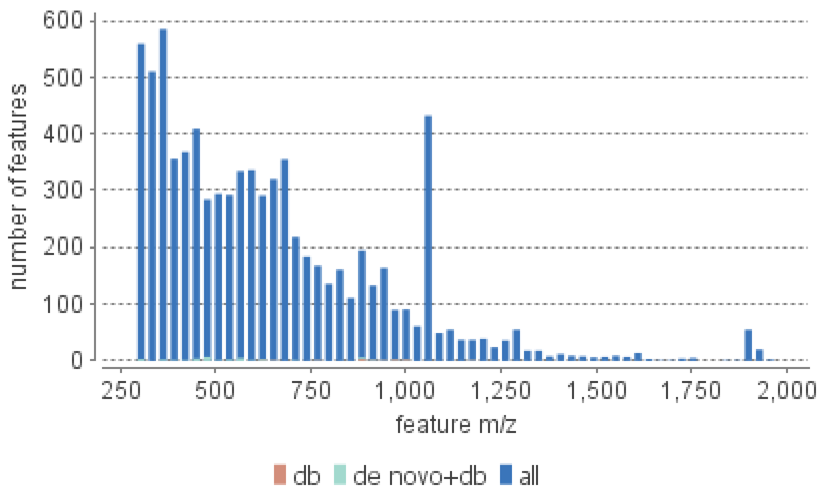

(b)

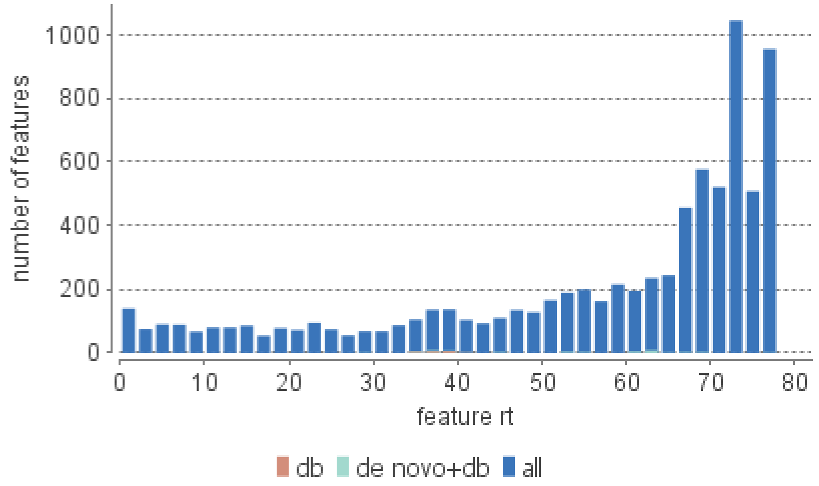

Figure 4. Distribution of identified peptide features. **(a)** Feature abundance distribution **(b)** *De novo* sequencing validation.

(a)

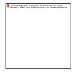

(b)

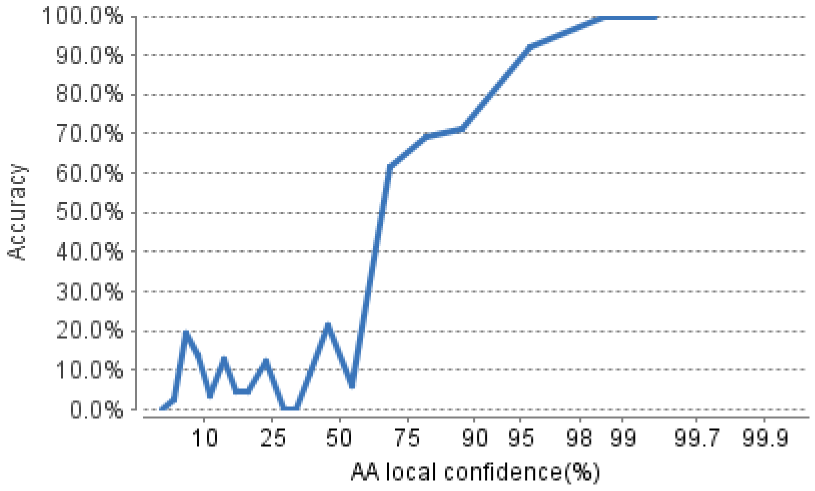

Table 1. Statistics of data.

# of MS scans	2748
# of MS/MS scans	1677
# of Features	7978
# of Chimera scans	237

Table 2. Result filtration parameters.

Peptide -10lgP	≥23.1
PTM Ascore	≥20
Protein -10lgP	≥20
Proteins unique peptides	≥1
De novo ALC Score	≥50%

Table 4. PTM profile.

**Name**	**ΔMass**	**Position**	**#PSM**	**-10lgP**	**Abundance**	**AScore**
Deamidation	.98	NQ	11	36.66		19.78
Carbamidomethyl	57.02	C	1	51.38	1.29E3	1000.00

Table 3. Statistics of filtered result.

Peptide-Spectrum Matches	27
Peptide sequences	19
Protein groups	3
Proteins	9
Proteins (#Unique Peptides)	3 (>2); 0 (=2); 6 (=1);
FDR (Peptide-Spectrum Matches)	0.0%
FDR (Peptide Sequences)	0.0%
FDR (Protein Group)	0.0%
De Novo Only Spectra	46
# of identified Features	47
# of identified MS/MS scans	26

3. Experiment Control

Figure 5. Precursor mass error of peptide-spectrum matches (PSM) in filtered result. **(a)** Distribution of precursor mass error in ppm; **(b)** Scatterplot of precursor m/z versus precursor mass error in ppm.

(a)

(b)

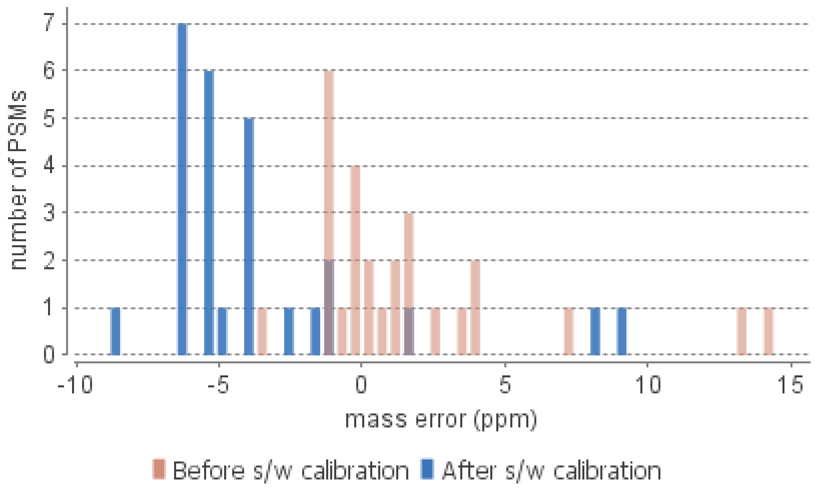

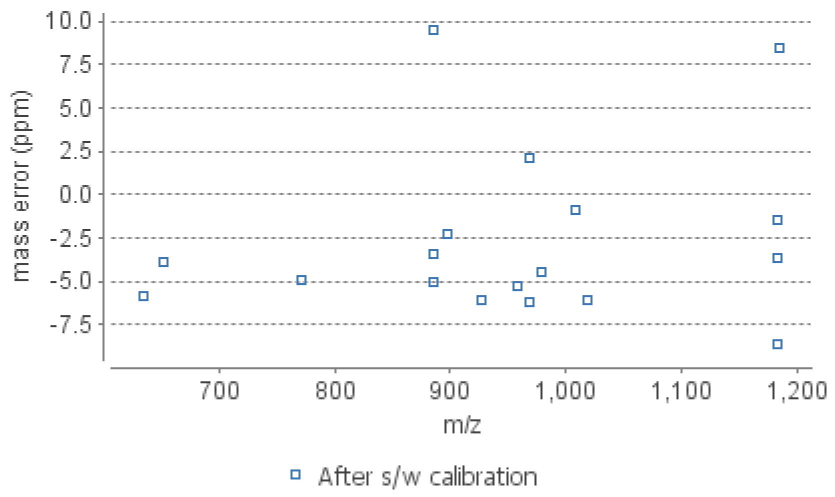

Table 5. Number of identified peptides in each sample by the number of missed cleavages

Missed Cleavages	0	1	2	3	4+
Digest traka A na 35-38 kDa	12	7	0	0	0

4. Other Information

Table 6. Search parameters.

Search Engine Name: PEAKS
Parent Mass Error Tolerance: 10.0 ppm
Fragment Mass Error Tolerance: 0.5 Da
Precursor Mass Search Type: monoisotopic
Enzyme: Trypsin
Max Missed Cleavages: 2
Digest Mode: Unspecific

Table 7. Instrument parameters.

Fractions: OB4066.raw
Ion Source: ESI(nano-spray)
Fragmentation Mode: CID, CAD(y and b ions)
MS Scan Mode: FT-ICR/Orbitrap
MS/MS Scan Mode: Linear Ion Trap

Fixed Modifications:
  Carbamidomethylation: 57.02
Variable Modifications:
  Deamidation (NQ): 0.98
  Oxidation (M): 15.99
  Hydroxylation Pro: 15.99
  Acetylation (K): 42.01
  Acetylation (Protein N-term): 42.01
  Acetylation (N-term): 42.01
  Amidation: -0.98
  Beta-methylthiolation: 45.99
  and 305 more...
Max Variable PTM Per Peptide: 5
Database: Uniprot_Peanut-3818_Jul18
Taxon: All
Contaminant Database: contaminantsMQ_mar19
Searched Entry: 1723
FDR Estimation: Enabled
De novo score (ALC%) threshold: 15
Peptide hit threshold (-10logP): 30.0
Peaks run ID: 502
Merge Options: no merge
Precursor Options: corrected
Charge Options: no correction
Filter Charge: 2 - 8
Process: true
Associate chimera: yes

Protein List

Protein Accession Contains:

Protein Description Contains:

Peptide Sample Area >=

Protein Ptm Contains:

Protein Group	Protein ID	Accession	-10lgP	Coverage (%)	Coverage (%) Digest traka A na 35-38 kDa	Area Digest traka A na 35-38 kDa	#Peptides	#Unique	#Spec Digest traka A na 35-38 kDa	PTM	Avg. Mass	Description
**1**	20574	tr|A1DZF0|A1DZF0_ARAHY	118.86	12	12	2.14E4	4	3	9	Y	60375	Arachin 6 OS=Arachis hypogaea OX=3818 PE=2 SV=1
**1**	20580	tr|Q647H4|Q647H4_ARAHY	118.86	11	11	2.14E4	4	3	9	Y	61506	Arachin Ahy-1 OS=Arachis hypogaea OX=3818 PE=2 SV=1
**1**	20591	tr|Q6T2T4|Q6T2T4_ARAHY	118.86	11	11	2.14E4	4	3	9	Y	61499	Storage protein OS=Arachis hypogaea OX=3818 PE=2 SV=1
**4**	20576	tr|Q9FZ11|Q9FZ11_ARAHY	93.24	5	5	5.49E3	2	1	3	N	60449	Gly1 OS=Arachis hypogaea OX=3818 GN=Gly1 PE=2 SV=1
**4**	20581	tr|Q647H3|Q647H3_ARAHY	93.24	4	4	5.49E3	2	1	3	N	61532	Arachin Ahy-2 OS=Arachis hypogaea OX=3818 PE=2 SV=1
**4**	20579	tr|Q5I6T2|Q5I6T2_ARAHY	93.24	5	5	5.49E3	2	1	3	N	60736	Arachin Ahy-4 OS=Arachis hypogaea OX=3818 PE=2 SV=1
**3**	20593	tr|Q6IWG5|Q6IWG5_ARAHY	36.32	5	5	9.07E4	1	1	5	Y	58061	Glycinin (Fragment) OS=Arachis hypogaea OX=3818 PE=2 SV=1
**3**	20595	tr|E5G077|E5G077_ARAHY	36.32	5	5	9.07E4	1	1	5	Y	58305	Ara h 3 allergen OS=Arachis hypogaea OX=3818 GN=ara h 3 PE=3 SV=1
**3**	20594	tr|Q0GM57|Q0GM57_ARAHY	36.32	5	5	9.07E4	1	1	5	Y	58263	Iso-Ara h3 OS=Arachis hypogaea OX=3818 PE=2 SV=1
**total 9 proteins**												

tr|A1DZF0|A1DZF0_ARAHY
back to list

| **Protein Coverage** | **Supporting Peptides** |

Protein Coverage:

d Deamidation (NQ) (+0.98)

```
  1 MAKLLELSFC FCFLVLGASS ISFRQQPEEN ACQFQRLNAQ RPDNRIESEG GYIETWNPNN QEFECAGVAL SRLVLRRNAL
 81 RRPFYSNAPQ EIFIQQGRGY FGLIFPGCPS TYEEPAQQGR RYQSQRPPRR LQEEDQSQQQ QDSHQKVHRF NEGDLIAVPT
161 GVAFWLYNDH DTDVVAVSLT DTNNNDNQLD QFPRRFNLAG NHEQEFLRYQ QQSRQSRRRS LPLSPYSPQP GQEDREFSPQ
241 GQHGRRERAG QEQENEGGNI FSGFTSEFLA QAFQVDDRQI VQNLRGENES EEQGAIVTVK GGLRILSPDR KSPDEEEEYD
321 EDEYAEEERQ QDRRRGRGSR GSGNGIEETI CTATVKKNIG RNRSPDIYNP QAGSLKTANE LNLLILRWLG LSAEYGNLYR
401 NALFVPHYNT NAHSIIYALR GRAHVQVVDS NGNRVYDEEL QEGHVLVVPQ NFAVAGKSQS ENFEYVAFKT DSRPSIANLA
481 GENSFIDNLP EEVVANSYGL PREQARQLKN NNPFKFFVPP FQQSPRAVA
```

496 d

d

Supporting Peptides:

Peptide	Uniq	-10lgP	Mass	Length	ppm	m/z	z	RT	Fraction	Scan	Source File	Area Digest traka A na 35-38 kDa	#Feature	#Feature Digest traka A na 35-38 kDa	Start	End	PTM
R.WLGLSAEYGNLYR.N	N	77.49	1540.7673	13	-4.9	771.3912	2	35.25	38	1765	OB4066.raw	1.85E4	1	1	388	400	
K.TANELNLLILR.W	Y	42.91	1268.7452	11	-5.9	635.3795	2	35.58	38	1789	OB4066.raw	2.19E3	1	1	377	387	
K.TDSRPSIAN(+.98)LAGEN(+.98)SFIDNLPEEVVANSYGLPR.E	Y	36.66	3546.7114	33	-3.6	1183.2463	3	37.90	38	1933	OB4066.raw	0	0	0	470	502	
K.TDSRPSIANLAGENSFIDN(+.98)LPEEVVAN(+.98)SYGLPR.E	Y	32.84	3546.7114	33	-8.6	1183.2405	3	38.24	38	1958	OB4066.raw	0	0	0	470	502	Deamidation (NQ)
K.TDSRPSIANLAGEN(+.98)SFIDN(+.98)LPEEVVANSYGLPREQAR.Q	Y	30.74	4030.9507	37	-0.8	1008.7494	4	36.78	38	1853	OB4066.raw	8.63E3	1	1	470	506	
K.TDSRPSIAN(+.98)LAGEN(+.98)SFIDN(+.98)LPEEVVANSYGLPR.E	Y	29.42	3547.6953	33	8.5	1183.5886	3	37.69	38	1923	OB4066.raw	1.92E3	1	1	470	502	
K.TDSRPSIANLAGENSFIDN(+.98)LPEEVVANSYGLPR.E	Y	28.62	3545.7273	33	-1.4	1182.9209	3	37.75	38	1923	OB4066.raw	0	0	0	470	502	
K.TDSRPSIANLAGENSFIDN(+.98)LPEEVVANSYGLPREQ(+.98)AR.Q	Y	28.04	4030.9507	37	-0.8	1008.7494	4	36.78	38	1864	OB4066.raw	8.63E3	1	1	470	506	
**total 8 peptides**																	

tr|Q647H4|Q647H4_ARAHY

back to list

| Protein Coverage | Supporting Peptides |

Protein Coverage:

```
  1 MGKLLALSVC FCFLVLGASS ISFRQQPEEN ACQFQRLNAQ RPDNRIESEG GYIETWNPNN QEFECAGVAL SRLVLRRNAL
 81 RRPFYSNAPQ EIFIQQGRGY FGLIFPGCPS TYEEPAQQGR RHQSQRPPRR FQGQDQSQQQ QDSHQKVHRF DEGDLIAVPT
161 GVAFWMYNDH DTDVVAVSLT DTNNNDNQLD QFPRRFNLAG NHEQEFLRYQ QQSRRRSLPY SPYSPQTQPK QEDREFSPRG
241 QHGRRERAGQ EQENEGGNIF SGFTPEFLAQ AFQVDDRQIL QNLRGENESD EQGAIVTVRG GLRILSPDRK RRQQYERPDE
321 EEEYDEDEYE YDEEERQHDR RRGRGSRGSG NGIEETICTA SFKKNIGRNR SPDIYNPQAG SLKTANELNL LILRWLGLSA
401 EYGNLYRNAL FVPHYNTNAH SIIYALRGRA HVQVVDSNGD RVFDEELQEG HVLVVPQNFA VAGKSQSENF EYVAFKTDSR
481 PSIANLAGEN SFIDNLPEEV VANSYGLPRE QARQLKNNNP FKFFVPPSEQ SLRAVA
```

503 d

d Deamidation (NQ) (+0.98)

Supporting Peptides:

Peptide	Uniq	-10lgP	Mass	Length	ppm	m/z	z	RT	Fraction	Scan	Source File	Area Digest traka A na 35-38 kDa	#Feature	#Feature Digest traka A na 35-38 kDa	Start	End	PTM
R.WLGLSAEYGNLYR.N	N	77.49	1540.7673	13	-4.9	771.3912	2	35.25	38	1765	OB4066.raw	1.85E4	1	1	395	407	
K.TANELNLLILR.W	Y	42.91	1268.7452	11	-5.9	635.3795	2	35.58	38	1789	OB4066.raw	2.19E3	1	1	384	394	
K.TDSRPSIAN(+.98)LAGEN(+.98)SFIDNLPEEVVANSYGLPR.E	Y	36.66	3546.7114	33	-3.6	1183.2463	3	37.90	38	1933	OB4066.raw	0	0	0	477	509	
K.TDSRPSIANLAGENSFIDN(+.98)LPEEVVAN(+.98)SYGLPR.E	Y	32.84	3546.7114	33	-8.6	1183.2405	3	38.24	38	1958	OB4066.raw	0	0	0	477	509	Deamidation (NQ)
K.TDSRPSIANLAGEN(+.98)SFIDN(+.98)LPEEVVANSYGLPREQAR.Q	Y	30.74	4030.9507	37	-0.8	1008.7494	4	36.78	38	1853	OB4066.raw	8.63E3	1	1	477	513	
K.TDSRPSIAN(+.98)LAGEN(+.98)SFIDN(+.98)LPEEVVANSYGLPR.E	Y	29.42	3547.6953	33	8.5	1183.5886	3	37.69	38	1923	OB4066.raw	1.92E3	1	1	477	509	
K.TDSRPSIANLAGENSFIDN(+.98)LPEEVVANSYGLPR.E	Y	28.62	3545.7273	33	-1.4	1182.9209	3	37.75	38	1923	OB4066.raw	0	0	0	477	509	
K.TDSRPSIANLAGENSFIDN(+.98)LPEEVVANSYGLPREQ(+.98)AR.Q	Y	28.04	4030.9507	37	-0.8	1008.7494	4	36.78	38	1864	OB4066.raw	8.63E3	1	1	477	513	
**total 8 peptides**																	

tr|Q6T2T4|Q6T2T4_ARAHY

back to list

| Protein Coverage | Supporting Peptides |

Protein Coverage:

d Deamidation (NQ) (+0.98)

```
  1 MGKLLALSVC FCFLVLGASS ISFRQQPEEN ACQFQRLNAQ RPDNRIESEG GYIETWNPNN QEFECAGVAL SRLVLRRNAL
 81 RRPFYSNAPQ EIFIQQGRAY FGLIFLGCPS TYEEPAQQGR RHQSQRPPRR FQGQDQSQQQ QDSHQKVHRF DEGDLIAVPT
161 GVAFWMYNDH DTDVVAVSLT DTNNNDNQLD QFPRRFNLAG NHEQEFLRYQ QQSRRRSLPY SPYSPQTQPK QEDREFSPRG
241 QHGRRERAGQ EQENEGGNIF SGFTPEFLAQ AFQVDDRQIL QNLRGENESD EQGAIVTVRG GLRILSPDRK KRQQYERPDE
321 EEEYDEDEYE YDEEERQQDR RRGRGSRGSG NGIEETICTA SFKKNIGRNR SPDIYNPQAG SLKTANELNL LILRWLGLSA
401 EYGNLYRNAL FVPHYNTNAH SIIYALRGRA HVQVVDSNGD RVFDEELQEG HVLVVPQNFA VAGKSQSENF EYVAFKTDSR
                                           503
                                            d
481 PSIANLAGEN SFIDNLPEEV VANSYGLPRE QARQLKNNNP FKFFVPPSEQ SLRAVA
                                            d
```

Supporting Peptides:

Peptide	Uniq	-10lgP	Mass	Length	ppm	m/z	z	RT	Fraction	Scan	Source File	Area Digest traka A na 35-38 kDa	#Feature	#Feature Digest traka A na 35-38 kDa	Start	End	PTM
R.WLGLSAEYGNLYR.N	N	77.49	1540.7673	13	-4.9	771.3912	2	35.25	38	1765	OB4066.raw	1.85E4	1	1	395	407	
K.TANELNLLILR.W	Y	42.91	1268.7452	11	-5.9	635.3795	2	35.58	38	1789	OB4066.raw	2.19E3	1	1	384	394	
K.TDSRPSIAN(+.98)LAGEN(+.98)SFIDNLPEEVVANSYGLPR.E	Y	36.66	3546.7114	33	-3.6	1183.2463	3	37.90	38	1933	OB4066.raw	0	0	0	477	509	
K.TDSRPSIANLAGENSFIDN(+.98)LPEEVVAN(+.98)SYGLPR.E	Y	32.84	3546.7114	33	-8.6	1183.2405	3	38.24	38	1958	OB4066.raw	0	0	0	477	509	Deamidation (NQ)
K.TDSRPSIANLAGEN(+.98)SFIDN(+.98)LPEEVVANSYGLPREQAR.Q	Y	30.74	4030.9507	37	-0.8	1008.7494	4	36.78	38	1853	OB4066.raw	8.63E3	1	1	477	513	
K.TDSRPSIAN(+.98)LAGEN(+.98)SFIDN(+.98)LPEEVVANSYGLPR.E	Y	29.42	3547.6953	33	8.5	1183.5886	3	37.69	38	1923	OB4066.raw	1.92E3	1	1	477	509	
K.TDSRPSIANLAGENSFIDN(+.98)LPEEVVANSYGLPR.E	Y	28.62	3545.7273	33	-1.4	1182.9209	3	37.75	38	1923	OB4066.raw	0	0	0	477	509	
K.TDSRPSIANLAGENSFIDN(+.98)LPEEVVANSYGLPREQ(+.98)AR.Q	Y	28.04	4030.9507	37	-0.8	1008.7494	4	36.78	38	1864	OB4066.raw	8.63E3	1	1	477	513	
**total 8 peptides**																	

tr|Q9FZ11|Q9FZ11_ARAHY

back to list

| Protein Coverage | Supporting Peptides |

Protein Coverage:

```
  1 MIRGRLALSV CFCFLVLGAS SISFRQQPEE NACQFQRLNA QRPDNRLESE GGYIETWNPN NQEFECAGVA LSRLVLRRNA
 81 LRRPFYSNAP QEIFIQQGRG YFGLIFPGCP STYEEPAQQG RRHQSQRAPR RFEGEDQSQQ QQQDSHQKVR RFDEGDLIAV
161 PTGVALWMFN DHDTDVVAVS LTDTNNNDNQ LDQFPRRFNL AGNHEQEFLR YQQQSRRRSL PYSPYSPQSQ PRQEEREFSP
241 RGQHSRRERA GQEEENEGGN IFSGFTPEFL AQAFQVDDRQ IVQNLRGENE SEEEGAIVTV KGGLRILSPD RKRGADEEEE
321 YDEDEYEYDE EDRRRGRGSR GRGNGIEETI CTASVKKNIG RNRSPDIYNP QAGSLKTAND LNLLILRWLG LSAEYGNLYR
401 NALFVPHYNT NAHSIIYALR GRAHVQVVDS NGNRVYDEEL QEGHVLVVPQ NFAVAGKSQS DNFEYVAFKT DSRPNIANFA
481 GENSIIDNLP EEVVANSYGL PREQARQLKN NNPFKFFVPP SQQSLRAVA
```

Supporting Peptides:

Peptide	Uniq	-10lgP	Mass	Length	ppm	m/z	z	RT	Fraction	Scan	Source File	Area Digest traka A na 35-38 kDa	#Feature	#Feature Digest traka A na 35-38 kDa	Start	End	PTM
R.WLGLSAEYGNLYR.N	N	77.49	1540.7673	13	-4.9	771.3912	2	35.25	38	1765	OB4066.raw	1.85E4	1	1	388	400	
K.TANDLNLLILRWLGLSAEYGNLYR.N	Y	31.49	2777.4863	24	-6.0	926.8353	3	41.00	38	2133	OB4066.raw	5.49E3	1	1	377	400	
**total 2 peptides**																	

tr|Q647H3|Q647H3_ARAHY

back to list

| Protein Coverage | Supporting Peptides |

Protein Coverage:

1 MAKLLALSVC FCFLVLGASS ISFRQQPEEN ACQFQRLNAQ RPDNRLESEG GYIETWNPNN QEFECAGVAL SRLVLRRNAL
81 RRPFYSNAPQ EIFIQQGRGY FGLIFPGCPS TYEEPAQQGR RHQSQRAPRR FEGEDQSQQQ QQDSHQKVRR FDEGDLIAVP
161 TGVALWMYND HDTDVVAVSL TDTNNNDNQL DQFPRRFNLA GNHEQEFLRY QQQSRRRSLP YSPYSPQSQP RQEEREFSPR
241 GQHSRRERAG QEQENEGGNI FSGFTPEFLA QAFQVDDRQI LQNLRGENES DEQGAIVTVR GGLRILSPDR KRRQQYERPD
321 EEEEYDEDEY EYDEEERQQD RRRGRGSRGR GNGIEETICT ASVKKNIGRN RSPDIYNPQA GSLK**TANDLN LLILRWLGLS**

401 **AEYGNLYR**NA LFVPHYNTNA HSIIYALRGR AHVQVVDSNG NRVYDEELQE GHVLVVPQNF AVAGKSQSDN FEYVAFKTDS

481 RPSIANLAGE NSIIDNLPEE VVANSYGLPR EQARQLKNNN PFKFFVPPSQ QSLGAVA

Supporting Peptides:

Peptide	Uniq	-10lgP	Mass	Length	ppm	m/z	z	RT	Fraction	Scan	Source File	Area Digest traka A na 35-38 kDa	#Feature	#Feature Digest traka A na 35-38 kDa	Start	End	PTM
R.WLGLSAEYGNLYR.N	N	77.49	1540.7673	13	-4.9	771.3912	2	35.25	38	1765	OB4066.raw	1.85E4	1	1	396	408	
K.TANDLNLLILRWLGLSAEYGNLYR.N	Y	31.49	2777.4863	24	-6.0	926.8353	3	41.00	38	2133	OB4066.raw	5.49E3	1	1	385	408	
**total 2 peptides**																	

tr|Q5I6T2|Q5I6T2_ARAHY

back to list

| Protein Coverage | Supporting Peptides |

Protein Coverage:

1 MAKLLELSFC FCFLVLGASS ISFRQQPEEN ACQFQRLNAQ RPDNRIESEG GYIETWNPNN QEFECAGVAL SRLVLRRNAL
81 RRPFYSNAPQ EIFIQQGRGY FGLIFPGCPS TYEEPAQQGR RSQSQRPPRR LQGEDQSQQQ QDSHQKVHRF DEGDLIAVPT
161 GVAFWLYNDH DTDVVAVSLT DTNNNDNQLD QFPRRFNLAG NHEQEFLRYQ QQSRQSRRRS LPYSPYSPQS QPRQEEREFS
241 PRGQHSRRER AGQEEENEGG NIFSGFTPEF LEQAFQVDDR QIVQNLRGEN ESEEEGAIVT VRGGLRILSP DRKRGADEEE
321 EYDEDEYEYD EEDRRRGRGS RGRGNGIEET ICTASVKKNI GRNRSPDIYN PQAGSLK**TAN DLNLLILRWL GLSAEYGNLY**

401 **R**NALFVPHYN TNAHSIIYAL RGRAHVQVVD SNGNRVYDEE LQEGHVLVVP QNFAVAGKSQ SDNFEYVAFK TDSRPSIANL

481 AGENSVIDNL PEEVVANSYG LQREQARQQL KNNNPFKFFV PPSQQSPRAV A

Supporting Peptides:

Peptide	Uniq	-10lgP	Mass	Length	ppm	m/z	z	RT	Fraction	Scan	Source File	Area Digest traka A na 35-38 kDa	#Feature	#Feature Digest traka A na 35-38 kDa	Start	End	PTM
R.WLGLSAEYGNLYR.N	N	77.49	1540.7673	13	-4.9	771.3912	2	35.25	38	1765	OB4066.raw	1.85E4	1	1	389	401	
K.TANDLNLLILRWLGLSAEYGNLYR.N	Y	31.49	2777.4863	24	-6.0	926.8353	3	41.00	38	2133	OB4066.raw	5.49E3	1	1	378	401	
**total 2 peptides**																	

tr|Q6IWG5|Q6IWG5_ARAHY
back to list

| Protein Coverage | Supporting Peptides |

Protein Coverage:

d Deamidation (NQ) (+0.98)

```
  1  KLLALSLCFC VLVLGASSVT FRQGGEENEC QFQRLNAQRP DNRIESEGGY IETWNPNNQE FQCAGVALSR TVLRRNALRR
 81  PFYSNAPLEI YVQQGSGYFG LIFPGCPSTY EEPAQEGRRY QSQKPSRRFQ VGQDDPSQQQ QDSHQKVHRF DEGDLIAVPT
161  GVAFWMYNDE DTDVVTVTLS DTSSIHNQLD QFPRRFYLAG NQEQEFLRYQ QQQGSRPHYR QISPRVRGDE QENEGSNIFS
241  GFAQEFLQHA FQVDRQTVEN LRGENEREEQ GAIVTVKGGL RILSPDEEDE SSRSPPSRRE EFDEDRSRPQ QRGKYDENRR
                                                   359            374
                                                   d              d
321  GYKNGIEETI CSASVKKNLG RSSNPDIYNP QAGSLRSVNE LDLPILGWLG LSAQHGTIYR NAMFVPHYTL NAHTIVVALN
401  GRAHVQVVDS NGNRVYDEEL QEGHVLVVPQ NFAVAAKAQS ENYEYLAFKT DSRPSIANLA GENSIIDNLP EEVVANSYRL
481  PREQARQLKN NNPFKFFVPP FDHQSMREVA
```

Supporting Peptides:

Peptide	Uniq	-10lgP	Mass	Length	ppm	m/z	z	RT	Fraction	Scan	Source File	Area Digest traka A na 35-38 kDa	#Feature	#Feature Digest traka A na 35-38 kDa	Start	End	PTM
R.SVNELDLPILGWLGLSAQ(+.98)HGTIYR.N	Y	36.32	2652.3911	24	-3.5	885.1392	3	39.89	38	2063	OB4066.raw	4.58E4	1	1	357	380	Deamidation (NQ)
R.SVNELDLPILGWLGLSAQHGTIYR.N	Y	28.55	2651.4070	24	-5.0	884.8098	3	39.58	38	2034	OB4066.raw	4.49E4	1	1	357	380	
R.SVN(+.98)ELDLPILGWLGLSAQ(+.98)HGTIYR.N	Y	24.35	2653.3750	24	9.5	885.4786	3	40.34	38	2091	OB4066.raw	0	0	0	357	380	Deamidation (NQ)
**total 3 peptides**																	

tr|E5G077|E5G077_ARAHY
back to list

| **Protein Coverage** | **Supporting Peptides** |

Protein Coverage:

d Deamidation (NQ) (+0.98)

```
  1  MAKLLALSLC FCVLVLGASS VTFRQGGEEN ECQFQRLNAQ RPDNRIESEG GYIETWNPNN QEFQCAGVAL SRTVLRRNAL
 81  RRPFYSNAPL EIYVQQGSGY FGLIFPGCPS TYEEPAQEGR RYQSQKPSRR FQVGQDDPSQ QQQDSHQKVH RFDEGDLIAV
161  PTGVAFWMYN DEDTDVVTVT LSDTSSIHNQ LDQFPRRFYL AGNQEQEFLR YQQQQGSRPH YRQISPRVRG DEQENEGSNI
241  FSGFAQEFLQ HAFQVDRQTV ENLRGENERE EQGAIVTVKG GLRILSPDEE DESSRSPPNR REEFDEDRSR PQQRGKYDEN
                                                 361            376
                                                  d              d
321  RRGYKNGIEE TICSASVKKN LGRSSNPDIY NPQAGSLRSV NELDLPILGW LGLSAQHGTI YRNAMFVPHY TLNAHTIVVA
401  LNGRAHVQVV DSNGNRVYDE ELQEGHVLVV PQNFAVAAKA QSENYEYLAF KTDSRPSIAN QAGENSIIDN LPEEVVANSY
481  RLPREQARQL KNNNPFKFFV PPFDHQSMRE VA
```

Supporting Peptides:

Peptide	Uniq	-10lgP	Mass	Length	ppm	m/z	z	RT	Fraction	Scan	Source File	Area Digest traka A na 35-38 kDa	#Feature	#Feature Digest traka A na 35-38 kDa	Start	End	PTM
R.SVNELDLPILGWLGLSAQ(+.98)HGTIYR.N	Y	36.32	2652.3911	24	-3.5	885.1392	3	39.89	38	2063	OB4066.raw	4.58E4	1	1	359	382	Deamidation (NQ)
R.SVNELDLPILGWLGLSAQHGTIYR.N	Y	28.55	2651.4070	24	-5.0	884.8098	3	39.58	38	2034	OB4066.raw	4.49E4	1	1	359	382	
R.SVN(+.98)ELDLPILGWLGLSAQ(+.98)HGTIYR.N	Y	24.35	2653.3750	24	9.5	885.4786	3	40.34	38	2091	OB4066.raw	0	0	0	359	382	Deamidation (NQ)
**total 3 peptides**																	

tr|Q0GM57|Q0GM57_ARAHY

back to list

| **Protein Coverage** | **Supporting Peptides** |

Protein Coverage:

d Deamidation (NQ) (+0.98)

```
  1 MAKLLALSLC FCVLVLGASS VTFRQGGEEN ECQFQRLNAQ RPDNRIESEG GYIETWNPNN QEFQCAGVAL SRTVLRRNAL
 81 RRPFYSNAPL EIYVQQGSGY FGLIFPGCPS TYEEPAQEGR RYQSQKPSRR FQVGQDDPSQ QQQDSHQKVH RFDEGDLIAV
161 PTGVAFWMYN DEDTDVVTVT LSDTSSIHNQ LDQFPRRFYL AGNQEQEFLR YQQQQGSRPH YRQISPRVRG DEQENEGSNI
241 FSGFAQEFLQ HAFQVDRQTV ENLRGENERE EQGAIVTVKG GLRILSPDEE DESSRSPPSR REEFDEDRSR PQQRGKYDEN
                                                    361         376
                                                     d           d
321 RRGYKNGIEE TICSASVKKN LGRSSNPDIY NPQAGSLRSV NELDLPILGW LGLSAQHGTI YRNAMFVPHY TLNAHTIVVA
401 LNGRAHVQVV DSNGNRVYDE ELQEGHVLVV PQNFAVAAKA QSENYEYLAF KTDSRPSIAN LAGENSIIDN LPEEVVANSY
481 RLPREQARQL KNNNPFKFFV PPFDHQSMRE VA
```

Supporting Peptides:

Peptide	Uniq	-10lgP	Mass	Length	ppm	m/z	z	RT	Fraction	Scan	Source File	Area Digest traka A na 35-38 kDa	#Feature	#Feature Digest traka A na 35-38 kDa	Start	End	PTM
R.SVNELDLPILGWLGLSAQ(+.98)HGTIYR.N	Y	36.32	2652.3911	24	-3.5	885.1392	3	39.89	38	2063	OB4066.raw	4.58E4	1	1	359	382	Deamidation (NQ)
R.SVNELDLPILGWLGLSAQHGTIYR.N	Y	28.55	2651.4070	24	-5.0	884.8098	3	39.58	38	2034	OB4066.raw	4.49E4	1	1	359	382	
R.SVN(+.98)ELDLPILGWLGLSAQ(+.98)HGTIYR.N	Y	24.35	2653.3750	24	9.5	885.4786	3	40.34	38	2091	OB4066.raw	0	0	0	359	382	Deamidation (NQ)
**total 3 peptides**																	

Peptide List

Summary

1. Notes Gastric Digest Raw peanut Band #11 38-41 kDa (PTM507)

2. Result Statistics

Figure 1. False discovery rate (FDR) curve. X axis is the number of peptide-spectrum matches (PSM) being kept. Y axis is the corresponding FDR.

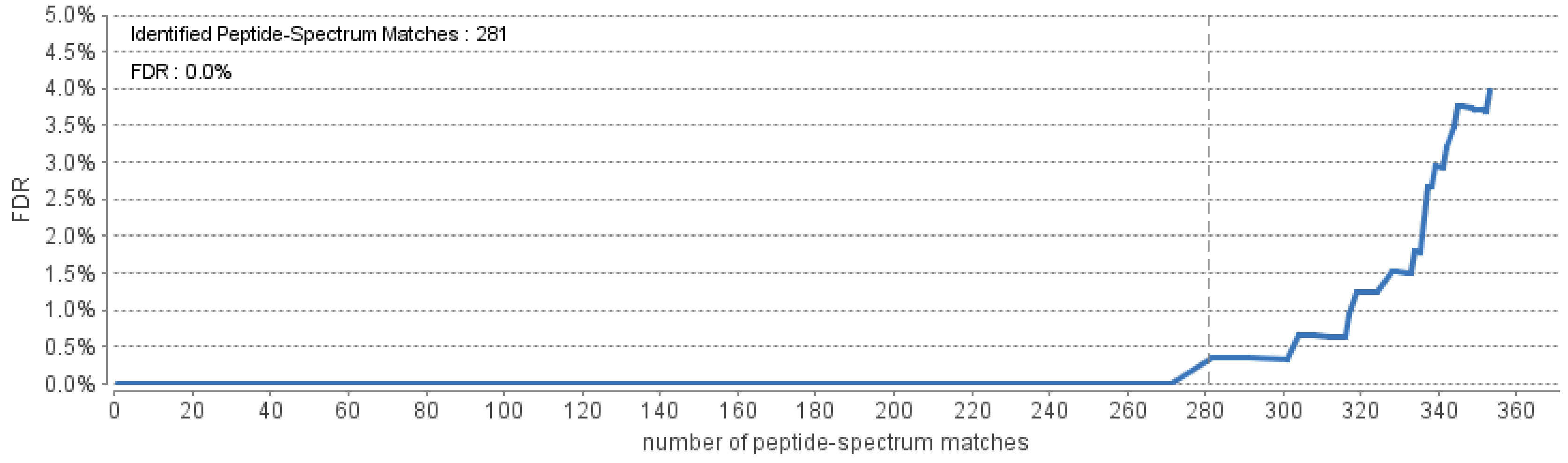

Figure 2. PSM score distribution. **(a)** Distribution of PEAKS peptide score; **(b)** Scatterplot of PEAKS peptide score versus precursor mass error.

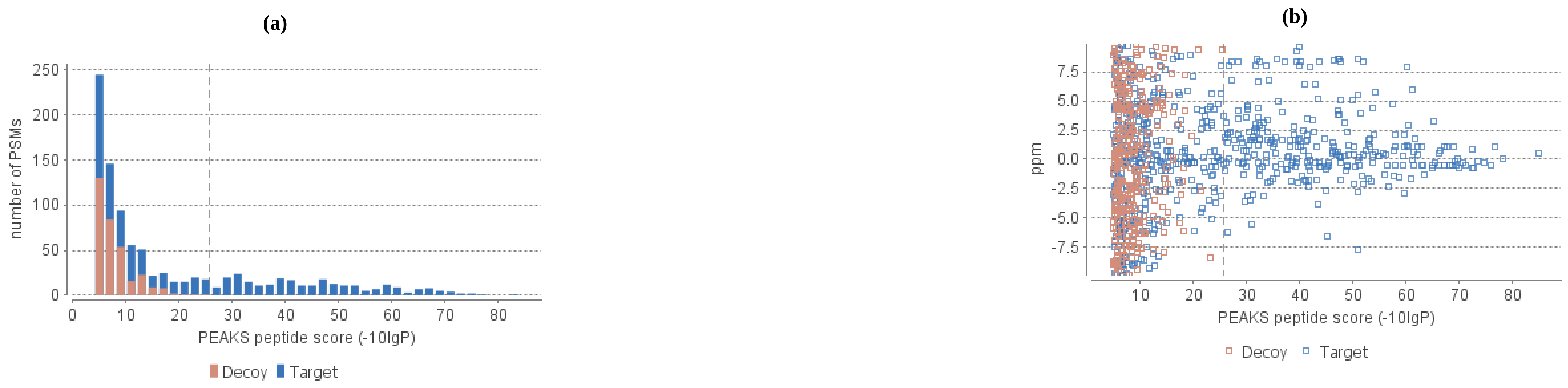

Figure 3. Distribution of peptide feature detection. **(a)** Feature m/z distribution; **(b)** Feature RT distribution

(a) (b)

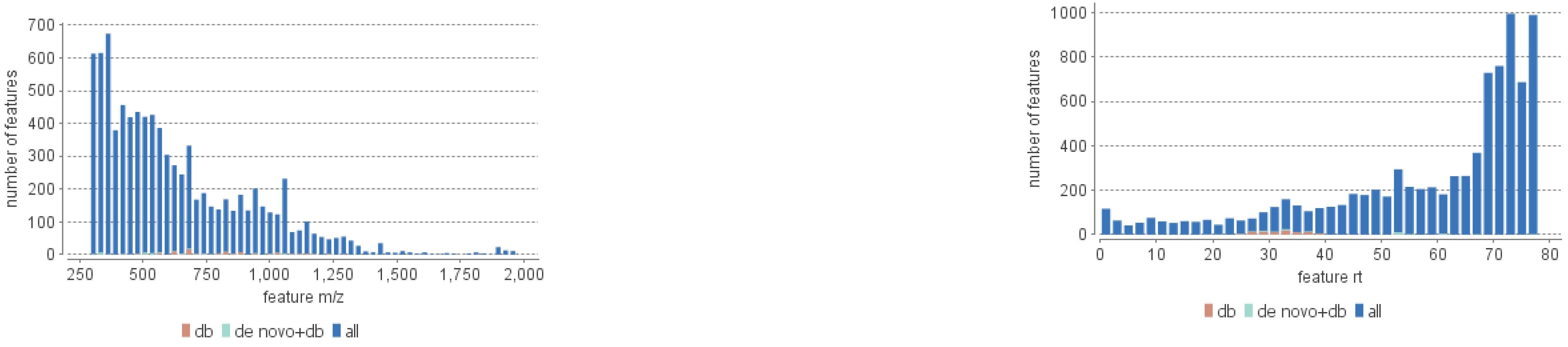

Figure 4. Distribution of identified peptide features. **(a)** Feature abundance distribution **(b)** *De novo* sequencing validation.

(a) (b)

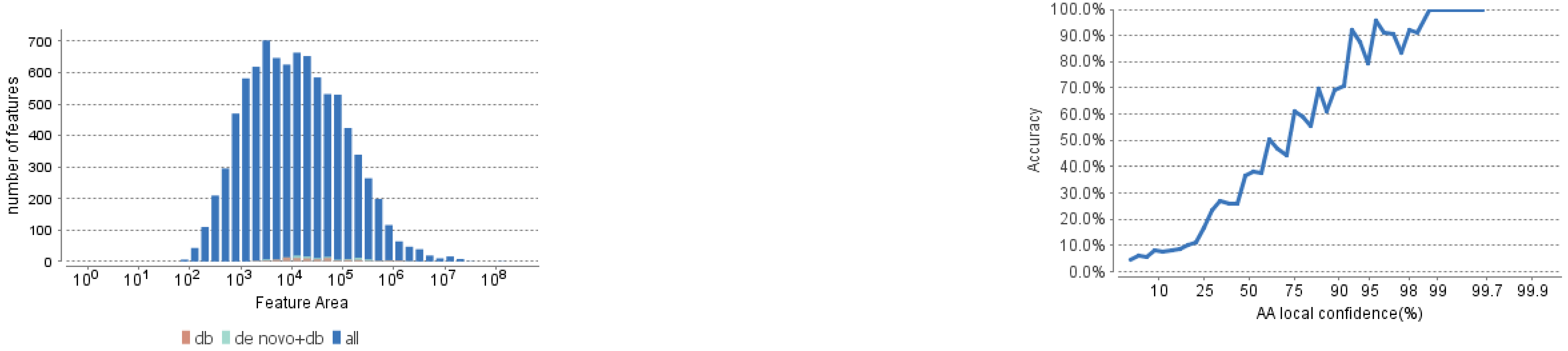

Table 1. Statistics of data.

# of MS scans	2371
# of MS/MS scans	2309
# of Features	8822
# of Chimera scans	382

Table 2. Result filtration parameters.

Peptide -10lgP	≥25.6

Table 4. PTM profile.

Name	ΔMass	Position	#PSM	-10lgP	Abundance	AScore
Deamidation	.98	NQ	219	76.60	1.18E5	9.34
Carbamidomethyl	57.02	C	98	76.60	1.18E5	1000.00
HydPro	15.99	P	22	61.12	4.97E3	59.53
Oxidation	15.99	M	10	47.71	3.54E3	1000.00

PTM Ascore	≥20
Protein -10lgP	≥20
Proteins unique peptides	≥1
De novo ALC Score	≥50%

Table 3. Statistics of filtered result.

Peptide-Spectrum Matches	281
Peptide sequences	157
Protein groups	6
Proteins	11
Proteins (#Unique Peptides)	9 (>2); 0 (=2); 2 (=1);
FDR (Peptide-Spectrum Matches)	0.0%
FDR (Peptide Sequences)	0.0%
FDR (Protein Group)	0.0%
De Novo Only Spectra	86
# of identified Features	132
# of identified MS/MS scans	251

Ethyl	28.03	E	2	34.53	7.07E3	28.11
Phosphorylation	79.97	T	1	26.69	6.76E3	11.06
Ethyl+Deamidated	29.02	Q	1	29.06	7.07E3	19.27
EtOH	44.03	K	1	26.69	5.42E4	91.60
Dimethylation(KR)	28.03	K	1	55.57	4E3	1000.00
Propionamide	71.04	C	1	25.86		1000.00

3. Experiment Control

Figure 5. Precursor mass error of peptide-spectrum matches (PSM) in filtered result. **(a)** Distribution of precursor mass error in ppm; **(b)** Scatterplot of precursor m/z versus precursor mass error in ppm.

(a)

(b)

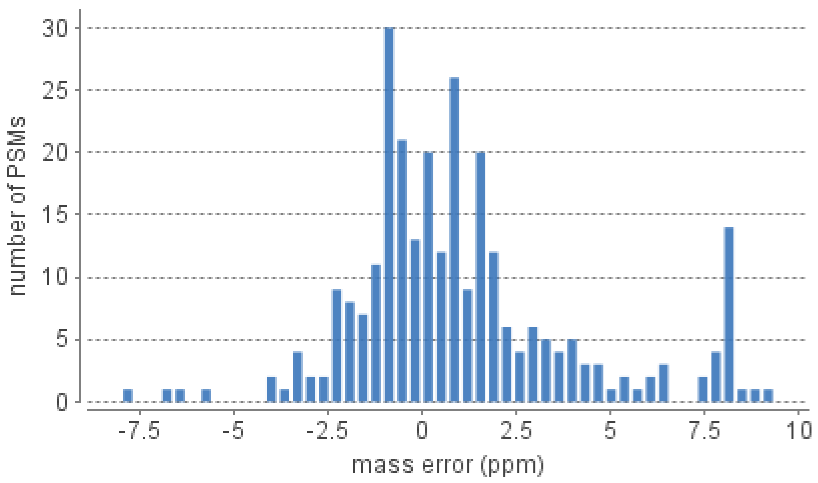

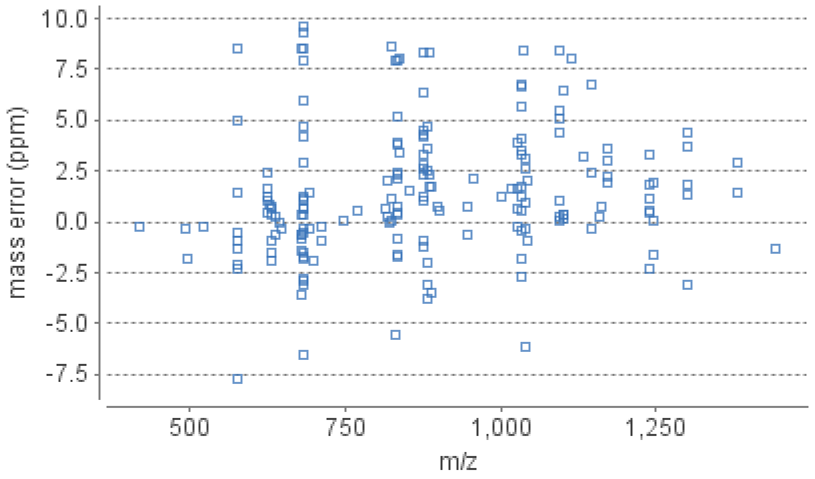

Table 5. Number of identified peptides in each sample by the number of missed cleavages

Missed Cleavages	0	1	2	3	4+
Digest 11 traka	57	89	11	0	0

4. Other Information

Table 6. Search parameters.

Search Engine Name: PEAKS
Parent Mass Error Tolerance: 10.0 ppm
Fragment Mass Error Tolerance: 0.5 Da
Precursor Mass Search Type: monoisotopic
Enzyme: Trypsin
Max Missed Cleavages: 2
Digest Mode: Unspecific
Fixed Modifications:
  Carbamidomethylation: 57.02
Variable Modifications:
  Deamidation (NQ): 0.98
  Oxidation (M): 15.99
  Hydroxylation Pro: 15.99
  Acetylation (K): 42.01
  Acetylation (Protein N-term): 42.01
  Acetylation (N-term): 42.01
  Amidation: -0.98
  Beta-methylthiolation: 45.99
  and 305 more...
Max Variable PTM Per Peptide: 5
Database: Uniprot_Peanut-3818_Jul18

Table 7. Instrument parameters.

Fractions: OB4061.raw
Ion Source: ESI(nano-spray)
Fragmentation Mode: CID, CAD(y and b ions)
MS Scan Mode: FT-ICR/Orbitrap
MS/MS Scan Mode: Linear Ion Trap

Taxon: All
Contaminant Database: contaminantsMQ_mar19
Searched Entry: 1723
FDR Estimation: Enabled
De novo score (ALC%) threshold: 15
Peptide hit threshold (-10logP): 30.0
Peaks run ID: 488
Merge Options: no merge
Precursor Options: corrected
Charge Options: no correction
Filter Charge: 2 - 8
Process: true
Associate chimera: yes

Protein List

Protein Accession Contains:
Protein Description Contains:
Peptide Sample Area >=
Protein Ptm Contains:

Protein Group	Protein ID	Accession	-10lgP	Coverage (%)	Coverage (%) Digest 11 traka	Area Digest 11 traka	#Peptides	#Unique	#Spec Digest 11 traka	PTM	Avg. Mass	Description
**2**	20576	tr|Q9FZ11|Q9FZ11_ARAHY	249.49	46	46	6.66E4	20	3	219	Y	60449	Gly1 OS=Arachis hypogaea OX=3818 GN=Gly1 PE=2 SV=1
**1**	20581	tr|Q647H3|Q647H3_ARAHY	245.95	45	45	1.9E5	19	3	216	Y	61532	Arachin Ahy-2 OS=Arachis hypogaea OX=3818 PE=2 SV=1
**6**	20849	tr|Q6PSU3|Q6PSU3_ARAHY	144.98	18	18	4.03E5	7	7	13	Y	66575	Conarachin (Fragment) OS=Arachis hypogaea OX=3818 PE=4 SV=1
**6**	20850	P43237|ALL11_ARAHY	144.98	17	17	4.03E5	7	7	13	Y	70283	Allergen Ara h 1, clone P17 OS=Arachis hypogaea OX=3818 PE=1 SV=1
**6**	20851	tr|B3IXL2|B3IXL2_ARAHY	144.98	17	17	4.03E5	7	7	13	Y	70283	Main allergen Ara h1 OS=Arachis hypogaea OX=3818 PE=2 SV=1
**5**	22697	tr|T2B9M0|T2B9M0_ARAHY	134.89	19	19	4.75E5	3	3	15	Y	38383	Fructose-bisphosphate aldolase OS=Arachis hypogaea OX=3818 PE=2 SV=1
**7**	20595	tr|E5G077|E5G077_ARAHY	93.15	16	16	8.13E5	4	3	14	Y	58305	Ara h 3 allergen OS=Arachis hypogaea OX=3818 GN=ara h 3 PE=3 SV=1
**7**	20594	tr|Q0GM57|Q0GM57_ARAHY	93.15	16	16	8.13E5	4	3	14	Y	58263	Iso-Ara h3 OS=Arachis hypogaea OX=3818 PE=2 SV=1
**7**	20593	tr|Q6IWG5|Q6IWG5_ARAHY	93.15	16	16	8.13E5	4	3	14	Y	58061	Glycinin (Fragment) OS=Arachis hypogaea OX=3818 PE=2 SV=1
**12**	21994	tr|A0A0A6ZDT0|A0A0A6ZDT0_ARAHY	61.73	8	8	6.8E3	1	1	1	N	20099	Glyceraldehyde-3-phosphate dehydrogenase C2 OS=Arachis hypogaea OX=3818 PE=4 SV=1
**12**	21405	tr|A0A0A6ZDP1|A0A0A6ZDP1_ARAHY	61.73	8	8	6.8E3	1	1	1	N	20101	Glyceraldehyde-3-phosphate dehydrogenase C2 OS=Arachis hypogaea OX=3818 PE=2 SV=1
**total 11 proteins**												

tr|Q9FZ11|Q9FZ11_ARAHY
back to list

| Protein Coverage | Supporting Peptides |

Protein Coverage:

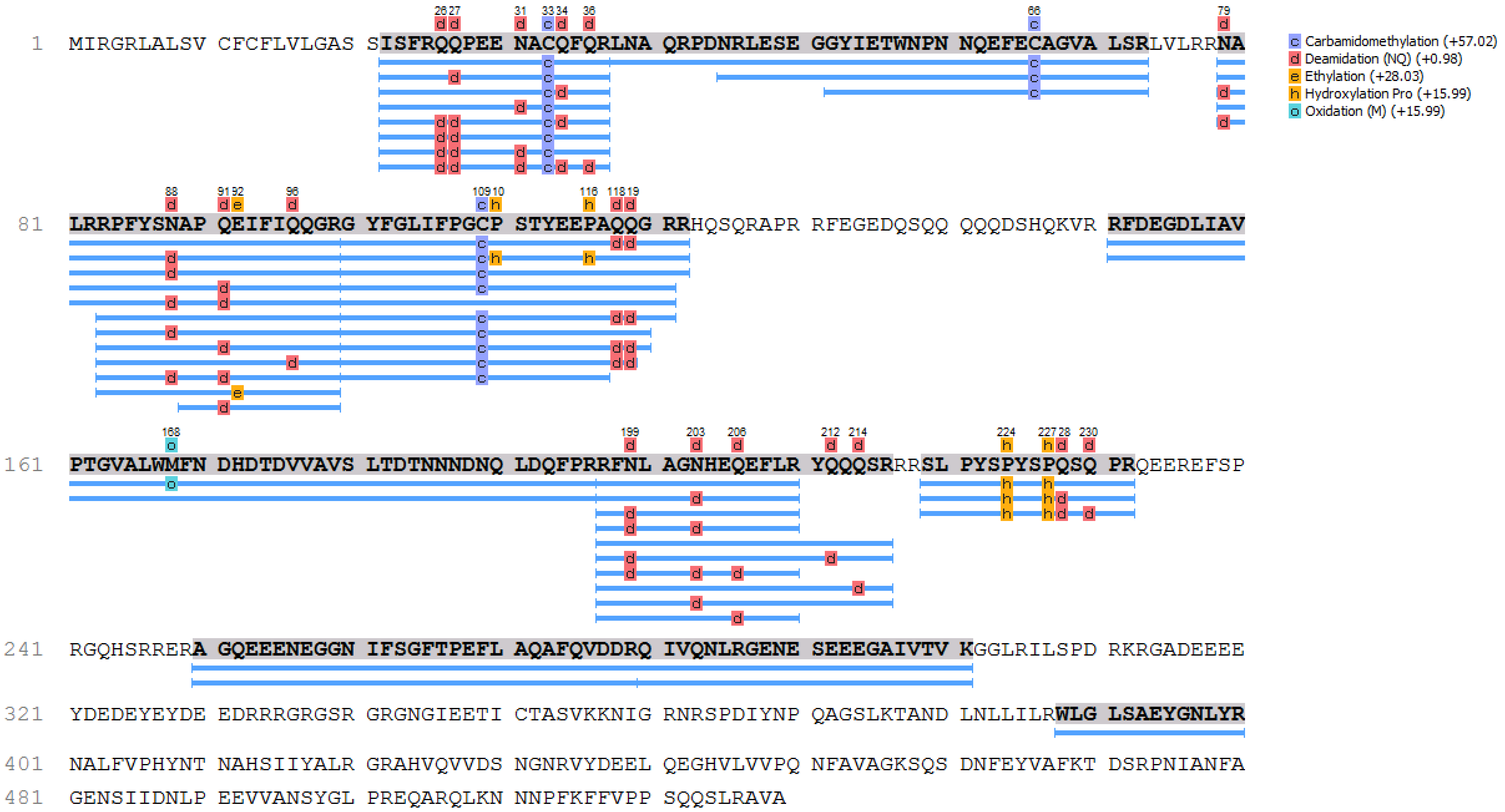

Supporting Peptides:

Peptide	Uniq	-10lgP	Mass	Length	ppm	m/z	z	RT	Fraction	Scan	Source File	Area Digest 11 traka	#Feature	#Feature Digest 11 traka	Start	End	PTM
R.WLGLSAEYGNLYR.N	N	85.02	1540.7673	13	0.5	771.3914	2	33.53	33	1880	OB4061.raw	6.78E4	1	1	388	400	
R.GYFGLIFPGC(+57.02)PSTYEEPAQQ(+.98)GR.R	N	76.60	2474.1211	22	1.1	1238.0692	2	35.30	33	1981	OB4061.raw	2.38E5	3	3	100	121	Carbamidomethylation
R.RFNLAGNHEQEFLR.Y	N	76.02	1729.8647	14	-0.6	577.6285	3	27.81	33	1524	OB4061.raw	2.56E5	2	2	197	210	
R.RPFYSNAPQEIFIQQGR.G	N	74.68	2050.0383	17	-0.3	684.3531	3	30.39	33	1681	OB4061.raw	7.21E5	2	2	83	99	
R.RPFYSNAPQ(+.98)EIFIQQGR.G	N	73.51	2051.0225	17	-0.2	1026.5183	2	30.94	33	1720	OB4061.raw	1.06E6	4	4	83	99	Deamidation (NQ)

Peptide	Uniq	-10lgP	Mass	Length	ppm	m/z	z	RT	Fraction	Scan	Source File	Area Digest 11 traka	#Feature	#Feature Digest 11 traka	Start	End	PTM
R.NALRRPFYSN(+.98)APQEIFIQQGR.G	N	72.57	2505.2876	21	-0.8	836.1025	3	31.01	33	1675	OB4061.raw	1.53E6	2	2	79	99	Deamidation (NQ)
R.GYFGLIFPGC(+57.02)PSTYEEPAQQGR.R	N	69.55	2473.1372	22	0.1	825.3864	3	35.12	33	1975	OB4061.raw	0	0	0	100	121	Carbamidomethylation
R.RPFYSN(+.98)APQEIFIQQGR.G	N	69.19	2051.0225	17	-0.2	1026.5183	2	30.94	33	1715	OB4061.raw	2.49E4	2	2	83	99	Deamidation (NQ)
R.RFNLAGN(+.98)HEQEFLR.Y	N	68.65	1730.8488	14	-0.6	577.9565	3	28.52	33	1578	OB4061.raw	3.38E5	1	1	197	210	Deamidation (NQ)
R.RPFYSNAPQEIFIQ(+.98)QGR.G	N	67.75	2051.0225	17	1.1	684.6822	3	30.94	33	1700	OB4061.raw	1.03E6	1	1	83	99	
R.GYFGLIFPGC(+57.02)PSTYEEPAQ(+.98)Q(+.98)GR.R	N	65.12	2475.1052	22	3.3	1238.5640	2	35.40	33	1993	OB4061.raw	0	0	0	100	121	Carbamidomethylation; Deamidation (NQ)
R.RFN(+.98)LAGN(+.98)HEQEFLR.Y	N	63.41	1731.8329	14	-1.4	578.2841	3	28.98	33	1589	OB4061.raw	1.14E5	1	1	197	210	Deamidation (NQ)
R.RFN(+.98)LAGNHEQEFLR.Y	N	62.87	1730.8488	14	-0.6	577.9565	3	28.52	33	1612	OB4061.raw	3.38E5	1	1	197	210	Deamidation (NQ)
R.AGQEEEN(+.98)EGGNIFSGFTPEFLAQAFQVDDR.Q	N	62.28	3302.4639	30	0.4	1101.8290	3	38.93	33	2211	OB4061.raw	4.04E4	1	1	250	279	
R.SLPYSP(+15.99)YSP(+15.99)Q(+.98)SQ(+.98)PR.Q	N	61.12	1639.7365	14	0.0	820.8755	2	26.89	33	1467	OB4061.raw	4.97E3	1	1	219	232	Hydroxylation Pro; Deamidation (NQ)
R.NALRRPFYSN(+.98)APQ(+.98)EIFIQQGR.G	N	60.81	2506.2717	21	-1.6	836.4298	3	31.36	33	1744	OB4061.raw	3.65E3	1	1	79	99	Deamidation (NQ)
R.RPFYSN(+.98)APQ(+.98)EIFIQQGR.G	N	60.39	2052.0063	17	1.1	685.0101	3	31.39	33	1765	OB4061.raw	5.14E5	3	3	83	99	Deamidation (NQ)
R.N(+.98)ALRRPFYSNAPQEIFIQQGR.G	N	60.26	2505.2876	21	7.9	836.1097	3	29.81	33	1643	OB4061.raw	1.27E6	2	2	79	99	
R.RPFYSNAPQ(+.98)EIFIQ(+.98)QGR.G	N	59.58	2052.0063	17	1.1	685.0101	3	31.39	33	1742	OB4061.raw	4.85E5	1	1	83	99	Deamidation (NQ)
R.NALRRPFYSNAPQEIFIQQGR.G	N	59.57	2504.3037	21	0.3	835.7755	3	29.80	33	1643	OB4061.raw	4.37E5	1	1	79	99	
R.N(+.98)ALRRPFYSNAPQ(+.98)EIFIQQGR.G	N	59.54	2506.2717	21	2.1	836.4329	3	30.96	33	1718	OB4061.raw	0	0	0	79	99	Deamidation (NQ)
S.ISFRQQPEENAC(+57.02)QFQR.L	N	57.69	2036.9486	16	-1.4	679.9892	3	26.80	33	1456	OB4061.raw	9.61E3	1	1	22	37	Carbamidomethylation
S.ISFRQQ(+.98)PEENAC(+57.02)Q(+.98)FQR.L	N	54.88	2038.9166	16	0.3	680.6464	3	27.60	33	1500	OB4061.raw	1.62E5	1	1	22	37	Carbamidomethylation; Deamidation (NQ)
R.LNAQ(+.98)RPDNRLESEGGYIETWN(+.98)PNN(+.98)QEFEC(+57.02)AGVALSR.L	N	54.28	4136.8770	36	5.7	1035.2324	4	32.85	33	1837	OB4061.raw	0	0	0	38	73	Carbamidomethylation
R.SLPYSP(+15.99)YSP(+15.99)QSQPR.Q	N	54.04	1637.7684	14	2.1	819.8932	2	26.31	33	1427	OB4061.raw	3.94E3	1	1	219	232	Hydroxylation Pro
R.AGQEEEN(+.98)EGGN(+.98)IFSGFTPEFLAQAFQVDDR.Q	N	53.61	3303.4480	30	0.3	1102.1570	3	39.26	33	2228	OB4061.raw	0	0	0	250	279	
R.GYFGLIFPGC(+57.02)PSTYEEPA.Q	N	52.94	2003.8975	18	1.3	1002.9573	2	37.84	33	2143	OB4061.raw	1.17E4	1	1	100	117	Carbamidomethylation
R.GYFGLIFPGC(+57.02)PSTYEEPAQ(+.98)Q(+.98)G.R	N	52.47	2319.0042	21	0.8	1160.5103	2	37.59	33	2125	OB4061.raw	2.49E4	1	1	100	120	Carbamidomethylation; Deamidation (NQ)
R.LN(+.98)AQRPDN(+.98)RLESEGGYIETWN(+.98)PNNQ(+.98)EFEC(+57.02)AGVALSR.L	N	51.97	4137.8608	36	8.4	1035.4812	4	32.26	33	1795	OB4061.raw	1.07E6	1	1	38	73	Carbamidomethylation

Peptide	Uniq	-10lgP	Mass	Length	ppm	m/z	z	RT	Fraction	Scan	Source File	Area Digest 11 traka	#Feature	#Feature Digest 11 traka	Start	End	PTM
R.NALRRPFYSNAPQ(+.98)EIFIQQGR.G	N	51.88	2505.2876	21	0.4	836.1035	3	32.40	33	1809	OB4061.raw	0	0	0	79	99	
R.RFNLAGNHEQ(+.98)EFLR.Y	N	51.23	1730.8488	14	-0.6	577.9565	3	28.52	33	1721	OB4061.raw	3.38E5	1	1	197	210	
R.AGQ(+.98)EEENEGGN(+.98)IFSGFTPEFLAQAFQVDDR.Q	N	51.09	3303.4480	30	0.1	1102.1567	3	39.16	33	2222	OB4061.raw	0	0	0	250	279	
N.APQ(+.98)EIFIQQGR.G	N	50.93	1286.6619	11	0.0	644.3382	2	28.98	33	1596	OB4061.raw	7.54E3	1	1	89	99	Deamidation (NQ)
R.RFN(+.98)LAGNHEQ(+.98)EFLR.Y	N	50.88	1731.8329	14	-7.7	578.2805	3	32.35	33	1805	OB4061.raw	1.84E3	1	1	197	210	Deamidation (NQ)
S.ISFRQ(+.98)Q(+.98)PEENAC(+57.02)Q(+.98)FQR.L	N	50.49	2039.9006	16	-0.6	680.9738	3	28.00	33	1549	OB4061.raw	1.73E5	1	1	22	37	Deamidation (NQ); Carbamidomethylation
R.AGQEEENEGGN(+.98)IFSGFTPEFLAQAFQVDDR.Q	N	50.23	3302.4639	30	0.4	1101.8290	3	38.93	33	2198	OB4061.raw	4.04E4	1	1	250	279	
R.LN(+.98)AQRPDNRLESEGGYIETWNPNNQ(+.98)EFEC(+57.02)AGVALSR.L	N	49.19	4135.8931	36	1.2	1034.9818	4	32.17	33	1795	OB4061.raw	0	0	0	38	73	Carbamidomethylation
S.ISFRQQ(+.98)PEENAC(+57.02)QFQR.L	N	48.97	2037.9326	16	-0.6	680.3177	3	27.19	33	1479	OB4061.raw	5.86E4	1	1	22	37	Deamidation (NQ); Carbamidomethylation
R.N(+.98)ALRRPFYSN(+.98)APQEIFIQQGR.G	N	48.84	2506.2717	21	2.4	836.4332	3	31.16	33	1731	OB4061.raw	1.63E4	1	1	79	99	Deamidation (NQ)
R.GYFGLIFPGC(+57.02)PSTYEEPAQ(+.98)Q(+.98).G	N	48.41	2261.9827	20	3.2	1132.0022	2	37.64	33	2132	OB4061.raw	1.17E4	1	1	100	119	Carbamidomethylation; Deamidation (NQ)
S.ISFRQQ(+.98)PEEN(+.98)AC(+57.02)QFQR.L	N	48.36	2038.9166	16	0.3	680.6464	3	27.60	33	1513	OB4061.raw	1.62E5	1	1	22	37	Deamidation (NQ); Carbamidomethylation
R.GYFGLIFPGC(+57.02)PSTYEEPAQ(+.98)Q(+.98)GRR.H	N	47.76	2631.2063	23	8.4	878.0834	3	35.60	33	2005	OB4061.raw	1.86E4	1	1	100	122	Carbamidomethylation; Deamidation (NQ)
R.SLPYSP(+15.99)YSP(+15.99)QSQ(+.98)PR.Q	N	47.57	1638.7524	14	0.2	820.3837	2	26.58	33	1450	OB4061.raw	1.37E4	1	1	219	232	Hydroxylation Pro
R.RPFYSN(+.98)APQEIFIQ(+.98)QGR.G	N	47.52	2052.0063	17	8.6	685.0153	3	34.02	33	1924	OB4061.raw	1.78E4	1	1	83	99	
R.GYFGLIFPGC(+57.02)PSTYEEPAQ(+.98)QG.R	N	47.49	2318.0200	21	0.3	1160.0176	2	37.39	33	2115	OB4061.raw	2.33E3	1	1	100	120	Carbamidomethylation
R.RPFYSN(+.98)APQEIFIQQ(+.98)GR.G	N	47.43	2052.0063	17	3.9	1027.0144	2	31.22	33	1751	OB4061.raw	1.22E4	1	1	83	99	Deamidation (NQ)
R.QIVQNLRGEN(+.98)ESEEEGAIVTVK.G	Y	47.29	2442.2236	22	0.6	815.0823	3	28.52	33	1568	OB4061.raw	7.94E3	1	1	280	301	
R.RFN(+.98)LAGNHEQ(+.98)EFLRYQ(+.98)QQ(+.98)SR.R	N	46.89	2524.1731	20	0.4	632.0508	4	29.52	33	1621	OB4061.raw	6.27E4	1	1	197	216	
R.RPFYSN(+.98)APQ(+.98)EIFIQ(+.98)QGR.G	N	46.74	2052.9905	17	0.7	685.3379	3	31.77	33	1777	OB4061.raw	8.91E4	2	2	83	99	Deamidation (NQ)
R.GYFGLIFPGC(+57.02)PSTYEEPAQ(+.98)QGR.R	N	46.44	2474.1211	22	1.1	825.7152	3	35.30	33	2012	OB4061.raw	1.05E5	1	1	100	121	Carbamidomethylation
R.LNAQRPDNRLESEGGYIETWNPNNQEFEC(+57.02)AGVALSR.L	N	46.11	4133.9248	36	0.5	1034.4890	4	31.62	33	1761	OB4061.raw	0	0	0	38	73	Carbamidomethylation
S.ISFRQ(+.98)Q(+.98)PEENAC(+57.02)QFQ(+.98)R.L	N	45.26	2039.9006	16	-0.6	680.9738	3	28.00	33	1537	OB4061.raw	1.73E5	1	1	22	37	Deamidation (NQ); Carbamidomethylation
R.LNAQRPDN(+.98)RLESEGGYIETWNPN(+.98)NQEFEC(+57.02)AGVALSR.L	N	44.87	4135.8931	36	-2.7	1034.9778	4	33.27	33	1862	OB4061.raw	0	0	0	38	73	Carbamidomethylation

Peptide	Uniq	-10lgP	Mass	Length	ppm	m/z	z	RT	Fraction	Scan	Source File	Area Digest 11 traka	#Feature	#Feature Digest 11 traka	Start	End	PTM
S.ISFRQ(+.98)Q(+.98)PEEN(+.98)AC(+57.02)Q(+.98)FQR.L	N	44.87	2040.8846	16	-0.9	681.3016	3	28.32	33	1561	OB4061.raw	5.57E4	1	1	22	37	Deamidation (NQ); Carbamidomethylation
R.LNAQ(+.98)RPDNRLESEGGYIETWN(+.98)PN(+.98)N(+.98)QEFEC(+57.02)AGVALSR.L	N	43.77	4137.8608	36	8.4	1035.4812	4	32.26	33	1837	OB4061.raw	1.07E6	1	1	38	73	Carbamidomethylation
R.GYFGLIFPGC(+57.02)PSTYEEP(+15.99)AQQGRR.H	N	43.45	2645.2332	23	-3.8	882.7483	3	34.22	33	1911	OB4061.raw	1.52E5	1	1	100	122	Carbamidomethylation
R.GYFGLIFPGC(+57.02)PSTYEEPAQQ(+.98)GRR.H	N	43.06	2630.2224	23	1.1	877.7490	3	33.73	33	1879	OB4061.raw	1.66E6	1	1	100	122	Carbamidomethylation
R.AGQ(+.98)EEEN(+.98)EGGN(+.98)IFSGFTPEFLAQAFQVDDR.Q	N	42.96	3304.4319	30	6.5	1102.4917	3	39.36	33	2234	OB4061.raw	0	0	0	250	279	
R.LNAQ(+.98)RPDNRLESEGGYIETWN(+.98)PNNQEFEC(+57.02)AGVALSR.L	N	42.45	4135.8931	36	3.4	1034.9840	4	34.49	33	1937	OB4061.raw	0	0	0	38	73	Carbamidomethylation
R.GYFGLIFPGC(+57.02)PSTYEEPAQ(+.98)QGRR.H	N	41.86	2630.2224	23	-0.9	877.7473	3	36.15	33	2038	OB4061.raw	1.66E6	1	1	100	122	Carbamidomethylation
S.ISFRQQ(+.98)PEENAC(+57.02)QFQ(+.98)R.L	N	41.56	2038.9166	16	0.3	680.6464	3	27.60	33	1608	OB4061.raw	1.62E5	1	1	22	37	Carbamidomethylation
R.LNAQRPDNRLESEGGYIETWN(+.98)PNNQEFEC(+57.02)AGVALSR.L	N	41.33	4134.9087	36	1.7	1034.7362	4	32.06	33	1772	OB4061.raw	7.3E5	1	1	38	73	Carbamidomethylation
R.LNAQRPDN(+.98)RLESEGGYIETWNPNNQ(+.98)EFEC(+57.02)AGVALSR.L	N	41.26	4135.8931	36	-1.8	1034.9786	4	34.69	33	1949	OB4061.raw	0	0	0	38	73	Carbamidomethylation
G.GYIETWNPN(+.98)NQEFEC(+57.02)AGVALSR.L	N	41.06	2555.1387	22	1.5	852.7214	3	33.24	33	1865	OB4061.raw	8.92E3	1	1	52	73	Carbamidomethylation
R.RFN(+.98)LAGNHEQ(+.98)EFLRYQ(+.98)Q(+.98)Q(+.98)SR.R	N	40.65	2525.1570	20	0.8	632.2970	4	29.72	33	1633	OB4061.raw	5.01E4	1	1	197	216	Deamidation (NQ)
R.LNAQRPDNRLESEGGYIETWN(+.98)PN(+.98)N(+.98)Q(+.98)EFEC(+57.02)AGVALSR.L	N	40.17	4137.8608	36	8.4	1035.4812	4	32.26	33	1873	OB4061.raw	1.07E6	1	1	38	73	Carbamidomethylation
R.RFN(+.98)LAGNHEQ(+.98)EFLRYQQ(+.98)Q(+.98)SR.R	N	39.71	2524.1731	20	-2.0	632.0493	4	30.07	33	1660	OB4061.raw	0	0	0	197	216	
R.LNAQRPDNRLESEGGYIETWNPNNQ(+.98)EFEC(+57.02)AGVALSR.L	N	39.69	4134.9087	36	1.7	1034.7362	4	32.06	33	1795	OB4061.raw	7.3E5	1	1	38	73	Carbamidomethylation
R.GYFGLIFPGC(+57.02)P(+15.99)STYEEPAQ(+.98)QGR.R	N	39.49	2490.1160	22	0.0	1246.0653	2	35.97	33	2027	OB4061.raw	0	0	0	100	121	Carbamidomethylation
R.LNAQ(+.98)RPDNRLESEGGYIETWNPNNQEFEC(+57.02)AGVALSR.L	N	39.09	4134.9087	36	1.7	1034.7362	4	32.06	33	1837	OB4061.raw	7.3E5	1	1	38	73	Carbamidomethylation
S.ISFRQQ(+.98)PEEN(+.98)AC(+57.02)Q(+.98)FQR.L	N	39.05	2039.9006	16	-0.6	680.9738	3	28.00	33	1655	OB4061.raw	1.73E5	1	1	22	37	Carbamidomethylation; Deamidation (NQ)
R.SLPYSP(+15.99)YSP(+15.99)Q(+.98)SQPR.Q	N	37.63	1638.7524	14	0.2	820.3837	2	26.58	33	1440	OB4061.raw	1.37E4	1	1	219	232	Hydroxylation Pro; Deamidation (NQ)
R.LN(+.98)AQ(+.98)RPDN(+.98)RLESEGGYIETWNPN(+.98)NQEFEC(+57.02)AGVALSR.L	N	37.29	4137.8608	36	8.4	1035.4812	4	32.26	33	1862	OB4061.raw	1.07E6	1	1	38	73	Carbamidomethylation
R.LNAQRPDNRLESEGGYIETWNPN(+.98)NQEFEC(+57.02)AGVALSR.L	N	36.66	4134.9087	36	1.7	1034.7362	4	32.06	33	1862	OB4061.raw	7.3E5	1	1	38	73	Carbamidomethylation
R.LNAQ(+.98)RPDNRLESEGGYIETWNPNNQ(+.98)EFEC(+57.02)AGVALSR.L	N	36.58	4135.8931	36	4.1	1034.9847	4	33.98	33	1906	OB4061.raw	0	0	0	38	73	Carbamidomethylation
R.LNAQRPDNRLESEGGYIETWNPNN(+.98)Q(+.98)EFEC(+57.02)AGVALSR.L	N	36.46	4135.8931	36	-0.4	1034.9801	4	33.44	33	1873	OB4061.raw	0	0	0	38	73	Carbamidomethylation
S.ISFRQQ(+.98)PEEN(+.98)AC(+57.02)Q(+.98)FQ(+.98)R.L	N	35.63	2040.8846	16	8.6	681.3080	3	29.16	33	1604	OB4061.raw	0	0	0	22	37	Deamidation (NQ); Carbamidomethylation

Peptide	Uniq	-10lgP	Mass	Length	ppm	m/z	z	RT	Fraction	Scan	Source File	Area Digest 11 traka	#Feature	#Feature Digest 11 traka	Start	End	PTM
R.RFDEGDLIAVP(+15.99)TGVALWMFNDHDTDVVAVSLTDTNN(+.98)N(+.98)DN(+.98)Q(+.98)LDQFPR.R	Y	35.49	5194.3623	46	1.8	1299.6002	4	37.67	33	2131	OB4061.raw	0	0	0	151	196	
S.ISFRQ(+.98)Q(+.98)PEEN(+.98)AC(+57.02)QFQR.L	N	35.33	2039.9006	16	-0.6	680.9738	3	28.00	33	1631	OB4061.raw	1.73E5	1	1	22	37	Deamidation (NQ); Carbamidomethylation
R.GYFGLIFPGC(+57.02)P(+15.99)STYEEPAQQGR.R	N	35.03	2489.1321	22	-1.6	1245.5713	2	35.79	33	2018	OB4061.raw	3.62E4	1	1	100	121	Carbamidomethylation
R.RPFYSNAPQ(+.98)E(+28.03)IFIQQGR.G	N	34.53	2079.0537	17	1.4	694.0262	3	32.01	33	1791	OB4061.raw	7.07E3	1	1	83	99	Ethylation
R.LN(+.98)AQRPDNRLESEGGYIETWN(+.98)PNNQEFEC(+57.02)AGVALSR.L	N	34.46	4135.8931	36	-1.8	1034.9786	4	34.26	33	1923	OB4061.raw	0	0	0	38	73	Carbamidomethylation
R.LNAQRPDN(+.98)RLESEGGYIETWNPNNQEFEC(+57.02)AGVALSR.L	N	33.89	4134.9087	36	1.7	1034.7362	4	32.06	33	1949	OB4061.raw	7.3E5	1	1	38	73	Carbamidomethylation
R.GYFGLIFPGC(+57.02)P(+15.99)STYEEPAQQGRR.H	N	33.80	2645.2332	23	-2.0	882.7499	3	35.56	33	2002	OB4061.raw	0	0	0	100	122	Carbamidomethylation
R.RFN(+.98)LAGNHEQ(+.98)EFLRYQ(+.98)Q(+.98)QSR.R	N	33.64	2524.1731	20	0.4	632.0508	4	29.52	33	1633	OB4061.raw	6.27E4	1	1	197	216	Deamidation (NQ)
D.NRLESEGGYIETWNPN(+.98)N(+.98)QEFEC(+57.02)AGVALSR.L	N	33.60	3341.4895	29	8.0	1114.8461	3	32.45	33	1829	OB4061.raw	1.54E4	1	1	45	73	Carbamidomethylation
R.GYFGLIFPGC(+57.02)PSTYEEP(+15.99)AQQ(+.98)GRR.H	N	33.28	2646.2173	23	-3.1	883.0770	3	34.33	33	1927	OB4061.raw	0	0	0	100	122	Carbamidomethylation
R.LNAQ(+.98)RPDNRLESEGGYIETWNP(+15.99)NNQEFEC(+57.02)AGVALSR.L	N	33.23	4150.9038	36	3.1	1038.7365	4	32.59	33	1821	OB4061.raw	0	0	0	38	73	Carbamidomethylation
S.ISFRQQ(+.98)PEEN(+.98)AC(+57.02)QFQ(+.98)R.L	N	32.96	2039.9006	16	-0.6	680.9738	3	28.00	33	1580	OB4061.raw	1.73E5	1	1	22	37	Deamidation (NQ); Carbamidomethylation
R.RFNLAGNHEQ(+.98)EFLRYQQQ(+.98)SR.R	N	32.49	2522.2051	20	-1.5	631.5576	4	28.58	33	1569	OB4061.raw	0	0	0	197	216	
R.LNAQ(+.98)RPDNRLESEGGYIETWN(+.98)PN(+.98)NQEFEC(+57.02)AGVALSR.L	N	32.40	4136.8770	36	2.9	1379.9702	3	32.28	33	1802	OB4061.raw	0	0	0	38	73	Carbamidomethylation
R.RFNLAGNHEQ(+.98)EFLRYQ(+.98)Q(+.98)QSR.R	N	32.35	2523.1890	20	-0.9	631.8040	4	28.96	33	1585	OB4061.raw	4.66E4	1	1	197	216	
R.LNAQRPDN(+.98)RLESEGGYIETWN(+.98)PNN(+.98)Q(+.98)EFEC(+57.02)AGVALSR.L	N	32.33	4137.8608	36	8.4	1035.4812	4	32.26	33	1889	OB4061.raw	1.07E6	1	1	38	73	Carbamidomethylation
R.LNAQRPDNRLESEGGYIETWN(+.98)PNN(+.98)Q(+.98)EFEC(+57.02)AGVALSR.L	N	32.16	4136.8770	36	6.7	1035.2334	4	34.10	33	1913	OB4061.raw	0	0	0	38	73	Carbamidomethylation
R.RFN(+.98)LAGN(+.98)HEQ(+.98)EFLR.Y	N	31.96	1732.8169	14	8.6	578.6179	3	29.66	33	1635	OB4061.raw	0	0	0	197	210	Deamidation (NQ)
R.AGQEEENEGGN(+.98)IFSGFTPEFLAQ(+.98)AFQVDDRQIVQ(+.98)N(+.98)LRGEN(+.98)ESEEEGAIVTVK.G	Y	31.92	5729.6289	52	2.4	1146.9358	5	38.87	33	2204	OB4061.raw	0	0	0	250	301	
R.N(+.98)ALRRPFYSN(+.98)APQ(+.98)EIFIQ(+.98)QGR.G	N	31.70	2508.2397	21	8.1	837.0939	3	31.77	33	1778	OB4061.raw	1.36E4	1	1	79	99	Deamidation (NQ)
R.RFDEGDLIAVPTGVALWM(+15.99)FNDHDTDVVAVSLTDTNNNDN(+.98)QLDQFPR.R	Y	31.16	5191.4106	46	-3.1	1298.8560	4	37.21	33	2103	OB4061.raw	0	0	0	151	196	Oxidation (M)
R.GYFGLIFPGC(+57.02)PSTYEEP(+15.99)AQQGR.R	N	31.00	2489.1321	22	-5.5	830.7134	3	35.79	33	2026	OB4061.raw	1.77E4	1	1	100	121	Carbamidomethylation
R.GYFGLIFPGC(+57.02)P(+15.99)STYEEP(+15.99)AQ(+.98)QGRR.H	N	30.73	2662.2122	23	-3.5	888.4082	3	33.92	33	1899	OB4061.raw	2.53E4	1	1	100	122	Carbamidomethylation; Hydroxylation Pro
R.RPFYSNAPQ(+.98)EIFIQQ(+.98)GR.G	N	30.13	2052.0063	17	-3.1	685.0073	3	37.03	33	2092	OB4061.raw	0	0	0	83	99	

Peptide	Uniq	-10lgP	Mass	Length	ppm	m/z	z	RT	Fraction	Scan	Source File	Area Digest 11 traka	#Feature	#Feature Digest 11 traka	Start	End	PTM
R.GYFGLIFP(+15.99)GC(+57.02)PSTYEEPAQQ(+.98)GRR.H	N	30.09	2646.2173	23	4.6	883.0838	3	34.74	33	1952	OB4061.raw	0	0	0	100	122	Carbamidomethylation
R.GYFGLIFPGC(+57.02)P(+15.99)STYEEPAQ(+.98)QGRR.H	N	30.00	2646.2173	23	3.6	883.0829	3	34.54	33	1940	OB4061.raw	0	0	0	100	122	Carbamidomethylation
R.RFN(+.98)LAGN(+.98)HEQ(+.98)EFLRYQQ(+.98)Q(+.98)SR.R	N	29.97	2525.1570	20	0.8	632.2970	4	29.72	33	1660	OB4061.raw	5.01E4	1	1	197	216	Deamidation (NQ)
S.ISFRQ(+.98)Q(+.98)PEEN(+.98)AC(+57.02)Q(+.98)FQ(+.98)R.L	N	29.57	2041.8687	16	-3.6	681.6277	3	28.86	33	1580	OB4061.raw	4.5E3	1	1	22	37	Deamidation (NQ); Carbamidomethylation
R.RPFYSNAPQ(+.98)E(+28.03)IFIQQ(+.98)GR.G	N	29.20	2080.0378	17	-0.4	694.3530	3	32.43	33	1811	OB4061.raw	0	0	0	83	99	
R.RPFYSNAPQ(+29.02)EIFIQQGR.G	N	29.06	2079.0537	17	1.4	694.0262	3	32.01	33	1786	OB4061.raw	7.07E3	1	1	83	99	
R.LNAQ(+.98)RPDNRLESEGGYIETWN(+.98)PN(+.98)NQ(+.98)EFEC(+57.02)AGVALSR.L	N	28.50	4137.8608	36	8.4	1035.4812	4	32.26	33	1913	OB4061.raw	1.07E6	1	1	38	73	Carbamidomethylation
R.NALRRPFYSNAPQ(+.98)EIFIQ(+.98)Q(+.98)GR.G	N	28.37	2507.2556	21	3.4	836.7620	3	33.21	33	1858	OB4061.raw	0	0	0	79	99	
R.LNAQRP(+15.99)DNRLESEGGYIETWNPNNQEFEC(+57.02)AGVALSR.L	N	27.91	4149.9199	36	1.0	1038.4883	4	32.51	33	1816	OB4061.raw	0	0	0	38	73	Carbamidomethylation
R.AGQ(+.98)EEENEGGNIFSGFTPEFLAQAFQVDDRQIVQ(+.98)NLRGEN(+.98)ESEEEGAIVTVK.G	Y	27.27	5727.6611	52	6.8	1146.5472	5	37.94	33	2180	OB4061.raw	5.86E4	1	1	250	301	
R.GYFGLIFPGCPST(+79.97)YEEPAQQGR.R	N	26.69	2496.0820	22	8.0	833.0413	3	35.30	33	1991	OB4061.raw	6.76E3	1	1	100	121	
R.AGQ(+.98)EEEN(+.98)EGGNIFSGFTPEFLAQ(+.98)AFQVDDRQ(+.98)IVQN(+.98)LRGENESEEEGAIVTVK.G	Y	25.63	5729.6289	52	-0.4	1146.9326	5	39.18	33	2223	OB4061.raw	0	0	0	250	301	
**total 113 peptides**																	

tr|Q647H3|Q647H3_ARAHY
back to list

| Protein Coverage | Supporting Peptides |

Protein Coverage:

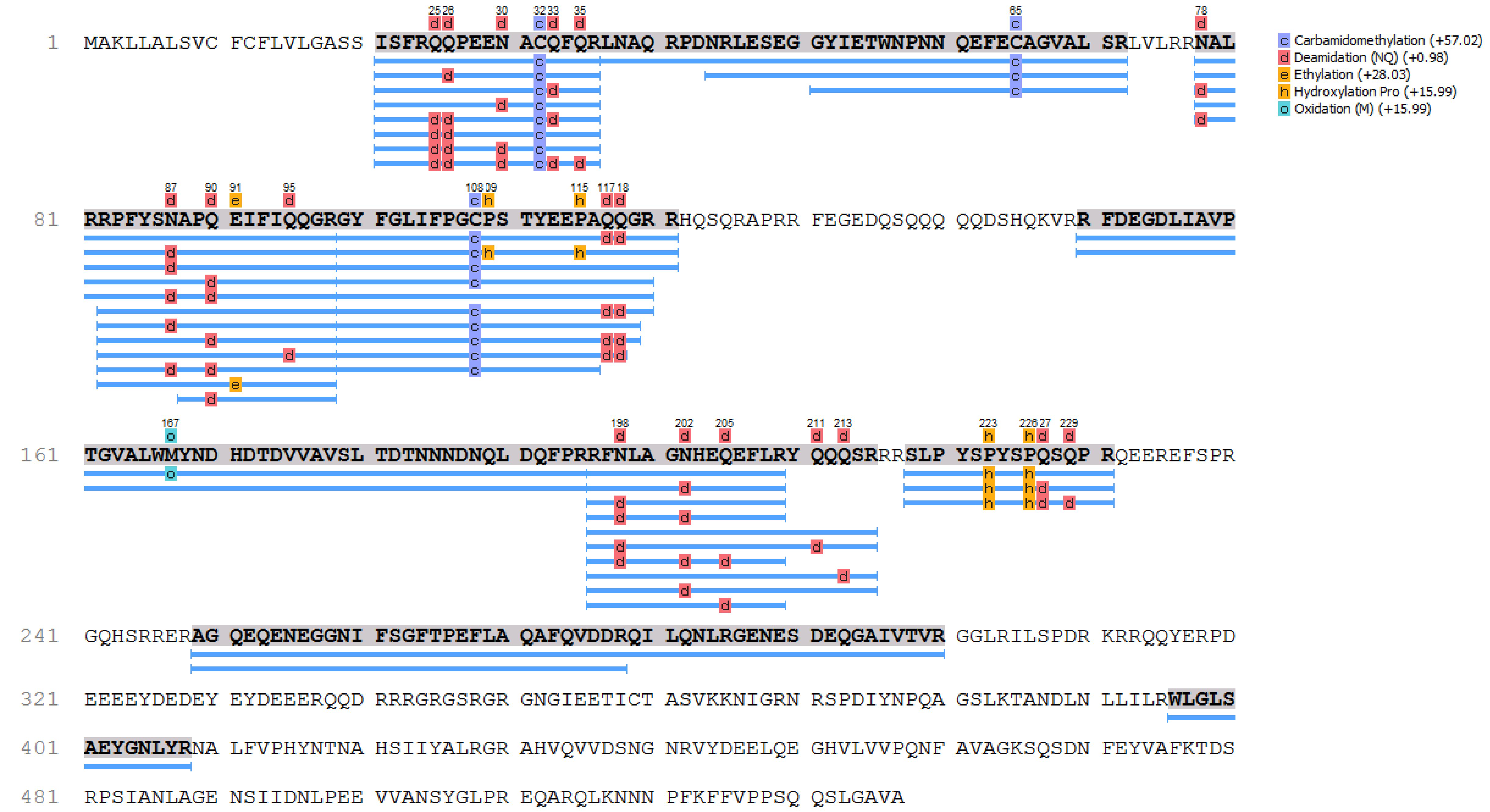

Supporting Peptides:

Peptide	Uniq	-10lgP	Mass	Length	ppm	m/z	z	RT	Fraction	Scan	Source File	Area Digest 11 traka	#Feature	#Feature Digest 11 traka	Start	End	PTM
R.WLGLSAEYGNLYR.N	N	85.02	1540.7673	13	0.5	771.3914	2	33.53	33	1880	OB4061.raw	6.78E4	1	1	396	408	
R.GYFGLIFPGC(+57.02)PSTYEEPAQQ(+.98)GR.R	N	76.60	2474.1211	22	1.1	1238.0692	2	35.30	33	1981	OB4061.raw	2.38E5	3	3	99	120	Carbamidomethylation
R.RFNLAGNHEQEFLR.Y	N	76.02	1729.8647	14	-0.6	577.6285	3	27.81	33	1524	OB4061.raw	2.56E5	2	2	196	209	
R.RPFYSNAPQEIFIQQGR.G	N	74.68	2050.0383	17	-0.3	684.3531	3	30.39	33	1681	OB4061.raw	7.21E5	2	2	82	98	
R.RPFYSNAPQ(+.98)EIFIQQGR.G	N	73.51	2051.0225	17	-0.2	1026.5183	2	30.94	33	1720	OB4061.raw	1.06E6	4	4	82	98	Deamidation (NQ)
R.NALRRPFYSN(+.98)APQEIFIQQGR.G	N	72.57	2505.2876	21	-0.8	836.1025	3	31.01	33	1675	OB4061.raw	1.53E6	2	2	78	98	Deamidation (NQ)
R.GYFGLIFPGC(+57.02)PSTYEEPAQQGR.R	N	69.55	2473.1372	22	0.1	825.3864	3	35.12	33	1975	OB4061.raw	0	0	0	99	120	Carbamidomethylation
R.RPFYSN(+.98)APQEIFIQQGR.G	N	69.19	2051.0225	17	-0.2	1026.5183	2	30.94	33	1715	OB4061.raw	2.49E4	2	2	82	98	Deamidation (NQ)
R.RFNLAGN(+.98)HEQEFLR.Y	N	68.65	1730.8488	14	-0.6	577.9565	3	28.52	33	1578	OB4061.raw	3.38E5	1	1	196	209	Deamidation (NQ)

Peptide	Uniq	-10lgP	Mass	Length	ppm	m/z	z	RT	Fraction	Scan	Source File	Area Digest 11 traka	#Feature	#Feature Digest 11 traka	Start	End	PTM
R.RPFYSNAPQEIFIQ(+.98)QGR.G	N	67.75	2051.0225	17	1.1	684.6822	3	30.94	33	1700	OB4061.raw	1.03E6	1	1	82	98	
R.GYFGLIFPGC(+57.02)PSTYEEPAQ(+.98)Q(+.98)GR.R	N	65.12	2475.1052	22	3.3	1238.5640	2	35.40	33	1993	OB4061.raw	0	0	0	99	120	Carbamidomethylation; Deamidation (NQ)
R.RFN(+.98)LAGN(+.98)HEQEFLR.Y	N	63.41	1731.8329	14	-1.4	578.2841	3	28.98	33	1589	OB4061.raw	1.14E5	1	1	196	209	Deamidation (NQ)
R.RFN(+.98)LAGNHEQEFLR.Y	N	62.87	1730.8488	14	-0.6	577.9565	3	28.52	33	1612	OB4061.raw	3.38E5	1	1	196	209	Deamidation (NQ)
R.SLPYSP(+15.99)YSP(+15.99)Q(+.98)SQ(+.98)PR.Q	N	61.12	1639.7365	14	0.0	820.8755	2	26.89	33	1467	OB4061.raw	4.97E3	1	1	218	231	Hydroxylation Pro; Deamidation (NQ)
R.NALRRPFYSN(+.98)APQ(+.98)EIFIQQGR.G	N	60.81	2506.2717	21	-1.6	836.4298	3	31.36	33	1744	OB4061.raw	3.65E3	1	1	78	98	Deamidation (NQ)
R.RPFYSN(+.98)APQ(+.98)EIFIQQGR.G	N	60.39	2052.0063	17	1.1	685.0101	3	31.39	33	1765	OB4061.raw	5.14E5	3	3	82	98	Deamidation (NQ)
R.N(+.98)ALRRPFYSNAPQEIFIQQGR.G	N	60.26	2505.2876	21	7.9	836.1097	3	29.81	33	1643	OB4061.raw	1.27E6	2	2	78	98	
R.RPFYSNAPQ(+.98)EIFIQ(+.98)QGR.G	N	59.58	2052.0063	17	1.1	685.0101	3	31.39	33	1742	OB4061.raw	4.85E5	1	1	82	98	Deamidation (NQ)
R.NALRRPFYSNAPQEIFIQQGR.G	N	59.57	2504.3037	21	0.3	835.7755	3	29.80	33	1643	OB4061.raw	4.37E5	1	1	78	98	
R.N(+.98)ALRRPFYSNAPQ(+.98)EIFIQQGR.G	N	59.54	2506.2717	21	2.1	836.4329	3	30.96	33	1718	OB4061.raw	0	0	0	78	98	Deamidation (NQ)
S.ISFRQQPEENAC(+57.02)QFQR.L	N	57.69	2036.9486	16	-1.4	679.9892	3	26.80	33	1456	OB4061.raw	9.61E3	1	1	21	36	Carbamidomethylation
R.AGQ(+.98)EQENEGGN(+.98)IFSGFTPEFLAQAFQVDDR.Q	Y	56.05	3302.4639	30	0.4	1101.8290	3	38.93	33	2203	OB4061.raw	4.04E4	1	1	249	278	
S.ISFRQQ(+.98)PEENAC(+57.02)Q(+.98)FQR.L	N	54.88	2038.9166	16	0.3	680.6464	3	27.60	33	1500	OB4061.raw	1.62E5	1	1	21	36	Carbamidomethylation; Deamidation (NQ)
R.LNAQ(+.98)RPDNRLESEGGYIETWN(+.98)PNN(+.98)QEFEC(+57.02)AGVALSR.L	N	54.28	4136.8770	36	5.7	1035.2324	4	32.85	33	1837	OB4061.raw	0	0	0	37	72	Carbamidomethylation
R.SLPYSP(+15.99)YSP(+15.99)QSQPR.Q	N	54.04	1637.7684	14	2.1	819.8932	2	26.31	33	1427	OB4061.raw	3.94E3	1	1	218	231	Hydroxylation Pro
R.GYFGLIFPGC(+57.02)PSTYEEPA.Q	N	52.94	2003.8975	18	1.3	1002.9573	2	37.84	33	2143	OB4061.raw	1.17E4	1	1	99	116	Carbamidomethylation
R.GYFGLIFPGC(+57.02)PSTYEEPAQ(+.98)Q(+.98)G.R	N	52.47	2319.0042	21	0.8	1160.5103	2	37.59	33	2125	OB4061.raw	2.49E4	1	1	99	119	Carbamidomethylation; Deamidation (NQ)
R.LN(+.98)AQRPDN(+.98)RLESEGGYIETWN(+.98)PNNQ(+.98)EFEC(+57.02)AGVALSR.L	N	51.97	4137.8608	36	8.4	1035.4812	4	32.26	33	1795	OB4061.raw	1.07E6	1	1	37	72	Carbamidomethylation
R.NALRRPFYSNAPQ(+.98)EIFIQQGR.G	N	51.88	2505.2876	21	0.4	836.1035	3	32.40	33	1809	OB4061.raw	0	0	0	78	98	
R.RFNLAGNHEQ(+.98)EFLR.Y	N	51.23	1730.8488	14	-0.6	577.9565	3	28.52	33	1721	OB4061.raw	3.38E5	1	1	196	209	
N.APQ(+.98)EIFIQQGR.G	N	50.93	1286.6619	11	0.0	644.3382	2	28.98	33	1596	OB4061.raw	7.54E3	1	1	88	98	Deamidation (NQ)
R.RFN(+.98)LAGNHEQ(+.98)EFLR.Y	N	50.88	1731.8329	14	-7.7	578.2805	3	32.35	33	1805	OB4061.raw	1.84E3	1	1	196	209	Deamidation (NQ)
S.ISFRQ(+.98)Q(+.98)PEENAC(+57.02)Q(+.98)FQR.L	N	50.49	2039.9006	16	-0.6	680.9738	3	28.00	33	1549	OB4061.raw	1.73E5	1	1	21	36	Deamidation (NQ); Carbamidomethylation
R.LN(+.98)AQRPDNRLESEGGYIETWNPNNQ(+.98)EFEC(+57.02)AGVALSR.L	N	49.19	4135.8931	36	1.2	1034.9818	4	32.17	33	1795	OB4061.raw	0	0	0	37	72	Carbamidomethylation
S.ISFRQQ(+.98)PEENAC(+57.02)QFQR.L	N	48.97	2037.9326	16	-0.6	680.3177	3	27.19	33	1479	OB4061.raw	5.86E4	1	1	21	36	Deamidation (NQ); Carbamidomethylation
R.N(+.98)ALRRPFYSN(+.98)APQEIFIQQGR.G	N	48.84	2506.2717	21	2.4	836.4332	3	31.16	33	1731	OB4061.raw	1.63E4	1	1	78	98	Deamidation (NQ)
R.GYFGLIFPGC(+57.02)PSTYEEPAQ(+.98)Q(+.98).G	N	48.41	2261.9827	20	3.2	1132.0022	2	37.64	33	2132	OB4061.raw	1.17E4	1	1	99	118	Carbamidomethylation; Deamidation (NQ)
S.ISFRQQ(+.98)PEEN(+.98)AC(+57.02)QFQR.L	N	48.36	2038.9166	16	0.3	680.6464	3	27.60	33	1513	OB4061.raw	1.62E5	1	1	21	36	Deamidation (NQ); Carbamidomethylation
R.GYFGLIFPGC(+57.02)PSTYEEPAQ(+.98)Q(+.98)GRR.H	N	47.76	2631.2063	23	8.4	878.0834	3	35.60	33	2005	OB4061.raw	1.86E4	1	1	99	121	Carbamidomethylation; Deamidation (NQ)
R.SLPYSP(+15.99)YSP(+15.99)QSQ(+.98)PR.Q	N	47.57	1638.7524	14	0.2	820.3837	2	26.58	33	1450	OB4061.raw	1.37E4	1	1	218	231	Hydroxylation Pro
R.RPFYSN(+.98)APQEIFIQ(+.98)QGR.G	N	47.52	2052.0063	17	8.6	685.0153	3	34.02	33	1924	OB4061.raw	1.78E4	1	1	82	98	
R.GYFGLIFPGC(+57.02)PSTYEEPAQ(+.98)QG.R	N	47.49	2318.0200	21	0.3	1160.0176	2	37.39	33	2115	OB4061.raw	2.33E3	1	1	99	119	Carbamidomethylation
R.RPFYSN(+.98)APQEIFIQQ(+.98)GR.G	N	47.43	2052.0063	17	3.9	1027.0144	2	31.22	33	1751	OB4061.raw	1.22E4	1	1	82	98	Deamidation (NQ)
R.RFN(+.98)LAGNHEQ(+.98)EFLRYQ(+.98)QQ(+.98)SR.R	N	46.89	2524.1731	20	0.4	632.0508	4	29.52	33	1621	OB4061.raw	6.27E4	1	1	196	215	
R.RPFYSN(+.98)APQ(+.98)EIFIQ(+.98)QGR.G	N	46.74	2052.9905	17	0.7	685.3379	3	31.77	33	1777	OB4061.raw	8.91E4	2	2	82	98	Deamidation (NQ)

Peptide	Uniq	-10lgP	Mass	Length	ppm	m/z	z	RT	Fraction	Scan	Source File	Area Digest 11 traka	#Feature	#Feature Digest 11 traka	Start	End	PTM
R.GYFGLIFPGC(+57.02)PSTYEEPAQ(+.98)QGR.R	N	46.44	2474.1211	22	1.1	825.7152	3	35.30	33	2012	OB4061.raw	1.05E5	1	1	99	120	Carbamidomethylation
R.LNAQRPDNRLESEGGYIETWNPNNQEFEC(+57.02)AGVALSR.L	N	46.11	4133.9248	36	0.5	1034.4890	4	31.62	33	1761	OB4061.raw	0	0	0	37	72	Carbamidomethylation
S.ISFRQ(+.98)Q(+.98)PEENAC(+57.02)QFQ(+.98)R.L	N	45.26	2039.9006	16	-0.6	680.9738	3	28.00	33	1537	OB4061.raw	1.73E5	1	1	21	36	Deamidation (NQ); Carbamidomethylation
R.LNAQRPDN(+.98)RLESEGGYIETWNPN(+.98)NQEFEC(+57.02)AGVALSR.L	N	44.87	4135.8931	36	-2.7	1034.9778	4	33.27	33	1862	OB4061.raw	0	0	0	37	72	Carbamidomethylation
S.ISFRQ(+.98)Q(+.98)PEEN(+.98)AC(+57.02)Q(+.98)FQR.L	N	44.87	2040.8846	16	-0.9	681.3016	3	28.32	33	1561	OB4061.raw	5.57E4	1	1	21	36	Deamidation (NQ); Carbamidomethylation
R.LNAQ(+.98)RPDNRLESEGGYIETWN(+.98)PN(+.98)N(+.98)QEFEC(+57.02)AGVALSR.L	N	43.77	4137.8608	36	8.4	1035.4812	4	32.26	33	1837	OB4061.raw	1.07E6	1	1	37	72	Carbamidomethylation
R.GYFGLIFPGC(+57.02)PSTYEEP(+15.99)AQQGRR.H	N	43.45	2645.2332	23	-3.8	882.7483	3	34.22	33	1911	OB4061.raw	1.52E5	1	1	99	121	Carbamidomethylation
R.GYFGLIFPGC(+57.02)PSTYEEPAQQ(+.98)GRR.H	N	43.06	2630.2224	23	1.1	877.7490	3	33.73	33	1879	OB4061.raw	1.66E6	1	1	99	121	Carbamidomethylation
R.LNAQ(+.98)RPDNRLESEGGYIETWN(+.98)PNNQEFEC(+57.02)AGVALSR.L	N	42.45	4135.8931	36	3.4	1034.9840	4	34.49	33	1937	OB4061.raw	0	0	0	37	72	Carbamidomethylation
R.GYFGLIFPGC(+57.02)PSTYEEPAQ(+.98)QGRR.H	N	41.86	2630.2224	23	-0.9	877.7473	3	36.15	33	2038	OB4061.raw	1.66E6	1	1	99	121	Carbamidomethylation
S.ISFRQQ(+.98)PEENAC(+57.02)QFQ(+.98)R.L	N	41.56	2038.9166	16	0.3	680.6464	3	27.60	33	1608	OB4061.raw	1.62E5	1	1	21	36	Carbamidomethylation
R.LNAQRPDNRLESEGGYIETWN(+.98)PNNQEFEC(+57.02)AGVALSR.L	N	41.33	4134.9087	36	1.7	1034.7362	4	32.06	33	1772	OB4061.raw	7.3E5	1	1	37	72	Carbamidomethylation
R.LNAQRPDN(+.98)RLESEGGYIETWNPNNQ(+.98)EFEC(+57.02)AGVALSR.L	N	41.26	4135.8931	36	-1.8	1034.9786	4	34.69	33	1949	OB4061.raw	0	0	0	37	72	Carbamidomethylation
G.GYIETWNPN(+.98)NQEFEC(+57.02)AGVALSR.L	N	41.06	2555.1387	22	1.5	852.7214	3	33.24	33	1865	OB4061.raw	8.92E3	1	1	51	72	Carbamidomethylation
R.RFN(+.98)LAGNHEQ(+.98)EFLRYQ(+.98)Q(+.98)Q(+.98)SR.R	N	40.65	2525.1570	20	0.8	632.2970	4	29.72	33	1633	OB4061.raw	5.01E4	1	1	196	215	Deamidation (NQ)
R.LNAQRPDNRLESEGGYIETWN(+.98)PN(+.98)N(+.98)Q(+.98)EFEC(+57.02)AGVALSR.L	N	40.17	4137.8608	36	8.4	1035.4812	4	32.26	33	1873	OB4061.raw	1.07E6	1	1	37	72	Carbamidomethylation
R.RFN(+.98)LAGNHEQ(+.98)EFLRYQQ(+.98)Q(+.98)SR.R	N	39.71	2524.1731	20	-2.0	632.0493	4	30.07	33	1660	OB4061.raw	0	0	0	196	215	
R.LNAQRPDNRLESEGGYIETWNPNNQ(+.98)EFEC(+57.02)AGVALSR.L	N	39.69	4134.9087	36	1.7	1034.7362	4	32.06	33	1795	OB4061.raw	7.3E5	1	1	37	72	Carbamidomethylation
R.GYFGLIFPGC(+57.02)P(+15.99)STYEEPAQ(+.98)QGR.R	N	39.49	2490.1160	22	0.0	1246.0653	2	35.97	33	2027	OB4061.raw	0	0	0	99	120	Carbamidomethylation
R.LNAQ(+.98)RPDNRLESEGGYIETWNPNNQEFEC(+57.02)AGVALSR.L	N	39.09	4134.9087	36	1.7	1034.7362	4	32.06	33	1837	OB4061.raw	7.3E5	1	1	37	72	Carbamidomethylation
S.ISFRQQ(+.98)PEEN(+.98)AC(+57.02)Q(+.98)FQR.L	N	39.05	2039.9006	16	-0.6	680.9738	3	28.00	33	1655	OB4061.raw	1.73E5	1	1	21	36	Carbamidomethylation; Deamidation (NQ)
R.SLPYSP(+15.99)YSP(+15.99)Q(+.98)SQPR.Q	N	37.63	1638.7524	14	0.2	820.3837	2	26.58	33	1440	OB4061.raw	1.37E4	1	1	218	231	Hydroxylation Pro; Deamidation (NQ)
R.LN(+.98)AQ(+.98)RPDN(+.98)RLESEGGYIETWNPN(+.98)NQEFEC(+57.02)AGVALSR.L	N	37.29	4137.8608	36	8.4	1035.4812	4	32.26	33	1862	OB4061.raw	1.07E6	1	1	37	72	Carbamidomethylation
R.LNAQRPDNRLESEGGYIETWNPN(+.98)NQEFEC(+57.02)AGVALSR.L	N	36.66	4134.9087	36	1.7	1034.7362	4	32.06	33	1862	OB4061.raw	7.3E5	1	1	37	72	Carbamidomethylation
R.LNAQ(+.98)RPDNRLESEGGYIETWNPNNQ(+.98)EFEC(+57.02)AGVALSR.L	N	36.58	4135.8931	36	4.1	1034.9847	4	33.98	33	1906	OB4061.raw	0	0	0	37	72	Carbamidomethylation
R.LNAQRPDNRLESEGGYIETWNPNN(+.98)Q(+.98)EFEC(+57.02)AGVALSR.L	N	36.46	4135.8931	36	-0.4	1034.9801	4	33.44	33	1873	OB4061.raw	0	0	0	37	72	Carbamidomethylation
S.ISFRQQ(+.98)PEEN(+.98)AC(+57.02)Q(+.98)FQ(+.98)R.L	N	35.63	2040.8846	16	8.6	681.3080	3	29.16	33	1604	OB4061.raw	0	0	0	21	36	Deamidation (NQ); Carbamidomethylation
S.ISFRQ(+.98)Q(+.98)PEEN(+.98)AC(+57.02)QFQR.L	N	35.33	2039.9006	16	-0.6	680.9738	3	28.00	33	1631	OB4061.raw	1.73E5	1	1	21	36	Deamidation (NQ); Carbamidomethylation
R.RFDEGDLIAVPTGVALWMYNDHDTDVVAVSLTDTNNNDNQ(+.98)LDQFPR.R	Y	35.26	5191.4106	46	1.4	1298.8617	4	37.11	33	2097	OB4061.raw	0	0	0	150	195	
R.GYFGLIFPGC(+57.02)P(+15.99)STYEEPAQQGR.R	N	35.03	2489.1321	22	-1.6	1245.5713	2	35.79	33	2018	OB4061.raw	3.62E4	1	1	99	120	Carbamidomethylation
R.RPFYSNAPQ(+.98)E(+28.03)IFIQQGR.G	N	34.53	2079.0537	17	1.4	694.0262	3	32.01	33	1791	OB4061.raw	7.07E3	1	1	82	98	Ethylation
R.LN(+.98)AQRPDNRLESEGGYIETWN(+.98)PNNQEFEC(+57.02)AGVALSR.L	N	34.46	4135.8931	36	-1.8	1034.9786	4	34.26	33	1923	OB4061.raw	0	0	0	37	72	Carbamidomethylation
R.LNAQRPDN(+.98)RLESEGGYIETWNPNNQEFEC(+57.02)AGVALSR.L	N	33.89	4134.9087	36	1.7	1034.7362	4	32.06	33	1949	OB4061.raw	7.3E5	1	1	37	72	Carbamidomethylation
R.GYFGLIFPGC(+57.02)P(+15.99)STYEEPAQQGRR.H	N	33.80	2645.2332	23	-2.0	882.7499	3	35.56	33	2002	OB4061.raw	0	0	0	99	121	Carbamidomethylation
R.RFN(+.98)LAGNHEQ(+.98)EFLRYQ(+.98)Q(+.98)QSR.R	N	33.64	2524.1731	20	0.4	632.0508	4	29.52	33	1633	OB4061.raw	6.27E4	1	1	196	215	Deamidation (NQ)
D.NRLESEGGYIETWNPN(+.98)N(+.98)QEFEC(+57.02)AGVALSR.L	N	33.60	3341.4895	29	8.0	1114.8461	3	32.45	33	1829	OB4061.raw	1.54E4	1	1	44	72	Carbamidomethylation
R.GYFGLIFPGC(+57.02)PSTYEEP(+15.99)AQQ(+.98)GRR.H	N	33.28	2646.2173	23	-3.1	883.0770	3	34.33	33	1927	OB4061.raw	0	0	0	99	121	Carbamidomethylation
R.LNAQ(+.98)RPDNRLESEGGYIETWNP(+15.99)NNQEFEC(+57.02)AGVALSR.L	N	33.23	4150.9038	36	3.1	1038.7365	4	32.59	33	1821	OB4061.raw	0	0	0	37	72	Carbamidomethylation

Peptide	Uniq	-10lgP	Mass	Length	ppm	m/z	z	RT	Fraction	Scan	Source File	Area Digest 11 traka	#Feature	#Feature Digest 11 traka	Start	End	PTM
S.ISFRQQ(+.98)PEEN(+.98)AC(+57.02)QFQ(+.98)R.L	N	32.96	2039.9006	16	-0.6	680.9738	3	28.00	33	1580	OB4061.raw	1.73E5	1	1	21	36	Deamidation (NQ); Carbamidomethylation
R.RFNLAGNHEQ(+.98)EFLRYQQQ(+.98)SR.R	N	32.49	2522.2051	20	-1.5	631.5576	4	28.58	33	1569	OB4061.raw	0	0	0	196	215	
R.LNAQ(+.98)RPDNRLESEGGYIETWN(+.98)PN(+.98)NQEFEC(+57.02)AGVALSR.L	N	32.40	4136.8770	36	2.9	1379.9702	3	32.28	33	1802	OB4061.raw	0	0	0	37	72	Carbamidomethylation
R.RFNLAGNHEQ(+.98)EFLRYQ(+.98)Q(+.98)QSR.R	N	32.35	2523.1890	20	-0.9	631.8040	4	28.96	33	1585	OB4061.raw	4.66E4	1	1	196	215	
R.LNAQRPDN(+.98)RLESEGGYIETWN(+.98)PNN(+.98)Q(+.98)EFEC(+57.02)AGVALSR.L	N	32.33	4137.8608	36	8.4	1035.4812	4	32.26	33	1889	OB4061.raw	1.07E6	1	1	37	72	Carbamidomethylation
R.LNAQRPDNRLESEGGYIETWN(+.98)PNN(+.98)Q(+.98)EFEC(+57.02)AGVALSR.L	N	32.16	4136.8770	36	6.7	1035.2334	4	34.10	33	1913	OB4061.raw	0	0	0	37	72	Carbamidomethylation
R.RFN(+.98)LAGN(+.98)HEQ(+.98)EFLR.Y	N	31.96	1732.8169	14	8.6	578.6179	3	29.66	33	1635	OB4061.raw	0	0	0	196	209	Deamidation (NQ)
R.N(+.98)ALRRPFYSN(+.98)APQ(+.98)EIFIQ(+.98)QGR.G	N	31.70	2508.2397	21	8.1	837.0939	3	31.77	33	1778	OB4061.raw	1.36E4	1	1	78	98	Deamidation (NQ)
R.GYFGLIFPGC(+57.02)PSTYEEP(+15.99)AQQGR.R	N	31.00	2489.1321	22	-5.5	830.7134	3	35.79	33	2026	OB4061.raw	1.77E4	1	1	99	120	Carbamidomethylation
R.GYFGLIFPGC(+57.02)P(+15.99)STYEEP(+15.99)AQ(+.98)QGRR.H	N	30.73	2662.2122	23	-3.5	888.4082	3	33.92	33	1899	OB4061.raw	2.53E4	1	1	99	121	Carbamidomethylation; Hydroxylation Pro
R.RPFYSNAPQ(+.98)EIFIQQ(+.98)GR.G	N	30.13	2052.0063	17	-3.1	685.0073	3	37.03	33	2092	OB4061.raw	0	0	0	82	98	
R.GYFGLIFP(+15.99)GC(+57.02)PSTYEEPAQQ(+.98)GRR.H	N	30.09	2646.2173	23	4.6	883.0838	3	34.74	33	1952	OB4061.raw	0	0	0	99	121	Carbamidomethylation
R.GYFGLIFPGC(+57.02)P(+15.99)STYEEPAQ(+.98)QGRR.H	N	30.00	2646.2173	23	3.6	883.0829	3	34.54	33	1940	OB4061.raw	0	0	0	99	121	Carbamidomethylation
R.RFN(+.98)LAGN(+.98)HEQ(+.98)EFLRYQQ(+.98)Q(+.98)SR.R	N	29.97	2525.1570	20	0.8	632.2970	4	29.72	33	1660	OB4061.raw	5.01E4	1	1	196	215	Deamidation (NQ)
S.ISFRQ(+.98)Q(+.98)PEEN(+.98)AC(+57.02)Q(+.98)FQ(+.98)R.L	N	29.57	2041.8687	16	-3.6	681.6277	3	28.86	33	1580	OB4061.raw	4.5E3	1	1	21	36	Deamidation (NQ); Carbamidomethylation
R.AGQ(+.98)EQ(+.98)ENEGGN(+.98)IFSGFTPEFLAQAFQVDDRQILQNLRGEN(+.98)ESDEQGAIVTVR.G	Y	29.38	5754.6831	52	-1.3	1439.6761	4	38.75	33	2197	OB4061.raw	0	0	0	249	300	
R.RPFYSNAPQ(+.98)E(+28.03)IFIQQ(+.98)GR.G	N	29.20	2080.0378	17	-0.4	694.3530	3	32.43	33	1811	OB4061.raw	0	0	0	82	98	
R.RPFYSNAPQ(+29.02)EIFIQQGR.G	N	29.06	2079.0537	17	1.4	694.0262	3	32.01	33	1786	OB4061.raw	7.07E3	1	1	82	98	
R.LNAQ(+.98)RPDNRLESEGGYIETWN(+.98)PN(+.98)NQ(+.98)EFEC(+57.02)AGVALSR.L	N	28.50	4137.8608	36	8.4	1035.4812	4	32.26	33	1913	OB4061.raw	1.07E6	1	1	37	72	Carbamidomethylation
R.NALRRPFYSNAPQ(+.98)EIFIQ(+.98)Q(+.98)GR.G	N	28.37	2507.2556	21	3.4	836.7620	3	33.21	33	1858	OB4061.raw	0	0	0	78	98	
R.RFDEGDLIAVPTGVALWM(+15.99)YNDHDTDVVAVSLTDTN(+.98)NNDNQ(+.98)LDQFPR.R	Y	28.36	5208.3892	46	2.1	1042.6873	5	36.09	33	2044	OB4061.raw	3.48E4	1	1	150	195	Oxidation (M)
R.LNAQRP(+15.99)DNRLESEGGYIETWNPNNQEFEC(+57.02)AGVALSR.L	N	27.91	4149.9199	36	1.0	1038.4883	4	32.51	33	1816	OB4061.raw	0	0	0	37	72	Carbamidomethylation
R.GYFGLIFPGCPST(+79.97)YEEPAQQGR.R	N	26.69	2496.0820	22	8.0	833.0413	3	35.30	33	1991	OB4061.raw	6.76E3	1	1	99	120	
R.RFDEGDLIAVPTGVALWM(+15.99)YNDHDTDVVAVSLTDTNN(+.98)NDNQ(+.98)LDQFPR.R	Y	26.48	5208.3892	46	2.1	1042.6873	5	36.09	33	2037	OB4061.raw	3.48E4	1	1	150	195	Oxidation (M)
R.RFDEGDLIAVPTGVALWM(+15.99)YN(+.98)DHDTDVVAVSLTDTNNNDN(+.98)Q(+.98)LDQFPR.R	Y	26.02	5209.3735	46	-1.0	1042.8810	5	36.34	33	2050	OB4061.raw	0	0	0	150	195	Oxidation (M)
R.RFDEGDLIAVPTGVALWMYN(+.98)DHDTDVVAVSLTDTN(+.98)NNDNQ(+.98)LDQFPR.R	Y	25.75	5193.3784	46	3.7	1299.3567	4	37.27	33	2097	OB4061.raw	8.04E4	1	1	150	195	
**total 109 peptides**																	

tr|Q6PSU3|Q6PSU3_ARAHY

back to list

| Protein Coverage | Supporting Peptides |

Protein Coverage:

1 MRGRVSPLML LLGILVLASV SATQAKSPYR KTENPCAQRC LQSCQQEPDD LKQKACESRC TKLEYDPRCV YDTGATNQRH

81 PPGERTRGRQ PGDYDDDRRQ PRREEGGRWG PAEPRERERE EDWRQPREDW RRPSHQQPRK IRPEGREGEQ EWGTPGSEVR

161 EETSR**NNPFY FPSRR**FSTRY GNQNGRIRVL QRFDQRSKQF QNLQNHRIVQ IEARPNTLVL PKHADADNIL VIQQGQATVT

241 VANGNNRK**SF NLDEGHALR**I PSGFISYILN RHDNQNLRVA K**ISMPVNTPG QFEDFFPASS RDQSSYLQGF SRNTLEAAFN**

321 **AEFNEIRRVL LEENAGGEQE ERGQR**RRSTR SSDNEGVIVK VSKEHVQELT KHAKSVSKKG SEEEDITNPI NLRDGEPDLS

401 NNFGRLFEVK PDKKNPQLQD LDMMLTCVEI KEGALMLPHF NSK**AMVIVVV NKGTGNLELV AVR**KEQQQRG RREQEWEEEE

481 EDEEEEGSNR EVRRYTARLK EGDVFIMPAA HPVAINASSE LHLLGFGINA ENNHRIFLAG DKDNVIDQIE KQAKDLAFPG

561 SGEQVEKLIK NQRESHFVSA

d Deamidation (NQ) (+0.98)
o Oxidation (M) (+15.99)

Supporting Peptides:

Peptide	Uniq	-10lgP	Mass	Length	ppm	m/z	z	RT	Fraction	Scan	Source File	Area Digest 11 traka	#Feature	#Feature Digest 11 traka	Start	End	PTM
K.SFNLDEGHALR.I	Y	72.51	1257.6101	11	0.9	629.8129	2	27.39	33	1494	OB4061.raw	6.44E3	1	1	249	259	
K.AM(+15.99)VIVVVNK.G	Y	47.71	987.5787	9	-0.3	494.7964	2	26.22	33	1432	OB4061.raw	3.54E3	1	1	444	452	Oxidation (M)
R.NTLEAAFNAEFNEIRR.V	Y	46.06	1893.9332	16	0.8	947.9746	2	33.24	33	1859	OB4061.raw	8.01E4	2	2	313	328	
R.NTLEAAFN(+.98)AEFNEIRR.V	Y	41.61	1894.9172	16	-0.6	948.4653	2	33.82	33	1894	OB4061.raw	9.83E3	1	1	313	328	
R.NNPFYFPSRR.F	Y	41.42	1296.6364	10	-0.3	649.3253	2	28.42	33	1563	OB4061.raw	1.26E4	1	1	166	175	
K.AM(+15.99)VIVVVNKGTGNLELVAVR.K	Y	38.52	2097.1980	20	-1.9	700.0720	3	32.04	33	1787	OB4061.raw	0	0	0	444	463	Oxidation (M)
K.ISM(+15.99)PVNTPGQ(+.98)FEDFFPASSRDQ(+.98)SSYLQ(+.98)GFSR.N	Y	32.30	3513.5669	31	3.6	1172.2004	3	34.61	33	1945	OB4061.raw	2.34E5	1	1	282	312	Oxidation (M); Deamidation (NQ)
K.ISM(+15.99)PVN(+.98)TPGQ(+.98)FEDFFPASSRDQ(+.98)SSYLQ(+.98)GFSR.N	Y	31.64	3514.5510	31	3.0	1172.5278	3	34.82	33	1957	OB4061.raw	0	0	0	282	312	Oxidation (M); Deamidation (NQ)
K.ISM(+15.99)PVNTPGQFEDFFPASSRDQSSYLQ(+.98)GFSR.N	Y	31.05	3511.5989	31	1.9	1171.5425	3	34.22	33	1925	OB4061.raw	4.02E4	1	1	282	312	Oxidation (M)
R.VLLEENAGGEQ(+.98)EERGQR.R	Y	30.05	1913.9078	17	0.2	638.9767	3	24.68	33	1341	OB4061.raw	9.24E3	1	1	329	345	
R.VLLEEN(+.98)AGGEQ(+.98)EERGQ(+.98)R.R	Y	29.98	1915.8759	17	-0.7	639.6321	3	25.68	33	1381	OB4061.raw	7.92E3	1	1	329	345	Deamidation (NQ)
K.ISM(+15.99)PVNTPGQFEDFFPASSRDQ(+.98)SSYLQ(+.98)GFSR.N	Y	26.46	3512.5830	31	2.2	1171.8708	3	34.42	33	1933	OB4061.raw	0	0	0	282	312	Oxidation (M)
**total 12 peptides**																	

P43237|ALL11_ARAHY
back to list

| Protein Coverage | Supporting Peptides |

Protein Coverage:

d Deamidation (NQ) (+0.98)
o Oxidation (M) (+15.99)

```
1   MRGRVSPLML LLGILVLASV SATQAKSPYR KTENPCAQRC LQSCQQEPDD LKQKACESRC TKLEYDPRCV YDTGATNQRH
81  PPGERTRGRQ PGDYDDDRRQ PRREEGGRWG PAEPRERERE EDWRQPREDW RRPSHQQPRK IRPEGREGEQ EWGTPGSEVR
161 EETSRNNPFY FPSRRFSTRY GNQNGRIRVL QRFDQRSKQF QNLQNHRIVQ IEARPNTLVL PKHADADNIL VIQQGQATVT
241 VANGNNRKSF NLDEGHALRI PSGFISYILN RHDNQNLRVA KISMPVNTPG QFEDFFPASS RDQSSYLQGF SRNTLEAAFN
321 AEFNEIRRVL LEENAGGEQE ERGQRRRSTR SSDNEGVIVK VSKEHVQELT KHAKSVSKKG SEEEDITNPI NLRDGEPDLS
401 NNFGRLFEVK PDKKNPQLQD LDMMLTCVEI KEGALMLPHF NSKAMVIVVV NKGTGNLELV AVRKEQQQRG RREQEWEEEE
481 EDEEEEGSNR EVRRYTARLK EGDVFIMPAA HPVAINASSE LHLLGFGINA ENNHRIFLAG DKDNVIDQIE KQAKDLAFPG
561 SGEQVEKLIK NQRESHFVSA RPQSQPSSSP EKEDQEEENQ GGKGPLLSIL KAFN
```

Supporting Peptides:

Peptide	Uniq	-10lgP	Mass	Length	ppm	m/z	z	RT	Fraction	Scan	Source File	Area Digest 11 traka	#Feature	#Feature Digest 11 traka	Start	End	PTM
K.SFNLDEGHALR.I	Y	72.51	1257.6101	11	0.9	629.8129	2	27.39	33	1494	OB4061.raw	6.44E3	1	1	249	259	
K.AM(+15.99)VIVVVNK.G	Y	47.71	987.5787	9	-0.3	494.7964	2	26.22	33	1432	OB4061.raw	3.54E3	1	1	444	452	Oxidation (M)
R.NTLEAAFNAEFNEIRR.V	Y	46.06	1893.9332	16	0.8	947.9746	2	33.24	33	1859	OB4061.raw	8.01E4	2	2	313	328	
R.NTLEAAFN(+.98)AEFNEIRR.V	Y	41.61	1894.9172	16	-0.6	948.4653	2	33.82	33	1894	OB4061.raw	9.83E3	1	1	313	328	
R.NNPFYFPSRR.F	Y	41.42	1296.6364	10	-0.3	649.3253	2	28.42	33	1563	OB4061.raw	1.26E4	1	1	166	175	
K.AM(+15.99)VIVVVNKGTGNLELVAVR.K	Y	38.52	2097.1980	20	-1.9	700.0720	3	32.04	33	1787	OB4061.raw	0	0	0	444	463	Oxidation (M)
K.ISM(+15.99)PVNTPGQ(+.98)FEDFFPASSRDQ(+.98)SSYLQ(+.98)GFSR.N	Y	32.30	3513.5669	31	3.6	1172.2004	3	34.61	33	1945	OB4061.raw	2.34E5	1	1	282	312	Oxidation (M); Deamidation (NQ)
K.ISM(+15.99)PVN(+.98)TPGQ(+.98)FEDFFPASSRDQ(+.98)SSYLQ(+.98)GFSR.N	Y	31.64	3514.5510	31	3.0	1172.5278	3	34.82	33	1957	OB4061.raw	0	0	0	282	312	Oxidation (M); Deamidation (NQ)
K.ISM(+15.99)PVNTPGQFEDFFPASSRDQSSYLQ(+.98)GFSR.N	Y	31.05	3511.5989	31	1.9	1171.5425	3	34.22	33	1925	OB4061.raw	4.02E4	1	1	282	312	Oxidation (M)
R.VLLEENAGGEQ(+.98)EERGQR.R	Y	30.05	1913.9078	17	0.2	638.9767	3	24.68	33	1341	OB4061.raw	9.24E3	1	1	329	345	
R.VLLEEN(+.98)AGGEQ(+.98)EERGQ(+.98)R.R	Y	29.98	1915.8759	17	-0.7	639.6321	3	25.68	33	1381	OB4061.raw	7.92E3	1	1	329	345	Deamidation (NQ)
K.ISM(+15.99)PVNTPGQFEDFFPASSRDQ(+.98)SSYLQ(+.98)GFSR.N	Y	26.46	3512.5830	31	2.2	1171.8708	3	34.42	33	1933	OB4061.raw	0	0	0	282	312	Oxidation (M)
total 12 peptides																	

tr|B3IXL2|B3IXL2_ARAHY
back to list

| Protein Coverage | Supporting Peptides |

Protein Coverage:

1 MRGRVSPLML LLGILVLASV SATQAKSPYR KTENPCAQRC LQSCQQEPDD LKQKACESRC TKLEYDPRCV YDTGATNQRH
81 PPGERTRGRQ PGDYDDDRRQ PRREEGGRWG PAEPRERERE EDWRQPREDW RRPSHQQPRK IRPEGREGEQ EWGTPGSEVR
161 EETSRNNPFY FPSRRFSTRY GNQNGRIRVL QRFDQRSKQF QNLQNHRIVQ IEARPNTLVL PKHADADNIL VIQQGQATVT
284 287 291 303 308
241 VANGNNRKSF NLDEGHALRI PSGFISYILN RHDNQNLRVA KISMPVNTPG QFEDFFPASS RDQSSYLQGF SRNTLEAAFN
334 339 344
321 AEFNEIRRVL LEENAGGEQE ERGQRRRSTR SSDNEGVIVK VSKEHVQELT KHAKSVSKKG SEEEDITNPI NLRDGEPDLS
445
401 NNFGRLFEVK PDKKNPQLQD LDMMLTCVEI KEGALMLPHF NSKAMVIVVV NKGTGNLELV AVRKEQQQRG RREQEWEEEE
481 EDEEEEGSNR EVRRYTARLK EGDVFIMPAA HPVAINASSE LHLLGFGINA ENNHRIFLAG DKDNVIDQIE KQAKDLAFPG
561 SGEQVEKLIK NQRESHFVSA RPQSQSPSSP EKEDQEEENQ GGKGPLLSIL KAFN

d Deamidation (NQ) (+0.98)
o Oxidation (M) (+15.99)

Supporting Peptides:

Peptide	Uniq	-10lgP	Mass	Length	ppm	m/z	z	RT	Fraction	Scan	Source File	Area Digest 11 traka	#Feature	#Feature Digest 11 traka	Start	End	PTM
K.SFNLDEGHALR.I	Y	72.51	1257.6101	11	0.9	629.8129	2	27.39	33	1494	OB4061.raw	6.44E3	1	1	249	259	
K.AM(+15.99)VIVVVNK.G	Y	47.71	987.5787	9	-0.3	494.7964	2	26.22	33	1432	OB4061.raw	3.54E3	1	1	444	452	Oxidation (M)
R.NTLEAAFNAEFNEIRR.V	Y	46.06	1893.9332	16	0.8	947.9746	2	33.24	33	1859	OB4061.raw	8.01E4	2	2	313	328	
R.NTLEAAFN(+.98)AEFNEIRR.V	Y	41.61	1894.9172	16	-0.6	948.4653	2	33.82	33	1894	OB4061.raw	9.83E3	1	1	313	328	
R.NNPFYFPSRR.F	Y	41.42	1296.6364	10	-0.3	649.3253	2	28.42	33	1563	OB4061.raw	1.26E4	1	1	166	175	
K.AM(+15.99)VIVVVNKGTGNLELVAVR.K	Y	38.52	2097.1980	20	-1.9	700.0720	3	32.04	33	1787	OB4061.raw	0	0	0	444	463	Oxidation (M)
K.ISM(+15.99)PVNTPGQ(+.98)FEDFFPASSRDQ(+.98)SSYLQ(+.98)GFSR.N	Y	32.30	3513.5669	31	3.6	1172.2004	3	34.61	33	1945	OB4061.raw	2.34E5	1	1	282	312	Oxidation (M); Deamidation (NQ)
K.ISM(+15.99)PVN(+.98)TPGQ(+.98)FEDFFPASSRDQ(+.98)SSYLQ(+.98)GFSR.N	Y	31.64	3514.5510	31	3.0	1172.5278	3	34.82	33	1957	OB4061.raw	0	0	0	282	312	Oxidation (M); Deamidation (NQ)
K.ISM(+15.99)PVNTPGQFEDFFPASSRDQSSYLQ(+.98)GFSR.N	Y	31.05	3511.5989	31	1.9	1171.5425	3	34.22	33	1925	OB4061.raw	4.02E4	1	1	282	312	Oxidation (M)
R.VLLEENAGGEQ(+.98)EERGQR.R	Y	30.05	1913.9078	17	0.2	638.9767	3	24.68	33	1341	OB4061.raw	9.24E3	1	1	329	345	
R.VLLEEN(+.98)AGGEQ(+.98)EERGQ(+.98)R.R	Y	29.98	1915.8759	17	-0.7	639.6321	3	25.68	33	1381	OB4061.raw	7.92E3	1	1	329	345	Deamidation (NQ)
K.ISM(+15.99)PVNTPGQFEDFFPASSRDQ(+.98)SSYLQ(+.98)GFSR.N	Y	26.46	3512.5830	31	2.2	1171.8708	3	34.42	33	1933	OB4061.raw	0	0	0	282	312	Oxidation (M)
**total 12 peptides**																	

tr|T2B9M0|T2B9M0_ARAHY

back to list

| Protein Coverage | Supporting Peptides |

Protein Coverage:

1 MSNFKSKYHD ELIANAAYIG TPGK**GILAAD ESTGTIGKR**L SSINVENVET NRRALRELLF TTPGALQYLS GVILFEETLY

81 QSTAAGKPFV EVLKEGGVLP GIKVDKGTVE LAGTNGETTT QGLDGLGQRC AKYYEAGARF AKWRAVLK**IG PTEPSELAIH**

161 **ENAYGLAR**YA AICQENGLVP IVEPEILVDG PHDIHKCAAV TERVLAACYK ALNDHHVLLE GTLLKPNMVT PGSDSAKVAP

241 EVVAEHTVRA LQR**TVPSAVP AVVFLSGGQS EEEATLNLNA INQVK**GKKPW SLSFSFGRAL QQSTLKAWSG KEENVKKAQE

321 AFLTRAKANS EATLGTYKGS ANLGEGASES LHVKDYKY

d Deamidation (NQ) (+0.98)

Modification sites: 162 d; 269 d; 277 d

Supporting Peptides:

Peptide	Uniq	-10lgP	Mass	Length	ppm	m/z	z	RT	Fraction	Scan	Source File	Area Digest 11 traka	#Feature	#Feature Digest 11 traka	Start	End	PTM
R.TVPSAVPAVVFLSGGQSEEEATLNLNAINQVK.G	Y	78.14	3281.7144	32	0.0	1094.9121	3	36.10	33	2035	OB4061.raw	0	0	0	254	285	
K.IGPTEPSELAIHENAYGLAR.Y	Y	65.53	2137.0803	20	-0.3	713.3672	3	30.01	33	1653	OB4061.raw	2.28E5	1	1	149	168	
R.TVPSAVPAVVFLSGGQ(+.98)SEEEATLNLN(+.98)AINQVK.G	Y	62.99	3283.6824	32	1.0	1095.5692	3	36.78	33	2077	OB4061.raw	0	0	0	254	285	Deamidation (NQ)
R.TVPSAVPAVVFLSGGQ(+.98)SEEEATLN(+.98)LNAINQVK.G	Y	50.97	3283.6824	32	5.5	1095.5741	3	37.13	33	2098	OB4061.raw	0	0	0	254	285	Deamidation (NQ)
R.TVPSAVPAVVFLSGGQ(+.98)SEEEATLNLNAINQVK.G	Y	49.88	3282.6982	32	5.1	1095.2456	3	36.18	33	2053	OB4061.raw	1.09E5	1	1	254	285	
R.TVPSAVPAVVFLSGGQSEEEATLNLN(+.98)AINQVK.G	Y	47.65	3282.6982	32	5.1	1095.2456	3	36.18	33	2061	OB4061.raw	1.09E5	1	1	254	285	
R.TVPSAVPAVVFLSGGQ(+.98)SEEEATLN(+.98)LNAINQ(+.98)VK.G	Y	46.48	3284.6663	32	8.4	1095.9053	3	36.92	33	2085	OB4061.raw	0	0	0	254	285	Deamidation (NQ)
K.GILAADESTGTIGKR.L	Y	40.82	1487.7943	15	-1.8	496.9378	3	26.07	33	1416	OB4061.raw	4.66E3	1	1	25	39	
K.IGPTEPSELAIHEN(+.98)AYGLAR.Y	Y	37.78	2138.0642	20	-0.9	713.6947	3	30.48	33	1695	OB4061.raw	2.38E4	1	1	149	168	Deamidation (NQ)
**total 9 peptides**																	

tr|E5G077|E5G077_ARAHY

back to list

| Protein Coverage | Supporting Peptides |

Protein Coverage:

1 MAKLLALSLC FCVLVLGASS VTFRQGGEEN ECQFQRLNAQ RPDNRIESEG GYIETWNPNN QEFQCAGVAL SRTVLRRNAL

- c Carbamidomethylation (+57.02)
- d Deamidation (NQ) (+0.98)
- P Propionamide (+71.04)

81 RRPFYSNAPL EIYVQQGSGY FGLIFPGCPS TYEEPAQEGR RYQSQKPSRR FQVGQDDPSQ QQQDSHQKVH RFDEGDLIAV

161 PTGVAFWMYN DEDTDVVTVT LSDTSSIHNQ LDQFPRRFYL AGNQEQEFLR YQQQQGSRPH YRQISPRVRG DEQENEGSNI

241 FSGFAQEFLQ HAFQVDRQTV ENLRGENERE EQGAIVTVKG GLRILSPDEE DESSRSPPNR REEFDEDRSR PQQRGKYDEN

321 RRGYKNGIEE TICSASVKKN LGRSSNPDIY NPQAGSLRSV NELDLPILGW LGLSAQHGTI YRNAMFVPHY TLNAHTIVVA

401 LNGRAHVQVV DSNGNRVYDE ELQEGHVLVV PQNFAVAAKA QSENYEYLAF KTDSRPSIAN QAGENSIIDN LPEEVVANSY

481 RLPREQARQL KNNNPFKFFV PPFDHQSMRE VA

Supporting Peptides:

Peptide	Uniq	-10lgP	Mass	Length	ppm	m/z	z	RT	Fraction	Scan	Source File	Area Digest 11 traka	#Feature	#Feature Digest 11 traka	Start	End	PTM
S.GYFGLIFPGC(+57.02)PSTYEEPA.Q	N	52.94	2003.8975	18	1.3	1002.9573	2	37.84	33	2143	OB4061.raw	1.17E4	1	1	99	116	Carbamidomethylation
R.SVNELDLPILGWLGLSAQ(+.98)HGTIYR.N	Y	44.67	2652.3911	24	1.7	885.1392	3	38.72	33	2212	OB4061.raw	6.47E4	2	2	359	382	Deamidation (NQ)
R.LNAQRPDN(+.98)RIESEGGYIETWNPN(+.98)N(+.98)Q(+.98)EFQC(+57.02)AGVALSR.T	Y	34.21	4136.8770	36	6.8	1035.2335	4	33.71	33	1889	OB4061.raw	0	0	0	37	72	Carbamidomethylation
R.SVNELDLPILGWLGLSAQHGTIYR.N	Y	34.03	2651.4070	24	2.3	884.8116	3	38.44	33	2170	OB4061.raw	1.78E4	1	1	359	382	
R.LNAQRPDNRIESEGGYIETWN(+.98)P(+15.99)NNQEFQC(+57.02)AGVALSR.T	Y	32.91	4149.9199	36	2.6	1038.4900	4	32.21	33	1797	OB4061.raw	0	0	0	37	72	Carbamidomethylation
R.LNAQRPDN(+.98)RIESEGGYIETWNPN(+.98)NQEFQC(+57.02)AGVALSR.T	Y	30.92	4134.9087	36	1.7	1034.7362	4	32.06	33	1889	OB4061.raw	7.3E5	1	1	37	72	Carbamidomethylation
R.LN(+.98)AQRPDNRIESEGGYIETWNP(+15.99)NN(+.98)Q(+.98)EFQC(+57.02)AGVALSR.T	Y	26.46	4151.8877	36	-6.2	1038.9728	4	31.93	33	1780	OB4061.raw	0	0	0	37	72	Carbamidomethylation
R.LN(+.98)AQRP(+15.99)DNRIESEGGYIETWNPNNQEFQC(+57.02)AGVALSR.T	Y	26.07	4149.9199	36	-0.3	1038.4869	4	32.89	33	1839	OB4061.raw	0	0	0	37	72	Carbamidomethylation
S.GYFGLIFPGC(+71.04)PSTYEEPAQEGR.R	Y	25.86	2488.1367	22	1.9	1245.0780	2	35.34	33	1989	OB4061.raw	0	0	0	99	120	Propionamide
**total 9 peptides**																	

tr|Q0GM57|Q0GM57_ARAHY

back to list

| Protein Coverage | Supporting Peptides |

Protein Coverage:

1 MAKLLALSLC FCVLVLGASS VTFRQGGEEN ECQFQR**LNAQ RPDNRIESEG GYIETWNPNN QEFQCAGVAL SR**TVLRRNAL

c Carbamidomethylation (+57.02)
d Deamidation (NQ) (+0.98)
P Propionamide (+71.04)

81 RRPFYSNAPL EIYVQQGS**GY FGLIFPGCPS TYEEPAQEGR** RYQSQKPSRR FQVGQDDPSQ QQQDSHQKVH RFDEGDLIAV

161 PTGVAFWMYN DEDTDVVTVT LSDTSSIHNQ LDQFPRRFYL AGNQEQEFLR YQQQQGSRPH YRQISPRVRG DEQENEGSNI

241 FSGFAQEFLQ HAFQVDRQTV ENLRGENERE EQGAIVTVKG GLRILSPDEE DESSRSPPSR REEFDEDRSR PQQRGKYDEN

321 RRGYKNGIEE TICSASVKKN LGRSSNPDIY NPQAGSLR**SV NELDLPILGW LGLSAQHGTI YR**NAMFVPHY TLNAHTIVVA

401 LNGRAHVQVV DSNGNRVYDE ELQEGHVLVV PQNFAVAAKA QSENYEYLAF KTDSRPSIAN LAGENSIIDN LPEEVVANSY

481 RLPREQARQL KNNNPFKFFV PPFDHQSMRE VA

Supporting Peptides:

Peptide	Uniq	-10lgP	Mass	Length	ppm	m/z	z	RT	Fraction	Scan	Source File	Area Digest 11 traka	#Feature	#Feature Digest 11 traka	Start	End	PTM
S.GYFGLIFPGC(+57.02)PSTYEEPA.Q	N	52.94	2003.8975	18	1.3	1002.9573	2	37.84	33	2143	OB4061.raw	1.17E4	1	1	99	116	Carbamidomethylation
R.SVNELDLPILGWLGLSAQ(+.98)HGTIYR.N	Y	44.67	2652.3911	24	1.7	885.1392	3	38.72	33	2212	OB4061.raw	6.47E4	2	2	359	382	Deamidation (NQ)
R.LNAQRPDN(+.98)RIESEGGYIETWNPN(+.98)N(+.98)Q(+.98)EFQC(+57.02)AGVALSR.T	Y	34.21	4136.8770	36	6.8	1035.2335	4	33.71	33	1889	OB4061.raw	0	0	0	37	72	Carbamidomethylation
R.SVNELDLPILGWLGLSAQHGTIYR.N	Y	34.03	2651.4070	24	2.3	884.8116	3	38.44	33	2170	OB4061.raw	1.78E4	1	1	359	382	
R.LNAQRPDNRIESEGGYIETWN(+.98)P(+15.99)NNQEFQC(+57.02)AGVALSR.T	Y	32.91	4149.9199	36	2.6	1038.4900	4	32.21	33	1797	OB4061.raw	0	0	0	37	72	Carbamidomethylation
R.LNAQRPDN(+.98)RIESEGGYIETWNPN(+.98)NQEFQC(+57.02)AGVALSR.T	Y	30.92	4134.9087	36	1.7	1034.7362	4	32.06	33	1889	OB4061.raw	7.3E5	1	1	37	72	Carbamidomethylation
R.LN(+.98)AQRPDNRIESEGGYIETWNP(+15.99)NN(+.98)Q(+.98)EFQC(+57.02)AGVALSR.T	Y	26.46	4151.8877	36	-6.2	1038.9728	4	31.93	33	1780	OB4061.raw	0	0	0	37	72	Carbamidomethylation
R.LN(+.98)AQRP(+15.99)DNRIESEGGYIETWNPNNQEFQC(+57.02)AGVALSR.T	Y	26.07	4149.9199	36	-0.3	1038.4869	4	32.89	33	1839	OB4061.raw	0	0	0	37	72	Carbamidomethylation
S.GYFGLIFPGC(+71.04)PSTYEEPAQEGR.R	Y	25.86	2488.1367	22	1.9	1245.0780	2	35.34	33	1989	OB4061.raw	0	0	0	99	120	Propionamide
**total 9 peptides**																	

tr|Q6IWG5|Q6IWG5_ARAHY

back to list

| **Protein Coverage** | **Supporting Peptides** |

Protein Coverage:

c	Carbamidomethylation (+57.02)
d	Deamidation (NQ) (+0.98)
p	Propionamide (+71.04)

```
  1  KLLALSLCFC VLVLGASSVT FRQGGEENEC QFQRLNAQRP DNRIESEGGY IETWNPNNQE FQCAGVALSR TVLRRNALRR
 81  PFYSNAPLEI YVQQGSGYFG LIFPGCPSTY EEPAQEGRRY QSQKPSRRFQ VGQDDPSQQQ QDSHQKVHRF DEGDLIAVPT
161  GVAFWMYNDE DTDVVTVTLS DTSSIHNQLD QFPRRFYLAG NQEQEFLRYQ QQQGSRPHYR QISPRVRGDE QENEGSNIFS
241  GFAQEFLQHA FQVDRQTVEN LRGENEREEQ GAIVTVKGGL RILSPDEEDE SSRSPPSRRE EFDEDRSRPQ QRGKYDENRR
321  GYKNGIEETI CSASVKKNLG RSSNPDIYNP QAGSLRSVNE LDLPILGWLG LSAQHGTIYR NAMFVPHYTL NAHTIVVALN
401  GRAHVQVVDS NGNRVYDEEL QEGHVLVVPQ NFAVAAKAQS ENYEYLAFKT DSRPSIANLA GENSIIDNLP EEVVANSYRL
481  PREQARQLKN NNPFKFFVPP FDHQSMREVA
```

Modification markers: 63 c; 106 c p; 374 d

Supporting Peptides:

Peptide	Uniq	-10lgP	Mass	Length	ppm	m/z	z	RT	Fraction	Scan	Source File	Area Digest 11 traka	#Feature	#Feature Digest 11 traka	Start	End	PTM
S.GYFGLIFPGC(+57.02)PSTYEEPA.Q	N	52.94	2003.8975	18	1.3	1002.9573	2	37.84	33	2143	OB4061.raw	1.17E4	1	1	97	114	Carbamidomethylation
R.SVNELDLPILGWLGLSAQ(+.98)HGTIYR.N	Y	44.67	2652.3911	24	1.7	885.1392	3	38.72	33	2212	OB4061.raw	6.47E4	2	2	357	380	Deamidation (NQ)
R.LNAQRPDN(+.98)RIESEGGYIETWNPN(+.98)N(+.98)Q(+.98)EFQC(+57.02)AGVALSR.T	Y	34.21	4136.8770	36	6.8	1035.2335	4	33.71	33	1889	OB4061.raw	0	0	0	35	70	Carbamidomethylation
R.SVNELDLPILGWLGLSAQHGTIYR.N	Y	34.03	2651.4070	24	2.3	884.8116	3	38.44	33	2170	OB4061.raw	1.78E4	1	1	357	380	
R.LNAQRPDNRIESEGGYIETWN(+.98)P(+15.99)NNQEFQC(+57.02)AGVALSR.T	Y	32.91	4149.9199	36	2.6	1038.4900	4	32.21	33	1797	OB4061.raw	0	0	0	35	70	Carbamidomethylation
R.LNAQRPDN(+.98)RIESEGGYIETWNPN(+.98)NQEFQC(+57.02)AGVALSR.T	Y	30.92	4134.9087	36	1.7	1034.7362	4	32.06	33	1889	OB4061.raw	7.3E5	1	1	35	70	Carbamidomethylation
R.LN(+.98)AQRPDNRIESEGGYIETWNP(+15.99)NN(+.98)Q(+.98)EFQC(+57.02)AGVALSR.T	Y	26.46	4151.8877	36	-6.2	1038.9728	4	31.93	33	1780	OB4061.raw	0	0	0	35	70	Carbamidomethylation
R.LN(+.98)AQRP(+15.99)DNRIESEGGYIETWNPNNQEFQC(+57.02)AGVALSR.T	Y	26.07	4149.9199	36	-0.3	1038.4869	4	32.89	33	1839	OB4061.raw	0	0	0	35	70	Carbamidomethylation
S.GYFGLIFPGC(+71.04)PSTYEEPAQEGR.R	Y	25.86	2488.1367	22	1.9	1245.0780	2	35.34	33	1989	OB4061.raw	0	0	0	97	118	Propionamide
**total 9 peptides**																	

tr|A0A0A6ZDT0|A0A0A6ZDT0_ARAHY

back to list

| Protein Coverage | Supporting Peptides |

Protein Coverage:

```
  1  MLVHYQIALF HLPRSSTTGL GIVEGLMTAV HSITATQKTV DGPSSKDWRG GRAASFNIIP SSTGAAKAVG KVLPSLNGKL
 81  TGMAFRVPTV DVSVVDLTVR LEKPATYDEI KQAIKEESEG KLKVILGYTE DDVVSTDFVG DSRSSIFDAK AGIALSKNFV
161  KIVSWYDNEW GYSTRVVDLV VHIAKQ
```

Supporting Peptides:

Peptide	Uniq	-10lgP	Mass	Length	ppm	m/z	z	RT	Fraction	Scan	Source File	Area Digest 11 traka	#Feature	#Feature Digest 11 traka	Start	End	PTM
K.IVSWYDNEWGYSTR.V	Y	61.73	1774.7950	14	1.8	888.4064	2	32.85	33	1841	OB4061.raw	6.8E3	1	1	162	175	
**total 1 peptides**																	

tr|A0A0A6ZDP1|A0A0A6ZDP1_ARAHY

back to list

| **Protein Coverage** | **Supporting Peptides** |

Protein Coverage:

```
  1  MLVHYQIALF HLPRSSTTGL GIVEGLMTTV HSITATQKTV DGPSSKDWRG GRAASFNIIP SSTGAAKAVG KVLPSLNGKL
 81  TGMAFRVPTV DVSVVDLTVR LEKPATYDEI KQAIKEESEG KLKGILGYTE DDVVSTDFVG DSRSSIFDAK AGIALSKNFV
161  KIVSWYDNEW GYSTRVVDLI VHIAKQ
```

Supporting Peptides:

Peptide	Uniq	-10lgP	Mass	Length	ppm	m/z	z	RT	Fraction	Scan	Source File	Area Digest 11 traka	#Feature	#Feature Digest 11 traka	Start	End	PTM
K.IVSWYDNEWGYSTR.V	Y	61.73	1774.7950	14	1.8	888.4064	2	32.85	33	1841	OB4061.raw	6.8E3	1	1	162	175	
**total 1 peptides**																	

Peptide List

Prepared with PEAKS ™ (bioinfor.com)

Summary

1. Notes Gastric Digest Raw peanut Band #12 41-41 kDa (PTM495)

2. Result Statistics

Figure 1. False discovery rate (FDR) curve. X axis is the number of peptide-spectrum matches (PSM) being kept. Y axis is the corresponding FDR.

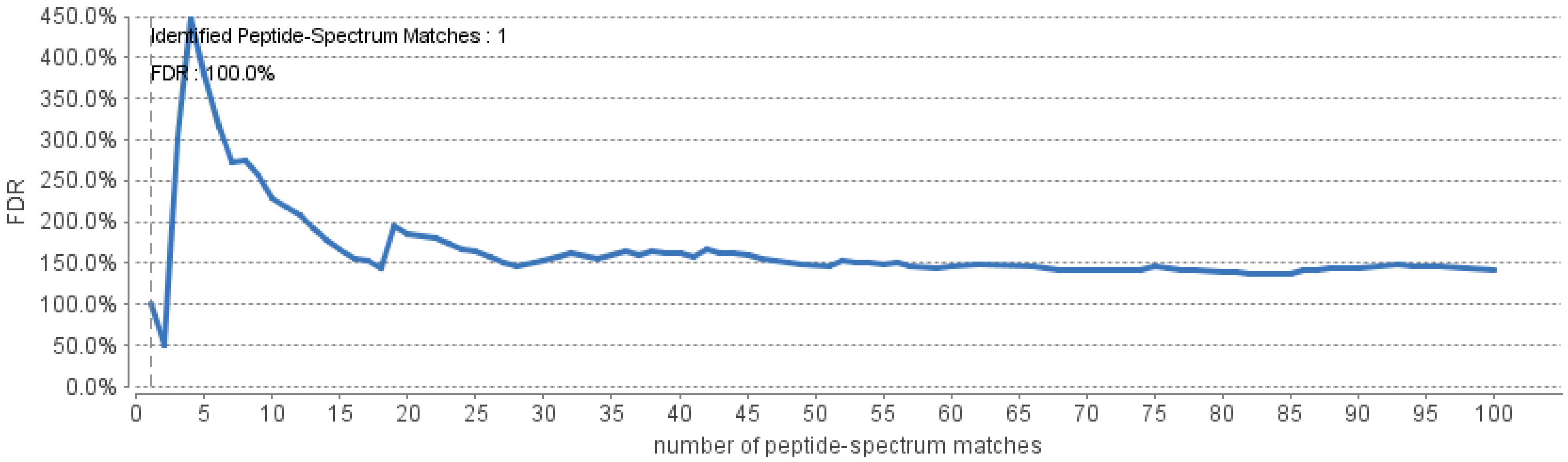

Figure 2. PSM score distribution. **(a)** Distribution of PEAKS peptide score; **(b)** Scatterplot of PEAKS peptide score versus precursor mass error.

(a)

(b)

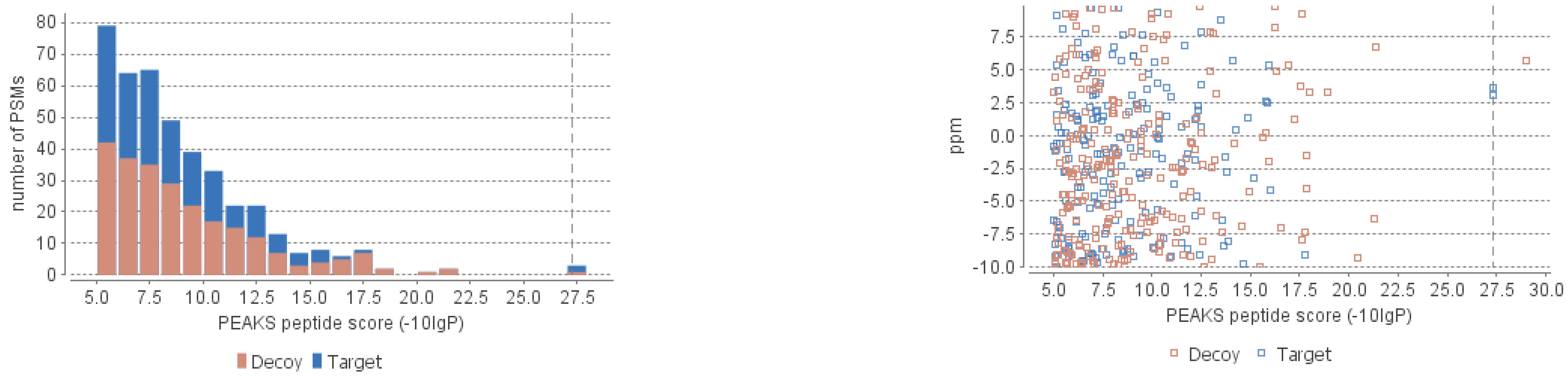

Figure 3. Distribution of peptide feature detection. **(a)** Feature m/z distribution; **(b)** Feature RT distribution

(a) **(b)**

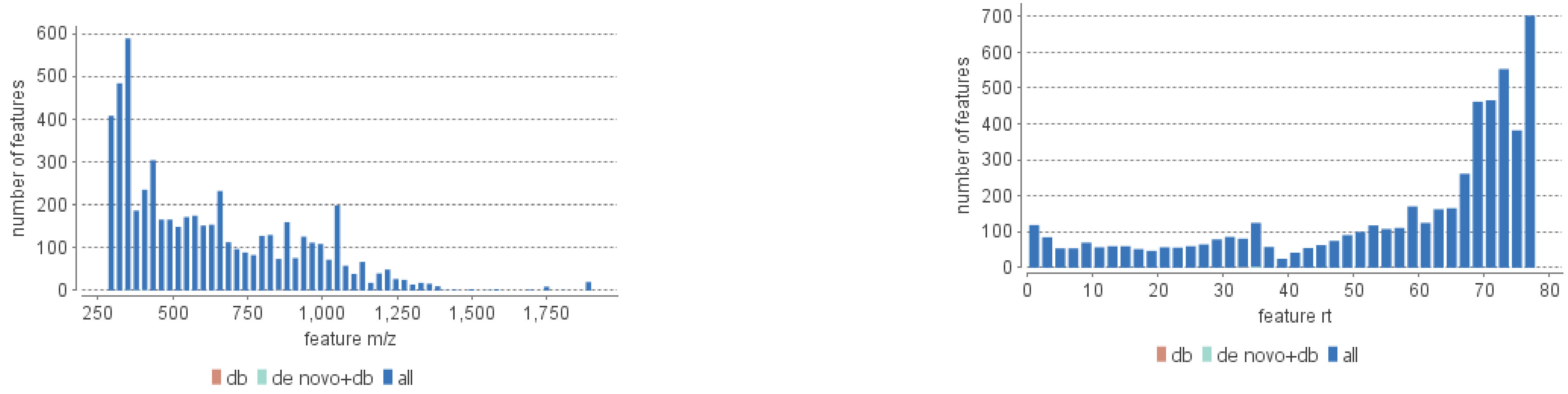

Figure 4. Distribution of identified peptide features. **(a)** Feature abundance distribution **(b)** *De novo* sequencing validation.

(a)

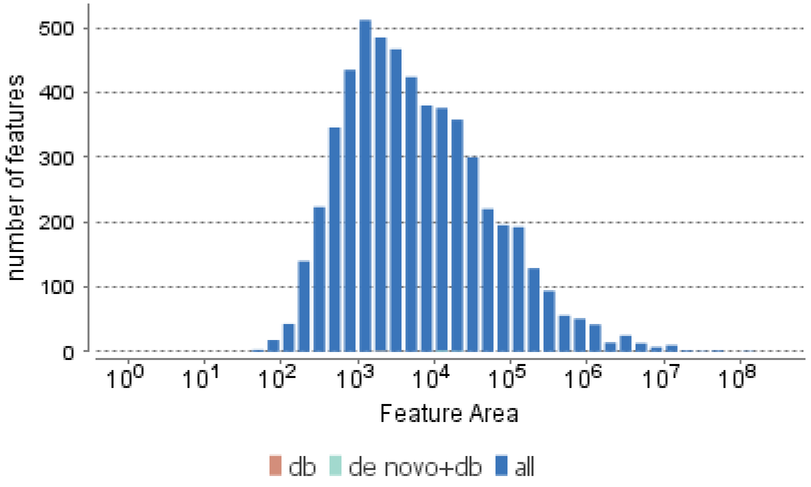

(b)

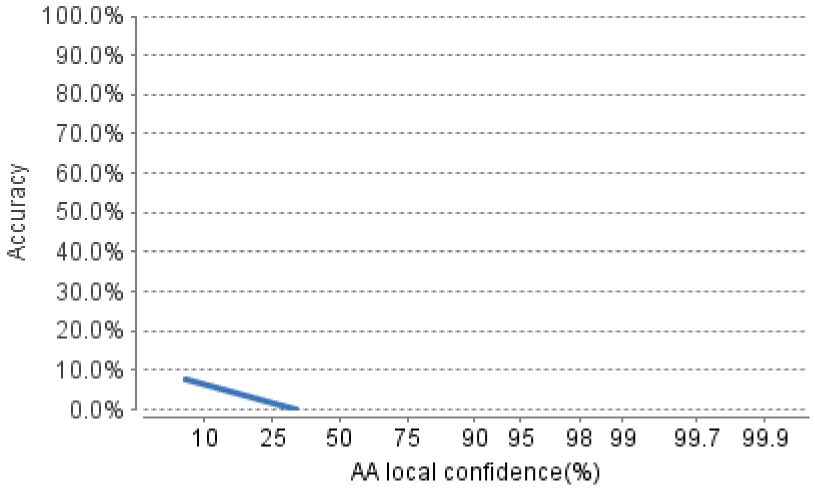

Table 1. Statistics of data.

# of MS scans	2835
# of MS/MS scans	1670
# of Features	5564
# of Chimera scans	247

Table 2. Result filtration parameters.

Peptide -10lgP	≥27.3
PTM Ascore	≥20
Protein -10lgP	≥20
Proteins unique peptides	≥1
De novo ALC Score	≥50%

Table 4. PTM profile.

**Name**	**ΔMass**	**Position**	**#PSM**	**-10lgP**	**Abundance**	**AScore**
Deamidation	.98	Q	1	27.34	1.65E3	1000.00
Carbamidomethyl	57.02	C	1	27.34	1.65E3	1000.00

Table 3. Statistics of filtered result.

Peptide-Spectrum Matches	1
Peptide sequences	1
Protein groups	1
Proteins	9
Proteins (#Unique Peptides)	0 (>2); 0 (=2); 9 (=1);
FDR (Peptide-Spectrum Matches)	100.0%
FDR (Peptide Sequences)	100.0%
FDR (Protein Group)	100.0%
De Novo Only Spectra	6
# of identified Features	4
# of identified MS/MS scans	1

3. Experiment Control

Figure 5. Precursor mass error of peptide-spectrum matches (PSM) in filtered result. **(a)** Distribution of precursor mass error in ppm; **(b)** Scatterplot of precursor m/z versus precursor mass error in ppm.

(a)

(b)

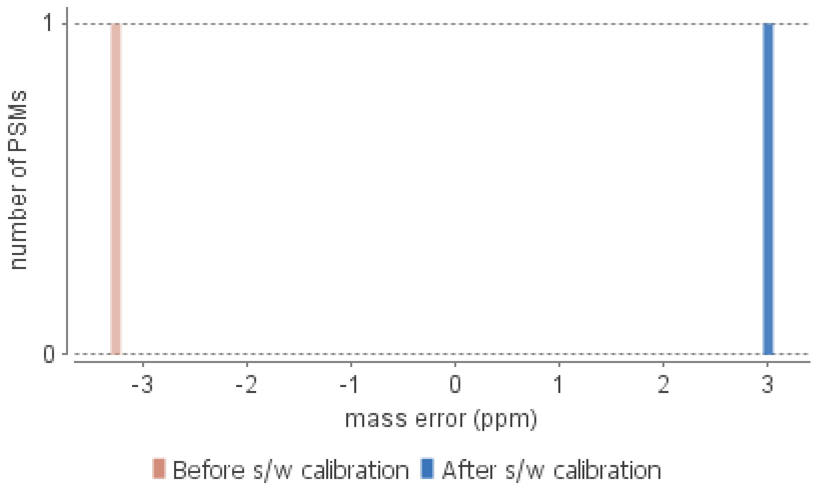

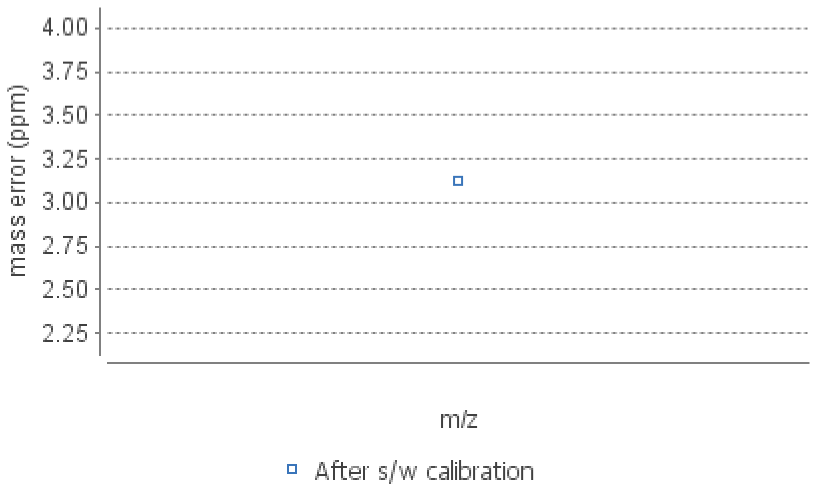

Table 5. Number of identified peptides in each sample by the number of missed cleavages

Missed Cleavages	0	1	2	3	4+
Digest 12. traka 45 kDa	0	1	0	0	0

4. Other Information

Table 6. Search parameters.

Search Engine Name: PEAKS
Parent Mass Error Tolerance: 10.0 ppm
Fragment Mass Error Tolerance: 0.5 Da
Precursor Mass Search Type: monoisotopic
Enzyme: Trypsin
Max Missed Cleavages: 2
Digest Mode: Unspecific

Table 7. Instrument parameters.

Fractions: OB4062.raw
Ion Source: ESI(nano-spray)
Fragmentation Mode: CID, CAD(y and b ions)
MS Scan Mode: FT-ICR/Orbitrap
MS/MS Scan Mode: Linear Ion Trap

Fixed Modifications:
  Carbamidomethylation: 57.02
Variable Modifications:
  Deamidation (NQ): 0.98
  Oxidation (M): 15.99
  Hydroxylation Pro: 15.99
  Acetylation (K): 42.01
  Acetylation (Protein N-term): 42.01
  Acetylation (N-term): 42.01
  Amidation: -0.98
  Beta-methylthiolation: 45.99
  and 305 more...
Max Variable PTM Per Peptide: 5
Database: Uniprot_Peanut-3818_Jul18
Taxon: All
Contaminant Database: contaminantsMQ_mar19
Searched Entry: 1723
FDR Estimation: Enabled
De novo score (ALC%) threshold: 15
Peptide hit threshold (-10logP): 30.0
Peaks run ID: 485
Merge Options: no merge
Precursor Options: corrected
Charge Options: no correction
Filter Charge: 2 - 8
Process: true
Associate chimera: yes

Protein List

Protein Accession Contains:

Protein Description Contains:

Peptide Sample Area >=

Protein Ptm Contains:

Protein Group	Protein ID	Accession	-10lgP	Coverage (%)	Coverage (%) Digest 12. traka 45 kDa	Area Digest 12. traka 45 kDa	#Peptides	#Unique	#Spec Digest 12. traka 45 kDa	PTM	Avg. Mass	Description
**1**	20576	tr|Q9FZ11|Q9FZ11_ARAHY	40.98	4	4	1.65E3	1	1	1	Y	60449	Gly1 OS=Arachis hypogaea OX=3818 GN=Gly1 PE=2 SV=1
**1**	20578	tr|B5TYU1|B5TYU1_ARAHY	40.98	4	4	1.65E3	1	1	1	Y	60624	Arachin Arah3 isoform OS=Arachis hy pogaea OX=3818 PE=1 SV=1
**1**	20579	tr|Q5I6T2|Q5I6T2_ARAHY	40.98	4	4	1.65E3	1	1	1	Y	60736	Arachin Ahy-4 OS=Arachis hypogaea OX=3818 PE=2 SV=1
**1**	20581	tr|Q647H3|Q647H3_ARAHY	40.98	4	4	1.65E3	1	1	1	Y	61532	Arachin Ahy-2 OS=Arachis hypogaea OX=3818 PE=2 SV=1
**1**	20574	tr|A1DZF0|A1DZF0_ARAHY	40.98	4	4	1.65E3	1	1	1	Y	60375	Arachin 6 OS=Arachis hypogaea OX=3 818 PE=2 SV=1
**1**	20577	tr|Q9SQH7|Q9SQH7_ARAHY	40.98	4	4	1.65E3	1	1	1	Y	61011	Glycinin OS=Arachis hypogaea OX=38 18 GN=Arah4 PE=2 SV=1
**1**	20580	tr|Q647H4|Q647H4_ARAHY	40.98	4	4	1.65E3	1	1	1	Y	61506	Arachin Ahy-1 OS=Arachis hypogaea OX=3818 PE=2 SV=1
**1**	20582	tr|Q8LKN1|Q8LKN1_ARAHY	40.98	4	4	1.65E3	1	1	1	Y	61738	Allergen Arah3/Arah4 OS=Arachis hyp ogaea OX=3818 PE=3 SV=1
**1**	20575	tr|Q8LL03|Q8LL03_ARAHY	40.98	11	11	1.65E3	1	1	1	Y	25499	Trypsin inhibitor (Fragment) OS=Arac his hypogaea OX=3818 PE=2 SV=1
**total 9 proteins**												

tr|Q9FZ11|Q9FZ11_ARAHY
back to list

| Protein Coverage | Supporting Peptides |

Protein Coverage:

1 MIRGRLALSV CFCFLVLGAS SISFRQQPEE NACQFQRLNA QRPDNRLESE GGYIETWNPN NQEFECAGVA LSRLVLRRNA

81 LRRPFYSNAP QEIFIQQGR**G YFGLIFPGCP STYEEPAQQG RR**HQSQRAPR RFEGEDQSQQ QQQDSHQKVR RFDEGDLIAV

161 PTGVALWMFN DHDTDVVAVS LTDTNNNDNQ LDQFPRRFNL AGNHEQEFLR YQQQSRRRSL PYSPYSPQSQ PRQEEREFSP

241 RGQHSRRERA GQEEENEGGN IFSGFTPEFL AQAFQVDDRQ IVQNLRGENE SEEEGAIVTV KGGLRILSPD RKRGADEEEE

321 YDEDEYEYDE EDRRRGRGSR GRGNGIEETI CTASVKKNIG RNRSPDIYNP QAGSLKTAND LNLLILRWLG LSAEYGNLYR

401 NALFVPHYNT NAHSIIYALR GRAHVQVVDS NGNRVYDEEL QEGHVLVVPQ NFAVAGKSQS DNFEYVAFKT DSRPNIANFA

481 GENSIIDNLP EEVVANSYGL PREQARQLKN NNPFKFFVPP SQQSLRAVA

c Carbamidomethylation (+57.02)
d Deamidation (NQ) (+0.98)

Supporting Peptides:

Peptide	Uniq	-10lgP	Mass	Length	ppm	m/z	z	RT	Fraction	Scan	Source File	Area Digest 12. traka 45 kDa	#Feature	#Feature Digest 12. traka 45 kDa	Start	End	PTM
R.GYFGLIFPGC(+57.02)PSTYEEPAQ(+.98)Q(+.98)GRR.H	Y	27.34	2631.2063	23	3.1	878.0731	3	33.17	34	1904	OB4062.raw	1.65E3	1	1	100	122	Carbamidomethylation; Deamidation (NQ)
**total 1 peptides**																	

tr|B5TYU1|B5TYU1_ARAHY

back to list

| Protein Coverage | Supporting Peptides |

Protein Coverage:

1	MAKLLELSFC	FCFLVLGASS	ISFRQQPEEN	ACQFQRLNAQ	RPDNRIESEG	GYIETWNPNN	QEFECAGVAL	SRLVLRRNAL	
81	RRPFYSNAPQ	EIFIQQGRGY	FGLIFPGCPS	TYEEPAQQGR	RYQSQRPPRR	LQEEDQSQQQ	QDSHQKVHRF	NEGDLIAVPT	
161	GVAFWLYNDH	DTDVVAVSLT	DTNNNDNQLD	QFPRRFNLAG	NHEQEFLRYQ	QQSRQSRRRS	LPYSPYSPQS	QPRQEEREFS	
241	PRGQHSRRER	AGQEEENEGG	NIFSGFTPEF	LAQAFQVDDR	QIVQNLRGEN	ESEEQGAIVT	VRGGLRILSP	DRKRGADEEE	
321	EYDEDEYEYD	EEDRRRGRGS	RGSGNGIEET	ICTATVKKNI	GRNRSPDIYN	PQAGSLKTAN	ELNLLILRWL	GLSAEYGNLY	
401	RNALFVPHYN	TNAHSIIYAL	RGRAHVQVVD	SNGNRVYDEE	LQEGHVLVVP	QNFAVAGKSQ	SDNFEYVAFK	TDSRPSIANL	
481	AGENSVIDNL	PEEVVANSYG	LPREQARQLK	NNNPFKFFVP	PSQQSPRAVA				

c Carbamidomethylation (+57.02)
d Deamidation (NQ) (+0.98)

108 c; 117 d; 118 d

Supporting Peptides:

Peptide	Uniq	-10lgP	Mass	Length	ppm	m/z	z	RT	Fraction	Scan	Source File	Area Digest 12. traka 45 kDa	#Feature	#Feature Digest 12. traka 45 kDa	Start	End	PTM
R.GYFGLIFPGC(+57.02)PSTYEEPAQ(+.98)Q(+.98)GRR.Y	Y	27.34	2631.2063	23	3.1	878.0731	3	33.17	34	1904	OB4062.raw	1.65E3	1	1	99	121	Carbamidomethylation; Deamidation (NQ)
**total 1 peptides**																	

tr|Q5I6T2|Q5I6T2_ARAHY
back to list

| Protein Coverage | Supporting Peptides |

Protein Coverage:

1 MAKLLELSFC FCFLVLGASS ISFRQQPEEN ACQFQRLNAQ RPDNRIESEG GYIETWNPNN QEFECAGVAL SRLVLRRNAL

81 RRPFYSNAPQ EIFIQQGRGY FGLIFPGCPS TYEEPAQQGR RSQSQRPPRR LQGEDQSQQQ QDSHQKVHRF DEGDLIAVPT

161 GVAFWLYNDH DTDVVAVSLT DTNNNDNQLD QFPRRFNLAG NHEQEFLRYQ QQSRQSRRRS LPYSPYSPQS QPRQEEREFS

241 PRGQHSRRER AGQEEENEGG NIFSGFTPEF LEQAFQVDDR QIVQNLRGEN ESEEEGAIVT VRGGLRILSP DRKRGADEEE

321 EYDEDEYEYD EEDRRRGRGS RGRGNGIEET ICTASVKKNI GRNRSPDIYN PQAGSLKTAN DLNLLILRWL GLSAEYGNLY

401 RNALFVPHYN TNAHSIIYAL RGRAHVQVVD SNGNRVYDEE LQEGHVLVVP QNFAVAGKSQ SDNFEYVAFK TDSRPSIANL

481 AGENSVIDNL PEEVVANSYG LQREQARQQL KNNNPFKFFV PPSQQSPRAV A

c Carbamidomethylation (+57.02)
d Deamidation (NQ) (+0.98)

Supporting Peptides:

Peptide	Uniq	-10lgP	Mass	Length	ppm	m/z	z	RT	Fraction	Scan	Source File	Area Digest 12. traka 45 kDa	#Feature	#Feature Digest 12. traka 45 kDa	Start	End	PTM
R.GYFGLIFPGC(+57.02)PSTYEEPAQ(+.98)Q(+.98)GRR.S	Y	27.34	2631.2063	23	3.1	878.0731	3	33.17	34	1904	OB4062.raw	1.65E3	1	1	99	121	Carbamidomethylation; Deamidation (NQ)
**total 1 peptides**																	

tr|Q647H3|Q647H3_ARAHY

back to list

| Protein Coverage | Supporting Peptides |

Protein Coverage:

1 MAKLLALSVC FCFLVLGASS ISFRQQPEEN ACQFQRLNAQ RPDNRLESEG GYIETWNPNN QEFECAGVAL SRLVLRRNAL

c Carbamidomethylation (+57.02)
d Deamidation (NQ) (+0.98)

108 c; 117 118 d d

81 RRPFYSNAPQ EIFIQQGR**GY FGLIFPGCPS TYEEPAQQGR R**HQSQRAPRR FEGEDQSQQQ QQDSHQKVRR FDEGDLIAVP

161 TGVALWMYND HDTDVVAVSL TDTNNNDNQL DQFPRRFNLA GNHEQEFLRY QQQSRRRSLP YSPYSPQSQP RQEEREFSPR

241 GQHSRRERAG QEQENEGGNI FSGFTPEFLA QAFQVDDRQI LQNLRGENES DEQGAIVTVR GGLRILSPDR KRRQQYERPD

321 EEEEYDEDEY EYDEEERQQD RRRGRGSRGR GNGIEETICT ASVKKNIGRN RSPDIYNPQA GSLKTANDLN LLILRWLGLS

401 AEYGNLYRNA LFVPHYNTNA HSIIYALRGR AHVQVVDSNG NRVYDEELQE GHVLVVPQNF AVAGKSQSDN FEYVAFKTDS

481 RPSIANLAGE NSIIDNLPEE VVANSYGLPR EQARQLKNNN PFKFFVPPSQ QSLGAVA

Supporting Peptides:

Peptide	Uniq	-10lgP	Mass	Length	ppm	m/z	z	RT	Fraction	Scan	Source File	Area Digest 12. traka 45 kDa	#Feature	#Feature Digest 12. traka 45 kDa	Start	End	PTM
R.GYFGLIFPGC(+57.02)PSTYEEPAQ(+.98)Q(+.98)GRR.H	Y	27.34	2631.2063	23	3.1	878.0731	3	33.17	34	1904	OB4062.raw	1.65E3	1	1	99	121	Carbamidomethylation; Deamidation (NQ)
**total 1 peptides**																	

tr|A1DZF0|A1DZF0_ARAHY

back to list

| Protein Coverage | Supporting Peptides |

Protein Coverage:

1 MAKLLELSFC FCFLVLGASS ISFRQQPEEN ACQFQRLNAQ RPDNRIESEG GYIETWNPNN QEFECAGVAL SRLVLRRNAL

81 RRPFYSNAPQ EIFIQQGR**GY FGLIFPGCPS TYEEPAQQGR R**YQSQRPPRR LQEEDQSQQQ QDSHQKVHRF NEGDLIAVPT

161 GVAFWLYNDH DTDVVAVSLT DTNNNDNQLD QFPRRFNLAG NHEQEFLRYQ QQSRQSRRRS LPLSPYSPQP GQEDREFSPQ

241 GQHGRRERAG QEQENEGGNI FSGFTSEFLA QAFQVDDRQI VQNLRGENES EEQGAIVTVK GGLRILSPDR KSPDEEEEYD

321 EDEYAEEERQ QDRRRGRGSR GSGNGIEETI CTATVKKNIG RNRSPDIYNP QAGSLKTANE LNLLILRWLG LSAEYGNLYR

401 NALFVPHYNT NAHSIIYALR GRAHVQVVDS NGNRVYDEEL QEGHVLVVPQ NFAVAGKSQS ENFEYVAFKT DSRPSIANLA

481 GENSFIDNLP EEVVANSYGL PREQARQLKN NNPFKFFVPP FQQSPRAVA

c Carbamidomethylation (+57.02)
d Deamidation (NQ) (+0.98)

Supporting Peptides:

Peptide	Uniq	-10lgP	Mass	Length	ppm	m/z	z	RT	Fraction	Scan	Source File	Area Digest 12. traka 45 kDa	#Feature	#Feature Digest 12. traka 45 kDa	Start	End	PTM
R.GYFGLIFPGC(+57.02)PSTYEEPAQ(+.98)Q(+.98)GRR.Y	Y	27.34	2631.2063	23	3.1	878.0731	3	33.17	34	1904	OB4062.raw	1.65E3	1	1	99	121	Carbamidomethylation; Deamidation (NQ)
**total 1 peptides**																	

tr|Q9SQH7|Q9SQH7_ARAHY

back to list

| Protein Coverage | Supporting Peptides |

Protein Coverage:

1 MAKLLELSFC FCFLVLGASS ISFRQQPEEN ACQFQRLNAQ RPDNRIESEG GYIETWNPNN QEFECAGVAL SRLVLRRNAL

81 RRPFYSNAPQ EIFIQQGR**GY FGLIFPGCPS TYEEPAQQGR R**YQSQRPPRR LQEEDQSQQQ QDSHQKVHRF NEGDLIAVPT

161 GVAFWLYNDH DTDVVAVSLT DTNNNDNQLD QFPRRFNLAG NHEQEFLRYQ QQSRQSRRRS LPYSPYSPHS RPRREEREFR

241 PRGQHSRRER AGQEEEDEGG NIFSGFTPEF LEQAFQVDDR QIVQNLWGEN ESEEEGAIVT VRGGLRILSP DGTRGADEEE

321 EYDEDQYEYH EQDGRRGRGS RGGGNGIEET ICTACVKKNI GGNRSPHIYD PQRWFTQNCH DLNLLILRWL GLSAEYGNLY

401 RNALFVPHYN TNAHSIIYAL RGRAHVQVVD SNGNRVYDEE LQEGHVLVVP QNFAVAGKSQ SENFEYVAFK TDSRPSIANF

481 AGENSFIDNL PEEVVANSYG LPREQARQLK NNNPFKFFVP PFQQSPRAVA

c Carbamidomethylation (+57.02)
d Deamidation (NQ) (+0.98)

108 c; 117 d, 118 d

Supporting Peptides:

Peptide	Uniq	-10lgP	Mass	Length	ppm	m/z	z	RT	Fraction	Scan	Source File	Area Digest 12. traka 45 kDa	#Feature	#Feature Digest 12. traka 45 kDa	Start	End	PTM
R.GYFGLIFPGC(+57.02)PSTYEEPAQ(+.98)Q(+.98)GRR.Y	Y	27.34	2631.2063	23	3.1	878.0731	3	33.17	34	1904	OB4062.raw	1.65E3	1	1	99	121	Carbamidomethylation; Deamidation (NQ)
**total 1 peptides**																	

tr|Q647H4|Q647H4_ARAHY

back to list

| Protein Coverage | Supporting Peptides |

Protein Coverage:

```
  1 MGKLLALSVC FCFLVLGASS ISFRQQPEEN ACQFQRLNAQ RPDNRIESEG GYIETWNPNN QEFECAGVAL SRLVLRRNAL
 81 RRPFYSNAPQ EIFIQQGRGY FGLIFPGCPS TYEEPAQQGR RHQSQRPPRR FQGQDQSQQQ QDSHQKVHRF DEGDLIAVPT
161 GVAFWMYNDH DTDVVAVSLT DTNNNDNQLD QFPRRFNLAG NHEQEFLRYQ QQSRRRSLPY SPYSPQTQPK QEDREFSPRG
241 QHGRRERAGQ EQENEGGNIF SGFTPEFLAQ AFQVDDRQIL QNLRGENESD EQGAIVTVRG GLRILSPDRK RRQQYERPDE
321 EEEYDEDEYE YDEEERQHDR RRGRGSRGSG NGIEETICTA SFKKNIGRNR SPDIYNPQAG SLKTANELNL LILRWLGLSA
401 EYGNLYRNAL FVPHYNTNAH SIIYALRGRA HVQVVDSNGD RVFDEELQEG HVLVVPQNFA VAGKSQSENF EYVAFKTDSR
481 PSIANLAGEN SFIDNLPEEV VANSYGLPRE QARQLKNNNP FKFFVPPSEQ SLRAVA
```

108: c; 117: d; 118: d

c Carbamidomethylation (+57.02)
d Deamidation (NQ) (+0.98)

Supporting Peptides:

Peptide	Uniq	-10lgP	Mass	Length	ppm	m/z	z	RT	Fraction	Scan	Source File	Area Digest 12. traka 45 kDa	#Feature	#Feature Digest 12. traka 45 kDa	Start	End	PTM
R.GYFGLIFPGC(+57.02)PSTYEEPAQ(+.98)Q(+.98)GRR.H	Y	27.34	2631.2063	23	3.1	878.0731	3	33.17	34	1904	OB4062.raw	1.65E3	1	1	99	121	Carbamidomethylation; Deamidation (NQ)
**total 1 peptides**																	

tr|Q8LKN1|Q8LKN1_ARAHY

back to list

| Protein Coverage | Supporting Peptides |

Protein Coverage:

```
  1 MGKLLALSVC FCFLVLGASS ISFRQQPEEN ACQFQRLNAQ RPDNRIESEG GYIETWNPNN QEFECAGVAL SRLVLRRNAL
                                          108        117 18
                                          c          d d
 81 RRPFYSNAPQ EIFIQQGRGY FGLIFPGCPS TYEEPAQQGR RHQSQRPPRR FQGQDQSQQQ QDSHQKVHRF DEGDLIAVPT
                                          c          d d
161 GVAFWMYNDH DTDVVAVSLT DTNNNDNQLD QFPRRFNLAG NHEQEFLRYQ QQSRRRSLPY SPYSPQTQPK QEDREFSPRG
241 QHGRRERAGQ EQENEGGNIF SGFTPEFLAQ AFQVDDRQIL QNLRGENESD EQGAIVTVRG GLRILSPDRK RRQQYERPDE
321 EEEYDEDEYE YDEEERQQDR RRGRGSRGSG NGIEETICTA SFKKNIGRNR SPDIYNPQAG SLKTANELQL NLLILRWLGL
401 SAEYGNLYRN ALFVPHYNTN AHSIIYALRG RAHVQVVDSN GDRVFDEELQ EGHVLVVPQN FAVAGKSQSE NFEYVAFKTD
481 SRPSIANLAG ENSFIDNLPE EVVANSYGLP REQARQLKNN NPFKFFVPPS EQSLRAVA
```

c Carbamidomethylation (+57.02)
d Deamidation (NQ) (+0.98)

Supporting Peptides:

Peptide	Uniq	-10lgP	Mass	Length	ppm	m/z	z	RT	Fraction	Scan	Source File	Area Digest 12. traka 45 kDa	#Feature	#Feature Digest 12. traka 45 kDa	Start	End	PTM
R.GYFGLIFPGC(+57.02)PSTYEEPAQ(+.98)Q(+.98)GRR.H	Y	27.34	2631.2063	23	3.1	878.0731	3	33.17	34	1904	OB4062.raw	1.65E3	1	1	99	121	Carbamidomethylation; Deamidation (NQ)
**total 1 peptides**																	

tr|Q8LL03|Q8LL03_ARAHY

back to list

| Protein Coverage | Supporting Peptides |

Protein Coverage:

```
  1 NYLHMLLALS VCFCFLVLGA SSISFRQQPE ENACQFQRLN AQRPDNRIES EGGYIETWNP NNQEFECAGV ALSRLVLRRN
                                            110        119 20
                                            c          d d
 81 ALRRPFYSNA PQEIFIQQGR GYFGLIFPGC PSTYEEPAQQ GRRHQSQRPP RRFQGQDQSQ QQQDSHQKVH RFDEGDLIAV
                                            c          d d
161 PTGVAFWMYN DHDTDVVAVS LTDTNNNDNQ LDQFPRRFNL AGNHEQEFLR YQQQSRRRS
```

c Carbamidomethylation (+57.02)
d Deamidation (NQ) (+0.98)

Supporting Peptides:

Peptide	Uniq	-10lgP	Mass	Length	ppm	m/z	z	RT	Fraction	Scan	Source File	Area Digest 12. traka 45 kDa	#Feature	#Feature Digest 12. traka 45 kDa	Start	End	PTM
R.GYFGLIFPGC(+57.02)PSTYEEPAQ(+.98)Q(+.98)GRR.H	Y	27.34	2631.2063	23	3.1	878.0731	3	33.17	34	1904	OB4062.raw	1.65E3	1	1	101	123	Carbamidomethylation; Deamidation (NQ)
**total 1 peptides**																	

Peptide List

Prepared with PEAKS ™ (bioinfor.com)

1. Notes Gastric Control Raw peanut Band #14 45-55 kDa (PTM464)

2. Result Statistics

Figure 1. False discovery rate (FDR) curve. X axis is the number of peptide-spectrum matches (PSM) being kept. Y axis is the corresponding FDR.

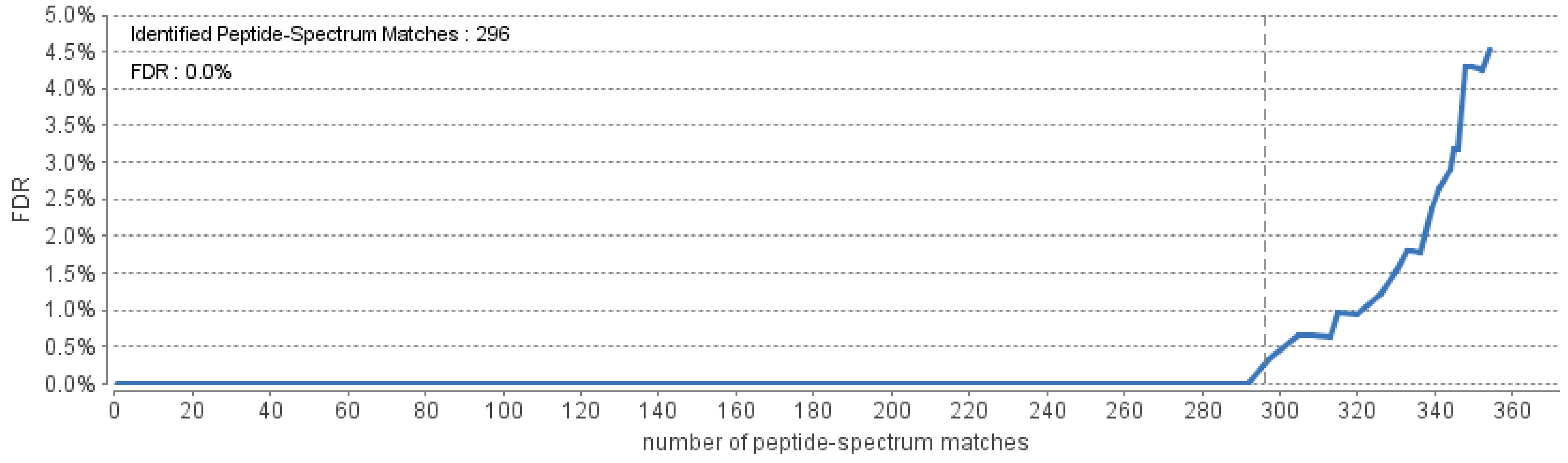

Figure 2. PSM score distribution. **(a)** Distribution of PEAKS peptide score; **(b)** Scatterplot of PEAKS peptide score versus precursor mass error.

(a)

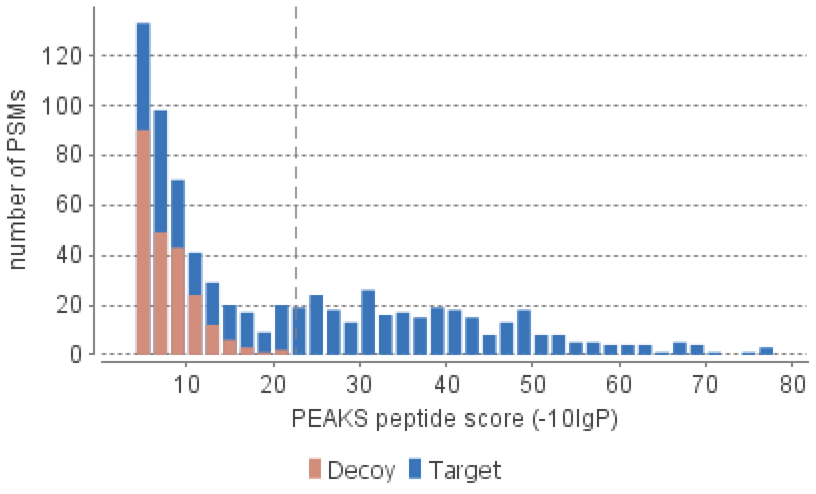

(b)

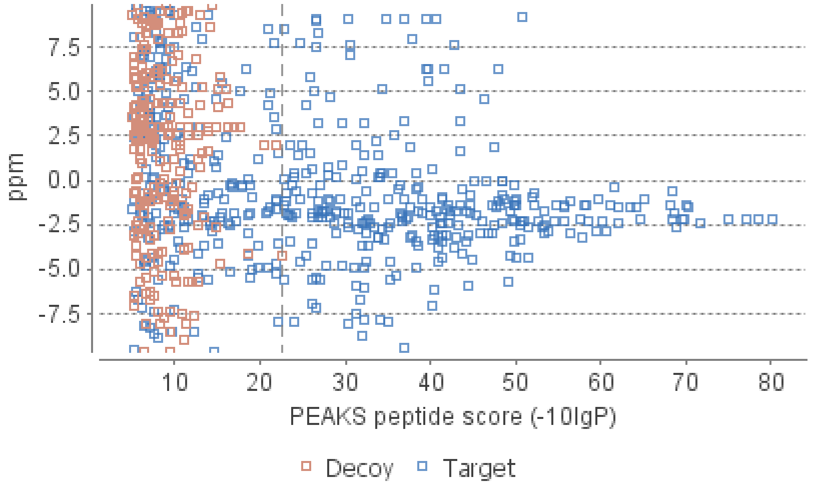

Figure 3. Distribution of peptide feature detection. **(a)** Feature m/z distribution; **(b)** Feature RT distribution

(a) (b)

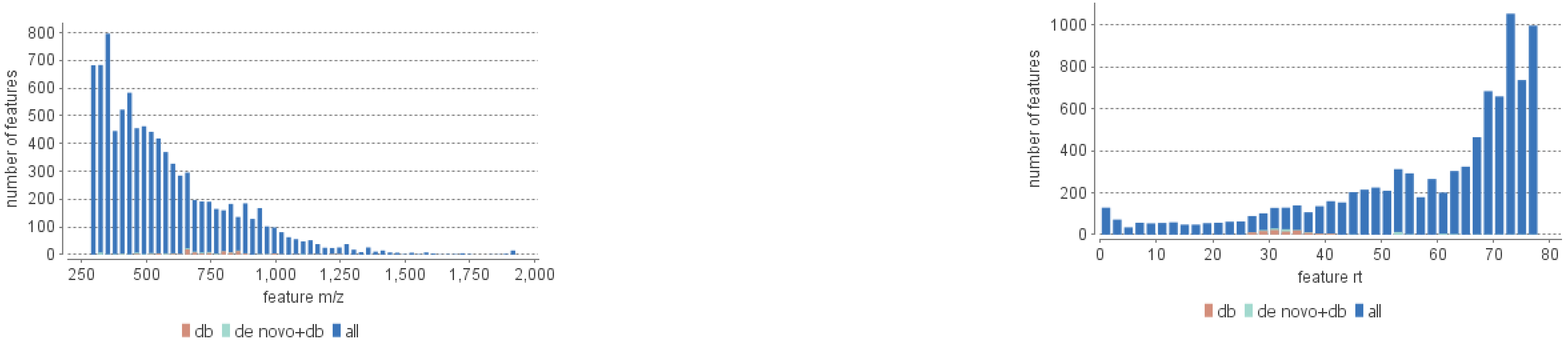

Figure 4. Distribution of identified peptide features. **(a)** Feature abundance distribution **(b)** *De novo* sequencing validation.

(a) (b)

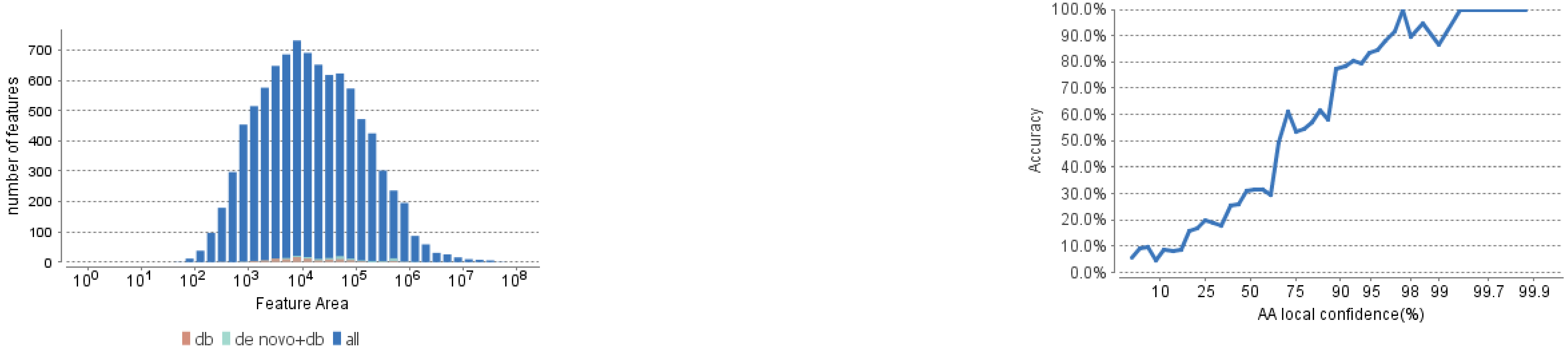

Table 1. Statistics of data.

# of MS scans	2679
# of MS/MS scans	1770
# of Features	9269
# of Chimera scans	168

Table 2. Result filtration parameters.

Peptide -10lgP	≥22.7

Table 4. PTM profile.

Name	ΔMass	Position	#PSM	-10lgP	Abundance	AScore
Deamidation	.98	NQ	206	68.89	8.57E3	54.70
Carbamidomethyl	57.02	C	83	68.44		1000.00
HydPro	15.99	P	35	44.45		56.47
Oxidation	15.99	M	7	42.90	2.74E3	1000.00

PTM Ascore	≥20
Protein -10lgP	≥20
Proteins unique peptides	≥1
De novo ALC Score	≥50%

Table 3. Statistics of filtered result.

Peptide-Spectrum Matches	296
Peptide sequences	154
Protein groups	6
Proteins	13
Proteins (#Unique Peptides)	9 (>2); 1 (=2); 3 (=1);
FDR (Peptide-Spectrum Matches)	0.0%
FDR (Peptide Sequences)	0.0%
FDR (Protein Group)	0.0%
De Novo Only Spectra	98
# of identified Features	164
# of identified MS/MS scans	280

Acetylation	42.01	N-term	3	32.39	7.36E3	1000.00
Dimethylation(KR)	28.03	K	2	53.40	9.75E3	1000.00
Methylation(others)	14.02	E	2	33.87	6.86E3	29.85
Phosphorylation	79.97	S	2	32.21	4.06E3	0.00
Ethyl	28.03	E	2	32.47	2.75E3	14.78
Carbamylation	43.01	N-term	1	31.32	5.56E3	1000.00
EtOH	44.03	K	1	27.61	4.36E4	59.20
Arg->Orn	-42.02	R	1	26.56		35.38
Dehydration	-18.01	T	1	32.21	4.06E3	0.00
Methylation(KR)	14.02	R	1	31.32	5.56E3	51.28

3. Experiment Control

Figure 5. Precursor mass error of peptide-spectrum matches (PSM) in filtered result. **(a)** Distribution of precursor mass error in ppm; **(b)** Scatterplot of precursor m/z versus precursor mass error in ppm.

(a)

(b)

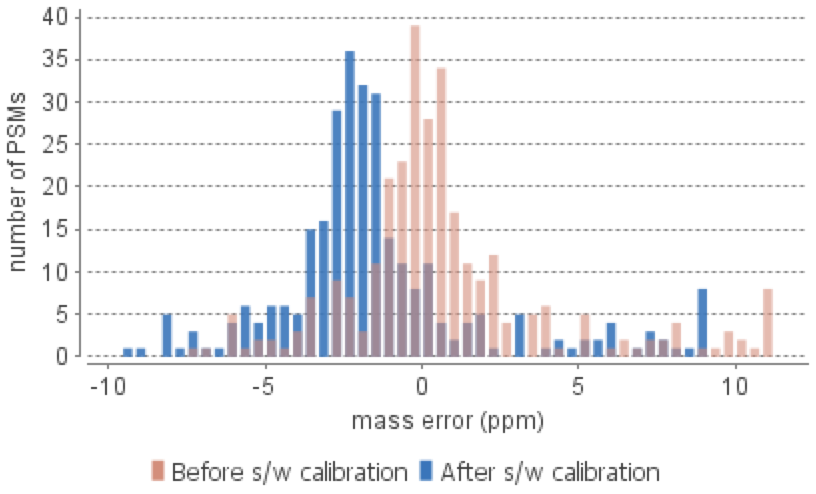

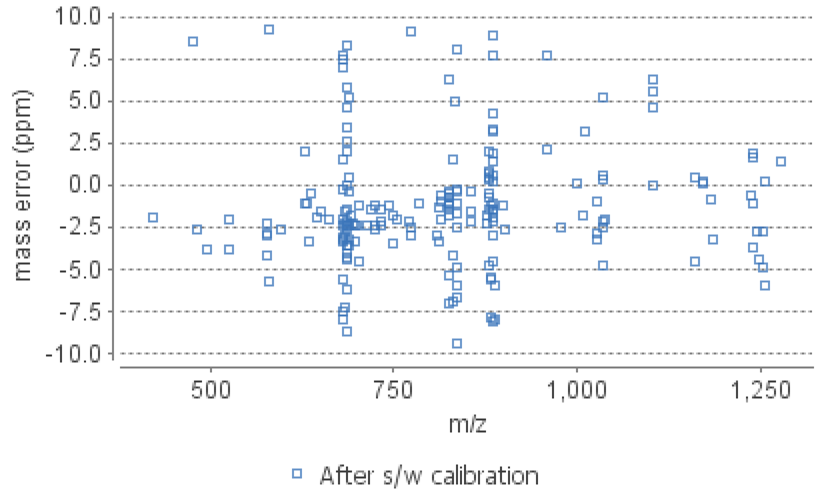

Table 5. Number of identified peptides in each sample by the number of missed cleavages

Missed Cleavages	0	1	2	3	4+
Kontrola 13. traka 50 kDa	93	60	1	0	0

4. Other Information

Table 6. Search parameters.

Search Engine Name: PEAKS
Parent Mass Error Tolerance: 10.0 ppm
Fragment Mass Error Tolerance: 0.5 Da
Precursor Mass Search Type: monoisotopic
Enzyme: Trypsin
Max Missed Cleavages: 2
Digest Mode: Unspecific
Fixed Modifications:
  Carbamidomethylation: 57.02
Variable Modifications:
  Deamidation (NQ): 0.98
  Oxidation (M): 15.99
  Hydroxylation Pro: 15.99
  Acetylation (K): 42.01
  Acetylation (Protein N-term): 42.01
  Acetylation (N-term): 42.01
  Amidation: -0.98
  Beta-methylthiolation: 45.99
  and 305 more...
Max Variable PTM Per Peptide: 5

Table 7. Instrument parameters.

Fractions: OB4042.raw
Ion Source: ESI(nano-spray)
Fragmentation Mode: CID, CAD(y and b ions)
MS Scan Mode: FT-ICR/Orbitrap
MS/MS Scan Mode: Linear Ion Trap

Database: Uniprot_Peanut-3818_Jul18
Taxon: All
Contaminant Database: contaminantsMQ_mar19
Searched Entry: 1723
FDR Estimation: Enabled
De novo score (ALC%) threshold: 15
Peptide hit threshold (-10logP): 30.0
Peaks run ID: 443
Merge Options: no merge
Precursor Options: corrected
Charge Options: no correction
Filter Charge: 2 - 8
Process: true
Associate chimera: yes

Protein List

Protein Accession Contains:
Protein Description Contains:
Peptide Sample Area >=
Protein Ptm Contains:

Protein Group	Protein ID	Accession	-10lgP	Coverage (%)	Coverage (%) Kontrola 13. traka 50 kDa	Area Kontrola 13. traka 50 kDa	#Peptides	#Unique	#Spec Kontrola 13. traka 50 kDa	PTM	Avg. Mass	Description
**1**	20580	tr|Q647H4|Q647H4_ARAHY	233.78	54	54	2.45E4	23	2	221	Y	61506	Arachin Ahy-1 OS=Arachis hypogaea OX=3818 PE=2 SV=1
**6**	20590	tr|O82580|O82580_ARAHY	168.47	23	23	7.13E4	10	1	92	Y	58350	Glycinin (Fragment) OS=Arachis hypogaea OX=3818 GN=Arah3 PE=2 SV=1
**9**	22400	tr|Q6PSU4|Q6PSU4_ARAHY	129.60	18	18	3.5E4	7	7	8	Y	48095	Conarachin (Fragment) OS=Arachis hypogaea OX=3818 PE=2 SV=1
**9**	20853	tr|N1NG13|N1NG13_ARAHY	129.60	12	12	3.5E4	7	7	8	Y	71345	Seed storage protein Ara h1 OS=Arachis hypogaea OX=3818 GN=ARAX_AHF417E07-017 PE=4 SV=1
**9**	20854	P43238|ALL12_ARAHY	129.60	12	12	3.5E4	7	7	8	Y	71345	Allergen Ara h 1, clone P41B OS=Arachis hypogaea OX=3818 PE=1 SV=1
**9**	20849	tr|Q6PSU3|Q6PSU3_ARAHY	127.53	13	13	3.5E4	7	7	8	Y	66575	Conarachin (Fragment) OS=Arachis hypogaea OX=3818 PE=4 SV=1
**9**	20850	P43237|ALL11_ARAHY	127.53	12	12	3.5E4	7	7	8	Y	70283	Allergen Ara h 1, clone P17 OS=Arachis hypogaea OX=3818 PE=1 SV=1
**9**	20851	tr|B3IXL2|B3IXL2_ARAHY	127.53	12	12	3.5E4	7	7	8	Y	70283	Main allergen Ara h1 OS=Arachis hypogaea OX=3818 PE=2 SV=1
**7**	20595	tr|E5G077|E5G077_ARAHY	123.43	17	17	2.72E5	4	4	33	Y	58305	Ara h 3 allergen OS=Arachis hypogaea OX=3818 GN=ara h 3 PE=3 SV=1
**7**	20593	tr|Q6IWG5|Q6IWG5_ARAHY	123.43	17	17	2.72E5	4	4	33	Y	58061	Glycinin (Fragment) OS=Arachis hypogaea OX=3818 PE=2 SV=1
**7**	20594	tr|Q0GM57|Q0GM57_ARAHY	123.43	17	17	2.72E5	4	4	33	Y	58263	Iso-Ara h3 OS=Arachis hypogaea OX=3818 PE=2 SV=1
**14**	22697	tr|T2B9M0|T2B9M0_ARAHY	35.36	6	6	2.3E4	1	1	2	N	38383	Fructose-bisphosphate aldolase OS=Arachis hypogaea OX=3818 PE=2 SV=1
**17**	22425	tr|C9W981|C9W981_ARAHY	22.72	1	1	3.94E3	1	1	1	N	116425	Phosphoenolpyruvate carboxylase OS=Arachis hypogaea OX=3818 GN=PEPC5 PE=2 SV=1
**total 13 proteins**												

tr|Q647H4|Q647H4_ARAHY

back to list

| Protein Coverage | Supporting Peptides |

Protein Coverage:

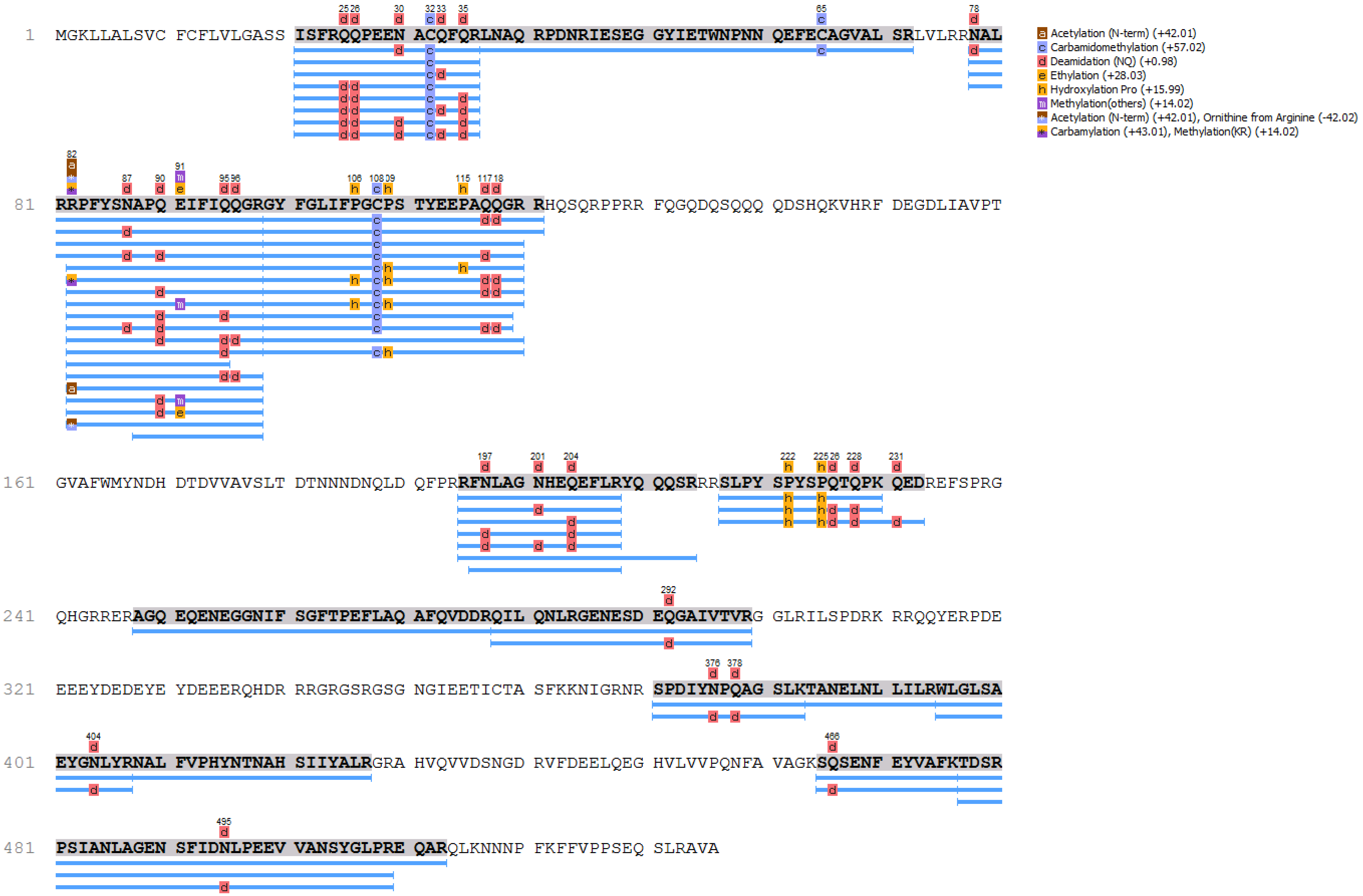

Supporting Peptides:

Peptide	Uniq	-10lgP	Mass	Length	ppm	m/z	z	RT	Fraction	Scan	Source File	Area Kontrola 13. traka 50 kDa	#Feature	#Feature Kontrola 13. traka 50 kDa	Start	End	PTM
R.WLGLSAEYGNLYR.N	N	80.29	1540.7673	13	-2.2	771.3909	2	33.47	14	1745	OB4042.raw	2.98E5	1	1	395	407	
K.SQSENFEYVAFK.T	N	71.67	1447.6619	12	-2.4	724.8380	2	30.46	14	1555	OB4042.raw	2.97E4	1	1	465	476	
K.SQ(+.98)SENFEYVAFK.T	N	68.89	1448.6459	12	-2.7	725.3298	2	30.85	14	1574	OB4042.raw	8.57E3	1	1	465	476	Deamidation (NQ)
R.GYFGLIFPGC(+57.02)PSTYEEPAQQGR.R	N	68.44	2473.1372	22	-0.6	1237.5778	2	35.08	14	1836	OB4042.raw	0	0	0	99	120	Carbamidomethylation
R.GYFGLIFPGC(+57.02)PSTYEEPAQQ(+.98)GR.R	N	65.18	2474.1211	22	-1.0	1238.0692	2	35.25	14	1842	OB4042.raw	6E4	1	1	99	120	Carbamidomethylation
R.RPFYSNAPQEIFIQQGR.G	N	64.23	2050.0383	17	-1.5	684.3538	3	30.27	14	1519	OB4042.raw	5.16E5	1	1	82	98	
R.RPFYSNAPQEIFIQ(+.98)QGR.G	N	61.11	2051.0225	17	-1.4	684.6819	3	30.85	14	1561	OB4042.raw	8.24E5	1	1	82	98	
R.RPFYSN(+.98)APQ(+.98)EIFIQQGR.G	N	60.71	2052.0063	17	-2.8	1027.0098	2	31.34	14	1612	OB4042.raw	4.16E5	5	5	82	98	Deamidation (NQ)
R.RPFYSN(+.98)APQEIFIQ(+.98)QGR.G	N	59.57	2052.0063	17	-2.8	1027.0098	2	31.34	14	1607	OB4042.raw	7.62E3	1	1	82	98	
R.RFNLAGNHEQEFLR.Y	N	58.53	1729.8647	14	-2.3	577.6288	3	27.72	14	1390	OB4042.raw	6.99E4	1	1	195	208	
R.RPFYSNAPQ(+.98)EIFIQQGR.G	N	58.35	2051.0225	17	-1.4	684.6819	3	30.85	14	1585	OB4042.raw	8.38E5	3	3	82	98	Deamidation (NQ)
R.RFN(+.98)LAGNHEQ(+.98)EFLR.Y	N	56.51	1731.8329	14	-3.0	578.2844	3	28.94	14	1473	OB4042.raw	7.03E4	1	1	195	208	Deamidation (NQ)
R.SPDIYNPQ(+.98)AGSLK.T	N	55.64	1389.6776	13	-2.4	695.8459	2	28.23	14	1414	OB4042.raw	9.57E3	1	1	371	383	
R.RFN(+.98)LAGNHEQEFLR.Y	N	54.65	1730.8488	14	-2.3	577.9568	3	28.40	14	1431	OB4042.raw	1.25E5	1	1	195	208	
R.RPFYSN(+.98)APQEIFIQQGR.G	N	53.76	2051.0225	17	-2.8	1026.5178	2	30.95	14	1582	OB4042.raw	7.44E3	1	1	82	98	
R.QILQ(+.98)NLRGENESDEQ(+.98)GAIVTVR.G	N	53.44	2470.2300	22	-1.8	824.4176	3	29.69	14	1502	OB4042.raw	3.89E4	1	1	278	299	Deamidation (NQ)
R.QILQNLRGENESDEQ(+.98)GAIVTVR.G	N	52.27	2469.2458	22	-1.4	824.0898	3	29.31	14	1490	OB4042.raw	4.9E4	1	1	278	299	
R.QILQNLRGENESDEQGAIVTVR.G	N	52.18	2468.2620	22	-0.7	823.7625	3	29.03	14	1462	OB4042.raw	1.36E4	1	1	278	299	
R.SPDIYN(+.98)PQAGSLK.T	N	51.09	1389.6776	13	-2.4	695.8459	2	28.23	14	1410	OB4042.raw	9.57E3	1	1	371	383	
R.RFNLAGNHEQ(+.98)EFLR.Y	N	50.78	1730.8488	14	-2.3	577.9568	3	28.40	14	1419	OB4042.raw	1.25E5	1	1	195	208	Deamidation (NQ)
R.RFN(+.98)LAGN(+.98)HEQ(+.98)EFLR.Y	N	50.69	1732.8169	14	9.3	578.6195	3	29.24	14	1480	OB4042.raw	1.04E4	1	1	195	208	Deamidation (NQ)
K.TANELNLLILR.W	N	50.47	1268.7452	11	-3.3	635.3792	2	33.96	14	1763	OB4042.raw	6.44E4	1	1	384	394	
R.NALRRPFYSN(+.98)APQEIFIQQGR.G	N	50.06	2505.2876	21	-1.1	836.1041	3	30.17	14	1539	OB4042.raw	2.18E4	1	1	78	98	Deamidation (NQ)
R.N(+.98)ALRRPFYSNAPQEIFIQQGR.G	N	49.89	2505.2876	21	-0.3	836.1047	3	30.02	14	1527	OB4042.raw	2.18E4	1	1	78	98	Deamidation (NQ)
R.QILQN(+.98)LRGENESDEQGAIVTVR.G	N	49.83	2469.2458	22	-1.4	824.0898	3	29.31	14	1482	OB4042.raw	4.9E4	1	1	278	299	
R.RFNLAGN(+.98)HEQEFLR.Y	N	49.75	1730.8488	14	-2.3	577.9568	3	28.40	14	1407	OB4042.raw	1.25E5	1	1	195	208	Deamidation (NQ)
R.QILQ(+.98)N(+.98)LRGENESDEQGAIVTVR.G	N	49.32	2470.2300	22	-1.8	824.4176	3	29.69	14	1522	OB4042.raw	3.89E4	1	1	278	299	
K.TDSRPSIANLAGENSFIDN(+.98)LPEEVVANSYGLPR.E	N	48.51	3545.7273	33	-0.8	1182.9180	3	36.33	14	1929	OB4042.raw	1.29E4	1	1	477	509	
S.ISFRQ(+.98)QPEENAC(+57.02)Q(+.98)FQR.L	N	48.47	2038.9166	16	-2.4	680.6460	3	27.52	14	1384	OB4042.raw	5.07E4	1	1	21	36	Carbamidomethylation; Deamidation (NQ)
R.RPFYSNAPQ(+.98)EIFIQ(+.98)QGR.G	N	48.46	2052.0063	17	0.0	685.0109	3	31.15	14	1609	OB4042.raw	4.03E5	2	2	82	98	Deamidation (NQ)
R.AGQEQEN(+.98)EGGN(+.98)IFSGFTPEFLAQAFQVDDR.Q	N	48.08	3302.4639	30	6.3	1101.8379	3	38.83	14	2079	OB4042.raw	1.58E4	1	1	248	277	
R.GYFGLIFPGC(+57.02)PSTYEEPAQ(+.98)QGR.R	N	48.04	2474.1211	22	-1.2	825.7151	3	35.25	14	1841	OB4042.raw	4.97E4	2	2	99	120	Carbamidomethylation; Deamidation (NQ)
R.GYFGLIFPGC(+57.02)PSTYEEPAQ(+.98)Q(+.98)GR.R	N	47.54	2475.1052	22	1.8	1238.5648	2	35.48	14	1860	OB4042.raw	0	0	0	99	120	Carbamidomethylation; Deamidation (NQ)
R.WLGLSAEYGN(+.98)LYR.N	N	47.46	1541.7513	13	-2.5	771.8827	2	33.91	14	1771	OB4042.raw	3.16E4	2	2	395	407	Deamidation (NQ)
R.QILQNLRGEN(+.98)ESDEQGAIVTVR.G	N	44.61	2469.2458	22	-1.4	824.0898	3	29.31	14	1514	OB4042.raw	4.9E4	1	1	278	299	
N.APQEIFIQQGR.G	N	44.59	1285.6779	11	-1.9	643.8464	2	28.31	14	1423	OB4042.raw	0	0	0	88	98	
S.ISFRQ(+.98)QPEENAC(+57.02)QFQR.L	N	44.48	2037.9326	16	-3.1	680.3175	3	27.08	14	1342	OB4042.raw	1.92E4	1	1	21	36	Carbamidomethylation
R.GYFGLIFP(+15.99)GC(+57.02)P(+15.99)STYEEPAQ(+.98)QGR.R	N	44.45	2506.1111	22	-5.9	1254.0581	2	35.51	14	1862	OB4042.raw	0	0	0	99	120	Hydroxylation Pro; Carbamidomethylation
R.NALRRPFYSN(+.98)APQ(+.98)EIFIQQGR.G	N	44.22	2506.2717	21	-1.7	836.4316	3	31.25	14	1615	OB4042.raw	3.78E4	1	1	78	98	Deamidation (NQ)

Peptide	Uniq	-10lgP	Mass	Length	ppm	m/z	z	RT	Fraction	Scan	Source File	Area Kontrola 13. traka 50 kDa	#Feature	#Feature Kontrola 13. traka 50 kDa	Start	End	PTM
S.ISFRQ(+.98)Q(+.98)PEENAC(+57.02)QFQ(+.98)R.L	N	44.16	2039.9006	16	-3.4	680.9733	3	27.93	14	1399	OB4042.raw	6.9E4	1	1	21	36	Deamidation (NQ); Carbamidomethylation
R.LNAQ(+.98)RPDNRIESEGGYIETWN(+.98)PN(+.98)N(+.98)QEFEC(+57.02)AGVALSR.L	N	43.45	4137.8608	36	5.2	1035.4801	4	32.41	14	1674	OB4042.raw	0	0	0	37	72	Carbamidomethylation
K.TDSRPSIANLAGEN(+.98)SFIDN(+.98)LPEEVVANSYGLPR.E	N	43.02	3546.7114	33	-3.2	1183.2432	3	36.67	14	1934	OB4042.raw	0	0	0	477	509	Deamidation (NQ)
R.GYFGLIFPGC(+57.02)PSTYEEPAQQ(+.98)GRR.H	N	42.93	2630.2224	23	-1.8	877.7484	3	33.77	14	1740	OB4042.raw	4.44E5	1	1	99	121	Carbamidomethylation
R.N(+.98)ALRRPFYSN(+.98)APQEIFIQQGR.G	N	42.27	2506.2717	21	-1.7	836.4316	3	31.25	14	1605	OB4042.raw	3.78E4	1	1	78	98	
S.ISFRQQ(+.98)PEEN(+.98)AC(+57.02)QFQR.L	N	42.02	2038.9166	16	-2.4	680.6460	3	27.52	14	1371	OB4042.raw	5.07E4	1	1	21	36	Carbamidomethylation
R.RPFYSNAPQEIFIQ.Q	N	41.89	1708.8573	14	-1.6	855.4364	2	32.20	14	1666	OB4042.raw	9.45E3	1	1	82	95	
R.NALFVPHYN(+.98)TNAHSIIYALR.G	N	41.84	2314.1858	20	-3.1	772.4019	3	32.10	14	1654	OB4042.raw	1.13E4	1	1	408	427	
R.SLPYSP(+15.99)YSP(+15.99)QTQ(+.98)PK.Q	Y	41.46	1624.7620	14	-1.0	813.3892	2	26.50	14	1305	OB4042.raw	1.24E4	1	1	217	230	Hydroxylation Pro
R.GYFGLIFPGC(+57.02)P(+15.99)STYEEPAQ(+.98)QGR.R	N	41.43	2490.1160	22	-4.4	1246.0625	2	35.95	14	1891	OB4042.raw	1.24E4	1	1	99	120	Carbamidomethylation
S.ISFRQQ(+.98)PEENAC(+57.02)QFQR.L	N	41.33	2037.9326	16	-3.1	680.3175	3	27.08	14	1359	OB4042.raw	1.92E4	1	1	21	36	Carbamidomethylation
R.RPFYSNAP(+15.99)QEIFIQQGR.G	N	41.19	2066.0332	17	-2.5	689.6848	3	31.98	14	1648	OB4042.raw	0	0	0	82	98	
R.SLPYSP(+15.99)YSP(+15.99)Q(+.98)TQ(+.98)PK.Q	Y	41.04	1625.7460	14	-2.0	813.8804	2	26.84	14	1332	OB4042.raw	8.82E3	1	1	217	230	Hydroxylation Pro; Deamidation (NQ)
S.ISFRQ(+.98)Q(+.98)PEENAC(+57.02)Q(+.98)FQ(+.98)R.L	N	40.88	2040.8846	16	-3.2	681.3015	3	28.28	14	1416	OB4042.raw	4.58E4	1	1	21	36	Deamidation (NQ); Carbamidomethylation
R.FNLAGNHEQEFLR.Y	N	40.84	1573.7637	13	-3.8	525.5943	3	29.11	14	1479	OB4042.raw	4.25E4	1	1	196	208	
R.GYFGLIFPGC(+57.02)PSTYEEPAQ(+.98)Q(+.98)G.R	N	40.82	2319.0042	21	-4.5	1160.5066	2	37.55	14	1990	OB4042.raw	4.46E3	1	1	99	119	Carbamidomethylation; Deamidation (NQ)
S.ISFRQQPEEN(+.98)AC(+57.02)QFQR.L	N	40.43	2037.9326	16	-3.1	680.3175	3	27.08	14	1337	OB4042.raw	1.92E4	1	1	21	36	Deamidation (NQ); Carbamidomethylation
R.SPDIYN(+.98)PQ(+.98)AGSLK.T	N	40.27	1390.6616	13	-3.3	696.3373	2	28.71	14	1454	OB4042.raw	1.04E3	1	1	371	383	Deamidation (NQ)
R.GYFGLIFP(+15.99)GC(+57.02)P(+15.99)STYEEPAQQGR.R	N	38.37	2505.1270	22	-4.9	1253.5674	2	35.32	14	1850	OB4042.raw	1.06E4	1	1	99	120	Carbamidomethylation
R.LN(+.98)AQRPDNRIESEGGYIETWN(+.98)PNNQEFEC(+57.02)AGVALSR.L	N	38.27	4135.8931	36	0.4	1034.9832	4	32.00	14	1663	OB4042.raw	1.58E5	1	1	37	72	Carbamidomethylation
S.ISFRQ(+.98)Q(+.98)PEENAC(+57.02)Q(+.98)FQR.L	N	38.25	2039.9006	16	-3.4	680.9733	3	27.93	14	1387	OB4042.raw	6.9E4	1	1	21	36	Deamidation (NQ); Carbamidomethylation
K.TANELN(+.98)LLILR.W	N	38.23	1269.7292	11	-0.5	635.8730	2	34.46	14	1808	OB4042.raw	2.71E4	1	1	384	394	
R.N(+.98)ALRRPFYSNAPQ(+.98)EIFIQQGR.G	N	37.77	2506.2717	21	8.0	836.4397	3	30.54	14	1559	OB4042.raw	3.78E4	1	1	78	98	
S.ISFRQ(+.98)Q(+.98)PEEN(+.98)AC(+57.02)QFQ(+.98)R.L	N	37.74	2040.8846	16	-3.2	681.3015	3	28.57	14	1439	OB4042.raw	4.58E4	1	1	21	36	Deamidation (NQ); Carbamidomethylation
R.RPFYSN(+.98)APQEIFIQ.Q	N	37.32	1709.8413	14	-2.1	855.9280	2	32.82	14	1698	OB4042.raw	4.44E3	1	1	82	95	
R.GYFGLIFP(+15.99)GC(+57.02)P(+15.99)STYEEPAQQ(+.98)GR.R	N	36.85	2506.1111	22	-9.4	836.3716	3	35.58	14	1866	OB4042.raw	0	0	0	99	120	Hydroxylation Pro; Carbamidomethylation
R.N(+.98)ALRRPFYSN(+.98)APQ(+.98)EIFIQQGR.G	N	36.44	2507.2556	21	-0.3	836.7607	3	31.54	14	1621	OB4042.raw	0	0	0	78	98	Deamidation (NQ)
R.GYFGLIFPGC(+57.02)PSTYEEPAQ(+.98)Q(+.98)GRR.H	N	35.54	2631.2063	23	0.4	878.0783	3	33.78	14	1757	OB4042.raw	0	0	0	99	121	Carbamidomethylation; Deamidation (NQ)
S.ISFRQ(+.98)Q(+.98)PEEN(+.98)AC(+57.02)Q(+.98)FQ(+.98)R.L	N	35.38	2041.8687	16	-5.6	681.6278	3	28.68	14	1448	OB4042.raw	2.82E3	1	1	21	36	Deamidation (NQ); Carbamidomethylation
R.RPFYSNAP(+15.99)Q(+.98)EIFIQQGR.G	N	34.27	2067.0173	17	5.2	690.0181	3	31.90	14	1648	OB4042.raw	4.55E3	1	1	82	98	
R.GYFGLIFPGC(+57.02)P(+15.99)STYEEP(+15.99)AQQGR.R	N	34.24	2505.1270	22	-2.7	1253.5701	2	35.41	14	1856	OB4042.raw	0	0	0	99	120	Carbamidomethylation; Hydroxylation Pro
R.GYFGLIFP(+15.99)GC(+57.02)P(+15.99)STYEEPAQ(+.98)Q(+.98)GR.R	N	34.16	2507.0950	22	0.2	1254.5577	2	35.45	14	1856	OB4042.raw	8.02E3	1	1	99	120	Hydroxylation Pro; Carbamidomethylation; Deamidation (NQ)
R.GYFGLIFPGC(+57.02)PSTYEEP(+15.99)AQQGRR.H	N	34.02	2645.2332	23	-7.9	882.7466	3	34.07	14	1773	OB4042.raw	4.78E4	1	1	99	121	Carbamidomethylation

Peptide	Uniq	-10lgP	Mass	Length	ppm	m/z	z	RT	Fraction	Scan	Source File	Area Kontrola 13. traka 50 kDa	#Feature	#Feature Kontrola 13. traka 50 kDa	Start	End	PTM
R.LNAQ(+.98)RPDNRIESEGGYIETWNPNNQ(+.98)EFEC(+57.02)AGVALSR.L	N	33.98	4135.8931	36	0.4	1034.9832	4	32.00	14	1644	OB4042.raw	1.58E5	1	1	37	72	Carbamidomethylation
R.RPFYSNAPQE(+14.02)IFIQQGR.G	N	33.87	2064.0540	17	-3.2	689.0245	3	30.85	14	1589	OB4042.raw	6.86E3	1	1	82	98	Methylation(others)
R.RPFYSNAPQ(+.98)E(+14.02)IFIQQGR.G	N	32.73	2065.0381	17	-3.6	689.3523	3	31.43	14	1617	OB4042.raw	9.36E3	1	1	82	98	Deamidation (NQ); Methylation(others)
R.RPFYSNAPQE(+28.03)IFIQQGR.G	N	32.47	2078.0696	17	-2.3	693.6970	3	31.61	14	1624	OB4042.raw	2.75E3	1	1	82	98	
R.SLPYSP(+15.99)YSP(+15.99)QTQPK.Q	Y	32.42	1623.7780	14	-0.6	812.8975	2	26.17	14	1286	OB4042.raw	0	0	0	217	230	Hydroxylation Pro
R.R(+42.01)PFYSNAP(+15.99)QEIFIQQGR.G	N	32.39	2108.0439	17	-4.6	703.6869	3	30.85	14	1577	OB4042.raw	7.36E3	1	1	82	98	Acetylation (N-term)
R.GYFGLIFPGC(+57.02)P(+15.99)S(+79.97)T(-18.01)YEEPAQQGR.R	N	32.21	2551.0879	22	1.4	1276.5558	2	36.85	14	1946	OB4042.raw	4.06E3	1	1	99	120	Carbamidomethylation; Hydroxylation Pro
R.N(+.98)ALRRPFYSNAPQ(+.98)EIFIQQ(+.98)GR.G	N	32.12	2507.2556	21	-4.9	836.7569	3	31.79	14	1636	OB4042.raw	0	0	0	78	98	
R.RPFYSNAP(+15.99)QEIFIQ(+.98)QGR.G	N	32.05	2067.0173	17	0.5	690.0149	3	32.17	14	1659	OB4042.raw	0	0	0	82	98	Deamidation (NQ)
R.RPFYSNAPQ(+.98)EIFIQ(+.98)Q(+.98)GR.G	N	32.01	2052.9905	17	-3.6	685.3364	3	31.85	14	1632	OB4042.raw	7.14E4	1	1	82	98	Deamidation (NQ)
R.RPFYSNAPQ(+.98)EIFIQQ(+.98)GR.G	N	31.95	2052.0063	17	-2.4	685.0092	3	37.08	14	1960	OB4042.raw	1.09E3	1	1	82	98	Deamidation (NQ)
R.GYFGLIFPGC(+57.02)P(+15.99)STYEEPAQQGR.R	N	31.46	2489.1321	22	-2.8	1245.5725	2	35.78	14	1878	OB4042.raw	0	0	0	99	120	Carbamidomethylation
R.R(+43.01)(+14.02)PFYSNAPQEIFIQQGR.G	N	31.32	2107.0598	17	-1.2	703.3612	3	30.49	14	1544	OB4042.raw	5.56E3	1	1	82	98	Carbamylation; Methylation(KR)
R.GYFGLIFPGC(+57.02)P(+15.99)STYEEP(+15.99)AQQGRR.H	N	31.32	2661.2280	23	-5.9	888.0800	3	33.77	14	1759	OB4042.raw	2.05E4	1	1	99	121	Carbamidomethylation
R.SLPYSP(+15.99)YSP(+15.99)Q(+.98)TQ(+.98)PKQ(+.98)ED.R	Y	31.20	1998.8582	17	0.1	1000.4386	2	27.17	14	1356	OB4042.raw	3.32E3	1	1	217	233	Hydroxylation Pro; Deamidation (NQ)
S.ISFRQ(+.98)Q(+.98)PEENAC(+57.02)QFQR.L	N	30.52	2038.9166	16	7.0	680.6524	3	27.00	14	1339	OB4042.raw	0	0	0	21	36	Carbamidomethylation
R.RPFYSNAPQ(+.98)E(+28.03)IFIQQGR.G	N	30.30	2079.0537	17	-2.4	694.0250	3	32.10	14	1653	OB4042.raw	5.75E3	1	1	82	98	Deamidation (NQ); Ethylation
K.TDSRPSIANLAGEN(+.98)SFIDN(+.98)LPEEVVANSYGLPREQ(+.98)AR.Q	N	29.53	4031.9348	37	3.2	1008.9964	4	35.25	14	1844	OB4042.raw	2.56E4	1	1	477	513	
K.TDSRPSIANLAGEN(+.98)SFIDNLPEEVVANSYGLPREQ(+.98)AR.Q	N	29.04	4030.9507	37	-1.8	1008.7453	4	35.22	14	1844	OB4042.raw	0	0	0	477	513	
R.RFN(+.98)LAGNHEQ(+.98)EFLRYQQ(+.98)Q(+.98)SR.R	N	28.14	2524.1731	20	-1.1	632.0512	4	29.57	14	1500	OB4042.raw	0	0	0	195	214	
R.LN(+.98)AQRPDNRIESEGGYIETWNPNNQEFEC(+57.02)AGVALSR.L	N	27.84	4134.9087	36	-4.8	1034.7317	4	32.66	14	1689	OB4042.raw	0	0	0	37	72	Carbamidomethylation
R.LN(+.98)AQRPDNRIESEGGYIETWN(+.98)PNN(+.98)QEFEC(+57.02)AGVALSR.L	N	27.21	4136.8770	36	-2.1	1035.2266	4	33.02	14	1711	OB4042.raw	0	0	0	37	72	Carbamidomethylation
R.GYFGLIFP(+15.99)GC(+57.02)PSTYEEPAQQ(+.98)GRR.H	N	26.99	2646.2173	23	-5.6	883.0767	3	34.26	14	1807	OB4042.raw	5.92E4	1	1	99	121	Carbamidomethylation
R.RPFYSN(+.98)APQEIFIQ(+.98)Q(+.98)GR.G	N	26.83	2052.9905	17	8.3	685.3446	3	37.13	14	1964	OB4042.raw	0	0	0	82	98	Deamidation (NQ)
R.GYFGLIFPGC(+57.02)PSTYEEPAQ(+.98)QGRR.H	N	26.76	2630.2224	23	-1.8	877.7484	3	33.77	14	1725	OB4042.raw	4.44E5	1	1	99	121	Carbamidomethylation
R.R(+42.01)(-42.02)PFYSNAPQEIFIQQGR.G	N	26.56	2050.0271	17	-7.2	684.3462	3	34.21	14	1783	OB4042.raw	0	0	0	82	98	Acetylation (N-term); Ornithine from Arginine
R.GYFGLIFPGCPS(+79.97)TYEEPAQQGR.R	N	26.28	2496.0820	22	5.0	833.0406	3	35.25	14	1845	OB4042.raw	2.98E3	1	1	99	120	
R.GYFGLIFPGC(+57.02)PSTYEEP(+15.99)AQQ(+.98)GRR.H	N	26.18	2646.2173	23	-5.6	883.0767	3	34.26	14	1787	OB4042.raw	5.92E4	1	1	99	121	Carbamidomethylation
R.LNAQ(+.98)RPDN(+.98)RIESEGGYIETWNPNN(+.98)QEFEC(+57.02)AGVALSR.L	N	26.06	4136.8770	36	0.6	1035.2294	4	32.92	14	1705	OB4042.raw	0	0	0	37	72	Carbamidomethylation
R.GYFGLIFP(+15.99)GC(+57.02)PSTYEEPAQQGR.R	N	26.02	2489.1321	22	-7.0	830.7140	3	35.75	14	1877	OB4042.raw	9.13E3	1	1	99	120	Carbamidomethylation
R.GYFGLIFP(+15.99)GC(+57.02)PSTYEEPAQQ(+.98)GR.R	N	25.48	2490.1160	22	-4.2	831.0443	3	35.88	14	1884	OB4042.raw	0	0	0	99	120	Carbamidomethylation
R.GYFGLIFPGC(+57.02)PSTYEEPAQ(+.98)QG.R	N	25.36	2318.0200	21	0.4	1160.0203	2	37.43	14	1983	OB4042.raw	0	0	0	99	119	Carbamidomethylation
R.R(+42.01)P(+15.99)FYSNAPQEIFIQQGR.G	N	24.64	2108.0439	17	-4.6	703.6869	3	30.85	14	1586	OB4042.raw	7.36E3	1	1	82	98	Acetylation (N-term)
R.GYFGLIFP(+15.99)GC(+57.02)PSTYEEPAQ(+.98)QGR.R	N	24.58	2490.1160	22	1.5	831.0490	3	35.35	14	1857	OB4042.raw	1.86E3	1	1	99	120	Carbamidomethylation
R.LNAQRPDN(+.98)RIESEGGYIETWNPN(+.98)NQEFEC(+57.02)AGVALSR.L	N	24.04	4135.8931	36	0.4	1034.9832	4	32.00	14	1699	OB4042.raw	1.58E5	1	1	37	72	Carbamidomethylation
R.GYFGLIFPGC(+57.02)P(+15.99)STYEEP(+15.99)AQ(+.98)Q(+.98)GRR.H	N	23.93	2663.1960	23	-8.0	888.7341	3	34.06	14	1790	OB4042.raw	4.49E4	1	1	99	121	Carbamidomethylation; Deamidation (NQ)
R.FNLAGN(+.98)HEQ(+.98)EFLR.Y	N	23.76	1575.7317	13	-2.1	526.2512	3	30.07	14	1525	OB4042.raw	6.86E3	1	1	196	208	
R.LNAQRP(+15.99)DNRIESEGGYIETWNPNNQ(+.98)EFEC(+57.02)AGVALSR.L	N	23.17	4150.9038	36	-2.0	1038.7334	4	32.52	14	1681	OB4042.raw	0	0	0	37	72	Carbamidomethylation

Peptide	Uniq	-10lgP	Mass	Length	ppm	m/z	z	RT	Fraction	Scan	Source File	Area Kontrola 13. traka 50 kDa	#Feature	#Feature Kontrola 13. traka 50 kDa	Start	End	PTM
R.GYFGLIFPGC(+57.02)P(+15.99)STYEEPAQQGRR.H	N	23.11	2645.2332	23	-1.2	882.7525	3	34.70	14	1813	OB4042.raw	0	0	0	99	121	Carbamidomethylation
R.GYFGLIFPGC(+57.02)P(+15.99)STYEEPAQQ(+.98)GRR.H	N	22.84	2646.2173	23	-5.6	883.0767	3	34.26	14	1801	OB4042.raw	5.92E4	1	1	99	121	Carbamidomethylation
**total 112 peptides**																	

tr|O82580|O82580_ARAHY

back to list

| Protein Coverage | Supporting Peptides |

Protein Coverage:

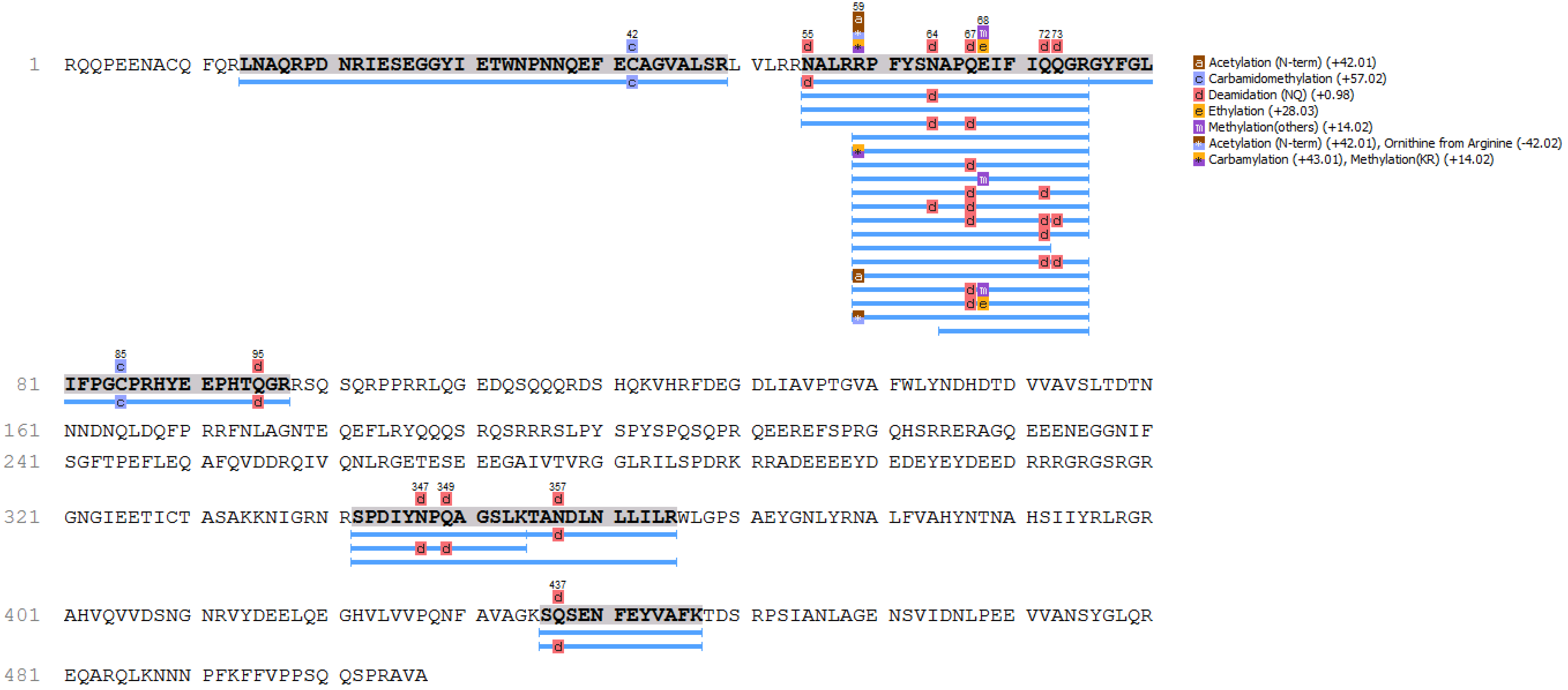

Supporting Peptides:

Peptide	Uniq	-10lgP	Mass	Length	ppm	m/z	z	RT	Fraction	Scan	Source File	Area Kontrola 13. traka 50 kDa	#Feature	#Feature Kontrola 13. traka 50 kDa	Start	End	PTM
K.SQSENFEYVAFK.T	N	71.67	1447.6619	12	-2.4	724.8380	2	30.46	14	1555	OB4042.raw	2.97E4	1	1	436	447	
K.SQ(+.98)SENFEYVAFK.T	N	68.89	1448.6459	12	-2.7	725.3298	2	30.85	14	1574	OB4042.raw	8.57E3	1	1	436	447	Deamidation (NQ)

Peptide	Uniq	-10lgP	Mass	Length	ppm	m/z	z	RT	Fraction	Scan	Source File	Area Kontrola 13. traka 50 kDa	#Feature	#Feature Kontrola 13. traka 50 kDa	Start	End	PTM
R.RPFYSNAPQEIFIQQGR.G	N	64.23	2050.0383	17	-1.5	684.3538	3	30.27	14	1519	OB4042.raw	5.16E5	1	1	59	75	
R.RPFYSNAPQEIFIQ(+.98)QGR.G	N	61.11	2051.0225	17	-1.4	684.6819	3	30.85	14	1561	OB4042.raw	8.24E5	1	1	59	75	
R.RPFYSN(+.98)APQ(+.98)EIFIQQGR.G	N	60.71	2052.0063	17	-2.8	1027.0098	2	31.34	14	1612	OB4042.raw	4.16E5	5	5	59	75	Deamidation (NQ)
R.RPFYSN(+.98)APQEIFIQ(+.98)QGR.G	N	59.57	2052.0063	17	-2.8	1027.0098	2	31.34	14	1607	OB4042.raw	7.62E3	1	1	59	75	
R.RPFYSNAPQ(+.98)EIFIQQGR.G	N	58.35	2051.0225	17	-1.4	684.6819	3	30.85	14	1585	OB4042.raw	8.38E5	3	3	59	75	Deamidation (NQ)
R.SPDIYNPQ(+.98)AGSLK.T	N	55.64	1389.6776	13	-2.4	695.8459	2	28.23	14	1414	OB4042.raw	9.57E3	1	1	342	354	
R.RPFYSN(+.98)APQEIFIQQGR.G	N	53.76	2051.0225	17	-2.8	1026.5178	2	30.95	14	1582	OB4042.raw	7.44E3	1	1	59	75	
R.SPDIYN(+.98)PQAGSLK.T	N	51.09	1389.6776	13	-2.4	695.8459	2	28.23	14	1410	OB4042.raw	9.57E3	1	1	342	354	
R.NALRRPFYSN(+.98)APQEIFIQQGR.G	N	50.06	2505.2876	21	-1.1	836.1041	3	30.17	14	1539	OB4042.raw	2.18E4	1	1	55	75	Deamidation (NQ)
R.N(+.98)ALRRPFYSNAPQEIFIQQGR.G	N	49.89	2505.2876	21	-0.3	836.1047	3	30.02	14	1527	OB4042.raw	2.18E4	1	1	55	75	Deamidation (NQ)
R.RPFYSNAPQ(+.98)EIFIQ(+.98)QGR.G	N	48.46	2052.0063	17	0.0	685.0109	3	31.15	14	1609	OB4042.raw	4.03E5	2	2	59	75	Deamidation (NQ)
N.APQEIFIQQGR.G	N	44.59	1285.6779	11	-1.9	643.8464	2	28.31	14	1423	OB4042.raw	0	0	0	65	75	
R.NALRRPFYSN(+.98)APQ(+.98)EIFIQQGR.G	N	44.22	2506.2717	21	-1.7	836.4316	3	31.25	14	1615	OB4042.raw	3.78E4	1	1	55	75	Deamidation (NQ)
R.LNAQ(+.98)RPDNRIESEGGYIETWN(+.98)PN(+.98)N(+.98)QEFEC(+57.02)AGVALSR.L	N	43.45	4137.8608	36	5.2	1035.4801	4	32.41	14	1674	OB4042.raw	0	0	0	14	49	Carbamidomethylation
R.N(+.98)ALRRPFYSN(+.98)APQEIFIQQGR.G	N	42.27	2506.2717	21	-1.7	836.4316	3	31.25	14	1605	OB4042.raw	3.78E4	1	1	55	75	
R.RPFYSNAPQEIFIQ.Q	N	41.89	1708.8573	14	-1.6	855.4364	2	32.20	14	1666	OB4042.raw	9.45E3	1	1	59	72	
R.RPFYSNAP(+15.99)QEIFIQQGR.G	N	41.19	2066.0332	17	-2.5	689.6848	3	31.98	14	1648	OB4042.raw	0	0	0	59	75	
R.SPDIYN(+.98)PQ(+.98)AGSLK.T	N	40.27	1390.6616	13	-3.3	696.3373	2	28.71	14	1454	OB4042.raw	1.04E3	1	1	342	354	Deamidation (NQ)
R.LN(+.98)AQRPDNRIESEGGYIETWN(+.98)PNNQEFEC(+57.02)AGVALSR.L	N	38.27	4135.8931	36	0.4	1034.9832	4	32.00	14	1663	OB4042.raw	1.58E5	1	1	14	49	Carbamidomethylation
R.N(+.98)ALRRPFYSNAPQ(+.98)EIFIQQGR.G	N	37.77	2506.2717	21	8.0	836.4397	3	30.54	14	1559	OB4042.raw	3.78E4	1	1	55	75	
R.RPFYSN(+.98)APQEIFIQ.Q	N	37.32	1709.8413	14	-2.1	855.9280	2	32.82	14	1698	OB4042.raw	4.44E3	1	1	59	72	
R.SPDIYNPQ(+.98)AGSLKTANDLNLLILR.W	N	36.80	2626.3965	24	-2.3	876.4727	3	34.16	14	1781	OB4042.raw	3.69E3	1	1	342	365	
R.N(+.98)ALRRPFYSN(+.98)APQ(+.98)EIFIQQGR.G	N	36.44	2507.2556	21	-0.3	836.7607	3	31.54	14	1621	OB4042.raw	0	0	0	55	75	Deamidation (NQ)
K.TAN(+.98)DLNLLILR.W	N	35.07	1255.7136	11	-1.1	628.8647	2	34.16	14	1788	OB4042.raw	2.89E4	1	1	355	365	Deamidation (NQ)
R.RPFYSNAP(+15.99)Q(+.98)EIFIQQGR.G	N	34.27	2067.0173	17	5.2	690.0181	3	31.90	14	1648	OB4042.raw	4.55E3	1	1	59	75	
R.LNAQ(+.98)RPDNRIESEGGYIETWNPNNQ(+.98)EFEC(+57.02)AGVALSR.L	N	33.98	4135.8931	36	0.4	1034.9832	4	32.00	14	1644	OB4042.raw	1.58E5	1	1	14	49	Carbamidomethylation
R.RPFYSNAPQE(+14.02)IFIQQGR.G	N	33.87	2064.0540	17	-3.2	689.0245	3	30.85	14	1589	OB4042.raw	6.86E3	1	1	59	75	Methylation(others)
R.RPFYSNAPQ(+.98)E(+14.02)IFIQQGR.G	N	32.73	2065.0381	17	-3.6	689.3523	3	31.43	14	1617	OB4042.raw	9.36E3	1	1	59	75	Deamidation (NQ); Methylation(others)
R.RPFYSNAPQE(+28.03)IFIQQGR.G	N	32.47	2078.0696	17	-2.3	693.6970	3	31.61	14	1624	OB4042.raw	2.75E3	1	1	59	75	
R.R(+42.01)PFYSNAP(+15.99)QEIFIQQGR.G	N	32.39	2108.0439	17	-4.6	703.6869	3	30.85	14	1577	OB4042.raw	7.36E3	1	1	59	75	Acetylation (N-term)
R.N(+.98)ALRRPFYSNAPQ(+.98)EIFIQQ(+.98)GR.G	N	32.12	2507.2556	21	-4.9	836.7569	3	31.79	14	1636	OB4042.raw	0	0	0	55	75	
R.RPFYSNAP(+15.99)QEIFIQ(+.98)QGR.G	N	32.05	2067.0173	17	0.5	690.0149	3	32.17	14	1659	OB4042.raw	0	0	0	59	75	Deamidation (NQ)
R.RPFYSNAPQ(+.98)EIFIQ(+.98)Q(+.98)GR.G	N	32.01	2052.9905	17	-3.6	685.3364	3	31.85	14	1632	OB4042.raw	7.14E4	1	1	59	75	Deamidation (NQ)
R.RPFYSNAPQ(+.98)EIFIQQ(+.98)GR.G	N	31.95	2052.0063	17	-2.4	685.0092	3	37.08	14	1960	OB4042.raw	1.09E3	1	1	59	75	Deamidation (NQ)
R.R(+43.01)(+14.02)PFYSNAPQEIFIQQGR.G	N	31.32	2107.0598	17	-1.2	703.3612	3	30.49	14	1544	OB4042.raw	5.56E3	1	1	59	75	Carbamylation; Methylation(KR)
R.RPFYSNAPQ(+.98)E(+28.03)IFIQQGR.G	N	30.30	2079.0537	17	-2.4	694.0250	3	32.10	14	1653	OB4042.raw	5.75E3	1	1	59	75	Deamidation (NQ); Ethylation
R.GYFGLIFPGC(+57.02)PRHYEEP(+15.99)HTQ(+.98)GR.R	Y	29.75	2634.2073	22	0.8	879.0790	3	33.77	14	1799	OB4042.raw	7.13E4	1	1	76	97	Carbamidomethylation; Deamidation (NQ)
R.LN(+.98)AQRPDNRIESEGGYIETWNPNNQEFEC(+57.02)AGVALSR.L	N	27.84	4134.9087	36	-4.8	1034.7317	4	32.66	14	1689	OB4042.raw	0	0	0	14	49	Carbamidomethylation
R.LN(+.98)AQRPDNRIESEGGYIETWN(+.98)PNN(+.98)QEFEC(+57.02)AGVALSR.L	N	27.21	4136.8770	36	-2.1	1035.2266	4	33.02	14	1711	OB4042.raw	0	0	0	14	49	Carbamidomethylation

Peptide	Uniq	-10lgP	Mass	Length	ppm	m/z	z	RT	Fraction	Scan	Source File	Area Kontrola 13. traka 50 kDa	#Feature	#Feature Kontrola 13. traka 50 kDa	Start	End	PTM
R.RPFYSN(+.98)APQEIFIQ(+.98)Q(+.98)GR.G	N	26.83	2052.9905	17	8.3	685.3446	3	37.13	14	1964	OB4042.raw	0	0	0	59	75	Deamidation (NQ)
R.R(+42.01)(-42.02)PFYSNAPQEIFIQQGR.G	N	26.56	2050.0271	17	-7.2	684.3462	3	34.21	14	1783	OB4042.raw	0	0	0	59	75	Acetylation (N-term); Ornithine from Arginine
R.LNAQ(+.98)RPDN(+.98)RIESEGGYIETWNPNN(+.98)QEFEC(+57.02)AGVALSR.L	N	26.06	4136.8770	36	0.6	1035.2294	4	32.92	14	1705	OB4042.raw	0	0	0	14	49	Carbamidomethylation
R.R(+42.01)P(+15.99)FYSNAPQEIFIQQGR.G	N	24.64	2108.0439	17	-4.6	703.6869	3	30.85	14	1586	OB4042.raw	7.36E3	1	1	59	75	Acetylation (N-term)
R.LNAQRPDN(+.98)RIESEGGYIETWNPN(+.98)NQEFEC(+57.02)AGVALSR.L	N	24.04	4135.8931	36	0.4	1034.9832	4	32.00	14	1699	OB4042.raw	1.58E5	1	1	14	49	Carbamidomethylation
R.LNAQRP(+15.99)DNRIESEGGYIETWNPNNQ(+.98)EFEC(+57.02)AGVALSR.L	N	23.17	4150.9038	36	-2.0	1038.7334	4	32.52	14	1681	OB4042.raw	0	0	0	14	49	Carbamidomethylation
**total 47 peptides**																	

tr|Q6PSU4|Q6PSU4_ARAHY
back to list

| **Protein Coverage** | **Supporting Peptides** |

Protein Coverage:

Oxidation (M) (+15.99)

1 GFDQRSRQFQ NLQNHRIVQI EAKPNTLVLP KHADADNILV IQQGQATVTV ANGNNRKSFN LDEGHALR**IP SGFISYILNR**

93

81 HDNQNLRVAK **ISMPVNTPGQ FEDFFPASSR DQSSYLQGFS R**NTLEAAFNA EFNEIRRVLL EENAGGEQEE RGQRRWSTRS

161 SENNEGVIVK VSKEHVEELT KHAKSVSKKG SEEGDITNPI NLREGEPDLS NNFGKLFEVK PDKKNPQLQD LDMMLTCVEI

255

241 KEGALMLPHF NSK**AMVIVVV NKGTGNLELV AVR**KEQQQRG RREEEEDEDE EEEGSNREVR RYTARLKEGD VFIMPAAHPV

321 AINASSELHL LGFGINAENN HRIFLAGDKD NVIDQIEKQA K**DLAFPGSGE QVEK**LIKNQK ESHFVSARPQ SQSQSPSSPE

401 KESPEKEDQE EENQGGKGPL LSILKAFN

Supporting Peptides:

Peptide	Uniq	-10lgP	Mass	Length	ppm	m/z	z	RT	Fraction	Scan	Source File	Area Kontrola 13. traka 50 kDa	#Feature	#Feature Kontrola 13. traka 50 kDa	Start	End	PTM
R.IPSGFISYILNR.H	Y	68.71	1378.7609	12	-1.8	690.3879	2	35.55	14	1865	OB4042.raw	3.09E4	1	1	69	80	
K.AM(+15.99)VIVVVNK.G	Y	42.90	987.5787	9	-3.8	494.7958	2	26.19	14	1290	OB4042.raw	2.74E3	1	1	254	262	Oxidation (M)
K.AM(+15.99)VIVVVNKGTGN(+.98)LELVAVR.K	Y	41.45	2098.1819	20	-2.4	700.4011	3	32.46	14	1677	OB4042.raw	0	0	0	254	273	Oxidation (M)
K.ISM(+15.99)PVNTPGQ(+.98)FEDFFPA.S	Y	33.70	1912.8552	17	2.2	957.4390	2	36.40	14	1917	OB4042.raw	0	0	0	91	107	Oxidation (M)
K.DLAFPGSGEQVEK.L	Y	27.01	1375.6619	13	-0.3	688.8395	2	29.21	14	1483	OB4042.raw	1.39E3	1	1	362	374	
K.ISM(+15.99)PVNTPGQ(+.98)FEDFFPASSRDQ(+.98)SSYLQ(+.98)GFSR.N	Y	24.01	3513.5669	31	0.2	1172.1990	3	34.49	14	1800	OB4042.raw	0	0	0	91	121	Oxidation (M)
K.ISM(+15.99)PVNTPGQ(+.98)FEDFFPASSR.D	Y	23.61	2243.0205	20	-3.5	748.6798	3	33.73	14	1754	OB4042.raw	0	0	0	91	110	Oxidation (M)
**total 7 peptides**																	

tr|N1NG13|N1NG13_ARAHY
back to list

| Protein Coverage | Supporting Peptides |

Protein Coverage:

Oxidation (M) (+15.99)

```
  1  MRGRVSPLML LLGILVLASV SATHAKSSPY QKKTENPCAQ RCLQSCQQEP DDLKQKACES RCTKLEYDPR CVYDPRGHTG
 81  TTNQRSPPGE RTRGRQPGDY DDDRRQPRRE EGGRWGPAGP REREREEDWR QPREDWRRPS HQQPRKIRPE GREGEQEWGT
161  PGSHVREETS RNNPFYFPSR RFSTRYGNQN GRIRVLQRFD QRSRQFQNLQ NHRIVQIEAK PNTLVLPKHA DADNILVIQQ
                                                                290
241  GQATVTVANG NNRKSFNLDE GHALRIPSGF ISYILNRHDN QNLRVAKISM PVNTPGQFED FFPASSRDQS SYLQGFSRNT
321  LEAAFNAEFN EIRRVLLEEN AGGEQEERGQ RRWSTRSSEN NEGVIVKVSK EHVEELTKHA KSVSKKGSEE EGDITNPINL
                                                                        453
401  REGEPDLSNN FGKLFEVKPD KKNPQLQDLD MMLTCVEIKE GALMLPHFNS KAMVIVVVNK GTGNLELVAV RKEQQQRGRR
481  EEEEDEDEEE EGSNREVRRY TARLKEGDVF IMPAAHPVAI NASSELHLLG FGINAENNHR IFLAGDKDNV IDQIEKQAKD
561  LAFPGSGEQV EKLIKNQKES HFVSARPQSQ SQSPSSPEKE SPEKEDQEEE NQGGKGPLLS ILKAFN
```

Supporting Peptides:

Peptide	Uniq	-10lgP	Mass	Length	ppm	m/z	z	RT	Fraction	Scan	Source File	Area Kontrola 13. traka 50 kDa	#Feature	#Feature Kontrola 13. traka 50 kDa	Start	End	PTM
R.IPSGFISYILNR.H	Y	68.71	1378.7609	12	-1.8	690.3879	2	35.55	14	1865	OB4042.raw	3.09E4	1	1	266	277	
K.AM(+15.99)VIVVVNK.G	Y	42.90	987.5787	9	-3.8	494.7958	2	26.19	14	1290	OB4042.raw	2.74E3	1	1	452	460	Oxidation (M)
K.AM(+15.99)VIVVVNKGTGN(+.98)LELVAVR.K	Y	41.45	2098.1819	20	-2.4	700.4011	3	32.46	14	1677	OB4042.raw	0	0	0	452	471	Oxidation (M)
K.ISM(+15.99)PVNTPGQ(+.98)FEDFFPA.S	Y	33.70	1912.8552	17	2.2	957.4390	2	36.40	14	1917	OB4042.raw	0	0	0	288	304	Oxidation (M)
K.DLAFPGSGEQVEK.L	Y	27.01	1375.6619	13	-0.3	688.8395	2	29.21	14	1483	OB4042.raw	1.39E3	1	1	560	572	
K.ISM(+15.99)PVNTPGQ(+.98)FEDFFPASSRDQ(+.98)SSYLQ(+.98)GFSR.N	Y	24.01	3513.5669	31	0.2	1172.1990	3	34.49	14	1800	OB4042.raw	0	0	0	288	318	Oxidation (M)
K.ISM(+15.99)PVNTPGQ(+.98)FEDFFPASSR.D	Y	23.61	2243.0205	20	-3.5	748.6798	3	33.73	14	1754	OB4042.raw	0	0	0	288	307	Oxidation (M)
**total 7 peptides**																	

P43238|ALL12_ARAHY

back to list

| Protein Coverage | Supporting Peptides |

Protein Coverage:

```
  1  MRGRVSPLML LLGILVLASV SATHAKSSPY QKKTENPCAQ RCLQSCQQEP DDLKQKACES RCTKLEYDPR CVYDPRGHTG
 81  TTNQRSPPGE RTRGRQPGDY DDDRRQPRRE EGGRWGPAGP REREREEDWR QPREDWRRPS HQQPRKIRPE GREGEQEWGT
161  PGSHVREETS RNNPFYFPSR RFSTRYGNQN GRIRVLQRFD QRSRQFQNLQ NHRIVQIEAK PNTLVLPKHA DADNILVIQQ
241  GQATVTVANG NNRKSFNLDE GHALRIPSGF ISYILNRHDN QNLRVAKISM PVNTPGQFED FFPASSRDQS SYLQGFSRNT
321  LEAAFNAEFN EIRRVLLEEN AGGEQEERGQ RRWSTRSSEN NEGVIVKVSK EHVEELTKHA KSVSKKGSEE EGDITNPINL
401  REGEPDLSNN FGKLFEVKPD KKNPQLQDLD MMLTCVEIKE GALMLPHFNS KAMVIVVVNK GTGNLELVAV RKEQQQRGRR
481  EEEEDEDEEE EGSNREVRRY TARLKEGDVF IMPAAHPVAI NASSELHLLG FGINAENNHR IFLAGDKDNV IDQIEKQAKD
561  LAFPGSGEQV EKLIKNQKES HFVSARPQSQ SQSPSSPEKE SPEKEDQEEE NQGGKGPLLS ILKAFN
```

Oxidation (M) (+15.99) — 290, 453

Supporting Peptides:

Peptide	Uniq	-10lgP	Mass	Length	ppm	m/z	z	RT	Fraction	Scan	Source File	Area Kontrola 13. traka 50 kDa	#Feature	#Feature Kontrola 13. traka 50 kDa	Start	End	PTM
R.IPSGFISYILNR.H	Y	68.71	1378.7609	12	-1.8	690.3879	2	35.55	14	1865	OB4042.raw	3.09E4	1	1	266	277	
K.AM(+15.99)VIVVVNK.G	Y	42.90	987.5787	9	-3.8	494.7958	2	26.19	14	1290	OB4042.raw	2.74E3	1	1	452	460	Oxidation (M)
K.AM(+15.99)VIVVVNKGTGN(+.98)LELVAVR.K	Y	41.45	2098.1819	20	-2.4	700.4011	3	32.46	14	1677	OB4042.raw	0	0	0	452	471	Oxidation (M)
K.ISM(+15.99)PVNTPGQ(+.98)FEDFFPA.S	Y	33.70	1912.8552	17	2.2	957.4390	2	36.40	14	1917	OB4042.raw	0	0	0	288	304	Oxidation (M)
K.DLAFPGSGEQVEK.L	Y	27.01	1375.6619	13	-0.3	688.8395	2	29.21	14	1483	OB4042.raw	1.39E3	1	1	560	572	
K.ISM(+15.99)PVNTPGQ(+.98)FEDFFPASSRDQ(+.98)SSYLQ(+.98)GFSR.N	Y	24.01	3513.5669	31	0.2	1172.1990	3	34.49	14	1800	OB4042.raw	0	0	0	288	318	Oxidation (M)
K.ISM(+15.99)PVNTPGQ(+.98)FEDFFPASSR.D	Y	23.61	2243.0205	20	-3.5	748.6798	3	33.73	14	1754	OB4042.raw	0	0	0	288	307	Oxidation (M)
**total 7 peptides**																	

tr|Q6PSU3|Q6PSU3_ARAHY

back to list

| Protein Coverage | Supporting Peptides |

Protein Coverage:

Oxidation (M) (+15.99)

```
1    MRGRVSPLML LLGILVLASV SATQAKSPYR KTENPCAQRC LQSCQQEPDD LKQKACESRC TKLEYDPRCV YDTGATNQRH
81   PPGERTRGRQ PGDYDDDRRQ PRREEGGRWG PAEPRERERE EDWRQPREDW RRPSHQQPRK IRPEGREGEQ EWGTPGSEVR
161  EETSRNNPFY FPSRRFSTRY GNQNGRIRVL QRFDQRSKQF QNLQNHRIVQ IEARPNTLVL PKHADADNIL VIQQGQATVT
241  VANGNNRKSF NLDEGHALRI PSGFISYILN RHDNQNLRVA KISMPVNTPG QFEDFFPASS RDQSSYLQGF SRNTLEAAFN
321  AEFNEIRRVL LEENAGGEQE ERGQRRRSTR SSDNEGVIVK VSKEHVQELT KHAKSVSKKG SEEEDITNPI NLRDGEPDLS
401  NNFGRLFEVK PDKKNPQLQD LDMMLTCVEI KEGALMLPHF NSKAMVIVVV NKGTGNLELV AVRKEQQQRG RREQEWEEEE
481  EDEEEEGSNR EVRRYTARLK EGDVFIMPAA HPVAINASSE LHLLGFGINA ENNHRIFLAG DKDNVIDQIE KQAKDLAFPG
561  SGEQVEKLIK NQRESHFVSA
```

Supporting Peptides:

Peptide	Uniq	-10lgP	Mass	Length	ppm	m/z	z	RT	Fraction	Scan	Source File	Area Kontrola 13. traka 50 kDa	#Feature	#Feature Kontrola 13. traka 50 kDa	Start	End	PTM
R.IPSGFISYILNR.H	Y	68.71	1378.7609	12	-1.8	690.3879	2	35.55	14	1865	OB4042.raw	3.09E4	1	1	260	271	
K.AM(+15.99)VIVVVNK.G	Y	42.90	987.5787	9	-3.8	494.7958	2	26.19	14	1290	OB4042.raw	2.74E3	1	1	444	452	Oxidation (M)
K.AM(+15.99)VIVVVNKGTGN(+.98)LELVAVR.K	Y	41.45	2098.1819	20	-2.4	700.4011	3	32.46	14	1677	OB4042.raw	0	0	0	444	463	Oxidation (M)
K.ISM(+15.99)PVNTPGQ(+.98)FEDFFPA.S	Y	33.70	1912.8552	17	2.2	957.4390	2	36.40	14	1917	OB4042.raw	0	0	0	282	298	Oxidation (M)
K.DLAFPGSGEQVEK.L	Y	27.01	1375.6619	13	-0.3	688.8395	2	29.21	14	1483	OB4042.raw	1.39E3	1	1	555	567	
K.ISM(+15.99)PVNTPGQ(+.98)FEDFFPASSRDQ(+.98)SSYLQ(+.98)GFSR.N	Y	24.01	3513.5669	31	0.2	1172.1990	3	34.49	14	1800	OB4042.raw	0	0	0	282	312	Oxidation (M)
K.ISM(+15.99)PVNTPGQ(+.98)FEDFFPASSR.D	Y	23.61	2243.0205	20	-3.5	748.6798	3	33.73	14	1754	OB4042.raw	0	0	0	282	301	Oxidation (M)
**total 7 peptides**																	

P43237|ALL11_ARAHY

back to list

| Protein Coverage | Supporting Peptides |

Protein Coverage:

Oxidation (M) (+15.99)

1 MRGRVSPLML LLGILVLASV SATQAKSPYR KTENPCAQRC LQSCQQEPDD LKQKACESRC TKLEYDPRCV YDTGATNQRH
81 PPGERTRGRQ PGDYDDDRRQ PRREEGGRWG PAEPRERERE EDWRQPREDW RRPSHQQPRK IRPEGREGEQ EWGTPGSEVR
161 EETSRNNPFY FPSRRFSTRY GNQNGRIRVL QRFDQRSKQF QNLQNHRIVQ IEARPNTLVL PKHADADNIL VIQQGQATVT

284
241 VANGNNRKSF NLDEGHALR**I PSGFISYILN R**HDNQNLRVA K**ISMPVNTPG QFEDFFPASS RDQSSYLQGF SR**NTLEAAFN

321 AEFNEIRRVL LEENAGGEQE ERGQRRRSTR SSDNEGVIVK VSKEHVQELT KHAKSVSKKG SEEEDITNPI NLRDGEPDLS

445
401 NNFGRLFEVK PDKKNPQLQD LDMMLTCVEI KEGALMLPHF NSK**AMVIVVV NKGTGNLELV AVR**KEQQQRG RREQEWEEEE

481 EDEEEEGSNR EVRRYTARLK EGDVFIMPAA HPVAINASSE LHLLGFGINA ENNHRIFLAG DKDNVIDQIE KQAK**DLAFPG**

561 **SGEQVEK**LIK NQRESHFVSA RPQSQSPSSP EKEDQEEENQ GGKGPLLSIL KAFN

Supporting Peptides:

Peptide	Uniq	-10lgP	Mass	Length	ppm	m/z	z	RT	Fraction	Scan	Source File	Area Kontrola 13. traka 50 kDa	#Feature	#Feature Kontrola 13. traka 50 kDa	Start	End	PTM
R.IPSGFISYILNR.H	Y	68.71	1378.7609	12	-1.8	690.3879	2	35.55	14	1865	OB4042.raw	3.09E4	1	1	260	271	
K.AM(+15.99)VIVVVNK.G	Y	42.90	987.5787	9	-3.8	494.7958	2	26.19	14	1290	OB4042.raw	2.74E3	1	1	444	452	Oxidation (M)
K.AM(+15.99)VIVVVNKGTGN(+.98)LELVAVR.K	Y	41.45	2098.1819	20	-2.4	700.4011	3	32.46	14	1677	OB4042.raw	0	0	0	444	463	Oxidation (M)
K.ISM(+15.99)PVNTPGQ(+.98)FEDFFPA.S	Y	33.70	1912.8552	17	2.2	957.4390	2	36.40	14	1917	OB4042.raw	0	0	0	282	298	Oxidation (M)
K.DLAFPGSGEQVEK.L	Y	27.01	1375.6619	13	-0.3	688.8395	2	29.21	14	1483	OB4042.raw	1.39E3	1	1	555	567	
K.ISM(+15.99)PVNTPGQ(+.98)FEDFFPASSRDQ(+.98)SSYLQ(+.98)GFSR.N	Y	24.01	3513.5669	31	0.2	1172.1990	3	34.49	14	1800	OB4042.raw	0	0	0	282	312	Oxidation (M)
K.ISM(+15.99)PVNTPGQ(+.98)FEDFFPASSR.D	Y	23.61	2243.0205	20	-3.5	748.6798	3	33.73	14	1754	OB4042.raw	0	0	0	282	301	Oxidation (M)
**total 7 peptides**																	

tr|B3IXL2|B3IXL2_ARAHY

back to list

| Protein Coverage | Supporting Peptides |

Protein Coverage:

Oxidation (M) (+15.99)

```
  1 MRGRVSPLML LLGILVLASV SATQAKSPYR KTENPCAQRC LQSCQQEPDD LKQKACESRC TKLEYDPRCV YDTGATNQRH
 81 PPGERTRGRQ PGDYDDDRRQ PRREEGGRWG PAEPRERERE EDWRQPREDW RRPSHQQPRK IRPEGREGEQ EWGTPGSEVR
161 EETSRNNPFY FPSRRFSTRY GNQNGRIRVL QRFDQRSKQF QNLQNHRIVQ IEARPNTLVL PKHADADNIL VIQQGQATVT
                                                 284
241 VANGNNRKSF NLDEGHALRI PSGFISYILN RHDNQNLRVA KISMPVNTPG QFEDFFPASS RDQSSYLQGF SRNTLEAAFN
321 AEFNEIRRVL LEENAGGEQE ERGQRRRSTR SSDNEGVIVK VSKEHVQELT KHAKSVSKKG SEEEDITNPI NLRDGEPDLS
                                                   445
401 NNFGRLFEVK PDKKNPQLQD LDMMLTCVEI KEGALMLPHF NSKAMVIVVV NKGTGNLELV AVRKEQQQRG RREQEWEEEE
481 EDEEEEGSNR EVRRYTARLK EGDVFIMPAA HPVAINASSE LHLLGFGINA ENNHRIFLAG DKDNVIDQIE KQAKDLAFPG
561 SGEQVEKLIK NQRESHFVSA RPQSQSPSSP EKEDQEEENQ GGKGPLLSIL KAFN
```

Supporting Peptides:

Peptide	Uniq	-10lgP	Mass	Length	ppm	m/z	z	RT	Fraction	Scan	Source File	Area Kontrola 13. traka 50 kDa	#Feature	#Feature Kontrola 13. traka 50 kDa	Start	End	PTM
R.IPSGFISYILNR.H	Y	68.71	1378.7609	12	-1.8	690.3879	2	35.55	14	1865	OB4042.raw	3.09E4	1	1	260	271	
K.AM(+15.99)VIVVVNK.G	Y	42.90	987.5787	9	-3.8	494.7958	2	26.19	14	1290	OB4042.raw	2.74E3	1	1	444	452	Oxidation (M)
K.AM(+15.99)VIVVVNKGTGN(+.98)LELVAVR.K	Y	41.45	2098.1819	20	-2.4	700.4011	3	32.46	14	1677	OB4042.raw	0	0	0	444	463	Oxidation (M)
K.ISM(+15.99)PVNTPGQ(+.98)FEDFFPA.S	Y	33.70	1912.8552	17	2.2	957.4390	2	36.40	14	1917	OB4042.raw	0	0	0	282	298	Oxidation (M)
K.DLAFPGSGEQVEK.L	Y	27.01	1375.6619	13	-0.3	688.8395	2	29.21	14	1483	OB4042.raw	1.39E3	1	1	555	567	
K.ISM(+15.99)PVNTPGQ(+.98)FEDFFPASSRDQ(+.98)SSYLQ(+.98)GFSR.N	Y	24.01	3513.5669	31	0.2	1172.1990	3	34.49	14	1800	OB4042.raw	0	0	0	282	312	Oxidation (M)
K.ISM(+15.99)PVNTPGQ(+.98)FEDFFPASSR.D	Y	23.61	2243.0205	20	-3.5	748.6798	3	33.73	14	1754	OB4042.raw	0	0	0	282	301	Oxidation (M)
**total 7 peptides**																	

tr|E5G077|E5G077_ARAHY

back to list

| Protein Coverage | Supporting Peptides |

Protein Coverage:

1 MAKLLALSLC FCVLVLGASS VTFRQGGEEN ECQFQRLNAQ RPDNRIESEG GYIETWNPNN QEFQCAGVAL SRTVLRRNAL

Carbamidomethylation (+57.02)
Deamidation (NQ) (+0.98)

81 RRPFYSNAPL EIYVQQGSGY FGLIFPGCPS TYEEPAQEGR RYQSQKPSRR FQVGQDDPSQ QQQDSHQKVH RFDEGDLIAV

161 PTGVAFWMYN DEDTDVVTVT LSDTSSIHNQ LDQFPRRFYL AGNQEQEFLR YQQQQGSRPH YRQISPRVRG DEQENEGSNI

241 FSGFAQEFLQ HAFQVDRQTV ENLRGENERE EQGAIVTVKG GLRILSPDEE DESSRSPPNR REEFDEDRSR PQQRGKYDEN

321 RRGYKNGIEE TICSASVKKN LGRSSNPDIY NPQAGSLRSV NELDLPILGW LGLSAQHGTI YRNAMFVPHY TLNAHTIVVA

401 LNGRAHVQVV DSNGNRVYDE ELQEGHVLVV PQNFAVAAKA QSENYEYLAF KTDSRPSIAN QAGENSIIDN LPEEVVANSY

481 RLPREQARQL KNNNPFKFFV PPFDHQSMRE VA

Supporting Peptides:

Peptide	Uniq	-10lgP	Mass	Length	ppm	m/z	z	RT	Fraction	Scan	Source File	Area Kontrola 13. traka 50 kDa	#Feature	#Feature Kontrola 13. traka 50 kDa	Start	End	PTM
K.AQSENYEYLAFK.T	Y	70.14	1461.6776	12	-1.4	731.8466	2	30.45	14	1553	OB4042.raw	0	0	0	440	451	
K.AQ(+.98)SENYEYLAFK.T	Y	68.32	1462.6616	12	-2.2	732.3381	2	30.66	14	1568	OB4042.raw	1.89E4	1	1	440	451	Deamidation (NQ)
R.SSNPDIYN(+.98)PQ(+.98)AGSLR.S	Y	49.84	1619.7427	15	-3.4	810.8776	2	28.64	14	1445	OB4042.raw	3.75E3	1	1	344	358	Deamidation (NQ)
R.SVNELDLPILGWLGLSAQHGTIYR.N	Y	47.81	2651.4070	24	-1.5	884.8102	3	38.45	14	2050	OB4042.raw	8.12E4	3	3	359	382	
R.SSNPDIYNPQ(+.98)AGSLR.S	Y	47.27	1618.7587	15	-1.4	810.3873	2	28.18	14	1422	OB4042.raw	1.12E4	1	1	344	358	
R.SSNPDIYNPQAGSLR.S	Y	45.82	1617.7747	15	-3.0	809.8939	2	27.55	14	1377	OB4042.raw	2.2E3	1	1	344	358	
R.SVNELDLPILGWLGLSAQ(+.98)HGTIYR.N	Y	45.49	2652.3911	24	-2.0	885.1378	3	38.78	14	2081	OB4042.raw	1.4E5	4	4	359	382	Deamidation (NQ)
R.SSNPDIYN(+.98)PQAGSLR.S	Y	43.54	1618.7587	15	-1.4	810.3873	2	28.18	14	1405	OB4042.raw	1.12E4	1	1	344	358	Deamidation (NQ)
R.SVN(+.98)ELDLPILGWLGLSAQ(+.98)HGTIYR.N	Y	40.16	2653.3750	24	0.6	885.4681	3	39.14	14	2092	OB4042.raw	3.18E3	1	1	359	382	Deamidation (NQ)
R.LNAQRPDNRIESEGGYIETWNPNNQEFQ(+.98)C(+57.02)AGVALSR.T	Y	32.11	4133.9248	36	-2.5	1034.4882	4	31.68	14	1629	OB4042.raw	0	0	0	37	72	Carbamidomethylation
**total 10 peptides**																	

tr|Q6IWG5|Q6IWG5_ARAHY

back to list

| Protein Coverage | Supporting Peptides |

Protein Coverage:

63 c

1 KLLALSLCFC VLVLGASSVT FRQGGEENEC QFQR**LNAQRP DNRIESEGGY IETWNPNNQE FQCAGVALSR** TVLRRNALRR

c Carbamidomethylation (+57.02)
d Deamidation (NQ) (+0.98)

81 PFYSNAPLEI YVQQGSGYFG LIFPGCPSTY EEPAQEGRRY QSQKPSRRFQ VGQDDPSQQQ QDSHQKVHRF DEGDLIAVPT
161 GVAFWMYNDE DTDVVTVTLS DTSSIHNQLD QFPRRFYLAG NQEQEFLRYQ QQQGSRPHYR QISPRVRGDE QENEGSNIFS
241 GFAQEFLQHA FQVDRQTVEN LRGENEREEQ GAIVTVKGGL RILSPDEEDE SSRSPPSRRE EFDEDRSRPQ QRGKYDENRR

349 d 351 d 359 d 374 d

321 GYKNGIEETI CSASVKKNLG R**SSNPDIYNP QAGSLRSVNE LDLPILGWLG LSAQHGTIYR** NAMFVPHYTL NAHTIVVALN

439 d

401 GRAHVQVVDS NGNRVYDEEL QEGHVLVVPQ NFAVAAK**AQS ENYEYLAFK**T DSRPSIANLA GENSIIDNLP EEVVANSYRL

481 PREQARQLKN NNPFKFFVPP FDHQSMREVA

Supporting Peptides:

Peptide	Uniq	-10lgP	Mass	Length	ppm	m/z	z	RT	Fraction	Scan	Source File	Area Kontrola 13. traka 50 kDa	#Feature	#Feature Kontrola 13. traka 50 kDa	Start	End	PTM
K.AQSENYEYLAFK.T	Y	70.14	1461.6776	12	-1.4	731.8466	2	30.45	14	1553	OB4042.raw	0	0	0	438	449	
K.AQ(+.98)SENYEYLAFK.T	Y	68.32	1462.6616	12	-2.2	732.3381	2	30.66	14	1568	OB4042.raw	1.89E4	1	1	438	449	Deamidation (NQ)
R.SSNPDIYN(+.98)PQ(+.98)AGSLR.S	Y	49.84	1619.7427	15	-3.4	810.8776	2	28.64	14	1445	OB4042.raw	3.75E3	1	1	342	356	Deamidation (NQ)
R.SVNELDLPILGWLGLSAQHGTIYR.N	Y	47.81	2651.4070	24	-1.5	884.8102	3	38.45	14	2050	OB4042.raw	8.12E4	3	3	357	380	
R.SSNPDIYNPQ(+.98)AGSLR.S	Y	47.27	1618.7587	15	-1.4	810.3873	2	28.18	14	1422	OB4042.raw	1.12E4	1	1	342	356	
R.SSNPDIYNPQAGSLR.S	Y	45.82	1617.7747	15	-3.0	809.8939	2	27.55	14	1377	OB4042.raw	2.2E3	1	1	342	356	
R.SVNELDLPILGWLGLSAQ(+.98)HGTIYR.N	Y	45.49	2652.3911	24	-2.0	885.1378	3	38.78	14	2081	OB4042.raw	1.4E5	4	4	357	380	Deamidation (NQ)
R.SSNPDIYN(+.98)PQAGSLR.S	Y	43.54	1618.7587	15	-1.4	810.3873	2	28.18	14	1405	OB4042.raw	1.12E4	1	1	342	356	Deamidation (NQ)
R.SVN(+.98)ELDLPILGWLGLSAQ(+.98)HGTIYR.N	Y	40.16	2653.3750	24	0.6	885.4681	3	39.14	14	2092	OB4042.raw	3.18E3	1	1	357	380	Deamidation (NQ)
R.LNAQRPDNRIESEGGYIETWNPNNQEFQ(+.98)C(+57.02)AGVALSR.T	Y	32.11	4133.9248	36	-2.5	1034.4882	4	31.68	14	1629	OB4042.raw	0	0	0	35	70	Carbamidomethylation
**total 10 peptides**																	

tr|Q0GM57|Q0GM57_ARAHY

back to list

| Protein Coverage | Supporting Peptides |

Protein Coverage:

c Carbamidomethylation (+57.02)
d Deamidation (NQ) (+0.98)

```
  1 MAKLLALSLC FCVLVLGASS VTFRQGGEEN ECQFQRLNAQ RPDNRIESEG GYIETWNPNN QEFQCAGVAL SRTVLRRNAL
 81 RRPFYSNAPL EIYVQQGSGY FGLIFPGCPS TYEEPAQEGR RYQSQKPSRR FQVGQDDPSQ QQQDSHQKVH RFDEGDLIAV
161 PTGVAFWMYN DEDTDVVTVT LSDTSSIHNQ LDQFPRRFYL AGNQEQEFLR YQQQQGSRPH YRQISPRVRG DEQENEGSNI
241 FSGFAQEFLQ HAFQVDRQTV ENLRGENERE EQGAIVTVKG GLRILSPDEE DESSRSPPSR REEFDEDRSR PQQRGKYDEN
321 RRGYKNGIEE TICSASVKKN LGRSSNPDIY NPQAGSLRSV NELDLPILGW LGLSAQHGTI YRNAMFVPHY TLNAHTIVVA
401 LNGRAHVQVV DSNGNRVYDE ELQEGHVLVV PQNFAVAAKA QSENYEYLAF KTDSRPSIAN LAGENSIIDN LPEEVVANSY
481 RLPREQARQL KNNNPFKFFV PPFDHQSMRE VA
```

Supporting Peptides:

Peptide	Uniq	-10lgP	Mass	Length	ppm	m/z	z	RT	Fraction	Scan	Source File	Area Kontrola 13. traka 50 kDa	#Feature	#Feature Kontrola 13. traka 50 kDa	Start	End	PTM
K.AQSENYEYLAFK.T	Y	70.14	1461.6776	12	-1.4	731.8466	2	30.45	14	1553	OB4042.raw	0	0	0	440	451	
K.AQ(+.98)SENYEYLAFK.T	Y	68.32	1462.6616	12	-2.2	732.3381	2	30.66	14	1568	OB4042.raw	1.89E4	1	1	440	451	Deamidation (NQ)
R.SSNPDIYN(+.98)PQ(+.98)AGSLR.S	Y	49.84	1619.7427	15	-3.4	810.8776	2	28.64	14	1445	OB4042.raw	3.75E3	1	1	344	358	Deamidation (NQ)
R.SVNELDLPILGWLGLSAQHGTIYR.N	Y	47.81	2651.4070	24	-1.5	884.8102	3	38.45	14	2050	OB4042.raw	8.12E4	3	3	359	382	
R.SSNPDIYNPQ(+.98)AGSLR.S	Y	47.27	1618.7587	15	-1.4	810.3873	2	28.18	14	1422	OB4042.raw	1.12E4	1	1	344	358	
R.SSNPDIYNPQAGSLR.S	Y	45.82	1617.7747	15	-3.0	809.8939	2	27.55	14	1377	OB4042.raw	2.2E3	1	1	344	358	
R.SVNELDLPILGWLGLSAQ(+.98)HGTIYR.N	Y	45.49	2652.3911	24	-2.0	885.1378	3	38.78	14	2081	OB4042.raw	1.4E5	4	4	359	382	Deamidation (NQ)
R.SSNPDIYN(+.98)PQAGSLR.S	Y	43.54	1618.7587	15	-1.4	810.3873	2	28.18	14	1405	OB4042.raw	1.12E4	1	1	344	358	Deamidation (NQ)
R.SVN(+.98)ELDLPILGWLGLSAQ(+.98)HGTIYR.N	Y	40.16	2653.3750	24	0.6	885.4681	3	39.14	14	2092	OB4042.raw	3.18E3	1	1	359	382	Deamidation (NQ)
R.LNAQRPDNRIESEGGYIETWNPNNQEFQ(+.98)C(+57.02)AGVALSR.T	Y	32.11	4133.9248	36	-2.5	1034.4882	4	31.68	14	1629	OB4042.raw	0	0	0	37	72	Carbamidomethylation
**total 10 peptides**																	

tr|T2B9M0|T2B9M0_ARAHY

back to list

| Protein Coverage | Supporting Peptides |

Protein Coverage:

```
  1 MSNFKSKYHD ELIANAAYIG TPGKGILAAD ESTGTIGKRL SSINVENVET NRRALRELLF TTPGALQYLS GVILFEETLY
 81 QSTAAGKPFV EVLKEGGVLP GIKVDKGTVE LAGTNGETTT QGLDGLGQRC AKYYEAGARF AKWRAVLKIG PTEPSELAIH
161 ENAYGLARYA AICQENGLVP IVEPEILVDG PHDIHKCAAV TERVLAACYK ALNDHHVLLE GTLLKPNMVT PGSDSAKVAP
241 EVVAEHTVRA LQRTVPSAVP AVVFLSGGQS EEEATLNLNA INQVKGKKPW SLSFSFGRAL QQSTLKAWSG KEENVKKAQE
321 AFLTRAKANS EATLGTYKGS ANLGEGASES LHVKDYKY
```

Supporting Peptides:

Peptide	Uniq	-10lgP	Mass	Length	ppm	m/z	z	RT	Fraction	Scan	Source File	Area Kontrola 13. traka 50 kDa	#Feature	#Feature Kontrola 13. traka 50 kDa	Start	End	PTM
K.IGPTEPSELAIHENAYGLAR.Y	Y	35.36	2137.0803	20	-2.4	713.3672	3	29.87	14	1515	OB4042.raw	2.3E4	1	1	149	168	

Peptide	Uniq	-10lgP	Mass	Length	ppm	m/z	z	RT	Fraction	Scan	Source File	Area Kontrola 13. traka 50 kDa	#Feature	#Feature Kontrola 13. traka 50 kDa	Start	End	PTM
**total 1 peptides**																	

tr|C9W981|C9W981_ARAHY

back to list

| Protein Coverage | Supporting Peptides |

Protein Coverage:

```
  1  MTDTTDDIAE EISFQSFDDD CRLLGNLLND ILQREVGTTV VDKLERIRVL AQSGCNMRQA GIVDMAEMLE KQLASELSKM
 81  TLEEALTLAR AFSHYLTLMG IAETHHRVRK RGNNIAQTAK SCDDIFNQLV QGGVSPDELY DTVCKQEVEI VLTAHPTQIN
161  RRTLQYKHIR IAHLLDYNDR PDLTIEDREM VIEDLVREIT SIWQTDELRR QKPTPVDEAR AGLNIVEQSL WKAVPHYLHR
241  VSNALKKHTG KPLPLTCTPI KFGSWMGGDR DGNPNVTAKV TKDVSLLSRW MAIDLYIREV DSLRFELSMN QCSESLSRLA
321  HEILEEANLE NRHENWNQPV SRSQSLPKQL PARAHLPSFA ENGEAQHPRL DIPGPDHSQH NHKEGEVSST LFKIGETSAN
401  SGASAAAISP SSSFNSIQQL GQRKSSAGSQ IGRSSFQKLM EPKLPQLPGI APYRVVLGNV KDKLERSRRR LELLLEDVSC
481  DNDPLDYYET TDQLLEPLLL CYESLQSCGS GVLADGRLAD LIRRVATFGM VLMKLDLRQE SGRHAETIDA ITKYLDLGTY
561  SEWDEEKKLE FLTRELKGKR PLVPHSIEVP HEVKEVLDTF RIAAELGSDS LGAYVISMAS NASDVLAVEL LQKDARLSVA
641  GDLGRECPGG TLRVVPLFET VKDLRGAGSV IRKLLSIDWY REHVIKNHNG HQEVMVGYSD SGKDAGRFTA AWELYKAQED
721  VVAACNEYGI KVTLFHGRGG SIGRGGGPTY LAIQSQPPGS VMGTLRSTEQ GEMIDAKFGL PQIAVRQLEI YTTAVLLATL
801  RPPHPPREEK WRKVIEEISN ISCQCYRSVV YENPEFLSYF HEATPEAELG FLNIGSRPAR RKSSKGIGHL RAIPWLFAWT
881  QTRFVLPAWL GVGAGLKGAC EKGHTEELKE MYKEWPFFQS TIDLIEMVLG KADIPIAKHY DEVLVSKERQ ELGRELRSEL
961  MTAEKFVLVI SGHEKLQQNN RSLRRLIENR LPFLNPLNML QVEILKRLRR EDDNRKIRDA LLITINGIAA GMKNTG
```

Supporting Peptides:

Peptide	Uniq	-10lgP	Mass	Length	ppm	m/z	z	RT	Fraction	Scan	Source File	Area Kontrola 13. traka 50 kDa	#Feature	#Feature Kontrola 13. traka 50 kDa	Start	End	PTM
R.FVLPAWLGVGAGLK.G	Y	22.72	1426.8336	14	8.6	476.6236	3	38.54	14	2054	OB4042.raw	3.94E3	1	1	884	897	
**total 1 peptides**																	

Peptide List

Prepared with PEAKS ™ (bioinfor.com)

1. Notes Gastric Digest Raw peanut Band #B 47-55 kDa (PTM505)

2. Result Statistics

Figure 1. False discovery rate (FDR) curve. X axis is the number of peptide-spectrum matches (PSM) being kept. Y axis is the corresponding FDR.

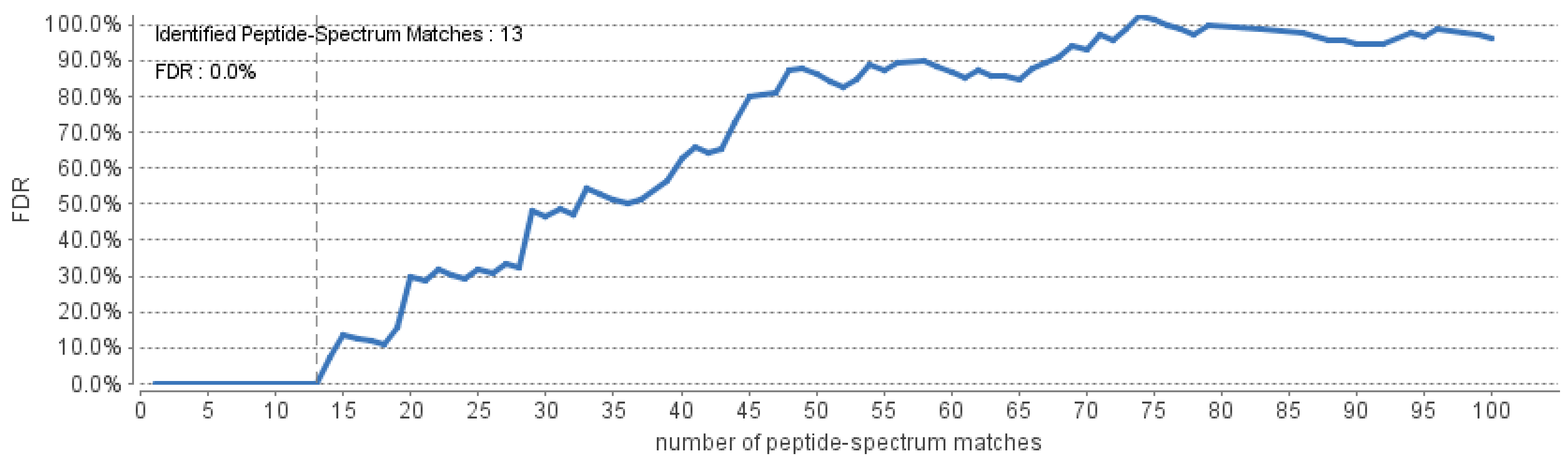

Figure 2. PSM score distribution. **(a)** Distribution of PEAKS peptide score; **(b)** Scatterplot of PEAKS peptide score versus precursor mass error.

(a)

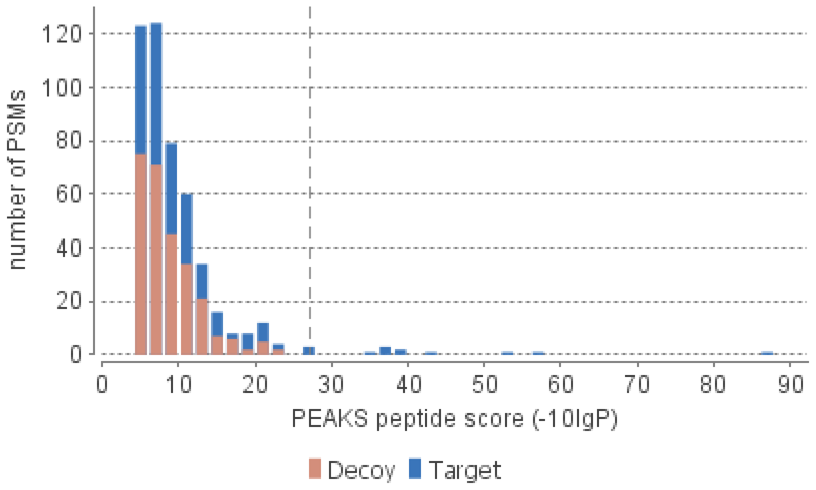

(b)

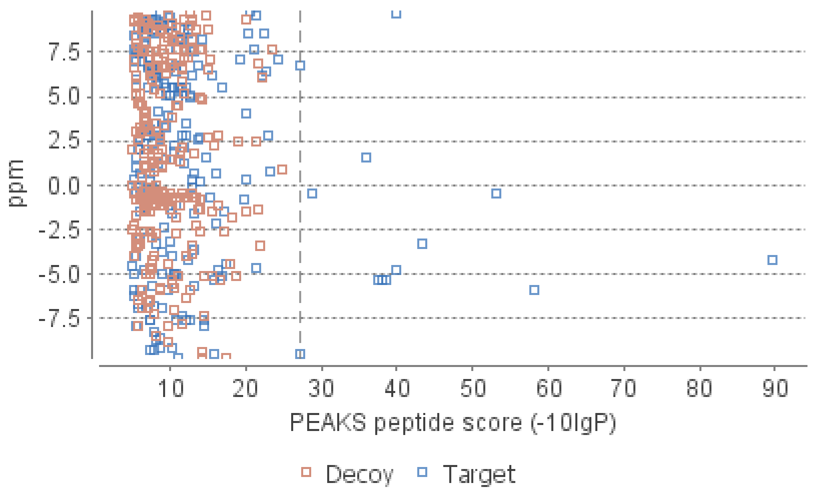

Figure 3. Distribution of peptide feature detection. **(a)** Feature m/z distribution; **(b)** Feature RT distribution

(a)

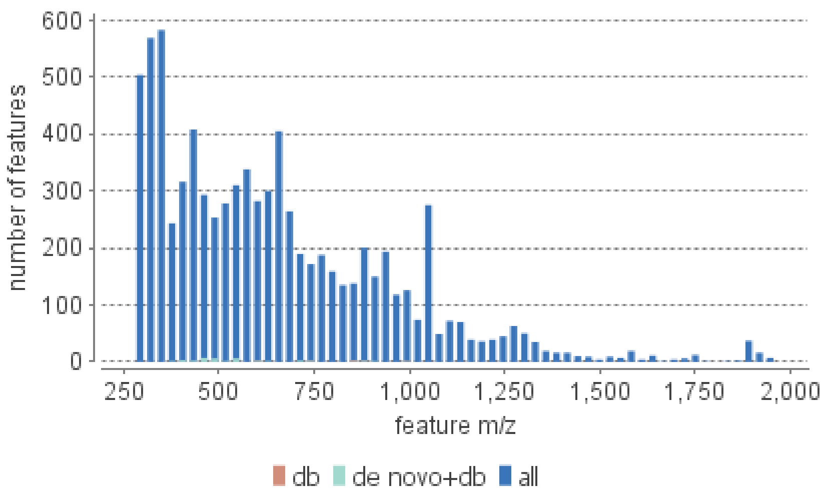

(b)

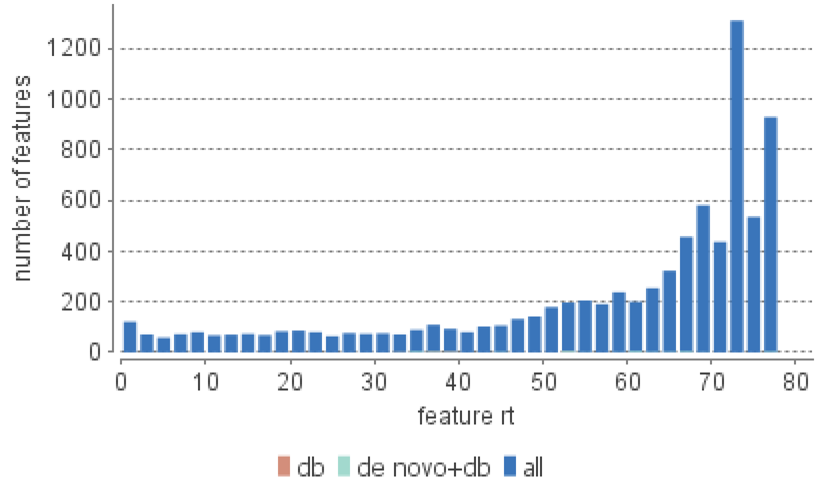

Figure 4. Distribution of identified peptide features. **(a)** Feature abundance distribution **(b)** *De novo* sequencing validation.

(a)

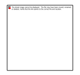

(b)

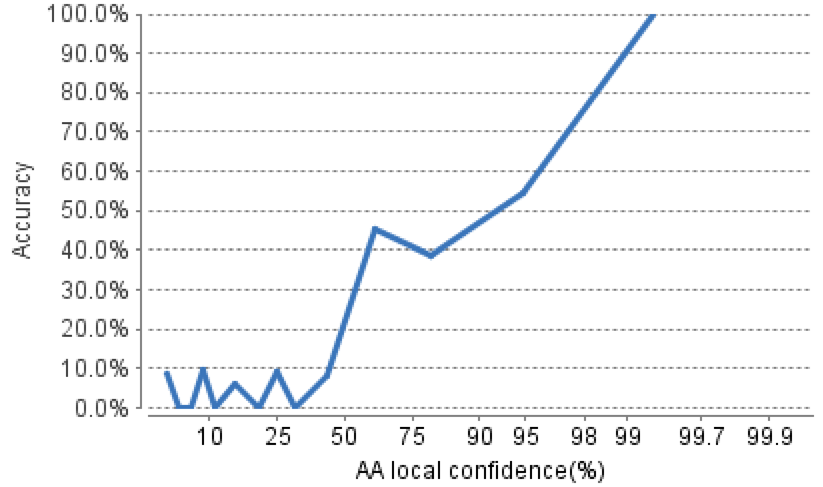

Table 1. Statistics of data.

# of MS scans	2741
# of MS/MS scans	1859
# of Features	8174
# of Chimera scans	324

Table 2. Result filtration parameters.

Peptide -10lgP	≥27.1
PTM Ascore	≥20

Table 4. PTM profile.

**Name**	**ΔMass**	**Position**	**#PSM**	**-10lgP**	**Abundance**	**AScore**
Deamidation	.98	NQ	6	53.02	2.53E3	0.00
Dimethylation(KR)	28.03	R	1	27.13	1.42E4	13.58
Carboxylation	43.99	E	1	27.13	1.42E4	6.65

Protein -10lgP	≥20
Proteins unique peptides	≥1
De novo ALC Score	≥50%

Table 3. Statistics of filtered result.

Peptide-Spectrum Matches	13
Peptide sequences	9
Protein groups	3
Proteins	13
Proteins (#Unique Peptides)	0 (>2); 1 (=2); 12 (=1);
FDR (Peptide-Spectrum Matches)	0.0%
FDR (Peptide Sequences)	0.0%
FDR (Protein Group)	0.0%
De Novo Only Spectra	47
# of identified Features	39
# of identified MS/MS scans	12

3. Experiment Control

Figure 5. Precursor mass error of peptide-spectrum matches (PSM) in filtered result. **(a)** Distribution of precursor mass error in ppm; **(b)** Scatterplot of precursor m/z versus precursor mass error in ppm.

(a)

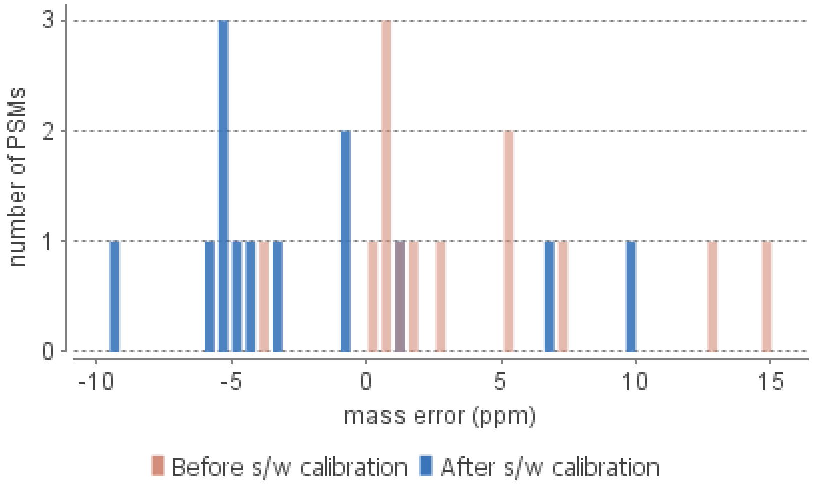

(b)

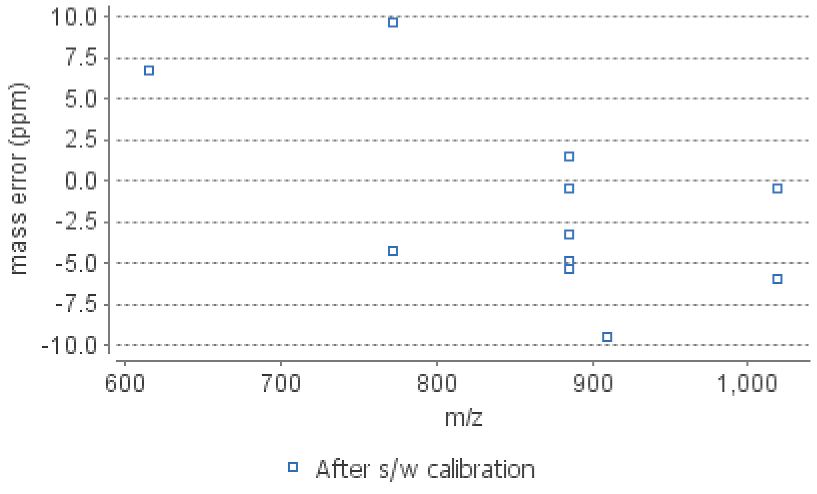

Table 5. Number of identified peptides in each sample by the number of missed cleavages

Missed Cleavages	0	1	2	3	4+
Digest traka B 45-50 kDa	5	3	1	0	0

4. Other Information

Table 6. Search parameters.

Search Engine Name: PEAKS
Parent Mass Error Tolerance: 10.0 ppm
Fragment Mass Error Tolerance: 0.5 Da
Precursor Mass Search Type: monoisotopic
Enzyme: Trypsin
Max Missed Cleavages: 2
Digest Mode: Unspecific
Fixed Modifications:
  Carbamidomethylation: 57.02
Variable Modifications:
  Deamidation (NQ): 0.98
  Oxidation (M): 15.99
  Hydroxylation Pro: 15.99
  Acetylation (K): 42.01
  Acetylation (Protein N-term): 42.01
  Acetylation (N-term): 42.01
  Amidation: -0.98
  Beta-methylthiolation: 45.99
  and 305 more...
Max Variable PTM Per Peptide: 5
Database: Uniprot_Peanut-3818_Jul18
Taxon: All
Contaminant Database: contaminantsMQ_mar19
Searched Entry: 1723
FDR Estimation: Enabled
De novo score (ALC%) threshold: 15
Peptide hit threshold (-10logP): 30.0
Peaks run ID: 499
Merge Options: no merge
Precursor Options: corrected
Charge Options: no correction
Filter Charge: 2 - 8
Process: true
Associate chimera: yes

Table 7. Instrument parameters.

Fractions: OB4067.raw
Ion Source: ESI(nano-spray)
Fragmentation Mode: CID, CAD(y and b ions)
MS Scan Mode: FT-ICR/Orbitrap
MS/MS Scan Mode: Linear Ion Trap

Protein List

Protein Accession Contains:
Protein Description Contains:
Peptide Sample Area >=
Protein Ptm Contains:

Protein Group	Protein ID	Accession	-10lgP	Coverage (%)	Coverage (%) Digest traka B 45-50 kDa	Area Digest traka B 45-50 kDa	#Peptides	#Unique	#Spec Digest traka B 45-50 kDa	PTM	Avg. Mass	Description
**2**	20576	tr|Q9FZ11|Q9FZ11_ARAHY	103.35	7	7	2.7E4	2	2	3	Y	60449	Gly1 OS=Arachis hypogaea OX=3818 GN=Gly1 PE=2 SV=1
**2**	20580	tr|Q647H4|Q647H4_ARAHY	89.78	2	2	1.28E4	1	1	2	Y	61506	Arachin Ahy-1 OS=Arachis hypogaea OX=3818 PE=2 SV=1
**2**	20591	tr|Q6T2T4|Q6T2T4_ARAHY	89.78	2	2	1.28E4	1	1	2	Y	61499	Storage protein OS=Arachis hypogaea OX=3818 PE=2 SV=1
**2**	20582	tr|Q8LKN1|Q8LKN1_ARAHY	89.78	2	2	1.28E4	1	1	2	Y	61738	Allergen Arah3/Arah4 OS=Arachis hypogaea OX=3818 PE=3 SV=1
**2**	20581	tr|Q647H3|Q647H3_ARAHY	89.78	2	2	1.28E4	1	1	2	Y	61532	Arachin Ahy-2 OS=Arachis hypogaea OX=3818 PE=2 SV=1
**2**	20574	tr|A1DZF0|A1DZF0_ARAHY	89.78	2	2	1.28E4	1	1	2	Y	60375	Arachin 6 OS=Arachis hypogaea OX=3818 PE=2 SV=1
**2**	20578	tr|B5TYU1|B5TYU1_ARAHY	89.78	2	2	1.28E4	1	1	2	Y	60624	Arachin Arah3 isoform OS=Arachis hypogaea OX=3818 PE=1 SV=1
**2**	20579	tr|Q5I6T2|Q5I6T2_ARAHY	89.78	2	2	1.28E4	1	1	2	Y	60736	Arachin Ahy-4 OS=Arachis hypogaea OX=3818 PE=2 SV=1
**2**	20577	tr|Q9SQH7|Q9SQH7_ARAHY	89.78	2	2	1.28E4	1	1	2	Y	61011	Glycinin OS=Arachis hypogaea OX=3818 GN=Arah4 PE=2 SV=1
**1**	20593	tr|Q6IWG5|Q6IWG5_ARAHY	43.22	5	5	9.75E4	1	1	7	Y	58061	Glycinin (Fragment) OS=Arachis hypogaea OX=3818 PE=2 SV=1
**1**	20595	tr|E5G077|E5G077_ARAHY	43.22	5	5	9.75E4	1	1	7	Y	58305	Ara h 3 allergen OS=Arachis hypogaea OX=3818 GN=ara h 3 PE=3 SV=1
**1**	20594	tr|Q0GM57|Q0GM57_ARAHY	43.22	5	5	9.75E4	1	1	7	Y	58263	Iso-Ara h3 OS=Arachis hypogaea OX=3818 PE=2 SV=1
**85**	21693	tr|A0A290G0J9|A0A290G0J9_ARAHY	27.13	2	2	1.72E4	1	1	1	N	57776	Resistance protein (Fragment) OS=Arachis hypogaea OX=3818 PE=2 SV=1
**total 13 proteins**												

tr|Q9FZ11|Q9FZ11_ARAHY
back to list

| Protein Coverage | Supporting Peptides |

Protein Coverage:

1 MIRGRLALSV CFCFLVLGAS SISFRQQPEE NACQFQRLNA QRPDNRLESE GGYIETWNPN NQEFECAGVA LSRLVLRRNA
81 LRRPFYSNAP QEIFIQQGRG YFGLIFPGCP STYEEPAQQG RRHQSQRAPR RFEGEDQSQQ QQQDSHQKVR RFDEGDLIAV
161 PTGVALWMFN DHDTDVVAVS LTDTNNNDNQ LDQFPRRFNL AGNHEQEFLR YQQQSRRRSL PYSPYSPQSQ PRQEEREFSP
241 RGQHSRRERA GQEEENEGGN IFSGFTPEFL AQAFQVDDRQ IVQNLR**GENE SEEEGAIVTV KGGLRILSPD R**KRGADEEEE
321 YDEDEYEYDE EDRRRGRGSR GRGNGIEETI CTASVKKNIG RNRSPDIYNP QAGSLKTAND LNLLILR**WLG LSAEYGNLYR**
401 NALFVPHYNT NAHSIIYALR GRAHVQVVDS NGNRVYDEEL QEGHVLVVPQ NFAVAGKSQS DNFEYVAFKT DSRPNIANFA
481 GENSIIDNLP EEVVANSYGL PREQARQLKN NNPFKFFVPP SQQSLRAVA

d Deamidation (NQ) (+0.98)
d Dimethylation(KR) (+28.03)

Supporting Peptides:

Peptide	Uniq	-10lgP	Mass	Length	ppm	m/z	z	RT	Fraction	Scan	Source File	Area Digest traka B 45-50 kDa	#Feature	#Feature Digest traka B 45-50 kDa	Start	End	PTM
R.WLGLSAEYGNLYR.N	Y	89.78	1540.7673	13	-4.2	771.3923	2	33.62	39	1867	OB4067.raw	1.28E4	1	1	388	400	
R.WLGLSAEYGN(+.98)LYR.N	Y	39.78	1541.7513	13	9.7	771.8950	2	33.53	39	1864	OB4067.raw	0	0	0	388	400	Deamidation (NQ)
R.GENESE(+43.99)EEGAIVTVKGGLRILSPDR(+28.03).K	Y	27.13	2726.3721	25	-9.5	909.7947	3	37.57	39	2118	OB4067.raw	1.42E4	1	1	287	311	Dimethylation(KR)
**total 3 peptides**																	

tr|Q647H4|Q647H4_ARAHY

back to list

| Protein Coverage | Supporting Peptides |

Protein Coverage:

1 MGKLLALSVC FCFLVLGASS ISFRQQPEEN ACQFQRLNAQ RPDNRIESEG GYIETWNPNN QEFECAGVAL SRLVLRRNAL
81 RRPFYSNAPQ EIFIQQGRGY FGLIFPGCPS TYEEPAQQGR RHQSQRPPRR FQGQDQSQQQ QDSHQKVHRF DEGDLIAVPT
161 GVAFWMYNDH DTDVVAVSLT DTNNNDNQLD QFPRRFNLAG NHEQEFLRYQ QQSRRRSLPY SPYSPQTQPK QEDREFSPRG
241 QHGRRERAGQ EQENEGGNIF SGFTPEFLAQ AFQVDDRQIL QNLRGENESD EQGAIVTVRG GLRILSPDRK RRQQYERPDE
321 EEEYDEDEYE YDEEERQHDR RRGRGSRGSG NGIEETICTA SFKKNIGRNR SPDIYNPQAG SLKTANELNL LILR**WLGLSA**
401 **EYGNLYR**NAL FVPHYNTNAH SIIYALRGRA HVQVVDSNGD RVFDEELQEG HVLVVPQNFA VAGKSQSENF EYVAFKTDSR
481 PSIANLAGEN SFIDNLPEEV VANSYGLPRE QARQLKNNNP FKFFVPPSEQ SLRAVA

d Deamidation (NQ) (+0.98)

Supporting Peptides:

Peptide	Uniq	-10lgP	Mass	Length	ppm	m/z	z	RT	Fraction	Scan	Source File	Area Digest traka B 45-50 kDa	#Feature	#Feature Digest traka B 45-50 kDa	Start	End	PTM
R.WLGLSAEYGNLYR.N	Y	89.78	1540.7673	13	-4.2	771.3923	2	33.62	39	1867	OB4067.raw	1.28E4	1	1	395	407	
R.WLGLSAEYGN(+.98)LYR.N	Y	39.78	1541.7513	13	9.7	771.8950	2	33.53	39	1864	OB4067.raw	0	0	0	395	407	Deamidation (NQ)
**total 2 peptides**																	

tr|Q6T2T4|Q6T2T4_ARAHY

back to list

| Protein Coverage | Supporting Peptides |

Protein Coverage:

d Deamidation (NQ) (+0.98)

```
  1 MGKLLALSVC FCFLVLGASS ISFRQQPEEN ACQFQRLNAQ RPDNRIESEG GYIETWNPNN QEFECAGVAL SRLVLRRNAL
 81 RRPFYSNAPQ EIFIQQGRAY FGLIFLGCPS TYEEPAQQGR RHQSQRPPRR FQGQDQSQQQ QDSHQKVHRF DEGDLIAVPT
161 GVAFWMYNDH DTDVVAVSLT DTNNNDNQLD QFPRRFNLAG NHEQEFLRYQ QQSRRRSLPY SPYSPQTQPK QEDREFSPRG
241 QHGRRERAGQ EQENEGGNIF SGFTPEFLAQ AFQVDDRQIL QNLRGENESD EQGAIVTVRG GLRILSPDRK KRQQYERPDE
321 EEEYDEDEYE YDEEERQQDR RRGRGSRGSG NGIEETICTA SFKKNIGRNR SPDIYNPQAG SLKTANELNL LILRWLGLSA
    404
     d
401 EYGNLYRNAL FVPHYNTNAH SIIYALRGRA HVQVVDSNGD RVFDEELQEG HVLVVPQNFA VAGKSQSENF EYVAFKTDSR
481 PSIANLAGEN SFIDNLPEEV VANSYGLPRE QARQLKNNNP FKFFVPPSEQ SLRAVA
```

Supporting Peptides:

Peptide	Uniq	-10lgP	Mass	Length	ppm	m/z	z	RT	Fraction	Scan	Source File	Area Digest traka B 45-50 kDa	#Feature	#Feature Digest traka B 45-50 kDa	Start	End	PTM
R.WLGLSAEYGNLYR.N	Y	89.78	1540.7673	13	-4.2	771.3923	2	33.62	39	1867	OB4067.raw	1.28E4	1	1	395	407	
R.WLGLSAEYGN(+.98)LYR.N	Y	39.78	1541.7513	13	9.7	771.8950	2	33.53	39	1864	OB4067.raw	0	0	0	395	407	Deamidation (NQ)
total 2 peptides																	

tr|Q8LKN1|Q8LKN1_ARAHY

back to list

| Protein Coverage | Supporting Peptides |

Protein Coverage:

d Deamidation (NQ) (+0.98)

```
  1 MGKLLALSVC FCFLVLGASS ISFRQQPEEN ACQFQRLNAQ RPDNRIESEG GYIETWNPNN QEFECAGVAL SRLVLRRNAL
 81 RRPFYSNAPQ EIFIQQGRGY FGLIFPGCPS TYEEPAQQGR RHQSQRPPRR FQGQDQSQQQ QDSHQKVHRF DEGDLIAVPT
161 GVAFWMYNDH DTDVVAVSLT DTNNNDNQLD QFPRRFNLAG NHEQEFLRYQ QQSRRRSLPY SPYSPQTQPK QEDREFSPRG
241 QHGRRERAGQ EQENEGGNIF SGFTPEFLAQ AFQVDDRQIL QNLRGENESD EQGAIVTVRG GLRILSPDRK RRQQYERPDE
321 EEEYDEDEYE YDEEERQQDR RRGRGSRGSG NGIEETICTA SFKKNIGRNR SPDIYNPQAG SLKTANELQL NLLILRWLGL
      406
       d
401 SAEYGNLYRN ALFVPHYNTN AHSIIYALRG RAHVQVVDSN GDRVFDEELQ EGHVLVVPQN FAVAGKSQSE NFEYVAFKTD
481 SRPSIANLAG ENSFIDNLPE EVVANSYGLP REQARQLKNN NPFKFFVPPS EQSLRAVA
```

Supporting Peptides:

Peptide	Uniq	-10lgP	Mass	Length	ppm	m/z	z	RT	Fraction	Scan	Source File	Area Digest traka B 45-50 kDa	#Feature	#Feature Digest traka B 45-50 kDa	Start	End	PTM
R.WLGLSAEYGNLYR.N	Y	89.78	1540.7673	13	-4.2	771.3923	2	33.62	39	1867	OB4067.raw	1.28E4	1	1	397	409	
R.WLGLSAEYGN(+.98)LYR.N	Y	39.78	1541.7513	13	9.7	771.8950	2	33.53	39	1864	OB4067.raw	0	0	0	397	409	Deamidation (NQ)
total 2 peptides																	

tr|Q647H3|Q647H3_ARAHY

back to list

| Protein Coverage | Supporting Peptides |

Protein Coverage:

1 MAKLLALSVC FCFLVLGASS ISFRQQPEEN ACQFQRLNAQ RPDNRLESEG GYIETWNPNN QEFECAGVAL SRLVLRRNAL

d Deamidation (NQ) (+0.98)

81 RRPFYSNAPQ EIFIQQGRGY FGLIFPGCPS TYEEPAQQGR RHQSQRAPRR FEGEDQSQQQ QQDSHQKVRR FDEGDLIAVP
161 TGVALWMYND HDTDVVAVSL TDTNNNDNQL DQFPRRFNLA GNHEQEFLRY QQQSRRRSLP YSPYSPQSQP RQEEREFSPR
241 GQHSRRERAG QEQENEGGNI FSGFTPEFLA QAFQVDDRQI LQNLRGENES DEQGAIVTVR GGLRILSPDR KRRQQYERPD
321 EEEEYDEDEY EYDEEERQQD RRRGRGSRGR GNGIEETICT ASVKKNIGRN RSPDIYNPQA GSLKTANDLN LLILR**WLGLS**

405 d

401 **AEYGNLYR**NA LFVPHYNTNA HSIIYALRGR AHVQVVDSNG NRVYDEELQE GHVLVVPQNF AVAGKSQSDN FEYVAFKTDS
481 RPSIANLAGE NSIIDNLPEE VVANSYGLPR EQARQLKNNN PFKFFVPPSQ QSLGAVA

Supporting Peptides:

Peptide	Uniq	-10lgP	Mass	Length	ppm	m/z	z	RT	Fraction	Scan	Source File	Area Digest traka B 45-50 kDa	#Feature	#Feature Digest traka B 45-50 kDa	Start	End	PTM
R.WLGLSAEYGNLYR.N	Y	89.78	1540.7673	13	-4.2	771.3923	2	33.62	39	1867	OB4067.raw	1.28E4	1	1	396	408	
R.WLGLSAEYGN(+.98)LYR.N	Y	39.78	1541.7513	13	9.7	771.8950	2	33.53	39	1864	OB4067.raw	0	0	0	396	408	Deamidation (NQ)
**total 2 peptides**																	

tr|A1DZF0|A1DZF0_ARAHY

back to list

| Protein Coverage | Supporting Peptides |

Protein Coverage:

1 MAKLLELSFC FCFLVLGASS ISFRQQPEEN ACQFQRLNAQ RPDNRIESEG GYIETWNPNN QEFECAGVAL SRLVLRRNAL

d Deamidation (NQ) (+0.98)

81 RRPFYSNAPQ EIFIQQGRGY FGLIFPGCPS TYEEPAQQGR RYQSQRPPRR LQEEDQSQQQ QDSHQKVHRF NEGDLIAVPT
161 GVAFWLYNDH DTDVVAVSLT DTNNNDNQLD QFPRRFNLAG NHEQEFLRYQ QQSRQSRRRS LPLSPYSPQP GQEDREFSPQ
241 GQHGRRERAG QEQENEGGNI FSGFTSEFLA QAFQVDDRQI VQNLRGENES EEQGAIVTVK GGLRILSPDR KSPDEEEEYD

397 d

321 EDEYAEEERQ QDRRRGRGSR GSGNGIEETI CTATVKKNIG RNRSPDIYNP QAGSLKTANE LNLLILR**WLG LSAEYGNLYR**
401 NALFVPHYNT NAHSIIYALR GRAHVQVVDS NGNRVYDEEL QEGHVLVVPQ NFAVAGKSQS ENFEYVAFKT DSRPSIANLA
481 GENSFIDNLP EEVVANSYGL PREQARQLKN NNPFKFFVPP FQQSPRAVA

Supporting Peptides:

Peptide	Uniq	-10lgP	Mass	Length	ppm	m/z	z	RT	Fraction	Scan	Source File	Area Digest traka B 45-50 kDa	#Feature	#Feature Digest traka B 45-50 kDa	Start	End	PTM
R.WLGLSAEYGNLYR.N	Y	89.78	1540.7673	13	-4.2	771.3923	2	33.62	39	1867	OB4067.raw	1.28E4	1	1	388	400	
R.WLGLSAEYGN(+.98)LYR.N	Y	39.78	1541.7513	13	9.7	771.8950	2	33.53	39	1864	OB4067.raw	0	0	0	388	400	Deamidation (NQ)
**total 2 peptides**																	

tr|B5TYU1|B5TYU1_ARAHY

back to list

| Protein Coverage | Supporting Peptides |

Protein Coverage:

1 MAKLLELSFC FCFLVLGASS ISFRQQPEEN ACQFQRLNAQ RPDNRIESEG GYIETWNPNN QEFECAGVAL SRLVLRRNAL

d Deamidation (NQ) (+0.98)

81 RRPFYSNAPQ EIFIQQGRGY FGLIFPGCPS TYEEPAQQGR RYQSQRPPRR LQEEDQSQQQ QDSHQKVHRF NEGDLIAVPT

161 GVAFWLYNDH DTDVVAVSLT DTNNNDNQLD QFPRRFNLAG NHEQEFLRYQ QQSRQSRRRS LPYSPYSPQS QPRQEEREFS

241 PRGQHSRRER AGQEEENEGG NIFSGFTPEF LAQAFQVDDR QIVQNLRGEN ESEEQGAIVT VRGGLRILSP DRKRGADEEE

398 d

321 EYDEDEYEYD EEDRRRGRGS RGSGNGIEET ICTATVKKNI GRNRSPDIYN PQAGSLKTAN ELNLLILR**WL GLSAEYGNLY**

401 **R**NALFVPHYN TNAHSIIYAL RGRAHVQVVD SNGNRVYDEE LQEGHVLVVP QNFAVAGKSQ SDNFEYVAFK TDSRPSIANL

481 AGENSVIDNL PEEVVANSYG LPREQARQLK NNNPFKFFVP PSQQSPRAVA

Supporting Peptides:

Peptide	Uniq	-10lgP	Mass	Length	ppm	m/z	z	RT	Fraction	Scan	Source File	Area Digest traka B 45-50 kDa	#Feature	#Feature Digest traka B 45-50 kDa	Start	End	PTM
R.WLGLSAEYGNLYR.N	Y	89.78	1540.7673	13	-4.2	771.3923	2	33.62	39	1867	OB4067.raw	1.28E4	1	1	389	401	
R.WLGLSAEYGN(+.98)LYR.N	Y	39.78	1541.7513	13	9.7	771.8950	2	33.53	39	1864	OB4067.raw	0	0	0	389	401	Deamidation (NQ)
**total 2 peptides**																	

tr|Q5I6T2|Q5I6T2_ARAHY

back to list

| Protein Coverage | Supporting Peptides |

Protein Coverage:

1 MAKLLELSFC FCFLVLGASS ISFRQQPEEN ACQFQRLNAQ RPDNRIESEG GYIETWNPNN QEFECAGVAL SRLVLRRNAL

d Deamidation (NQ) (+0.98)

81 RRPFYSNAPQ EIFIQQGRGY FGLIFPGCPS TYEEPAQQGR RSQSQRPPRR LQGEDQSQQQ QDSHQKVHRF DEGDLIAVPT

161 GVAFWLYNDH DTDVVAVSLT DTNNNDNQLD QFPRRFNLAG NHEQEFLRYQ QQSRQSRRRS LPYSPYSPQS QPRQEEREFS

241 PRGQHSRRER AGQEEENEGG NIFSGFTPEF LEQAFQVDDR QIVQNLRGEN ESEEEGAIVT VRGGLRILSP DRKRGADEEE

398 d

321 EYDEDEYEYD EEDRRRGRGS RGRGNGIEET ICTASVKKNI GRNRSPDIYN PQAGSLKTAN DLNLLILR**WL GLSAEYGNLY**

401 **R**NALFVPHYN TNAHSIIYAL RGRAHVQVVD SNGNRVYDEE LQEGHVLVVP QNFAVAGKSQ SDNFEYVAFK TDSRPSIANL

481 AGENSVIDNL PEEVVANSYG LQREQARQQL KNNNPFKFFV PPSQQSPRAV A

Supporting Peptides:

Peptide	Uniq	-10lgP	Mass	Length	ppm	m/z	z	RT	Fraction	Scan	Source File	Area Digest traka B 45-50 kDa	#Feature	#Feature Digest traka B 45-50 kDa	Start	End	PTM
R.WLGLSAEYGNLYR.N	Y	89.78	1540.7673	13	-4.2	771.3923	2	33.62	39	1867	OB4067.raw	1.28E4	1	1	389	401	
R.WLGLSAEYGN(+.98)LYR.N	Y	39.78	1541.7513	13	9.7	771.8950	2	33.53	39	1864	OB4067.raw	0	0	0	389	401	Deamidation (NQ)
**total 2 peptides**																	

tr|Q9SQH7|Q9SQH7_ARAHY

back to list

| Protein Coverage | Supporting Peptides |

Protein Coverage:

1 MAKLLELSFC FCFLVLGASS ISFRQQPEEN ACQFQRLNAQ RPDNRIESEG GYIETWNPNN QEFECAGVAL SRLVLRRNAL

d Deamidation (NQ) (+0.98)

81 RRPFYSNAPQ EIFIQQGRGY FGLIFPGCPS TYEEPAQQGR RYQSQRPPRR LQEEDQSQQQ QDSHQKVHRF NEGDLIAVPT

161 GVAFWLYNDH DTDVVAVSLT DTNNNDNQLD QFPRRFNLAG NHEQEFLRYQ QQSRQSRRRS LPYSPYSPHS RPRREEREFR

241 PRGQHSRRER AGQEEEDEGG NIFSGFTPEF LEQAFQVDDR QIVQNLWGEN ESEEEGAIVT VRGGLRILSP DGTRGADEEE

398 d

321 EYDEDQYEYH EQDGRRGRGS RGGGNGIEET ICTACVKKNI GGNRSPHIYD PQRWFTQNCH DLNLLILR**WL GLSAEYGNLY**

d

401 **R**NALFVPHYN TNAHSIIYAL RGRAHVQVVD SNGNRVYDEE LQEGHVLVVP QNFAVAGKSQ SENFEYVAFK TDSRPSIANF

481 AGENSFIDNL PEEVVANSYG LPREQARQLK NNNPFKFFVP PFQQSPRAVA

Supporting Peptides:

Peptide	Uniq	-10lgP	Mass	Length	ppm	m/z	z	RT	Fraction	Scan	Source File	Area Digest traka B 45-50 kDa	#Feature	#Feature Digest traka B 45-50 kDa	Start	End	PTM
R.WLGLSAEYGNLYR.N	Y	89.78	1540.7673	13	-4.2	771.3923	2	33.62	39	1867	OB4067.raw	1.28E4	1	1	389	401	
R.WLGLSAEYGN(+.98)LYR.N	Y	39.78	1541.7513	13	9.7	771.8950	2	33.53	39	1864	OB4067.raw	0	0	0	389	401	Deamidation (NQ)
**total 2 peptides**																	

tr|Q6IWG5|Q6IWG5_ARAHY

back to list

| Protein Coverage | Supporting Peptides |

Protein Coverage:

1 KLLALSLCFC VLVLGASSVT FRQGGEENEC QFQRLNAQRP DNRIESEGGY IETWNPNNQE FQCAGVALSR TVLRRNALRR

d Deamidation (NQ) (+0.98)

81 PFYSNAPLEI YVQQGSGYFG LIFPGCPSTY EEPAQEGRRY QSQKPSRRFQ VGQDDPSQQQ QDSHQKVHRF DEGDLIAVPT

161 GVAFWMYNDE DTDVVTVTLS DTSSIHNQLD QFPRRFYLAG NQEQEFLRYQ QQQGSRPHYR QISPRVRGDE QENEGSNIFS

241 GFAQEFLQHA FQVDRQTVEN LRGENEREEQ GAIVTVKGGL RILSPDEEDE SSRSPPSRRE EFDEDRSRPQ QRGKYDENRR

359 d 374 d

321 GYKNGIEETI CSASVKKNLG RSSNPDIYNP QAGSLR**SVNE LDLPILGWLG LSAQHGTIYR** NAMFVPHYTL NAHTIVVALN

d

d d

401 GRAHVQVVDS NGNRVYDEEL QEGHVLVVPQ NFAVAAKAQS ENYEYLAFKT DSRPSIANLA GENSIIDNLP EEVVANSYRL

481 PREQARQLKN NNPFKFFVPP FDHQSMREVA

Supporting Peptides:

Peptide	Uniq	-10lgP	Mass	Length	ppm	m/z	z	RT	Fraction	Scan	Source File	Area Digest traka B 45-50 kDa	#Feature	#Feature Digest traka B 45-50 kDa	Start	End	PTM
R.SVN(+.98)ELDLPILGWLGLSAQ(+.98)HGTIYR.N	Y	43.22	2653.3750	24	-3.3	885.4680	3	39.12	39	2218	OB4067.raw	0	0	0	357	380	Deamidation (NQ)
R.SVNELDLPILGWLGLSAQ(+.98)HGTIYR.N	Y	39.91	2652.3911	24	-4.8	885.1387	3	38.76	39	2194	OB4067.raw	4.61E4	1	1	357	380	Deamidation (NQ)
R.SVNELDLPILGWLGLSAQHGTIYR.N	Y	38.53	2651.4070	24	-5.3	884.8102	3	38.53	39	2169	OB4067.raw	5.14E4	1	1	357	380	
**total 3 peptides**																	

tr|E5G077|E5G077_ARAHY

back to list

| Protein Coverage | Supporting Peptides |

Protein Coverage:

1 MAKLLALSLC FCVLVLGASS VTFRQGGEEN ECQFQRLNAQ RPDNRIESEG GYIETWNPNN QEFQCAGVAL SRTVLRRNAL

d Deamidation (NQ) (+0.98)

81 RRPFYSNAPL EIYVQQGSGY FGLIFPGCPS TYEEPAQEGR RYQSQKPSRR FQVGQDDPSQ QQQDSHQKVH RFDEGDLIAV

161 PTGVAFWMYN DEDTDVVTVT LSDTSSIHNQ LDQFPRRFYL AGNQEQEFLR YQQQQGSRPH YRQISPRVRG DEQENEGSNI

241 FSGFAQEFLQ HAFQVDRQTV ENLRGENERE EQGAIVTVKG GLRILSPDEE DESSRSPPNR REEFDEDRSR PQQRGKYDEN

361 d, 376 d

321 RRGYKNGIEE TICSASVKKN LGRSSNPDIY NPQAGSLR**SV NELDLPILGW LGLSAQHGTI YR**NAMFVPHY TLNAHTIVVA

401 LNGRAHVQVV DSNGNRVYDE ELQEGHVLVV PQNFAVAAKA QSENYEYLAF KTDSRPSIAN QAGENSIIDN LPEEVVANSY

481 RLPREQARQL KNNNPFKFFV PPFDHQSMRE VA

Supporting Peptides:

Peptide	Uniq	-10lgP	Mass	Length	ppm	m/z	z	RT	Fraction	Scan	Source File	Area Digest traka B 45-50 kDa	#Feature	#Feature Digest traka B 45-50 kDa	Start	End	PTM
R.SVN(+.98)ELDLPILGWLGLSAQ(+.98)HGTIYR.N	Y	43.22	2653.3750	24	-3.3	885.4680	3	39.12	39	2218	OB4067.raw	0	0	0	359	382	Deamidation (NQ)
R.SVNELDLPILGWLGLSAQ(+.98)HGTIYR.N	Y	39.91	2652.3911	24	-4.8	885.1387	3	38.76	39	2194	OB4067.raw	4.61E4	1	1	359	382	Deamidation (NQ)
R.SVNELDLPILGWLGLSAQHGTIYR.N	Y	38.53	2651.4070	24	-5.3	884.8102	3	38.53	39	2169	OB4067.raw	5.14E4	1	1	359	382	
**total 3 peptides**																	

tr|Q0GM57|Q0GM57_ARAHY

back to list

| Protein Coverage | Supporting Peptides |

Protein Coverage:

1 MAKLLALSLC FCVLVLGASS VTFRQGGEEN ECQFQRLNAQ RPDNRIESEG GYIETWNPNN QEFQCAGVAL SRTVLRRNAL

d Deamidation (NQ) (+0.98)

81 RRPFYSNAPL EIYVQQGSGY FGLIFPGCPS TYEEPAQEGR RYQSQKPSRR FQVGQDDPSQ QQQDSHQKVH RFDEGDLIAV

161 PTGVAFWMYN DEDTDVVTVT LSDTSSIHNQ LDQFPRRFYL AGNQEQEFLR YQQQQGSRPH YRQISPRVRG DEQENEGSNI

241 FSGFAQEFLQ HAFQVDRQTV ENLRGENERE EQGAIVTVKG GLRILSPDEE DESSRSPPSR REEFDEDRSR PQQRGKYDEN

361 d, 376 d

321 RRGYKNGIEE TICSASVKKN LGRSSNPDIY NPQAGSLR**SV NELDLPILGW LGLSAQHGTI YR**NAMFVPHY TLNAHTIVVA

401 LNGRAHVQVV DSNGNRVYDE ELQEGHVLVV PQNFAVAAKA QSENYEYLAF KTDSRPSIAN LAGENSIIDN LPEEVVANSY

481 RLPREQARQL KNNNPFKFFV PPFDHQSMRE VA

Supporting Peptides:

Peptide	Uniq	-10lgP	Mass	Length	ppm	m/z	z	RT	Fraction	Scan	Source File	Area Digest traka B 45-50 kDa	#Feature	#Feature Digest traka B 45-50 kDa	Start	End	PTM
R.SVN(+.98)ELDLPILGWLGLSAQ(+.98)HGTIYR.N	Y	43.22	2653.3750	24	-3.3	885.4680	3	39.12	39	2218	OB4067.raw	0	0	0	359	382	Deamidation (NQ)
R.SVNELDLPILGWLGLSAQ(+.98)HGTIYR.N	Y	39.91	2652.3911	24	-4.8	885.1387	3	38.76	39	2194	OB4067.raw	4.61E4	1	1	359	382	Deamidation (NQ)
R.SVNELDLPILGWLGLSAQHGTIYR.N	Y	38.53	2651.4070	24	-5.3	884.8102	3	38.53	39	2169	OB4067.raw	5.14E4	1	1	359	382	
**total 3 peptides**																	

tr|A0A290G0J9|A0A290G0J9_ARAHY

back to list

| Protein Coverage | Supporting Peptides |

Protein Coverage:

1 RLTGEIPASI GRLSKLAILK LSNNSLSGRI PPELGDCHSL IWLDLNTNSL TGSVPAELFK QSGKIAVNFI SGKTFVYIKN
81 DGSKMFHGAG SLLEFAGIRQ EQLNRISSRN SWNVTRVYQG SLGKLLPPTF NNNGSMIFLD VSHNMLSGSI PKEIGSMNYL
161 YILYMGHNNI SGNIPQDLGM LKNLGILDLS SNRLEGRIPE SLAGLSFLTE IDLSNNFLSG LVPSSGQFYT FPASRFLNNS
241 GLCGVPLAPC KDDSASGANG QRHKSHRRQV TLVGNVAMGL LSSLFCLFVL VIIAIETRKR RKKKEPALDF YVDSRSHSGT
321 ANAGWKMITA REALSINLET FEQRPLRKLT FADLLEATNG FHDDSIIGSG GFGVVYK**AQL KDGSVVAIK**K LIHISGQGDR
401 EFTAEMETIG RIKHKNLVPL LGYCKVGEER LLVYEYMKYG SLDILHDQKK AGIKLNWAAR KKIAVGAARG LAFLHHTCNP
481 NIIHRDMKSS NVLLDENLEA RVADFGMARL MSNVDTHLSV STLAGTPG

Supporting Peptides:

Peptide	Uniq	-10lgP	Mass	Length	ppm	m/z	z	RT	Fraction	Scan	Source File	Area Digest traka B 45-50 kDa	#Feature	#Feature Digest traka B 45-50 kDa	Start	End	PTM
K.AQLKDGSVVAIK.K	Y	27.13	1227.7186	12	6.7	614.8744	2	53.64	39	3057	OB4067.raw	1.72E4	1	1	378	389	
**total 1 peptides**																	

Peptide List

Prepared with PEAKS ™ (bioinfor.com)

1. Notes Gastric Digest Raw peanut Band #13 55-60 kDa (PTM504)

2. Result Statistics

Figure 1. False discovery rate (FDR) curve. X axis is the number of peptide-spectrum matches (PSM) being kept. Y axis is the corresponding FDR.

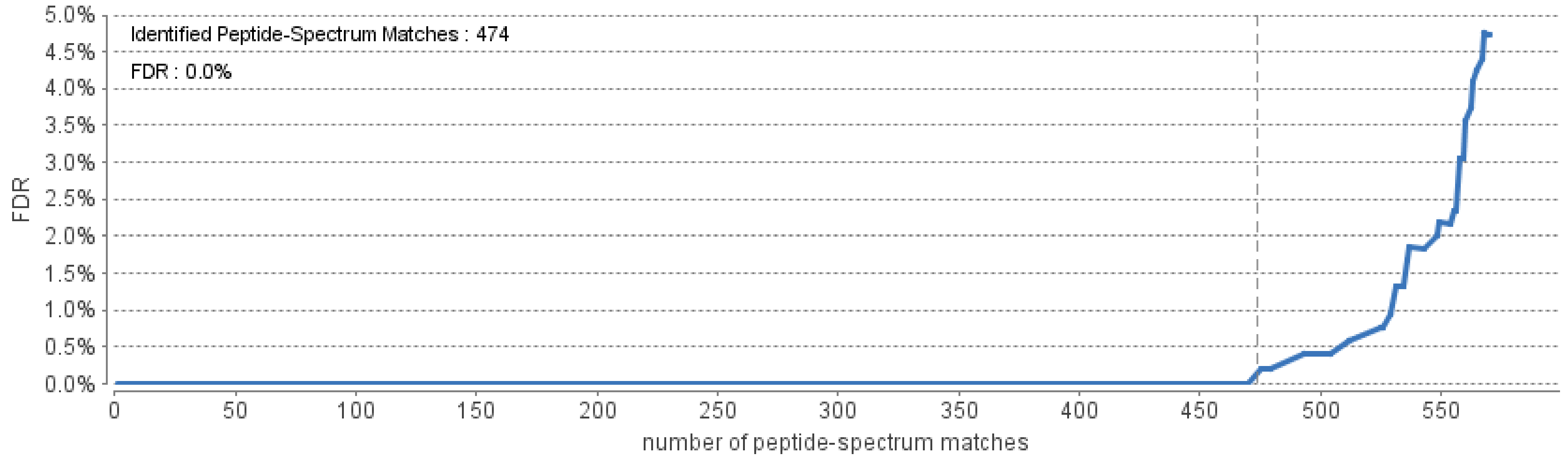

Figure 2. PSM score distribution. **(a)** Distribution of PEAKS peptide score; **(b)** Scatterplot of PEAKS peptide score versus precursor mass error.

(a) **(b)**

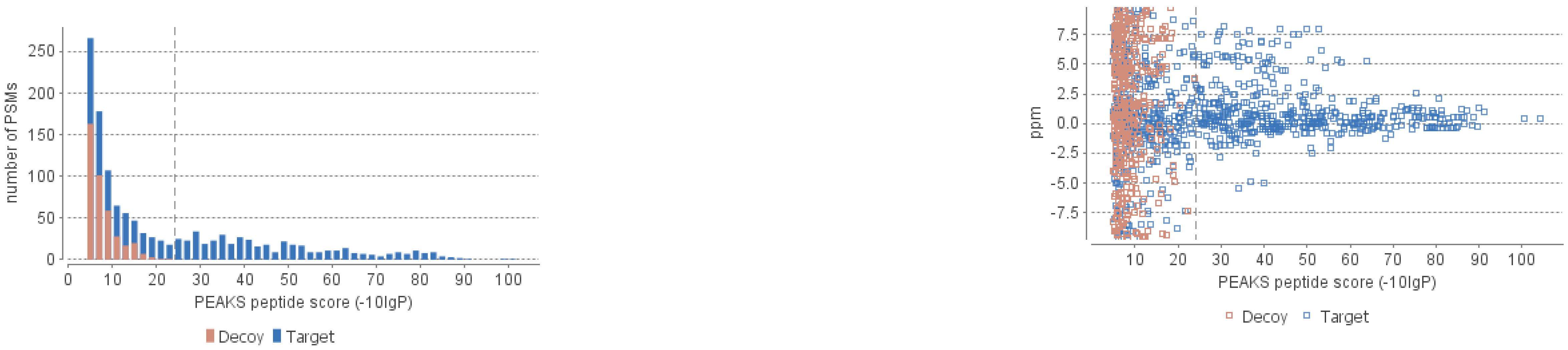

Figure 3. Distribution of peptide feature detection. **(a)** Feature m/z distribution; **(b)** Feature RT distribution

(a)

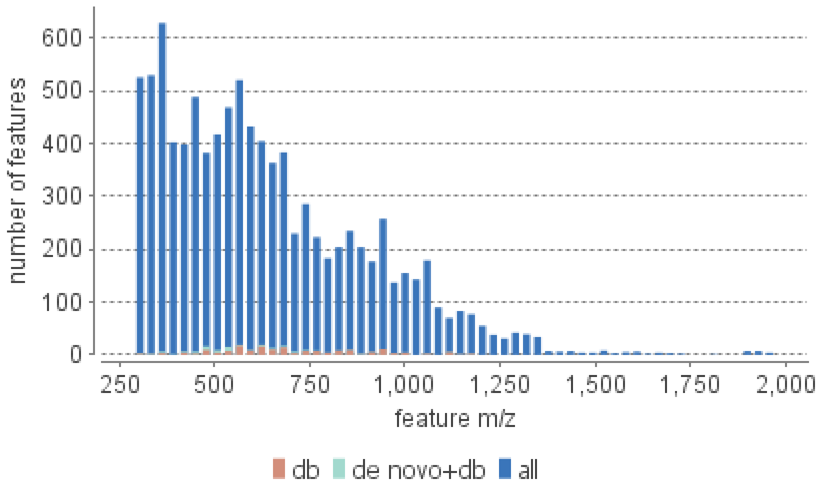

(b)

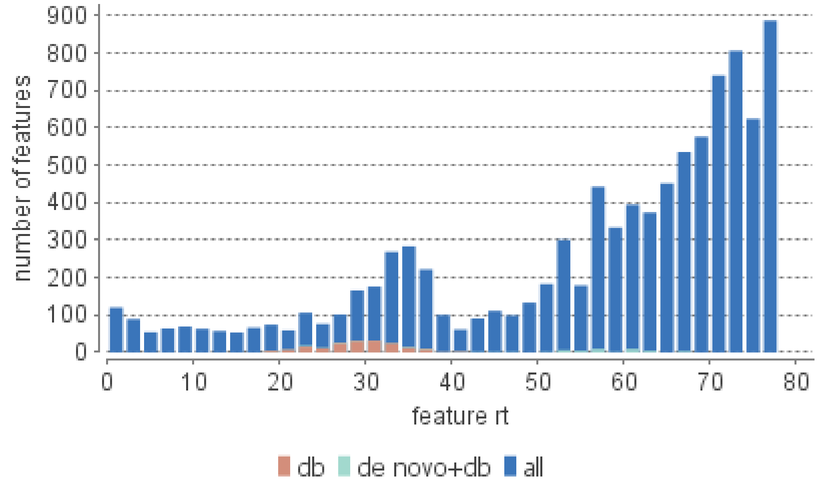

Figure 4. Distribution of identified peptide features. **(a)** Feature abundance distribution **(b)** *De novo* sequencing validation.

(a)

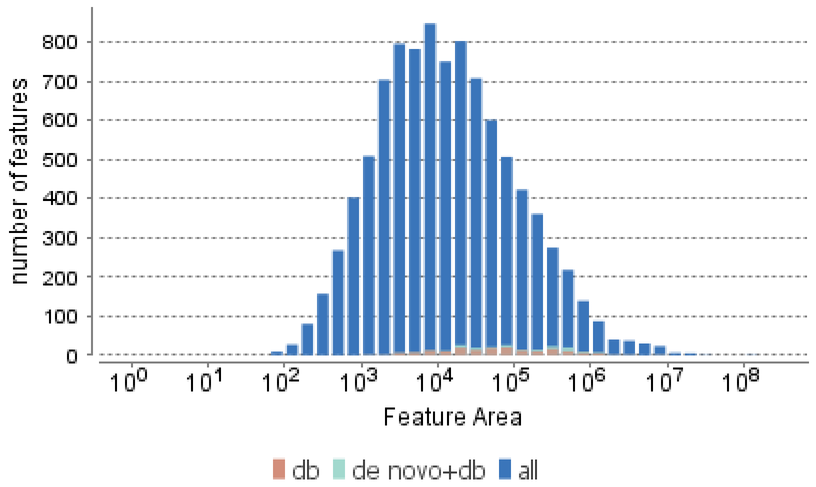

(b)

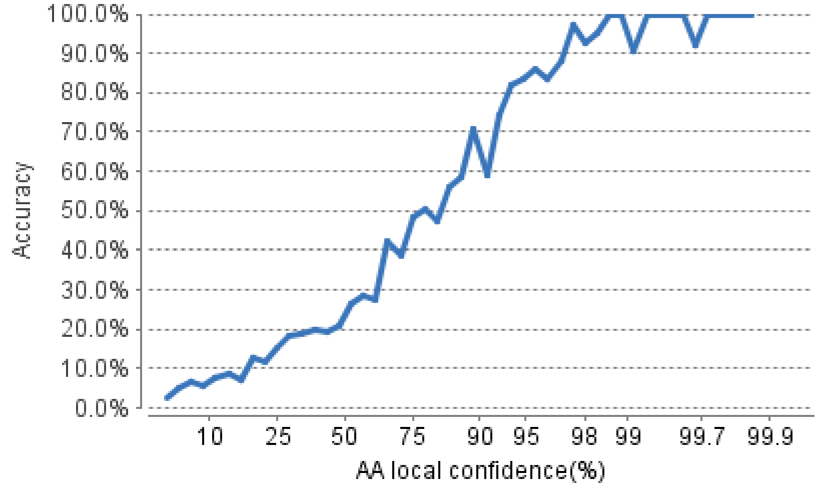

Table 1. Statistics of data.

# of MS scans	1874
# of MS/MS scans	2919
# of Features	9587
# of Chimera scans	362

Table 2. Result filtration parameters.

Peptide -10lgP	≥24.1

Table 4. PTM profile.

Name	ΔMass	Position	#PSM	-10lgP	Abundance	AScore
Deamidation	.98	NQ	288	89.93	2.62E5	1000.00
Oxidation	15.99	M	61	91.29	6.36E4	1000.00
Pyro-glu from Q	-17.03	N-term	4	45.70	4.01E3	1000.00
Carbamidomethyl	57.02	C	3	32.90		1000.00

PTM Ascore	≥20
Protein -10lgP	≥20
Proteins unique peptides	≥1
De novo ALC Score	≥50%

Table 3. Statistics of filtered result.

Peptide-Spectrum Matches	474
Peptide sequences	219
Protein groups	3
Proteins	14
Proteins (#Unique Peptides)	5 (>2); 0 (=2); 9 (=1);
FDR (Peptide-Spectrum Matches)	0.0%
FDR (Peptide Sequences)	0.0%
FDR (Protein Group)	0.0%
De Novo Only Spectra	80
# of identified Features	220
# of identified MS/MS scans	442

Ethyl	28.03	E,N-term	3	51.23	5.41E3	27.19
Methylation(others)	14.02	ES	2	55.55	8.52E3	30.46
Pyro-glu from E	-18.01	N-term	2	36.72	3.58E3	1000.00
Carbamylation	43.01	N-term	1	44.07	9.55E3	1000.00
HydPro	15.99	P	1	29.84		8.26
Sodium	21.98	E	1	31.12	1.34E3	0.00

3. Experiment Control

Figure 5. Precursor mass error of peptide-spectrum matches (PSM) in filtered result. **(a)** Distribution of precursor mass error in ppm; **(b)** Scatterplot of precursor m/z versus precursor mass error in ppm.

(a)

(b)

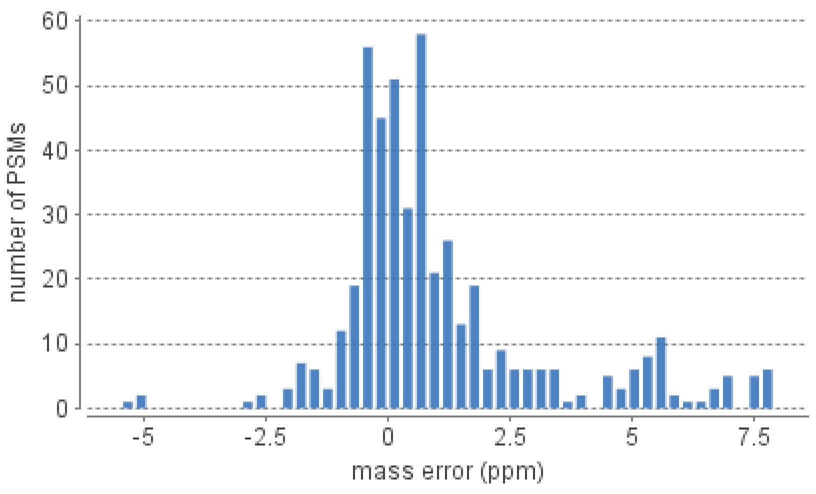

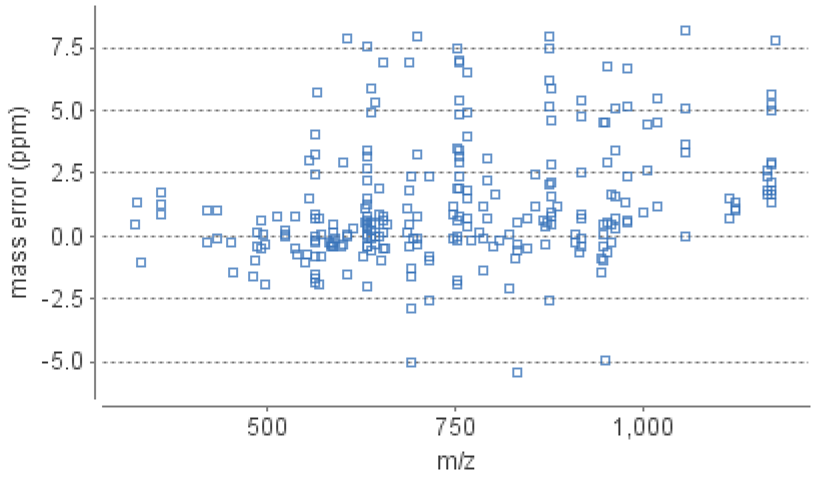

Table 5. Number of identified peptides in each sample by the number of missed cleavages

Missed Cleavages	0	1	2	3	4+
Digest 13 traka 60 kDa	80	102	37	0	0

4. Other Information

Table 6. Search parameters.

Search Engine Name: PEAKS
Parent Mass Error Tolerance: 10.0 ppm
Fragment Mass Error Tolerance: 0.5 Da
Precursor Mass Search Type: monoisotopic
Enzyme: Trypsin
Max Missed Cleavages: 2
Digest Mode: Unspecific
Fixed Modifications:
Carbamidomethylation: 57.02
Variable Modifications:
Deamidation (NQ): 0.98
Oxidation (M): 15.99
Hydroxylation Pro: 15.99
Acetylation (K): 42.01
Acetylation (Protein N-term): 42.01
Acetylation (N-term): 42.01
Amidation: -0.98
Beta-methylthiolation: 45.99
and 305 more...
Max Variable PTM Per Peptide: 5

Table 7. Instrument parameters.

Fractions: OB4063.raw
Ion Source: ESI(nano-spray)
Fragmentation Mode: CID, CAD(y and b ions)
MS Scan Mode: FT-ICR/Orbitrap
MS/MS Scan Mode: Linear Ion Trap

Database: Uniprot_Peanut-3818_Jul18
Taxon: All
Contaminant Database: contaminantsMQ_mar19
Searched Entry: 1723
FDR Estimation: Enabled
De novo score (ALC%) threshold: 15
Peptide hit threshold (-10logP): 30.0
Peaks run ID: 498
Merge Options: no merge
Precursor Options: corrected
Charge Options: no correction
Filter Charge: 2 - 8
Process: true
Associate chimera: yes

Protein List

Protein Accession Contains:
Protein Description Contains:
Peptide Sample Area >=
Protein Ptm Contains:

Protein Group	Protein ID	Accession	-10lgP	Coverage (%)	Coverage (%) Digest 13 traka 60 kDa	Area Digest 13 traka 60 kDa	#Peptides	#Unique	#Spec Digest 13 traka 60 kDa	PTM	Avg. Mass	Description
**1**	20853	tr|N1NG13|N1NG13_ARAHY	367.01	54	54	9.5E6	56	15	423	Y	71345	Seed storage protein Ara h1 OS=Arachis hypogaea OX=3818 GN=ARAX_AHF417E07-017 PE=4 SV=1
**1**	20854	P43238|ALL12_ARAHY	367.01	54	54	9.5E6	56	15	423	Y	71345	Allergen Ara h 1, clone P41B OS=Arachis hypogaea OX=3818 PE=1 SV=1
**2**	20849	tr|Q6PSU3|Q6PSU3_ARAHY	354.63	56	56	3.88E6	50	9	379	Y	66575	Conarachin (Fragment) OS=Arachis hypogaea OX=3818 PE=4 SV=1
**2**	20850	P43237|ALL11_ARAHY	354.63	53	53	3.88E6	50	9	379	Y	70283	Allergen Ara h 1, clone P17 OS=Arachis hypogaea OX=3818 PE=1 SV=1
**2**	20851	tr|B3IXL2|B3IXL2_ARAHY	354.63	53	53	3.88E6	50	9	379	Y	70283	Main allergen Ara h1 OS=Arachis hypogaea OX=3818 PE=2 SV=1
**5**	20577	tr|Q9SQH7|Q9SQH7_ARAHY	88.43	2	2	1.85E4	1	1	1	N	61011	Glycinin OS=Arachis hypogaea OX=3818 GN=Arah4 PE=2 SV=1
**5**	20581	tr|Q647H3|Q647H3_ARAHY	88.43	2	2	1.85E4	1	1	1	N	61532	Arachin Ahy-2 OS=Arachis hypogaea OX=3818 PE=2 SV=1
**5**	20580	tr|Q647H4|Q647H4_ARAHY	88.43	2	2	1.85E4	1	1	1	N	61506	Arachin Ahy-1 OS=Arachis hypogaea OX=3818 PE=2 SV=1
**5**	20582	tr|Q8LKN1|Q8LKN1_ARAHY	88.43	2	2	1.85E4	1	1	1	N	61738	Allergen Arah3/Arah4 OS=Arachis hypogaea OX=3818 PE=3 SV=1
**5**	20576	tr|Q9FZ11|Q9FZ11_ARAHY	88.43	2	2	1.85E4	1	1	1	N	60449	Gly1 OS=Arachis hypogaea OX=3818 GN=Gly1 PE=2 SV=1
**5**	20579	tr|Q5I6T2|Q5I6T2_ARAHY	88.43	2	2	1.85E4	1	1	1	N	60736	Arachin Ahy-4 OS=Arachis hypogaea OX=3818 PE=2 SV=1
**5**	20591	tr|Q6T2T4|Q6T2T4_ARAHY	88.43	2	2	1.85E4	1	1	1	N	61499	Storage protein OS=Arachis hypogaea OX=3818 PE=2 SV=1
**5**	20574	tr|A1DZF0|A1DZF0_ARAHY	88.43	2	2	1.85E4	1	1	1	N	60375	Arachin 6 OS=Arachis hypogaea OX=3818 PE=2 SV=1
**5**	20578	tr|B5TYU1|B5TYU1_ARAHY	88.43	2	2	1.85E4	1	1	1	N	60624	Arachin Arah3 isoform OS=Arachis hypogaea OX=3818 PE=1 SV=1
**total 14 proteins**												

tr|N1NG13|N1NG13_ARAHY
back to list

| Protein Coverage | Supporting Peptides |

Protein Coverage:

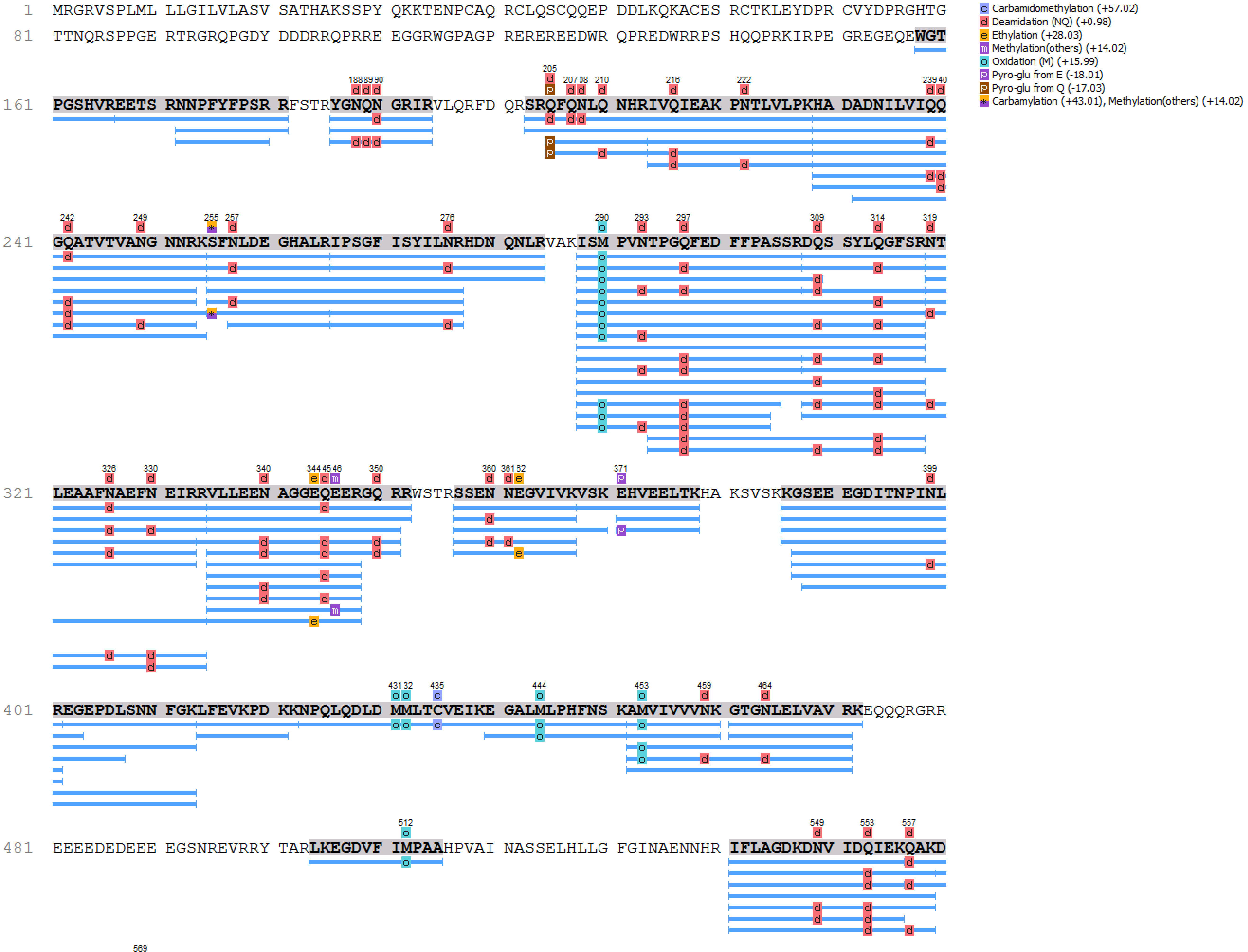
1 MRGRVSPLML LLGILVLASV SATHAKSSPY QKKTENPCAQ RCLQSCQQEP DDLKQKACES RCTKLEYDPR CVYDPRGHTG
81 TTNQRSPPGE RTRGRQPGDY DDDRRQPRRE EGGRWGPAGP REREREEDWR QPREDWRRPS HQQPRKIRPE GREGEQEWGT
161 PGSHVREETS RNNPFYFPSR RFSTRYGNQN GRIRVLQRFD QRSRQFQNLQ NHRIVQIEAK PNTLVLPKHA DADNILVIQQ
241 GQATVTVANG NNRKSFNLDE GHALRIPSGF ISYILNRHDN QNLRVAKISM PVNTPGQFED FFPASSRDQS SYLQGFSRNT
321 LEAAFNAEFN EIRRVLLEEN AGGEQEERGQ RRWSTRSSEN NEGVIVKVSK EHVEELTKHA KSVSKKGSEE EGDITNPINL
401 REGEPDLSNN FGKLFEVKPD KKNPQLQDLD MMLTCVEIKE GALMLPHFNS KAMVIVVVNK GTGNLELVAV RKEQQQRGRR
481 EEEEDEDEEE EGSNREVRRY TARLKEGDVF IMPAAHPVAI NASSELHLLG FGINAENNHR IFLAGDKDNV IDQIEKQAKD
Carbamidomethylation (+57.02)
Deamidation (NQ) (+0.98)
Ethylation (+28.03)
Methylation(others) (+14.02)
Oxidation (M) (+15.99)
Pyro-glu from E (-18.01)
Pyro-glu from Q (-17.03)
Carbamylation (+43.01), Methylation(others) (+14.02)

Supporting Peptides:

Peptide	Uniq	-10lgP	Mass	Length	ppm	m/z	z	RT	Fraction	Scan	Source File	Area Digest 13 traka 60 kDa	#Feature	#Feature Digest 13 traka 60 kDa	Start	End	PTM
R.NTLEAAFNAEFNEIR.R	N	104.37	1737.8322	15	0.4	869.9237	2	34.24	35	1984	OB4063.raw	2.88E5	1	1	319	333	
K.ISM(+15.99)PVNTPGQFEDFFPASSR.D	N	91.29	2242.0364	20	1.0	1122.0266	2	32.92	35	1895	OB4063.raw	1.87E5	2	2	288	307	Oxidation (M)
K.ISM(+15.99)PVN(+.98)TPGQ(+.98)FEDFFPASSR.D	N	89.93	2244.0044	20	1.3	1123.0110	2	33.63	35	1944	OB4063.raw	3.04E5	2	2	288	307	Oxidation (M); Deamidation (NQ)
R.NTLEAAFN(+.98)AEFNEIR.R	N	89.50	1738.8162	15	-0.3	870.4151	2	34.79	35	2026	OB4063.raw	1.46E5	1	1	319	333	Deamidation (NQ)
K.HADADNILVIQQGQATVTVAN(+.98)GNNR.K	N	88.85	2619.3000	25	0.6	874.1078	3	29.47	35	1680	OB4063.raw	7.3E4	1	1	229	253	
K.ISM(+15.99)PVN(+.98)TPGQFEDFFPASSR.D	N	87.13	2243.0205	20	1.1	1122.5188	2	33.35	35	1914	OB4063.raw	3.87E5	1	1	288	307	Oxidation (M)
K.SFNLDEGHALR.I	N	86.49	1257.6101	11	0.6	629.8127	2	26.97	35	1516	OB4063.raw	1.94E6	2	2	255	265	
R.DQSSYLQGFSR.N	N	86.47	1286.5891	11	0.0	644.3018	2	29.29	35	1662	OB4063.raw	1.76E5	1	1	308	318	
K.KGSEEEGDITNPINLR.E	Y	85.75	1770.8748	16	1.2	886.4457	2	27.65	35	1568	OB4063.raw	1.36E5	2	2	386	401	
R.DQ(+.98)SSYLQ(+.98)GFSR.N	N	84.94	1288.5571	11	-0.3	645.2856	2	30.41	35	1733	OB4063.raw	1.05E5	1	1	308	318	Deamidation (NQ)
R.IPSGFISYILNR.H	N	84.71	1378.7609	12	-0.4	690.3875	2	35.35	35	2010	OB4063.raw	1.15E6	1	1	266	277	
K.GSEEEGDITNPINLR.E	Y	83.02	1642.7798	15	0.1	822.3972	2	29.29	35	1667	OB4063.raw	4.09E4	1	1	387	401	
R.DQSSYLQ(+.98)GFSR.N	N	82.67	1287.5731	11	0.5	644.7941	2	29.84	35	1698	OB4063.raw	2.3E5	1	1	308	318	Deamidation (NQ)
K.ISM(+15.99)PVNTPGQ(+.98)FEDFFPASSR.D	N	81.23	2243.0205	20	1.1	1122.5188	2	33.35	35	2029	OB4063.raw	6.87E5	2	2	288	307	Oxidation (M); Deamidation (NQ)
K.ISMPVNTPGQ(+.98)FEDFFPASSR.D	N	81.21	2227.0254	20	0.7	1114.5208	2	34.60	35	2017	OB4063.raw	4.86E4	1	1	288	307	Deamidation (NQ)
K.HADADNILVIQ(+.98)QGQATVTVAN(+.98)GNNR.K	N	80.52	2620.2842	25	2.1	874.4371	3	30.26	35	1728	OB4063.raw	0	0	0	229	253	
K.AM(+15.99)VIVVVNKGTGNLELVAVR.K	N	79.29	2097.1980	20	0.8	700.0739	3	31.57	35	1810	OB4063.raw	3.05E5	1	1	452	471	Oxidation (M)
R.VLLEENAGGEQEER.G	N	79.16	1571.7427	14	1.1	786.8795	2	25.50	35	1436	OB4063.raw	3.06E4	1	1	335	348	
R.DQ(+.98)SSYLQGFSR.N	N	77.03	1287.5731	11	0.5	644.7941	2	29.84	35	1711	OB4063.raw	2.3E5	1	1	308	318	Deamidation (NQ)
K.HADADNILVIQQGQ(+.98)ATVTVAN(+.98)GNNRK.S	N	75.31	2748.3792	26	2.5	917.1360	3	28.64	35	1624	OB4063.raw	0	0	0	229	254	Deamidation (NQ)
R.NTLEAAFNAEFNEIRR.V	N	75.09	1893.9332	16	0.5	947.9744	2	33.09	35	1877	OB4063.raw	4.8E6	3	3	319	334	
R.SSENNEGVIVK.V	Y	74.55	1174.5830	11	-0.4	588.2985	2	22.43	35	1245	OB4063.raw	2.18E5	2	2	357	367	
R.IFLAGDKDNVIDQIEK.Q	N	74.42	1816.9570	16	0.1	909.4858	2	30.71	35	1760	OB4063.raw	6.31E5	2	2	541	556	
K.HADADNILVIQQGQ(+.98)ATVTVANGN(+.98)NRK.S	N	71.87	2748.3792	26	0.7	917.1343	3	29.19	35	1658	OB4063.raw	0	0	0	229	254	
R.VLLEEN(+.98)AGGEQEER.G	N	71.84	1572.7267	14	-0.1	787.3705	2	26.03	35	1466	OB4063.raw	1.83E4	1	1	335	348	Deamidation (NQ)
K.ISMPVN(+.98)TPGQ(+.98)FEDFFPASSR.D	N	71.81	2228.0095	20	1.5	1115.0137	2	35.06	35	2042	OB4063.raw	2.31E4	1	1	288	307	Deamidation (NQ)
R.SSENN(+.98)EGVIVK.V	Y	71.14	1175.5670	11	0.2	588.7909	2	23.17	35	1297	OB4063.raw	1.14E5	1	1	357	367	
R.EGEPDLSN(+.98)NFGK.L	Y	70.08	1306.5676	12	0.6	654.2915	2	27.35	35	1553	OB4063.raw	1.64E4	1	1	402	413	
R.IFLAGDKDNVIDQ(+.98)IEKQAK.D	N	69.30	2145.1316	19	-0.9	716.0505	3	31.66	35	1816	OB4063.raw	4.92E5	2	2	541	559	Deamidation (NQ)
K.AM(+15.99)VIVVVN(+.98)KGTGNLELVAVR.K	N	69.22	2098.1819	20	-0.3	700.4010	3	32.05	35	1835	OB4063.raw	1.92E5	1	1	452	471	Oxidation (M)
R.VLLEEN(+.98)AGGEQ(+.98)EER.G	N	68.37	1573.7107	14	-1.3	787.8616	2	26.47	35	1490	OB4063.raw	2.78E3	1	1	335	348	Deamidation (NQ)
K.AM(+15.99)VIVVVNKGTGN(+.98)LELVAVR.K	N	67.58	2098.1819	20	-0.3	700.4010	3	32.05	35	1849	OB4063.raw	1.92E5	1	1	452	471	Oxidation (M)
K.HADADNILVIQ(+.98)QGQATVTVANGNNR.K	N	67.11	2619.3000	25	0.6	874.1078	3	29.47	35	1693	OB4063.raw	7.3E4	1	1	229	253	
R.SSEN(+.98)NEGVIVK.V	Y	67.08	1175.5670	11	0.2	588.7909	2	23.17	35	1281	OB4063.raw	1.14E5	1	1	357	367	Deamidation (NQ)
K.GTGNLELVAVR.K	N	66.74	1127.6299	11	-0.2	564.8221	2	28.83	35	1636	OB4063.raw	3.43E5	1	1	461	471	
R.VLLEENAGGEQ(+.98)EER.G	N	66.37	1572.7267	14	-0.1	787.3705	2	26.03	35	1454	OB4063.raw	1.83E4	1	1	335	348	Deamidation (NQ)
K.VSKEHVEELTK.H	Y	66.05	1297.6877	11	1.9	649.8524	2	20.30	35	1112	OB4063.raw	7.09E5	2	2	368	378	
K.HADADNILVIQQ(+.98)GQATVTVANGNN(+.98)R.K	N	65.90	2620.2842	25	2.1	874.4371	3	30.04	35	1714	OB4063.raw	0	0	0	229	253	
R.NTLEAAFNAEFN(+.98)EIR.R	N	65.73	1738.8162	15	-0.3	870.4151	2	34.79	35	2041	OB4063.raw	1.46E5	1	1	319	333	

Peptide	Uniq	-10lgP	Mass	Length	ppm	m/z	z	RT	Fraction	Scan	Source File	Area Digest 13 traka 60 kDa	#Feature	#Feature Digest 13 traka 60 kDa	Start	End	PTM
K.AMVIVVVNK.G	N	65.53	971.5837	9	-0.4	486.7990	2	28.34	35	1602	OB4063.raw	2.96E4	1	1	452	460	
R.EGEPDLSNNFGK.L	Y	64.64	1305.5836	12	0.2	653.7992	2	26.67	35	1510	OB4063.raw	4.34E4	1	1	402	413	
K.HADADNILVIQ(+.98)QGQ(+.98)ATVTVAN(+.98)GNNRK.S	N	64.16	2749.3630	26	0.9	917.4624	3	29.40	35	1668	OB4063.raw	7.4E4	1	1	229	254	
K.HADADNILVIQQGQATVTVANGN(+.98)NRK.S	N	64.04	2747.3950	26	-0.6	916.8051	3	29.05	35	1614	OB4063.raw	9.54E5	2	2	229	254	
K.HADADNILVIQQGQATVTVAN(+.98)GNNRK.S	N	63.48	2747.3950	26	-0.6	916.8051	3	29.05	35	1604	OB4063.raw	8.5E4	1	1	229	254	
K.HADADNILVIQQ(+.98)GQ(+.98)ATVTVANGN(+.98)NRK.S	N	62.90	2749.3630	26	0.9	917.4624	3	29.40	35	1692	OB4063.raw	7.4E4	1	1	229	254	Deamidation (NQ)
K.SFN(+.98)LDEGHALR.I	N	62.86	1258.5941	11	1.1	630.3051	2	27.44	35	1546	OB4063.raw	2.89E4	1	1	255	265	Deamidation (NQ)
R.IFLAGDKDNVIDQ(+.98)IEK.Q	N	62.82	1817.9410	16	-0.2	909.9776	2	31.18	35	1796	OB4063.raw	4.46E5	2	2	541	556	Deamidation (NQ)
R.IFLAGDKDNVIDQ(+.98)IEKQ(+.98)AK.D	N	62.39	2146.1157	19	-0.8	716.3786	3	32.23	35	1852	OB4063.raw	5.46E5	2	2	541	559	Deamidation (NQ)
R.NTLEAAFN(+.98)AEFNEIRR.V	N	62.26	1894.9172	16	0.1	948.4659	2	33.70	35	1943	OB4063.raw	8.02E5	3	3	319	334	Deamidation (NQ)
R.IVQIEAKPNTLVLPK.H	Y	62.06	1662.0079	15	0.6	832.0117	2	29.01	35	1638	OB4063.raw	1.36E6	2	2	214	228	
K.ISM(+15.99)PVNTPGQ(+.98)FEDFFPA.S	N	61.02	1912.8552	17	0.4	957.4353	2	36.10	35	2100	OB4063.raw	1.6E5	1	1	288	304	Oxidation (M); Deamidation (NQ)
K.AMVIVVVNKGTGNLELVAVR.K	N	60.67	2081.2031	20	-0.1	694.7416	3	33.00	35	1897	OB4063.raw	4.48E4	1	1	452	471	
K.ISM(+15.99)PVN(+.98)TPGQFEDFFPASSRDQ(+.98).S	N	60.43	2487.0898	22	-0.9	830.0365	3	33.35	35	1927	OB4063.raw	4.2E4	1	1	288	309	Oxidation (M); Deamidation (NQ)
K.AM(+15.99)VIVVVNK.G	N	60.32	987.5787	9	0.0	494.7966	2	25.83	35	1453	OB4063.raw	2.71E5	1	1	452	460	Oxidation (M)
R.IVQ(+.98)IEAKPN(+.98)TLVLPK.H	Y	58.59	1663.9760	15	-0.5	832.9948	2	29.84	35	1700	OB4063.raw	4.49E5	2	2	214	228	Deamidation (NQ)
R.IVQ(+.98)IEAKPNTLVLPK.H	Y	58.54	1662.9919	15	-0.3	832.5030	2	29.39	35	1661	OB4063.raw	2.25E6	2	2	214	228	Deamidation (NQ)
K.HADADNILVIQQ(+.98)GQ(+.98)ATVTVAN(+.98)GNNR.K	N	57.94	2621.2681	25	5.2	874.7678	3	30.83	35	1763	OB4063.raw	0	0	0	229	253	
R.IFLAGDKDN(+.98)VIDQ(+.98)IEKQAK.D	N	57.84	2146.1157	19	-0.8	716.3786	3	32.23	35	1840	OB4063.raw	9.45E4	1	1	541	559	Deamidation (NQ)
K.HADADNILVIQ(+.98)Q(+.98)GQ(+.98)ATVTVAN(+.98)GN(+.98)NRK.S	N	57.10	2751.3311	26	5.4	918.1226	3	30.12	35	1704	OB4063.raw	9.83E3	1	1	229	254	Deamidation (NQ)
K.HADADNILVIQ(+.98)QGQ(+.98)ATVTVANGNN(+.98)RK.S	N	56.78	2749.3630	26	0.9	917.4624	3	29.40	35	1681	OB4063.raw	7.4E4	1	1	229	254	Deamidation (NQ)
K.ISM(+15.99)PVNTPGQ(+.98)FEDFFPAS.S	N	56.37	1999.8873	18	1.0	1000.9519	2	35.53	35	2072	OB4063.raw	3.74E4	1	1	288	305	Oxidation (M); Deamidation (NQ)
K.GSEEEGDITNPINLREGEPDLSNNFGK.L	Y	55.88	2930.3530	27	1.3	977.7929	3	30.71	35	1751	OB4063.raw	6.55E4	1	1	387	413	
K.ISM(+15.99)PVNTPGQ(+.98)FEDFFPASSRDQ(+.98)SSYLQGFSR.N	N	55.84	3512.5830	31	2.1	879.1549	4	34.15	35	1978	OB4063.raw	0	0	0	288	318	Oxidation (M)
K.GTGNLELVAVRK.E	N	55.77	1255.7249	12	-0.8	628.8692	2	26.17	35	1475	OB4063.raw	1.95E4	1	1	461	472	
K.GSEEEGDITNPIN(+.98)LR.E	Y	55.59	1643.7638	15	-2.1	822.8875	2	29.76	35	1694	OB4063.raw	6.19E3	1	1	387	401	Deamidation (NQ)
R.VLLEENAGGEQE(+14.02)ER.G	N	55.55	1585.7583	14	0.7	793.8870	2	26.47	35	1488	OB4063.raw	8.52E3	1	1	335	348	Methylation(others)
R.IFLAGDKDN(+.98)VIDQ(+.98)IEK.Q	N	55.37	1818.9250	16	-1.5	607.3147	3	31.57	35	1804	OB4063.raw	4.47E4	1	1	541	556	Deamidation (NQ)
K.HADADNILVIQ(+.98)Q(+.98)GQATVTVAN(+.98)GN(+.98)NRK.S	N	54.33	2750.3472	26	4.7	917.7940	3	30.50	35	1742	OB4063.raw	0	0	0	229	254	
R.IVQIEAKPN(+.98)TLVLPK.H	Y	54.24	1662.9919	15	-0.3	832.5030	2	29.39	35	1675	OB4063.raw	2.25E6	2	2	214	228	
K.SFNLDEGHALRIPSGFISYILNR.H	N	53.34	2618.3604	23	0.8	655.5979	4	36.38	35	2140	OB4063.raw	4.23E5	2	2	255	277	
K.HADADNILVIQ(+.98)Q(+.98)GQATVTVAN(+.98)GNNR.K	N	53.21	2621.2681	25	6.2	874.7687	3	30.67	35	1753	OB4063.raw	0	0	0	229	253	
K.HADADNILVIQQ(+.98)GQ(+.98)ATVTVAN(+.98)GNNRK.S	N	53.18	2749.3630	26	0.9	917.4624	3	29.40	35	1704	OB4063.raw	7.4E4	1	1	229	254	
K.DLAFPGSGEQ(+.98)VEKLIK.N	N	53.12	1730.9089	16	0.6	866.4623	2	32.33	35	1862	OB4063.raw	2.24E4	1	1	560	575	Deamidation (NQ)
K.GSEEEGDITNPIN(+.98)LREGEPDLSNNFGK.L	Y	52.83	2931.3369	27	1.4	978.1209	3	30.92	35	1769	OB4063.raw	0	0	0	387	413	
K.ISM(+15.99)PVNTPGQ(+.98)FEDFFPASSRDQSSYLQ(+.98)GFSR.N	N	52.83	3512.5830	31	2.8	879.1555	4	33.97	35	1966	OB4063.raw	0	0	0	288	318	Oxidation (M); Deamidation (NQ)
K.HADADNILVIQQ(+.98)GQ(+.98)ATVTVANGNN(+.98)R.K	N	52.71	2621.2681	25	8.0	874.7703	3	29.80	35	1714	OB4063.raw	2.91E4	1	1	229	253	Deamidation (NQ)
R.IPSGFISYILN(+.98)R.H	N	51.41	1379.7449	12	2.4	690.8813	2	35.86	35	2080	OB4063.raw	9.57E4	1	1	266	277	Deamidation (NQ)
R.VLLEENAGGE(+28.03)QEER.G	N	51.23	1599.7739	14	-0.4	800.8939	2	27.35	35	1555	OB4063.raw	5.41E3	1	1	335	348	Ethylation

Peptide	Uniq	-10lgP	Mass	Length	ppm	m/z	z	RT	Fraction	Scan	Source File	Area Digest 13 traka 60 kDa	#Feature	#Feature Digest 13 traka 60 kDa	Start	End	PTM
R.VLLEENAGGEQEERGQ(+.98)R.R	N	51.22	1913.9078	17	-0.1	638.9765	3	24.27	35	1349	OB4063.raw	1.43E5	1	1	335	351	
R.NNPFYFPSR.R	N	51.10	1140.5352	9	-0.8	571.2744	2	30.41	35	1744	OB4063.raw	2.15E5	1	1	172	180	
K.HADADNILVIQQ(+.98)GQATVTVAN(+.98)GNNR.K	N	51.07	2620.2842	25	-2.6	874.4331	3	30.38	35	1735	OB4063.raw	0	0	0	229	253	
R.EETSRN(+.98)NPFYFPSRR.F	N	50.93	1899.8864	15	-0.4	634.3025	3	27.55	35	1559	OB4063.raw	3E4	1	1	167	181	
R.NTLEAAFNAEFN(+.98)EIRR.V	N	50.87	1894.9172	16	-2.0	632.6451	3	32.34	35	1858	OB4063.raw	0	0	0	319	334	
K.HADADNILVIQQ(+.98)GQ(+.98)ATVTVAN(+.98)GN(+.98)N(+.98)RK.S	N	50.75	2751.3311	26	5.4	918.1226	3	30.12	35	1716	OB4063.raw	9.83E3	1	1	229	254	
K.GSEEEGDITNPIN(+.98)LREGEPDLSNN(+.98)FGK.L	Y	50.68	2932.3210	27	0.6	978.4482	3	31.37	35	1813	OB4063.raw	6.9E4	1	1	387	413	
R.NNPFYFPSRR.F	N	50.48	1296.6364	10	0.0	649.3254	2	28.25	35	1595	OB4063.raw	1E6	2	2	172	181	
K.AM(+15.99)VIVVVN(+.98)KGTGN(+.98)LELVAVR.K	N	49.92	2099.1660	20	8.0	700.7349	3	32.92	35	1885	OB4063.raw	4.36E3	1	1	452	471	Oxidation (M); Deamidation (NQ)
R.IPSGFISYILNRHDN(+.98)QNLR.V	N	49.71	2257.1604	19	-0.8	565.2969	4	33.29	35	1919	OB4063.raw	0	0	0	266	284	
K.ISM(+15.99)PVNTPGQ(+.98)FEDFFPASSRDQSSYLQGFSR.N	N	49.65	3511.5989	31	1.0	878.9078	4	34.14	35	1949	OB4063.raw	6.17E5	1	1	288	318	Oxidation (M)
R.IPSGFISYILN(+.98)RHDNQ(+.98)NLR.V	N	49.47	2258.1443	19	2.5	565.5447	4	33.47	35	1931	OB4063.raw	0	0	0	266	284	Deamidation (NQ)
K.ISM(+15.99)PVNTPGQFEDFFPASSRDQ(+.98)SSYLQGFSR.N	N	49.33	3511.5989	31	1.8	1171.5424	3	33.82	35	1955	OB4063.raw	0	0	0	288	318	Oxidation (M)
R.IPSGFISYILNRHDNQ(+.98)NLR.V	N	49.25	2257.1604	19	-0.2	753.3939	3	33.45	35	1950	OB4063.raw	6.13E4	1	1	266	284	
K.KGSEEEGDITNPINLREGEPDLSNNFGK.L	Y	49.15	3058.4478	28	0.4	765.6195	4	29.60	35	1686	OB4063.raw	0	0	0	386	413	
R.IFLAGDKDNVIDQIEKQAK.D	N	48.22	2144.1477	19	2.3	715.7249	3	31.31	35	1777	OB4063.raw	9.33E3	1	1	541	559	
K.HADADNILVIQ(+.98)Q(+.98)GQ(+.98)ATVTVAN(+.98)GNNR.K	N	47.99	2622.2522	25	7.5	875.0979	3	30.51	35	1753	OB4063.raw	2.28E4	1	1	229	253	Deamidation (NQ)
K.ISM(+15.99)PVNTPGQ(+.98)FEDFFPASSRDQ(+.98)SSYLQ(+.98)GFSR.N	N	46.92	3513.5669	31	0.8	879.3997	4	34.35	35	1991	OB4063.raw	8.02E6	2	2	288	318	Oxidation (M); Deamidation (NQ)
K.HADADNILVIQQGQATVTVANGNN(+.98)RK.S	N	46.77	2747.3950	26	1.1	687.8568	4	28.16	35	1594	OB4063.raw	0	0	0	229	254	
R.Q(-17.03)FQNLQNHR.I	N	45.70	1166.5581	9	-0.2	584.2862	2	26.87	35	1514	OB4063.raw	4.01E3	1	1	205	213	Pyro-glu from Q
R.SSEN(+.98)N(+.98)EGVIVK.V	Y	45.49	1176.5510	11	0.5	589.2831	2	24.08	35	1343	OB4063.raw	1.3E4	1	1	357	367	Deamidation (NQ)
K.GSEEEGDITN(+.98)PINLREGEPDLSNNFGK.L	Y	45.47	2931.3369	27	0.5	978.1201	3	31.11	35	1781	OB4063.raw	0	0	0	387	413	
K.GSEEEGDITNPIN(+.98)LREGEPDLSN(+.98)NFGK.L	Y	45.47	2932.3210	27	6.7	978.4542	3	30.99	35	1790	OB4063.raw	6.95E4	1	1	387	413	
R.NTLEAAFN(+.98)AEFN(+.98)EIRR.V	N	44.87	1895.9012	16	4.5	948.9622	2	35.87	35	2089	OB4063.raw	1.14E6	1	1	319	334	Deamidation (NQ)
K.KGSEEEGDITNPIN(+.98)LREGEPDLSNNFGK.L	Y	44.18	3059.4319	28	0.7	765.8658	4	29.94	35	1710	OB4063.raw	4.16E5	1	1	386	413	
K.S(+43.01)(+14.02)FNLDEGHALR.I	N	44.07	1314.6316	11	-0.5	658.3228	2	27.05	35	1530	OB4063.raw	9.55E3	1	1	255	265	Carbamylation; Methylation(others)
K.HADADNILVIQ(+.98)Q(+.98)GQ(+.98)ATVTVANGNNR.K	N	43.79	2621.2681	25	8.0	874.7703	3	29.80	35	1693	OB4063.raw	2.91E4	1	1	229	253	Deamidation (NQ)
R.VLLEEN(+.98)AGGEQ(+.98)EERGQ(+.98)R.R	N	43.74	1915.8759	17	-0.6	639.6322	3	25.11	35	1409	OB4063.raw	8.19E4	1	1	335	351	Deamidation (NQ)
R.YGN(+.98)QN(+.98)GRIR.V	N	43.38	1078.5155	9	-0.7	540.2646	2	20.07	35	1082	OB4063.raw	3.11E5	2	2	186	194	Deamidation (NQ)
R.N(+.98)TLEAAFNAEFNEIR.R	N	43.25	1738.8162	15	-0.3	870.4151	2	34.79	35	2053	OB4063.raw	1.46E5	1	1	319	333	Deamidation (NQ)
K.EHVEELTK.H	Y	42.61	983.4924	8	-0.5	492.7532	2	19.96	35	1113	OB4063.raw	3.18E4	2	2	371	378	
R.SSENNEGVIVKVSK.E	Y	42.61	1488.7783	14	-0.4	497.2665	3	23.50	35	1304	OB4063.raw	1.83E4	1	1	357	370	
K.ISM(+15.99)PVN(+.98)TPGQ(+.98)FEDFFPA.S	N	42.42	1913.8392	17	-0.3	957.9266	2	36.47	35	2128	OB4063.raw	5.88E4	1	1	288	304	Oxidation (M); Deamidation (NQ)
R.DQSSYLQ(+.98)GFSRN(+.98)TLEAAFN(+.98)AEFNEIRR.V	N	42.25	3165.4639	27	3.3	1056.1654	3	36.32	35	2118	OB4063.raw	0	0	0	308	334	
R.SSENNE(+28.03)GVIVK.V	Y	42.11	1202.6143	11	-0.3	602.3142	2	26.10	35	1467	OB4063.raw	7.44E3	1	1	357	367	Ethylation
N.TPGQ(+.98)FEDFFPASSRDQ(+.98)SSYLQ(+.98)GFSR.N	N	41.87	2856.2515	25	6.8	953.0975	3	33.96	35	1980	OB4063.raw	2.48E5	1	1	294	318	Deamidation (NQ)
K.KGSEEEGDITNPIN(+.98)LREGEPDLSN(+.98)NFGK.L	Y	41.78	3060.4158	28	4.0	766.1143	4	30.44	35	1739	OB4063.raw	0	0	0	386	413	
R.DQSSYLQ(+.98)GFSRN(+.98)TLEAAFNAEFNEIRR.V	N	41.74	3164.4797	27	0.0	1055.8339	3	36.20	35	2118	OB4063.raw	7.48E4	1	1	308	334	
R.Q(-17.03)FQNLQ(+.98)NHR.I	N	41.25	1167.5421	9	-0.4	584.7781	2	27.85	35	1580	OB4063.raw	1.89E4	1	1	205	213	Pyro-glu from Q

Peptide	Uniq	-10lgP	Mass	Length	ppm	m/z	z	RT	Fraction	Scan	Source File	Area Digest 13 traka 60 kDa	#Feature	#Feature Digest 13 traka 60 kDa	Start	End	PTM
K.ISMPVNTPGQ(+.98)FEDFFPASSRDQ(+.98)SSYLQGFSR.N	N	40.49	3496.5881	31	1.6	1166.5386	3	35.06	35	2021	OB4063.raw	5.34E5	1	1	288	318	Deamidation (NQ)
R.VLLEENAGGEQ(+.98)EERGQRR.W	N	39.96	2070.0090	18	-5.0	691.0068	3	24.83	35	1391	OB4063.raw	4.28E4	1	1	335	352	
K.LFEVKPDK.K	N	39.73	974.5436	8	0.2	488.2792	2	24.38	35	1358	OB4063.raw	1.2E5	2	2	414	421	
K.ISM(+15.99)PVNTPGQFEDFFPASSRDQ(+.98)SSYLQ(+.98)GFSR.N	N	39.69	3512.5830	31	5.0	1171.8741	3	33.88	35	1960	OB4063.raw	0	0	0	288	318	Oxidation (M)
K.HADADNILVIQQ(+.98)GQATVTVANGN(+.98)N(+.98)RK.S	N	39.50	2749.3630	26	6.9	688.3528	4	31.09	35	1842	OB4063.raw	5.25E4	1	1	229	254	
R.IPSGFISYILN(+.98)RHDNQ(+.98)N(+.98)LR.V	N	38.84	2259.1284	19	5.7	565.7926	4	33.53	35	2009	OB4063.raw	1.27E6	1	1	266	284	
R.IPSGFISYILN(+.98)RHDNQN(+.98)LR.V	N	38.53	2258.1443	19	-1.8	753.7207	3	33.54	35	1936	OB4063.raw	0	0	0	266	284	Deamidation (NQ)
K.ISMPVNTPGQFEDFFPASSRDQ(+.98)SSYLQGFSR.N	N	38.43	3495.6040	31	2.4	1166.2114	3	34.55	35	2004	OB4063.raw	0	0	0	288	318	
K.KGSEEEGDITNPINLREGEPDLSNN(+.98)FGK.L	Y	37.80	3059.4319	28	0.7	765.8658	4	29.94	35	1784	OB4063.raw	4.16E5	1	1	386	413	
R.YGNQ(+.98)N(+.98)GRIR.V	N	37.77	1078.5155	9	-0.7	540.2646	2	20.07	35	1094	OB4063.raw	3.11E5	2	2	186	194	
K.GSEEEGDITNPINLREGEPDLSN(+.98)NFGK.L	Y	37.53	2931.3369	27	2.4	978.1219	3	31.51	35	1806	OB4063.raw	0	0	0	387	413	
R.VLLEENAGGEQ(+.98)EERGQ(+.98)R.R	N	36.86	1914.8918	17	0.2	639.3047	3	24.64	35	1374	OB4063.raw	1.77E5	1	1	335	351	
F.NLDEGHALR.I	N	36.61	1023.5097	9	0.8	512.7625	2	26.97	35	1531	OB4063.raw	1.56E4	1	1	257	265	
R.VLLEEN(+.98)AGGEQ(+.98)EERGQR.R	N	36.15	1914.8918	17	0.2	639.3047	3	24.64	35	1398	OB4063.raw	1.77E5	1	1	335	351	
R.IPSGFISYILNRHDN(+.98)Q(+.98)NLR.V	N	36.15	2258.1443	19	0.8	565.5438	4	34.62	35	2009	OB4063.raw	0	0	0	266	284	
R.IPSGFISYILN(+.98)RHDN(+.98)QNLR.V	N	36.12	2258.1443	19	-1.6	565.5424	4	35.07	35	2038	OB4063.raw	7.93E4	1	1	266	284	
R.IPSGFISYILN(+.98)RHDNQNLR.V	N	36.08	2257.1604	19	0.6	753.3945	3	33.08	35	1904	OB4063.raw	0	0	0	266	284	
R.LKEGDVFIM(+15.99)PAA.H	N	35.74	1305.6638	12	-0.5	653.8389	2	29.47	35	1678	OB4063.raw	3.05E4	1	1	504	515	Oxidation (M)
R.N(+.98)NPFYFPSRR.F	N	35.51	1297.6204	10	0.8	649.8180	2	29.01	35	1644	OB4063.raw	4.08E4	1	1	172	181	
R.Q(-17.03)FQN(+.98)LQ(+.98)NHR.I	N	35.33	1168.5261	9	-0.3	585.2701	2	28.34	35	1609	OB4063.raw	1.07E4	1	1	205	213	Pyro-glu from Q; Deamidation (NQ)
K.Q(+.98)AKDLAFPGSGEQ(+.98)VEK.L	N	35.23	1704.8206	16	-1.9	569.2797	3	26.67	35	1500	OB4063.raw	8.39E3	1	1	557	572	Deamidation (NQ)
K.SFN(+.98)LDEGHALRIPSGFISYILNR.H	N	35.13	2619.3445	23	6.9	655.8479	4	40.04	35	2348	OB4063.raw	0	0	0	255	277	Deamidation (NQ)
K.ISM(+15.99)PVN(+.98)TPGQFEDFFPASSRDQ(+.98)SSYLQ(+.98)GFSR.N	N	35.04	3513.5669	31	5.7	1172.2029	3	34.24	35	1990	OB4063.raw	8E6	1	1	288	318	Oxidation (M); Deamidation (NQ)
K.KGSEEEGDITN(+.98)PINLREGEPDLSN(+.98)NFGK.L	Y	34.14	3060.4158	28	6.5	766.1162	4	30.54	35	1745	OB4063.raw	0	0	0	386	413	
R.VLLEENAGGEQ(+.98)EERGQR.R	N	34.10	1913.9078	17	-0.1	638.9765	3	24.27	35	1364	OB4063.raw	1.43E5	1	1	335	351	
R.IPSGFISYILN(+.98)RHDN(+.98)Q(+.98)NLR.V	N	33.82	2259.1284	19	7.5	754.0557	3	33.45	35	1950	OB4063.raw	1.33E6	2	2	266	284	Deamidation (NQ)
R.IPSGFISYILNRHDNQN(+.98)LR.V	N	33.54	2257.1604	19	-0.2	753.3939	3	33.45	35	1936	OB4063.raw	6.13E4	1	1	266	284	
R.VLLEEN(+.98)AGGEQ(+.98)EERGQRR.W	N	33.54	2070.9929	18	-1.6	691.3372	3	23.68	35	1319	OB4063.raw	3.7E4	1	1	335	352	Deamidation (NQ)
R.YGN(+.98)QNGRIR.V	N	33.50	1077.5315	9	0.9	360.1848	3	19.20	35	1038	OB4063.raw	1.04E5	1	1	186	194	
R.YGN(+.98)Q(+.98)N(+.98)GRIR.V	N	33.15	1079.4995	9	1.3	360.8409	3	20.88	35	1147	OB4063.raw	6.14E4	1	1	186	194	Deamidation (NQ)
K.E(-18.01)HVEELTK.H	Y	33.15	965.4818	8	-1.0	483.7477	2	23.89	35	1329	OB4063.raw	1.77E4	1	1	371	378	Pyro-glu from E
K.NPQ(+.98)LQ(+.98)DLDM(+15.99)M(+15.99)LTC(+57.02)VEIKEGALM(+15.99)LPHFNSK.A	N	32.90	3421.5913	29	2.5	856.4072	4	34.21	35	1981	OB4063.raw	0	0	0	423	451	Oxidation (M); Carbamidomethylation
K.ISM(+15.99)PVN(+.98)TPGQ(+.98)FEDFFPASSRDQ(+.98)SSYLQGFSR.N	N	32.83	3513.5669	31	5.7	1172.2029	3	34.24	35	2188	OB4063.raw	8E6	1	1	288	318	Oxidation (M); Deamidation (NQ)
K.LFEVKPDKK.N	N	32.65	1102.6385	9	-1.0	552.3260	2	22.20	35	1226	OB4063.raw	3.34E3	1	1	414	422	
K.NPQ(+.98)LQDLDM(+15.99)M(+15.99)LTC(+57.02)VEIKEGALM(+15.99)LPHFNSK.A	N	32.51	3420.6074	29	1.2	856.1602	4	33.80	35	1981	OB4063.raw	1.32E5	1	1	423	451	Oxidation (M); Carbamidomethylation
R.YGNQN(+.98)GRIR.V	N	32.16	1077.5315	9	0.9	360.1848	3	19.20	35	1048	OB4063.raw	1.04E5	1	1	186	194	
R.SSENN(+.98)EGVIVKVSK.E	Y	32.08	1489.7623	14	-1.9	497.5938	3	24.27	35	1361	OB4063.raw	1.23E4	1	1	357	370	
N.TPGQ(+.98)FEDFFPASSRDQSSYLQ(+.98)GFSR.N	N	31.78	2855.2673	25	-0.6	952.7625	3	33.71	35	1948	OB4063.raw	0	0	0	294	318	Deamidation (NQ)

Peptide	Uniq	-10lgP	Mass	Length	ppm	m/z	z	RT	Fraction	Scan	Source File	Area Digest 13 traka 60 kDa	#Feature	#Feature Digest 13 traka 60 kDa	Start	End	PTM
R.Q(-17.03)FQN(+.98)LQNHR.I	N	31.44	1167.5421	9	-0.4	584.7781	2	27.85	35	1554	OB4063.raw	1.89E4	1	1	205	213	Pyro-glu from Q
K.ISMPVNTPGQ(+.98)FEDFFPASSRDQ(+.98)SSYLQ(+.98)GFSR.N	N	31.28	3497.5720	31	2.6	1166.8677	3	35.17	35	2044	OB4063.raw	0	0	0	288	318	Deamidation (NQ)
R.SSE(+21.98)NNEGVIVK.V	Y	31.12	1196.5649	11	-0.4	599.2895	2	22.43	35	1243	OB4063.raw	1.34E3	1	1	357	367	
R.DQ(+.98)SSYLQ(+.98)GFSRN(+.98)TLEAAFN(+.98)AEFN(+.98)EIRR.V	N	30.59	3167.4319	27	8.2	1056.8265	3	36.57	35	2129	OB4063.raw	1.42E5	1	1	308	334	Deamidation (NQ)
K.EGALM(+15.99)LPHFNSK.A	N	30.42	1358.6653	12	-0.2	453.8956	3	29.01	35	1643	OB4063.raw	1.66E5	1	1	440	451	Oxidation (M)
K.ISM(+15.99)P(+15.99)VNTPGQ(+.98)FEDFFPASSRDQ(+.98)SSYLQ(+.98)GFSR.N	N	29.84	3529.5620	31	7.8	1177.5371	3	34.77	35	2018	OB4063.raw	0	0	0	288	318	Oxidation (M)
R.VLLEENAGGEQEERGQ(+.98)RR.W	N	29.67	2070.0090	18	-1.3	691.0094	3	23.17	35	1278	OB4063.raw	4.28E4	1	1	335	352	
R.RVLLEEN(+.98)AGGEQ(+.98)EERGQ(+.98)R.R	N	29.64	2071.9771	18	-2.9	691.6643	3	24.08	35	1347	OB4063.raw	1.44E4	1	1	334	351	Deamidation (NQ)
R.SRQ(+.98)FQ(+.98)N(+.98)LQ(+.98)NHRIVQIEAKPN(+.98)TLVLPK.H	Y	29.31	3075.6353	26	0.3	616.1345	5	30.41	35	1741	OB4063.raw	8.17E4	1	1	203	228	
R.SRQ(+.98)FQ(+.98)N(+.98)LQN(+.98)HRIVQ(+.98)IEAKPNTLVLPK.H	Y	28.93	3075.6353	26	0.3	616.1345	5	30.41	35	1715	OB4063.raw	8.17E4	1	1	203	228	Deamidation (NQ)
R.VLLEEN(+.98)AGGEQEERGQ(+.98)R.R	N	28.61	1914.8918	17	0.2	639.3047	3	24.64	35	1422	OB4063.raw	1.77E5	1	1	335	351	
K.SFNLDEGHALRIPSGFISYILNRHDNQN(+.98)LR.V	N	28.47	3496.7600	30	-0.1	700.3592	5	35.63	35	2074	OB4063.raw	1.68E5	1	1	255	284	
R.YGN(+.98)Q(+.98)NGRIR.V	N	28.16	1078.5155	9	1.7	360.5131	3	20.06	35	1088	OB4063.raw	2.55E5	1	1	186	194	
K.Q(+.98)AKDLAFPGSGEQVEK.L	N	28.09	1703.8365	16	0.7	568.9532	3	26.48	35	1483	OB4063.raw	1.25E4	1	1	557	572	Deamidation (NQ)
G.SEEEGDITNPINLREGEPDLSN(+.98)NFGK.L	Y	28.08	2874.3154	26	1.7	959.1140	3	30.99	35	1778	OB4063.raw	2.76E4	1	1	388	413	
K.SFNLDEGHALRIPSGFISYILN(+.98)RHDNQN(+.98)LR.V	N	27.94	3497.7439	30	3.3	700.5583	5	35.72	35	2074	OB4063.raw	3.15E5	1	1	255	284	
K.KGSEEEGDITNPINLREGEPDL.S	Y	27.39	2411.1453	22	1.7	804.7237	3	30.21	35	1730	OB4063.raw	1.15E4	1	1	386	407	
E.WGTPGSHVR.E	Y	26.65	995.4937	9	-1.0	332.8382	3	21.98	35	1214	OB4063.raw	2.53E4	1	1	158	166	
K.KGSEEEGDITN(+.98)PINLREGEPDLSNNFGK.L	Y	26.43	3059.4319	28	0.7	765.8658	4	29.94	35	1772	OB4063.raw	4.16E5	1	1	386	413	
R.VLLEENAGGEQEERGQR.R	N	26.31	1912.9238	17	0.6	638.6489	3	23.89	35	1337	OB4063.raw	4.66E4	1	1	335	351	
K.N(+.98)PQLQDLDM(+15.99)M(+15.99)LTC(+57.02)VEIKEGALM(+15.99)LPHFNSK.A	N	25.90	3420.6074	29	1.2	856.1602	4	33.80	35	1994	OB4063.raw	1.32E5	1	1	423	451	Oxidation (M); Carbamidomethylation
R.DQ(+.98)SSYLQGFSRN(+.98)TLEAAFN(+.98)AEFN(+.98)EIRR.V	N	25.88	3166.4478	27	5.1	1056.4952	3	36.89	35	2153	OB4063.raw	0	0	0	308	334	Deamidation (NQ)
D.ADNILVIQQ(+.98)GQATVTVANGNNRK.S	N	25.85	2424.2720	23	-0.2	809.0978	3	28.93	35	1652	OB4063.raw	8.86E3	1	1	232	254	
R.S(+28.03)SENNEGVIVK.V	Y	25.46	1202.6143	11	2.9	602.3162	2	25.05	35	1406	OB4063.raw	2.16E3	1	1	357	367	
R.DQ(+.98)SSYLQGFSRNTLEAAFNAEFN(+.98)EIRR.V	N	25.25	3164.4797	27	0.0	1055.8339	3	36.20	35	2153	OB4063.raw	7.48E4	1	1	308	334	
R.DQ(+.98)SSYLQGFSRN(+.98)TLEAAFNAEFNEIRR.V	N	25.24	3164.4797	27	2.2	792.1290	4	36.28	35	2105	OB4063.raw	2.6E5	1	1	308	334	
R.VLLEENAGGEQEERGQRR.W	N	25.15	2069.0249	18	0.5	690.6826	3	22.58	35	1271	OB4063.raw	1.8E4	1	1	335	352	
K.KGSEEEGDITNPINLREG.E	Y	24.90	1956.9388	18	-1.0	653.3196	3	27.75	35	1573	OB4063.raw	3.41E3	1	1	386	403	
R.DQ(+.98)SSYLQ(+.98)GFSRNTLEAAFNAEFN(+.98)EIRR.V	N	24.52	3165.4639	27	3.1	792.3757	4	36.40	35	2123	OB4063.raw	0	0	0	308	334	
K.HADADNILVIQ(+.98)QGQ(+.98)ATVTVAN(+.98)GNN(+.98)RK.S	N	24.44	2750.3472	26	1.8	688.5953	4	32.27	35	1854	OB4063.raw	0	0	0	229	254	
**total 185 peptides**																	

P43238|ALL12_ARAHY

back to list

| Protein Coverage | Supporting Peptides |

Protein Coverage:

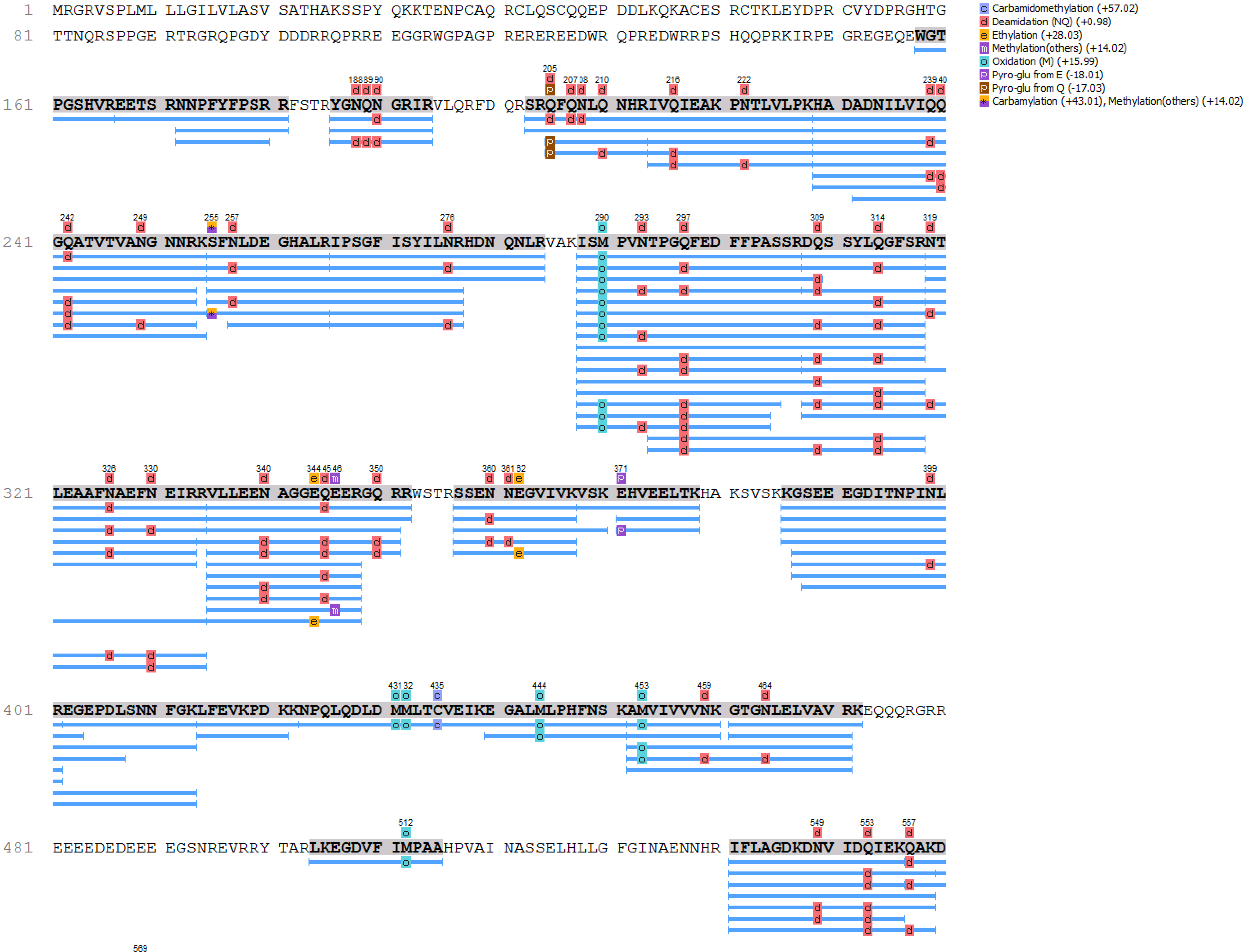
1 MRGRVSPLML LLGILVLASV SATHAKSSPY QKKTENPCAQ RCLQSCQQEP DDLKQKACES RCTKLEYDPR CVYDPRGHTG
81 TTNQRSPPGE RTRGRQPGDY DDDRRQPRRE EGGRWGPAGP REREREEDWR QPREDWRRPS HQQPRKIRPE GREGEQEWGT
161 PGSHVREETS RNNPFYFPSR RFSTRYGNQN GRIRVLQRFD QRSRQFQNLQ NHRIVQIEAK PNTLVLPKHA DADNILVIQQ
241 GQATVTVANG NNRKSFNLDE GHALRIPSGF ISYILNRHDN QNLRVAKISM PVNTPGQFED FFPASSRDQS SYLQGFSRNT
321 LEAAFNAEFN EIRRVLLEEN AGGEQEERGQ RRWSTRSSEN NEGVIVKVSK EHVEELTKHA KSVSKKGSEE EGDITNPINL
401 REGEPDLSNN FGKLFEVKPD KKNPQLQDLD MMLTCVEIKE GALMLPHFNS KAMVIVVVNK GTGNLELVAV RKEQQQRGRR
481 EEEEDEDEEE EGSNREVRRY TARLKEGDVF IMPAAHPVAI NASSELHLLG FGINAENNHR IFLAGDKDNV IDQIEKQAKD
c Carbamidomethylation (+57.02)
d Deamidation (NQ) (+0.98)
e Ethylation (+28.03)
m Methylation(others) (+14.02)
o Oxidation (M) (+15.99)
p Pyro-glu from E (-18.01)
p Pyro-glu from Q (-17.03)
Carbamylation (+43.01), Methylation(others) (+14.02)

Supporting Peptides:

Peptide	Uniq	-10lgP	Mass	Length	ppm	m/z	z	RT	Fraction	Scan	Source File	Area Digest 13 traka 60 kDa	#Feature	#Feature Digest 13 traka 60 kDa	Start	End	PTM
R.NTLEAAFNAEFNEIR.R	N	104.37	1737.8322	15	0.4	869.9237	2	34.24	35	1984	OB4063.raw	2.88E5	1	1	319	333	
K.ISM(+15.99)PVNTPGQFEDFFPASSR.D	N	91.29	2242.0364	20	1.0	1122.0266	2	32.92	35	1895	OB4063.raw	1.87E5	2	2	288	307	Oxidation (M)
K.ISM(+15.99)PVN(+.98)TPGQ(+.98)FEDFFPASSR.D	N	89.93	2244.0044	20	1.3	1123.0110	2	33.63	35	1944	OB4063.raw	3.04E5	2	2	288	307	Oxidation (M); Deamidation (NQ)
R.NTLEAAFN(+.98)AEFNEIR.R	N	89.50	1738.8162	15	-0.3	870.4151	2	34.79	35	2026	OB4063.raw	1.46E5	1	1	319	333	Deamidation (NQ)
K.HADADNILVIQQGQATVTVAN(+.98)GNNR.K	N	88.85	2619.3000	25	0.6	874.1078	3	29.47	35	1680	OB4063.raw	7.3E4	1	1	229	253	
K.ISM(+15.99)PVN(+.98)TPGQFEDFFPASSR.D	N	87.13	2243.0205	20	1.1	1122.5188	2	33.35	35	1914	OB4063.raw	3.87E5	1	1	288	307	Oxidation (M)
K.SFNLDEGHALR.I	N	86.49	1257.6101	11	0.6	629.8127	2	26.97	35	1516	OB4063.raw	1.94E6	2	2	255	265	
R.DQSSYLQGFSR.N	N	86.47	1286.5891	11	0.0	644.3018	2	29.29	35	1662	OB4063.raw	1.76E5	1	1	308	318	
K.KGSEEEGDITNPINLR.E	Y	85.75	1770.8748	16	1.2	886.4457	2	27.65	35	1568	OB4063.raw	1.36E5	2	2	386	401	
R.DQ(+.98)SSYLQ(+.98)GFSR.N	N	84.94	1288.5571	11	-0.3	645.2856	2	30.41	35	1733	OB4063.raw	1.05E5	1	1	308	318	Deamidation (NQ)
R.IPSGFISYILNR.H	N	84.71	1378.7609	12	-0.4	690.3875	2	35.35	35	2010	OB4063.raw	1.15E6	1	1	266	277	
K.GSEEEGDITNPINLR.E	Y	83.02	1642.7798	15	0.1	822.3972	2	29.29	35	1667	OB4063.raw	4.09E4	1	1	387	401	
R.DQSSYLQ(+.98)GFSR.N	N	82.67	1287.5731	11	0.5	644.7941	2	29.84	35	1698	OB4063.raw	2.3E5	1	1	308	318	Deamidation (NQ)
K.ISM(+15.99)PVNTPGQ(+.98)FEDFFPASSR.D	N	81.23	2243.0205	20	1.1	1122.5188	2	33.35	35	2029	OB4063.raw	6.87E5	2	2	288	307	Oxidation (M); Deamidation (NQ)
K.ISMPVNTPGQ(+.98)FEDFFPASSR.D	N	81.21	2227.0254	20	0.7	1114.5208	2	34.60	35	2017	OB4063.raw	4.86E4	1	1	288	307	Deamidation (NQ)
K.HADADNILVIQ(+.98)QGQATVTVAN(+.98)GNNR.K	N	80.52	2620.2842	25	2.1	874.4371	3	30.26	35	1728	OB4063.raw	0	0	0	229	253	
K.AM(+15.99)VIVVVNKGTGNLELVAVR.K	N	79.29	2097.1980	20	0.8	700.0739	3	31.57	35	1810	OB4063.raw	3.05E5	1	1	452	471	Oxidation (M)
R.VLLEENAGGEQEER.G	N	79.16	1571.7427	14	1.1	786.8795	2	25.50	35	1436	OB4063.raw	3.06E4	1	1	335	348	
R.DQ(+.98)SSYLQGFSR.N	N	77.03	1287.5731	11	0.5	644.7941	2	29.84	35	1711	OB4063.raw	2.3E5	1	1	308	318	Deamidation (NQ)
K.HADADNILVIQQGQ(+.98)ATVTVAN(+.98)GNNRK.S	N	75.31	2748.3792	26	2.5	917.1360	3	28.64	35	1624	OB4063.raw	0	0	0	229	254	Deamidation (NQ)
R.NTLEAAFNAEFNEIRR.V	N	75.09	1893.9332	16	0.5	947.9744	2	33.09	35	1877	OB4063.raw	4.8E6	3	3	319	334	
R.SSENNEGVIVK.V	Y	74.55	1174.5830	11	-0.4	588.2985	2	22.43	35	1245	OB4063.raw	2.18E5	2	2	357	367	
R.IFLAGDKDNVIDQIEK.Q	N	74.42	1816.9570	16	0.1	909.4858	2	30.71	35	1760	OB4063.raw	6.31E5	2	2	541	556	
K.HADADNILVIQQGQ(+.98)ATVTVANGN(+.98)NRK.S	N	71.87	2748.3792	26	0.7	917.1343	3	29.19	35	1658	OB4063.raw	0	0	0	229	254	
R.VLLEEN(+.98)AGGEQEER.G	N	71.84	1572.7267	14	-0.1	787.3705	2	26.03	35	1466	OB4063.raw	1.83E4	1	1	335	348	Deamidation (NQ)
K.ISMPVN(+.98)TPGQ(+.98)FEDFFPASSR.D	N	71.81	2228.0095	20	1.5	1115.0137	2	35.06	35	2042	OB4063.raw	2.31E4	1	1	288	307	Deamidation (NQ)
R.SSENN(+.98)EGVIVK.V	Y	71.14	1175.5670	11	0.2	588.7909	2	23.17	35	1297	OB4063.raw	1.14E5	1	1	357	367	
R.EGEPDLSN(+.98)NFGK.L	Y	70.08	1306.5676	12	0.6	654.2915	2	27.35	35	1553	OB4063.raw	1.64E4	1	1	402	413	
R.IFLAGDKDNVIDQ(+.98)IEKQAK.D	N	69.30	2145.1316	19	-0.9	716.0505	3	31.66	35	1816	OB4063.raw	4.92E5	2	2	541	559	Deamidation (NQ)
K.AM(+15.99)VIVVVN(+.98)KGTGNLELVAVR.K	N	69.22	2098.1819	20	-0.3	700.4010	3	32.05	35	1835	OB4063.raw	1.92E5	1	1	452	471	Oxidation (M)
R.VLLEEN(+.98)AGGEQ(+.98)EER.G	N	68.37	1573.7107	14	-1.3	787.8616	2	26.47	35	1490	OB4063.raw	2.78E3	1	1	335	348	Deamidation (NQ)
K.AM(+15.99)VIVVVNKGTGN(+.98)LELVAVR.K	N	67.58	2098.1819	20	-0.3	700.4010	3	32.05	35	1849	OB4063.raw	1.92E5	1	1	452	471	Oxidation (M)
K.HADADNILVIQ(+.98)QGQATVTVANGNNR.K	N	67.11	2619.3000	25	0.6	874.1078	3	29.47	35	1693	OB4063.raw	7.3E4	1	1	229	253	
R.SSEN(+.98)NEGVIVK.V	Y	67.08	1175.5670	11	0.2	588.7909	2	23.17	35	1281	OB4063.raw	1.14E5	1	1	357	367	Deamidation (NQ)
K.GTGNLELVAVR.K	N	66.74	1127.6299	11	-0.2	564.8221	2	28.83	35	1636	OB4063.raw	3.43E5	1	1	461	471	
R.VLLEENAGGEQ(+.98)EER.G	N	66.37	1572.7267	14	-0.1	787.3705	2	26.03	35	1454	OB4063.raw	1.83E4	1	1	335	348	Deamidation (NQ)
K.VSKEHVEELTK.H	Y	66.05	1297.6877	11	1.9	649.8524	2	20.30	35	1112	OB4063.raw	7.09E5	2	2	368	378	
K.HADADNILVIQQ(+.98)GQATVTVANGNN(+.98)R.K	N	65.90	2620.2842	25	2.1	874.4371	3	30.04	35	1714	OB4063.raw	0	0	0	229	253	
R.NTLEAAFNAEFN(+.98)EIR.R	N	65.73	1738.8162	15	-0.3	870.4151	2	34.79	35	2041	OB4063.raw	1.46E5	1	1	319	333	

Peptide	Uniq	-10lgP	Mass	Length	ppm	m/z	z	RT	Fraction	Scan	Source File	Area Digest 13 traka 60 kDa	#Feature	#Feature Digest 13 traka 60 kDa	Start	End	PTM
K.AMVIVVVNK.G	N	65.53	971.5837	9	-0.4	486.7990	2	28.34	35	1602	OB4063.raw	2.96E4	1	1	452	460	
R.EGEPDLSNNFGK.L	Y	64.64	1305.5836	12	0.2	653.7992	2	26.67	35	1510	OB4063.raw	4.34E4	1	1	402	413	
K.HADADNILVIQ(+.98)QGQ(+.98)ATVTVAN(+.98)GNNRK.S	N	64.16	2749.3630	26	0.9	917.4624	3	29.40	35	1668	OB4063.raw	7.4E4	1	1	229	254	
K.HADADNILVIQQGQATVTVANGN(+.98)NRK.S	N	64.04	2747.3950	26	-0.6	916.8051	3	29.05	35	1614	OB4063.raw	9.54E5	2	2	229	254	
K.HADADNILVIQQGQATVTVAN(+.98)GNNRK.S	N	63.48	2747.3950	26	-0.6	916.8051	3	29.05	35	1604	OB4063.raw	8.5E4	1	1	229	254	
K.HADADNILVIQQ(+.98)GQ(+.98)ATVTVANGN(+.98)NRK.S	N	62.90	2749.3630	26	0.9	917.4624	3	29.40	35	1692	OB4063.raw	7.4E4	1	1	229	254	Deamidation (NQ)
K.SFN(+.98)LDEGHALR.I	N	62.86	1258.5941	11	1.1	630.3051	2	27.44	35	1546	OB4063.raw	2.89E4	1	1	255	265	Deamidation (NQ)
R.IFLAGDKDNVIDQ(+.98)IEK.Q	N	62.82	1817.9410	16	-0.2	909.9776	2	31.18	35	1796	OB4063.raw	4.46E5	2	2	541	556	Deamidation (NQ)
R.IFLAGDKDNVIDQ(+.98)IEKQ(+.98)AK.D	N	62.39	2146.1157	19	-0.8	716.3786	3	32.23	35	1852	OB4063.raw	5.46E5	2	2	541	559	Deamidation (NQ)
R.NTLEAAFN(+.98)AEFNEIRR.V	N	62.26	1894.9172	16	0.1	948.4659	2	33.70	35	1943	OB4063.raw	8.02E5	3	3	319	334	Deamidation (NQ)
R.IVQIEAKPNTLVLPK.H	Y	62.06	1662.0079	15	0.6	832.0117	2	29.01	35	1638	OB4063.raw	1.36E6	2	2	214	228	
K.ISM(+15.99)PVNTPGQ(+.98)FEDFFPA.S	N	61.02	1912.8552	17	0.4	957.4353	2	36.10	35	2100	OB4063.raw	1.6E5	1	1	288	304	Oxidation (M); Deamidation (NQ)
K.AMVIVVVNKGTGNLELVAVR.K	N	60.67	2081.2031	20	-0.1	694.7416	3	33.00	35	1897	OB4063.raw	4.48E4	1	1	452	471	
K.ISM(+15.99)PVN(+.98)TPGQFEDFFPASSRDQ(+.98).S	N	60.43	2487.0898	22	-0.9	830.0365	3	33.35	35	1927	OB4063.raw	4.2E4	1	1	288	309	Oxidation (M); Deamidation (NQ)
K.AM(+15.99)VIVVVNK.G	N	60.32	987.5787	9	0.0	494.7966	2	25.83	35	1453	OB4063.raw	2.71E5	1	1	452	460	Oxidation (M)
R.IVQ(+.98)IEAKPN(+.98)TLVLPK.H	Y	58.59	1663.9760	15	-0.5	832.9948	2	29.84	35	1700	OB4063.raw	4.49E5	2	2	214	228	Deamidation (NQ)
R.IVQ(+.98)IEAKPNTLVLPK.H	Y	58.54	1662.9919	15	-0.3	832.5030	2	29.39	35	1661	OB4063.raw	2.25E6	2	2	214	228	Deamidation (NQ)
K.HADADNILVIQQ(+.98)GQ(+.98)ATVTVAN(+.98)GNNR.K	N	57.94	2621.2681	25	5.2	874.7678	3	30.83	35	1763	OB4063.raw	0	0	0	229	253	
R.IFLAGDKDN(+.98)VIDQ(+.98)IEKQAK.D	N	57.84	2146.1157	19	-0.8	716.3786	3	32.23	35	1840	OB4063.raw	9.45E4	1	1	541	559	Deamidation (NQ)
K.HADADNILVIQ(+.98)Q(+.98)GQ(+.98)ATVTVAN(+.98)GN(+.98)NRK.S	N	57.10	2751.3311	26	5.4	918.1226	3	30.12	35	1704	OB4063.raw	9.83E3	1	1	229	254	Deamidation (NQ)
K.HADADNILVIQ(+.98)QGQ(+.98)ATVTVANGNN(+.98)RK.S	N	56.78	2749.3630	26	0.9	917.4624	3	29.40	35	1681	OB4063.raw	7.4E4	1	1	229	254	Deamidation (NQ)
K.ISM(+15.99)PVNTPGQ(+.98)FEDFFPAS.S	N	56.37	1999.8873	18	1.0	1000.9519	2	35.53	35	2072	OB4063.raw	3.74E4	1	1	288	305	Oxidation (M); Deamidation (NQ)
K.GSEEEGDITNPINLREGEPDLSNNFGK.L	Y	55.88	2930.3530	27	1.3	977.7929	3	30.71	35	1751	OB4063.raw	6.55E4	1	1	387	413	
K.ISM(+15.99)PVNTPGQ(+.98)FEDFFPASSRDQ(+.98)SSYLQGFSR.N	N	55.84	3512.5830	31	2.1	879.1549	4	34.15	35	1978	OB4063.raw	0	0	0	288	318	Oxidation (M)
K.GTGNLELVAVRK.E	N	55.77	1255.7249	12	-0.8	628.8692	2	26.17	35	1475	OB4063.raw	1.95E4	1	1	461	472	
K.GSEEEGDITNPIN(+.98)LR.E	Y	55.59	1643.7638	15	-2.1	822.8875	2	29.76	35	1694	OB4063.raw	6.19E3	1	1	387	401	Deamidation (NQ)
R.VLLEENAGGEQE(+14.02)ER.G	N	55.55	1585.7583	14	0.7	793.8870	2	26.47	35	1488	OB4063.raw	8.52E3	1	1	335	348	Methylation(others)
R.IFLAGDKDN(+.98)VIDQ(+.98)IEK.Q	N	55.37	1818.9250	16	-1.5	607.3147	3	31.57	35	1804	OB4063.raw	4.47E4	1	1	541	556	Deamidation (NQ)
K.HADADNILVIQ(+.98)Q(+.98)GQATVTVAN(+.98)GN(+.98)NRK.S	N	54.33	2750.3472	26	4.7	917.7940	3	30.50	35	1742	OB4063.raw	0	0	0	229	254	
R.IVQIEAKPN(+.98)TLVLPK.H	Y	54.24	1662.9919	15	-0.3	832.5030	2	29.39	35	1675	OB4063.raw	2.25E6	2	2	214	228	
K.SFNLDEGHALRIPSGFISYILNR.H	N	53.34	2618.3604	23	0.8	655.5979	4	36.38	35	2140	OB4063.raw	4.23E5	2	2	255	277	
K.HADADNILVIQ(+.98)Q(+.98)GQATVTVAN(+.98)GNNR.K	N	53.21	2621.2681	25	6.2	874.7687	3	30.67	35	1753	OB4063.raw	0	0	0	229	253	
K.HADADNILVIQQ(+.98)GQ(+.98)ATVTVAN(+.98)GNNRK.S	N	53.18	2749.3630	26	0.9	917.4624	3	29.40	35	1704	OB4063.raw	7.4E4	1	1	229	254	
K.DLAFPGSGEQ(+.98)VEKLIK.N	N	53.12	1730.9089	16	0.6	866.4623	2	32.33	35	1862	OB4063.raw	2.24E4	1	1	560	575	Deamidation (NQ)
K.GSEEEGDITNPIN(+.98)LREGEPDLSNNFGK.L	Y	52.83	2931.3369	27	1.4	978.1209	3	30.92	35	1769	OB4063.raw	0	0	0	387	413	
K.ISM(+15.99)PVNTPGQ(+.98)FEDFFPASSRDQSSYLQ(+.98)GFSR.N	N	52.83	3512.5830	31	2.8	879.1555	4	33.97	35	1966	OB4063.raw	0	0	0	288	318	Oxidation (M); Deamidation (NQ)
K.HADADNILVIQQ(+.98)GQ(+.98)ATVTVANGNN(+.98)R.K	N	52.71	2621.2681	25	8.0	874.7703	3	29.80	35	1714	OB4063.raw	2.91E4	1	1	229	253	Deamidation (NQ)
R.IPSGFISYILN(+.98)R.H	N	51.41	1379.7449	12	2.4	690.8813	2	35.86	35	2080	OB4063.raw	9.57E4	1	1	266	277	Deamidation (NQ)
R.VLLEENAGGE(+28.03)QEER.G	N	51.23	1599.7739	14	-0.4	800.8939	2	27.35	35	1555	OB4063.raw	5.41E3	1	1	335	348	Ethylation

Peptide	Uniq	-10lgP	Mass	Length	ppm	m/z	z	RT	Fraction	Scan	Source File	Area Digest 13 traka 60 kDa	#Feature	#Feature Digest 13 traka 60 kDa	Start	End	PTM
R.VLLEENAGGEQEERGQ(+.98)R.R	N	51.22	1913.9078	17	-0.1	638.9765	3	24.27	35	1349	OB4063.raw	1.43E5	1	1	335	351	
R.NNPFYFPSR.R	N	51.10	1140.5352	9	-0.8	571.2744	2	30.41	35	1744	OB4063.raw	2.15E5	1	1	172	180	
K.HADADNILVIQQ(+.98)GQATVTVAN(+.98)GNNR.K	N	51.07	2620.2842	25	-2.6	874.4331	3	30.38	35	1735	OB4063.raw	0	0	0	229	253	
R.EETSRN(+.98)NPFYFPSRR.F	N	50.93	1899.8864	15	-0.4	634.3025	3	27.55	35	1559	OB4063.raw	3E4	1	1	167	181	
R.NTLEAAFNAEFN(+.98)EIRR.V	N	50.87	1894.9172	16	-2.0	632.6451	3	32.34	35	1858	OB4063.raw	0	0	0	319	334	
K.HADADNILVIQQ(+.98)GQ(+.98)ATVTVAN(+.98)GN(+.98)N(+.98)RK.S	N	50.75	2751.3311	26	5.4	918.1226	3	30.12	35	1716	OB4063.raw	9.83E3	1	1	229	254	
K.GSEEEGDITNPIN(+.98)LREGEPDLSNN(+.98)FGK.L	Y	50.68	2932.3210	27	0.6	978.4482	3	31.37	35	1813	OB4063.raw	6.9E4	1	1	387	413	
R.NNPFYFPSRR.F	N	50.48	1296.6364	10	0.0	649.3254	2	28.25	35	1595	OB4063.raw	1E6	2	2	172	181	
K.AM(+15.99)VIVVVN(+.98)KGTGN(+.98)LELVAVR.K	N	49.92	2099.1660	20	8.0	700.7349	3	32.92	35	1885	OB4063.raw	4.36E3	1	1	452	471	Oxidation (M); Deamidation (NQ)
R.IPSGFISYILNRHDN(+.98)QNLR.V	N	49.71	2257.1604	19	-0.8	565.2969	4	33.29	35	1919	OB4063.raw	0	0	0	266	284	
K.ISM(+15.99)PVNTPGQ(+.98)FEDFFPASSRDQSSYLQGFSR.N	N	49.65	3511.5989	31	1.0	878.9078	4	34.14	35	1949	OB4063.raw	6.17E5	1	1	288	318	Oxidation (M)
R.IPSGFISYILN(+.98)RHDNQ(+.98)NLR.V	N	49.47	2258.1443	19	2.5	565.5447	4	33.47	35	1931	OB4063.raw	0	0	0	266	284	Deamidation (NQ)
K.ISM(+15.99)PVNTPGQFEDFFPASSRDQ(+.98)SSYLQGFSR.N	N	49.33	3511.5989	31	1.8	1171.5424	3	33.82	35	1955	OB4063.raw	0	0	0	288	318	Oxidation (M)
R.IPSGFISYILNRHDNQ(+.98)NLR.V	N	49.25	2257.1604	19	-0.2	753.3939	3	33.45	35	1950	OB4063.raw	6.13E4	1	1	266	284	
K.KGSEEEGDITNPINLREGEPDLSNNFGK.L	Y	49.15	3058.4478	28	0.4	765.6195	4	29.60	35	1686	OB4063.raw	0	0	0	386	413	
R.IFLAGDKDNVIDQIEKQAK.D	N	48.22	2144.1477	19	2.3	715.7249	3	31.31	35	1777	OB4063.raw	9.33E3	1	1	541	559	
K.HADADNILVIQ(+.98)Q(+.98)GQ(+.98)ATVTVAN(+.98)GNNR.K	N	47.99	2622.2522	25	7.5	875.0979	3	30.51	35	1753	OB4063.raw	2.28E4	1	1	229	253	Deamidation (NQ)
K.ISM(+15.99)PVNTPGQ(+.98)FEDFFPASSRDQ(+.98)SSYLQ(+.98)GFSR.N	N	46.92	3513.5669	31	0.8	879.3997	4	34.35	35	1991	OB4063.raw	8.02E6	2	2	288	318	Oxidation (M); Deamidation (NQ)
K.HADADNILVIQQGQATVTVANGNN(+.98)RK.S	N	46.77	2747.3950	26	1.1	687.8568	4	28.16	35	1594	OB4063.raw	0	0	0	229	254	
R.Q(-17.03)FQNLQNHR.I	N	45.70	1166.5581	9	-0.2	584.2862	2	26.87	35	1514	OB4063.raw	4.01E3	1	1	205	213	Pyro-glu from Q
R.SSEN(+.98)N(+.98)EGVIVK.V	Y	45.49	1176.5510	11	0.5	589.2831	2	24.08	35	1343	OB4063.raw	1.3E4	1	1	357	367	Deamidation (NQ)
K.GSEEEGDITN(+.98)PINLREGEPDLSNNFGK.L	Y	45.47	2931.3369	27	0.5	978.1201	3	31.11	35	1781	OB4063.raw	0	0	0	387	413	
K.GSEEEGDITNPIN(+.98)LREGEPDLSN(+.98)NFGK.L	Y	45.47	2932.3210	27	6.7	978.4542	3	30.99	35	1790	OB4063.raw	6.95E4	1	1	387	413	
R.NTLEAAFN(+.98)AEFN(+.98)EIRR.V	N	44.87	1895.9012	16	4.5	948.9622	2	35.87	35	2089	OB4063.raw	1.14E6	1	1	319	334	Deamidation (NQ)
K.KGSEEEGDITNPIN(+.98)LREGEPDLSNNFGK.L	Y	44.18	3059.4319	28	0.7	765.8658	4	29.94	35	1710	OB4063.raw	4.16E5	1	1	386	413	
K.S(+43.01)(+14.02)FNLDEGHALR.I	N	44.07	1314.6316	11	-0.5	658.3228	2	27.05	35	1530	OB4063.raw	9.55E3	1	1	255	265	Carbamylation; Methylation(others)
K.HADADNILVIQ(+.98)Q(+.98)GQ(+.98)ATVTVANGNNR.K	N	43.79	2621.2681	25	8.0	874.7703	3	29.80	35	1693	OB4063.raw	2.91E4	1	1	229	253	Deamidation (NQ)
R.VLLEEN(+.98)AGGEQ(+.98)EERGQ(+.98)R.R	N	43.74	1915.8759	17	-0.6	639.6322	3	25.11	35	1409	OB4063.raw	8.19E4	1	1	335	351	Deamidation (NQ)
R.YGN(+.98)QN(+.98)GRIR.V	N	43.38	1078.5155	9	-0.7	540.2646	2	20.07	35	1082	OB4063.raw	3.11E5	2	2	186	194	Deamidation (NQ)
R.N(+.98)TLEAAFNAEFNEIR.R	N	43.25	1738.8162	15	-0.3	870.4151	2	34.79	35	2053	OB4063.raw	1.46E5	1	1	319	333	Deamidation (NQ)
K.EHVEELTK.H	Y	42.61	983.4924	8	-0.5	492.7532	2	19.96	35	1113	OB4063.raw	3.18E4	2	2	371	378	
R.SSENNEGVIVKVSK.E	Y	42.61	1488.7783	14	-0.4	497.2665	3	23.50	35	1304	OB4063.raw	1.83E4	1	1	357	370	
K.ISM(+15.99)PVN(+.98)TPGQ(+.98)FEDFFPA.S	N	42.42	1913.8392	17	-0.3	957.9266	2	36.47	35	2128	OB4063.raw	5.88E4	1	1	288	304	Oxidation (M); Deamidation (NQ)
R.DQSSYLQ(+.98)GFSRN(+.98)TLEAAFN(+.98)AEFNEIRR.V	N	42.25	3165.4639	27	3.3	1056.1654	3	36.32	35	2118	OB4063.raw	0	0	0	308	334	
R.SSENNE(+28.03)GVIVK.V	Y	42.11	1202.6143	11	-0.3	602.3142	2	26.10	35	1467	OB4063.raw	7.44E3	1	1	357	367	Ethylation
N.TPGQ(+.98)FEDFFPASSRDQ(+.98)SSYLQ(+.98)GFSR.N	N	41.87	2856.2515	25	6.8	953.0975	3	33.96	35	1980	OB4063.raw	2.48E5	1	1	294	318	Deamidation (NQ)
K.KGSEEEGDITNPIN(+.98)LREGEPDLSN(+.98)NFGK.L	Y	41.78	3060.4158	28	4.0	766.1143	4	30.44	35	1739	OB4063.raw	0	0	0	386	413	
R.DQSSYLQ(+.98)GFSRN(+.98)TLEAAFNAEFNEIRR.V	N	41.74	3164.4797	27	0.0	1055.8339	3	36.20	35	2118	OB4063.raw	7.48E4	1	1	308	334	
R.Q(-17.03)FQNLQ(+.98)NHR.I	N	41.25	1167.5421	9	-0.4	584.7781	2	27.85	35	1580	OB4063.raw	1.89E4	1	1	205	213	Pyro-glu from Q

Peptide	Uniq	-10lgP	Mass	Length	ppm	m/z	z	RT	Fraction	Scan	Source File	Area Digest 13 traka 60 kDa	#Feature	#Feature Digest 13 traka 60 kDa	Start	End	PTM
K.ISMPVNTPGQ(+.98)FEDFFPASSRDQ(+.98)SSYLQGFSR.N	N	40.49	3496.5881	31	1.6	1166.5386	3	35.06	35	2021	OB4063.raw	5.34E5	1	1	288	318	Deamidation (NQ)
R.VLLEENAGGEQ(+.98)EERGQRR.W	N	39.96	2070.0090	18	-5.0	691.0068	3	24.83	35	1391	OB4063.raw	4.28E4	1	1	335	352	
K.LFEVKPDK.K	N	39.73	974.5436	8	0.2	488.2792	2	24.38	35	1358	OB4063.raw	1.2E5	2	2	414	421	
K.ISM(+15.99)PVNTPGQFEDFFPASSRDQ(+.98)SSYLQ(+.98)GFSR.N	N	39.69	3512.5830	31	5.0	1171.8741	3	33.88	35	1960	OB4063.raw	0	0	0	288	318	Oxidation (M)
K.HADADNILVIQQ(+.98)GQATVTVANGN(+.98)N(+.98)RK.S	N	39.50	2749.3630	26	6.9	688.3528	4	31.09	35	1842	OB4063.raw	5.25E4	1	1	229	254	
R.IPSGFISYILN(+.98)RHDNQ(+.98)N(+.98)LR.V	N	38.84	2259.1284	19	5.7	565.7926	4	33.53	35	2009	OB4063.raw	1.27E6	1	1	266	284	
R.IPSGFISYILN(+.98)RHDNQN(+.98)LR.V	N	38.53	2258.1443	19	-1.8	753.7207	3	33.54	35	1936	OB4063.raw	0	0	0	266	284	Deamidation (NQ)
K.ISMPVNTPGQFEDFFPASSRDQ(+.98)SSYLQGFSR.N	N	38.43	3495.6040	31	2.4	1166.2114	3	34.55	35	2004	OB4063.raw	0	0	0	288	318	
K.KGSEEEGDITNPINLREGEPDLSNN(+.98)FGK.L	Y	37.80	3059.4319	28	0.7	765.8658	4	29.94	35	1784	OB4063.raw	4.16E5	1	1	386	413	
R.YGNQ(+.98)N(+.98)GRIR.V	N	37.77	1078.5155	9	-0.7	540.2646	2	20.07	35	1094	OB4063.raw	3.11E5	2	2	186	194	
K.GSEEEGDITNPINLREGEPDLSN(+.98)NFGK.L	Y	37.53	2931.3369	27	2.4	978.1219	3	31.51	35	1806	OB4063.raw	0	0	0	387	413	
R.VLLEENAGGEQ(+.98)EERGQ(+.98)R.R	N	36.86	1914.8918	17	0.2	639.3047	3	24.64	35	1374	OB4063.raw	1.77E5	1	1	335	351	
F.NLDEGHALR.I	N	36.61	1023.5097	9	0.8	512.7625	2	26.97	35	1531	OB4063.raw	1.56E4	1	1	257	265	
R.VLLEEN(+.98)AGGEQ(+.98)EERGQR.R	N	36.15	1914.8918	17	0.2	639.3047	3	24.64	35	1398	OB4063.raw	1.77E5	1	1	335	351	
R.IPSGFISYILNRHDN(+.98)Q(+.98)NLR.V	N	36.15	2258.1443	19	0.8	565.5438	4	34.62	35	2009	OB4063.raw	0	0	0	266	284	
R.IPSGFISYILN(+.98)RHDN(+.98)QNLR.V	N	36.12	2258.1443	19	-1.6	565.5424	4	35.07	35	2038	OB4063.raw	7.93E4	1	1	266	284	
R.IPSGFISYILN(+.98)RHDNQNLR.V	N	36.08	2257.1604	19	0.6	753.3945	3	33.08	35	1904	OB4063.raw	0	0	0	266	284	
R.LKEGDVFIM(+15.99)PAA.H	N	35.74	1305.6638	12	-0.5	653.8389	2	29.47	35	1678	OB4063.raw	3.05E4	1	1	504	515	Oxidation (M)
R.N(+.98)NPFYFPSRR.F	N	35.51	1297.6204	10	0.8	649.8180	2	29.01	35	1644	OB4063.raw	4.08E4	1	1	172	181	
R.Q(-17.03)FQN(+.98)LQ(+.98)NHR.I	N	35.33	1168.5261	9	-0.3	585.2701	2	28.34	35	1609	OB4063.raw	1.07E4	1	1	205	213	Pyro-glu from Q; Deamidation (NQ)
K.Q(+.98)AKDLAFPGSGEQ(+.98)VEK.L	N	35.23	1704.8206	16	-1.9	569.2797	3	26.67	35	1500	OB4063.raw	8.39E3	1	1	557	572	Deamidation (NQ)
K.SFN(+.98)LDEGHALRIPSGFISYILNR.H	N	35.13	2619.3445	23	6.9	655.8479	4	40.04	35	2348	OB4063.raw	0	0	0	255	277	Deamidation (NQ)
K.ISM(+15.99)PVN(+.98)TPGQFEDFFPASSRDQ(+.98)SSYLQ(+.98)GFSR.N	N	35.04	3513.5669	31	5.7	1172.2029	3	34.24	35	1990	OB4063.raw	8E6	1	1	288	318	Oxidation (M); Deamidation (NQ)
K.KGSEEEGDITN(+.98)PINLREGEPDLSN(+.98)NFGK.L	Y	34.14	3060.4158	28	6.5	766.1162	4	30.54	35	1745	OB4063.raw	0	0	0	386	413	
R.VLLEENAGGEQ(+.98)EERGQR.R	N	34.10	1913.9078	17	-0.1	638.9765	3	24.27	35	1364	OB4063.raw	1.43E5	1	1	335	351	
R.IPSGFISYILN(+.98)RHDN(+.98)Q(+.98)NLR.V	N	33.82	2259.1284	19	7.5	754.0557	3	33.45	35	1950	OB4063.raw	1.33E6	2	2	266	284	Deamidation (NQ)
R.IPSGFISYILNRHDNQN(+.98)LR.V	N	33.54	2257.1604	19	-0.2	753.3939	3	33.45	35	1936	OB4063.raw	6.13E4	1	1	266	284	
R.VLLEEN(+.98)AGGEQ(+.98)EERGQRR.W	N	33.54	2070.9929	18	-1.6	691.3372	3	23.68	35	1319	OB4063.raw	3.7E4	1	1	335	352	Deamidation (NQ)
R.YGN(+.98)QNGRIR.V	N	33.50	1077.5315	9	0.9	360.1848	3	19.20	35	1038	OB4063.raw	1.04E5	1	1	186	194	
R.YGN(+.98)Q(+.98)N(+.98)GRIR.V	N	33.15	1079.4995	9	1.3	360.8409	3	20.88	35	1147	OB4063.raw	6.14E4	1	1	186	194	Deamidation (NQ)
K.E(-18.01)HVEELTK.H	Y	33.15	965.4818	8	-1.0	483.7477	2	23.89	35	1329	OB4063.raw	1.77E4	1	1	371	378	Pyro-glu from E
K.NPQ(+.98)LQ(+.98)DLDM(+15.99)M(+15.99)LTC(+57.02)VEIKEGALM(+15.99)LPHFNSK.A	N	32.90	3421.5913	29	2.5	856.4072	4	34.21	35	1981	OB4063.raw	0	0	0	423	451	Oxidation (M); Carbamidomethylation
K.ISM(+15.99)PVN(+.98)TPGQ(+.98)FEDFFPASSRDQ(+.98)SSYLQGFSR.N	N	32.83	3513.5669	31	5.7	1172.2029	3	34.24	35	2188	OB4063.raw	8E6	1	1	288	318	Oxidation (M); Deamidation (NQ)
K.LFEVKPDKK.N	N	32.65	1102.6385	9	-1.0	552.3260	2	22.20	35	1226	OB4063.raw	3.34E3	1	1	414	422	
K.NPQ(+.98)LQDLDM(+15.99)M(+15.99)LTC(+57.02)VEIKEGALM(+15.99)LPHFNSK.A	N	32.51	3420.6074	29	1.2	856.1602	4	33.80	35	1981	OB4063.raw	1.32E5	1	1	423	451	Oxidation (M); Carbamidomethylation
R.YGNQN(+.98)GRIR.V	N	32.16	1077.5315	9	0.9	360.1848	3	19.20	35	1048	OB4063.raw	1.04E5	1	1	186	194	
R.SSENN(+.98)EGVIVKVSK.E	Y	32.08	1489.7623	14	-1.9	497.5938	3	24.27	35	1361	OB4063.raw	1.23E4	1	1	357	370	
N.TPGQ(+.98)FEDFFPASSRDQSSYLQ(+.98)GFSR.N	N	31.78	2855.2673	25	-0.6	952.7625	3	33.71	35	1948	OB4063.raw	0	0	0	294	318	Deamidation (NQ)

Peptide	Uniq	-10lgP	Mass	Length	ppm	m/z	z	RT	Fraction	Scan	Source File	Area Digest 13 traka 60 kDa	#Feature	#Feature Digest 13 traka 60 kDa	Start	End	PTM
R.Q(-17.03)FQN(+.98)LQNHR.I	N	31.44	1167.5421	9	-0.4	584.7781	2	27.85	35	1554	OB4063.raw	1.89E4	1	1	205	213	Pyro-glu from Q
K.ISMPVNTPGQ(+.98)FEDFFPASSRDQ(+.98)SSYLQ(+.98)GFSR.N	N	31.28	3497.5720	31	2.6	1166.8677	3	35.17	35	2044	OB4063.raw	0	0	0	288	318	Deamidation (NQ)
R.SSE(+21.98)NNEGVIVK.V	Y	31.12	1196.5649	11	-0.4	599.2895	2	22.43	35	1243	OB4063.raw	1.34E3	1	1	357	367	
R.DQ(+.98)SSYLQ(+.98)GFSRN(+.98)TLEAAFN(+.98)AEFN(+.98)EIRR.V	N	30.59	3167.4319	27	8.2	1056.8265	3	36.57	35	2129	OB4063.raw	1.42E5	1	1	308	334	Deamidation (NQ)
K.EGALM(+15.99)LPHFNSK.A	N	30.42	1358.6653	12	-0.2	453.8956	3	29.01	35	1643	OB4063.raw	1.66E5	1	1	440	451	Oxidation (M)
K.ISM(+15.99)P(+15.99)VNTPGQ(+.98)FEDFFPASSRDQ(+.98)SSYLQ(+.98)GFSR.N	N	29.84	3529.5620	31	7.8	1177.5371	3	34.77	35	2018	OB4063.raw	0	0	0	288	318	Oxidation (M)
R.VLLEENAGGEQEERGQ(+.98)RR.W	N	29.67	2070.0090	18	-1.3	691.0094	3	23.17	35	1278	OB4063.raw	4.28E4	1	1	335	352	
R.RVLLEEN(+.98)AGGEQ(+.98)EERGQ(+.98)R.R	N	29.64	2071.9771	18	-2.9	691.6643	3	24.08	35	1347	OB4063.raw	1.44E4	1	1	334	351	Deamidation (NQ)
R.SRQ(+.98)FQ(+.98)N(+.98)LQ(+.98)NHRIVQIEAKPN(+.98)TLVLPK.H	Y	29.31	3075.6353	26	0.3	616.1345	5	30.41	35	1741	OB4063.raw	8.17E4	1	1	203	228	
R.SRQ(+.98)FQ(+.98)N(+.98)LQN(+.98)HRIVQ(+.98)IEAKPNTLVLPK.H	Y	28.93	3075.6353	26	0.3	616.1345	5	30.41	35	1715	OB4063.raw	8.17E4	1	1	203	228	Deamidation (NQ)
R.VLLEEN(+.98)AGGEQEERGQ(+.98)R.R	N	28.61	1914.8918	17	0.2	639.3047	3	24.64	35	1422	OB4063.raw	1.77E5	1	1	335	351	
K.SFNLDEGHALRIPSGFISYILNRHDNQN(+.98)LR.V	N	28.47	3496.7600	30	-0.1	700.3592	5	35.63	35	2074	OB4063.raw	1.68E5	1	1	255	284	
R.YGN(+.98)Q(+.98)NGRIR.V	N	28.16	1078.5155	9	1.7	360.5131	3	20.06	35	1088	OB4063.raw	2.55E5	1	1	186	194	
K.Q(+.98)AKDLAFPGSGEQVEK.L	N	28.09	1703.8365	16	0.7	568.9532	3	26.48	35	1483	OB4063.raw	1.25E4	1	1	557	572	Deamidation (NQ)
G.SEEEGDITNPINLREGEPDLSN(+.98)NFGK.L	Y	28.08	2874.3154	26	1.7	959.1140	3	30.99	35	1778	OB4063.raw	2.76E4	1	1	388	413	
K.SFNLDEGHALRIPSGFISYILN(+.98)RHDNQN(+.98)LR.V	N	27.94	3497.7439	30	3.3	700.5583	5	35.72	35	2074	OB4063.raw	3.15E5	1	1	255	284	
K.KGSEEEGDITNPINLREGEPDL.S	Y	27.39	2411.1453	22	1.7	804.7237	3	30.21	35	1730	OB4063.raw	1.15E4	1	1	386	407	
E.WGTPGSHVR.E	Y	26.65	995.4937	9	-1.0	332.8382	3	21.98	35	1214	OB4063.raw	2.53E4	1	1	158	166	
K.KGSEEEGDITN(+.98)PINLREGEPDLSNNFGK.L	Y	26.43	3059.4319	28	0.7	765.8658	4	29.94	35	1772	OB4063.raw	4.16E5	1	1	386	413	
R.VLLEENAGGEQEERGQR.R	N	26.31	1912.9238	17	0.6	638.6489	3	23.89	35	1337	OB4063.raw	4.66E4	1	1	335	351	
K.N(+.98)PQLQDLDM(+15.99)M(+15.99)LTC(+57.02)VEIKEGALM(+15.99)LPHFNSK.A	N	25.90	3420.6074	29	1.2	856.1602	4	33.80	35	1994	OB4063.raw	1.32E5	1	1	423	451	Oxidation (M); Carbamidomethylation
R.DQ(+.98)SSYLQGFSRN(+.98)TLEAAFN(+.98)AEFN(+.98)EIRR.V	N	25.88	3166.4478	27	5.1	1056.4952	3	36.89	35	2153	OB4063.raw	0	0	0	308	334	Deamidation (NQ)
D.ADNILVIQQ(+.98)GQATVTVANGNNRK.S	N	25.85	2424.2720	23	-0.2	809.0978	3	28.93	35	1652	OB4063.raw	8.86E3	1	1	232	254	
R.S(+28.03)SENNEGVIVK.V	Y	25.46	1202.6143	11	2.9	602.3162	2	25.05	35	1406	OB4063.raw	2.16E3	1	1	357	367	
R.DQ(+.98)SSYLQGFSRNTLEAAFNAEFN(+.98)EIRR.V	N	25.25	3164.4797	27	0.0	1055.8339	3	36.20	35	2153	OB4063.raw	7.48E4	1	1	308	334	
R.DQ(+.98)SSYLQGFSRN(+.98)TLEAAFNAEFNEIRR.V	N	25.24	3164.4797	27	2.2	792.1290	4	36.28	35	2105	OB4063.raw	2.6E5	1	1	308	334	
R.VLLEENAGGEQEERGQRR.W	N	25.15	2069.0249	18	0.5	690.6826	3	22.58	35	1271	OB4063.raw	1.8E4	1	1	335	352	
K.KGSEEEGDITNPINLREG.E	Y	24.90	1956.9388	18	-1.0	653.3196	3	27.75	35	1573	OB4063.raw	3.41E3	1	1	386	403	
R.DQ(+.98)SSYLQ(+.98)GFSRNTLEAAFNAEFN(+.98)EIRR.V	N	24.52	3165.4639	27	3.1	792.3757	4	36.40	35	2123	OB4063.raw	0	0	0	308	334	
K.HADADNILVIQ(+.98)QGQ(+.98)ATVTVAN(+.98)GNN(+.98)RK.S	N	24.44	2750.3472	26	1.8	688.5953	4	32.27	35	1854	OB4063.raw	0	0	0	229	254	
**total 185 peptides**																	

tr|Q6PSU3|Q6PSU3_ARAHY

back to list

| Protein Coverage | Supporting Peptides |

Protein Coverage:

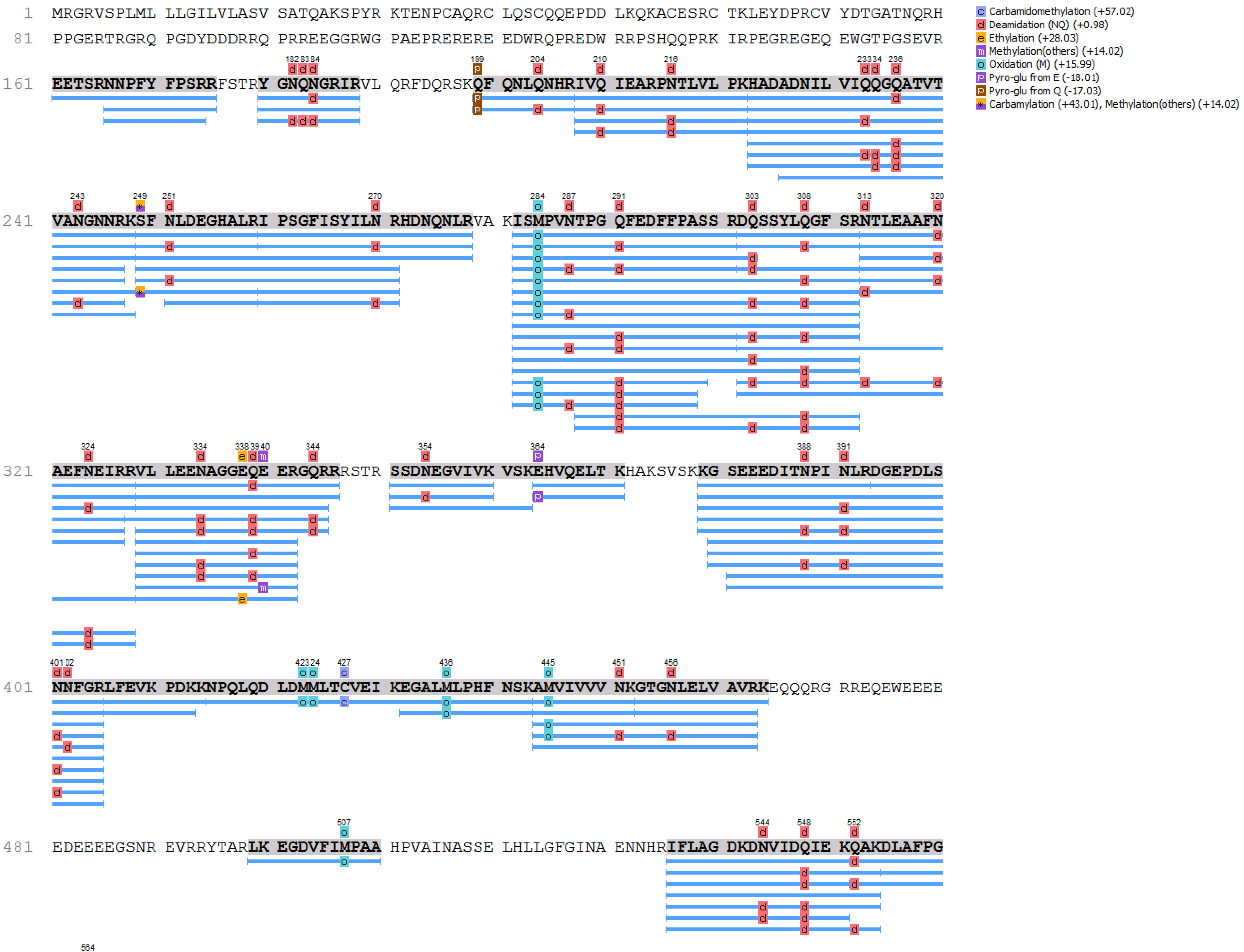
1 MRGRVSPLML LLGILVLASV SATQAKSPYR KTENPCAQRC LQSCQQEPDD LKQKACESRC TKLEYDPRCV YDTGATNQRH
81 PPGERTRGRQ PGDYDDDRRQ PRREEGGRWG PAEPRERERE EDWRQPREDW RRPSHQQPRK IRPEGREGEQ EWGTPGSEVR
161 EETSRNNPFY FPSRRFSTRY GNQNGRIRVL QRFDQRSKQF QNLQNHRIVQ IEARPNTLVL PKHADADNIL VIQQGQATVT
241 VANGNNRKSF NLDEGHALRI PSGFISYILN RHDNQNLRVA KISMPVNTPG QFEDFFPASS RDQSSYLQGF SRNTLEAAFN
321 AEFNEIRRVL LEENAGGEQE ERGQRRRSTR SSDNEGVIVK VSKEHVQELT KHAKSVSKKG SEEEDITNPI NLRDGEPDLS
401 NNFGRLFEVK PDKKNPQLQD LDMMLTCVEI KEGALMLPHF NSKAMVIVVV NKGTGNLELV AVRKEQQQRG RREQEWEEEE
481 EDEEEEGSNR EVRRYTARLK EGDVFIMPAA HPVAINASSE LHLLGFGINA ENNHRIFLAG DKDNVIDQIE KQAKDLAFPG
Carbamidomethylation (+57.02)
Deamidation (NQ) (+0.98)
Ethylation (+28.03)
Methylation(others) (+14.02)
Oxidation (M) (+15.99)
Pyro-glu from E (-18.01)
Pyro-glu from Q (-17.03)
Carbamylation (+43.01), Methylation(others) (+14.02)

Supporting Peptides:

Peptide	Uniq	-10lgP	Mass	Length	ppm	m/z	z	RT	Fraction	Scan	Source File	Area Digest 13 traka 60 kDa	#Feature	#Feature Digest 13 traka 60 kDa	Start	End	PTM
R.NTLEAAFNAEFNEIR.R	N	104.37	1737.8322	15	0.4	869.9237	2	34.24	35	1984	OB4063.raw	2.88E5	1	1	313	327	
K.ISM(+15.99)PVNTPGQFEDFFPASSR.D	N	91.29	2242.0364	20	1.0	1122.0266	2	32.92	35	1895	OB4063.raw	1.87E5	2	2	282	301	Oxidation (M)
K.ISM(+15.99)PVN(+.98)TPGQ(+.98)FEDFFPASSR.D	N	89.93	2244.0044	20	1.3	1123.0110	2	33.63	35	1944	OB4063.raw	3.04E5	2	2	282	301	Oxidation (M); Deamidation (NQ)
R.NTLEAAFN(+.98)AEFNEIR.R	N	89.50	1738.8162	15	-0.3	870.4151	2	34.79	35	2026	OB4063.raw	1.46E5	1	1	313	327	Deamidation (NQ)
K.HADADNILVIQQGQATVTVAN(+.98)GNNR.K	N	88.85	2619.3000	25	0.6	874.1078	3	29.47	35	1680	OB4063.raw	7.3E4	1	1	223	247	
K.ISM(+15.99)PVN(+.98)TPGQFEDFFPASSR.D	N	87.13	2243.0205	20	1.1	1122.5188	2	33.35	35	1914	OB4063.raw	3.87E5	1	1	282	301	Oxidation (M)
K.SFNLDEGHALR.I	N	86.49	1257.6101	11	0.6	629.8127	2	26.97	35	1516	OB4063.raw	1.94E6	2	2	249	259	
R.DQSSYLQGFSR.N	N	86.47	1286.5891	11	0.0	644.3018	2	29.29	35	1662	OB4063.raw	1.76E5	1	1	302	312	
R.DQ(+.98)SSYLQ(+.98)GFSR.N	N	84.94	1288.5571	11	-0.3	645.2856	2	30.41	35	1733	OB4063.raw	1.05E5	1	1	302	312	Deamidation (NQ)
R.IPSGFISYILNR.H	N	84.71	1378.7609	12	-0.4	690.3875	2	35.35	35	2010	OB4063.raw	1.15E6	1	1	260	271	
R.DQSSYLQ(+.98)GFSR.N	N	82.67	1287.5731	11	0.5	644.7941	2	29.84	35	1698	OB4063.raw	2.3E5	1	1	302	312	Deamidation (NQ)
K.ISM(+15.99)PVNTPGQ(+.98)FEDFFPASSR.D	N	81.23	2243.0205	20	1.1	1122.5188	2	33.35	35	2029	OB4063.raw	6.87E5	2	2	282	301	Oxidation (M); Deamidation (NQ)
K.ISMPVNTPGQ(+.98)FEDFFPASSR.D	N	81.21	2227.0254	20	0.7	1114.5208	2	34.60	35	2017	OB4063.raw	4.86E4	1	1	282	301	Deamidation (NQ)
K.HADADNILVIQ(+.98)QGQATVTVAN(+.98)GNNR.K	N	80.52	2620.2842	25	2.1	874.4371	3	30.26	35	1728	OB4063.raw	0	0	0	223	247	
K.AM(+15.99)VIVVVNKGTGNLELVAVR.K	N	79.29	2097.1980	20	0.8	700.0739	3	31.57	35	1810	OB4063.raw	3.05E5	1	1	444	463	Oxidation (M)
R.VLLEENAGGEQEER.G	N	79.16	1571.7427	14	1.1	786.8795	2	25.50	35	1436	OB4063.raw	3.06E4	1	1	329	342	
R.DQ(+.98)SSYLQGFSR.N	N	77.03	1287.5731	11	0.5	644.7941	2	29.84	35	1711	OB4063.raw	2.3E5	1	1	302	312	Deamidation (NQ)
K.HADADNILVIQQGQ(+.98)ATVTVAN(+.98)GNNRK.S	N	75.31	2748.3792	26	2.5	917.1360	3	28.64	35	1624	OB4063.raw	0	0	0	223	248	Deamidation (NQ)
R.NTLEAAFNAEFNEIRR.V	N	75.09	1893.9332	16	0.5	947.9744	2	33.09	35	1877	OB4063.raw	4.8E6	3	3	313	328	
R.IFLAGDKDNVIDQIEK.Q	N	74.42	1816.9570	16	0.1	909.4858	2	30.71	35	1760	OB4063.raw	6.31E5	2	2	536	551	
K.HADADNILVIQQGQ(+.98)ATVTVANGN(+.98)NRK.S	N	71.87	2748.3792	26	0.7	917.1343	3	29.19	35	1658	OB4063.raw	0	0	0	223	248	
R.VLLEEN(+.98)AGGEQEER.G	N	71.84	1572.7267	14	-0.1	787.3705	2	26.03	35	1466	OB4063.raw	1.83E4	1	1	329	342	Deamidation (NQ)
K.ISMPVN(+.98)TPGQ(+.98)FEDFFPASSR.D	N	71.81	2228.0095	20	1.5	1115.0137	2	35.06	35	2042	OB4063.raw	2.31E4	1	1	282	301	Deamidation (NQ)
R.IFLAGDKDNVIDQ(+.98)IEKQAK.D	N	69.30	2145.1316	19	-0.9	716.0505	3	31.66	35	1816	OB4063.raw	4.92E5	2	2	536	554	Deamidation (NQ)
K.AM(+15.99)VIVVVN(+.98)KGTGNLELVAVR.K	N	69.22	2098.1819	20	-0.3	700.4010	3	32.05	35	1835	OB4063.raw	1.92E5	1	1	444	463	Oxidation (M)
R.VLLEEN(+.98)AGGEQ(+.98)EER.G	N	68.37	1573.7107	14	-1.3	787.8616	2	26.47	35	1490	OB4063.raw	2.78E3	1	1	329	342	Deamidation (NQ)
K.AM(+15.99)VIVVVNKGTGN(+.98)LELVAVR.K	N	67.58	2098.1819	20	-0.3	700.4010	3	32.05	35	1849	OB4063.raw	1.92E5	1	1	444	463	Oxidation (M)
K.HADADNILVIQ(+.98)QGQATVTVANGNNR.K	N	67.11	2619.3000	25	0.6	874.1078	3	29.47	35	1693	OB4063.raw	7.3E4	1	1	223	247	
K.GTGNLELVAVR.K	N	66.74	1127.6299	11	-0.2	564.8221	2	28.83	35	1636	OB4063.raw	3.43E5	1	1	453	463	
R.VLLEENAGGEQ(+.98)EER.G	N	66.37	1572.7267	14	-0.1	787.3705	2	26.03	35	1454	OB4063.raw	1.83E4	1	1	329	342	Deamidation (NQ)
K.HADADNILVIQQ(+.98)GQATVTVANGNN(+.98)R.K	N	65.90	2620.2842	25	2.1	874.4371	3	30.04	35	1714	OB4063.raw	0	0	0	223	247	
R.NTLEAAFNAEFN(+.98)EIR.R	N	65.73	1738.8162	15	-0.3	870.4151	2	34.79	35	2041	OB4063.raw	1.46E5	1	1	313	327	
K.AMVIVVVNK.G	N	65.53	971.5837	9	-0.4	486.7990	2	28.34	35	1602	OB4063.raw	2.96E4	1	1	444	452	
K.GSEEEDITNPINLRDGEPDLSNNFGR.L	Y	64.97	2887.3220	26	0.7	963.4486	3	31.18	35	1782	OB4063.raw	9.64E4	1	1	380	405	
K.HADADNILVIQ(+.98)QGQ(+.98)ATVTVAN(+.98)GNNRK.S	N	64.16	2749.3630	26	0.9	917.4624	3	29.40	35	1668	OB4063.raw	7.4E4	1	1	223	248	
K.HADADNILVIQQGQATVTVANGN(+.98)NRK.S	N	64.04	2747.3950	26	-0.6	916.8051	3	29.05	35	1614	OB4063.raw	9.54E5	2	2	223	248	
K.HADADNILVIQQGQATVTVAN(+.98)GNNRK.S	N	63.48	2747.3950	26	-0.6	916.8051	3	29.05	35	1604	OB4063.raw	8.5E4	1	1	223	248	
K.GSEEEDITNPINLRDGEPDLSN(+.98)NFGR.L	Y	63.35	2888.3059	26	1.6	963.7774	3	31.38	35	1805	OB4063.raw	7.59E4	1	1	380	405	Deamidation (NQ)
R.DGEPDLSNNFGR.L	Y	63.14	1319.5742	12	0.4	660.7947	2	27.65	35	1560	OB4063.raw	9.38E3	1	1	394	405	

Peptide	Uniq	-10lgP	Mass	Length	ppm	m/z	z	RT	Fraction	Scan	Source File	Area Digest 13 traka 60 kDa	#Feature	#Feature Digest 13 traka 60 kDa	Start	End	PTM
K.HADADNILVIQQ(+.98)GQ(+.98)ATVTVANGN(+.98)NRK.S	N	62.90	2749.3630	26	0.9	917.4624	3	29.40	35	1692	OB4063.raw	7.4E4	1	1	223	248	Deamidation (NQ)
K.SFN(+.98)LDEGHALR.I	N	62.86	1258.5941	11	1.1	630.3051	2	27.44	35	1546	OB4063.raw	2.89E4	1	1	249	259	Deamidation (NQ)
R.IFLAGDKDNVIDQ(+.98)IEK.Q	N	62.82	1817.9410	16	-0.2	909.9776	2	31.18	35	1796	OB4063.raw	4.46E5	2	2	536	551	Deamidation (NQ)
R.IFLAGDKDNVIDQ(+.98)IEKQ(+.98)AK.D	N	62.39	2146.1157	19	-0.8	716.3786	3	32.23	35	1852	OB4063.raw	5.46E5	2	2	536	554	Deamidation (NQ)
R.NTLEAAFN(+.98)AEFNEIRR.V	N	62.26	1894.9172	16	0.1	948.4659	2	33.70	35	1943	OB4063.raw	8.02E5	3	3	313	328	Deamidation (NQ)
K.ISM(+15.99)PVNTPGQ(+.98)FEDFFPA.S	N	61.02	1912.8552	17	0.4	957.4353	2	36.10	35	2100	OB4063.raw	1.6E5	1	1	282	298	Oxidation (M); Deamidation (NQ)
K.AMVIVVVNKGTGNLELVAVR.K	N	60.67	2081.2031	20	-0.1	694.7416	3	33.00	35	1897	OB4063.raw	4.48E4	1	1	444	463	
K.ISM(+15.99)PVN(+.98)TPGQFEDFFPASSRDQ(+.98).S	N	60.43	2487.0898	22	-0.9	830.0365	3	33.35	35	1927	OB4063.raw	4.2E4	1	1	282	303	Oxidation (M); Deamidation (NQ)
K.AM(+15.99)VIVVVNK.G	N	60.32	987.5787	9	0.0	494.7966	2	25.83	35	1453	OB4063.raw	2.71E5	1	1	444	452	Oxidation (M)
K.HADADNILVIQQ(+.98)GQ(+.98)ATVTVAN(+.98)GNNR.K	N	57.94	2621.2681	25	5.2	874.7678	3	30.83	35	1763	OB4063.raw	0	0	0	223	247	
R.IFLAGDKDN(+.98)VIDQ(+.98)IEKQAK.D	N	57.84	2146.1157	19	-0.8	716.3786	3	32.23	35	1840	OB4063.raw	9.45E4	1	1	536	554	Deamidation (NQ)
K.HADADNILVIQ(+.98)Q(+.98)GQ(+.98)ATVTVAN(+.98)GN(+.98)NRK.S	N	57.10	2751.3311	26	5.4	918.1226	3	30.12	35	1704	OB4063.raw	9.83E3	1	1	223	248	Deamidation (NQ)
K.HADADNILVIQ(+.98)QGQ(+.98)ATVTVANGNN(+.98)RK.S	N	56.78	2749.3630	26	0.9	917.4624	3	29.40	35	1681	OB4063.raw	7.4E4	1	1	223	248	Deamidation (NQ)
K.ISM(+15.99)PVNTPGQ(+.98)FEDFFPAS.S	N	56.37	1999.8873	18	1.0	1000.9519	2	35.53	35	2072	OB4063.raw	3.74E4	1	1	282	299	Oxidation (M); Deamidation (NQ)
K.ISM(+15.99)PVNTPGQ(+.98)FEDFFPASSRDQ(+.98)SSYLQGFSR.N	N	55.84	3512.5830	31	2.1	879.1549	4	34.15	35	1978	OB4063.raw	0	0	0	282	312	Oxidation (M)
K.GTGNLELVAVRK.E	N	55.77	1255.7249	12	-0.8	628.8692	2	26.17	35	1475	OB4063.raw	1.95E4	1	1	453	464	
R.VLLEENAGGEQE(+14.02)ER.G	N	55.55	1585.7583	14	0.7	793.8870	2	26.47	35	1488	OB4063.raw	8.52E3	1	1	329	342	Methylation(others)
R.IFLAGDKDN(+.98)VIDQ(+.98)IEK.Q	N	55.37	1818.9250	16	-1.5	607.3147	3	31.57	35	1804	OB4063.raw	4.47E4	1	1	536	551	Deamidation (NQ)
K.HADADNILVIQ(+.98)Q(+.98)GQATVTVAN(+.98)GN(+.98)NRK.S	N	54.33	2750.3472	26	4.7	917.7940	3	30.50	35	1742	OB4063.raw	0	0	0	223	248	
K.GSEEEDITNPIN(+.98)LRDGEPDLSN(+.98)NFGR.L	Y	53.72	2889.2900	26	0.3	964.1042	3	31.76	35	1824	OB4063.raw	7.11E4	1	1	380	405	
K.SFNLDEGHALRIPSGFISYILNR.H	N	53.34	2618.3604	23	0.8	655.5979	4	36.38	35	2140	OB4063.raw	4.23E5	2	2	249	271	
K.HADADNILVIQ(+.98)Q(+.98)GQATVTVAN(+.98)GNNR.K	N	53.21	2621.2681	25	6.2	874.7687	3	30.67	35	1753	OB4063.raw	0	0	0	223	247	
K.HADADNILVIQQ(+.98)GQ(+.98)ATVTVAN(+.98)GNNRK.S	N	53.18	2749.3630	26	0.9	917.4624	3	29.40	35	1704	OB4063.raw	7.4E4	1	1	223	248	
K.DLAFPGSGEQ(+.98)VEKLIK.N	N	53.12	1730.9089	16	0.6	866.4623	2	32.33	35	1862	OB4063.raw	2.24E4	1	1	555	570	Deamidation (NQ)
K.ISM(+15.99)PVNTPGQ(+.98)FEDFFPASSRDQSSYLQ(+.98)GFSR.N	N	52.83	3512.5830	31	2.8	879.1555	4	33.97	35	1966	OB4063.raw	0	0	0	282	312	Oxidation (M); Deamidation (NQ)
K.HADADNILVIQQ(+.98)GQ(+.98)ATVTVANGNN(+.98)R.K	N	52.71	2621.2681	25	8.0	874.7703	3	29.80	35	1714	OB4063.raw	2.91E4	1	1	223	247	Deamidation (NQ)
R.IPSGFISYILN(+.98)R.H	N	51.41	1379.7449	12	2.4	690.8813	2	35.86	35	2080	OB4063.raw	9.57E4	1	1	260	271	Deamidation (NQ)
R.VLLEENAGGE(+28.03)QEER.G	N	51.23	1599.7739	14	-0.4	800.8939	2	27.35	35	1555	OB4063.raw	5.41E3	1	1	329	342	Ethylation
R.VLLEENAGGEQEERGQ(+.98)R.R	N	51.22	1913.9078	17	-0.1	638.9765	3	24.27	35	1349	OB4063.raw	1.43E5	1	1	329	345	
R.NNPFYFPSR.R	N	51.10	1140.5352	9	-0.8	571.2744	2	30.41	35	1744	OB4063.raw	2.15E5	1	1	166	174	
K.HADADNILVIQQ(+.98)GQATVTVAN(+.98)GNNR.K	N	51.07	2620.2842	25	-2.6	874.4331	3	30.38	35	1735	OB4063.raw	0	0	0	223	247	
R.EETSRN(+.98)NPFYFPSRR.F	N	50.93	1899.8864	15	-0.4	634.3025	3	27.55	35	1559	OB4063.raw	3E4	1	1	161	175	
R.NTLEAAFNAEFN(+.98)EIRR.V	N	50.87	1894.9172	16	-2.0	632.6451	3	32.34	35	1858	OB4063.raw	0	0	0	313	328	
K.HADADNILVIQQ(+.98)GQ(+.98)ATVTVAN(+.98)GN(+.98)N(+.98)RK.S	N	50.75	2751.3311	26	5.4	918.1226	3	30.12	35	1716	OB4063.raw	9.83E3	1	1	223	248	
R.NNPFYFPSRR.F	N	50.48	1296.6364	10	0.0	649.3254	2	28.25	35	1595	OB4063.raw	1E6	2	2	166	175	
K.AM(+15.99)VIVVVN(+.98)KGTGN(+.98)LELVAVR.K	N	49.92	2099.1660	20	8.0	700.7349	3	32.92	35	1885	OB4063.raw	4.36E3	1	1	444	463	Oxidation (M); Deamidation (NQ)
R.IPSGFISYILNRHDN(+.98)QNLR.V	N	49.71	2257.1604	19	-0.8	565.2969	4	33.29	35	1919	OB4063.raw	0	0	0	260	278	
K.ISM(+15.99)PVNTPGQ(+.98)FEDFFPASSRDQSSYLQGFSR.N	N	49.65	3511.5989	31	1.0	878.9078	4	34.14	35	1949	OB4063.raw	6.17E5	1	1	282	312	Oxidation (M)

Peptide	Uniq	-10lgP	Mass	Length	ppm	m/z	z	RT	Fraction	Scan	Source File	Area Digest 13 traka 60 kDa	#Feature	#Feature Digest 13 traka 60 kDa	Start	End	PTM
R.IPSGFISYILN(+.98)RHDNQ(+.98)NLR.V	N	49.47	2258.1443	19	2.5	565.5447	4	33.47	35	1931	OB4063.raw	0	0	0	260	278	Deamidation (NQ)
K.ISM(+15.99)PVNTPGQFEDFFPASSRDQ(+.98)SSYLQGFSR.N	N	49.33	3511.5989	31	1.8	1171.5424	3	33.82	35	1955	OB4063.raw	0	0	0	282	312	Oxidation (M)
G.SEEEDITNPINLRDGEPDLSN(+.98)NFGR.L	Y	49.27	2831.2844	25	-0.9	944.7679	3	31.48	35	1802	OB4063.raw	1.99E4	1	1	381	405	Deamidation (NQ)
R.IPSGFISYILNRHDNQ(+.98)NLR.V	N	49.25	2257.1604	19	-0.2	753.3939	3	33.45	35	1950	OB4063.raw	6.13E4	1	1	260	278	
R.IFLAGDKDNVIDQIEKQAK.D	N	48.22	2144.1477	19	2.3	715.7249	3	31.31	35	1777	OB4063.raw	9.33E3	1	1	536	554	
K.HADADNILVIQ(+.98)Q(+.98)GQ(+.98)ATVTVAN(+.98)GNNR.K	N	47.99	2622.2522	25	7.5	875.0979	3	30.51	35	1753	OB4063.raw	2.28E4	1	1	223	247	Deamidation (NQ)
R.SSDNEGVIVK.V	Y	47.01	1046.5244	10	0.1	524.2695	2	22.58	35	1248	OB4063.raw	9.14E4	1	1	351	360	
K.ISM(+15.99)PVNTPGQ(+.98)FEDFFPASSRDQ(+.98)SSYLQ(+.98)GFSR.N	N	46.92	3513.5669	31	0.8	879.3997	4	34.35	35	1991	OB4063.raw	8.02E6	2	2	282	312	Oxidation (M); Deamidation (NQ)
K.HADADNILVIQQGQATVTVANGNN(+.98)RK.S	N	46.77	2747.3950	26	1.1	687.8568	4	28.16	35	1594	OB4063.raw	0	0	0	223	248	
R.IVQIEARPNTLVLPK.H	Y	46.22	1690.0140	15	0.7	846.0149	2	29.29	35	1670	OB4063.raw	4.5E5	2	2	208	222	
K.Q(-17.03)FQNLQNHR.I	N	45.70	1166.5581	9	-0.2	584.2862	2	26.87	35	1514	OB4063.raw	4.01E3	1	1	199	207	Pyro-glu from Q
K.KGSEEEDITNPINLRDGEPDLSNNFGR.L	Y	45.15	3015.4170	27	0.8	754.8621	4	30.03	35	1712	OB4063.raw	2.92E5	1	1	379	405	
R.NTLEAAFN(+.98)AEFN(+.98)EIRR.V	N	44.87	1895.9012	16	4.5	948.9622	2	35.87	35	2089	OB4063.raw	1.14E6	1	1	313	328	Deamidation (NQ)
K.KGSEEEDITN(+.98)PIN(+.98)LRDGEPDLSNN(+.98)FGR.L	Y	44.31	3018.3689	27	4.4	1007.1347	3	30.80	35	1754	OB4063.raw	4.1E3	1	1	379	405	Deamidation (NQ)
K.S(+43.01)(+14.02)FNLDEGHALR.I	N	44.07	1314.6316	11	-0.5	658.3228	2	27.05	35	1530	OB4063.raw	9.55E3	1	1	249	259	Carbamylation; Methylation(others)
K.HADADNILVIQ(+.98)Q(+.98)GQ(+.98)ATVTVANGNNR.K	N	43.79	2621.2681	25	8.0	874.7703	3	29.80	35	1693	OB4063.raw	2.91E4	1	1	223	247	Deamidation (NQ)
R.VLLEEN(+.98)AGGEQ(+.98)EERGQ(+.98)R.R	N	43.74	1915.8759	17	-0.6	639.6322	3	25.11	35	1409	OB4063.raw	8.19E4	1	1	329	345	Deamidation (NQ)
K.KGSEEEDITNPINLRDGEPDLSNN(+.98)FGR.L	Y	43.70	3016.4009	27	1.9	755.1089	4	30.42	35	1738	OB4063.raw	0	0	0	379	405	
R.YGN(+.98)QN(+.98)GRIR.V	N	43.38	1078.5155	9	-0.7	540.2646	2	20.07	35	1082	OB4063.raw	3.11E5	2	2	180	188	Deamidation (NQ)
R.N(+.98)TLEAAFNAEFNEIR.R	N	43.25	1738.8162	15	-0.3	870.4151	2	34.79	35	2053	OB4063.raw	1.46E5	1	1	313	327	Deamidation (NQ)
K.KGSEEEDITNPIN(+.98)LRDGEPDLSNN(+.98)FGR.L	Y	43.06	3017.3850	27	7.0	755.3588	4	30.41	35	1738	OB4063.raw	2.91E5	1	1	379	405	Deamidation (NQ)
R.IVQIEARPN(+.98)TLVLPK.H	Y	43.01	1690.9980	15	-0.5	846.5059	2	29.57	35	1703	OB4063.raw	9.29E5	2	2	208	222	Deamidation (NQ)
R.SSDN(+.98)EGVIVK.V	Y	42.66	1047.5084	10	0.0	524.7615	2	23.50	35	1306	OB4063.raw	1.58E4	1	1	351	360	Deamidation (NQ)
K.KGSEEEDITN(+.98)PINLRDGEPDLSN(+.98)NFGR.L	Y	42.56	3017.3850	27	2.6	1006.8049	3	30.69	35	1754	OB4063.raw	0	0	0	379	405	Deamidation (NQ)
K.ISM(+15.99)PVN(+.98)TPGQ(+.98)FEDFFPA.S	N	42.42	1913.8392	17	-0.3	957.9266	2	36.47	35	2128	OB4063.raw	5.88E4	1	1	282	298	Oxidation (M); Deamidation (NQ)
R.DQSSYLQ(+.98)GFSRN(+.98)TLEAAFN(+.98)AEFNEIRR.V	N	42.25	3165.4639	27	3.3	1056.1654	3	36.32	35	2118	OB4063.raw	0	0	0	302	328	
R.IVQ(+.98)IEARPNTLVLPK.H	Y	42.18	1690.9980	15	0.0	564.6733	3	29.57	35	1685	OB4063.raw	9.29E5	2	2	208	222	Deamidation (NQ)
K.EHVQELTK.H	Y	42.04	982.5084	8	0.7	492.2618	2	19.02	35	1040	OB4063.raw	5.16E3	1	1	364	371	
N.TPGQ(+.98)FEDFFPASSRDQ(+.98)SSYLQ(+.98)GFSR.N	N	41.87	2856.2515	25	6.8	953.0975	3	33.96	35	1980	OB4063.raw	2.48E5	1	1	288	312	Deamidation (NQ)
R.DQSSYLQ(+.98)GFSRN(+.98)TLEAAFNAEFNEIRR.V	N	41.74	3164.4797	27	0.0	1055.8339	3	36.20	35	2118	OB4063.raw	7.48E4	1	1	302	328	
K.Q(-17.03)FQNLQ(+.98)NHR.I	N	41.25	1167.5421	9	-0.4	584.7781	2	27.85	35	1580	OB4063.raw	1.89E4	1	1	199	207	Pyro-glu from Q
K.ISMPVNTPGQ(+.98)FEDFFPASSRDQ(+.98)SSYLQGFSR.N	N	40.49	3496.5881	31	1.6	1166.5386	3	35.06	35	2021	OB4063.raw	5.34E5	1	1	282	312	Deamidation (NQ)
R.VLLEENAGGEQ(+.98)EERGQRR.R	N	39.96	2070.0090	18	-5.0	691.0068	3	24.83	35	1391	OB4063.raw	4.28E4	1	1	329	346	
R.LFEVKPDK.K	N	39.73	974.5436	8	0.2	488.2792	2	24.38	35	1358	OB4063.raw	1.2E5	2	2	406	413	
K.ISM(+15.99)PVNTPGQFEDFFPASSRDQ(+.98)SSYLQ(+.98)GFSR.N	N	39.69	3512.5830	31	5.0	1171.8741	3	33.88	35	1960	OB4063.raw	0	0	0	282	312	Oxidation (M)
K.HADADNILVIQQ(+.98)GQATVTVANGN(+.98)N(+.98)RK.S	N	39.50	2749.3630	26	6.9	688.3528	4	31.09	35	1842	OB4063.raw	5.25E4	1	1	223	248	
K.KGSEEEDITN(+.98)PIN(+.98)LRDGEPDLSNNFGR.L	Y	39.49	3017.3850	27	7.0	755.3588	4	30.41	35	1721	OB4063.raw	2.91E5	1	1	379	405	
R.IPSGFISYILN(+.98)RHDNQ(+.98)N(+.98)LR.V	N	38.84	2259.1284	19	5.7	565.7926	4	33.53	35	2009	OB4063.raw	1.27E6	1	1	260	278	
R.IPSGFISYILN(+.98)RHDNQN(+.98)LR.V	N	38.53	2258.1443	19	-1.8	753.7207	3	33.54	35	1936	OB4063.raw	0	0	0	260	278	Deamidation (NQ)
K.ISMPVNTPGQFEDFFPASSRDQ(+.98)SSYLQGFSR.N	N	38.43	3495.6040	31	2.4	1166.2114	3	34.55	35	2004	OB4063.raw	0	0	0	282	312	

Peptide	Uniq	-10lgP	Mass	Length	ppm	m/z	z	RT	Fraction	Scan	Source File	Area Digest 13 traka 60 kDa	#Feature	#Feature Digest 13 traka 60 kDa	Start	End	PTM
K.GSEEEDITN(+.98)PIN(+.98)LRDGEPDLSNN(+.98)FGR.L	Y	37.86	2890.2739	26	3.5	964.4352	3	32.00	35	1836	OB4063.raw	0	0	0	380	405	Deamidation (NQ)
R.YGNQ(+.98)N(+.98)GRIR.V	N	37.77	1078.5155	9	-0.7	540.2646	2	20.07	35	1094	OB4063.raw	3.11E5	2	2	180	188	
R.VLLEENAGGEQ(+.98)EERGQ(+.98)R.R	N	36.86	1914.8918	17	0.2	639.3047	3	24.64	35	1374	OB4063.raw	1.77E5	1	1	329	345	
K.E(-18.01)HVQELTK.H	Y	36.72	964.4978	8	-1.6	483.2554	2	23.08	35	1279	OB4063.raw	3.58E3	1	1	364	371	Pyro-glu from E
F.NLDEGHALR.I	N	36.61	1023.5097	9	0.8	512.7625	2	26.97	35	1531	OB4063.raw	1.56E4	1	1	251	259	
R.VLLEEN(+.98)AGGEQ(+.98)EERGQR.R	N	36.15	1914.8918	17	0.2	639.3047	3	24.64	35	1398	OB4063.raw	1.77E5	1	1	329	345	
R.IPSGFISYILNRHDN(+.98)Q(+.98)NLR.V	N	36.15	2258.1443	19	0.8	565.5438	4	34.62	35	2009	OB4063.raw	0	0	0	260	278	
R.IPSGFISYILN(+.98)RHDN(+.98)QNLR.V	N	36.12	2258.1443	19	-1.6	565.5424	4	35.07	35	2038	OB4063.raw	7.93E4	1	1	260	278	
R.IPSGFISYILN(+.98)RHDNQNLR.V	N	36.08	2257.1604	19	0.6	753.3945	3	33.08	35	1904	OB4063.raw	0	0	0	260	278	
R.LKEGDVFIM(+15.99)PAA.H	N	35.74	1305.6638	12	-0.5	653.8389	2	29.47	35	1678	OB4063.raw	3.05E4	1	1	499	510	Oxidation (M)
R.N(+.98)NPFYFPSRR.F	N	35.51	1297.6204	10	0.8	649.8180	2	29.01	35	1644	OB4063.raw	4.08E4	1	1	166	175	
K.KGSEEEDITNPIN(+.98)LRDGEPDLSNNFGR.L	Y	35.45	3016.4009	27	3.1	755.1099	4	31.31	35	1793	OB4063.raw	0	0	0	379	405	
K.Q(-17.03)FQN(+.98)LQ(+.98)NHR.I	N	35.33	1168.5261	9	-0.3	585.2701	2	28.34	35	1609	OB4063.raw	1.07E4	1	1	199	207	Pyro-glu from Q; Deamidation (NQ)
K.Q(+.98)AKDLAFPGSGEQ(+.98)VEK.L	N	35.23	1704.8206	16	-1.9	569.2797	3	26.67	35	1500	OB4063.raw	8.39E3	1	1	552	567	Deamidation (NQ)
K.SFN(+.98)LDEGHALRIPSGFISYILNR.H	N	35.13	2619.3445	23	6.9	655.8479	4	40.04	35	2348	OB4063.raw	0	0	0	249	271	Deamidation (NQ)
K.ISM(+15.99)PVN(+.98)TPGQFEDFFPASSRDQ(+.98)SSYLQ(+.98)GFSR.N	N	35.04	3513.5669	31	5.7	1172.2029	3	34.24	35	1990	OB4063.raw	8E6	1	1	282	312	Oxidation (M); Deamidation (NQ)
R.IVQ(+.98)IEARPN(+.98)TLVLPK.H	Y	34.74	1691.9822	15	-0.2	565.0012	3	30.12	35	1720	OB4063.raw	2.65E5	1	1	208	222	Deamidation (NQ)
R.SSDNEGVIVKVSK.E	Y	34.34	1360.7197	13	-1.4	454.5799	3	23.74	35	1322	OB4063.raw	6.99E3	1	1	351	363	
R.VLLEENAGGEQ(+.98)EERGQR.R	N	34.10	1913.9078	17	-0.1	638.9765	3	24.27	35	1364	OB4063.raw	1.43E5	1	1	329	345	
K.KGSEEEDITNPINLRDGEPDLSN(+.98)NFGR.L	Y	34.10	3016.4009	27	4.8	755.1111	4	31.93	35	1832	OB4063.raw	0	0	0	379	405	
R.IPSGFISYILN(+.98)RHDN(+.98)Q(+.98)NLR.V	N	33.82	2259.1284	19	7.5	754.0557	3	33.45	35	1950	OB4063.raw	1.33E6	2	2	260	278	Deamidation (NQ)
R.IPSGFISYILNRHDNQN(+.98)LR.V	N	33.54	2257.1604	19	-0.2	753.3939	3	33.45	35	1936	OB4063.raw	6.13E4	1	1	260	278	
R.VLLEEN(+.98)AGGEQ(+.98)EERGQRR.R	N	33.54	2070.9929	18	-1.6	691.3372	3	23.68	35	1319	OB4063.raw	3.7E4	1	1	329	346	Deamidation (NQ)
R.YGN(+.98)QNGRIR.V	N	33.50	1077.5315	9	0.9	360.1848	3	19.20	35	1038	OB4063.raw	1.04E5	1	1	180	188	
R.YGN(+.98)Q(+.98)N(+.98)GRIR.V	N	33.15	1079.4995	9	1.3	360.8409	3	20.88	35	1147	OB4063.raw	6.14E4	1	1	180	188	Deamidation (NQ)
K.NPQ(+.98)LQ(+.98)DLDM(+15.99)M(+15.99)LTC(+57.02)VEIKEGALM(+15.99)LPHFNSK.A	N	32.90	3421.5913	29	2.5	856.4072	4	34.21	35	1981	OB4063.raw	0	0	0	415	443	Oxidation (M); Carbamidomethylation
K.ISM(+15.99)PVN(+.98)TPGQ(+.98)FEDFFPASSRDQ(+.98)SSYLQGFSR.N	N	32.83	3513.5669	31	5.7	1172.2029	3	34.24	35	2188	OB4063.raw	8E6	1	1	282	312	Oxidation (M); Deamidation (NQ)
R.LFEVKPDKK.N	N	32.65	1102.6385	9	-1.0	552.3260	2	22.20	35	1226	OB4063.raw	3.34E3	1	1	406	414	
K.NPQ(+.98)LQDLDM(+15.99)M(+15.99)LTC(+57.02)VEIKEGALM(+15.99)LPHFNSK.A	N	32.51	3420.6074	29	1.2	856.1602	4	33.80	35	1981	OB4063.raw	1.32E5	1	1	415	443	Oxidation (M); Carbamidomethylation
R.YGNQN(+.98)GRIR.V	N	32.16	1077.5315	9	0.9	360.1848	3	19.20	35	1048	OB4063.raw	1.04E5	1	1	180	188	
K.KGSEEEDITNPINLR.D	Y	31.84	1713.8533	15	0.1	572.2917	3	27.95	35	1586	OB4063.raw	9.33E3	1	1	379	393	
N.TPGQ(+.98)FEDFFPASSRDQSSYLQ(+.98)GFSR.N	N	31.78	2855.2673	25	-0.6	952.7625	3	33.71	35	1948	OB4063.raw	0	0	0	288	312	Deamidation (NQ)
G.SEEEDITNPIN(+.98)LRDGEPDLSNN(+.98)FGR.L	Y	31.76	2832.2686	25	-1.4	945.0955	3	31.76	35	1802	OB4063.raw	2.16E4	1	1	381	405	
K.Q(-17.03)FQN(+.98)LQNHR.I	N	31.44	1167.5421	9	-0.4	584.7781	2	27.85	35	1554	OB4063.raw	1.89E4	1	1	199	207	Pyro-glu from Q
K.ISMPVNTPGQ(+.98)FEDFFPASSRDQ(+.98)SSYLQ(+.98)GFSR.N	N	31.28	3497.5720	31	2.6	1166.8677	3	35.17	35	2044	OB4063.raw	0	0	0	282	312	Deamidation (NQ)
R.DQ(+.98)SSYLQ(+.98)GFSRN(+.98)TLEAAFN(+.98)AEFN(+.98)EIRR.V	N	30.59	3167.4319	27	8.2	1056.8265	3	36.57	35	2129	OB4063.raw	1.42E5	1	1	302	328	Deamidation (NQ)
K.EGALM(+15.99)LPHFNSK.A	N	30.42	1358.6653	12	-0.2	453.8956	3	29.01	35	1643	OB4063.raw	1.66E5	1	1	432	443	Oxidation (M)
K.ISM(+15.99)P(+15.99)VNTPGQ(+.98)FEDFFPASSRDQ(+.98)SSYLQ(+.98)GFSR.N	N	29.84	3529.5620	31	7.8	1177.5371	3	34.77	35	2018	OB4063.raw	0	0	0	282	312	Oxidation (M)

Peptide	Uniq	-10lgP	Mass	Length	ppm	m/z	z	RT	Fraction	Scan	Source File	Area Digest 13 traka 60 kDa	#Feature	#Feature Digest 13 traka 60 kDa	Start	End	PTM
R.VLLEENAGGEQEERGQ(+.98)RR.R	N	29.67	2070.0090	18	-1.3	691.0094	3	23.17	35	1278	OB4063.raw	4.28E4	1	1	329	346	
R.RVLLEEN(+.98)AGGEQ(+.98)EERGQ(+.98)R.R	N	29.64	2071.9771	18	-2.9	691.6643	3	24.08	35	1347	OB4063.raw	1.44E4	1	1	328	345	Deamidation (NQ)
R.VLLEEN(+.98)AGGEQEERGQ(+.98)R.R	N	28.61	1914.8918	17	0.2	639.3047	3	24.64	35	1422	OB4063.raw	1.77E5	1	1	329	345	
K.SFNLDEGHALRIPSGFISYILNRHDNQN(+.98)LR.V	N	28.47	3496.7600	30	-0.1	700.3592	5	35.63	35	2074	OB4063.raw	1.68E5	1	1	249	278	
R.YGN(+.98)Q(+.98)NGRIR.V	N	28.16	1078.5155	9	1.7	360.5131	3	20.06	35	1088	OB4063.raw	2.55E5	1	1	180	188	
K.Q(+.98)AKDLAFPGSGEQVEK.L	N	28.09	1703.8365	16	0.7	568.9532	3	26.48	35	1483	OB4063.raw	1.25E4	1	1	552	567	Deamidation (NQ)
K.SFNLDEGHALRIPSGFISYILN(+.98)RHDNQN(+.98)LR.V	N	27.94	3497.7439	30	3.3	700.5583	5	35.72	35	2074	OB4063.raw	3.15E5	1	1	249	278	
R.VLLEENAGGEQEERGQR.R	N	26.31	1912.9238	17	0.6	638.6489	3	23.89	35	1337	OB4063.raw	4.66E4	1	1	329	345	
K.N(+.98)PQLQDLDM(+15.99)M(+15.99)LTC(+57.02)VEIKEGALM(+15.99)LPHFNSK.A	N	25.90	3420.6074	29	1.2	856.1602	4	33.80	35	1994	OB4063.raw	1.32E5	1	1	415	443	Oxidation (M); Carbamidomethylation
R.DQ(+.98)SSYLQGFSRN(+.98)TLEAAFN(+.98)AEFN(+.98)EIRR.V	N	25.88	3166.4478	27	5.1	1056.4952	3	36.89	35	2153	OB4063.raw	0	0	0	302	328	Deamidation (NQ)
D.ADNILVIQQ(+.98)GQATVTVANGNNRK.S	N	25.85	2424.2720	23	-0.2	809.0978	3	28.93	35	1652	OB4063.raw	8.86E3	1	1	226	248	
R.DQ(+.98)SSYLQGFSRNTLEAAFNAEFN(+.98)EIRR.V	N	25.25	3164.4797	27	0.0	1055.8339	3	36.20	35	2153	OB4063.raw	7.48E4	1	1	302	328	
R.DQ(+.98)SSYLQGFSRN(+.98)TLEAAFNAEFNEIRR.V	N	25.24	3164.4797	27	2.2	792.1290	4	36.28	35	2105	OB4063.raw	2.6E5	1	1	302	328	
R.VLLEENAGGEQEERGQRR.R	N	25.15	2069.0249	18	0.5	690.6826	3	22.58	35	1271	OB4063.raw	1.8E4	1	1	329	346	
R.DQ(+.98)SSYLQ(+.98)GFSRNTLEAAFNAEFN(+.98)EIRR.V	N	24.52	3165.4639	27	3.1	792.3757	4	36.40	35	2123	OB4063.raw	0	0	0	302	328	
K.HADADNILVIQ(+.98)QGQ(+.98)ATVTVAN(+.98)GNN(+.98)RK.S	N	24.44	2750.3472	26	1.8	688.5953	4	32.27	35	1854	OB4063.raw	0	0	0	223	248	
**total 171 peptides**																	

P43237|ALL11_ARAHY
back to list

| Protein Coverage | Supporting Peptides |

Protein Coverage:

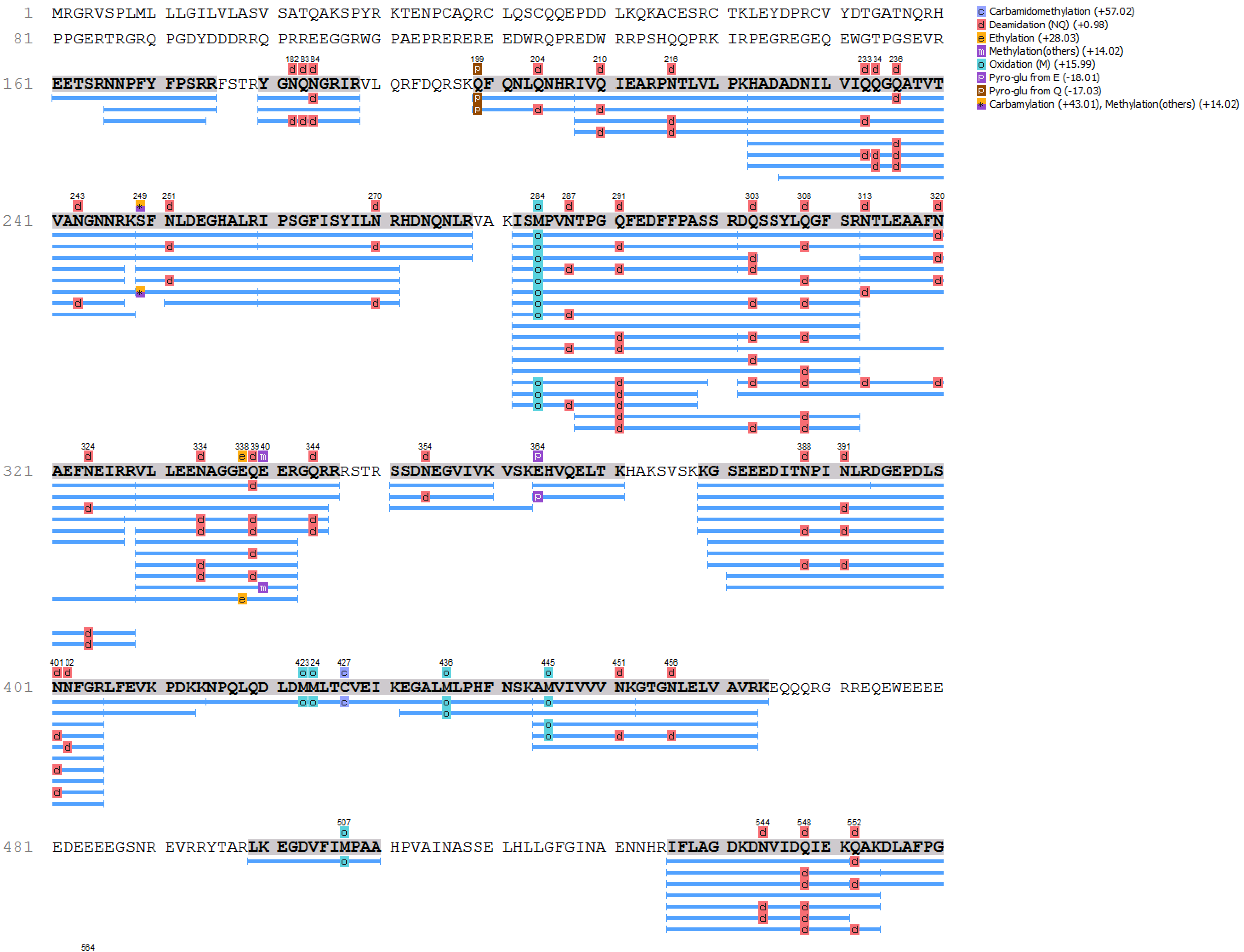
1 MRGRVSPLML LLGILVLASV SATQAKSPYR KTENPCAQRC LQSCQQEPDD LKQKACESRC TKLEYDPRCV YDTGATNQRH
81 PPGERTRGRQ PGDYDDDRRQ PRREEGGRWG PAEPRERERE EDWRQPREDW RRPSHQQPRK IRPEGREGEQ EWGTPGSEVR
161 EETSRNNPFY FPSRRFSTRY GNQNGRIRVL QRFDQRSKQF QNLQNHRIVQ IEARPNTLVL PKHADADNIL VIQQGQATVT
241 VANGNNRKSF NLDEGHALRI PSGFISYILN RHDNQNLRVA KISMPVNTPG QFEDFFPASS RDQSSYLQGF SRNTLEAAFN
321 AEFNEIRRVL LEENAGGEQE ERGQRRRSTR SSDNEGVIVK VSKEHVQELT KHAKSVSKKG SEEEDITNPI NLRDGEPDLS
401 NNFGRLFEVK PDKKNPQLQD LDMMLTCVEI KEGALMLPHF NSKAMVIVVV NKGTGNLELV AVRKEQQQRG RREQEWEEEE
481 EDEEEEGSNR EVRRYTARLK EGDVFIMPAA HPVAINASSE LHLLGFGINA ENNHRIFLAG DKDNVIDQIE KQAKDLAFPG
182 183 184 199 204 210 216 233 234 236
243 249 251 270 284 287 291 303 308 313 320
324 334 338 339 340 344 354 364 388 391
401 402 423 424 427 436 445 451 456
507 544 548 552
564
Carbamidomethylation (+57.02)
Deamidation (NQ) (+0.98)
Ethylation (+28.03)
Methylation(others) (+14.02)
Oxidation (M) (+15.99)
Pyro-glu from E (-18.01)
Pyro-glu from Q (-17.03)
Carbamylation (+43.01), Methylation(others) (+14.02)

Supporting Peptides:

Peptide	Uniq	-10lgP	Mass	Length	ppm	m/z	z	RT	Fraction	Scan	Source File	Area Digest 13 traka 60 kDa	#Feature	#Feature Digest 13 traka 60 kDa	Start	End	PTM
R.NTLEAAFNAEFNEIR.R	N	104.37	1737.8322	15	0.4	869.9237	2	34.24	35	1984	OB4063.raw	2.88E5	1	1	313	327	
K.ISM(+15.99)PVNTPGQFEDFFPASSR.D	N	91.29	2242.0364	20	1.0	1122.0266	2	32.92	35	1895	OB4063.raw	1.87E5	2	2	282	301	Oxidation (M)
K.ISM(+15.99)PVN(+.98)TPGQ(+.98)FEDFFPASSR.D	N	89.93	2244.0044	20	1.3	1123.0110	2	33.63	35	1944	OB4063.raw	3.04E5	2	2	282	301	Oxidation (M); Deamidation (NQ)
R.NTLEAAFN(+.98)AEFNEIR.R	N	89.50	1738.8162	15	-0.3	870.4151	2	34.79	35	2026	OB4063.raw	1.46E5	1	1	313	327	Deamidation (NQ)
K.HADADNILVIQQGQATVTVAN(+.98)GNNR.K	N	88.85	2619.3000	25	0.6	874.1078	3	29.47	35	1680	OB4063.raw	7.3E4	1	1	223	247	
K.ISM(+15.99)PVN(+.98)TPGQFEDFFPASSR.D	N	87.13	2243.0205	20	1.1	1122.5188	2	33.35	35	1914	OB4063.raw	3.87E5	1	1	282	301	Oxidation (M)
K.SFNLDEGHALR.I	N	86.49	1257.6101	11	0.6	629.8127	2	26.97	35	1516	OB4063.raw	1.94E6	2	2	249	259	
R.DQSSYLQGFSR.N	N	86.47	1286.5891	11	0.0	644.3018	2	29.29	35	1662	OB4063.raw	1.76E5	1	1	302	312	
R.DQ(+.98)SSYLQ(+.98)GFSR.N	N	84.94	1288.5571	11	-0.3	645.2856	2	30.41	35	1733	OB4063.raw	1.05E5	1	1	302	312	Deamidation (NQ)
R.IPSGFISYILNR.H	N	84.71	1378.7609	12	-0.4	690.3875	2	35.35	35	2010	OB4063.raw	1.15E6	1	1	260	271	
R.DQSSYLQ(+.98)GFSR.N	N	82.67	1287.5731	11	0.5	644.7941	2	29.84	35	1698	OB4063.raw	2.3E5	1	1	302	312	Deamidation (NQ)
K.ISM(+15.99)PVNTPGQ(+.98)FEDFFPASSR.D	N	81.23	2243.0205	20	1.1	1122.5188	2	33.35	35	2029	OB4063.raw	6.87E5	2	2	282	301	Oxidation (M); Deamidation (NQ)
K.ISMPVNTPGQ(+.98)FEDFFPASSR.D	N	81.21	2227.0254	20	0.7	1114.5208	2	34.60	35	2017	OB4063.raw	4.86E4	1	1	282	301	Deamidation (NQ)
K.HADADNILVIQ(+.98)QGQATVTVAN(+.98)GNNR.K	N	80.52	2620.2842	25	2.1	874.4371	3	30.26	35	1728	OB4063.raw	0	0	0	223	247	
K.AM(+15.99)VIVVVNKGTGNLELVAVR.K	N	79.29	2097.1980	20	0.8	700.0739	3	31.57	35	1810	OB4063.raw	3.05E5	1	1	444	463	Oxidation (M)
R.VLLEENAGGEQEER.G	N	79.16	1571.7427	14	1.1	786.8795	2	25.50	35	1436	OB4063.raw	3.06E4	1	1	329	342	
R.DQ(+.98)SSYLQGFSR.N	N	77.03	1287.5731	11	0.5	644.7941	2	29.84	35	1711	OB4063.raw	2.3E5	1	1	302	312	Deamidation (NQ)
K.HADADNILVIQQGQ(+.98)ATVTVAN(+.98)GNNRK.S	N	75.31	2748.3792	26	2.5	917.1360	3	28.64	35	1624	OB4063.raw	0	0	0	223	248	Deamidation (NQ)
R.NTLEAAFNAEFNEIRR.V	N	75.09	1893.9332	16	0.5	947.9744	2	33.09	35	1877	OB4063.raw	4.8E6	3	3	313	328	
R.IFLAGDKDNVIDQIEK.Q	N	74.42	1816.9570	16	0.1	909.4858	2	30.71	35	1760	OB4063.raw	6.31E5	2	2	536	551	
K.HADADNILVIQQGQ(+.98)ATVTVANGN(+.98)NRK.S	N	71.87	2748.3792	26	0.7	917.1343	3	29.19	35	1658	OB4063.raw	0	0	0	223	248	
R.VLLEEN(+.98)AGGEQEER.G	N	71.84	1572.7267	14	-0.1	787.3705	2	26.03	35	1466	OB4063.raw	1.83E4	1	1	329	342	Deamidation (NQ)
K.ISMPVN(+.98)TPGQ(+.98)FEDFFPASSR.D	N	71.81	2228.0095	20	1.5	1115.0137	2	35.06	35	2042	OB4063.raw	2.31E4	1	1	282	301	Deamidation (NQ)
R.IFLAGDKDNVIDQ(+.98)IEKQAK.D	N	69.30	2145.1316	19	-0.9	716.0505	3	31.66	35	1816	OB4063.raw	4.92E5	2	2	536	554	Deamidation (NQ)
K.AM(+15.99)VIVVVN(+.98)KGTGNLELVAVR.K	N	69.22	2098.1819	20	-0.3	700.4010	3	32.05	35	1835	OB4063.raw	1.92E5	1	1	444	463	Oxidation (M)
R.VLLEEN(+.98)AGGEQ(+.98)EER.G	N	68.37	1573.7107	14	-1.3	787.8616	2	26.47	35	1490	OB4063.raw	2.78E3	1	1	329	342	Deamidation (NQ)
K.AM(+15.99)VIVVVNKGTGN(+.98)LELVAVR.K	N	67.58	2098.1819	20	-0.3	700.4010	3	32.05	35	1849	OB4063.raw	1.92E5	1	1	444	463	Oxidation (M)
K.HADADNILVIQ(+.98)QGQATVTVANGNNR.K	N	67.11	2619.3000	25	0.6	874.1078	3	29.47	35	1693	OB4063.raw	7.3E4	1	1	223	247	
K.GTGNLELVAVR.K	N	66.74	1127.6299	11	-0.2	564.8221	2	28.83	35	1636	OB4063.raw	3.43E5	1	1	453	463	
R.VLLEENAGGEQ(+.98)EER.G	N	66.37	1572.7267	14	-0.1	787.3705	2	26.03	35	1454	OB4063.raw	1.83E4	1	1	329	342	Deamidation (NQ)
K.HADADNILVIQQ(+.98)GQATVTVANGNN(+.98)R.K	N	65.90	2620.2842	25	2.1	874.4371	3	30.04	35	1714	OB4063.raw	0	0	0	223	247	
R.NTLEAAFNAEFN(+.98)EIR.R	N	65.73	1738.8162	15	-0.3	870.4151	2	34.79	35	2041	OB4063.raw	1.46E5	1	1	313	327	
K.AMVIVVVNK.G	N	65.53	971.5837	9	-0.4	486.7990	2	28.34	35	1602	OB4063.raw	2.96E4	1	1	444	452	
K.GSEEEDITNPINLRDGEPDLSNNFGR.L	Y	64.97	2887.3220	26	0.7	963.4486	3	31.18	35	1782	OB4063.raw	9.64E4	1	1	380	405	
K.HADADNILVIQ(+.98)QGQ(+.98)ATVTVAN(+.98)GNNRK.S	N	64.16	2749.3630	26	0.9	917.4624	3	29.40	35	1668	OB4063.raw	7.4E4	1	1	223	248	
K.HADADNILVIQQGQATVTVANGN(+.98)NRK.S	N	64.04	2747.3950	26	-0.6	916.8051	3	29.05	35	1614	OB4063.raw	9.54E5	2	2	223	248	
K.HADADNILVIQQGQATVTVAN(+.98)GNNRK.S	N	63.48	2747.3950	26	-0.6	916.8051	3	29.05	35	1604	OB4063.raw	8.5E4	1	1	223	248	
K.GSEEEDITNPINLRDGEPDLSN(+.98)NFGR.L	Y	63.35	2888.3059	26	1.6	963.7774	3	31.38	35	1805	OB4063.raw	7.59E4	1	1	380	405	Deamidation (NQ)
R.DGEPDLSNNFGR.L	Y	63.14	1319.5742	12	0.4	660.7947	2	27.65	35	1560	OB4063.raw	9.38E3	1	1	394	405	

Peptide	Uniq	-10lgP	Mass	Length	ppm	m/z	z	RT	Fraction	Scan	Source File	Area Digest 13 traka 60 kDa	#Feature	#Feature Digest 13 traka 60 kDa	Start	End	PTM
K.HADADNILVIQQ(+.98)GQ(+.98)ATVTVANGN(+.98)NRK.S	N	62.90	2749.3630	26	0.9	917.4624	3	29.40	35	1692	OB4063.raw	7.4E4	1	1	223	248	Deamidation (NQ)
K.SFN(+.98)LDEGHALR.I	N	62.86	1258.5941	11	1.1	630.3051	2	27.44	35	1546	OB4063.raw	2.89E4	1	1	249	259	Deamidation (NQ)
R.IFLAGDKDNVIDQ(+.98)IEK.Q	N	62.82	1817.9410	16	-0.2	909.9776	2	31.18	35	1796	OB4063.raw	4.46E5	2	2	536	551	Deamidation (NQ)
R.IFLAGDKDNVIDQ(+.98)IEKQ(+.98)AK.D	N	62.39	2146.1157	19	-0.8	716.3786	3	32.23	35	1852	OB4063.raw	5.46E5	2	2	536	554	Deamidation (NQ)
R.NTLEAAFN(+.98)AEFNEIRR.V	N	62.26	1894.9172	16	0.1	948.4659	2	33.70	35	1943	OB4063.raw	8.02E5	3	3	313	328	Deamidation (NQ)
K.ISM(+15.99)PVNTPGQ(+.98)FEDFFPA.S	N	61.02	1912.8552	17	0.4	957.4353	2	36.10	35	2100	OB4063.raw	1.6E5	1	1	282	298	Oxidation (M); Deamidation (NQ)
K.AMVIVVVNKGTGNLELVAVR.K	N	60.67	2081.2031	20	-0.1	694.7416	3	33.00	35	1897	OB4063.raw	4.48E4	1	1	444	463	
K.ISM(+15.99)PVN(+.98)TPGQFEDFFPASSRDQ(+.98).S	N	60.43	2487.0898	22	-0.9	830.0365	3	33.35	35	1927	OB4063.raw	4.2E4	1	1	282	303	Oxidation (M); Deamidation (NQ)
K.AM(+15.99)VIVVVNK.G	N	60.32	987.5787	9	0.0	494.7966	2	25.83	35	1453	OB4063.raw	2.71E5	1	1	444	452	Oxidation (M)
K.HADADNILVIQQ(+.98)GQ(+.98)ATVTVAN(+.98)GNNR.K	N	57.94	2621.2681	25	5.2	874.7678	3	30.83	35	1763	OB4063.raw	0	0	0	223	247	
R.IFLAGDKDN(+.98)VIDQ(+.98)IEKQAK.D	N	57.84	2146.1157	19	-0.8	716.3786	3	32.23	35	1840	OB4063.raw	9.45E4	1	1	536	554	Deamidation (NQ)
K.HADADNILVIQ(+.98)Q(+.98)GQ(+.98)ATVTVAN(+.98)GN(+.98)NRK.S	N	57.10	2751.3311	26	5.4	918.1226	3	30.12	35	1704	OB4063.raw	9.83E3	1	1	223	248	Deamidation (NQ)
K.HADADNILVIQ(+.98)QGQ(+.98)ATVTVANGNN(+.98)RK.S	N	56.78	2749.3630	26	0.9	917.4624	3	29.40	35	1681	OB4063.raw	7.4E4	1	1	223	248	Deamidation (NQ)
K.ISM(+15.99)PVNTPGQ(+.98)FEDFFPAS.S	N	56.37	1999.8873	18	1.0	1000.9519	2	35.53	35	2072	OB4063.raw	3.74E4	1	1	282	299	Oxidation (M); Deamidation (NQ)
K.ISM(+15.99)PVNTPGQ(+.98)FEDFFPASSRDQ(+.98)SSYLQGFSR.N	N	55.84	3512.5830	31	2.1	879.1549	4	34.15	35	1978	OB4063.raw	0	0	0	282	312	Oxidation (M)
K.GTGNLELVAVRK.E	N	55.77	1255.7249	12	-0.8	628.8692	2	26.17	35	1475	OB4063.raw	1.95E4	1	1	453	464	
R.VLLEENAGGEQE(+14.02)ER.G	N	55.55	1585.7583	14	0.7	793.8870	2	26.47	35	1488	OB4063.raw	8.52E3	1	1	329	342	Methylation(others)
R.IFLAGDKDN(+.98)VIDQ(+.98)IEK.Q	N	55.37	1818.9250	16	-1.5	607.3147	3	31.57	35	1804	OB4063.raw	4.47E4	1	1	536	551	Deamidation (NQ)
K.HADADNILVIQ(+.98)Q(+.98)GQATVTVAN(+.98)GN(+.98)NRK.S	N	54.33	2750.3472	26	4.7	917.7940	3	30.50	35	1742	OB4063.raw	0	0	0	223	248	
K.GSEEEDITNPIN(+.98)LRDGEPDLSN(+.98)NFGR.L	Y	53.72	2889.2900	26	0.3	964.1042	3	31.76	35	1824	OB4063.raw	7.11E4	1	1	380	405	
K.SFNLDEGHALRIPSGFISYILNR.H	N	53.34	2618.3604	23	0.8	655.5979	4	36.38	35	2140	OB4063.raw	4.23E5	2	2	249	271	
K.HADADNILVIQ(+.98)Q(+.98)GQATVTVAN(+.98)GNNR.K	N	53.21	2621.2681	25	6.2	874.7687	3	30.67	35	1753	OB4063.raw	0	0	0	223	247	
K.HADADNILVIQQ(+.98)GQ(+.98)ATVTVAN(+.98)GNNRK.S	N	53.18	2749.3630	26	0.9	917.4624	3	29.40	35	1704	OB4063.raw	7.4E4	1	1	223	248	
K.DLAFPGSGEQ(+.98)VEKLIK.N	N	53.12	1730.9089	16	0.6	866.4623	2	32.33	35	1862	OB4063.raw	2.24E4	1	1	555	570	Deamidation (NQ)
K.ISM(+15.99)PVNTPGQ(+.98)FEDFFPASSRDQSSYLQ(+.98)GFSR.N	N	52.83	3512.5830	31	2.8	879.1555	4	33.97	35	1966	OB4063.raw	0	0	0	282	312	Oxidation (M); Deamidation (NQ)
K.HADADNILVIQQ(+.98)GQ(+.98)ATVTVANGNN(+.98)R.K	N	52.71	2621.2681	25	8.0	874.7703	3	29.80	35	1714	OB4063.raw	2.91E4	1	1	223	247	Deamidation (NQ)
R.IPSGFISYILN(+.98)R.H	N	51.41	1379.7449	12	2.4	690.8813	2	35.86	35	2080	OB4063.raw	9.57E4	1	1	260	271	Deamidation (NQ)
R.VLLEENAGGE(+28.03)QEER.G	N	51.23	1599.7739	14	-0.4	800.8939	2	27.35	35	1555	OB4063.raw	5.41E3	1	1	329	342	Ethylation
R.VLLEENAGGEQEERGQ(+.98)R.R	N	51.22	1913.9078	17	-0.1	638.9765	3	24.27	35	1349	OB4063.raw	1.43E5	1	1	329	345	
R.NNPFYFPSR.R	N	51.10	1140.5352	9	-0.8	571.2744	2	30.41	35	1744	OB4063.raw	2.15E5	1	1	166	174	
K.HADADNILVIQQ(+.98)GQATVTVAN(+.98)GNNR.K	N	51.07	2620.2842	25	-2.6	874.4331	3	30.38	35	1735	OB4063.raw	0	0	0	223	247	
R.EETSRN(+.98)NPFYFPSRR.F	N	50.93	1899.8864	15	-0.4	634.3025	3	27.55	35	1559	OB4063.raw	3E4	1	1	161	175	
R.NTLEAAFNAEFN(+.98)EIRR.V	N	50.87	1894.9172	16	-2.0	632.6451	3	32.34	35	1858	OB4063.raw	0	0	0	313	328	
K.HADADNILVIQQ(+.98)GQ(+.98)ATVTVAN(+.98)GN(+.98)N(+.98)RK.S	N	50.75	2751.3311	26	5.4	918.1226	3	30.12	35	1716	OB4063.raw	9.83E3	1	1	223	248	
R.NNPFYFPSRR.F	N	50.48	1296.6364	10	0.0	649.3254	2	28.25	35	1595	OB4063.raw	1E6	2	2	166	175	
K.AM(+15.99)VIVVVN(+.98)KGTGN(+.98)LELVAVR.K	N	49.92	2099.1660	20	8.0	700.7349	3	32.92	35	1885	OB4063.raw	4.36E3	1	1	444	463	Oxidation (M); Deamidation (NQ)
R.IPSGFISYILNRHDN(+.98)QNLR.V	N	49.71	2257.1604	19	-0.8	565.2969	4	33.29	35	1919	OB4063.raw	0	0	0	260	278	
K.ISM(+15.99)PVNTPGQ(+.98)FEDFFPASSRDQSSYLQGFSR.N	N	49.65	3511.5989	31	1.0	878.9078	4	34.14	35	1949	OB4063.raw	6.17E5	1	1	282	312	Oxidation (M)

Peptide	Uniq	-10lgP	Mass	Length	ppm	m/z	z	RT	Fraction	Scan	Source File	Area Digest 13 traka 60 kDa	#Feature	#Feature Digest 13 traka 60 kDa	Start	End	PTM
R.IPSGFISYILN(+.98)RHDNQ(+.98)NLR.V	N	49.47	2258.1443	19	2.5	565.5447	4	33.47	35	1931	OB4063.raw	0	0	0	260	278	Deamidation (NQ)
K.ISM(+15.99)PVNTPGQFEDFFPASSRDQ(+.98)SSYLQGFSR.N	N	49.33	3511.5989	31	1.8	1171.5424	3	33.82	35	1955	OB4063.raw	0	0	0	282	312	Oxidation (M)
G.SEEEDITNPINLRDGEPDLSN(+.98)NFGR.L	Y	49.27	2831.2844	25	-0.9	944.7679	3	31.48	35	1802	OB4063.raw	1.99E4	1	1	381	405	Deamidation (NQ)
R.IPSGFISYILNRHDNQ(+.98)NLR.V	N	49.25	2257.1604	19	-0.2	753.3939	3	33.45	35	1950	OB4063.raw	6.13E4	1	1	260	278	
R.IFLAGDKDNVIDQIEKQAK.D	N	48.22	2144.1477	19	2.3	715.7249	3	31.31	35	1777	OB4063.raw	9.33E3	1	1	536	554	
K.HADADNILVIQ(+.98)Q(+.98)GQ(+.98)ATVTVAN(+.98)GNNR.K	N	47.99	2622.2522	25	7.5	875.0979	3	30.51	35	1753	OB4063.raw	2.28E4	1	1	223	247	Deamidation (NQ)
R.SSDNEGVIVK.V	Y	47.01	1046.5244	10	0.1	524.2695	2	22.58	35	1248	OB4063.raw	9.14E4	1	1	351	360	
K.ISM(+15.99)PVNTPGQ(+.98)FEDFFPASSRDQ(+.98)SSYLQ(+.98)GFSR.N	N	46.92	3513.5669	31	0.8	879.3997	4	34.35	35	1991	OB4063.raw	8.02E6	2	2	282	312	Oxidation (M); Deamidation (NQ)
K.HADADNILVIQQGQATVTVANGNN(+.98)RK.S	N	46.77	2747.3950	26	1.1	687.8568	4	28.16	35	1594	OB4063.raw	0	0	0	223	248	
R.IVQIEARPNTLVLPK.H	Y	46.22	1690.0140	15	0.7	846.0149	2	29.29	35	1670	OB4063.raw	4.5E5	2	2	208	222	
K.Q(-17.03)FQNLQNHR.I	N	45.70	1166.5581	9	-0.2	584.2862	2	26.87	35	1514	OB4063.raw	4.01E3	1	1	199	207	Pyro-glu from Q
K.KGSEEEDITNPINLRDGEPDLSNNFGR.L	Y	45.15	3015.4170	27	0.8	754.8621	4	30.03	35	1712	OB4063.raw	2.92E5	1	1	379	405	
R.NTLEAAFN(+.98)AEFN(+.98)EIRR.V	N	44.87	1895.9012	16	4.5	948.9622	2	35.87	35	2089	OB4063.raw	1.14E6	1	1	313	328	Deamidation (NQ)
K.KGSEEEDITN(+.98)PIN(+.98)LRDGEPDLSNN(+.98)FGR.L	Y	44.31	3018.3689	27	4.4	1007.1347	3	30.80	35	1754	OB4063.raw	4.1E3	1	1	379	405	Deamidation (NQ)
K.S(+43.01)(+14.02)FNLDEGHALR.I	N	44.07	1314.6316	11	-0.5	658.3228	2	27.05	35	1530	OB4063.raw	9.55E3	1	1	249	259	Carbamylation; Methylation(others)
K.HADADNILVIQ(+.98)Q(+.98)GQ(+.98)ATVTVANGNNR.K	N	43.79	2621.2681	25	8.0	874.7703	3	29.80	35	1693	OB4063.raw	2.91E4	1	1	223	247	Deamidation (NQ)
R.VLLEEN(+.98)AGGEQ(+.98)EERGQ(+.98)R.R	N	43.74	1915.8759	17	-0.6	639.6322	3	25.11	35	1409	OB4063.raw	8.19E4	1	1	329	345	Deamidation (NQ)
K.KGSEEEDITNPINLRDGEPDLSNN(+.98)FGR.L	Y	43.70	3016.4009	27	1.9	755.1089	4	30.42	35	1738	OB4063.raw	0	0	0	379	405	
R.YGN(+.98)QN(+.98)GRIR.V	N	43.38	1078.5155	9	-0.7	540.2646	2	20.07	35	1082	OB4063.raw	3.11E5	2	2	180	188	Deamidation (NQ)
R.N(+.98)TLEAAFNAEFNEIR.R	N	43.25	1738.8162	15	-0.3	870.4151	2	34.79	35	2053	OB4063.raw	1.46E5	1	1	313	327	Deamidation (NQ)
K.KGSEEEDITNPIN(+.98)LRDGEPDLSNN(+.98)FGR.L	Y	43.06	3017.3850	27	7.0	755.3588	4	30.41	35	1738	OB4063.raw	2.91E5	1	1	379	405	Deamidation (NQ)
R.IVQIEARPN(+.98)TLVLPK.H	Y	43.01	1690.9980	15	-0.5	846.5059	2	29.57	35	1703	OB4063.raw	9.29E5	2	2	208	222	Deamidation (NQ)
R.SSDN(+.98)EGVIVK.V	Y	42.66	1047.5084	10	0.0	524.7615	2	23.50	35	1306	OB4063.raw	1.58E4	1	1	351	360	Deamidation (NQ)
K.KGSEEEDITN(+.98)PINLRDGEPDLSN(+.98)NFGR.L	Y	42.56	3017.3850	27	2.6	1006.8049	3	30.69	35	1754	OB4063.raw	0	0	0	379	405	Deamidation (NQ)
K.ISM(+15.99)PVN(+.98)TPGQ(+.98)FEDFFPA.S	N	42.42	1913.8392	17	-0.3	957.9266	2	36.47	35	2128	OB4063.raw	5.88E4	1	1	282	298	Oxidation (M); Deamidation (NQ)
R.DQSSYLQ(+.98)GFSRN(+.98)TLEAAFN(+.98)AEFNEIRR.V	N	42.25	3165.4639	27	3.3	1056.1654	3	36.32	35	2118	OB4063.raw	0	0	0	302	328	
R.IVQ(+.98)IEARPNTLVLPK.H	Y	42.18	1690.9980	15	0.0	564.6733	3	29.57	35	1685	OB4063.raw	9.29E5	2	2	208	222	Deamidation (NQ)
K.EHVQELTK.H	Y	42.04	982.5084	8	0.7	492.2618	2	19.02	35	1040	OB4063.raw	5.16E3	1	1	364	371	
N.TPGQ(+.98)FEDFFPASSRDQ(+.98)SSYLQ(+.98)GFSR.N	N	41.87	2856.2515	25	6.8	953.0975	3	33.96	35	1980	OB4063.raw	2.48E5	1	1	288	312	Deamidation (NQ)
R.DQSSYLQ(+.98)GFSRN(+.98)TLEAAFNAEFNEIRR.V	N	41.74	3164.4797	27	0.0	1055.8339	3	36.20	35	2118	OB4063.raw	7.48E4	1	1	302	328	
K.Q(-17.03)FQNLQ(+.98)NHR.I	N	41.25	1167.5421	9	-0.4	584.7781	2	27.85	35	1580	OB4063.raw	1.89E4	1	1	199	207	Pyro-glu from Q
K.ISMPVNTPGQ(+.98)FEDFFPASSRDQ(+.98)SSYLQGFSR.N	N	40.49	3496.5881	31	1.6	1166.5386	3	35.06	35	2021	OB4063.raw	5.34E5	1	1	282	312	Deamidation (NQ)
R.VLLEENAGGEQ(+.98)EERGQRR.R	N	39.96	2070.0090	18	-5.0	691.0068	3	24.83	35	1391	OB4063.raw	4.28E4	1	1	329	346	
R.LFEVKPDK.K	N	39.73	974.5436	8	0.2	488.2792	2	24.38	35	1358	OB4063.raw	1.2E5	2	2	406	413	
K.ISM(+15.99)PVNTPGQFEDFFPASSRDQ(+.98)SSYLQ(+.98)GFSR.N	N	39.69	3512.5830	31	5.0	1171.8741	3	33.88	35	1960	OB4063.raw	0	0	0	282	312	Oxidation (M)
K.HADADNILVIQQ(+.98)GQATVTVANGN(+.98)N(+.98)RK.S	N	39.50	2749.3630	26	6.9	688.3528	4	31.09	35	1842	OB4063.raw	5.25E4	1	1	223	248	
K.KGSEEEDITN(+.98)PIN(+.98)LRDGEPDLSNNFGR.L	Y	39.49	3017.3850	27	7.0	755.3588	4	30.41	35	1721	OB4063.raw	2.91E5	1	1	379	405	
R.IPSGFISYILN(+.98)RHDNQ(+.98)N(+.98)LR.V	N	38.84	2259.1284	19	5.7	565.7926	4	33.53	35	2009	OB4063.raw	1.27E6	1	1	260	278	
R.IPSGFISYILN(+.98)RHDNQN(+.98)LR.V	N	38.53	2258.1443	19	-1.8	753.7207	3	33.54	35	1936	OB4063.raw	0	0	0	260	278	Deamidation (NQ)
K.ISMPVNTPGQFEDFFPASSRDQ(+.98)SSYLQGFSR.N	N	38.43	3495.6040	31	2.4	1166.2114	3	34.55	35	2004	OB4063.raw	0	0	0	282	312	

Peptide	Uniq	-10lgP	Mass	Length	ppm	m/z	z	RT	Fraction	Scan	Source File	Area Digest 13 traka 60 kDa	#Feature	#Feature Digest 13 traka 60 kDa	Start	End	PTM
K.GSEEEDITN(+.98)PIN(+.98)LRDGEPDLSNN(+.98)FGR.L	Y	37.86	2890.2739	26	3.5	964.4352	3	32.00	35	1836	OB4063.raw	0	0	0	380	405	Deamidation (NQ)
R.YGNQ(+.98)N(+.98)GRIR.V	N	37.77	1078.5155	9	-0.7	540.2646	2	20.07	35	1094	OB4063.raw	3.11E5	2	2	180	188	
R.VLLEENAGGEQ(+.98)EERGQ(+.98)R.R	N	36.86	1914.8918	17	0.2	639.3047	3	24.64	35	1374	OB4063.raw	1.77E5	1	1	329	345	
K.E(-18.01)HVQELTK.H	Y	36.72	964.4978	8	-1.6	483.2554	2	23.08	35	1279	OB4063.raw	3.58E3	1	1	364	371	Pyro-glu from E
F.NLDEGHALR.I	N	36.61	1023.5097	9	0.8	512.7625	2	26.97	35	1531	OB4063.raw	1.56E4	1	1	251	259	
R.VLLEEN(+.98)AGGEQ(+.98)EERGQR.R	N	36.15	1914.8918	17	0.2	639.3047	3	24.64	35	1398	OB4063.raw	1.77E5	1	1	329	345	
R.IPSGFISYILNRHDN(+.98)Q(+.98)NLR.V	N	36.15	2258.1443	19	0.8	565.5438	4	34.62	35	2009	OB4063.raw	0	0	0	260	278	
R.IPSGFISYILN(+.98)RHDN(+.98)QNLR.V	N	36.12	2258.1443	19	-1.6	565.5424	4	35.07	35	2038	OB4063.raw	7.93E4	1	1	260	278	
R.IPSGFISYILN(+.98)RHDNQNLR.V	N	36.08	2257.1604	19	0.6	753.3945	3	33.08	35	1904	OB4063.raw	0	0	0	260	278	
R.LKEGDVFIM(+15.99)PAA.H	N	35.74	1305.6638	12	-0.5	653.8389	2	29.47	35	1678	OB4063.raw	3.05E4	1	1	499	510	Oxidation (M)
R.N(+.98)NPFYFPSRR.F	N	35.51	1297.6204	10	0.8	649.8180	2	29.01	35	1644	OB4063.raw	4.08E4	1	1	166	175	
K.KGSEEEDITNPIN(+.98)LRDGEPDLSNNFGR.L	Y	35.45	3016.4009	27	3.1	755.1099	4	31.31	35	1793	OB4063.raw	0	0	0	379	405	
K.Q(-17.03)FQN(+.98)LQ(+.98)NHR.I	N	35.33	1168.5261	9	-0.3	585.2701	2	28.34	35	1609	OB4063.raw	1.07E4	1	1	199	207	Pyro-glu from Q; Deamidation (NQ)
K.Q(+.98)AKDLAFPGSGEQ(+.98)VEK.L	N	35.23	1704.8206	16	-1.9	569.2797	3	26.67	35	1500	OB4063.raw	8.39E3	1	1	552	567	Deamidation (NQ)
K.SFN(+.98)LDEGHALRIPSGFISYILNR.H	N	35.13	2619.3445	23	6.9	655.8479	4	40.04	35	2348	OB4063.raw	0	0	0	249	271	Deamidation (NQ)
K.ISM(+15.99)PVN(+.98)TPGQFEDFFPASSRDQ(+.98)SSYLQ(+.98)GFSR.N	N	35.04	3513.5669	31	5.7	1172.2029	3	34.24	35	1990	OB4063.raw	8E6	1	1	282	312	Oxidation (M); Deamidation (NQ)
R.IVQ(+.98)IEARPN(+.98)TLVLPK.H	Y	34.74	1691.9822	15	-0.2	565.0012	3	30.12	35	1720	OB4063.raw	2.65E5	1	1	208	222	Deamidation (NQ)
R.SSDNEGVIVKVSK.E	Y	34.34	1360.7197	13	-1.4	454.5799	3	23.74	35	1322	OB4063.raw	6.99E3	1	1	351	363	
R.VLLEENAGGEQ(+.98)EERGQR.R	N	34.10	1913.9078	17	-0.1	638.9765	3	24.27	35	1364	OB4063.raw	1.43E5	1	1	329	345	
K.KGSEEEDITNPINLRDGEPDLSN(+.98)NFGR.L	Y	34.10	3016.4009	27	4.8	755.1111	4	31.93	35	1832	OB4063.raw	0	0	0	379	405	
R.IPSGFISYILN(+.98)RHDN(+.98)Q(+.98)NLR.V	N	33.82	2259.1284	19	7.5	754.0557	3	33.45	35	1950	OB4063.raw	1.33E6	2	2	260	278	Deamidation (NQ)
R.IPSGFISYILNRHDNQN(+.98)LR.V	N	33.54	2257.1604	19	-0.2	753.3939	3	33.45	35	1936	OB4063.raw	6.13E4	1	1	260	278	
R.VLLEEN(+.98)AGGEQ(+.98)EERGQRR.R	N	33.54	2070.9929	18	-1.6	691.3372	3	23.68	35	1319	OB4063.raw	3.7E4	1	1	329	346	Deamidation (NQ)
R.YGN(+.98)QNGRIR.V	N	33.50	1077.5315	9	0.9	360.1848	3	19.20	35	1038	OB4063.raw	1.04E5	1	1	180	188	
R.YGN(+.98)Q(+.98)N(+.98)GRIR.V	N	33.15	1079.4995	9	1.3	360.8409	3	20.88	35	1147	OB4063.raw	6.14E4	1	1	180	188	Deamidation (NQ)
K.NPQ(+.98)LQ(+.98)DLDM(+15.99)M(+15.99)LTC(+57.02)VEIKEGALM(+15.99)LPHFNSK.A	N	32.90	3421.5913	29	2.5	856.4072	4	34.21	35	1981	OB4063.raw	0	0	0	415	443	Oxidation (M); Carbamidomethylation
K.ISM(+15.99)PVN(+.98)TPGQ(+.98)FEDFFPASSRDQ(+.98)SSYLQGFSR.N	N	32.83	3513.5669	31	5.7	1172.2029	3	34.24	35	2188	OB4063.raw	8E6	1	1	282	312	Oxidation (M); Deamidation (NQ)
R.LFEVKPDKK.N	N	32.65	1102.6385	9	-1.0	552.3260	2	22.20	35	1226	OB4063.raw	3.34E3	1	1	406	414	
K.NPQ(+.98)LQDLDM(+15.99)M(+15.99)LTC(+57.02)VEIKEGALM(+15.99)LPHFNSK.A	N	32.51	3420.6074	29	1.2	856.1602	4	33.80	35	1981	OB4063.raw	1.32E5	1	1	415	443	Oxidation (M); Carbamidomethylation
R.YGNQN(+.98)GRIR.V	N	32.16	1077.5315	9	0.9	360.1848	3	19.20	35	1048	OB4063.raw	1.04E5	1	1	180	188	
K.KGSEEEDITNPINLR.D	Y	31.84	1713.8533	15	0.1	572.2917	3	27.95	35	1586	OB4063.raw	9.33E3	1	1	379	393	
N.TPGQ(+.98)FEDFFPASSRDQSSYLQ(+.98)GFSR.N	N	31.78	2855.2673	25	-0.6	952.7625	3	33.71	35	1948	OB4063.raw	0	0	0	288	312	Deamidation (NQ)
G.SEEEDITNPIN(+.98)LRDGEPDLSNN(+.98)FGR.L	Y	31.76	2832.2686	25	-1.4	945.0955	3	31.76	35	1802	OB4063.raw	2.16E4	1	1	381	405	
K.Q(-17.03)FQN(+.98)LQNHR.I	N	31.44	1167.5421	9	-0.4	584.7781	2	27.85	35	1554	OB4063.raw	1.89E4	1	1	199	207	Pyro-glu from Q
K.ISMPVNTPGQ(+.98)FEDFFPASSRDQ(+.98)SSYLQ(+.98)GFSR.N	N	31.28	3497.5720	31	2.6	1166.8677	3	35.17	35	2044	OB4063.raw	0	0	0	282	312	Deamidation (NQ)
R.DQ(+.98)SSYLQ(+.98)GFSRN(+.98)TLEAAFN(+.98)AEFN(+.98)EIRR.V	N	30.59	3167.4319	27	8.2	1056.8265	3	36.57	35	2129	OB4063.raw	1.42E5	1	1	302	328	Deamidation (NQ)
K.EGALM(+15.99)LPHFNSK.A	N	30.42	1358.6653	12	-0.2	453.8956	3	29.01	35	1643	OB4063.raw	1.66E5	1	1	432	443	Oxidation (M)
K.ISM(+15.99)P(+15.99)VNTPGQ(+.98)FEDFFPASSRDQ(+.98)SSYLQ(+.98)GFSR.N	N	29.84	3529.5620	31	7.8	1177.5371	3	34.77	35	2018	OB4063.raw	0	0	0	282	312	Oxidation (M)

Peptide	Uniq	-10lgP	Mass	Length	ppm	m/z	z	RT	Fraction	Scan	Source File	Area Digest 13 traka 60 kDa	#Feature	#Feature Digest 13 traka 60 kDa	Start	End	PTM
R.VLLEENAGGEQEERGQ(+.98)RR.R	N	29.67	2070.0090	18	-1.3	691.0094	3	23.17	35	1278	OB4063.raw	4.28E4	1	1	329	346	
R.RVLLEEN(+.98)AGGEQ(+.98)EERGQ(+.98)R.R	N	29.64	2071.9771	18	-2.9	691.6643	3	24.08	35	1347	OB4063.raw	1.44E4	1	1	328	345	Deamidation (NQ)
R.VLLEEN(+.98)AGGEQEERGQ(+.98)R.R	N	28.61	1914.8918	17	0.2	639.3047	3	24.64	35	1422	OB4063.raw	1.77E5	1	1	329	345	
K.SFNLDEGHALRIPSGFISYILNRHDNQN(+.98)LR.V	N	28.47	3496.7600	30	-0.1	700.3592	5	35.63	35	2074	OB4063.raw	1.68E5	1	1	249	278	
R.YGN(+.98)Q(+.98)NGRIR.V	N	28.16	1078.5155	9	1.7	360.5131	3	20.06	35	1088	OB4063.raw	2.55E5	1	1	180	188	
K.Q(+.98)AKDLAFPGSGEQVEK.L	N	28.09	1703.8365	16	0.7	568.9532	3	26.48	35	1483	OB4063.raw	1.25E4	1	1	552	567	Deamidation (NQ)
K.SFNLDEGHALRIPSGFISYILN(+.98)RHDNQN(+.98)LR.V	N	27.94	3497.7439	30	3.3	700.5583	5	35.72	35	2074	OB4063.raw	3.15E5	1	1	249	278	
R.VLLEENAGGEQEERGQR.R	N	26.31	1912.9238	17	0.6	638.6489	3	23.89	35	1337	OB4063.raw	4.66E4	1	1	329	345	
K.N(+.98)PQLQDLDM(+15.99)M(+15.99)LTC(+57.02)VEIKEGALM(+15.99)LPHFNSK.A	N	25.90	3420.6074	29	1.2	856.1602	4	33.80	35	1994	OB4063.raw	1.32E5	1	1	415	443	Oxidation (M); Carbamidomethylation
R.DQ(+.98)SSYLQGFSRN(+.98)TLEAAFN(+.98)AEFN(+.98)EIRR.V	N	25.88	3166.4478	27	5.1	1056.4952	3	36.89	35	2153	OB4063.raw	0	0	0	302	328	Deamidation (NQ)
D.ADNILVIQQ(+.98)GQATVTVANGNNRK.S	N	25.85	2424.2720	23	-0.2	809.0978	3	28.93	35	1652	OB4063.raw	8.86E3	1	1	226	248	
R.DQ(+.98)SSYLQGFSRNTLEAAFNAEFN(+.98)EIRR.V	N	25.25	3164.4797	27	0.0	1055.8339	3	36.20	35	2153	OB4063.raw	7.48E4	1	1	302	328	
R.DQ(+.98)SSYLQGFSRN(+.98)TLEAAFNAEFNEIRR.V	N	25.24	3164.4797	27	2.2	792.1290	4	36.28	35	2105	OB4063.raw	2.6E5	1	1	302	328	
R.VLLEENAGGEQEERGQRR.R	N	25.15	2069.0249	18	0.5	690.6826	3	22.58	35	1271	OB4063.raw	1.8E4	1	1	329	346	
R.DQ(+.98)SSYLQ(+.98)GFSRNTLEAAFNAEFN(+.98)EIRR.V	N	24.52	3165.4639	27	3.1	792.3757	4	36.40	35	2123	OB4063.raw	0	0	0	302	328	
K.HADADNILVIQ(+.98)QGQ(+.98)ATVTVAN(+.98)GNN(+.98)RK.S	N	24.44	2750.3472	26	1.8	688.5953	4	32.27	35	1854	OB4063.raw	0	0	0	223	248	
**total 171 peptides**																	

tr|B3IXL2|B3IXL2_ARAHY

back to list

| Protein Coverage | Supporting Peptides |

Protein Coverage:

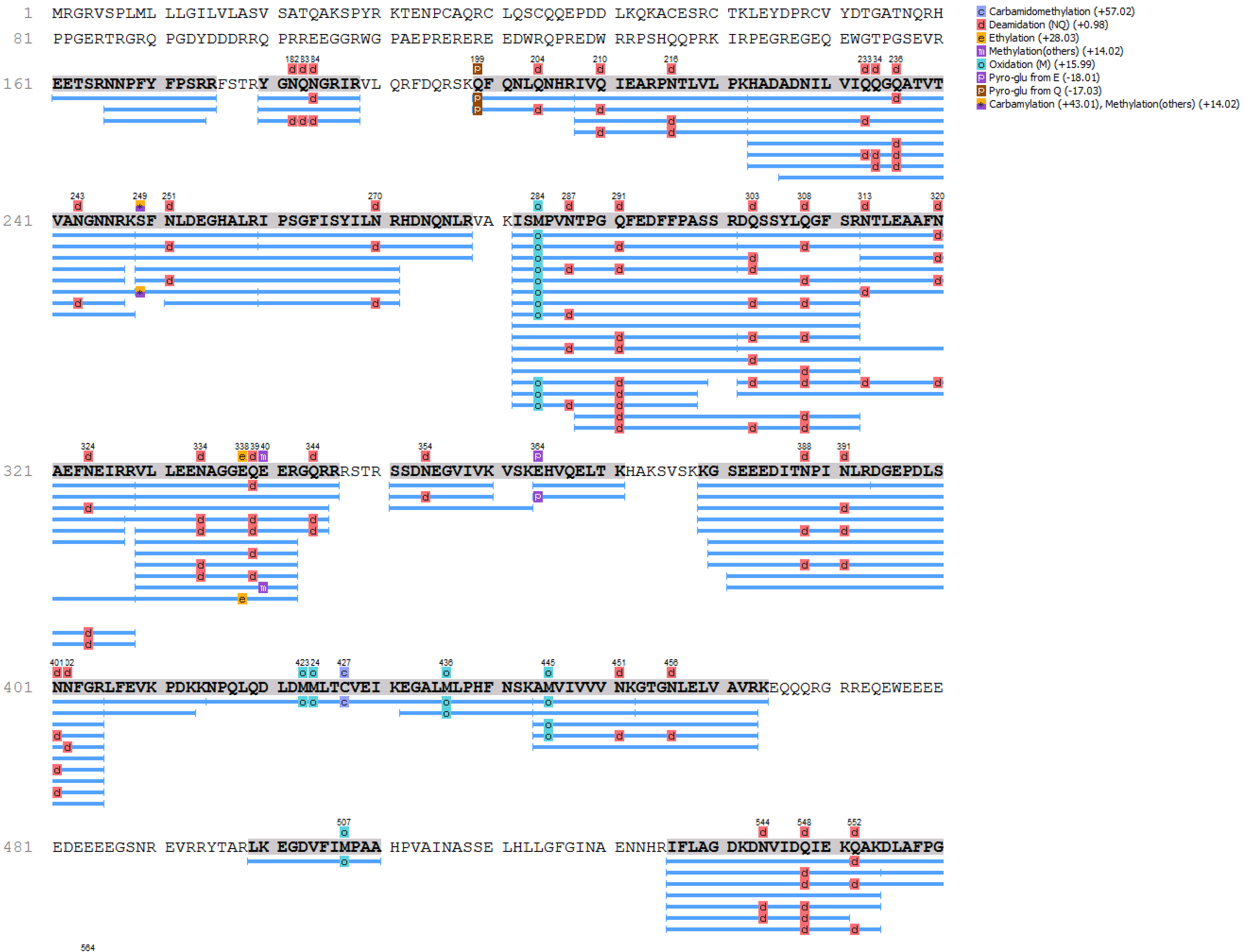
1 MRGRVSPLML LLGILVLASV SATQAKSPYR KTENPCAQRC LQSCQQEPDD LKQKACESRC TKLEYDPRCV YDTGATNQRH
81 PPGERTRGRQ PGDYDDDRRQ PRREEGGRWG PAEPRERERE EDWRQPREDW RRPSHQQPRK IRPEGREGEQ EWGTPGSEVR
161 EETSRNNPFY FPSRRFSTRY GNQNGRIRVL QRFDQRSKQF QNLQNHRIVQ IEARPNTLVL PKHADADNIL VIQQGQATVT
241 VANGNNRKSF NLDEGHALRI PSGFISYILN RHDNQNLRVA KISMPVNTPG QFEDFFPASS RDQSSYLQGF SRNTLEAAFN
321 AEFNEIRRVL LEENAGGEQE ERGQRRRSTR SSDNEGVIVK VSKEHVQELT KHAKSVSKKG SEEEDITNPI NLRDGEPDLS
401 NNFGRLFEVK PDKKNPQLQD LDMMLTCVEI KEGALMLPHF NSKAMVIVVV NKGTGNLELV AVRKEQQQRG RREQEWEEEE
481 EDEEEEGSNR EVRRYTARLK EGDVFIMPAA HPVAINASSE LHLLGFGINA ENNHRIFLAG DKDNVIDQIE KQAKDLAFPG
Carbamidomethylation (+57.02)
Deamidation (NQ) (+0.98)
Ethylation (+28.03)
Methylation(others) (+14.02)
Oxidation (M) (+15.99)
Pyro-glu from E (-18.01)
Pyro-glu from Q (-17.03)
Carbamylation (+43.01), Methylation(others) (+14.02)

Supporting Peptides:

Peptide	Uniq	-10lgP	Mass	Length	ppm	m/z	z	RT	Fraction	Scan	Source File	Area Digest 13 traka 60 kDa	#Feature	#Feature Digest 13 traka 60 kDa	Start	End	PTM
R.NTLEAAFNAEFNEIR.R	N	104.37	1737.8322	15	0.4	869.9237	2	34.24	35	1984	OB4063.raw	2.88E5	1	1	313	327	
K.ISM(+15.99)PVNTPGQFEDFFPASSR.D	N	91.29	2242.0364	20	1.0	1122.0266	2	32.92	35	1895	OB4063.raw	1.87E5	2	2	282	301	Oxidation (M)
K.ISM(+15.99)PVN(+.98)TPGQ(+.98)FEDFFPASSR.D	N	89.93	2244.0044	20	1.3	1123.0110	2	33.63	35	1944	OB4063.raw	3.04E5	2	2	282	301	Oxidation (M); Deamidation (NQ)
R.NTLEAAFN(+.98)AEFNEIR.R	N	89.50	1738.8162	15	-0.3	870.4151	2	34.79	35	2026	OB4063.raw	1.46E5	1	1	313	327	Deamidation (NQ)
K.HADADNILVIQQGQATVTVAN(+.98)GNNR.K	N	88.85	2619.3000	25	0.6	874.1078	3	29.47	35	1680	OB4063.raw	7.3E4	1	1	223	247	
K.ISM(+15.99)PVN(+.98)TPGQFEDFFPASSR.D	N	87.13	2243.0205	20	1.1	1122.5188	2	33.35	35	1914	OB4063.raw	3.87E5	1	1	282	301	Oxidation (M)
K.SFNLDEGHALR.I	N	86.49	1257.6101	11	0.6	629.8127	2	26.97	35	1516	OB4063.raw	1.94E6	2	2	249	259	
R.DQSSYLQGFSR.N	N	86.47	1286.5891	11	0.0	644.3018	2	29.29	35	1662	OB4063.raw	1.76E5	1	1	302	312	
R.DQ(+.98)SSYLQ(+.98)GFSR.N	N	84.94	1288.5571	11	-0.3	645.2856	2	30.41	35	1733	OB4063.raw	1.05E5	1	1	302	312	Deamidation (NQ)
R.IPSGFISYILNR.H	N	84.71	1378.7609	12	-0.4	690.3875	2	35.35	35	2010	OB4063.raw	1.15E6	1	1	260	271	
R.DQSSYLQ(+.98)GFSR.N	N	82.67	1287.5731	11	0.5	644.7941	2	29.84	35	1698	OB4063.raw	2.3E5	1	1	302	312	Deamidation (NQ)
K.ISM(+15.99)PVNTPGQ(+.98)FEDFFPASSR.D	N	81.23	2243.0205	20	1.1	1122.5188	2	33.35	35	2029	OB4063.raw	6.87E5	2	2	282	301	Oxidation (M); Deamidation (NQ)
K.ISMPVNTPGQ(+.98)FEDFFPASSR.D	N	81.21	2227.0254	20	0.7	1114.5208	2	34.60	35	2017	OB4063.raw	4.86E4	1	1	282	301	Deamidation (NQ)
K.HADADNILVIQ(+.98)QGQATVTVAN(+.98)GNNR.K	N	80.52	2620.2842	25	2.1	874.4371	3	30.26	35	1728	OB4063.raw	0	0	0	223	247	
K.AM(+15.99)VIVVVNKGTGNLELVAVR.K	N	79.29	2097.1980	20	0.8	700.0739	3	31.57	35	1810	OB4063.raw	3.05E5	1	1	444	463	Oxidation (M)
R.VLLEENAGGEQEER.G	N	79.16	1571.7427	14	1.1	786.8795	2	25.50	35	1436	OB4063.raw	3.06E4	1	1	329	342	
R.DQ(+.98)SSYLQGFSR.N	N	77.03	1287.5731	11	0.5	644.7941	2	29.84	35	1711	OB4063.raw	2.3E5	1	1	302	312	Deamidation (NQ)
K.HADADNILVIQQGQ(+.98)ATVTVAN(+.98)GNNRK.S	N	75.31	2748.3792	26	2.5	917.1360	3	28.64	35	1624	OB4063.raw	0	0	0	223	248	Deamidation (NQ)
R.NTLEAAFNAEFNEIRR.V	N	75.09	1893.9332	16	0.5	947.9744	2	33.09	35	1877	OB4063.raw	4.8E6	3	3	313	328	
R.IFLAGDKDNVIDQIEK.Q	N	74.42	1816.9570	16	0.1	909.4858	2	30.71	35	1760	OB4063.raw	6.31E5	2	2	536	551	
K.HADADNILVIQQGQ(+.98)ATVTVANGN(+.98)NRK.S	N	71.87	2748.3792	26	0.7	917.1343	3	29.19	35	1658	OB4063.raw	0	0	0	223	248	
R.VLLEEN(+.98)AGGEQEER.G	N	71.84	1572.7267	14	-0.1	787.3705	2	26.03	35	1466	OB4063.raw	1.83E4	1	1	329	342	Deamidation (NQ)
K.ISMPVN(+.98)TPGQ(+.98)FEDFFPASSR.D	N	71.81	2228.0095	20	1.5	1115.0137	2	35.06	35	2042	OB4063.raw	2.31E4	1	1	282	301	Deamidation (NQ)
R.IFLAGDKDNVIDQ(+.98)IEKQAK.D	N	69.30	2145.1316	19	-0.9	716.0505	3	31.66	35	1816	OB4063.raw	4.92E5	2	2	536	554	Deamidation (NQ)
K.AM(+15.99)VIVVVN(+.98)KGTGNLELVAVR.K	N	69.22	2098.1819	20	-0.3	700.4010	3	32.05	35	1835	OB4063.raw	1.92E5	1	1	444	463	Oxidation (M)
R.VLLEEN(+.98)AGGEQ(+.98)EER.G	N	68.37	1573.7107	14	-1.3	787.8616	2	26.47	35	1490	OB4063.raw	2.78E3	1	1	329	342	Deamidation (NQ)
K.AM(+15.99)VIVVVNKGTGN(+.98)LELVAVR.K	N	67.58	2098.1819	20	-0.3	700.4010	3	32.05	35	1849	OB4063.raw	1.92E5	1	1	444	463	Oxidation (M)
K.HADADNILVIQ(+.98)QGQATVTVANGNNR.K	N	67.11	2619.3000	25	0.6	874.1078	3	29.47	35	1693	OB4063.raw	7.3E4	1	1	223	247	
K.GTGNLELVAVR.K	N	66.74	1127.6299	11	-0.2	564.8221	2	28.83	35	1636	OB4063.raw	3.43E5	1	1	453	463	
R.VLLEENAGGEQ(+.98)EER.G	N	66.37	1572.7267	14	-0.1	787.3705	2	26.03	35	1454	OB4063.raw	1.83E4	1	1	329	342	Deamidation (NQ)
K.HADADNILVIQQ(+.98)GQATVTVANGNN(+.98)R.K	N	65.90	2620.2842	25	2.1	874.4371	3	30.04	35	1714	OB4063.raw	0	0	0	223	247	
R.NTLEAAFNAEFN(+.98)EIR.R	N	65.73	1738.8162	15	-0.3	870.4151	2	34.79	35	2041	OB4063.raw	1.46E5	1	1	313	327	
K.AMVIVVVNK.G	N	65.53	971.5837	9	-0.4	486.7990	2	28.34	35	1602	OB4063.raw	2.96E4	1	1	444	452	
K.GSEEEDITNPINLRDGEPDLSNNFGR.L	Y	64.97	2887.3220	26	0.7	963.4486	3	31.18	35	1782	OB4063.raw	9.64E4	1	1	380	405	
K.HADADNILVIQ(+.98)QGQ(+.98)ATVTVAN(+.98)GNNRK.S	N	64.16	2749.3630	26	0.9	917.4624	3	29.40	35	1668	OB4063.raw	7.4E4	1	1	223	248	
K.HADADNILVIQQGQATVTVANGN(+.98)NRK.S	N	64.04	2747.3950	26	-0.6	916.8051	3	29.05	35	1614	OB4063.raw	9.54E5	2	2	223	248	
K.HADADNILVIQQGQATVTVAN(+.98)GNNRK.S	N	63.48	2747.3950	26	-0.6	916.8051	3	29.05	35	1604	OB4063.raw	8.5E4	1	1	223	248	
K.GSEEEDITNPINLRDGEPDLSN(+.98)NFGR.L	Y	63.35	2888.3059	26	1.6	963.7774	3	31.38	35	1805	OB4063.raw	7.59E4	1	1	380	405	Deamidation (NQ)
R.DGEPDLSNNFGR.L	Y	63.14	1319.5742	12	0.4	660.7947	2	27.65	35	1560	OB4063.raw	9.38E3	1	1	394	405	

Peptide	Uniq	-10lgP	Mass	Length	ppm	m/z	z	RT	Fraction	Scan	Source File	Area Digest 13 traka 60 kDa	#Feature	#Feature Digest 13 traka 60 kDa	Start	End	PTM
K.HADADNILVIQQ(+.98)GQ(+.98)ATVTVANGN(+.98)NRK.S	N	62.90	2749.3630	26	0.9	917.4624	3	29.40	35	1692	OB4063.raw	7.4E4	1	1	223	248	Deamidation (NQ)
K.SFN(+.98)LDEGHALR.I	N	62.86	1258.5941	11	1.1	630.3051	2	27.44	35	1546	OB4063.raw	2.89E4	1	1	249	259	Deamidation (NQ)
R.IFLAGDKDNVIDQ(+.98)IEK.Q	N	62.82	1817.9410	16	-0.2	909.9776	2	31.18	35	1796	OB4063.raw	4.46E5	2	2	536	551	Deamidation (NQ)
R.IFLAGDKDNVIDQ(+.98)IEKQ(+.98)AK.D	N	62.39	2146.1157	19	-0.8	716.3786	3	32.23	35	1852	OB4063.raw	5.46E5	2	2	536	554	Deamidation (NQ)
R.NTLEAAFN(+.98)AEFNEIRR.V	N	62.26	1894.9172	16	0.1	948.4659	2	33.70	35	1943	OB4063.raw	8.02E5	3	3	313	328	Deamidation (NQ)
K.ISM(+15.99)PVNTPGQ(+.98)FEDFFPA.S	N	61.02	1912.8552	17	0.4	957.4353	2	36.10	35	2100	OB4063.raw	1.6E5	1	1	282	298	Oxidation (M); Deamidation (NQ)
K.AMVIVVVNKGTGNLELVAVR.K	N	60.67	2081.2031	20	-0.1	694.7416	3	33.00	35	1897	OB4063.raw	4.48E4	1	1	444	463	
K.ISM(+15.99)PVN(+.98)TPGQFEDFFPASSRDQ(+.98).S	N	60.43	2487.0898	22	-0.9	830.0365	3	33.35	35	1927	OB4063.raw	4.2E4	1	1	282	303	Oxidation (M); Deamidation (NQ)
K.AM(+15.99)VIVVVNK.G	N	60.32	987.5787	9	0.0	494.7966	2	25.83	35	1453	OB4063.raw	2.71E5	1	1	444	452	Oxidation (M)
K.HADADNILVIQQ(+.98)GQ(+.98)ATVTVAN(+.98)GNNR.K	N	57.94	2621.2681	25	5.2	874.7678	3	30.83	35	1763	OB4063.raw	0	0	0	223	247	
R.IFLAGDKDN(+.98)VIDQ(+.98)IEKQAK.D	N	57.84	2146.1157	19	-0.8	716.3786	3	32.23	35	1840	OB4063.raw	9.45E4	1	1	536	554	Deamidation (NQ)
K.HADADNILVIQ(+.98)Q(+.98)GQ(+.98)ATVTVAN(+.98)GN(+.98)NRK.S	N	57.10	2751.3311	26	5.4	918.1226	3	30.12	35	1704	OB4063.raw	9.83E3	1	1	223	248	Deamidation (NQ)
K.HADADNILVIQ(+.98)QGQ(+.98)ATVTVANGNN(+.98)RK.S	N	56.78	2749.3630	26	0.9	917.4624	3	29.40	35	1681	OB4063.raw	7.4E4	1	1	223	248	Deamidation (NQ)
K.ISM(+15.99)PVNTPGQ(+.98)FEDFFPAS.S	N	56.37	1999.8873	18	1.0	1000.9519	2	35.53	35	2072	OB4063.raw	3.74E4	1	1	282	299	Oxidation (M); Deamidation (NQ)
K.ISM(+15.99)PVNTPGQ(+.98)FEDFFPASSRDQ(+.98)SSYLQGFSR.N	N	55.84	3512.5830	31	2.1	879.1549	4	34.15	35	1978	OB4063.raw	0	0	0	282	312	Oxidation (M)
K.GTGNLELVAVRK.E	N	55.77	1255.7249	12	-0.8	628.8692	2	26.17	35	1475	OB4063.raw	1.95E4	1	1	453	464	
R.VLLEENAGGEQE(+14.02)ER.G	N	55.55	1585.7583	14	0.7	793.8870	2	26.47	35	1488	OB4063.raw	8.52E3	1	1	329	342	Methylation(others)
R.IFLAGDKDN(+.98)VIDQ(+.98)IEK.Q	N	55.37	1818.9250	16	-1.5	607.3147	3	31.57	35	1804	OB4063.raw	4.47E4	1	1	536	551	Deamidation (NQ)
K.HADADNILVIQ(+.98)Q(+.98)GQATVTVAN(+.98)GN(+.98)NRK.S	N	54.33	2750.3472	26	4.7	917.7940	3	30.50	35	1742	OB4063.raw	0	0	0	223	248	
K.GSEEEDITNPIN(+.98)LRDGEPDLSN(+.98)NFGR.L	Y	53.72	2889.2900	26	0.3	964.1042	3	31.76	35	1824	OB4063.raw	7.11E4	1	1	380	405	
K.SFNLDEGHALRIPSGFISYILNR.H	N	53.34	2618.3604	23	0.8	655.5979	4	36.38	35	2140	OB4063.raw	4.23E5	2	2	249	271	
K.HADADNILVIQ(+.98)Q(+.98)GQATVTVAN(+.98)GNNR.K	N	53.21	2621.2681	25	6.2	874.7687	3	30.67	35	1753	OB4063.raw	0	0	0	223	247	
K.HADADNILVIQQ(+.98)GQ(+.98)ATVTVAN(+.98)GNNRK.S	N	53.18	2749.3630	26	0.9	917.4624	3	29.40	35	1704	OB4063.raw	7.4E4	1	1	223	248	
K.DLAFPGSGEQ(+.98)VEKLIK.N	N	53.12	1730.9089	16	0.6	866.4623	2	32.33	35	1862	OB4063.raw	2.24E4	1	1	555	570	Deamidation (NQ)
K.ISM(+15.99)PVNTPGQ(+.98)FEDFFPASSRDQSSYLQ(+.98)GFSR.N	N	52.83	3512.5830	31	2.8	879.1555	4	33.97	35	1966	OB4063.raw	0	0	0	282	312	Oxidation (M); Deamidation (NQ)
K.HADADNILVIQQ(+.98)GQ(+.98)ATVTVANGNN(+.98)R.K	N	52.71	2621.2681	25	8.0	874.7703	3	29.80	35	1714	OB4063.raw	2.91E4	1	1	223	247	Deamidation (NQ)
R.IPSGFISYILN(+.98)R.H	N	51.41	1379.7449	12	2.4	690.8813	2	35.86	35	2080	OB4063.raw	9.57E4	1	1	260	271	Deamidation (NQ)
R.VLLEENAGGE(+28.03)QEER.G	N	51.23	1599.7739	14	-0.4	800.8939	2	27.35	35	1555	OB4063.raw	5.41E3	1	1	329	342	Ethylation
R.VLLEENAGGEQEERGQ(+.98)R.R	N	51.22	1913.9078	17	-0.1	638.9765	3	24.27	35	1349	OB4063.raw	1.43E5	1	1	329	345	
R.NNPFYFPSR.R	N	51.10	1140.5352	9	-0.8	571.2744	2	30.41	35	1744	OB4063.raw	2.15E5	1	1	166	174	
K.HADADNILVIQQ(+.98)GQATVTVAN(+.98)GNNR.K	N	51.07	2620.2842	25	-2.6	874.4331	3	30.38	35	1735	OB4063.raw	0	0	0	223	247	
R.EETSRN(+.98)NPFYFPSRR.F	N	50.93	1899.8864	15	-0.4	634.3025	3	27.55	35	1559	OB4063.raw	3E4	1	1	161	175	
R.NTLEAAFNAEFN(+.98)EIRR.V	N	50.87	1894.9172	16	-2.0	632.6451	3	32.34	35	1858	OB4063.raw	0	0	0	313	328	
K.HADADNILVIQQ(+.98)GQ(+.98)ATVTVAN(+.98)GN(+.98)N(+.98)RK.S	N	50.75	2751.3311	26	5.4	918.1226	3	30.12	35	1716	OB4063.raw	9.83E3	1	1	223	248	
R.NNPFYFPSRR.F	N	50.48	1296.6364	10	0.0	649.3254	2	28.25	35	1595	OB4063.raw	1E6	2	2	166	175	
K.AM(+15.99)VIVVVN(+.98)KGTGN(+.98)LELVAVR.K	N	49.92	2099.1660	20	8.0	700.7349	3	32.92	35	1885	OB4063.raw	4.36E3	1	1	444	463	Oxidation (M); Deamidation (NQ)
R.IPSGFISYILNRHDN(+.98)QNLR.V	N	49.71	2257.1604	19	-0.8	565.2969	4	33.29	35	1919	OB4063.raw	0	0	0	260	278	
K.ISM(+15.99)PVNTPGQ(+.98)FEDFFPASSRDQSSYLQGFSR.N	N	49.65	3511.5989	31	1.0	878.9078	4	34.14	35	1949	OB4063.raw	6.17E5	1	1	282	312	Oxidation (M)

Peptide	Uniq	-10lgP	Mass	Length	ppm	m/z	z	RT	Fraction	Scan	Source File	Area Digest 13 traka 60 kDa	#Feature	#Feature Digest 13 traka 60 kDa	Start	End	PTM
R.IPSGFISYILN(+.98)RHDNQ(+.98)NLR.V	N	49.47	2258.1443	19	2.5	565.5447	4	33.47	35	1931	OB4063.raw	0	0	0	260	278	Deamidation (NQ)
K.ISM(+15.99)PVNTPGQFEDFFPASSRDQ(+.98)SSYLQGFSR.N	N	49.33	3511.5989	31	1.8	1171.5424	3	33.82	35	1955	OB4063.raw	0	0	0	282	312	Oxidation (M)
G.SEEEDITNPINLRDGEPDLSN(+.98)NFGR.L	Y	49.27	2831.2844	25	-0.9	944.7679	3	31.48	35	1802	OB4063.raw	1.99E4	1	1	381	405	Deamidation (NQ)
R.IPSGFISYILNRHDNQ(+.98)NLR.V	N	49.25	2257.1604	19	-0.2	753.3939	3	33.45	35	1950	OB4063.raw	6.13E4	1	1	260	278	
R.IFLAGDKDNVIDQIEKQAK.D	N	48.22	2144.1477	19	2.3	715.7249	3	31.31	35	1777	OB4063.raw	9.33E3	1	1	536	554	
K.HADADNILVIQ(+.98)Q(+.98)GQ(+.98)ATVTVAN(+.98)GNNR.K	N	47.99	2622.2522	25	7.5	875.0979	3	30.51	35	1753	OB4063.raw	2.28E4	1	1	223	247	Deamidation (NQ)
R.SSDNEGVIVK.V	Y	47.01	1046.5244	10	0.1	524.2695	2	22.58	35	1248	OB4063.raw	9.14E4	1	1	351	360	
K.ISM(+15.99)PVNTPGQ(+.98)FEDFFPASSRDQ(+.98)SSYLQ(+.98)GFSR.N	N	46.92	3513.5669	31	0.8	879.3997	4	34.35	35	1991	OB4063.raw	8.02E6	2	2	282	312	Oxidation (M); Deamidation (NQ)
K.HADADNILVIQQGQATVTVANGNN(+.98)RK.S	N	46.77	2747.3950	26	1.1	687.8568	4	28.16	35	1594	OB4063.raw	0	0	0	223	248	
R.IVQIEARPNTLVLPK.H	Y	46.22	1690.0140	15	0.7	846.0149	2	29.29	35	1670	OB4063.raw	4.5E5	2	2	208	222	
K.Q(-17.03)FQNLQNHR.I	N	45.70	1166.5581	9	-0.2	584.2862	2	26.87	35	1514	OB4063.raw	4.01E3	1	1	199	207	Pyro-glu from Q
K.KGSEEEDITNPINLRDGEPDLSNNFGR.L	Y	45.15	3015.4170	27	0.8	754.8621	4	30.03	35	1712	OB4063.raw	2.92E5	1	1	379	405	
R.NTLEAAFN(+.98)AEFN(+.98)EIRR.V	N	44.87	1895.9012	16	4.5	948.9622	2	35.87	35	2089	OB4063.raw	1.14E6	1	1	313	328	Deamidation (NQ)
K.KGSEEEDITN(+.98)PIN(+.98)LRDGEPDLSNN(+.98)FGR.L	Y	44.31	3018.3689	27	4.4	1007.1347	3	30.80	35	1754	OB4063.raw	4.1E3	1	1	379	405	Deamidation (NQ)
K.S(+43.01)(+14.02)FNLDEGHALR.I	N	44.07	1314.6316	11	-0.5	658.3228	2	27.05	35	1530	OB4063.raw	9.55E3	1	1	249	259	Carbamylation; Methylation(others)
K.HADADNILVIQ(+.98)Q(+.98)GQ(+.98)ATVTVANGNNR.K	N	43.79	2621.2681	25	8.0	874.7703	3	29.80	35	1693	OB4063.raw	2.91E4	1	1	223	247	Deamidation (NQ)
R.VLLEEN(+.98)AGGEQ(+.98)EERGQ(+.98)R.R	N	43.74	1915.8759	17	-0.6	639.6322	3	25.11	35	1409	OB4063.raw	8.19E4	1	1	329	345	Deamidation (NQ)
K.KGSEEEDITNPINLRDGEPDLSNN(+.98)FGR.L	Y	43.70	3016.4009	27	1.9	755.1089	4	30.42	35	1738	OB4063.raw	0	0	0	379	405	
R.YGN(+.98)QN(+.98)GRIR.V	N	43.38	1078.5155	9	-0.7	540.2646	2	20.07	35	1082	OB4063.raw	3.11E5	2	2	180	188	Deamidation (NQ)
R.N(+.98)TLEAAFNAEFNEIR.R	N	43.25	1738.8162	15	-0.3	870.4151	2	34.79	35	2053	OB4063.raw	1.46E5	1	1	313	327	Deamidation (NQ)
K.KGSEEEDITNPIN(+.98)LRDGEPDLSNN(+.98)FGR.L	Y	43.06	3017.3850	27	7.0	755.3588	4	30.41	35	1738	OB4063.raw	2.91E5	1	1	379	405	Deamidation (NQ)
R.IVQIEARPN(+.98)TLVLPK.H	Y	43.01	1690.9980	15	-0.5	846.5059	2	29.57	35	1703	OB4063.raw	9.29E5	2	2	208	222	Deamidation (NQ)
R.SSDN(+.98)EGVIVK.V	Y	42.66	1047.5084	10	0.0	524.7615	2	23.50	35	1306	OB4063.raw	1.58E4	1	1	351	360	Deamidation (NQ)
K.KGSEEEDITN(+.98)PINLRDGEPDLSN(+.98)NFGR.L	Y	42.56	3017.3850	27	2.6	1006.8049	3	30.69	35	1754	OB4063.raw	0	0	0	379	405	Deamidation (NQ)
K.ISM(+15.99)PVN(+.98)TPGQ(+.98)FEDFFPA.S	N	42.42	1913.8392	17	-0.3	957.9266	2	36.47	35	2128	OB4063.raw	5.88E4	1	1	282	298	Oxidation (M); Deamidation (NQ)
R.DQSSYLQ(+.98)GFSRN(+.98)TLEAAFN(+.98)AEFNEIRR.V	N	42.25	3165.4639	27	3.3	1056.1654	3	36.32	35	2118	OB4063.raw	0	0	0	302	328	
R.IVQ(+.98)IEARPNTLVLPK.H	Y	42.18	1690.9980	15	0.0	564.6733	3	29.57	35	1685	OB4063.raw	9.29E5	2	2	208	222	Deamidation (NQ)
K.EHVQELTK.H	Y	42.04	982.5084	8	0.7	492.2618	2	19.02	35	1040	OB4063.raw	5.16E3	1	1	364	371	
N.TPGQ(+.98)FEDFFPASSRDQ(+.98)SSYLQ(+.98)GFSR.N	N	41.87	2856.2515	25	6.8	953.0975	3	33.96	35	1980	OB4063.raw	2.48E5	1	1	288	312	Deamidation (NQ)
R.DQSSYLQ(+.98)GFSRN(+.98)TLEAAFNAEFNEIRR.V	N	41.74	3164.4797	27	0.0	1055.8339	3	36.20	35	2118	OB4063.raw	7.48E4	1	1	302	328	
K.Q(-17.03)FQNLQ(+.98)NHR.I	N	41.25	1167.5421	9	-0.4	584.7781	2	27.85	35	1580	OB4063.raw	1.89E4	1	1	199	207	Pyro-glu from Q
K.ISMPVNTPGQ(+.98)FEDFFPASSRDQ(+.98)SSYLQGFSR.N	N	40.49	3496.5881	31	1.6	1166.5386	3	35.06	35	2021	OB4063.raw	5.34E5	1	1	282	312	Deamidation (NQ)
R.VLLEENAGGEQ(+.98)EERGQRR.R	N	39.96	2070.0090	18	-5.0	691.0068	3	24.83	35	1391	OB4063.raw	4.28E4	1	1	329	346	
R.LFEVKPDK.K	N	39.73	974.5436	8	0.2	488.2792	2	24.38	35	1358	OB4063.raw	1.2E5	2	2	406	413	
K.ISM(+15.99)PVNTPGQFEDFFPASSRDQ(+.98)SSYLQ(+.98)GFSR.N	N	39.69	3512.5830	31	5.0	1171.8741	3	33.88	35	1960	OB4063.raw	0	0	0	282	312	Oxidation (M)
K.HADADNILVIQQ(+.98)GQATVTVANGN(+.98)N(+.98)RK.S	N	39.50	2749.3630	26	6.9	688.3528	4	31.09	35	1842	OB4063.raw	5.25E4	1	1	223	248	
K.KGSEEEDITN(+.98)PIN(+.98)LRDGEPDLSNNFGR.L	Y	39.49	3017.3850	27	7.0	755.3588	4	30.41	35	1721	OB4063.raw	2.91E5	1	1	379	405	
R.IPSGFISYILN(+.98)RHDNQ(+.98)N(+.98)LR.V	N	38.84	2259.1284	19	5.7	565.7926	4	33.53	35	2009	OB4063.raw	1.27E6	1	1	260	278	
R.IPSGFISYILN(+.98)RHDNQN(+.98)LR.V	N	38.53	2258.1443	19	-1.8	753.7207	3	33.54	35	1936	OB4063.raw	0	0	0	260	278	Deamidation (NQ)
K.ISMPVNTPGQFEDFFPASSRDQ(+.98)SSYLQGFSR.N	N	38.43	3495.6040	31	2.4	1166.2114	3	34.55	35	2004	OB4063.raw	0	0	0	282	312	

Peptide	Uniq	-10lgP	Mass	Length	ppm	m/z	z	RT	Fraction	Scan	Source File	Area Digest 13 traka 60 kDa	#Feature	#Feature Digest 13 traka 60 kDa	Start	End	PTM
K.GSEEEDITN(+.98)PIN(+.98)LRDGEPDLSNN(+.98)FGR.L	Y	37.86	2890.2739	26	3.5	964.4352	3	32.00	35	1836	OB4063.raw	0	0	0	380	405	Deamidation (NQ)
R.YGNQ(+.98)N(+.98)GRIR.V	N	37.77	1078.5155	9	-0.7	540.2646	2	20.07	35	1094	OB4063.raw	3.11E5	2	2	180	188	
R.VLLEENAGGEQ(+.98)EERGQ(+.98)R.R	N	36.86	1914.8918	17	0.2	639.3047	3	24.64	35	1374	OB4063.raw	1.77E5	1	1	329	345	
K.E(-18.01)HVQELTK.H	Y	36.72	964.4978	8	-1.6	483.2554	2	23.08	35	1279	OB4063.raw	3.58E3	1	1	364	371	Pyro-glu from E
F.NLDEGHALR.I	N	36.61	1023.5097	9	0.8	512.7625	2	26.97	35	1531	OB4063.raw	1.56E4	1	1	251	259	
R.VLLEEN(+.98)AGGEQ(+.98)EERGQR.R	N	36.15	1914.8918	17	0.2	639.3047	3	24.64	35	1398	OB4063.raw	1.77E5	1	1	329	345	
R.IPSGFISYILNRHDN(+.98)Q(+.98)NLR.V	N	36.15	2258.1443	19	0.8	565.5438	4	34.62	35	2009	OB4063.raw	0	0	0	260	278	
R.IPSGFISYILN(+.98)RHDN(+.98)QNLR.V	N	36.12	2258.1443	19	-1.6	565.5424	4	35.07	35	2038	OB4063.raw	7.93E4	1	1	260	278	
R.IPSGFISYILN(+.98)RHDNQNLR.V	N	36.08	2257.1604	19	0.6	753.3945	3	33.08	35	1904	OB4063.raw	0	0	0	260	278	
R.LKEGDVFIM(+15.99)PAA.H	N	35.74	1305.6638	12	-0.5	653.8389	2	29.47	35	1678	OB4063.raw	3.05E4	1	1	499	510	Oxidation (M)
R.N(+.98)NPFYFPSRR.F	N	35.51	1297.6204	10	0.8	649.8180	2	29.01	35	1644	OB4063.raw	4.08E4	1	1	166	175	
K.KGSEEEDITNPIN(+.98)LRDGEPDLSNNFGR.L	Y	35.45	3016.4009	27	3.1	755.1099	4	31.31	35	1793	OB4063.raw	0	0	0	379	405	
K.Q(-17.03)FQN(+.98)LQ(+.98)NHR.I	N	35.33	1168.5261	9	-0.3	585.2701	2	28.34	35	1609	OB4063.raw	1.07E4	1	1	199	207	Pyro-glu from Q; Deamidation (NQ)
K.Q(+.98)AKDLAFPGSGEQ(+.98)VEK.L	N	35.23	1704.8206	16	-1.9	569.2797	3	26.67	35	1500	OB4063.raw	8.39E3	1	1	552	567	Deamidation (NQ)
K.SFN(+.98)LDEGHALRIPSGFISYILNR.H	N	35.13	2619.3445	23	6.9	655.8479	4	40.04	35	2348	OB4063.raw	0	0	0	249	271	Deamidation (NQ)
K.ISM(+15.99)PVN(+.98)TPGQFEDFFPASSRDQ(+.98)SSYLQ(+.98)GFSR.N	N	35.04	3513.5669	31	5.7	1172.2029	3	34.24	35	1990	OB4063.raw	8E6	1	1	282	312	Oxidation (M); Deamidation (NQ)
R.IVQ(+.98)IEARPN(+.98)TLVLPK.H	Y	34.74	1691.9822	15	-0.2	565.0012	3	30.12	35	1720	OB4063.raw	2.65E5	1	1	208	222	Deamidation (NQ)
R.SSDNEGVIVKVSK.E	Y	34.34	1360.7197	13	-1.4	454.5799	3	23.74	35	1322	OB4063.raw	6.99E3	1	1	351	363	
R.VLLEENAGGEQ(+.98)EERGQR.R	N	34.10	1913.9078	17	-0.1	638.9765	3	24.27	35	1364	OB4063.raw	1.43E5	1	1	329	345	
K.KGSEEEDITNPINLRDGEPDLSN(+.98)NFGR.L	Y	34.10	3016.4009	27	4.8	755.1111	4	31.93	35	1832	OB4063.raw	0	0	0	379	405	
R.IPSGFISYILN(+.98)RHDN(+.98)Q(+.98)NLR.V	N	33.82	2259.1284	19	7.5	754.0557	3	33.45	35	1950	OB4063.raw	1.33E6	2	2	260	278	Deamidation (NQ)
R.IPSGFISYILNRHDNQN(+.98)LR.V	N	33.54	2257.1604	19	-0.2	753.3939	3	33.45	35	1936	OB4063.raw	6.13E4	1	1	260	278	
R.VLLEEN(+.98)AGGEQ(+.98)EERGQRR.R	N	33.54	2070.9929	18	-1.6	691.3372	3	23.68	35	1319	OB4063.raw	3.7E4	1	1	329	346	Deamidation (NQ)
R.YGN(+.98)QNGRIR.V	N	33.50	1077.5315	9	0.9	360.1848	3	19.20	35	1038	OB4063.raw	1.04E5	1	1	180	188	
R.YGN(+.98)Q(+.98)N(+.98)GRIR.V	N	33.15	1079.4995	9	1.3	360.8409	3	20.88	35	1147	OB4063.raw	6.14E4	1	1	180	188	Deamidation (NQ)
K.NPQ(+.98)LQ(+.98)DLDM(+15.99)M(+15.99)LTC(+57.02)VEIKEGALM(+15.99)LPHFNSK.A	N	32.90	3421.5913	29	2.5	856.4072	4	34.21	35	1981	OB4063.raw	0	0	0	415	443	Oxidation (M); Carbamidomethylation
K.ISM(+15.99)PVN(+.98)TPGQ(+.98)FEDFFPASSRDQ(+.98)SSYLQGFSR.N	N	32.83	3513.5669	31	5.7	1172.2029	3	34.24	35	2188	OB4063.raw	8E6	1	1	282	312	Oxidation (M); Deamidation (NQ)
R.LFEVKPDKK.N	N	32.65	1102.6385	9	-1.0	552.3260	2	22.20	35	1226	OB4063.raw	3.34E3	1	1	406	414	
K.NPQ(+.98)LQDLDM(+15.99)M(+15.99)LTC(+57.02)VEIKEGALM(+15.99)LPHFNSK.A	N	32.51	3420.6074	29	1.2	856.1602	4	33.80	35	1981	OB4063.raw	1.32E5	1	1	415	443	Oxidation (M); Carbamidomethylation
R.YGNQN(+.98)GRIR.V	N	32.16	1077.5315	9	0.9	360.1848	3	19.20	35	1048	OB4063.raw	1.04E5	1	1	180	188	
K.KGSEEEDITNPINLR.D	Y	31.84	1713.8533	15	0.1	572.2917	3	27.95	35	1586	OB4063.raw	9.33E3	1	1	379	393	
N.TPGQ(+.98)FEDFFPASSRDQSSYLQ(+.98)GFSR.N	N	31.78	2855.2673	25	-0.6	952.7625	3	33.71	35	1948	OB4063.raw	0	0	0	288	312	Deamidation (NQ)
G.SEEEDITNPIN(+.98)LRDGEPDLSNN(+.98)FGR.L	Y	31.76	2832.2686	25	-1.4	945.0955	3	31.76	35	1802	OB4063.raw	2.16E4	1	1	381	405	
K.Q(-17.03)FQN(+.98)LQNHR.I	N	31.44	1167.5421	9	-0.4	584.7781	2	27.85	35	1554	OB4063.raw	1.89E4	1	1	199	207	Pyro-glu from Q
K.ISMPVNTPGQ(+.98)FEDFFPASSRDQ(+.98)SSYLQ(+.98)GFSR.N	N	31.28	3497.5720	31	2.6	1166.8677	3	35.17	35	2044	OB4063.raw	0	0	0	282	312	Deamidation (NQ)
R.DQ(+.98)SSYLQ(+.98)GFSRN(+.98)TLEAAFN(+.98)AEFN(+.98)EIRR.V	N	30.59	3167.4319	27	8.2	1056.8265	3	36.57	35	2129	OB4063.raw	1.42E5	1	1	302	328	Deamidation (NQ)
K.EGALM(+15.99)LPHFNSK.A	N	30.42	1358.6653	12	-0.2	453.8956	3	29.01	35	1643	OB4063.raw	1.66E5	1	1	432	443	Oxidation (M)
K.ISM(+15.99)P(+15.99)VNTPGQ(+.98)FEDFFPASSRDQ(+.98)SSYLQ(+.98)GFSR.N	N	29.84	3529.5620	31	7.8	1177.5371	3	34.77	35	2018	OB4063.raw	0	0	0	282	312	Oxidation (M)

Peptide	Uniq	-10lgP	Mass	Length	ppm	m/z	z	RT	Fraction	Scan	Source File	Area Digest 13 traka 60 kDa	#Feature	#Feature Digest 13 traka 60 kDa	Start	End	PTM
R.VLLEENAGGEQEERGQ(+.98)RR.R	N	29.67	2070.0090	18	-1.3	691.0094	3	23.17	35	1278	OB4063.raw	4.28E4	1	1	329	346	
R.RVLLEEN(+.98)AGGEQ(+.98)EERGQ(+.98)R.R	N	29.64	2071.9771	18	-2.9	691.6643	3	24.08	35	1347	OB4063.raw	1.44E4	1	1	328	345	Deamidation (NQ)
R.VLLEEN(+.98)AGGEQEERGQ(+.98)R.R	N	28.61	1914.8918	17	0.2	639.3047	3	24.64	35	1422	OB4063.raw	1.77E5	1	1	329	345	
K.SFNLDEGHALRIPSGFISYILNRHDNQN(+.98)LR.V	N	28.47	3496.7600	30	-0.1	700.3592	5	35.63	35	2074	OB4063.raw	1.68E5	1	1	249	278	
R.YGN(+.98)Q(+.98)NGRIR.V	N	28.16	1078.5155	9	1.7	360.5131	3	20.06	35	1088	OB4063.raw	2.55E5	1	1	180	188	
K.Q(+.98)AKDLAFPGSGEQVEK.L	N	28.09	1703.8365	16	0.7	568.9532	3	26.48	35	1483	OB4063.raw	1.25E4	1	1	552	567	Deamidation (NQ)
K.SFNLDEGHALRIPSGFISYILN(+.98)RHDNQN(+.98)LR.V	N	27.94	3497.7439	30	3.3	700.5583	5	35.72	35	2074	OB4063.raw	3.15E5	1	1	249	278	
R.VLLEENAGGEQEERGQR.R	N	26.31	1912.9238	17	0.6	638.6489	3	23.89	35	1337	OB4063.raw	4.66E4	1	1	329	345	
K.N(+.98)PQLQDLDM(+15.99)M(+15.99)LTC(+57.02)VEIKEGALM(+15.99)LPHFNSK.A	N	25.90	3420.6074	29	1.2	856.1602	4	33.80	35	1994	OB4063.raw	1.32E5	1	1	415	443	Oxidation (M); Carbamidomethylation
R.DQ(+.98)SSYLQGFSRN(+.98)TLEAAFN(+.98)AEFN(+.98)EIRR.V	N	25.88	3166.4478	27	5.1	1056.4952	3	36.89	35	2153	OB4063.raw	0	0	0	302	328	Deamidation (NQ)
D.ADNILVIQQ(+.98)GQATVTVANGNNRK.S	N	25.85	2424.2720	23	-0.2	809.0978	3	28.93	35	1652	OB4063.raw	8.86E3	1	1	226	248	
R.DQ(+.98)SSYLQGFSRNTLEAAFNAEFN(+.98)EIRR.V	N	25.25	3164.4797	27	0.0	1055.8339	3	36.20	35	2153	OB4063.raw	7.48E4	1	1	302	328	
R.DQ(+.98)SSYLQGFSRN(+.98)TLEAAFNAEFNEIRR.V	N	25.24	3164.4797	27	2.2	792.1290	4	36.28	35	2105	OB4063.raw	2.6E5	1	1	302	328	
R.VLLEENAGGEQEERGQRR.R	N	25.15	2069.0249	18	0.5	690.6826	3	22.58	35	1271	OB4063.raw	1.8E4	1	1	329	346	
R.DQ(+.98)SSYLQ(+.98)GFSRNTLEAAFNAEFN(+.98)EIRR.V	N	24.52	3165.4639	27	3.1	792.3757	4	36.40	35	2123	OB4063.raw	0	0	0	302	328	
K.HADADNILVIQ(+.98)QGQ(+.98)ATVTVAN(+.98)GNN(+.98)RK.S	N	24.44	2750.3472	26	1.8	688.5953	4	32.27	35	1854	OB4063.raw	0	0	0	223	248	
**total 171 peptides**																	

tr|Q9SQH7|Q9SQH7_ARAHY
back to list

| Protein Coverage | Supporting Peptides |

Protein Coverage:

```
  1 MAKLLELSFC FCFLVLGASS ISFRQQPEEN ACQFQRLNAQ RPDNRIESEG GYIETWNPNN QEFECAGVAL SRLVLRRNAL
 81 RRPFYSNAPQ EIFIQQGRGY FGLIFPGCPS TYEEPAQQGR RYQSQRPPRR LQEEDQSQQQ QDSHQKVHRF NEGDLIAVPT
161 GVAFWLYNDH DTDVVAVSLT DTNNNDNQLD QFPRRFNLAG NHEQEFLRYQ QQSRQSRRRS LPYSPYSPHS RPRREEREFR
241 PRGQHSRRER AGQEEEDEGG NIFSGFTPEF LEQAFQVDDR QIVQNLWGEN ESEEEGAIVT VRGGLRILSP DGTRGADEEE
321 EYDEDQYEYH EQDGRRGRGS RGGGNGIEET ICTACVKKNI GGNRSPHIYD PQRWFTQNCH DLNLLILRWL GLSAEYGNLY
401 RNALFVPHYN TNAHSIIYAL RGRAHVQVVD SNGNRVYDEE LQEGHVLVVP QNFAVAGKSQ SENFEYVAFK TDSRPSIANF
481 AGENSFIDNL PEEVVANSYG LPREQARQLK NNNPFKFFVP PFQQSPRAVA
```

Supporting Peptides:

Peptide	Uniq	-10lgP	Mass	Length	ppm	m/z	z	RT	Fraction	Scan	Source File	Area Digest 13 traka 60 kDa	#Feature	#Feature Digest 13 traka 60 kDa	Start	End	PTM
R.WLGLSAEYGNLYR.N	Y	88.43	1540.7673	13	-0.2	771.3908	2	33.27	35	1916	OB4063.raw	1.85E4	1	1	389	401	
**total 1 peptides**																	

tr|Q647H3|Q647H3_ARAHY
back to list

| Protein Coverage | Supporting Peptides |

Protein Coverage:

1 MAKLLALSVC FCFLVLGASS ISFRQQPEEN ACQFQRLNAQ RPDNRLESEG GYIETWNPNN QEFECAGVAL SRLVLRRNAL
81 RRPFYSNAPQ EIFIQQGRGY FGLIFPGCPS TYEEPAQQGR RHQSQRAPRR FEGEDQSQQQ QQDSHQKVRR FDEGDLIAVP
161 TGVALWMYND HDTDVVAVSL TDTNNNDNQL DQFPRRFNLA GNHEQEFLRY QQQSRRRSLP YSPYSPQSQP RQEEREFSPR
241 GQHSRRERAG QEQENEGGNI FSGFTPEFLA QAFQVDDRQI LQNLRGENES DEQGAIVTVR GGLRILSPDR KRRQQYERPD
321 EEEEYDEDEY EYDEEERQQD RRRGRGSRGR GNGIEETICT ASVKKNIGRN RSPDIYNPQA GSLKTANDLN LLILR**WLGLS**
401 **AEYGNLYR**NA LFVPHYNTNA HSIIYALRGR AHVQVVDSNG NRVYDEELQE GHVLVVPQNF AVAGKSQSDN FEYVAFKTDS
481 RPSIANLAGE NSIIDNLPEE VVANSYGLPR EQARQLKNNN PFKFFVPPSQ QSLGAVA

Supporting Peptides:

Peptide	Uniq	-10lgP	Mass	Length	ppm	m/z	z	RT	Fraction	Scan	Source File	Area Digest 13 traka 60 kDa	#Feature	#Feature Digest 13 traka 60 kDa	Start	End	PTM
R.WLGLSAEYGNLYR.N	Y	88.43	1540.7673	13	-0.2	771.3908	2	33.27	35	1916	OB4063.raw	1.85E4	1	1	396	408	
**total 1 peptides**																	

tr|Q647H4|Q647H4_ARAHY

back to list

| Protein Coverage | Supporting Peptides |

Protein Coverage:

1 MGKLLALSVC FCFLVLGASS ISFRQQPEEN ACQFQRLNAQ RPDNRIESEG GYIETWNPNN QEFECAGVAL SRLVLRRNAL
81 RRPFYSNAPQ EIFIQQGRGY FGLIFPGCPS TYEEPAQQGR RHQSQRPPRR FQGQDQSQQQ QDSHQKVHRF DEGDLIAVPT
161 GVAFWMYNDH DTDVVAVSLT DTNNNDNQLD QFPRRFNLAG NHEQEFLRYQ QQSRRRSLPY SPYSPQTQPK QEDREFSPRG
241 QHGRRERAGQ EQENEGGNIF SGFTPEFLAQ AFQVDDRQIL QNLRGENESD EQGAIVTVRG GLRILSPDRK RRQQYERPDE
321 EEEYDEDEYE YDEEERQHDR RRGRGSRGSG NGIEETICTA SFKKNIGRNR SPDIYNPQAG SLKTANELNL LILR**WLGLSA**
401 **EYGNLYR**NAL FVPHYNTNAH SIIYALRGRA HVQVVDSNGD RVFDEELQEG HVLVVPQNFA VAGKSQSENF EYVAFKTDSR
481 PSIANLAGEN SFIDNLPEEV VANSYGLPRE QARQLKNNNP FKFFVPPSEQ SLRAVA

Supporting Peptides:

Peptide	Uniq	-10lgP	Mass	Length	ppm	m/z	z	RT	Fraction	Scan	Source File	Area Digest 13 traka 60 kDa	#Feature	#Feature Digest 13 traka 60 kDa	Start	End	PTM
R.WLGLSAEYGNLYR.N	Y	88.43	1540.7673	13	-0.2	771.3908	2	33.27	35	1916	OB4063.raw	1.85E4	1	1	395	407	
**total 1 peptides**																	

tr|Q8LKN1|Q8LKN1_ARAHY

back to list

| Protein Coverage | Supporting Peptides |

Protein Coverage:

1 MGKLLALSVC FCFLVLGASS ISFRQQPEEN ACQFQRLNAQ RPDNRIESEG GYIETWNPNN QEFECAGVAL SRLVLRRNAL
81 RRPFYSNAPQ EIFIQQGRGY FGLIFPGCPS TYEEPAQQGR RHQSQRPPRR FQGQDQSQQQ QDSHQKVHRF DEGDLIAVPT
161 GVAFWMYNDH DTDVVAVSLT DTNNNDNQLD QFPRRFNLAG NHEQEFLRYQ QQSRRRSLPY SPYSPQTQPK QEDREFSPRG
241 QHGRRERAGQ EQENEGGNIF SGFTPEFLAQ AFQVDDRQIL QNLRGENESD EQGAIVTVRG GLRILSPDRK RRQQYERPDE
321 EEEYDEDEYE YDEEERQQDR RRGRGSRGSG NGIEETICTA SFKKNIGRNR SPDIYNPQAG SLKTANELQL NLLILR**WLGL**
401 **SAEYGNLYR**N ALFVPHYNTN AHSIIYALRG RAHVQVVDSN GDRVFDEELQ EGHVLVVPQN FAVAGKSQSE NFEYVAFKTD
481 SRPSIANLAG ENSFIDNLPE EVVANSYGLP REQARQLKNN NPFKFFVPPS EQSLRAVA

Supporting Peptides:

Peptide	Uniq	-10lgP	Mass	Length	ppm	m/z	z	RT	Fraction	Scan	Source File	Area Digest 13 traka 60 kDa	#Feature	#Feature Digest 13 traka 60 kDa	Start	End	PTM
R.WLGLSAEYGNLYR.N	Y	88.43	1540.7673	13	-0.2	771.3908	2	33.27	35	1916	OB4063.raw	1.85E4	1	1	397	409	
**total 1 peptides**																	

tr|Q9FZ11|Q9FZ11_ARAHY

back to list

| Protein Coverage | Supporting Peptides |

Protein Coverage:

1 MIRGRLALSV CFCFLVLGAS SISFRQQPEE NACQFQRLNA QRPDNRLESE GGYIETWNPN NQEFECAGVA LSRLVLRRNA
81 LRRPFYSNAP QEIFIQQGRG YFGLIFPGCP STYEEPAQQG RRHQSQRAPR RFEGEDQSQQ QQQDSHQKVR RFDEGDLIAV
161 PTGVALWMFN DHDTDVVAVS LTDTNNNDNQ LDQFPRRFNL AGNHEQEFLR YQQQSRRRSL PYSPYSPQSQ PRQEEREFSP
241 RGQHSRRERA GQEEENEGGN IFSGFTPEFL AQAFQVDDRQ IVQNLRGENE SEEEGAIVTV KGGLRILSPD RKRGADEEEE
321 YDEDEYEYDE EDRRRGRGSR GRGNGIEETI CTASVKKNIG RNRSPDIYNP QAGSLKTAND LNLLILR**WLG LSAEYGNLYR**
401 NALFVPHYNT NAHSIIYALR GRAHVQVVDS NGNRVYDEEL QEGHVLVVPQ NFAVAGKSQS DNFEYVAFKT DSRPNIANFA
481 GENSIIDNLP EEVVANSYGL PREQARQLKN NNPFKFFVPP SQQSLRAVA

Supporting Peptides:

Peptide	Uniq	-10lgP	Mass	Length	ppm	m/z	z	RT	Fraction	Scan	Source File	Area Digest 13 traka 60 kDa	#Feature	#Feature Digest 13 traka 60 kDa	Start	End	PTM
R.WLGLSAEYGNLYR.N	Y	88.43	1540.7673	13	-0.2	771.3908	2	33.27	35	1916	OB4063.raw	1.85E4	1	1	388	400	
**total 1 peptides**																	

tr|Q5I6T2|Q5I6T2_ARAHY

back to list

| Protein Coverage | Supporting Peptides |

Protein Coverage:

1 MAKLLELSFC FCFLVLGASS ISFRQQPEEN ACQFQRLNAQ RPDNRIESEG GYIETWNPNN QEFECAGVAL SRLVLRRNAL
81 RRPFYSNAPQ EIFIQQGRGY FGLIFPGCPS TYEEPAQQGR RSQSQRPPRR LQGEDQSQQQ QDSHQKVHRF DEGDLIAVPT
161 GVAFWLYNDH DTDVVAVSLT DTNNNDNQLD QFPRRFNLAG NHEQEFLRYQ QQSRQSRRRS LPYSPYSPQS QPRQEEREFS
241 PRGQHSRRER AGQEEENEGG NIFSGFTPEF LEQAFQVDDR QIVQNLRGEN ESEEEGAIVT VRGGLRILSP DRKRGADEEE
321 EYDEDEYEYD EEDRRRGRGS RGRGNGIEET ICTASVKKNI GRNRSPDIYN PQAGSLKTAN DLNLLILR**WL GLSAEYGNLY**
401 **R**NALFVPHYN TNAHSIIYAL RGRAHVQVVD SNGNRVYDEE LQEGHVLVVP QNFAVAGKSQ SDNFEYVAFK TDSRPSIANL
481 AGENSVIDNL PEEVVANSYG LQREQARQQL KNNNPFKFFV PPSQQSPRAV A

Supporting Peptides:

Peptide	Uniq	-10lgP	Mass	Length	ppm	m/z	z	RT	Fraction	Scan	Source File	Area Digest 13 traka 60 kDa	#Feature	#Feature Digest 13 traka 60 kDa	Start	End	PTM
R.WLGLSAEYGNLYR.N	Y	88.43	1540.7673	13	-0.2	771.3908	2	33.27	35	1916	OB4063.raw	1.85E4	1	1	389	401	
**total 1 peptides**																	

tr|Q6T2T4|Q6T2T4_ARAHY

back to list

| Protein Coverage | Supporting Peptides |

Protein Coverage:

1 MGKLLALSVC FCFLVLGASS ISFRQQPEEN ACQFQRLNAQ RPDNRIESEG GYIETWNPNN QEFECAGVAL SRLVLRRNAL
81 RRPFYSNAPQ EIFIQQGRAY FGLIFLGCPS TYEEPAQQGR RHQSQRPPRR FQGQDQSQQQ QDSHQKVHRF DEGDLIAVPT
161 GVAFWMYNDH DTDVVAVSLT DTNNNDNQLD QFPRRFNLAG NHEQEFLRYQ QQSRRRSLPY SPYSPQTQPK QEDREFSPRG
241 QHGRRERAGQ EQENEGGNIF SGFTPEFLAQ AFQVDDRQIL QNLRGENESD EQGAIVTVRG GLRILSPDRK KRQQYERPDE
321 EEEYDEDEYE YDEEERQQDR RRGRGSRGSG NGIEETICTA SFKKNIGRNR SPDIYNPQAG SLKTANELNL LILR**WLGLSA**
401 **EYGNLYR**NAL FVPHYNTNAH SIIYALRGRA HVQVVDSNGD RVFDEELQEG HVLVVPQNFA VAGKSQSENF EYVAFKTDSR
481 PSIANLAGEN SFIDNLPEEV VANSYGLPRE QARQLKNNNP FKFFVPPSEQ SLRAVA

Supporting Peptides:

Peptide	Uniq	-10lgP	Mass	Length	ppm	m/z	z	RT	Fraction	Scan	Source File	Area Digest 13 traka 60 kDa	#Feature	#Feature Digest 13 traka 60 kDa	Start	End	PTM
R.WLGLSAEYGNLYR.N	Y	88.43	1540.7673	13	-0.2	771.3908	2	33.27	35	1916	OB4063.raw	1.85E4	1	1	395	407	
**total 1 peptides**																	

tr|A1DZF0|A1DZF0_ARAHY

back to list

| Protein Coverage | Supporting Peptides |

Protein Coverage:

1 MAKLLELSFC FCFLVLGASS ISFRQQPEEN ACQFQRLNAQ RPDNRIESEG GYIETWNPNN QEFECAGVAL SRLVLRRNAL
81 RRPFYSNAPQ EIFIQQGRGY FGLIFPGCPS TYEEPAQQGR RYQSQRPPRR LQEEDQSQQQ QDSHQKVHRF NEGDLIAVPT
161 GVAFWLYNDH DTDVVAVSLT DTNNNDNQLD QFPRRFNLAG NHEQEFLRYQ QQSRQSRRRS LPLSPYSPQP GQEDREFSPQ
241 GQHGRRERAG QEQENEGGNI FSGFTSEFLA QAFQVDDRQI VQNLRGENES EEQGAIVTVK GGLRILSPDR KSPDEEEEYD
321 EDEYAEEERQ QDRRRGRGSR GSGNGIEETI CTATVKKNIG RNRSPDIYNP QAGSLKTANE LNLLILR**WLG LSAEYGNLYR**
401 NALFVPHYNT NAHSIIYALR GRAHVQVVDS NGNRVYDEEL QEGHVLVVPQ NFAVAGKSQS ENFEYVAFKT DSRPSIANLA
481 GENSFIDNLP EEVVANSYGL PREQARQLKN NNPFKFFVPP FQQSPRAVA

Supporting Peptides:

Peptide	Uniq	-10lgP	Mass	Length	ppm	m/z	z	RT	Fraction	Scan	Source File	Area Digest 13 traka 60 kDa	#Feature	#Feature Digest 13 traka 60 kDa	Start	End	PTM
R.WLGLSAEYGNLYR.N	Y	88.43	1540.7673	13	-0.2	771.3908	2	33.27	35	1916	OB4063.raw	1.85E4	1	1	388	400	
**total 1 peptides**																	

tr|B5TYU1|B5TYU1_ARAHY

back to list

| Protein Coverage | Supporting Peptides |

Protein Coverage:

1 MAKLLELSFC FCFLVLGASS ISFRQQPEEN ACQFQRLNAQ RPDNRIESEG GYIETWNPNN QEFECAGVAL SRLVLRRNAL
81 RRPFYSNAPQ EIFIQQGRGY FGLIFPGCPS TYEEPAQQGR RYQSQRPPRR LQEEDQSQQQ QDSHQKVHRF NEGDLIAVPT
161 GVAFWLYNDH DTDVVAVSLT DTNNNDNQLD QFPRRFNLAG NHEQEFLRYQ QQSRQSRRRS LPYSPYSPQS QPRQEEREFS
241 PRGQHSRRER AGQEEENEGG NIFSGFTPEF LAQAFQVDDR QIVQNLRGEN ESEEQGAIVT VRGGLRILSP DRKRGADEEE
321 EYDEDEYEYD EEDRRRGRGS RGSGNGIEET ICTATVKKNI GRNRSPDIYN PQAGSLKTAN ELNLLILR**WL GLSAEYGNLY**
401 **R**NALFVPHYN TNAHSIIYAL RGRAHVQVVD SNGNRVYDEE LQEGHVLVVP QNFAVAGKSQ SDNFEYVAFK TDSRPSIANL
481 AGENSVIDNL PEEVVANSYG LPREQARQLK NNNPFKFFVP PSQQSPRAVA

Supporting Peptides:

Peptide	Uniq	-10lgP	Mass	Length	ppm	m/z	z	RT	Fraction	Scan	Source File	Area Digest 13 traka 60 kDa	#Feature	#Feature Digest 13 traka 60 kDa	Start	End	PTM
R.WLGLSAEYGNLYR.N	Y	88.43	1540.7673	13	-0.2	771.3908	2	33.27	35	1916	OB4063.raw	1.85E4	1	1	389	401	
**total 1 peptides**																	

Peptide List

1. Notes Gastric Digest Raw peanut Band #14 60-66 kDa (PTM496)

2. Result Statistics

Figure 1. False discovery rate (FDR) curve. X axis is the number of peptide-spectrum matches (PSM) being kept. Y axis is the corresponding FDR.

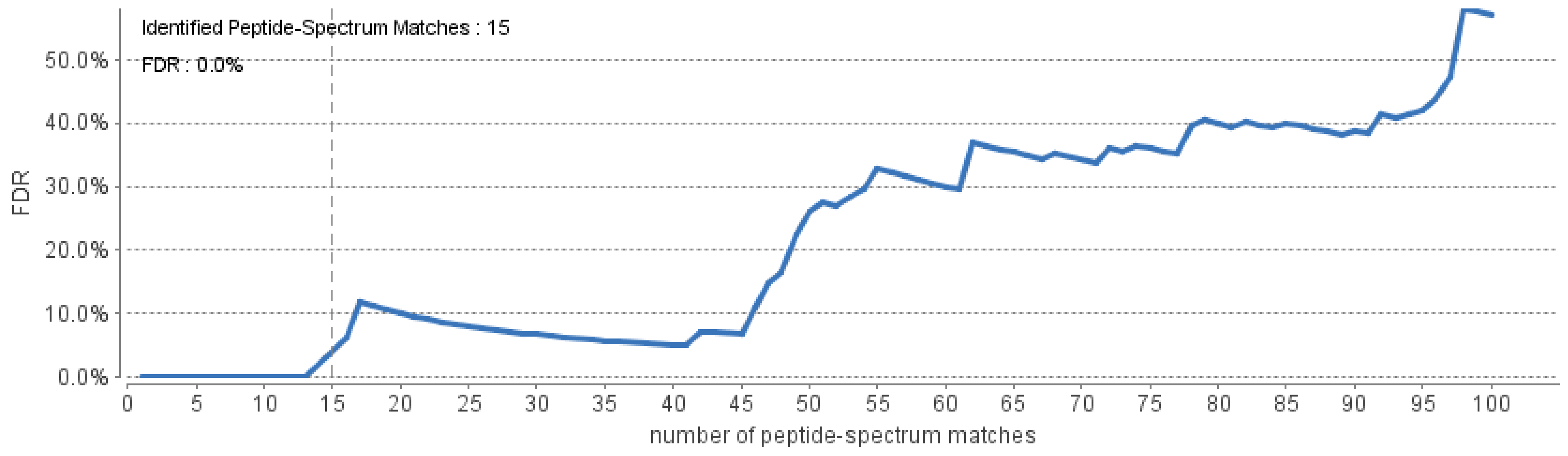

Figure 2. PSM score distribution. **(a)** Distribution of PEAKS peptide score; **(b)** Scatterplot of PEAKS peptide score versus precursor mass error.

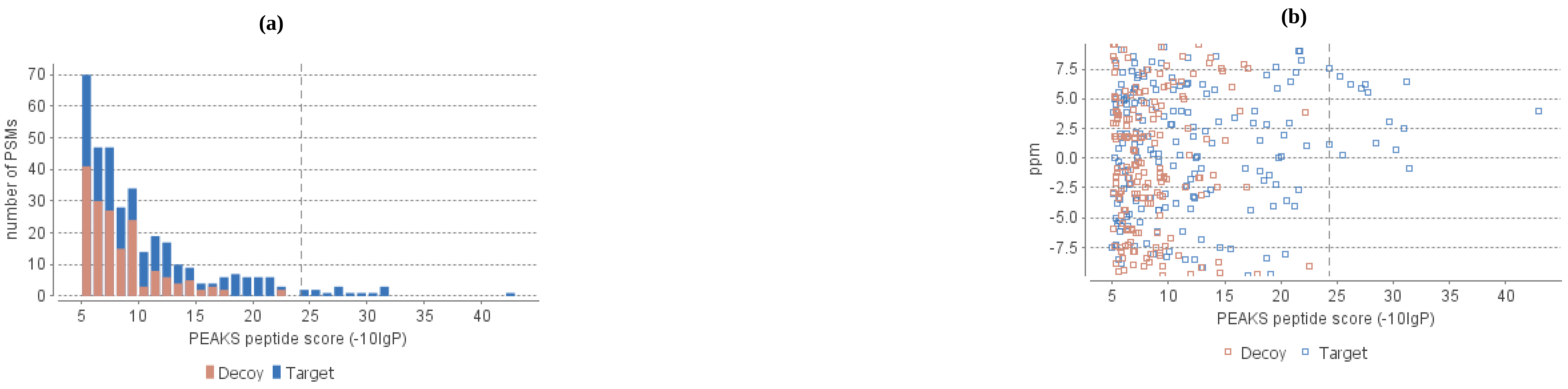

Figure 3. Distribution of peptide feature detection. **(a)** Feature m/z distribution; **(b)** Feature RT distribution

(a)

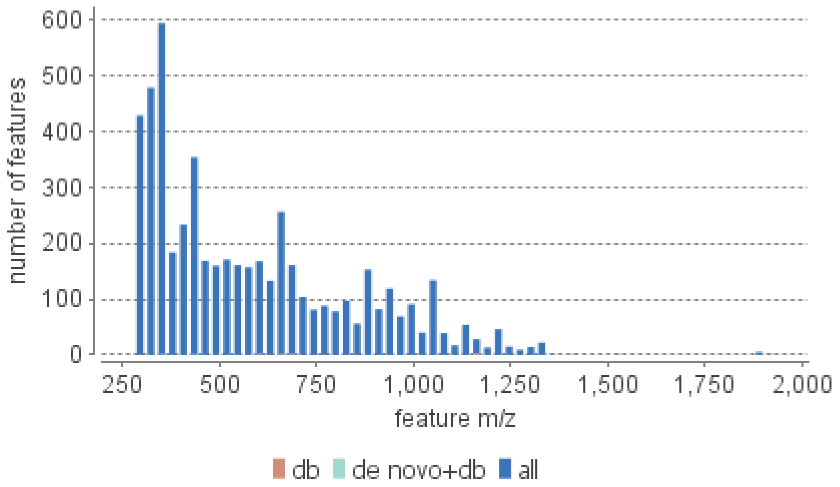

(b)

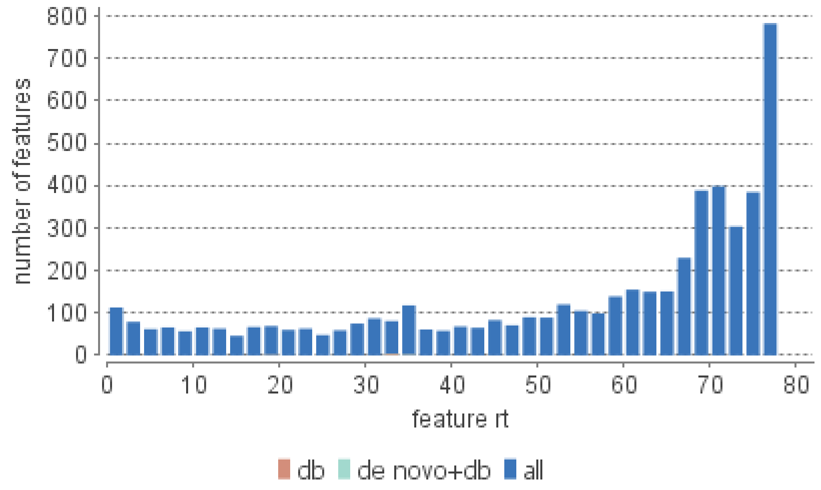

Figure 4. Distribution of identified peptide features. **(a)** Feature abundance distribution **(b)** *De novo* sequencing validation.

(a)

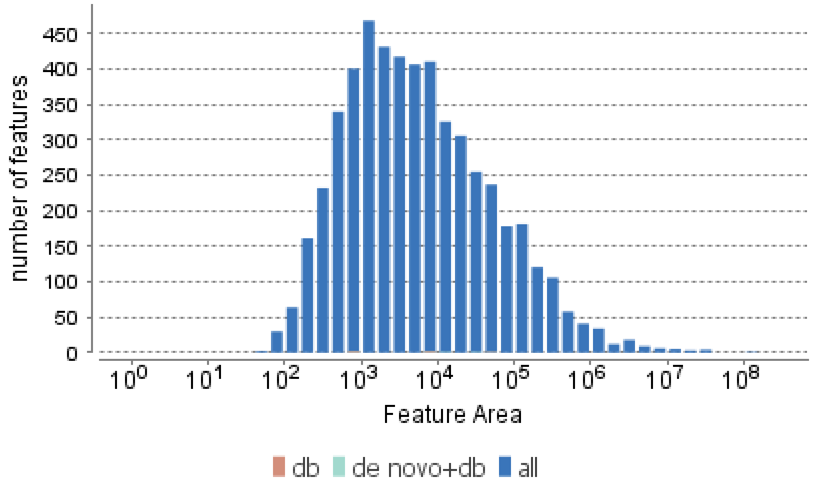

(b)

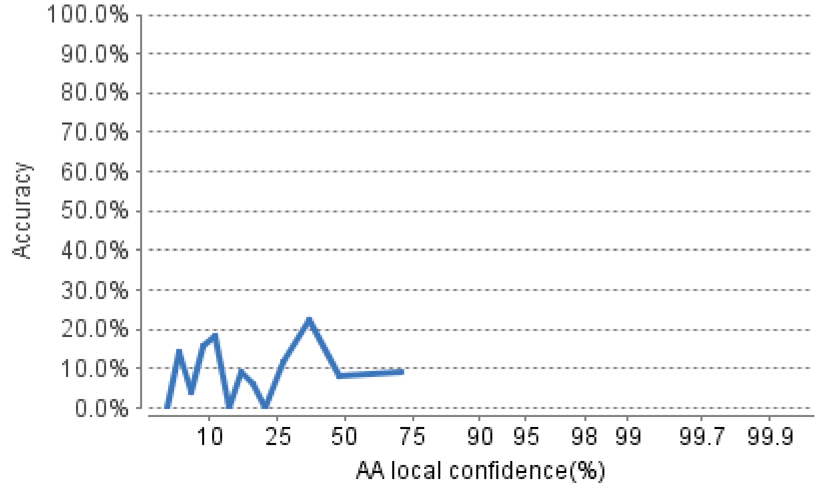

Table 1. Statistics of data.

# of MS scans	3005
# of MS/MS scans	1307
# of Features	5268
# of Chimera scans	117

Table 2. Result filtration parameters.

Peptide -10lgP	≥24.3

Table 4. PTM profile.

Name	ΔMass	Position	#PSM	-10lgP	Abundance	AScore
Deamidation	.98	NQ	11	31.40		0.00
Oxidation	15.99	M	2	31.17	7.51E3	1000.00
Dihydroxy	31.99	K	1	24.30	8.97E2	4.05

PTM Ascore	≥20
Protein -10lgP	≥20
Proteins unique peptides	≥1
De novo ALC Score	≥50%

Table 3. Statistics of filtered result.

Peptide-Spectrum Matches	15
Peptide sequences	10
Protein groups	2
Proteins	7
Proteins (#Unique Peptides)	6 (>2); 0 (=2); 1 (=1);
FDR (Peptide-Spectrum Matches)	0.0%
FDR (Peptide Sequences)	0.0%
FDR (Protein Group)	0.0%
De Novo Only Spectra	2
# of identified Features	7
# of identified MS/MS scans	15

3. Experiment Control

Figure 5. Precursor mass error of peptide-spectrum matches (PSM) in filtered result. **(a)** Distribution of precursor mass error in ppm; **(b)** Scatterplot of precursor m/z versus precursor mass error in ppm.

(a)

(b)

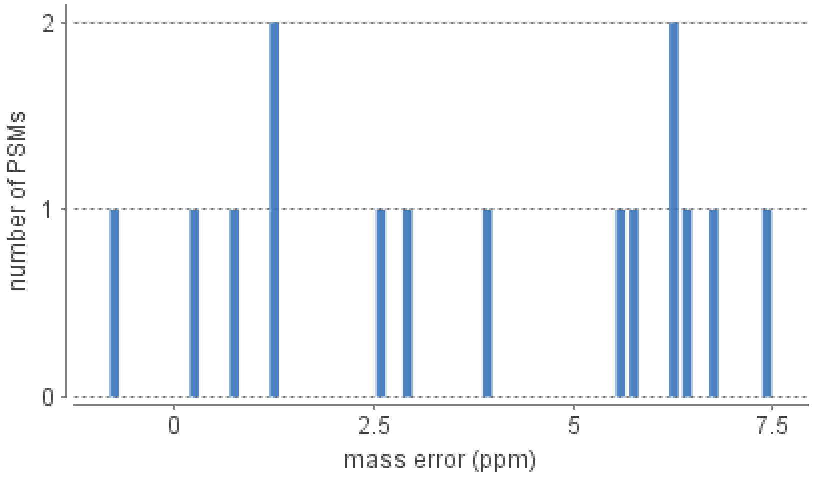

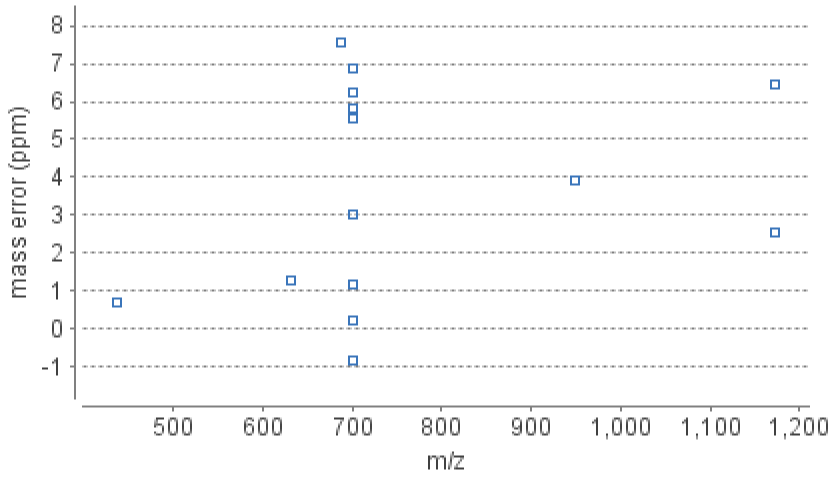

Table 5. Number of identified peptides in each sample by the number of missed cleavages

Missed Cleavages	0	1	2	3	4+
Digest 14. traka 67 kDa	1	4	5	0	0

4. Other Information

Table 6. Search parameters.

Search Engine Name: PEAKS
Parent Mass Error Tolerance: 10.0 ppm
Fragment Mass Error Tolerance: 0.5 Da
Precursor Mass Search Type: monoisotopic
Enzyme: Trypsin
Max Missed Cleavages: 2
Digest Mode: Unspecific
Fixed Modifications:
  Carbamidomethylation: 57.02
Variable Modifications:
  Deamidation (NQ): 0.98
  Oxidation (M): 15.99
  Hydroxylation Pro: 15.99
  Acetylation (K): 42.01
  Acetylation (Protein N-term): 42.01
  Acetylation (N-term): 42.01
  Amidation: -0.98
  Beta-methylthiolation: 45.99
  and 305 more...
Max Variable PTM Per Peptide: 5

Table 7. Instrument parameters.

Fractions: OB4064.raw
Ion Source: ESI(nano-spray)
Fragmentation Mode: CID, CAD(y and b ions)
MS Scan Mode: FT-ICR/Orbitrap
MS/MS Scan Mode: Linear Ion Trap

Database: Uniprot_Peanut-3818_Jul18
Taxon: All
Contaminant Database: contaminantsMQ_mar19
Searched Entry: 1723
FDR Estimation: Enabled
De novo score (ALC%) threshold: 15
Peptide hit threshold (-10logP): 30.0
Peaks run ID: 486
Merge Options: no merge
Precursor Options: corrected
Charge Options: no correction
Filter Charge: 2 - 8
Process: true
Associate chimera: yes

Protein List

Protein Accession Contains:
Protein Description Contains:
Peptide Sample Area >=
Protein Ptm Contains:

Protein Group	Protein ID	Accession	-10lgP	Coverage (%)	Coverage (%) Digest 14. traka 67 kDa	Area Digest 14. traka 67 kDa	#Peptides	#Unique	#Spec Digest 14. traka 67 kDa	PTM	Avg. Mass	Description
**1**	20853	tr|N1NG13|N1NG13_ARAHY	75.17	14	14	1.09E5	4	4	14	Y	71345	Seed storage protein Ara h1 OS=Arachis hypogaea OX=3818 GN=ARAX_AHF417E07-017 PE=4 SV=1
**1**	20854	P43238|ALL12_ARAHY	75.17	14	14	1.09E5	4	4	14	Y	71345	Allergen Ara h 1, clone P41B OS=Arachis hypogaea OX=3818 PE=1 SV=1
**1**	22400	tr|Q6PSU4|Q6PSU4_ARAHY	75.17	21	21	1.09E5	4	4	14	Y	48095	Conarachin (Fragment) OS=Arachis hypogaea OX=3818 PE=2 SV=1
**1**	20849	tr|Q6PSU3|Q6PSU3_ARAHY	75.17	15	15	1.09E5	4	4	14	Y	66575	Conarachin (Fragment) OS=Arachis hypogaea OX=3818 PE=4 SV=1
**1**	20850	P43237|ALL11_ARAHY	75.17	14	14	1.09E5	4	4	14	Y	70283	Allergen Ara h 1, clone P17 OS=Arachis hypogaea OX=3818 PE=1 SV=1
**1**	20851	tr|B3IXL2|B3IXL2_ARAHY	75.17	14	14	1.09E5	4	4	14	Y	70283	Main allergen Ara h1 OS=Arachis hypogaea OX=3818 PE=2 SV=1
**2**	21956	tr|E5FHY1|E5FHY1_ARAHY	30.20	14	14	0E0	1	1	1	N	10140	Late embryogenesis abundant protein group 1 protein OS=Arachis hypogaea OX=3818 GN=LEA1-2 PE=2 SV=1
**total 7 proteins**												

tr|N1NG13|N1NG13_ARAHY
back to list

| Protein Coverage | Supporting Peptides |

Protein Coverage:

1 MRGRVSPLML LLGILVLASV SATHAKSSPY QKKTENPCAQ RCLQSCQQEP DDLKQKACES RCTKLEYDPR CVYDPRGHTG
81 TTNQRSPPGE RTRGRQPGDY DDDRRQPRRE EGGRWGPAGP REREREEDWR QPREDWRRPS HQQPRKIRPE GREGEQEWGT
161 PGSHVREETS RNNPFYFPSR RFSTRYGNQN GRIRVLQRFD QRSRQFQNLQ NHRIVQIEAK PNTLVLPKHA DADNILVIQQ
241 GQATVTVANG NNRK**SFNLDE GHALRIPSGF ISYILNRHDN QNLR**VAK**ISM PVNTPGQFED FFPASSRDQS SYLQGFSRNT**
321 **LEAAFNAEFN EIRR**VLLEEN AGGEQEERGQ RRWSTRSSEN NEGVIVKVSK EHVEELTKHA KSVSKKGSEE EGDITNPINL
401 REGEPDLSNN FGKLFEVKPD KKNPQLQDLD MMLTCVEIK**E GALMLPHFNS K**AMVIVVVNK GTGNLELVAV RKEQQQRGRR
481 EEEEDEDEEE EGSNREVRRY TARLKEGDVF IMPAAHPVAI NASSELHLLG FGINAENNHR IFLAGDKDNV IDQIEKQAKD
561 LAFPGSGEQV EKLIKNQKES HFVSARPQSQ SQSPSSPEKE SPEKEDQEEE NQGGKGPLLS ILKAFN

276 d; 290 o; 293 d

d Deamidation (NQ) (+0.98)
o Oxidation (M) (+15.99)

Supporting Peptides:

Peptide	Uniq	-10lgP	Mass	Length	ppm	m/z	z	RT	Fraction	Scan	Source File	Area Digest 14. traka 67 kDa	#Feature	#Feature Digest 14. traka 67 kDa	Start	End	PTM
R.NTLEAAFNAEFNEIRR.V	Y	43.01	1893.9332	16	3.9	947.9776	2	33.04	36	1823	OB4064.raw	7.01E3	1	1	319	334	
K.SFNLDEGHALRIPSGFISYILN(+.98)RHDNQN(+.98)LR.V	Y	31.40	3497.7439	30	-0.8	700.5555	5	35.06	36	1948	OB4064.raw	4.66E4	1	1	255	284	
K.ISM(+15.99)PVN(+.98)TPGQFEDFFPASSRDQ(+.98)SSYLQGFSR.N	Y	31.17	3512.5830	31	6.5	1171.8759	3	33.88	36	1874	OB4064.raw	7.51E3	1	1	288	318	Oxidation (M)
K.ISM(+15.99)PVN(+.98)TPGQFEDFFPASSRDQ(+.98)SSYLQ(+.98)GFSR.N	Y	31.02	3513.5669	31	2.5	1172.1992	3	34.36	36	1906	OB4064.raw	7.78E2	1	1	288	318	Oxidation (M); Deamidation (NQ)
K.SFN(+.98)LDEGHALRIPSGFISYILNRHDNQN(+.98)LR.V	Y	29.62	3497.7439	30	3.0	700.5582	5	35.41	36	1970	OB4064.raw	0	0	0	255	284	
K.SFN(+.98)LDEGHALRIPSGFISYILN(+.98)RHDNQ(+.98)NLR.V	Y	27.81	3498.7280	30	5.6	700.7568	5	34.81	36	1932	OB4064.raw	0	0	0	255	284	Deamidation (NQ)
K.SFN(+.98)LDEGHALRIPSGFISYILNRHDNQ(+.98)NLR.V	Y	27.51	3497.7439	30	6.2	700.5604	5	33.95	36	1886	OB4064.raw	4.66E4	1	1	255	284	
K.SFN(+.98)LDEGHALRIPSGFISYILN(+.98)RHDNQN(+.98)LR.V	Y	27.18	3498.7280	30	5.8	700.7570	5	34.49	36	1913	OB4064.raw	0	0	0	255	284	
K.EGALMLPHFNSK(+31.99).A	Y	24.30	1374.6602	12	7.5	688.3425	2	19.81	36	1068	OB4064.raw	8.97E2	1	1	440	451	
**total 9 peptides**																	

P43238|ALL12_ARAHY

back to list

| Protein Coverage | Supporting Peptides |

Protein Coverage:

1 MRGRVSPLML LLGILVLASV SATHAKSSPY QKKTENPCAQ RCLQSCQQEP DDLKQKACES RCTKLEYDPR CVYDPRGHTG
81 TTNQRSPPGE RTRGRQPGDY DDDRRQPRRE EGGRWGPAGP REREREEDWR QPREDWRRPS HQQPRKIRPE GREGEQEWGT
161 PGSHVREETS RNNPFYFPSR RFSTRYGNQN GRIRVLQRFD QRSRQFQNLQ NHRIVQIEAK PNTLVLPKHA DADNILVIQQ
241 GQATVTVANG NNRK**SFNLDE GHALRIPSGF ISYILNRHDN QNLR**VAK**ISM PVNTPGQFED FFPASSRDQS SYLQGFSRNT**
321 **LEAAFNAEFN EIRR**VLLEEN AGGEQEERGQ RRWSTRSSEN NEGVIVKVSK EHVEELTKHA KSVSKKGSEE EGDITNPINL
401 REGEPDLSNN FGKLFEVKPD KKNPQLQDLD MMLTCVEIK**E GALMLPHFNS K**AMVIVVVNK GTGNLELVAV RKEQQQRGRR
481 EEEEDEDEEE EGSNREVRRY TARLKEGDVF IMPAAHPVAI NASSELHLLG FGINAENNHR IFLAGDKDNV IDQIEKQAKD
561 LAFPGSGEQV EKLIKNQKES HFVSARPQSQ SQSPSSPEKE SPEKEDQEEE NQGGKGPLLS ILKAFN

d Deamidation (NQ) (+0.98)
o Oxidation (M) (+15.99)

Supporting Peptides:

Peptide	Uniq	-10lgP	Mass	Length	ppm	m/z	z	RT	Fraction	Scan	Source File	Area Digest 14. traka 67 kDa	#Feature	#Feature Digest 14. traka 67 kDa	Start	End	PTM
R.NTLEAAFNAEFNEIRR.V	Y	43.01	1893.9332	16	3.9	947.9776	2	33.04	36	1823	OB4064.raw	7.01E3	1	1	319	334	
K.SFNLDEGHALRIPSGFISYILN(+.98)RHDNQN(+.98)LR.V	Y	31.40	3497.7439	30	-0.8	700.5555	5	35.06	36	1948	OB4064.raw	4.66E4	1	1	255	284	
K.ISM(+15.99)PVN(+.98)TPGQFEDFFPASSRDQ(+.98)SSYLQGFSR.N	Y	31.17	3512.5830	31	6.5	1171.8759	3	33.88	36	1874	OB4064.raw	7.51E3	1	1	288	318	Oxidation (M)
K.ISM(+15.99)PVN(+.98)TPGQFEDFFPASSRDQ(+.98)SSYLQ(+.98)GFSR.N	Y	31.02	3513.5669	31	2.5	1172.1992	3	34.36	36	1906	OB4064.raw	7.78E2	1	1	288	318	Oxidation (M); Deamidation (NQ)
K.SFN(+.98)LDEGHALRIPSGFISYILNRHDNQN(+.98)LR.V	Y	29.62	3497.7439	30	3.0	700.5582	5	35.41	36	1970	OB4064.raw	0	0	0	255	284	
K.SFN(+.98)LDEGHALRIPSGFISYILN(+.98)RHDNQ(+.98)NLR.V	Y	27.81	3498.7280	30	5.6	700.7568	5	34.81	36	1932	OB4064.raw	0	0	0	255	284	Deamidation (NQ)
K.SFN(+.98)LDEGHALRIPSGFISYILNRHDNQ(+.98)NLR.V	Y	27.51	3497.7439	30	6.2	700.5604	5	33.95	36	1886	OB4064.raw	4.66E4	1	1	255	284	
K.SFN(+.98)LDEGHALRIPSGFISYILN(+.98)RHDNQN(+.98)LR.V	Y	27.18	3498.7280	30	5.8	700.7570	5	34.49	36	1913	OB4064.raw	0	0	0	255	284	
K.EGALMLPHFNSK(+31.99).A	Y	24.30	1374.6602	12	7.5	688.3425	2	19.81	36	1068	OB4064.raw	8.97E2	1	1	440	451	
**total 9 peptides**																	

tr|Q6PSU4|Q6PSU4_ARAHY

back to list

| Protein Coverage | Supporting Peptides |

Protein Coverage:

1 GFDQRSRQFQ NLQNHRIVQI EAKPNTLVLP KHADADNILV IQQGQATVTV ANGNNRK**SFN LDEGHALRIP SGFISYILNR**
81 **HDNQNLR**VAK **ISMPVNTPGQ FEDFFPASSR DQSSYLQGFS RNTLEAAFNA EFNEIRR**VLL EENAGGEQEE RGQRRWSTRS
161 SENNEGVIVK VSKEHVEELT KHAKSVSKKG SEEGDITNPI NLREGEPDLS NNFGKLFEVK PDKKNPQLQD LDMMLTCVEI
241 K**EGALMLPHF NSK**AMVIVVV NKGTGNLELV AVRKEQQQRG RREEEEDEDE EEEGSNREVR RYTARLKEGD VFIMPAAHPV
321 AINASSELHL LGFGINAENN HRIFLAGDKD NVIDQIEKQA KDLAFPGSGE QVEKLIKNQK ESHFVSARPQ SQSQSPSSPE
401 KESPEKEDQE EENQGGKGPL LSILKAFN

d Deamidation (NQ) (+0.98)
o Oxidation (M) (+15.99)

Supporting Peptides:

Peptide	Uniq	-10lgP	Mass	Length	ppm	m/z	z	RT	Fraction	Scan	Source File	Area Digest 14. traka 67 kDa	#Feature	#Feature Digest 14. traka 67 kDa	Start	End	PTM
R.NTLEAAFNAEFNEIRR.V	Y	43.01	1893.9332	16	3.9	947.9776	2	33.04	36	1823	OB4064.raw	7.01E3	1	1	122	137	
K.SFNLDEGHALRIPSGFISYILN(+.98)RHDNQN(+.98)LR.V	Y	31.40	3497.7439	30	-0.8	700.5555	5	35.06	36	1948	OB4064.raw	4.66E4	1	1	58	87	
K.ISM(+15.99)PVN(+.98)TPGQFEDFFPASSRDQ(+.98)SSYLQGFSR.N	Y	31.17	3512.5830	31	6.5	1171.8759	3	33.88	36	1874	OB4064.raw	7.51E3	1	1	91	121	Oxidation (M)
K.ISM(+15.99)PVN(+.98)TPGQFEDFFPASSRDQ(+.98)SSYLQ(+.98)GFSR.N	Y	31.02	3513.5669	31	2.5	1172.1992	3	34.36	36	1906	OB4064.raw	7.78E2	1	1	91	121	Oxidation (M); Deamidation (NQ)
K.SFN(+.98)LDEGHALRIPSGFISYILNRHDNQN(+.98)LR.V	Y	29.62	3497.7439	30	3.0	700.5582	5	35.41	36	1970	OB4064.raw	0	0	0	58	87	
K.SFN(+.98)LDEGHALRIPSGFISYILN(+.98)RHDNQ(+.98)NLR.V	Y	27.81	3498.7280	30	5.6	700.7568	5	34.81	36	1932	OB4064.raw	0	0	0	58	87	Deamidation (NQ)
K.SFN(+.98)LDEGHALRIPSGFISYILNRHDNQ(+.98)NLR.V	Y	27.51	3497.7439	30	6.2	700.5604	5	33.95	36	1886	OB4064.raw	4.66E4	1	1	58	87	
K.SFN(+.98)LDEGHALRIPSGFISYILN(+.98)RHDNQN(+.98)LR.V	Y	27.18	3498.7280	30	5.8	700.7570	5	34.49	36	1913	OB4064.raw	0	0	0	58	87	
K.EGALMLPHFNSK(+31.99).A	Y	24.30	1374.6602	12	7.5	688.3425	2	19.81	36	1068	OB4064.raw	8.97E2	1	1	242	253	
**total 9 peptides**																	

tr|Q6PSU3|Q6PSU3_ARAHY
back to list

| Protein Coverage | Supporting Peptides |

Protein Coverage:

```
  1 MRGRVSPLML LLGILVLASV SATQAKSPYR KTENPCAQRC LQSCQQEPDD LKQKACESRC TKLEYDPRCV YDTGATNQRH
 81 PPGERTRGRQ PGDYDDDRRQ PRREEGGRWG PAEPRERERE EDWRQPREDW RRPSHQQPRK IRPEGREGEQ EWGTPGSEVR
161 EETSRNNPFY FPSRRFSTRY GNQNGRIRVL QRFDQRSKQF QNLQNHRIVQ IEARPNTLVL PKHADADNIL VIQQGQATVT
241 VANGNNRKSF NLDEGHALRI PSGFISYILN RHDNQNLRVA KISMPVNTPG QFEDFFPASS RDQSSYLQGF SRNTLEAAFN
321 AEFNEIRRVL LEENAGGEQE ERGQRRRSTR SSDNEGVIVK VSKEHVQELT KHAKSVSKKG SEEEDITNPI NLRDGEPDLS
401 NNFGRLFEVK PDKKNPQLQD LDMMLTCVEI KEGALMLPHF NSKAMVIVVV NKGTGNLELV AVRKEQQQRG RREQEWEEEE
481 EDEEEEGSNR EVRRYTARLK EGDVFIMPAA HPVAINASSE LHLLGFGINA ENNHRIFLAG DKDNVIDQIE KQAKDLAFPG
561 SGEQVEKLIK NQRESHFVSA
```

Modification sites: 270 d, 284 o, 287 d

d Deamidation (NQ) (+0.98)
o Oxidation (M) (+15.99)

Supporting Peptides:

Peptide	Uniq	-10lgP	Mass	Length	ppm	m/z	z	RT	Fraction	Scan	Source File	Area Digest 14. traka 67 kDa	#Feature	#Feature Digest 14. traka 67 kDa	Start	End	PTM
R.NTLEAAFNAEFNEIRR.V	Y	43.01	1893.9332	16	3.9	947.9776	2	33.04	36	1823	OB4064.raw	7.01E3	1	1	313	328	
K.SFNLDEGHALRIPSGFISYILN(+.98)RHDNQN(+.98)LR.V	Y	31.40	3497.7439	30	-0.8	700.5555	5	35.06	36	1948	OB4064.raw	4.66E4	1	1	249	278	
K.ISM(+15.99)PVN(+.98)TPGQFEDFFPASSRDQ(+.98)SSYLQGFSR.N	Y	31.17	3512.5830	31	6.5	1171.8759	3	33.88	36	1874	OB4064.raw	7.51E3	1	1	282	312	Oxidation (M)
K.ISM(+15.99)PVN(+.98)TPGQFEDFFPASSRDQ(+.98)SSYLQ(+.98)GFSR.N	Y	31.02	3513.5669	31	2.5	1172.1992	3	34.36	36	1906	OB4064.raw	7.78E2	1	1	282	312	Oxidation (M); Deamidation (NQ)
K.SFN(+.98)LDEGHALRIPSGFISYILNRHDNQN(+.98)LR.V	Y	29.62	3497.7439	30	3.0	700.5582	5	35.41	36	1970	OB4064.raw	0	0	0	249	278	
K.SFN(+.98)LDEGHALRIPSGFISYILN(+.98)RHDNQ(+.98)NLR.V	Y	27.81	3498.7280	30	5.6	700.7568	5	34.81	36	1932	OB4064.raw	0	0	0	249	278	Deamidation (NQ)
K.SFN(+.98)LDEGHALRIPSGFISYILNRHDNQ(+.98)NLR.V	Y	27.51	3497.7439	30	6.2	700.5604	5	33.95	36	1886	OB4064.raw	4.66E4	1	1	249	278	
K.SFN(+.98)LDEGHALRIPSGFISYILN(+.98)RHDNQN(+.98)LR.V	Y	27.18	3498.7280	30	5.8	700.7570	5	34.49	36	1913	OB4064.raw	0	0	0	249	278	
K.EGALMLPHFNSK(+31.99).A	Y	24.30	1374.6602	12	7.5	688.3425	2	19.81	36	1068	OB4064.raw	8.97E2	1	1	432	443	

Peptide	Uniq	-10lgP	Mass	Length	ppm	m/z	z	RT	Fraction	Scan	Source File	Area Digest 14. traka 67 kDa	#Feature	#Feature Digest 14. traka 67 kDa	Start	End	PTM
**total 9 peptides**																	

P43237|ALL11_ARAHY
back to list

| Protein Coverage | Supporting Peptides |

Protein Coverage:

```
  1  MRGRVSPLML LLGILVLASV SATQAKSPYR KTENPCAQRC LQSCQQEPDD LKQKACESRC TKLEYDPRCV YDTGATNQRH
 81  PPGERTRGRQ PGDYDDDRRQ PRREEGGRWG PAEPRERERE EDWRQPREDW RRPSHQQPRK IRPEGREGEQ EWGTPGSEVR
161  EETSRNNPFY FPSRRFSTRY GNQNGRIRVL QRFDQRSKQF QNLQNHRIVQ IEARPNTLVL PKHADADNIL VIQQGQATVT
241  VANGNNRKSF NLDEGHALRI PSGFISYILN RHDNQNLRVA KISMPVNTPG QFEDFFPASS RDQSSYLQGF SRNTLEAAFN
321  AEFNEIRRVL LEENAGGEQE ERGQRRRSTR SSDNEGVIVK VSKEHVQELT KHAKSVSKKG SEEEDITNPI NLRDGEPDLS
401  NNFGRLFEVK PDKKNPQLQD LDMMLTCVEI KEGALMLPHF NSKAMVIVVV NKGTGNLELV AVRKEQQQRG RREQEWEEEE
481  EDEEEEGSNR EVRRYTARLK EGDVFIMPAA HPVAINASSE LHLLGFGINA ENNHRIFLAG DKDNVIDQIE KQAKDLAFPG
561  SGEQVEKLIK NQRESHFVSA RPQSQSPSSP EKEDQEEENQ GGKGPLLSIL KAFN
```

Modification markers above line 241: 270 d; 284 o; 287 d

d Deamidation (NQ) (+0.98)
o Oxidation (M) (+15.99)

Supporting Peptides:

Peptide	Uniq	-10lgP	Mass	Length	ppm	m/z	z	RT	Fraction	Scan	Source File	Area Digest 14. traka 67 kDa	#Feature	#Feature Digest 14. traka 67 kDa	Start	End	PTM
R.NTLEAAFNAEFNEIRR.V	Y	43.01	1893.9332	16	3.9	947.9776	2	33.04	36	1823	OB4064.raw	7.01E3	1	1	313	328	
K.SFNLDEGHALRIPSGFISYILN(+.98)RHDNQN(+.98)LR.V	Y	31.40	3497.7439	30	-0.8	700.5555	5	35.06	36	1948	OB4064.raw	4.66E4	1	1	249	278	
K.ISM(+15.99)PVN(+.98)TPGQFEDFFPASSRDQ(+.98)SSYLQGFSR.N	Y	31.17	3512.5830	31	6.5	1171.8759	3	33.88	36	1874	OB4064.raw	7.51E3	1	1	282	312	Oxidation (M)
K.ISM(+15.99)PVN(+.98)TPGQFEDFFPASSRDQ(+.98)SSYLQ(+.98)GFSR.N	Y	31.02	3513.5669	31	2.5	1172.1992	3	34.36	36	1906	OB4064.raw	7.78E2	1	1	282	312	Oxidation (M); Deamidation (NQ)
K.SFN(+.98)LDEGHALRIPSGFISYILNRHDNQN(+.98)LR.V	Y	29.62	3497.7439	30	3.0	700.5582	5	35.41	36	1970	OB4064.raw	0	0	0	249	278	
K.SFN(+.98)LDEGHALRIPSGFISYILN(+.98)RHDNQ(+.98)NLR.V	Y	27.81	3498.7280	30	5.6	700.7568	5	34.81	36	1932	OB4064.raw	0	0	0	249	278	Deamidation (NQ)
K.SFN(+.98)LDEGHALRIPSGFISYILNRHDNQ(+.98)NLR.V	Y	27.51	3497.7439	30	6.2	700.5604	5	33.95	36	1886	OB4064.raw	4.66E4	1	1	249	278	
K.SFN(+.98)LDEGHALRIPSGFISYILN(+.98)RHDNQN(+.98)LR.V	Y	27.18	3498.7280	30	5.8	700.7570	5	34.49	36	1913	OB4064.raw	0	0	0	249	278	
K.EGALMLPHFNSK(+31.99).A	Y	24.30	1374.6602	12	7.5	688.3425	2	19.81	36	1068	OB4064.raw	8.97E2	1	1	432	443	
**total 9 peptides**																	

tr|B3IXL2|B3IXL2_ARAHY
back to list

| Protein Coverage | Supporting Peptides |

Protein Coverage:

1 MRGRVSPLML LLGILVLASV SATQAKSPYR KTENPCAQRC LQSCQQEPDD LKQKACESRC TKLEYDPRCV YDTGATNQRH

81 PPGERTRGRQ PGDYDDDRRQ PRREEGGRWG PAEPRERERE EDWRQPREDW RRPSHQQPRK IRPEGREGEQ EWGTPGSEVR

161 EETSRNNPFY FPSRRFSTRY GNQNGRIRVL QRFDQRSKQF QNLQNHRIVQ IEARPNTLVL PKHADADNIL VIQQGQATVT

241 VANGNNRK**SF NLDEGHALRI PSGFISYILN RHDNQNLR**VA K**ISMPVNTPG QFEDFFPASS RDQSSYLQGF SRNTLEAAFN**

321 **AEFNEIRR**VL LEENAGGEQE ERGQRRRSTR SSDNEGVIVK VSKEHVQELT KHAKSVSKKG SEEEDITNPI NLRDGEPDLS

401 NNFGRLFEVK PDKKNPQLQD LDMMLTCVEI K**EGALMLPHF NSK**AMVIVVV NKGTGNLELV AVRKEQQQRG RREQEWEEEE

481 EDEEEEGSNR EVRRYTARLK EGDVFIMPAA HPVAINASSE LHLLGFGINA ENNHRIFLAG DKDNVIDQIE KQAKDLAFPG

561 SGEQVEKLIK NQRESHFVSA RPQSQSPSSP EKEDQEEENQ GGKGPLLSIL KAFN

d Deamidation (NQ) (+0.98)
o Oxidation (M) (+15.99)

Supporting Peptides:

Peptide	Uniq	-10lgP	Mass	Length	ppm	m/z	z	RT	Fraction	Scan	Source File	Area Digest 14. traka 67 kDa	#Feature	#Feature Digest 14. traka 67 kDa	Start	End	PTM
R.NTLEAAFNAEFNEIRR.V	Y	43.01	1893.9332	16	3.9	947.9776	2	33.04	36	1823	OB4064.raw	7.01E3	1	1	313	328	
K.SFNLDEGHALRIPSGFISYILN(+.98)RHDNQN(+.98)LR.V	Y	31.40	3497.7439	30	-0.8	700.5555	5	35.06	36	1948	OB4064.raw	4.66E4	1	1	249	278	
K.ISM(+15.99)PVN(+.98)TPGQFEDFFPASSRDQ(+.98)SSYLQGFSR.N	Y	31.17	3512.5830	31	6.5	1171.8759	3	33.88	36	1874	OB4064.raw	7.51E3	1	1	282	312	Oxidation (M)
K.ISM(+15.99)PVN(+.98)TPGQFEDFFPASSRDQ(+.98)SSYLQ(+.98)GFSR.N	Y	31.02	3513.5669	31	2.5	1172.1992	3	34.36	36	1906	OB4064.raw	7.78E2	1	1	282	312	Oxidation (M); Deamidation (NQ)
K.SFN(+.98)LDEGHALRIPSGFISYILNRHDNQN(+.98)LR.V	Y	29.62	3497.7439	30	3.0	700.5582	5	35.41	36	1970	OB4064.raw	0	0	0	249	278	
K.SFN(+.98)LDEGHALRIPSGFISYILN(+.98)RHDNQ(+.98)NLR.V	Y	27.81	3498.7280	30	5.6	700.7568	5	34.81	36	1932	OB4064.raw	0	0	0	249	278	Deamidation (NQ)
K.SFN(+.98)LDEGHALRIPSGFISYILNRHDNQ(+.98)NLR.V	Y	27.51	3497.7439	30	6.2	700.5604	5	33.95	36	1886	OB4064.raw	4.66E4	1	1	249	278	
K.SFN(+.98)LDEGHALRIPSGFISYILN(+.98)RHDNQN(+.98)LR.V	Y	27.18	3498.7280	30	5.8	700.7570	5	34.49	36	1913	OB4064.raw	0	0	0	249	278	
K.EGALMLPHFNSK(+31.99).A	Y	24.30	1374.6602	12	7.5	688.3425	2	19.81	36	1068	OB4064.raw	8.97E2	1	1	432	443	
**total 9 peptides**																	

tr|E5FHY1|E5FHY1_ARAHY

back to list

| Protein Coverage | Supporting Peptides |

Protein Coverage:

1 MASKQRQDLN EKAKQGETVV PGGTGGKTLE AQEHLAEGRS KGGQTRKEQL GTEGYQEMGR K**GGLSTMEKS GGER**AEEEGV

81 EIDESKFATK NQNK

Supporting Peptides:

Peptide	Uniq	-10lgP	Mass	Length	ppm	m/z	z	RT	Fraction	Scan	Source File	Area Digest 14. traka 67 kDa	#Feature	#Feature Digest 14. traka 67 kDa	Start	End	PTM
K.GGLSTMEKSGGER.A	Y	30.20	1307.6139	13	0.7	436.8789	3	57.59	36	3183	OB4064.raw	0	0	0	62	74	
**total 1 peptides**																	

Peptide List

Summary

1. Notes Gastric Control Raw peanut Band #15 67-250 kDa (PTM469)

2. Result Statistics

Figure 1. False discovery rate (FDR) curve. X axis is the number of peptide-spectrum matches (PSM) being kept. Y axis is the corresponding FDR.

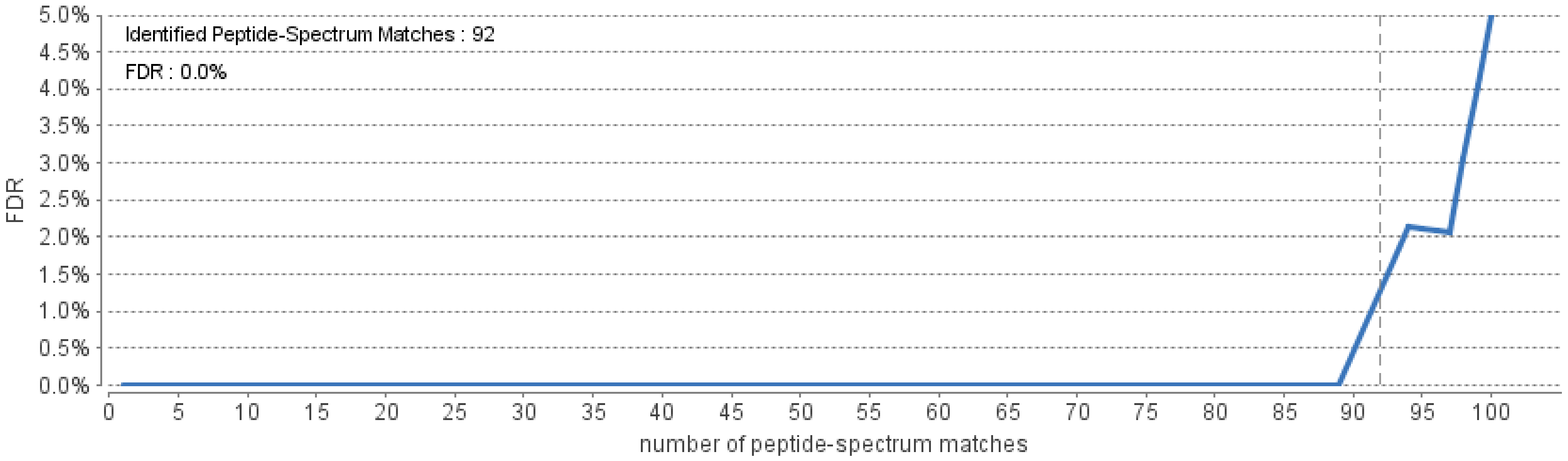

Figure 2. PSM score distribution. **(a)** Distribution of PEAKS peptide score; **(b)** Scatterplot of PEAKS peptide score versus precursor mass error.

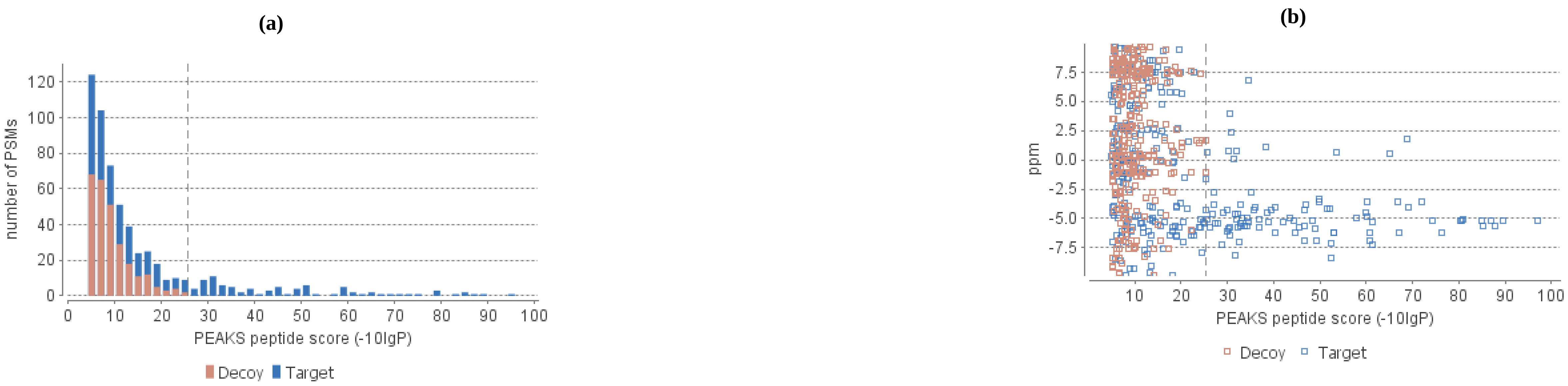

Figure 3. Distribution of peptide feature detection. **(a)** Feature m/z distribution; **(b)** Feature RT distribution

(a)

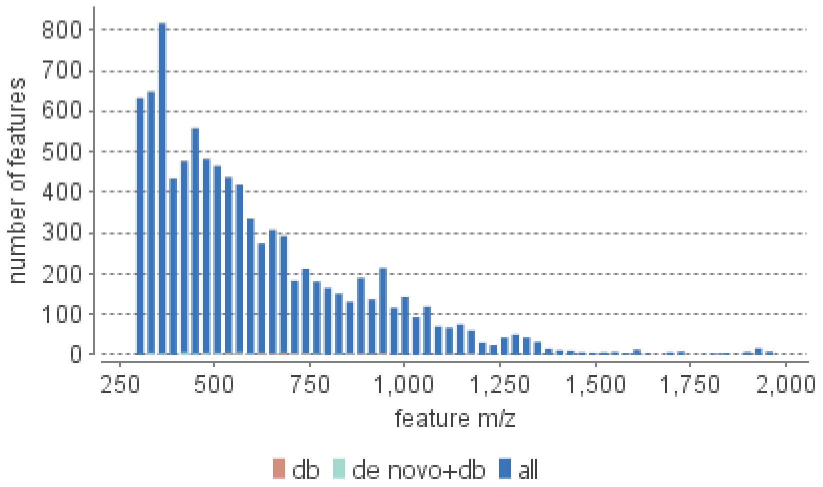

(b)

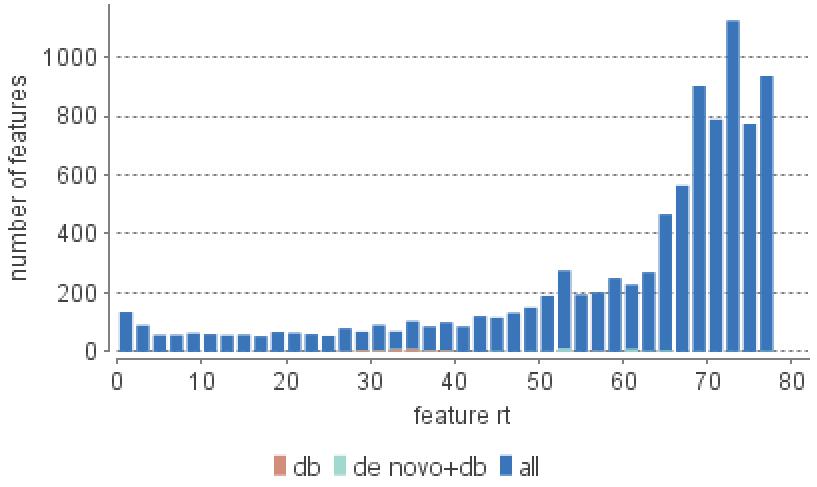

Figure 4. Distribution of identified peptide features. **(a)** Feature abundance distribution **(b)** *De novo* sequencing validation.

(a)

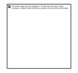

(b)

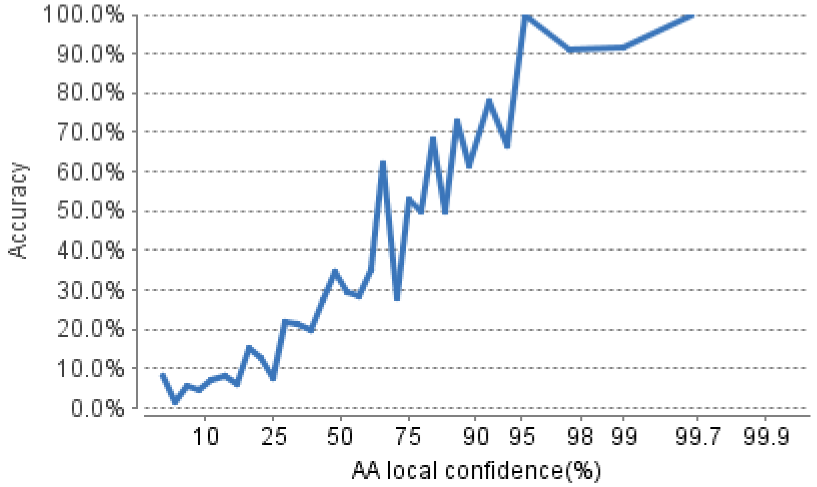

Table 1. Statistics of data.

# of MS scans	2790
# of MS/MS scans	1797
# of Features	9241
# of Chimera scans	194

Table 2. Result filtration parameters.

Peptide -10lgP	≥25.5
PTM Ascore	≥20

Table 4. PTM profile.

**Name**	**ΔMass**	**Position**	**#PSM**	**-10lgP**	**Abundance**	**AScore**
Deamidation	.98	NQ	43	72.00	1.16E4	12.72
Oxidation	15.99	M	34	72.00	1.16E4	1000.00
HydPro	15.99	P	5	52.86	5.17E3	1000.00
Amidine	41.03	N-term	3	52.86	5.17E3	1000.00

Protein -10lgP	≥20
Proteins unique peptides	≥1
De novo ALC Score	≥50%

Table 3. Statistics of filtered result.

Peptide-Spectrum Matches	92
Peptide sequences	47
Protein groups	6
Proteins	18
Proteins (#Unique Peptides)	3 (>2); 0 (=2); 15 (=1);
FDR (Peptide-Spectrum Matches)	0.0%
FDR (Peptide Sequences)	0.0%
FDR (Protein Group)	0.0%
De Novo Only Spectra	64
# of identified Features	78
# of identified MS/MS scans	90

3. Experiment Control

Figure 5. Precursor mass error of peptide-spectrum matches (PSM) in filtered result. **(a)** Distribution of precursor mass error in ppm; **(b)** Scatterplot of precursor m/z versus precursor mass error in ppm.

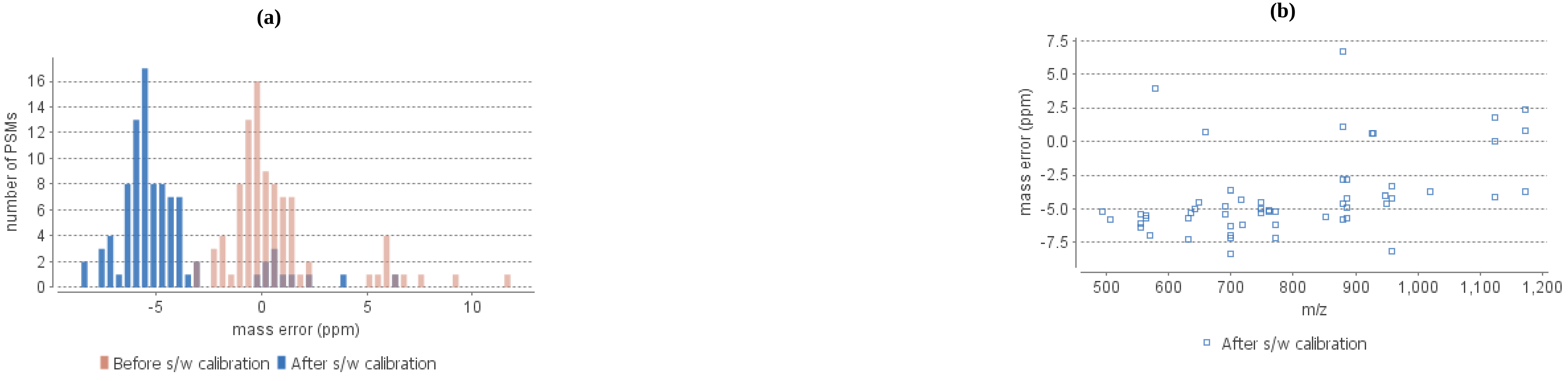

Table 5. Number of identified peptides in each sample by the number of missed cleavages

Missed Cleavages	0	1	2	3	4+
Kontrola 15. traka 67-100 kDa	31	16	0	0	0

4. Other Information

Table 6. Search parameters.

Search Engine Name: PEAKS
Parent Mass Error Tolerance: 10.0 ppm
Fragment Mass Error Tolerance: 0.5 Da
Precursor Mass Search Type: monoisotopic
Enzyme: Trypsin
Max Missed Cleavages: 2
Digest Mode: Unspecific
Fixed Modifications:
  Carbamidomethylation: 57.02
Variable Modifications:
  Deamidation (NQ): 0.98
  Oxidation (M): 15.99
  Hydroxylation Pro: 15.99
  Acetylation (K): 42.01
  Acetylation (Protein N-term): 42.01
  Acetylation (N-term): 42.01
  Amidation: -0.98
  Beta-methylthiolation: 45.99
  and 305 more...
Max Variable PTM Per Peptide: 5
Database: Uniprot_Peanut-3818_Jul18
Taxon: All
Contaminant Database: contaminantsMQ_mar19
Searched Entry: 1723
FDR Estimation: Enabled
De novo score (ALC%) threshold: 15
Peptide hit threshold (-10logP): 30.0
Peaks run ID: 452
Merge Options: no merge
Precursor Options: corrected
Charge Options: no correction
Filter Charge: 2 - 8
Process: true
Associate chimera: yes

Table 7. Instrument parameters.

Fractions: OB4044.raw
Ion Source: ESI(nano-spray)
Fragmentation Mode: CID, CAD(y and b ions)
MS Scan Mode: FT-ICR/Orbitrap
MS/MS Scan Mode: Linear Ion Trap

Protein List

Protein Accession Contains:

Protein Description Contains:

Peptide Sample Area >=

Protein Ptm Contains:

Protein Group	Protein ID	Accession	-10lgP	Coverage (%)	Coverage (%) Kontrola 15. traka 67-100 kDa	Area Kontrola 15. traka 67-100 kDa	#Peptides	#Unique	#Spec Kontrola 15. traka 67-100 kDa	PTM	Avg. Mass	Description
**2**	20850	P43237|ALL11_ARAHY	173.86	17	17	9.78E4	9	1	53	Y	70283	Allergen Ara h 1, clone P17 OS=Arachis hypogaea OX=3818 PE=1 SV=1
**2**	20851	tr|B3IXL2|B3IXL2_ARAHY	173.86	17	17	9.78E4	9	1	53	Y	70283	Main allergen Ara h1 OS=Arachis hypogaea OX=3818 PE=2 SV=1
**2**	20849	tr|Q6PSU3|Q6PSU3_ARAHY	173.86	18	18	9.78E4	9	1	53	Y	66575	Conarachin (Fragment) OS=Arachis hypogaea OX=3818 PE=4 SV=1
**1**	20853	tr|N1NG13|N1NG13_ARAHY	172.12	16	16	5.11E4	9	1	53	Y	71345	Seed storage protein Ara h1 OS=Arachis hypogaea OX=3818 GN=ARAX_AHF417E07-017 PE=4 SV=1
**1**	20854	P43238|ALL12_ARAHY	172.12	16	16	5.11E4	9	1	53	Y	71345	Allergen Ara h 1, clone P41B OS=Arachis hypogaea OX=3818 PE=1 SV=1
**3**	21031	tr|Q9M5D3|Q9M5D3_ARAHY	170.26	8	8	6.68E4	5	5	16	Y	97616	Lipoxygenase OS=Arachis hypogaea OX=3818 PE=2 SV=1
**3**	21032	tr|Q4JME6|Q4JME6_ARAHY	170.26	8	8	6.68E4	5	5	16	Y	97476	Lipoxygenase OS=Arachis hypogaea OX=3818 GN=Pnlox3 PE=2 SV=1
**3**	21033	tr|Q4JME7|Q4JME7_ARAHY	170.26	8	8	6.68E4	5	5	16	Y	97596	Lipoxygenase OS=Arachis hypogaea OX=3818 GN=Pnlox2 PE=2 SV=1
**4**	20579	tr|Q5I6T2|Q5I6T2_ARAHY	139.76	8	8	7.99E2	3	1	9	Y	60736	Arachin Ahy-4 OS=Arachis hypogaea OX=3818 PE=2 SV=1
**4**	20576	tr|Q9FZ11|Q9FZ11_ARAHY	139.76	8	8	7.99E2	3	1	9	Y	60449	Gly1 OS=Arachis hypogaea OX=3818 GN=Gly1 PE=2 SV=1
**4**	20581	tr|Q647H3|Q647H3_ARAHY	139.76	8	8	7.99E2	3	1	9	Y	61532	Arachin Ahy-2 OS=Arachis hypogaea OX=3818 PE=2 SV=1
**5**	20574	tr|A1DZF0|A1DZF0_ARAHY	126.79	8	8	4.28E3	3	1	8	Y	60375	Arachin 6 OS=Arachis hypogaea OX=3818 PE=2 SV=1
**5**	20578	tr|B5TYU1|B5TYU1_ARAHY	126.79	8	8	4.28E3	3	1	8	Y	60624	Arachin Arah3 isoform OS=Arachis hypogaea OX=3818 PE=1 SV=1
**5**	20580	tr|Q647H4|Q647H4_ARAHY	126.79	8	8	4.28E3	3	1	8	Y	61506	Arachin Ahy-1 OS=Arachis hypogaea OX=3818 PE=2 SV=1
**5**	20591	tr|Q6T2T4|Q6T2T4_ARAHY	126.79	8	8	4.28E3	3	1	8	Y	61499	Storage protein OS=Arachis hypogaea OX=3818 PE=2 SV=1
**6**	20593	tr|Q6IWG5|Q6IWG5_ARAHY	57.81	5	5	9.74E4	1	1	7	Y	58061	Glycinin (Fragment) OS=Arachis hypogaea OX=3818 PE=2 SV=1
**6**	20595	tr|E5G077|E5G077_ARAHY	57.81	5	5	9.74E4	1	1	7	Y	58305	Ara h 3 allergen OS=Arachis hypogaea OX=3818 GN=ara h 3 PE=3 SV=1
**6**	20594	tr|Q0GM57|Q0GM57_ARAHY	57.81	5	5	9.74E4	1	1	7	Y	58263	Iso-Ara h3 OS=Arachis hypogaea OX=3818 PE=2 SV=1
**total 18 proteins**												

P43237|ALL11_ARAHY
back to list

| Protein Coverage | Supporting Peptides |

Protein Coverage:

1 MRGRVSPLML LLGILVLASV SATQAKSPYR KTENPCAQRC LQSCQQEPDD LKQKACESRC TKLEYDPRCV YDTGATNQRH

81 PPGERTRGRQ PGDYDDDRRQ PRREEGGRWG PAEPRERERE EDWRQPREDW RRPSHQQPRK IRPEGREGEQ EWGTPGSEVR

161 EETSR**NNPFY FPSR**RFSTRY GNQNGRIRVL QRFDQRSKQF QNLQNHR**IVQ IEARPNTLVL PK**HADADNIL VIQQGQATVT

241 VANGNNRKSF NLDEGHALR**I PSGFISYILN R**HDNQNLRVA K**ISMPVNTPG QFEDFFPASS RDQSSYLQGF SRNTLEAAFN**

321 **AEFNEIRR**VL LEENAGGEQE ERGQRRRSTR SSDNEGVIVK VSKEHVQELT KHAKSVSKKG SEEEDITNPI NLRDGEPDLS

401 NNFGRLFEVK PDKKNPQLQD LDMMLTCVEI KEGALMLPHF NSK**AMVIVVV NKGTGNLELV AVR**KEQQQRG RREQEWEEEE

481 EDEEEEGSNR EVRRYTARLK EGDVFIMPAA HPVAINASSE LHLLGFGINA ENNHRIFLAG DKDNVIDQIE KQAKDLAFPG

561 SGEQVEKLIK NQRESHFVSA RPQSQSPSSP EKEDQEEENQ GGKGPLLSIL KAFN

a Amidination of lysines or N-terminal amines with methyl acetimidate (+41.03)
d Deamidation (NQ) (+0.98)
h Hydroxylation Pro (+15.99)
o Oxidation (M) (+15.99)

Supporting Peptides:

Peptide	Uniq	-10lgP	Mass	Length	ppm	m/z	z	RT	Fraction	Scan	Source File	Area Kontrola 15. traka 67-100 kDa	#Feature	#Feature Kontrola 15. traka 67-100 kDa	Start	End	PTM
K.AM(+15.99)VIVVVN(+.98)KGTGNLELVAVR.K	N	72.00	2098.1819	20	-3.6	700.4025	3	32.23	16	1743	OB4044.raw	1.16E4	1	1	444	463	Oxidation (M)
K.ISM(+15.99)PVNTPGQ(+.98)FEDFFPASSR.D	N	69.11	2243.0205	20	-4.1	1122.5189	2	33.58	16	1822	OB4044.raw	0	0	0	282	301	Oxidation (M); Deamidation (NQ)
K.ISM(+15.99)PVN(+.98)TPGQ(+.98)FEDFFPASSR.D	N	68.85	2244.0044	20	1.8	1123.0175	2	33.95	16	1843	OB4044.raw	1.6E3	1	1	282	301	Oxidation (M); Deamidation (NQ)
K.AM(+15.99)VIVVVNKGTGNLELVAVR.K	N	66.99	2097.1980	20	-6.3	700.0726	3	31.77	16	1708	OB4044.raw	2.4E4	2	2	444	463	Oxidation (M)
K.AM(+15.99)VIVVVNKGTGN(+.98)LELVAVR.K	N	66.89	2098.1819	20	-3.6	700.4025	3	32.23	16	1732	OB4044.raw	1.16E4	1	1	444	463	Oxidation (M)
R.IPSGFISYILNR.H	N	61.22	1378.7609	12	-5.3	690.3877	2	35.47	16	1968	OB4044.raw	2.55E4	1	1	260	271	
R.IPSGFISYILN(+.98)R.H	N	60.08	1379.7449	12	-4.8	690.8801	2	35.84	16	1968	OB4044.raw	4.21E3	1	1	260	271	Deamidation (NQ)
R.I(+41.03)P(+15.99)SGFISYILNR.H	N	52.86	1435.7823	12	-6.2	718.8978	2	35.52	16	1946	OB4044.raw	5.17E3	1	1	260	271	Amidination of lysines or N-terminal amines with methyl acetimidate; Hydroxylation Pro
K.ISM(+15.99)PVNTPGQ(+.98)FEDFFPA.S	N	52.23	1912.8552	17	-4.2	957.4360	2	36.27	16	1987	OB4044.raw	3.37E3	1	1	282	298	Oxidation (M); Deamidation (NQ)
K.ISM(+15.99)PVN(+.98)TPGQFEDFFPA.S	N	49.70	1912.8552	17	-3.4	957.4368	2	36.34	16	1993	OB4044.raw	0	0	0	282	298	Oxidation (M)
K.ISM(+15.99)PVNTPGQ(+.98)FEDFFPASSRDQ(+.98)SSYLQGFSR.N	N	49.07	3512.5830	31	-5.8	879.1526	4	34.26	16	1862	OB4044.raw	5.39E4	2	2	282	312	Oxidation (M)
K.AM(+15.99)VIVVVNK.G	N	48.39	987.5787	9	-5.2	494.7967	2	26.12	16	1394	OB4044.raw	2.62E3	1	1	444	452	Oxidation (M)
R.IVQ(+.98)IEARPNTLVLPK.H	Y	46.53	1690.9980	15	-5.7	564.6731	3	29.74	16	1588	OB4044.raw	3.81E4	1	1	208	222	Deamidation (NQ)
R.NTLEAAFNAEFNEIRR.V	N	45.50	1893.9332	16	-5.7	632.3181	3	33.12	16	1796	OB4044.raw	7.55E4	2	2	313	328	

Peptide	Uniq	-10lgP	Mass	Length	ppm	m/z	z	RT	Fraction	Scan	Source File	Area Kontrola 15. traka 67-100 kDa	#Feature	#Feature Kontrola 15. traka 67-100 kDa	Start	End	PTM
K.ISM(+15.99)PVN(+.98)TPGQFEDFFPASSR.D	N	43.39	2243.0205	20	-5.0	748.6810	3	33.61	16	1824	OB4044.raw	0	0	0	282	301	Oxidation (M)
K.ISM(+15.99)PVN(+.98)TPGQ(+.98)FEDFFPASSRDQSSYLQ(+.98)GFSR.N	N	38.31	3513.5669	31	1.1	879.4047	4	34.42	16	1874	OB4044.raw	1.16E5	1	1	282	312	Oxidation (M)
K.ISMP(+15.99)VNTPGQ(+.98)FEDFFPASSRDQSSYLQGFSR.N	N	35.09	3511.5989	31	-2.8	878.9092	4	34.36	16	1870	OB4044.raw	0	0	0	282	312	
R.IVQIEARPN(+.98)TLVLPK.H	Y	32.96	1690.9980	15	-5.7	564.6731	3	29.74	16	1611	OB4044.raw	3.81E4	1	1	208	222	Deamidation (NQ)
R.NNPFYFPSR.R	N	32.57	1140.5352	9	-7.0	571.2739	2	30.63	16	1640	OB4044.raw	3.93E3	1	1	166	174	
K.ISM(+15.99)PVNTPGQ(+.98)FEDFFPASSRDQSSYLQGFSR.N	N	32.17	3511.5989	31	-4.6	878.9076	4	34.00	16	1848	OB4044.raw	0	0	0	282	312	Oxidation (M)
K.ISM(+15.99)PVNTPGQ(+.98)FEDFFPASSRDQ(+.98)SSYLQ(+.98)GFSR.N	N	32.07	3513.5669	31	0.8	1172.2034	3	34.39	16	1881	OB4044.raw	1.16E5	1	1	282	312	Oxidation (M)
R.NTLEAAFN(+.98)AEFNEIRR.V	N	31.66	1894.9172	16	-4.6	948.4666	2	33.79	16	1828	OB4044.raw	1.24E4	2	2	313	328	
K.ISM(+15.99)PVN(+.98)TPGQ(+.98)FEDFFPA.S	N	31.57	1913.8392	17	-8.1	957.9242	2	36.64	16	2011	OB4044.raw	8.44E2	1	1	282	298	Oxidation (M); Deamidation (NQ)
K.ISMP(+15.99)VN(+.98)TPGQ(+.98)FEDFFPASSR.D	N	31.32	2244.0044	20	0.0	1123.0155	2	34.05	16	1851	OB4044.raw	0	0	0	282	301	Hydroxylation Pro; Deamidation (NQ)
K.ISM(+15.99)PVN(+.98)TPGQ(+.98)FEDFFPASSRDQ(+.98)SSYLQ(+.98)GFSR.N	N	30.89	3514.5510	31	2.4	1172.5333	3	34.78	16	1897	OB4044.raw	0	0	0	282	312	Oxidation (M); Deamidation (NQ)
R.IVQIEARPNTLVLPK.H	Y	27.91	1690.0140	15	-5.5	564.3452	3	29.41	16	1577	OB4044.raw	2.16E4	1	1	208	222	
K.ISM(+15.99)PVNTPGQ(+.98)FEDFFPASSRDQSSYLQ(+.98)GFSR.N	N	26.94	3512.5830	31	-3.8	1171.8701	3	34.22	16	1857	OB4044.raw	3.9E4	1	1	282	312	Oxidation (M)
**total 27 peptides**																	

tr|B3IXL2|B3IXL2_ARAHY

back to list

| Protein Coverage | Supporting Peptides |

Protein Coverage:

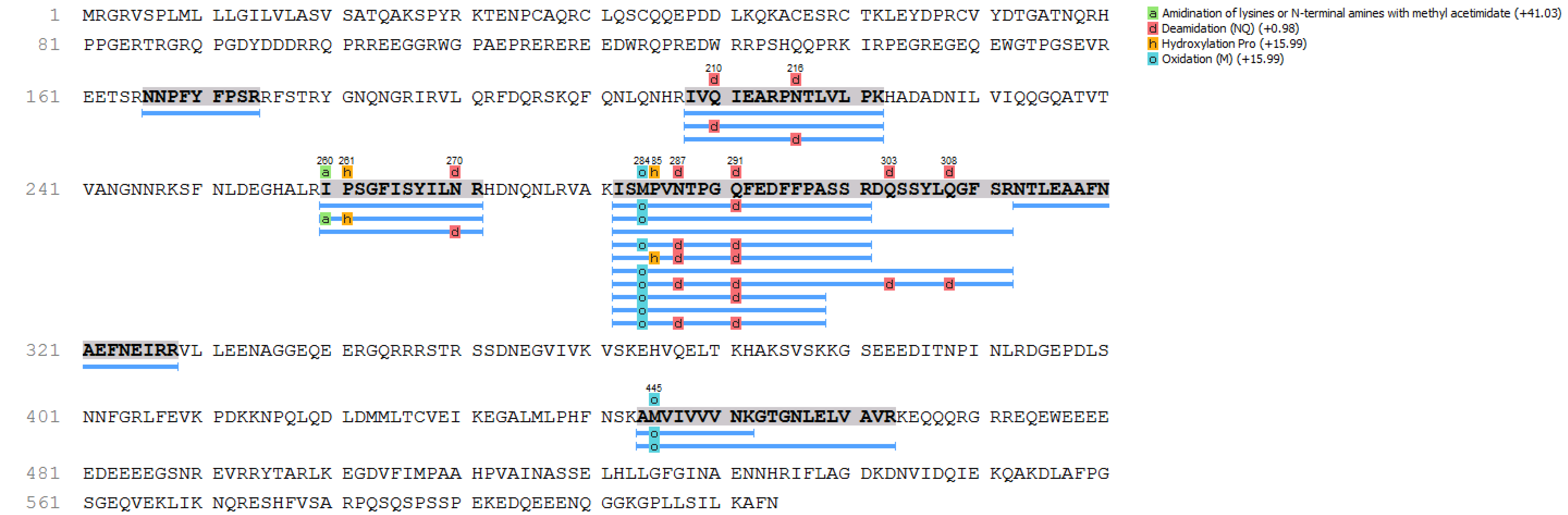

Supporting Peptides:

Peptide	Uniq	-10lgP	Mass	Length	ppm	m/z	z	RT	Fraction	Scan	Source File	Area Kontrola 15. traka 67-100 kDa	#Feature	#Feature Kontrola 15. traka 67-100 kDa	Start	End	PTM
K.AM(+15.99)VIVVVN(+.98)KGTGNLELVAVR.K	N	72.00	2098.1819	20	-3.6	700.4025	3	32.23	16	1743	OB4044.raw	1.16E4	1	1	444	463	Oxidation (M)
K.ISM(+15.99)PVNTPGQ(+.98)FEDFFPASSR.D	N	69.11	2243.0205	20	-4.1	1122.5189	2	33.58	16	1822	OB4044.raw	0	0	0	282	301	Oxidation (M); Deamidation (NQ)
K.ISM(+15.99)PVN(+.98)TPGQ(+.98)FEDFFPASSR.D	N	68.85	2244.0044	20	1.8	1123.0175	2	33.95	16	1843	OB4044.raw	1.6E3	1	1	282	301	Oxidation (M); Deamidation (NQ)
K.AM(+15.99)VIVVVNKGTGNLELVAVR.K	N	66.99	2097.1980	20	-6.3	700.0726	3	31.77	16	1708	OB4044.raw	2.4E4	2	2	444	463	Oxidation (M)
K.AM(+15.99)VIVVVNKGTGN(+.98)LELVAVR.K	N	66.89	2098.1819	20	-3.6	700.4025	3	32.23	16	1732	OB4044.raw	1.16E4	1	1	444	463	Oxidation (M)
R.IPSGFISYILNR.H	N	61.22	1378.7609	12	-5.3	690.3877	2	35.47	16	1968	OB4044.raw	2.55E4	1	1	260	271	
R.IPSGFISYILN(+.98)R.H	N	60.08	1379.7449	12	-4.8	690.8801	2	35.84	16	1968	OB4044.raw	4.21E3	1	1	260	271	Deamidation (NQ)
R.I(+41.03)P(+15.99)SGFISYILNR.H	N	52.86	1435.7823	12	-6.2	718.8978	2	35.52	16	1946	OB4044.raw	5.17E3	1	1	260	271	Amidination of lysines or N-terminal amines with methyl acetimidate; Hydroxylation Pro
K.ISM(+15.99)PVNTPGQ(+.98)FEDFFPA.S	N	52.23	1912.8552	17	-4.2	957.4360	2	36.27	16	1987	OB4044.raw	3.37E3	1	1	282	298	Oxidation (M); Deamidation (NQ)
K.ISM(+15.99)PVN(+.98)TPGQFEDFFPA.S	N	49.70	1912.8552	17	-3.4	957.4368	2	36.34	16	1993	OB4044.raw	0	0	0	282	298	Oxidation (M)
K.ISM(+15.99)PVNTPGQ(+.98)FEDFFPASSRDQ(+.98)SSYLQGFSR.N	N	49.07	3512.5830	31	-5.8	879.1526	4	34.26	16	1862	OB4044.raw	5.39E4	2	2	282	312	Oxidation (M)
K.AM(+15.99)VIVVVNK.G	N	48.39	987.5787	9	-5.2	494.7967	2	26.12	16	1394	OB4044.raw	2.62E3	1	1	444	452	Oxidation (M)
R.IVQ(+.98)IEARPNTLVLPK.H	Y	46.53	1690.9980	15	-5.7	564.6731	3	29.74	16	1588	OB4044.raw	3.81E4	1	1	208	222	Deamidation (NQ)
R.NTLEAAFNAEFNEIRR.V	N	45.50	1893.9332	16	-5.7	632.3181	3	33.12	16	1796	OB4044.raw	7.55E4	2	2	313	328	

Peptide	Uniq	-10lgP	Mass	Length	ppm	m/z	z	RT	Fraction	Scan	Source File	Area Kontrola 15. traka 67-100 kDa	#Feature	#Feature Kontrola 15. traka 67-100 kDa	Start	End	PTM
K.ISM(+15.99)PVN(+.98)TPGQFEDFFPASSR.D	N	43.39	2243.0205	20	-5.0	748.6810	3	33.61	16	1824	OB4044.raw	0	0	0	282	301	Oxidation (M)
K.ISM(+15.99)PVN(+.98)TPGQ(+.98)FEDFFPASSRDQSSYLQ(+.98)GFSR.N	N	38.31	3513.5669	31	1.1	879.4047	4	34.42	16	1874	OB4044.raw	1.16E5	1	1	282	312	Oxidation (M)
K.ISMP(+15.99)VNTPGQ(+.98)FEDFFPASSRDQSSYLQGFSR.N	N	35.09	3511.5989	31	-2.8	878.9092	4	34.36	16	1870	OB4044.raw	0	0	0	282	312	
R.IVQIEARPN(+.98)TLVLPK.H	Y	32.96	1690.9980	15	-5.7	564.6731	3	29.74	16	1611	OB4044.raw	3.81E4	1	1	208	222	Deamidation (NQ)
R.NNPFYFPSR.R	N	32.57	1140.5352	9	-7.0	571.2739	2	30.63	16	1640	OB4044.raw	3.93E3	1	1	166	174	
K.ISM(+15.99)PVNTPGQ(+.98)FEDFFPASSRDQSSYLQGFSR.N	N	32.17	3511.5989	31	-4.6	878.9076	4	34.00	16	1848	OB4044.raw	0	0	0	282	312	Oxidation (M)
K.ISM(+15.99)PVNTPGQ(+.98)FEDFFPASSRDQ(+.98)SSYLQ(+.98)GFSR.N	N	32.07	3513.5669	31	0.8	1172.2034	3	34.39	16	1881	OB4044.raw	1.16E5	1	1	282	312	Oxidation (M)
R.NTLEAAFN(+.98)AEFNEIRR.V	N	31.66	1894.9172	16	-4.6	948.4666	2	33.79	16	1828	OB4044.raw	1.24E4	2	2	313	328	
K.ISM(+15.99)PVN(+.98)TPGQ(+.98)FEDFFPA.S	N	31.57	1913.8392	17	-8.1	957.9242	2	36.64	16	2011	OB4044.raw	8.44E2	1	1	282	298	Oxidation (M); Deamidation (NQ)
K.ISMP(+15.99)VN(+.98)TPGQ(+.98)FEDFFPASSR.D	N	31.32	2244.0044	20	0.0	1123.0155	2	34.05	16	1851	OB4044.raw	0	0	0	282	301	Hydroxylation Pro; Deamidation (NQ)
K.ISM(+15.99)PVN(+.98)TPGQ(+.98)FEDFFPASSRDQ(+.98)SSYLQ(+.98)GFSR.N	N	30.89	3514.5510	31	2.4	1172.5333	3	34.78	16	1897	OB4044.raw	0	0	0	282	312	Oxidation (M); Deamidation (NQ)
R.IVQIEARPNTLVLPK.H	Y	27.91	1690.0140	15	-5.5	564.3452	3	29.41	16	1577	OB4044.raw	2.16E4	1	1	208	222	
K.ISM(+15.99)PVNTPGQ(+.98)FEDFFPASSRDQSSYLQ(+.98)GFSR.N	N	26.94	3512.5830	31	-3.8	1171.8701	3	34.22	16	1857	OB4044.raw	3.9E4	1	1	282	312	Oxidation (M)
**total 27 peptides**																	

tr|Q6PSU3|Q6PSU3_ARAHY

back to list

| Protein Coverage | Supporting Peptides |

Protein Coverage:

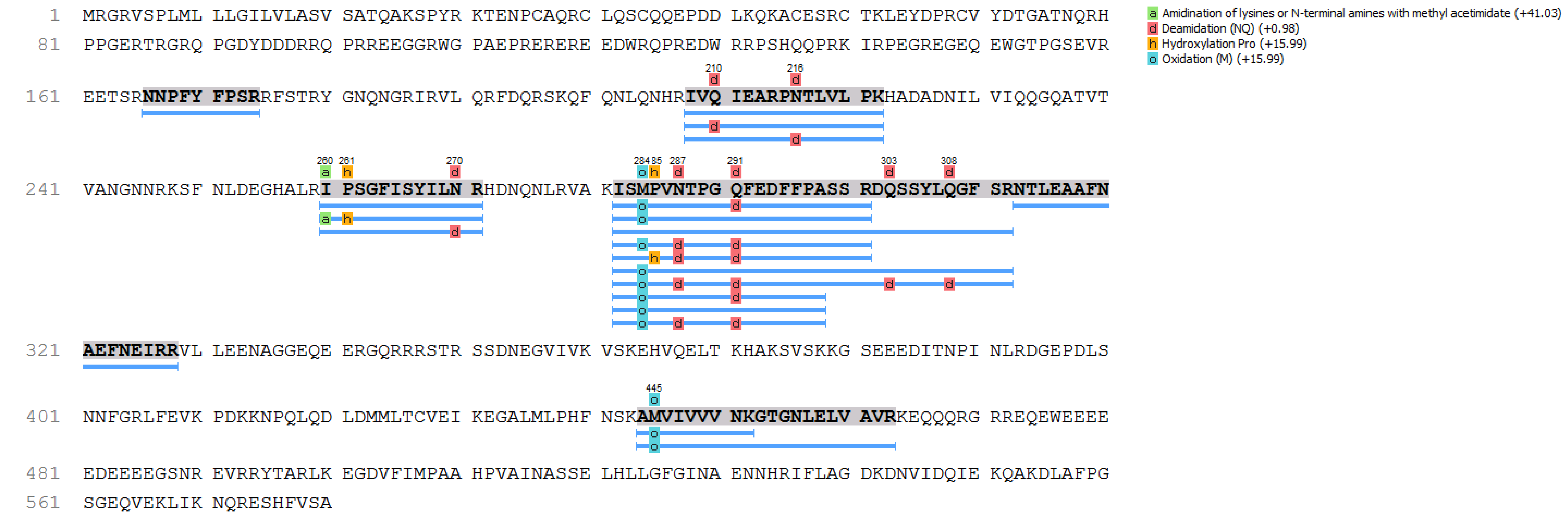

Supporting Peptides:

Peptide	Uniq	-10lgP	Mass	Length	ppm	m/z	z	RT	Fraction	Scan	Source File	Area Kontrola 15. traka 67-100 kDa	#Feature	#Feature Kontrola 15. traka 67-100 kDa	Start	End	PTM
K.AM(+15.99)VIVVVN(+.98)KGTGNLELVAVR.K	N	72.00	2098.1819	20	-3.6	700.4025	3	32.23	16	1743	OB4044.raw	1.16E4	1	1	444	463	Oxidation (M)
K.ISM(+15.99)PVNTPGQ(+.98)FEDFFPASSR.D	N	69.11	2243.0205	20	-4.1	1122.5189	2	33.58	16	1822	OB4044.raw	0	0	0	282	301	Oxidation (M); Deamidation (NQ)
K.ISM(+15.99)PVN(+.98)TPGQ(+.98)FEDFFPASSR.D	N	68.85	2244.0044	20	1.8	1123.0175	2	33.95	16	1843	OB4044.raw	1.6E3	1	1	282	301	Oxidation (M); Deamidation (NQ)
K.AM(+15.99)VIVVVNKGTGNLELVAVR.K	N	66.99	2097.1980	20	-6.3	700.0726	3	31.77	16	1708	OB4044.raw	2.4E4	2	2	444	463	Oxidation (M)
K.AM(+15.99)VIVVVNKGTGN(+.98)LELVAVR.K	N	66.89	2098.1819	20	-3.6	700.4025	3	32.23	16	1732	OB4044.raw	1.16E4	1	1	444	463	Oxidation (M)
R.IPSGFISYILNR.H	N	61.22	1378.7609	12	-5.3	690.3877	2	35.47	16	1968	OB4044.raw	2.55E4	1	1	260	271	
R.IPSGFISYILN(+.98)R.H	N	60.08	1379.7449	12	-4.8	690.8801	2	35.84	16	1968	OB4044.raw	4.21E3	1	1	260	271	Deamidation (NQ)
R.I(+41.03)P(+15.99)SGFISYILNR.H	N	52.86	1435.7823	12	-6.2	718.8978	2	35.52	16	1946	OB4044.raw	5.17E3	1	1	260	271	Amidination of lysines or N-terminal amines with methyl acetimidate; Hydroxylation Pro
K.ISM(+15.99)PVNTPGQ(+.98)FEDFFPA.S	N	52.23	1912.8552	17	-4.2	957.4360	2	36.27	16	1987	OB4044.raw	3.37E3	1	1	282	298	Oxidation (M); Deamidation (NQ)
K.ISM(+15.99)PVN(+.98)TPGQFEDFFPA.S	N	49.70	1912.8552	17	-3.4	957.4368	2	36.34	16	1993	OB4044.raw	0	0	0	282	298	Oxidation (M)
K.ISM(+15.99)PVNTPGQ(+.98)FEDFFPASSRDQ(+.98)SSYLQGFSR.N	N	49.07	3512.5830	31	-5.8	879.1526	4	34.26	16	1862	OB4044.raw	5.39E4	2	2	282	312	Oxidation (M)
K.AM(+15.99)VIVVVNK.G	N	48.39	987.5787	9	-5.2	494.7967	2	26.12	16	1394	OB4044.raw	2.62E3	1	1	444	452	Oxidation (M)
R.IVQ(+.98)IEARPNTLVLPK.H	Y	46.53	1690.9980	15	-5.7	564.6731	3	29.74	16	1588	OB4044.raw	3.81E4	1	1	208	222	Deamidation (NQ)
R.NTLEAAFNAEFNEIRR.V	N	45.50	1893.9332	16	-5.7	632.3181	3	33.12	16	1796	OB4044.raw	7.55E4	2	2	313	328	

Peptide	Uniq	-10lgP	Mass	Length	ppm	m/z	z	RT	Fraction	Scan	Source File	Area Kontrola 15. traka 67-100 kDa	#Feature	#Feature Kontrola 15. traka 67-100 kDa	Start	End	PTM
K.ISM(+15.99)PVN(+.98)TPGQFEDFFPASSR.D	N	43.39	2243.0205	20	-5.0	748.6810	3	33.61	16	1824	OB4044.raw	0	0	0	282	301	Oxidation (M)
K.ISM(+15.99)PVN(+.98)TPGQ(+.98)FEDFFPASSRDQSSYLQ(+.98)GFSR.N	N	38.31	3513.5669	31	1.1	879.4047	4	34.42	16	1874	OB4044.raw	1.16E5	1	1	282	312	Oxidation (M)
K.ISMP(+15.99)VNTPGQ(+.98)FEDFFPASSRDQSSYLQGFSR.N	N	35.09	3511.5989	31	-2.8	878.9092	4	34.36	16	1870	OB4044.raw	0	0	0	282	312	
R.IVQIEARPN(+.98)TLVLPK.H	Y	32.96	1690.9980	15	-5.7	564.6731	3	29.74	16	1611	OB4044.raw	3.81E4	1	1	208	222	Deamidation (NQ)
R.NNPFYFPSR.R	N	32.57	1140.5352	9	-7.0	571.2739	2	30.63	16	1640	OB4044.raw	3.93E3	1	1	166	174	
K.ISM(+15.99)PVNTPGQ(+.98)FEDFFPASSRDQSSYLQGFSR.N	N	32.17	3511.5989	31	-4.6	878.9076	4	34.00	16	1848	OB4044.raw	0	0	0	282	312	Oxidation (M)
K.ISM(+15.99)PVNTPGQ(+.98)FEDFFPASSRDQ(+.98)SSYLQ(+.98)GFSR.N	N	32.07	3513.5669	31	0.8	1172.2034	3	34.39	16	1881	OB4044.raw	1.16E5	1	1	282	312	Oxidation (M)
R.NTLEAAFN(+.98)AEFNEIRR.V	N	31.66	1894.9172	16	-4.6	948.4666	2	33.79	16	1828	OB4044.raw	1.24E4	2	2	313	328	
K.ISM(+15.99)PVN(+.98)TPGQ(+.98)FEDFFPA.S	N	31.57	1913.8392	17	-8.1	957.9242	2	36.64	16	2011	OB4044.raw	8.44E2	1	1	282	298	Oxidation (M); Deamidation (NQ)
K.ISMP(+15.99)VN(+.98)TPGQ(+.98)FEDFFPASSR.D	N	31.32	2244.0044	20	0.0	1123.0155	2	34.05	16	1851	OB4044.raw	0	0	0	282	301	Hydroxylation Pro; Deamidation (NQ)
K.ISM(+15.99)PVN(+.98)TPGQ(+.98)FEDFFPASSRDQ(+.98)SSYLQ(+.98)GFSR.N	N	30.89	3514.5510	31	2.4	1172.5333	3	34.78	16	1897	OB4044.raw	0	0	0	282	312	Oxidation (M); Deamidation (NQ)
R.IVQIEARPNTLVLPK.H	Y	27.91	1690.0140	15	-5.5	564.3452	3	29.41	16	1577	OB4044.raw	2.16E4	1	1	208	222	
K.ISM(+15.99)PVNTPGQ(+.98)FEDFFPASSRDQSSYLQ(+.98)GFSR.N	N	26.94	3512.5830	31	-3.8	1171.8701	3	34.22	16	1857	OB4044.raw	3.9E4	1	1	282	312	Oxidation (M)
**total 27 peptides**																	

tr|N1NG13|N1NG13_ARAHY
back to list

| Protein Coverage | Supporting Peptides |

Protein Coverage:

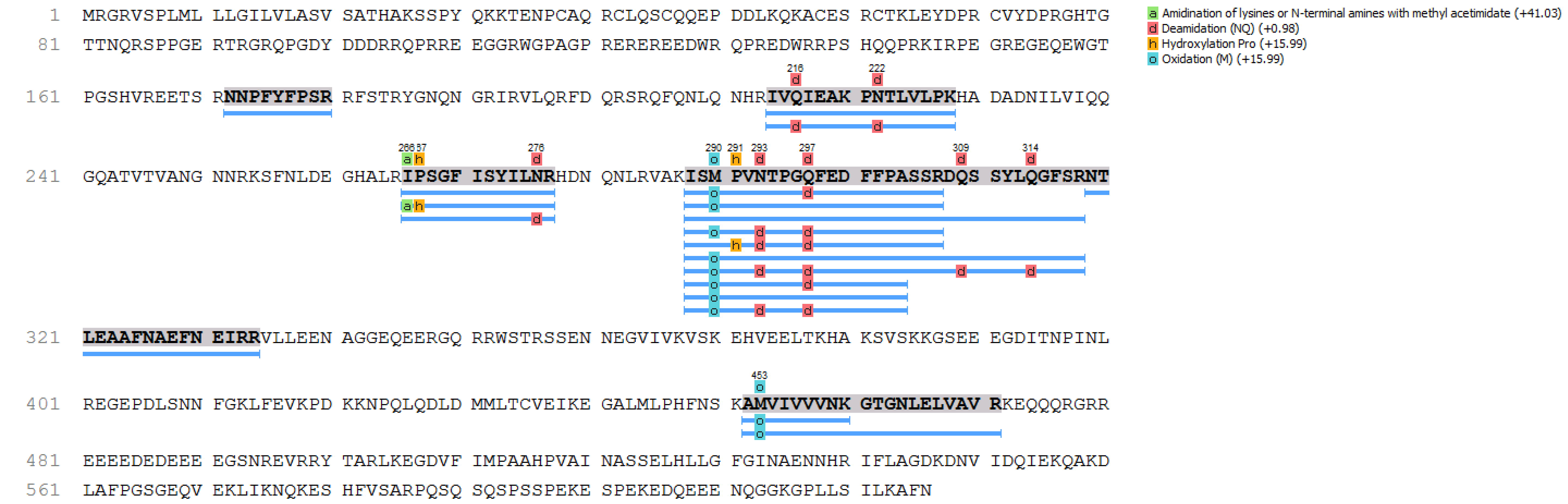

Supporting Peptides:

Peptide	Uniq	-10lgP	Mass	Length	ppm	m/z	z	RT	Fraction	Scan	Source File	Area Kontrola 15. traka 67-100 kDa	#Feature	#Feature Kontrola 15. traka 67-100 kDa	Start	End	PTM
K.AM(+15.99)VIVVVN(+.98)KGTGNLELVAVR.K	N	72.00	2098.1819	20	-3.6	700.4025	3	32.23	16	1743	OB4044.raw	1.16E4	1	1	452	471	Oxidation (M)
K.ISM(+15.99)PVNTPGQ(+.98)FEDFFPASSR.D	N	69.11	2243.0205	20	-4.1	1122.5189	2	33.58	16	1822	OB4044.raw	0	0	0	288	307	Oxidation (M); Deamidation (NQ)
K.ISM(+15.99)PVN(+.98)TPGQ(+.98)FEDFFPASSR.D	N	68.85	2244.0044	20	1.8	1123.0175	2	33.95	16	1843	OB4044.raw	1.6E3	1	1	288	307	Oxidation (M); Deamidation (NQ)
K.AM(+15.99)VIVVVNKGTGNLELVAVR.K	N	66.99	2097.1980	20	-6.3	700.0726	3	31.77	16	1708	OB4044.raw	2.4E4	2	2	452	471	Oxidation (M)
K.AM(+15.99)VIVVVNKGTGN(+.98)LELVAVR.K	N	66.89	2098.1819	20	-3.6	700.4025	3	32.23	16	1732	OB4044.raw	1.16E4	1	1	452	471	Oxidation (M)
R.IPSGFISYILNR.H	N	61.22	1378.7609	12	-5.3	690.3877	2	35.47	16	1968	OB4044.raw	2.55E4	1	1	266	277	
R.IPSGFISYILN(+.98)R.H	N	60.08	1379.7449	12	-4.8	690.8801	2	35.84	16	1968	OB4044.raw	4.21E3	1	1	266	277	Deamidation (NQ)
R.I(+41.03)P(+15.99)SGFISYILNR.H	N	52.86	1435.7823	12	-6.2	718.8978	2	35.52	16	1946	OB4044.raw	5.17E3	1	1	266	277	Amidination of lysines or N-terminal amines with methyl acetimidate; Hydroxylation Pro
K.ISM(+15.99)PVNTPGQ(+.98)FEDFFPA.S	N	52.23	1912.8552	17	-4.2	957.4360	2	36.27	16	1987	OB4044.raw	3.37E3	1	1	288	304	Oxidation (M); Deamidation (NQ)
K.ISM(+15.99)PVN(+.98)TPGQFEDFFPA.S	N	49.70	1912.8552	17	-3.4	957.4368	2	36.34	16	1993	OB4044.raw	0	0	0	288	304	Oxidation (M)
K.ISM(+15.99)PVNTPGQ(+.98)FEDFFPASSRDQ(+.98)SSYLQGFSR.N	N	49.07	3512.5830	31	-5.8	879.1526	4	34.26	16	1862	OB4044.raw	5.39E4	2	2	288	318	Oxidation (M)
K.AM(+15.99)VIVVVNK.G	N	48.39	987.5787	9	-5.2	494.7967	2	26.12	16	1394	OB4044.raw	2.62E3	1	1	452	460	Oxidation (M)
R.NTLEAAFNAEFNEIRR.V	N	45.50	1893.9332	16	-5.7	632.3181	3	33.12	16	1796	OB4044.raw	7.55E4	2	2	319	334	
K.ISM(+15.99)PVN(+.98)TPGQFEDFFPASSR.D	N	43.39	2243.0205	20	-5.0	748.6810	3	33.61	16	1824	OB4044.raw	0	0	0	288	307	Oxidation (M)
K.ISM(+15.99)PVN(+.98)TPGQ(+.98)FEDFFPASSRDQSSYLQ(+.98)GFSR.N	N	38.31	3513.5669	31	1.1	879.4047	4	34.42	16	1874	OB4044.raw	1.16E5	1	1	288	318	Oxidation (M)

Peptide	Uniq	-10lgP	Mass	Length	ppm	m/z	z	RT	Fraction	Scan	Source File	Area Kontrola 15. traka 67-100 kDa	#Feature	#Feature Kontrola 15. traka 67-100 kDa	Start	End	PTM
K.ISMP(+15.99)VNTPGQ(+.98)FEDFFPASSRDQSSYLQGFSR.N	N	35.09	3511.5989	31	-2.8	878.9092	4	34.36	16	1870	OB4044.raw	0	0	0	288	318	
R.IVQIEAKPNTLVLPK.H	Y	32.75	1662.0079	15	-5.4	555.0099	3	29.19	16	1557	OB4044.raw	1.44E4	1	1	214	228	
R.NNPFYFPSR.R	N	32.57	1140.5352	9	-7.0	571.2739	2	30.63	16	1640	OB4044.raw	3.93E3	1	1	172	180	
K.ISM(+15.99)PVNTPGQ(+.98)FEDFFPASSRDQSSYLQGFSR.N	N	32.17	3511.5989	31	-4.6	878.9076	4	34.00	16	1848	OB4044.raw	0	0	0	288	318	Oxidation (M)
K.ISM(+15.99)PVNTPGQ(+.98)FEDFFPASSRDQ(+.98)SSYLQ(+.98)GFSR.N	N	32.07	3513.5669	31	0.8	1172.2034	3	34.39	16	1881	OB4044.raw	1.16E5	1	1	288	318	Oxidation (M)
R.NTLEAAFN(+.98)AEFNEIRR.V	N	31.66	1894.9172	16	-4.6	948.4666	2	33.79	16	1828	OB4044.raw	1.24E4	2	2	319	334	
K.ISM(+15.99)PVN(+.98)TPGQ(+.98)FEDFFPA.S	N	31.57	1913.8392	17	-8.1	957.9242	2	36.64	16	2011	OB4044.raw	8.44E2	1	1	288	304	Oxidation (M); Deamidation (NQ)
K.ISMP(+15.99)VN(+.98)TPGQ(+.98)FEDFFPASSR.D	N	31.32	2244.0044	20	0.0	1123.0155	2	34.05	16	1851	OB4044.raw	0	0	0	288	307	Hydroxylation Pro; Deamidation (NQ)
K.ISM(+15.99)PVN(+.98)TPGQ(+.98)FEDFFPASSRDQ(+.98)SSYLQ(+.98)GFSR.N	N	30.89	3514.5510	31	2.4	1172.5333	3	34.78	16	1897	OB4044.raw	0	0	0	288	318	Oxidation (M); Deamidation (NQ)
R.IVQIEAKPN(+.98)TLVLPK.H	Y	30.55	1662.9919	15	-6.4	555.3373	3	29.46	16	1592	OB4044.raw	2.92E4	1	1	214	228	
R.IVQ(+.98)IEAKPN(+.98)TLVLPK.H	Y	30.04	1663.9760	15	-6.1	555.6655	3	30.00	16	1616	OB4044.raw	7.43E3	1	1	214	228	Deamidation (NQ)
K.ISM(+15.99)PVNTPGQ(+.98)FEDFFPASSRDQSSYLQ(+.98)GFSR.N	N	26.94	3512.5830	31	-3.8	1171.8701	3	34.22	16	1857	OB4044.raw	3.9E4	1	1	288	318	Oxidation (M)
**total 27 peptides**																	

P43238|ALL12_ARAHY

back to list

| Protein Coverage | Supporting Peptides |

Protein Coverage:

1 MRGRVSPLML LLGILVLASV SATHAKSSPY QKKTENPCAQ RCLQSCQQEP DDLKQKACES RCTKLEYDPR CVYDPRGHTG

81 TTNQRSPPGE RTRGRQPGDY DDDRRQPRRE EGGRWGPAGP REREREEDWR QPREDWRRPS HQQPRKIRPE GREGEQEWGT

161 PGSHVREETS RNNPFYFPSR RFSTRYGNQN GRIRVLQRFD QRSRQFQNLQ NHRIVQIEAK PNTLVLPKHA DADNILVIQQ

241 GQATVTVANG NNRKSFNLDE GHALRIPSGF ISYILNRHDN QNLRVAKISM PVNTPGQFED FFPASSRDQS SYLQGFSRNT

321 LEAAFNAEFN EIRRVLLEEN AGGEQEERGQ RRWSTRSSEN NEGVIVKVSK EHVEELTKHA KSVSKKGSEE EGDITNPINL

401 REGEPDLSNN FGKLFEVKPD KKNPQLQDLD MMLTCVEIKE GALMLPHFNS KAMVIVVVNK GTGNLELVAV RKEQQQRGRR

481 EEEEDEDEEE EGSNREVRRY TARLKEGDVF IMPAAHPVAI NASSELHLLG FGINAENNHR IFLAGDKDNV IDQIEKQAKD

561 LAFPGSGEQV EKLIKNQKES HFVSARPQSQ SQSPSSPEKE SPEKEDQEEE NQGGKGPLLS ILKAFN

a Amidination of lysines or N-terminal amines with methyl acetimidate (+41.03)
d Deamidation (NQ) (+0.98)
h Hydroxylation Pro (+15.99)
o Oxidation (M) (+15.99)

Modification sites: 216 d, 222 d, 266 a, 267 h, 276 d, 290 o, 291 h, 293 d, 297 d, 309 d, 314 d, 453 o

Supporting Peptides:

Peptide	Uniq	-10lgP	Mass	Length	ppm	m/z	z	RT	Fraction	Scan	Source File	Area Kontrola 15. traka 67-100 kDa	#Feature	#Feature Kontrola 15. traka 67-100 kDa	Start	End	PTM
K.AM(+15.99)VIVVVN(+.98)KGTGNLELVAVR.K	N	72.00	2098.1819	20	-3.6	700.4025	3	32.23	16	1743	OB4044.raw	1.16E4	1	1	452	471	Oxidation (M)
K.ISM(+15.99)PVNTPGQ(+.98)FEDFFPASSR.D	N	69.11	2243.0205	20	-4.1	1122.5189	2	33.58	16	1822	OB4044.raw	0	0	0	288	307	Oxidation (M); Deamidation (NQ)
K.ISM(+15.99)PVN(+.98)TPGQ(+.98)FEDFFPASSR.D	N	68.85	2244.0044	20	1.8	1123.0175	2	33.95	16	1843	OB4044.raw	1.6E3	1	1	288	307	Oxidation (M); Deamidation (NQ)
K.AM(+15.99)VIVVVNKGTGNLELVAVR.K	N	66.99	2097.1980	20	-6.3	700.0726	3	31.77	16	1708	OB4044.raw	2.4E4	2	2	452	471	Oxidation (M)
K.AM(+15.99)VIVVVNKGTGN(+.98)LELVAVR.K	N	66.89	2098.1819	20	-3.6	700.4025	3	32.23	16	1732	OB4044.raw	1.16E4	1	1	452	471	Oxidation (M)
R.IPSGFISYILNR.H	N	61.22	1378.7609	12	-5.3	690.3877	2	35.47	16	1968	OB4044.raw	2.55E4	1	1	266	277	
R.IPSGFISYILN(+.98)R.H	N	60.08	1379.7449	12	-4.8	690.8801	2	35.84	16	1968	OB4044.raw	4.21E3	1	1	266	277	Deamidation (NQ)
R.I(+41.03)P(+15.99)SGFISYILNR.H	N	52.86	1435.7823	12	-6.2	718.8978	2	35.52	16	1946	OB4044.raw	5.17E3	1	1	266	277	Amidination of lysines or N-terminal amines with methyl acetimidate; Hydroxylation Pro
K.ISM(+15.99)PVNTPGQ(+.98)FEDFFPA.S	N	52.23	1912.8552	17	-4.2	957.4360	2	36.27	16	1987	OB4044.raw	3.37E3	1	1	288	304	Oxidation (M); Deamidation (NQ)
K.ISM(+15.99)PVN(+.98)TPGQFEDFFPA.S	N	49.70	1912.8552	17	-3.4	957.4368	2	36.34	16	1993	OB4044.raw	0	0	0	288	304	Oxidation (M)
K.ISM(+15.99)PVNTPGQ(+.98)FEDFFPASSRDQ(+.98)SSYLQGFSR.N	N	49.07	3512.5830	31	-5.8	879.1526	4	34.26	16	1862	OB4044.raw	5.39E4	2	2	288	318	Oxidation (M)
K.AM(+15.99)VIVVVNK.G	N	48.39	987.5787	9	-5.2	494.7967	2	26.12	16	1394	OB4044.raw	2.62E3	1	1	452	460	Oxidation (M)
R.NTLEAAFNAEFNEIRR.V	N	45.50	1893.9332	16	-5.7	632.3181	3	33.12	16	1796	OB4044.raw	7.55E4	2	2	319	334	
K.ISM(+15.99)PVN(+.98)TPGQFEDFFPASSR.D	N	43.39	2243.0205	20	-5.0	748.6810	3	33.61	16	1824	OB4044.raw	0	0	0	288	307	Oxidation (M)
K.ISM(+15.99)PVN(+.98)TPGQ(+.98)FEDFFPASSRDQSSYLQ(+.98)GFSR.N	N	38.31	3513.5669	31	1.1	879.4047	4	34.42	16	1874	OB4044.raw	1.16E5	1	1	288	318	Oxidation (M)

Peptide	Uniq	-10lgP	Mass	Length	ppm	m/z	z	RT	Fraction	Scan	Source File	Area Kontrola 15. traka 67-100 kDa	#Feature	#Feature Kontrola 15. traka 67-100 kDa	Start	End	PTM
K.ISMP(+15.99)VNTPGQ(+.98)FEDFFPASSRDQSSYLQGFSR.N	N	35.09	3511.5989	31	-2.8	878.9092	4	34.36	16	1870	OB4044.raw	0	0	0	288	318	
R.IVQIEAKPNTLVLPK.H	Y	32.75	1662.0079	15	-5.4	555.0099	3	29.19	16	1557	OB4044.raw	1.44E4	1	1	214	228	
R.NNPFYFPSR.R	N	32.57	1140.5352	9	-7.0	571.2739	2	30.63	16	1640	OB4044.raw	3.93E3	1	1	172	180	
K.ISM(+15.99)PVNTPGQ(+.98)FEDFFPASSRDQSSYLQGFSR.N	N	32.17	3511.5989	31	-4.6	878.9076	4	34.00	16	1848	OB4044.raw	0	0	0	288	318	Oxidation (M)
K.ISM(+15.99)PVNTPGQ(+.98)FEDFFPASSRDQ(+.98)SSYLQ(+.98)GFSR.N	N	32.07	3513.5669	31	0.8	1172.2034	3	34.39	16	1881	OB4044.raw	1.16E5	1	1	288	318	Oxidation (M)
R.NTLEAAFN(+.98)AEFNEIRR.V	N	31.66	1894.9172	16	-4.6	948.4666	2	33.79	16	1828	OB4044.raw	1.24E4	2	2	319	334	
K.ISM(+15.99)PVN(+.98)TPGQ(+.98)FEDFFPA.S	N	31.57	1913.8392	17	-8.1	957.9242	2	36.64	16	2011	OB4044.raw	8.44E2	1	1	288	304	Oxidation (M); Deamidation (NQ)
K.ISMP(+15.99)VN(+.98)TPGQ(+.98)FEDFFPASSR.D	N	31.32	2244.0044	20	0.0	1123.0155	2	34.05	16	1851	OB4044.raw	0	0	0	288	307	Hydroxylation Pro; Deamidation (NQ)
K.ISM(+15.99)PVN(+.98)TPGQ(+.98)FEDFFPASSRDQ(+.98)SSYLQ(+.98)GFSR.N	N	30.89	3514.5510	31	2.4	1172.5333	3	34.78	16	1897	OB4044.raw	0	0	0	288	318	Oxidation (M); Deamidation (NQ)
R.IVQIEAKPN(+.98)TLVLPK.H	Y	30.55	1662.9919	15	-6.4	555.3373	3	29.46	16	1592	OB4044.raw	2.92E4	1	1	214	228	
R.IVQ(+.98)IEAKPN(+.98)TLVLPK.H	Y	30.04	1663.9760	15	-6.1	555.6655	3	30.00	16	1616	OB4044.raw	7.43E3	1	1	214	228	Deamidation (NQ)
K.ISM(+15.99)PVNTPGQ(+.98)FEDFFPASSRDQSSYLQ(+.98)GFSR.N	N	26.94	3512.5830	31	-3.8	1171.8701	3	34.22	16	1857	OB4044.raw	3.9E4	1	1	288	318	Oxidation (M)
**total 27 peptides**																	

tr|Q9M5D3|Q9M5D3_ARAHY

back to list

| Protein Coverage | Supporting Peptides |

Protein Coverage:

Oxidation (M) (+15.99)

1 MVSVRRSAGI RGRAGILNRG HKIKWTVVLM RKNVLDVDTF TDVAATANIG GLIGTGIN**VI GSTVDALTAF LGR**SVSLQLI

81 SSTQSDVNGN GKVVKDTFLE GIIASLPTLE LENLAFSIHF EWDDSMGIPG AFYIKNYMQV EFFLKTLTLE DVPNQGTIHF

161 VCNSWVYNSK LYKSPR**IFFS NKPYLPSETP APLVK**YREED LKILRGDGKG ERQEHERIYD YDVYNDLGNP DRNENHARPI

241 LGGSTTFLTS QGRTGRYPAR NDPNSEKPGD VYVPRDENFG HLKSSDFLAN SIKFLTRYVL PAFESVFDLN LTPNEFDSFQ

321 DVRDLYEGGI RLPTEVISTK RRLLVIKELF RTDGEQVLKF PPPRFLQVNE ENSAWMTDEE FVIINELLVS HPCMDPTIYG

437

401 DQNSKIPAEV LDLEGCSLEE AINGRR**LFIL DYHDVFMPYV R**RINETHAKA YATRTILFLK EDGTLNPVAI ELSLPHPDGD

481 KSGAISDVIL PAKGRCRKHN LATSQSLCHI NDSCYHHLMS HWLNTHAVIE PFVIATNRQL SVIHPIYKLL SPHYRDTMNI

561 NALARQNLIN SDGIIERTFL PSKFSVEMSS AVYKNWVFTD QALPADLIKR GMAVEDSSSP YGIRLVIEDY PYAVDGLEIW

641 FAIKEWVQDY VSLYYPTDND LKKDPELQNW WKEAVEVGHG DLKDKPWWPK MQTVEELVES CTTIIWTASA LHAAVNFGQY

721 PYGGLILNRP TLSRRLLPEQ GTAEYEEMVK SHQKAYLR**TI TPKLETLIDL TTIEILSK**HA SDEVYLGERD NPHWTFDSRA

801 LEAFQRFGNK LSEIEEKLTE KNKDGRLSNR IGPVELPYTL LHPTSNEGLT FRGVPNSISI

Supporting Peptides:

Peptide	Uniq	-10lgP	Mass	Length	ppm	m/z	z	RT	Fraction	Scan	Source File	Area Kontrola 15. traka 67-100 kDa	#Feature	#Feature Kontrola 15. traka 67-100 kDa	Start	End	PTM
K.LETLIDLTTIEILSK.H	Y	87.78	1700.9811	15	-5.6	851.4976	2	37.65	16	2082	OB4044.raw	9.15E3	1	1	764	778	

Peptide	Uniq	-10lgP	Mass	Length	ppm	m/z	z	RT	Fraction	Scan	Source File	Area Kontrola 15. traka 67-100 kDa	#Feature	#Feature Kontrola 15. traka 67-100 kDa	Start	End	PTM
N.VIGSTVDALTAFLGR.S	Y	84.90	1518.8406	15	-5.2	760.4277	2	36.00	16	1964	OB4044.raw	2.21E4	3	3	59	73	
R.LFILDYHDVFM(+15.99)PYVR.R	Y	59.84	1942.9651	15	-4.5	648.6628	3	35.12	16	1913	OB4044.raw	1.32E4	1	1	427	441	Oxidation (M)
R.IFFSNKPYLPSETPAPLVK.Y	Y	46.74	2147.1665	19	-4.3	716.7302	3	31.65	16	1704	OB4044.raw	4.55E3	1	1	177	195	
R.TITPKLETLIDLTTIEILSK.H	Y	41.98	2241.3083	20	-5.3	748.1101	3	38.12	16	2128	OB4044.raw	1.02E4	1	1	759	778	
R.LFILDYHDVFMPYVR.R	Y	36.81	1926.9702	15	-5.0	643.3309	3	36.27	16	1983	OB4044.raw	7.58E3	1	1	427	441	
**total 6 peptides**																	

tr|Q4JME6|Q4JME6_ARAHY

back to list

| Protein Coverage | Supporting Peptides |

Protein Coverage:

Oxidation (M) (+15.99)

1 MFSGVAGILN RGHKIKGTVV LMRKNVLDVD TFTDVAATAN IGGLIGTGIN **VIGSTVDALT AFLGR**SVSLQ LISSTQSDEN
81 GNGKVVKDTF LEGIIASLPT LGAGESAFSI HFEWDDSMGI PGAFYIKNYM QVEFFLKTLT LEDVPNQGTI HFVCNSWVYN
161 SKLYKSPR**IF FSNKPYLPSE TPAPLVK**YRE EDLKILRGDG KGERQEHERI YDYDVYNDLG NPDRNENHAR PILGGSTTFP
241 YPRRGRTGRY PARNDPNSEK PGDVYVPRDE NFGHLKSSDF LANSIKFLTR YVLPAFESVF DLNLTPNEFD SFQDVRDLYE
321 GGIRLPTEVI STISPLPVIK ELFRTDGEQV LKFPPPHIIQ VNKSAWMTDE EFAREMIAGV NPCMIRSLQE FPPKSNLDPT
440
401 IYGDQNSKIP AEVLDLEGCS LEEAINGRR**L FILDYHDVFM PYVR**RINETH AKAYATRTIL FLKEDGTLKP VAIELSLPHP
481 DGDKSGAISE VILPAKEGVE STIWLLAKAY VIVNDSCYHQ LMSHWLNTHA VIEPFVIATN RQLSVIHPIY KLLSPHYLDT
561 MNINALARQN LINSDGIIER TFLPSKFSVE MSSAVYKNWV FTDQALPADL IKRGMAVEDS SSPYGIRLVI EDYPYAVDGL
641 EIWFAIKEWV QDYVSLYYPT DNDLKKDPEL QNWWKEAVEV GHGDLKDKPW WPKMQTVEEL VESCTTIIWT ASALHAAVNF
721 GQYPYGGLIL NRPTLSRRLL PEQGTAEYEE MVKSHQKAYL R**TITPKLETL IDLTTIEILS K**HASDEVYLG ERDNPHWTFD
801 SRALEAFQRF GNKLSEIEEK LTEKNKDGRL SNRIGPVELP YTLLHPTSNE GLTFRGVPNS ISI

Supporting Peptides:

Peptide	Uniq	-10lgP	Mass	Length	ppm	m/z	z	RT	Fraction	Scan	Source File	Area Kontrola 15. traka 67-100 kDa	#Feature	#Feature Kontrola 15. traka 67-100 kDa	Start	End	PTM
K.LETLIDLTTIEILSK.H	Y	87.78	1700.9811	15	-5.6	851.4976	2	37.65	16	2082	OB4044.raw	9.15E3	1	1	767	781	
N.VIGSTVDALTAFLGR.S	Y	84.90	1518.8406	15	-5.2	760.4277	2	36.00	16	1964	OB4044.raw	2.21E4	3	3	51	65	
R.LFILDYHDVFM(+15.99)PYVR.R	Y	59.84	1942.9651	15	-4.5	648.6628	3	35.12	16	1913	OB4044.raw	1.32E4	1	1	430	444	Oxidation (M)
R.IFFSNKPYLPSETPAPLVK.Y	Y	46.74	2147.1665	19	-4.3	716.7302	3	31.65	16	1704	OB4044.raw	4.55E3	1	1	169	187	
R.TITPKLETLIDLTTIEILSK.H	Y	41.98	2241.3083	20	-5.3	748.1101	3	38.12	16	2128	OB4044.raw	1.02E4	1	1	762	781	
R.LFILDYHDVFMPYVR.R	Y	36.81	1926.9702	15	-5.0	643.3309	3	36.27	16	1983	OB4044.raw	7.58E3	1	1	430	444	
**total 6 peptides**																	

tr|Q4JME7|Q4JME7_ARAHY

back to list

| Protein Coverage | Supporting Peptides |

Protein Coverage:

1 MFSGVTGMLN RGHKIKGTVV LMRKNVLDVD TFTDVVATAN IGGLIGTGIN **VIGSTVDALT AFLGR**SVSLQ LISSTQSDEN

Oxidation (M) (+15.99)

81 GNGKVVKDTF LEGIIASLPT LGAGESAFSI HFEWDDSMGI PGAFYIKNYM QVEFFLKTLT LEDVPNQGTI HFVCNSWVYN

161 SKLYKSPR**IF FSNKPYLPSE TPAPLVK**YRE EDLKNLRGDG KGERQEHERI YDYDVYNDLG NPDRNENHAR PILGGSTTFP

241 YPRRGRTGRY PARNDPNSEK PGDVYVPRDE NFGHLKSSDF LANSIKFLTR YVLPAFESVF DLNLTPNEFD SFQDVRDLYE

321 GGIRLPTEVI STISPLPVIK ELFRTDGEQV LKFPPPHIIQ VNKSAWMTDE EFAREMIAGV NPCMIRSLQE FPPKSNLDPT

440

401 IYGDQNSKIP AEVLDLEGCS LEEAINGRR**L FILDYHDVFM PYVR**RINETH AKAYATRTIL FLKEDGTLKP VAIELSLPHP

481 DGDKSGAISE VILPAKEGVE STIWLLAKAY VIVNDSCYHQ LMSHWLNTHA VIEPFVIATN RQLSVIHPIY KLLSPHYRDT

561 MNINALARQN LINSDGIIER TFLPSKFSVE MSSAVYKNWV FTDQALPADL IKRGMAVEDS SSPYGIRLVI EDYPYAVDGL

641 EIWFAIKEWV QDYVSLYYPT DNDLKKDPEL QNWWKEAVEV GHGDLKDKPW WPKMQTVEEL VESCTTIIWT ASALHAAVNF

721 GQYPYGGLIL NRPTLSRRLL PEQGTAEYEE MVKSHQKAYL R**TITPKLETL IDLTTIEILS K**HASDEVYLG ERDNPHWTFD

801 SRALEAFQRF GNKLSEIEEK LTEKNKDGRL SNRIGPVELP YTLLHPTSNE GLTFRGVPNS ISI

Supporting Peptides:

Peptide	Uniq	-10lgP	Mass	Length	ppm	m/z	z	RT	Fraction	Scan	Source File	Area Kontrola 15. traka 67-100 kDa	#Feature	#Feature Kontrola 15. traka 67-100 kDa	Start	End	PTM
K.LETLIDLTTIEILSK.H	Y	87.78	1700.9811	15	-5.6	851.4976	2	37.65	16	2082	OB4044.raw	9.15E3	1	1	767	781	
N.VIGSTVDALTAFLGR.S	Y	84.90	1518.8406	15	-5.2	760.4277	2	36.00	16	1964	OB4044.raw	2.21E4	3	3	51	65	
R.LFILDYHDVFM(+15.99)PYVR.R	Y	59.84	1942.9651	15	-4.5	648.6628	3	35.12	16	1913	OB4044.raw	1.32E4	1	1	430	444	Oxidation (M)
R.IFFSNKPYLPSETPAPLVK.Y	Y	46.74	2147.1665	19	-4.3	716.7302	3	31.65	16	1704	OB4044.raw	4.55E3	1	1	169	187	
R.TITPKLETLIDLTTIEILSK.H	Y	41.98	2241.3083	20	-5.3	748.1101	3	38.12	16	2128	OB4044.raw	1.02E4	1	1	762	781	
R.LFILDYHDVFMPYVR.R	Y	36.81	1926.9702	15	-5.0	643.3309	3	36.27	16	1983	OB4044.raw	7.58E3	1	1	430	444	
**total 6 peptides**																	

tr|Q5I6T2|Q5I6T2_ARAHY

back to list

| Protein Coverage | Supporting Peptides |

Protein Coverage:

1 MAKLLELSFC FCFLVLGASS ISFRQQPEEN ACQFQRLNAQ RPDNRIESEG GYIETWNPNN QEFECAGVAL SRLVLRRNAL

d Deamidation (NQ) (+0.98)

81 RRPFYSNAPQ EIFIQQGRGY FGLIFPGCPS TYEEPAQQGR RSQSQRPPRR LQGEDQSQQQ QDSHQKVHRF DEGDLIAVPT

161 GVAFWLYNDH DTDVVAVSLT DTNNNDNQLD QFPRRFNLAG NHEQEFLRYQ QQSRQSRRRS LPYSPYSPQS QPRQEEREFS

241 PRGQHSRRER AGQEEENEGG NIFSGFTPEF LEQAFQVDDR QIVQNLRGEN ESEEEGAIVT VRGGLRILSP DRKRGADEEE

398 d

321 EYDEDEYEYD EEDRRRGRGS RGRGNGIEET ICTASVKKNI GRNRSPDIYN PQAGSLK**TAN DLNLLILRWL GLSAEYGNLY**

d

401 **RNALFVPHYN TNAHSIIYAL R**GRAHVQVVD SNGNRVYDEE LQEGHVLVVP QNFAVAGKSQ SDNFEYVAFK TDSRPSIANL

481 AGENSVIDNL PEEVVANSYG LQREQARQQL KNNNPFKFFV PPSQQSPRAV A

Supporting Peptides:

Peptide	Uniq	-10lgP	Mass	Length	ppm	m/z	z	RT	Fraction	Scan	Source File	Area Kontrola 15. traka 67-100 kDa	#Feature	#Feature Kontrola 15. traka 67-100 kDa	Start	End	PTM
R.WLGLSAEYGNLYR.N	N	97.15	1540.7673	13	-5.2	771.3911	2	33.39	16	1811	OB4044.raw	3.81E4	1	1	389	401	
K.TANDLN(+.98)LLILRWLGLSAEYGNLYR.N	Y	64.94	2778.4705	24	0.6	927.1696	3	39.99	16	2224	OB4044.raw	7.99E2	1	1	378	401	
K.TANDLNLLILRWLGLSAEYGNLYR.N	Y	53.61	2777.4863	24	0.6	926.8416	3	39.97	16	2224	OB4044.raw	0	0	0	378	401	
R.WLGLSAEYGN(+.98)LYR.N	N	52.28	1541.7513	13	-7.2	771.8815	2	33.73	16	1831	OB4044.raw	0	0	0	389	401	Deamidation (NQ)
R.NALFVPHYN(+.98)TNAHSIIYALR.G	N	30.39	2314.1858	20	3.9	579.5591	4	31.52	16	1697	OB4044.raw	0	0	0	402	421	
**total 5 peptides**																	

tr|Q9FZ11|Q9FZ11_ARAHY

back to list

| Protein Coverage | Supporting Peptides |

Protein Coverage:

1 MIRGRLALSV CFCFLVLGAS SISFRQQPEE NACQFQRLNA QRPDNRLESE GGYIETWNPN NQEFECAGVA LSRLVLRRNA

d Deamidation (NQ) (+0.98)

81 LRRPFYSNAP QEIFIQQGRG YFGLIFPGCP STYEEPAQQG RRHQSQRAPR RFEGEDQSQQ QQQDSHQKVR RFDEGDLIAV

161 PTGVALWMFN DHDTDVVAVS LTDTNNNDNQ LDQFPRRFNL AGNHEQEFLR YQQQSRRRSL PYSPYSPQSQ PRQEEREFSP

241 RGQHSRRERA GQEEENEGGN IFSGFTPEFL AQAFQVDDRQ IVQNLRGENE SEEEGAIVTV KGGLRILSPD RKRGADEEEE

397 d

321 YDEDEYEYDE EDRRRGRGSR GRGNGIEETI CTASVKKNIG RNRSPDIYNP QAGSLK**TAND LNLLILRWLG LSAEYGNLYR**

d

401 **NALFVPHYNT NAHSIIYALR** GRAHVQVVDS NGNRVYDEEL QEGHVLVVPQ NFAVAGKSQS DNFEYVAFKT DSRPNIANFA

481 GENSIIDNLP EEVVANSYGL PREQARQLKN NNPFKFFVPP SQQSLRAVA

Supporting Peptides:

Peptide	Uniq	-10lgP	Mass	Length	ppm	m/z	z	RT	Fraction	Scan	Source File	Area Kontrola 15. traka 67-100 kDa	#Feature	#Feature Kontrola 15. traka 67-100 kDa	Start	End	PTM
R.WLGLSAEYGNLYR.N	N	97.15	1540.7673	13	-5.2	771.3911	2	33.39	16	1811	OB4044.raw	3.81E4	1	1	388	400	
K.TANDLN(+.98)LLILRWLGLSAEYGNLYR.N	Y	64.94	2778.4705	24	0.6	927.1696	3	39.99	16	2224	OB4044.raw	7.99E2	1	1	377	400	
K.TANDLNLLILRWLGLSAEYGNLYR.N	Y	53.61	2777.4863	24	0.6	926.8416	3	39.97	16	2224	OB4044.raw	0	0	0	377	400	
R.WLGLSAEYGN(+.98)LYR.N	N	52.28	1541.7513	13	-7.2	771.8815	2	33.73	16	1831	OB4044.raw	0	0	0	388	400	Deamidation (NQ)

Peptide	Uniq	-10lgP	Mass	Length	ppm	m/z	z	RT	Fraction	Scan	Source File	Area Kontrola 15. traka 67-100 kDa	#Feature	#Feature Kontrola 15. traka 67-100 kDa	Start	End	PTM
R.NALFVPHYN(+.98)TNAHSIIYALR.G	N	30.39	2314.1858	20	3.9	579.5591	4	31.52	16	1697	OB4044.raw	0	0	0	401	420	
**total 5 peptides**																	

tr|Q647H3|Q647H3_ARAHY
back to list

| Protein Coverage | Supporting Peptides |

Protein Coverage:

```
  1 MAKLLALSVC FCFLVLGASS ISFRQQPEEN ACQFQRLNAQ RPDNRLESEG GYIETWNPNN QEFECAGVAL SRLVLRRNAL
 81 RRPFYSNAPQ EIFIQQGRGY FGLIFPGCPS TYEEPAQQGR RHQSQRAPRR FEGEDQSQQQ QQDSHQKVRR FDEGDLIAVP
161 TGVALWMYND HDTDVVAVSL TDTNNNDNQL DQFPRRFNLA GNHEQEFLRY QQQSRRRSLP YSPYSPQSQP RQEEREFSPR
241 GQHSRRERAG QEQENEGGNI FSGFTPEFLA QAFQVDDRQI LQNLRGENES DEQGAIVTVR GGLRILSPDR KRRQQYERPD
321 EEEEYDEDEY EYDEEERQQD RRRGRGSRGR GNGIEETICT ASVKKNIGRN RSPDIYNPQA GSLKTANDLN LLILRWLGLS
401 AEYGNLYRNA LFVPHYNTNA HSIIYALRGR AHVQVVDSNG NRVYDEELQE GHVLVVPQNF AVAGKSQSDN FEYVAFKTDS
481 RPSIANLAGE NSIIDNLPEE VVANSYGLPR EQARQLKNNN PFKFFVPPSQ QSLGAVA
```

d Deamidation (NQ) (+0.98)

405 d

Supporting Peptides:

Peptide	Uniq	-10lgP	Mass	Length	ppm	m/z	z	RT	Fraction	Scan	Source File	Area Kontrola 15. traka 67-100 kDa	#Feature	#Feature Kontrola 15. traka 67-100 kDa	Start	End	PTM
R.WLGLSAEYGNLYR.N	N	97.15	1540.7673	13	-5.2	771.3911	2	33.39	16	1811	OB4044.raw	3.81E4	1	1	396	408	
K.TANDLN(+.98)LLILRWLGLSAEYGNLYR.N	Y	64.94	2778.4705	24	0.6	927.1696	3	39.99	16	2224	OB4044.raw	7.99E2	1	1	385	408	
K.TANDLNLLILRWLGLSAEYGNLYR.N	Y	53.61	2777.4863	24	0.6	926.8416	3	39.97	16	2224	OB4044.raw	0	0	0	385	408	
R.WLGLSAEYGN(+.98)LYR.N	N	52.28	1541.7513	13	-7.2	771.8815	2	33.73	16	1831	OB4044.raw	0	0	0	396	408	Deamidation (NQ)
R.NALFVPHYN(+.98)TNAHSIIYALR.G	N	30.39	2314.1858	20	3.9	579.5591	4	31.52	16	1697	OB4044.raw	0	0	0	409	428	
**total 5 peptides**																	

tr|A1DZF0|A1DZF0_ARAHY
back to list

| Protein Coverage | Supporting Peptides |

Protein Coverage:

1 MAKLLELSFC FCFLVLGASS ISFRQQPEEN ACQFQRLNAQ RPDNRIESEG GYIETWNPNN QEFECAGVAL SRLVLRRNAL

d Deamidation (NQ) (+0.98)

81 RRPFYSNAPQ EIFIQQGRGY FGLIFPGCPS TYEEPAQQGR RYQSQRPPRR LQEEDQSQQQ QDSHQKVHRF NEGDLIAVPT

161 GVAFWLYNDH DTDVVAVSLT DTNNNDNQLD QFPRRFNLAG NHEQEFLRYQ QQSRQSRRRS LPLSPYSPQP GQEDREFSPQ

241 GQHGRRERAG QEQENEGGNI FSGFTSEFLA QAFQVDDRQI VQNLRGENES EEQGAIVTVK GGLRILSPDR KSPDEEEEYD

397 d

321 EDEYAEEERQ QDRRRGRGSR GSGNGIEETI CTATVKKNIG RNRSPDIYNP QAGSLK**TANE LNLLILRWLG LSAEYGNLYR**

401 **NALFVPHYNT NAHSIIYALR** GRAHVQVVDS NGNRVYDEEL QEGHVLVVPQ NFAVAGKSQS ENFEYVAFKT DSRPSIANLA

481 GENSFIDNLP EEVVANSYGL PREQARQLKN NNPFKFFVPP FQQSPRAVA

Supporting Peptides:

Peptide	Uniq	-10lgP	Mass	Length	ppm	m/z	z	RT	Fraction	Scan	Source File	Area Kontrola 15. traka 67-100 kDa	#Feature	#Feature Kontrola 15. traka 67-100 kDa	Start	End	PTM
R.WLGLSAEYGNLYR.N	N	97.15	1540.7673	13	-5.2	771.3911	2	33.39	16	1811	OB4044.raw	3.81E4	1	1	388	400	
R.WLGLSAEYGN(+.98)LYR.N	N	52.28	1541.7513	13	-7.2	771.8815	2	33.73	16	1831	OB4044.raw	0	0	0	388	400	Deamidation (NQ)
K.TANELNLLILR.W	Y	39.00	1268.7452	11	-5.3	635.3799	2	33.87	16	1837	OB4044.raw	4.28E3	1	1	377	387	
R.NALFVPHYN(+.98)TNAHSIIYALR.G	N	30.39	2314.1858	20	3.9	579.5591	4	31.52	16	1697	OB4044.raw	0	0	0	401	420	
**total 4 peptides**																	

tr|B5TYU1|B5TYU1_ARAHY

back to list

| Protein Coverage | Supporting Peptides |

Protein Coverage:

1 MAKLLELSFC FCFLVLGASS ISFRQQPEEN ACQFQRLNAQ RPDNRIESEG GYIETWNPNN QEFECAGVAL SRLVLRRNAL

d Deamidation (NQ) (+0.98)

81 RRPFYSNAPQ EIFIQQGRGY FGLIFPGCPS TYEEPAQQGR RYQSQRPPRR LQEEDQSQQQ QDSHQKVHRF NEGDLIAVPT

161 GVAFWLYNDH DTDVVAVSLT DTNNNDNQLD QFPRRFNLAG NHEQEFLRYQ QQSRQSRRRS LPYSPYSPQS QPRQEEREFS

241 PRGQHSRRER AGQEEENEGG NIFSGFTPEF LAQAFQVDDR QIVQNLRGEN ESEEQGAIVT VRGGLRILSP DRKRGADEEE

398 d

321 EYDEDEYEYD EEDRRRGRGS RGSGNGIEET ICTATVKKNI GRNRSPDIYN PQAGSLK**TAN ELNLLILRWL GLSAEYGNLY**

401 **RNALFVPHYN TNAHSIIYAL R**GRAHVQVVD SNGNRVYDEE LQEGHVLVVP QNFAVAGKSQ SDNFEYVAFK TDSRPSIANL

481 AGENSVIDNL PEEVVANSYG LPREQARQLK NNNPFKFFVP PSQQSPRAVA

Supporting Peptides:

Peptide	Uniq	-10lgP	Mass	Length	ppm	m/z	z	RT	Fraction	Scan	Source File	Area Kontrola 15. traka 67-100 kDa	#Feature	#Feature Kontrola 15. traka 67-100 kDa	Start	End	PTM
R.WLGLSAEYGNLYR.N	N	97.15	1540.7673	13	-5.2	771.3911	2	33.39	16	1811	OB4044.raw	3.81E4	1	1	389	401	
R.WLGLSAEYGN(+.98)LYR.N	N	52.28	1541.7513	13	-7.2	771.8815	2	33.73	16	1831	OB4044.raw	0	0	0	389	401	Deamidation (NQ)
K.TANELNLLILR.W	Y	39.00	1268.7452	11	-5.3	635.3799	2	33.87	16	1837	OB4044.raw	4.28E3	1	1	378	388	
R.NALFVPHYN(+.98)TNAHSIIYALR.G	N	30.39	2314.1858	20	3.9	579.5591	4	31.52	16	1697	OB4044.raw	0	0	0	402	421	
**total 4 peptides**																	

tr|Q647H4|Q647H4_ARAHY

back to list

| **Protein Coverage** | **Supporting Peptides** |

Protein Coverage:

d Deamidation (NQ) (+0.98)

```
  1  MGKLLALSVC FCFLVLGASS ISFRQQPEEN ACQFQRLNAQ RPDNRIESEG GYIETWNPNN QEFECAGVAL SRLVLRRNAL
 81  RRPFYSNAPQ EIFIQQGRGY FGLIFPGCPS TYEEPAQQGR RHQSQRPPRR FQGQDQSQQQ QDSHQKVHRF DEGDLIAVPT
161  GVAFWMYNDH DTDVVAVSLT DTNNNDNQLD QFPRRFNLAG NHEQEFLRYQ QQSRRRSLPY SPYSPQTQPK QEDREFSPRG
241  QHGRRERAGQ EQENEGGNIF SGFTPEFLAQ AFQVDDRQIL QNLRGENESD EQGAIVTVRG GLRILSPDRK RRQQYERPDE
321  EEEYDEDEYE YDEEERQHDR RRGRGSRGSG NGIEETICTA SFKKNIGRNR SPDIYNPQAG SLKTANELNL LILRWLGLSA
        404
         d
401  EYGNLYRNAL FVPHYNTNAH SIIYALRGRA HVQVVDSNGD RVFDEELQEG HVLVVPQNFA VAGKSQSENF EYVAFKTDSR
481  PSIANLAGEN SFIDNLPEEV VANSYGLPRE QARQLKNNNP FKFFVPPSEQ SLRAVA
```

Supporting Peptides:

Peptide	Uniq	-10lgP	Mass	Length	ppm	m/z	z	RT	Fraction	Scan	Source File	Area Kontrola 15. traka 67-100 kDa	#Feature	#Feature Kontrola 15. traka 67-100 kDa	Start	End	PTM
R.WLGLSAEYGNLYR.N	N	97.15	1540.7673	13	-5.2	771.3911	2	33.39	16	1811	OB4044.raw	3.81E4	1	1	395	407	
R.WLGLSAEYGN(+.98)LYR.N	N	52.28	1541.7513	13	-7.2	771.8815	2	33.73	16	1831	OB4044.raw	0	0	0	395	407	Deamidation (NQ)
K.TANELNLLILR.W	Y	39.00	1268.7452	11	-5.3	635.3799	2	33.87	16	1837	OB4044.raw	4.28E3	1	1	384	394	
R.NALFVPHYN(+.98)TNAHSIIYALR.G	N	30.39	2314.1858	20	3.9	579.5591	4	31.52	16	1697	OB4044.raw	0	0	0	408	427	
total 4 peptides																	

tr|Q6T2T4|Q6T2T4_ARAHY

back to list

| **Protein Coverage** | **Supporting Peptides** |

Protein Coverage:

d Deamidation (NQ) (+0.98)

```
  1  MGKLLALSVC FCFLVLGASS ISFRQQPEEN ACQFQRLNAQ RPDNRIESEG GYIETWNPNN QEFECAGVAL SRLVLRRNAL
 81  RRPFYSNAPQ EIFIQQGRAY FGLIFLGCPS TYEEPAQQGR RHQSQRPPRR FQGQDQSQQQ QDSHQKVHRF DEGDLIAVPT
161  GVAFWMYNDH DTDVVAVSLT DTNNNDNQLD QFPRRFNLAG NHEQEFLRYQ QQSRRRSLPY SPYSPQTQPK QEDREFSPRG
241  QHGRRERAGQ EQENEGGNIF SGFTPEFLAQ AFQVDDRQIL QNLRGENESD EQGAIVTVRG GLRILSPDRK KRQQYERPDE
321  EEEYDEDEYE YDEEERQQDR RRGRGSRGSG NGIEETICTA SFKKNIGRNR SPDIYNPQAG SLKTANELNL LILRWLGLSA
        404
         d
401  EYGNLYRNAL FVPHYNTNAH SIIYALRGRA HVQVVDSNGD RVFDEELQEG HVLVVPQNFA VAGKSQSENF EYVAFKTDSR
481  PSIANLAGEN SFIDNLPEEV VANSYGLPRE QARQLKNNNP FKFFVPPSEQ SLRAVA
```

Supporting Peptides:

Peptide	Uniq	-10lgP	Mass	Length	ppm	m/z	z	RT	Fraction	Scan	Source File	Area Kontrola 15. traka 67-100 kDa	#Feature	#Feature Kontrola 15. traka 67-100 kDa	Start	End	PTM
R.WLGLSAEYGNLYR.N	N	97.15	1540.7673	13	-5.2	771.3911	2	33.39	16	1811	OB4044.raw	3.81E4	1	1	395	407	
R.WLGLSAEYGN(+.98)LYR.N	N	52.28	1541.7513	13	-7.2	771.8815	2	33.73	16	1831	OB4044.raw	0	0	0	395	407	Deamidation (NQ)
K.TANELNLLILR.W	Y	39.00	1268.7452	11	-5.3	635.3799	2	33.87	16	1837	OB4044.raw	4.28E3	1	1	384	394	
R.NALFVPHYN(+.98)TNAHSIIYALR.G	N	30.39	2314.1858	20	3.9	579.5591	4	31.52	16	1697	OB4044.raw	0	0	0	408	427	

Peptide	Uniq	-10lgP	Mass	Length	ppm	m/z	z	RT	Fraction	Scan	Source File	Area Kontrola 15. traka 67-100 kDa	#Feature	#Feature Kontrola 15. traka 67-100 kDa	Start	End	PTM
total 4 peptides																	

tr|Q6IWG5|Q6IWG5_ARAHY

back to list

| Protein Coverage | Supporting Peptides |

Protein Coverage:

```
  1 KLLALSLCFC VLVLGASSVT FRQGGEENEC QFQRLNAQRP DNRIESEGGY IETWNPNNQE FQCAGVALSR TVLRRNALRR
 81 PFYSNAPLEI YVQQGSGYFG LIFPGCPSTY EEPAQEGRRY QSQKPSRRFQ VGQDDPSQQQ QDSHQKVHRF DEGDLIAVPT
161 GVAFWMYNDE DTDVVTVTLS DTSSIHNQLD QFPRRFYLAG NQEQEFLRYQ QQQGSRPHYR QISPRVRGDE QENEGSNIFS
241 GFAQEFLQHA FQVDRQTVEN LRGENEREEQ GAIVTVKGGL RILSPDEEDE SSRSPPSRRE EFDEDRSRPQ QRGKYDENRR
                                                    359              374
                                                    d                d
321 GYKNGIEETI CSASVKKNLG RSSNPDIYNP QAGSLRSVNE LDLPILGWLG LSAQHGTIYR NAMFVPHYTL NAHTIVVALN
                                                                     d
                                                    d                d
401 GRAHVQVVDS NGNRVYDEEL QEGHVLVVPQ NFAVAAKAQS ENYEYLAFKT DSRPSIANLA GENSIIDNLP EEVVANSYRL
481 PREQARQLKN NNPFKFFVPP FDHQSMREVA
```

d Deamidation (NQ) (+0.98)

Supporting Peptides:

Peptide	Uniq	-10lgP	Mass	Length	ppm	m/z	z	RT	Fraction	Scan	Source File	Area Kontrola 15. traka 67-100 kDa	#Feature	#Feature Kontrola 15. traka 67-100 kDa	Start	End	PTM
R.SVNELDLPILGWLGLSAQHGTIYR.N	Y	57.81	2651.4070	24	-5.0	884.8099	3	38.47	16	2125	OB4044.raw	3.02E4	1	1	357	380	
R.SVNELDLPILGWLGLSAQ(+.98)HGTIYR.N	Y	39.39	2652.3911	24	-4.2	885.1386	3	38.78	16	2157	OB4044.raw	6.72E4	1	1	357	380	Deamidation (NQ)
R.SVN(+.98)ELDLPILGWLGLSAQ(+.98)HGTIYR.N	Y	34.29	2653.3750	24	-5.6	885.4653	3	39.22	16	2177	OB4044.raw	0	0	0	357	380	Deamidation (NQ)
total 3 peptides																	

tr|E5G077|E5G077_ARAHY

back to list

| Protein Coverage | Supporting Peptides |

Protein Coverage:

```
  1 MAKLLALSLC FCVLVLGASS VTFRQGGEEN ECQFQRLNAQ RPDNRIESEG GYIETWNPNN QEFQCAGVAL SRTVLRRNAL
 81 RRPFYSNAPL EIYVQQGSGY FGLIFPGCPS TYEEPAQEGR RYQSQKPSRR FQVGQDDPSQ QQQDSHQKVH RFDEGDLIAV
161 PTGVAFWMYN DEDTDVVTVT LSDTSSIHNQ LDQFPRRFYL AGNQEQEFLR YQQQQGSRPH YRQISPRVRG DEQENEGSNI
241 FSGFAQEFLQ HAFQVDRQTV ENLRGENERE EQGAIVTVKG GLRILSPDEE DESSRSPPNR REEFDEDRSR PQQRGKYDEN
                                                       361              376
                                                       d                d
321 RRGYKNGIEE TICSASVKKN LGRSSNPDIY NPQAGSLRSV NELDLPILGW LGLSAQHGTI YRNAMFVPHY TLNAHTIVVA
                                                                        d
                                                       d                d
401 LNGRAHVQVV DSNGNRVYDE ELQEGHVLVV PQNFAVAAKA QSENYEYLAF KTDSRPSIAN QAGENSIIDN LPEEVVANSY
481 RLPREQARQL KNNNPFKFFV PPFDHQSMRE VA
```

d Deamidation (NQ) (+0.98)

Supporting Peptides:

Peptide	Uniq	-10lgP	Mass	Length	ppm	m/z	z	RT	Fraction	Scan	Source File	Area Kontrola 15. traka 67-100 kDa	#Feature	#Feature Kontrola 15. traka 67-100 kDa	Start	End	PTM
R.SVNELDLPILGWLGLSAQHGTIYR.N	Y	57.81	2651.4070	24	-5.0	884.8099	3	38.47	16	2125	OB4044.raw	3.02E4	1	1	359	382	
R.SVNELDLPILGWLGLSAQ(+.98)HGTIYR.N	Y	39.39	2652.3911	24	-4.2	885.1386	3	38.78	16	2157	OB4044.raw	6.72E4	1	1	359	382	Deamidation (NQ)
R.SVN(+.98)ELDLPILGWLGLSAQ(+.98)HGTIYR.N	Y	34.29	2653.3750	24	-5.6	885.4653	3	39.22	16	2177	OB4044.raw	0	0	0	359	382	Deamidation (NQ)
**total 3 peptides**																	

tr|Q0GM57|Q0GM57_ARAHY

back to list

| Protein Coverage | Supporting Peptides |

Protein Coverage:

d Deamidation (NQ) (+0.98)

```
  1 MAKLLALSLC FCVLVLGASS VTFRQGGEEN ECQFQRLNAQ RPDNRIESEG GYIETWNPNN QEFQCAGVAL SRTVLRRNAL
 81 RRPFYSNAPL EIYVQQGSGY FGLIFPGCPS TYEEPAQEGR RYQSQKPSRR FQVGQDDPSQ QQQDSHQKVH RFDEGDLIAV
161 PTGVAFWMYN DEDTDVVTVT LSDTSSIHNQ LDQFPRRFYL AGNQEQEFLR YQQQQGSRPH YRQISPRVRG DEQENEGSNI
241 FSGFAQEFLQ HAFQVDRQTV ENLRGENERE EQGAIVTVKG GLRILSPDEE DESSRSPPSR REEFDEDRSR PQQRGKYDEN
321 RRGYKNGIEE TICSASVKKN LGRSSNPDIY NPQAGSLRSV NELDLPILGW LGLSAQHGTI YRNAMFVPHY TLNAHTIVVA
401 LNGRAHVQVV DSNGNRVYDE ELQEGHVLVV PQNFAVAAKA QSENYEYLAF KTDSRPSIAN LAGENSIIDN LPEEVVANSY
481 RLPREQARQL KNNNPFKFFV PPFDHQSMRE VA
```

(361 d, 376 d — deamidation sites marked over SV NELDLPILGW LGLSAQHGTI YR)

Supporting Peptides:

Peptide	Uniq	-10lgP	Mass	Length	ppm	m/z	z	RT	Fraction	Scan	Source File	Area Kontrola 15. traka 67-100 kDa	#Feature	#Feature Kontrola 15. traka 67-100 kDa	Start	End	PTM
R.SVNELDLPILGWLGLSAQHGTIYR.N	Y	57.81	2651.4070	24	-5.0	884.8099	3	38.47	16	2125	OB4044.raw	3.02E4	1	1	359	382	
R.SVNELDLPILGWLGLSAQ(+.98)HGTIYR.N	Y	39.39	2652.3911	24	-4.2	885.1386	3	38.78	16	2157	OB4044.raw	6.72E4	1	1	359	382	Deamidation (NQ)
R.SVN(+.98)ELDLPILGWLGLSAQ(+.98)HGTIYR.N	Y	34.29	2653.3750	24	-5.6	885.4653	3	39.22	16	2177	OB4044.raw	0	0	0	359	382	Deamidation (NQ)
**total 3 peptides**																	

Peptide List

Prepared with PEAKS ™ (bioinfor.com)

Summary

1. Notes Gastric Digest Raw peanut Band #C 67-80 kDa (PTM506)

2. Result Statistics

Figure 1. False discovery rate (FDR) curve. X axis is the number of peptide-spectrum matches (PSM) being kept. Y axis is the corresponding FDR.

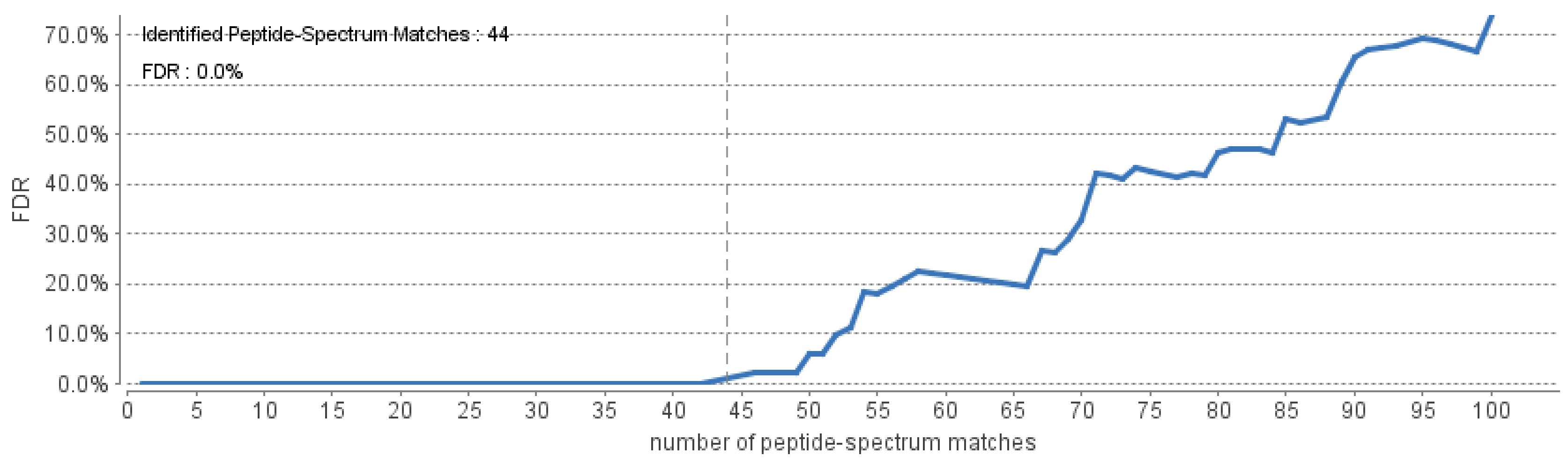

Figure 2. PSM score distribution. **(a)** Distribution of PEAKS peptide score; **(b)** Scatterplot of PEAKS peptide score versus precursor mass error.

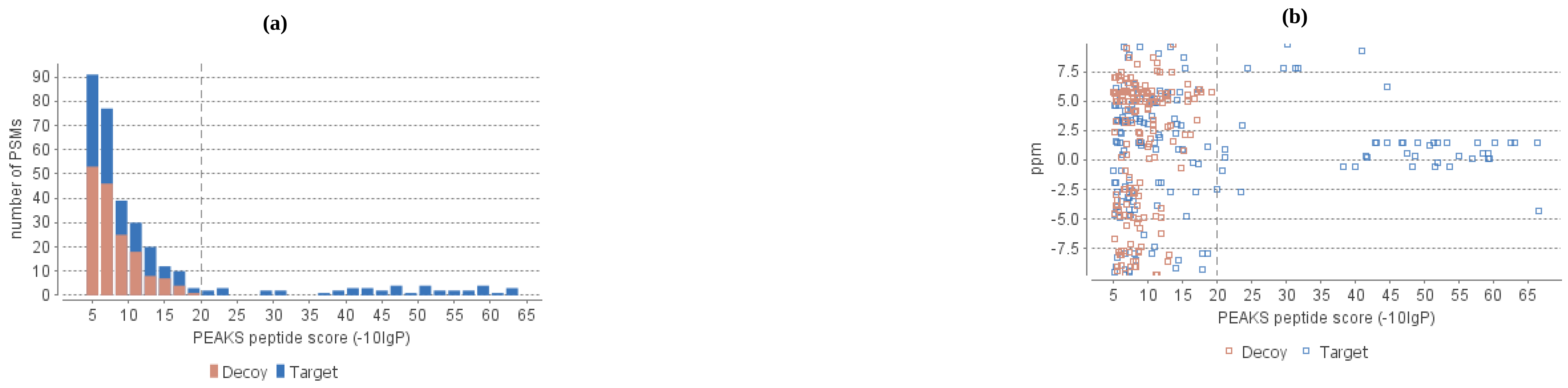

Figure 3. Distribution of peptide feature detection. **(a)** Feature m/z distribution; **(b)** Feature RT distribution

(a) (b)

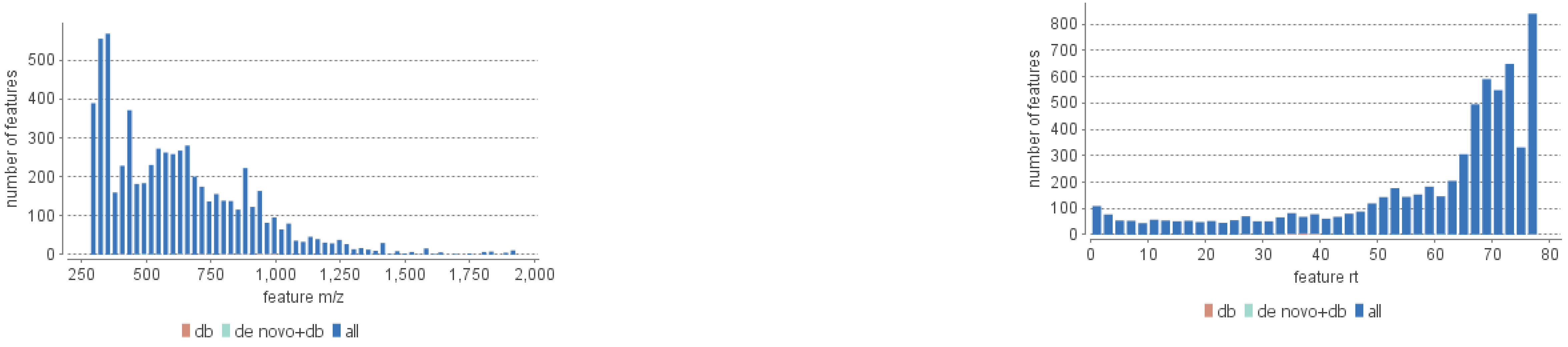

Figure 4. Distribution of identified peptide features. **(a)** Feature abundance distribution **(b)** *De novo* sequencing validation.

(a) (b)

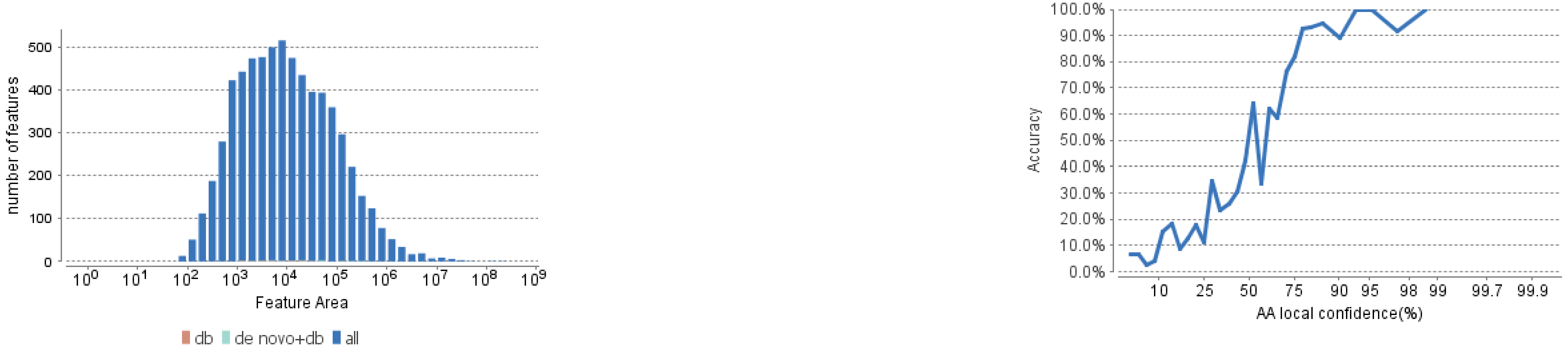

Table 1. Statistics of data.

# of MS scans	3071
# of MS/MS scans	1199
# of Features	6545
# of Chimera scans	126

Table 2. Result filtration parameters.

Peptide -10lgP	≥20

Table 4. PTM profile.

Name	ΔMass	Position	#PSM	-10lgP	Abundance	AScore
Deamidation	.98	NQ	7	44.63		0.00
Carbamidomethyl	57.02	C	2	50.67	2.39E3	1000.00
HydPro	15.99	P	1	20.79	3.67E4	0.00
Phosphorylation	79.97	S	1	23.43		3.04

PTM Ascore	≥20
Protein -10lgP	≥20
Proteins unique peptides	≥1
De novo ALC Score	≥50%

ISD_z+2_ion	-15.01	N-term	1	21.09	1000.00
Methylation(KR)	14.02	K	1	21.09	80.56

Table 3. Statistics of filtered result.

Peptide-Spectrum Matches	44
Peptide sequences	19
Protein groups	4
Proteins	13
Proteins (#Unique Peptides)	0 (>2); 0 (=2); 13 (=1);
FDR (Peptide-Spectrum Matches)	0.0%
FDR (Peptide Sequences)	0.0%
FDR (Protein Group)	0.0%
De Novo Only Spectra	12
# of identified Features	22
# of identified MS/MS scans	44

3. Experiment Control

Figure 5. Precursor mass error of peptide-spectrum matches (PSM) in filtered result. **(a)** Distribution of precursor mass error in ppm; **(b)** Scatterplot of precursor m/z versus precursor mass error in ppm.

(a)

(b)

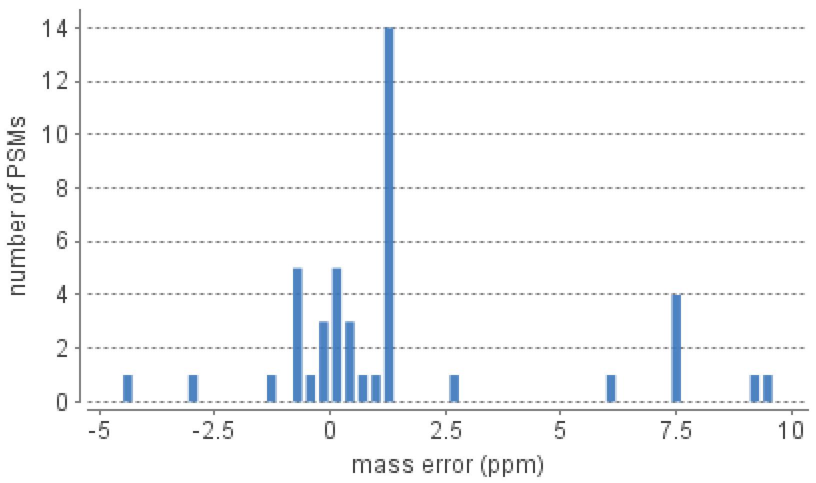

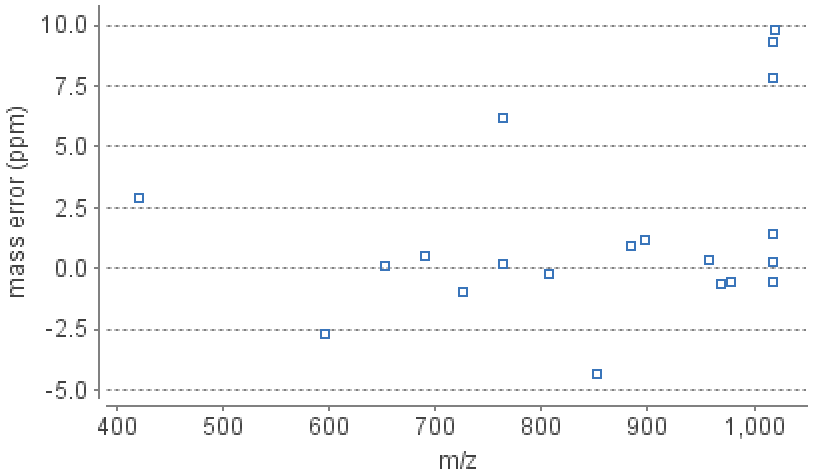

Table 5. Number of identified peptides in each sample by the number of missed cleavages

Missed Cleavages	0	1	2	3	4+
Digest traka C 65-70 kDa	7	11	1	0	0

4. Other Information

Table 6. Search parameters.

Search Engine Name: PEAKS
Parent Mass Error Tolerance: 10.0 ppm
Fragment Mass Error Tolerance: 0.5 Da
Precursor Mass Search Type: monoisotopic
Enzyme: Trypsin
Max Missed Cleavages: 2
Digest Mode: Unspecific
Fixed Modifications:
  Carbamidomethylation: 57.02
Variable Modifications:
  Deamidation (NQ): 0.98
  Oxidation (M): 15.99
  Hydroxylation Pro: 15.99
  Acetylation (K): 42.01
  Acetylation (Protein N-term): 42.01
  Acetylation (N-term): 42.01
  Amidation: -0.98
  Beta-methylthiolation: 45.99
  and 305 more...
Max Variable PTM Per Peptide: 5

Table 7. Instrument parameters.

Fractions: OB4068.raw
Ion Source: ESI(nano-spray)
Fragmentation Mode: CID, CAD(y and b ions)
MS Scan Mode: FT-ICR/Orbitrap
MS/MS Scan Mode: Linear Ion Trap

Database: Uniprot_Peanut-3818_Jul18
Taxon: All
Contaminant Database: contaminantsMQ_mar19
Searched Entry: 1723
FDR Estimation: Enabled
De novo score (ALC%) threshold: 15
Peptide hit threshold (-10logP): 30.0
Peaks run ID: 494
Merge Options: no merge
Precursor Options: corrected
Charge Options: no correction
Filter Charge: 2 - 8
Process: true
Associate chimera: yes

Protein List

Protein Accession Contains:
Protein Description Contains:
Peptide Sample Area >=
Protein Ptm Contains:

Protein Group	Protein ID	Accession	-10lgP	Coverage (%)	Coverage (%) Digest traka C 65-70 kDa	Area Digest traka C 65-70 kDa	#Peptides	#Unique	#Spec Digest traka C 65-70 kDa	PTM	Avg. Mass	Description
**5**	21031	tr|Q9M5D3|Q9M5D3_ARAHY	66.63	2	2	1.35E3	1	1	1	N	97616	Lipoxygenase OS=Arachis hypogaea OX=3818 PE=2 SV=1
**5**	21032	tr|Q4JME6|Q4JME6_ARAHY	66.63	2	2	1.35E3	1	1	1	N	97476	Lipoxygenase OS=Arachis hypogaea OX=3818 GN=Pnlox3 PE=2 SV=1
**5**	21033	tr|Q4JME7|Q4JME7_ARAHY	66.63	2	2	1.35E3	1	1	1	N	97596	Lipoxygenase OS=Arachis hypogaea OX=3818 GN=Pnlox2 PE=2 SV=1
**3**	20853	tr|N1NG13|N1NG13_ARAHY	59.25	2	2	3.97E3	1	1	3	N	71345	Seed storage protein Ara h1 OS=Arachis hypogaea OX=3818 GN=ARAX_AHF417E07-017 PE=4 SV=1
**3**	20854	P43238|ALL12_ARAHY	59.25	2	2	3.97E3	1	1	3	N	71345	Allergen Ara h 1, clone P41B OS=Arachis hypogaea OX=3818 PE=1 SV=1
**3**	20850	P43237|ALL11_ARAHY	59.25	2	2	3.97E3	1	1	3	N	70283	Allergen Ara h 1, clone P17 OS=Arachis hypogaea OX=3818 PE=1 SV=1
**3**	20851	tr|B3IXL2|B3IXL2_ARAHY	59.25	2	2	3.97E3	1	1	3	N	70283	Main allergen Ara h1 OS=Arachis hypogaea OX=3818 PE=2 SV=1
**3**	20849	tr|Q6PSU3|Q6PSU3_ARAHY	59.25	2	2	3.97E3	1	1	3	N	66575	Conarachin (Fragment) OS=Arachis hypogaea OX=3818 PE=4 SV=1
**3**	22400	tr|Q6PSU4|Q6PSU4_ARAHY	59.25	3	3	3.97E3	1	1	3	N	48095	Conarachin (Fragment) OS=Arachis hypogaea OX=3818 PE=2 SV=1
**9**	22753	tr|A0A126DIH0|A0A126DIH0_ARAHY	23.66	2	2	1.33E4	1	1	1	N	39071	DnaJ OS=Arachis hypogaea OX=3818 PE=2 SV=1
**7**	20593	tr|Q6IWG5|Q6IWG5_ARAHY	21.09	5	5	3.91E3	1	1	1	N	58061	Glycinin (Fragment) OS=Arachis hypogaea OX=3818 PE=2 SV=1
**7**	20595	tr|E5G077|E5G077_ARAHY	21.09	5	5	3.91E3	1	1	1	N	58305	Ara h 3 allergen OS=Arachis hypogaea OX=3818 GN=ara h 3 PE=3 SV=1
**7**	20594	tr|Q0GM57|Q0GM57_ARAHY	21.09	5	5	3.91E3	1	1	1	N	58263	Iso-Ara h3 OS=Arachis hypogaea OX=3818 PE=2 SV=1
**total 13 proteins**												

tr|Q9M5D3|Q9M5D3_ARAHY
back to list

| Protein Coverage | Supporting Peptides |

Protein Coverage:

1 MVSVRRSAGI RGRAGILNRG HKIKWTVVLM RKNVLDVDTF TDVAATANIG GLIGTGINVI GSTVDALTAF LGRSVSLQLI
81 SSTQSDVNGN GKVVKDTFLE GIIASLPTLE LENLAFSIHF EWDDSMGIPG AFYIKNYMQV EFFLKTLTLE DVPNQGTIHF
161 VCNSWVYNSK LYKSPRIFFS NKPYLPSETP APLVKYREED LKILRGDGKG ERQEHERIYD YDVYNDLGNP DRNENHARPI
241 LGGSTTFLTS QGRTGRYPAR NDPNSEKPGD VYVPRDENFG HLKSSDFLAN SIKFLTRYVL PAFESVFDLN LTPNEFDSFQ
321 DVRDLYEGGI RLPTEVISTK RRLLVIKELF RTDGEQVLKF PPPRFLQVNE ENSAWMTDEE FVIINELLVS HPCMDPTIYG
401 DQNSKIPAEV LDLEGCSLEE AINGRRLFIL DYHDVFMPYV RRINETHAKA YATRTILFLK EDGTLNPVAI ELSLPHPDGD
481 KSGAISDVIL PAKGRCRKHN LATSQSLCHI NDSCYHHLMS HWLNTHAVIE PFVIATNRQL SVIHPIYKLL SPHYRDTMNI
561 NALARQNLIN SDGIIERTFL PSKFSVEMSS AVYKNWVFTD QALPADLIKR GMAVEDSSSP YGIRLVIEDY PYAVDGLEIW
641 FAIKEWVQDY VSLYYPTDND LKKDPELQNW WKEAVEVGHG DLKDKPWWPK MQTVEELVES CTTIIWTASA LHAAVNFGQY
721 PYGGLILNRP TLSRRLLPEQ GTAEYEEMVK SHQKAYLRTI TPK**LETLIDL TTIEILSK**HA SDEVYLGERD NPHWTFDSRA

801 LEAFQRFGNK LSEIEEKLTE KNKDGRLSNR IGPVELPYTL LHPTSNEGLT FRGVPNSISI

Supporting Peptides:

Peptide	Uniq	-10lgP	Mass	Length	ppm	m/z	z	RT	Fraction	Scan	Source File	Area Digest traka C 65-70 kDa	#Feature	#Feature Digest traka C 65-70 kDa	Start	End	PTM
K.LETLIDLTTIEILSK.H	Y	66.63	1700.9811	15	-4.3	851.4941	2	37.42	40	1931	OB4068.raw	1.35E3	1	1	764	778	
**total 1 peptides**																	

tr|Q4JME6|Q4JME6_ARAHY
back to list

| Protein Coverage | Supporting Peptides |

Protein Coverage:

1 MFSGVAGILN RGHKIKGTVV LMRKNVLDVD TFTDVAATAN IGGLIGTGIN VIGSTVDALT AFLGRSVSLQ LISSTQSDEN
81 GNGKVVKDTF LEGIIASLPT LGAGESAFSI HFEWDDSMGI PGAFYIKNYM QVEFFLKTLT LEDVPNQGTI HFVCNSWVYN
161 SKLYKSPRIF FSNKPYLPSE TPAPLVKYRE EDLKILRGDG KGERQEHERI YDYDVYNDLG NPDRNENHAR PILGGSTTFP
241 YPRRGRTGRY PARNDPNSEK PGDVYVPRDE NFGHLKSSDF LANSIKFLTR YVLPAFESVF DLNLTPNEFD SFQDVRDLYE
321 GGIRLPTEVI STISPLPVIK ELFRTDGEQV LKFPPPHIIQ VNKSAWMTDE EFAREMIAGV NPCMIRSLQE FPPKSNLDPT
401 IYGDQNSKIP AEVLDLEGCS LEEAINGRRL FILDYHDVFM PYVRRINETH AKAYATRTIL FLKEDGTLKP VAIELSLPHP
481 DGDKSGAISE VILPAKEGVE STIWLLAKAY VIVNDSCYHQ LMSHWLNTHA VIEPFVIATN RQLSVIHPIY KLLSPHYLDT
561 MNINALARQN LINSDGIIER TFLPSKFSVE MSSAVYKNWV FTDQALPADL IKRGMAVEDS SSPYGIRLVI EDYPYAVDGL
641 EIWFAIKEWV QDYVSLYYPT DNDLKKDPEL QNWWKEAVEV GHGDLKDKPW WPKMQTVEEL VESCTTIIWT ASALHAAVNF
721 GQYPYGGLIL NRPTLSRRLL PEQGTAEYEE MVKSHQKAYL RTITPK**LETL IDLTTIEILS K**HASDEVYLG ERDNPHWTFD

801 SRALEAFQRF GNKLSEIEEK LTEKNKDGRL SNRIGPVELP YTLLHPTSNE GLTFRGVPNS ISI

Supporting Peptides:

Peptide	Uniq	-10lgP	Mass	Length	ppm	m/z	z	RT	Fraction	Scan	Source File	Area Digest traka C 65-70 kDa	#Feature	#Feature Digest traka C 65-70 kDa	Start	End	PTM
K.LETLIDLTTIEILSK.H	Y	66.63	1700.9811	15	-4.3	851.4941	2	37.42	40	1931	OB4068.raw	1.35E3	1	1	767	781	
**total 1 peptides**																	

tr|Q4JME7|Q4JME7_ARAHY
back to list

| Protein Coverage | Supporting Peptides |

Protein Coverage:

1 MFSGVTGMLN RGHKIKGTVV LMRKNVLDVD TFTDVVATAN IGGLIGTGIN VIGSTVDALT AFLGRSVSLQ LISSTQSDEN
81 GNGKVVKDTF LEGIIASLPT LGAGESAFSI HFEWDDSMGI PGAFYIKNYM QVEFFLKTLT LEDVPNQGTI HFVCNSWVYN
161 SKLYKSPRIF FSNKPYLPSE TPAPLVKYRE EDLKNLRGDG KGERQEHERI YDYDVYNDLG NPDRNENHAR PILGGSTTFP
241 YPRRGRTGRY PARNDPNSEK PGDVYVPRDE NFGHLKSSDF LANSIKFLTR YVLPAFESVF DLNLTPNEFD SFQDVRDLYE
321 GGIRLPTEVI STISPLPVIK ELFRTDGEQV LKFPPPHIIQ VNKSAWMTDE EFAREMIAGV NPCMIRSLQE FPPKSNLDPT
401 IYGDQNSKIP AEVLDLEGCS LEEAINGRRL FILDYHDVFM PYVRRINETH AKAYATRTIL FLKEDGTLKP VAIELSLPHP
481 DGDKSGAISE VILPAKEGVE STIWLLAKAY VIVNDSCYHQ LMSHWLNTHA VIEPFVIATN RQLSVIHPIY KLLSPHYRDT
561 MNINALARQN LINSDGIIER TFLPSKFSVE MSSAVYKNWV FTDQALPADL IKRGMAVEDS SSPYGIRLVI EDYPYAVDGL
641 EIWFAIKEWV QDYVSLYYPT DNDLKKDPEL QNWWKEAVEV GHGDLKDKPW WPKMQTVEEL VESCTTIIWT ASALHAAVNF
721 GQYPYGGLIL NRPTLSRRLL PEQGTAEYEE MVKSHQKAYL RTITPK**LETL IDLTTIEILS K**HASDEVYLG ERDNPHWTFD
801 SRALEAFQRF GNKLSEIEEK LTEKNKDGRL SNRIGPVELP YTLLHPTSNE GLTFRGVPNS ISI

Supporting Peptides:

Peptide	Uniq	-10lgP	Mass	Length	ppm	m/z	z	RT	Fraction	Scan	Source File	Area Digest traka C 65-70 kDa	#Feature	#Feature Digest traka C 65-70 kDa	Start	End	PTM
K.LETLIDLTTIEILSK.H	Y	66.63	1700.9811	15	-4.3	851.4941	2	37.42	40	1931	OB4068.raw	1.35E3	1	1	767	781	
**total 1 peptides**																	

tr|N1NG13|N1NG13_ARAHY
back to list

| Protein Coverage | Supporting Peptides |

Protein Coverage:

1 MRGRVSPLML LLGILVLASV SATHAKSSPY QKKTENPCAQ RCLQSCQQEP DDLKQKACES RCTKLEYDPR CVYDPRGHTG
81 TTNQRSPPGE RTRGRQPGDY DDDRRQPRRE EGGRWGPAGP REREREEDWR QPREDWRRPS HQQPRKIRPE GREGEQEWGT
161 PGSHVREETS RNNPFYFPSR RFSTRYGNQN GRIRVLQRFD QRSRQFQNLQ NHRIVQIEAK PNTLVLPKHA DADNILVIQQ
241 GQATVTVANG NNRKSFNLDE GHALR**IPSGF ISYILNR**HDN QNLRVAKISM PVNTPGQFED FFPASSRDQS SYLQGFSRNT
321 LEAAFNAEFN EIRRVLLEEN AGGEQEERGQ RRWSTRSSEN NEGVIVKVSK EHVEELTKHA KSVSKKGSEE EGDITNPINL
401 REGEPDLSNN FGKLFEVKPD KKNPQLQDLD MMLTCVEIKE GALMLPHFNS KAMVIVVVNK GTGNLELVAV RKEQQQRGRR
481 EEEEDEDEEE EGSNREVRRY TARLKEGDVF IMPAAHPVAI NASSELHLLG FGINAENNHR IFLAGDKDNV IDQIEKQAKD
561 LAFPGSGEQV EKLIKNQKES HFVSARPQSQ SQSPSSPEKE SPEKEDQEEE NQGGKGPLLS ILKAFN

Supporting Peptides:

Peptide	Uniq	-10lgP	Mass	Length	ppm	m/z	z	RT	Fraction	Scan	Source File	Area Digest traka C 65-70 kDa	#Feature	#Feature Digest traka C 65-70 kDa	Start	End	PTM
R.IPSGFISYILNR.H	Y	59.25	1378.7609	12	0.5	690.3881	2	35.25	40	1805	OB4068.raw	3.97E3	1	1	266	277	

Peptide	Uniq	-10lgP	Mass	Length	ppm	m/z	z	RT	Fraction	Scan	Source File	Area Digest traka C 65-70 kDa	#Feature	#Feature Digest traka C 65-70 kDa	Start	End	PTM
total 1 peptides																	

P43238|ALL12_ARAHY
back to list

| Protein Coverage | Supporting Peptides |

Protein Coverage:

```
  1  MRGRVSPLML LLGILVLASV SATHAKSSPY QKKTENPCAQ RCLQSCQQEP DDLKQKACES RCTKLEYDPR CVYDPRGHTG
 81  TTNQRSPPGE RTRGRQPGDY DDDRRQPRRE EGGRWGPAGP REREREEDWR QPREDWRRPS HQQPRKIRPE GREGEQEWGT
161  PGSHVREETS RNNPFYFPSR RFSTRYGNQN GRIRVLQRFD QRSRQFQNLQ NHRIVQIEAK PNTLVLPKHA DADNILVIQQ
241  GQATVTVANG NNRKSFNLDE GHALRIPSGF ISYILNRHDN QNLRVAKISM PVNTPGQFED FFPASSRDQS SYLQGFSRNT
321  LEAAFNAEFN EIRRVLLEEN AGGEQEERGQ RRWSTRSSEN NEGVIVKVSK EHVEELTKHA KSVSKKGSEE EGDITNPINL
401  REGEPDLSNN FGKLFEVKPD KKNPQLQDLD MMLTCVEIKE GALMLPHFNS KAMVIVVVNK GTGNLELVAV RKEQQQRGRR
481  EEEEDEDEEE EGSNREVRRY TARLKEGDVF IMPAAHPVAI NASSELHLLG FGINAENNHR IFLAGDKDNV IDQIEKQAKD
561  LAFPGSGEQV EKLIKNQKES HFVSARPQSQ SQSPSSPEKE SPEKEDQEEE NQGGKGPLLS ILKAFN
```

Supporting Peptides:

Peptide	Uniq	-10lgP	Mass	Length	ppm	m/z	z	RT	Fraction	Scan	Source File	Area Digest traka C 65-70 kDa	#Feature	#Feature Digest traka C 65-70 kDa	Start	End	PTM
R.IPSGFISYILNR.H	Y	59.25	1378.7609	12	0.5	690.3881	2	35.25	40	1805	OB4068.raw	3.97E3	1	1	266	277	
total 1 peptides																	

P43237|ALL11_ARAHY
back to list

| Protein Coverage | Supporting Peptides |

Protein Coverage:

```
  1  MRGRVSPLML LLGILVLASV SATQAKSPYR KTENPCAQRC LQSCQQEPDD LKQKACESRC TKLEYDPRCV YDTGATNQRH
 81  PPGERTRGRQ PGDYDDDRRQ PRREEGGRWG PAEPRERERE EDWRQPREDW RRPSHQQPRK IRPEGREGEQ EWGTPGSEVR
161  EETSRNNPFY FPSRRFSTRY GNQNGRIRVL QRFDQRSKQF QNLQNHRIVQ IEARPNTLVL PKHADADNIL VIQQGQATVT
241  VANGNNRKSF NLDEGHALRI PSGFISYILN RHDNQNLRVA KISMPVNTPG QFEDFFPASS RDQSSYLQGF SRNTLEAAFN
321  AEFNEIRRVL LEENAGGEQE ERGQRRRSTR SSDNEGVIVK VSKEHVQELT KHAKSVSKKG SEEEDITNPI NLRDGEPDLS
401  NNFGRLFEVK PDKKNPQLQD LDMMLTCVEI KEGALMLPHF NSKAMVIVVV NKGTGNLELV AVRKEQQQRG RREQEWEEEE
481  EDEEEEGSNR EVRRYTARLK EGDVFIMPAA HPVAINASSE LHLLGFGINA ENNHRIFLAG DKDNVIDQIE KQAKDLAFPG
561  SGEQVEKLIK NQRESHFVSA RPQSQSPSSP EKEDQEEENQ GGKGPLLSIL KAFN
```

Supporting Peptides:

Peptide	Uniq	-10lgP	Mass	Length	ppm	m/z	z	RT	Fraction	Scan	Source File	Area Digest traka C 65-70 kDa	#Feature	#Feature Digest traka C 65-70 kDa	Start	End	PTM
R.IPSGFISYILNR.H	Y	59.25	1378.7609	12	0.5	690.3881	2	35.25	40	1805	OB4068.raw	3.97E3	1	1	260	271	
total 1 peptides																	

tr|B3IXL2|B3IXL2_ARAHY
back to list

| Protein Coverage | Supporting Peptides |

Protein Coverage:

1 MRGRVSPLML LLGILVLASV SATQAKSPYR KTENPCAQRC LQSCQQEPDD LKQKACESRC TKLEYDPRCV YDTGATNQRH
81 PPGERTRGRQ PGDYDDDRRQ PRREEGGRWG PAEPRERERE EDWRQPREDW RRPSHQQPRK IRPEGREGEQ EWGTPGSEVR
161 EETSRNNPFY FPSRRFSTRY GNQNGRIRVL QRFDQRSKQF QNLQNHRIVQ IEARPNTLVL PKHADADNIL VIQQGQATVT
241 VANGNNRKSF NLDEGHALRI PSGFISYILN RHDNQNLRVA KISMPVNTPG QFEDFFPASS RDQSSYLQGF SRNTLEAAFN
321 AEFNEIRRVL LEENAGGEQE ERGQRRRSTR SSDNEGVIVK VSKEHVQELT KHAKSVSKKG SEEEDITNPI NLRDGEPDLS
401 NNFGRLFEVK PDKKNPQLQD LDMMLTCVEI KEGALMLPHF NSKAMVIVVV NKGTGNLELV AVRKEQQQRG RREQEWEEEE
481 EDEEEEGSNR EVRRYTARLK EGDVFIMPAA HPVAINASSE LHLLGFGINA ENNHRIFLAG DKDNVIDQIE KQAKDLAFPG
561 SGEQVEKLIK NQRESHFVSA RPQSQSPSSP EKEDQEEENQ GGKGPLLSIL KAFN

Supporting Peptides:

Peptide	Uniq	-10lgP	Mass	Length	ppm	m/z	z	RT	Fraction	Scan	Source File	Area Digest traka C 65-70 kDa	#Feature	#Feature Digest traka C 65-70 kDa	Start	End	PTM
R.IPSGFISYILNR.H	Y	59.25	1378.7609	12	0.5	690.3881	2	35.25	40	1805	OB4068.raw	3.97E3	1	1	260	271	
**total 1 peptides**																	

tr|Q6PSU3|Q6PSU3_ARAHY

back to list

| Protein Coverage | Supporting Peptides |

Protein Coverage:

1 MRGRVSPLML LLGILVLASV SATQAKSPYR KTENPCAQRC LQSCQQEPDD LKQKACESRC TKLEYDPRCV YDTGATNQRH
81 PPGERTRGRQ PGDYDDDRRQ PRREEGGRWG PAEPRERERE EDWRQPREDW RRPSHQQPRK IRPEGREGEQ EWGTPGSEVR
161 EETSRNNPFY FPSRRFSTRY GNQNGRIRVL QRFDQRSKQF QNLQNHRIVQ IEARPNTLVL PKHADADNIL VIQQGQATVT
241 VANGNNRKSF NLDEGHALRI PSGFISYILN RHDNQNLRVA KISMPVNTPG QFEDFFPASS RDQSSYLQGF SRNTLEAAFN
321 AEFNEIRRVL LEENAGGEQE ERGQRRRSTR SSDNEGVIVK VSKEHVQELT KHAKSVSKKG SEEEDITNPI NLRDGEPDLS
401 NNFGRLFEVK PDKKNPQLQD LDMMLTCVEI KEGALMLPHF NSKAMVIVVV NKGTGNLELV AVRKEQQQRG RREQEWEEEE
481 EDEEEEGSNR EVRRYTARLK EGDVFIMPAA HPVAINASSE LHLLGFGINA ENNHRIFLAG DKDNVIDQIE KQAKDLAFPG
561 SGEQVEKLIK NQRESHFVSA

Supporting Peptides:

Peptide	Uniq	-10lgP	Mass	Length	ppm	m/z	z	RT	Fraction	Scan	Source File	Area Digest traka C 65-70 kDa	#Feature	#Feature Digest traka C 65-70 kDa	Start	End	PTM
R.IPSGFISYILNR.H	Y	59.25	1378.7609	12	0.5	690.3881	2	35.25	40	1805	OB4068.raw	3.97E3	1	1	260	271	
**total 1 peptides**																	

tr|Q6PSU4|Q6PSU4_ARAHY

back to list

| Protein Coverage | Supporting Peptides |

Protein Coverage:

1 GFDQRSRQFQ NLQNHRIVQI EAKPNTLVLP KHADADNILV IQQGQATVTV ANGNNRKSFN LDEGHALR**IP SGFISYILNR**

81 HDNQNLRVAK ISMPVNTPGQ FEDFFPASSR DQSSYLQGFS RNTLEAAFNA EFNEIRRVLL EENAGGEQEE RGQRRWSTRS

161 SENNEGVIVK VSKEHVEELT KHAKSVSKKG SEEGDITNPI NLREGEPDLS NNFGKLFEVK PDKKNPQLQD LDMMLTCVEI

241 KEGALMLPHF NSKAMVIVVV NKGTGNLELV AVRKEQQQRG RREEEEDEDE EEEGSNREVR RYTARLKEGD VFIMPAAHPV

321 AINASSELHL LGFGINAENN HRIFLAGDKD NVIDQIEKQA KDLAFPGSGE QVEKLIKNQK ESHFVSARPQ SQSQSPSSPE

401 KESPEKEDQE EENQGGKGPL LSILKAFN

Supporting Peptides:

Peptide	Uniq	-10lgP	Mass	Length	ppm	m/z	z	RT	Fraction	Scan	Source File	Area Digest traka C 65-70 kDa	#Feature	#Feature Digest traka C 65-70 kDa	Start	End	PTM
R.IPSGFISYILNR.H	Y	59.25	1378.7609	12	0.5	690.3881	2	35.25	40	1805	OB4068.raw	3.97E3	1	1	69	80	
**total 1 peptides**																	

tr|A0A126DIH0|A0A126DIH0_ARAHY

back to list

| Protein Coverage | Supporting Peptides |

Protein Coverage:

1 MAQLLSPVYA AEAFKIQNPS LNWWSSSRSS WRLLSNKVAS SCNFVTPPLC GGGKRRGGGD SRVRVAAEDS VSRSESVADD

81 YYAVLGLLPD ATPSQIKKAY YDCMKSCHPD LSGNDSETTN FCVFINEVYA VLSDPVQRKV YDEIHGYSLT SMNPFVDDSC

161 PKDHAFVDEF SCIGCKNCTN VAPDVFAIEE DFGRARVYSQ CGNPELVQQA IDSCPVDCIH WTSAAQLSLL EDEMRRIERV

241 NVALMLSGMG GASFDVFRMA SSRWEKRQSK VLERAKMRMM KQQRSDRTDS YWENLWGNPK DYESSEEEVK ERAK**RAAAAA**

321 **RR**WREYSRKG VDKPPTFKLP EASSGKDS

Supporting Peptides:

Peptide	Uniq	-10lgP	Mass	Length	ppm	m/z	z	RT	Fraction	Scan	Source File	Area Digest traka C 65-70 kDa	#Feature	#Feature Digest traka C 65-70 kDa	Start	End	PTM
K.RAAAAARR.W	Y	23.66	841.4994	8	2.9	421.7582	2	26.25	40	1344	OB4068.raw	1.33E4	1	1	315	322	
**total 1 peptides**																	

tr|Q6IWG5|Q6IWG5_ARAHY

back to list

| Protein Coverage | Supporting Peptides |

Protein Coverage:

1 KLLALSLCFC VLVLGASSVT FRQGGEENEC QFQRLNAQRP DNRIESEGGY IETWNPNNQE FQCAGVALSR TVLRRNALRR

81 PFYSNAPLEI YVQQGSGYFG LIFPGCPSTY EEPAQEGRRY QSQKPSRRFQ VGQDDPSQQQ QDSHQKVHRF DEGDLIAVPT

161 GVAFWMYNDE DTDVVTVTLS DTSSIHNQLD QFPRRFYLAG NQEQEFLRYQ QQQGSRPHYR QISPRVRGDE QENEGSNIFS

241 GFAQEFLQHA FQVDRQTVEN LRGENEREEQ GAIVTVKGGL RILSPDEEDE SSRSPPSRRE EFDEDRSRPQ QRGKYDENRR

321 GYKNGIEETI CSASVKKNLG RSSNPDIYNP QAGSLR**SVNE LDLPILGWLG LSAQHGTIYR** NAMFVPHYTL NAHTIVVALN

401 GRAHVQVVDS NGNRVYDEEL QEGHVLVVPQ NFAVAAKAQS ENYEYLAFKT DSRPSIANLA GENSIIDNLP EEVVANSYRL

481 PREQARQLKN NNPFKFFVPP FDHQSMREVA

Supporting Peptides:

Peptide	Uniq	-10lgP	Mass	Length	ppm	m/z	z	RT	Fraction	Scan	Source File	Area Digest traka C 65-70 kDa	#Feature	#Feature Digest traka C 65-70 kDa	Start	End	PTM
R.SVNELDLPILGWLGLSAQHGTIYR.N	Y	21.09	2651.4070	24	0.9	884.8104	3	38.12	40	1981	OB4068.raw	3.91E3	1	1	357	380	
**total 1 peptides**																	

tr|E5G077|E5G077_ARAHY

back to list

| Protein Coverage | Supporting Peptides |

Protein Coverage:

```
  1 MAKLLALSLC FCVLVLGASS VTFRQGGEEN ECQFQRLNAQ RPDNRIESEG GYIETWNPNN QEFQCAGVAL SRTVLRRNAL
 81 RRPFYSNAPL EIYVQQGSGY FGLIFPGCPS TYEEPAQEGR RYQSQKPSRR FQVGQDDPSQ QQQDSHQKVH RFDEGDLIAV
161 PTGVAFWMYN DEDTDVVTVT LSDTSSIHNQ LDQFPRRFYL AGNQEQEFLR YQQQQGSRPH YRQISPRVRG DEQENEGSNI
241 FSGFAQEFLQ HAFQVDRQTV ENLRGENERE EQGAIVTVKG GLRILSPDEE DESSRSPPNR REEFDEDRSR PQQRGKYDEN
321 RRGYKNGIEE TICSASVKKN LGRSSNPDIY NPQAGSLRSV NELDLPILGW LGLSAQHGTI YRNAMFVPHY TLNAHTIVVA
401 LNGRAHVQVV DSNGNRVYDE ELQEGHVLVV PQNFAVAAKA QSENYEYLAF KTDSRPSIAN QAGENSIIDN LPEEVVANSY
481 RLPREQARQL KNNNPFKFFV PPFDHQSMRE VA
```

Supporting Peptides:

Peptide	Uniq	-10lgP	Mass	Length	ppm	m/z	z	RT	Fraction	Scan	Source File	Area Digest traka C 65-70 kDa	#Feature	#Feature Digest traka C 65-70 kDa	Start	End	PTM
R.SVNELDLPILGWLGLSAQHGTIYR.N	Y	21.09	2651.4070	24	0.9	884.8104	3	38.12	40	1981	OB4068.raw	3.91E3	1	1	359	382	
**total 1 peptides**																	

tr|Q0GM57|Q0GM57_ARAHY

back to list

| Protein Coverage | Supporting Peptides |

Protein Coverage:

```
  1 MAKLLALSLC FCVLVLGASS VTFRQGGEEN ECQFQRLNAQ RPDNRIESEG GYIETWNPNN QEFQCAGVAL SRTVLRRNAL
 81 RRPFYSNAPL EIYVQQGSGY FGLIFPGCPS TYEEPAQEGR RYQSQKPSRR FQVGQDDPSQ QQQDSHQKVH RFDEGDLIAV
161 PTGVAFWMYN DEDTDVVTVT LSDTSSIHNQ LDQFPRRFYL AGNQEQEFLR YQQQQGSRPH YRQISPRVRG DEQENEGSNI
241 FSGFAQEFLQ HAFQVDRQTV ENLRGENERE EQGAIVTVKG GLRILSPDEE DESSRSPPSR REEFDEDRSR PQQRGKYDEN
321 RRGYKNGIEE TICSASVKKN LGRSSNPDIY NPQAGSLRSV NELDLPILGW LGLSAQHGTI YRNAMFVPHY TLNAHTIVVA
401 LNGRAHVQVV DSNGNRVYDE ELQEGHVLVV PQNFAVAAKA QSENYEYLAF KTDSRPSIAN LAGENSIIDN LPEEVVANSY
481 RLPREQARQL KNNNPFKFFV PPFDHQSMRE VA
```

Supporting Peptides:

Peptide	Uniq	-10lgP	Mass	Length	ppm	m/z	z	RT	Fraction	Scan	Source File	Area Digest traka C 65-70 kDa	#Feature	#Feature Digest traka C 65-70 kDa	Start	End	PTM
R.SVNELDLPILGWLGLSAQHGTIYR.N	Y	21.09	2651.4070	24	0.9	884.8104	3	38.12	40	1981	OB4068.raw	3.91E3	1	1	359	382	
**total 1 peptides**																	

Peptide List

Summary

1. Notes Gastric Digest Raw peanut Band #15 80-150 kDa (PTM500)

2. Result Statistics

Figure 1. False discovery rate (FDR) curve. X axis is the number of peptide-spectrum matches (PSM) being kept. Y axis is the corresponding FDR.

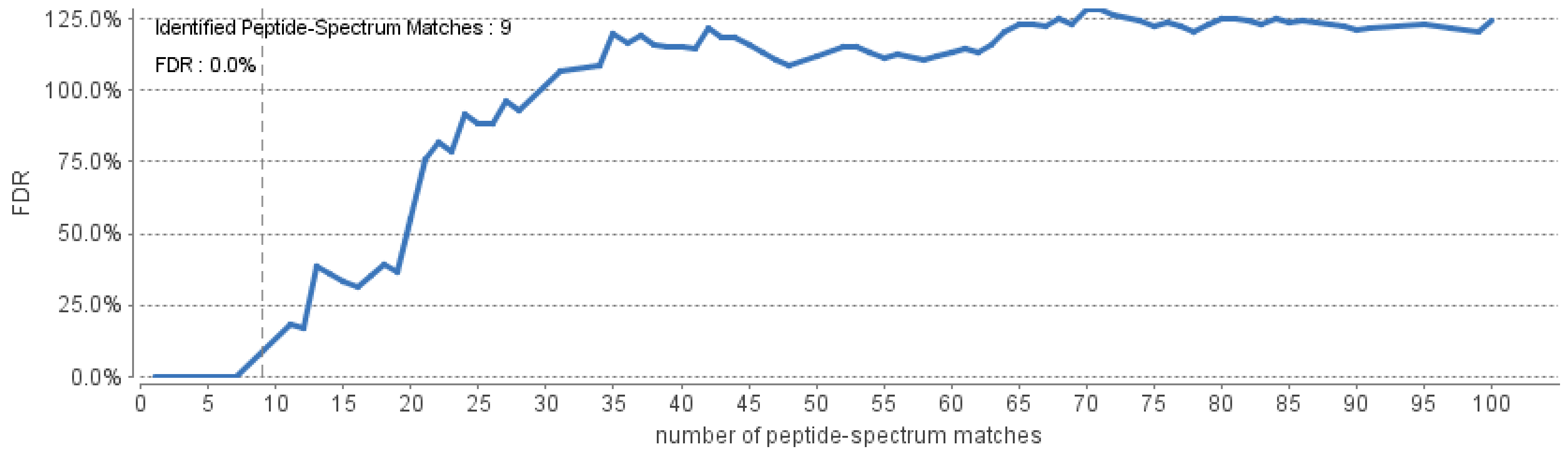

Figure 2. PSM score distribution. **(a)** Distribution of PEAKS peptide score; **(b)** Scatterplot of PEAKS peptide score versus precursor mass error.

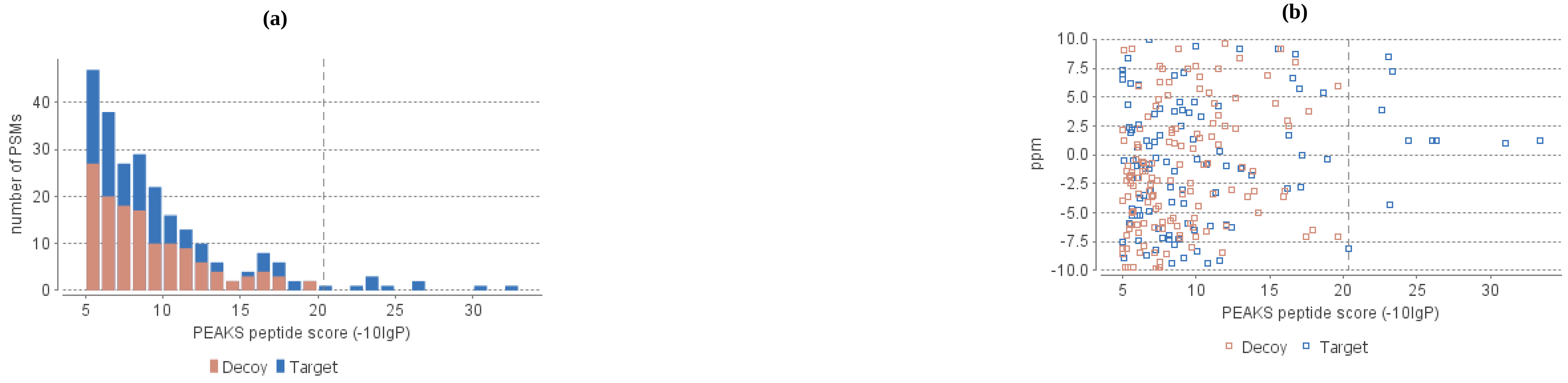

Figure 3. Distribution of peptide feature detection. **(a)** Feature m/z distribution; **(b)** Feature RT distribution

(a) **(b)**

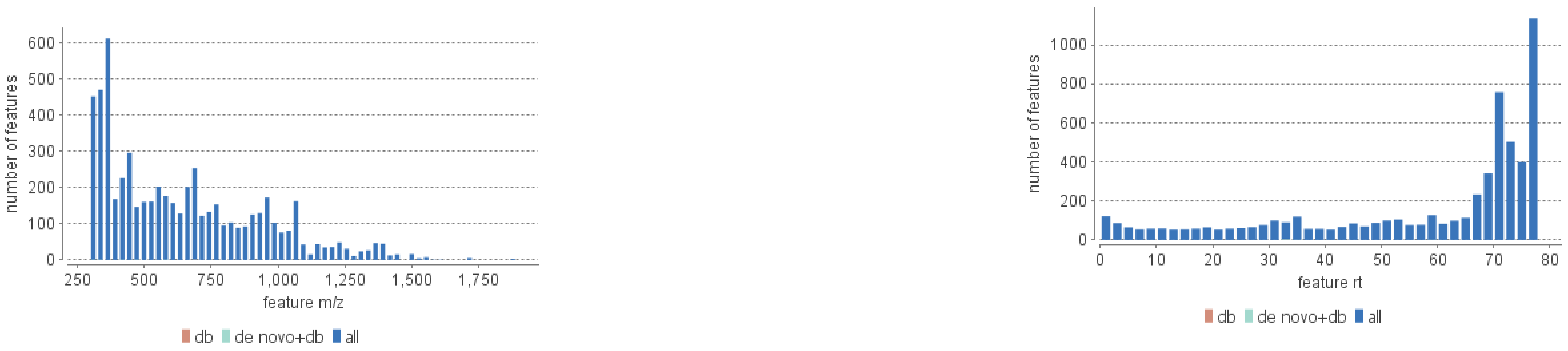

Figure 4. Distribution of identified peptide features. **(a)** Feature abundance distribution **(b)** *De novo* sequencing validation.

(a) **(b)**

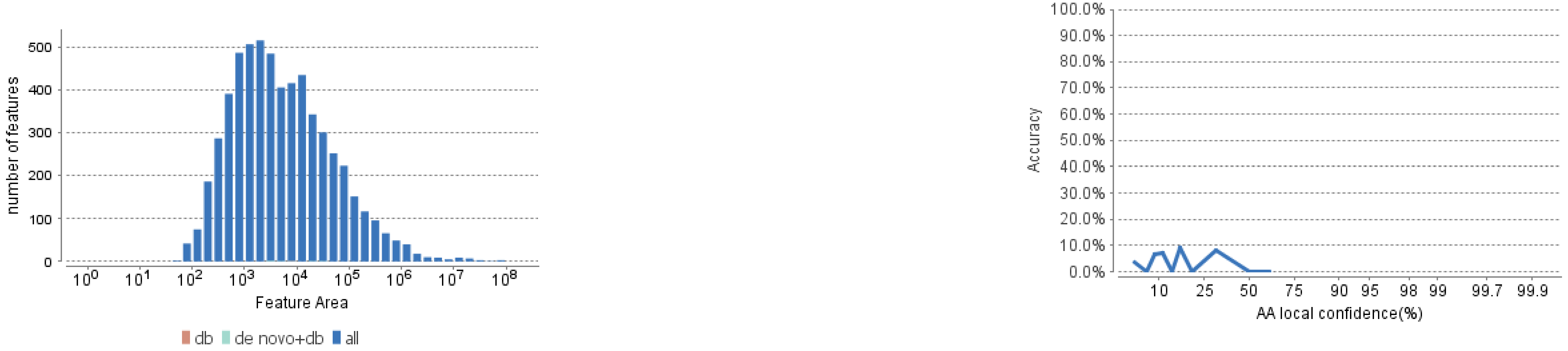

Table 1. Statistics of data.

# of MS scans	3186
# of MS/MS scans	976
# of Features	5939
# of Chimera scans	66

Table 2. Result filtration parameters.

Peptide -10lgP	≥20.4

Table 4. PTM profile.

Name	ΔMass	Position	#PSM	-10lgP	Abundance	AScore
Deamidation	.98	NQ	9	33.37	9.5E3	0.00
Oxidation	15.99	M	1	23.18		1000.00

PTM Ascore	≥20
Protein -10lgP	≥20
Proteins unique peptides	≥1
De novo ALC Score	≥50%

Table 3. Statistics of filtered result.

Peptide-Spectrum Matches	9
Peptide sequences	9
Protein groups	1
Proteins	6
Proteins (#Unique Peptides)	0 (>2); 6 (=2); 0 (=1);
FDR (Peptide-Spectrum Matches)	0.0%
FDR (Peptide Sequences)	0.0%
FDR (Protein Group)	0.0%
De Novo Only Spectra	5
# of identified Features	6
# of identified MS/MS scans	5

3. Experiment Control

Figure 5. Precursor mass error of peptide-spectrum matches (PSM) in filtered result. **(a)** Distribution of precursor mass error in ppm; **(b)** Scatterplot of precursor m/z versus precursor mass error in ppm.

(a)

(b)

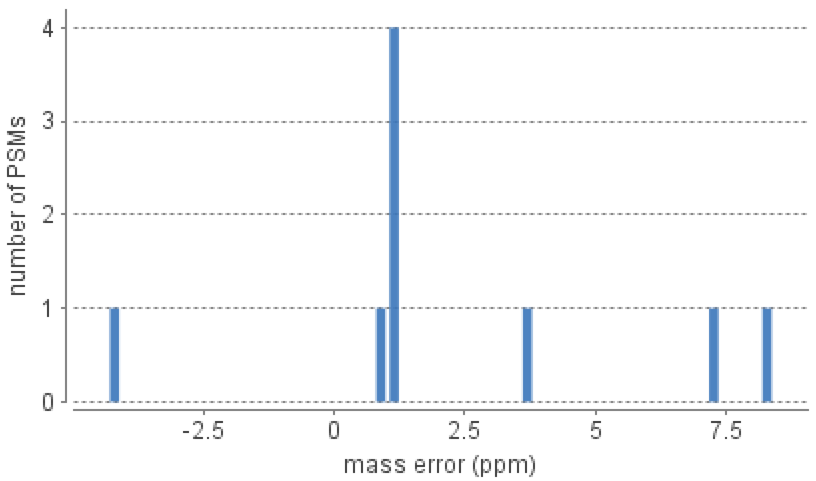

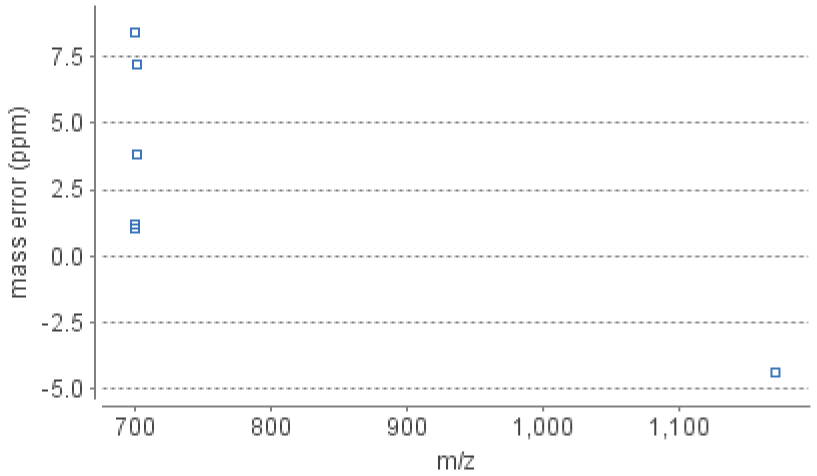

Table 5. Number of identified peptides in each sample by the number of missed cleavages

Missed Cleavages	0	1	2	3	4+
Digest 15. traka 100 kDa	0	1	8	0	0

4. Other Information

Table 6. Search parameters.

Search Engine Name: PEAKS
Parent Mass Error Tolerance: 10.0 ppm
Fragment Mass Error Tolerance: 0.5 Da
Precursor Mass Search Type: monoisotopic
Enzyme: Trypsin
Max Missed Cleavages: 2
Digest Mode: Unspecific
Fixed Modifications:
  Carbamidomethylation: 57.02
Variable Modifications:
  Deamidation (NQ): 0.98
  Oxidation (M): 15.99
  Hydroxylation Pro: 15.99
  Acetylation (K): 42.01
  Acetylation (Protein N-term): 42.01
  Acetylation (N-term): 42.01
  Amidation: -0.98
  Beta-methylthiolation: 45.99
  and 305 more...
Max Variable PTM Per Peptide: 5

Table 7. Instrument parameters.

Fractions: OB4065.raw
Ion Source: ESI(nano-spray)
Fragmentation Mode: CID, CAD(y and b ions)
MS Scan Mode: FT-ICR/Orbitrap
MS/MS Scan Mode: Linear Ion Trap

Database: Uniprot_Peanut-3818_Jul18
Taxon: All
Contaminant Database: contaminantsMQ_mar19
Searched Entry: 1723
FDR Estimation: Enabled
De novo score (ALC%) threshold: 15
Peptide hit threshold (-10logP): 30.0
Peaks run ID: 490
Merge Options: no merge
Precursor Options: corrected
Charge Options: no correction
Filter Charge: 2 - 8
Process: true
Associate chimera: yes

Protein List

Protein Accession Contains:
Protein Description Contains:
Peptide Sample Area >=
Protein Ptm Contains:

Protein Group	Protein ID	Accession	-10lgP	Coverage (%)	Coverage (%) Digest 15. traka 100 kDa	Area Digest 15. traka 100 kDa	#Peptides	#Unique	#Spec Digest 15. traka 100 kDa	PTM	Avg. Mass	Description
**1**	20853	tr|N1NG13|N1NG13_ARAHY	51.75	10	10	3.8E4	2	2	9	Y	71345	Seed storage protein Ara h1 OS=Arachis hypogaea OX=3818 GN=ARAX_AHF417E07-017 PE=4 SV=1
**1**	20854	P43238|ALL12_ARAHY	51.75	10	10	3.8E4	2	2	9	Y	71345	Allergen Ara h 1, clone P41B OS=Arachis hypogaea OX=3818 PE=1 SV=1
**1**	20849	tr|Q6PSU3|Q6PSU3_ARAHY	51.75	11	11	3.8E4	2	2	9	Y	66575	Conarachin (Fragment) OS=Arachis hypogaea OX=3818 PE=4 SV=1
**1**	20850	P43237|ALL11_ARAHY	51.75	10	10	3.8E4	2	2	9	Y	70283	Allergen Ara h 1, clone P17 OS=Arachis hypogaea OX=3818 PE=1 SV=1
**1**	20851	tr|B3IXL2|B3IXL2_ARAHY	51.75	10	10	3.8E4	2	2	9	Y	70283	Main allergen Ara h1 OS=Arachis hypogaea OX=3818 PE=2 SV=1
**1**	22400	tr|Q6PSU4|Q6PSU4_ARAHY	51.75	14	14	3.8E4	2	2	9	Y	48095	Conarachin (Fragment) OS=Arachis hypogaea OX=3818 PE=2 SV=1
**total 6 proteins**												

tr|N1NG13|N1NG13_ARAHY
back to list

| Protein Coverage | Supporting Peptides |
Protein Coverage:

1 MRGRVSPLML LLGILVLASV SATHAKSSPY QKKTENPCAQ RCLQSCQQEP DDLKQKACES RCTKLEYDPR CVYDPRGHTG
81 TTNQRSPPGE RTRGRQPGDY DDDRRQPRRE EGGRWGPAGP REREREEDWR QPREDWRRPS HQQPRKIRPE GREGEQEWGT
161 PGSHVREETS RNNPFYFPSR RFSTRYGNQN GRIRVLQRFD QRSRQFQNLQ NHRIVQIEAK PNTLVLPKHA DADNILVIQQ
276 d 290 o
241 GQATVTVANG NNRK**SFNLDE GHALRIPSGF ISYILNRHDN QNLR**VAK**ISM PVNTPGQFED FFPASSRDQS SYLQGFSR**NT
321 LEAAFNAEFN EIRRVLLEEN AGGEQEERGQ RRWSTRSSEN NEGVIVKVSK EHVEELTKHA KSVSKKGSEE EGDITNPINL
401 REGEPDLSNN FGKLFEVKPD KKNPQLQDLD MMLTCVEIKE GALMLPHFNS KAMVIVVVNK GTGNLELVAV RKEQQQRGRR
481 EEEEDEDEEE EGSNREVRRY TARLKEGDVF IMPAAHPVAI NASSELHLLG FGINAENNHR IFLAGDKDNV IDQIEKQAKD
561 LAFPGSGEQV EKLIKNQKES HFVSARPQSQ SQSPSSPEKE SPEKEDQEEE NQGGKGPLLS ILKAFN

d Deamidation (NQ) (+0.98)
o Oxidation (M) (+15.99)

Supporting Peptides:

Peptide	Uniq	-10lgP	Mass	Length	ppm	m/z	z	RT	Fraction	Scan	Source File	Area Digest 15. traka 100 kDa	#Feature	#Feature Digest 15. traka 100 kDa	Start	End	PTM
K.SFNLDEGHALRIPSGFISYILN(+.98)RHDNQ(+.98)NLR.V	Y	33.37	3497.7439	30	1.2	700.5569	5	33.33	37	1790	OB4065.raw	9.5E3	1	1	255	284	
K.SFNLDEGHALRIPSGFISYILNRHDNQ(+.98)NLR.V	Y	30.99	3496.7600	30	1.0	700.3600	5	33.38	37	1790	OB4065.raw	0	0	0	255	284	
K.SFN(+.98)LDEGHALRIPSGFISYILNRHDNQ(+.98)NLR.V	Y	26.30	3497.7439	30	1.2	700.5569	5	33.33	37	1798	OB4065.raw	9.5E3	1	1	255	284	
K.SFNLDEGHALRIPSGFISYILNRHDN(+.98)Q(+.98)NLR.V	Y	26.06	3497.7439	30	1.2	700.5569	5	33.33	37	1796	OB4065.raw	9.5E3	1	1	255	284	
K.SFNLDEGHALRIPSGFISYILN(+.98)RHDN(+.98)QNLR.V	Y	24.43	3497.7439	30	1.2	700.5569	5	33.33	37	1786	OB4065.raw	9.5E3	1	1	255	284	
K.SFN(+.98)LDEGHALRIPSGFISYILN(+.98)RHDNQ(+.98)NLR.V	Y	23.38	3498.7280	30	7.2	700.7579	5	33.50	37	1798	OB4065.raw	0	0	0	255	284	Deamidation (NQ)
K.ISM(+15.99)PVN(+.98)TPGQFEDFFPASSRDQ(+.98)SSYLQ(+.98)GFSR.N	Y	23.18	3513.5669	31	-4.3	1172.1912	3	33.52	37	1799	OB4065.raw	0	0	0	288	318	Oxidation (M)
K.SFNLDEGHALRIPSGFISYILNRHDN(+.98)QNLR.V	Y	23.06	3496.7600	30	8.4	700.3652	5	33.31	37	1786	OB4065.raw	0	0	0	255	284	
K.SFNLDEGHALRIPSGFISYILN(+.98)RHDN(+.98)Q(+.98)NLR.V	Y	22.67	3498.7280	30	3.8	700.7556	5	33.47	37	1796	OB4065.raw	0	0	0	255	284	
**total 9 peptides**																	

P43238|ALL12_ARAHY
back to list

| Protein Coverage | Supporting Peptides |

Protein Coverage:

1 MRGRVSPLML LLGILVLASV SATHAKSSPY QKKTENPCAQ RCLQSCQQEP DDLKQKACES RCTKLEYDPR CVYDPRGHTG
81 TTNQRSPPGE RTRGRQPGDY DDDRRQPRRE EGGRWGPAGP REREREEDWR QPREDWRRPS HQQPRKIRPE GREGEQEWGT
161 PGSHVREETS RNNPFYFPSR RFSTRYGNQN GRIRVLQRFD QRSRQFQNLQ NHRIVQIEAK PNTLVLPKHA DADNILVIQQ
276 d 290 o
241 GQATVTVANG NNRK**SFNLDE GHALRIPSGF ISYILNRHDN QNLR**VAK**ISM PVNTPGQFED FFPASSRDQS SYLQGFSR**NT
321 LEAAFNAEFN EIRRVLLEEN AGGEQEERGQ RRWSTRSSEN NEGVIVKVSK EHVEELTKHA KSVSKKGSEE EGDITNPINL
401 REGEPDLSNN FGKLFEVKPD KKNPQLQDLD MMLTCVEIKE GALMLPHFNS KAMVIVVVNK GTGNLELVAV RKEQQQRGRR
481 EEEEDEDEEE EGSNREVRRY TARLKEGDVF IMPAAHPVAI NASSELHLLG FGINAENNHR IFLAGDKDNV IDQIEKQAKD
561 LAFPGSGEQV EKLIKNQKES HFVSARPQSQ SQSPSSPEKE SPEKEDQEEE NQGGKGPLLS ILKAFN

d Deamidation (NQ) (+0.98)
o Oxidation (M) (+15.99)

Supporting Peptides:

Peptide	Uniq	-10lgP	Mass	Length	ppm	m/z	z	RT	Fraction	Scan	Source File	Area Digest 15. traka 100 kDa	#Feature	#Feature Digest 15. traka 100 kDa	Start	End	PTM
K.SFNLDEGHALRIPSGFISYILN(+.98)RHDNQ(+.98)NLR.V	Y	33.37	3497.7439	30	1.2	700.5569	5	33.33	37	1790	OB4065.raw	9.5E3	1	1	255	284	
K.SFNLDEGHALRIPSGFISYILNRHDNQ(+.98)NLR.V	Y	30.99	3496.7600	30	1.0	700.3600	5	33.38	37	1790	OB4065.raw	0	0	0	255	284	
K.SFN(+.98)LDEGHALRIPSGFISYILNRHDNQ(+.98)NLR.V	Y	26.30	3497.7439	30	1.2	700.5569	5	33.33	37	1798	OB4065.raw	9.5E3	1	1	255	284	
K.SFNLDEGHALRIPSGFISYILNRHDN(+.98)Q(+.98)NLR.V	Y	26.06	3497.7439	30	1.2	700.5569	5	33.33	37	1796	OB4065.raw	9.5E3	1	1	255	284	
K.SFNLDEGHALRIPSGFISYILN(+.98)RHDN(+.98)QNLR.V	Y	24.43	3497.7439	30	1.2	700.5569	5	33.33	37	1786	OB4065.raw	9.5E3	1	1	255	284	
K.SFN(+.98)LDEGHALRIPSGFISYILN(+.98)RHDNQ(+.98)NLR.V	Y	23.38	3498.7280	30	7.2	700.7579	5	33.50	37	1798	OB4065.raw	0	0	0	255	284	Deamidation (NQ)
K.ISM(+15.99)PVN(+.98)TPGQFEDFFPASSRDQ(+.98)SSYLQ(+.98)GFSR.N	Y	23.18	3513.5669	31	-4.3	1172.1912	3	33.52	37	1799	OB4065.raw	0	0	0	288	318	Oxidation (M)
K.SFNLDEGHALRIPSGFISYILNRHDN(+.98)QNLR.V	Y	23.06	3496.7600	30	8.4	700.3652	5	33.31	37	1786	OB4065.raw	0	0	0	255	284	
K.SFNLDEGHALRIPSGFISYILN(+.98)RHDN(+.98)Q(+.98)NLR.V	Y	22.67	3498.7280	30	3.8	700.7556	5	33.47	37	1796	OB4065.raw	0	0	0	255	284	
**total 9 peptides**																	

tr|Q6PSU3|Q6PSU3_ARAHY
back to list

| Protein Coverage | Supporting Peptides |

Protein Coverage:

```
  1 MRGRVSPLML LLGILVLASV SATQAKSPYR KTENPCAQRC LQSCQQEPDD LKQKACESRC TKLEYDPRCV YDTGATNQRH
 81 PPGERTRGRQ PGDYDDDRRQ PRREEGGRWG PAEPRERERE EDWRQPREDW RRPSHQQPRK IRPEGREGEQ EWGTPGSEVR
161 EETSRNNPFY FPSRRFSTRY GNQNGRIRVL QRFDQRSKQF QNLQNHRIVQ IEARPNTLVL PKHADADNIL VIQQGQATVT
                                          270                     284
                                          d                       o
241 VANGNNRKSF NLDEGHALRI PSGFISYILN RHDNQNLRVA KISMPVNTPG QFEDFFPASS RDQSSYLQGF SRNTLEAAFN
321 AEFNEIRRVL LEENAGGEQE ERGQRRRSTR SSDNEGVIVK VSKEHVQELT KHAKSVSKKG SEEEDITNPI NLRDGEPDLS
401 NNFGRLFEVK PDKKNPQLQD LDMMLTCVEI KEGALMLPHF NSKAMVIVVV NKGTGNLELV AVRKEQQQRG RREQEWEEEE
481 EDEEEEGSNR EVRRYTARLK EGDVFIMPAA HPVAINASSE LHLLGFGINA ENNHRIFLAG DKDNVIDQIE KQAKDLAFPG
561 SGEQVEKLIK NQRESHFVSA
```

d Deamidation (NQ) (+0.98)
o Oxidation (M) (+15.99)

Supporting Peptides:

Peptide	Uniq	-10lgP	Mass	Length	ppm	m/z	z	RT	Fraction	Scan	Source File	Area Digest 15. traka 100 kDa	#Feature	#Feature Digest 15. traka 100 kDa	Start	End	PTM
K.SFNLDEGHALRIPSGFISYILN(+.98)RHDNQ(+.98)NLR.V	Y	33.37	3497.7439	30	1.2	700.5569	5	33.33	37	1790	OB4065.raw	9.5E3	1	1	249	278	
K.SFNLDEGHALRIPSGFISYILNRHDNQ(+.98)NLR.V	Y	30.99	3496.7600	30	1.0	700.3600	5	33.38	37	1790	OB4065.raw	0	0	0	249	278	
K.SFN(+.98)LDEGHALRIPSGFISYILNRHDNQ(+.98)NLR.V	Y	26.30	3497.7439	30	1.2	700.5569	5	33.33	37	1798	OB4065.raw	9.5E3	1	1	249	278	
K.SFNLDEGHALRIPSGFISYILNRHDN(+.98)Q(+.98)NLR.V	Y	26.06	3497.7439	30	1.2	700.5569	5	33.33	37	1796	OB4065.raw	9.5E3	1	1	249	278	
K.SFNLDEGHALRIPSGFISYILN(+.98)RHDN(+.98)QNLR.V	Y	24.43	3497.7439	30	1.2	700.5569	5	33.33	37	1786	OB4065.raw	9.5E3	1	1	249	278	
K.SFN(+.98)LDEGHALRIPSGFISYILN(+.98)RHDNQ(+.98)NLR.V	Y	23.38	3498.7280	30	7.2	700.7579	5	33.50	37	1798	OB4065.raw	0	0	0	249	278	Deamidation (NQ)
K.ISM(+15.99)PVN(+.98)TPGQFEDFFPASSRDQ(+.98)SSYLQ(+.98)GFSR.N	Y	23.18	3513.5669	31	-4.3	1172.1912	3	33.52	37	1799	OB4065.raw	0	0	0	282	312	Oxidation (M)
K.SFNLDEGHALRIPSGFISYILNRHDN(+.98)QNLR.V	Y	23.06	3496.7600	30	8.4	700.3652	5	33.31	37	1786	OB4065.raw	0	0	0	249	278	
K.SFNLDEGHALRIPSGFISYILN(+.98)RHDN(+.98)Q(+.98)NLR.V	Y	22.67	3498.7280	30	3.8	700.7556	5	33.47	37	1796	OB4065.raw	0	0	0	249	278	
**total 9 peptides**																	

P43237|ALL11_ARAHY
back to list

| Protein Coverage | Supporting Peptides |

Protein Coverage:

```
  1  MRGRVSPLML LLGILVLASV SATQAKSPYR KTENPCAQRC LQSCQQEPDD LKQKACESRC TKLEYDPRCV YDTGATNQRH
 81  PPGERTRGRQ PGDYDDDRRQ PRREEGGRWG PAEPRERERE EDWRQPREDW RRPSHQQPRK IRPEGREGEQ EWGTPGSEVR
161  EETSRNNPFY FPSRRFSTRY GNQNGRIRVL QRFDQRSKQF QNLQNHRIVQ IEARPNTLVL PKHADADNIL VIQQGQATVT
                                          270                           284
                                           d                             o
241  VANGNNRKSF NLDEGHALRI PSGFISYILN RHDNQNLRVA KISMPVNTPG QFEDFFPASS RDQSSYLQGF SRNTLEAAFN
321  AEFNEIRRVL LEENAGGEQE ERGQRRRSTR SSDNEGVIVK VSKEHVQELT KHAKSVSKKG SEEEDITNPI NLRDGEPDLS
401  NNFGRLFEVK PDKKNPQLQD LDMMLTCVEI KEGALMLPHF NSKAMVIVVV NKGTGNLELV AVRKEQQQRG RREQEWEEEE
481  EDEEEEGSNR EVRRYTARLK EGDVFIMPAA HPVAINASSE LHLLGFGINA ENNHRIFLAG DKDNVIDQIE KQAKDLAFPG
561  SGEQVEKLIK NQRESHFVSA RPQSQSPSSP EKEDQEEENQ GGKGPLLSIL KAFN
```

d Deamidation (NQ) (+0.98)
o Oxidation (M) (+15.99)

Supporting Peptides:

Peptide	Uniq	-10lgP	Mass	Length	ppm	m/z	z	RT	Fraction	Scan	Source File	Area Digest 15. traka 100 kDa	#Feature	#Feature Digest 15. traka 100 kDa	Start	End	PTM
K.SFNLDEGHALRIPSGFISYILN(+.98)RHDNQ(+.98)NLR.V	Y	33.37	3497.7439	30	1.2	700.5569	5	33.33	37	1790	OB4065.raw	9.5E3	1	1	249	278	
K.SFNLDEGHALRIPSGFISYILNRHDNQ(+.98)NLR.V	Y	30.99	3496.7600	30	1.0	700.3600	5	33.38	37	1790	OB4065.raw	0	0	0	249	278	
K.SFN(+.98)LDEGHALRIPSGFISYILNRHDNQ(+.98)NLR.V	Y	26.30	3497.7439	30	1.2	700.5569	5	33.33	37	1798	OB4065.raw	9.5E3	1	1	249	278	
K.SFNLDEGHALRIPSGFISYILNRHDN(+.98)Q(+.98)NLR.V	Y	26.06	3497.7439	30	1.2	700.5569	5	33.33	37	1796	OB4065.raw	9.5E3	1	1	249	278	
K.SFNLDEGHALRIPSGFISYILN(+.98)RHDN(+.98)QNLR.V	Y	24.43	3497.7439	30	1.2	700.5569	5	33.33	37	1786	OB4065.raw	9.5E3	1	1	249	278	
K.SFN(+.98)LDEGHALRIPSGFISYILN(+.98)RHDNQ(+.98)NLR.V	Y	23.38	3498.7280	30	7.2	700.7579	5	33.50	37	1798	OB4065.raw	0	0	0	249	278	Deamidation (NQ)
K.ISM(+15.99)PVN(+.98)TPGQFEDFFPASSRDQ(+.98)SSYLQ(+.98)GFSR.N	Y	23.18	3513.5669	31	-4.3	1172.1912	3	33.52	37	1799	OB4065.raw	0	0	0	282	312	Oxidation (M)
K.SFNLDEGHALRIPSGFISYILNRHDN(+.98)QNLR.V	Y	23.06	3496.7600	30	8.4	700.3652	5	33.31	37	1786	OB4065.raw	0	0	0	249	278	
K.SFNLDEGHALRIPSGFISYILN(+.98)RHDN(+.98)Q(+.98)NLR.V	Y	22.67	3498.7280	30	3.8	700.7556	5	33.47	37	1796	OB4065.raw	0	0	0	249	278	
**total 9 peptides**																	

tr|B3IXL2|B3IXL2_ARAHY

back to list

| Protein Coverage | Supporting Peptides |

Protein Coverage:

1 MRGRVSPLML LLGILVLASV SATQAKSPYR KTENPCAQRC LQSCQQEPDD LKQKACESRC TKLEYDPRCV YDTGATNQRH

d Deamidation (NQ) (+0.98)
o Oxidation (M) (+15.99)

81 PPGERTRGRQ PGDYDDDRRQ PRREEGGRWG PAEPRERERE EDWRQPREDW RRPSHQQPRK IRPEGREGEQ EWGTPGSEVR

161 EETSRNNPFY FPSRRFSTRY GNQNGRIRVL QRFDQRSKQF QNLQNHRIVQ IEARPNTLVL PKHADADNIL VIQQGQATVT

241 VANGNNRK**SF NLDEGHALRI PSGFISYILN RHDNQNLR**VA K**ISMPVNTPG QFEDFFPASS RDQSSYLQGF SR**NTLEAAFN

321 AEFNEIRRVL LEENAGGEQE ERGQRRRSTR SSDNEGVIVK VSKEHVQELT KHAKSVSKKG SEEEDITNPI NLRDGEPDLS

401 NNFGRLFEVK PDKKNPQLQD LDMMLTCVEI KEGALMLPHF NSKAMVIVVV NKGTGNLELV AVRKEQQQRG RREQEWEEEE

481 EDEEEEGSNR EVRRYTARLK EGDVFIMPAA HPVAINASSE LHLLGFGINA ENNHRIFLAG DKDNVIDQIE KQAKDLAFPG

561 SGEQVEKLIK NQRESHFVSA RPQSQPSSSP EKEDQEEENQ GGKGPLLSIL KAFN

Supporting Peptides:

Peptide	Uniq	-10lgP	Mass	Length	ppm	m/z	z	RT	Fraction	Scan	Source File	Area Digest 15. traka 100 kDa	#Feature	#Feature Digest 15. traka 100 kDa	Start	End	PTM
K.SFNLDEGHALRIPSGFISYILN(+.98)RHDNQ(+.98)NLR.V	Y	33.37	3497.7439	30	1.2	700.5569	5	33.33	37	1790	OB4065.raw	9.5E3	1	1	249	278	
K.SFNLDEGHALRIPSGFISYILNRHDNQ(+.98)NLR.V	Y	30.99	3496.7600	30	1.0	700.3600	5	33.38	37	1790	OB4065.raw	0	0	0	249	278	
K.SFN(+.98)LDEGHALRIPSGFISYILNRHDNQ(+.98)NLR.V	Y	26.30	3497.7439	30	1.2	700.5569	5	33.33	37	1798	OB4065.raw	9.5E3	1	1	249	278	
K.SFNLDEGHALRIPSGFISYILNRHDN(+.98)Q(+.98)NLR.V	Y	26.06	3497.7439	30	1.2	700.5569	5	33.33	37	1796	OB4065.raw	9.5E3	1	1	249	278	
K.SFNLDEGHALRIPSGFISYILN(+.98)RHDN(+.98)QNLR.V	Y	24.43	3497.7439	30	1.2	700.5569	5	33.33	37	1786	OB4065.raw	9.5E3	1	1	249	278	
K.SFN(+.98)LDEGHALRIPSGFISYILN(+.98)RHDNQ(+.98)NLR.V	Y	23.38	3498.7280	30	7.2	700.7579	5	33.50	37	1798	OB4065.raw	0	0	0	249	278	Deamidation (NQ)
K.ISM(+15.99)PVN(+.98)TPGQFEDFFPASSRDQ(+.98)SSYLQ(+.98)GFSR.N	Y	23.18	3513.5669	31	-4.3	1172.1912	3	33.52	37	1799	OB4065.raw	0	0	0	282	312	Oxidation (M)
K.SFNLDEGHALRIPSGFISYILNRHDN(+.98)QNLR.V	Y	23.06	3496.7600	30	8.4	700.3652	5	33.31	37	1786	OB4065.raw	0	0	0	249	278	
K.SFNLDEGHALRIPSGFISYILN(+.98)RHDN(+.98)Q(+.98)NLR.V	Y	22.67	3498.7280	30	3.8	700.7556	5	33.47	37	1796	OB4065.raw	0	0	0	249	278	
**total 9 peptides**																	

tr|Q6PSU4|Q6PSU4_ARAHY

back to list

| Protein Coverage | Supporting Peptides |

Protein Coverage:

1 GFDQRSRQFQ NLQNHRIVQI EAKPNTLVLP KHADADNILV IQQGQATVTV ANGNNRK**SFN LDEGHALRIP SGFISYILNR**

d Deamidation (NQ) (+0.98)
o Oxidation (M) (+15.99)

81 **HDNQNLR**VAK **ISMPVNTPGQ FEDFFPASSR DQSSYLQGFS R**NTLEAAFNA EFNEIRRVLL EENAGGEQEE RGQRRWSTRS

161 SENNEGVIVK VSKEHVEELT KHAKSVSKKG SEEGDITNPI NLREGEPDLS NNFGKLFEVK PDKKNPQLQD LDMMLTCVEI

241 KEGALMLPHF NSKAMVIVVV NKGTGNLELV AVRKEQQQRG RREEEEDEDE EEEGSNREVR RYTARLKEGD VFIMPAAHPV

321 AINASSELHL LGFGINAENN HRIFLAGDKD NVIDQIEKQA KDLAFPGSGE QVEKLIKNQK ESHFVSARPQ SQSQSPSSPE

401 KESPEKEDQE EENQGGKGPL LSILKAFN

Supporting Peptides:

Peptide	Uniq	-10lgP	Mass	Length	ppm	m/z	z	RT	Fraction	Scan	Source File	Area Digest 15. traka 100 kDa	#Feature	#Feature Digest 15. traka 100 kDa	Start	End	PTM
K.SFNLDEGHALRIPSGFISYILN(+.98)RHDNQ(+.98)NLR.V	Y	33.37	3497.7439	30	1.2	700.5569	5	33.33	37	1790	OB4065.raw	9.5E3	1	1	58	87	
K.SFNLDEGHALRIPSGFISYILNRHDNQ(+.98)NLR.V	Y	30.99	3496.7600	30	1.0	700.3600	5	33.38	37	1790	OB4065.raw	0	0	0	58	87	
K.SFN(+.98)LDEGHALRIPSGFISYILNRHDNQ(+.98)NLR.V	Y	26.30	3497.7439	30	1.2	700.5569	5	33.33	37	1798	OB4065.raw	9.5E3	1	1	58	87	
K.SFNLDEGHALRIPSGFISYILNRHDN(+.98)Q(+.98)NLR.V	Y	26.06	3497.7439	30	1.2	700.5569	5	33.33	37	1796	OB4065.raw	9.5E3	1	1	58	87	
K.SFNLDEGHALRIPSGFISYILN(+.98)RHDN(+.98)QNLR.V	Y	24.43	3497.7439	30	1.2	700.5569	5	33.33	37	1786	OB4065.raw	9.5E3	1	1	58	87	
K.SFN(+.98)LDEGHALRIPSGFISYILN(+.98)RHDNQ(+.98)NLR.V	Y	23.38	3498.7280	30	7.2	700.7579	5	33.50	37	1798	OB4065.raw	0	0	0	58	87	Deamidation (NQ)
K.ISM(+15.99)PVN(+.98)TPGQFEDFFPASSRDQ(+.98)SSYLQ(+.98)GFSR.N	Y	23.18	3513.5669	31	-4.3	1172.1912	3	33.52	37	1799	OB4065.raw	0	0	0	91	121	Oxidation (M)
K.SFNLDEGHALRIPSGFISYILNRHDN(+.98)QNLR.V	Y	23.06	3496.7600	30	8.4	700.3652	5	33.31	37	1786	OB4065.raw	0	0	0	58	87	
K.SFNLDEGHALRIPSGFISYILN(+.98)RHDN(+.98)Q(+.98)NLR.V	Y	22.67	3498.7280	30	3.8	700.7556	5	33.47	37	1796	OB4065.raw	0	0	0	58	87	
**total 9 peptides**																	

Peptide List

Prepared with PEAKS ™ (bioinfor.com)
